# A Primary Sequence Analysis of the ARGONAUTE Protein Family in Plants

**DOI:** 10.3389/fpls.2016.01347

**Published:** 2016-08-31

**Authors:** Daniel Rodríguez-Leal, Amanda Castillo-Cobián, Isaac Rodríguez-Arévalo, Jean-Philippe Vielle-Calzada

**Affiliations:** ^1^Grupo de Desarrollo Reproductivo y Apomixis, Unidad de Genómica Avanzada Laboratorio Nacional de Genómica para la Biodiversidad, CINVESTAV IrapuatoIrapuato, Mexico; ^2^Departamento de Ingeniería Genética de Plantas, CINVESTAV IrapuatoIrapuato, Mexico

**Keywords:** ARGONAUTE evolution, phylogenetics, MEME, maximum likelihood, Bayesian inference

## Abstract

Small RNA (sRNA)-mediated gene silencing represents a conserved regulatory mechanism controlling a wide diversity of developmental processes through interactions of sRNAs with proteins of the ARGONAUTE (AGO) family. On the basis of a large phylogenetic analysis that includes 206 *AGO* genes belonging to 23 plant species, *AGO* genes group into four clades corresponding to the phylogenetic distribution proposed for the ten family members of *Arabidopsis thaliana*. A primary analysis of the corresponding protein sequences resulted in 50 sequences of amino acids (blocks) conserved across their linear length. Protein members of the AGO4/6/8/9 and AGO1/10 clades are more conserved than members of the AGO5 and AGO2/3/7 clades. In addition to blocks containing components of the PIWI, PAZ, and DUF1785 domains, members of the AGO2/3/7 and AGO4/6/8/9 clades possess other consensus block sequences that are exclusive of members within these clades, suggesting unforeseen functional specialization revealed by their primary sequence. We also show that AGO proteins of animal and plant kingdoms share linear sequences of blocks that include motifs involved in posttranslational modifications such as those regulating AGO2 in humans and the PIWI protein AUBERGINE in *Drosophila*. Our results open possibilities for exploring new structural and functional aspects related to the evolution of AGO proteins within the plant kingdom, and their convergence with analogous proteins in mammals and invertebrates.

## Introduction

Small RNA (sRNA)-mediated gene silencing has proven to be one of several important mechanisms that regulate plant growth and development. Its action relies on the production of 20–30 nucleotides (nt) double stranded RNAs (dsRNA) that are produced by proteins of the DICER-LIKE (DCL) family after cleavage of long single-stranded RNA precursors that are copied by a family of RNA-dependent RNA polymerases (RDRs) to generate long dsRNA. sRNAs can bind to one or several ARGONAUTE (AGO) proteins and use complementary base pairing to identify and silence their targets after binding single-stranded sRNAs. The mechanisms of AGO-mediated target silencing include transcript cleavage, translational repression, methylation, and subsequent chromatin modifications (Ghildiyal and Zamore, 2009). AGO proteins of plants and animals are classified in three categories based on their association with different types of sRNAs. Whereas, some AGOs in plants and animals interact primarily with microRNAs and short-interfering RNAs (siRNAs), others exclusive of the animal kingdom associate with so-called piwi-interacting RNAs (piRNAs; also known as PIWI proteins). A third group is composed of AGO proteins only present in the worm *Caenorhabditis elegans* (Vaucheret, 2008). Several studies have revealed that AGO proteins contain an N-terminal domain, a PAZ and MID domains, and a PIWI domain that adopts an RNaseH conformation with endonuclease activity (Song et al., 2004); however, to this date no three dimensional conformation of a plant AGO protein has been elucidated, and therefore the evolutionary trends that have shaped the structure and function of these proteins remains to be determined.

Several phylogenetic studies have analyzed the evolutionary relationships of major gene families involved in sRNA mediated silencing in animals and plants (Cerutti and Casas-Mollano, 2006; Kapoor et al., 2008; Murphy et al., 2008; de Jong et al., 2009; Tabach et al., 2013; Swarts et al., 2014). In flowering plants, phylogenetic studies of *AGO, RNA-DEPENDENT RNA POLYMERASE* (*RDR*), and other gene components of the silencing machinery have been restricted to a few species such as *Arabidopsis thaliana* (Arabidopsis), *Populus trichocarpa* (poplar), and *Oryza sativa* (rice; Kapoor et al., 2008; Qian et al., 2011; Bai et al., 2012; Huang et al., 2015), resulting in a structure coalescing around four clades that follows the distribution of the ten *AGO* genes found in the Arabiodpsis genome. Expression and functional analysis of members of particular clades relates these phylogenetic relationships to their potential function (Nonomura et al., 2007; Kapoor et al., 2008; Havecker et al., 2010); however, the evolutionary implications of this phylogenetic architecture cannot be understood without a in-depth analysis that incorporates genes from a wide array of land plants, including algae, bryophytes, and flowering plants for which a complete genome sequence is available.

Here we present a phylogenetic approximation of 206 *AGO* genes contained in 23 different genomes, including algae, bryophytes, gymnosperms, and flowering plants. After showing that all genes were included in one of four major clades encompassing all the diversity of plant AGOs, we performed a sequence analysis that included 185 proteins corresponding to genes included in our phylogeny. We show that the AGO4/6/8/9 and AGO1/10 clades show the most conserved linear organization of 50 identified amino acid sequences (named blocks), whereas this linear organization is more variable in members of the AGO2/3/7 and AGO5 clades. In addition to components of the PIWI, PAZ, and DUF1785 domains, we identified several blocks of unknown function that show a predictable position within a conserved primary structure and are exclusive of proteins from a specific clade. Our analysis also revealed specific block series that are conserved between flowering plants and animal organisms, including mammals, insects, nematodes, and fungi. In particular, we identified blocks containing specific motifs involved in posttranslational modifications of AGO2 and AUBERGINE in humans and *Drosophila*, respectively. Our overall results expand the phylogenetic understanding of AGO proteins in plants by establishing new linear patterns associated with their evolutionary relationship.

## Materials and methods

### Sequence selection and construction of the primary database

Database searching was conducted in Phytozome version 5.0 (http://www.phytozome.net) for 15 of the 23 genomes included in this study, using the keywords *ARGONAUTE* and *PIWI* in the biomart tool. BLAST searches, using the *Arabidopsis lyrata AGO* genes as query, were conducted for the *A. thaliana, O. sativa*, and *Zea mays* genomes, under default parameters in TAIR v.9 (http://www.arabidopsis.org/), TIGR (http://blast.jcvi.org/euk-blast/index.cgi?project=osa1), and Maizesequence.org (http://maizesequence.org/index.html), respectively. Additional *AGO* sequences were obtained by assembling ESTs coming from *Pinus taeda, Pinus radiata, Lactuca sativa, Citrus sinensis, and Amborella trichopoda* from the KEGG database and added to the sequences used for the subsequent analysis. Nucleotide sequences (coding sequence) were gathered from all selected genomes (Supplementary File 1). To confirm that the selected sequences belong to the AGO family, the coding region was translated into amino acid sequence using Seaview (Gouy et al., 2010) and used to perform a domain annotation using the batch search tool in Pfam protein database (Finn et al., 2010; Supplementary File 2), discarding the sequences that did not contain the canonical domains reported for AGO proteins (PAZ and PIWI), likely due to misannotations in the original databases.

### Sequence edition and phylogenetic reconstruction

Nucleotide sequences were edited to extract conserved domains, and used to construct phylogenetic trees under both Bayesian inference (BI) and maximum likelihood (ML) frameworks. Amino acid guided alignments were generated using MUSCLE in SeaView (Gouy et al., 2010). Because many inter-domain regions showed inconsistencies such as long-branch attraction and obvious misannotations resulting in misplaced taxon outgroups, a *perl* script (Supplementary File 3) was designed to extract the DNA sequence encoding the domains DUF1785 (recently renamed as Argonaute linker 1 domain), PAZ and PIWI from the total sequences, using the coordinates found in the Pfam batch search output. This resulted in an elimination of inter-domain regions, reducing the total length of analyzed sequences (Supplementary File 4).

Multiple sequence alignments were generated using Muscle (Edgar, 2004) by translating DNA into amino acid sequence before performing the alignment and then untranslating the sequences to maintain the coding frame at the DNA level using SeaView. Following alignment verification and editing between species, intra-domain regions showing obvious misalignments were trimmed using Gblocks (Talavera and Castresana, 2007; Supplementary File 5). For each phylogenetic reconstruction by BI and ML, the evolutionary model was chosen using *Modelgenerator* (Keane et al., 2006), resulting in the selection of the General Time Reversible (GTR) model, including the proportion of invariable sites and the gamma distribution into four categories (*GTR*+*I*+*G*). The ML analysis was conducted using *PhyML* version 3 (Guindon et al., 2009); nucleotide frequencies, alpha value, and proportion of invariable sites were fixed according to the values obtained from *Modelgenerato*r, and the rest of parameters were optimized in the ML framework. Statistical support for the analysis was obtained by performing 2000 bootstrap replicates. Bayesian analyses were conducted using MrBayes 3.1 (http://mrbayes.csit.fsu.edu/; Ronquist and Huelsenbeck, 2003) as follows: two independent runs were performed for 10 million generations with eight chains (two cold and six heated), using fixed priors and sampling frequency of each 100 generations, and discarding 25% as burn-in and computing a majority-rule consensus of the trees sampled during the run. *Chlamydomonas reinhardtii AGO* sequences were selected as the outgroup. To support the the Bayesian inference search and ensure accurate tree sampling, the trace files generated by MrBayes were analyzed by Tracer v1.6 (Rambaut and Drummond, 2007), generating estimates, density and analysis of variance values (Supplementary Figure 1). An independent BI analysis was conducted, in which sequences from Bryophytes (*Sellaginella moellendorffii* and *Physcomitrella patens*) and Gymnosperms (*P. taeda*) were excluded, using the same parameters as described above (Supplementary Figures 1, 2). Both ML and BI trees were visualized and edited in FigTree (http://tree.bio.ed.ac.uk/software/figtree/).

### Identification of conserved amino acid sequence blocks and comparison with AGOs from other organisms

The MEME-suite (Bailey et al., 2009) was used to search for relatively short sequences of conserved amino acids (named blocks) among plant AGOs, and subsequently compare their patterns of distribution to AGOs from other non-plant organisms. Selected full-length coding sequences of plant *AGOs* were translated into amino acid sequences and searched for conserved sequence blocks using MEME in two independent runs (maximum number of motifs = 50, minimum width = 8, maximum width = 50). The motifs found after the second run were used as query to search against Genbank and Swissprot to look for their distribution in both databases using MAST tool. To generate a “Block Consensus Sequence” within each clade, all proteins were carefully analyzed to identify the sequence of blocks represented in at least 90% of all members within each clade. Blocks exclusively present in at least one member of a clade but not in any member of a different clade were named “Clade-Specific Blocks.” On the basis of the block consensus sequences for all four clades and the MAST results, we created the “Viridiplantae Consensus” by selecting all blocks present in at least two out four of the clades. The Viridiplantae consensus was compared to the representation of a random selection of 10 AGO proteins belonging to mammals, insects, nematodes, and fungi, to identify equivalent sequence blocks. The MEME block in LOGOs format and MAST-suite HTML output files are available upon request.

## Results

### Phylogenetic relationships within the plant ARGONAUTE gene family

**Figure 1:**
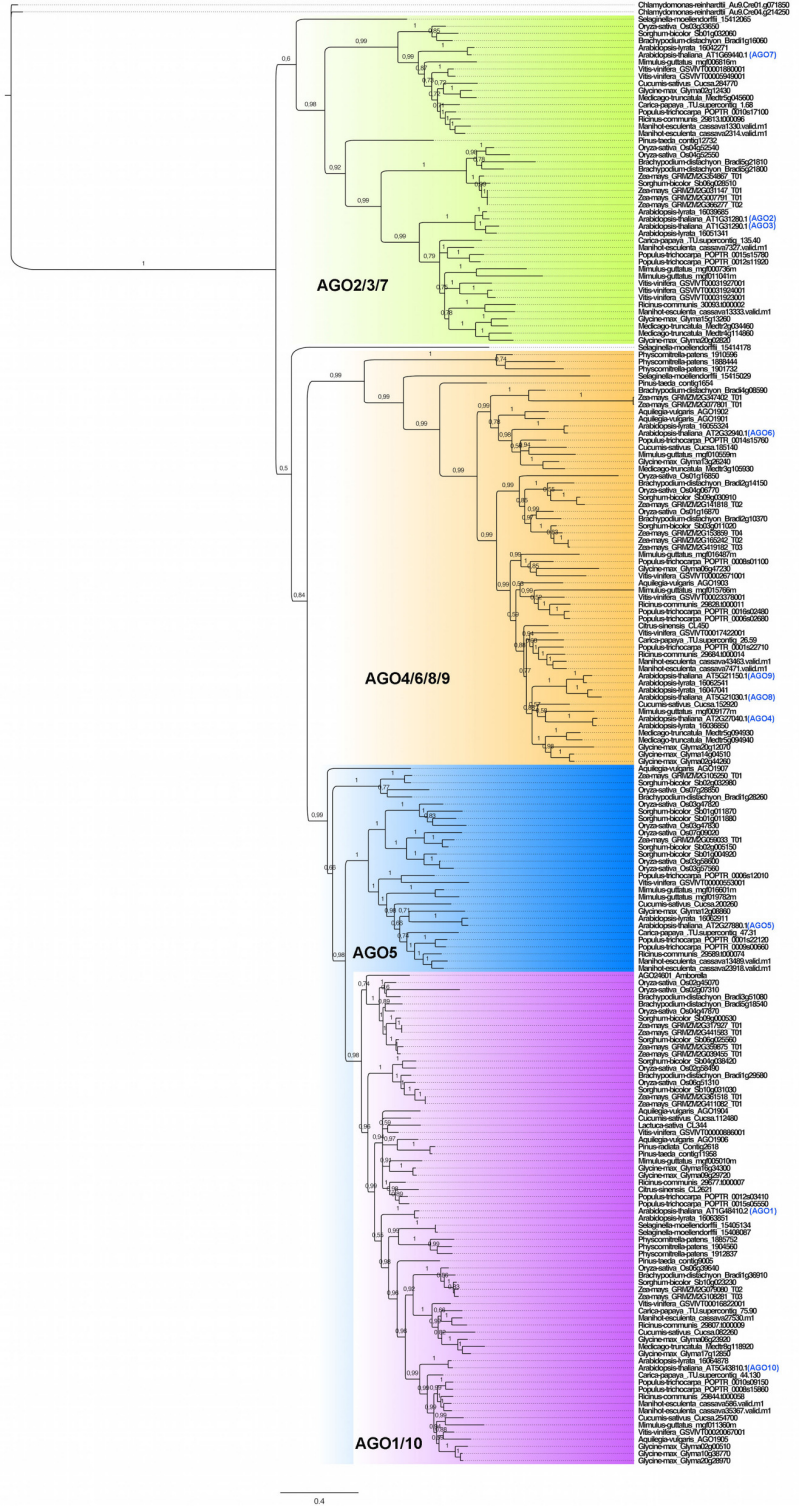
**Bayesian-derived phylogenetic structure of 206 *AGO* genes contained in 23 plant genomes**. The *AGO2/3/7* clade is highlighted in green; the *AGO4/6/8/9* clade is highlighted in orange; the *AGO5* clade is highlighted in blue; the *AGO1/10* clade is highlighted in lavender. The nomenclature of the *Arabidopsis thaliana* AGO proteins is indicated in blue. Posterior probability values are shown in the branches of the tree.

Previous analyses showed that the plant *AGO* family is monophyletic (Cerutti and Casas-Mollano, 2006; Kapoor et al., 2008; Murphy et al., 2008). The *AGO* phylogenetic gene tree illustrated in Figure 1 is represented by 206 sequences obtained by both Bayesian inference (BI) and maximum likelihood (ML) analyses, subdivided into four clades: *AGO2/3/7, AGO4/6/8/9, AGO5*, and *AGO1/10*, following the nomenclature of the ten *AGO* genes of Arabidopsis. In comparison to Kapoor et al. (2008), our phylogenetic analysis grouped *AGO4/6/8/9* closer to the *AGO5-AGO1/10* clade. In both BI and ML, the clade distribution was equivalent, supporting the robustness of the analysis (Supplementary Figure 1), and patterns of *AGO* distribution reflecting the Angiosperm phylogeny divided in the two major groups of monocots and dicots. Little changes in topology or statistical values were observed when sequences from Bryophytes (*P. patens* and *Selaginella moellendorffii*), Gymnosperms (*P. taeda* and *P. radiata*) and genomes with an incomplete set of genes (*L. sativa, C. sinensis*, and *A. trichopoda***)** were excluded, suggesting that our topology is robust and that evolutionary relationships are well defined in spite of a over-representation of Angiosperm lineages (Supplementary Figure 2). Remarkably, almost all clades exhibited the presence of paralogs coming from *S. moellendorffii* and *P. patens*, suggesting that this clade organization pre-dates the emergence of vascular plants (Gymnosperms and Angiosperms).

The number of *AGO* genes per genome ranged from 2 in *C. reinhardtii* to 20 predicted for *Z. mays* (maize), reflecting the great diversity of the family size. The genome of other species such as *Carica papaya* and *Cucumis sativus* exhibited reduced copy number in several clades of the tree, which is correlated with an absence of recent genome duplications (Ming et al., 2008; Huang et al., 2009), in contrast with those of monocots and some dicots such as Arabidopsis, *Glycine max*, and *Vitis vinifera* that experienced extensive genome duplications (Van de Peer et al., 2009). Additionally, it was common to find in different groups multiple paralogs for most of the *AGO* genes from Arabidopsis, suggesting that there is a trend for increasing gene number in Angiosperms. In almost all clades, monocots exhibited a considerable number of paralogs for almost all *AGO* genes from Arabidopsis, indicating that lineage-specific gene duplications have occurred during evolution. Following the same trend, previous genomic analysis pointed to the presence of a genome triplication in *V. vinifera* (grapevine), and gene pairs such as *AGO2-AGO3* and *AGO8-AGO9* seem to be the result of lineage-specific gene duplication within the Arabidopsis genus, contrary to *C. papaya* in which this type of duplications are absent. Due to the lack of sequences from other closely related species, we are not able to discard the possibility of a wider, older event of gene duplication within the *Brassicaceae*. In contrast, given the distribution of the phylogeny with regard to sequences from *Pinus*, the duplication that gave rise to the *AGO1* and *AGO10* lineages seems to pre-date the emergence of vascular plants. Finally, there are certain lineages, such as the one represented by maize and sorghum (*Sorghum bicolor*), but also in poplar (*P. trichocarpa*), in which several *AGO1* or *AGO10*-like paralogs are present, mostly likely coming from duplications that occurred after the divergence between monocots and dicots. In summary, our phylogenetic results indicate that clade distribution of *AGO* family pre-dates the emergence of Angiosperms, and that there is a lineage-specific evolution of the different clades of the family mediated by lineage- and species-specific gene duplications, suggesting that one-to-one functional analogies among AGO proteins might not be predictable on the basis of protein sequence, due to significant diversification within the *AGO* gene family.

### ARGONAUTE protein clades show a specific distribution of amino acid blocks

**Figure 2:**
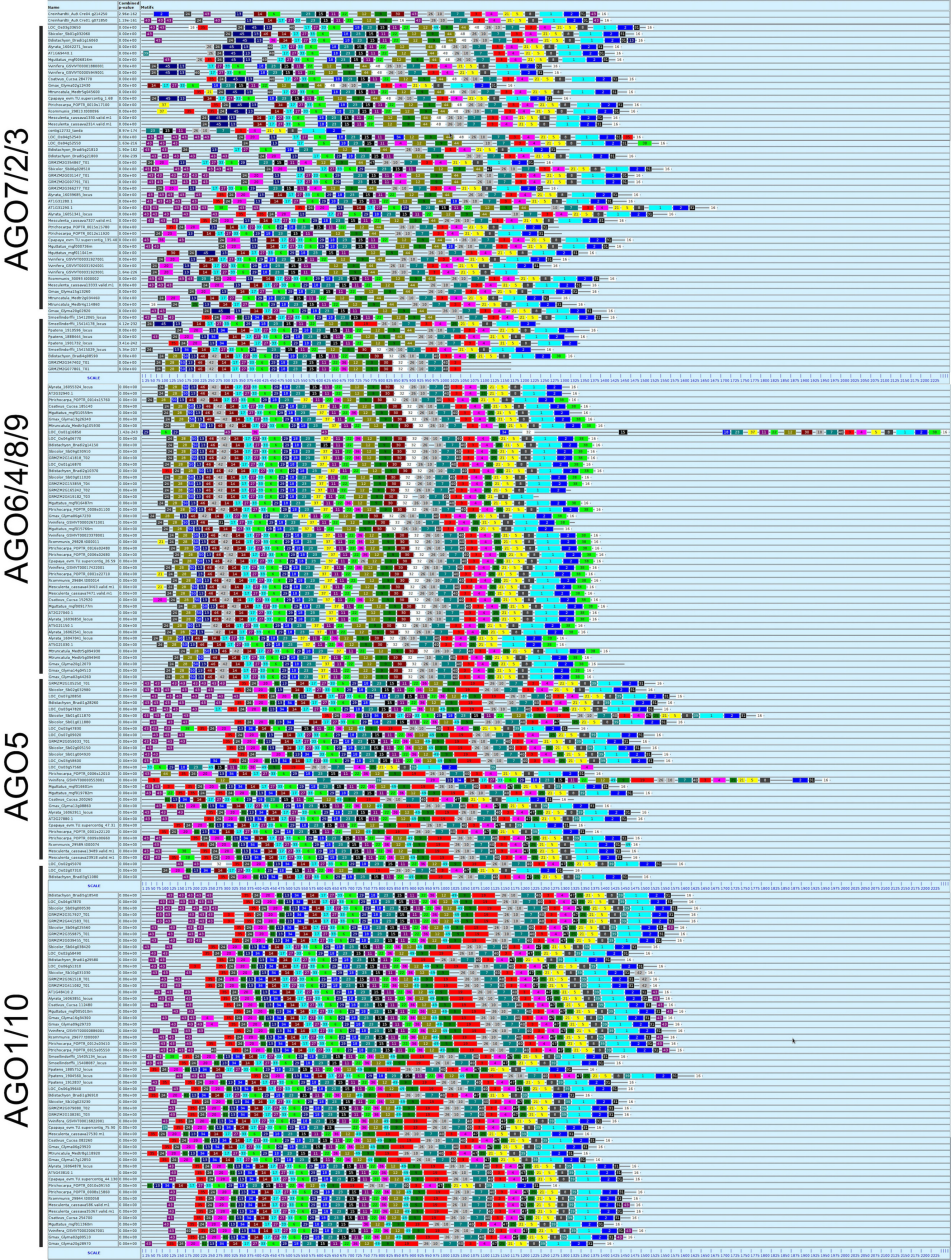
**Primary analysis of 185 AGO proteins in plants**. The different plant AGO clades showed particular distribution of sequence blocks. A black line below the name of each clade delimits the members of each clade; numbers correspond to blocks depicted in Table 1.

After discarding 21 sequences corresponding to AGO proteins from Bryophytes, Gymnosperms, or corresponding to incomplete sequences from a few species of flowering plants (*L. sativa* and *Citrus* sp.), a total of 185 sequences were used to generate a MEME-driven search for short but conserved amino acid sequences (named blocks), resulting in a dynamic distribution within AGO proteins that is correlated with the previously described phylogenetic architecture (Table 1 and Figure 1). The analysis yielded 50 sequence blocks with a variable length ranging between eight (block 47) and 50 (block 1) amino acids (Table 1 and Figure 2). For all clades of the phylogeny, the PIWI domain, responsible for endonuclease cleavage during AGO interaction with sRNAs, is represented by amino acid components present in blocks 1 to 5, 7, 8, 21, 25, 26, 31, 39, 40, 42, and 48; whereas the PAZ domain, responsible for anchoring sRNA during interaction with target transcripts, is represented by components present in blocks 11, 15, 18, 22, 23, 29, and 37 (Table 1). The catalytic residues DDH/DDD, previously described as included in the PIWI domain, are embedded within sequence block 5 (DGVSEGQFYQVLNYELDAIRKACA), 3 (PTIIFGMDVTHPHPGEDSSPSIAAV), and 2 (ELQTLTNNLCYTYARCTRSVSIVPPAYYAHLAA), as expected for essential components of the PIWI-dependent RNA silencing machinery (Table 1). Our analysis also identified components of the DUF1785 domain present in all AGO proteins in blocks 6, 27, and 33.

**Table 1:** **Consensus sequence blocks of plant ARGONAUTE proteins**.

**Block^a^**	**Length^b^**	**Consensus sequence^c^**	**Domain Component^d^**
1	50	*GNIPPGTVVDTKICHPTEFDFYLCSHAGMQGTSRPTHYHVLWDENNFTAD*	PIWI
2	33	*ELQTLTNNLCYTYARCTRSVSIVPPAYYAHLAA*	PIWI
3	25	*PTIIFGMDVTHPHPGEDSSPSIAAV*	PIWI
4	26	*DWPEVTKYAALVCAQAHRQEMIQDLF*	PIWI
5	24	*DGVSEGQFYQVLNYELDAIRKACA*	PIWI
6	27	*GFYQSFRPTQMGLSLNIDMSTTMFIEP*	DUF1785
7	29	*KMNDQYLANVALKINAKMGGRNTVLVDAL*	PIWI
8	21	*NWQPPVTFIVVQKRHHTRLFP*	PIWI
9	27	*PQNGQWNMMNKKMVNGGTVERWACINF*	–
10	18	*YGDWKWICETDLGIVTQC*	PIWI
11	21	*RPNYLPMELCKIVEGQRYTKR*	PAZ
12	29	*YAKEFGISISEKMTQVEARVLPAPWLKYH*	–
13	15	*AYDGRKSLYTAGPLP*	–
14	27	*FKVEIKFAAKADMHHLAQFLAGRQADA*	–
15	21	*VEYFWEMYGYTIQHTHWPCLQ*	PAZ
16	15	*LPALHENVKNVMFYC*	–
17	15	*QEALQVLDIVLREHP*	–
18	17	*DWVKIKKALKNVKVEVT*	PAZ
19	50	*NVQESVARGFCHELAQMCQISGMEFNPEPVIPIYSARPDQVEKALKHVYH*	–
20	29	*PDKDLHHYDVTITPEVTSRGVNRAIMAEL*	–
21	15	*RKATGQKPQRIIFYR*	PIWI
22	16	*QITALLKMTCQRPQER*	PAZ
23	29	*NMNQKYRITGLTEQPCRELWFPMDDKNTM*	PAZ
24	15	*GTKCILKANHFFVEF*	–
25	15	*GTVDGGMIRELLISF*	PIWI
26	15	*ELQLLICILPDNNGS*	PIWI
27	15	*WRCCPVGRSFFSPDM*	DUF1785
28	29	*FYHYSVALKYEDGRPVDGKGIGRKVIDKV*	–
29	15	*LPVIDFVAQNLNKDD*	PAZ
30	28	*SARCDVRHLVRDLIKCGMMKGIMIEPPF*	–
31	11	*FRARFYMEPEM*	PIWI
32	29	*FEENPQFRRAPPMVRVEKMFEQIQSKLPG*	–
33	11	*QDLGGGVEGWR*	DUF1785
34	21	*FTWKEFEITLVDEDDGTGGPR*	–
35	21	*QPPPASSKSLRFPLRPGFGTV*	–
36	15	*DILQTVHHNAYHQDP*	–
37	21	*YFVNHRNIELRYSGDLPCINV*	PAZ
38	29	*AQMGQFMKFEDMSETSSSHGGHTSAGAVP*	–
39	11	*NHNDRNSTDKS*	PIWI
40	8	*RIPLVSDI*	PIWI
41	11	*YRESHLGMRLP*	–
42	21	*GNGSPNESDRKRMRRPYQSKT*	PIWI
43	15	*GGGRGGGGGGRGGGG*	–
44	21	*FINQLIQRCCQLGIFMNKNTW*	–
45	41	*SSQRIFHYNVEISPNPSKEVARMIKQKLVEENSAVLSGALP*	–
46	21	*QNKMEFTVVLEDVSSNRNNGN*	–
47	8	*KTWQDPQR*	PIWI
48	29	*SPQFEPTQVLNNVSLLESKLKKIHKAASN*	–
49	8	*DTGREKDC*	–
50	11	*TYDSELAGKDF*	–

a *Block numbers correspond to those shown in Figure 1*.

b *Block length is given as the total number of amino acids*.

c *The catalytic residues of the PIWI domain are shown in red and underlined*.

d *Only blocks containing components of DUF, PAZ, and PIWI domains are indicated*.

With the exception of AGO proteins from the moss *P. patens* and the lycophyte *S. moellendorffii* that show a unique distribution and sequence of blocks, all four clades show a generally conserved trend of linear organization (Figure 2). In addition to the PIWI, PAZ, and DUF1785 domains, we found two new series of conserved amino acid blocks that were named domain A (comprising a variables series of blocks 13, 14, 17, 20, 24, 28, 34, and 35) and domain B (comprising a variable series of blocks 9, 12, 19, 30, 32, 36, 44, and 49). These domains are described in Table 2. The AGO1/10 clade shows the most conserved sequence of blocks of all four clades, with a linear series of four domains that includes A-1 (block sequence: 24-13-17), DUF1785-1 (block sequence: 27-6), PAZ-1 (block sequence: 18-23-15-11), B-1 (block sequence 12-9-44), and the PIWI-1 domain (block sequence: 26-10-7-3-4-21-58-1-2) that incorporated block 31 as specificity of proteins from this clade (Figure 2 and Table 2). Although, no clade-specific blocks were identified in members of the AGO1/10 clade, Figure 2 shows a highly variable number of copies of block 43 (GGGRGGGGGGRGGGG) at the N-terminal region of numerous protein members.

**Table 2:** **Consensus domain sequence distribution in four clades of plant ARGONAUTE proteins**.

**Clade^a^**	**Consensus domain sequence^b^**	**Specific blocks^c^**
AGO2/3/7 (44)	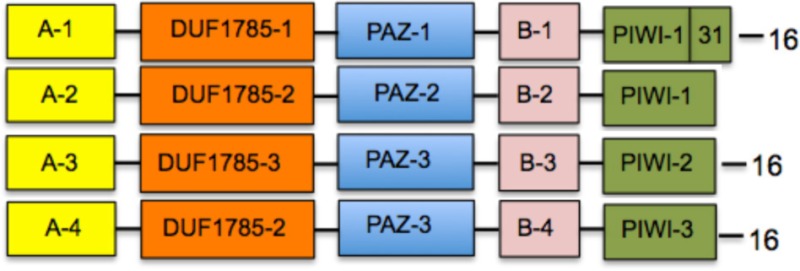	45, 48
AGO4/6/8/9 (55)		28, 30, 32, 37, 42, 46, 50
AGO5 (28)		none
AGO1/10 (58)		none
A-1: 24-13-17	DUF1785-1: 27-6	PIWI-1: 26-10-7-3-4-21-5-8-1-2
A-2: 24-28^*^-13-14-17	DUF1785-2: 27-33-6	PIWI-2: 26-10-7-40-3-4-21-5-8-1-2-31
A-3: 35-24-20-13-14-17	DUF1785-3: 33-6	PIWI-3: 26-10-7-40-3-4-47-21-5-39-1-2-3
A-4: 35-24-20-41-13-34-14-17		
B-1: 12-9-44	PAZ-1: 18-23-15-11	
B-2: 12-9-30^*^-32^*^	PAZ-2: 29-18-15-12-22	
B-3: 12-9-19	PAZ-3: 29-18-23-15-11-22	
B-4: 36-12-49-9-19		

a *Clades follow the Arabidopsis thaliana nomenclature; numbers between brackets indicate the number of proteins included in each clade*.

b *Asterisks indicate blocks present in Angiosperms but not in proteins from Physcomitrella and Selaginella sp*.

c *Blocks exclusively found in at least one member of the corresponding clade, but not in other clades*.

The AGO4/6/8/9 and AGO5 clades also show a highly conserved series of blocks that are present in at least 90% of the proteins analyzed within each clade (Figure 2 and Table 2). Whereas, the AGO4/6/8/9 clade is characterized by containing the A-2 (24-28-13-14-17), DUF1785-2 (27-33-6), PAZ-2 (29-18-15-12-22), B-2 (12-9-30-32), and PIWI-1 domains, the AGO5 clade has the A-3 (35-24-20-13-14-17), DUF1785-3 (33-6), PAZ-3 (29-18-23-15-11-22), B-3 (12-9-19), and PIWI-2 domains (26-10-7-40-3-4-21-5-8-2-31). Contrary to the AGO4/6/8/9 clade, members of the AGO1/10 clade have a strong tendency to include blocks 40 (RIPLVSDI), 47 (KTWQDPQR), 39 (NHNDRNSTDKS), and 31 (FRARFYMEPEM), all components of the PIWI domain that are also partially included in the consensus of the AGO5 (blocks 40 and 31) and AGO2/3/7 (block 31) clades (Table 2). In addition, in the AGO4/6/8/9 clade we found four exceptional proteins (one from *Ricinus communis*, one from *Cocumis sativus*, one from *P. trichocarpa*, and one from *Manihot esculenta*) containing a copy of block 20 (PDKDLHHYDVTITPEVTSRGVNRA IMAEL; of unknown function) or 21 (RKATGQKPQRIIFYR; a component of the PIWI domain) at the N-terminal region (Figure 2). Additional off-types of this clade at the N-terminal domain are Bradi2g10370 from *Brachypodium dystachion* that contains block 35 (QPPPASSKSLRFPLRPGFGTV; of unknown function) in the N-terminal region, and LOC Os01gA16850 from rice that is exceptionally long and contains an overall unique sequence of block distribution, suggesting a case of sequence misannotation. By contrast to the AGO4/6/8/9 and AGO2/3/7 clades, the AGO5 clade does not contain specific blocks absent from members of all other clades.

Finally, the AGO2/3/7 clade shows a conserved sequence of blocks present in at least 90% of the proteins analyzed within the clade that includes domains A-4 (35-24-20-41-13-34-14-17), DUF1785-2 (as the AGO4/6/8/9 clade), PAZ-3 (as the AGO5 clade), B-4 (36-12-49-9-19), and PIWI-3 (26-10-7-40-3-4-47-21-5-8-39-1-2-3) as shown in Table 2. Blocks 45 (SSQRIFHYNVEISPNPSKEVARMIKQKLVEENSAVLSGALP) and 48 (SPQFEPTQVLNNVSLLESKLKKIHKAASN) that contain consensus sequences of unknown function are exclusively found in this clade, clearly showing the divergence that some primary sequence components have among members of the AGO family of proteins in plants.

Although being present in < 90% of proteins analyzed, a few additional blocks are abundantly present in members of specific clades, often in multiple copies (Figure 2 and Table 1). In the case of the AGO2/3/7 clade, these include blocks 20 (PDKDLHHYDVTITPEVTSRGVNRAIMAEL), 22 (QIT ALLKMTCQRPQER), 43 (GRGGGRGRGGR), and 45 (SSQRIFHYNVEISPN PSKEVARMIKQKLVEENSAVLSGALP). Whereas, block 20 is usually present at the N-terminal region of many proteins, block 22 represents a component of the PAZ domain that is usually located in close association of block 11 (which is also a component of PAZ; Table 1). Block 45 is a large element of unknown function found at the N-terminal region, and in close association with block 13. Of particular interest is block 43, abundantly present in up to eight copies at the N-terminal region of a multitude of proteins belonging to three clades (AGO2/3/7, AGO5, and AGO1/10), but present in members of *C. reinhardtii, P. patens*, and *S. moellendorffii*, which suggests an ancestral origin for the block (Table 1). In the case of the AGO4/6/8/9 clade, block 38 is often present in at the C-terminal region of many proteins, and in close association with components of the PIWI domain (blocks 1 and 2). In the case of the AGO5 clade, blocks 35 (QPPPASSKSLRFPLRPGFGTV) and 49 (*DTGREKDC*) are often present in a single copy at the N-terminal or mid region, respectively. Finally, in the case of the AGO1/10 clade, block 43 is also abundantly present in multiple copies at the N-terminal region, and additionally, only in some proteins belonging to highly distinct families of flowering plants (*Brassicaceae, Cucurbitaceae, Fabaceae*, and *Phrymaceae*), also present as a single copy located at the C-terminal region, and in close association with components of the PIWI domain.

### Consensus block sequences are conserved between plant and non-plant organisms

After determining the distribution of linear blocks within the plant clades, we looked for the possible presence of similar or identical primary sequence blocks in other AGO proteins belonging to non-plant organisms. Using the motif alignment and search tool (MAST) of the MEME suite, we searched protein databases such as NCBI non-redundant protein database and SWISSPROT, comparing the sequences in terms of presence or absence of specific blocks, and the organization of their linear arrangement. We performed six independent MAST runs using as input all sequence blocks, changing the parameters from standard to stringent values by modifying the sequence composition thresholds, as well as the *E*- and *P*-values. We found that specific blocks defined domains that were conserved between plants, animals, and fungi (Table 3), supporting the robustness of our analysis and extending the functional conservation of domains across kingdoms. For example, the mammalian consensus block sequence contains domains A-3, DUF1785-1, and B-3 that are also contained in the plant consensus sequence (Table 3); it also contains the PAZ-3 domain in conjunction to block 4 that correspond to a component of the PIWI domain (Table 3), and the PIWI-1 domain supplemented with block 31 (Table 3). The insect consensus block sequence also contains the DUF1785-1 and PAZ-3 domains, but is characterized by different variants of the A, B, and PIWI domains. By contrast, nematodes and fungi are characterized by consensus sequences that largely diverge from the plant consensus sequence, although in general terms, only the insertion of a few individual blocks distinguished the flowering plant consensus from these highly divergent non-plant groups of organisms. Interestingly, the consensus of the PIWI subfamily of AGO proteins was more divergent than the rest of the consensus (Tables 2, 3), suggesting that PIWI subgroup has considerably diverged as compared to the rest of the AGO proteins.

**Table 3:** **Consensus domain sequences among ARGONAUTE proteins from different group of organisms**.

**Organism group**	**Consensus domain sequence**^a^	
Viridiplantae	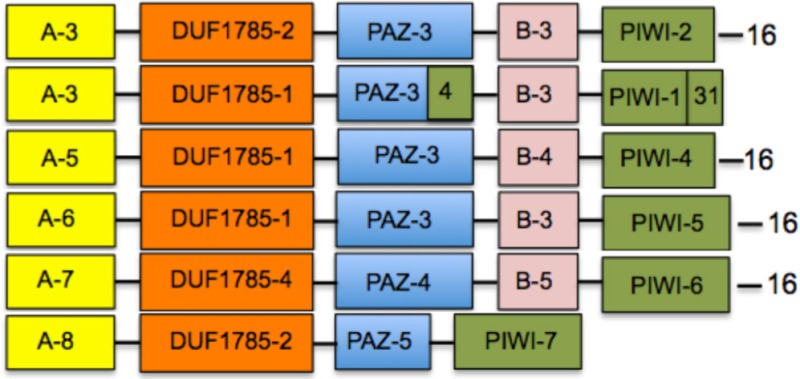
Mammals
Insects
Nematodes
Fungi
PIWI subfamily
A-5: 20-13-14-17	DUF1785-4: 27-33	PIWI-4: 26-10-7-3-4-21-5-8-1-2-31
A-6: 24-20-13-14-17		PIWI-5: 10-7-3-4-21-5-8-1-2-31
A-7: 43-35-24-13-14-23-17	PAZ-4: 18-11-22	PIWI-6: 7-3-4-21-5-8-1-2-31
A-8: 43-24-20	PAZ-5: 15-12	PIWI-7: 10-4-21-5-8-2-1
B-5: 12-9		

a *The nomenclature of domains is equivalent to Table 2*.

### Specific functional motifs are conserved between animal and plant ago proteins

**Figure 3:**
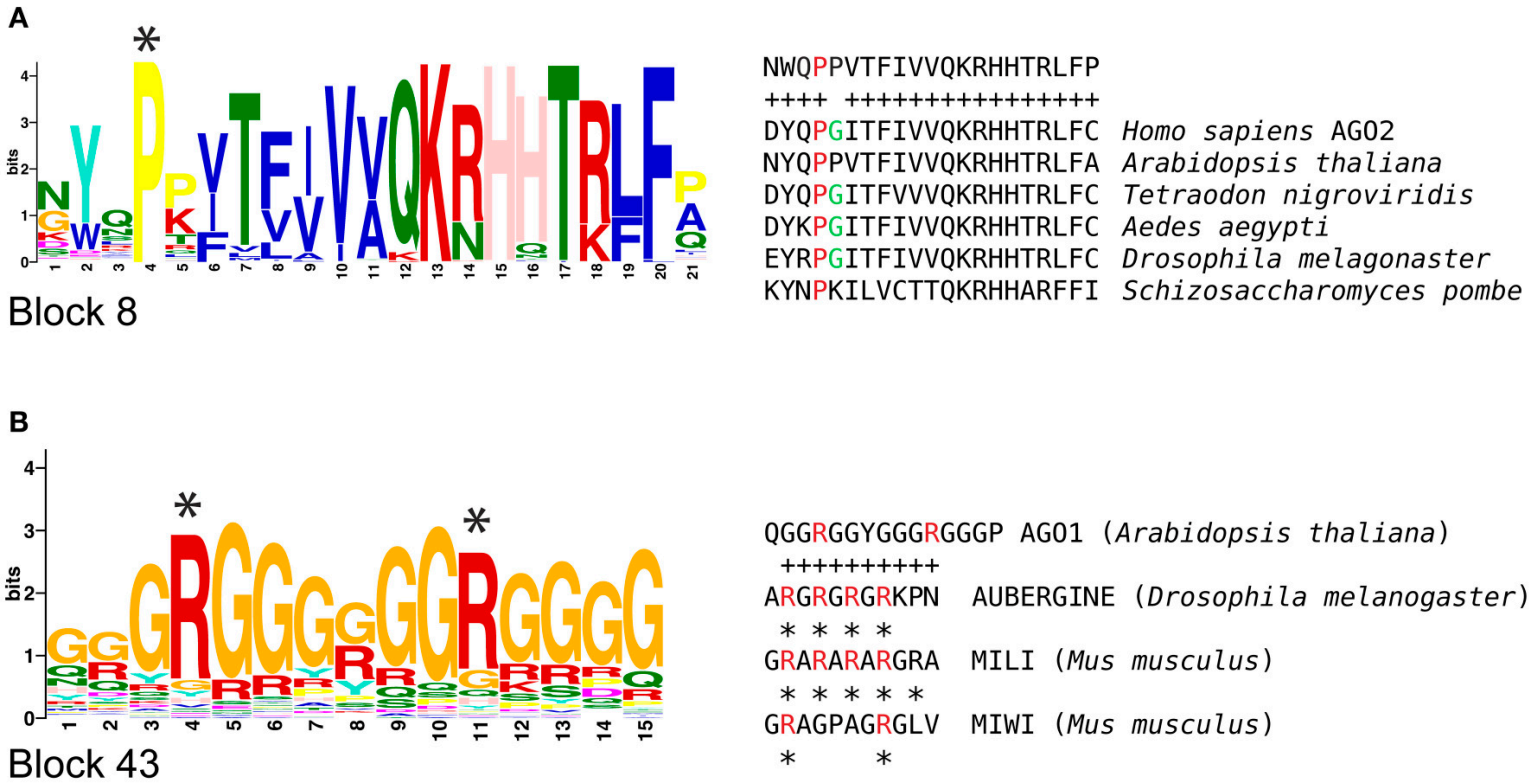
**Animal functional motifs found in sequence blocks of plant AGOs. (A)** A proline residue that is hydroxylated in human AGO2 is highly conserved in block 8 of AGO proteins from several plant organisms, including Arabidopsis. **(B)** A motif that contains arginine residues that are methylated in PIWI proteins is similar to the glycine-arginine enriched sequence found in block 43; asterisks indicate arginine residues that are targets of methylation.

We also analyzed plant AGOs in search for motifs previously reported for animal AGO proteins, and found two types of sequences that could be related to similar or equivalent functions across kingdoms. Qi et al. (2008) reported that human AGO2 is post-translationally modified to confer stability and allow protein-protein interactions with members of the RNA interference silencing complex (RISC). This posttranslational modification depends on the proline residue of a *X-P-G* motif that is recognized and hydroxylated by a collagen prolyl-4-hydroxylase. Mutations that suppress this modification result in AGO2 instability and defective RISC activity. We found that the proline residue of the *X-P-G* motif is present in all AGO proteins containing block 8 (Figure 3A). In plants, the proline and glycine residues were highly conserved in all phylogenetic groups, suggesting that it might be a site for posttranslational modifications. Recent evidence supports the presence and function of prolyl-hydroxylases in Arabidopsis, supporting the possibility that this type of modifications could occur in plant AGO proteins (Tiainen et al., 2005; Vlad et al., 2007; Asif et al., 2009; Velasquez et al., 2015).

Our analysis also revealed that block 43 (GRGGGRGRGGR) is abundantly present in a variable number of consecutive copies at the N-terminal region of members of the AGO2/3/7, AGO5, and AGO1/10 clades, but completely absent from the AGO4/6/8/9 clade (Figure 2). This conservation appears to be related to mechanisms such as those regulating posttranslational modification in AUBERGINE (AUB), a PIWI protein of *Drosophila melanogaster* that is modified by a symmetrical arginine methylation in an amino acid motif rich in glycine and arginine residues. This modification allows AUB to interact with proteins of the TUDOR (TUD) family (Kirino et al., 2009, 2010; Siomi et al., 2010). Based on our MAST analysis, we found a close association between sequence block 43 (GRGGGRGRGGR), and the proposed region to be modified in AUB and MIWI (Siomi et al., 2010; Figure 3B), raising the possibility that the sequence corresponding to block 43 could function as an evolutionary conserved protein-protein interaction and localization signal in plant AGOs. These results suggest that non-canonical functional motifs involved AGO protein function might be shared across kingdoms, opening possibilities for new experimental assays.

## Discussion

Our phylogenetic analysis based on 23 plant genomes defined that the *AGO* gene family is divided into four major clades that recapitulate the relationships among Arabidopsis *AGO* genes. Although previous studies already classified *AGO* into these clades, there are specific differences in the resulting clade distribution and statistical support of our phylogeny. In contrast to previous reports (Cerutti and Casas-Mollano, 2006; Kapoor et al., 2008; Murphy et al., 2008), our phylogeny placed *AGO2/3/7* as a distinct sister clade to the common branch that includes all other three clades, although with moderate to weak statistical support (up to 60% and 0.9 in bootstrap and posterior probability). In our analysis, the ML and BI statistical frameworks allowed the establishment of phylogenetic relationships that use nucleotide sequences as markers of phylogenetic reconstructions. Although, we cannot rule out the influence of several parameters such as out-group selection, taxon sampling, or molecular edition in the outcome of the topology or the statistical support of the phylogenetic tree, a recent analysis based on a larger number of plant and animal genomes that included 19 of the 23 plant genomes included here resulted in a closely related but less detailed topology (Singh et al., 2015), providing independent support to our analysis.

While our phylogeny is mostly composed of genes from flowering plants, sequences from *P. patens* and *S. moellendorffii* also clustered in at least one of the four major *AGO* clades, suggesting that the divergence of these clades could precede the divergence of Gymnosperms and Angiosperms. Although, there is little functional information for the *AGO* genes of *C. reinhardtii* (Casas-Mollano et al., 2008), *P. patens*, or *S. moellendorffii*, we hypothesize that in these ancient plant organisms the RNAi machinery is dependent on AGO proteins that are capable of ubiquitously fulfill functions that in Angiosperms are executed separately by different AGOs from the four major clades (Bartel, 2004; Bonnet et al., 2006; Casas-Mollano et al., 2008; Cho et al., 2008). According to the hypothesis of the origins of sRNAs in eukaryotic evolution (Shabalina and Koonin, 2008) we propose that even in *C. reinhardtii, AGO* genes are functional and participate in basic processes such as transposon silencing and possibly gene regulation through a pathway reminiscent of the miRNA-dependent pathway of flowering plants (Molnar et al., 2007).

The evolutionary trend observed in AGO proteins could be associated with the diversification and function of key developmental processes in flowering plants (Cibrian-Jaramillo and Martienssen, 2009). The AGO4/6/8/9 clade, which in our analysis shows a high level of block conservation and possibly functional specialization, is directly involved in epigenetic silencing of heterochromatin, including transposons, retrotransposons, and other repetitive elements (Matzke et al., 2015). Specialization of this specific clade could have involved the acquisition of restricted temporal or spatial patterns of protein expression (Havecker et al., 2010; Olmedo-Monfil et al., 2010), recent genomic duplications (Takeda et al., 2008), or sRNA interaction and regulation through *de novo* DNA methylation. Although several RNA-DEPENDENT RNA POLYMERASE (RDR) proteins have also been implicated in these mechanisms, a possible phylogenetic relationship between AGOs and RDRs has not yet been investigated, even if a genome phylogeny that used measures of congruence suggested that AGO1 and RDR6 are of critical importance in the evolution of seed plants (Cibrian-Jaramillo et al., 2010). The presence of some consensus sequences corresponding to blocks that are elements of the PIWI and PAZ domains suggest that the clade might include canonical components that are not functionally represented in any of the three other clades. In addition to these blocks, several other newly defined domains such as A-2 and B-2 of unknown tertiary structure or function are exclusive of proteins either included in the AGO2/3/7 or AGO4/6/8/9 clades, suggesting that some of corresponding sequences might represent a clade-specific degree of specialization with possible structural or biochemical functions. Their identification could serve as the basis for subsequent experimental analysis.

The AGO1/10 members also have a tightly conserved primary sequence, probably related to their almost exclusive association with microRNAs, being AGO1 the best characterized AGO protein to this date (Kidner and Martienssen, 2005; Vaucheret, 2008). The AGO2/3/7 clade has been implicated in *tasiRNA*-dependent posttranscriptional regulation, particularly during leaf development (Montgomery et al., 2008; Chitwood et al., 2009); the function of members of this clade also include SHOOTLESS4 (SHL4)/SHOOT ORGANIZATION2 (SHO2) in rice (LOC_Os03g33650 in the phylogeny; Nagasaki et al., 2007), and RAGGED SEEDLING2 in maize (GRMZM2G365589 in the phylogeny; Douglas et al., 2010). In the case of the *AGO5* clade, evidence in Arabidopsis and rice indicates broad sRNA binding capacity by corresponding proteins and specific functions during gametogenesis and meiosis. In rice, *MEIOSIS ARRESTED AT LEPTOTENE* (*MEL1*) is expressed during meiosis and is important for functional megaspore development as well as both male and female gametogenesis (Nonomura et al., 2007).

The structure of the two *C. reinhardtii* AGO sequences containing the majority of blocks found in all four major clades supports the hypothesis of a monophyletic and multifunctional ancestral origin for the family. The conservation of specific blocks and motifs across the animal and plant kingdom also suggests that specific elements of unknown function could have prevailed after the divergence and specialization of AGO proteins in plants. In some cases such as the A-3, B-3, and PAZ-3 domains conserved between plants and mammals, the corresponding block elements include sequences of unknown function associated with elements included in canonical domains, opening possibilities for experimentally testing whether this primary sequence conservation could be a consequence of functional elements that were conserved after the split between plants and animals.

Functional motifs present in animal AGO proteins were identified in one or more of the conserved sequence blocks generated by our analysis, within regions subject for posttranslational modifications such as hydroxylation (Qi et al., 2008) or arginine methylation (Kirino et al., 2009, 2010). In the former case, we found a block that is highly conserved in all AGOs from different kingdoms, which points to the possibility of a functional conservation across kingdoms. In the latter, the conserved motif was described as a site for protein-protein interaction and localization signals related to the *D. melanogaster* AUB protein, suggesting a function beyond a specific AGO clade or specific developmental process due to divergent functional evolution. Our overall results provide a first analysis of AGO linear sequences, providing new regions that might conserve ancestral elements related to unforeseen functions requiring experimental assessment before being further defined or elucidated.

## Author contributions

DR and JV conceived and designed the research; AC and IR provided computational support; DR and JV analyzed the data, interpreted the results, and wrote the paper.

### Conflict of interest statement

The authors declare that the research was conducted in the absence of any commercial or financial relationships that could be construed as a potential conflict of interest. The reviewer MB and handling Editor declared their shared affiliation, and the handling Editor states that the process nevertheless met the standards of a fair and objective review.

## Supplementary material

Sequences and script files can be downloaded from: http://datos.langebio.cinvestav.mx/~vielle/Rodriguez-Leal_etal_2016_suppData/

## Abbreviations

ML: Maximum likelihood
BI: Bayesian inference
AGO: ARGONAUTE.
