# Supplementary material for: A Primary Sequence Analysis of the ARGONAUTE Protein Family in Plants
Source: Front Plant Sci. 2016 Aug 31;7:1347. doi: 10.3389/fpls.2016.01347 (PMC5007885; doi:10.3389/fpls.2016.01347)
Supplement: Supplementary file 1 [file DataSheet1.PDF]

Supplementary Material

A primary structural analysis of the ARGONAUTE protein family in plants.

Daniel Rodríguez-Leal[1,2], Amanda Castillo-Cobián[2], Isaac Rodríguez-Arévalo[1] and Jean-Philippe Vielle-Calzada[1]*.

A

B

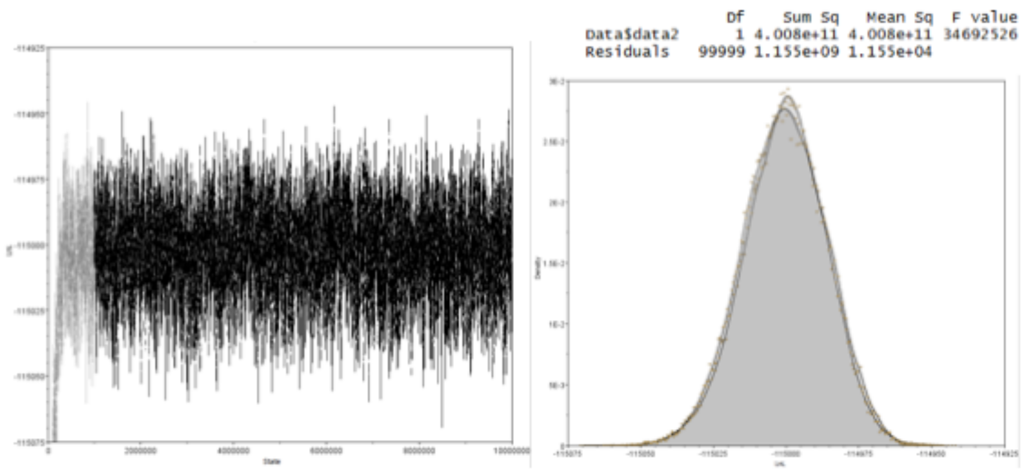

C

Summary Statistic	AGO-final_8cat.run2.p	AGO-final_8cat.run1.p
mean	-114999.9394	-115000.9367
stderr of mean	0.6031	0.5217
stdev	14.3003	14.1867
variance	204.4983	201.2631
median	-114999.685	-115000.69
mode	n/a	n/a
geometric mean	n/a	n/a
95% HPD Interval	[-115028.037, -114972.092]	[-115029.073, -114973.921]
auto-correlation time (ACT)	16007.9614	12172.7498
effective sample size (ESS)	562.2265	739.3646

D

```
                                      95% Cred. Interval
                                     ----------------------
Parameter      Mean        Variance     Lower        Upper        Median      PSRF *
-------------------------------------------------------------------------------------
TL           30.386861    8.650841    28.905000    32.918000    29.990000    1.000
-------------------------------------------------------------------------------------
* Convergence diagnostic (PSRF = Potential scale reduction factor [Gelman
  and Rubin, 1992], uncorrected) should approach 1 as runs converge. The
  values may be unreliable if you have a small number of samples. PSRF should
  only be used as a rough guide to convergence since all the assumptions
  that allow one to interpret it as a scale reduction factor are not met in
  the phylogenetic context.
```

Supplementary Figure 1. Analysis of the phylogenetic output.
(**A**) Sample correlation. (**B**) Analysis of variance. (**C**) Statistical comparison summary; it includes the value of the auto-correlation time (ACT; corresponding to the number of states in the MCMC chain that the two samples have to be from each other for them to be uncorrelated; the ACT is estimated from the samples in the trace, excluding the burn-in) and the effective sample size (EES, corresponding to the number of independent samples that the trace is equivalent to; essentially the chain length, excluding the burn-in, divided by the ACT). (**D**) Convergence diagnostic on the basis of the potential scale reduction factor (PSRF; Gelman and Rubin, 1992).

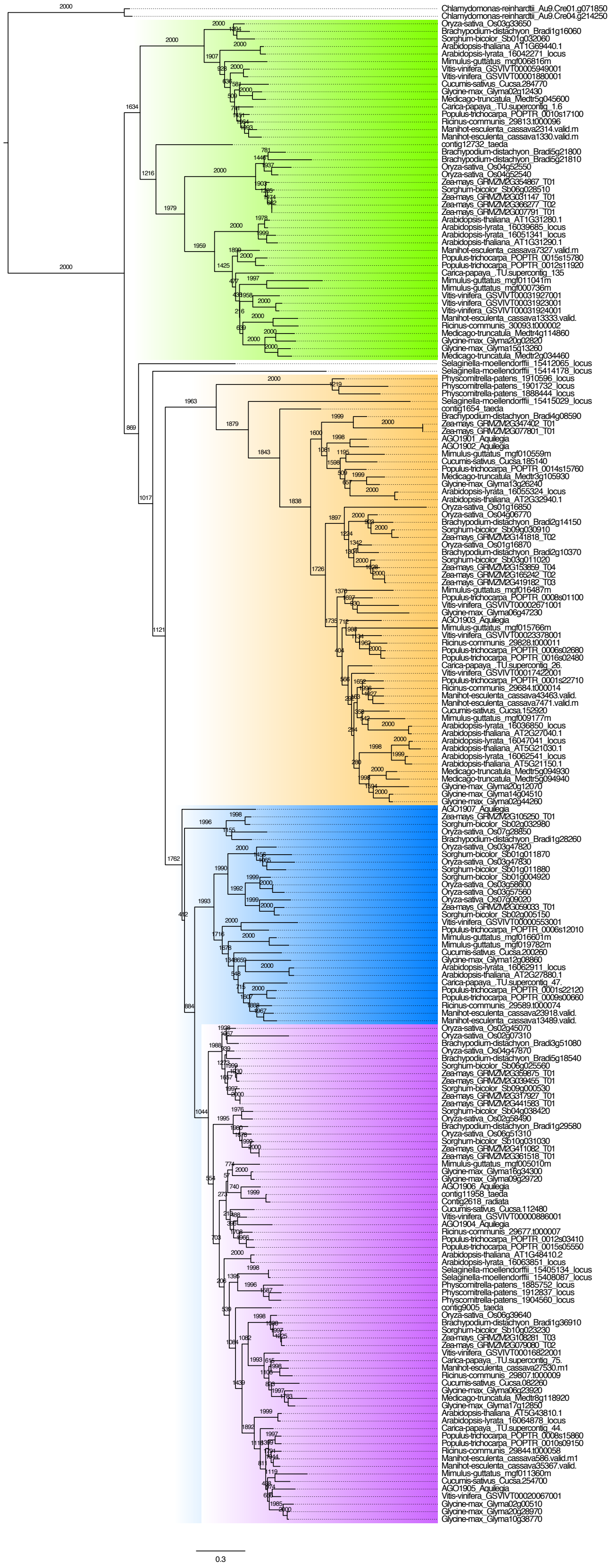
Chlamydomonas-reinhardtii_Au9.Cre01.g071850
Chlamydomonas-reinhardtii_Au9.Cre04.g214250
Oryza-sativa_Os03g33650
Brachypodium-distachyon_Bradi1g16060
Sorghum-bicolor_Sb01g032060
Arabidopsis-thaliana_AT1G69440.1
Arabidopsis-lyrata_16042271_locus
Mimulus-guttatus_mgf006816m
Vitis-vinifera_GSVIVT00005949001
Vitis-vinifera_GSVIVT00001880001
Cucumis-sativus_Cucsa.284770
Glycine-max_Glyma02g12430
Medicago-truncatula_Medtr5g045600
Carica-papaya_.TU.supercontig_1.6
Populus-trichocarpa_POPTR_0010s17100
Ricinus-communis_29813.t000096
Manihot-esculenta_cassava2314.valid.m
Manihot-esculenta_cassava1330.valid.m
contig12732_taeda
Brachypodium-distachyon_Bradi5g21800
Brachypodium-distachyon_Bradi5g21810
Oryza-sativa_Os04g52550
Oryza-sativa_Os04g52540
Zea-mays_GRMZM2G354867_T01
Sorghum-bicolor_Sb06g028510
Zea-mays_GRMZM2G031147_T01
Zea-mays_GRMZM2G366277_T02
Zea-mays_GRMZM2G007791_T01
Arabidopsis-thaliana_AT1G31280.1
Arabidopsis-lyrata_16039685_locus
Arabidopsis-lyrata_16051341_locus
Arabidopsis-thaliana_AT1G31290.1
Manihot-esculenta_cassava7327.valid.m
Populus-trichocarpa_POPTR_0015s15780
Populus-trichocarpa_POPTR_0012s11920
Carica-papaya_.TU.supercontig_135
Mimulus-guttatus_mgf011041m
Mimulus-guttatus_mgf000736m
Vitis-vinifera_GSVIVT00031927001
Vitis-vinifera_GSVIVT00031923001
Vitis-vinifera_GSVIVT00031924001
Manihot-esculenta_cassava13333.valid.
Ricinus-communis_30093.t000002
Medicago-truncatula_Medtr4g114860
Glycine-max_Glyma20g02820
Glycine-max_Glyma15g13260
Medicago-truncatula_Medtr2g034460
Selaginella-moellendorffii_15412065_locus
Selaginella-moellendorffii_15414178_locus
Physcomitrella-patens_1910596_locus
Physcomitrella-patens_1901732_locus
Physcomitrella-patens_1888444_locus
Selaginella-moellendorffii_15415029_locus
contig1654_taeda
Brachypodium-distachyon_Bradi4g08590
Zea-mays_GRMZM2G347402_T01
Zea-mays_GRMZM2G077801_T01
AGO1901_Aquilegia
AGO1902_Aquilegia
Mimulus-guttatus_mgf010559m
Cucumis-sativus_Cucsa.185140
Populus-trichocarpa_POPTR_0014s15760
Medicago-truncatula_Medtr3g105930
Glycine-max_Glyma13g26240
Arabidopsis-lyrata_16055324_locus
Arabidopsis-thaliana_AT2G32940.1
Oryza-sativa_Os01g16850
Oryza-sativa_Os04g06770
Brachypodium-distachyon_Bradi2g14150
Sorghum-bicolor_Sb09g030910
Zea-mays_GRMZM2G141818_T02
Oryza-sativa_Os01g16870
Brachypodium-distachyon_Bradi2g10370
Sorghum-bicolor_Sb03g011020
Zea-mays_GRMZM2G153859_T04
Zea-mays_GRMZM2G165242_T02
Zea-mays_GRMZM2G419182_T03
Mimulus-guttatus_mgf016487m
Populus-trichocarpa_POPTR_0008s01100
Vitis-vinifera_GSVIVT00002671001
Glycine-max_Glyma06g47230
AGO1903_Aquilegia
Mimulus-guttatus_mgf015766m
Vitis-vinifera_GSVIVT00023378001
Ricinus-communis_29828.t000011
Populus-trichocarpa_POPTR_0006s02680
Populus-trichocarpa_POPTR_0016s02480
Carica-papaya_.TU.supercontig_26.
Vitis-vinifera_GSVIVT00017422001
Populus-trichocarpa_POPTR_0001s22710
Ricinus-communis_29684.t000014
Manihot-esculenta_cassava43463.valid.
Manihot-esculenta_cassava7471.valid.m
Cucumis-sativus_Cucsa.152920
Mimulus-guttatus_mgf009177m
Arabidopsis-lyrata_16036850_locus
Arabidopsis-thaliana_AT2G27040.1
Arabidopsis-lyrata_16047041_locus
Arabidopsis-thaliana_AT5G21030.1
Arabidopsis-lyrata_16062541_locus
Arabidopsis-thaliana_AT5G21150.1
Medicago-truncatula_Medtr5g094930
Medicago-truncatula_Medtr5g094940
Glycine-max_Glyma20g12070
Glycine-max_Glyma14g04510
Glycine-max_Glyma02g44260
AGO1907_Aquilegia
Zea-mays_GRMZM2G105250_T01
Sorghum-bicolor_Sb02g032980
Oryza-sativa_Os07g28850
Brachypodium-distachyon_Bradi1g28260
Oryza-sativa_Os03g47820
Sorghum-bicolor_Sb01g011870
Oryza-sativa_Os03g47830
Sorghum-bicolor_Sb01g011880
Sorghum-bicolor_Sb01g004920
Oryza-sativa_Os03g58600
Oryza-sativa_Os03g57560
Oryza-sativa_Os07g09020
Zea-mays_GRMZM2G059033_T01
Sorghum-bicolor_Sb02g005150
Vitis-vinifera_GSVIVT00000553001
Populus-trichocarpa_POPTR_0006s12010
Mimulus-guttatus_mgf016601m
Mimulus-guttatus_mgf019782m
Cucumis-sativus_Cucsa.200260
Glycine-max_Glyma12g08860
Arabidopsis-lyrata_16062911_locus
Arabidopsis-thaliana_AT2G27880.1
Carica-papaya_.TU.supercontig_47.
Populus-trichocarpa_POPTR_0001s22120
Populus-trichocarpa_POPTR_0009s00660
Ricinus-communis_29589.t000074
Manihot-esculenta_cassava23918.valid.
Manihot-esculenta_cassava13489.valid.
Oryza-sativa_Os02g45070
Oryza-sativa_Os02g07310
Brachypodium-distachyon_Bradi3g51080
Oryza-sativa_Os04g47870
Brachypodium-distachyon_Bradi5g18540
Sorghum-bicolor_Sb06g025560
Zea-mays_GRMZM2G359875_T01
Zea-mays_GRMZM2G039455_T01
Sorghum-bicolor_Sb09g000530
Zea-mays_GRMZM2G317927_T01
Zea-mays_GRMZM2G441583_T01
Sorghum-bicolor_Sb04g038420
Oryza-sativa_Os02g58490
Brachypodium-distachyon_Bradi1g29580
Oryza-sativa_Os06g51310
Sorghum-bicolor_Sb10g031030
Zea-mays_GRMZM2G411082_T01
Zea-mays_GRMZM2G361518_T01
Mimulus-guttatus_mgf005010m
Glycine-max_Glyma16g34300
Glycine-max_Glyma09g29720
AGO1906_Aquilegia
contig11958_taeda
Contig2618_radiata
Cucumis-sativus_Cucsa.112480
Vitis-vinifera_GSVIVT00000886001
AGO1904_Aquilegia
Ricinus-communis_29677.t000007
Populus-trichocarpa_POPTR_0012s03410
Populus-trichocarpa_POPTR_0015s05550
Arabidopsis-thaliana_AT1G48410.2
Arabidopsis-lyrata_16063851_locus
Selaginella-moellendorffii_15405134_locus
Selaginella-moellendorffii_15408087_locus
Physcomitrella-patens_1885752_locus
Physcomitrella-patens_1912837_locus
Physcomitrella-patens_1904560_locus
contig9005_taeda
Oryza-sativa_Os06g39640
Brachypodium-distachyon_Bradi1g36910
Sorghum-bicolor_Sb10g023230
Zea-mays_GRMZM2G108281_T03
Zea-mays_GRMZM2G079080_T02
Vitis-vinifera_GSVIVT00016822001
Carica-papaya_.TU.supercontig_75.
Manihot-esculenta_cassava27530.m1
Ricinus-communis_29807.t000009
Cucumis-sativus_Cucsa.082260
Glycine-max_Glyma06g23920
Medicago-truncatula_Medtr8g118920
Glycine-max_Glyma17g12850
Arabidopsis-thaliana_AT5G43810.1
Arabidopsis-lyrata_16064878_locus
Carica-papaya_.TU.supercontig_44.
Populus-trichocarpa_POPTR_0008s15860
Populus-trichocarpa_POPTR_0010s09150
Ricinus-communis_29844.t000058
Manihot-esculenta_cassava586.valid.m1
Manihot-esculenta_cassava35367.valid.
Mimulus-guttatus_mgf011360m
Cucumis-sativus_Cucsa.254700
AGO1905_Aquilegia
Vitis-vinifera_GSVIVT00020067001
Glycine-max_Glyma02g00510
Glycine-max_Glyma20g28970
Glycine-max_Glyma10g38770
0.3

A

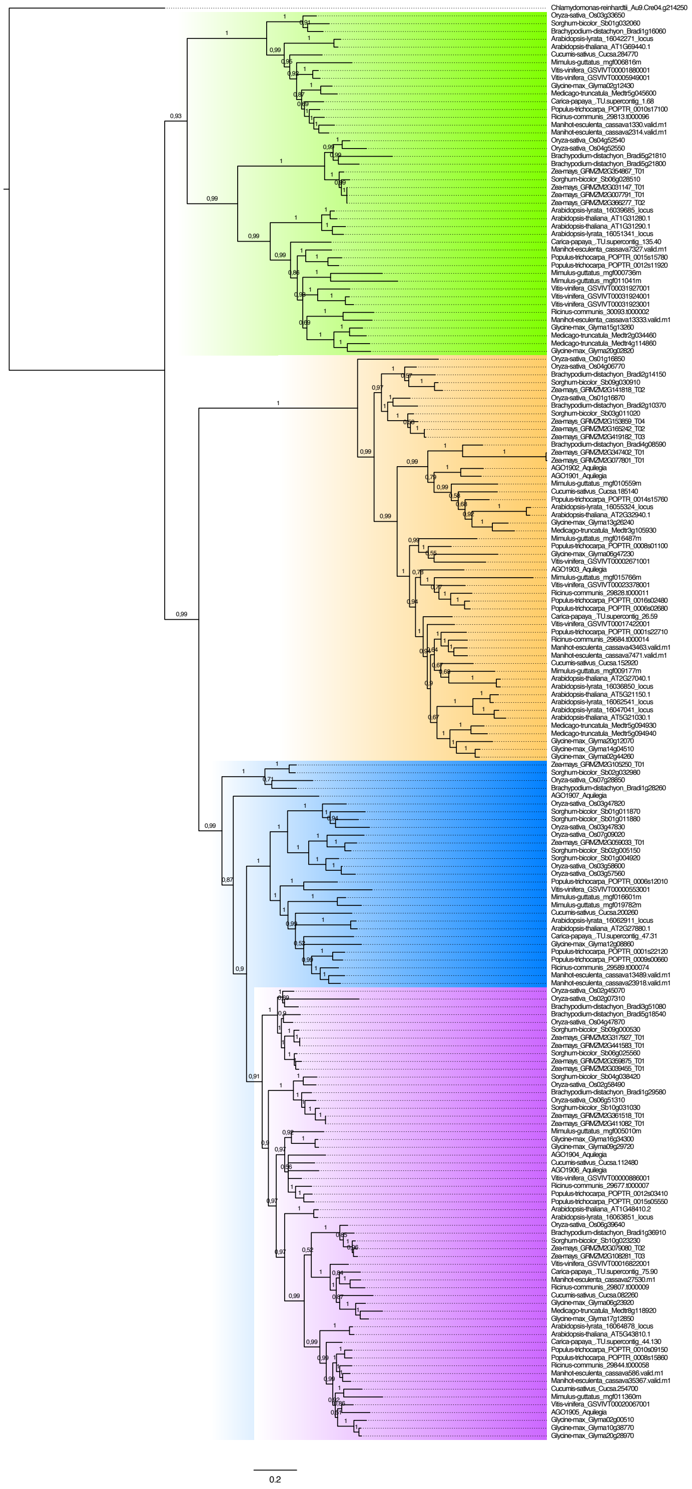

B

Supplementary Figure 2. Additional phylogenetic structures. (A) Maximum likelihood-derived phylogenetic structure of 206 *AGO* genes. (B) Bayesian-derived phylogenetic structure that includes 185 genes corresponding to protein sequences described in Figure 1.

Supplementary File 1. Coding sequences from all AGO proteins used in this study.

>Arabidopsis-thaliana_AT1G31280.1
ATGGAGAGAGGTGGTTATCGAGGAGGTCGTGGTGATGGCCGTGGTAGAGGTGGCCGTGGT
TATGGCGGAGGCGGAGGAGGAGGAGAACAAGGTCGTGATCGTGGCTACGGCGGCGGAGAA
CAAGGTCGTGGTCGTGGCTCAGAGCGAGGCGGCGGAAATCGTGGTCAAGGTCGTGGTGAA
CAACAGGATTTTCGAAGCCAGAGTCAGCGGGGACCTCCGCCAGGTCACGGTGGCCGTGGG
ACGACGCAGTTCCAACAGCCTCGACCACAGGTGGCTCCGCAGCCGTCGCAGGCTCCGGCG
AGTTATGCCGGTTCAGTAGGAGGAGTCGCTGGTAGAGGCGCGTGGGGTCGTAAGCCACAG
GTTCCGTCTGATTCGGCTTCTCCGTCCACCAGCACCACCGTGGTTTCTGAACCCGTTCGT
GTAGCTGAAGTTATGAATCTGAAGCCATCGGTGCAAGTTGCGACTTCTGATAGGAAAGAA
CCAATGAAGCGACCTGATAGAGGCGGAGTTGTGGCTGTGCGGCGTGTTAATCTATATGTG
AATCATTATAAAGTGAATTTCAATCCTGAAAGTGTTATAAGACATTATGATGTTGAAATC
AAAGGAGAAATTCCTACCAAGAAGGTTTCGAGGTTTGAGCTAGCTATGGTTAGGGACAAG
GTGTTCACTGACAATCCCGATGAGTTTCCCTTAGCTATGACAGCTTATGATGGTCAGAAG
AACATTTTCAGTGCGGTTGAGTTACCTACGGGGTCATACAAGGTGGAGTATCCTAAAACT
GAAGAGATGAGAGGTCGAAGCTATACGTTCACTATCAAACAGGTGAATGTGCTGAAGCTA
GGTGACTTGAAAGAGTACATGACAGGGAGATCGTCTTTCAATCCGCGTGATGTGTTGCAA
GGAATGGATGTTGTGATGAAGGAGCATCCTTCCAAGTGTATGATCACTGTTGGTAAAAGC
TTTTTCACTCGTGAAACTGAGCCAGATGAAGATTTTCGTTTCGGGGTTATAGCTGCGAAA
GGGTATCGCCACACTCTGAAGCCCACAGCACAAGGTTTGTCTTTGTGTTTGGATTACTCG
GTGTTGGCGTTCCGCAAAGCAATGTCGGTCATTGAATACCTGAAGTTGTACTTTAACTGG
TCTGATATGCGTCAGTTTAGGAGGCGTGATGTGGAAGAGGAATTGATTGGTTTGAAAGTC
ACTGTCAATCATCGGAAGAACAAGCAGAAACTCACCATTGTAGGGCTGAGTATGCAAAAC
ACAAAAGACATCAAATTTGATCTTATTGATCAAGAGGGAAACGAGCCGCCAAGGAAGACG
TCCATTGTTGAGTATTTCAGGATAAAGTATGGAAGACACATTGTTCACAAGGATATACCT
TGCTTGGATTTGGGAAAAAACGGTAGGCAAAATTTTGTGCCCATGGAATTCTGTGACTTG
GTTGAGGGACAGATATATCCAAAGGATAACTTGGATAAAGATTCAGCTTTGTGGCTAAAA
AAGTTGTCACTGGTCAATCCACAACAAAGGCAGAGGAATATAGATAAGATGATAAAGGCT
CGTAATGGACCGAGCGGTGGTGAAATCATTGGAAACTTTGGATTGAAAGTGGATACAAAC
ATGACACCGGTGGAAGGTCGTGTACTCAAGGCTCCATCATTGAAGTTGGCAGAGAGAGGG
AGAGTTGTGCGTGAGGAACCCAACCCGAGACAGAACAACCAATGGAACCTTATGAAGAAG
GGAGTCACAAGGGGATCTATAGTCAAGCATTGGGCTGTACTTGACTTCACTGCATCCGAG
AGATTTAACAAGATGCCTAATGACTTTGTGGATAACCTTATTGATCGTTGTTGGAGACTT
GGGATGCAGATGGAGGCTCCTATCGTTTACAAAACATCGAGAATGGAAACACTTTCTAAT
GGTAATGCTATTGAGGAATTGCTTCGATCCGTGATAGATGAAGCTTCTCGTAAGCATGGT
GGGGCTCGTCCAACTCTTGTTCTGTGTGCTATGTCTCGGAAAGACGATGGCTATAAGACT
CTGAAATGGATAGCCGAGACCAAACTTGGTCTGGTGACTCAGTGTTTCTTGACTGGTCCT
GCCACTAAAGGAGGTGATCAGTACCGGGCAAATCTTGCCCTCAAGATGAACGCAAAGGTT
GGTGGAAGCAATGTCGAGCTTATGGATACTTTCTCTTTCTTCAAAAAAGAGGATGAGGTC
ATGTTCATTGGTGCTGATGTCAATCATCCCGCTGCTCGGGACAAGATGAGCCCGTCCATT
GTTGCTGTTGTGGGAACTCTTAACTGGCCTGAAGCAAATCGCTATGCAGCTAGAGTCATT
GCCCAGCCTCACCGCAAAGAGGAAATACAAGGATTTGGCGACGCTTGTTTGGAGCTTGTC
AAAGCTCATGTTCAGGCCACAGGGAAACGGCCTAACAAGATTGTGATATTCCGTGATGGT
GTCAGCGATGCTCAGTTCGATATGGTTCTCAATGTGGAGTTGCTTGATGTTAAGCTAACT
TTTGAGAAGAATGGTTACAATCCAAAGATAACGGTAATCGTAGCCCAGAAACGTCATCAA
ACCCGTTTCTTCCCAGCCACAAATAATGATGGAAGTGATAAGGGCAATGTGCCTTCAGGT
ACGGTTGTTGATACTAAAGTTATTCACCCGTATGAGTATGATTTCTACCTCTGCAGTCAC
CACGGAGGGATAGGGACAAGCAAACCGACTCATTACTACACTCTTTGGGACGAACTTGGA
TTCACTTCGGATCAGGTGCAGAAGCTCATCTTCGAGATGTGCTTCACTTTCACTCGCTGC
ACCAAACCCGTCTCTCTTGTTCCGCCGGTGTATTATGCTGACATGGTTGCTTTTAGAGGA
AGGATGTACCACGAGGCAAGCTCTCGTGAGAAGAACTTTAAGCAGCCGCGGGGAGCGTCA
ACCTCTGCTGCTTCGCTTGCCTCTTCATTATCTTCTCTTACAATTGAGGACAAAGCGATT
TTCAAGCTGCATGCAGAGCTTGAGAATGTTATGTTCTTCGTCTGA
>Arabidopsis-thaliana_AT1G31290.1
ATGGATCGAGGTGGTTACCGAGGAGGTCGTGGTGATGGCCGTGGCAGAGGCGGCGGCGGA
GATCGTGGTCGTGGTTACAGCGGTCGTGGTGATGGCCGTGGCAGAGGCGGCGACGGAGAT
CGTGGTTACAGCGGTCGTGGTGATGGCCATGGCAGAGGCGGCGGCGGAGATCGTGGTCGT
GGTTACAGCGGTCGTGGTGATGGCCGTGGCAGAGGCGGCGGCGGAGATCGTGGTCGTGGT
TACAGCGGTCGTGGTGATGGCCATGGCAGAGGCGGCGGCGGAGATCGTGGTCGTGGTTAC

AGCGGTCGTGGTCGTGGCTTTGTTCAAGATAGGGATGGTGGTTGGGTAAATCCAGGGCAA
AGTTCAGGTGGTCATGTTCGTGGTCGTGGGACGCAGTTGCAACAACCTCCGCCACAGGAA
GTTCCTCCGTCGTCGTCGCAAGCTCAAGTGAGTCAAGGCGTTGCACCAGGAGACGTGGGT
CAAGGCGGCGTCGGAGACGTGGGTAGAGACGGCGTCGGAGACGTGGGTAGAGACGGCGTC
GGAGACGTGGGTCAAGGTGGCGTCGGAGACGTGGGTCAAGTAGGCGTCGGAGACGTGGGT
CAAGGCGGCGTCGGAGACGTGGGTCAAGGCGGCGTCGGAGACGTGGGTAGAGACGGCGTC
GGAGACGTGGGTAGAGACGGCGTCGGAGACGTGGGTAGAGGCGGCGTCGGAGACCGTGGA
CAGAGTCAGTCGGGACTATCGTCGGGTCACTTTGGTCGTGGGACGCAGTTGCAACAACCT
CAGCCACAGGCGGTTTCTCAGTCGTCTTCGCAGGGTCAAGTGAGTCAATCCTTTGCAACA
GGAGGCGTGGGTTTAGGCGCGTGGGCTCGTAAGCCTCAACTTTTCTCTGATTCGACGGTC
TTACCTTCTAGTTCTTCGTCTAACGTCGTTGCTTCTCACACCGCTAGTGGTTCTCAAGTT
ATGACTCCGAAACCATCTTCTTCTGATAAGAAAGAACCAGTAAAGCGTCCTGATAAAGGT
GGTAATATAAAAGTGAAAGGCGTAATTAATCTCTCCGTCAATCATTTTAGAGTGAGTTTC
TCAACAGAAAGTGTCATAAGGCACTACGATGTTGATATCAAAGGAGAAAATTCTTCAAAA
AAGATATCAAGATTTGAGCTAGCTATGGTCAAAGAAAAGCTGTTCAAGGACAATAATGAC
TTTCCTAACGCCATGACAGCGTACGATGGTCAGAAGAACATTTTCAGCGCTGTTGAATTA
CCTACGGGATCATTTAAAGTTGATTTCTCAGAAACCGAGGAGATCATGAGAGGTAGAAGC
TATACGTTCATCATCAAACAAGTGAAAGAGCTAAAGTTACTTGACTTGCAAGCCTACATA
GATGGACGTTCCACTTTTATTCCGCGTGATGTGTTGCAAGGAATGGATGTTGTGATGAAG
GAACATCCTTCAAAACGTATGATCACTGTTGGTAAAAGATTCTTTAGTACTCGGCTAGAG
ATTGACTTTGGATACGGAGTTGGAGCTGCGAAAGGGTTTCACCACACTCTCAAGCCCACA
GTACAAGGTTTATCATTATGTTTGAACTCCTCTTTGTTGGCGTTCCGCAAAGCAATTTCA
GTCATCGAATACCTGAAGTTGTACTTTGGGTGGAGAAATATACGTCAGTTTAAGAATTGT
AGGCCTGATGACGTGGTACAAGAATTGATTGGTTTGAAAGTCACTGTTGATCATCGAAAG
ACCAAACAGAAATTCATCATTATGGGGTTGAGTAAGGACGACACAAAAGATATCAAATTC
GATTTTATTGATCATGCTGGAAACCAGCCTCCAAGGAAAATATCCATTGTTGAGTATTTC
AAGGAAAAGTATGGAAGAGACATTGATCACAAGGATATTCCTTGCTTGAATTTGGGGAAA
AAGGGTCGGGAAAATTTTGTACCCATGGAGTTCTGTAACTTAGTCGAGGGGCAGATTTTT
CCAAAAGAGAAATTGTATAGAGATTCAGCCGCGTGGTTAAAGGAGCTTTCACTAGTCACT
CCACAACAAAGACTGGAGAATATAAATAAGATGATAAAGTCTAGTGATGGACCTAGAGGT
GGAGATATCATTGGAAACTTCGGATTGAGAGTGGATCCAAACATGACAACGGTGGAAGGT
CGTGTACTCGAGGCTCCTACACTGAAATTGACTGATCGGAGAGGCAATCCTATCCATGAG
AAACTCATGAGCGAGAGCAATCAATGGAACCTTACGACAAAGGGAGTCACAAAGGGTTCA
ATAATCAAGCATTGGGCTGTTCTTGACTTCACTGCATCCGAGAGTTTGAAAAAAAAGATG
CCTGGTTACTTTGTAAATAAACTCATCGAACGTTGTAAGGGACTTGGGATGCAGATGGAG
GCTCCTATCGTTTGCAAAACATCGAGTATGGAAACACTTTATGATGGTAATGCTCTTGAG
GAATTGCTTCGATCCGTGATAGATGAGGCTTCTCATAACCATGGTGGGGCTTGTCCAACT
CTTGTTCTGTGTGCTATGACTGGGAAGCACGATGGATACAAGACTCTGAAATGGATAGCC
GAGACCAAACTTGGTCTAGTGACTCAGTGTTTCTTGACCATATCTGCCATTAAAGGAGAA
ACCGTTTCTGATCAGTACTTGGCAAATCTCGCCCTCAAGATAAACGCAAAGGTTGGTGGA
ACGAACGTGGAGTTGGTGGATAATATTTTCTCTTTCTTCAAAAAAGAAGATAAGGTCATG
TTCATTGGTGCTGATGTCAATCATCCCGCTGCTCACGACAATATGAGTCCATCCATTGTT
GCTGTTGTAGGCACTCTTAACTGGCCTGAAGCTAACCGCTACGCAGCTAGAGTCAAAGCT
CAGAGTCACCGTAAAGAAGAGATACAAGGGTTTGGTGAAACTTGCTGGGAGCTTATCGAA
GCTCATTCTCAGGCCCCCGAGAAACGACCTAACAAGATTGTGATATTCCGTGATGGTGTC
AGCGATGGTCAGTTCGATATGGTTCTCAATGTGGAGTTACAGAATGTTAAGGACGTTTTT
GCCAAGGTTGGCTATAATCCGCAGATAACTGTAATTGTGGCACAGAAACGTCATCAAACC
CGTTTCTTCCCCGCCACAACTAGCAAAGATGGAAGGGCTAAGGGCAATGTGCCTTCAGGT
ACGGTCGTTGATACCACGATCATTCACCCGTTTGAGTATGATTTCTACCTCTGTAGTCAA
CATGGAGCGATAGGTACAAGCAAACCAACTCATTATTATGTTCTTTCGGACGAAATCGGG
TTCAACTCGAATCAGATTCAGAAACTCATCTTTGACTTGTGCTTCACGTTTACTCGCTGC
ACCAAACCGGTCGCTCTGGTTCCTCCGGTTTCTTATGCTGACAAGGCTGCTTCTAGAGGA
AGGGTGTACTACGAGGCAAGCTTAATGAAGAAGAATTCTAAGCAGTCGCGTGGAGCGTCT
TCGTCTTCTGCCTCGGTTGCCTCTTCATCCTCTTCTGTCACAATGGAGGACAAAGAGATC
TTCAAGGTTCACGCAGGCATCGAGAACTTTATGTTCTTTGTCTGA
>Arabidopsis-thaliana_AT1G48410.2
ATGGTGAGAAAGAGAAGAACGGATGCTCCATCTGAAGGAGGTGAAGGCTCTGGGTCTCGT
GAAGCTGGTCCAGTCTCAGGTGGTGGACGTGGTTCACAGCGAGGTGGTTTCCAGCAGGGA
GGAGGACAACACCAAGGTGGAAGGGGTTATACTCCTCAACCTCAACAGGGAGGTCGTGGT
GGTCGTGGATATGGGCAACCACCACAACAGCAACAACAGTATGGAGGACCACAAGAGTAC
CAAGGAAGAGGAAGAGGAGGACCTCCTCATCAAGGAGGTCGAGGAGGGTATGGCGGTGGC
CGTGGAGGTGGACCTTCTTCTGGACCACCGCAGAGACAATCAGTTCCCGAGCTGCATCAA

GCTACCTCACCTACTTATCAAGCGGTGTCTTCTCAGCCTACACTGTCTGAGGTGAGTCCT
ACCCAGGTACCAGAACCTACTGTTCTGGCTCAGCAATTTGAACAACTCTCTGTTGAACAA
GGAGCTCCCAGTCAGGCAATCCAGCCTATACCTTCTTCTAGCAAGGCTTTCAAGTTTCCA
ATGAGGCCTGGTAAAGGACAGAGTGGAAAGCGTTGCATTGTGAAGGCTAACCATTTCTTT
GCTGAACTGCCTGATAAGGATTTGCACCATTATGATGTTACCATTACTCCGGAAGTTACA
TCAAGGGGTGTCAATCGTGCTGTGATGAAACAACTTGTTGATAATTATCGTGATTCTCAC
CTTGGAAGTCGTCTTCCAGCGTATGATGGTCGAAAAAGTCTTTACACTGCTGGTCCACTT
CCCTTTAACTCCAAGGAGTTCAGAATCAATCTTCTTGACGAAGAAGTAGGGGCTGGAGGT
CAAAGACGAGAAAGGGAATTTAAAGTTGTGATCAAGCTAGTTGCACGTGCTGATCTGCAT
CACCTAGGAATGTTTTTGGAGGGGAAACAATCAGATGCCCCACAGGAAGCTCTGCAGGTT
CTTGACATTGTTCTTCGTGAGCTGCCGACCTCTAGAATCAGGTATATTCCGGTGGGCCGG
TCCTTTTATTCCCCTGATATAGGAAAAAAACAATCATTGGGGGATGGCTTGGAGAGCTGG
CGTGGATTCTACCAAAGCATTCGTCCTACACAGATGGGCTTATCACTCAATATTGATATG
TCATCGACAGCCTTCATAGAGGCAAACCCTGTGATTCAGTTTGTCTGTGATTTGCTTAAC
CGGGATATTTCTTCTCGACCTTTATCTGATGCTGATCGTGTTAAGATAAAAAAGGCTCTT
AGAGGTGTCAAAGTTGAAGTGACTCATCGAGGAAACATGCGCCGGAAGTACCGCATTTCC
GGTTTGACTGCTGTGGCCACTCGGGAATTGACATTCCCAGTAGATGAAAGAAATACTCAG
AAATCTGTTGTAGAATACTTCCACGAAACATATGGTTTTCGCATTCAGCACACTCAACTA
CCATGCTTGCAAGTTGGGAATTCTAATAGGCCTAATTACTTACCAATGGAGGTATGCAAG
ATTGTTGAAGGCCAGCGGTATTCCAAAAGATTGAATGAGAGACAGATCACTGCTTTGCTG
AAGGTTACCTGTCAGCGCCCGATAGATCGAGAAAAAGATATCTTACAGACGGTGCAACTC
AATGATTATGCTAAAGATAATTATGCTCAAGAGTTTGGCATCAAAATAAGTACTTCTCTG
GCTTCTGTTGAGGCTCGTATACTGCCTCCTCCATGGCTTAAGTACCACGAGTCTGGAAGG
GAAGGGACTTGTCTGCCACAAGTTGGTCAATGGAACATGATGAATAAGAAAATGATCAAT
GGTGGAACGGTGAATAATTGGATCTGCATCAACTTTTCTAGGCAAGTGCAGGACAATCTA
GCGCGTACATTTTGTCAGGAACTTGCTCAAATGTGTTACGTATCTGGCATGGCATTTAAT
CCGGAACCAGTCCTCCCACCAGTCAGTGCTCGCCCTGAGCAAGTAGAGAAGGTCTTGAAG
ACTAGATATCATGATGCCACATCAAAACTCTCCCAAGGAAAAGAAATTGATCTGCTTATT
GTCATTCTGCCCGATAATAATGGATCATTATACGGTGATTTGAAACGCATATGTGAGACT
GAACTCGGCATAGTCTCTCAATGTTGCCTGACAAAGCATGTCTTTAAGATGAGCAAACAA
TACATGGCTAATGTTGCGCTGAAGATTAATGTGAAGGTTGGAGGAAGAAACACAGTGCTT
GTTGATGCTCTATCTAGGCGGATTCCTCTAGTCAGTGATCGACCCACCATTATATTTGGT
GCTGATGTTACCCACCCTCACCCTGGAGAGGATTCAAGCCCATCTATTGCTGCTGTTGTG
GCATCTCAGGATTGGCCTGAAATCACTAAATATGCTGGATTAGTTTGCGCTCAAGCGCAT
AGGCAGGAGCTCATTCAGGATCTGTTCAAAGAGTGGAAGGATCCTCAGAAAGGTGTGGTG
ACTGGTGGCATGATAAAGGAGTTGCTCATAGCCTTCCGTAGATCAACTGGGCATAAACCA
CTAAGGATCATCTTCTACAGGGATGGAGTCAGTGAGGGACAATTTTACCAAGTTTTGCTC
TATGAACTTGATGCCATCCGCAAGGCCTGTGCTTCGCTGGAAGCAGGTTATCAACCACCA
GTGACATTTGTGGTGGTGCAGAAGCGTCATCACACGAGGCTGTTTGCTCAGAACCACAAT
GATCGCCATTCGGTGGACAGAAGTGGGAATATTTTACCTGGCACTGTTGTGGACTCTAAA
ATCTGCCACCCTACAGAGTTTGACTTTTACCTCTGTAGTCATGCTGGTATTCAGGGCACT
TCTCGACCTGCTCATTACCACGTTCTTTGGGATGAGAACAACTTTACTGCAGATGGACTT
CAATCTCTGACCAATAACTTATGTTACACGTATGCAAGATGCACACGCTCAGTTTCAATT
GTTCCCCCTGCATATTATGCACATCTAGCAGCTTTTAGGGCTCGATTCTACATGGAGCCA
GAGACATCAGACAGTGGCTCAATGGCTAGTGGGAGCATGGCACGTGGAGGTGGAATGGCT
GGTAGAAGCACACGCGGGCCTAATGTCAATGCTGCAGTGAGGCCACTCCCAGCTCTGAAA
GAGAATGTGAAGCGTGTCATGTTCTACTGCTGA
>Arabidopsis-thaliana_AT1G69440.1
ATGGAAGAAAAAACTCATCATCATCATCACAGTACCAACAAACACATCCCTAGTTCCAAA
TCAAGAACACCTCTTCTCCACAAGCCTTATCATCACCATGTTCAAACCAATCCTCCTCCT
TTTCTTTTGCACCCTTCTTCTCACCAAAACCTCAATCTTGTTGCCTCAAATCTTCCTTCT
AGCTACTACTACTATTACTACTGCTACTTCTACTCTCAATTTCACAACTCTCTTCCTCCT
CCTCCTCCTCCTCATCTTCTTCCTCTTTCTCCTCCTCTTCCTCCTTTACTTCCTCTTCCT
CCTCCTCACTCAATGACCCGTTTCCACAAATCTCTCCCTGTTTCTCAAGTTGTGGAGAGA
AAGCAGCAACACCAACAGAAGAAGAAGATACAAGTTTCTAACAACAAAGTGAGTGGATCC
ATAGCAATAGAAGAAGCAGCATTAGTGGTTGCTAAAAGACCGGACTTTGGTGGTCAAGAT
GGTTCTGTCATTTATCTCCTCGCCAACCATTTTCTTGTCAAGTTTGATTCGTCACAGAGG
ATTTACCATTACAATGTTGAGATATCTCCACAGCCTTCAAAGGAAATTGCTAGAATGATC
AAACAGAAGCTTGTTGAGACAGATCGTAATAGCTTCTCTGGTGTTGTTCCGGCTTTTGAC
GGCAGGCAAAACATTTACAGCCCTGTGGAGTTTCAGGGAGATAGGCTTGAGTTCTTTGTT
AATCTCCCTATCCCATCTTGCAAGGCTGTGATGAATTACGGTGACTTGCGTGAGAAGCAA
CCTCAGAAGAAGATTGAGAAACTGTTTAGGGTTAATATGAAGCTTGTGTCCAAGTTTGAT

GGTAAAGAACAGAGAAAGGAAGGAGAGGATTGGGCTCCTCTGCCTCCAGAATACATTCAT
GCTCTTGATGTTATCTTGAGAGAGAATCCAATGGAGAAGTGTACATCGATCGGAAGATCG
TTTTACTCGAGTTCTATGGGTGGATCTAAGGAGATTGGAGGAGGAGCTGTTGGACTCAGA
GGGTTTTTCCAGAGTCTTAGACATACTCAGCAAGGTTTAGCACTTAACATGGATCTCTCA
ATCACAGCTTTCCATGAAAGTATTGGAGTAATAGCTTACTTGCAGAAGCGGCTCGAGTTT
CTTACGGACCTTCCTAGGAACAAAGGTAGAGAATTGAGTTTAGAGGAAAAGAGAGAAGTG
GAGAAAGCACTTAAGAACATAAGAGTCTTTGTTTGCCATAGAGAAACAGTTCAAAGGTAT
CGAGTTTATGGGTTAACAGAGGAAATTACGGAGAATATATGGTTTCCTGATAGAGAAGGG
AAATATCTAAGGCTTATGAGTTACTTCAAAGATCATTATGGTTATGAGATTCAGTTCAAG
AACTTGCCGTGTCTGCAAATCAGTAGGGCAAGACCTTGTTACCTTCCTATGGAGCTATGC
ATGATTTGTGAAGGTCAAAAGTTTCTTGGAAAGCTATCAGATGATCAAGCTGCAAAGATC
ATGAAAATGGGCTGCCAAAAACCTAATGAAAGGAAAGCTATTATTGATAAGGTTATGACA
GGATCGGTCGGTCCATCGAGCGGAAACCAAACAAGAGAATTCAACCTTGAGGTTTCAAGA
GAAATGACATTGCTGAAAGGAAGAATACTTCAGCCACCAAAGCTTAAACTTGACCGGCCA
AGGAACCTTAAAGAAAGTAAAGTTTTAAAGGAACTAGAATTGAGAGATGGGCTTTGATG
AGTATTGGAGGCAGCTCTGATCAGAAATCTACCATTCCCAAGTTCATAAACGAGCTTACT
CAAAAGTGTGAGCATTTGGGAGTCTTCTTAAGCAAGAACACATTAAGCAGTACCTTCTTT
GAACCATCACACATACTCAACAACATTTCGCTTCTCGAATCAAAACTGAAGGAGATTCAA
AGAGCGGCATCGAACAATCTCCAGCTGATTATTTGTGTAATGGAGAAAAAACATAAAGGG
TACGGAGATCTAAAGAGGATATCAGAGACAAGAATTGGTGTTGTGACACAATGCTGCTTA
TACCCTAACATCACTAAGCTCAGTTCTCAGTTCGTTTCGAACTTAGCTCTCAAGATAAAC
GCCAAGATCGGTGGATCCATGACCGAGCTCTACAACTCGATACCTTCTCACATCCCTAGA
CTGCTTAGACCCGATGAGCCGGTTATCTTCATGGGAGCTGATGTAACGCATCCTCATCCA
TTCGATGATTGTAGCCCTTCAGTAGCGGCTGTGGTAGGGAGCATAAACTGGCCAGAAGCT
AACCGATACGTCTCAAGAATGAGGTCTCAGACTCATAGGCAAGAGATCATACAAGATCTT
GACTTGATGGTCAAGGAACTTCTTGATGATTTTTACAAAGCGGTAAAAAAGCTTCCGAAT
CGAATCATATTCTTCAGGGACGGTGTTAGCGAGACACAGTTCAAGAAAGTTCTCCAAGAA
GAGCTTCAATCGATAAAAACTGCTTGTTCGAAGTTCCAAGATTACAATCCAAGCATCACA
TTCGCCGTGGTTCAGAAAAGACACCACACAAGGCTGTTCCGGTGCGATCCAGACCATGAG
AACATACCTCCTGGTACAGTGGTTGATACAGTGATAACTCATCCGAAAGAGTTTGATTTT
TATCTCTGTAGCCATTTAGGAGTGAAGGGCACGAGCAGGCCAACGCATTACCATATTCTA
TGGGACGAGAACGAGTTCACTTCAGACGAATTGCAGAGACTTGTGTATAATTTGTGTTAC
ACTTTCGTGAGGTGCACGAAACCTATATCGATTGTGCCACCGGCTTATTATGCTCACCTT
GCTGCGTACAGAGGAAGGCTATACATCGAGAGATCATCTGAATCTAATGGAGGATCCATG
AATCCTTCTTCTGTGTCTCGTGTTGGTCCTCCAAAGACGATTCCTCTTCCTAAATTAAGT
GATAATGTCAAGAATCTCATGTTTTACTGCTGA
>Arabidopsis-thaliana_AT2G27040.1
ATGGATTCAACAAATGGTAACGGAGCTGATCTTGAATCAGCAAATGGGGCAAACGGGAGT
GGGGTTACTGAGGCATTACCACCTCCTCCACCAGTTATACCTCCAAATGTGGAACCAGTT
CGTGTTAAAACTGAACTTGCTGAGAAGAAGGGGCCAGTTCGAGTTCCTATGGCTCGAAAA
GGATTTGGAACAAGGGGCCAAAAGATCCCCTTGTTAACAAATCATTTCAAAGTCGATGTG
GCTAATCTTCAGGGTCATTTCTTCCACTACAGTGTGGCTCTATTCTATGATGATGGTCGT
CCTGTTGAACAAAAGGGTGTTGGAAGAAAAATCCTTGACAAGGTGCATCAGACTTACCAT
TCTGATCTGGATGGTAAAGAGTTTGCTTATGACGGTGAGAAGACGTTGTTTACATATGGA
GCTTTGCCTAGTAACAAGATGGATTTTTCTGTGGTGCTTGAGGAAGTATCTGCTACAAGG
GCTAATGGAAACGGAAGCCCCAATGGGAATGAAAGTCCAAGTGATGGTGATAGGAAAAGA
CTGCGTAGGCCTAACCGGTCCAAAAACTTTAGAGTGGAGATCAGCTATGCGGCCAAAATT
CCTCTTCAAGCTCTTGCTAATGCAATGCGGGGACAAGAATCAGAGAATTCCCAGGAGGCA
ATACGGGTTCTTGATATCATATTGAGGCAACATGCTGCTAGACAAGGTTGCTTGCTTGTT
CGACAGTCTTTTTTCCACAATGATCCAACCAACTGTGAACCAGTTGGTGGTAACATCTTA
GGATGTAGGGGATTTCACTCCAGTTTCAGAACAACGCAGGGTGGCATGTCACTTAATATG
GATGTTACAACCACCATGATCATCAAGCCTGGTCCAGTGGTTGATTTCCTAATTGCTAAC
CAAAATGCTAGGGACCCTTATTCGATTGACTGGTCTAAGGCTAAACGAACCCTTAAGAAC
CTAAGGGTAAAGGTCAGCCCCTCAGGCCAAGAATTCAAGATAACCGGATTGAGTGACAAG
CCTTGCAGGGAACAAACGTTTGAATTGAAGAAAAGGAACCCAAATGAAAATGGAGAGTTC
GAAACTACTGAAGTTACAGTTGCTGACTACTTCCGCGATACAAGGCATATTGATTTGCAA
TATTCTGCGGATTTGCCTTGCATCAATGTTGGGAAGCCAAAGCGACCCACTTACATTCCT
CTCGAGCTCTGCGCGTTGGTTCCACTTCAGAGGTACACAAAAGCACTTACCACGTTCCAA
AGATCTGCCCTTGTTGAGAAATCCAGACAGAAACCCCAAGAGAGGATGACTGTTCTGTCC
AAAGCTCTGAAAGTTAGCAACTATGATGCGGAACCACTCCTGCGATCCTGTGGCATTTCG
ATCAGCTCCAACTTTACTCAGGTGGAGGGTCGTGTTCTACCAGCTCCCAAGCTGAAAATG
GGATGTGGATCTGAAACCTTTCCCAGAAATGGTCGCTGGAACTTCAACAACAAGGAATTT

GTTGAGCCCACCAAAATTCAACGATGGGTTGTTGTCAATTTCTCTGCTCGCTGTAATGTA
CGTCAAGTTGTTGATGATCTGATAAAAATTGGAGGATCAAAAGGAATTGAAATTGCTTCT
CCCTTTCAAGTGTTTGAGGAGGGTAATCAATTCCGCCGTGCTCCTCCTATGATTCGTGTT
GAGAACATGTTTAAGGACATCCAATCGAAACTCCCTGGTGTCCCACAATTCATACTATGT
GTGCTCCCTGACAAAAAGAACAGTGATCTCTATGGTCCATGGAAGAAAAAAAACTTAACT
GAATTTGGCATTGTTACTCAATGCATGGCTCCAACGCGGCAACCTAATGATCAGTATCTT
ACTAACTTACTTCTGAAGATTAATGCAAAGCTTGGAGGCCTGAACTCAATGTTAAGTGTA
GAGCGTACACCTGCGTTCACTGTGATTTCTAAGGTTCCAACCATTATCCTTGGGATGGAT
GTTTCACATGGATCTCCTGGACAGTCTGATGTCCCGTCCATCGCTGCTGTGGTGAGTTCT
AGGGAGTGGCCACTGATATCCAAATATAGAGCATCTGTTCGGACACAGCCTTCTAAGGCT
GAGATGATTGAGTCCCTTGTCAAGAAAAATGGAACTGAAGACGATGGCATTATCAAGGAG
TTGCTGGTAGATTTCTACACCAGCTCGAATAAGAGAAAACCAGAGCATATCATAATTTTC
AGGGATGGTGTGAGTGAATCTCAATTCAATCAGGTTCTGAATATTGAACTTGATCAGATC
ATCGAGGCTTGCAAGCTCTTAGACGCAAATTGGAACCCAAAGTTCCTTTTGTTGGTGGCT
CAAAAGAATCATCATACCAAGTTCTTCCAGCCAACGTCTCCTGAAAATGTTCCTCCAGGG
ACAATCATTGACAACAAAATATGTCACCCAAAGAACAATGATTTCTACCTCTGTGCTCAC
GCTGGAATGATTGGAACTACCCGCCCAACTCACTACCACGTCCTGTATGATGAGATTGGT
TTTTCAGCTGACGAACTTCAGGAACTTGTCCACTCGCTCTCCTATGTGTACCAAAGAAGC
ACCAGTGCCATTTCTGTTGTTGCGCCGATCTGCTATGCTCACTTGGCAGCTGCTCAGCTT
GGGACGTTCATGAAGTTTGAAGATCAGTCTGAGACATCATCAAGCCATGGTGGTATCACA
GCTCCAGGACCAATCTCTGTTGCACAGCTCCCAAGACTCAAAGACAACGTCGCCAACTCC
ATGTTCTTCTGTTAA
>Arabidopsis-thaliana_AT2G27880.1
ATGTCAAATCGTGGTGGTGGTGGTCACGGCGGCGCTAGTCGTGGTCGAGGAGGTGGACGA
AGGTCTGACCAGAGACAAGATCAGTCTTCTGGTCAAGTTGCTTGGCCGGGTTTACAACAA
AGCTACGGTGGTCGTGGTGGTTCTGTTTCCGCCGGTAGAGGTCGTGGAAACGTCGGAAGA
GGTGAAAATACTGGAGATCTGACGGCGACGCAAGTTCCGGTTGCTTCTGCTGTTTCTGGC
GGGAGAGGTCGTGGTAACATCGGAGATCCGACGTTTTCTGTTGCGTCTTCGTCTAAGACG
GTGTCTGTTGCTTCTTCATCTAAAGAGGAAAGTAAGAATACGGAGGTTTCTGAGACCATG
TCGAACCTTCAGATTACTTCGACGGAGACGAAACCTGAAATGACGTCACTTCCTCCGGCG
TCATCTAAAGCGGTTACGTTTCCGGTACGGCCAGGACGTGGTACTTTGGGGAAGAAAGTC
ATGGTTCGTGCGAATCATTTCCTTGTTCAAGTTGCTGATCGTGATCTCTACCATTACGAT
GTTTCGATCAATCCTGAGGTTATATCAAAGACAGTGAACAGAAACGTGATGAAACTTCTG
GTTAAGAATTATAAAGACTCTCACTTGGGAGGGAAGTCACCAGCGTATGATGGAAGGAAA
AGCCTCTATACTGCTGGTCCATTACCTTTTGACTCGAAAGAGTTTGTTGTGAATCTGGCG
GAGAAAAGAGCTGACGGTTCCTCTGGGAAGGACAGACCGTTTAAAGTTGCTGTAAAGAAT
GTGACAAGCACTGATCTTTATCAGTTGCAACAGTTCCTTGATCGTAAGCAAAGAGAGGCT
CCATATGATACTATCCAAGTGCTTGATGTTGTTCTTAGGGATAAGCCCTCTAATGATTAT
GTCTCTGTTGGGAGGTCTTTTTTCCACACTAGTTTGGGAAAGGACGCAAGAGATGGTAGG
GGTGAGCTTGGAGATGGTATTGAGTACTGGAGAGGTTATTTCCAAAGTCTAAGGCTGACT
CAGATGGGTTTGTCTCTGAACATTGACGTTTCAGCAAGATCATTTTATGAACCGATTGTT
GTCACTGACTTTATTAGCAAGTTTCTGAATATAAGGGACTTAAACAGGCCACTTAGAGAC
TCAGATCGACTTAAGGTGAAGAAAGTTTTGAGGACACTGAAAGTTAAGTTGCTTCACTGG
AACGGCACAAAAAGTGCCAAAATTAGTGGGATTTCTAGTCTACCCATCAGGGAGCTAAGG
TTCACTCTGGAGGACAAATCAGAGAAGACGGTTGTTCAATATTTTGCTGAAAAATATAAT
TATAGAGTGAAATACCAGGCTCTACCTGCTATTCAAACAGGGAGTGACACAAGACCCGTC
TACCTACCAATGGAGCTCTGCCAAATTGACGAAGGGCAAAGATACACCAAAAGGCTCAAT
GAGAAGCAAGTGACTGCATTGCTAAAAGCTACCTGCCAACGACCCCCTGATAGAGAGAAC
TCGATCAAAAACTTGGTTGTGAAAAATAATTACAATGATGATCTGAGCAAGGAGTTTGGG
ATGTCAGTGACTACCCAACTAGCCTCGATTGAAGCTCGTGTACTTCCCCCACCGATGTTG
AAGTACCATGACAGTGGTAAAGAGAAAATGGTAAATCCAAGGCTAGGACAGTGGAACATG
ATTGACAAGAAAATGGTTAATGGAGCAAAAGTCACTTCTTGGACTTGCGTAAGTTTCTCT
ACTCGGATTGACCGTGGTTTACCCCAAGAGTTCTGCAAACAGTTGATTGGGATGTGTGTC
AGCAAAGGAATGGAATTTAAGCCTCAACCTGCTATTCCGTTCATCTCTTGTCCCCCTGAA
CATATTGAGGAAGCTCTTCTCGATATCCACAAAAGGGCACCTGGTCTCCAACTGTTGATT
GTAATATTGCCTGATGTGACTGGATCATATGGAAAAATAAAAAGGATCTGTGAAACAGAA
TTGGGGATTGTCTCTCAGTGTTGCCAACCTAGACAAGTTAATAAACTCAACAAGCAGTAC
ATGGAAAATGTTGCCTTGAAGATCAATGTCAAGACTGGGGGAAGGAACACTGTTCTTAAT
GATGCTATTAGAAGAAACATACCTCTTATTACTGATCGTCCAACCATCATCATGGGTGCT
GATGTGACTCACCCACAGCCTGGAGAGGACTCAAGTCCTTCTATTGCTGCTGTTGTGGCC
TCTATGGACTGGCCTGAGATAAACAAATACCGAGGATTGGTTTCTGCTCAAGCTCATAGG
GAAGAAATTATTCAGGACCTGTATAAGCTGGTTCAGGATCCACAACGTGGGCTAGTCCAC

TCTGGTTTGATAAGGGAACATTTCATAGCATTCAGGAGAGCTACAGGCCAGATACCTCAA
AGGATCATCTTCTATCGTGACGGAGTAAGCGAAGGGCAGTTTAGTCAGGTTCTGCTACAT
GAGATGACTGCTATCCGCAAGGCTTGTAACTCTCTCCAAGAGAATTATGTTCCTCGTGTT
ACTTTCGTGATTGTCCAGAAACGTCACCACACACGTTTGTTCCCTGAGCAACACGGGAAT
CGTGATATGACTGATAAGAGTGGCAATATTCAACCAGGTACTGTCGTGGACACTAAAATC
TGTCACCCTAATGAATTCGACTTCTATTTGAACAGCCATGCTGGTATTCAGGGAACAAGC
AGGCCGGCACATTACCATGTACTTCTCGATGAGAACGGTTTCACCGCTGATCAGTTGCAA
ATGCTCACAAACAACCTCTGCTACACGTATGCGAGGTGTACAAAATCTGTGTCAATTGTG
CCACCAGCCTACTACGCTCACTTGGCTGCATTCCGTGCCCGCTACTACATGGAGAGTGAG
ATGTCTGATGGAGGTTCGAGCAGGTCCAGGAGCTCAACAACAGGTGTGGGTCAAGTCATT
TCGCAGCTCCCAGCAATAAAAGATAACGTCAAGGAGGTTATGTTTTATTGCTAA
>Arabidopsis-thaliana_AT2G32940.1
ATGGAGACATCTTCATCTCTGCCACTGTCACCCATAAGTATAGAACCAGAACAGCCCAGC
CATCGAGATTATGACATCACAACTAGACGTGGGGTTGGAACCACAGGCAACCCAATAGAA
TTGTGCACCAATCACTTTAATGTTTCTGTGAGACAGCCGGATGTAGTATTTTACCAGTAC
ACTGTTAGTATCACCACAGAAAACGGTGATGCTGTTGATGGGACAGGAATAAGCAGAAAG
CTTATGGACCAACTATTCAAGACTTACTCTTCTGATCTTGATGGTAAAAGGTTGGCATAT
GATGGAGAGAAGACTTTATATACTGTTGGTCCTCTACCACAGAACGAATTTGACTTTCTA
GTCATCGTGGAAGGGTCATTTTCAAAACGTGATTGTGGTGTCTCTGATGGTGGTAGTTCT
TCCGGTACTTGCAAGCGATCAAAACGTTCTTTCTTGCCAAGAAGTTACAAGGTTCAGATA
CATTATGCTGCAGAAATACCGTTGAAGACTGTTCTTGGCACTCAGAGAGGAGCTTATACA
CCAGATAAGAGTGCTCAGGATGCACTTAGAGTGCTTGACATTGTACTGAGGCAGCAAGCA
GCTGAAAGGGGATGCCTTTTGGTCAGACAGGCATTTTTCCATAGCGATGGGCACCCCATG
AAAGTTGGAGGAGGTGTTATAGGTATTCGAGGTTTACATTCGAGCTTTCGTCCAACTCAT
GGAGGACTCTCGCTTAACATTGATGTGTCAACAACAATGATCTTAGAACCTGGTCCAGTT
ATTGAATTCCTGAAAGCCAATCAGAGTGTTGAGACACCACGGCAAATCGACTGGATTAAG
GCTGCGAAAATGCTTAAACATATGAGGGTTAAGGCAACACACCGTAACATGGAATTTAAA
ATTATAGGTCTAAGTTCGAAACCGTGCAATCAGCAACTGTTTTCAATGAAGATTAAAGAT
GGTGAACGTGAGGTACCTATTAGAGAGATTACTGTGTATGACTATTTCAAGCAAACCTAC
ACAGAGCCTATTTCTTCTGCGTACTTTCCATGCCTTGATGTTGGCAAGCCAGATCGGCCC
AACTATCTTCCACTGGAGTTTTGTAATCTTGTATCTCTGCAACGTTATACAAAACCATTG
TCAGGGAGGCAAAGAGTTTTACTTGTTGAAAGTTCAAGACAAAAACCTCTAGAGAGAATT
AAAACACTCAATGATGCAATGCATACCTACTGCTATGACAAGGACCCATTTTTGGCTGGA
TGTGGCATCTCCATTGAAAAAGAAATGACTCAAGTTGAAGGCCGTGTTCTCAAGCCCCCA
ATGCTGAAGTTTGGCAAGAATGAAGATTTTCAACCTTGCAACGGACGGTGGAACTTTAAC
AACAAGATGCTTCTAGAACCAAGAGCTATTAAAAGCTGGGCTATCGTCAACTTTTCTTTT
CCATGCGACAGTAGTCACATTTCCCGTGAGCTTATAAGCTGTGGCATGAGGAAAGGCATT
GAAATTGATCGACCTTTTGCACTAGTTGAAGAGGATCCACAGTACAAAAAAGCAGGTCCT
GTTGAAAGGGTAGAAAAAATGATTGCAACGATGAAGTTAAAATTTCCAGATCCTCCTCAT
TTTATCCTCTGTATTCTGCCAGAACGGAAAACTTCTGATATCTATGGTCCCTGGAAGAAG
ATATGCCTCACTGAAGAAGGGATCCACACACAATGCATCTGCCCAATCAAGATCAGTGAC
CAATATCTCACCAATGTACTTCTGAAGATAAATTCGAAGCTTGGAGGCATCAATTCTCTG
TTGGGAATAGAGTACTCTTACAACATTCCATTGATAAACAAAATTCCCACCTTGATTTTG
GGTATGGATGTATCTCATGGGCCTCCAGGTCGGGCAGATGTTCCTTCAGTAGCAGCGGTT
GTTGGTTCAAAATGCTGGCCCTTAATCTCAAGGTATAGGGCAGCTGTAAGAACTCAGTCA
CCACGTCTGGAGATGATTGATTCGCTCTTCCAACCTATCGAGAACACTGAGAAAGGAGAT
AACGGTATCATGAACGAATTATTTGTAGAGTTCTATCGGACAAGCAGGGCACGGAAACCT
AAGCAGATTATCATTTTCAGGGATGGTGTGAGTGAATCACAGTTCGAGCAAGTCTTGAAG
ATCGAAGTGGACCAAATTATAAAGGCGTATCAACGTCTTGGTGAATCTGATGTGCCAAAA
TTCACTGTCATTGTGGCTCAGAAGAACCACCACACCAAGTTGTTTCAAGCTAAGGGTCCT
GAAAATGTTCCTGCAGGAACTGTCGTGGACACCAAGATCGTACACCCGACAAACTACGAT
TTTTACATGTGTGCTCATGCAGGAAAAATAGGAACTTCAAGACCGGCTCATTACCATGTA
TTACTTGATGAGATTGGTTTCTCCCCCGATGACTTGCAGAATCTCATCCATTCTCTCTCC
TATGTCAACCAACGTAGCACAACTGCAACTTCGATCGTGGCTCCAGTACGATACGCTCAT
CTTGCAGCAGCTCAAGTTGCGCAATTCACAAAGTTTGAAGGTATATCGGAGGACGGGAAA
GTTCCAGAGCTTCCACGTCTGCACGAGAATGTCGAAGGCAACATGTTCTTCTGCTGA
>Arabidopsis-thaliana_AT5G21030.1
ATGGATACGACTCTACCGCCTCCTCAACACATGGAACGTGAACCTTTGAAGAGCAAGAGT
AGTCTTCTTCCAATGACTCGCCGTGGCAACGGTTCCAAAGGACAGAAGATTCTTCTGCTC
ACTAACCACTTCAGAGTCAACTTCAGAAAACCAAACAGTCATAATTTCTTCCATTACAGC
GTTACTATCACATATGAAGATGGTAGTCCACTTTTAGCCAAAGGTTTTGGCAGAAAGATT
CTTGAAAAAGTTCAACAGACTTGTCAAGCTGATTTGGGTTGCAAACATTTTGCTTATGAT

GGCGACAAGAATCTTTATACTGTTGGTCCTCTTCCTAGATCCAGTCTAGACTTCTCTGTT
GTTCTTGAAACCGCGCCTTCTAGGAGAAACGCAGACAAAAGACTAAAGCTTCCTCACCAA
TCCAAGAAATTCAATGTTGCAATCCTTTTTGCGCCTCCAGAAATCCCAATGGAAGCTATT
GCAAATGCTCTTCAAGGAAAGAAAACTAAACATCTTCTAGATGCTATCAGAGTGATGGAC
TGTATTTTGAGCCAAAATGCAGCTAGACAAGGTTGCCTCCTAGTTCGGCAATCTTTCTTC
CACAATGACGCAAAGTACTTTGCGAATATCGGTGAAGGTGTAGATTGTTGCAAAGGATTT
CATTCAAGCTTCAGAACTACTCAGGGAGGCTTGTCCCTCAATATTGATGTTTCGACTGCT
ATGATAGTAAAACCTGGTCCTGTTGTTGATTTTCTTATTGCAAATCAAGGTGTGAACGAT
CCATTCTCTATTAACTGGAAAAAGGCTAAAAATACTCTGAAAAATCTTAGAGTTAAAGTC
CTCCCTTCAAATCAAGAATACAAGATAACCGGACTAAGTGGACTACACTGCAAAGATCAA
ACGTTTACTTGGAAGAAAAGGAACCAAAACAGGGAATTTGAGGAGGTTGAGATTACAGTG
TCCGATTACTTCACTAGGATCCGTGAAATCGAACTGCGTTACTCGGGTGGCTTACCTTGT
ATCAATGTTGGTAAGCCAAATCGTCCTACCTACTTTCCCATTGAGCTCTGTGAGCTTGTA
TCTCTACAACGCTATACTAAAGCGCTAACCAAATTCCAGAGGAGTAACCTTATCAAAGAA
TCAAGGCAGAATCCACAACAAAGAATTGGTGTGTTAACCAGAGCTCTCAAGACTAGTAAT
TATAATGATGACCCAATGTTGCAAGAATGTGGTGTTAGGATTGGCTCTGACTTCACTCAA
GTCGAAGGCCGCGTGTTACCAACACCAAAGCTTAAAGCTGGCAAAGAACAAGACATCTAT
CCTATTAATGGGAGTTGGAACTTCAAGAACAAGCCAGCCACAGTTACTAGATGGGCTGTT
GTGAACTTCTCTGCTCGCTGTGACCCGCAAAAGATTATTGATGATTTGACCAGATGTGGA
AAGATGAAAGGAATTAACGTAGATTCTCCATACCATGTCGTCTTTGAAGAAAATCCTCAG
TTTAAGGATGCCACTGGTTCTGTAAGAGTTGATAAGATGTTTCAACATTTACAATCAATA
CTCGGCGAAGTTCCTCCAAAATTCCTTCTATGCATACTCGAAAAGAAAAACTCTGATGTT
TATGAAAAATCTTGTTCAATGTGGAATTGTGAATGTATTGTTCCTCCTCAAAACTTAAAT
GATCAGTATCTCACAAATCTTCTACTAAAGATAAATGCCAAGCTTGGTGGATTGAATTCA
GTTTTGGATATGGAGCTGTCAGGAACAATGCCTCTGGTAATGAGAGTTCCTACCATCATT
ATTGGAATGGATGTATCTCATGGTTCTCCTGGACAGTCTGATCATATACCATCCATTGCC
GCGGTTGTGAGCTCCAGAGAGTGGCCACTGATCTCAAAATACAGGGCTTGTGTGCGTACA
CAGTCGCCTAAAGTTGAAATGATCGATAGCCTCTTTAAACCCGTCTCTGACAAAGATGAT
CAAGGTATCATGAGAGAGCTCTTGCTTGACTTTCACTCAAGTTCTGGAAAGAAACCGAAT
CACATTATCATTTTCAGGGATGGTGTCAGTGAATCTCAGTTTAACCAAGTTCTTAATATT
GAACTGGATCAGATGATGCAAATAAACCACCACACGAAGTTCTTCCAAACCGAAAGCCCT
AATAATGTTCTTCCAGGAACAATAATTGACAGCAACATCTGTCACCAACACAACAACGAT
TTCTATCTTTGTGCTCATGCTGGAAAGATTGGAACTACAAGGCCAACACATTACCATGTG
CTCTACGACGAGATTGGATTTGACACAGATCAACTCCAAGAACTTGTGCATTCACTATCC
TATGTCTACCAGCGGAGCACAACTGCCATCTCTCTTGTTGCGCCGATATGTTATGCTCAT
TTGGCGGCTGCACAGATGGCAACTGCAATGAAGTTTGAGGACATGTCTGAGACTTCATCG
AGCCACGGCGGGATCACCACAGCTGGAGCAGTCCCTGTGCCTCCCATGCCGAAGCTGAAC
ACCAACGTTGCTAGTTCAATGTTCTTTTGCTGA
>Arabidopsis-thaliana_AT5G21150.1
ATGGATTCTGATGAACCGAATGGGAGTGGATTACCACCTCCACCACCTTTCGTTCCAGCA
AATCTTGTCCCTGAAGTGGAGCCTGTAAAAAAGAACATTCTTCTCCCAATGGCTCGGCCT
CGAGGCAGCGGCTCCAAAGGACAGAAGATTCCTCTTCTTACTAATCACTTTGGAGTCAAG
TTCAACAAACCAAGCGGTTACTTCTTTCATTACAGTGTTGCTATCAATTATGAAGATGGC
CGTCCAGTGGAGGCTAAAGGTATCGGCCGAAAGATTCTTGACAAAGTTCAGGAGACCTAT
CAAAGTGATTTGGGTGCCAAATACTTTGCTTATGATGGCGAGAAGACTCTCTTCACTGTT
GGTGCTCTTCCCAGCAACAAACTTGACTTCTCTGTTGTTCTTGAAGAAATACCTTCTAGC
AGAAATCACGCTGGAAATGATACAAATGATGCTGATAGAAAAAGATCAAGGCGTCCCAAC
CAAACTAAGAAATTCATGGTTGAGATAAGTTATGCTGCAAAGATCCCCATGCAGGCTATT
GCAAGCGCTCTTCAAGGGAAGGAGACAGAGAATCTTCAAGACGCTCTGAGAGTGCTGGAT
ATTATTTTGCGCCAGAGTGCAGCTAGGCAAGGTTGCCTCCTTGTTCGCCAGTCCTTTTTC
CACAATGACGTAAAGAACTTTGTACCTATTGGTGGAGGTGTCAGTGGTTGCAGAGGGTTC
CATTCAAGTTTCAGAACTACTCAGGGAGGCTTATCCCTGAATATTGACACTTCAACTACG
ATGATAGTACAACCTGGACCTGTAGTTGATTTCCTGCTTGCTAACCAGAACAAGAAAGAT
CCATACGGAATGGACTGGAACAAGGCTCGACGTGTCCTCAAGAATCTGAGAGTTCAAATT
ACTCTTTCCAATAGAGAATACAAGATAAGTGGACTAAGTGAACACAGCTGCAAAGATCAA
CTATTTACATGGAGGAAACCTAACGACAAGGGAGAATTTGAGGAGGTTGAGATCACAGTG
CTCAATTACTATAAAGAGCGTAACATTGAAGTGCGTTATTCAGGTGACTTCCCTTGCATC
AATGTTGGTAAGCCGAAGCGTCCCACTTACTTCCCCATTGAGTTCTGTAATCTTGTGTCT
CTACAGCGATACACAAAATCGCTTACCAATTTTCAGAGGGCTGCCCTAGTTGAAAAGTCT
AGGCAGAAGCCACCTGAAAGGATGGCCTCGCTAACCAAAGGTCTGAAGGACAGCAATTAC
AATGCCGACCCGGTATTGCAAGATAGTGGTGTTAGCATTATCACCAATTTTACCCAAGTT
GAAGGCCGTATCTTACCAACACCAATGCTGAAAGTGGGCAAGGGAGAAAACCTTTCCCCA

ATCAAAGGAAAATGGAACTTTATGCGTAAGACACTCGCTGAGCCAACGACGGTTACTAGA
TGGGCTGTTGTGAACTTCTCTGCTCGCTGTGATACAAATACACTTATTCGTGACTTGATT
AAATGTGGACGGGAGAAAGGAATTAATGTAGAGCCTCCATTCAAGGATGTCATCAACGAG
AATCCTCAGTTTAGGAATGCACCAGCTACTGTGAGAGTAGAGAATATGTTTGAGCAGATA
AAATCCAAACTCCCAAAGCCGCCTCTGTTCCTTCTTTGCATACTCGCTGAAAGGAAAAAC
TCTGATGTTTATGGCCCTTGGAAAAAAAAGAATCTTGTTGATCTTGGAATTGTGACTCAG
TGCATTGCTCCCACCAGACTGAACGATCAGTATCTCACCAATGTTCTCCTGAAGATAAAT
GCCAAGCTTGGTGGATTGAATTCGTTGTTAGCTATGGAGCGCTCACCAGCAATGCCAAAA
GTAACGCAAGTTCCTACCATCATTGTTGGGATGGATGTATCCCATGGTTCCCCTGGCCAG
TCTGATATACCATCAATTGCTGCTGTTGTGAGCTCAAGACAATGGCCACTCATCTCAAAA
TATAAGGCATGTGTACGCACACAATCACGCAAAATGGAAATGATTGATAATCTCTTCAAA
CCCGTCAATGGCAAAGACGAAGGAATGTTCAGGGAACTCTTGTTAGACTTTTACTACAGT
TCAGAGAATAGGAAACCAGAGCACATCATTATTTTCAGGGATGGTGTAAGCGAGTCTCAG
TTCAATCAAGTTCTTAATATTGAATTGGATCAGATGATGCAGGCATGCAAGTTTCTTGAT
GATACGTGGCATCCGAAGTTTACAGTGATAGTTGCCCAGAAGAACCACCACACAAAGTTC
TTCCAGTCTCGAGGCCCTGATAATGTTCCTCCAGGAACAATCATTGACAGCCAGATCTGT
CACCCACGCAACTTTGATTTCTATCTCTGCGCCCATGCTGGCATGATTGGAACTACAAGG
CCAACACATTACCATGTTCTGTATGACGAGATTGGGTTTGCCACAGACGACCTCCAAGAA
CTTGTGCATTCTCTGTCCTATGTCTACCAGAGGAGCACCACTGCGATCTCAGTCGTTGCA
CCTGTATGTTACGCTCATTTGGCAGCTGCACAGATGGGAACTGTGATGAAGTATGAAGAG
TTGTCTGAGACTTCTTCGAGCCATGGAGGAATCACCACACCTGGAGCAGTCCCTGTGCCA
CCTATGCCGCAGCTGCACAATAATGTTTCAACCTCCATGTTCTTCTGTTGA
>Arabidopsis-thaliana_AT5G43810.1
ATGCCGATTAGGCAAATGAAAGATAGCTCTGAGACTCACTTAGTTATCAAAACCCAACCT
TTAAAGCACCACAATCCAAAAACCGTTCAAAACGGTAAAATCCCTCCTCCTTCTCCTTCT
CCGGTGACGGTGACTACTCCGGCGACGGTTACTCAGAGTCAAGCTTCTTCACCTTCACCA
CCGTCAAAGAATCGTAGCCGGAGGAGAAACCGTGGTGGAAGAAAATCTGATCAAGGAGAT
GTTTGTATGAGACCTAGCTCTCGTCCTCGTAAACCGCCACCGCCAAGTCAAACCACTTCC
TCCGCCGTCTCCGTCGCCACCGCCGGTGAGATTGTCGCTGTGAATCATCAGATGCAGATG
GGTGTTCGTAAAAACTCAAACTTTGCTCCAAGACCTGGATTTGGAACACTTGGAACTAAA
TGCATTGTTAAAGCTAACCACTTTCTCGCTGATTTGCCTACCAAGGATTTGAATCAGTAT
GATGTTACAATTACTCCTGAAGTGTCATCAAAGAGTGTTAACAGAGCTATAATTGCTGAG
TTAGTTAGACTTTACAAAGAGTCTGATCTCGGGAGGAGACTTCCGGCTTACGATGGCCGG
AAAAGTCTTTACACTGCTGGAGAACTTCCTTTTACTTGGAAGGAGTTCAGTGTTAAGATT
GTTGATGAAGATGACGGTATCATCAATGGCCCTAAAAGGGAGAGATCATATAAGGTGGCA
ATCAAGTTTGTTGCACGGGCAAATATGCATCACTTAGGCGAGTTTCTAGCTGGTAAACGG
GCAGATTGTCCGCAAGAGGCGGTGCAGATTCTTGATATTGTACTCAGGGAGTTGTCGGTT
AAGAGGTTTTGTCCCGTTGGAAGATCTTTCTTTTCGCCTGATATTAAAACACCGCAGCGA
CTCGGTGAAGGGTTAGAGTCATGGTGTGGGTTTTACCAGAGTATTAGACCAACTCAAATG
GGTTTATCACTAAATATCGATATGGCTTCAGCTGCATTCATCGAGCCTCTTCCAGTGATA
GAGTTTGTAGCACAGCTTCTTGGAAAGGATGTCTTGTCGAAGCCATTGTCGGATTCTGAT
CGCGTCAAGATTAAGAAGGGTCTTAGAGGAGTGAAAGTAGAGGTTACTCACAGAGCGAAT
GTAAGAAGGAAATACCGTGTTGCGGGTTTAACAACTCAACCAACAAGAGAGCTAATGTTT
CCAGTAGATGAGAACTGTACTATGAAGTCAGTTATTGAGTATTTCCAAGAGATGTATGGA
TTCACGATCCAGCACACGCATTTGCCATGTCTCCAAGTTGGAAACCAAAAGAAGGCAAGC
TATTTGCCGATGGAGGCATGCAAAATTGTCGAGGGACAACGGTACACGAAAAGGTTGAAT
GAGAAGCAGATTACTGCTCTCTTGAAAGTTACATGCCAAAGGCCGAGGGACAGAGAAAAC
GATATTTTGCGGACTGTCCAACACAACGCATATGATCAAGATCCATATGCAAAGGAGTTT
GGCATGAACATAAGCGAAAAGTTAGCTTCTGTTGAAGCTCGTATTCTTCCAGCTCCATGG
CTTAAGTATCACGAGAACGGGAAAGAAAAAGATTGTCTCCCGCAAGTTGGTCAGTGGAAT
ATGATGAACAAGAAAATGATCAACGGGATGACTGTGAGCAGATGGGCCTGTGTTAACTTC
TCACGCAGCGTTCAAGAAAACGTTGCTCGTGGATTTTGTAATGAACTTGGTCAGATGTGT
GAAGTCTCAGGCATGGAGTTTAATCCAGAACCCGTGATACCAATATATAGTGCGAGGCCC
GATCAAGTCGAGAAAGCTCTAAAGCATGTTTATCACACTTCAATGAACAAAACCAAAGGC
AAAGAGTTAGAGCTTCTGCTGGCAATATTACCTGATAACAACGGTTCACTTTATGGTGAT
CTTAAGAGAATCTGTGAAACCGAGCTTGGTTTGATATCTCAATGTTGTCTCACAAAACAT
GTGTTCAAGATTAGCAAACAGTATCTGGCAAATGTATCCCTTAAAATCAACGTAAAGATG
GGAGGAAGGAACACAGTTCTAGTAGACGCCATAAGCTGTAGAATACCACTGGTTAGCGAT
ATACCGACAATCATTTTTGGCGCAGACGTGACTCACCCAGAGAACGGGGAAGAGTCAAGC
CCTTCAATCGCTGCTGTTGTTGCTTCTCAAGACTGGCCTGAAGTGACAAAATATGCGGGT
TTAGTTTGTGCTCAAGCTCACAGGCAAGAACTTATACAAGATTTGTATAAAACATGGCAA
GATCCTGTTCGCGGTACTGTTAGTGGCGGTATGATCAGGGACCTTCTTATCTCATTTAGA

AAAGCAACAGGGCAAAAACCGCTTCGAATTATCTTTTATCGTGATGGAGTAAGCGAAGGG
CAATTCTATCAAGTTTTACTCTATGAGTTGGATGCAATTCGAAAGGCTTGTGCATCGCTT
GAACCGAATTATCAGCCACCGGTGACATTCATAGTTGTACAGAAGCGTCACCACACTCGT
TTGTTTGCTAATAATCACCGAGACAAAAACAGTACTGACCGAAGCGGAAATATCTTACCA
GGTACTGTAGTTGACACTAAAATATGTCATCCAACTGAATTCGACTTCTACCTTTGTAGC
CATGCGGGTATTCAGGGAACAAGCAGGCCTGCACATTACCATGTTCTTTGGGACGAGAAC
AATTTCACAGCAGATGGTATTCAATCTCTGACTAACAATCTCTGTTATACCTATGCGCGG
TGCACTCGGTCGGTCTCTATAGTTCCTCCAGCGTATTATGCTCATCTTGCAGCATTTCGA
GCACGTTTCTACCTGGAACCGGAGATAATGCAAGACAACGGATCACCGGGTAAAAAGAAC
ACGAAAACAACAACTGTCGGAGACGTAGGTGTGAAGCCTTTACCAGCCTTGAAGGAGAAT
GTGAAGAGAGTAATGTTCTACTGCTAA
>Arabidopsis-lyrata__16036850_locus
ATGGATTCAACAAATGGTAACGGTGCTGATCTTGAATCAGCAAATGGAGCAAACGGGAGT
GGGGTGGTTACTGATGCATTGCCACCTCCTCCACCAGTTATACCCCCAAATGTGGAACCA
GTTCGTGTTAAAACTGAACTTGCTGAGAAGAAGAACTTGCGAGTTCCTATGGCTAGAAAA
GGCTTTGGAACAAGGGGACAAAAGATTCCATTGTTGACTAATCATTTCAAAGTCGATGTG
GCTAATCTTCAGGGCCATTTCTTCCACTACAGTGTTGCTCTATTCTATGATGATGGTCGT
CCTGTTGAAGCAAAGGGTGTTGGAAGAAAAATCCTTGACAAGGTGCATGAGACTTACCAT
TCTGATCTAGATGGTAAAGAGTTTGCTTATGATGGTGAGAAGACGCTGTTTACATATGGA
GCTTTGCCTGATAACAAGATGGATTTTTCTGTGGTGCTTGAGGAAGTATCTGCTGCAAGG
ACTAATGGAAACGGTAGCCCCAATGGGAATGAAAGTCCAAGTGATGGTGATAGGAAAAGA
CTGCGTAGGCCTAACCGGTCCAAAAACTTTAGAGTTGAGATCAGCTATGCGGCCAAAATT
CCTCTACAAGCTCTTGCTAATGCAATGCGGGGACAGGAATCAGAGAACTCCCAGGAGGCA
ATACGGGTTCTTGATATCATATTGAGGCAACATGCTGCTAGACAAGGTTGCTTGCTTGTT
CGACAGTCTTTTTTCCACAATGATCCAAGTAACTGTGAACAAGTTGGTGGTAACATCTTA
GGCTGTAGGGGATTTCACTCCAGTTTCAGAACAACGCAGGGTGGCATGTCACTTAATATG
GATGTTACAACCACCATGATCATCAAGCCTGGTCCAGTGGTTGATTTCCTAATTGCTAAC
CAAAATGCGAGGGATCCTTATTCGATTGACTGGTCTAAGGCTAAAAGAACCCTTAAGAAC
CTAAGGGTTAAGGTCAGCCCCTCAAACCAAGAATTCAGGATCACCGGATTGAGTGACAAG
CCTTGCAGGGAACAAACGTTTGAATTGAAGAAAAGGAACCCAAATGAAAATGGAGAGTTC
GATACTACTGAAGTGACAGTTGCTGACTACTTCCGCGAAATAAGGCATATTGATTTGCAA
TATTCTGCGGATCTTCCGTGCATCAATGTTGGGAAGCCAAAGCGACCCACTTACATTCCT
CTTGAGCTCTGCGCCTTGATTCCACTTCAGAGGTACACAAAAGCACTTAACACATTCCAA
AGATCTGCCCTTGTTGAGAAATCCAGACAGAAACCCCAAGAGAGGATGACTGTTCTGTCC
AAAGCTCTTAAAGTTAGCAACTATGATGCTGAACCACTCCTGCGTTCCTGTGGCATTTCG
ATCAGCTCCAACTTTACTCAGGTGGAGGGTCGTGTTCTACCAGCTCCCAAGCTGAAAATG
GGATGTGGATCTGAAACCTTTCCCAGAAATGGTCGCTGGAATTTCAACAACAAGCAATTT
GTTGAGCCCACCAAAATTGAACGATGGGTTGTTGTCAATTTCTCCGCTCGCTGTAATGTA
CGCCAAGTTGTTGATGATCTGATCAAAATTGGGGGATCAAAAGGAATTGAAATTGCTCCT
CCCTTTCAAGTGTTTGAGGAGGGTAATCAATTCCGCCGCGCTCCTCCTATGAATCGTGTT
GAGAACATGTTTAAGGACATCCAGTCTAAACTCCCTGGTGTCCCACAATTCATACTATGT
GTGCTCCCAGAGAAAAAGAACTGCGATCTCTACGGTCCATGGAAGAAGAAAAACTTAACT
GAATTCGGCATTGTTACTCAATGCATGGCCCCGACGCGGCAACCTAATGATCAGTATCTT
ACAAACTTACTTTTGAAGATTAATGCTAAGCTTGGAGGCCTGAACTCAATGTTAAGTGTA
GAGCGTACACCTGCCTTCACTGTGATTTCCAAGGTTCCAACCATTATCCTTGGGATGGAT
GTTTCACATGGATCTCCTGGACAGTCTGATGTCCCGTCCATTGCTGCTGTGGTGAGTTCG
AGGGAGTGGCCACTAATCTCCAAATATAGAGCATCTGTTCGGACACAGCCTTCTAAGGCT
GAGATGATTGAGTCCCTTTTCAAGAAAAATGGAACTGAAGACGATGGCATCATCAAGGAG
TTGCTGGTTGATTTCTACACCAGCTCGAATAAGAGAAAGCCAGAACATATCATAATTTTC
AGGGATGGTGTGAGTGAATCTCAATTCAATCAGGTTCTGAATATCGAACTCGATCAGATC
ATCGAGGCTTGCAAGCTCCTTGATGCAAATTGGAATCCAAAATTCCTCTTGTTGGTGGCT
CAAAAGAATCATCATACAAAGTTCTTCCAGACATCGTCTCCTGATAATGTTCCCCCAGGG
ACAATCATTGACAACAAAATATGTCACCCAAAGAACAATGATTTCTACCTCTGTGCTCAC
GCTGGAATGATTGGAACTACCCGTCCAACGCACTACCATGTCCTGTATGATGAGATTGGT
TTTTCACCTGATGAACTTCAGGAACTTGTCCACTCGCTCTCCTATGTGTACCAAAGAAGC
ACCACTGCCATTTCAGTTGTTGCGCCGATCTGCTATGCTCACTTGGCAGCTGCTCAGCTT
GGGACGTTCATGAAGTTTGAAGATCAGTCTGAGACATCATCAAGCCATGGTGGAATCACA
GCTCCAGGACCAATCTCTGTTGCACAGCTCCCAAAACTCAAAGACAACGTCGCCAACTCC
ATGTTCTTCTGTTAA
>Arabidopsis-lyrata__16039685_locus
ATGGAGAGAGGTGGTTATCGAGGTGGTCGTGGTGATGGCCGTGGTAGAGGCGGCGGAGGA
GGAGGCCGTGGCTATGGCGGCGGCGGAGGAGGAGGAGGAGGAGGAGAACAAGGTCGTGGT

CGTGGCTATGGCGGCGGAGGAGGAGAACAAGGTCGTGGTCGTGGCTCAGAGCGTGGCGGT
GGAAATCGTGGGCAGGGACGTGGTGAACAGCAGGATTTTCGTAGCCAGAGTCAGTGGGGA
CCTCCGCCAGGTCACGGTGGTCGTGGTACGCAGTCGCAACAACCTCGACCACAGGTGGTT
CCGCAGCCGTCGCAGGCTCCGGTGAGTTATGCTGGTTCGGTAGGAGGAGTCGCTGGTAGA
GGCGCGTGGGGTCGTAGGCCACAGGTTTCTTCTGATTCGGCTTCTCCGTCCACCACCGTC
GTGGTTTCTGAGCCCGTTCGTGTAGCTGAAGTTATGAATCCGAGGCCATCGGTGCAAGTT
GTGTCTTCTGATAGGAAAGAACCGATGAAGCGACCTGATAGAGGCGGTGTTGTGGCTGTG
CGGCGGGTTAATCTCTATGTGAATCATTTTAGAGTGAATTTCGATCCGGAAAGTGTCATA
AGACATTATGATGTTGAAATCAAAGGAGAAAATCCTACGAAGAAGATTTCGAGGTTTGAG
CTAGCTATGGTTAGGGACAAGGTGTTCACTGATAATCCTAATGAGTTTCCCTTCGCTATG
ACAGCTTATGATGGTCAGAAGAACATTTTCAGCGCAGCTGAATTGCCTACAGGTTCATAT
AAGGTGGAGTTCCCTAAAACTGAAGAGATGAGAGGTCGAAGCTATACATTCACTATCAAA
CAGGTGAATGAGCTTAAGCTACGCGACTTGAAAGAGTACATGACAGGGGGTTCGTCTTGC
AATCCGCGTGATGTGTTGCAAGGAATGGATGTTGTGATGAAGGAGCATCCTTCAAAGTGT
ATGATCACTGTTGGTAAAAGCTTTTTCACTCGTGAAACTGAGCGAGATGAAGACTTTGGC
TTCGGGGTTGCAGCTGCGAAAGGGTATCGCCACACTCTGAAGCCCACAGCACAAGGTTTG
TCTTTGTGTTTGGACTACTCAGTGTTGGCGTTCCGCAAAGCAATGTCGGTCATTGAATAC
CTGAAGTTGTACTTTAACTGGTCCGATATGCGTCAGTTTAGGAATTGTAGGCGTGATGTG
GAAAAGGAACTGACTGGTTTGAAAGTCACTGTGAATCATCGGAAGAACAAGCAGAAACTC
ACCATTGTAGGGCTGAGTATGCAAGACACAAAAGACATCAAATTCGATCTTATTGATCAA
GAGGGAAACGAGCCACCAAGGAAAACGTCCATTGTTGAGTATTTCAGGATAAAGTATGGA
AGAGACATTGTTCACAAAGATATTCCTTGCTTGGATTTGGGGAAAAACGGTAGGCAAAAT
TTTGTCCCCATGGAGTTCTGCGACTTGGTTGAGGGACAGATTTATCCAAAGGACGACTTG
GATAAAGATTCAGCTTTGTGGTTAAAAAAGTTGTCGCTAGTCAATCCACAACAAAGGCAG
AGGAATATAGATAAGATGATAAAGTCTCGTAATGGACCGAGCGGTGGAGAAATAATTGGA
AACTTCGGATTGAAAGTGGATACAAACATGACACCGGTGGAAGGTCGTGTACTCAAGGCT
CCAACATTGAAGTTGGCAGAGAGAGGGAGAGCTGTGCGTGAGGAACCCAACCCAAGACAG
AACAACCAATGGAACCTTATGAAGAAGGGAGTCACAAGGGGATCTATAGTCAAGCATTGG
GCTGTACTTGACTTCACCGCATCCGAGAGATTTAACAGGATGCCTAATGACTTTGTGGAT
AACCTCATCGACCGTTGTTGGAGACTTGGGATGCAGATGGAGGCTCCTATAGTTTACAAA
ACATCGAGAATGGAAACACTTTCTAATGGTAATGCTCTTGAGGAATTGCTTCGATCCGTG
ATAGATGAGGCTTTTCGTAAGCATGATGGGGCTCGTCCAACTCTTGTTCTGTGTGCTATG
TCTCGGAAGGACGATGGCTACAAGACTCTGAAATGGATAGCCGAGACCAAACTTGGTCTT
GTGACTCAGTGTTTCTTGACCGGTTCTGCCACTAAAGGAGGTGATCAGTACTGGGCAAAT
CTTGCCCTCAAGATGAATGCAAAGGTTGGTGGAAGCAACGTTGAGTTGATGGATACGTTC
TCTTTTTTCCAAAAAGAGGATGAGGTCATGTTCATTGGTGCCGATGTCAATCATCCTGCT
GCTCGGGACAAGATGAGCCCGTCTATTGTTGCTGTTGTGGGTACTCTAAACTGGCCTGCA
GCTAACCGTTATGCAGCTAGAGTCATTGCCCAGCCTCACCGTAAAGAGGAGATACAAGGA
TTTGGCGATGCTTGCCTGGAGCTTGTCAAAGCTCATGTTCAGGCCACCGGGAAACGACCT
AACAAGATTGTGATATTCCGTGATGGTGTCAGCGACGCTCAGTTCGATATGGTTCTCAAT
GTGGAGTTGCTTGATGTTAAGCTGACTTTTGAGAAGAATGGTTACAATCCAAAGATAACG
GTAATCGTAGCCCAGAAACGCCATCAAACCCGTTTCTTCCCTGCCACAAGCAATGATGGA
AGTGATAAAGGCAATGTGCCTTCAGGTACGGTCGTTGATACTAAAGTCATTCACCCATAT
GAGTATGATTTCTACATCTGCAGTCACCATGGAGGGATCGGGACAAGCAAACCGACTCAT
TATTACACTCTTTGGGACGAACTTGGATTCACTTCAGATCAGGTTCAGAAGCTCATCTTT
GAGATGTGCTTCACTTTCACTCGCTGCACCAAACCTGTCTCTCTTGTTCCTCCGGTGTAT
TATGCTGACATGGTTGCCTTTAGAGGAAGGATGTACCACGAGGCAAGCTCTCGAGAGAAG
AACTTTAGGCAGCCGTGGGGAGCGTCTACCTCAGCTGCTTCGCTTGCCTCTTCATTATCT
TCTCTTACAGTTGAGGACAAAGCGATATTCAAGCTGCATGCAGAGCTTGAGAACGTTATG
TTCTTCGTCTGA
>Arabidopsis-lyrata__16042271_locus
ATGGAAGAAAAAACTCAGAGTCATCATCATCATCACAGTTCCAACAAAAACAACCCCAAT
TCCAAATCAAGAACACCTCTTCTCCACAAGCCTTGTCATCATCACCATGTTCAAACCAAT
CCTCCTCCTTTTCTTTTGCACCCTTCTTCTCACCAAAACCTCAATCTTGTTGCCTCAAAT
CTTCCTTCTAGCTACTACTACTATTACTACTGCTACTTCTACTCTCAATTTCACAACTCT
CTTCCTCCTCCTCCTCCTCTTCTTCCTCTTCCTCCTCCTCCTTTACTTCCTCTTCCTCCT
CCTCACTCAATGACCCGTTTCCACAAATCTCTCCCTGTTTCTCAAGTTGTGGAGAGAAAG
CAGCAACAACAGCAGAAGAAGAAGATACAAGTTTCTAACAACAAAGCGAGTGGGTCCATA
GCAATAGAAGAAGCAGCATTAGTGGTTGCTAAAAGACCTGACTTTGGTGGTCAAGAAGGT
TCTGTCATTTATCTCCTCGCCAACCATTTTCTTGTCAAGTTTGATTCTTCACAGAGGATT
TACCATTACAACGTTGAGATCTCTCCTCAGCCTTCAAAGGAAATTGCTCGGATGATCAAA
CAGAAGCTTGTGGAGACCGATGTGAATAGTTTCTCCGGTGTTGTTCCGGCTTTTGATGGT

AGGCAAAACATTTATAGTCCTGTGGAGTTTCAGGGCGATAGGCTTGAGTTCTTTGTTAAT
CTCTCTATCCCATCTTGCAAGGGTGTAATGAATTACGGTGACTTGCGAGAGAAGCAACCT
CAGAAGAAGATTGATAAACTGTTTAGGGTTAACATGAGGCTTGTGTCTAAGTTTGATGGT
AAGGAACAGAGAAAGGAAGGAGAAGATTGGGCTCCTCTGCCTCCAGAATACATTCATGCT
CTTGATGTTATCTTGAGGGAGAATCCAATGGAGAAGTGTACGTCGATTGGAAGATCGTTT
TACTCGAGTTCTATGGGTGGTTCCAAGGAGATTGGAGGAGGAGCTGTTGGACTCAGAGGG
TTTTTCCAGAGTCTTAGGCAGACTCAACAAGGTTTAGCACTTAACATGGATCTCTCAATC
ACAGCTTTCCATGAAAGCATTGGAGTAATAGCTTACTTGCAGAAGCGGCTCGAGTTTCTC
AAGGACCTTTCTAGGAACAAAGATACAGAATTGAATCTAGAGGAAAAGAGAGAAGTGGAG
AAAGCACTTAAGAACATAAGAATCTTTGTTTGCCATAGAGAAACAGTTCAAAGGTATCGG
GTTTACGGGTTAACAGAGGAGATTACCGATAATATATGGTTTCCGGATAGAGATGGGAAG
AACTTAAGGCTTATGAGCTACTTTAAAGATCATTATGGTTATGAGATTCAGTATAAGAAC
TTACCGTGTCTGCAAATCAGTAGGGCAAGACCTTGCTACCTTCCTATGGAACTATGTATG
ATCTGTGAAGGTCAAAAGTTTCTTGGGAAGCTTTCGGATGATCAAGCTGCAAAGATCATG
AAAATGGGCTGCCAAAAACCAAATGAAAGGAAAGCTATTATTGATAAGGTTATGACAGGA
TTGGTCGGTCCGTCGAGCGGAAACCAAACAAGAGAATTCAACCTCGAGGTTTCAAGAGAA
ATGACATTGCTGAAAGGAAGAATACTTCAGCCTCCAAAGCTTAAACTTGACCGGCCAAGG
AACCTTAAAGAAAGTAGAGCTTTTAAAGGAACCCGGATCGAGAGATGGGCTTTGATGAGT
ATTGGAGGCAGCTCGGATCAGAAATCTACAATTTCCAAGTTCATAAACGAGCTCACTCAA
AAGTGTGAGCATTTGGGAGTCTTCTTAAGCAAGAACACATTAAGCAGCACTTTCTTTGAA
CCATCACACATACTCAACAACATTTCGCTTCTCGAATCGAAACTGAAGGAGATTCAAAGA
GCGGCGTCCAACAATCTCCAGCTGATTATCTGTGTAATGGAGAAAAAACATAAAGGGTAC
GGAGATCTTAAGCGAATAGCAGAGACAAGAATTGGTGTTGTGACGCAATGCTGCTTATAC
CCTAACATCACTAAGCTCAGTTCTCAGTTCGTTTCAAACTTAGCTCTCAAGATAAACGCC
AAGATCGGCGGATCCATGACCGAGCTCTACAACTCAATACCTTCTCACATCCCAAGACTA
CTTAGACCCGATGAGCCGGTTATCTTCATGGGAGCTGATGTAACGCATCCTCATCCATTC
GATGACTGTAGCCCTTCAGTAGCGGCTGTGGTCGGGAGCATAAACTGGCCAGAAGCTAAC
AGATACGTCTCGAGAATGAGATCTCAGACTCATAGGCAAGAGATCATTCAAGATCTTGAC
TTGATGGTCAAGGAACTTCTTGACGATTTCTACAAAGCGGTAAACAAGCTTCCGAATCGA
ATCATATTCTTCAGAGACGGTGTGAGCGAGACACAGTTCAAGAAAATCCTCCAAGAAGAG
CTTCAATCCATCAAAATCGCTTGCTCTAAGTTCCAGGATTACAATCCAAGCATCACATTC
GCCGTGGTCCAGAAAAGACACCACACAAGGCTGTTCCGGTGCAATCCAGACAATGAGAAC
ATCCCTCCTGGCACAGTGGTTGATACAGTGATAACTCATCCGAAAGAGTTTGATTTCTAT
CTCTGTAGCCACTTAGGAGTAAAAGGCACGAGCAGGCCAACGCATTACCACATCCTATGG
GACGAGAACGAGTTTACTTCAGACGAATTGCAGAGACTTGTGTATAACTTGTGTTACACT
TTCGTGAGGTGCACGAAACCTATTTCGATTGTACCACCGGCTTATTATGCACACCTTGCT
GCGTACAGAGGAAGGCTCTACATCGAGAGATCATCTGAATCTAATGGAGGATCCATGAAT
CCTTCTTCTGTCTCTCGAGTTGGTCCTCCAAAGACGATTCCTCTCCCTAAATTAAGTGAT
AATGTCAAGAATCTCATGTTTTACTGCTGA
>Arabidopsis-lyrata__16047041_locus
ATGGAACGGGAATCTGTGAAGAACAAGAGTATACTTCTTCCAATGGCTCGGCGTGGCAAC
GGTTCCAAAGGAAAGAAGATTCATCTGCTCACTAACCACTTTAGAGTTAACTTCAGTCAA
CCAAACAATCATGATTTCTTCCATTACAGCGTTGCTATCACATATGAAGATGGTAGTCCA
GTTGAAGCCAAGGGTATTGGTAGAAAGATTCTTGAAAAAGTTCAACAGACTTATCAAACT
GATTTGGGTTTCAAACACTTTGCTTATGACGGCGACAAGAATCTTTTCACTGCCGGTCCT
CTTCCCGGATCCAATCTAGAATTCTCTGTTGTTCTTGAAGACATGTCTTCTAGGAGAAAT
GCAGGCAAAAGACTAAGGCTTTCTCACCAATCCAAGAAATTCAATGTTGCAATCAGCTTT
GCTGCAAAAATCCCTATGAAAGCTATTGCAAATGCTCTTCAAGGAAAGGAAACAAACCAT
TTTCAAGATGCTATCAGAGTGATGGATGTTATTTTGTGCCAAAATGCAGCTCGGAAAGGT
TGCCTCCTAGTTCGCCAATCTTTCTTCCACAATGATGCAAAGTACTTTACGAATATCGGT
GAAGGTGTTGATTGTTGCAAAGGATTCCATTCAAGCTTCAGAACTACTCAGGGAGGCTTG
TCCCTCAATATTGATGTTTCGACTACGATGATAGTAAAACCTGGTCCTGTTGTTGGTTTT
CTTATTGGAAACCAAGGTGTGAAAGATCCATTCTCTATTAACTGGAAAAAGGCTAAAAGT
ACTCTCAAGAATCTTAGAGTTAAAGTCATCCCCTCAAATCAAGAATACAAGATAACCGGA
CTAAGTGGACTACACTGCAAAGATCAAATGTTTACTTGGAAGAAAAAGAACCAAAACGGG
GAAGTTGAGGAGGTTGAGATTACAGTGTTTGATTACTTCACCAAAATCCGTGACATCAAA
CTGCATTATTCAGGTGGCTTACCATGTATCAATGTTGGTAAGCCAAATCGTCCTACCTAC
TTTCCCATTGAGCTCTGTGAGCTTGTATCTCTACAACGCTATACTAAAGCTCTTACAAGT
TTTCAGAGGAGTAACCTTGTCAAAGAATCAAGGCAAAACCCTCATCAAAGAATGGAAGTG
TTAACCAGAGCTCTCAAGAACAGTAATTATAGTGATGACCCAATGTTGCAAGAATGTGGT
GTTAGAATAGGCTCTGACTTCACTCAAGTCGAAGGTCGCTTGTTACCAACACCCAAGCTT
AAAGCTGGCAATGAACAAGACATCTATCCTAAGAATGGGCGTTGGAACTTCAATAACAAG

ATAGTTTTTGAGTCAGCAACAGTTACTAGATGGGCCGTTGTGAACTTCTCTGCTCGCTGT
GACCCGCGCAAGATTGTTCGTGATTTGATCAGATGTGGAAATATGAAAGGAATTAACGTA
GATCCTCCATACAAAGTTGTCTTTGAAGAAGATCCTCAGTTTAAGGATGCACCAGGTTCT
GTAAGAGTTGAAAAGATGTTTGAACGTTTAGAATCCACACTCGGTGACGTTCCTCCAAAT
TTCCTTCTATGCATACTCGAAAAGAAAAACTCTGATGTTTATGGTCCTTGGAAAAAGAAA
AATCTTGTTCAAGTTGGAATTGTGAATCAGTGTATTGCTCCTCCTCAAAACGTTAATGAT
CATTATCTCACAAATGTTCTCCTCAAGATAAATGCCAAGCTTGGTGGATTGAATTCAGTG
TTGGATATGGAGCGGTCACGGGCAATGCCTTTGGTAATGAAAGTTCCTACCATCATTATT
GGGATGGATGTATCTCATGGTTCTCCTGGACAGTCTGATGTACCATCCATTGCCGCGGTT
GTGAGCTCCAGAGAATGGCCACTGATCTCAAAATACAGGGCTTGTGTGCGTACACAGTCG
CGTAAAGTTGAAATGATCGATAACCTCTTTAAACCAGTCTCTGACAAAGATGATGAAGGT
ATCATGAGGGAGCTCTTGCTTGACTTTTACTCAAGTTCTGCAGTCAAACCCAATCACATT
ATTATTTTCAGGGATGGTGTGAGTGAATCTCAGTTTAACCAAGTTCTTAATATTGAACTG
GATCAGATGAAGCAAAAAAACCACCACACCAAGTTCTTCCAGACCAGGAGCCCTGATAAT
GTTCCTCCAGGAACAATAATTGATAGCAACATCTGTCACCAACACAACAACGATTTCTAT
CTTTGTGCTCATGCTGGAATGATTGGAACTACAAGGCCAACACATTACCATGTGCTGTAT
GACGAGATTGGATTTGACACAGATCAACTCCAAGAACTTGTGCATTCCCTATCCTATGTC
TACCAGCGGAGCACAACTGCAATCTCTCTTGTTGCGCCGATATGTTATGCTCATTTGGCG
GCTGCACAGATGGGAACTGCGATGAAGTTTGAGGACATTTCTGAGACTTCGTCGAGCCAC
GGCGGGATCACCATAGCTGGAGCAGTCTCTGTGCCACCAATGCCGAAGCTGAACACCAAG
GTTGCTAGTTCAATGTTCTTCTGCTGA
>Arabidopsis-lyrata__16051341_locus
ATGGAGCGAGGTGGTTACCGAGGAGGTCGTGGTGATGGCCGTGGTAGAGGCGGCGTCGGA
TATCGTGGTCGTGGTTACAGCGGTCGTGGTCGTGGCTTCGATCAAGACAGAGATGGTGGT
TTGGTAAATCGAGGGCAGAGTTCCGGTGGTCAGGTTCACGGTGGTCGTGGGACGCAATTG
CAACAACCTCGGCCACAGGCGGTTTCTCAGTCGTCTTCGCAGGTTCAAGTGACTCAAGGC
GTTGTAGCCGGAGGCGTGGGTAGAGGCGGCGTCGGAGACCGTGGTCAGAGTCAGTTGGGA
CCATCGTTTGGTCACGGTGGTCGTGGGACGCAGTTGCAACAACCTCGGCCACAAGCGGTT
CCTCCGTCGACGTCGCAGGCACGAGTGAGTCAAGCCTTTGCAACCGGAGGCGTGGGTAGA
GGTGGCGTTGGAGACCGTGGTCGTGGTTATGGCGGAGGTGAACGCGGTCGTGGTCGTGGC
TTCAATCAAGGCGGCGATGGTGGTTTGGTAAATCGTGGGCAGAGTTCCGGTGGTCAGAGT
CAGTTGGGACCATCATCTGGTCGGCCACAGCCGATTCCTCAGTCGTCGTCGCAGGCTCAA
GTGGCTCAAGCCGTTGCAGCAGGAGGCGCGTGGGCTCGTAGGCCACAACTTTTCTCTGAT
TCGGCGGTCTTGCCTTCTTCTTCGTCGACCGTCGTGGCTTCTCAAACCGCTAGTGGTTCT
CAAGTTATGACTCCGAAGCCATCTTCTTCTGATAAGAAAGAACCAATGAAGCGTCCTGAT
AGAGGTGGTAGTAAGCTTGTGCAACGCGTAAATCTCTCTGTCAATCATTTCAATGTGAGT
TTCCCTTCTGAAAGTGAAAGTGTCATAAGGCACTACGATGTTGATATCAAAGGAGAAAAT
CCTTTAAAAAAGATATCAAGATATGAGCTAGCTATGGTCAAAGAGAAGGTGTTCACCGAC
AATCCCGATAAGTTTCCCTTCGCTATGACAGCTTATGATGGTCAGAAGAACATTTTCAGC
GCAGCTGAATTGTCTACAGGTTCATATAAGGTGGAGTTCCCTGAAACTGATGAGATGAGA
GCTCGAAGCTATACATTCACCATCAAACAGGTGAATGATGAGCTTAAGCTACGTGACTTG
GAAGACTACATAAGGGGAAGTTCCTCTTTCATTCCGCGTGATGTGTTGCAAGGAATGGAT
GTTGTGATGAAGGAACATCCTTCAAAGCGTATGATGACTGTTGGTAAAAGCTTTTTCACT
CGTGAGCCAGATGAAGACTTTCGCTTCGGGGTTGTAGCTGCGAAAGGGTATCGCCACACT
CTAAAGCCCACAGCACAAGGTTTGTCTTTGTGTTTGGACTACTCGGTTTTGGCATTCCGC
AATGCAATGTCGGTCATTGACTACCTGAAGTTGTACTTTGGCTGGTCCGATATGCGTCAG
TTTAGGAATTGTAGGCGTGATGTGGAAAAGGAACTGACTGGTTTGAAAGTCACTGTTAAC
CATCGGAAGAACAAGCAGAAACTCACCATTGTAGGGTTGAGTGAGTACAACACAAAAGAT
ATCACATTCGATCTTATTGATCATGCGGGAGACGAGCCTCCAAGGAAAGTATCCATTGTT
AAGTATTTCATGGAAAAGTATGGAAAAGACATCCGTTACAAGGATATTCCTTGCCTAAGT
TTGGGGAAAAAAGGTCGGCAAAATTATGTACCCATGGAGTTTTGTAACTTGGTCGAGGGG
CAGATTTATCCAAAAGAAAAATTGAAGGGTAATTCAGCTTCCCGGTTAAAACACTTGTCC
CTTGTCAATCCACAACGAAGGAAGGAGAATATAGAAAATATGATAAAGCTTAGGGATGGA
CCAAGCGGTGGAGATATCATTGGGAATTTTGGACTGAAAGTGGCTACAAACATGACAACG
GTGGAAGGTCGTGTACTCAAGGCTCCTACACTGATGTTGACTGATCAGAAAGGAAATCCT
GTCACTGAGGAACCCCGCAAGAACAATCAATGGAACCTTACAATAAAGCGAGTCACAAAG
GGATCAAAAATCAAGCATTGGGCTGTTCTTGACTTCACCGCGTCTAAGAAGCCGCACAAT
TACAAGATGCCTGATAACTTTGTTGAGGAACTCACTGCGCGTTGTTCCAGACTTGGGATG
ACGTTGGAGAATCCTATCGTTCGCAAAACATTAAGTATGGATACGCTTTCTAATGGTAAT
GATCTTGAGGAATTGCTTCGATCCGTGATTGATGAGGCTTTACTTAACTATCGGGCTCGT
CCAACTCTTGTTCTGTGTGCTATGTCTGGGAAAGTCGATGGCTACAAGACTCTGAAATGG
CTAGCCGAGACCAAACTTGGTCTGGTGACTCAGTGTTTCTTGACCGGTTCTGCGAATAGG

GGAGGTGATCAGTACTTGGCAAATCTCGCCCTCAAGATAAACGCAAAGGTTGGTGGAACG
AACGTGGAGCTGGTGGACAATTATTTCTCTTTCTTCAATAAAGAAGATGAGGTCATGTTC
ATTGGTGCTGATGTCAATCATCCCGCTGCTCACGACAAGATGAGTCCATCCATTGTTGCT
GTTGTAGGCACTCTTAACTGGCCTGAAGCTAACCGCTACGCAGCTAGAGTCAAAGCTCAG
ACTCACCGTAAAGAAGAGATACAAGGGTTTGGTGAAACTTGCTTAGAGCTTGTCAATGCT
CATTCTAATGCCACCAAGAAACGACCTAACAAGATTGTGATATTCCGTGATGGTGTCAGT
GATGGTCAGTTCGATATGGTTCTCAATGTGGAGTTACAGAATGTTAAGGACACTTTTAAA
AAGATTGAATATAATCCGTTGATAACGGTAATTGTGGCACAGAAGCGTCATCAAACCCGT
TTCTTCCCTGCCACAAGCAATGATAAGGACAATGTGCTTTCAGGTACGGTCGTTGATACA
AAGATCATTCACCCATTTGAGTATGATTTCTATCTCTGTAGTCACCATGGAGCGATTGGG
ACAAGCAAACCAACTCATTACTATGTTCTGTACGACGAAATCGGGTTCAAGTCGGATCAG
ATTCAGAAGCTCATATTTGACGTGTGCTTCACGTTTACTCGCTGCACCAAACCTGTCGCT
CTGGTTCCTCCAGTTTCTTATGCTGACAAGGCTGCTTCTAGAGGAAGGTTGTACTACGAG
GCAAGCTTTATGGAGAAGAACTCTAAGCAGTCGCGTGGAGCGTCTTCATCTTCTGCTGCT
TCGGTTGCCTCTTCATCCTCTTCTCTCACAGTGGAGGACAAAGAGATCTTCAAAGTTCAC
ACAGAGATTGAGAACAATATGTTCTTCGTCTGA
>Arabidopsis-lyrata__16055324_locus
ATGGAGAAATCTTCATCTCTGCCACTGTCACCCATAAGTATAGAACTAGAAAAGCCCAGC
CATCGAGATTATGACATCACAACTAGACGTGGGGTTGGAACCACAGGCAACCCAATAGAA
TTGTGCACCAATCACTTTAATGTTTCTGTGAGACAGCCGGATGTAGTATTTTACCAGTAC
ACTGTTAGTATCACCTCAGAAAATGGTGATGCTGTCGATGGGAAAGGAATAAGCAGAAAG
CTTATGGACCAACTATTCAAGACTTACTCTTCTGACCTTGATGGTAAAAGGTTGGCATAT
GATGGAGAGAAGACTTTATATACTGTTGGTCCTCTGCCACAGAACAACTTTGACTTTCAA
GTCATCTTGGAAGGTTCATTTTCAAAACGTGATTGTAGTGTCTCTGATGGTGGTAGTCCT
TCCGGTACTTGCAAGAGATCAAAACGTTCTTTCTTGCCAAGAAGTTACAAGGTTCAGATA
CACTTTGCTGCAAAAATACCGTTGAAGACTATTCTTGTCACTCAGAGAGGATCTTATACA
GCAGATAAGAGTGCTCAGGATGCACTTAGAGTGCTTGACATTGTACTGAGGCAGCAAGCA
GCTGAAAGGGGATGCCTTTTGGTCAGGCAGGCTTTTTTCCATAGCGATGGCCACCCCATG
GAAGTTGGAGGAGGTGTAAAAGGTATTCGAGGTTTCCATTCGAGCTTTCGTCCAACCCAT
GGAGGACTCTCGCTTAACATTGATGTGTCAACAACAATAATCTTAGAACCGGGTCCAGTT
CTTGAATTCCTGAAAGCCAATCAGAGTGTTGAGACACCACGACAAATCGACTGGGTTAAG
GCTGCGAAAATGCTTAAACATATGAGGGTAAAGGCAACACACCGTAACATGGAATTTAAA
ATTATAGGTCTAAGTCAGAAACCGTGCAATCAGCAACTGTTTTCGATGAAGATTAAAGAT
GGTGAACGTGAAGGACAGACTAGAGATATTACAGTGTATGACTATTTCAAGCAAACTTAC
ACAGAACCTATTTCTTCTGCGTACTTGCCATGCCTTGATGTTGGCAAGCCAAATCGCCCC
AACTATCTTCCACTGGAGTTTTGTAATCTTGTATCTCTGCAACGTTATACAAAAGCATTG
TCCGGAAGGCAAAGAGCTCTACTTGTTGAAAAATCAAGACAAAAACCTCTTGAGAGAATT
AAGACACTCAATGATGCAATGCATACCTACTGCTTTGACAAGGACCCATTTTTGGCTGGA
TGTGGGATCTCCATTGAAAAACAAATGACTCAAGTTGAAGGCCGGGTTCTCAAGCCCCCA
ATGCTGAAGTTTGGCAAGAATGAAGATTTTGAACCTTGCAACGGACGGTGGAACTTTAAC
AACAAGATGCTTCTGGAGCCAAAAGCTATTAAAAACTGGGCTATTGTCAACTTTTCTTTT
CCATGCGACAGTAGTCACATTTCCCGTGAGCTTATAAGCTGTGGCATGAGGAAAGGCATT
GAAATTGATCGACCTTTTGCACTAGTTGAAGAGGATCCACAGTATAAAAAAGCAGCTCCT
GTTGAAAGGGTAGAAAAGATGATTGCAAAGATGAAGCTAAAATTTCCAGATCCTCCTCAT
TTTATCCTCTGTGTCCTGCCAGAACGGAAAACTTCTGATATCTATGGTCCCTGGAAGAAG
ATATGCCTCACTGAAGAAGGGATCCACACACAATGCATCTGCCCTGTCAAGATCAGTGAC
CAATATCTCACCAATGTACTTCTGAAGATTAATTCAAAGCTTGGAGGCATCAATTCTTTG
TTGGGAATAGAGTACTCTTACAACATTCCATTGATAAACAAAATTCCCACCTTGATTTTG
GGTATGGATGTATCTCATGGGTCTCCAGGTCGGGCAGATGTTCCTTCAGTAGCAGCGGTT
GTTGGTTCAAAATGCTGGCCCTTAATCTCAAGGTATAGGGCAGCTGCAAGAACTCAGTCA
CCACGTCTGGAGATGATTGATTCGCTCTTCCAACCTATCGAGAACACTGAGAAAGGAGAT
AACGGTATTATGAACGAATTATTTGTAGAGTTCTACCGGACAAGCAGGTCACGGAAGCCT
AAGCAGATTATCATTTTCAGGGATGGTGTGAGTGAATCACAGTTCAACCAAGTCTTGAAC
ATCGAGGTGGACCAAATTATAAAGGCGTATCAACGTCTTGGTGAATCTGATGTGCCAAAA
TTCACTGTCATTGTGGCTCAGAAGAACCACCACACCAAGTTGTTTAAAGCTAAGGGTCCT
GAAAATGTTCCTGCAGGAACTGTTGTGGACACCAAGATCGTACACCCGACAAACTACGAT
TTTTACATGTGTGCTCATGCAGGAATAATAGGAACTTCAAGACCGGCTCATTACCATGTA
TTACTTGATGAGATTGGTTTCTCCCCCGATGACTTGCAGAATCTCATCCATTCTCTCTCC
TATGTCAACCAACGTAGCACAACTGCAACTTCGATTGTGGCTCCAGTGCGATACGCTCAT
CTTGCAGCAGCTCAATTTGCTAAGTTTGAAGATGTATCGGAGGACGGGAAAGTTCCAGAG
CTTCCACGTCTGCACGAGAATGTGGAAACCAACATGTTCTTCTGCTTCTGCTGA
>Arabidopsis-lyrata__16062541_locus

ATGGATTCTAATGAACCGAATGGGAGTGGCGTAATGCAAAAGGGAGGGTTACCACCTCCA
CCACCTGTCGTTCCAACAAATGTTGTCCCTGAAGTGGAGCCTGTAAAAAAGAACATTCTT
CTCCCAATGGCTCGGCCTAGAGGCAGTGGTTCCAAAGGACAGAAGATTCCTCTTCTTACT
AATCACTTTGGAGTCAAGTTTAACAAAGCAAGCGGTTACTTCTTTCATTACAGTGTTGCT
ATCTCATATGAAGATGGCCGTCCAGTGGAGGCTAAAGGTATTGGCAGAAAGATTCTTGAC
AAAGTTCAGGAGACCTATCAAAGTGATTTGGGGTCCAAATACTTTGCTTATGATGGCGAG
AAGACTCTCTTCACTGTTGGTGCTCTTCCCAGCAACAAACTTGACTTCTCTGTTGTTCTT
GAAGACATACCTTCTAGCAGAAATAACGCTGGAAATGATACAAATGATGGGGATAGGAAA
AGATCAAGGCGTCCCAACCAATCTAAGAAATTCATGGTTGAGATAAGTTATGCTGCAAAG
ATCCCCATGCAGGCTATTGCAAGCGCTCTTCAAGGGAAGGAGACAGAGAATCTTCAAGAC
GCTCTAAGAGTGCTGGATATTATTTTGCGCCAGAGTGCAGCTAGGCAAGGTTGCCTCCTT
GTTCGCCAGTCTTTTTTCCACAATGACGTAAAGAACTTTGTACCTATTGGTGGAGGTGTC
AGTGGTTGCAGAGGGTTCCATTCAAGTTTCAGAACTACTCAGGGAGGCTTATCCCTGAAT
ATTGACACTTCAACTACGATGATTGTACAACCTGGACCTGTTGTTGATTTCCTGCTTGCA
AACCAGAACAAGAAAGATCCATACGGTGTGGACTGGAACAAGGCTCGTCGTGTTCTCAAG
AATCTGAGAGTTCAAGTTACTCTTTCAAATAGAGAATACAAGATAAGCGGGCTAAGTGAA
CACAGCTGCAAAGATCAAATGTTTACATGGAGGAAACCTAACGACAAGGGGGAATTTGAG
GAGGTTGAGATCACAGTGCTCAATTACTATAAAGAGCGTAATATTGAAGTGCGATATTCA
GGTGACTTCCCTTGCATCAATGTTGGTAAGCCGAAGCGTCCCACTTACTTCCCCATTGAG
TTCTGTAATCTTGTGTCTCTTCAGCGATACACAAAATCGCTTACCAATTTTCAGAGGGCT
GCACTAGTAGAAAAGTCTAGGCAGAAGCCACCAGAAAGGATGGCCTCACTAACCAAAGGT
CTAAAGGACAGCAATTATAATGCCGACCCGGTGTTGCAAGATAGTGGTGTCAGCATTATT
ACCAACTTCACCCAAGTTGAAGGCCGCATCTTACCAACACCAAAGCTGAAAGTGGGGAAC
GGACAGGACTTTACCCCAAACAACGGGCGCTGGAACTTTAATAGCAGGAAACTTGTTGAG
CCAACGACTGTTACTAGATGGGCTGTTGTGAACTTTTCTGCTCGTTGTGATACAAATGCG
CTTATTCGCGACTTGATTAGATGTGGACAGTCAAAAGGAATTAATGTAGAGCCTCCATTC
AAGGATGTCATTAACGAAAATCCTCAGTTTAGGAATGCACCAGCTACTGTGAGAGTAGAG
AATATGTTTGAGCAGATAAAATCCAAACTCCCAGGGCAGCCTCTGTTCCTACTTTGCATA
CTCTCAGAAAGGAAAAACTCTGATGTTTATGGCCCTTGGAAAAAAAAGAATCTTGTTGAT
CTTGGAATTGTGACTCAGTGCATTGCTCCCACAAGAGTGAACGATCAGTATCTCACCAAT
GTTCTCCTGAAGATAAATGCCAAGCTTGGTGGATTGAATTCGCTGTTAGCAATAGAGCGC
TCACCAGCAATGCCAAAAGTAACGCAAGTTCCTACTATCATTGTTGGGATGGATGTATCC
CATGGTTCCCCTGGCCAGTCTGATATACCATCAATTGCAGCGGTTGTGAGCTCCAGACAA
TGGCCACTCATCTCAAAATATAAGGCATGTGTACGCACACAATCGCGCAAAATGGAAATG
ATTGATAATCTCTTCAAACCCGTCTCTGGCAAAGACGAAGGAATGTTCAGGGAGCTGTTG
CTCGACTTTTACTACAGTTCTGAGAAGAGGAAACCAGAGCACATCATTATATTCAGGGAT
GGTGTGAGTGAGTCTCAGTTCAATCAAGTTCTTAATATTGAATTGGATCAGATGATGCAG
GCATGCAAGTTTCTTGACGAACACTGGAATCCAAAGTTTACAGTGATCGTTGCCCAGAAG
AACCACCACACCAAGTTCTTCCAGTCGAGTCGCCCTGATAATGTTCCTCCAGGAACAATA
ATTGACAGCCAGATCTGTCACCCACGCAACTTTGATTTCTATCTCTGCGCCCATGCCGGC
ATGATTGGAACTACAAGGCCAACACATTACCATGTGCTGTATGACGAGATTGGATTTGCC
ACAGATGACCTTCAAGAACTTGTGCATTCTCTATCCTATGTCTACCAGAGGAGCACCACT
GCGATCTCAGTCGTTGCACCAGTTTGTTATGCGCATTTGGCAGCTGCACAGATGGGAACT
GTGATGAAGTATGAAGAGTTGTCTGAGACTTCTTCGAGCCATGGGGGAATCACCACACCC
GGAGCAGTCCCTGTGCCTCCCATGCCGCAGCTGAACGATAAAGTTGCAACCTCCATGTTC
TTCTGCTGA
>Arabidopsis-lyrata__16062911_locus
ATGTCAAATCGTGGTGGTCACGGCGGCGCTAGTCGTGGTCGAGGAGGACGTAGGTCTGAT
CAGAGACAAAATCAGTCTTCTGGTCAGGCTGCTTGGCCGGGTTTACAACAAAGCTCCGGT
GGTCGTGGTGGTTCTGTTTCTGGCGGGAGAGGTCGTGGTAACGTCGGAAGAGGTGAAACT
AGTGGAGATCTGACGGCGACGCAAGTTCCGGTTGCTTCTGTTTCTGGCGGGAGAGGTCGT
GGTAACGTCGGACGAAGCGATTCTACCGGAGATCCGACGGCGACGTCTGTTGCTTCTTCG
TCTAAGACGATGTCTGTTTCTTCTTCGTCTAAAGAGGAAAGTAAGGTTACGGAGGTTTCT
GAGGCTTTGGCGAAGGTTGAGATTACTCCGACGGAGACGAAACCGGAAACGACGCTTCCT
CCGGCGTCATCTAAGGCGATTACGTATCCGGTACGGCCAGGGCGTGGTACTTTGGGGAAG
AAAGTCCTTATTCGTGCGAATCATTTCCTTGTTCAAATTGCTGATTGTGATCTCTATCAC
TACGATGTTTCGATTAATCCTGAGGTGATATCAAAGGCAGTGAACAGGAACGTGATGAAA
CTTTTGGTTAAGAACTATAAAGATTCTCACTTGGGAGGGAAGGCTCCAGCGTATGATGGA
AGGAAAAGCCTCTATACTGCTGGTGCATTACCTTTTGAATCGAAAGAGTTTGTTGTGAAT
CTGGCGGAAAAAAAGAGCTGATGGTTCTTCTGGGAAGGACAGATCGTTTAAAGTTGCTATA
AAGCTGGCGTCAAGACCTGATCTTTATCAGTTGCAACAGTTCCTTGCTCATAGGCAAAGA
GATGCTCCATATGATACTATCCAAGTGCTTGATGTTGTTCTCAGGGACAAGCCCTCTAAT

GATTATGTCTCTGTTGGGAGGTCTTTTTCCACACTAGTTTGGGAAAGGACACCAGAGAT
GGTAGGGGTGAGCTTGGAGATGGTATTGAGTACTGGAGAGGTTTTTCCAAAGTCTAAGG
CTGACTCAGATGGGTTTGTCTCTTAACATCGACGTTTCAGCAAGATCATTCTATGAACCA
ATCGTTGTCACTGAGTTTATTAGCAAGTTTCTGAATATAAGAGACTTAAACAGACCACTT
AGAGACTCAGATCGACTTAAGGTGAAGAAAGTTTTGAGGACACTAAAAGTTAAGTTGCTA
CATTGGAACAGCACAAAAAGTGCCAAGATTAGTGGGATTTCCAGCTGTCCCATCAGTCAG
CTAAGGTTCACTTTAGAGGACAAATCAGAGAAGACGGTTATACAATATTTTGCTGAAAAA
TATAATTATAGAGTGAAATACCCGGCTCTACCTGCTATTCAAACTGGGAGTGACACAAGA
CCCGTCTACCTACCTATGGAACTCTGCCAAATTGACGAAGGGCAAAGATACACCAAAAGG
CTTAATGAGAAGCAAGTGACTGCGTTGCTAAGAGCTACCTGCCAACGACCTCAAGAGAGA
GAGAACTCGATCAAAAACTTGGTTGTGAAAAATAATTATAACAATGTTCATGGTCTGAGC
AAGGAGTTTGGGATGTCAGTGACTAGCCAACTAGCCTCGATTGAAGCTCGTGTACTTCCT
CCACCGATGTTGAAGTACCATGAAAGCGGTAGAGAGAAGATGGTCAATCCAAGCCTGGGA
CAGTGGAACATGATTAACAAGAAAATGGTTAATGGAGCAAGAGTTGCATCTTGGACTTGC
GTAAATTTCTCTACGCGGATTGACCGAGGTTTACCCCAAGAGTTCTGCAAACAATTGACT
GGGATGTGCGTCAGCAAAGGAATGGAATTTAATCCACAACCTGCTATTCCGTTCATCTCT
TATCCGCCTCAAAGAATTGAGGAAGCTCTTCATGATATCCACAATAGGGCACCTGGTCTC
CAACTGTTGATTGTAATATTGCCTGATGTGACTGGATCATATGGACAAATCAAAAGGATC
TGTGAAACAGAATTGGGGATCGTCTCTCAGTGTTGCCAACCAAGACAAGCTTCTAAACTC
AATAAGCAGTACATGGAAAATGTTGCCTTGAAGATTAATGTCAAGACTGGGGGAAGGAAC
ACTGTTCTTAATGATGCTATTAGAAGAAACATACCTCTTATTACTGATCGCCCAACCATA
ATCATGGGTGCTGATGTGACTCATCCGCAACCTGGAGAGGACTCGAGTCCTTCTATTGCT
GCTGTTGTGGCCTCTATGGACTGGCCTGAGATAACTAAATACCGAGGACTGGTTTCTGCT
CAAGCTCATAGGGAAGAAATTATCCAGGACCTGTATAAGCTGGTTCAGGATCCACAACGT
GGGCTAGTCCACTCTGGTTTGATAAGGGAACATTTCATAGCATTCAGGAGAGCTACGGGC
CAGATACCTCAAAGGATTATCTTCTATCGCGACGGAGTAAGCGAAGGGCAGTTTAGTCAG
GTTCTGCTGCATGAGATGACAGCTATACGAAAGGCTTGTAACTCTCTCCAAGAGAACTAT
GTTCCTCGTGTCACTTTCGTGATTGTCCAGAAACGTCACCACACTCGTTTGTTCCCTGAG
CAACACGGGAATCGTGATACGACTGATAAAAGTGGCAATATTCAACCAGGTACTGTCGTT
GACACTACAATCTGTCACCCGAATGAGTTCGACTTCTATTTGAACAGCCACGCTGGTATT
CAGGGAACAAGCAGGCCGGCTCATTACCATGTTCTTCTCGACGAGAACGGTTTCACTGCT
GATCAGTTGCAAATGCTCACCAACAACCTCTGCTACACGTTTGCGAGGTGCACAAGATCT
GTGTCAATTGTGCCACCAGCCTACTACGCTCACTTGGCTGCTTTCCGTGCCCGCTACTAC
ATGGAGAGTGAGATGTCTGATGGAGGCTCGAGCAGGTCGAGGAACACAACAACAGGTGCG
GGTCAAGTCATTTCGCAGCTCCCAGCAATAAAAGATAACGTCAAGGACGTTATGTTTTAC
TGCTGA
>Arabidopsis-lyrata__16063851_locus
ATGGTGAGAAAGAGAAGAACGGATGCTCCATCTGCAGGAGGTGAAGCCTCTGGGTCTCGT
GAAGCTGGTCCAGTCTCAGGTGGTGGACGTGGTTCACAAAGAGGTGGTTTCCAGCAGGGA
GGTGGAGGAGGACAACAGCAAGGTGGAAGAGGTTATACTCCTCAGTCTCAACAGGGAGGT
CGTGGTGGTCGTGGATATGGGCAACCACCACAACAGCAACAGCAGTATGGTGGTCCACAA
GAGTACCAAGGAAGAGGAAGAGGAGGACCTCCTCATCAAGGAGGTCGAGGAGGGTATGGC
GGTGGCCGTGGAGGTGGACCTTCTTCTGGACCACCGCAGAGACAATCAGTTCCCGAGCTG
CATCAAGCTACCTCACCTACTTATCAAGCGGTGTCTTCTCAGCCTACACTGTCTGAGGTG
AGTCCTACCCAGGTGCCGGAACCTACTGTTCTGGCTCAGCAATTGGAACAGCTCTCTGTT
GAACAAGGAGCTCCCAGTCAGGCAATCCAGCCTATACCTTCTTCTAGCAAGGCTTACAAG
TTTCCAATGAGGCCTGGTAAAGGACAGAGTGGAAAGCGTTGCGTTGTGAAGGCTAACCAT
TTCTTTGCTGAACTGCCTGATAAGGATTTGCACCAGTATGATGTAACCATTACTCCGGAA
GTTACATCAAGGGGTGTTAATCGCGCTGTGATGAAACAACTTGTTGATAGTTATCGTGAA
TCTCACCTTGGAAATCGTCTTCCTGCCTATGATGGTCGGAAAAGTCTGTACACTGCTGGA
CCACTTCCGTTTACTTCCAAGGAGTTCAGAATCAATCTTCTTGATGAGGAAGAAGGGGCG
GGAGGACAAAGACGAGAAAGGGAATTCAAAGTTGTGATCAAGCTAGTTGCACGTGCTGAT
CTGCATCACCTAGGATTGTTTTTGGAGGGGAAACAACCAGATGCCCCACAGGAAGCTCTG
CAGGTTCTTGACATTGTTCTTCGTGAGCTGCCGACCTCTAGCATCAGGTATACTCCGGTG
GGCCGGTCCTTTTATTCCCCTGATATAGGAAGAAAGCAATCTTTGGGGGATGGCTTGGAG
AGCTGGCGTGGATTCTACCAAAGCATTCGTCCTACACAGATGGGCTTATCACTCAATATT
GATATGTCATCGACAGCATTCATAGAGGCATCCCCTGTGATTAAGTTTGTCTGTGATTTG
CTTAACCGGGATATTTCATCTCGACCTTTATCTGATGCTGATCGTGTTAAGATAAAAAAG
GCTCTTAGAGGTGTCAAGGTTGAAGTGACTCATCGAGGAAACATGCGCCGGAAGTACCGT
ATTTCTGGCTTGACTGCTGTGGCCACTCGGGAATTGACATTCCCAGTAGATGAACGAAAT
ACTCAGAAATCTGTTGTAGAATACTTCCACGAAACATATGGTTTTCGCATTCAGCACACT
CAACTACCATGCTTGCAAGTTGGGAATTCTAACAGGCCAAATTACTTACCAATGGAGGTA

TGCAAGATTGTTGAAGGCCAGCGATATTCGAAAAGATTGAATGAGAGACAGATCACTGCT
TTGCTGAAGGTTACCTGTCAGCGCCCCCTAGAACGAGAAAAAGATATCTTACGGACGGTA
GAACTCAATAATTATAAGGAAGATCCCTATGCTAAGGAGTTTGGCATCAAAATAAGTACG
TCTCTGGCTTCTGTTGAGGCTCGAATACTGCCTCCACCATGGCTTAAGTACCACGAGTCT
GGAAGGGAAGGGACTTGTCTGCCACAAGTTGGTCAATGGAACATGATGAATAAGAAAATG
ATCAATGGTGGAACGGTGAATAATTGGATCTGCATCAACTTTTCTAGGCAAGTGCAGGAC
AATCTAGCTCGTACGTTTTGTCAGGAACTTGCTCAAATGTGCTATGTATCTGGCATGGCC
TTTAATCCTGAACCAGTCCTCCCACCAGTCAGTGCTCGCCCTGAGCAAGTAGAGAAGGTC
TTGAAGACGAGATATCATGATGCCACCTCAAAACTCTCCCAAGGAAAGGAAATTGATCTG
CTTATTGTCATTCTTCCAGACAATAACGGATCATTATATGGTGATTTAAAACGCATATGT
GAGACTGAACTTGGCATTGTCTCTCAATGCTGCCTGACAAAACATGTCTTTAAGATGAGC
AAACAATACATGGCTAATGTTGCGCTGAAGATTAATGTGAAGGTTGGAGGAAGGAATACA
GTGCTTGTTGATGCTTTATCAAGGCGGATTCCACTAGTCAGTGATCGACCCACCATTATA
TTTGGTGCTGATGTTACCCATCCTCACCCTGGAGAGGATTCAAGCCCATCTATTGCTGCT
GTTGTGGCATCCCAGGATTGGCCTGAAATCACTAAATATGCTGGATTAGTTTGCGCTCAA
GCGCATAGGCAGGAGCTCATTCAGGATCTGTTCAAAGAGTGGAAGGATCCTCAGAAAGGG
GTGGTGACTGGTGGCATGATAAAGGAGTTACTCATAGCCTTCCGTAGATCAACTGGGCAT
AAACCGCTAAGGATCATCTTCTACAGGGATGGAGTCAGTGAAGGACAATTTTACCAAGTT
TTACTCTATGAACTTGATGCTATCCGCAAGGCCTGTGCTTCGCTGGAAGCTGGTTATCAG
CCACCAGTGACATTTGTGGTGGTGCAGAAGCGCCATCACACGAGGCTGTTTGCTCACAAC
CACAATGATCGTCATTCGGTGGACAGAAGTGGGAATATCTTACCTGGCACTGTTGTGGAC
TCTAAAATCTGTCACCCTACCGAGTTTGACTTTTACCTCTGTAGTCATGCTGGTATACAG
GGAACTTCTCGACCAGCTCATTACCATGTTCTTTGGGATGAGAACAACTTTACGGCAGAT
GGACTTCAATCTCTGACCAATAACTTATGTTACACGTATGCAAGATGCACACGATCAGTT
TCAATTGTTCCCCCTGCATATTATGCACATCTAGCAGCTTTTAGGGCTCGATTCTACATG
GAGCCGGAGACATCAGACAGTGGTTCGATGGCTAGCGGGAGCATGGCACGTGGAGGTGGA
ATGGCTGGTAGAAACACACGTGGGCCTAACATCAATGCTGCAGTGAGGCCACTCCCAGCA
CTGAAAGAGAATGTGAAGCGTGTTATGTTCTACTGCTGA
>Arabidopsis-lyrata__16064878_locus
ATGCCGATTAGGCAAATGAAAGATAGCTCTGAGACTCATTTAGTCATCAAAACCCAACCT
TTAAAGAACCATAATCCTAAAACCGTCCAAAACGGCAAAATCCCTCCTTCTCCGGTGACT
ACTCCGGCGACGGTGACTCAGAGTCAAGCTTCTTCACCTTCACCACCGTCAAAGAATCGT
AGCCGGAGGAGAAATCGTGGTGGGAGAAAATCTGATCAAGGAGATGTTTGTATGAGACCT
AGCTCTCGTCCTCGTAAACCACCACCGCCGTGTCAAACCACTTCCGCCGTCGTCTCAGCC
TCCACCGCCGGTGAGATTGTTGCCGTGAGTCATCAGATGCAGATGGGTGTTCGTAAAAAC
TCAAACTTTGCGCCAAGACCTGGATTTGGAACACTTGGAACTAAATGCATCGTTAAAGCT
AATCATTTCCTCGCTGATTTGCCTACCAAGGATTTGAATCAATATGATGTTACAATAACT
CCTGAAGTGTCATCAAAGAGTGTAAACAGAGCTATAATTGCTGAGTTAGTTAGACTTTAC
AAAGAGTCTGATCTCGGGAAGAGACTTCCGGCTTACGATGGCCGGAAAAGTCTTTACACT
GCCGGAGAACTTCCTTTTACTTGGAAGGAGTTCACTGTTAAGATTGTTGATGAAGATGAC
GGTATCATCAATGGACCTAAAAGGGAGAGATCATATAAGGTGGCTATTAAGTTTGTTGCA
CGGGCAAATATGCATCACTTAGGCGAGTTTCTAGCTGGTAAACGGGCAGATTGTCCGCAA
GAGGCGGTGCAGATTCTTGACATTGTACTCAGGGAATTGTCGGTTAAGAGGTTTTGTCCG
GTTGGAAGATCTTTCTTTTCGCCTGATATTAGAACACCGCAGCGACTCGGTGAAGGGTTA
GAGTCATGGTGTGGGTTTTACCAGAGTATTAGACCAACTCAAATGGGTTTATCACTAAAT
ATCGATATGGCTTCAGCTGCGTTCATCGAGCCTCTTCCGGTAATAGAGTTTGTAGCACAG
CTTCTTGGAAAGGATGTCTTGTCGAAGCCATTGTCTGATTCTGATCGGGTTAAGATTAAG
AAGGGTCTCAGAGGAGTGAAAGTAGAGGTTACTCACAGAGCGAATGTAAGGAGGAAATAC
CGTGTTGCGGGTTTAACAACTCAACCTACAAGAGAGCTAATGTTTCCAGTTGATGAGAAC
TGTACGATGAAGTCGGTTATTGAGTACTTCCAAGAGATGTATGGATTCACGATCCAGCAC
ACGCATTTGCCGTGTCTCCAAGTTGGAAACCAAAAGAAAGCAAGCTATTTGCCAATGGAG
GCCTGCAAAATTGTTGAGGGACAACGATACACGAAAAGGTTGAATGAGAAGCAGATTACT
GCTCTCTTGAAAGTTACATGTCAAAGGCCAAGGGACAGAGAAAACGATATTTTGCGGACG
GTCCAACACAACGCATATGATCAAGATCCATATGCGAAGGAGTTTGGCATGAACATAAGC
GAAAAGTTAGCTTCTGTTGAAGCTCGGATTCTTCCAGCTCCATGGCTTAAGTATCACGAG
AACGGGAAAGAAAAAGATTGTCTCCCGCAAGTCGGTCAGTGGAATATGATGAACAAGAAA
ATGATCAATGGGATGACGGTGAGCAGATGGGCCTGTGTTAACTTCTCACGCAGCGTTCAA
GAAAACGTTGCTCGTGCATTTTGTAATGAACTTGGTCAGATGTGTGAAGTCTCGGGCATG
GAGTTCAATCCAGAACCCGTGATCCCAATATATAATGCGAGGCCCGATCAAGTCGAGAAA
GCTCTAAAGCATGTTTATCACACTGCAATGAACAAAACCAAAGGCAGAGAGTTAGAGCTT
CTGTTGGCAATTTTACCTGATAACAACGGTTCACTTTATGGTGATCTTAAGAGAATCTGC
GAAACCGAGCTTGGTTTGATATCTCAATGTTGTCTCACAAAACATGTGTTCAAGATTAGC

AAACAGTATCTGGCAAATGTATCCCTTAAAATCAATGTCAAGATGGGAGGAAGGAACACA
GTTCTTTTAGACGCCATAAGCTGTAGAATTCCACTGGTTAGCGATATACCGACAATTATA
TTTGGCGCAGACGTGACTCACCCAGAGAACGGGGAAGAGTCAAGCCCTTCAATCGCTGCT
GTTGTTGCTTCTCAAGACTGGCCTGAAGTCACAAAATATGCGGGTTTAGTTTGTGCTCAA
GCTCACAGGCAAGAACTTATACAAGATTTGTATAAAACATGGCAAGATCCTGTACGCGGT
ACTGTTAGTGGCGGTATGATCAGGGACCTTCTGATCTCATTTAGGAAAGCAACAGGGCAG
AAACCGCTTCGAATTATCTTTTACCGTGATGGAGTAAGCGAAGGGCAATTCTATCAAGTT
TTACTCTATGAGTTGGATGCAATTCGAAAGGCTTGTGCGTCGCTTGAACCGAATTATCAG
CCACCGGTGACATTCATTGTTGTACAGAAGCGTCACCACACTCGTTTGTTTGCTAATAAT
CACCGAGACAAAAGCAGTACTGACCGAAGCGGAAATATCTTACCTGGTACTGTAGTTGAC
ACCAAAATATGTCATCCAACTGAATTCGACTTCTACCTTTGTAGCCATGCGGGTATTCAG
GGAACAAGCAGGCCTGCACATTACCATGTTCTTTGGGACGAGAACAATTTCACAGCTGAT
GGTATTCAATCTTTGACTAATAATCTCTGTTATACCTATGCACGATGCACTCGATCAGTC
TCGATAGTTCCTCCAGCGTATTATGCGCATCTTGCAGCATTTCGAGCACGTTTCTACATG
GAACCGGAGATAATGCAAGACAACGGATCACCCGGAAAAAAGAACACGAAAACTACAACT
GTGGGAGACTCAGGTGTGAAGCCTTTACCGGCTTTGAAGGAGAATGTTAAGAGAGTAATG
TTCTACTGCTAA
>Brachypodium-distachyon_Bradi1g16060
ATGGAGAGGGAGGGGGAGGCCAAGAACGAGAGGAAGGCCAGAGGTGGAGGAGGCGATAGC
AGAACCGCTGCTAGTAATGGTGGCGGCGGGAAAGGGAGGAGGACGTGGAAGGGTGCTTAC
GGTGGTGGGGGCGGCGGCTACAGACAGTACCCGATCATCCAGGCCTACCCGGCGCTCCTG
CCGCTGCCGGTAAACGCCGGCCGCAACCGCACGCATATTAATGGCGCGGTCGCGCTGCCT
CTGCCGCCGCCGGTACTGCTATACCTGCAGCCGCCGCCGCCGCTGCACCTGCTCTCCACG
TGCTACGGGAAGCCCATGCCGGGCGCGGTGCAGAGGGGGGCCAATGTGGACGCACAAGCCA
TCCAAGAAGCCGCCGCCGCCCCCACACGCCGTCACCGCCGCGCTGCTGCCGCTCCCCAAG
GGTTCCAACACACTTCAGACTGAAAAGTTTTTCATGCATAAGAAAACATCTCAGGTGGGG
ATGGATAATGGAAACACCCATCACAGGTCCTCTAACAATCAACAGGGAACACCCATTGCA
CGGAGACCTGACTCTGGTGGGGTTGAGGGGGCTGTGATTCCTCTTTCTGCAAACCATTTC
CTTGTGCGGTTTGATCCTGATCAAAAGATATTCCATTACGATGTTAACATATTCCCACAT
CCATCAAAGGAAACTGCAAGAATGATTAAGAACAAACTTGTAGAAGAAAATTCAAGCGTT
CTATCGGGTGCTCTGCCGGCTTTTGATGGCCGCAGGGACCTTTATAGTCCTTTTGAGTTT
CAGGAGGACAAGGCTGAGTTCTTTGTCAGTCTTCCAGTTACATCAGCAAGATGTTCAGTA
GTTAAAAATAATGGTTACATACTTGACCAACAGAAATTTAAGGTTTTTAAGGTGAACATT
CGATTGGTTTCCAAGCTAAGTGGTGAGTACTTGAACAAGTATTTGAGCAAAGATAAGGAT
GGCATTTCTCTTCCTCAAGATTACCTCCATGCGTTGGATGTCATCTTGCGAGAAGGTGCT
ATGGAAAGTTCTGTTCTGGTAGGCCGGTCTTTGTATCCACGCTCCATGGGAGAAGCAAAG
GATATTGGTGGTGGGGCTGTTGGTTTAAGAGGTTTCTTTCAGAGCTTGAGGCCAACCAAG
CAAGGTCTTGCACTTAATGTTGATCTCTCACTCACAGCATTCCATGAGAGCACGGGCATG
ATTGCATACTTGCAGAAGCGGTGTGACTTCTTGAAAGACCTTCCGCATGACAAAAATAGG
GCTTTGGCACAAGAGGAGAGGAGGGACGTCGAGAAAGCACTGAGAAACATTAGGGTTTTT
GTGTGCCACCGTGAGACCAACCAAAGGTACCACGTGCATAGCTTGACCGAGGAGACAACA
GAAAATCTCAAGTTTCGAGACCGAAGTGGGAAAGACCTTATGGTGATGGATTACTTCAAG
GAGCAATACAATCATGATATCCAATTCAGGAACCTGCCATGCTTGCAGATCGGTAGAAGC
AAACCATGTTATGTGCCAATGGAGCTTTGTGTAGTTTGTGAGGGTCAGAAGTTTCTTGGC
AAGCTCTCAGATGAACAAACATCCAAGATTCTCAAGATGGGTTGCCAAAGACCAAGTGAG
AGAAAAGGAATCATAAATGGCGCTGTTGAAGAAGCCTTTGGTGCAAAACGTAATTCTTAT
GCCGATCAATTCAATCTCCAAGTGTCCAAGGACATGACACAACTCTCTGGGAGGGTTCTC
TTGCCACCAAAACTGAAATTCGGCGGTGGAGGGCGCATTAAGGATATAACACCACAGCGA
TTTGATCGGCAATGGAGCTTGCTGGACAGCCATGTTACTGAGGGCTCCAAGATAAAGAGC
TGGGCCTTGATAAGCTTTGGTGGTACCCCGGAGCAGCACTCCTGCATTCCAAAATTTGTC
AACCAGCTATCAAGTCGGTGTGAGCAGCTGGGAATTTATCTAAACAAGAAAACTGTGATC
AGCCCGTTGTTTGAACGGATTCAACTCCTTAACAGTGTGTGCATTTTGGAGAGCAAGCTC
AAGAAAATTCAAGAAGCTGCATCAGGCAACCTGCAATTGCTAATATGTGTCATGGAACGG
AGGCACCGAGGCTATGCTGATTTGAAGCGAATTGCTGAAACATCCATTGGTGTTGTGACA
CAGTGTTGCCTTTACCCCAACTTAAGCAAGCTGACCGTGCAATTTGTGGCCAATTTAGCC
CTGAAGATGAATGCTAAGCTTGGTGGATGCAATGTTTCCCTCTACAACAGCTTGCCATGC
CAAATTCCTAGAATATTTTCAGATGATGAACCAGTGATGTTCATGGGTGCTGATGTTACA
CACCCACATCCACTTGACGACTCGAGCCCATCAGTGGTTGCTGTAGTTGCAAGCATGAAT
TGGCCTTCAGCAAATAAGTACATCTCCAGAATGAGGTCGCAGACGCACCGGAAAGAAATA
ATTGAACACCTGGATGTGATGGCTGGTGAACTACTTGAAGAGTTCCTGAAAGAAGTTGGC
AAGCTCCCAGCTCGAATCATATTTTTTCGAGATGGTGTGAGCGAGACACAGTTCGACAAG
GTCCTGAAGGAGGAGATGCATGCTGTGCGAATGACATGTTCGAGGTACCCAGGCTACAAG

CCCTTAATCACGTTCATCGTTGTACAGAAGAGGCATCACACGAGGCTCTTCCACAGGGAG
AAAAATGGCGGCTCGACACACTATTCTGATCAGAACATACCACCTGGAACGGTTGTTGAC
ACCGTGATCACGCACCCAAGGGAATTCGACTTCTACCTGTGCAGCCATTGGGGCACAAAG
GGGACAAGCCGTCCGACTCATTACCACATCTTGTTGGATGAGAACAAGTTTGGGTCCGAC
GAATTGCAGCAGCTGATACATAATCTTTGCTACACTTTTGTGCGGTGCACCAGGCCAGTC
TCCCTTGTTCCTCCGGCATACTATGCGCATCTTGCTGCCTATAGAGGCAAGCTGTACCTT
GAGAGGTCAGATTCAGTGCCAACCAGCCGCACAACTCTGTACAGTACCACTCCATTGCAG
ACGCCGCCGCTGCCTAAGCTCAGTGACAGCGTGAAGAGGCTCATGTTCTATTGCTGA
>Brachypodium-distachyon_Bradi1g28260
ATGGCGAATCGAGGGGGACGCGGGGGCCATGACGGCGGCCAGCACTACGGAGGCCGAGAT
GGCGGCGGCGGCGGCGGCCAGTACTACGGAGGCCGCGGTGGCGGCGGCCGCGGCAATGTC
GAGGGCGGCGATGGCCGTGGACGAGGGTACTACCGAGGCGGTGGTGATGGTGGCGGCCGC
CGCGGCCGTGGGTACTACCAAGGGGAAGGTGATGTCCGAGGGTTCTACCAAGGACGCGGC
GGTGATGGCGGCCGCGGGAACCAAGGACGCGGGTACCACGGCGGCGGCGAGGGCGTCATC
CGTGGTCGCGGGCGTGGTTACCAAGGGGATGGTGATGTCCGAGGCCGCGGCCGCGGATAC
GATGGGGGTGGCGACCGAGGCCGAGGCCGTGGCCGTGGCTATCAGCAAGGCGGCAACGAC
TACGGCCGCGGCCGTGGCGGCTATCAGCAACAAGGCGGCGACAACTACGGAGGCGGCCGC
GGAGGCAGGGGCGGAGGAGGCTACCCAGATTGGCCCCAACCCGTCGGGCCTCCCCTCGCC
GAGCGCTACGCGACCGAGGCGGCCCAGCTCCGGGAGAAGTTCAAGGCGATGGACATCAGC
CGCGCCGAGCCCACGTTCCCGGCCCGCCCCGGGTTCGGCAGCGCCGGGAAGGCGTGCATC
GTGAAGGCCAACCACTTCTTCGTCGGCCTCGTCGACAAGGGCCTGCACCAATACGACGTC
ACCGTCTCGCCGGAGCCGACTCTAACGGGCGTCTACCGGGCCGTCATGTCGAGGCTTGTG
TCGGAGCACCAGCATACCAGCCTTGGCGGCCGCCTCCCCGCTTATGACGGCCGCAAGACC
CTGTACACCGCTGGCCAGCTGCCGTTCAACAGCAAGGAGTTTGAGGTCATCTTGTCTGAC
AATAAGACAGGTTCATCTGGGCATAGCAGGGAAAGGAAGTACGTGGTGGCCATCAAGCAC
GTCACCTTGGTCAGCCTGCAGCAGCTGCAGATGCTCATGGCTGGGTACTCGACAGACATA
CCATCTCAGGCGCTGCAGGTGCTCGACATTGTGCTGCGCGACATGATTCTCAACGAGCGG
AGTGACATGGGGTATGTCGTGGTAGGCCGGTCCTTCTTCTCCGCAAGCATTGACGACCCC
AGGCATCTTGGCCTGGGTATTGAAGGATGGAAGGGGTTCTACCAGAGTATCAGGCCTACG
CAGAGCGGATTGTCTCTGAACATAGATATGTCTTCGACAGCTTTCGTTAAAGCTCAGTCA
GTGATTAAGTTTGTTCAAGATATTCTTAAGAAACCTGATCTCCGCCATGTTACTGGTCCT
GATTGTCAGAAGATTAAGAAAGCCCTCAAGGGTGTGAGGGTTGAAGTGACACATCGAGGA
GATGTACGCAGAAAGTACTGCATTTCTGGCTTAGCTGGTACTGCTCGAGATCTGAGGTTC
CAATCATCAACTGGCGTGTCCAAGACAGTCATGGATTATTTTAGAGAGACATACAAGCTG
CAACTGCGTTATGATTTTCTCCCATGCCTCGATGTTGGTACAACACAGAAACCAAACTAT
CTTCCGATGGAGGTTTGCAACATAGTTCCAGGACAGCGGTACCAGAAGAAGCTGGATGAA
AATCAGGTTTCTAACATGATGCAAATAACTTGCCAACAACCCACTTCAACGTGAGGGATTC
ATTCGTCAGACTGTTAGGTGTAACAATTACAATAATACCAAACGCGCAAATGAATTTGGC
ATAGAAGTTGACTACGAGCCTACTTCTGTTCAGGCTAGAGTTCTGCCTGCTCCAATGCTG
AAGTACCATCCTTCTGGATCTGACAACATGTGCAACCCAAGTAATGGAGCTTGGAACATG
AGAGGCAAGAAAGTCGTTGATGGTGCCCGTGTTGTCAATTGGCTATGTATAAACTTTTGC
GTTGATTTGCCTGAGGCTGATGTTCGTCGGTTCTGCAATGGACTGAGTAACATGTGCTGC
AATACTGGACTGTTCGTCAACATTGGAGGTCTTAAACTATTCAGTGCTGATCCATTGAAG
TTTGAAGCTAATCTCCACAATGTCCGCAATTTCTGTCAGCAAACACGGCAGATGTCGGGG
GTTCAAAAGATTGACCTCCTACTTGCTCTATTGCCAGATAAAAATGACAGCTTATATGGT
TATATTTCTTATCAGAAGCTTCCTTCCGTTACTGATTTATTACATATCCAATGCCTGATG
TCATTTTTGACCACAGGTGATATTAAAAGAATTTGCGAAACAGACATCGGTGTGATGTCA
CAGTGTTGTCTAAGGAAGAATGTCTTAAAGTCGAGTCCTCAATTTTTTGCAAATGTTGCT
ATTAAGATCAATGCCAAGTGTGGGGGAAGGAACTCAGTATTTGCCAATAGACAAGCAAGT
TTACCGGTGGTTTCAGCCAAGCCAACGATTATCTTCGGTGCAGATGTTACTCATCCAAGT
GCCCTAGATGATGCTACCCCTTCCATCGCTTCTGTTGTTGCCTCTAAAGACTGGCCTGAG
GTGACTAAGTATCATGGTGTGGTTCGTGCACAAGGTCACCGCGAAGAGCTCATCCAAGGT
CTTGAGGACATTGTTAGGGAACTCCTTCGTTCATTCGAAAAAGAATCTAACCGTAGGCCT
GAGCAGCTGATATTCTACAGGGATGGTGTAAGCGAGGGTCAGTTCAAGCAGGTTCTGGAG
AAGGAAATCCCAGAGATAGAGAAGGCATGGAAGGCAATATACAACGAGGAGCCACAGATC
ACCTTCATAGTGGTGCAGAAGAGGCACCACACAAGACTGTTCCCGAACAATCACAGTGAT
ATGAGCAGCAAGGACAGCAGTGGCAATGTTCTGCCAGGGACAGTTGTTGATAGACAGGTC
TGCCACCCAACAGAGTTTGATTTCTTCCTGTGCAGCCATGCTGGGATCAAGGGAACAAGC
CGTCCAACACATTACCATGTGCTGCGAGATGACAACAAGTTCACCGCTGATGCACTGCAG
TCGCTTACGAACAACCTCTGCTATACGTATGCAAGCTGCACTCGCTCGGTGTCGATTGCT
CCTCCCGTCTATTATGCTCATAAGCTTGCTTTCCGTGCTCGGTTCTACCAAACCCAAGGC
TCAGATGTGGAGTCGGTGGCAAGTTCAGGCAGCACAACTCAACCCGGTGCGATCAAGGCA

CTTCCTGAGATAAAAGATGAGGTGAAAAGGCTAATGTTCTACTGCTAG
>Brachypodium-distachyon_Bradi1g29580
ATGGGATCGAGGAGACCAAGACTGCCTGGATTTGGTGATGATGGTGGCGGGAGAGGGGGT
CAGGGAAGAGGCGGACGTGGCCGGGGCGGCTTCTATCCACAGCAATACCAGCAAGGTGGC
CGTGGAGCTGCGGGCTCCTACCATTACAATGGCCAGGGAGCTGCTCCTCAGCCCCGTGGC
GCAACAATGGTGCCGACACAGCAATGGCGTCCAGCTGGTCCTGCTGCTGCAGAGGATTCC
GGCCATGGACAGCCCTACAGGGAAGTGCACCCACAGCAACACAACAATGGTGGCCCGCCT
GCGACAATAACTCCCGAACTGCACCAAGCAATGGTTGATGATGCTCCACATGAGCCTGCT
GACATGATCTCCTCACCAGAGGCAGCTGGCTCATTGGAGACATCACCACCACAGGCTCTC
GAGGTGGTCACCGAGCACCTCGAGGTCTTGTCCATGCAGAGTGAATTGAGTGCCAGCCAG
GGGATTGTCCAAGCAATTCAGTTGTCCAGCAGCTCTTACAAGTTTCCTCACCGCCCGGGG
CGTGGAAGTATCGGCACGAGGTGCTTGGTAAAGGCAAATCACTTCCTTGCTGAACTGCCA
GACAAGGATCTTCATCAGTATGATGTTTCAATCACCCCGGAGATTACGTCACGGATTGTA
AGCCGTGCTGTGATGGAAGAGCTGGTGAAGCTGCACAAGGTATCATATTTGGGTGGGCGG
CTTCCAGCCTATGATGGTAGGAAGAGCATGTACACGGCTGGTCCGCTGCCATTTGTTTCA
AAAGAGTTTCACATCAATTTGCTTGACGAAGATGATGGTTCTGGTTTAGAGAGGCGTCAG
AGAACTTTTAAGGTGGTGATTAAGTTTGCCGCAAGAGCTGATCTTCATCGGCTCGAGCAA
TTTCTAGCTGGAAGGCAGGCAGAGGCTCCCCAAGAGGCCTTGCAAGTTCTTGATATCGTC
CTGCGTGAGCTGCCAACAGCTAGATATGCATCATATGGTCGATCCTTCTTCTCGCCTGAC
CTGGGGAGGAGGCGATCCCTTGGTGAAGGAATAGAAAGCTGGCGTGGGTTTTATCAGAGC
ATTCGTCCTACTCAGATGGGATTATCACTAAATATCGACATGTCAGCGACATCTTTCTTC
GAGCCACTACCTGTCATTGATTTTGTTGCACAGCTTCTAAACACTGATGTCTACTCAAGG
CCCCTGTCAGATGCTGATCGTGTCAAGATCAAGAAGGCCTTAAGAGGAGTGAAGGTGGAA
GTTACTCATCGTGGCAACATACGGCGGAAGTACCGTATATCTGGTTTAACATCTCAGGCA
ACTCGGGAGTTAAGTTTTCCTGTTGATCAAGGGGGCATGGTGAAGTCTGTTGTACAATAT
TTTCAGGAGACATATGGTTTTGCTATCCAACACACCTACCTTCCCTGTCTGCAAGTTGGT
AATCAGCAGCGTCCAAATTATCTCCCCATGGAGGTCTGCAAAATAGTGGAGGGACAGAGA
TATTCCAAGAGACTGAACCAGAGTCAGATAAGAGTTCTCTTAGAGGAGACATGTCAGCGC
CCACATGATCGGGAGCGTGACATAATTCAGAAAATGGTGAATGGTGGTAGAGTCAGGAGC
TGGCTGTGTGTCAATTTTGCTCGAAACGTGCAAGAGAGCGTTGCTACTGGATTTTGTCGT
GAACTTGCTCGCATGTGCCAAGCCTCAGGAATGGACTTTGCTTTGGAGCCTGTTCTTCCG
CCTATATATGTGCGTCCGGATCAAGTGGAGCGAGCTTTGAAAGCCAGGTTCCATGATGCA
ATGACCATACTTGGACCTCAGCGCAAGGAGCTTGAATTGCTTATTGGAATACTTCCTGAT
AACAATGGCTCACTTTATGGTGACTTGAAGCGTGTCTGTGAAATCGACCTTGGGCTAGTT
TCCCAGTGCTGTTTAACGAAACAAGTGTTTAAAATGAACAAACAGATCCTGGCAAATCTT
GCGCTGAAGATAAATGTCAAGGTTGGGGGAAGGAACACTGTACTGGCTGATGCATTGTCA
AGGCGCATTCCTTTGGTTACTGACAGGCCTACGATTATATTTGGTGCCGATGTCACCCAT
CCTCATCCTGGTGAAGACAGCAGCCCTTCCATTGCTGCAGTTGTGGCCTCCCAGGATTGG
CCTGAGGTGACAAAATATGCTGGCCTAGTTTCTGCTCAATCTCACAGGCAAGAATTGATA
GAGGATCTATATAAAGTCACACATGATCCACAGAGAGGAACCATCCATGGTGGCATGATC
AGGGAGCTTCTTATATCCTTTAAAAGGTCAACTGGAGAAAAGCCTCAGCGAATTATATTC
TATAGGGATGGTGTCAGTGAAGGCCAGTTTTACCAAGTTCTACTGCATGAGCTTGATGCG
ATCCGAAAGGCATGTGCATCGCTGGAAGCAAATTACCAACCGCAGGTTACTTTCGTCGTG
GTTCAGAAGCGCCACCACACCAGGTTATTTGCACACAACCACAATGACCAAAACTCTGTT
GACAGGAGCGGCAACATACTTCCTGGTACTGTTGTTGACTCCAAGATCTGCCATCCTACA
GAGTTTGACTTCTTCCTGTGCAGCCATGCTGGCATTAAGGGCACAAGCCGTCCTGCTCAT
TACCATGTACTGTGGGATGAAAACAACTTCACCGCCGATGGGCTGCAGACCCTCACTAAC
AACCTCTGCTACACTTATGCAAGGTGCACGCGTTCTGTATCTATTGTTCCTCCTGCATAT
TATGCTCATCTGGCTGCCTTCCGTGCCCGTTTCTACATGGAGCCAGATAGCTCCGACAGT
GGCTCTACAGCGAGTGCGCGTGGAGGCCTGTCCGGCTCGTCGACGTCTCGCAGTACCCGC
GCCGCTGGGGGTGGAATCGTCAGGCCCCTTCCTGCACTCAAGGACAGCGTGAAGAGGGTC
ATGTTTTACTGTTGA
>Brachypodium-distachyon_Bradi1g36910
ATGGCGCAGACGGCGGCGGCGGCGGCGCCGCCGACGCGCGCCGTCATTGGGCCGCCGGTG
CCGAGCAAGGGGCTGGCGTTCTGCCGGCGGCCGGGGTTCGGGACGGTGGGCGCGCGCTGC
GTCGTCAAGGCCAACCACTTCCTCGCCGAGATTCCCGACAAGGACCTTACCCAGTACGAC
GTCAAGATCACGCCGGAGGTGAGCTCCCGATGCGTGAACCGGGCCATCATCGCGGAGCTG
GTCCGCCTCTACCGCGCATCCGATCTCGGAATGCGCCTCCCGGCTTACGACGGCCGCAAG
AGCCTCTACACCGCCGGGACGCTTCCGTTCGACGCGCGCGAGTTCGTCGTGCGCCTAACC
GACGACGACGGCGGCACCGGCGTCCCCCCACGCGAGAGGGAATACAGGGTCGTCATCAAG
TTTGCCGCGCGCGCCGACCTCCACCACCTCCGCCAGTTCATCGCCGGGCGGCAGGCAGAC
GCGCCGCAGGAGGCCGTCCAGGTCCTCGACATCGTCCTCCGGGAGCTCGCCAACCAAAGG

TACGTGCCGATAGGGCGCTCGTTCTACTCGCCAGACATACGGAAGCCACAGCGTCTCGGC
GACGGTCTGCAGTCCTGGTGTGGGTTCTACCAGAGCATTCGGCCAACTCAGATGGGCTTG
TCGCTTAACATCGATATGTCGTCCACTGCATTCATCGAGCCGCTGCCGGTGATCGAGTTC
GTGGCCCAGATTTTAGGGAAGGATGTCATGTCAAGGCCATTGTCTGATGCAAACAGAATT
AAGATCAAGAAAGCACTGCGGGGTGTGAAAGTTGAAGTTACTCACCGGGAAAATGTAAGG
CGGAAGTACCGTATTTCAGGGGTGACAGCACAACCAACACACGAACTGATTTTCCCAATT
GACGATCAAATGAATATGAAATCAGTTGTAGAGTATTTCAAGGAAATGTATGGGTTCACA
ATTCAGCATGCCCATCTTCCTTGCCTTATGGTGGGAAACCAAAAGAAGGCAAACTATCTA
CCAATGGAGGCTTGCAAGATTGTTGAGGGCCAGAGATACACAAAGAGGTTGAATGAAAAG
CAGATCACCTCGCTGCTAAAGGTTACATGCCAAAGGCCTAGAGAGAAGGAAATGGATATT
CTACAGACAGTTCATCAAAACGGATATGACCAAGATCCTTACGCGAAGGAATTTGGGATC
AACATAAGTGAGAAGTTAACGTCTGTTGAAGCTCGTGTCCTTCCTGCACCTTGGCTGAAA
TATCATGACGCTGGAAAAGAAAAGGAGTGCTTGCCACAGGTTGGTCAATGGAACATGGTC
AACAAGAAAGTGATAAATGGGGGCAAGGTGAGTCACTGGGCCTGCATAAACTTCTCAAGA
AATGTTCAAGAAACCACTGCTCGGGGATTCTGCCAGGAGTTGGCACAAATGTGTCAGATT
TCGGGCATGGAATTCAACAGTGAACCTGTGCTACCAATATATTCAGCTAGACCAGATCAA
GTAGCTAAGGCACTTAAGCATGTGTATAATGTGGCACTACACAAACTCAAGGGTAAAGAA
CTTGAGCTTCTTTTGGCCATTCTCCCTGACAACAATGGTGCTTTATATGGTGATATCAAA
CGTATCTGTGAAACTGATTTGGGATTGATATCACAATGTTGCTTAACTAAGCATGTTTTT
AAGATTAGCAAGCAGTACTTGGCAAATGTTTCGCTTAAAATCAATGTTAAGATGGGAGGA
AGAAACACTGTGCTTGTGGATGCGCTAAGTTGGAGGATTCCATTGGTCAGTGACATACCA
ACTATTATATTTGGTGCGGATGTAACACATCCTGAAACCGGGGAGGACTCTAGTCCATCC
ATCGCTGCAGTTGTGGCTTCTCAAGACTGGCCAGAAGTTACGAAGTATGCTGGATTGGTG
TGCGCGCAGGCGCATCGGCAAGAGCTCATTCAGGACCTTTATAAGACATGGCATGACCCT
CAGAGAGGCACGGTCACAGGAGGCATGGTCAGGGAACTCTTAATATCCTTCAGGAAGGCC
ACTGGGCAGAAGCCATTAAGAATAATTTTCTACCGTGATGGTGTTAGCGAAGGCCAATTC
TACCAAGTTCTCCTGTATGAGTTAGATGCTATCCGTAAGGCATGTGCATCCCTGGAACCA
AATTACCAACCTCCTGTAACATTCGTGGTCGTTCAAAAGCGTCACCATACAAGGCTGTTT
GCAAACAATCACAAGGACAGAAGTAGCATGGACAAGAGTGGAAATATTTTGCCTGGAACT
GTTGTTGATTCGAAGATATGCCATCCAACGGAGTTTGATTTCTACCTCTGTAGTCATGCT
GGAATCCAGGGAACAAGTAGGCCAGCTCACTACCATGTCCTCTGGGATGAGAACAATTTC
TCAGCAGACGAGATGCAGACGTTGACAAACAACCTTTGCTACACGTACGCACGGTGCACA
CGCTCTGTTTCTGTCGTCCCTCCTGCATACTATGCGCATTTGGCTGCATTCCGGGCACGG
TTCTACATGGAGCCGGAGCTCTCAGAGAACCACACATCGAAGAGCTCTAGCGGAACGAAC
GGAACCTCGGTGAAGCCTTTGCCTGCTGTGAAAGAGAAGGTGAAGAGGGTGATGTTCTAC
TGTTGA
>Brachypodium-distachyon_Bradi2g10370
ATGGAGTCGCATGATGGCGTGGCTGATGACTTGCCACCACCACCACCTCTGCCGCCAAGC
ATAGAGCCTCTTAAACCTGAGGAAGCTGATGACTTGCCACCTCCACCACCACTACCACCA
AACGTTGAGCCTATTAAAGTTGAGGATGCAAAGAAGCCTAGGAGGACGCTGATCTCACGT
CCTGGGTTTGGCAAGAATGGGAAGCCGATACAGCTTGTGACAAACCATTTTAAAGTTTCC
TTGAAAACAACTGATGAGTTCTTTCACCATTATTATGTAAATCTGAAGTATGAGGATGAC
AGGCCAGTTGATGGAAAAGGAGTGGGTAGAAAAGTCATTGATAAGCTACAACAAACTTAT
GCATCTGAGCTTGCGCATAAAGATTTTGCTTATGATGGGGAAAAGAGTCTTTTTACCATA
GGTGCTCTACCACAAGTTAACAATGAGTTCGTTGTGGTGCTTGAAGATGTTTCTAGTGGA
AAGACTGCTGCAAATGGCAGCCCTGGAAATGACAACTCTCCAGGAAGTGATAGGAAAAGA
GTCAGAAGGCCATATCAGACTAAAACTTTTAAAGTGGAGCTCAACTTTGCTTCTAGGATT
CCAATGAGTTCTATCGCGATGGCTCTGCAAGGTCAGGAATCAGAGCACACGCAGGAAGCA
ATTCGGGTTATTGATATCATATTGAGACAACACGCCGCCAAGCAGGGTTGCCTTTTAGTC
CGCCAATCCTTTTTCCATAACAATCCATCACAATTTGTGGATCTGGGAGGCGGTGTAATG
GGCTGCAGGGGATTTCACTCAAGCTTCCGAGCCACAAAGAGCGGGCTTTCTCTTAATATT
GATGTATCTACAACAATGATTGTGAAACCTGGCGCTGTCGTTGATTTTCTAATTGCCAAC
CAGAAGGTAGATCACCCCAATAAAATTGATTGGGCAAAGGCTAAGCGTGCACTGAAGAAT
CTAAGGATAAAAACAAGTCCAGCAAACACAGAATTTAAGATTGTTGGACTGAGTGAAAGG
AACTGTTATGAACAAATGTTTTCACTGAAGCAAAGGAATAATGGGAATGGTGACTCTGAA
GCCATAGAAATATCTGTTTATGATTACTTTGTGAAGAATCGGGGCATAGAGCTAAGATAT
TCTGGTGATTTCCCCTGTATCAATGTGGGGAAACCAAAGCGCCCAACATATTTTCCTATT
GAGCTCTGCTCGCTTGTGCCTTTGCAAAGATATACCAAGTCTTTAAGTACTCTCCAAAGG
TCCTCTCTTGTCGAGAAGTCGAGGCAGAAGCCTCAAGAAAGGATGTCTGTTTTGTCTGAT
GTACTTAAACGCAGCAGCTATGACACAGATCCCATGCTAAAGGCATGTGGAATTTCGATA
GCTCAAGGTTTTACGCAAGTGCCTGGTAGGGTACTGCAGCCCCCCAAGCTCAAAGCTGGA
AATGGTGAAGATATTTTCACAAGGAATGGCCGATGGAATTTCAATAACAAGAGGCTTGTT

AGAGCTAGCTGTGTGGAAAGATGGGCAGTGGTTAACTTTTCGGCACGGTGTAACTGCAAT
GATCTTGTTCGAGACCTCATCAAGTGTGGAGGCATGAAGGGCATTAAAGTAGATCAGCCT
TTTGACGTGTTTGAAGAGAATTCTTCAATGAGAAGAGCACCTGCTCCAAAAAGGGTGGAG
GCTATGTTTGAAACAGTGAAGACAAAGCTTCCTGGAGCACCTAAGTTTTTGTTGTGTATC
CTTGCTGAGAGGAAGAATTCAGATGTTTATGGGCCTTGGAAGCGAAAATGTCTAGCTGAA
TTTGGGATTGTCACACAATGTGTGGCCCCAACTAGGGTCAATGACCAATATCTGACAAAT
GTCCTGCTAAAAATAAATGCAAAGCTTGGTGGGATGAATTCTTTGCTGCAAATTGAACTG
TCCCCTGCAATCCCTCTTGTATCAAAGGTCCCAACTATGATTTTGGGCATGGATGTGTCC
CATGGGTCCCCTGGACAGGCTGATACACCATCTATTGCTGCAGTCGTTAGTTCTCGTGAA
TGGCCTCTTGTCTCAAAGTACAGGGCCTCAGTGCGTTCTCAGTCACCAAAATCAGAAATG
ATTGATTCACTGTTTAAGCCACAAGGAACTGAAGACGATGGCCTTATTAGGGAGTGCCTA
ATAGACTTCTATACCAGTTCTGGAAAAAGAAAACCTGATCAGATTATAATCTTCAGGGAT
GGTGTTAGTGAGAGCCAATTTACTCAGGTGCTAAACAAGGAGTTAGATCAAATAAATGAG
GCATGCAAATTCTTGGATGAAAGTTGGTCCCCCAAATTCACACTAATTGTTGCGCAGAAA
AACCACCACACAAAGTTTTCATCCCTGGATCACCGGATAATGTCCCTCCCGGCACTGTT
GTTGATAACGTAGTTTGCCATCCAAAGAACTATGATTTCTACATGTGCGCACATGCAGGA
ATGATAGGAACAACAAGGCCAACACATTACCATATCCTGCATGATGATATACACTTTACC
GCAGATGATCTGCAGGATCTCGTGCACTCGCTCTCTTATGTGTACCAAAGAAGCACAACG
GCCATATCAGTAGTCTCTCCAATCTGCTATGCCCATCTTGCTGCTGCCCAAGTGTCACAG
TTCGTCAAGTTTGATGAGATGTCTGAGACGTCTTCAAGTCAGGGAGGCGGAGGCCACACC
TCTGCAGGCAGCACCCCAGTGCAAGAGCTGCCTCGCCTGCACGAGAAAGTCAGGAGCTCC
ATGTTCTTCTGTTGA
>Brachypodium-distachyon_Bradi2g14150
ATGGGCTCTGATGATGGAGGGGCTGAGGAGTTGCCACCACCACCTCCTCTGCCTCCAAAT
GTGGTGCCCATCAAAGCTGATGATGTCGTCAAGGAATCGCCACCGGACCAGCCAATGAAG
CCCAAGAGGTCCCTGGTGGCCAGGCCTGGTTTAGGAAGGATCGGGCAGCCCATCCAGCTC
CTCTCTAATCACTTTAAAGTCTCCGTGAAGAGTTCACAGGACTTCTTCCATCACTACGAT
GTAAGTATGCCTTTGAAGTATGAGGATGATACACCAGTTGATAGGCTGAAAGGGATAGGC
AGAAAGGTGATTGATAAACTACAGCATACATACCTCTCCGAACTTGCAAACAAGGACTTC
GCTTATGATGGTGAAAAGAGCCTTTTTACTATCGGCGCTCTTCCTCAAGTCACCAATGAA
TTCATCGTGATCTTGGAGGATCTTGGAACTGGAAAGACTGCTGCCAATGGGAGCCCTGGA
GGTAATGGCAGTCCTGGAGGGAGTGACAAGAAGAGAGTCAGGAGGCCGTACCAGACAAAG
ACTTTCAAAGTGGAGCTGCGCTTTGCAGCAAAAATACCCATGGGTGCAATTGCTGATGCC
ATCAGAGGTCAAGAATCAGAGAACTCCCTGGAGGCTATCCGAGTTCTTGATATCATACTG
AGGCAGCATGCTGCAAAACAGGGCTGCCTTTTAGTCCGCCAGTCATTTTTCCACAACAAT
CCTCGTAATTTTGTTGACCTGGGCGGTGGTGTGTTGGGGTGCCGAGGATTTCACTCAAGT
TTCCGTGGTACACAGAGTGGACTCTCCCTGAACATTGATGTTTCCACTACAATGATTGTT
CAACCTGGTCCTGTTATTGATTTCCTTAAAGCTAACCAGAAAGTTGATCAGCCTGCCAGA
ATTGACTGGTCAAAAGCCAAGCGTGCTCTTAAGAACTTGAGGATAAAAACAATTCCCGCA
AATACAGAATTCAAAATTGTTGGTTTGAGTGACAGAAATTGCAATGAACAGACGTTTGAA
TGGAGGCAGAGGAATGGTAGTGGAGGTATCGATACTGTTGAAATAACAGTCTATGAGTAC
TTCGTTAAGATTAGAGGCATAGAACTGCAGTATGGTAGTCTTCCCTGTATCAATGTAGGG
AGGCCAAAGCGTCCAACGTATTTTCCTGCGGAGCTTTGCATGCTTCTTCCACTGGAAAGA
TACACCAAAGCTCTGTCTACTCTGCAAAGGTCCTCGCTTGTTGAGAAATCCAGACAGAAG
CCTCAGGAAAGAATGTCAACCCTTGATGAGGCACTTAAACGTAGCAACTATGAATCTGAC
CCCATGCTGAGGGCATGTGGCATTTCAATTGCGCGGAATTTTACTCAAATTGAAGGGAGG
GTCCTGCAAGCGCCCAGGCTGAGAGTTGGCAACAATGAAGATATCTTAACACATAAGGGA
CGTTGGAGTTTGAAACACAAGAAGCTGTATCAGACCTGCTCTGTCGAGAGGTGGGCGGTT
GTTAATTTCTCTGCGCGATGTGATGTTCGGGGTCTTGTCCGAGACCTCAAAAGGAATGGA
ATTGCGAAGGGACTTAAAATACAGGACCCTTTTGATCATGTATTTGAAGAGAGCCCGTCG
ATGAGACGAGCGCCTGCGGCACAAAGAGTGGATGCTATGCTTGCTCAGTTAGAAAAGAAA
CTACCTGATAAACCCAATTTCCTTCTGTGCCTTCTCCCTGATAGGAAAAACTGCGAAGTT
TATGATTCAAATATATATTCTTTCCTTCAACCAGGCCCTTGGAAGAAGAAGTGTCTTGCT
GATCTTGGTATTGTTACACAATGTCTAGCTCCTCCAGCAAGAGTAAATGATCAGTACATA
GATAATGTGCTGTTGAAGATAAATGCTAAGCTTGGTGGGCTGAACTCATTGCTGAGAATT
GAAGTAGAACGCACAATACCTCTTGTGTCAAAGGTGCCTACTATCATCTTGGGCATCGAC
GTGTCACATGGTCCACCTGGGCAATCTGATAGACCTTCCATTGCCGCGGTGGTTAGCTCT
CGAGAGTGGCCTTACATCTCTAAATATAGAGCAACAGTGAACACTCAGTCACCCAAACTA
GAGATGGTGTCCTCCTTGTTTAAACCACAGGGGCCTGAAGATGATGATGGCCTCATTCGG
GTATCACTTATTGACTTCTACAACACTAGTGGGAAGCGCAAACCAGATCACGTTATTATT
TTCAGGGATGGAGTTAGTGAAAGCCAATTTACTCAGGTCATAAACATCGAGCTTGAAAAG
ATCATTGAGGCATGCAAGTTCCTCGATGAGAAGTGGTCGCCTAAGTTCACAGTCATTGTT

GCTCAGAAGAACCATCATACCAAATTTTTCCAGACTGGATCCCCGGATAATGTTCCTCCC
GGCACTGTGGTGGACAAACAAGTGTGTCATCCCAAGAATTTCGACTTCTACATGTGTGCA
CATGCTGGCATGATCGGAACATCAAGGCCAACACATTATCATGTTCTGCACGATGAGATC
GGCTTCACAGCTGACGAACTTGAGGAATTTGTGCATTCACTCTCCTATGTGTACCAGAGG
AGCACAACGGCGGTATCAGTCGTTGCTCCAATTTGCTATGCGCATCTGGCGGCTGCCCAG
GTCGGCACATTCCTGAAGTTTGAAGACATGTCGGACGCTTCGTCAAGCCAGGGAAGAGGC
CATACATCTGCGGGCAGCGCTGCGGTGCAGCCGCTGCCTGGGCTCCATGAGAGAGTGAGG
AACACCATGTTCTTCTGCTGA
>Brachypodium-distachyon_Bradi3g51080
ATGCTGCCATTAGCTCCGGGGAAAGTTCCAAAACTCAGGAAGCTACAAGAACCCCTGGAC
AGGGCCCTTCACAGAGAGTTGAGAGAGGTCAGCAGCGTGGAGGTAGCAACCGGGCACATG
CCAATACTCAATATTCTCAACAAGGAGGTCGTGGTGGTGGGCATTACCAGGATAGCATCA
CAGCAACCACCTGGGGGTCCTGTTAAGTATCAAGCACATGGGTATTATGGCCACGGTGGC
CCAAACCAAAGAGGAATGTCACAACCATACCATGACGGGCGTAGGAGTGGCGGTGGTGGA
AGAGGAGTTCCTATTACTCCATCAATAACACTTCCCGAACTGCACCAAGCCCCACAAGTT
CAGCACCAAGTTCCTGTGGTTACTCCTTCACCGCGGGAAACTGGCTCATCCTCACTAGGT
GTTGATATGAACACTGGACAACTCCAGCTACAGTTTCAGCAACTTGTTAACCTAGGTCAA
AGTTCCTCTGGACAGGGTATCCAATTAGCACCACCATCGAGTAAATCTGTAAGATTTCCA
ATGCGCCCTGGCAAGGGTAAATTAGGCAATAGGTGCATCGTGAAAGCAAATCATTTCTCT
GCTGAACTGCCTGATAAAGATCTTCATCAATATGATGTCTCTATAACTCCCGACGTCCCT
TCACGTGGTGTCAATCGTGCAGTCATGGGGCAGCTTGTAACCCTCTTCAGGCAATCTCAT
TTGGGTGGTAGTCTTCCTGCCTATGATGGAAGGAAGAGCCTATATACTGCTGGGCCATTG
CCATTCACTTCTAGGACCTTTGAAATTATTCTTCAGGATGAGGAGGATAGACTTGGTGGT
GCGCAAGCTGCACAAAGGCGTGAAAAACATTTTACGGTCGTCATAAAGTTCGCGGCGCGC
GCTGATCTCCATCATTTAGCTATGTTTTTGGCTGGGAGGCAAGCAGATGCTCCTCAAGAA
GCTCTTCAAGTGCTTGACATCGTTCTACGTGAACTGCCTACAGCAAGGTATTCCCCAGTT
GCCAGGTCATTTTATTCACCTAACTTAGGGAGGCGCCAGCAACTCGGTGATGGCTTGGAA
AGTTGGCGTGGCTTTTACCAGAGCATACGGCCCACACAGATGGGACTTTCACTGAATATT
GATATGTCATCTACAGCATTCATCGAGCCTTTGCCTGTGATTGATTTTGTTGCACAACTT
TTGAACAGAAACGTATCAGTTAGACCATTATCAGATGCTGATCGGGTGAAGATCAAGAAG
GCTCTACGAGGTGTAAAGGTTGAGGTCACACATAGAGGCAATATGCGCAGAAAGTATCGT
ATATCTGGCCTTACCTCGCAAGCAACAAGAGAGTTAACTTTCCCTATAGATAATCATGGT
ACTGTGAAGACAGTAGTGCGATACTTCCAGGAGACATACGGTTTTAACATTCAGCATACT
ACTTTGCCTTGCTTGCAAGTGGGCAATCCACAGAGGCCAAATTACCTTCCCATGGAGGTC
TGTAAGATTATCGAGGGACAGCGTTACTCAAAACGACTGAATGAGAAGCAGATAACTGCT
CTTCTTAAAGTGACCTGCCAGCGCCCCCAACAGAGGGAGCTGGACATTTTGCAGCTTAAA
TACCACGATAGTGGCAGAGAGAAAGATGTATTACCAAGAATTGGCCAATGGAATATGAAG
AATAAGAAAATGGTCAATGGTGGGAGAGTGAAGGACTGGACATGCATTAACTTTTCACGA
CATGTCCAAGATAGTGCAGCAAAGAGTTTCTGTCATGAGTTGGCTGTCATGTGCCAAATA
TCTGGGATGGAGTTTTCAATCGATCCTCTGCTTCCTCCTTTAACTGCGAGACCTGAACAT
GTAGAAAGAGCACTCAAGGCACGCTATCAAGATTCCATGACTGTGTTGAAACCACAGGGC
AGGGAACTTGACCTGCTTATTGTAATTTTACCTGACAATAATGGTTCTCTTTATGGTGAT
CTCAAAAGAATATGTGAGACAGATCTTGGATTGGTCTCTCAATGCTGTCTCACGAAACAT
GTTTTTAAGATGAACCAGCAGTATCTTGCAAATGTCGCCCTTAAAATCAATGTTAAGGTG
GGAGGAAGAAATACTGTACTTGTCAATGCTTTGTCAAGGAGAATTCCCCTTGTCAGTGAC
AGACCAACCATTATATTCGGTGCAGATGTTACCCATCCTCATCCTGGAGAGGATTCTAGC
CCTTCCATTGCAGCTGTTGTTGCTTCTCAAGATTGGCCTGAGATCACCAAGTATGCTGGA
TTAGTGAGTGCACAAACCCGTCGCCAGGAGTTGATACAAGATCTTTTTAAAGTGCAGCAA
GATCCTCAGAGAGGGAGTATAGCTGGTGGAATGGTTAGAGAACTTCTCATTTCTTTTAAG
AGATCAACTGGACAGAAACCCCAGAGGATCATATTCTATAGAGATGGTGTTAGTGAAGGG
CAGTTCTATCAGGTTCTGTTGTATGAGCTTGATGCCATTAGAAAGGCTTGTGCATCATTG
GAGCCCAATTATCAGCCACCTGTTACCTTTGTGGTGGTCCAGAAGCGTCATCATACACGG
CTCTTTGCTAATAACCACAATGATCAGCACAGTGTTGATAGAAAAAGTGGAAACATATTA
CCTGGAACTGTGGTTGATTCAAAGATATGTCATCCTACCGAGTTTGATTTCTACCTTTGT
AGCCATGCTGGCATTCAGGGAACAAGCCGCCCTGCGCATTACCATGTCCTGTGGGATGAA
AACAAATTTACTGCCGACGGTTTGCAGACTCTCACGAACAACTTGTGCTACACCTATGCA
AGATGCACTCGTTCTGTATCAATTGTGCCTCCTGCATACTATGCTCATCTTGCGGCTTTT
CGAGCTCGGTTCTACTTGGAACCGGATACTTCCGACAGTGGGTCGGCCATGAGTGGTGCC
ACGACAAGCCGTGGCCCTGCTAGTGCGCGCAGCAATAGAGCTGCTGGGAACGTTGCTGTT
AAACCGCTGCCTGACCTAAAGGACAACGTGAAGCGTGTCATGTTTTACTGTTAA
>Brachypodium-distachyon_Bradi4g08590
ATGCCTGATAGTATCAGTATGGCGGTTAAAGAGGGTGGGGCTGCCCAAGTGCATAAGGAT

GACACTGTTAAACGCACAGCAATGGCACGGCCTGGCTTTGGCCGTGAAGGAAAGCCCATT
AGGTTGATGTCAAACCACTTTGCTGTGAAGCTTAGCAGGACTGATGCTGTTTTCTACCAA
TACAGTGTGTCCATAAAATCTGATGATGATAAGGTGGTTGATGGAAAGGGTATTGGACGA
AAGGTCATTGATAAAATGTTGCAAACATACAGTTCTGAGCTTGCTGGGAAGGAATTTGCG
TACGATGGTGAGAAATGTCTATTTACAGTGGGGCCTCTTCCGCAGAACAACTTCGAGTTC
ACTGTTATCTTGGAGGAAACTTCTTCAAGGGCTGTTGGTAGGAGTCCAGAACATGGAAGT
CCTGGTCTGGGCGACAAGAAACGAGCAAAGCGGTCACATCTTCCGAAACAATTTGTAGTG
GGTATTAGTTATGCAGCAAAGATTCCCCTCAGAGCAGTTGCTTTAGCGCTTCGAGGAAGT
GACTCTGATCATGCTCAGGATGCTCTGAGAGTACTTGACATTGTTTTAAGGCAACAACAG
GCTAAGCAAGGTTGCCTGCTTGTAAGACAATCATTTTTCAGCGATGACAATAGGAACTTA
GTTGATTTAACTGGTGGTGTCAGTGGATGTCGTGGACTTCACTCTAGTTTCCGTACTACT
ATGGGTGGTTTGTCTTTAAATATGGATGTTTCAACTACGATGATTGTGACTCCTGGACCT
GTTGTTCACTTCCTCCTTACAAATCAAAATGTTAGAGATGTTCAAGATCTTGACTGGCCC
AAGATTTGTAGAACTTGTCACAAAATTCTAGTTACTTTTTTGCAGGCCAAAAAAATGTTG
AAGAATCTGAGAGTTAAAGCTACGCACAACAACATGGAATTCAAGATCATTGGTCTTAGT
GATCAACCATGTTCTAGACAGACATTTCCAATGAAAGTTCGGAATGGATGCACTGAAAGT
CAGACTGTTGATATTACTGTTGAGGAATATTTTAAATCCAAGGAGGTATTTTTGGCAAAG
CCTTATCTGCCATGTCTTGATGTGGGAAAACCAAAACGCCCGAATTATCTCCCAATTGAG
CTAGCGAACATGGTATCACTTCAACGTTATACGAAGGCACTGTCTTCTCAGCAAAGAGCA
ACGTTGGTTGAAAAATCACGACAGAAACCTCAGGATCGCATAAGAGTTATCACCGATTTG
GTTGTGGGAAATAGTGAAGATTGTATTCCAAACAGAGGCAGGTGGAATTATAATAACAAG
AAGCTATTTGAGCCAGTCAGGATTGAGCGTTGGGCAATTGTTAATTTCTCTGCTCGTTGT
GATATGAGCCGGATATCAAGAGATCTGATAAACTGTGGACGGAGCAAAGGCATTATTATT
GAAGGCCCTCACAGCTTGGTGGATGAAGATAGTCAAGCTAGGAGATGTGCTCCTATTGTA
AGAGTTGAAAGGATGTTCGAAAAAGTTAAAGCAAACCTTCCTGGTCCTCCGGAATTTCTG
CTGTGTGTTTTACCAGAGAGGAAGAATTGTGATATATACGGCCCTTGGAAGAAGAAAAAT
CTTCATGAGATGGGTATTGTCACTCAATGCATTGTTCCTAGTAATAAGATGAATGATCAA
TACTTCACCAATGTTCTTCTAAAGATCAATGCTAAGCTGGGCGGAATGAACTCCAAACTG
GCACTGGAACATAGCCATATGATTCCAATTGTCAATAAGAAACCGACATTAATTTTGGGA
ATGGATGTTTCACATGGTTCTCCAGGTCGATCAGATATACCATCAATTGCTGCTGTTGTT
GGGTCTAGATGTTGGCCACTGATATCACGGTACAGGGCATCTGTCCGGACCCAGTCTCCG
AAGGTAGAGATGATTGATTCACTTTTTAAGCCACTGGAGGATGGAAAGGATGATGGTATA
ATAAGGGAACTTCTGTTAGACTTCTACCAAACCAGTCAACAAAGAAAGCCAACACAGATA
ATCATTTTCAGGGATGGTGTGAGCGAGTCTCAATTTAGCCAAGTACTGAATCTTGAGGTT
AATCAAATAATAAAGGCTTACCAGAATATGGGTCAGGGGGATCCTCCAAAGGTTACAGTT
ATCATTGCTCAAAAGAATCATCACACAAAACTCTTCCAAGCTGAAGCATCAGATAATGTT
CCACCTGGGACTGTTGTGGACTCTGGTATTGTTCATCCAAAACAGTATGATTTCTACATG
TGTGCTCATGCGGGACCTATAGGTACCTCAAGGCCCACCCATTATCATGTCTTGCTTGAT
GAGATTGGCTTCTCACCAGATGACCTCCAGAAGCTAGTTCTTTCGCTTTCATATGTGTAC
CAGAGGAGCACTACTGCGATATCTGTCGTGGCACCTATCTGTTATGCTCACCTTGCAGCA
GCGCAGATGAGCCAGTTCATGAAATTTGAGGAGTTTGCTGATACTTCATCTGGGTCTGGT
GTCCCTTCGGCATCGACAGCAACAGTCCCTGAGCTGCCCCGGTTGCATGCTGATGTCTGC
AGTTCTATGTTCTTTTGTTGA
>Brachypodium-distachyon_Bradi5g18540
ATGGTTAGGAAGAAGAGAACTGGTCCTGCCAGCCCTGGAGAGAGTTCTGGGGAGGCTTCG
GGAGCTTCTGGGCAGGGTTCCTCACAGCGAACTGAAAGAGCTCCTCAACAGCATGGTGGT
GGACGTGGTTGGGTACCTCCACAGGGTGGCCGTGGTGGTGGGCCACACCAGGGGCGTGGT
GGACCCTATCAGGGCCCTGGTGGGCCAGCTCCACCGGAATTTCAACAGCGTGATTATCAG
GGACGTGGACATGCAGGTGGTGGCCCACCTGAATATCAACAGCGTGACTATCAGGGACGT
GGACATCCAGGTGGTGGTCCACCTGAATATCAGCCGCGTGACTATCAGGGACGTGGTGGT
CCAAGGCCCAGAGGTGGTGGAATGCCGCAGCCATACTATGGTGGGCATAGGGGAGGTAGT
GGTGGACGCAATGTTCCCCCAGGTCCATCAAGAACAGTTCCCGAGCTGCACCAAGCCCCT
CATGTCCAATATCAAGCCCCGATGGTTTCCCCATCCGCATCGGGAGCTGGCTCATCCTCT
CAGCCCGTGGTGGAGGTGAGCAGTGGACAAGTCCAGCAACAGTTTCAAAAACTTGCCATC
ATTGACCAAAGTTCGACCAGCCAAGCCTCTCAACTGGCACCAGCGTCGAGCAAATCAGTT
AGATTCCCATTGCGCCCTGGAAAGGGTACATATGGGGATAGGTGTGTCGTCAAGGCAAAC
CATTTCTTTGCTGAGCTTCCTGATAAGGACCTCCACCAATACGATGTGACCATAACGCCA
GAGGTTACATCACGTGGTGTTAATCGTGCTGTGATGGCAGAACTTGTTAAGCTTTACAGA
CAATCTCATTTGGATGGACGTTTACCTGCTTATGATGGAAGGAAGAGCCTCTATACAGCT
GGGCCGTTGCCGTTTACGTCAAGGACATTTGAAATTACTCTGCAAGACGAAGAAGAAAGC
CTTGGTGGTGGACAAGTCGTGCCAAGGCGCGAGAGACAATTCAGGGTGGTGATCAAATTT
GCTGCTCGTGCTGATCTCCACCATTTGGCTATGTTTCTAGCTGGGAGGCAGCCTGATGCA

CCACAAGAAGCTCTTCAGGTGCTTGATATTGTGCTACGTGAATTGCCTACTGCAAGGTAT
TCTCCAGTTGGCCGATCATTTTATTCTCCAAACTTAGGGAGACGTCAGAAACTTGGTGAA
GGTTTGGAAAGTTGGCGTGGATTTTACCAAAGTATAAGACCTACACAGATGGGACTATCG
CTTAATATTGATATGTCTTCTACTGCATTTATTGAGCCTCTCCCTGTGATTGAATTTGTT
GCTCAGCTTCTGTGTAGAGACATCTCAGTTAGACCGCTGTCTGATTCAGATCGTGTGAAG
ATTAAAAAGGCGTTGCGAGGTGTAAAGGTGGAGGTGACGCATCGAGGGAACATGCGTAGG
AAATATCGTATATCTGGTCTAACGTCACAAGCTACACGAGAGCTATCATTCCCTGTCGAT
GAACGTGGTACTGTAAAAACCGTGGTGCAATACTTCCTGGAGACATATGGTTTTAATATT
CAGCACACCACTCTGCCTTGCTTGCAAGTGGGCAATCAGCAGAGGCCGAATTATCTTCCT
ATGGAGGTTTGTAAGATTGTTGAAGGACAACGTTACTCGAAGCGCCTGAATGAGAAACAG
ATAACTGCTCTACTGAAAGTCACTTGCCAACGCCCTCAGGAGCGCGAAAAGGATATCTTG
ACGACTGTGCATCACAATGCATACTATGAGGATCCATATGCACAGGAATTTGGCATAAAA
ATTGATGAGCGCCTTGCATCGGTTGAAGCTCGTGTTCTTCCCCCGCCAAGACTTAAGTAC
CATGATAGTGGCAGAGAGAAGGATGTCTTGCCCAGAATCGGCCAGTGGAATATGATGAAC
AAGAAAATGGTGAATGGTGGTCGAGTCAGTAACTGGGCATGTATTAACTTCTCTCGGAAT
GTTCAAGATAGTGCTGCCAAGGGTTTCTGCCATGAGCTGGCTATAATGTGCCAAATATCT
GGAATGGACTTTGCACCTGAACCTGTGCTACCCCCACTTACCGCGAGACCTGAGCATGTG
GAAAGAGCACTAAAGGCTCGTTATCAAGATGCCATGAACATAATCAGACCCCAGGGCAGA
GAACTTGATCTGCTAATTGTTATACTGCCTGACAATAATGGTTCTCTTTATGGGGATCTC
AAAAGGATTTGCGAGACTGATCTGGGATTGGTATCTCAATGTTGTCTCACAAAGCACGTC
TTTAAGATGAGCAAGCAGTATCTTGCCAATGTAGCACTTAAAATTAATGTTAAGGTAGGA
GGAAGGAATACTGTACTTGTTGATGCTTTGACAAGGAGGATTCCCCTTGTTAGTGACAGA
CCAACCATAATATTTGGTGCTGATGTTACACATCCTCATCCTGGTGAAGATTCCAGTCCT
TCCATTGCCGCAGTGGTTGCTTCCCAAGACTGGCCTGAGGTCACCAAGTATGCAGGATTA
GTGAGCGCACAAGCCCATAGACAAGAGTTGATACAGGATCTATTTAAAGTATGGCAGGAT
CCCCAAAGAGGAACTGTGACTGGTGGCATGATCAAGGAGCTTCTCATTTCTTTCAAGAGG
GCAACTGGGCAAAAGCCACAGCGAATCATATTTTACAGGGATGGTGTCAGCGAAGGACAG
TTCTATCAAGTTCTGTTGTATGAGCTTGATGCCATTAGAAAGGCCTGTGCATCCTTGGAG
CCAAATTATCAGCCTCCAGTTACCTTCGTAGTTGTCCAGAAGCGTCATCATACCAGGCTT
TTTGCTAATAACCACAACGATCAGCGTACTGTGGATAGAAGCGGGAATATACTGCCTGGC
ACTGTTGTTGATTCCAAGATTTGCCATCCGACAGAGTTTGATTTCTACTTGTGTAGCCAT
GCTGGCATTCAGGGAACAAGCCGCCCTGCTCATTATCATGTTCTGTGGGATGAGAACAAA
TTTACTGCTGATGAGTTGCAAACTCTCACAAACAACTTGTGCTACACGTACGCAAGGTGC
ACCCGCTCTGTATCAATAGTGCCGCCGGCATATTATGCACATTTGGCGGCCTTCCGAGCA
AGATTTTACATGGAGCCAGATACCTCTGACAGTGGGTCCATGGCAAGTGGTGCCCGTGGC
CCTCCACAGGGTGGTTCGCGCAGTACAAGGGCGTTTGGGAATGTTGCTGTCAGGCCTCTG
CCTGCCCTCAAGGAAAACGTGAAGCGTGTCATGTTTTACTGTTAA
>Brachypodium-distachyon_Bradi5g21800
ATGGAGCACGAGCAAGGCGGCGGCCGCGGCCGCGGGAAAGCTCGCGGCGGAGGCGGGCGT
GGCGGTGGGCGAGGGGGTGGCTACGGGCCTCAAGGAGGCGGCGGAGGCTACGGACGAGGT
GAGGGCCAGGGCCGTGGAGGCGGAGGCGGCTACGCGCCTCGCGGGAGAGGCTACGGCCCT
CAGGGAGGCGGCGAGGGCCGCGGCGGAGGAGGCTACGGGTACCAGGGCCGCGGCGGAGGA
GGCTACGGCTCTCAGGGAGGCGGCGAGGGCCGCGGAAGAGGCTACGGGCCTCAGGGGCGC
GGCGAGGGACGGGGAGGAGGCTATGGCGCCCATGGGCGCGGCGAGGGAAGGGGTGGAGGC
TATGGCGCTCAGGGGCGCGGCGAGGGCCTTGGAGCCGGAGGAGGCCACGGGCCTCAGGGA
AGCGGAGGGCACGATGGTGGGCGTGGTACTGGCGGTGGTGGACGTGGGTACGGGCCAGGC
GGCGGCAGCGCGTGGGCGCAGCCGGGGAGAGGGCGTGGAGGTGGAGGTGGAGGTGGAGCG
GTCCCGGCGTGGGGGCCGGCGCCAGCGCCAGCTCCAGCCCCGGAAGCGAGGAGGATCAAA
GCAGAGGAGGCGGGGAGCTCGTCTGGATCCGTCGAACGCATATCCGACAAAGTGGCAAAA
ATAGAACCATCAGCACCCCAAGTTGTTGTGTCTCCTAATGGCACACGGGTGCCAATGCAA
AGACCTGATCGTGGAGGATCATCATTTCGAAACCAGGTCCAACTTTTGGTGAACTACTTC
ATAGTCAACTACCAAAATGTGTCAACCATATTTCACTACGACATAGACATCAAGTTTGAT
CAATCATCTTCAGAGTCTTCAGGCAAGGAGCTCTCCAATGCAGATTTTCATTCTGCAAAG
GCCGAGCTTTTCAAGGATGACAGCTTTCGGAATCTTTCATCAGCTGTTGCTTATGATGGA
AAGAGAAATCTATTTACTTGCACTGAACTGCCAGAAGGCTTATTCCGTGTGAGAGTTCGT
TCACGGACCTACATTGTATCAGTAGAGTTCAAGAAGCGGCTTCCTTTAAACCAACACCCA
CAATTGCCTAGGGAGGTCTTGCAGGGTCTTGATGTCGTCGATGTTGTAGCTATGAAAGGA
ACCAAACAGACCCTTAAACACACCCAGCAAGGGCTGGTCCTATGTGTTGACCATTCAGTT
ATGCCATTCCGCAAAGCTGGACCTGTCCTGGATGTTGTCAGGCAGTTCATAAGAAGGAAC
CTTGACTACAGGACAAATCTGACCGACAGTGAATGGGATAATTTGTCATCTGAACTGAAA
GGCCAACGAGTTACTGTGAACCACCGGATGACTAACCAGAAGTACACTATTCGAGGCTTA
ACAAAGGACCCTGCCAGAATGATCACCTTTGAAGATTCTGAATCAGGGCAGCAAAAGAGG

CTTGTTGATTATTTTGCTCAGCAGTATGGCAAGGTGACTGAGTATGAGATGCTTCCATGC
TTGGATTTGTCCAAGACGAAAAAGAACTATGTGCCGATTGAGCTTTGTGATTTCCTTGAA
GGACAGAGGTATCCAAAGGCAAATTTGCCAAGGAATATTGATACAAGCCTGAAAAGGATG
GCTTTAATCGGTGTAGATGAACGGAAGGCAGAGATTATGATGTCGGTAGGAGCTACACAT
GGGCCTTGCAGGGGAGAGATAGCTGAGAAGTTCGGGGTGTCTTTGGATGTAAAGATGACA
GAAGTCACCGGTAGGATCCTTCCTCCTCCAGTCCTGAAACTTGGCGGCCCCAAAGGCCAG
ACTTGCAAATTCAATATCAATCAGCCTACCTGCCAATGGAACCTCATGAGGAATAAACTG
GTAGAAGGCAAGGCCCTCAATTACTGGGGCATTATCGACTTCAGTACAGATTCCAGGCAG
CCGCTTCACCGAGAAATGTTTCTTAATTATATCGTTGGCAAGTGCCAAATGGCAGTGCTA
TGGGATGCAGACGAATTATACAGGGTGCTGAACGAAGCAAAGCAGTCTGCAGAAGAAAAG
AAGCAAAAGCTGCAGCTGCTCTTCTGCCCGATGTTTGAGCAGCATCCGGGGTACAAGACA
CTGAAGCTGATCTGCGAGACGAAACTGGGGATCCAGACCCAGTGTTTCTTGAGCAACGTC
GCGAACAATCCCCGGGGCCAGGACCAGTACATGTCCAACCTTGCTCTCAAAATCAACAGC
AAGATCGGTGGGAGCAACGTCCAGCTCTTTGATCTGCTCCCAAGGGACACGGGCGCTCCT
TTCATGCTCATCGGCGCGGACGTGAACCACCCGTCGCCCGGTAATCTGGAGAGCCCGTCT
ATAGCAGCCGTGGTGGCGTCCATGGACAAAGGAGCCACCAAGTATGCGTCCAGGATTCGC
GCGCAGCCACACCGCTGCGAGGTGATCAAGCACCTCGGCGAGATCTGCCAAGAGCTCATC
AGTGTCTTTGAGAACAAGAACAAGGTCAAGCCACATAAGATCATTTACTTCCGCGATGGC
GTGAGCGATGGGCAGTTCGACATGGTCCTGAATGAGGAGCTGGCGGACATGGAGAAGAGG
ATCAAGGTGAACGGCTACTCGCCGACGATCACCGTCATCGTGGCCAAGAAGCGGCACCAC
ACGCGGCTGTTCCCCAAGGATAAGCAGGCGGGTAAGGGCAACGTGCTCCCTGGCACGGTG
GTGGACACCAAAGTGGTCGACCCCTCGGCATACGACTTCTACCTGTGCAGCCACAACGGG
CTGATCGGGACGAGCCGGCCGACGCACTACTACAGCCTCATGGACGAGCATAACTACAGC
TCGGACGACCTCCAAAAGCTGATCTACAACCTCTGCTTCGTCTTCGCCCGCTGCACCAAG
CCTGTGTCGCTGGCCACGCCCGTCTACTATGCCGACCTGGCGGCGTACCGCGGCAGGCTC
TACTACGAGGGCATGACGATGGCGTTCCCGACCCAGGAGCGTGGGTCTTCATCCTCGTCC
TCTTCCTCCGCAGCTGCTCCTGCTCCTGCTCCAGAGTTCCCGAGGCTGCACGAGGATATT
CAGAACAACATGTTCTTCATCTGA
>Brachypodium-distachyon_Bradi5g21810
ATGGCGTATCGGAACGGCCGTGATCGCGGGGGCCGCGGCGGCGGTCGCGTGGGCCTGGGA
GAGGAGAGAGGTCACCGAGAACGCCACTTCCGCGGAGGCGGCAGCAGGAATCAAGGCGGC
TACCAGCATGCTAGAGGGAGAAACGGAGGAGGAGAGAACGGTGGGCGCTACAACCGCCAA
GGGCCGAACGCCCTGGAAACGGATAAGCGGAATAAGACCGAGGATCCTCCTGTTGACCCA
AAGATTAGCCCCAGTGAAGGGGTTGAAGTCAAACTTCTGGTGAACCACTTTACAGTCAAG
TTTGAGGAATCAACTATGTTTCACTATGATATCAAGCTAGACCAAGATTCTCCAGGGGCT
TCAGGCACGGGGCTACCCAATGCAGATAATTTTGCAAAGGCTGAACTCGTCAAGGTACTG
CAGCGGCCTCCACATTCATTGACTGTTGCTTATAATGGCATGGGACGTCTGTTCACCTTT
GCCGAACTGCCAGAAGGTCCATTCACTGTGAAAGTTGGGTCACGGGCCTACAGTGCGTTT
GCAAAGCTCGAGAACAAGGTGTCATTGAGCGAACTCTCTGAGCGGCCTGTGCCTGAATAT
TTATCGCAGGGTCTTGATTGCATTGTTCGCGAGGCCTCTAGCTTGGGAAAGATTATCGTT
GGTCAGACGTTCTATTCACCGGAAGAGGTGCCTGGGAACGAAGCAGACCCTAACACTGAC
CAACCAAGTGCTGTTCCTCCCGTTGCTCTGAGAGGAACCAAGCAGACCCTAAAACACACC
AACCAAGGGCCGATCCTCTGTGTGGACTATTCATTTATGGACTTCTGCAAAATGGGAGGC
AGTGTTCGGAGCCTTGTTAAGCACTTGGTGAAACGCCTTGACGGCACGATCCTTGACATC
CATACAACTCTAGGCGAGAAACAGTTGGTGCATTTGGAACGTCATCTCAAGGGCCTCTAC
GTTACCCTGAATTACCAGAAATCCCCTGAAGGAAAAAGTGATGGGACCACAGCTCGGAAG
TACAAGGTTCACGGCCTGACAAAGCAGCTTGCCCACCAGATCACCTTTCCGGATTTCAAA
TCAGGGGACCAGCGGAAGCTTCTTGAGTATTATCGTCAGCAGTATGGGAAGAGGTATCCC
AAAGAAAGTTCGCGCGAGAATTCCAACCAGCAACCAAACAACAGGCCACCCAAATTATCT
GAGCGGAAAAAGGAGATTCTGCGCATGGTAAAAGATGTAGATGGGCCTTGCAGAGGTCTG
GGAGGTGAGCAATTTAAGATTTCATTGGGCGAACAGATGACAGAAGTCATGGGTAGGATC
CTTCCTCCCCCAATGTTAAAACTTAGGGGATTTAATGGCAATTCCTACAGATTAAGTATC
GATCGTCAGCGTCAGCCTAAGTGCCAGTGGAACATCACGAGGAAGAAAGTGGCAGATGGA
ATTAACCTCCAGTACTGGGGCATTCTCGACTTCAGTGCAAGGCGGTCTCTGTCTCGCCGC
TGGGAGGAAGCGCTTCACAGAAGAAGGTTTGTTCGTGACATCTTCTTCAAGTGCAATGAG
CTTGGCATTCGGATGGCCGAGAAGCCATGCTACGACGAGGAATCAAAAATGTCAGTGCTA
TCGGATGCAGGCGAATTATACAAGGTGCTCAGCGCAGCAAAGCTGTCTGTAGAAGAGAAT
AAGCAGAAGCTGCAGCTCCTCTTCTGCCCGATGTCTGAGCAGCATCCGGGGTACAAGACA
CTGAAGCAGATCTGCGAGACGAAGCTGGGGATCCAGACTCAGTGTTTGTTGAGCGAAGCC
GCGAATAAAGACAACGTCCGGGACCGGGACCAGTACATGTCCAACCTTGCTCTCAAGATC
AACAGCAAGCTCGGGGGCAGCAATGTCCAGCTACTATCTGACGGGCTTCCAAAGATGGCT
GGCAGCCATTTCATGTTCATCGGCGCGGACGTGAACCATCCATCTCCCAACGACAACTTG

AGTCACTCGATAGCAGCTGTGGTCGCGTCCATGGATTGCCCTGGCGCCAGCAAGTACGTG
CCTAGAATCCGTGCCCAGAAGAACCGCTGTGAGGAGATCGTGGAGCTCGGTCAGATGTGC
AAAGAGCTCATCCAAGTCTACGAGAAGAAGAACGGTGTCAAGCCACAGAAGATCATTTAC
TTCCGCGATGGCGTGAGCGATAATCAGTTCGAGATGGTCCTGAAACAGGAGCTGAAGCAG
CTGGAGAACATGCTGAAGGCGCTCAAGGAGGGCTACTCGCCGACAATCACAGCGATCGTG
GCCAAGAAGCGGCACCACACACGGCTGTTCCCCAAGGACGAAGACCGCAACGTGCTCCCT
GGCACGGTGGTCGACACCGATGTGGTCAACACGGCAGACCAAGACTTCTTCCTGTGCAGC
CACGACGGGCTGCACGGGACGAGCCGGCCAACGCACTACCACAGGCTCAAGGACGACCAC
GGCTTCGAGCCCGTCGACCTGCAGAAGCTGGTGTACAACATGTGCTTCCTGTTCGCGCGC
TGCACCAAGCCGGTGTCGCTCACGACGCCCGTCAAGTACGCCGACCTTGCGGCGTACCGC
GGCAGGGACTACTACGACATTATGAAGACGGAGTCCCAGCACAGTGAGCTGCTTAAGAAG
ACGGGTGGATTCCCGATCCTTCTGCACGTGGACCTCGAGGACAGAATGGTCTTCATCTGA
>Carica-papaya_.TU.supercontig_135.40
ATGGAGAGACGAGGATATAGGGAAAGAGGAAGAGGCGGACGTGGTGGCGGTGGTCATAGA
GGTGGCAATGTTGGCGGCTCCAACGGTTTTGGAGGCCACCATCATCATCATCAGCAGCGT
AATCAGAATTCATACGAGTATCAGGATCATCATCAGAGGCAACGGAGGCCTGATGATCAT
GGAGGATTCCAGCGCCGACAGACGCATTCATTGTTTGATTCGAGTCATGATGGTGGTCGT
CGTTGTGGTACGGGTGGAGCTGGGCCAGGGAAGGGGGAAAACAGTCGACGAGGAAGAGAG
GTTTGGATCCCAAACAGAGGTGGAGTTCCAGTGCCGCCATATAGACCTCCGTTTACTGAG
CCTTTTCCTGATTCTTCCTCTCAAGAGATACAATCAGTGACATTATCAGAGAAATTGCCA
CCTTCATCTTCTACACCCAAAAACACTTCTAAAAGATTTCCAGTTACACGACCTGATGAA
GGTGGTACCTTGGCCATTCATACTGCAAGACTTCGTGTCAACCATTTTCTAGTTAAGTTT
AGTGTCGGCAGTACTATAATGCACTATGACATTAATATTAGACAAGAGGTGGTCCCTGAA
AATAGTTGCCCAGGGAAAATTTCAAAGTTAAACATGACTATGATCAGGAAGAAACTTTTC
ACTGATAATCCCATCAAATTCCCGCTCTCAGCTACAGCATATGACGGTGAGAAGAATATT
TATAGTTTAGTTCCACTTGAAGAGGGAGTATATAAGGTGGAATTGTCTGAGGTGGAAGAC
TCGAGGATACGAGTATACATGTGTACTATAAAGCTGGTGAATGAACTCAAGCTTCACAAG
TTGCGGGATTATTTGACTGGGAATGTCTGCACAGTACCCCGTGATATACTGCAAGGAATG
GATGTCGTAATGAAGGAGAATCCAACCAAATGTATGATTTCTGCTGGTCGTGGCTTTCAT
TTCATTAACCCTGATCCAGATGATGATCTTGGATTTGGTCTTACTGCATCCAGAGGGTAT
CGACATAGCCTCAAGCCCACCTCACAAGGTCTAGCACTCTGTGTGGACTATTCCGTGTTG
GCATTTTGGAAAAAGATGCCAGTTATTGAGTTTCTGAAGCAGCATATTCATAGGTTTTCT
TTAAACAATTTTGATGTCTCTAGGAATGCTGTTGCAAATGCTTTGACAAACTTAAAAGTT
ACAGTAACCCACCGTAAAACCAAACAAAAGTACACTATTGTTTGTTTGACCAAGGAGAGA
ACAAAGAATATTAAATTCAATATGGAAGACCCAGATGGCAAATGTCCAAAAAGACAAGTT
AGTATAGTTGATTATTTCAGAGAGAAATACGGCAGGGATATTGTGCATAAAGATATTCCT
TGCCTTGATCTAGGAAAAAACAATAGGGCGAGCTATGTACCAATGGAATTCTGCGTCTTA
GTGGAGGGACAGATTTATCCGAAAGAGCAGCTGCATACTGATGCAGCCTGGAGGTTGAAG
AACATGTCACTGGCAAAACCAGAAGATAGAGAGAGCAGGATTTGTAAGATTGTAGGGGCT
ACTGATGGACCATATAGTGGATATGGCATCCGAAATTTTGGAATTGAGGTTGATGTGAAT
ATGACATCAGTTATTGGACGAATTATCCGGCCTCCCGACTTGAAGTTAGGTGCTCCTGAT
GGTCGACTGATTAAGGTAACAGTTGACAGGAAGAGATGCCAGTGGAATCTTCTTGGCAAA
GGAGTAGTGGAAGGAAAACCTGTTGAGAGATGGGCTGTACTTGACTTTAGCTCATCTGAT
CGATGTAGGTTGAATGCTGACCTAGTTATTCCGAAGCTTATTAATCGCTGCAGGACTCTA
GGGATGCATATGAAAGAACCTGTTTTATATCAATGCACTGGAATGGATAAATTGTCTAAC
ACTGATGATCTTTATGAATTGCTTGAAAGTGTTATTAGTGGAGCTTACTACTGTGGTGGA
GGTCATGTACAATTCATTCTCTGTGTGATGTCGAGAAAGGATCCTGGCTACAAGTATCTC
AAGTGGATCTCTGAGACGAGAACTGGTGTGGTGACACAATGTTGTTTGTCCACTAGTGCC
AACAAAGTGAATGATCAGTATCTTGCTAATCTTGCTCTTAAGATCAATGCTAAGCTTGGA
GGTAGTAACGTAGAGTTGGTTGATGCCCTTGCTCATTTCAAAAGAGAAGACCATGTTATG
TTCGTGGGAGCTGACGTCAATCATCCTGCTGCACGGAACACAACAAGTCCATCGATAGCA
GCTGTAGTTGCCACTATAAATTGGCCAGCAGCTAACCAATACGCAGCAAGGATTCGTGCT
CAAAACCATCGTGAAGAGAGGATTGTTAATTACGGGAGTATGTGTCTGGATCTTGCTGAA
ACTTATGCTCGGCTAAATAAAGGAGTGAAACCTAAAAAAGTTGTGGTCTTCCGCGATGGG
GTGAGTGAGGGGCAGTTTGATATGGTTCTTAACGAAGAGTTACTTGACATGAAAGACGCA
TTTCAAAAAGTCAGTTATTTCCCAAATATAACTATTGTTGTGGCACAGAAGCGTCATCAA
ACTCGTTTCTTTCCAGAGAGTGAGAAAGATGGGGGTCTTACCGGTAATATACCTCCTGGT
ACTGTTGTGGATACAAAAATCATTCACCCTTTTGAGTTCGACTTTTATCTTTGTAGTCAC
TATGGGAGTATTGGGACAAGCAAGCCTACACACTACCATGTGTTGTGGGATGAGAATGGA
TTTTCTTCTGATCAGTTGCAGAAGCTCATTTATGACATGTGCTTCACATTTGCCCGATGC
ACTAAATCTGTATCCTTGATACCTCCAGTGTACTATGCAGATCTTGTTGCTTACAGGGGG
CGGCTGTATCATGAAGCAGTTATAGAGAAGCAGTCTCCACCTTCTGCTATATCATCTTCT

TCCACATTGACTATGTCTTCATTGTCTGCAGAGGAATCTTTTGATGAGAAGTTCTATAAG
GTGCATGCTGATCTAGAAAACATTATGTTTTTGTTTGA
>Carica-papaya_.TU.supercontig_1.68
ATGAGCGCTGCAGCACCACCATTAGTGGCTGCAAGGAGACCGGATTCTGGTGGCACAGAA
GGGCCAGTGATATCTCTACTTGCTAACCATTTTCTAGTCCAGTTTGATTCCTCACAAAGA
ATATACCATTACAATGTTGAAATCTCTCCAAGTCCATCCAAAGAAGTTGCTCGAATGATC
AAACAGAAACTGGTGCAGGACAACCCAGTTACTCTCTCTGGGGCTCTTCCTGCCTATGAT
GGCAGAAAGAATCTTTACAGCCCTATAGAATTCCAAAATGATAGGTTTGAGCTTTTCATT
AGCCTCCCAATCACCACTAGCAGCAATTCATCAGCTTTACCTTTTGGAGAGTTAAGTGAC
TTGGAAGGGAAGAAACAACAGTTCAAACTCTTCCGAATTAATATCAGACTTGTGTCAAAG
CTTGATGGGAAGGAATTGAATAGCTATCTGAGCAAGGAAGCTGATGATTGGATCCCACTG
CCTCAAGATTACCTCCATGCTTTGGATGTTGTTTTGAGAGAGAGCCCAACTGAGAAGTGT
ATATCTGTAGGGAGATCTTTCTATTCAAGTTCAATGGGAGGTGCTAAGGAAATTGGGGGA
GGAGCTGTTGGTTTGAGAGGGTTCTTTCAGAGTCTTCGACCAACCCAGCAGGGCCTTGCT
CTCAATGTGGATTTCTCTGTTACTGCTTTCCATGAGAGCATTGGAGTGATTCCATACTTG
CAGAAGCGTCTCGAGTTTCTCCAAGATCTTCCTCAAAGGAAAACGAGGAGGTTGAGTGGT
GAAGAAAGGAAAGAAGTAGAGAAGGCATTGAGGAACATCCGGGTTTTTGTTTGCCATAGA
GAAACTGTTCAGAGGTACCGGGTTTACGGCTTAACTGAGGAAGCTACCGAAAATCTTTGG
TTTGCAGACAGGGATGGGAAGAATCTGAGACTGCTAACTTACTTCAAAGATCATTACAAC
TATGATATACAATTCAGGAACTTGCCATGCTTGCAGATTAGTAGAAGCAAGCCATGTTAT
CTTCCTATGGAGCTTTGTATGATATGTGAAGGCCAAAAGTTTCTTGGTAAGCTTTCAGAT
GATCAGACTGCAAGAATACTTAAGATGGCCTGCCAAAGACCAAAAGAAAGAAAAGCCCTT
ATAGACGGAGTGATGGGAGGACCTGTTGGACCAACAAGTGGGAATCAGGGAAGAGAGTTC
AAATTCAATGTTTCAAGGGAAATGACAAGATTGAGTGGGAGAATTCTACAACCTCCAAAG
CTTAAGCTTGGTGATGGTGGTCATGTAAGAGATTTAACTCCCTTTCGCCACAACCGACAG
TGGAACCTTCTGGACAGCCATGTCTTTGAAGGAACTCGAATAGAGAGGTGGGCATTGATA
AGTTTTGGAGGCACACCTGATCAGAAGTCTAACATTTCAAAATTCATAAACCAGTTGTCT
CAAAGGTGCGAACAGTTAGGCATCTTTCTTAACAAGAACACAATAATAAGTCCCCAGTTT
GAGCCAACACAGTTACTTAACAACGTCTTCCTTCTTGAGTCGAAGCTTAAGAAAATCCAC
AAAGCTGCATCAAACAATCTGCAGCTGCTGATGTGCATAATGGAGAGAAAGCACAAGGGA
TATGCAGATTTGAAGCGAATAGCAGAGACAAGTGTGGGAGTAGTAACCCAATGTTGTTTG
TATCCAAACCTTAGCAAGTTGAGTTCACAATTCCTGGCTAACTTAGCTCTCAAAATGAAT
GCTAAGGTTGGGGGCTGCACAGTTGCTTTGTACAACTCGCTACCGTCTCAAATTCCACGT
ATCCTTCAGCTCGATGAGCCTGTGATGTTTATGGGTGCTGATGTTACTCATCCTCATCCT
CTCGATGATTTCAGCCCATCTGTTGCTGCTGTTGTAGGAAGCATGAACTGGCCTGCAGCC
AACAAGTATGTTTCAAGAATGAGGTCCCAAACCCATAGACAAGAAATTATCCAGGATCTT
GCTTCGATGGTAGGGGAGTTACTAGATGATTTCCTCCAAGAAATAACCCAACTTCCCAAA
AGAATCATATTCTTCCGGGATGGAGTAAGTGAAACCCAATTTAACAAGGTGCTTAAAGAG
GAGCTACAAGCTATTAGAGAAGCATGTTCAAGATTTCCTGGTTATAGACCTCCCATTACT
TTTTCTGTAGTTCAAAAGAGGCATCACACAAGGTTGTTTCCCTATGATCTTGATTCATCT
TCCTCCCAAAATCAGTTCTTTGAAGAAAACATTCCTCCGGGAACTGTGGTAGACACCGTG
ATTACTCACCCGAGAGAATTTGATTTCTACCTGTGTAGCCACTGGGGAGTAAAAGGAACC
AGCCGTCCAACACATTACCATGTCCTGTGGGATGAGAATCATTTCACTTCTGATGAACTC
CAAAAGCTGGTTTACAATCTGTGCTACACATTTGTGAGGTGTACGAAGCCAGTTTCTTTA
GTTCCCCCTGCCTACTATGCTCACTTGGCTGCATATAGAGGCAGACTTTACCTTGAGCGA
TCTTCTGAACCTACAGCTTCTGTGAGAAATACTTGTACTATTTCACGAGCTGCACCTCCA
AAGACAACACCTCTACCCAAACTTCGTGAAAATGTAAAGAAGCTCATGTTTTACTGCTAG
>Carica-papaya_.TU.supercontig_26.59
ATGCTTGAAATATACATTCCTAGGTATATGGTCGCTGACATTGATATGGAAGGAAATGGG
GCACATGAAGGAAATGGGGCTCAGGACATGCTACCTCCTCCCCCTGCTGTTATCCCACCA
GACGTAACTCCAGTGAAAGTGGAGCCTGAAAAGAAAAAGGTTACGCGGGTGCCAATGGCT
AGACGTGGGGTTGGGTCCAAAGGGCAGAAGATCCAGTTGCTTACAAATCATTTCAAAGTT
AACGTTTCTAGTGTGGAGGGTCACTTCTACCATTACAGTGTTGCTCTCAAATATGAAGAT
GGCCGCCCTGTAGAGGGCAAGGGTGTGGGAAGAAAATTGATTGATAAGGTGCAAGAGACT
TATCATAGCGAGTTAGACGGTAAAAACTTTGCTTATGATGGAGAGAAGAGCTTATTCTCT
GCTGGTCCCCTTCCGCGTAACAAACTTGAATTCACTGTTGTGCTTGAAGAAATTACCTCT
AGCAGGAATAATGGGCTTGGTAGCCCAGGTGGAGATGCAAGTCCTAATGAGACCGACAGG
AAAAGGCTTAGAAGGCCCTACCAATCCAAAACTTTTAGGGTTGAAATTAGCTTTGCTGCA
AAGATTCCCATGCAGGCTATTGCAAATGCATTGCGTGGACAGGAATCGGAAAATTCACAA
GAAGCCTTGAGGGTTCTGGATATAATATTACGGCAACATGCATCAAGACAAGGATGCCTT
CTTGTTAGGCAGTCTTTCTTTCATAATGATCCGAAGAACTTCGCGGATGTGGGTGGTGGT
GTCTTAGGTTGTAGAGGATTTCATTCAAGCTTCAGGGCCTCTCAGGGAGGCTTGTCTTTA

AATATTGATGTGTCAACTACAATGATAATACGGCCTGGGCCTGTGGTGGATTTCTTAATT
GAGAATCAAAATGTCAGAGATCCCTACTCCATTGATTGGTCAAAGGCTAAAAGAACACTG
AAAAATTTGAGGATCAAGGCCAATCCCTCAAATCAGGAATACAAAATAACTGGTTTGAGT
GAGAGACCATGCAAGGAGCAAACGTTTGTATTGAAGAATAGGAAGGATGATAATGGGGAA
GAAGCTGAAGTGACTGTGTATGATTACTTTGTTAATACTCGTGGTATAGAGTTGAGGTAC
TCTGGTGATTTGCCTTGCATTAATGTTGGAAAGCCGAAACGCCCCACTTTTATCCCACTT
GAGCTTTGCTCTTTGGTGCCCTTGCAGCGATACACAAAAGCACTTTCAACTCTCCAAAGA
GCTTCGCTAGTGGAGAAATCAAGGCAGAAGCCACAGGAACGGATGAATGCTTTATCTATT
GCTTTGAGAAACAGCAATTATGGTTCTGAACCTATGCTCCGGCAATGTGGCATCTCAATC
AGTACGAACTTTACTCAAATTGAAGGCCGTGTTCTGCCAGCTCCCAGGTTAAAAGTGGGC
AATGGGGAAGATTTCTTTCCTCGAAATGGGCGTTGGAATTTCAATAATAAGAAACTCGCG
GAACCAACTAAGATTGAAAGATGGGCAGTTGTGAATTTCTCGGCACGTTGTGATATTCGT
GGTCTTGTGCGAGATCTAATAAAATGTGGAGAAATGAAAGGAATTCGCCTAGAACCTCCA
TTTGAGGTTTTTGAAGAAAGTCATCAGTTTAGACGAGCCCCAGCTTTGGTTAGAGTGGAG
AAGATGTTTGAGGAAATACAAGCTAAACTTCCAGGGGCTCCTGAGTTCCTTCTTTGCTTA
CTTCCTGAGAGAAAGAACTGTGACATTTATGGTCCTTGGAAACGGAAGAATCTTGCTGAA
TTTGGTATAGTCACTCAATGCATTGCTCCCACAAGGGTCAATGACCAATATCTCACTAAT
GTTTTACTGAAGATCAATGCAAAGCTTGGTGGATTGAATTCAATATTAGCAATTGAACAT
TCTCCTGCAATCCCTATTGTTTCAAAGGCTCCTACTATCATCCTTGGGATGGATGTTTCA
CACGGCTCTCCTGGGCAATCTGATGTTCCATCAATTGCAGCAGTTGTCAGCTCTAGGCAG
TGGCCATTGATCTCACGATATAGAGCTTCTGTGCGTACACAGTCCCCCAAGGTTGAAATG
ATTGATTCTCTTTTCAAGCGAGTGTCGGAGACTGAGGATGATGGTATCATGAGGGAGCTG
CTGCTGGACTTCTATACGAGTTCTGGGAAAAGAAAACCTGATCAGATCATCATATTCAGG
GATGGAGTAAGTGAGTCACAATTCAATCAAGTTCTTAACATCGAACTTGATCAAATTATT
GAGGCCTGTAAATTTCTTGATGAATCCTGGTCCCCAAAGTTCTTGGTGATCATTGCACAG
AAGAACCATCATACGAAATTTTTCCAGTCTGGCTCCCCAGATAATGTTCCACCTGGAACT
GTTATTGACAGTAAAATTTGTCATCCACGAAACAACGATTTTTATCTATGTGCTCATGCT
GGCATGATTGGAACTACAAGGCCTACACACTATCATGTTCTGTTAGATGAGATTGGCTTC
TCTGCAGATGATCTCCAAGAGCTAGTTCATTCACTATCTTATGTATATCAAAGAAGCACA
ACTGCCATTTCTGTAGTTGCACCTGTATGCTATGCTCATCTGGCAGCCACCCAGATGGGA
CAGTTTATTAAGTTTGAAGATGCTTCTGAGACATCCTCGAGTCATGGAGGAACAACAACT
GCTGGAGCTGTTGCAGTGCCACAACTGCCCAGATTGAAGGAAAACGTATCGAGTTCAATG
TTCTTTTGTTGA
>Carica-papaya_.TU.supercontig_44.130
ATGCCTATAAGGCAAATGAAAGACGGCTCAGAGCAGCACTTTGTAATCAAAACCCACCTC
CAAAACTCCATGAATCCCACACAAAAACATCCCAAAGCTACCCAAAATGGTAAAGGACCA
CCACCCCAAGAACAATCCCACAATAACAAACCCCATAACCAAGCATCACCTCCAACGAGA
AATAGAGGAAGGAGGAGAGGCAGAGGTGGCAGGAAATCTGATCAAGGAGATGTCTGTATG
AGACCGAGTTCCAGGCCTTGCACAGTTGCACTTAAGTCCGTCAACCCGGCTGGTGATCTT
GTACCAAGCAACCAAATTAAGTCTATTGAAAATGTTGAGAGTGTGTGTCAAATGGAAACG
GGTTTTCCTACTTCAAGCAAGTCTTTGAGTTTCGCACGTAGGCCTGGTTATGGACAAATT
GGGACAAAGTGCATTGTCAAGGCTAACCATTTCTTTGCTGAGTTAAATGAGAAAGATTTG
AACCAGTATGATGTCACAATAACTCCGGAGGTGCCATCAAGAACTGTGAACAGAGCAATT
ATAGCAGAGCTTGTCAGATTATATAAAGAATCGGACCTAGGAATGAGACTACCTGCTTAT
GATGGCCGAAAGAGCTTGTATACAGCTGGCGAACTTCCCTTTGCTTGGAAAGAGTTTACC
ATTAAGCTTATAGATGAAGAGGATGGAATCAATGGCCCCAAAAGGGAAAGAGAATACAAA
GTGGTCATTAAATTTGTAGCTCGAGCCAACATGTATCAATTAGGACAGTTTCTAGCGGGT
AAGCGAGCAGATGCACCACAAGAAGCTTTGCAAATTCTTGACATTGTGCTGAGGGAGCTC
TCAAACAAGAGATATTGCCCTATTGGAAGGTCATTCTTCTCTCCTGATATTAGAAACCCG
CAACGCCTTGGTGAGGGTTTGGAGTCATGGTGTGGCTTTTATCAGAGTATTAGGCCTACA
CAGATGGGCTTGTCCTTAAATATTGATATGGCTTCAGCTGCATTTATTGAGGCTCTCCCT
GTGGTAGAGTTTGTAGCCCAGCTTTTGGGAAAGGATGTTTTATCAAGGCCATTATCTGAT
TCTGATCGTGTGAAGATTAAGAAGGCTCTTAGGGGAGTAAAAGTGGAGGTTACACACCGA
GGAAACGTGAGAAGGAAGTACCGTGTTGCAGGATTGACATCCCAGCCAACAAGAGAACTA
GTGTTTCCCGTTGATGACAACTCTACCATGAAGTCAGTTGTTGAGTACTTCCAAGAAATG
TATGGCTTCACCATTCAACATACACATCTGCCTTGTCTTCAAGTAGGAAATCAAAAGAAG
GCAAACTATTTGCCTATGGAGGCCTGCAAAATTGTTGAGGGACAGAGATATACAAAAAGG
TTGAATGAGAAGCAAATTACTTCCCTTTTGAGAGTTACTTGCCAGAGACCTAGGGATCGG
GAACATGATATTTTGCAGACAGTTCAACATAATGCTTATGATCAAGATCCTTATGCAAAG
GAATTTGGAATCAAGATCAGTGAAAAACTAGCTTCTGTTGAGGCTCGCATTCTTCCTGCT
CCTTGGCTTAAATATCATGAAACTGGAAAGGAAAAGGATTGCCTGCCCCAAGTTGGCCAG
TGGAATATGATGAACAAGAAAATGATTAATGGGATGACTGTAACGCGATGGGCCTGTATT

AACTTTTCACGTAGTGTACAAGAGAGTGTTGCTCGTGGGTTCTGCCATGAACTTGCGCAG
ATGTGCCAAGTGTCTGGCATGGAATTCAATCCAGAGCCTGTCATTCCAATCTATAGTGCC
AGGCCAGAGCAAGTGGAGAAAGCTTTGAAGCATGTATACCATGCATCCATGAACAAAACT
AAAGGAAAAGAATTAGAGCTTCTATTAGCTATCTTACCTGACAATAATGGGTCCCTGTAT
GGTGATCTTAAGCGAATCTGTGAAACTGATCTAGGTTTAATATCACAATGCTGTCTCACA
AAACATGTCTTCAAGATCAGCAAGCAATACCTGGCTAATGTGTCCCTAAAAATTAATGTT
AAGATGGGTGGTAGAAACACTGTTCTTTTGGATGCTATCAGTTGCAGAATTCCTTTAGTT
AGTGATATACCAACTATAATATTTGGAGCAGATGTAACCCACCCAGAGAATGGGGAAGAC
TCCAGCCCGTCGATAGCTGCTGTAGTAGCTTCTCAAGATTGGCCGGAGGTTACTAAATAT
GCTGGGTTAGTTTGTGCTCAAGCTCACAGACAGGAACTCATACAAGATTTGTACAAAACG
TGGCAGGATCCTGTTCGTGGCAATGTTAGCGGTGGCATGATCAGGGACCTTCTTGTTTCC
TTTAGGAAGGCAACAGGGCAGAAACCATTAAGGATTATATTTTACAGGGATGGTGTAAGT
GAAGGGCAATTTTATCAAGTTCTACTTTATGAGTTAGATGCAATTCGAAAGGCTTGTGCC
TCTCTAGAACCAGATTATCAACCACCAGTTACTTTCATTGTTGTACAAAAGCGGCACCAT
ACTCGATTATTTGCAAGCAACCACAGAGACAGGAGCAGCACAGACAAGAGTGGGAACATT
CTGCCTGGCACCGTGGTTGATTCAAAAATCTGTCATCCATCAGAATTTGATTTTTACCTC
TGTAGCCACGCTGGTATTCAGGGGACTAGTAGACCAGCTCACTACCATGTGCTATGGGAT
GAGAACAATTTTACAGCAGATGGGATGCAGTCTTTGACAAACAACCTCTGTTATACCTAT
GCAAGATGCACCCGCTCTGTCTCCGTTGTCCCTCCAGCATATTATGCACATTTAGCTGCT
TTTCGTGCTCGATTCTACATGGAACCAGAGATGCAGGAGAATGGGATGACAGGGCATGCT
GCTAAGGGCACACGACCAGCAGGAGAGTCAGGAAAAGTCCGGCCATTGCCTGCACTAAAG
GAGAATGTGAAGAGGGTAATGTTTTACTGCTAA
>Carica-papaya_.TU.supercontig_47.31
ATGTTCCTATGCCGGTCGCTGCGGCTCCTCCCACATCGCGACCAGCATTCATTCCGGTGG
CGGCTCCCAAGTGTGGCCATGTCTCCTGGTCCTTCCTCCTTAGCCTCGACCTCATCCTCA
CTAGTTCCATCTGACAAGCCAGCTGCAATTTCGGCTTCTGTCTCCGAACTGAACACTAAC
TTGGCGGGGAGACTGACATTGGAGCCGGCGCCGATGCCACCCGCATCGTCCAAGGCTATT
AGGTTCCCGGCGAGGCCAGGTGTTGGAAAGTCAGGATACAAGTGTCAGATTCGAGCAAAC
CACTTTTTAGTTGAGTTTGCGTATAGAGATTTATTTCAGTATGATGTGTCAATCACTCCT
GAGGTATCATCTAAGAAGACAAATCGCTTGGTTATAGAAGAGCTAGTAGTTTCATGCAGA
GCTTCTCACCTGAATAATCGACAACCGGCTTATGATGGAAGGAAATTCCTCTATACAGCT
GGAGCGCTGCCTTTTACATCTATGGAATTTGTGGTCAAGTTAACTAACATAGATGCTAGT
GTCAGCTCATCGACCTCCAAGAGGAATGAACGTGAATTTAAGGTGGCACTCAAATTGGCT
GGAAAGGCAGACCTTGATCATCTACAACAGTTTTTATCTGCCAGACAAATAGATGCGCCA
TATGAAACAATTCAGGCTTTGGATATTGTCCTCAGACAAATGGCATCGAAAGATAACATT
ATTGTGGGAAGATCGTTTTTCTCACCTGACATGAGGGGCAACCTTGGAGATGGCGTGGAC
TTTTGGTGGGGACATTACCAAAGCTTGAGACCAACCCAGATGGGTTTATCCCTTAATATT
GATTTGTCTGCCACGGCATTCTATCAGCCAATTCTGGTGACTGATTTTTTAAAAGAGAAT
TTGAAAATCAGAGACCTTTCGAGGCCATTGTCTGATCAGGATCGGGTGAAGGTCAGAAGG
GCCTTGAAATTGTTAAAGGTATCACTTACCTGTGGCCGATATCCTAGGGCTTATAAAATT
TTTGGCATATCAGATCAGCCATTAAGCGAACTAACGTTTTATCTTGATGAAGAGAGGCCA
AATATGTCCGTGGTCCAATATTTTTATGAAAAATACAAAACTAAGCTCAAATTTACTTCT
TTGCCTGCAATTCAAGCTGGAAGTGTATCAAGTCCTGCATTTCTGCCTATGGAGGTTTGC
CAAATTGCTGATAGACAGAGATATACAAAGAAGTTAAACGCTAGACAGATAACTAACCTG
TTAAGAGCAACCTGTCGGCGTCCTAATCAACGAGAAGATAATATTAAAGAGATTGCTCAA
TCAGTTAAGTTCAGTGATGCGCTTGTAACGACTGAGTTTGGAATTCATGTGGGACGGGAA
CTTGCTAAGGTTGAAGCTCGAGTTCTGCGCTCACCAACGCTGAAATATCACGATACTGGG
GTCCAGTCAAGGGTTGATCCCCGTTTAGGTCAATGGAATATGATTAACAAGAAAATGGTC
AATGGTGGGAGGGTTGAGTTCTGGACATGTGTGAACTTCTCTGAACAGGTGGATAGAAAT
TTGCCCTTCCAATTCTGTAGACAATTGATTGAAATGTGTAACAGCAAGGGAATGGAATTC
AACCCAAACCCACTACTTCCTATATGCTCATATAAACCAACTGAGATTGAGAGGGTTCTT
GTGGACATTTACAATCAATCGATGGAAAAACTTCAAAACATGAAGGGGAAACAACTTCAG
TTATTGATTGTAATTTTACCCGATGTGACTGACTCATATGGAAAAATTAAAAGAGTGTGT
GAAACAGAATTAGGAATTGTCTCACAATGCTGTCAACCTGGACAAGCAAAGAAACTCAGC
AAGCAATACTTTGAAAATATCGCTCTCAAGATAAATGTGAAGGCTGGGGGACGAAACACT
GTATTAGATGATGCTATTGAGGGAAGAATTCCTTTTCTTACTGATCGTCCTACCATCATT
CTTGGAGCTGATGTGACCCACCCACAACCTGGCAATGATTCTTTTCCTTCAATAGCAGCA
GTGGTGGCTTCCATGGACTGGCCTGAAGCAACCAAGTATCGAGGAATTGTATCTGCTCAG
TCACACCGAGAAGAAATTATACAAGATCTTTATAAAATAATTCACGATCCTGAAAGAGGT
CGTGCTTGTGCCTCCCTGGAGGAAGGATATCTTCCGCCAGTTACATTTATTGTGGTGCAG
AAAAGGCATCATACACGTCTTTTCCCTGCTGACCACACAAGGCACAACCTGACAGACAAG
AGTGGCAATATTCTACCAGGTACCGTGGTTGACACCAAGATTTGCCATCCCAGAGAATTC

GATTTTTATCTGAATAGCCATGCTGGAATTCAGGGCACTAGTCGTCCTGTGCACTACCAT
GTTCTGTGGGATGAGAACAAATTCACTGCTGATGCATTGCAAGTGCTCACTAATAATTTG
TGTTACACCTATGCAAGGTGCACACGGGCTGTTTCAATTGTGCCTCCTGCTTATTATGCG
CATCTAGCTGCTTTTCGAGCTCGGTATTATTTAGAAGAAACGATGGAGTCGGATAGTGAA
TCTGCAAGTGGAGGGAGGAGGGCGATCACGCCATTACCTTCCATCAAAGACAATGTGAAG
AACGTTATGTTCTATTGCTGA
>Carica-papaya_.TU.supercontig_75.90
ATGAGAGACCCAGATCAGGAGAACCGTTTGGTATCTAGGGAAAATCTAGACAGCTCCAAG
AATCAGAATAAATCATCCCAAATATCACCAAAAATCAACGTTGAAAACTTTGAAGAAGCA
AAGAAGAAGACGGTAAATAACGGAAGAAGAAGACGCCATAACAAGAGTAATCATAAAGCT
CTGAAGCTTGATTCTAATTCTGTGTTGTCTCCTCCTGCTCCATGCAAAGGCCTTGTGTTC
CATAAACGACCTGGTTATGGCCAGTTAGGAACTAAATGCGTGGTCAAAGCTAACCATTTT
CTTGCTCAGATGTCAATCTCCGACTTGTTTCATTACAGTGTTCAAATAAACCCAGAAGTC
ACTTCTCGTAAATTGGGAAAAGCTATAATGACCCAGTTGGTGAAAGTTCATAGAGACAAT
GAACTGGGAATGAGGCTGCCTGTTTATGATGGAGGAAGAAATCTTTATACTGCTGGATTT
TTACCTTTTACCTCTAAAGAGTTTACTGTAAAACTGGTTGACGAAGATGAGGGAACGGGC
AATATTACCAGGGAAAGAGAATTTAAAGTAACAATCAAGTTCGTTGCTGTTGCTAGTATG
GTGAAATTACAAGAACTTCTTTCAGGAAAACAAGTTGATAACCCTCAAGAAGCCATTAAT
GTTATTGATATTGTGTTGAGGGAACTGGCAGCTCAGAGGTATATATCAGTTGGGAGATTC
CTCTATTCTCCTGAGCTCAAGAAGCCGCAGCAGCTGGGCGGAGGACTGGAGTCATGGCGC
GGATTTTACCAGAGTATACGGCCTACCCAGATGGGATTATCATTGAATATTGACATGTCA
ACGACAGCATTCATTGAACCTCTTCCGGTTATTGAATTTGTTGCCAAATTACTGGGTAAA
GACGTGTATTCAAGGCCGTTATCTGACGCAGATAGAGTTAAGGTGAAGAAAGCTCTTAGA
GGTGTAAAGGTTGAAGTAACACATAGAAGACATGTTCGAAGAAAATACCGGATTTCTGGG
CTGACATCTCAGCCAACAAGGGAGCTAATTTTCCCTCTTGATGAGCACATGAACATGAAA
TCAGTTGTTGAGTATTTCCATGAAATGTATGGTTATACCATTCAATATGCCCATTTACCT
TGCCTTCAAGTCGGAAATCCAAGAAAAATCAACTATNNNNNNNNNNNNNNNNNNNNNNNN
NNNNNNNNNNNNNNNNNNNNNNNNNNNNNNNNNNNNNNNNNAGGGCTGAATGAGAAGCAA
ATTACTTCGCTTCTAAAAGTTTCATGTCAAAGGCCTTGTGAACAAGAAACTGATATTTTA
CAGACAATTCGCCAAAATGCATACGATCGAGATCCATATGCAAAGGAGTTTGGGATCAAT
ATAGACAGCAAGCTCGCATCAATCGAGGCTCGGGTTCTTCCACCTCCATGGCTCAAGTAC
AATGACGATGGAAAGGAGAAAGAACATTTGCCACAAGTTGGTCAGTGGAATATGATGAAC
AAGAAAGTGATAAATGGAAGCAGTGTAAGATACTGGGCCTGCATCAACTTCTCACGAAGT
GTCCAAGAGAGCACTGCCAATGGTTTCTGTCAACAATTGGTTCAAATGTGCCAAATCTCT
GGCATGGAATTCAACCAGGATCCTGTTATTCCCATATATTCAGCAAGGCCAGATCAGGTT
AAGAAAGCACTAAAATACGTATATCATGCAGCTTCAAACAAGCTCGAAGGGAAAGAATTG
GAATTACTGATTGCTATTCTTCCAGACAATAATGGTTCTTTGTATGGTGATTTGAAACGA
ATTTGTGAAACAGATCTCGGGTTGATTTCTCAGTGCTGTCTCACAAAACATGTTTTCAAG
ATTAGCAGACAGTATCTAGCAAACTTGTCACTTAAAATCAACGTCAAGATGGGAGGAAGA
AACACCGTGCTTTTAGATGCTTTAAGCTGGAGAATTCCATTGGTCAGTGACATTCCAACA
ATTATTTTCGGAGCTGATGTAACTCATCCGGAGACAGGAGAGGACTCGAATCCATCAATT
GCTGCTGTTGTAGCGTCCCAAGACTGGCCGGAGGTTACGAAATATGCAGGATTGGTATGT
GCTCAGCCTCATCGACAAGAACTTATTCAAGATTTATACAAAACTTGGCAGGATCCTCAA
GGAGGCACAGTTAGTGGAGGGATGATTAGGGATGGTGTAAGTGAAGGACAATTCTACCAG
GTTTTGCTATTTGAACTTGATGCCATTCGCAAGGCTTGTGCGTCACTAGAACCCGGTTAT
CAACCTCCGGTAACATTTGTTATAGTCCAAAAGCGCCACCATACTAGACTCTTTGCAAGC
AATCACAACGACAGAAGTCGTACTGACAAGAGTGGGAATATCTTGCCAGGCACGGTTGTG
GACTCTAAGATTTGCCATCCAACTGAGTTTGACTTCTATCTATGCAGCCATGCAGGAATT
CAGGGAACAAGTAGACCTGCTCATTATCATGTTCTGTGGGATGAGAACAATTTTACTGCT
GATGAGATTCAGACTCTGACAAACAATCTCTGCTATACGTATGCTAGATGCACTCGATCG
GTATCAGTAGTGCCTCCTGCATATTATGCTCATTTAGCGGCTTATCGAGCTCGATTCTAC
ATGGAACCAGATGCACCGGATAATGCTAAAACACGATGCTCCCGCCCAGGTGTCCGTCCC
TTGCCGGCATTGAAGGAGAAAGTGAAGAATGTGATGTTCTACTGCTAA
>Chlamydomonas-reinhardtii_Au9.Cre01.g071850
ATGTCGGGCCGAGGTCGCGGCGGCGGCCGTGGCGGCGGCGGCGGTGACCGCGGCGGCGGC
GGATACGGCGGCGGCGGTCGCGGCGGTGGTGGCGGTGGTGGCTATGGCGGTGGCGGTGGC
GGCGGTGGTCGTGGAGGGGGCGGTGGCGGTGGCTACGGCGGTGGCGGCGGACGCGGAGGC
GGTGGCGGCGGCGGTGGCGGCGGTGTGGTGTCTCCGGCGCAGGCGGCGGCCTCGCTGGAC
GCCATCCTCAAGTCCGCCAAGTCCCTCACCGCACCCGTCAAGGTGGAGACCACCGCCGAC
GGCCGGCCGCTGGGCATCACTCGGCGGCCCAGTGCGGGCAGTGTGGGCAAGGCGGTGGCG
CTGCTGGCCAACTACTTCGCACTGGCCACCACACCCGCCTTCCCGGGCCAGGCCTACCAC
TATGATGTGGAGATCCGCTCCGTTGAGACCCCCGGAGGTGGTGGTGGTGGCGGCCGCGGC

GGCGGCCGGGGCGGCGGCCGTGGCGGCGGCCGCGGCGGCCGTGGCGGCGCCGCCCCCGCG
CCCGCCACTGCCGAGGAGTCTGCCGGCGCGCCTGCCGCCGAGGCGGGCGGTGAGGACCTG
CCGCCGCGCCTGGCACACCGCGTGATGGCGTCGGCGGCGGCGGCGCACGGCTGGCCGGCG
GGGGCGTGGCGCTTCGACGGCCGCAAGAACCTGTTCCTGCCGGGAGAGCTTCTGCCGCGC
GAGGTGCGGGAGTGGCCTGTGACGCTCAAGCCGCGCGAGGGCGACAAGAGTGAGCGCGAC
AAGGGCTTCGTGGTGACCACCAAGTGGGCGGCGTGTGTGGGGCTGACGCAGCTGCAGGAC
TACCTGGCCAGGCGGCAGCAGACGGCGCCGCGCGACGCCATGCAGGTGTTGGACATTGTG
ATCCGGCACGCCTTCGCGATCGACCCGCGCTGCACCGTGGTGGGCCGCGGCTTCTACTAC
GGCGGGGAGGGCGTCATGCCGCTGGGGGGCGGCGCGGAGGTGTGGAGTGGATTCCAGCAG
GTGGAGTTCCCCATGCCCGGCGGCCGCGCGCGGCGCAAGGCGCTGGTGGGTCTGAGCGAG
CAGGGCGCCGACCGCACCATGTTCATGAACGAGAAGGAGGGGCGTGAGATGTCGGTGGCC
GAGTACTTCCGCTCCACGGGCCGCCCGCTGCGCCACCCCGGCCTGCCCTGCGCCAACGTG
GGCGACCGCCGCCGCGCCGTGTTCATTCCCGTGGAGCTGTGCACTGTGGTTGCTGGCCAG
CGGCGCATGAAGCTGGACGCCACGCAGTCGGCCGGCATGATCACCGCCGCCAAACAGGAC
CCGGCAGTCAAGAAGGAGGCATGCGACAAGCAGGCCAAGCGGGTGGCTGAGGCTCTGGCG
GCGGGCGGCACGGACAGGTGCTGGGGCCTGAAGCTGGCCACTGGCATGCTGCCGGTGCAG
GGCCGCGTGCTGCCCAACCCGGTGCTGCAGTACGGCGACCCGCAGGCCTTCGACGCGGGG
CCGCTGGGCAGCTGGAACCTGCGCAATGTCAAGTTTGTGGACCCGCGCGCTCTGGACTCC
TGGGGCGTCGCCGTCATGATGAACCAGGCGGACGTGGACTTTGACGGCGACAACAGCCTG
TGGCAGTTCCTGGAGGACCTCACCGGCGGCATGATCCGCTGCGGCATGCGCGTGGCCAGC
CCCGTCACCGCAGCCAGCACAGACAGCCCGCCCGTGGAGTTTGGCGGCATGCCGGGCGGC
GGCGGCCGCGGCGGCGGGCGCGGCATCGAGGCCACCATGCGCGCGGCGGCGGACGCGGCG
GCGGCGCGGTACAAGAAGCCGGCGCAGCTGGTGCTGGTGGTGCTGCCGGAGAAGACCGCG
GACGAGTACCGGGAGGTGAAGCGCGTGTCGGACATTGAGCTGGGCATACCCAGCCAGGTG
GTGGTGGCGTCCAAGGCGCGTGTGGGCTACCGCGCGCACAAGGGCGGCGGCCCGCAGTAC
TGCGCCAATGTGGCCATGAAGATCAACAACAAGCTGGGCGGAGTCAACGTGCAGCTCAGC
GGCGGACTGCGCAACATGCCGGTGCTGGGCGGCGCGGGCGCCGTGCCCTTCATGGTGCTG
GGCGCCGACGTGACGCACCCCACCGGCGCCGCGGCGCGCGCCGACAGCCGCGACCCCTCC
GTGGCGGCCGTGGTGGCCAGCCTGGACGCCAGCCTGGGCCGCTGGGCCAGCCGCGTGCTG
CTGCAGGCGGGGCGGCAGGAGGTGATCACGGGCATGTGCGGCGCCACAAAGGAGCTGCTG
CTGGAGTTCTACAGGGCAAACAAGCAGGTCAAGCCGCAGCGGCTGGTCATGTACCGCGAC
GGCGTGTCGGAGGGCCAGTTCGAGCAGGTCCTGGCTGAGGAGTACACGGCCCTGCGCCGC
GCCTGCCGCGAGCTGGAGGAGGGCTACCGGCCCGCCATCACCTTCGTGGTGGTGCAGAAG
CGCCACAACACCCGCCTGCTGCCCAGCGACCGAGCCGCGTCCGACCAGAAGGGCAACGTG
GTTCCGGGCACTGTGGTGGACAGCGGCATCACCGCCCCGGACGGCTTCGACTTCTACCTC
AACAGCCACGCCGGGCTGCAGGGCACCAACAAGCCCGCCCACTACCACGTGCTGGTGGAC
GAGATCGGCTTCGGCGCCGACGGCATCCAGCTGCTCACCTACTGGCTGTGCTACCTCTAC
CAGCGCACAACAAAGTCGGTCAGCTACTGCCCGCCCGCCTACTACGCCGACCGCGCCGCC
TTCCGCGGCCGCACGCTGCTGGCCGCCTCCTCCTCCGCCTCGGACAGCGCCTCCGAGACC
GCCTCCCGCAGCGGCCGCGGCGCAGGCGCGGCTGAGGGCGGCGCCAGCGCCCCGCCCACC
TTCGCAGGCATCCACCGCAACCTGACCAACGTGCTGTACTTCATGTAG
>Chlamydomonas-reinhardtii_Au9.Cre04.g214250
ATGGCGGAGGCTACGGCGGAGGCGGGGGAGGCTCCCGCGGCGGCGGCGGCGGCGGTGGGA
GCGGTGGCGGCGACCGAGGATACGGCGGTGGCGGCGGCGGCCGCGGAGGTGGCGGCTATG
GTGGCGGCAGCGCTGGCGGCGGTGGCTACGGTGGCGGCGGCGGTCGGGGCGGCGGAGGCG
GCGGAGGCGGCTATGGTGGAGGCGGCGGCGGCCGCGGAGGTGGAGACGACGCCCGACGGC
CGGCCGCTGGGCATCACGCGCCGCCCCAACGCCGGCACCGTTGGCAAGGCGGTGGCGCTG
CTGGCCAATTACTTCGCACTGGCCACCACACCCGCCTTCCCGGGGCAGGCCTACCACTAC
GATGTGGAGATACGCTCCGTTGAAGAGGCGGCAGGAGGAGGAGGAGGCGGCGGCCGTGGC
GGCGGCCGTGGCGGCGGCGGCGGCGGCGGCGGCGGGCGCGGGGGCCGGCGTGACCTGGCG
CCTGCTAGTGCTACTGGCGACGTGCCGGGTGGCGCCGCCGCCGCCGCCGCCGCTGAGGAC
CTGCCGCCGCGGCTGGCGCACCGCGTGATGGCGGCGGCGGCGGCGGCGCACGGCTGGCCG
GCGGGGGCGTGGCGGTTTGATGGGCGGAAGAACCTGTTCCTGCCGGGGGAGCTGCTGCCG
CGCGAGGTGCGGGAGTGGCCTGTGACGCTCAAGCCGCGCGAGGGCGACAAGAGCGAGCGC
GACAAGGGCTTCGTGGTGGCTACCAAGTGGGCGGCGTGTGTGGGGCTGACGCAGCTGCAG
GACTACCTGGCGCAGAGGCAGCAGACGGCGCCGCGCGACGCCATGCAGTCCTTCAAGGCG
GTCCAGGCCGGCCTCATGCTCAACCTGGACTCCTCCTTCGCAGCCTTCATGTCCGCCCGC
CCGCTGCCCGAGCTGCTGGCGGAGGGCGCGGGCGTGCGCGGCGACCCGGCGCAACTGGCG
GCGGCCGACCCACGCCGGCTGAGGGCGGCTGCCAGGTCGCTGGTGGGGTTCAAGGTGGAG
TTCCCCATGCCCGGCGGCCGCGCTCGGCGCAAGATGCTGACGGGGCTGAGCGAGCAGGGA
GCAGACCGCACCATGTTCATGAACGAGAAGGAGGGGCGTGAGATGTCAGTGGCCGAGTAC
TTCCGCTCCACTGGCCGCCCGCTGCGCCACCCCGGCCTGCCCTGCGCCAACGTGGGCGAC

CGCCGCCGCGCAGTCTACATTCCGCTGGAGCTGTGCACTGTGGTTGCTGGCCAGCGGCGC
ATGAAGCTGGACGCCACGCAGTCGGCCGGCATGATCACCGCCGCCAAACAGGATCCGGCG
GTGAAGGCCGAGGCATGCAACAAGCAGGCCAAGCGGGTGGCCGAGGCTCTGGCTGCGGGC
GGCACTGAGCGGTCCTGGGGCCTGAAGCTGGGCACCGGCATGCTGCCGGTGCAGGGGCGC
GTGCTGCCCAACCCCGTGCTGCAGTACGGCAACCGACAGGACTTCGACGCGGGGCCGCTG
GGCAGCTGGAACACCCTCAATGTCAAGTTTGTGGACGCACGCGCACTGGACTCGTGGGCC
GTGGCCGTCATGATGAACCAGGCGGACGTGGACTTTGACGGCGACAACAGCATCTGGAAA
TTCCTGGAGGACCTCTGCTCGGCCATGATCACCCGCGGCATGCGCGTGGCCAGCCCCGTG
ACCCGGGGCTCCAACGACAGCCCGCCAGTGGAGTACGGCGGCGCGGCGGCGGGCGGCGGC
GCAGCAGGCCGCGGCGGCGGGGCCCGCGGCGTCGAGGCGACCCTGCGCGCGGCGGCAGAC
GCGGCGGCGGCGCGGTACAAGAAGCCGGCGCAGCTGGTGCTGGTGGTGCTGCCGGTCAAG
CCCTCGGACGAGTACCGGGAGGTGAAGCGCGTGTCGGACATCGAGTTGGGCATACCCAGC
CAGGTGGTGGTGGGGGGCAAGGCGCGTGTGGGCTACCGCGAGCAGAGGGGCGGCGGCCCG
CAGTACTGCGCCAACGTGGCCATGAAGATTAACAACAAGCTGGGCGGGGTCAACGTGCAG
CTCAGCGGCGGACTGCGCTACATGCCCGTGCTGGGCGGCGCGGGCTCCGTGCCCTTCATG
GTGTTGGGCGCCGACGTGACGCACCCCACCGGCGCCGCCGCGCGCGCCGACAGCCGCGAC
CCCTCCGTGGCGGCTGTGGTGGGCAGCCTGGACGCCAGCCTGGGCCGCTGGGCCAGCCGC
GTGCTGCTGCAGGCGGGGCGCCAGGAGGTGATCACGGGCATGTGCGGCGCCACAAAGGAG
CTGCTGCTGGAGTTCTACAGGGCAAACAAGCAGGTCAAGCCGCAGCGGCTGGTCATGTAC
CGCGACGGCGTGTCGGAGGGCCAGTTCGAGCAGGTCCTAGCGGAGGAGTTCACGGCCCTG
CGCCGCGCCTGCCGCGAGCTGGAGGAGGGCTACCGGCCCGCCATCACCTTCGTGGTGGTG
CAGAAGCGCCACAACACCCGCCTGCTGCCCAACGACCGCGCGTCGGCCGACCCCAAGGGC
AACGTGGTCCCCGGCACGGTGGTGGACAGTGGCATCACCGCCCCGGACGGCTTCGACTTC
TACCTCAACAGCCACTCCGGACTGCAGGGCACCAACAAGCCCGCCCACTACCACGTGCTG
GTGGACGAGATCGGGTTCGGCGCCGACGGCATGCAGCTGCTCACCTACTGGCTGTGCTAC
CTCTACCAGCGCACCACCAAGTCGGTCAGCTACTGCCCGGCCGCCTACTACGCCGACCGC
GCCGCCTTCAGGGGCCGCACACTGCTGGCCGCCTCCTCCTCCGCCTCTGACAGCGCCTCC
GAGTCCGGCAGCCGAGCCGGCCGCGGCGCGGGCGCGGCTGAGGGCGGCGCCAGCGCCCCG
CCCACCTTCGCCGGCATCCACCGCAACCTGTCCAACGTGCTGTACTTCATGTAG
>Cucumis-sativus_Cucsa.082260
aTGCCCTACATGCAAATGGAGCCAGAAGATAAATATGTGATACCAGCTTCTCAAATGGCG
TTGAAATCCCACCATGGCTCAAATGATGGTGAGAAGACTAGTTTATGGAAAAGGAAAGGA
AGGAGGAGAGCAAAAGGACTTCCCATGGAAGGATTGAAGCAAAAGGAGCTCGATCCAAAT
CTGGAGTTTCCCTCTTCTTCTTGCAAAAGCTTAACATTTCCAAGAAGGCCAAACTTTGGC
CAGCTGGGGACTAAATGCTTGGTTAAAGCCAACCACTTCCTAGCCATAATACCTGAATCA
GACATAAGCCATTATAACGTTAAGATAACTCCAGAAGTTACTTCTCGTAAAATGAAGAAG
AATATACTGACCGAGTTGGTCAAACAATATAGAACCACAGAGCTGGGGATGCGTCTGCCG
GTTTATGATGGAGGAAGTAACCTGTACACAGCTGGATTGCTGCCTTTCATATCAAAAGAG
TTCACTGTCATTTTGGCTAATGAAGAAGAGGGGACAGGCACACCCAGGGAACGAGAATTT
AAAGTGCAGATTAAGTTTGTAACTTTGGCTAGCATGCACCAGTTAAGAGAATTACTCGCT
GGCAAACAAGTTAATAACCCTCAAGAAGCTTTGACCATTATTGACATCGTCTTGAGGGAG
CTCCATGCTCAAAGATATATATCAGTTGGACGATCCTTTTACTCTCCTTGTATTAAGAAG
CCTCAGCATGTTGGGGGTGGTCTGCAAGCATGGCGAGGCTTCTATCAAAGCATCCGGCCT
ACTCAAATGGGATTGTCTCTAAATATTGACATGTCATCCACTGCATTCATTGAAGCAATC
CCGGTCATTGATTTTGTCGCTCAACTCTTAAACAAAGACGTGTACTCTAGAACATTGTCT
GATGCTGATCGTATCAAGGTCAAAAAAGTACTTAGAGGTGTAAAAGTTGAAGTTACACAT
AGAGGAAACGTACGAAGGAAATATCGGATTTCAGGGCTAACATCACAGCCTACAAGAGAG
CTAATTTTCCCTCTTGATGAGCAGATGAACATGAAATCTGTAGTTGAGTACTTCCAAGAG
ATGTATGGGTATACCATTCAGTATACACATTTGCCTTGCCTTCAAGTAGGAAACCAGAAG
AAGGTGAATTATCTACCCATGGAGGCATGTAAGATACTCAAAGGACAGAGATACACAAAA
GGACTTAACGAAAAGCAAATAACTTCCTTGTTGAAGGTTTCATGCCAAAGACCCTCTGAT
CAGGAAATGGACATTTTACAGACTGTTCATGAGAATGCATATGAGGCAGATCCATACGCA
AAAGAATTTCGAATCAGCATTGACAACAAGCTTACATCAGTTGAAGCACGAGTCCTTCCA
TCTCCATGGCTAAAATTTCATGATACTGGAAAAGAAAaGGAACATCAGCCTCAAGTTGGT
CAGTGGAATATGATGAATAAGAAAGTTATAGATGGAAGTGTTATAAGATACTGGGCTTGT
ATAAATTTCTCACGTAACGTTCAAGAGAGCATAGCTCGTGGTTTCTGTCAACAGCTGGTC
CAGATGTGCCAGATCTCTGGCATGGAATTTAACCCAGATCCTGCAATTCCTATATACTCA
GCAAGACCAGATCAAGTGAAGAAGGCCTTAAAATTTGTATATGGTGCGGTTTATAACAAA
AATGAAGGGAAAGAGTTAGATTTGCTGATAGCTATTCTTCCTGACAACAATGGCTCATTA
TATGGTGATCTTAAAAGGATCTGTGAAACAGAATTGGGATTGATATCCCAGTGTTGTCTA
ACAAAACATGTCTTCAAGAAGAGCAGACAGTACTTGGCGAACGTGTCTCTTAAGATCAAT
GTCAAGATGGGTGGAAGAAACACTGTtCTTTTAGATGCTTTACGGGCAAGAATTCCTCTA

GTCAGTGACATACCAACCATTATTTTGGAGCTGACGTCACACATCCAGAATCTGGAGAG
GATTCTCTTCCATCAATAGCAGCTGTTGTGGCTTCCCAAGATTGGCCAGAAGTTACAAAA
TATGCTGGATTGGTATGTGCGCAGCCGCACAGGGAGGAACTAATTCAAGATTTATTCAAA
ACGTGGAAGGATCCTCATCGGGGAACAGTTGCCGGGGGTATGATAAGGGAGCTCTTGCTT
TCATTTAAGAAGGCCACTGGACAAAAaCCATTAAGGATAATATTCTATAGGGATGGGGTC
AGTGAGGGTCAGTTCTACCAGGTTCTACTGCACGAACTCGATGCCATACGCAAGGCCTGT
GCTTCACTAGAACCCAGTTACCAACCTCCAGTAACTTTTATCATAGTCCAAAAGCGACAC
CACACCAGACTCTTTGCATCCAATCACAACGATAGGAGTAGCACTGACAAGAGTGGTAAC
ATTTTACCAGGTACTGTCGTGGATTCAAAAATATGTCATCCAACTGAGTTCGACTTTTAT
CTATGCAGTCACGCAGGAATCCAGGGAACAAGTCGTCCTGCACATTATCACGTTCTTTGG
GATGAGAACAATTTCAGTGCGGATGAAATTCAATCTCTGACTAATAATCTCTGTTACACG
TATGCTCGGTGCACAAGATCGGTCTCAGTAGTGCCTCCAGCATACTACGCTCATTTAGCA
GCATACAGAGCTCGATTCTACGTAGAACCCGACGCACAGGAGAATGCCAAAAGATGCTGC
ACTCGAACCACAAATGGGTCCTGTGTCCGGCCTCTGCCTGCGCTGAAAGAGAGAGTGAAA
AATGTAATGTTCTATTGCTGA
>Cucumis-sativus_Cucsa.112480
ATGGGGAGGAAGAAGAGAACCGATGCTGGTGGTGAAAGTTCTGAGTCTCAAGATGGTGGT
GGTCGTGGCTCCCAAAGATCAGCTGAAAGGAGAGACACCCCACAGCAACATGGTGGTAGT
GGGTACCAGCGTGGGAGAGGTTGGGGTTCTCAAGGGGGACGTGGAGGCCAAGGTGGTGGA
GGGCGTGGCCGAGGAACGTCTCAACATCAGCACTATGGAGGACCTCCCGATCACCAAGGC
AgGGGTAGAGGTGGGCCATATCATGGAGGACATAACAACTATGGTGGTGGTGGTGGTAAT
CGTGGTGGCATGGGTGGTGGTGGCATTgGGGGAGGACCTTCTTCTGGTGGACCATCCAGG
TCACTAGTTCCCGAGCTGCACCAAGCAACCCCAATGTATCAAGGAGGGATGACTCAGCCA
GTCTCATCTGGGGCgAGTTCTTCCTCCCATCCATCTGACACTTCATCAATTGATCAACAA
TTTCAACAAAtTTCCATTCAACAGGAGTCCTCTCAAAGTCAAGCAATTCAACCTGCACCA
CCATCTAGTAAATCCTTGAGATTCCCACTGAGACCAGGAAAGGGTAGCTCTGGCACAAGG
TGTATTGTTAAGGCTAACCATTTCTTTGCTGAGTTACCCGACAAAGATCTTCATCAGTAT
GATGTTACTATTACCCCAGAAGTTACTTCACGTGTCTACAACCGAGCTGTAATGGAACAG
CTAGTTAAATTATATAGAGTCTCACACCTTGGAGATCGGCTTCCTGCTTATGATGGAAGA
AAAAGTTTATATACGGCTGGTCCCCTCCCTTTTACATCTAATGAGTTTAGAATAACTCTT
TTTGATGAAGAAGATGGATCTGGTGGACAAAGACGGGAAAGGGAATTTAAAGTTGTAATC
AAATTGGCTGCACGTGCTGATCTACACCATCTTGGACTCTTTTTGCAAGGCAGACAGGCC
GATGCCCCTCAAGAAGCTCTTCAAGTTCTGGATATTGTATTACGAGAATTACCTACTTCA
AGGTATTGTCCAGTGGCCCGATCATTTTACTCTCCAGACCTTGGCAGACGTCAGACACTG
GGTGAGGGGCTGGAAAGTTGGCGTGGTTTCTATCAGAGTATCCGCCCTACTCAAATGGGA
CTCTCCCTTAATATTGATATGTCATCTACTGCTTTCATAGAGCCTTTGCATGTCATTGAA
TTTGTCACTCAACTTCTCAATCGTGATGTGTCGTCCAGACCATTATCTGATGCTGACCGT
GTGAAGATAAAGAAGGCTCTTCGAGGTGTCAAGGTTGAAGTGACACATCGTGGGAATATG
CGCAGAAAATATCGCATTTCTGGTCTGACATCACAAGCAACACGCGAGCTAACTTTCCCT
GTCGATGAGAGAGGTACTATGAAGTCAGTGGTTGAGTACTTCTACGAAACGTATGGGTTT
GTTATCCAACATACCCAGTGGCCTTGTCTTCAAGTAGGGAATCAGCAGAGACCTAATTAT
CTACCGATGGAAGTATGTAAGATTGTTGAAGGCCAGAGGTATTCCAAAAGGCTTAATGAA
AGACAAATAACTGCTCTGCTCAAGGTTACATGCCAACGGCCTAAAGATAGAGAGGAAGAC
ATTATGCAGACGGTACACCATAATGCATACCATAATGATCCATATGCCAAGGAGTTTGGA
ATCAAGATTAGTGAGAAGCTTGCTTCTGTCGAAGCTCGTATTCTTCCAGCACCATGGCTA
AAATATCATGACACCGGCAGAGAAAAAGATTGTTTGCCTCAAGTTGGACAATGGAATATG
ATGAATAAGAAAATGTTCAATGGTGGTACTGTTAACAACTGGATGTGCATTAATTTCTCT
CGCTATGTGCAAGACAGTGTTACTCGTGGCTTTTGTTATGAACTTGCTCAAATGTGTTAC
ATTTCTGGCATGGCTTTCAATCCAGAACCAGTTCTTCCCCCTATTTTTGCTCGTCCAGAT
CATGTGGAAAAGGCCCTTAAAACTAGGTACCATGATGCAATGAGCATTCTCCAACCCCAG
GGCAAGGAGCTTGATTTGCTTATTGTTGTATTACCAGATAACAACGGATCCCTTTATGGT
GATTTAAAGCGTATCTGTGAAACTGATCTTGGCCTCGTTTCCCAGTGCTGTTTGACTAAA
CATGTTTTCAAAATGAGTAAACAGTATTTGGCCAATGTGGCCTTAAAAATAAATGTTAAG
GTTGGGGGAAGGAATACGGTTCTTGTTGATGCATTATCTAGGCGCATACCTTTGGTTAGC
GACCGGCCTACCATTATATTTGGCGCCGATGTAACTCATCCTCACCCAGGAGAAGACTCC
AGTCCATCCATTGCTGCTGTTGTGGCTTCTCAAGATTGGCCGGAAGTTACAAAATATGCT
GGTTTGGTTAGTGCTCAAGCCCACAGGCAAGAACTCATTCAAGATCTTTTCAAAACATGG
CAGGATCCTGTTAGGGGGACAGTAACGGGTGGAATGATCAAGGAACTCCTCATCTCCTTC
CGTCGAGCAACTGGACAGAAACCTCAACGCATTATATTTTACAGGGATGGGGTTAGCGAA
GGACAGTTCTACCAAGTTTTGTTGCATGAGCTGGATGCCATTAGAAAGGCATGTGCTTCT
TTGGAACCAAACTACCAACCTCCAGTGACATTTGTGGTGGTACAGAAGCGTCATCACACT
AGGTTGTTTGCTAATAACCATTCTGATCGCCATACAGTCGATAAGAGTGGGAACATATTA

CCTGGCACGGTTGTCGATTCTAAAATTTGTCACCCCACGGAGTTCGATTTTTACCTGTGC
AGTCATGCTGGAATCCAGGGTACAAGTCGTCCTGCTCATTACCATGTTCTATGGGATGAG
AACAAGTTCACTGCTGATGGATTGCAGACCCTAACGAATAATCTTTGTTACACATATGCT
AGGTGTACTCGCTCCGTTTCTATTGTGCCCCCAGCGTACTACGCTCATCTTGCTGCTTTC
CGAGCTCGTTTCTATATGGAACCTGAAACCTCTGACAGTGGGTCAATTAGCAGTGAAGTT
GCTGGACGTGGTGGCGTCGGTGGTGCTCGCAGTACACGGGCACCAGGTTTGAATGCTGCT
GTTCGACCTTTGCCAGCTCTGAAAGAGAATGTCAAGAGGGTTATGTTCTATTGTTGA
>Cucumis-sativus_Cucsa.152920
ATGAGTTCAAATGAGCCAGATGGAAATGGAGCTGATGCAGTGTTGCCTCCTCCACCTCCG
CCTCCCCCAATTCCCCCAAATGTCGTTCCAATTCAAGCTGAGCTAGAGCAGGCTCCCGAA
ATTGTGAAAAAAAaGGTTGTGCGAGTTCCAATTGCACGACGTGGCCTTGCTTCAAAAGGC
CAGAAGATATCTCTGCTCACGAATCATTTTAAAGTGAATGTGACCAATATAGAAGGGCAC
TTTTTTCACTATAGTGTTGCACTTGCATATGAAGATGGTCGCCCAGTTGATGGCAAGGGC
GTAGGAAGAAAGGTTATCGACAAGGTTCATGAAACATATAATTCTGAACTGGCCGGTAAA
GATTTTGCTTACGATGGAGAAAAGAGTTTGTTTACTGTTGGGCCTCTCCCAAGGAATAAA
CTTGAATTTACTGTTGTTCTTGAGGATATTACGTCAAACAGGAACAATGGTAACTGTAGT
CCCGATGGGCATGGAAGTCCCAATAATGGAGACCGAAAAAGGATGAAACGACCCTACCGT
TCAAAATCATTTAAGGTAGAAATCAGCTTTGCGGCTAAGATACCAATGCAGGCTATAGCA
AGTGCTTTACGTGGTCAGGAATCTGAGAATTTCCAAGAGGCCATCAGGGTGTTGGATATT
ATCTTACGGCAGAATGCATCAAAGCAAGGTTGCCTACTTGTCAGACAGTCATTTTTCCAT
AATGACCCAAACTCTTGTACTGATGTAGGCGGTGGAGTTCTTGGCTGTAGAGGCTTCCAC
TCTAGTTTTAGAACAACACAGAGTGGCCTCTCTTTGAATATTGATGTTTCCACAACCATG
ATTATACAGCCTGGTCCTGTTGTGGACTTTTTAATTGCAAACCAGAATGTCAGAGATCCT
TTCTCACTTGACTGGACCAAGGCTAAACGGACACTCAAGAATTTGAGGATTAAAGCAAGC
CCCTCCAATGCAGAATACAAGATAACTGGATTAAGTGAAAAGCCTTGTAAAGAGCAAACG
TTTACTTTGAAACAGAAAGGTGGAAATGATGAAGACTGCATTGAAATCACTGTTTATGAT
TATTTTGTCAAGCATCGGAACATCGAACTTCGATATTCATCAGATCTTCCTTGTATAAAT
GTGGGGAAGCCCAAGCGTCCCACTTTTATCCCTGTTGAGTTGTGCTCTTTGGTATCACTA
CAACGATACACAAAAGCACTGTCCACATTTCAAAGAGCCTCACTTGTTGAGAAATCGAGG
CAAAAGCCACAAGAAAGGATGAGGGTTTTGTCCGATTCGTTGAGAAGGAACAAATATGAT
GCTGAGCCGATGTTACGGTCATGTGGAATTGCTATTAATTCAAGCTTCATTCAAGTTGAA
GGCCGTGTTCTACCTGCACCCAAGTTGAAAGTGGGCAATGGGGAAGATTTTTTTCCTCGT
AATGGCCGATGGAATTTCAATAATAAGAAATTGGCTCAACCTACTAAAATAGAGCGATGG
GCTGTGGTGAACTTCTCAGCGCGCTGTGATACACGTGGCCTTGTCAGAGACCTGATAAAA
TGTGGTGATATGAAGGGCATTGCAATAGAGGCTCCTTTTGATGTTTTTGAAGAAAATCCA
CAGTTTAGACGTGCCCCACCTATGGTTAGAGTGGAGAAGATGTTTGAGGAAGTTCAGTCT
AAACTTCCTGGACAACCACAATTTCTTCTTTGTCTACTACCTGAGAGAAAGAATTCTGAT
CTTTATGGTCCTTGGAAGAAAAAAAATCTTGCAGAATTTGGAATTGTTACTCAGTGTATT
GCTCCTACACGGGTAAATGACCAATACCTCACAAATGTACTCCTTAAGATCAACGCCAAG
CTTGGTGGGCTTAATTCCTTGTTAGCAGTGGAGCATTCTCCATCCATTCCTATGGTTTCA
AAAGTTCCAACAATTATCCTGGGTATGGATGTTTCACATGGTTCTCCAGGACAGTCAGAT
ATTCCGTCTATTGCTGCGGTGGTCAGTTCTAGACAGTGGCCATTGATCTCTCGTTACCGA
GCTGCAGTACGTACCCAATCTCCAAAAGTGGAGATGATTGATTCTTTGTACAAGCGTATT
TCTGATACTGAAGATGATGGAATAATGAGGGAGCTTCTACTTGACTTCTATACTAGTTCA
GGAAAAaGAAAGCCAGATCAGATTATCATATTCAGGGATGGTGTCAGTGAATCCCAATTT
AACCAAGTGCTAAATGTTGAATTAGATCAGATTATCCAGTCTTGCAAGTTCCTTGATGAA
AATTGGAACCCCAAGTTTGTGGTGATCGTGGCCCAGAAGAACCATCACACTAAGTTCTTT
CAGCCTGGATCTCCTGACAACGTTCCTCCCGGAACTATTATTGACAACAAAATTTGTCAT
CCAAGAAACAACGATTTCTATCTCTGTGCTCATGCTGGAATGATTGGTACTACAAGGCCA
ACTCATTATCACGTTCTGTTAGATGAAGTTGGTTTTTCTGCAGATGACCTGCAAGAACTA
GTGCACTCTCTATCCTACGTATACCAAAGAAGTACTACAGCGATTTCTGTTGTTGCTCCC
GTCTGCTACGCTCATTTGGCAGCCACCCAGATTGGGCAGTTTATAAAGTTTGAAGAAACA
TCAGAGACAGCCTCGAGCGATGGCGGGCTGACAAGTGCAGGAGCAGTTCCTGTGCCCCAA
CTGCCGAGATTGCAGGAGAAGGTTTGCAATTCCATGTTCTTCTGTTGA
>Cucumis-sativus_Cucsa.185140
ATGGTTAACATAACTAATACCGAAGGGAAAATGAGCGAAACCTCTCCTTTGCCCCTAGAG
CCATCTCTACCTCCTGATATGAAGCCAGAAAAAGCAATGCCTCAGTATACGATCATGAGT
AGACGTGGTGTAGGAAGTAAAGGGAGACGCATTCCTTTGCTCACTAACCACTTCAGAGTA
TCTCTTAATGCTCCAGATTTAGTTTTCTATCAATACGCTGTTTCAATATGCTATGAAGAT
GGCAGGCCCGTTGAAGGGAAGGACATTGGGCGGAAATTGATGGATAAACTTTATCAAACT
TACTCTACTGAACTTGCTAATAAAAGGTTTGCATACGATGGAGAAAAAATGTCTTTACACC
ATTGGTCCCCTGCCACaAAAAAAGCTTGAGTTCTCTGTGGTGCTAGAGGGATTCTGTGCA

AAAATAGAAACAGGTAGCTCTGGGGGAAGTGGGAGCCCAAATCGGACTGGAAAGAGGTTT
AAGCGTAGTTCTCAGTCGAAGACTTTTAAGATTGAGCTAAGCTTTGCTACTAAAATTCCA
ATGAAGTCCATTTTTACTGCCCTCAAGGGATCAGAGGAAGATAATGGCAGCACTCAGGAT
GCATTAAGAGTGCTTGACATTATCCTGCGGCAGCAAGCAGCTAACCGGGGATGCCTTTTG
GTAAGGCAGTCGTTCTTTCATGATGACTCAAGGAACTTTGCTGATGTAGGAGGAGGGGTA
ACAGGAGTACGGGGATTCCATTCTAGCTTTAGGTTGGCACAGGATGGATTATCATTGAAT
ATGGATGTTTCTACCACAATGATCCTGAAGCCTGGGCCAGTTATTGATTTCCTAATAGCA
AATCAGAATGTACGGGAACCACGCTATATTGATTGGGGGAAGGCaAAAAAAATGTTAAAG
AATTTGAGAGTCAAGGCAAGACATCGGAACATGGAATTTAAAATCATTGGTTTAAGTGAA
AAGCCTTGTAACCAACAATTTTTTTCCATGAAACTGAAGAATAATGGCAGCACTGATGGG
GAGATGGTTGATATTACTGTTTATGAATACTTTGTCAGACACTGTGGCATTGAACTGACT
CATTCTGCTTATTTGCCATGTCTAGATGTTGGGAAACCTAAACGACCAACTTATATACCT
TTGGAGTTGTGCTCACTTGTTTCACTTCAACGGTACACAAAAGCTTTGTCTTCAATGCAA
AGAGCATCTTTGGTAGAGAAATCAAGGCAGAAGCCTCAAGaAAAAATaAAAATTGTTACT
GATGCTTTGAAAAATTATCGATACGATGAAGATCCAGTGTTAGCTCAGTGTGGAGTAAAA
ATTGATAGACAACTGACACAGCTTGATGGTCGTGTACTCGAATCTCCAAAGTTAAAGGTT
GGTAGAAGTGATGATTGTATTCCACGTAATGGACGGTGGAACTTTAATAACAAGACACTT
CTGAATCCCACTCGCATTAACCGTTGGATTGTTGTCAATTTCTCGGCACGTTGTGATATT
AGCCACATATCACGTGAACTTATAAATTGTGGAAGAAATAAAGGAATTCATATTGAAAGA
CCAATTACCTTGATTGAGGAAGACCAGCATTCTAGAAGAGCCAGCCCTGTTGACAGGGTG
GAAAATATGTTCGAACAGATGATGGCAAAAATGTCAGATGCACCAATGTTTATTCTTTGT
GTCCTCCCAGAAAAGAAAAATTCGAATATTTACGGACCCTGGAAGAAAAAGTGTTTGTGC
GACTTTGGGATTTTTACACAGTGCATTTCCCCCACTAAAATTAATGATCAGTACATTACT
AATGTACTTCTTAAGATTAACTCCAAGCTGGGAGGTATAAACTCATTGTTGGCCATTGAG
CATGCATCATGTGTTCCATTGATAAAAGATACTCCAACGTTGATCTTGGGAATGGATGTG
TCTCATGGGTCTCCTGGACGATCAGATGTTCCATCCATTGCTGCAGTTGTTGGATCCAGA
TCCTGGCCTTTGATATCAAGGTATAGAGCAGCTGTACGGACCCAGTCGCCTAAGGTGGAA
ATGATTGATGCTCTATTTAAGCCTCTGGAGAATGGCAAGGATGATGGTATCATTAGAGAA
TTGCTTTTAGATTTCTATAGCACCAGCAAGGGCCGCAAACCAACTCAGATTGTTGTCTTT
AGAGACGGAGTTAGTGAATCTCAATTTAATCAAGTTTTGAACATTGAGTTGGATCAAATA
GTCAAGGCTTACCAACATCTTGGAGAGGTTAACATTCCAAAGTTCACGGTTATTATAGCA
CaAAAAAATCACCATACAAGAtTTTTTCTACCTGGTGCCTCTGAAAATGTTCCACCTGGA
ACGGTTGTTGACACGAAAGTTGTACATCCAAAAAATTACGACTTCTACATGTGTGCTCAT
GCGGGAATGATCGGCACGTCAAGGCCAGCACACTACCATGTCCTGCTCGATGAAATAGGT
TTTTCTCCTGATGATCTTCAAAATTTTGTTCATTCACTTTCATACGTGTATCAAAGGAGC
ACAACCGCACTTTCAATTGCTGCACCAATATGTTATGCGCATCTTGCTGCAGCACAGATG
AGCCAGTTTATTAAGTTTGAGGAACTGTCTGAAACCTCCTCCGAACGAGGAGGTGTTACT
TCATTGGGAAGTCTATCGATTCCAGAACTTCCACGGCTGCACGACGATGTTAACGGCTCC
ATGTTTTTCTGCTGA
>Cucumis-sativus_Cucsa.200260
ATGGAACCGCTGGTCGAAAAGTTGTTGTTCGTGCCAATCATTTCCTGGTTCAAGTTGCAG
ACAAAGATTtCCACCATTACGATGTTTCAATCACACCTGAGGTTACATCCAAAAaGGgTC
TGCCGAGATATAGTAAACCAGCTTGCAAATACGTATAGAGAATCTCATTTAGGTGGAAGA
TATCTAGCATATGATGGAGGAAAAGgTGTTTATGCTGCTGGTCAACTTCCATTTTCGTCC
AAAGAGTTCATGATTAAATTAGTCCGTAAAGATGGTGCTGGGTCTTCTCAACCAACAAGG
AAAGAACGAGAGTTCAAAGTATCTATCAAATTTGCCTCCAAACCAGACCTCCATCATCTA
CAACAGTTTATACATaGACaGCAGCGAGATGCACCACAGGAGACTATACAAGTTCTTGAT
GTTGTTTTGAGAACAAAACCATCTGTCGATTACACAGTTGTTGGAAGGTCATTTTTCTCG
CATGAACTGGGGCAGCCAGGTGAACTTGGTAATGGTGTTGAGTATTGGAGGGGATATTAC
CAAAGTCTACGACCTGTACAAATGGGTTTATCTTTAAATATAGATGTGTCAGCTAGATCA
TTTTATGAACCAATcAGTGTGACAGAATATGTTGTTAAACACTTCAACCTAAGGAaTCTC
TCAAAGCCAATGTCTGATCAGGATTGTCGCAAGATCAAAAAGGTACTTAGAGGAGTGAAG
GTTGGATTAAcGTGTAGGGAGCATGCCAGGACCTACAAGATTACTGGGATATCATCAGAG
CCTGTTAATAGATTAATGTTTACTCTTGAcGATCAGAAAACCCGAaTCTCTGTTGCACAG
TACTTTCATGAAAAATATGGCGTGGCACTCAAATATCCATTTCTACCAGCTATACAAGCT
GGTAATGATGCCAAGCCGGTTTATTTGCCTATGGAGGTTTGTAAGATTGTTGCTGGGCAG
AGATATACCAAAAAATTGAATGAACGCCAAGTAACTgAGATGCTAAGAGCGACTTGTCAG
AGACCTCCGAACAGGGAaGATAGTATAGGGAAGATGATTGGTAAGATTGATCATTCTAAA
GATGACATTGTCAATGATTTTGGCATTGTTGTATCATCAAGGTTATGTGATGTGGGTGCT
CGTGTTTTACCGTCCCCGATGCTAAAATATCATGATACTGGCgAAGAATCACGAGTTGAT
CCAAGGATGGGTCAGTGGAATATGATTAACAAGAAAATGATCAATGGTGGTAGAGTTGAT
TATTGGGGCTGTGTGAACTTCTCTTCACGGcTGGATCCAGGCTTGCCATCTGAATTTTGC

CATCAGTTGGTTAGCATGTGCAATAGCAAAGGAATGGTTTTCAACCCTACACCGCTGTTT
CCTGTACGTAATGCACATGCAAACCAAATAGATGGTGCACTTGgAGACATTCATAGCCAG
TCTTTAAAAAGTTTGGGACCCCAGGGCAAATCTCTTCAGTTGCTCATAATTATTTTACCT
GATATTTCTGGTTCCTATGGGAAGATCAAAAGGATATGTGAGACTGAGCTTGGAATTGTT
TCACAATGTTGCCAACCTAGGCAAGCACAAAAGCTGAACAAGCAGTACTTTGAAAATGTG
GCCCTTAAAATTAACGTTAAGGTTGGGGGAAGAAACAATGCCcTAAATGATGCTATTCAG
CGGAAAATTCCACTTGTTTCAGATCGGCCTACAATAATCTTTGGAGCAGATGTAACACAC
CCACAACCTGGAGAGGACTCCAGTCCTTCAATAGCAGCTGTTGTTGCCTCAATGGACTGG
CCCGAGGTAACGAAGTATAGAGGAaTTGTTTCAGCTCAGGGCCACCGggATGAAATTATA
CAAGATTTGTACAGAGAAGATAAAGATCCTCAAAAAGGGTTGGTGtGTGCAGGAATGATC
AGGGAGCTGTTCATTGCTTTTAGAAGGTCAACAAATCTGAAACCGCACAGAATAATATTT
TACAGAGATGGTGTAAGTGAAGGACAGTTCTCACAAGTTTTATTTTATGAGGTGGATGCA
ATTAGGAAGGCATGTGCCTCTCTTGAAGAGGGTTACCAGCCTCCGATTACCTTTGTTGTG
GTGCAAAAGAGACATCATACCCGTCTTTTCCCTATCAGCGGTGCGGATACTGATCGGAGT
GGAAACATCCTTCCAGGTACTGTTGTTGACACCAATATTTGCCATCCAACTGAATTTGAT
TTCTATTTAAACAGCcATGCCGGCATTCAGGGGACGAGTAGGCCAACACATTATCACGTT
TTGTATGATGAAAACAAATTCACTGCCGATGCAATGCAGATGCTTACTAATAATCTTTGC
TACACGTATGCAAGGTGCACTCGGTCGgTTTCCATTGTTCCACCAGCATACTATGCACAT
CTTGCAGCCTTTCGTGCCCGTTATTACATAGAGGGTGATTCATCTGACAGCGGTTCCACA
AGTTCAGGTGGTGGGAATGTAGATATTCAACGGCTGCCGAGCATAAAAGAAAATGTGAAG
GATGTGATGTTTTATGTTGAGCGTGTGGGGGGAGAGGATGTGGTTGTGTTTGGTGGGTTT
TGA
>Cucumis-sativus_Cucsa.254700
ATGCCAGTACGGCAAATGAAAGAGAGCTCCGAGCAGCACCTTGTAATCAAAACCCACTTG
CAGAACACTGTTCAAAAAGCTCCAAAATCAACCCAAAATGGCAAAGGTCCGCCGAATCTG
GAACACCAGAACATCAAATTTCGAAACCCCTCTTCCCCTCCGAGTAAAAACAGGGGTCGG
AGAAGGAGCAGAGGTGGTCGGAAATCCGATCAGGGAGATGTGTTTATGAGGCCCAGTTCT
CGGCCCTGCACGGTGGCACGTAAACCCGATGAACCTGAGTTTAACGCCGGAGCTATGGTG
GCGAGTACTAATCCAAATGGTGGAATCATTAGTGGGATGCAAATGGGTTTTCGTAATTCA
AGCAAGTCTTTGAGTTTTGCCCCGAGGCCTGGATTTGGTCAAGTTGGAACAAAGTGTATT
GTAAAAGCAAACCATTTCTTTGCAGAGTTACCAGACAAAGATTTGAATCAATATGATGTT
ACAATTACTCCTGAAGTGGCATCAAGAACTGTGAACAGAGCCATCATGGCTGAATTGGTG
AGATTATACAGAGAATCTGATTTGGGAAAAAGATTACCAGCTTATGATGGCAGAAAGAGT
CTGTATACAGCTGGTGAGCTTCCTTTTGTATGGAAAGAGTTTACCATCAAGCTTGTGGAT
GAAGAAGATGGAGTCAGTGGTCCCAAGAGGGAGAGGGAATATAAAGTACTTATTAAGTTC
GTTGCACGTGCAAATTTACACCATCTGGGTCAGTTTCTGGCGGGCAAGCGTGCTGATGCT
CCACAAGAAGCTCTTCAGATACTGGACATTGTATTGAGAGAGCTTTCATCCAAAAGGTAC
TGTCCTATAGGGAGATCTTTCTTTTCTCCTGATATTAGATCACCTCAGCGGCTCGGTGAT
GGGCTAGAATCATGGTGTGGATTTTATCAGAGTATTAGACCTACTCAAATGGGACTGTCT
CTGAACATAGATATGGCTTCAGCTGCGTTCATTGAGCCTCTCCCTGTCCTTGAGTTTGTT
GCTCAGCTTCTAGGGAAAGATGTTTTGTCACGGCCACTGTCCGACTCCGATCGAGTAAAG
ATTAAAAAGGCTCTGAGAGGGGTGAAAGTTGAAGTAACACACCGTGGAAATGTTAGGCGG
AAGTATCGAGTTTCGGGTCTGACATCACAGCCTACAAGAGAATTAGTATTTCCTGTTGAT
GACAACTCAACCATGAAGTCAGTTGTTGAATACTTCCAGGAGATGTATGGCTTCACCATT
CAGCATGCACATCTCCCTTGCCTTCAAGTAGGAAACCAGAAGAAGGCAAATTATTTGCCA
ATGGAGGCCTGCAAAATTGTGGGGGGGCAAAGATATACAAAGAGATTGAATGAAAAGCAA
ATAACAGCACTTCTAAAAGTCACGTGTCAAAGACCTAGGGATCGAGAAAACGACATTTTG
CAGACTGTCCAACATAACGCTTATGACAATGATCCTTATGCTAAAGAGTTTGGGATTAAA
ATCAGTGAAAAATTGGCTTCTGTTGAAGCTAGAATTCTTCCTCCTCCATGGCTAAAATAT
CATGATACTGGTAAAGAGAAGGATTGTTTGCCTCAAGTGGGTCAATGGAATATGATGAAC
AAGAAAATGATTAATGGAATGACTGTTAACCGGTGGGCATGTATCAACTTCTCTAGGAGC
GTGCAAGAGAGCGTAGCTCGTGGGTTTTGTTCTGAACTTGCTCAGATGTGTCAAGTGTCT
GGAATGGAATTCAATCCAGAACCAGTTATTCCAATATACAATGCAAGGCCAGAACAGGTA
GAGAAAGCTTTGAAGCATGTGTACCATGCTTCCATGAACAAGACTAAAGGAAAAGAGTTG
GAGTTATTATTAGCTATTTTACCTGACAATAACGGATCGCTTTATGGTGATCTTAAGCGA
ATTTGTGAAACTGATCTTGGTTTAATATCACAATGCTGTCTTACAAAGCACGTGTTCAAG
ATAAGCAAGCAATACCTTGCTAATGTGTCACTGAAGATCAATGTCAAGATGGGTGGAAGA
AACACCGTTCTTCTAGATGCTATCAGCTGCAGGATACCACTTGTGAGTGACATACCAACA
ATTATATTTGGAGCAGACGTGACCCATCCAGAGAATGGCGAAGATTCCAGCCCTTCAATA
GCTGCTGTAGTAGCCTCTCAGGATTGGCCTGAAGTGACAAAATATGCAGGGCTAGTATGT
GCTCAAGCTCATAGACAAGAACTTATACAAGACTTGTACAAAACTTGGCAGGATCCTGTC
CGTGGCACTGTCAGTGGTGGCATGATCAGGGATCTTCTGATTTCGTTTAGGAAAGCAACA

GGGCAGAAGCCTCTCAGGATAATATTTTACAGGGACGGCGTGAGCGAAGGACAATTTTAT
CAAGTATTACTTTATGAGTTGGATGCAATCAGGAAGGCATGTGCTTCTTTAGAACCGAAT
TACCAACCACCTGTAACATTCATTGTCGTACAAAAGCGACACCACACCCGATTGTTTGCC
AACAACTATAGAGATAGAAGTAGCACAGACAAGAGTGGGAACATTTTACCTGGAACTGTA
GTCGACTCCAAAATATGCCACCCAACAGAATTTGATTTCTATCTCTGTAGTCATGCTGGA
ATTCAGGGAACGAGTCGGCCAGCTCACTACCATGTTCTTTGGGATGAAAACAATTTCACT
GCAGATGGAATTCAGTCGTTAACAAACAATCTTTGCTACACGTATGCAAGATGTACACGT
TCAGTTTCTGTCGTCCCTCCAGCATACTATGCACATTTAGCTGCATTCCGAGCTCGATTC
TACATGGAACCGGAGATGCAGGaGAATGGCTCAGCTGGTCGTTCGGCCAAGAGTACACGT
GTCACCGGTGAATGTGGGGTTCGTCCTTTGCCAGCTCTAAAAGaAAACGTGAAGAGAGTA
ATGTTTTACTGTTAG
>Cucumis-sativus_Cucsa.284770
ATGGAAGAGTCAGATGAGCCTAAGACTACCAACAAATTCAACAACAAAACTGGGACTTTC
AAAGCCAGGATTAACCCTCATCATCCTCATCCCCATCCCCATCCTCATCTTCTTCATCAT
CAATATCAGCATCATCAGCTATGGCAGTACTCAAATCAGTATGCTTTCTGTAACCAGAAT
CATTTCCAAAGATGCTGTCCTGTCCTTCTTCCCCTTCCCCTTCCACTTCCTCTTCCTTCT
CCTATTCCTCTGCAACTTGCTCTTCCTCCAAGTAAAACCACTAGACCAAAGCCCCATTTG
CAGAAGCCTTCTTGTAAGCTCAGTAACCCTCCTTTAGCTGCCACCTCTGATACCCATGTC
CCAATAATAAAGATTTCTCCAGCTCCAGAAGGGCTACGACTGAAAAGCAGTTTGCCACTT
AAAGGAGAAAATGGAAAAGTGAGTCAAAGTAAACCAGAAGTATTAGTGGCTGCAAGAAGA
CCAGATTCTGGAGGTGTGGAAGGGCCAGTTATTCCTCTTTTAGCTAACCATTTCTTGGTT
CAATTTGACCCTTCACAACGTATTTACCATTACAATGTAGAAATTTCTCCCAATCCTTCT
AAAGAAGTTGCTAGAATGATTAAACAAAAACTTGTCGAAGATAACTCAGATTTGCTCTCT
GGTGCGTCTCCTGCTTATGATGGCCGCAAGAATCTTTACAGCCCAATTGAGTTTGTAAGA
GATAGGCTTGAGTTCTATGTCAGCCTTCCAATTCCTTCCAGCAGGATGAAATTTAGTGAA
GGAGAGATTGAACATAAGCAAGCCTTTAAACTCTTCAGGATAAGTATTAAACTTGTCTCG
AAATTTGATGGGATGGAACTGAATAGATACATGACAAAAGAGGGAGATGATTGGATTCCA
ATTCCTCAGGATTATCTTCATGCCCTAGATGTTGTATTGAGGGAAGGTCCAAATGAGAAA
TGTATACCTGTGGGAAGATCTCTGTATTCCAGTTCAATGTGGGGAGGCAAAGAAATTGGG
GGAGGAGTTGTTGGCTTAAGAGGCTTTTTCCAAAGCCTTAGACCCACACAACAAGGCTTA
GCTATGAATGTAGATTGTTCTGTCACTGCTTTCCATGAAAGCATTGGAGTGATTCCTTAC
TTACAAAAACGTCTTGATTTTCTTAGAGATCTTTCTCAGAGGAAGACAAGAGGTTTAACC
ATTGAAGAAAAGAAGGAAGTGGAGAAGGCTTTAATGCATATCAGAGTATTTGTTTGTCAC
AGAGAGAGTGTTCAAAGATATAGAGTTTATGGCTTGACAGATGAACCTACTGAGAGTCTC
TGGTTTGCTGATAGAAATGGGAAGAATCTAAGATTGGTGGGTTACTTCAAGGATCATTAT
AACTATGACATTCAATTCAGGAATTTGCCTTGTTTGCAGATTAGTAGGAGTAAGCCATGT
TATCTTCCTATGGAGCTTTGTATGATTTGTGAAGGGCAAAAGTTTCTTGGGAAGCTCACT
GATGAACAGACTACAAGAATGCTTAAGATGGGTTGCCAGAGGCCAAAGGAAAGAAAAGCT
AATATTGATGGTGTAATGCAAGGTCCTGTTGGGCCAACCAGTGGGGTCCAGGGAAGAGAA
TTCAGCCTCCAAGTTTCAAAGGaAATGACCAAATTAAGTGGGAGAGTTCTTCAACCACCC
AAGCTAAAGCTTGGCGAAGGTGGCCAGATTCGAGATCTAATTCCCTCCCGCCATGACCGA
CAGTGGAACCTTGTCGATTGCCACGTTTTCGAGGGAACGAGAATTGAGAGATGGGCATTG
ATAAGTTTTGGTGGTGTCCCGGATCAGAGGTCAAACATTCCTAGATTTATAAATCAGCTC
TCAAGAAGATGTGACCAATTAGGCATATTTCTCAACAGTAAAACAGTTGTACCACCTCAA
TTTGAATCAACCCAAGTACTCAACAGTGTCTCACTGtTGGAATCTAAGCTTAAGAAGATT
CATGGAGCTGCATCTAACAACCTTCAGCTCTTGATATGTGTGATGGAGAGAAAACACAAA
GGCTATGCAGATTTGAAACGCATTGCCGAGACCAGTATCGGCGTTGTAAGTCAATGTTGC
CTTTATCCTAATCTTGCCAAGTTGAGCTCTCAATTTCTGGCTAATTTGGCTCTTAAGATT
AATGCCAAGGTTGGTGGTTGCACTGTTGCTTTATATAATTCGTTACATTCACAAGTACCT
CGACTGCTCCGAATCGATAAGCCTGTGATCTTCATGGGTGCAGATGTAACTCATCCTCAT
CCCTTGGATGATTTTAGCCCCTCCATCGCTGCTGTGGTTGGTAGCATGAATTGGCCAGCA
GCAAACAAGTACGTCTCCAGAATGAGATCACAAACACACAGACAAGAAATTATTGTGGAT
CTTGGAACAATGGTCGAAGAGCTGCTTGAGGAATTCTATCAAGAAGTGAATGAATTGCCA
TCACGAATCATATTCTTCAGAGACGGTGTAAGTGAAACCCAGTTCTACAAGGTGCTTCAA
GAGGAATTGCAAGCTATAAAAACAGCCTGTTCTAGGTTTCTCAATTATAAACCTCCTATA
ACTTTTGCTGTTGTTCAGAAGAGACATCACACAAGATTGTTCCCATTCAAAGTCGATCCA
TCTTCTAATCAAACTGAGGTTCTCGATGAAAATATTCCTCCCGGGACAGTCGTTGATACC
GTAATTACGCACCCGAAGGAATTCGATTTCTATCTCTGCAGCCATTGGGGGGTGAAGGGA
ACAAGCAGGCCTACACATTATCATATTCTGTGTGATGAGAATCAATTCACTTCTGATGAA
CTACAAAAGCTAGTTTACAACTTATGCTACACATACAACAGGTGCACAAAGCCTGTTTCA
TTAGTACCTCCAGCTTATTATGCTCATCTTGCTGCTTATAGAGGCAGACTTTACCTTGAA
CGATCCGACTCAACAACTTATACTCGAGGAATATCCACTGTCTCTCGAGCTGCCCCTCCA

AAAACAACGCCTCTACCGAAACTTAAAGAAAATGTTAGGAAACTAATGTTCTACTGTTGA
>Glycine-max_Glyma01g06370
CAAGGAAGCCACATGCAGTACTTGTTGCAAGTAGGCCAGACTCTGGTGGCAGAGAAGGCT
CTGTGATCTCTCTTCTTGCCAACCACTTTTTGGTGCAATTTGATCCATCACAGAAGATAT
ATCATTACAATGTTGAAATCACTCCTCATCCCTCCAAGGATGTTGCCAGAGCAATCAAGC
AGAAGTTGGTAAATAACAATTCTGCAGTCCTCTCAGGTGCTACTCCAGCATATGATGGTA
GAAAGAATCTTTATAGTCCAGTTGAATTCCAAAATGACAAGCTTGAGTTCTACATAAGCC
TCCCAATCCCCACTAGCAAGTTGAATTCACCTTATGGAGAAATGCCTGATTTGAAAGAGA
AGCATGAACAGCTTAAACTTTTCAGGATAAATGTCAAGTTGGTCTCAAAGATCAATGGGA
AGGAGTTGAGTAATTACTTGAGCAACGAGGGTGATGATTGGATTCCACTTCCACAGGATT
ATCTGCATGCTTTGGATGTAGTTCTTAGGGAAAGTCCAACTGAGAAATGCATACCTGTAG
GGAGGTCATTCTATTCAAGTTCAATGGGAAGAAGCAAAGACATTGGTGGAGGAGCTGTTG
GATTGAGAGGCTTCTTTCAGAGTCTTAGACCAACACAACAAGGACTTGCTCTCAATGTGG
ATTTCTCGGTAACTGCTTTCCATGAGAGCATAGGAGTGATTGCATACTTGCAGAAGCGCG
TCGAGTTTCTTCGAGACCTGTCTCAAAGGAAGACAGCTCAATTAACTGGCGAAGAGAGGA
AGGAAGTGGAGAAGGCGTTGAAGAGCATCAGGGTCTTTGTTTGCCACAGAGAAACTGTTC
AGCGATATCGTGTCTATGGCTTGACTGAGGAGGTTACTGAAAATCTTTGGTTTGCTGACA
GAGATGGGAAGAATCTGAGGTTGGTGAATTACTTTAAAGATCAATATAACTATGACATAC
AATTCAGAAAACTGCCATGCTTGCAAATTAGTAGGAGTAAGCCTTGTTATCTCCCTATGG
AGCTTTGTGTGATCTGTGAAGGCCAGAAGTTCCTTGGGAAACTGTCTGATGATCAAACAG
CAAGAATACTCAAAATGGGCTGCCAAAGACCGGCAGAACGAAAAACCATTGTCGAAGGAG
TCATGAGAGGAACTGTTGGGCCTACCAGTGGTGATCAGGAAAAAGAATTCAAACTCCAAG
TATCAAGAGAAATGACAAAGTTGACTGGTAGAATTCTTCACCCTCCCAAACTAAAGCTTG
GAGATGGAGGTCATGTAAGAAATCTGACTCCTTCGCGTCACGACCGCCAATGGAACCTTC
TTGACGGCCATGTCTTTGAAGGAACTACTATTGAAAGGTGGGCACTAATTAGTTTTGGGG
GCACACCTGAGCAGAAGTCCAATGTCCCCAGATTTATAAACCAGTTATGTCAAAGGTGTG
AACAATTGGGCATTTTTCTCAACAAGAACACTGTTATTAGTCCCCAGTTTGAATCTATCC
AAATTCTTAACAATGTCACCCTTTTGGAATCTAAGCTCAAGAGAATCCAGAGGACAGCCT
CAAACAATCTCCAGCTTCTTATTTGCATAATGGAGAGAAAACACAAAGGGTATGCTGACT
TGAAGCGAATTGCCGAGACAAGTGTTGGTGTCATGAGCCAATGCTGCCTGTACCCCAACC
TCAACAAGTTGAGTTCACAATTTTTGGCTAATTTGGTCCTCAAAATCAATGCCAAAGTTG
GTGGATGCACAGTTGCCTTATACAACTCATTGCCTTCGCAGTTACCGCGCCTCTTTCATA
TTGATGAGCCAGTGATATTCATGGGTGCTGATGTGACACATCCTCACCCTCTTGATGATG
TCAGTCCATCTGTTGCTGCTGTTGTTGGTAGCATGAATTGGCCGACAGCAAACAAGTACA
TTTCAAGAATAAGGTCTCAAACACATAGACAAGAAATCATCCAGGATCTCGGTGCAATGG
TGGGGGAATTGCTTGATGATTTTTACCAGGAGGTAGAGAAACTCCCCAATAGAATCATTT
TCTTCAGAGACGGGGTTAGTGAAACTCAGTTTTACAAAGTGCTGGAAGAGGAACTTCAAT
CCATCAGGTTTGCATGTTCAAGGTTTCCTGGCTACAAACCTACCATTACTTTTGCAGTTG
TGCAAAAGAGGCATCACACAAGGTTGTTTCCCTTTGAAACTGACCAGTCTTCAACTCAAA
ACAATTTTCTATATGAAAACATTCCTCCTGGGACTGTGGTTGATTCTGTGATCACTCATC
CAAAGGAATTTGACTTCTATCTTTGTAGCCATTGGGGTGTTAAAGGAACAAGTAGGCCAA
CTCACTACCATGTCTTGTGGGATGAAAACCAGTTTACTTCTGATGAACTACAGAAACTGG
TTTACAACTTATGCTACACTTTTGTTAGGTGTACCAAGCCAATTTCTTTGGTGCCTCCTG
CATATTATGCACACTTAGCTGCATATAGAGGCAGACTCTACCTTGAGAGATCAGAGTCCT
TAGGTTTGTTCCGAAGCACATCTACACTATCCAGAGCTGCTCCTCCAAAGACAGCAGCTC
TACCTAAACTTA
>Glycine-max_Glyma02g00510
ATGCCAGTGAGACAGATGAGGGAGAGCTCAGAACAACACCTTGTGATCAAACCCCATTTG
CAGAACCCCATGAATGGAGCTAAGAAGGTTCCTAGAGCTGTTCAAAATGGCAAAGGTCCA
CCACCACCGCCACTGCCACAACAGGAACTACCCCACAACCAAACTTCACCTCATGTAAGG
AACAAGGGGAGAAGAAGGAGCAGAGGTGGAGGAAGAAAATGTGATCAAGGAGATGTTTTG
ATGAGACCTATTGTGGCAAGTGGTGTTGAAAATGGAAGCACCATGTGTGGTGAAATAGAG
ATGAGTTGCCCCACTTCAAGCAAGTCTTTGAGCTTTGCTCCTAGGCCTGGCTATGGACAA
GTTGGGACAAAGTGCATTGTGAAGGCTAACCACTTCTTTGCAGAGTTACCAGACAAGGAC
TTGAACCAGTATGATGTCAGTATTACCCCCGAAGTGTCTTCCAAAGCAGTGAACAGGTCC
ATTATAGCAGAACTTGTGAGGCTGTATAAAGAGTCTGACCTTGGGATGAGGCTTCCAGCA
TATGATGGCAGAAAAAGTCTGTACACTGCAGGGCCACTTCCCTTTTCCTGGAGAGAGTTC
AAGATAAAGGTTGTGGATGATGAGGATAGAGTTAATGGCCCCAAAAGGGAAAGAGATTAT
AGAGTGGTGATCAAGTTTGTTGCCAGGGCCAATTTGCACCACTTGGGCCAGTTTCTAGCG
GGTAAATGTGCTGAAGCTCCACAAGAGGCACTCCAAATTCTTGACATTGTATTAAGAGAG
CTATCATCTAAGAGGTTTTGCCCAATTGGGAGGTCCTTCTTTTCACCTGATATTAGAACA
CCACAACGGCTTGGAGAGGGTTTGGAATCGTGGTGTGGATTTTACCAAAGCATAAGGCCT

ACTCAGATGGGTCTTTCACTCAATATTGATATGGCTTCTGCTGCGTTCATTGAACCTCTT
CCAGTTGTGGAATATGTTGGCCAGCTATTAGGAAAAGATATTCTGTCGAGGCAATTGTCT
GATGCTGATCGCATTAAAATTAAGAAAGCCCTTAGAGGAGTTAAAGTCGAAGTAACCCAC
CGAGGGAGTGTTAGAAGAAAGTATCGAGTTTCTGGTTTGACTTGTCAACCAACCAGAGAA
CTTGTGTTTCCTGTTGATGAGAATTCAACTATGAAGTCAGTAGTTGAATACTTTCAAGAG
ATGTATGGTTTCACAATTAAATATACTCACCTTCCTTGCCTTCAAGTAGGAAATCAAAAG
AAGGCAAATTATTTACCTATGGAGGCCTGCAAAATTGTTGAGGGACAACGATATACAAAA
AGGTTGAATGAGAAGCAAATCACTGCTCTCCTGAAAGTTACTTGTCAAAGACCTCGTGAT
CGAGAAAATGACATTTTGCAGACCATTCAGCACAATGCTTATGGTCAAGATCCTTATGCA
AAGGAATTTGGGATTAAAATTAGTGAAAAGCTAGCTTCTGTAGAAGCGCGAATTCTCCCT
GCCCCATGGCTTAAATATCATGAAAGTGGGAAAGAAAAGAATTGTTTACCCCAAGTTGGT
CAGTGGAATATGATGAACAAGAAAATGATAAATGGGATGACTGTCAGCCAGTGGGCATGC
ATAAATTTTTCACGAAGTGTACAAGACAGTGTTGCTCGCACTTTTTGTACCGAGCTAGCT
CAAATGTGCCAAGTATCTGGCATGGAATTTAATCCAGAGCCTGTTATCCCCATCTACAAT
GCCAAACCTGAGCATGTGGAGAAAGCTTTGAAGCATGTTTACCATGCGTCAACAAACAAA
ACCAAAGGAAAGGAATTGGAGCTTTTGTTAGCAATATTGCCGGACAACAATGGTTCTCTC
TATGGTGATCTCAAGCGTATTTGTGAAACCGACCTTGGTTTAATTTCTCAATGTTGTTTG
ACAAAGCATGTCTTTAAGATCACAAAGCAGTATTTGGCTAATGTGTCTCTGAAAATCAAT
GTCAAGATGGGAGGTAGAAACACCGTTCTTGTTGATGCTGTAAGCTGTAGAATACCATTG
GTTAGTGACATACCAACTATAATATTTGGAGCAGATGTTACCCATCCTGAAAATGGAGAA
GACTCCAGCCCTTCAATAGCAGCCGTTGTAGCATCTCAAGACTGGCCTGAAGTGACGAAA
TATGCCGGTTTAGTGTGTGCTCAAGCTCATAGACAGGAACTCATACAAGATTTGTACAAA
ATGTGGCACGACCCTGTTCGTGGCTTAGTCAGTGGTGGCATGATCCGAGACTTGTTGATT
TCCTTTAGAAAGGCAACAGGGCAGAAGCCTCTAAGAATTATCTTTTATAGGGATGGTGTA
AGTGAAGGGCAGTTTTACCAAGTTCTACTTTATGAGTTGGATGCAATTCGGAAGGCATGT
GCTTCTTTAGAACCAAACTATCAACCTCCAGTAACTTTCATAGTTGTACAAAAAAGACAC
CATACCCGGTTGTTTGCAAATAACCACAGGGACAGGAACAGTACAGATAAGAGTGGAAAT
ATATTGCCTGGGACTGTTGTTGATTCCAAAATCTGTCATCCAACAGAATTTGATTTTTAT
CTGTGCAGTCATGCTGGCATCCAGGGTACGAGTCGTCCAGCTCATTATCATGTTCTGTGG
GATGAAAACAACTTCACAGCAGATGGAATTCAGTCTTTGACAAACAATCTCTGTTATACA
TATGCCAGGTGTACACGCTCCGTATCTGTTGTTCCTCCAGCGTATTATGCACATTTGGCA
GCTTTTCGAGCGCGGTTCTATATGGAGCCAGACCTGCAGCAGGAGAATGGCTCCTCAGGT
GGTGGCTCCAAGGCAACAAGAGCAGGTGGGGTGTGTGGTGGGGTGAAGCCATTGCCAGCA
TTGAAAGAAAATGTGAAGAGAGTCATGTTTTATTGTTAG
>Glycine-max_Glyma02g12430
ATGTCTGACTTGAAAGAGAAGCATGAACAGCTTAAACTTTTCAGGATAAATATCAAACTG
GTCTCGAAGATCAATGGGAAGGAGTTGAGTAACTATTTGAGCAAAGAGGATGATGATTGG
ATTCCACTTCCACAGGATTATCTGCATGCTTTGGATGTAGTTCTTAGGGAAAGTCCAACC
GAGAAATGCATACCTGTAGGGAGGTCATTCTATTCAAGTTCAATGGGAAGAAGCAAAGAC
ATTGGTGGAGGAGCTGTTGGATTGAGAGGCTTCTTTCAGAGTCTTAGACCAACACAACAA
GGACTTGCTCTCAACGTGGATTTCTCAGTAACTGCTTTCCATGAGAGCATAGGAGTGATT
GCATACTTGCAGAAGCGCCTCGAGTTTCTTCGAGACCTGTCTCAAAGAAAGACAGCTCAA
TTAACCGGTGAAGAGAGGAAGGAAGTGGAGAAGGCATTGAAGAACATCAGGGTCTTCGTT
TGCCATAGAGAAACTGTTCAACGATATCGTGTCTATGGCTTGACTGAGGAGGTTACTGAA
AATCTTTGGTTTGCTGACAGAGATGGGAAGAATCTGAGGTTGGTGAATTACTTTAAAGAT
CAGTATAACTATGACATACAATTCAGAAAACTGCCATGCTTGCAAATTAGTAGGAGTAAG
CCTTGTTATCTCCCTATGGAGCTTTGTGTGATCTGTGAAGGCCAGAAGTTCCTTGGGAAA
CTGTCTGATGATCAAACAGCAAGAATACTCAAAATGGGCTGCCAAAGACCGGGAGAACGA
AAAACCATTGTCGAAGGAGTCATGAGAGGAACTGTTGGGCCTACCAGTGGTGATCAGGAA
AAAGAATTCAAACTCCAAGTATCAAGAGAAATGACAAAGTTGACTGGTAGAATTCTTCAC
CCTCCCAAACTAAAGCTTGGAGATGGAGGTCATGTGAGAAACCTGACTCCTTCACGTCAC
GACCGCCAGTGGAACCTTCTTGATGGACATGTCTTCGAAGGAACTACAATTGAAAGGTGG
GCACTAATTAGTTTTGGGGGCACACCTGACCAGAAGTCCAATGTCCCCAGATTCATAAAC
CAGTTATGTCAAAGGTGTGAACAATTGGGCATTTTTCTTAACAAGAACACAGTTATTAGT
CCCCAGTTTGAATCAATCCAAATTCTTAACAATGTCACCCTTTTGGAATCTAAGCTCAAG
AGGATCCTGAGGACAGCCTCAAACAATCTCCAGCTTCTTATTTGCATAATGGAGAGAAAA
CACAAAGGGTATGCAGACTTGAAACGAATTGCTGAGACAAGTGTTGGTGTTGTGAGCCAA
TGCTGCCTGTACCCCAATCTCAACAAGTTGAGTTCACAATTTTTGGCCAATTTGGCCCTC
AAAATCAACGCCAAAGTTGGTGGATGCACAGTTGCCTTGTACAACTCATTGCCATCGCAG
TTACCGCGCCTCTTTCATATTGACGAGCCGGTGATATTCATGGGTGCAGATGTGACACAT
CCTCACCCTCTAGATGATGTCAGTCCATCTGTCGCTGCTGTCGTCGGCAGCATGAATTGG
CCAACAGCAAACAAGTACATTTCAAGAATAAGGTCTCAAACACATAGACAAGAAATCATC

CTGGACCTAGGCGCAATGGTGGGGGAGTTGCTTGATGATTTTTACCAGGAGGTAGAGAAA
CTCCCTAACAGAATCATTTTCTTCCGAGACGGGGTCAGTGAAACTCAGTTTTACAAAGTG
CTGGAAGAGGAACTTCAATCCATCAGGTGTGCATGCTCAAGGTTTCCTGGCTACAAACCT
ACCATTACTTTTGCAGTTGTGCAAAAGAGGCATCACACAAGGTTGTTTCCCTTTGAAACT
GACCAGTCTTCAACTCAAAAAAACAATTTTCTATATGAAAACATTCCTCCAGGGACTGTG
GTTGATTCTGTGATCACTCATCCAAAGGAGTTTGATTTCTATCTTTGTAGCCATTGGGGT
GTTAAAGGAACAAGTAGGCCAACTCACTACCATGTCTTGTGGGATGAAAACCAGTTTACT
TCTGATGAACTTCAGAAACTGGTTTACAACTTGTGCTACACTTTTGTTAGGTGTACCAAG
CCAATTTCTTTGGTGCCTCCTGCATATTATGCACATTTGGCTGCATATAGAGGCAGACTC
TACCTTGAGAGATCAGAGTCCTTAGGTTTATTCAGAAGCACATCTACACTATCCAGAGCT
GCTCCTCCAAAGACAGCACCTCTACCTAAACTTAGTGAAAACATCAAGAAGCTCATGTTC
TATTGCTAG
>Glycine-max_Glyma02g44260
ATGGCTTCTTCGGGGCCAAATGGAACTGACAACGGGGATTCGTTGCCTCCCCCGCCTCCT
GTTGTCCCATCAGATGTTGTACCTGTCAAAGCTGAGCTGGAGAAGAAGAAGGCTTTGCGG
CTTCCAATAGCCAGGCGTGGCCTTGCTTCAAAAGGGACAAAGCTGCAACTTCTCACCAAT
CACTACAGGGTGAACGTTGCAAATACTGACGGGCATTTTTATCAGTATAGTGTTGCTCTT
TTTTATGATGATGGACGACCTGTGGAGGGTAAGGGTGTAGGGAGGAAGCTTCTGGACAGG
GTGCATGAGACGTATGATTCTGAATTGAATGGGAAGGATTTTGCATATGATGGTGAGAAG
ACTCTGTTTACTCTTGGGTCTCTTGCTCGCAACAAGCTTGAGTTTACGGTCGTTTTGGAG
GATATTATTGCTTCCAGAAACAATGGAAACTGCAGCCCAGATGGCAATGGAGAACTGAAT
GAGAGTGACAAAAAGAGGATGAGGCGCCCTAATAGTTCCAAGGCATTTAAAGTTGAGCTT
AGCTATGCTTCAAAGATTCCATTGCAGGCCATTGCCAATGCTCTACGTGGACAGGAATCC
GAGAATTATCAAGAAGCTATAAGAGTGCTTGATATCATTTTGAGGCAACATGCTGCTAAG
CAAGGCTGTTTGCTTGTGAGGCAATCGTTCTTCCACAATGATCCCAAGAATTTTGCTGAT
GTAGGAGGAGGTGTACTTGGATGCAGGGGTTTCCATTCTAGCTTTAGAACTACACAAAGT
GGACTGTCTTTGAACATAGATGTCTCAACCACGATGATAATTACCCCTGGGCCTGTGGTG
GACTTCCTAATATCCAATCAAAATGTGAGAGACCCCTTTTCACTTGACTGGGCCAAGGCC
AAGAGGACATTAAAAAATCTGAGGATTAAAGCGAGCCCATCTAATCAAGAATTCAAAATA
ACTGGGATTAGTGAATTCCCTTGCAAAGATCAAACGTTTACCTTGAAGAGAAAAGGTGGT
GATGATGTTGCTGAGGAAGAAGTGACAGTATATGATTATTTTGTTAATATCCGCAAGATA
GATCTTCGATATTCTGGGGATCTCCCATGTATTAACGTTGGAAAGCCAAAAAGGCCAACT
TATATCCCTCTTGAGCTTTGCTCTTTGGTATCCCTGCAACGTTATACAAAAGCACTATCC
ACTCTTCAAAGGGCTTCATTGGTGGAGAAGTCCAGGCAGAAGCCACAGGAGAGGATGAGG
GTTTTGACTGATGCACTTAAAAGTAGCAATTATGGTTCCGAACCAATGCTACGTAATTGT
GGGATCTCTATAAGCCCCAACTTTACTGAAGTTGAGGGTCGGGTTTTACAAGCTCCGCGG
TTAAAATTTGGTAATGGGGAAGACTTCAATCCTAGAAATGGGAGGTGGAACTTCAATAAC
AAGAAAATTGTCAAGCCAACTAAAATAGAAAGATGGGCTGTGGTTAACTTCTCTGCACGC
TGTGATACACGAGGGCTTGTGAGGGATCTCATTAAGTGTGGTGGAATGAAAGGAATTGTG
ATAGATCAGCCATTTGATGTGTTTGAAGAAAATGGTCAGTTTAGGCGTGCACCGCCTGTG
GTTCGAGTAGAGAAGATGTTTGAATTGGTTCAGTCTAAACTTCCTGGGGCTCCTCAGTTT
CTTCTTTGTCTGCTTCCTGAGAGGAAAAATTCTGATCTTTATGGTCCATGGAAGAAGAAG
AATCTTGCTGAGTTTGGAATCGTGACTCAGTGTATAGCTCCGACCAGGGTCAATGACCAA
TATTTGACTAATGTTCTGTTGAAAATCAATGCTAAGCTTGGTGGCCTGAACTCAATACTA
GGTGTTGAGCATTCTCCTTCTATTCCTATTGTTTCTAGAGCACCAACCATTATTATTGGC
ATGGATGTGTCTCATGGTTCGCCAGGGCAAACAGATATTCCTTCAATTGCCGCGGTGGTC
AGCTCCCGAGAATGGCCACTAATATCAAAGTATAGGGCTAGCGTCCGTACCCAGTCTCCA
AAGATGGAAATGATTGATAATTTGTTCAAGAAGGTTTCTGACAAGGAGGATGAAGGCATA
ATGAGGGAGCTTCTACTTGATTTCTATACAAGTTCTGGGAATAGAAAGCCCGATAATATA
ATCATATTCAGGGACGGTGTTAGTGAGTCCCAGTTCAATCAAGTTTTGAACATTGAACTT
GATCAAATTATCGAGGCTTGCAAGTTTTTAGATGAAAAGTGGAACCCCAAGTTTTTGGTG
ATTGTTGCTCAAAAGAACCATCATACTAAATTCTTTCAACCTGGAGCTCCTGACAATGTT
CCTCCTGGAACTGTAATTGATAACAAAATTTGCCATCCTCGGAATTATGATTTCTATATG
TGTGCACATGCTGGAATGATTGGTACTAGCAGGCCCACACACTACCATGTTCTGTTAGAC
GAGATTGGCTTTTCACCTGATGATCTACAAGAGCTTGTACATTCATTATCATATGTGTAT
CAGAGGAGCACCACTGCCATTTCTGTCGTTGCTCCAATTTGCTATGCTCATCTGGCTGCT
ACTCAGATGGGTCAGTTTATGAAATTTGAAGACAAATCTGAGACATCTTCGAGTCATGGT
GGTTCTGGTATGCCTGCTCCTCCCGTCCCTCAGTTGCCAAGATTGCAGGAAAATGTTTCG
AGCTCAATGTTCTTTTGTTGA
>Glycine-max_Glyma06g23920
ATGAAGGATCCAGAGGAGCACCAAGTGAAGTCTAGAAGGCCTCTTCAGAAATCTGCAGAA
CGTAGAAGAAGGTGCAGGAGAAGGAGCCAGTGCAAGATAGAGAAATTGCAAGAAGCAGAA

CTTGATTCACACCTTGGTTTCTCTTCTGCCACTTGCAAGAGTCTTGTGTTTCATCAAAGG
CCTGGCTTTGGCCAACTTGGTACTAAGTGTGTCATCAAAGCTAACCACTTCCTTGCAGAT
ATATCAGTTTCTGACTTGAGCCATTACAATGTTATAATAACACCTGAAGTCACTTCTCGT
AAAACAAGCAAGGCTATCATAGCAGAGTTGGTGAGGCTTCACAGGAACACTGAATTAGCC
ACTAGGCTCCCTGTGTATGATGGAGGGAGAAACCTTTACACGGCTGGTTTGCTTCCTTTT
ACATACAAAGAGTTCAATGTAACATTGAGTGAGAATGATGATGTTACTTGTGGTACCAGG
GAAAGGGAGTTTAAGGTGGTAATCAAGTTTGCAACTCATGTTAGCATGCATCAGCTACGT
GAGCTTCTTAGCGGGAAACAAGTGAAGAATCCGCAGGAAGCTATAAGCGTTTTTGACATT
GTGTTAAGGGAGCTTGCGGCTCAGAGTTACGTGTCCATTGGGAGGTTTCTGTATTCTCCT
GATGTAAGAAAACCACAGCAGTTGGGTGGTGGTCTTGAATCATGGAGGGGCTTCTATCAG
AGTATAAGGCCTACTCAGATGGGATTGTCACTTAACATTGATATGTCATCAATGGCATTT
ATCGAACCACTACCTGTGATTGACTTTGTTGCTCAAATTTTGGGAAAAGATGTTCACTCA
AAGCCGTTGTTGGATGCAGATCGTGTCAAGATCAAAAAGGCCCTAAGAGGTGTAAAAGTT
GAAGTTACACATAGAGGAAATTTTCGAAGGAAGTATAGAATATCAGGACTGACTTCACAG
CCTACAAGGGAGCTTATTTTCCCTCTTGATGACCAAATGAACATGAAATCAGTAGTTGAT
TATTTTCAAGAAATGTATGGATTTACAATCAAATATTCTCATCTACCTTGCCTTCAAGTA
GGAAGTCAAAGGAAGGTGAACTATTTGCCAATGGAGGCATGCAAGATAGTTGGGGGCCAG
AGATATACAAAAGGGCTAAATGAAAAGCAGATAACTTCTCTGCTGAAGGTCTCATGTCAA
AGGCCCCGCGAACAAGAGACAGACATTTTACAGACAATTCAACAAAACAATTATGAGAAT
AATCCCTATGCAAAGGAGTTTGGCATCAGCATAGACAGCAAGCTTGCATCAGTTGAGGCT
CGGGTTCTTCCGGCTCCATGGTTGAAATATCATGACACTGGAAGAGAGAAAGAATACTTG
CCACAAGTTGGTCAGTGGAATATGATGAACAAGAAAGTTATAAATGGAAGTACTGTAAGA
TATTGGGCATGTATCAACTTCTCACGAAGTGTACAGGAAAGTGCAGCTCGTGGATTTTGC
CAACAGTTAGTTCAGATGTGCCAAATCTCAGGCATGGAATTCAGTCAGGACCCTGCGATT
CCTATACATTCGGCTAGACCAGATCAGGTAAAGAAGGCTTTGAAGTATGTACATTCTGCT
GTTATAGACAAACTTGATGGGAAAGAGCTAGAGTTGCTGATTGCCCTTCTTCCAGACAAT
AATGGCTCTTTGTATGGTGATCTCAAAAGAATCTGCGAAACAGATCTGGGGTTGATTTCT
CAATGCTGCCTTACAAAACATGTATTCAAGATCAATAGACAGTACCTGGCTAACGTGGCA
CTAAAAATCAATGTCAAGATGGGAGGAAGAAACACAGTACTTTTGGATGCTTTAAGTTGG
AGGATTCCACTGGTTAGTGATATTCCAACAATAATATTTGGAGCTGATGTAACTCATCCA
GAATCAGGAGAGGATTCTTGTCCATCCATTGCTGCTGTTGTAGCCTCCCAGGACTGGCCT
GAAGTAACAAAGTATGCAGGGTTGGTTTGTGCTCAGCCTCATCGAGAAGAACTAATTCAA
GATCTTTTCCGATGTTGGAAAGATCCACAGAGGGGTGTAATGTACGGTGGTATGATCAGG
GAGCTTTTACTATCATTTAAGAAGGCAACTGGACAAAAACCATTGAGAATAATCTTTTAC
AGGGATGGGGTAAGTGAGGGACAGTTCTACCAGGTTTTGCTATATGAGCTTGATGCCATA
CGCAAGGCTTGTGCATCTTTGGAACCTAGTTACCAACCTCCAGTAACATTTGTTATTGTT
CAAAAACGGCATCACACTAGACTCTTCGCAAACAATCATGATGACAGAAATAGCACTGAT
AAGAGTGGGAATATCTTACCTGGTACTGTGGTAGATTCTAAGATCTGTCATCCTTCGGAA
TTCGACTTCTACTTGTGCAGTCATGCAGGAATTCAGGGTACAAGTAGACCAGCACACTAT
CACGTCCTCTGGGACGAGAACAATTTCACTGCAGATGAGATTCAGTCTCTAACCAACAAT
TTGTGCTACACTTATGCAAGATGTACGCGGTCTGTTTCAGTAGTGCCTCCAGCATACTAT
GCTCATCTAGCAGCCTACAGGGCTCGATTCTACATGGAACCTGATGTTGCTGAGATTTCA
AAACTGCGAGGAACAAGATCGAAAGAAGGGCCTGTTCGGGCACTGCCTGCTCTGAAAGAG
AAGGTGAAGAATGTTATGTTTTACTGTTGA
>Glycine-max_Glyma06g47230
GTCTCAAAACGTGTGCCTATGGCGAGGAAAGAGGTTGGGTCCAAGGGAGAGCCCAGACAA
CTATTAGCCAACCATTTTGGAGTTTGCCTAGTCAAGCCCAAGGATGATATTGATGGCTAC
TTCTACCATTATGATGTTGCCATGAGTTATGAAGATGGGAACCCTGTTGAGGCCAAGGGT
GTAGGGAGAAAGGTCTTAAATCAAGTTTGTGAAACATATGTTGAACTGAGAAACATGAGT
TTTGCATATGATGGTGAAAAAAGTCTATTCACTCTTGGTCCTCTAGCAAGTCAGAGGCTT
CAATATCCTGTTGTGTTGGAGGATGTTTCATCAAGGAGGGTTGGAAAGAATGGAAACCCT
GCTGAGAGTCCTAAGGGAGGATATACAAAAAGAATGAGAATTAGGCATCAGTTTCGGCCA
AAAACTATCAACGTGGATATCAAATATGCAGCCAAAATTCCATTGCAAGCAATTGAAGAT
GCATTGCGTGGTCGGGATTCTGAGAAATCTCAGGAAGCAGTGAGGGTTCTAGACATCATA
CTGAGACAGCATTCTGCAAACCAGGGATACCTCCTTGTTCGCCAATCCTTCTTTCATGAC
AATCGAAGAACCTGTACTGATATAGGAGGTGGTGTGCAAGGGTGCCGCGGTTTTCATTCA
AGCTTTCGAGTCACACAAGGAGGTTTATCTCTCAACATGGATGTGACAACCCACAATGATT
GTAAAGCCTGGCCCTGTGGTGGACTTCCTTCTCCAGAATCAGAGTGTTCAGAACCCAAAC
TACATTGATTGGACCAAGGCAAAGAGAATGCTGAAGAACCTAAGAATCAGGGCTAATGGT
GTTGAGTTCAAAATCTCTGGATTGAGTGATAACACTTGCAGGAATCAGAAATTTCTTTTA
AGGCAAAAGGGTACGAATGGTGAAGTGCAGGAAAGGGAAATCACAGTTCATGACTACTTT
ACACGCCAAAAACTGATTGGCCTGAACTATTCTGCTGACATGCCATGCATCAATGTTGGC

AAACCAAAACGCCCGTCTTATTTTCCAATAGAGCTATGTGAAATGGTCTCGTTGCAGCGT
TACACCAAGGCTCTAACAAATTTGCAAAGGGCTCAGCTAGTGGAGAAGACAAGGCAAAAG
CCCCAAGTTAGGAGACAAGCTTTGGAAGATGCCTTAAGAAGCAGCAGATATGACGATGAA
CCTATGCTTCGCTCTTCTGGGATTACCATTGAACCTAACTTTGTTAGACTCGTTGGTCGT
GTATTGGAACCCCCAAAGCTTATAGTTGGAGGAGAGAAGAGCATCATTCCCCGGAATGGG
CGCTGGAATTTCAACAATAAGAAACTATATGAACCTTTGATGATTGGTCGTTGGGCCATT
GTAAACTTCTCAAGTCGGTGTGACACAAGACTCCTGATAGAACTCATTAGGAGGTGTGCT
GCAGCTAAAGGGATGACCATGAGCAATAGTCTCTTTGATAAAGTAATTGAAGAGGATGGT
TGCTTCATACGTGAACCACCAAATGTCAGGGTAGAGAGAATGTATGCAAAGCTGAGGACC
ACTCTGCCGCACGAGAAACCTCACTTTCTGTTGTGTATTCTTCCAGAGAAAAAGAATTCT
GACATTTATGGTCCTTGGAAGAAGAAAAGTCTTGTTGAAGAAGGGATTGTAACACAATGT
ATTGCACCAACAAAGATTAATGATCAATACATTACTAATGTACTTTTGAAAATTAATGCA
AAGTATGGTGGGATGAATTCGTATCTGTCAGTTGAGCTTTGTAATTCCATTCCATTTGTT
TCAGCTGTACCAACCTTGATTCTTGGTATGGACGTTTCTCATGGATCTCCTGGTCGATCA
GATGTGCCCTCTATTGCTGCGGTTGTGAGCTCAAGATGTTGGCCTCAAATTTCACGTTAT
AGAGCTTCAGTTCGCACACAATCATCAAAAGTTGAGATGATTCAATCTCTATTCAAGCCT
GTGGCTAATACTAATAAGGATGAAGGTATCATCAGGGAGGTGCTGTTGGACTTTGAAATT
ACTTCATTCAAAAGAAAGCCTCAACAAATTATAATTTTCAGGGATGGAGTGAGTGAATCA
CAGTTCAACCAGGTGCTCAATATTGAGTTAAGTCAAATCATTGAGGCATGCAAACACCTT
GATGAAAAATGGGATCCAAAGTTCACTTTGATTATTGCCCAGAAGAATCATCATACTAGG
TTCTTTCAGGCCAATGCTAGAGATCAAACTAATGTTCCACCAGGAACCGTTATTGACAAC
ACTGTCTGTCATCCCAAAAACAATGACTTCTACTTGTGTGCTCAAGCTGGGATGATTGGG
ACAACTCGACCTACTCACTACCATGTTTTACACGATGAAATCGGATTCTCAGCTGATGAA
GTGCAAGAATTAGTGCATTCATTGTCTTATACGTATCAGAGGAGCACAACTGCTGTATCA
TTAGTCGCTCCCATTTGCTATGCCCACTTGGCAGCAGCCCAGATGGCACAATTTATGAAG
TTTGATGAACATTCTGAGACTTCTTCAACCCATGGTGGACTCACATCTGCTAGCGCACCT
TTAGTACCTCAATTACCACGTCTTCACAAACAAGTCATCAACTCCATGTTCTTTTGTTAA
>Glycine-max_Glyma09g29720
ATGATTTGGTGTTGCCATTTGTTTCTTTTTCTTGTTATCATGGTCAGAAAGAGAAGAACT
GAACTACCCAGTGGGGGTGAAAGCTCTGAGGCTCAACGCCCTGCTGAAAGGAGTGCACCA
CCCCAACAACAGGCTGCTGCTGCTGCCCCAGGAGGGGCTGGACCCCAAGGAGGCAGAGGT
TGGGGTCCCCAAGGAGGACGAGGAGGCTATGGTGGGGGCCGCAGTCGTGGGATGCCCCAA
CAGCAATATGGTGCCCCTCCTGAATATCAAGGTAGGGGAAGGGGAGGGCCTTCTCAGCAA
GGAGGCCGTGGAGGGTATGGCGGTGGCCGAAGTGGTGGTGGTATGGGCAGTGGCCGTGGC
GTAGGTCCTTCATATGGTGGCCCATCCAGGCCACCGGCACCCGAGCTGCACCAAGCAACC
TCAGTTCAATTCTATCAAACTGGGGTGAGTTCTCAGCCTGCATTATCTGAGGCCAGTTCA
TCACTGCCGCCGCCGGAACCTGTTGATTTGGAACAGTCAATGGCGCAGATGGTGCTTCAT
TCTGAAGCTGCTCCTTCTCCGCCTCCTGCAAGTAAATCATCAATGAGGTTCCCTCTTCGA
CCAGGAAAGGGTAGCTATGGCACCAAATGTGTTGTCAAGGCTAATCATTTCTTTGCCGAG
TTGCCCAACAAAGATCTGCATCAATATGATGTAACAATTACTCCTGAAGTGACATCAAGA
GGAGTGAACCGTGCTGTTATGGAGCAGTTGGTGAGGCTGTATCGGGAATCTCACTTGGGT
AAGAGACTTCCTGCTTACGATGGGCGCAAGAGCCTCTATACTGCTGGACCACTTCCTTTT
ATGTCAAAGGAGTTCAGAATTGTTCTTGCTGATGATGATGAAGGAGCTGGAGGCCAGAGG
AGGGACAGGGAATTCAAGGTTGTGATAAAATTGGCTGCACGGGCAGATCTTCACCATTTA
GGACTCTTTTTACAGGGAAGGCAAACTGATGCTCCTCAAGAGGCTTTGCAGGTCCTTGAC
ATTGTTCTGCGTGAACTCCCTACTACAAGGTATTGTCCTGTAGGAAGATCATTTTATTCA
CCTGATTTGGGTAGAAGACAGCCTTTAGGTGAGGGATTGGAAAGCTGGCGTGGTTTCTAC
CAGAGTATTCGGCCTACACAGATGGGGCTATCCCTGAACATTGATATGTCTTCCACTGCA
TTTATTGAGCCATTGCCGGTAATTGACTTCGTAAATCAACTGCTGAACAGAGATGTATCT
GCCCGGCCATTATCTGATGCTGATCGTGTTAAGATCAAGAAAGCTCTTCGAGGTATCAAA
GTTGAAGTAACACATCGTGGAAACATGAGAAGGAAATATCGTATCTCTGGTCTGACTTCA
CAGGCAACCAGAGAATTGACATTCCCAGTAGATGAAAGGGGAACCATGAAATCTGTTGTG
GAGTACTTCTATGAGACATATGGGTTTGTCATTCAACATACTCAGTGGCCTTGTCTGCAA
GTTGGCAATACACAGAGACCTAACTATTTGCCAATGGAGGTTTGCAAGATAGTGGAAGGT
CAAAGGTACTCAAAAAGGCTTAATGAGAGGCAAATCACCGCTTTGCTGAAAGTTACATGC
CAGCGTCCTGTTGAGAGGGAGCGTGATATCATGCAGACAGTACACCACAATGCATACCAT
GAAGATCCTTATGCCAAAGAATTTGGGATCAAGATCAGTGAGAAGCTTGCTCAAGTTGAA
GCTCGCATCCTTCCTGCTCCATGGCTCAAATATCACGATACGGGCAGAGAAAAGGATTGT
CTTCCTCAAGTTGGGCAATGGAATATGATGAATAAGAAAATGGTTAATGGGGGAACAGTT
AACAACTGGTTCTGCATAAACTTTTCGAGGAATGTTCAAGATAGTGTTGCCCGCGGTTTT
TGCTATGAACTTGCTCAGATGTGTTATATATCTGGAATGGCATTTACACCTGAGCCAGTA
GTTCCCCCAGTCAGTGCTCGCCCTGATCAAGTGGAAAAGGTTCTTAAAACTCGGTATCAC

GATGCCAAGAATAAACTGCAAGGAAAAGAGCTTGATTTACTCATTGTTATCTTGCCGGAT
AATAATGGATCACTATATGGTGACCTCAAACGTATTTGTGAGACAGATCTAGGACTTGTT
TCACAATGTTGCTTAACTAAGCATGTCTTCAAAATGAGCAAGCAGTACCTTGCAAATGTT
GCTTTGAAAATTAATGTCAAAGTTGGAGGGAGAAACACTGTACTGGTTGATGCGCTCTCA
CGACGCATTCCCTTGGTCAGTGACAGACCTACAATTATTTTGGAGCTGATGTGACTCAT
CCACATCCTGGAGAGGATTCAAGTCCATCAATTGCAGCAGTTGTGGCTTCGCAAGACTAT
CCTGAAATTACAAAGTATGCTGGTTTAGTTTGTGCCCAAGCTCATAGGCAGGAACTCATC
CAGGATCTTTTCAAACAATGGCAAGATCCAGTCAGAGGAACAGTGACTGGTGGAATGATC
AAGGAACTTCTTATATCTTTTAGGAGAGCTACAGGACAAAAGCCACAACGCATCATATTT
TATAGGGATGGTGTTAGTGAGGGTCAATTTTATCAGGTTCTACTGTTTGAGCTTGATGCT
ATTCGAAAGGCATGTGCATCCCTGGAACCCAACTATCAGCCTCCTGTGACTTTTGTGGTG
GTTCAAAAGCGTCACCACACAAGGCTCTTTGCCAGCAACCATCACGATAAGAGTTCTTTT
GACAGGAGTGGCAACATATTGCCTGGTACTGTTGTTGACTCCAAAATCTGCCATCCCACC
GAATTTGACTTTTATCTCTGCAGCCATGCTGGAATACAGGGTACAAGCCGTCCTGCTCAC
TACCATGTGTTGTGGGATGAAAACAATTTTACTGCTGATGCCTTGCAAACACTCACCAAT
AATCTTTGCTACACATATGCTCGGTGCACCCGATCTGTTTCAATTGTGCCTCCTGCATAC
TATGCTCACCTTGCTGCATTCCGTGCAAGGTTTTACATGGAACCCGAGACTTCGGATAGT
GGCTCTATGACAAGTGGTGCTGTTGCAGGCCGTGGGATGGGTGGCGGCGGTGGTGGTGGT
GTAGGGCGTAGCACCCGGGCACCTGGTGCTAATGCTGCTGTGAGACCATTGCCTGCACTC
AAAGAGAACGTTAAGAGAGTTATGTTTTATTGTTAA
>Glycine-max_Glyma10g38770
ATGCCAGTGAGGCAGATGAAAGAAAGTTCGGAGCAACACCTTGTGATCAAACCACATTTG
CAGAATCCTATGAATCAAGCCAAGAAGACAACCAAAGCTGCACAAAATGGCAAAGGTCCA
CCGCCACAAGAAAACCATAACCAAACTTCGCCACACTCAAAGAACAAGGGAAGGAGAAGA
GGGAGAGGTGGCAGAAAACCTGATCAAGGAGATGTTATGATGAGGCCTAGTTGCAGGCCA
TGCACTGCAACACTAACAAGTACTGCAAATGAGAATGCTGAAAATGGTTGCATCTCTGAT
ATGGGTTTTCCCACTTCAAGCAAGTCTTTGACCTTTGCTCCTAGGCCTGGATATGGACAA
GTTGGGACAAAATGCATTGTGAAGGCTAACCACTTCTTTGCAGAGTTACCAGACAAGGAC
TTGAACCAATATGATGTTACTATTACCCCAGAAGTGTCTTCTAGAACAGTAAACAGGTCT
ATCATAGCAGAACTAGTGAGGCTGTATAAAGAGTCTGACTTGGGGATGAGACTTCCAGCA
TATGATGGCAGAAAAAGTTTGTACACTGCAGGGCAGCTTCCCTTTGCTTGGAGAGAGTTT
AAGATTAAGCTTATAGATGAAGAGGATGGAGTTAATGGCCCTAAGGAAAGAGAGTACAGG
GTGGTGATCAAGTTCGTTGCTCGGGCTAACTTGTATCACTTGGGACAGTTTCTAGCTGGT
AGGCGTGCTGATGCACCGCAAGAGGCACTTCAAATTCTTGACATTGTATTAAGAGAGCTG
TCAACTAAGAGGTATTGCCCTATTGGGAGGTCCTTCTTTTCACCTGATATTAGAACACCG
CAACGGCTTGGAGAGGGATTAGAATCATGGTGTGGATTTTACCAGAGTATAAGGCCTACA
CAAATGGGCCTTTCCCTTAATATTGATATGGCGTCTGCTGCGTTTATTGAGCCTCTTCCA
GTAGTGGAATTTGTTGGCCAGCTATTAGCAAAAGATGTGCTGTCAAGGCCATTGTCAGAT
GCTGATCGCATTAAGATTAAGAAAGCCCTTAGAGGAGTTAAAGTTGAAGTAACACACAGA
GGAAGTGTGAGAAGAAAATATCGTGTTTCTGGATTGACTTCTCAACCAACCAGAGAACTT
GTGTTTCCTGTTGATGAGAACTCAACTATGAAATCAGTAGTTGAATACTTCCAAGAGATG
TATGGTTTCACTATTCAATATACTCACCTTCCTTGCCTTCAAGTAGGAAACCAAAAGAAG
GCTAACTATTTACCTATGGAGGCCTGCAAAATTGTTGAGGGGCAACGTTATACAAAAAGA
TTGAATGAGAAGCAAATTACAGCTCTGTTGAAAGTTACTTGCCAGAGACCTCGCGATCGG
GAAAATGACATTTTACGGACCGTTCAACATAATGCTTATGATCAAGATCCTTATGCAAAG
GAATTTGGAATTAAAATCAGTGAAAAGCTAGCTTCTGTTGAAGCACGAATTCTTCCGGCC
CCTTGGCTTAAATATCACGAAAGTGGGAAAGAGAAGAACTGTTTACCCCAAGTTGGTCAG
TGGAATATGATGAACAAGAAAATGATTAATGGAATGACTGTTAGCCGGTGGGCATGCATA
AATTTTTCAAGGAGCGTGCAAGATAGTGTTGCTCGCACTTTTTGTAATGAACTTGCTCAA
ATGTGTCAAGTATCTGGCATGGAATTTAATCCAGAGTCTGTTATTCCCATCTACAATGCC
AAACCTGAACAGGTGGAAAAAGCTTTGAAACATGTTTACCATGTGTCAGGGAGCAAAATT
AAAGGAAAGGAATTGGAGCTTTTGTTAGCAATATTGCCAGACAATAACGGGTCTCTCTAT
GGTGATCTCAAGCGAATTTGTGAAACTGACCTTGGTTTAATTTCACAATGCTGTCTGACA
AAGCATGTCTTCAAAATCACTAAACAGTACTTGGCTAATGTGTCTCTGAAGATCAATGTG
AAGATGGGAGGTAGAAACACTGTACTTCTTGATGCTGTAAGCAGCAGAATACCATTGGTT
AGTGACATGCCAACCATAATTTTCGGAGCAGATGTAACCCACCCTGAAAATGGAGAAGAA
TTGAGCCCTTCAATAGCAGCTGTAGTCGCATCCCAGGACTGGCCCGAAGTGACAAAATAT
GCCGGTTTAGTATGTGCTCAAGCTCATAGGCAGGAACTTATACAAGATTTGTACAAAACT
TGGCAAGACCCTGTTCGTGGCACAGTTAGTGGTGGCATGATCCGAGATTTACTGGTTTCC
TTCAGAAAGGCAACAGGACAAAAGCCACTACGAATTATATTTTACAGGGATGGTGTAAGT
GAAGGACAATTTTACCAAGTTTTACTTTATGAGTTAGATGCAATTCGGAAGGCATGTGCT
TCCTTAGAACCAAACTACCAGCCTCCAGTAACTTTCATAGTTGTGCAAAAAAGACATCAT

ACCCGGTTATTTGCAAACAACTACAGGGACAGAAGCAGTACAGATCGGAGTGGGAATATA
TTGCCTGGGACTGTTGTTGATACCAAAATCTGCCATCCAACAGAATTTGATTTTTATCTC
TGCAGCCATGCTGGCATCCAGGGTACTAGTCGGCCAGCTCATTATCATGTCCTGTGGGAT
GAAAACAACTTCACACCTGATGGAATTCAGTCTCTGACAAACAACCTTTGTTATACATAT
GCCAGGTGTACACGCTCAGTATCAGTTGTTCCTCCAGCATATTATGCACATTTAGCAGCG
TTTCGAGCACGTTTCTATATGGAACCAGATATGCAAGACAATGGCTCTGCAGGTGACGGT
AATGGTCATGGTGCCAAAGCAACACGAGCAGCTGGTGATTATAGTGTCAAGCCATTGCCA
GACTTGAAAGAAAATGTGAAGAGAGTCATGTTTTACTGTTAG
>Glycine-max_Glyma12g08860
ATGTCTCCTCGCGGTGGCTCCAAGCAGCAACCAGATTCACGCCGTCAAGCGCCACCGTCC
GCGGCGGCGGCGGTGCCTTCTCCTCCCGGAAGAGGACGCGGCCGTGGACGCGGAGAATCC
TCCGTTCCCGCGCCTTCTCTGCAACTTACAATGCAACCTTCGGCGCCGTCGTCGTCGAAG
GCGGTGAGGTTCAAGGAACGGCCAGGGTTCGGTCTTGCTGGGGAGAAAATCAAAGTTCGA
GCGAATCATTTTCAAGTCCAAGTCGCCGAACAAGATCTATTTCATTATGACGTCTCTATC
AATCCTGAGATTACTTCGAAGAAGGTTTCTAGAGATGTTATGACTTTGCTTGTGCAAGCG
CATCGTGAAAAGATTCTCGGAAATCGCATACCAGCCTACGATGGGGGAAAGAGTCTTTTC
ACCGCTGGATCCTTGCCCTTCGAATCTAAGGATTTTGTGATCGTGCTAAAAGACGACGAT
GAACCAGGCTCGTCGTCTTCTTCCTCTCCTACTAGGAAAAAACGTGAACGCGAGTATAGA
GTCACCATCAGGCTTGCTTCCAGAACTGACATTCACCACCTCAGTCAGTTTCTCAGGCGC
CGTCAGTTGGATTGTCCTTATGAGACTATCCAGGCTCTTGATGTTGTTCTGCGTGCTACA
CCGTCTGAAAGGTTCGTTGTTGTGGGAAGATCGTTCTTCTCACCTTCTTTGGGGAAACCT
GGATCGCTTGGTAGCGGAACGGAGTACTGGAGGGGCTATTACCAGAGCCTTCGCCCAACT
CAGATGGGCCTGTCTCTTAACATTAATGTGTCGGCGAGGGCTTTTTATGAGCCTATTCCT
GTGATTGATTTCATTGAAAGTCATTTTAGGGCCAATCCTTCCAGGCCTTTGCCTGATCAG
GATCGAATCAAGCTTAAGAGAGTACTGAGAGGAGTGAAGGTAGAAGTGACTCATGGAAAG
AATCTTAGACGTTACAAGATCACTGGAGTCACAAAAGAACAACTCAGAAAGTTAATGTTT
ACTCTTGATGACAATAGAACAAAAAGCTCAGTTGTTCAATATTTTCATGAGAAATACAAT
ATTGTGTTGAAGCATACGCTTCTTCCTGCTCTTCAAGCTGGTAGTGACATTAAACCAATT
TTTCTGCCTATGGAGCTTTGTCAAATTGTGGCTGGACAAAGATATACAAAGAGATTGAAT
GAGGAGCAAGTAACTAATCTTTTAAGGGCATCTTGTCAGCGTCCTCGTGATAGAGAAAAC
TCTATCAGACAGGTGGTGAGGCAAAGTAATTTCAGCACAGACAAATTTGTGAGTCACTTT
GGAATTCAAGTGAGGGAGGATCCAGCATTGCTTGATGCTCGAGTTCTTCCTGCACCAATG
CTAAAATATCATGACACAGGTAGAGAATCAAGTGTTGAACCCAAAATGGGCCAATGGAAT
ATGATTGATAAGAAAATGTTTAATGCTGGTGTTGTGGAACATTGGACTTGCCTCAACTTT
TCTGGAAAAATAAACAGAGAGTTTCCAAGTGCATTTTGTCATAAGTTGGCCAGAATGTGT
AGTAACAAGGGCATGCGTTTTAATTCAAAGCCTTTACTGCCCATAACATCTGCTCAAAGT
AGTCAAATAGAGAGTGCTCTTGTAAATTTGCATAAGCAGTCTATTACAAGACTAGCAAAC
CAAGGAAGACTCCAATTGTTGATCATAATTTTGCCAGATTTCGAGGGGTCCTATGAAAAA
ATAAAGCGTATTTGTGAAACTGAGCTAGGAATAGTGTCTCAGTGTTGTCAGCCGAGGCAT
GTTTGCCAGATGAAGCCACAATATCTTGAAAATGTGGCCCTCAAGATAAATGTGAAGGTT
GGTGGCAGTAACACAGTATTGAATGATGCAATTGCTAGAATAATTCCTCGTGTGTCTGAC
AGACCTACATTAATCTTGGGTGCGGATGTAACACATCCACAGCCAGGGGAAGATTCTAGT
CCTTCTATTGCTGCAGTAGTTGCATCTATGGATTGGCCTTATGTAACAAGGTACAGAGGA
GTTGTTTCTGCTCAGACTCACCGTGAAGAAATCATCCAAGATCTTTATAATACATGTGAA
GATCCTGTGAAGGGGAAGGTGCATTCGGGAATTATCAGGGAGTTACTTCGCGCTTTCCGT
TTGTCTACTAATCAGAAGCCAGAGAGGATTATATTCTACAGGGATGGAGTAAGTGAGGGC
CAATTCAGCCAGGTTTTGCTGTACGAGATGGATGCAATACGGCGGGCTTGTGCTTCACTA
CAAGAAGGCTATTTACCCCGTGTTACTTTTGTGGTGGTCCAAAAACGACACCACACAAGG
TTATTTCCTGTAGATCATGGAAGTCATGATCAGACAAATAAAAGTGGAAATATAATGCCA
GGGACTGTCGTAGACACACACATATGCCACCCTCGGGAGTTTGATTTTTACCTCAACAGT
CATGCTGGAATGCAAGGAACTAGTCGACCAACACATTATCATGTGCTGTTCGATGAAAAC
AACTTCACTGCTGACGGGTTGCAAATGTTTACTAATAATTTGTGTTATACGTATGCAAGG
TGTACTCGATCAGTCTCAATAGTTCCACCTGTGTATTATGCACATTTGGCTGCCTTCAGG
GCTCGCTGTTACATTGAAGTTGCAACATCAGATTCTGGTTCTGCAAGTGGAGGCCGGGCT
GCTAACTGTGAGGTTAGATTGCCTTCGGTTAAGGAAAATGTGAAAGATGTGATGTTTTTC
TGTTAA
>Glycine-max_Glyma13g26240
ATGACGGAGGAAGTTCCTGGGATTGTAGAAGCTGCTGAACCTCCAACATCATCACAATCA
CCTGCTGATGTGCCACATAATTTGGAAACAGAACAAATGACTCCTACAAAGTATTCTATT
ATCAGTAGGAATGGGGTTGGGACCACAGGAAAACACATACCTTTACTTGTGAATCTCTTT
GAAGTTGCTGTTAATGTTCCAGACACAGTATTTTTTCAGTATAGTGTTGCTATTACTTTC
GAAGATAAACAAGCTGTTGAAAGCAAGGGCATTGGGAGAAAAGTGATTGATAGGCTTTAC

CAAACATACTCTTCTGAACTTGGTGGCAAAAGGTTTGTATATGATGGAGGGAAAACTTTG
TACACAGTGGGTCCTCTGCCACTAAATAAGTACGAGTTCAAAGTGTTGCTGGAGAAATCA
TTTACAAAACGGTATTTCTTGTGTATGACTGTCTTATTTATCAGTAGTGCCAAGAGTCCT
GGTGCTAATGGAAGCCTTCATGAGGAAACTAAAAGGTCAAAACATTCATTTCAGTCAAAG
ACATTCATGGTGGAGATAAGTTTTGCTACCAAAATACCACTTCAGTCCATTGTTATTTCC
TTGAAAGAGGTTGAGTCGGATACCAATTCTCAAGATGCTTTGAGAGTGCTTGATACTATA
CTGAGGCAGCGTGCAGCTAACTGTGGGTGTCTCTTGGTAAGGCAATCTTTTTTTCATGAT
GACTCAAGAAATTTCAATGATGTTGGGGCTGGTGTAACAGCAGTCTCAGGTTTTCATTCC
AGTTTTCGTTCCACACAGCGAGGATTGTCTCTCAATATTGACGTGTCAACAACTATTATC
ATAAAACCTGGACCTGTAATTGATTTTCTGTTATCAAATCAGCAAGTGAAGGAACCCCGT
TATATTGACTGGGAAAAGGCGAAAAAAATGCTTAAAAATTTAAGGGTGCAAGCAACTCAT
CATAACCAGGAATTTAAAATTTCTGGGTTGAGTGAGAAACCATGCATTCAGCAACTTTTT
AGTATGAAGGTTAAAAATGATGACAACAATAGCAGAGGGCAGACAGTGGATATTACTGTT
TATGAATATTTTGCCAAACACTGTGGCATAGAGTTGACCTCATCTGCTTATCTTCCATGT
CTTGATGTTGGGAAACCAAAACGTCCCGTCTATCTTCCCTTGGAGCTATGTTCACTTGTT
TCCCTCCAACGGTATACAAAGGTTTTATCTCTGATGCAAAGAGCATCTTTAGTTGAAAAA
TCACGCCAAAAGCCTCAAGATAGAATCAAAATTTTAAAAAGTGCTGTAGGAAAATGCTAT
GATGATGATCCTGTTCTTGCTGCATGTGGCATTTCTATTGAGAAACAACTGAATCTGATT
GAAGGTCGTGTTCTTGAAACACCAAAGTTGAAGGTTGGTAAGAATGATGATTGCATCCCC
CATAACGGAAGGTGGAATTTTAATAAGAAGACACTTCTACAAGCATCACATATTGATTAT
TGGGCTGTTGTCAACTTTTCTGCAAGTTGTGATACTAGTTACATATCAAGGGAGCTGATC
AGATGTGGAATGAGCAAGGGCATTAACATTGAACGGCCATATACTTTGATAGAGGAGGAA
CCACAGTTGAGAAAATCTCACCCTGTTGCAAGGGTTGAAAGAATGTTTGACCTGCTTGCA
TCAAAACTGAATAGAGAGCCAAAGTTGATTCTTTGTGTCTTGCCAGAGAGGAAAATTTGT
GATATATATGGGCCTTGGAAAAAGAAGTGTCTGAGTGAAATTGGTGTTGTCACACAGTGC
ATTGCCCCTGTCAAGATCACTAATCAATACCTTACTAATGTTCTTCTTAAAATCAATTCT
AAGCTTGGAGGAATAAATTCTCTGTTGGCAATAGAGCATTCTGGGCATCTTCCCCTGATT
AAAGATACCCCAACGATGATTTTGGGGATGGATGTCTCTCACAATTCACTTGGTCGATTA
GATAGTCCATCAATAGCAGCTGTTGTTGGATCTCGACATTGGCCACTGATTTCAAGGTAT
AGAGCGTCTGTGAGAATGCAGGCATCCAAGGTGGAGATGATTGATGCTCTATACAAGCCA
TTGGAAAATGGGAGTGATGATGGTATTATCAGAGAATTGCTTTTAGATTTCTATGATTCT
AGTAATGGACGCAAGCCAACTCAATTTATTGTATTCAGGGATGGAGTTAGTGAATCACAA
TTCGAACAAGTTTTAACTATCGAGCTTAACCAGATAATCAAGGCCTATCAACATCTTGGT
GAGGTTAATGTTCCCCAGTTCACTGTGATTGTGGCACAAAAAAAGCATCATATTAAGCTG
TTTCTGCCTAATGGCCCAGAAAATGTTCCTCCTGGGACGGTTGTGGATACAACAATTACG
CATCCAAGAAATTATGATTTTTACATGTGTGCTCATGCAGGGATGCTTGGAACATCCAGG
CCTGTACATTATCATGTGCTACTTGATGAGATTGGTTTCTCGGCAGATGGCTTGCAAAAT
TTGATCCATTCGTTGTCCTATGTGAACCAAAGGAGCACAATTGCAACCTCAGTTGTGGCA
CCCATATGCTATGCTCACCATGCTGCAGCTCAGATGGGACAACTTTTGAATTTTGATGAT
TCATCAGAAACAGGTTCAAGTCCTGCTTCAGAGGGAGGCATCCCCATCCCAGAGCTTCCA
AGGTTGCACAGGAATGTCAGGAGTTCCATGTTCTTCTGTTGA
>Glycine-max_Glyma14g04510
ATGGCTTCTTTCGGGCCAAATGGAACTGAAAATGAGGATTCGTTGCCTCCCCCACCTCCC
GTTGTACCATCAGATGTTGTACCTGTTAAAGCTGAGCCGGAGAAGAAAAAGGCTTCAAGG
TTTCCAATAGCCAGGCGTGGCCTTGCTTCAAAAGGGACGAAGCTGCAACTTCTCACCAAT
CACTACAGAGTGAACGTTGCAAACACTGATGGGCATTTTTATCAGTATAGTGTTGCTCTT
TTTTATGATGATGGACGACCTGTGGAGGGTAAGGGTGTAGGGAGGAAGCTTCTGGACAGG
GTGCATGAAACGTATGATTCTGAATTGAATGGGAAAGATTTTGCATATGATGGTGAGAAG
ACTCTGTTTACTCTCGGGTCTCTTGCTCGCAACAAGCTTGAGTTTACGGTTGTTCTGGAG
GATGTTATTGCTACCAGAAACAATGGAAACTGCAGCCCAGAGGGCAATGGAGAACTGAAT
GAGAGTGACAAAAAGAGGATGAGGCGCCCTAATAGATCCAAGGCATTTAAAGTGGAACTT
AGCTATGCTTCAAAGATTCCATTGCAGGCCATTGCCAATGCTCTACGTGGACAGGAATCT
GAGAATTATCAAGAAGCAATAAGAGTGCTTGATATCATTTTGAGGCAACATGCTGCTAAG
CAAGGGTGTTTGCTTGTGCGGCAATCGTTCTTTCACAACAATCCCAAGAATTTTGCTGAT
GTAGGAGGAGGTGTACTTGGTTGCAGGGGCTTCCACTCTAGTTTTAGAACTACTCAAAGT
GGACTGTCTTTGAACATAGATGTCTCAACCACCATGATAATTACCCCTGGGCCTGTGGTT
GACTTCCTAATATCCAATCAAAATGTGAGAGACCCCTTTTCACTTGATTGGGCAAAGGCC
AAGAGGACATTAAAAAACCTGAGGATTAAATCGAGCCCATCTAATCAAGAATTCAAAATC
ACTGGGCTTAGTGAACTCCCTTGCAAAGATCAAATGTTTACATTGAAGAAAAAAGGTGGT
GATGATGACACTGAGGAAGAAGTGACAGTATATGATTATTTTGTTAATATCCGCAAGATA
GATCTTCGGTATTCTGGGGATCTCCCATGTATCAACGTTGGGAAACCAAAAAGGCCAACT
TACATTCCTCTTGAGCTGTGTTCTTTGGTATCACTTCAACGTTATACAAAAGCACTATCC

ACACTTCAAAGGTCTTCATTGGTGGAGAAGTCCAGGCAGAAGCCACAAGAGAGGATGAGG
GTCTTGTCTGATGCACTTAAAAGTAGCAATTATGGTTCTGAACCAATGCTACGCAATTGT
GGAATCTCTATAAGCCCCAACTTTACTGAAGTTGAGGGTCGGGTTTTACAAGCTCCACGG
TTGAAATTTGGTAATGGGGAGGACTTCAATCCTAGAAATGGGAGGTGGAACTTCAATAAC
AAGAAAATTGTCAAGCCAACTAAAATAGAAAGATGGGCTGTGGTTAACTTCTCTGCACGC
TGTGATATACGAGGGCTTGTGAGGGATCTCATTAAGTGTGGTGGAATGAAAGGCATTGTG
ATAGATCAGCCGTTTGATGTGTTTGAAGAAAATGGTCAGTTTAGGCGTGCACCGCCAGTG
GTTCGAGTTGAGAAGATGTTTGAGTTGGTCCAGTCAAAACTTCCTGGGGCTCCTCAGTTT
CTTCTTTGTCTGCTTCCTGAGAGGAAAAATTCTGATCTTTATGGTCCATGGAAGAAGAAG
AATCTTGCTGAGTTTGGAATTGTGACTCAGTGTATAGCTCCGACAAGGGTCAATGACCAA
TATTTGACTAATGTTCTGTTGAAAATCAATGCTAAGCTTGGTGGTCTGAACTCAATGTTA
GGCGTTGAGCATTCTCCTTCTATTCCTATTGTTTCTAGAGCGCCAACCATCATTATTGGC
ATGGATGTGTCTCATGGTTCGCCAGGGCAAACAGATATTCCTTCAATTGCTGCGGTGGTC
AGCTCTAGAGAATGGCCTCTAATATCAAAGTATAGGGCCAGTGTCCGTACGCAGTCTCCA
AAGATGGAAATGATTGATAATTTGTTCAAGAAAGTTTCCGACAAGGAGGATGAAGGCATA
ATGAGGGAGCTTCTACTTGATTTCTATACAAGTTCTGGAAATAGAAAGCCCGATAATATA
ATCATATTCAGGGATGGTGTTAGTGAGTCCCAGTTCAATCAAGTTTTGAACATTGAACTT
GATCAAATTATCGAGGCTTGCAAGTTTTTAGATGAAAAGTGGAATCCCAAGTTTTTGGTG
ATTGTTGCTCAAAAGAACCATCATACTAAATTCTTTCAACCTGGAGCTCCTGACAATGTT
CCTCCTGGAACGGTAATTGATAACAAAATTTGTCATCCTCGGAATTATGATTTCTACATG
TGTGCACATGCTGGAATGATTGGTACTAGCAGGCCTACACACTACCATGTTCTCTTAGAC
GAGATTGGCTTTTCACCTGATGATCTACAGGAGCTTGTGCATTCATTATCATATGTGTAT
CAGAGGAGCACAACTGCCATTTCTGTCGTTGCTCCAATATGCTATGCTCATCTGGCTGCA
ACTCAGATGGGTCAATTTATGAAATTTGAGGACAAATCTGAGACATCTTCAAGTCATGGT
GGTTCTGGTATACCTGCTCCTCCCGTCCCTCAGTTGCCAAGATTGCAGGACAAAGTTTCG
AGCTCAATGTTCTTTTGTTGA
>Glycine-max_Glyma15g13260
ATGGCGTCGAGTTCCGGCGGCGGCGGCGGGGTTGAAAGACGCGGAGACCCTAACGACCTT
TGGCGACGACCTGCTCCTCCGGCTACAGATAAGAAAACAAGAAAGGCAAAAGAATTGAAT
TTTTTCTGTATGTTAAAATCAACTTACGCATTCCATATTTTGGAAACGTTAAATGAGAGT
ATAAAAGATGTTTTGGAAATGTTAAATGAGAGTATAAAAAATATCGAAACAGTTACCTGCC
TCAACTTGTACCCTTGAAAAAAAGGATAAAATTTCACCTATTCAGAGACCTGACAATGGT
GGCACGTTAGCAATTCTAACAAGCAGACTTCGTGTCAATCATTTCCCAGTTAAGTTTGAT
CCAGAGAGTATAATAATGCATTATAGTGTTGGTGTCAAACCTAAGGTTTCTTCAAAATTT
GGCCAGCCTCAGAAATTATCAAATTCTGATCTGTCCATGATCAGAGAGAAACTGTTTTCT
GATGATCCTGAGAGGTTGCCCTTGGAGATGACTGCACATGATGGTGCAAAAAATATTTAT
AGTGCTGTACAATTACCAGAAGAGACCTTTACTGTGGAGATCTCTGAAGGAGAGAATGAA
AAGGCCATTTCATATAGTGTTACCTTAACTCTTGTTAATAAACTCAGGCTTTGCAAGTTA
ATGGACTATCTTAGTGGGCATAACCTCTCCATTCCTAGGGACATTTTACAAGGGATGGAT
GTGGTGGTGAAAGAGAATCCAGCTAGACGTGCTGTTTCTGTAGGACGACACTTCTATCCC
ACAAATCCTCCTGTCATAATGAAGGATCTTCACCACGGGATAATTGCTATTGGAGGGTTT
CAGCATAGTTTGAAGCCTACATCTCAGGGTCTGTCCTTATGTGTAGACTACTCAGTATTG
GCTTTTCGAAAGCAAATGTCAGTCTTGGATTTCTTGCATGAGCGTATTGATAACTTTAAA
TTGGATGAATTCGAAAAATTCAGAAAATTCATTGAGGAGGCACTTATTGGATTGAAAGTT
AATGTGACTCATCGTAAATGCAATCGGAAATACATTATTTCAAGATTAACACCTATGATT
ACTAGGTATGTCACTTTTCCCATTGACAATACTGGTGGATGGAACTCAAATGATGTTAGT
CTTATTACCTTTTTTAAAGAAAAATATGGCAAGGATATCGTGTACAAAGATATTCCTTGT
CTAGATTTAGGGAAAGACAGGAAGAAGAACTATGTACCCATGGAATTCTGTGTTTTGGTT
GAGGGCCAAAGATATCCCAAAGAGCGTCTGGATGGTATTTCTGCGAATACATTGAAAGCA
ATGTCACTAGCTCACCCAAATGAGAGGGAGTGTGCAATACAAAAGATGGTGCAATCTAGT
GATGGACCTTGCAGTGATCTTATTCAAAATTTTGGAATAAGTGTCAACACAACCATGACA
ACTATTGTAGGACGTGTACTTGGTCCTCCAGAGTTGAAGTTAGGTGATCCAAATGGAAAG
ATAATCAAATTAACAGTGGATATGGAGAAATGTCACTGGAATCTTGCTGGAAAATCAATG
GTGGAAGGTAAACCAGTTGAGTATTGGGGTGTTCTTGATTTCACTAGTTGTGGGCCATAT
AAGTACAAATTAAGAGGCAAGGAATTCATTCAAAAGCTTATTGGTAAATACAAGAAATTG
GGTATATACATGCAGGAGCCCATTTGGTATGAAGAATCTTCAATGAAGATACTTGCGAGT
TATGATTTACTATCCGAATTACTTGAAAAAATTAACTACATTTGTAAATATAACCAAGTT
CACCCGCAATTTCTTCTCTGTGTAATGGCTAAAAAAAGTCCCGGTTATAAGTACCTAAAG
TGGATTTCTGAGACCAAACTTGGTATACTGACACAATGCTGCTTGTCTAATAGTGCTAAT
GAAGGGGAGGACAAATTTTATACTAATCTGGCTCTCAAGATCAATGCCAAGCTTGGCGGC
AGTAACGTGGAGCTCAGTAATGGACTACCTTACTTTGAGGATGAAGGAGATGTTATGTTT
TTAGGGGCTGATGTCAACCATCCTGGTTATCAAGACACCAGGAGTCCATCAATTGCAGCT

GTGGTTGCTACTGTTAATTGGCCTGCTGCAAATCGTTATGCAGCACGTGTTTTCCCACAA
TACAATCGAAGTGAGAAAATATTGAACTTTGGGGATGTTTGTCTTGAGCTTGTTGCATGC
TATAGGAGGATGAATGGAGTTAGGCCAGAAAGAATTGTTATTTTTCGTGATGGGGTGAGC
GAATACCAGTTTGACATGGTTCTTAATGAAGAGCTACTAGATTTGAAGGGAGTATTTCAA
AGAGTAAATTACTTTCCAACAATCACTCTTATTGTAACACAAAAACGACATCATACTCGA
TTTTTTCCAGAGGGCTGGAGAGATGGATCTTCCAGTGGCAATGTTTTGCCGGGAACAGTT
GTCGACACAAAAGTTATACACCCTTATGAGTTTGACTTCTACCTTTGTAGTTACTATGGA
AACCTAGGTACAAGCAAGCCTACTCATTACCATGTCTTATGGGACGAGCACAAGTTTACA
TCTGATTTATTGCAGAAGCTAATATACGAGATGTGTTTCACCTTTGCAAAGTGCACTAAA
CCTGTATCATTAGTCCCTCCTGTGTATTATGCTGACCTTGCTGCTTATAGAGGACGATTA
TACCATGAAGCAAGGATCAGGATGCAATCTCCAAAGTCAACAGCATTTTCATCTTCTAAA
GATGCTTCATCAGCTTCACGAACTGCTTCATTTGAACAGGGATTTTACACATTGCATGCT
GACCTGGAAAACATAATGTTCTTCATCTAA
>Glycine-max_Glyma16g34300
ATGGTCAGAAAGAGAAGAACTGAACTACCAAGTGGGGGTGAAAGCTCTGAGGCTCAACAC
CCTTCTGAAAGGAGTGCACCACCGCCCCAACAACAAGCTGCTGCTGCTGCCCCAGGAGGG
GCTGGACCCCAAGGAGGCAGAGGCTGGGGTCCCCAAGGAGGAAGAGGAGGGGGCCGCAGC
CGTGGGATGCCCCAACAGCAATATGGTGCCCCTCCTGATTATCAAGGTAGGGGAAGGGGA
GGGCCTTCTCAGCAAGGAGGCCGTGGAGGATATGGCAGTGGCCGAAGTGGTGGTGGTGGT
GGTGGTATGGGCAGTGGCCGTGGCGTTGGTCCTTCATATGGTGGCCCATCGAGGCCACCG
GCACCCGAGCTGCACCAAGCAACCTCAGTTCAGTCATATCAAACTGGGGTGAGTTCTCAG
CCTGCATCATCTGAGGCCAGTTCATCCCTGCCGCCAGAGCCTATTGATTTGGAACAGTCA
ATGGGGCAGATGGTGCTTCATTCTGAACCTGCTCCTACTCCTCCTCCTGCAAGTAAATCA
TCAATGAGGTTTCCCCTTCGACCTGGAAAGGGTAGCTATGGCACCAAGTGTGTTGTTAAG
GCTAATCATTTCTTTGCTGAGTTACCCAACAAAGATCTGCATCAATATGATGTTACAATT
ACTCCAGAAGTGATATCAAGAGGGGTGAACCGTGCTGTTATGGAGCAGTTGGTGAGACTG
TACCGGGAATCTCACTTGGGTAAGAGACTCCCTGCTTATGATGGACGCAAGAGCCTCTAT
ACTGCTGGACCACTTCCTTTTATGTCAAAGGAGTTCAGAATTGTTCTCGTTGATGATGAT
GAAGGAGCTGGAGGCCAGAGGAGGGACAGGGAGTTCAAGGTTGTGATAAAATTGGCTGCA
CGTGCAGACCTTCACCATTTGGGACTCTTTTTACAAGGAAGGCAAACTGATGCTCCTCAA
GAGGCTTTGCAGGTCCTTGACATTGTTCTGCGCGAACTCCCTACTACAAGGTATTGTCCT
GTTGGAAGATCATTTTATTCACCTGATTTGGGCAGAAGACAGCCTTTAGGTGAGGGATTG
GAAAGCTGGCGTGGTTTCTACCAGAGTATTCGGCCTACACAGATGGGGCTATCACTGAAC
ATTGATATGTCTTCCACTGCATTTATTGAGCCATTGCCAGTAATTGATTTCGTAACTCAA
CTGCTGAACAGAGATGTATCCGCCCGGCCACTTTCTGATGCTGATCGTGTGAAGATCAAG
AAAGCTCTCCGGGGTATCAAAGTTGAAGTGACACATCGTGGAAACATGAGAAGAAAATAT
CGTATCTCTGGTCTGACTTCACAGGCAACCAGAGAATTGACATTCCCGGTAGATGAAAGG
GGAACCATGAAATCTGTTGTGGAGTACTTCTATGAGACATATGGGTTTGTCATTCAACAT
ACTCAGTGGCCTTGTCTGCAAGTTGGCAATGCACAGAGACCAAACTATTTGCCAATGGAG
GTTTGCAAGATAGTGGAGGGTCAAAGGTACTCGAAAAGGCTTAATGAGAGGCAAATCACT
AATTTGCTGAGAGTTACATGCCAGCGTCCTGGTGAGAGGGAGCGTGATATCATGCAGACA
GTACACCACAACGCATACCACGAAGATCCTTATGCTAAAGAATTTGGAATCAAGATTAGC
GAGAAACTTGCTCAAGTTGAAGCTCGCATCCTTCCTGCTCCATGGCTCAAATATCACGAT
ACGGGCAGAGAAAAGGATTGTCTTCCTCAAGTTGGGCAATGGAATATGATGAATAAGAAA
ATGGTTAATGGGGGGACAGTTAACAACTGGTTCTGCATAAACTTTTCAAGGAATGTTCAA
GATAGTGTTGCCCGTGGTTTTTGCTATGAACTTGCTCAGATGTGTTATATATCTGGAATG
GCATTTACACCTGAGCCAGTAGTCCCCCCAGTCAGTGCTCGCCCTGATCAAGTGGAAAAG
GTTCTTAAAACTCGGTATCATGATGCCAAGAACAAACTGCAAGGAAGAGAGCTTGATTTG
CTCATCGTTATCTTGCCCGATAATAATGGATCTCTTTATGGTGATCTCAAACGGATATGT
GAGACTGACCTAGGACTTGTTTCACAATGTTGCTTAACTAAGCATGTCTTCAAAATGAGC
AAGCAGTACCTTGCAAATGTTGCTTTGAAAATTAATGTCAAAGTTGGAGGGAGAAACACT
GTACTGGTTGATGCGCTCTCACGACGCATTCCCTTGGTCAGTGACAGACCTACAATTATT
TTTGGAGCTGATGTGACTCATCCACATCCTGGAGAGGATTCAAGCCCATCAATTGCAGCA
GTTGTGGCTTCGCAAGACTATCCTGAAATTACAAAGTATGCTGGTTTAGTTTGTGCCCAA
GTTCATAGGCAGGAACTCATTCAGGATCTTTTCAAACAATGGCAAGATCCAGTCAGAGGA
ACAGTGACTGGTGGAATGATCAAGGAACTTCTTATATCTTTTAGGAGAGCTACAGGACAA
AAGCCACAACGCATCATATTTTATAGGGATGGTGTGAGTGAGGGACAGTTTTATCAGGTT
CTACTGTTTGAGCTTGATGCTATTCGAAAGGCATGTGCATCCCTGGAACCCAATTATCAG
CCTCCTGTGACTTTTGTGGTGGTTCAAAAGCGTCACCACACAAGGCTCTTTGCTAGCAAC
CATCATGATAAGAGTTCTGTTGACAAGAGTGGCAACATATTGCCTGGCACTGTTGTTGAC
TCCAAAATCTGCCATCCCACCGAATTTGACTTTTATCTCTGCAGCCATGCTGGAATACAG
GGTACAAGCCGTCCTGCTCACTACCATGTGTTGTGGGATGAAAACAATTTTACTGCTGAT

GCCTTGCAAACACTCACCAACAATCTTTGCTACACATATGCTCGGTGCACCCGGTCTGTT
TCAATCGTGCCTCCTGCATACTATGCTCACCTTGCTGCATTCCGCGCAAGGTTTTACATG
GAACCAGAGACGTCGGATAGTGGCTCTATGACAAGTGGTGCTGTTGCAGGCCGTGGGATG
GGCGGTGTAGGGCGTAGCACGCGTGTACCTGGTGCCAATGCTGCTGTGAGACCATTGCCT
GCACTCAAGGAGAACGTCAAGAGAGTTATGTTTTATTGTTAA
>Glycine-max_Glyma17g12850
ATGAAGGATCCAGTGGAGGATCAAGTGAAATCTAGGAGGGCAGTGCAGAAACCCAAGGAT
TTTAGAAGAAGAGATAGAAGAAGAAACCAATGCAAACAAGAGAAATTACAAGACACACAA
TTGGTTTCCTCTGCTTGCAAAAGTCTTGTGTTTCCTGCAAGGCCTGGCTATGGCCAGCTG
GGGACAAAGTGTCTGGTCAAAGCCAACCACTTCCTGGCAGATATATCCGCATCTGACTTG
AGCCATTACAATGTTAAAATAACACCTGAGGTTACTTCTCGTAAAACAAGCAAAGCCATC
ATAGCTGAGTTAGTGAGGCTTCACAGGAACACTGATTTGGCAATGAAGCTTCCTGTTTAT
GATGGAGGAAGAAATCTCTACACTGCTGGCTTGCTTTCTTTTGCATACAAAGAGTTCACC
ATACTATTGAGAGAGGATGATGAGGGCACTGGTTCTACCAGGGAAAGAGAATTTGAAGTG
GTGATCAGGTTTGCAGCTCGTGTTAGCATGAATCAGTTACGTGAGCTTCTGAGTGGCAAG
CAAGTGGACACTCCACAAGAAGCACTTACTGTCATTGACACTGTATTGAGGGAGCTTGCA
GCTCAGAGCTACGTGTCAATTGGGAGGTTTCTATATTCTCCGGATTTAAGAAAACCACAG
CAGCTAGGTGGTGGCCTGGAATCGTGGTGCGGCTTCTATCAGAGTATAAGGCCAACTCAG
ATGGGATTGTCACTTAATATTGACATGTCATCAATGGCGTTTATTGAACCACTTCCTGTA
ATTGACTTTGTTGCTCAAATTTTGGGAAAAGATGTGCTCTCAAAGCCATTGTCAGATGCA
GATCGTGTCAAGATTAAGAAGGCCTTAAGAGGTGTGAAAGTTGAGGTTACACATAGAGGA
AGTTTTCGAAGGAAGTACAGGATTACAGGATTGACATCACAGCCTACAAGGGAGCTTAAT
TTCCCCGTCGATGAGAAAATGAACATGAAATCAGTAGTTGATTACTTTCAAGAAATGTAT
GGATATACAATCATATATTCTCATCTACCCTGCCTTCAAGTAGGAAGCCAAAAGAAGGTG
AACTATTTGCCTATGGAGGCATGCAAGATAGTTGGGGGTCAGAGATATACAAAAGGGCTT
AACGAAAAGCAGATAACTTCTCTGCTAAAGGTCTCATGCCAGAGACCACGTGAACAAGAG
ACAGATATTCTACAGACAATTCACCAAAATGATTATGAGTATAATCCCTATGCCAAGGAG
TTTGGGATCAGCATTGACAGCAAGCTTGCATCAGTTGAGGCTCGGGTTCTTCCTGCTCCA
TGGTTGAAATATCACGAGACTGGAAGGGAGAAAGAATACCTGCCACAAGTTGGTCAATGG
AACATGATGAACAAGAAAGTTATAAACGGAAGCACTGTAAGATATTGGGCTTGTATTAAT
TTCTCACGAAGTATCCAAGAAAGTACTGCTCGCGGGTTTTGCCAACAGTTAGTTCAAATA
TGCCAAATCTCAGGCATGGAATTTAGTCAAGACCCTGTGATTCCAATATATTCAGCAAAA
CCTGATCTGGTAAAGAAAGCCTTGAAGTATGTACATTCTGCTGTACTTGATAAACTTGGT
GGGAAAGAACTAGAGTTGTTGATTGCCATTCTTCCAGACAACAATGGCTCTCTGTATGGC
GATCTCAAAAGAATCTGTGAAACCGATCTGGGGTTGATTTCTCAGTGCTGTCTTACAAAA
CACGTATTCAAGATCAATAGGCAGTATTTGGCAAATGTGGCACTAAAGATCAATGTCAAG
ATGGGAGGAAGGAACACAGTACTTTTGGATGCCCTAAGTTGGAGGATCCCATTGGTTAGT
GACATTCCAACAATAATTTTTGGAGCAGATGTAACACATCCAGAATCTGGAGAGGACCCT
TGTCCATCCATTGCTGCTGTTGTAGCCTCCCAGGACTGGCCGGAAGTAACAAAGTACGCA
GGATTGGTATGCGCTCAGCCTCATCGTGAGGAACTCATTCAAGATCTTTTTAAATGTTGG
AAGGATCCTCATCATGGTATAGTTTATGGTGGCATGATCAGAGAGCTGTTACTCTCTTTT
AAGAAGGCAACCGGACAAAAACCATTGAGGATAATATTTTACAGGGATGGGGTAAGTGAA
GGACAGTTCTACCAGGTTTTGTTGTATGAGCTTGATGCCATCCGTAAGGCTTGTGCATCT
TTGGAACCTAGTTACCAACCTCCGGTAACATTTGTTGTGGTTCAAAAGCGACATCACACT
AGACTCTTCTCAAACAATCATGACGACAGAAATAGCACTGATAAGAGTGGGAATATCTTA
CCTGGTACTGTGGTGGATTCTAAGATCTGTCATCCTACGGAATTCGACTTCTATTTATGC
AGTCATGCGGGAATTCAGGGTACAAGTAGACCAGCTCATTATCATGTTCTGTGGGACGAG
AACAATTTCACTGCTGATGAGATCCAATCTCTGACCAACAACTTGTGCTACACCTATGCA
AGATGTACACGATCAGTTTCTGTAGTGCCTCCTGCGTACTATGCTCATTTGGCAGCTTAC
AGAGCTCGATTCTACATGGAACCTAATGTCCATGAAATTGCTAAATCTCGAGGTGCAAGG
TCAAAAGATGAGTCAGTTCGGCCACTACCTGCTCTGAAAGAGAAGGTGAAGAATGTAATG
TTTTATTGTTGA
>Glycine-max_Glyma20g02820
ATGGAAAGAGGTGCTTACAGAGGCCGTGGCCGTAACGGTAATGGTGGCCGTAACAGTTAC
GGTGGTGGTCGCGGTAGTTACGGTGGTGGCCGTGACGGTAACGGTGACCATAACAGTTAC
GGTGGTGGCCGCGGTGGTCGTAGCAGTTACGGTGATAGAAGTTCTCCTTCTCAATCACAG
TGGCAACCAAGGTCAAACCCTTCTTTTTCTTCTCCAAAACCTAACAATTCTCATTCTCAA
GTTCAAACACAAACTCAAGCTCATCCAGGTATTCAGTATCAGCCACTAATTTTTCTTTTC
TTACAGTTTTTAGTCTACATTGGATCTTCGAAGATACCAGAGAAGAAAATGGACACCATC
ACACCTGTGCGTAGGCCTGACAATGGGGGAACAGTTGCAGTGCGAAAGTGCTATCTCCGT
GTGAACCATTTCCCTGTTTCCTTCAATCCACAGAGTATAATTATGCATTATAATGTGGAA
GTGAAGGCCAAGGCTCCTCCACTGAAGAACAATCGTCCTCCCAAGAAGATTTCGAAGTAC

GACTTGTCATTGATTCGGGATAAGCTGTTTTCCGACAATTCACTGCCAGCTTCAGCGTAT
GATGGTGAAAAGAACATCTTCAGCGCGGTGCCTTTGCCGGAGGAAACATTTACCGTGGAC
GTGTCCAAAGGAGAGGACGAAAGGCCTGTTTCTTATTTGGTCTCTCTGACATTGGTGAGT
AGGCTCGAGCTTCGGAAGTTGAGGGATTACCTCAGTGGAAGCGTGCTTTCGATCCCTAGG
GATGTTTTGCACGGCTTGGATTTGGTGGTGAAGGAAAATCCTTCGAAGCAGTGTGTTTCC
TTGGGGCGGTGCTTCTTCCCCATGAACCCTCCTTTGAGGAAGAAAGATCTTAACCATGGC
ATAATTGCGATTGGAGGGTTTCAGCAGAGTCTTAAGTCTACTTCTCAGGGATTGTCCTTG
TGCCTGGACTATTCGGTTTTGTCCTTTCGGAAGAAGCTGTTGGTGTTGGATTTTCTGCAC
GAGCATATTAGGGACTTCAATTTAAGGGAGTTTGGGCGGTTCAGGAGACAAGTTGAGCAT
GTACTTATTGGGTTGAAGGTTAATGTTAAACACCGGAAGACAAAGCAGAAGTACACTATT
ACTAGGTTGACACCCAAGGTTACGAGACATATCACATTCCCTATTTTGGATCCCGAGGGC
CGGAATCCCCCAAAGGAAGCTACTCTGGTTGGTTACTTTCTAGAGAAGTATGGTGTGAAC
ATTGAATACAAGGACATTCCTGCCTTGGATTTTGGAGGCAACAAGACGAATTTTGTGCCT
ATGGAGTTGTGTGAGTTGGTTGAGGGGCAGAGATATCCCAAAGAGAATTTGGACAAATAT
GCTGCCAAGGACTTAAAAGACATGTCAGTGGCTCCTCCAAGGGTGAGGCAAAGTACAATA
CAAGCAATGGTAAACTCAGAGGACGGACCGTGCGGAGGTGGTGTTATTAAAAATTTTGGA
ATGAGTGTCAACACTTCCATGACAAATGTGACAGGACGTGTAATTCAGCCTCCACAATTG
AAGCTAGGTAATCCAAATGGCCAGACTGTTAGTATGACACTTGAAGTAGAGAAATGTCAG
TGGAATCTAGTGGGACGATCAATGGTGGAAGGCAAGCCAGTTGAGTGTTGGGGCATTCTT
GATTTTACCAGCCAGGAGTCTGGTTGGCGCAAATTAAACAGCAAACAATTCATTGAGAAC
CTTATGGGTAAGTATAGAAAATTGGGTATTGGCATGAAGGAGCCAGTTTGGCGTGAACAA
TCTAGTATGTGGAGTCTTGGGGATTACAATTCGCTGTGTAAATTACTTGAAAATATTGAG
GATAAGGTTCAAAAAAGATATCGACGAAAACTACAATTTCTTCTGTGTGTGATGTCCGAC
AAGCATCAAGGTTACAAGTGCCTCAAATGGATTGCTGAGACCAAGGTTGGCATAGTGACA
CAATGCTGCTTGTCTGGTATTGCTAATGAAGGGAAGGACCAATATCTTACAAATCTTGCC
CTCAAGATCAATGCCAAAATTGGAGGAAGTAATGTGGAGCTCATCAATAGGCTACCACAC
TTTGAGGGTGAAGGTCATGTTATGTTCATAGGGGCTGATGTCAATCATCCAGCTTCCCGG
GACATCAACAGTCCATCAATTGCTGCTGTAGTTGCCACTGTTAATTGGCCTGCTGCAAAT
CGCTATGCAGCACGTGTTTGTGCTCAAGGTCATCGGGTTGAGAAAATTTTGAATTTTGGG
AGAATTTGCTATGAACTTGTTTCGTATTACGATAGGCTGAACAAAGTCAGGCCTGAAAAA
ATTGTTGTCTTTCGTGATGGCGTGAGCGAAAGTCAATTCCATATGGTTCTCACAGAGGAG
TTACAAGATTTGAAATCGGTGTTTAGTGATGCAAATTACTTCCCAACCATCACTATTATT
GTCGCACAAAAGCGACATCAAACTCGATTTTTTCCTGTGGGTCCAAAGGATGGGATTCAA
AATGGCAATGTGTTTCCAGGTACAGTTGTGGACACAAAAGTAGTACATCCTTTTGAATTT
GACTTTTACCTTTGTAGTCACTATGGAAGCTTGGGTACTAGTAAGCCCACTCACTATCAT
GTCTTATGGGATGAGCATAAATTTAACTCTGATGATTTGCAGAAACTGATATATGACATG
TGCTTTACCTTTGCAAGGTGCACTAAACCTGTATCTTTAGTCCCTCCAGTGTACTATGCT
GATCTCACTGCATATAGAGGACGGTTATACTATGAAGCAATGAATCAAATGCAATCTCCT
GGTTCAGCTGTGTCGTCTTCATCATCACAGATTACTTCTTTGTCAATTTCTTCAACAGGC
TCAAGTTTAAATGATCCGGGGTATTACAAGTTGCATGCTGATGTGGAAAATATAATGTTC
TTCGTTTGA
>Glycine-max_Glyma20g12070
ATGGATTCATTTGAGCCAGATGGAAATGGGAAGGAGTCACTGCCACCACCACCTCCTGTT
GTTCCCTCTGATATTGTACCTCTCAAAGCAGAGGAGGTGCTCTGTACCCCTACCGAGCAT
AATAAGAAAAAGGCTTCCCGACTTCCAATAGCCAGATCTGGTCTGGGATCAAAAGGAAAT
AAAATACAATTACTAACCAATCACTTCAAAGTTAATGTTGCTAAAAATGATGGGCATTTC
TTCCATTATAGTGTGGCTTTTACTTATGAAGATGGACGCCCTGTAGAAGGTAAGGGTGTA
GGGAGAAAGATAATAGATAGGGTGCAGGAGACATATCATTCTGACTTAAATGGTAAGGAC
TTTGCATATGATGGGGAGAAAAGTCTGTTTACTGTTGGCTCTCTTCCTCAAAACAAGCTT
GAGTTTGAAGTTGTTCTTGAGGATGTCACCTCTAACAGGAATAATGGCAATTGCAGCCCT
GATGGTCTAGGGGACAATGAGAGTGACAGAAAGAGGATGCGACGTCCTTATCGTTCGAAG
TCATTCAAAGTAGAGATAAGCTTTGCTGCAAAAATTCCAATGCAGGCCATTGCCAGTGCC
TTACGCGGGCAAGAGACTGAGAATTTTCAAGAAGCCATCAGAGTTCTTGATATCATTTTG
AGGCAGCATGCTGCTAAGCAAGGCTGCTTACTTGTACGCCAATCCTTTTTCCACAATAAT
CCAAATAATTTTGCTGATGTAGGAGGTGGTGTCCTAGGCTGTAGAGGATTCCACTCAAGC
TTTAGAACTACACAGAGTGGCCTGTCTCTTAACATAGATGTGTCAACTACAATGATAATT
TCTCCTGGGCCTGTGGTGGATTTCTTAATTTCCAATCAAAATGTGAGAGATCCTTTTCAA
CTTGACTGGGCTAAGGCCAAAAGGACCCTAAAAAATCTGAGGATTAAAACTAGCCCATCC
AATCAAGAATTCAAAATTTCTGGGCTCAGTGAACTCCCATGCAGAGAGCAGACTTTTACT
TTGAAAGGTAAAGGTGGGGGGGATGGTGAAGATGGTAATGAGGAAATCACTGTATATGAT
TATTTTGTTAAGGTTCGTAAGATAGATCTCCGATACTCTGCTGACCTTCCATGTATCAAT
GTTGGCAAGCCTAAACGACCAACATTTTTCCCCATTGAGGTTTGTGAATTGGTATCATTG

CAACGATATACAAAAGCTCTGTCCACGCTTCAAAGGGCTTCATTAGTGGAGAAGTCGAGG
CAGAAGCCACAAGAGAGGATGAAAATTTTGTCTGATGCACTGAGAACAAGCAACTATGGT
GCTGAACCTATGCTCCGGAATTGTGGAATTTCTATAAGCACTGGCTTCACTGAAGTGGAG
GGCCGGGTGTTGCCTGCACCAAGGTTGAAGTTTGGCAATGGTGAGGATCTCAATCCTAGG
AATGGGAGATGGAATGTCAGCAGAGTGAAATTTGTGGAACCATCAAAGATAGAAAGATGG
GCTGTTGCTAACTTTTCTGCACGCTGTGATGTACGAGGACTTGTACGGGACCTCATTAGA
ATTGGAGATATGAAAGGAATTACTATAGAACAACCATTTGACGTGTTTGATGAGAATCCA
CAGTTTAGGCGTGCCCCCCCTATGGTTAGAGTGGAGAAAATGTTCGAGCATATCCAATCT
AAACTTCCTGGGGCTCCTCAGTTCCTTCTCTGTTTGCTTCCTGATCGGAAAAATTGTGAT
ATTTATGGTCCATGGAAAAAGAAGAATCTTGCTGATTTTGGAATCATAAATCAGTGTATG
TGTCCTTTAAGGGTCAATGACCAGTACCTGACTAATGTTATGTTGAAGATCAATGCCAAG
CTTGGTGGGTTGAATTCATTGTTAGGCGTTGAACATTCTCCTTCTCTTCCTGTTGTTTCC
AAAGCTCCCACCCTCATTCTGGGAATGGACGTGTCACATGGCTCACCTGGGCAGACTGAC
ATTCCTTCAATTGCTGCGGTGGTCAGCTCTAGACACTGGCCTCTGATATCAAAGTATAGG
GCATGTGTTCGTACGCAATCTGCAAAGATGGAAATGATTGATAATTTGTTCAAGCTAGTA
TCTGAAAAGGAAGATGAAGGCATCATAAGGGAACTTTTGCTTGATTTCTATACAACTTCT
GGGAGGAGAAAACCGGAAAATATAATCATATTCAGGGATGGGGTTAGTGAGTCACAATTC
AATCAAGTTTTGAATATTGAACTCGATCGAATCATTGAGGCTTGCAAATTTCTCGATGAA
AATTGGGAGCCAAAATTTGTGGTAATTGTTGCTCAGAAGAACCACCACACTAGATTTTTC
CAGCCTGGCTCTCCCGACAATGTCCCACCTGGCAAGTGTTCTGGGTCTTTACAGCATTTA
GGGAGTTGTGAAAAAGGTGCCCCTTCACCTGCCCTCAATTACTGTGGAACTAGTAGGCCT
ACCCATTATCATGTGCTGCTTGATCAGGTTGGTTTCTCTCCGGATCAGCTGCAGGAGCTT
GTCCATTCATTATCATATGTGTATCAGAGGAGCACTACTGCCATTTCTGTTGTTGCTCCA
ATATGCTATGCGCACTTGGCTGCTACTCAGTTGGGGCAGTTCATGAAATTTGAGGACAAA
TCTGAAACATCTTCAAGCCATGGTGGATTGAGCGGTGCAGGTGCTGTTCCCGTCCCTCAG
TTGCCTCCCTTGCAGGAGAATGTCCGCAACACAATGATTACTGGGAGACTGCTTCCATCA
ATGTTCAAGACCACTCCACCGTTGTGGGGGAGGATGCCACTGAACCCAAGTATCAGGCGG
AGAATCTCTGGCGTTGTACCCTTTGTTGTCGCTGAGGCTTCTGTTTTCCCCAACCTGTGG
TCAGTTAGGGTTTTCATAGCTTTTGCTTTCGCCCACCGTGTCGGTTCCTAG
>Glycine-max_Glyma20g28970
TTTTTGGTAGGGGACAAGCCTAACAAGGGAAGAAGAAGGGGGAGAGGTGGCAGAAAATCT
GATCAAGGAGATGTTATGATGAGGCCTAGTTGCAGGCCATGCACTGCACCACTAACAAGT
AGTGCAAATGGGAACGCTGAAAATGGTTGTATCTCTGATACGGGTTTTCCCACTTCAAGC
AAGTCTTTGACCTTTGCTCGTAGGCCTGGGTATGGACAAGTTGGGACAAAATGCATTGTG
AAGGCTAACCACTTCTTTGCAGAGTTACCAGACAAGGACTTGAACCAATATGATGTTACT
ATTACCCCAGAAGTGTCTTCTAGAACAGTAAACAGGTCTATCATAGCAGAACTTGTGAGG
CTGTATAAAGAGTCTGACTTGGGGATGAGACTTCCAGCATATGATGGCAGAAAAAGTCTG
TACACTGCAGGGCAGCTTCCCTTTGCTTGGAGAGAGTTTAAGATTAAGCTTGTAGATGAA
GAGGATGGAGTTAATGGCCCCAAAAGGGAAAGAGAGTACAGGGTGGTGATCAAGTTTGTT
GCTCGGGCAAATTTGTATCACTTGGGCCAATTTCTAGCTGGTAAGCGTGCTGATGCACCG
CAAGAGGCACTTCAAATTCTTGACATTGTACTAAGAGAGCTGTCAACTAAGAGGTATTGC
CCCATTGGGAGGTCCTTCTTTTCACCTGATATTAGAACACCGCAACGGCTTGGAGAGGGA
TTAGAGTCATGGTGTGGATTTTACCAGAGTATAAGGCCTACACAAATGGGCCTTTCCCTT
AATATTGATATGGCATCTGCTGCGTTTATTGAGCCTCTGCCAGTAGTGGAATTTGTTGGT
CAGCTATTAGGAAAAGATGTGCTGTCAAGGCCATTGTCAGATGCTGATCGCATTAAGATT
AAGAAAGCCCTTAGAGGAGTTAAAGTTGAAGTAACACACAGAGGAAGTGTGAGAAGAAAG
TATCGTGTTTCTGGGTTGACTTCTCAACCTACCAGAGAACTTGTGTTTCCTGTTGATGAG
AACTCAACTATGAAATCAGTAGTTGAATACTTCCAAGAGATGTATGGTTTCACTATTCAA
TATACTCACCTTCCTTGCCTTCAAGTAGGAAACCAAAAGAAGGCGAACTATTTACCTATG
GAGGCCTGCAAAATTGTTGAGGGGCAACGTTATACAAAAAGATTGAATGAGAAGCAAATT
ACGGCTCTATTGAAAGTTACTTGCCAGAGGCCTCGCGATCGGGAAAATGACATTTTACGG
ACCGTTCAACATAATGCTTATGATCAAGATCCTTATGCAAAGGAATTTGGAATTAAAATC
AGTGAAAAGCTAGCTTCTGTTGAAGCACGAATTCTTCCTGCTCCTTGGCTTAAATATCAT
GAAAGTGGGAAAGAAAAGAACTGTTTACCCCAAGTTGGTCAGTGGAATATGATGAACAAG
AAAATGATTAATGGAATGACTGTTAGCCGGTGGGCATGCATAAATTTTTCACGGAGCGTG
CAAGATAGTGTTGCTCGTACTTTTTGTAATGAGCTTGCTCAAATGTGTCAAGTATCTGGC
ATGGAATTTAATCCAGAGCCCGTTATCCCCATCTACAATGCCAAGCCTGAACAGGTGGAG
AAAGCTTTGAAACATGTTTACCATGTGGCAGGGAGCAAAACTAAAGCAAAGGAATTGGAG
CTTTTGTTAGCAATATTGCCAGACAATAATGGGTCTCTCTATGGTGATCTCAAGCGAATT
TGTGAAACTGACCTTGGCTTAATTTCACAATGCTGTCTGACAAAGCATGTCTTCAAAATC
ACTAAACAGTACCTGGCTAATGTGTCTCTGAAGATCAATGTGAAGATGGGAGGTAGAAAC
ACTGTACTTCTTGATGCTGTAAGCTGCAGAATACCATTGGTTAGTGACATACCAACCATA

ATTTTTGGAGCAGATGTAACCCACCCAGAAAATGGAGAAGACTCGAGCCCTTCAATAGCA
GCTGTAGTAGCATCCCAGGACTGGCCCGAAGTGACAAAATATGCCGGTTTGGTATGCGCC
CAAGCTCATAGGCAGGAACTTATACAAGATTTGTACAAAACTTGGCAAGACCCTGTTCGT
GGCACAGTTAGTGGTGGCATGATCCGAGATTTACTGGTTTCCTTTAGAAAGGCAACAGGA
CAAAAGCCACTACGAATTATATTTTACAGGGACGGTGTAAGTGAAGGGCAATTTTACCAA
GTTTTACTTTATGAGTTGGATGCGATTCGGAAGGCATGTGCTTCCTTAGAACCAAACTAC
CAGCCTCCAGTAACATTCATAGTTGTGCAAAAAAGACATCATACCCGGTTATTTGCAAAC
AACTACAGGGACAGAAGTAGTACAGATAGGAGTGGGAATATATTGCCTGGGACTGTCGTT
GATTCCAAAATCTGCCATCCAACAGAATTTGATTTTTATCTCTGCAGCCATGCTGGCATC
CAGGGTACTAGTCGGCCAGCTCATTATCATGTCCTGTGGGATGAAAACAACTTCACAGCA
GATGGAATTCAGTCTCTGACAAACAATCTTTGTTATACATATGCCAGGTGTACGCGCTCA
GTATCAGTTGTTCCTCCAGCATATTATGCACATTTAGCAGCGTTCCGAGCACGTTTCTAT
ATGGAGCCAGATATGCAAGACAATGGCTCTGCAGGTGATGGCAATGGTTATGGTGCCAAA
GCAACACGAGCAGCTGGTGATTATAGTGTCAAGCCATTGCCAGACTTGAAAGAAAATGTG
AAGAGGGTCATGTTTTACTGTTAG
>Manihot-esculenta__cassava1330.valid.m1
ATGGAGGATTCGGAAGAATCCAATGCTAGCAAGAAATGCACCGCTAAGACTAGGACTCTT
AGGGGCAGGACTAACTCCGACCATAAGCATCAGTATCAGTATCAATATCAGCATCACCTC
TTTCAATACTCTAACCAGTTTGGTTTGTTGAACCATGGCCAGTATCCAAGTTACTGCCCA
GCTCTTCTTCCTCTGCCTCCGCCGATACCTCTCCAACTTGCTCTTGTTCCACCTTTCCCT
CAAAACCATAGCTTCAGATCAAAAACCCATTTTCAGAAACCTTCGTTTAAGCTAAATAAT
CATCCACCTCCTCCTCTTCCCACCCCTTTTGTTAGCCAAGGACCAGTTGCAGCAATTTCA
TCAGCTGCAAGGAGACCAGATTCTGGTGGAGTAGAAGGACATGTTATTACTCTTCTTGCT
AACCATTTCCTTGTCCGATTCAATTCCTCGCAGAGAATTTTCCATTACAATGTGGATATT
TTTCCCAGTCCTTCAAAGGAAGTTGCCCGAATGATCAAACAAAAACTTGTAGAGGATAAT
TCAGCTGTGCTCTCTGGGGCTTTTCCAGCCTATGATGGTCGAAAGAATTTTTACAGTCCA
GTTGAGTTCCGCAATGATAGGCTTGAATTCTACATTAGCCTCCCAATCCCAACTAGCAAA
TCATCTTTGCCTTTTGGAGAACTGAGTGACTTTCAAGAGAAGCATCAACAGTTGAAACTA
TTTCGAATAAATATCAAGCTTGTATCAAAGCTGGATGGAAAGGAACTGAGCAGATACTTG
AGCAAAGAAGGTGATGACTGGATCCCACTTCCTCAGGATTATCTACATGCGTTGGATGTT
GTTCTGAGAGAAAGTCCCATGGAGAAATGTATACCTGTGGGGAGATCATTCTATTCAAGT
TCAATGGGAGGAACAAAGGAAATTGGAGGAGGAGCCATTGGATTGAGAGGATTCTTTCAA
AGTCTCAGACCCACCCAACAAGGATTAGCTCTCAATGTGGATTTTTCAGTGACTGCTTTT
CATGAAAGTATTGGAGTGATCCCTTACTTGCAAAAGCGTCTCAAGTTTCTTAGGGACCTC
CCACAGAACAAAACAAGGAGTTTGATTGGTGAAGAAAGGAAGGAAGTGGAGAAGGCTTTG
AAGAACATCAGGGTCTTTGTTTGCCACAGAGAAACAGTCCAGAGGTACCGAGTTTTCGGC
TTAACTGAAGAAGCTACGGACAATCTTTGGTTCGCGGACAGGGATGGGAAGAATCTGAGG
CTGGTGAGTTACTTCAAGGACCACTACAATTATGATATAAAATTCCGGAACTTGCCATGT
TTGCAGATCAGTAGAAGTAAACCATGTTATCTTCCTATGGAGCTTTGTATGATATGTGAA
GGCCAGAAGTTTCTTGGTAAGCTGTCAGATGATCAGACTGCAAAAATACTCAAGATGGGC
TGCCAAAGGCCTAAAGAACGAAGAACAATTATACATGAAGTCATGAGAGGTTCTGTTGGG
CCAACCAGATCCAAGACGTATATGAGCAGAGAATTCAAACTCCATGTTTCAAGAGAAATG
ACAAGATTGAATGGGAGAATCCTACAACCTCCAAAACTAAGACTTGGTGACGGTGGCCTT
ATAAGAGATCTGATACCTTCTCGCCAGGATCGCCAATGGAACCTTGCCGACAGCCATGTC
TTTGAAGGAACAAGAATAGAAAGATGGGCGCTGATAAGTTTTGGAGGCACCCcTGAACAG
AAGTCTAACATTCCAAAATTCATAAACCAGCTATCCCAAAGGTGTGAACACTTAGGCATC
TTTCTTAGCAAAAACACAATTATTAGCCCACAATATGAGCCAACTCAAGTGCTCAATAAT
GTGGCCCTTCTGGAATCAAAACTCAAGAAAATCCAGAAAGCTGCCTCAAACAATCTCCAG
CTGCTTATTTGTATAAtGGAGAAGAGACACAGAGGATATGCAGATTTGAAGCGGATAGCA
GAGACAAATGTAGGTGTTGTAAGCCAGTGCTGCTTGTTTACAAATCTTGGCAAGTTGAGT
TCACAATTTCTTTCCAATTTGTCTCTCAAGATCAATGCCAAACTTGGAGGATGTACAGTT
GCTTTGTACAATTCATTACCCTCTCAGATTCCCCGCCTTCTTCATTCCGACGAGCCCGTG
ATCTTCATGGGAGCTGACGTCACTCATCCTCACCCTCTCGATGACTTCAGTCCATCAGTT
GCTGCTGTTGTTGGTAGCATGAACTGGCCAGCAGCAAATAAGTATGCATCTCGAATGAGG
TCGCAAACGCATCGCCAAGAAATCATCCAGGATCTTGGTTCAATGGTGAAAGAATTGCTA
GATGATTTTTACAAAGAAGCAAACAAACTTCCCAAAAGGATAATGTTCTTCAGGGATGGA
GTAAGTGAAACTCAGTTTCATAAGGTTCTCCAAGAAGAGCTGAAATCAATTCGAGAAGCT
TGTTCTAGATTCCCTTGTTATAAACCTCCCATTACCTTTGCAGTTGTCCAAAAGAGGCAT
CATACAAGGTTGTTTCCCTGCGAAACAGATCTATCTTCTTCTATTCAGGACCAGTTTTAC
AATGAAAATATCCCACCAGGGACAGTTGTGGACACTGTGATTACACATCCAAAGGAATTT
GATTTTTATTTGTGCAGCCATTGGGGGGTgAAAGGAACAAGCAGGCCAACACATTACCAT
GTGTTGTGGGATGAGAACCAATTCACTTCTGATGAATTACAGAAGTTGGTATACAATCTC

TGCTACACATTTGTAAGATGCACCAAGCCAGTTTCTTTGGTGCCTCCTGCTTACTACGCC
CACTTAGCTGCATACAGGGGCAGGCTTTACATTGAACGGTCTGAGTCGTCCATGAGAAAT
GCTTGTACGATTTCAAGAGCTTCCCCACCAAAAGCAACACCTCTACCGAAGCTCAGTGAG
AAAGTAAAGAATCTCATGTTCTACTGCTGA
>Manihot-esculenta__cassava13333.valid.m1
ATGGAGAGATACTCTGCTGCCGCTAGTGGAAGAGGAAGGGACGGTCAGGATGGTGGCCGC
GGTCGTCGTGGTCGTGGTCGTGGTGGTGGTCTTGAGGGTGGTCCTGCACGAGGCGCTGGA
AGaGGTGGAAGAGGTGTAAGAGGAGGGAGAGGAGGAGGAGTAGGAGGAATAGTAGACTGG
GGGCCTGCCGGTGGCAGGTTTCGTGGATcTACTAGCTCTGCtCCTTCTGTTCAAACACGG
ATAGAGCCACAGAATATTTCTACTGAAGAACTTCCTGAGCCTATTGTTCCAGCAATGCAA
TCATTAGCAATTTCTACATCTTCAGCTCCTGAAGATTCAGGCAAACGCGTTCCAATTAAA
CGACCTGACAATGGTGGGAAAAATTGTGACCGGCCTGTACCGCTCCGTGTTAATCATTTT
CTGGTTCGTTATGACCCCGAGAGAATCATAAGGCACTATGATGTTAATGTCAAACCGGAT
TTGCGATCTGAAGATGGTCGGGCTGTGAAGTTGCCGAAGACTGTTCTGTCTATGATTCGC
AACAAGTTATTCTCAGATGATCCTGACAACTTTCCCTTGTTAAAGTCTGCATATGATGGT
GAGAAAAACATTTTCAGTGCAGAGCCACTACCCACTGGAACATTTAAGGTGGGATTATCG
AATGAGGAAGGGATAAAGATCCGATACTTCACAGTTGAAGTGCAGCTCGTGAATGAGCTT
AAATGTAGCAAGTTGGGTGATTATATAAGGGGCGAAATTTTGTCCATTCCTCGTGATGTG
CTACAAGCTTTGGATGTGATCATGAAGGAGAATCCAACAAGGAACATGATTTTTGCTAGT
CGAAGTTTTCATCCTATCGAACCTGATACACAAGTTGACCTTCACCGTGGCATCATTGCT
TCTAGAGGGATTAAACATAGCTTGAAGCCAACCTTCCAGGGTCTAGCCTTGTGTATGGAC
TATTCCGTTGTGCCATttCGCAAGCAAATGCCCGTTATAGATTATCTACAGGAACACATT
CCTGGCTTCAATCCAAACAATTTCAGAAGATTCAAGAAGCAAGTCAAGGAAGCTTTGGAG
GGATTGAATGTTACTGTGACTCACAGAACTACGAATCAGAAATACAAAATTGCTGGATTA
ACCGATCAGAACACACGGGATATTTCATTTGACGTGGAAAACCCGAGTGACCAGAACTCT
CAAGGGAAAGTTAACCTTGTTTCTTATTTTAGAAAAAAaTACAACAAGGATATTATCCAC
ATGGATATACCATGCTTAGCTTTGGGGAAAAGCAACAGGAAAAATTACGTTCCTATGGAG
TTCTGCATCATAGCTGGGTGGCAGAGATATGCAAAGGAGCTTTTGGACGTAAATCAATTA
GGAGAGTTAAGGAAGATTTCATTAGTTGCACCAAAGGATAGACAAAGAACAATACGTGAC
ATGGTAGAAAATAGAGTTGGACCCTGCAGTGGAGATATTATTCAAAATTTTGGAATCAGT
GTGGATGTGGAAATGACAACACTTATTGGTCGGGTTATTAAGCCACCAGAGTTGAAGCTT
GACACTAATCTGAGAAGAAGCTTGGTGATTACTAGCGAACCAATCAGGGAATGGGGAATA
CTTTTCTTTGGCGAGTATAGTCTTGAAAATTTCGCTCCTGAGCTGATCTCTCGCAGCCAA
AGAATGAACATCCGCATGGACCAGCCTCGTTTAAACGAACTTCATCCAAGGAAATTGTTA
CTTGATGTTCAGAAACTCGAAGAACTGCTTGAAAGAATTAAGAGTGCACGTTTACAGATT
CTTGTTTGTGTGTTGCCTAAGGAGCCTAAGGAGCCGAAGGAGCCTAAGGAGGGTCCTGCT
GGTTACAATAATCTCAAGTGGATAAGTGAGAAAAAAGTTGGCATCCTAACTCAATGTTGT
TTAGCCAAAAATTGCAGTAGACCTAAAGATCAGTTTCTCGCAAATCTTGCTCTAAACATC
AATGCGAAGCTTGGGGGCAGTAATGTAGAGCTCATCAAGCAGCCCAATTTCTTGCAAATT
AAGGGCCATTTTATGTTTGTTGGTGCTGATGTTAATCATCCTCGCTCTTACAACACAACA
AGTCCTTCAATTGCAGCTGTTGTCGGCACCATGAATTGGCCAGCAGCAAATAAATATAGT
GCACGGATTTGTCCTcAGGATCATAGGACAGAGAAGATTCTGAAGTTTGGAAGCATGTGC
ATGGAGCTTGTAAATACATATACCAGCCTAAATCAGGCAATGCCAAAGAATATTATCCTA
TTCCGTGATGGTGTAAGTGATGGGCAGTTTGATATGGTTCTGAATGAAGAACTCACGGAT
CTCAAGATGACATTTCAAGCATCGAATTACTCCCCCAGAATTACTGTTGTTGTTGCACAG
AAGCGACACACAACTCGGATGTTTCTAGATGGCAACCAGGATGGAAATGTGCCCCCAGGA
ACTGTGGTAGACACAAAAATCACACATCCTTGTGGATTTGACTTCTACATTTGCAGCCAT
TATGGTAGTATAGGAACAAGCAAGCCAGCCCACTACCATGTGTTGTGGGATGAGAATGGT
TTTACTTCTGATGAATTGCAGGAGCTTATTCATGGCATGTGCTTCACCTGTGCCCAGTGC
ACCAAGTCTGTCTCACTGGtCCCTCCAGTGTTGTATGCCGACCGTGCTGCTTATAGAGGT
CGGGTTTACCATGATGCAATGGAGGCATATCAGTCATCAGCACCACCACCTTCAACATCA
TCAACGCCTTCCTTTGATGAGCAGCTGTACAAGTTGCACCCTGACCTGGAAAACTCCAtG
TTTTTCATTTGA
>Manihot-esculenta__cassava13489.valid.m1
ATGTCTCGCCGTGGTGGTGGTCGCCGGCAGGATTTTCGCCGACACCAGCAATCATCCTCT
CAATTTCCGTCGTTCCAGCGTGGTGGTGGCGGTAGAGGTGGAAGAGATGGCGGTGGACGA
GCTAGCCGCGGTTTCAATCCGGCAGCAACTGTCACTCAATCTGCTCCTACAATATCTCGT
TCTTCCTCGTCGGCCTTCCCTCCTCCTACCACGTCTGTTGCTCAGCGTACGACTGCTCCT
CAATCTGTGGCTTCTTCGTCAAGAGCATCGTCGTCTCAGGCGACAGTAATGAGTTCGGCG
GCAGTGGAGGAGCTGAGACGAGAGGTGGAGCAGAAGCTTACAACTGGAGATAAGGTGACC
AAGGCTGAGACGCTGCCGGCGTCGTCAAAGGCCGTTCGTTTCACTCCTAGGCCTGGCTTT
GGTACTGCCGGCGAGAAGTGTGTGGTTAAGGCCAACCATTTTTTGGTCGAAATAGCTGAT

AGAGATCTCTGTCATTATGACGTAACTGTGACTCCCGAAGTAACCTCAAAGAAATTAAAC
AGGGTTATAATATCTCAGCTTGTTAGAATGTATCGTGAGTCACATTTGGGCAACCGACTG
CCAGCTTATGATGGCAGGAAAAGCCTCTATACGGCAGGACCATTGCCTTTTGAATCTAAG
GAATTTGTCGTTAAGCTGGTTGAGGAGAATAATGGTGCCGTGTCTTCTGCTTCGACAAGG
AGGGAACGGCAATTTAAAGTGGCTATCAAATTTGCTTCTAAGGCAAACATTCATCACCTG
CGACAATTTTTAAGTGGTAGACAATTGGATGCCCCACAAGAAACCATACAAGTTCTTGAT
ATTGTTCTTAGGGAATCACCATCTGAAAAGTATACAACTGTCGGGAGGTCATTCTTCTCA
ACTAATTTGGGGCCAAGGGGTGAGCTTGGTGATGGTATAGAATATTGGAGAGGATACTAT
CAAAGCCTTCGCCCAGCCCAGATGGGACTGTCTTTTAATATTGATGTGTCAGCCAGATCC
TTCTTTGAGCCAATTATGGTGACTGACTTTCTAGCTAAATACTTTAGGTTGAGAGACATG
TCAAGTCCTCTGTTGGAACAAGATCGTATTAAGGTGAAAAGGGCCTTAAGAGGAGTAAAA
GTAGAGCTAAGTCATTGGGATTATGCTAAAAGCTGCAAGATCATTGATTTATCCAATCAG
CCCCTGAATCAAATATTCTTCACTTTTGGAGATAAGAGTGTGTCAGTGGTTCAGTATTTC
CGTGATCGATACAATATTGGTCTTAAATATACAACATTGCCTGCTATTCAAGCTGGGAGT
GATTCAAAGCCAATTTTCTTGCCTATGGAGGTTTGTAGAATTGTTGAAGGACAGAGATAC
TCAATGAAATTGAATGATAGGCAAGTAACTGAATTGTTAAAAGCAACATGTCAACGCCCT
TGTGCTCGGGAAGATAGCATTAAGCAGATTGTTATGCGGAATGATTACAGCAGTGATAAG
CTTGTGaGgAATGAATTTGGAATTCAAGTTAAGGAAGAGCTTACTTTCATTGATGCTCGA
GTTTTACCTCCTCCGATGCTTAAATATCATGACACTGGCGGTGAACCAAGGATTGAACCC
CTCTTAGGACAATGGAATATGAAGAACAAGAAAATGGTGAATGGTGGAAGAGTGGAATTC
TGGACCTGtGTTAACTTCTCTTTGAAGGTCAACCAAAATTTGCCTGTTGAATTTTGTAGG
CAATTAATTGAGATGTGTGTTAGCAAAGGAATGGGATTCAACCCCAACCCCGTTCTTCCA
ATACACTCGGCTCATCCCAACCAAATAGAAAGGGCTCTTGCTGATGTCCACGAGCAGTGT
ACTGCAAAACTTTCTAATGAGAAGAATCAGCTTCAGTTGTTGATAATCATTCTACCTGAT
GTCATGGGTTCATATGGGAAAATTAAAAGGATATGCGAAACAGAATTGGGTATTGTATCT
CAGTGCTGTCAACCAAGTCAGGCTGCAAAGCTACGTAGACCGTATTTTGAAAATGTTTCC
CTCAAAATCAATGTTAAGGTTGGTGGTCGAAATACTGTGTTAAATGATGCTATTCAAAGG
AGTATTCCTCTAGTGACTGATATTCCTACCATTATTTTTGGTGCTGATGTAACTCATCCG
CCTCCTGGGGAGGATACTAATCCGTCAATAGCAGCAGTAGTGGCTTCAATGGACTGGCCA
GAGGTAACAAAGTATAGAGGAAATGTCTCTGCACAGGCTCATCGTGAAGAAATTATCCAG
GATCTCTATAAATCATATCACGATCCGGTTAGAGGATTAATTCATTCCGGAATGATCAGG
GAATTATGTATAGCATTTAGGAGAGCAACTGGGCATAAACCCAACAGGATGATATTCTAC
AGAGATGGTGTTAGTGAGGGCCAATTTAGCCAGGTTTTGCTACATGAGATGGATGCCATA
CGAAAGGCTTGTTGCTCACTTGAGGAGGGATACCTTCCACGTGTTACCTTCATTGTGGTG
CAGAAAAGGCATCATACACGGCTTTTTCCTGTTCAGGGTGGTGAGACAGATCACAGTGGC
AATATTCGACCAGGCACAGTTATTGATACCAAGATTTGCCACCAGAATGAATTTGATTTC
TACCTCAACAGCCATGCTGGAATTCAGGGAACAAGCAGGCCTGCACACTATCATGTGTTG
TATGATGAGAATTGTTTCACAGCTGATAAGTTGCAAGTTCTGACTAATAACATGTGTTAC
ACGTATGCAAGGTGTACTCGCTCTGTTTCCGTAGTTCCTCCTGCCTATTATGCCCACTTA
GCTGCTTTTCGGGCTCGATATTACATTGAGGGGGAAACATTGGATGGTGGACCTTCAGGT
GGTAGGAGTACAACTGGGAGGAGCAGGGAAGTTCAGCCGCTGCCTGTGATCAAAGACAAT
GTTAAAGATGTTATGTTTTATTGCTGA
>Manihot-esculenta__cassava2314.valid.m1
ATGGAGGACTCAGATGAATCCAATGGTGGCAACAAATGCACCACCAAGGCTAGAACTTTG
AGAGCCAGGGCTAAGACCAACTCCACCCATAAGCATGAGTATCAATATCAATATCAGCAT
CAGCTCTTTCAATATTCAAACCAGTATGGGTTCTTTAATCACAACCAAATCCAATACCCA
AGTTACTACCCAGCTCTTCTTCCTCTGCCTCCTCCAATACCTATTCAACTTGCTCTGACC
CCACCTTTGCTTCAAAATCATAGCTTTAGATCAAAAACCCATTTGCAGAAACCTTCTTGT
AATCTAAATAATCATCCTCTTCCTACCTCTTCTGTTAGTCAAGGACCTGTTGTCACAATC
TCATCAGCTGCAAGGAGACCAGATTCTGGTGGTGTTGAAGGATCAGTTGTTACTCTTGTG
GCCAACCATTTTCTTGTCCAATTCAATTCGTCGCAGAGAATTTTCCATTACAATGTAGAA
ATTTCTCCAAATCCTTCCAAGGAAGTTGCCAGGATGATCAAAGAAAAACTGGTCCAAGAC
AATTCAGCTGTGTTCTCTGGAACTTTTCCAGCCTATGATGGCCGGAAGAATCTTTACAGC
CCAGTTGAATTCCAAAATGATAGGCTTGAATTCTACATGAGCCTCCCAATCCCAAGTAGC
AAATCATCATTGCCTCTTGGAGAACTAAATGACTTTCAAGAGAAGCATCTGCAGCTCAAA
CTATTCCGGATAAACATCAAGTTGGCGTCGAAGTTGGACGGGAAGGAACTGAGCCGGTAC
TTGAGCAAAGAAGGTGATGATTGGATCCCACTTCCTCAGGATTATCTACATGCTTTGGAT
GTTGTTTTGCGCGAGAGTCCGATGGAGAAATGCATACCTGTAGGGAGATCATTCTATTCA
AGTTTAATGGGCGGAACAAAGGAAATTGGAGGAGGAGCTGTTGGATTGAGAGGATTCTTT
CAAAGTCTTAGACCCACACAACAAGGACTAGCTCTCAATGTAGATTTCTCCGTGACCGCT
TTTCATGAAAGTATCGGAGTGATCCCCTACCTGCAAAAGCGTCTCAAGTTTCTGAGGGAC
CTTCCTCAAAACAAAACAAGGAGTTTGATTATTGAAGAAAGGAAAGAAGTGGAGATAACT

TTGAAGAATATCAGGGTCTTTGTTTGCCACAGAGAAACTGTTCAGAGATACCGGGTTTAT
GGCTTGACTGAAGAAGCTACGGATAATATCTGGTTCGCCGACAGGGACGGGAAGAAACTG
AGGCTGGTGAGTTACTTCAAGGATCACTACAATTATGACATAAAATTCAGGAACTTGCCA
TGTTTGCAGATTAGTAGAAGTAAACCATGTTATCTTCCCATGGAGCTTTGTATGATTTGT
GAAGGCCAGAAGTTTCTTGGGAAACTGTCCGATGATCAGACAGCAAAAATTCtcAAGATG
GGCTGCCAAAGACCTAAAGAACGAAAAACCATTATTAATGAGGTCATGAGaGGATCTGTT
GGACCAACAAGTGGCAAGCAGAGCAGAGAATTCAAACTCAATGTTTCAAGaGAAATGACA
AGACTGAACGGGAGGATCCTGCAGCCTCCGAAATTAAGACTTGGTGATGGTGGACTGGTA
AGAGATCTGACTCCTTCTCGCCATGATCGGCAATGGAACCTTGTGGACAGCCATGTCTTT
GAAGGAACAAGAATAGAAAGGTGGGCACTAATGAGTTTTGGAGGCACCCTTGATCAGAAG
TCCAACATTCCAAAATTTATAAACCAACTATCACAAAGGTGTGAACAGTTAGGCATTTTt
CTTACTAAAAaCACAATAATTAGCCCACAATACGAGTCGACTCAAGTGCTTAACAATGTC
GCCCTTCTGGAATCGAAACTGAAGAAAATCCAGAAGGCTGCCGCCAACAATCTCCAGCTG
CTTATTTGTATAATGGAGAAGAGACACAAAGGATATGCAGATTTGAAGCGAATAGCAGAA
ACAAATGTTGGTGTTGTAAGCCAGTGCTGCTTGTTTCCGAACCTCGGCAAGTTGAATCCA
CAATTTCTAGCTAATTTGGCTCTCAAGATCAATGCTAAAGTTGGAGGATGCACAGTCGCT
TTGTACAATTCGTTACCCTCGCAGATTCCACGCCTATTTCATTCTGATGAACCTGTGATC
TTCATGGGAGCTGATGTAACTCATCCTCACCCTCTTGATGACTTCAGTCCATCAGTTGCT
GCTGTTGTTGGTAGCATGAACTGGCCAGAAACAAACAAGTATGCATCACGAATGAGGTCG
CAAACGCATCGACAAGAAATTATTCAGGACCTTGGTGCAATGGTGAAAGAATTGCTAGAT
GAATTTTATCAAGAAGCAAGAAAACTTCCCAAGAGGATAATATTCTTCAGGGATGGAGTA
AGCGAAACCCAGTTCTACAAGGTGCTTCAAGAGGAGTTGCGGGCGATTCAAGAAGCATGT
TCGAGATACCCAGGTTATAGACCTCTCATTACATTTGCAGTTGTCCAAAAGAGACACCAT
ACAAGGTTGTTTCACTGTGAAACAGATGAATCTTCCATTCAAAACCAGTTTTACGGCGAA
AACATACCCCCAGGAACAGTTGTGGATACTGTTATTACTCATCCAAAGGAATTTGATTTT
TATCTATGCAGCCATTGGGGAGTAAAAGGAACAAGTAGGCCAACTCATTACCATGTCTTG
TGGGATGAGAACCAATTCACTTCTGATGAATTACAGAAGCTAGTTTACAATCTCTGCTAC
ACATTTGTAAGGTGCACAAAGCCAATTTCTTTGGTGCCTCCTGCTTACTATGCACACTTA
GCTGCATACAGGGGCAGGCTTTACCTCGAACGATCGGAGTCAATGGCTTCCATGAGAAAT
GGTTCTAAGATCTCAAGAGCTGCTCCACCAAAGGCAACACCTCTGCCAAAGCTCAATGAG
AATGTAAAGAATCTCATGTTCTATTGCTGA
>Manihot-esculenta__cassava23918.valid.m1
ATGTCTCGACGTGGCGGTGGCCGCCGGCCGGATTCTCGCTCGGACCAGCAATCATCATCC
CCATCGCCGTCATTTCAGCGCGGTGGTGGAGGCGGTAGGGGTGGTAGAGGTGGAAGAGGT
GGCCGAGGACGTGAAGGACCTGGCCATAGTCCGCTTCCAACTTTCACTCAATCTGCTTCC
ACGCCGGCTACGTCCTTCTCTCCAATATCTCGTCCGCCGGTGCCTGCTTACTCTCCTGAA
GCTCAAACCCCGTCGGTTGCTCCGTCGCCTCCTCAAGCTGTGGCTTCTTCGTCGCGAGCA
CCGCCGTCTAAGGCGGCAGCTTCGAGTTCGACGGCGGAGGAGATTCGACGAGAGGTCGAA
CAAAAGCTGTCGCTTGGAGATCAGGCGACCAAGGCTGTTCGTTTCCCGTCTAGGCCTGGG
TTTGGGACTGTCGGCATGAAGTGCGTGGTTAAGGCGAACCAtTTTTTGGtCCAAGTCGCT
GACAGGGATCTCTGCCAATATGATGTCACCATAACTCCTGAAGTAACTTCAAAGAAGGTA
AACAGGGATATAATATCTGAGCTTGTTCGAATGTATCGTGCATCACATTTGGGCAACCGA
ATGCCTGCTTATGATGGCAGGAAAAACCTCTATACAGCAGGGCCTTTGCCTTTTGAATCT
AAAGAATTTATTGTCAAGCTGGTTGAGAAGAATAATGCAGCTGGGTCTTCTGGTTCATCC
AAGAGGGAAAGACAATTTAAAGTGGCAATCAAATTTGCGTCTAAGGCGGACCTTCATCAT
TTGCAACAATTTTTGTATGGCAGACAAGCAGATGCTCCGCAAGAAACCGTACAAGTTCTT
GATATTGTACTCAGGGCATCACCATCAGAAAAGTATACAACTGTTGGGAGGTCATTTTTC
TCACCTGATCTGGGGCCAAGGGGTGAGCTTGGTGATGGTATAGAATATTGGAGAGGATAT
TATCAAAGCCTTCGCCCAACCCAGATGGGACTGTCTTTTAATGTTGATGTGTCAGCCAGA
TCCTTCTTTGAGCCAATTATGGTGACTGACTTTGTAGCTAAGTACTTTAGGTTGAGAGAT
TTGTCAAGGCCTCTGTCTGAACAAGATCGCATAAAGGTGAAGAAGTCTTTAAAAGGAGTA
AAAGTAGAGCTACATCACAGGGAGTATCCTAAAAGCTACAAGATCACTAGCTTATCTAAT
CAGCCAATGAATCAAACTTTCTTCACTCTCGATGATAAGAATTCAAAAGTATCAGTTGTT
CAATACTTTCGTGAGAGATACAACATTATGCTCAAATATACGTCCTTGCCTGCCCCTTCAA
GCCGGCAGTGATTCAAAGCCCGTTTATTTGCCTATGGAGCTTTGTAGGATTGTTGAAGGA
CAGAGGTACACAAAGAAATTGAACGAAAGGCAAGTTACTCAGCTGTTAAGGGCAACTTGT
CAACGCCCTCATGATCGGGAAAATAGCATTAAGCAGATGGTTAGGCGGAATAATTACAGT
AGAGATGAGCTTGTGGCAAATGAATTTGGAATTCAAGTTAAAGAAGAACTTGCACTCGTT
GATGCTCGAGTTTTGCCGCCTCCAATGCTTAAGTATCATGAAACTGGGGGTGAATCAAGG
GTTGATCCACGCTTAGGCCAATGGAATATGATAAACAAGAAAATGGTGAATGGAGGGAGA
GTGGATTTTTGGACATGTGTGAACTTCTCTTCGCAAATCCATCCAAATTTGCCTCGTGAC
TTTTGTCAGCAGTTAGTTCAGATGTGTGTCAGCAAAGGAATGGGATTCAATCCAAACCCT

ATTCTGCCAATACAATCAGCTCATCCTAGCCAGATTGAGAGGGCTCTGGCTGATGTTCAC
AAGCAGTGTACTGCAAAACTTGCAAATGAGAAGAAACAGCTTCAGTTGTTGATAATCATT
TTACCAGATTTCACTGGGTCTTATGGGAAGATCAAACGAATCTGTGAAACAGAATTTGGA
ATTGTCTCTCAGTGCTGTCAACCAAAGCAGGCAGCAAAGCTAAGTAAACAGTATTTTGAA
AATGTTGCCCTCAAAATCAATGTTAAGGTTGGCGGTAGAAATACTGTGTTGAATGATGCT
ATTCAAAGGAGAATTCCCCTTGTGACTGATCTTCCTACAATTATTTTTGGTGCTGATGTA
ACTCATCCAGCTCCTGGGGAGGACTCTAATCCATCGATAGCTGCAGTAGTAGCTTCAATG
GACTGGCCAGAGGTAACAAAGTATAGAGGCCTTGTCTCTGCACAAGCTCATCGAGAAGAA
ATTATCCAGGATCTCTATAAATCATATCATAATCCAGATAAGGGATTAGTTCATTTAGGA
ATGATAAGGGAATTACTAATATCCTTCAGAAAATCAACTGGGCATAAACCTGGCAGGATT
ATATTCTACAGAGATGGTGTTAGTGAGGGCCAATTTAGCCAGGTCTTGCTACATGAGATG
GATGCCATACGAAAGGCTTGTTCTTCACTTGAGGAGGGATACCTTCCACGTGTTACCTTT
GTTGTAGTTCAGAAGAGACATCATACACGACTTTTTCCTGTTGATCGTGGTCAGACAGAC
AGGAGTGGCAATATTCTGCCAGGCACTGTTATTGATACTAAGATATGCCACCCGAAGGAG
TTCGATTTCTACCTCAACAGCCATGCTGGAATTCAGGGAACAAGCAGACCTACACACTAC
CATGTGTTGTATGATGAGAATGGGTTCACTGCCGATGGGTTGCAAATTCTGACTAACAAT
CTTTGTTATACGTATGCGAGGTGTACCCGTTCTGTCTCAATAGTCCCTCCTGCCTATTAT
GCGCATTTAGCTGCTTTTCGGGCTCGATATTACATAGAGGGCGAAACATCAGATAGTGGA
TCTTCAAGTGGTAGGAATACAATAGGGAGGAGTAAGGAGGTCCAGCCTCTTCCTGTGATC
AAAGATAATGTGAAAGATGTTATGtTTATTGCTGA
>Manihot-esculenta__cassava27530.m1
ATGGATTCCAAGAATATGTTATTATCAGAAGCTAAAGGAACTCCTCCTGCTTCATGCAAA
AGCCTCTTCATGTTTCATCGCAGGCCTGGGTATGGTCAGCTGGGGACGAAGTGCATGGTT
AAAGCCAATCATTTTCTCGCTGAGATACCCGACACAGACTTGAGTCACTACAGTGTAGAA
ATAACCCCAGAAGTTACATCTCGTAAACTGAGCAAAGCCATCATGACCCAGTTGGTAAAA
CTGCACAGAGGAACTGATTTAGGGATGAGGCTACCTGTTTATGATGGAGGAAGAAACCTT
TACACAGCAAGATCATTGCCTTTTATCTCCAAAGACTTCACTGTAACTTTGGTTCACGAA
GATGAGGCAACAGGCAATTTCAAGAAAAGAGAATTCAAAGTGACAATAAAGTTTGAAGCA
CTTGCTAGTATGCTGCAATTAAGGGAGCTTTTTTCTGGAAAACCAGTTGATACTCCTCAA
GAAGCGATTACTATTATTGACATCATACTAAGGGAATTTGCTGCTCAAAGGTACGTATCA
ATTGGAAGGTCCTTCTATTCTCCTGATATAAAGAAACCACAGAAGCTGGATGGAGGTTTA
GAATCATGGCGGGGCTTCTACCAAAGTATAAGACCAACTCAAATGGGGCTATCACTGAAT
ATTGATATGTCGGCTACGGCTTTCATTGAACCACTCCTTGTTATTGATTTTGTTGCTCAA
ATTTTGTGCAAGGACGTGTATAAAAGGCCATTATCAGATGCAGATCGCGTTAAGGTCAAG
AAAGCTCTTAGAGGTGTCAAAGTTGAAGTCACTCACAGAGGAAATGTACGAAGAAAATAC
CGAATTTCCGGGTTGACAACACAGCCTACAAGAGAGCTAATCTTCCCACTCGATGGGCAT
ATGAACATGAAATCAGTTGTTGAGTACTTTCAAGAGATGTACGGCTATACCATTCAGTAT
CCTCATCTACCTTGCCTGCAAGTAGGAAACCAGAGGAAGGTGAACTATTTGCCGATGGAG
GCTTGCAAGATAGTCCAGGGACAAAGATATACAAAAGGGCTAAATGAGAAGCAGATCACT
TCTTTGCTAAAAGTTTCATGTCAAAGACCCCGTGATCAAGAATCAGATATTTTACAGACA
ATTCAACAAAATGGGTACAACCAAGATCCATATGCGAAAGAGTTTGGCATCAGCATAGAT
AGCAAGCTTGCATCTATTGAGGCTCGAGTTCTACCGGCTCCATGGCTGAAGTACAATGAT
ACTGGAAAAGTAAAAGAATATCTTCCTCAAGTTGGCCAGTGGAATATGATGAACAAGAAA
GTGATAAATGGAAGTACCGTAAGATATTGGGCTTGCATCAACTTCTCACGAAGTGTCCAA
GAAACCACAGCTAGAGGCTTTTGCCAGCAGTTGGGTCAGATGTGCCAAATCTCTGGCATG
GATTTTAACTGCGAACCAGTACTTCCAATATATTCAGCAAGACCTGATCAGGTAAAGAAG
GCCTTGAAATATGTTTATCATGCAGCTGCAAAGAAACTTGAAGGGAAAGAACTGGAGTTG
CTTATTGCAATTCTTCCTGACAGCAATGGCTCATTGTATGGTGATTTAAAAAGAATTTGT
GAAACTGATCTCGGGTTGATTTCCCAGTGCTGCCTTACCAAACATGTCTTCAAAATTAAC
AGACAGTACTTGGCAAATGTGTCACTCAAAATCAACGTTAAGATGGGAGGAAGAAATACT
GTGCTTTTGGATGCTTTGAGTTGGAGAATTCCCTTGGTCAGCGACATCCCAACAATAATT
TTTGGGGCTGATGTAACACATCCAGAGTCAGGAGAGGACACTAGTCCATCTATAGCTGCT
GTTGTAGCCTCCCAAGACTGGCCTGAAGTTACAAAATATGCCGGATTAGTATGCGCTCAG
CCTCACCGGCAAGAACTTATTCAAGATTTATTCAAAACATGGCAGGACCCTCAACAGGGA
ACTGTCTCTGGAGGCATGATCAGGGAGCTCTTACTTTCATTTAAGAAAGCCACTGGGCAA
AAGCCATTGAGGATAATATTTTACAGGGATGGTGTAAGCGAAGGACAGTTCTACCAGGTT
CTGTTGTATGAACTTGACGCGATTCGAAAGGCTTGTGCATCGCTAGAACCCAGTTACCAA
CCTCCAGTGACATTTGTGGTTGTCCAAAAGCGGCACCATACTAGACTCTTCACGAGCAAT
CACAACGACAGAAATAGCACTGACAAGAGTGGAAATATTTTACCTGGTACTGTAGTGGAT
TCTAAAATATGTCATCCGACTGAATTCGACTTCTATTTATGTAGTCATGCAGGAATCCAG
GGCACCAGCCGGCCTGCCCATTATCATGTGCTGTGGGATGAGAACAACTTCACTGCAGAT
GAGATTCAATCTCTGACAAACAACCTCTGCTATACGTATGCAAGGTGCACACGCTCGGTT

TCTGTAGTTCCTCCTGCATATTATGCTCATTTGGCAGCTTATCGAGCTCGGTTTTACATG
GAACCTGAGGACGCGCCGGAGAATCCTAAGATGCGTTGCACACGCGCCGCCAATGGATCA
TGCGTCCGCCCCTTGCCTGCATTGAAAGATAAGGTGAAGAGCGTCATGTTTtACTGCTAA
>Manihot-esculenta__cassava35367.valid.m1
ATGCCTATAAGGCAAATGAAAGAGAGCTCAGAGCAGCACTTAGTGATTAAAACCCACTTG
CAGAACACTATGAACCATCCTCAAAAACACCCTAAAACTTCCCAAAATGGCAAGGGACCT
CCACCTCAGGAAACCCACAACaGCAAAACTCAGAACCAGACCTTACCTCCAGCaAAAAAC
AGAGGAAGGAGAAGAGGCAGAGGTGGTAGAAAGTCTGACCAAGGAGATGCCGGTATGAGA
CCCAGTTCAAGGCCGTGtACTTTGGCACATAAGCCTGTGAATCCAGCAGGTGATCTCTTG
GCAAGTGCTCCAAATGGTTCTATTGGAAATGCTGGAAGTACTTGTGAAATAGAGATGGGT
TTGGGGTTCCCTACTTCTAGCAAGTCTTTGAGTTACGCTGCTAGGCCTGGTTATGGTCAA
ATGGGGACAAAGTGTGTTGTTAAGGCCAACCATTTCTTCGCAGAATTACCAGACAAGGAC
TTGAACCAATATGATGTTACCATAACTCCTGAAGTGGCATCAAGAACTACAAACAGAGCT
ATCATGGCAGAGCTTGTGAGGCTTTACAAAGAATCTGACTTAGGAATGAGACTTCCTGCT
TACGATGGCAGAAAGAGTCTGTACACAGCTGGGCAACTTCCCTTTGCTTGGAAGGAATTC
ACGATTAAGCTAGTTGATGAGGAGGATGGAATCAATGGCCCTAAGAGAGAGAGAGAGTAC
AAAGTGGTGATCAAGTTTGTAGCAAGGGCCAATATGCATCATTTGGGTCAATTTTTAGCT
GGTAAACGTGCTGATGCTCCACAGGAAGCTTTACAAATTCTTGACATTGTATTGAGAGAG
CTCTCAACAAAGAGGTACTGTCCCATAGGAAGATCCTTCTTTTCACCAGATATTAGAGCA
CCACAACGGCTTGGTGATGGCTTGGAGTCTTGGTGTGGGTTTTACCAGAGTATAAGGCCT
ACACAAATGGGCCTCTCTTTAAATATTGATATGGCTTCTGCTGCATTCATCGAGCCTCTC
CCTGTAATTGAGTTTGTTGCCCAGCTTCTAGGCAAGGATGTGTTATCCAGACCATTGTCT
GATTCTGATAGAATCAAGATTAAAAAGGCCCTCAGGGGAGTGAAAGTTGAAGTAACTCAT
AGAGGACATGTACGGAGAAAGTATCGTGTCTCAGGATTGACATCTCAACCTACAAGAGAA
CTTGTATTTCCTGTTGATGATAACTCCACTATGAAGTCAGTAGTTGAGTACTTCCAAGAG
ATGTATGGCTTCACCATTCAGCATTCTCATCTCCCTTGCCTTCAAGTAGGAAACCAGAAG
AAGGCAAACTATCTCCCGATGGAGGCATGCAAAATTGTAGAAGGTCAACGATACACTAAA
AGGTTGAATGAGAGACAAATTACTGCACTGTTGaAAGTTACATGCCAAAGACCGAGGGAT
CGAGAAAATGACATTTTGCAGACAGTTCAGCATAATGCTTATGATCAAGATCCTTATGCA
AAGGAGTTTGGGATCAAAATCAGTGAAAAGCTAGCTTCTGTTGAAGCTCGAATTCTACCT
GCTCCTTGGCTGAAATATCATGAAACAGGAAGGGAAAAGGACtGTTTGCCACAAGTTGGG
CAATGGAACATGATGAACAAGAAAATGATCAATGGGATGACCGTAAGCCGATGGGCTTGT
ATAAACTTTTCAAGGAGTGTTCAAGAAAGTGTTGCTCGTGGATTTTGCAATGAACTTGCT
CAGATGTGCCAAGTATCTGGCATGGAATTCAATCCAGAGCCTGTTATTCCAATCTACAAT
GCCAGGCCAGATCAAGTAGAGAAAGCCTTGAAGCATGTATATCATTCTTCTATGAGCAAA
ACCAAAGGAAAAGAACTAGAGCTTTTATTAGCTATTCTGCCCGACAACAACGGATCCCTT
TATGGTGACCTTAAGCGGATATGTGAAACTGATCTTGGTTTAATATCACAATGCTGTCTT
ACAAAACATGTCTTCAAGATCAGCAAGCAGTATTTGGCTAATGTGTCACTGAAGATTAAT
GTTAAGATGGGTGGTAGAAACACTGTCCTTTTAGACGCCATTAGCTGCAGGATACCATTA
GTTAGTGACATACCAACCATTATATTTGGAGCAGATGTGACTCACCCAGAGAATGGGGAG
GACTCAAGCCCTTCAATTGCAGCAGTAGTAGCTTCTCAAGACTGGCCTGAAGTGACAAAA
TATGCTGGATTAGTTTGTGCTCAAGCTCACAGACAAGAACTCATACAAGACTTGTATAAA
ACATGGCAAGATCCAGTTCGTGGAACTGTTAGTGGGGGCATGATCAGAGATCTTCTGGTT
TCCTTTAGGAAGGCAACAGGACAGAAGCCGCTGAGGATTATATTCTACAGGGATGGTGTT
AGCGAAGGGCAATTCTATCAAGTTCTGCTTTATGAATTAGATGCAATCCGGAAGGCTTGT
GCCTCTTTAGAACCAAACTATCAACCACCTGTGACTTTCATAGTTGTACAGAAACGGCAT
CaTACTCGATTATTTGCTAACAACCACAGGGaTAGGAGTAGCACGGaCAAAAGTGGGAAC
ATATTACCTGGTACTGTGGTTGACTCTAAAATCTGTCACCCTACAGAATTTGATTTTTAT
CTCTGTAGCCATGCTGGTATTCAGGGAACAAGTCGGCCTGCTCACTACCATGTTTTGTGG
GATGAAAACAACTTCACTGCTGATGGAATCCAGTCATTGACAAACAATCTATGCTATACA
TATGCAAGATGCACTCGCTCTGTTTCTGTCGTTCCTCCAGCATATTATGCACATTTGGCT
GCTTTTCGCGCTCGATTTTACATGGAGCCAGAAATGCAGGAAAATGGTTCGTCAGGAGTT
GGTTCTGGGCATGGTAGCAAGGGAACACGGGTAGGAGAATCTGGAGTACGGCCATTGCCT
GCCTTGAAGGAAAACGTGAAGAGAGTAATGTTTTATTGTTGA
>Manihot-esculenta__cassava43463.valid.m1
ATGGATTCGATTGAGCCGGAGAAAAACGGGTCACAGCAGGATTTGCCTCCTCCTCCACCG
GTTGTTCCACCGAATTTAGTTCCGCTCAAAGTAGAGACTGAGCCGGTGAAGAAGAAGCCT
GCTAGGGTTCCGATAGCTAGGCGTGGGTTTGGATCTAAAGGACAGAAGATTTCTCTTCTT
ACCAACCACTTTAAAGTGAATGTGACCAATGTTGAGGGTTACTTTTACCATTATAGTGTG
TCTCTCTCATATGAAGATGGTCGTCCTGTTGATGGAAAAGGCGTTGGGAGGAAGGTCATC
GATCGAGTTCAGGAGACTTACGATTCTGAGTTAGATGGAAAGAACTTTGCATATGATGGG
GAGAAGAGTTTATTCACTATTGGTTCTCTTCCTCGCAACAAGCTTGAGTTCACCGTTGTG

CTTGAGGACGTCACGTCTAACAGAAACAATGGAAATGCAAGCCCTGAAGGTCATGGTAGT
CCTAATGAGAGTGATCGGAAAAGGTTGCGGCGACCATATCATTCTAAAACGTTCAAGGTC
GAGATCAGTTTTGCTGCAAAAATACCCATGCAAGCCATTGCAAATGCGCTTCGGGGGCAG
GAGTCGGAGAACTCTCAAGAAGCCATAAGGGTTCTGGACATCATATTGCGTCAGCATGCA
GCTAAACaGGGTTGCCTCCTTGTTCGCCAGAACTTTTTTCACAATGATCCAAGGAATTTC
GCAGATGTTGGGGGTGGTGTTCTGGGTTGTAAAGGGTTTCACTCGAGTTTTAGAACCACT
CAGGGGGGCTTATCTCTGAATATTGATGTATCTACAACCATGATAATTCAGCCTGGGCCG
GTTGTGGATTTTCTAATTGCCAACCAAAATGCAAGAGACCCCTTTCAACTTGACTGGGCT
AAGGCAAAACGAACTCTTAAAAACCTGAGGATCAAAGCTAGCCCCTCGAACCAGGAGTAC
AAGATAACTGGTCTGAGTGACAAACCATGCAGAGAGCAAATGTTTCAACTTAAACAAAAA
AGGGATGGGGATGGTGAACCTCTGGAATTAACTGTTTATGACTATTTTGTTAATCATCGT
CATATAGATTTGCGCTATTCTGCTGATTTGCCTTGCATTAACGTTGGGAAGCCAAAGCGC
CCTACTTATATTCCGATTGAGCTTTGTACTTTGGTGTCCTTGCAACGATACACGAAAGCA
CTCAATACTCTCCAAAGAGCTTCCCTGGTGGAGAAATCAAGGCAAAAACCCCAAGAGAGG
ATGAGTACTTTAACAAATGCTCTTAAAAGCAGCAAATATGATGCTGAACCTATGCTACGT
TCTTGTGGCATTTCAATCAGTACTAATTTTGCTGATATTGAAGGCCGTGTTTTGCCTGCT
CCAAGGCTAAAAGTAGGCAATGGGGAGGATTTCTTTCCCCGGAATGGGCGGTGGAATTTT
AATAACAAGAAACTCGTGGAGCCTAGTAAGATAGAAAGATGGGCTGTTGTGAACTTCTCA
GCGCGATGTGATGTTCGTAGCCTTGTAAGAGACCTTACAAGATGTGCAGATATGAAAGGA
ATTCCTATGGAGGCTCCCTTTGATGTATTTGAGGAGAATCCACAGTTTAGGCGAGCTCCA
CCTACAGTTAGAGTGGAGAAAATGTTTGAGGAGATACAGTCTAAACTTCCTGGTGCCCCC
AAGTTCCTATTGTGTTTACTCCCGGaGAGGAAAAACTCAGATATATATGGCCCTTGGAAG
AAGAAAAATCTTGCTGAGTTTGGAATTGTCACCCAGTGCCTGGCTCCTCAAAGGGTCAAT
GACCAGTATCTGACTAATCTTCTGTTGAAGATCAATGCAAAGCTTGGTGGGTTAAATTCT
ATGTTATCAGTTGAACATACTCCTTCTATTCCGGTTGTTTCTAAGGTTCCTACAATCATA
CTTGGGATGGATGTATCACATGGCTCTCCTGGGCATTCTGATTGCCCGTCCATTGCTGCG
GTGGTCAGTTCCAGGAATTGGCCATTGATTTCTCGTTACAGGGCATCAGTTCGCACTCAG
TCTCCAAAGGTGGAAATGATAGACTCTCTATACAAACGAGTGTCAGAAACTGAGGATGAA
GGAATGATTAGGGAGCTTCTGTTGGATTTTTATACCAGTTCAGGTAAAAGGAAACCAGAT
CAGATCATCATATTCAGGGATGGTGTAAGTGAATCTCAATTTAATCAAGTATTGAATATT
GAACTGGATCAGATAATTGAGGCATGCAAGTTTCTTGATGAGAAATGGAACCCCAAGTTT
GTGGTGATTGTAGCTCAGAAGAACCACCATACAAAATTCTTCCAAAAGGGATCTCCTGAT
AATGTTCCACCTGGTACTGTCATAGACAATAAAGTCTGTCATCCAAGGAACAATGATTTC
TATCTCTGTGCTCATGCAGGAATGATTGGTACTACAAGACCCACACATTACCATGTTTTA
TTAGATGAGGTTGGCTTTTCAGCAGACGATCTTCAGGACCTAGTGCATTCTCTATCATAT
GTTTATCAAAGAAGTACAACTGCCATTTCTGTAGTTGCGCCAATATGCTATGCTCACCTG
GCTGCCACCCAGATGGGATCGTTCATGAAGTTCGAAGACACTTCTGAGACGTCATCGAGT
CACGGTGGTGTAACCTCTGCTGGAGCTGTTCCTGTGCCTCAGCTGCCCAAATTGCAGGAT
AAAGTATGCAATTCTATGTTCTTCTGTTGA
>Manihot-esculenta__cassava586.valid.m1
ATGCCTATAAGGCAAATGAAAGAGAGCTCAGAGCAGCACTTGGTGATAAAAACCCACTTG
CAGAACACAATGAACCAACCTCAAAAACACCATAAAACCCCTCAAAATGGCAAGGGACCG
CCATCTCAGGAAACCCACAGCAAGAGCCAGACCTCAcCCCCAACAAAAaACAGGGGAAGA
AGAAGAAACAGAGGTGGTAGAAAGTCTGATCAAGGAGATGTCTGTATGAGACCCAGTTCA
AGGCCATGTACTGTGGCGCATAAGCCCGTGAATCCAGCAGGTAATCCCTTGGCAAGAGCT
CCAAATGGGTCCGTTAGAAATGCTGGAAATATTTGTGAAATGGAGATGGGTTTGGGGTTC
CCTAcTTCTAGCAAGTCTTTGAGTTTTGCTCCTAGGCCTGGTTATGGTCAGCTGGGGACA
AAGTGTATTGTTAAGGCCAACCACTTCTTTGCAGAGTTACCAGACAAGGACTTGAACCAC
TATGATGTTACCATAACTCCTGAAGTGGCATCAAGAGCTAAAAACAGAGCTATCATGGCG
GAGCTGGTGAGGCTTTACAAAGAATCTGACTTGGGAAGGAGACTTCCTGCTTACGATGGC
AGAAAGAGTCTGTATACTGCTGGTGAACTTCCCTTTGCTTGGAAGGAGTTCGTGATTAAA
CTTGTTGATGAAGAAGATGGAATAAATGGTCCCAAGAGGGAAAGAGAGTACAAAGTGGTG
ATCAAGTTTGTAGCAAGGGCCAATATGCATCACTTGGGTCAATTTTTAGCTGGTAAACGT
GCTGATGCTCCACAGGAGGCTTTACAAATTCTTGACATTGTGCTGAGGGAGCTCTCAACA
AAGAGGTACTGCCCCATTGGAAGATCCTTCTTTTCACCGGATATTAGAGCACCACAACGA
CTTGGTGATGGCTTGGAGTCCTGGTGTGGGTTTTACCAGAGTATAAGGCCTACACAAATG
GGCCTATCCTTGAATATTGATATGGCTTCAGCTGCATTCATTGAGCCTCTCCCAGTAATT
GAGTTTGTTGCACAGCTTCTGGGCAAGGATGTATTATCAAGGCCATTATGTGATTCCGAT
AGAATCAAGATTAAGAAGGCCCTCAGAGGAGTAAAAGTTGAAGTAACTCATAGAGGGAAT
ATACGGAGAAAGTATCGTGTCTCAGGATTGACATCTCAACCTACAAGAGAACTTGTATTT
CCTGTTGATGATAACTCAACTATGAAGTCAGTAGTTGAATATTTCCAAGAGATGTATGGA
TTCACCATTCAACATACGCATCTACCTTGCCTTCAAGTAGGAAACCAGAAGAAGGCGAAC

TATCTACCTATGGAGGCTTGCAAAATTGTGGAGGGCCAACGATATACGAAAAGGTTGAAT
GAGAGACAAATTACTGCCCTGTTGAAAGTTACATGCCAAAGGCCAAGGGATCGAGAAAAT
GACATTTTGCAGACAGTTCAGCATAATGCTTATGATCATGATCCTTTTGCAAAGGAGTTT
GGGATCAAAATCAGTGAAAAGCTAGCTTCTGTTGAGGCTCGAATTCTCCCTGCTCCCTGG
CTGAAATATCATGAAACTGGAAAGGAAAAGGACTGCTTGCCACAAGTTGGCCAATGGAAC
ATGATGAATAAGAAAATGATTAATGGAATGACTGTGAGCCGATGGTCATGTATCAACTTT
TCAAGAAGTGTTCAAGAAAGTGTTGCTCGGGGGTTTTGCAATGAGCTTGCTCAGATGTGC
CAAGTATCTGGCATGGAATTCAATCCAGAGCCTGTTATTCCAATCTATAATGCCAGGCCT
GATCAAGTAGAAAAAGCTTTGAAGCATGTTTATCATGCTTCCATGAACAAAACCAAAGGa
AAAGAATTGGAGCTTTTATTAGCTATTCTACCTGACAATAATGGATCCCTATATGGGGAT
CTTAAGCGGATATGTGAAACTGATCTTGGTTTAATATCACAATGCTGTCTTACTAAACAT
GtTTTCAAAATCAGCAAGCAGTATTTGGCTAATGTATCACTGAAGATTAATGTTAAGATG
GGTGGTAGAAACACTGTCCTTTTAGATGCCATTAGCTGCAGAATACCATTGGTTAGTGAC
ATACCAACTATTATATTTGGAGCAGATGTAACTCACCCAGAGAATGGGGAGGACTCAAGC
CCCTCCATTGCAGCTGTGGTAGCTTCTCAGGACTGGCCTGAAGTGACAAAATATGCTGGA
TTGGTTTGTGCTCAAGCTCACAGACAAGAACTCATACAAGACTTGTACAAAACATGGCAA
GATCCAGTTCGTGGAACTGTTAGTGGTGGCATGATCAGAGATCTTCTAGTTTCCTTTCGG
AAGGCAACAGGACAAAAGCCACTAAGGATTATATTTTACAGGGATGGTGTTAGTGAAGGG
CAATTTTATCAAGTTTTGCTTTATGAATTGGATGCAATCCGGAAGGCTTGTGCTTCTCTA
GAACCAAACTATCAACCACCTGTGACttTCATAGTTGTACAAAAACGACACCATACTCGA
TTATTTGCTAACAACCACAGGGATAGAAGTAGCATAGATAAGAGTGGAAACATATTACCC
GGCACTGTGGTTGATTCGAAAATCTGCCATCCAACAGAGTTTGATTTTTATCTCTGCAGT
CATGCAGGTATTCAGGGGACAAGTCGGCCTGCTCACTACCATGTTCTGTGGGATGAGAAC
AACTTCACTGCTGACGGAATCCAGTCATTGACGAACAATTTATGTTACACATATGCAAGA
TGCACTCGCTCTGTTTCTGTTGTTCCTCCTGCATATTACGCACATTTAGCTGCTTTTCGT
GCCCGATTTTACACGGAGCCAGAAATGCAGGAAAATGGCTCCAGCGGTGGGGGCTCAGGA
TATGGCACCAAGGGAGCAAGAGCAGGAGAAACAGGAGTCCGGCCATTGCCTGCCTTGAAG
GAAAATGTGAAGAGAGTAATGTTTTACTGTTAA
>Manihot-esculenta__cassava7327.valid.m1
ATGGAGAGAGGAGGGTATGGTTATAGAGGAAGACGTACGGGTGGAAGAGGTCAAAGAGGA
AGAGGTCGAGGTGGTAGTGGCGGCTTTGGCAAGGAGCAACAATGGATACCATCAACTCAA
GGTGGAGGTGGAGCCCAGGGTCACATGGGGTCTAACCAGAGTGCTAGTAGCGGTGGTTCG
GCGTGGCACCCAAGTGGTGGCTCTAGTAGGGGTGGTGGAgAACGTGGAGGATCGACCTCA
ACTAGGGGTAGGGAGCACATGATTCCCGCTCCACCTCCCTCTGCTCATCGGAGTTCTGAT
GTCCCTGAATCAGGGCCTGCTGTATCTGGTCCTAGCACTCGACCTGTGAATATTTCACAA
CATTTGGCTTCCTCTTCTTCTCCTCAAGATGCAAGCAATAGATTTGTCCCAGTCAAACGG
CCAGATAAAGGTGGCACACTGGCTGTTCGAACTGCTAGCCTTCATGTTAATCATTTCCCT
GTCAGGTTCAATTCAGATAGTATCATAAGGCATTATGATATTGATGTTAAACCAGACGTG
TCTCCCAAGAATGGTCGATCTGCGAAAATATCAAAGTCTGATTTGGCAATGATCAGGAGC
AAGTTATTTACTGATGATCCTTCTCAGTTTCCCTTGTCAATGACTGCATATGATGGTGAG
AAGAACATTTTCAGTGCTGTTCCTTTGCCCACTGGAAAGATTAAGGTGGAGTTCTCTGAG
GGGGAAGACATGAAGGATCGTGCTTACATAATTAATATAAAGCTTGTGAATGAACTCAAG
CTTTGCAAGTTGAAGGATTACTTAAGTGGGAAACTGTTTTCAATCCCTCGTGACATATTG
CAAGGGATGGATGTTGTAATGAAGGAGAACCCAGCTAGGCATATGGTTTCTGTCGGCCGC
AGCTTTCACTCAGTTGAAGCTAATGAAGAAGATGACCTTGGATTTGGCATCACAGCTTCC
AGAGGGTTTCAACATAGCCTTAAGCCCACCTTCCAGGGTCTAGCCATGTGCTTGGACTAT
TCTGTCCTGGCATTTCGCAAGCGACTTCCAGTAATAAATTTTCTTATGGAGCATATTCCA
GGGTTTAACTTAAATGATTTCGGAAGCTTTAGAAAAGATGTTGAAAATGCACTGAAGGGA
CTGAAAGTTACAGTGACTCACCGTGTTACCAAACAAAAATATACTATAGCTGGACTTACA
AAGGATAATGCACTGTATCTTTCATTTTTGTCTGAAGACCCAAATGGCAAAACCCCACCT
AAGAAAGTACATCTTGTTGAATATTTCAGGCAAAAATACCAGGATATAAAATTCAAAGAC
ATTCCTTGCCTTGATTTGGGCACAAACAACCGAAAAAATTATGTACCCATGGAGTTCTGT
GTCCTAGTTGAGGGTCAGATTTATCCAAAGGAGCATTTGGATAGAAATGCAGCCCTCTTT
TTGAAGAAGATGTCGCTGGCAACTCCAAGGGATCGGCAGAGAATAATATGTGATATGGTA
TCTGCTGGAGATGGGCCTTGTGGTGGGAACATAATCCGGAATTTTGGAATGGAAGTTGAC
ATGAACATGACTTCAGTGGTGGGCCGTGTGATTGGGCCACCAGAGTTGAAACTAAGTGCT
CCCAATGGAAGGGTAATTAGAATTGCTGTTGACAAGGAGAAGTGTCACTGGAATCTTCTT
GGAAAAGGAGTGGTGGAAGGGAAACCAGCTGATCGGTGGGCTGTGCTTGACTTTAGTTCC
TCTGAACGCGGTCGTTATAAATTAAGGTCAGAGCAATTCATTCCAAAGCTTCGAGCTCGG
TGTGAGAATCTGAGAATTTCTATGAAAGAGCCTCTCTTCTACCATCCTGCTACAATGCAT
GCATTCTCAAATATTGATTTACTTCATCATCTGCTTGAAGTTGCTAATGATCGTGCATAT
AAGATTTGCGGAGGCCGGTTGCAATTTATTCTTTGTGTGATGTCTAGGAAGGATTCTGGT

TACAAGTATCTTAAGTGGATTTCTGAGACCAGAGTTGGTGTAGTGACTCAATGTTGCTTG
TCTGATCTTGCTAACAATGGGAATGACCAGTATCTTGCTAATCTTGCTCTGAAGATAAAT
GCAAAGCTTGGAGGTAGCAATGTGGAGCTCATTGATCGACTTCCATTGTTTGAAGGTGAG
GGCCATGTTATGTTTgTTGGGGCTGATGTTAATCATCCTGGCTCCCGGAACAAGACAAGT
CCATCTATAGCTGCTGTTGTTGCCACTGTAAATTGGCCTGCTGCAAACCGCTATGCAGCT
CGTGTTCGTCCCCAGGAACATCGTAAGGAGAAGATTATCAATTTTGGAGATATGTGTGTT
GAGCTTGTTGAAACTTATGTTCGGCTGAATAGAGTCAAACCAGGTAATATTGTAATATTC
CGTGACGGGGTAAGTGAGGGCCAGTTTGATATGGTTCTCAATGAAGAGTTAATCGATCTC
AAGAGAGCATTTAGATCAATCAATTATGCACCAACTGTTACACTTATTGTGGCCCAAAAG
CGGCACCAGACTCGTTTGTTTCCTGCAGGCAGGAGTGGTGGGAACTCAAATGGGAATGTG
TCTCCTGGCACAGTTGTGGACTCTAAAATTGTGCATCCATTTGAGTATGATTTCTATCTT
TGTAGCCATTACGGAAGCCTTGGGACGAGCAAGCCCACACACTATCATGTACTGTGGGAT
GAACATGGTTTCAGTTCTGATCAATTGCAGAAGCTCATATATAATATGTGCTACACATTT
GCTCGATGCACAAAACCTGTGTCCTTGGTTCCACCAGTGTACTATGCTGACCTTGTTGCT
TACAGGGGAAGGCTGTATTATGAAGCAGTAATGGATGGGCAATCTCCAGCTTCAGCATCA
TCTTCAACCTCTTCAGCAGCGACATCATCGCTTTCATCGGCTGCTTCATTGGATGACAGA
TTCTACAGATTACATGCTGAACTGGAGAACATGATGTTCTTCGTTTGA
>Manihot-esculenta__cassava7471.valid.m1
ATGGACTCGTTTGAGCCAGATAAAAATGGTTCGCATCAGGACTTGCCTCCTCCTCCACCG
CTTGTTCCGCCGGATGTTGTTCCTCTCAGGGTAGAGCCTGAGCCGGTGAAGAAGAAGGCT
GTTAGGGTTCCGATAGCTAGGCGTGGTCTTGGATCGAAAGGGCAGAAGATTCCACTTGTT
ACTAACCACTTCAAAGTGAATGTGACTAATGTTGAGGGCTATTTCTTCCATTACAGTGTT
GCTCTCTTTTATGAAGATGGTCGTCCTGTTGATGGAAAAGGCATTGGAAGGAAGGTTATT
GATCGAGTGCAGGAAACTTACGATTCTGAGTTAGATGGAAAGCAATTTGCATATGATGGG
GAGAAGAGCTTATTCACTGTTGGTTCTCTTCCGCGCAACAAGCTCGAGTTTACTGTGGTT
CTTGAGGACATCTCGTCTAACAGAAGCAACGGAAATGCAAGCCCCGAAGACCATGGCAGT
CCAAATCAGAGTGATCGAAAGAGGTTACGACGACCATATCAGTCTAAAACATTCAAAGTA
GAGATCAGTTTTGCTGCTAAAATACCCATGCAAGCTATTGCAAATGCACTTCGGGGACAG
GAGTCGGAGAACTCTCAAGAAGCGATTAGGGTACTGGATATTATATTGCGTCAGCATGCA
GCTAAACAGGGCTGCCTCCTTGTTCGCCAGAACTTTTTTCATAATGATCCAAGGAATTTC
GCAGATGTTgGGGGTGGTGTTCTCGGCTGTAGAGGGTTTCATTCAAGTTTTAGAACCACT
CAGGGCGGCTTGTCTCTGAATATTGATGTATCTACAACCATGATAATTCAGCCTGGTCCT
GTTGTGGACTTTCTAATTGCCAACCAAAATGCGAGAGACCCCTTTCAACTTGACTGGGCA
AAGGCAAAACGAACTTTAAAAAATCTGAGGATCAAGGCTAGGCCGTCCAATCAAGAGTAC
AGGATAACTGGTCTGAGTGAGAAACCTTGCAGCGAGCAAACCTTTCAACTAAAGCAAAAA
AGTGGGGATGGCGAACCTCTTGAGTTAACTGTTTACGAATATTTTGTTAATCACCGCCGT
ATAGAATTGAGGTATTCTGCGGATTTGCCATGCATCAACGTTGGCAAGCCAAAGCGTCCT
ACTTATATTCCAATTGAACTTTGTGATTTGGTTTCCCTGCAACGTTATACAAAAGCACTT
AATACGCACCAAAGAGCTTCTTTGGTGGAGAGATCAAGACAAAAACCACAAGAAAGGATG
AGTACATTATCAAATGCTCTGAAAAATAGCAAATATGATGCTGAACCTCTGCTAAGATCA
TGTGGGATTTCTATAAGTAATAGTTTTGCCCAAGTTGATGGGCGTGTTTTGCCAGCACCA
AGGCTAAAAGTGGGCAATGGTGAGGATTTCTTTCCCCGAAATGGGCGCTGGAACCTTAAT
AATAAGAGACTTGTGGAACCATGTAAGATAGAGAGATGGGCTGTTGTGAACTTTTCAGCA
CGTTGTGATGTTCGTAACCTGGTACGAGATCTGACAAGATGCGCGGAGATGAAAGGAATT
TCCGTAGAGCCTCCTTTCGATGTATTTGAGGAGAGCCCCCAGCTTAGACGAGCCCCACCT
ACAGTAAGAGTGGAGAAAAtGTTTGAGGAGATTCAGTCTAAGCTCCCTGGTGCACCGAAG
TTTCTTTTGTGCTTACTCCCAGAGAGGAAAAACTCTGACATATATGGTCCTTGGAAGAAG
AAAAATCTTTCTGAGTTTGGAATTTTCAATCAGTGCTTGGCTCCGCAGAGGGTCAATGAC
CAGTATCTTACAAATCTTCTCTTGAAGATCAATGCAAAGCTTGGCGGGTTAAATACTTTG
TTAGCAGTGGAGCAAACACCTTCTATCCCTTTGGTTTCTAAGGTTCCTACAATTATCCTT
GGGATGGATGTGTCACATGGCTCTCCTGGGCATTCTGATGTCCCATCGATTGCTGCGGTG
GTCAGTTCCAGGCAGTGGCCATTGATTTCTCGTTACAGGGCATCTGTCCGTACACAGTCT
CCAAAGGTTGAAATGATCGACTCTCTGTTTAAAAGGGTATCTGAGACCGATGATGAAGGA
ATGATTAGGGAGCTTCTGTTGGACTTCTATACTAGTTCAGGGAAAAGAAAACCTGATCAG
ATCATCATATTCAGGGATGGTGTCAGTGAATCTCAATTCAATCAAGTATTAAATATTGAA
CTGGATCAGATAATTGAGGCATGCAAGTTTCTTGATGAGAAATGGAACCCCAAGTTTGTT
GTCATTGTTGCCCAGAAAAATCACCATACAAAATTCTTCCAGCCAGGAGCTCCTGAAAAT
GTTCCACCTGGTACTGTCATAGACAACAAAGTCTGTCATCCTAGGAACTATGATTTCTAT
CTCTGTGCACATGCAGGAATGATTGGAACTACAAGGCCCACACATTACCATGTTTTATTA
GATGAGGTTGGGTTTTCAGCAGATGATCTTCAGGAACTAGTGCATTCTCTATCATATGTG
TATCAAAGAAGCACAACTGCCATATCTGTAGTTGCGCCAATATGCTACGCACACCTGGCT
GCAACCCAGATGGGAACATTCATGAAGTTTGAAGACACTTCTGAGACATCCTCGAGCCAT

GGTGGTGTAACCTCTGCTGGAGCTGTTCCTGTGCCTCAGCTGCCAAGATTGCAAGAGAAA
GTATGCAATTCTATGTTCTTTTGTTGA
>Mimulus-guttatus_mgf000736m
ATGGATAGCAGCAGAAACAACAATTATGGACGCGGCCGTGGTCGTGGCCGTGGGAATTAC
CAGAATCAAGAGAGCGGCGGAAGAGGGCGGGGTTCTCAAAATCACAGCCGGCCTCCAGTT
CAGGGGAATCAGCCCCAACAACCTCACCATGCGTCCGGCTACAGACCGCCGCAGACGACG
GCACCGCAACTGCCTCATCAACCCGTCGTTGGCGTTGCTACTGCAAGACCCAACGCGTGG
GTGCGGGGCCCACCTTCTCCTACTCCTCCCCCATTTCAGGCGACGCAACAACCTCAACAC
CTCGGTGCTGCAAGACCAAACCCGTGGACTCAGAGGCCGTTGGGCCCATCTCCTCCACGT
CATACGCCTCCGCAGCCATCTGCTGTCGATCTTGACATTCAAAAGGTCAAGATTTCAGAA
GAGAAGCCCGTGTCATCTTCATCCGAAAACAAAGATAATCAGATTCAGCCAATCAAAAGG
CCTGATAAAGGTGGGATGCTTTCCATCAGGTCCATCCCACTTCTTGCCAATCATTTTCCG
GTCAATTTCAATCCACAGAACACCATTTTCCATTACGATGTCGATGTGAAGCCCATGTCG
TCATCGCCTAACGACAGTAAGCTCACTAAGAAGAAACTCAGAAAATCCGAATTGCGTTTC
ATCAAAGACCAGTTATTTTCCGGTGAGGACCAATTGAAGACAGCTTATGATGGCGAGAAG
AGCATCTTCAGTGCAGTTCCTTTGCGTGAAGGACAGTTTCAGGTGGATATTTCTCAAGGT
GAAGATGTAAGGAGCGGCTCGTATACGTTCGTTATCAAACTTGTGAACGAACTGAAGCTA
TCGAAGCTGAGAGACTACCTTAGAGGAAACCTCTCTTATGTTCCTCGTGATATATTACAA
GGGATGGACTTGGTAATGAAAGAGAATCCTTCTAGAAAGAGAATCACAGTCGGTCGAAGT
TTTTTCTCTCCCGAATTCCGGAGCCGTGACGACTTTCGTAACGGACTCGCTGCATACAGA
GGGTTGCAACAGAGTTTGAAGCCTACTTTGCAGGGTCTTGCTCTGTGTTTGGACTACTCT
GTTTTAGCATTTCGAAAGCCGTGGCCAGTGATGGAGTTTCTCAAAGAGCATTTTAAAGGG
TTTCAAGTGCAAGATGTAAAAAAGTCACGACGAGCTGTCAACGATGCTTTGAGAGGGTTG
AAAGTGACGGTTACACACCGTCGCACGAAGCAAAAGTATACTGTTGCAGGGCTAAGTGAA
GAGGACACCTGTGATATTTACTTTGATCTTGTTGATGTGGAAGGCAAGAACCCTCCTCAA
AGAACTAGCCTTGTTAAGTATTTCAAGGAAAAATGGGGTAAGGATATTGTGTACCAGAAT
ATACCTTGCTTGGAACTTGGCAAAGGTAGTAAGTCGAACAAAGTACCGATGGAGTTCTGT
GTGTTAGTCGAAGGGCAGAGGTACCCGAAGGAGAATCTGGACAGAAACACGGCTGTGCTT
TTGAAAAACATATCACTTGCGGATCCTGGTGACAGAAGGGACACACAATAAATGAGATGGTG
CGGAGTGATGATGGACCTTGCGGGGATGTTGCTAGAAATTTTGGATTTCAGGTTGATAGA
AACATGACAAAGGTTTCAGGCCGTGTTATCGGTTCGCCCGATTTGAAATTAGGAGCTCCA
CGACCCGTCAAAGTGGACGCAGTAAAGTGTCAGTGGAACCTTCTCGGAAAATCTTTTGTC
GATGCAAAACCAATCGAGCGTTGGGCTCTATTAGATTTAACAGGTGGCGATCGATACAAG
CTGCAAGAACACACTTTTATCAGCAATCTGATTGGCCGGTGCAGAAGCCTCGGTAACCGC
ATTGAGGAACCTGTTGTGTACCGTTTGGCTCGAATGTCTGACCTGTCTTCCATCAACAGA
GTCGAAAAGCTTCTCAAAGGCATTATGGAAGAATCGAACAAAATCAGTGAAGGGAAGTTG
CAGTTGATTGTTTGTGTAATGACCAAAAAGGACCCGGGCTACAAGAATCTCAAGTGGGTT
TCCGAGACAAAACTCGGTGTGGTGACTCAGTGTTGTCTTTCCGGTTCTGCCAGCAAACCG
CAGGGGCAGGACCAGTATTTTGCCAACCTCTGTCTGAAAATCAATGCTAAATTAGGTGGC
AATAATTTCGAAGTGGCGGGAAACTTTACCCATTTCGATGCGAGAGATCATGTGATGTTT
ATAGGAGCTGACGTGAACCATCCTGCACCTATGAACTCGTCTTGCCCCTCCATAGCTGCA
GTTGTGGCGACCGTTAACTGGCCAGCTGCAAATAAATATGCAGCCAGAGTTAGCCCACAG
AAACACCGCTGTGAAAAGATCGAAAATTTCGGAGCAATGTGTTTGGATTTGGTCAACACA
TACGCCAAATTTAATAAAGTCAAGCCGAAGAGAATCGTGGTGTTTCGTGATGGTGTTAGT
GAGGGGCAGTTCGAAATGGTCCTAAGTCAGGAGCTGTTGGATTTGAAGAACTCGATTTAT
GACGGTGATTATCAGCCGTCGATCACTCTCGTTGTTGCTCAGAAGCGTCACCAGACGAGG
CTTTTTATCGATAATGGAAACAGCGGGGGTAGAATCGGAAATGTACCTCCTGGAACTGTT
GTGGATACGAGGATTGTGCATCCGTTCGATTTTGACTTTTATCTGTGCAGCCACTATGGA
GCTCTTGGAACGAGCAAGCCTACTCATTACTATGTGCTTTGGGATGAGAATTCGTTTACT
TCTGACCAATTGCAGAAGCTTATAAACGACATGTGCTACACATTTGTTCGGTGCACTAAA
CCCGTTTCGCTTGTGCCACCTGTCTACTATGCTGACCTGGTTGCTTACAGGGGAAGAATG
TTCCAAGAGGTTGCTATGGAGAATCAATATGATTCGTCTAATGCTGCGTTTGACCAGAGT
TTCTACAGCCTGCACCCGGATTTGGAGAACTTTATGTTTTTCGTTTGA
>Mimulus-guttatus_mgf005010m
ATGGTGAGGAAGAGGAGAAGTGACATTCCTGGCTCTGGTGAGGGCTCCGAACCTCAGGAA
ACTGGTGTAGGACGCGGTGCCGTTCAACGCCCACCTGCACAGCTGCAACAGCCCCACCAG
CCTCAACCGCCCTACCAGCCGCAACACCCACAACAGCCACAGCAGCCACAACAGCCACAA
CAGCAAGGAGGAGGAGGAAGAGGCTGGACACCTCAACGTGGAGGATATGGTGGCCGTGGT
GGTGGAGGCGGCAGAATGGCTCCCCCGCAACACTATGGTGGGCCTCCCGAGTATCAACAG
CAAGGTCGGGGCGGTCAGCAATATCAGCGAGGAGGGGGTGCACCACGACGTGGCGCTTAT
TCAGGTGGCCGAGGCGAGGCACCATTTGCTGGTGGGCCCTCTAGGCCACCCGCTCCCGAG
CTGCACCAAGCTGCTACCCAGTCCCCGTATCAAGCGACAATGACTCAGCCAATTCAGTAT

GGGAGTCCTGCGGAGACACTCAGAGGGGCTAGCTCTTCTTTCCAGCCTCCTGAACCGACA
GATGTAGAAGTGTCTGAGCAGATCCAGCAACTCTCTATCCAGCCAGATGTTGCTCCAAGC
CAAGAAATGCAACCTGCTTCAAGCAAGTCAGTGAGATTTCCTCTGAGGCCTGGCAAGGGT
AGCAATGGTACCAGGTGTATTGTCAAAGCGAATCATTTCTTTGCTGAGCTGCCTGACAAA
GACTTGCATCAGTATGATGTCTCAATTACTCCTGAAGTCACATCCCGAGGAGTAAACCGT
GCTGTGATGGCGCAGTTAGTGAAGCATTATAGGGATTCTCATCTTGGAAAGAGGCTCCCT
GCCTATGACGGAAGAAAGAGTCTGTACACTGCTGGCCCTCTACCTTTTGTGGCAAAGGAG
TTCAAAATAACTCTTACCGACGAAGAAGATGGTCCTGGAACTGCCAGGCGAGAAAGGGAA
TTCAAAGTGGTTATCAAGTTCGCTGCACGCGCAGACTTGCACCATTTAGGCATGTTCTTA
CAGGGTAGACAAGCTGATGCACCCCAAGAAGCTCTTCAGGTTCTTGACATTGTACTGCGT
GAACTTCCAACCTCTCGGTATTCTCCAGTTGGTCGATCTTTCTATTCTCCTGATTTAGGT
AAAAGGCAGCCTCTTGGTGAAGGACTGGAAAGTTGGCGTGGATTCTACCAGAGTATTCGT
CCTACCCAGATGGGTTTGTCACTGAATATTGATATGTCATCCACTGCGTTCATTGAGCCT
CTCCCGGTGATTGATTTTGTCACCCAGCTTCTGAACAGAGATGTATCAGCTAGACCATTA
TCTGATGCTGACAGAGTCAAGATTAAGAAAGCCCTTAGGGGAGTAAAGGTGGAAGTTACT
CACAGAGGAAACATGCGAAGGAAATACCGTATCTCTGGTTTGACATCACAAGCAACTCGT
GAACTAACATTTCCTGTTGACGAGAGAGGCACTATGAAATCTGTTGTTGAGTATTTTCAA
GAAACCTATGGCTTTGTTATCCAACACACTCAGTGGCCTTGTCTGCAAGTTGGGAATACA
CAGAGGCCAAACTATCTTCCCATGGAGGTGTGCAAGATAGTAGAGGGCCAGAGGTATTCT
AAGAGGTTAAATGAGAGACAGATTACTGCACTACTCAAGGTCACTTGTCAGCGTCCTCAG
GAAAGGGAGCTTGACATTCTCCAGACTGTACACCATAATGCTTATGCTGAAGACCCTTAT
GCTAAAGAATTTGGAATTAAGATTAGTGAGAAGCTAGCGCAGGTCGAAGCTCGTGTTCTA
CCTCCACCTTGGCTCAAATATCATGACTCGGGTCGGGAGAAGGACTGTCTCCCACAAGTT
GGACAGTGGAATATGATGAACAAGAGGATGGTCAATGGTGGGACAGTGAACAGTTGGATA
TGCATCAACTTTTCACGCAATGTTCAAGACAGCGTGGCACGTAGTTTCTGCCACGAGCTT
GCTCAGATGTGCAGCACTTCTGGCATGGCTTTTAATCCTGAACCCGTCCTACCAGTTTTG
ACTGGTCGCCCAGATCAGGTAGAACGAGTTTTGAAAGCTCGGTATCATGATGTGACAACT
AAATTGCAGCCCCATAAGAAGGAGCTTGACTTGTTAATTGTTATATTGCCAGACAACAAT
GGCTCTCTTTACGGTGATCTAAAACGGATATGCGAAACGGATCTTGGAATCGTCTCACAG
TGCTGTCTTCAGAAGCATGTGTACAGGATGAGCAAACAGTATCTTGCCAACGTGTCTCTG
AAAATTAATGTCAAAGTTGGTGGGAGGAATACAGTGCTGGTGGATGCGCTCTCCAGGCGT
ATTCCTCTTGTCAGTGATCGACCAACTATCATATTTGGTGCAGATGTTACACATCCCCAT
CCTGGAGAGGACTCCAGCCCATCCATTGCTGCTGTTGTTGCTTCCCAGGATTGGCCTGAA
GTCACAAAGTATGCAGGCTTGGTTTGTGCTCAGGCACATAGGCAGGAACTCATCCAGGAT
CTCTATAAAACCTGGCAGGATCCGGTTAAGGGAACCATGCATGGTGGCATGATCAAGGAA
CTACTTATTTCATTCCGCAGAGCAACTGGGCAGAAACCACAGCGAATTATCTTCTATAGG
GATGGTGTCAGTGAAGGCCAGTTTTATCAAGTTTTGCTTTATGAACTCGATGCTATACGA
AAGGCATGTGCCTCGTTGGAGCCAAACTATCAGCCTACTGTTACCTTTGTTGTGGTCCAG
AAACGTCATCATACTCGATTGTTTGCTAATAACCACCATGACCGACATGCAGTTGATAGA
AGTGGAAACATTCTTCCTGGTACTGTTGTTGACTCCAAAATCTGTCACCCAACTGAGTTT
GACTTCTACCTCTGCAGCCATGCTGGTATACAGGGTACTAGCCGTCCAGCACATTACCAT
GTTCTCTGGGATGAGAACAAATTTACAGCTGATGCTCTTCAAAGTCTTACTAATAACCTT
TGCTACACATACGCCAGGTGTACTCGCTCTGTTTCCATTGTGCCTCCTGCATATTATGCA
CATCTGGCAGCTTTTAGAGCTCGATTCTACATGGAGCCAGAAACATCAGACAGTGGTTCA
ATGACGAGCAGTGCAGTCGGTGGTAGAGGTGCGGGTCCCGGTGGAAGGACCACTAGGCCT
CCGGGGGCTAATGCTGCAGTTAGACCCCTCCCTCAGCTTAGGGATAACGTCAAAAGGGTC
ATGTTTTACTGTTGA
>Mimulus-guttatus_mgf006816m
ATGGAAGAAGAAAAACAACAAGAAGAAAAGTCCAACACTAATAATAATAAAACAAGCACA
AAATGCAGTCCAAGAACAAGCAGTTACAGAGGCAGTGAAACTGCTTTTACCTACCATCAT
CATCATCACCAGCAGTTGATTCAGAACCCATATGGGTACGGGTACGGGTACGGATTCGGG
TTCGGGTTCGGTTACACGAATCAATACCAAACTTTCCCTGCTCTGCTTCCCCTGCCTCCC
ACCATACCCCTGCATCTCTCAGTTGCTCCTTGTTTCCCTCAAAACCATGGATTCGGGTCA
AGAACCCATTTCAAGAAACCCACAAGGAATCACAATTACCCTGTTCCACCTGCACCAGCA
GAAGTGGAGAAGAGAGTGGTTTGTCACCCTAAAGAAAATGGAAGAAAGATTGTTAGTGAT
GCAAATAAAAAACAATTCCAAGTTGCAAGAAGACCAGATTCCGGCGGAATCGAAGGCAAG
CCGATAACCCTCCTAGCCAATCATTTCCTAGTCGAATTCGACCCGTCTCAAAAGATCTTC
CACTACGACATCGAAATCCATCCAAACCCATCGAAAGAATTCGCAAGAATGATCAAACAG
AAACTTGTCGAACAAAATTCGTCCCTTCTCTCCAATGCAGTACCCGTATTCGACGGCCGG
AGAACCATTTACAGCCCTATCGAATTCCAAGAAAAGAATCTCGATCTCTACATTAGCCTC
CCGATATCTCCTCTTACGCCGTCGAAGGTTTTCCGCGTAAATATCAAGCTCGTATCGAAA
TTCGACGGAAAAGCCCCTAGCAATTACCTAAACAATGGAGTCGAATACGATTCGACCCCT

CTTCCTCAAGAGTACATCCACGCATTGGATGTAGTTCTCCGCGAGGGTCCCACCGAGAAG
TGTATACCTTCCGGAAGGTCGTTCTACTCGAGCTCAGTGGGAGGTGCGAAAGAAATCGGA
GGTGGGGCTATTGCACTTAGAGGATTCTTTCAAAGCTTGAGACCTACACAACAAGGGCTT
GCTCTCAACGTCGACTTCTCGGTTACTGCCTTCCACGAGAGCATCGGAGTGATCCCCTAT
TTGCAGAAACGGCTCGACTTTATGCGGGACCTATCTTGCAAAAGGGCTTTGAGTTTGACG
AGCGAGGAGAAGGATGAAGTGGAGAAAGCACTCAAGAATATGAGGGTTTTTGTCTGCCAT
AGAGAAACCATGCAGAGATACCGAGTTTACGGGTTGACTGAAAAGGTTACGGAAGATCTT
TGGTTTCCCGATAGAGATGGGACGAGTATGAGACTTACGAGCTATTTCAAGGAGCAATAT
GGGTACGATATACAGTATATGAATTTGCCTTGCTTGCAGATTAGTAGAAGGAAGCCGTGT
TATTTGCCTATGGAGCTTTGTGTGATTTGTGAAGGGCAGAAGTTTCTCGGAAAACTCTCG
GATGATCAAACGGCGAAAATACTCAAAATGGGCTGTCAAAGGCCGAAAGAACGAAGGGCG
ATTATCGATAAAGTTATGAAAGGATCGTTTGGGCCAGCCAGCGGTGATCAAGGAAAAGAA
TTTAAGCTTCGAGTTTCGAAAGAAATGACGAGATTGACGGGGAGAATTCTTCAGCCTCCG
AAGCTGAAGCTCGGAAACGGTGGCAATGTAAGAAATTTAACACCTTTCCGAAACGATCGA
CAATGGAACCTTTTAGACAGCCACGTCTTCGAGAGCACGCGTGTCGAGAGGTGGGCGATC
ATGAGTTTTGGCGGGACCCACGAACAGAAATCCACCATAGGCAAATTCACAAACCACCTC
TCTCAAAGATGCCAACACTTGGGTATATACCTCCACAAGAACACAGTAGTCGAACCGATT
TACGAGCCGATGCACGTGCTCGGAAATCCGAAGCTTTTGGAGTCCAAACTCAAGAAAATT
TATAACTCTTCTTCGGGAAATCTCCAATTGCTCGTTTGTGTTATGGAGAGGAAACACAAA
GGGTACGCGGACTTAAAAAGAATTGCCGAAACTAGGATCGGAATAGTGAGCCAATGCTGT
CTGTACTCGAATCTTGAAAAAATGAGCTCGCAGTTTCTCGCAAATTTAGCTCTCAAGATA
AACGCGAAAGTCGGTGGATGCACTGTCGCCCTCTACAACACACTCCCTTCTCAAATCCCG
AGGCTTTTCAGACAAGAAGATCCCGTTATTTTCATGGGCGCTGATGTCACTCATCCTCAT
CCATTGGACGATTCTACCCCTTCCGTCGCCGCTGTTGTAGGCAGTGTGAACTGGCCCGCT
TCGAATAAATACGTCTCGAGAATGAGGTCGCAAACTCACCGGCAAGAAATAATCGAAGAT
CTTTGTAATATGGTTACGGAAATATTGGAGGATTTCTTACACGAGCTCTCAAAACTCCCG
ACTAGGATTGTATTCTTTCGAGATGGAGTAAGCGAAACGCAGTTCCACAAAGTGATGCAT
GAGGAGCTGAAAGCTATTAAAGAGGCTTGCTCGAGATTTTCCGATTATGCCCCTCCGATC
ACTTTCGCTGTGGTGCAGAAGCGCCACCACACCAGATTATTCCCCACCGAGAATCAGTTA
CCGCCGGACGAGAATGTTTCGCCGGGGACAGTGGTCGACAGTGTGATCGTTCATCCGAGG
GAGTTCGATTTCTATCTTTGTAGCCATTGGGGTGTGAAGGGGACGAGCAGGCCGATTCAT
TACCATGTATTATGGGACGAAAACAAATTTACGTCCGACGAAGTGCAGAAGTTGGTCTAC
AATCTTTGCTACACGTTTGTGAGGTGTACGAAGCCTGTATCTATTGTGCCTCCCGTTTAT
TATGCGCATCTTGCTGCATATAGAGGCAGCTTGTATCTCGACCGATCGGATTTGAGTATA
ACTATTTCGAGGGATTCGATACCGAAGGCGATGCCTTTACCTAAACTTAGTGAGAATATT
AGGAAGTTGATGTTTTATTGCTGA
>Mimulus-guttatus_mgf009177m
ATGGATCCCTCTGAGCTAAACGGTAGTGAGGCAGAGCTAAATGGTAATGGGGCGGAGCTA
AATGGTAACGGGGCGGAGCTGAATGGTAATGGGGCGGCAGAAGGTAATGGAGGTTCAGAA
GAAGCACTGCCTCCTCCTCCACCTATCCCCCCAAACGTTACTCCAATAAAAGCCGAGCCA
GAAGTGAAAAAGATCACCCGTGTTCCAATGGCTAGACGAGGCCTTGGAACCAAAGGAAAC
AAAGTTCCGATTCTGACCAATCACTTCAAAGTGAATGTCAACAGTGTTGATGGCTACTTC
TTCCATTACAGTGTTGCCCTTGCATATGAAGATGGCCGCCCTGTTGATGGCAAGGGCATT
GGTCGGAAAGTGCTCGACAGAGTTCACGAGACGTACGATTCTGAGTTGGCTGGCAAAGAG
TTTGCTTATGATGGGGAGAAGAGTTTGTTCACTGTGGGCCCGCTTCCGAGGAATAAACTC
GAGTTCACTGTTGTTCTTGAGGACGTTACCTCGAGCAGGAACAATGGAAATGCGAGTCCA
GGTTCTGAAAATCCCAACGACAGTGACAGGAAAAGGCTGAGGCGACCGTACCAGTCTAAA
ACTTTCCAAGTCGAGATCAGCTTTGCTGCAAAGATTCCCATGCAGGCAATTGCCAACGCG
TTACGCGGACAAGAATCTGAAAACTCTCAAGAGGCTTTACGTGTTCTTGATATCATACTA
AGGCAACATGCAGCAAAGCAGGGTTGTTTGTTGGTTCGCCAGTCGTTTTTCCACAACGAC
CCGAAGAATTTCTCTGATGTTGGAGGTGGGGTCCTCGGCTGCCGAGGCTTCCACTCGAGT
TTTAGGACAACCCAGAGTGGATTGTCTTTGAACATTGATGTATCTACAACAATGATAATC
CAGCCTGGTGATGTAGCGAACTTCCTTGTTGCTAACCAAAATTGCAGGGACCCCTTTTCG
GTCGATTGGGCAAAGGCTAAACGAACGCTGAAGAATTTGAGGATCACCGTAAGTCCGACT
AACCAGGAGTTCAAAATAACTGGTCTAAGCGAGAAGTCTTGCAGAGAGCAAACTTTCACA
TTGAAGCAGAAAAGCAAGGATGGCGATGGTGAATTTCAAACAACAGAAGTGACTGTGTAC
GACTACTTTGTGAACCAGCGGAACATCGACTTGCGATTCTCTGCTGACTTACCATGCATT
AATGTCGGAAAGCCAAAGCGCCCGACTTACTTCCCAGTCGAGCTATGCTCTCTTGTGTCA
CTGCAACGCTACACGAAAGCTCTATCTACACTTCAAAGAGCTTCATTGGTCGAGAAATCA
CGCCAAAAGCCACAAGAAAGGATGTCTGTTCTGAGCAATGCTTTGAAAATCAACAAGTAT
GATTCAGAGCCTATGCTTCGTGCTTGTGGTGTCTCGATCAATAACAACTTCACTCAGGTT
GAAGGGCGTGTGCTGCCAGCCCCTAAGCTCAAAGTTGGTAATGGAGAGGACTTGTTTGCT

AGGAACGGCCGATGGAACTTCAACAACAAGAGGTTTGTTAATGCGTGTAAGGTTGAGCGA
TGGGCTGTGGTGAACTTCTCTGCTCGTTGCGATGTGCGTGGCCTGATCAGAGATTTGATT
AAAGTCGGCGAATCGAAAGGAATCATTGTAGAAGATCCGTTTGATGTTTTCGAAGAGAAT
CAGCAGTTTCGAAGAGCTCCGCCTATGGTTAGGGTTGAGAAGATGTTTGAAGAGGTGCAG
TCCAAGCTTCCGGGCCCACCAAAATTCCTACTCTGTTTGCTTCCCGAGCGTAAAAACTGT
GCACTTTATGGCCCGTGGAAGCGCAAAAACTTGTCCGAATTCGGAGTTGTTACCCAATGC
CTGGCTCCACAGCGAGTTAACGATCAGTATCTGACAAACCTTCTGCTGAAGATAAATGCA
AAGCTTGGTGGGTTGAACTCCGTGTTAGCTAGTGAACTTTCGCCTACAATTCCGATGGTG
TCAAAGCTGCCGACATTGATTCTTGGTATGGACGTCTCTCATGGTTCGCCTGGACAGTCT
GATATTCCATCGATTGCTGCGGTTGTTAGCTCTAGGCAATGGCCTTCGGTTTCTCGTTAC
AGAGCATGTGTTCGCACTCAATCTCCAAAGATGGAAATGATTGATTCCCTGTTCAAACGT
GTTTCCGATTCTGAAGATGATGGAATTATGAGGGAGGCTCTGCTGGATTTCTATGTTAGT
TCTGGGAAACGAAAGCCCGACCAAATCATCATTTTCAGGGATGGTGTGAGTGAATCTCAA
TTCAATCAAGTCCTCAATATTGAACTGAGCCAAATTATAGAGGCTTGCAAATTCCTTGAC
GAAAAATGGAATCCCAAGTTTGTCGTAATCATTGCACAGAAGAATCACCACACTAAGTTT
TTCCAGCCGAATTCTCCCGATAATGTGCAGCCAGGGACTGTGATTGATAACAAAGTTTGT
CATCCGAGGAACAACGACTTCTACCTGTGTGCCCATGCGGGCATGATCGGAACAACGAGG
CCTACACATTACCATGTTCTGCTCGACGAGATGGGTTTTTCTACCGATGATTTACAGGAG
CTTGTTCACTCACTTTCTTACGTATACCAGCGCAGCACCACTGCTATATCGATTGTTGCT
CCAATCTGCTACGCGCACTTGGCAGCGACTCAGTTGGGGCAGTGGATGAAGTTCGAAGAC
ACATCGGAGACTTCGTCAAGCCTTAACGGGGCCGCGCCAGGTGGCGCGCCAGCTGTACCG
CCTATGCCTAAGTTATCGGAGAGCGTCCGCAACTCCATGTTTTTCTGTTAA
>Mimulus-guttatus_mgf010559m
ATGGGAACAGATGGTGGGGCAGACCATACTTCTCCATCTTTGGACCTACCACCACCAACT
ATACCACCAAATGTAAAGGTAGTACGAGAAGACCTGCCTAATAGATCTATAATTACACGT
CCTGGATTCGGGACTTCTGGCAAGCGCATCTCTTTGCTCGCTAATCATTTCAAAGTTTCT
ATCACAAATCCAGACGAAACTTTTTACCAGTACAGCGTTGCTATTAGTTTTGAGGATAAT
AAAGCGGTTGAGAGCAAGGTACTAGGGAGGAAAGTTATCGATAAACTTTACCAAACATAC
TCTTCTGAACTTGCTGGGAAGAGATTTGCTTATGATGGGGATGCGAGTTTGTACACTGTG
GGGCCCTTACCGCAGAACAACTTGGAATATACAGTGGTTCTTGAAGAATCTATTGCCAAG
TGCAGTGGGAATCCTTCTGATAACGGAACCCAAATCGAGTCTTACAAAAGATCTAAGAGC
TCTCTACACTCAAAAACTTTCAAGGTAGAGATCAGTTATGCTGCAAAAGTACCATTGAAT
TCCATATCTCATGCCCTTCAAAAAGCTGATCCAGAAAAGGCTCAAGATGCTCTGAGGGTT
TTAGACATCGTACTAAGGCAAGATGCAGCTAAAAGAGGGTGCCTTTTGGTTGGTCAATCA
TTCTTTCATGATGACTCGCGGACGTTCACTGATGTTGGAGGGGGGGTGACAGGTGTGAGA
GGTTTCCACTCCAGCTTTCGTCCAACTCTGAGTGGTTTGTCTCTGAATATGGATGCATCG
ACAACCTTGATCTTGACGCCTGGACCAGTCGTGGACTTCCTGCTTGTTAATCAGAATGTG
AAGGAAACTCGGTATATTGACTGGGCAAAGGCCAAAAAGATGCTCAAGAATATGAGGGTT
AAGGCGAGGCACAATAATATGGAATTCAAAATTGCAGGTTTGAGTGAGAAACCTTGCAAT
CAGCAGTTATTTTCGATAAAAGTTAATAGTGGTGGTAGTCCCGGAGGTGATGGAGAAACC
GTGGAGATTACTGTTTACGACTATTTTGTTAAACACCGTAACATAGAACTTATATCTTCA
TCATATATGCCATGCATTGATGTTGGGAAACCGAAAAGACCGATCTATCTGCCTATAGAG
CTCTGTTCTCTAGTCTCTCTTCAGAGATACACAAAAGCACTGTCGGTAACACAAAGAGCA
TCCTTGGTTGAAAAATCAAGGCAGAAGCCTCCTGAGAAAATTCGAGTTGTAACCGATGCT
ATCAAAAACTCTCACTATGATGAGAATCCTGTCCTTGTCGCTTGTGGTATTTCAGTTGAA
AAGCATCTTAGTCAATTAGATGGACGGATTCTTGATGCACCAAAGTTAAAGGTTGGCAAC
AGTGAAGACTGTTTACCCCAAAATGGCCGGTGGAACTTCAATAACAAGAAACTTCTGAAC
CCCAGCCAGATAGATCATTGGGCACTTGTCAATTTCTCTGCACGTGCAGATTGTAGCCAT
CTTTCGAGGGAGCTAATCAACTGTGGGAGGAACAAGGGCATTCATATTGAACGCCCATAT
GCAATATTCGAGGAAGATCCACAATATAGAAAAGCTAGCCCTGTGACACGTGTAGAAAAG
ATGGTTGAACATATTACGGCTACACTACCTGGTCCTCCTGAGTTTTTGCTTTGTGTTTTG
CCCGAACGAAAAAATTGTGATCTTTATGGACCTTGGAAGAGAAAATGTCTATGTAACTTG
GGTATCGTCACCCAATGCGTCTCTCCTATCAAAATCAACGACCAGTACCTGACAAATGTA
CTTCTCAAAATGAATTCTAAGTTAGGGGGGATCAACTCATTGTTGGCAATAGAGAATTCC
CGTCGAATTCCACTCATTACGGATAAGCCGACCATGATCTTGGGAATGGATGTATCGCAT
GGTTCTCCTGGTCGATCTGATATCCCTTCAATTGCTGCGGTTGTTGGATCTCGGAGTTGG
CCATTGATATCAAGGTATAGAGCAGCTGTACGAACCCAATCTTCAAGGGTGGAGATGATT
GAAAGTTTGTTCAAGCCTCTAGCAAATGGGGAGGATGACGGCATTATGAGGGAACTGCTT
AAGGATTTCTACGAAACCAGTAATGGACGCAAACCAACTCAGATTATTATTTTCAGGGAT
GGTGTGAGTGAATCCCAGTTTACACAAGTCATCGACATTGAACTCAATCAAATTATCAAG
GCTTATCAGCATCTAGGCGAGACCGAGATTCCGAAATTCACAGTGATAGTGGCCCAGAAG
AATCACCATACGAGACTTTTCCAAGCTACAGCTGCTGAGAATGTTCCACCCGGTACTGTT

GTGGACACAAACATTGTTCATCCTACAAATTACGATTTCTATATGTGCTCTCAGGCAGGG
AAAATAGGAACTTCTCGACCTGCACATTATCATGTATTGCTCGACGAGATTGGTTTCTCC
CCAGACGATATGCAGAATCTCATCCATTCCCTATCATATGTATATCAGAGGAGCACTACT
GCCATCTCCATTGTTGCACCGGTATGTTATGCTCATCTTGCAGCCCAACAAATGAGTCAG
TTTATAAAATTTGAAGACTCGTCCGAACAAAAAAACACGACAACAGGAGGAAGCATACCT
TTTCCAGAGCTGCCCAGGCTCCACAAAAACGTTGCTGGATCGATGTTCTTTTGTTGA
>Mimulus-guttatus_mgf011041m
ATGGACGGCGATCGACAACCCCCAGCGGCGGTGAGTGAGTATCCTTTCCTTTTGCTTCTG
CTAATACCACTAGCCCTAACACTAGATTTTCTTTTCCCCACAACCAGCCACCACCGGCG
CAGAAGCAGTCGGGGAAGTACAGGCCGCCGCACCTCCGGAACCAGGAGAGTAGCGGCGAC
GATGCTAGCAAACTTTGCTGTCAATTGCCGAAAAAATCGGAATCGAAGCAGCCTCAATCA
GGTCTTCCAGAAGTTACCGATAACAAAATTTTGCCTGCCAAGCGGCCTGTGGGCAGTACA
CATGGGGGAAGACCAGTTAACCTTGTAGTAAACCATTTTCTTGTCAAGTTCAACCCTAGA
GTCACCATATTTCATTATAATCTCGATATTAAACAAGTGATATCTCATGGAAAAAGACCA
GCGAAAAAGTCGAAGAAAAAGGTGAACCTACGTTTGATCAGAGACAAACTCTGCCTCGAT
GATCCAGCTCGATTTCCGTTGAATCGGACCGCATATGACGGTGAAAAGAATCTCTACAGT
GCAGTGCCGCTGCCCACCGGTCAATTCAATGTAGAACTCTGCGATATTGAAGATCTGTTG
ACTCGCACGTATGTCGTTTCAATCAAGTTAATGAATGAGCTAAAGCTTTCCAAGCTGGAA
GACTATTTAAGCGGGAAGGTGACGTACGTGCCTCGTGATATACTGCAAGGGATGGATCTT
GTGATGAAGGAGAATCCTTCTAGGCATAGAATCTCTATCGATCGTCACTTTTACTCTTCC
AGTTTTAACGTGGAAGATGATTTTAAGCACGGTGTTGCAGCGTATCGAGGCTTCCAATCA
ACTTTAAGACCGACGTCGCAGGGTCTGGCCTTGTGCCTCGACAGCTCGGTTTTGGCGTTT
CGTAAGCCGTTGGCAGTTATGGATTTCCTTAAAGAGAATATTCCTGAATTCGACGGAATG
TACCTCGGTTTCAATTTGAGGCGGAGAGTTGCACATGCGTTGAAAGGATTGACGGTTAGA
GTGACTCACCGTGTTACGAAACAGCGGTTCACCATAGCTGGTTTGACTGCCAAAAACACC
CGTGATCTTTGGTTCGATTTTGTCGACCCGAAAGGGAGGGACCCGACTGTTAACGTCAGC
CTTGTGCAGTACTTCAGGCATAAGTATGGCAAGGACATTGTGTACCAGGATATTCCTTGC
TTGATTCTTGGCAGAAATAATCGGACGAATCACGTACCTATGGAATTCTGTATCTTGTCT
GTCGGACAACGCTACAGAAAAGAGCTTTTGGATGAAGTTTCGCAGGATAAATACGATGCA
AAATGCCTGGCTTCGCCGCCAGAAAGGAGAAAAACAATCTGCGAGACGATGCAAGCTTAC
GATGGACCTTGTGGGGATGTCACTCAAAATTTTGGACTTCGAATCGATAAGAACATGACA
TCTGTCGAAGGTCGAGTTATATCTCCACCCGATTTGAACTTTGGTGCTCCAGATGGTAGT
GTTGATATAGTAAGAGTTGAAAACGAGAAATGCCAGTGGGACATAGCTGAAAACTCTGTT
GTGGAGGGCAAACAAATCGAAAGATGGGCCTTGATCGACTTCAGCTCTTCTGATTCTTCG
AGAGCCAAAGACTTCATCAGGAATCTGAGAAACCGATCCACGAGTTTAGGTATCTATATG
GACGAGCCTCTACTTTGCCATTTCACCGGCATGCGCGAGTTCTCTTCGGTCAACAGGCTA
GAAGGACTTCTCCGAAGTGTCGTCGAGGAAGCCAGCAGAAAAAATCCGAATAGATTACAG
ATAATAATATGCGTAATGGCGGAAAAACATCACGGCTACAAGTACCTTAAGTTCGTGTCG
GAAACACGAATTGGTGTAGTGACTCAGTGTTGCCTGTCTGGTCACGCATTCAGAGGAGAC
GAAAAGTTTCTCGGAAATCTGTGTCTCAAGATTAATGCAAAGCTCGGAGGAAGTAATGTT
GAGTTGACTCAGAGACTTGCTGATTTCGAGGAGGAAGATCATGTTATGTTCATTGGAGCC
GATGTGAACCATCCAGTCTCGAAGAAATCAACAACTCCATCCATAGCAGCAGTTGTCTCT
ACAGTGAACTGGCCTGCAGTGAACCGCTATGCCGCAAGAGTTTGCCCTCAAGACCACAGA
ACCGAGAAGATTCTCGAATTCGGTTCCATGTGCCGCGATCTCGTCAACACTTATTTCCAG
GTCAACAAAGTCAAACCGAAGAAGATTGTTGTTTTTCGAGACGGTGTGAGCGATGGGCAA
TTCGATATGGTACTGAACGAAGAATTATCCGACTTAAAAAGGTCTGTCTGCGATGAGAAT
TACAAGCCAACAATCACTCTTGTTCTAGCTCAGAAGAGGCACCAGACTCGTCTCTTTCTC
GAAAATGTTCTGGATGGTGGGGCCACTGGGAATGTGCCACCTGGCACCGTTGTAGACACG
AAGATTGTCCATCCGTTTGAGTTCGATTTTTACCTCTGCAGTCACTATGGAAGGATTGGG
ACTAGCAAGGCGGTGAGGTACTGTGTACTGTGGGACGAAAATTCGTTCACGTCCGATGAA
TTACAGAAGCTTATATATAATTTATGCTTCACGTTTGCACGTAGTACGAGGCCTGTTTCG
CTGGTGCCACCTGTATACTACGCTGACCTGGTCGCTTACAGGGGTCGTATTTTTCAGGAG
GTGGCGAAGGAGTTTGAATCTCGTTCATCGTTTTCGTCGTCGTCGAATTGTACTACTGCA
TTTGATAGAAGGTTCTACAACCTTCATCCGGATCTTCAGAATATCATGTTCTTTGTTTGA
>Mimulus-guttatus_mgf011360m
ATGAAACCCCAGTTACCACCGCCACCAAACACCGCCACCAAAAACCCTAAAACCGCTCAA
ACCGGCAAGGGGGCCGCGGCCCCCCCACCACCGTTATTACCACAAGAACCTCAAAACCAG
TCGCCGTCACCCCCATCAAGAAACAGGGGAAGAAGAAGGGGGCGAGGGGGCCGAAAATCC
GACCAAGGTGAAGTCTTTATGCGGCCAAGCTCACGCCCATGCACCGCAGCGGATAAACCC
GGAACCAGAGAAACGGGTCGGGTCATGATCGACCCGGTTCCACCAAACGGGTCGGGTACA
AGTTTCCCCTGTTCAAGCAAGTCTTTGACTTTCCCAACTAGGCCTGGTTTTGGGCAGCTT
GGGACAAAATGTATTGTCAAGGCAAATCATTTCTTTGCTCAGTTGCCAGACAAAGACTTA

AACCAATATGATGTAACTATTACCCCCGAAGTCGCATCGAGGGCGGTAAATCGAGCTATA
ATAGCGGAACTCGTGAAAGTATACAAGGAATCCGATTTAGGGAAGAGATTACCTGCGTAT
GATGGGCGAAAGAGTCTTTACACCGCAGGGGAACTTCCTTTCGCTCGGAAGGAGTTTATT
GTTAAGCTTGTAGACGAAGAAGACACTATCAACGGCCCTAAGAGAGTGAGGGAATACAAA
GTAGCAATCAAATTTGTCGCACGTGCAAATTTACATCATTTGGAGCAATTTCTAGCCGGT
AAACGAGCAGATGGTCCAAAGGAGGCACTACAAATTCTCGACATTGTATTGAGAGAGCTC
TCCGTGAAAAGATTCTGTCCTGTCGGAAGATCGTTTTTTTCTCCGGATATAAGGAAACCG
CAAAGACTAGGTGACGGTTTGGAGGCGTGGTGTGGATTCTATCAGAGCATACGGCCCACT
CAGATGGGATTGTCACTTAACATAGATATGGCTTCGGCTGCATTTATTGAGGCTCTTCCG
GTTATCGAATTCGTCGCTCAATTATTGGGGAAAGATGTTTTGTCGAGGCCGTTATCCGAT
TCGGATCGCATTAAGGTTAAAAAAGCACTTAGAGGAGTAAAAGTTGAAGTGACTCATAGA
AGAGATGTTCGAAGAAAATATCGAGTTTGCGGCATTACGACTCAGCCCACTCGTGAGCTA
GTATTTCCGGTGGATGACGACTCGAACATGAAATCCGTGGTCGAGTATTTCCAAGAAATG
TACGGTTTCACAATACAGCACACTCATCTGCCTTGCCTACAAGTAGGGAATCAGAAGAAG
GCAAACTATTTACCCATGGAGGTCAGATGTTGTTGGCTTGTAAAAATTGTGGAGGGACAG
AGATATACTAAAAGGCATGTGATAACTGTTCAACACAATGCTTATGAAGAAGATCCGTAC
GCAAATGAATTCGGAATCAAAATCAGCGAAAAAATGACATCTGTCGAGGCCCGAGTTCTC
CCCGCTCCGTGGTTGAAATATCATGACACGGGAAAAGAAAAAGATTGCTTGCCCCAAGTT
GGCCAATGGAATATGATGAACAAGAAAATGATAAACGGCATGAACGTGAACCGTTGGGCA
TGTATTAACTTCTCACGGAGCGTACAAGATAGCGTTGCTCGTGGATTTTGCAATGAGCTA
GCTCAAATGTGTCAAGTATCGGGCATGGAATTTAATCCGGAACCCGTTATTCCAATCTAC
ATAGCTCGGCCGGATCAAGTCGAAAAAGCTTTGAAGCATGTTTATCATGCATGCACCAAT
AAATTAAAAGGAAAAGAATTGGAGCTTTTATTAGCTATTTTGCCCGACAACAACGGCTCT
CTATACGGTGATTTGAAGCGAATATGTGAAACCGATCTTGGTATAATATCCCAATGTTGT
CTCACGAAACACGTTTTCAAGATCAACAAGCAATATCTTGCTAATGTGTCCTTAAAAATA
AATGTCAAGATGGGTGGAAGAAATACGGTTTTATTGGATGCAATAAGCTGTAGAATACCG
CTAGTGAGTGATATACCTACGATTATATTCGGAGCTGATGTAACGCATCCCGAAAACGGA
GAGGAGTCGAGTCCATCTATAGCTGCTGTTGTAGCTTCTCAAGATTGGCCCGAGGTGACA
AAGTACGCGGGGTTGGTTTGCGCGCAAGCGCATAGACAAGAATTGATACAAGATTTGTAC
AAGACTTGGCAAGATCCTGTTCGTGGGACCGTGAGTGGCGGCATGATCAGGGATCTTTTG
GTTTCGTTTAGAAAAGCAACTGGACAAAAGCCACAGAGGATAATATTTTACAGGGATGGT
GTGAGTGAAGGGCAATTTTACCAAGTGCTACTATTTGAGTTGGATGCTATTCGCAAGGCT
TGCGCCTCGTTGGAGCCAAACTATCAACCACCGGTGACTTTTATCGTAGTTCAAAAACGG
CATCACACAAGACTTTTTGCTAACAATCATAGGGATAAAAGCAGCACCGACAAGAGCGGA
AACGTATTACCTGGTACGGTGGTCGATTCGAAAATCTGTCATCCTACAGAATTCGACTTT
TATCTTTGTAGTCATGCTGGAATTCAGGGAACGAGTCGACCTGCTCACTACCATGTACTC
TGGGATGAGAACAATTTCACTGCCGACGGAATTCAGTCACTGACGAACAATCTATGTTAT
ACATACGCCAGGTGTACACGCTCCGTTTCAGTTGTTCCCCCAGCTTACTATGCACATTTG
GCTGCATTCCGGGCGAGATTCTATTTGGAACCGGACTTGCCGGAAAGTGGCAGCGGCGGT
GGTGGCAAGGTGGGAAGAGTGGCGGGAGAAACGGGTGTGCGTCCTTTACCGGCTCTCAAA
GAAAATGTAAAAAGAGTAATGTTTTATTGTTGA
>Mimulus-guttatus_mgf015766m
ATGGAACCACCAACACCTCCGCCGCAAATTCTGCCGCCACCACCTCCCACCGGAATCCCG
TCAAAAAATTTTCCGATCAAAATTGAGCGCATCGAAAAATCTCCACCGATGAACTTCCCT
AGCCCGGGTTCGAAAGGGAAAGGAGTATCTTTATTAACAAACCACTTCAAAGTTACCGTC
GGAAGTAACGGTGTTGGTGATTTCTTTCACTATGATATATTGATATCTTACGAAGATGGC
CGTGAAGTAACTCGAAAGAATCTTAAGATGGCTTTGGTTGATAAACTATCGGAGGTTTAT
AAAACAGAGCTAGCTGGGAAAAGTCTCGTTTACGATGGAGATCGAAGCTTATTCGCCCTC
GGCCCACTCCCGCAAAACAACCTTGAGTTTTCTATTGTCCTAAATCCCGTTTTAGCAAAA
TGTGTTAGTGGGAATGAACAGAAGAGGCAGAGGCTTTCGTCTACTTCGAAAATGTTTAAA
TTGCGGTTGAAATTTGCGTCTCGAATATCGATGCAGAGGAGATTAATCGACTGCAGTAGC
TTAAGAGTGTTGAACGCAATTCTTCGACAACAAGCATTAAGACAGGGTTGCCTTGCCGTT
CACCAATCGTTCTTTTTGAACGATACGAAGAATTTAATCGACTTAGGTGGCGGCATCCAT
GGATGCAGAGGATTCTTTTCTAGCTTTAAAGCTCTTCAAGGTGGAATGTATTTGAACCAT
GATGTATGTATGACCACAATTGTACAATCGGGTTCGGTGGTGGATTTTCTTATTCGCAAT
CAAAATGTGAAAACTCCGTTCGAAATCGACTGGAATAAGGCTAGAGGGACGCTAAAAAAC
TTGAGGTTTAAAGTAAATCACTTGAAATTCGAGTTCAAAATCACGGGATTTAGCGACAGG
CCTTGCAAAGAACAGAAATTCTCTCTGAAGCTGGGAGAAGCAAACGTTGAGACTACGGTT
TACGATTATTTTGTGAAGACGCGTGGAATTAAGCTGAGTTTTAGTGCAAACTTACCATGC
ATCAACGCTGGGAAGCCTCGTAAACCGAAATTCTTTCCAATAGAGCTTTGTTCGTTGGTT
CCTTTGCAACGTTACAAGAACGAGTTAACCGGTTATCAAACTTCTGCAATGATTAAAAAA
TCAAGCCAAAAACCAGAGGAATTGCTTCAGTTTTTGGCTGATACTGGGAAAATGATTAAG

CATGAGGCTGAACCACTGTTGAAATCTTGTGGCATTTCAATCGACGGCTCGTTTGTTCAA
GCTGAAGGCCGTGTTCTTTCAGCCCCACGCTTAATAATGGGAAATGGAGAACATATCTTT
CCTAGAAATGGCTTCTGGAACATCAAAGATAAGAAACTTTTGGCGCCAAAAAAACTCGAA
AACTGGGCTGTGGTGAACTTCTCAACTAGCTGCGATATACGTAGTACGTGCCTAAAATTG
GCGAAAGTGAGCTCGAATTACGGAATGGTAGTAAGTCCTCCGGCATGCGTTATTCAAGAA
AATCCCAAGTATAAAAAGAAACCGGCTTCTGTTCGAGTGGAAGTGATGTTCCAACAAATA
AAATCGAAATTCCGACAAAATCCACCTCGCTTCATTCTTTGCCTCCTTTCCGATAAGAAA
TTCTGCGACTTATACGGTCCGTGGAAAAAGAAGACCATTGTAGAATTCGGAATTCTCGAT
CAATGCGTTTCGAATAATAAAATCGACGAGAAGTATCTCATGAATCTCATGCTCAAGATA
AACGCAAAGCTCGGTGGTTTTAATCATACTATTCTTTCGGAAATAACGAGAAGTATCCCA
TTTGTTAGTAAGATTCCTACAATGATATTTGGAATGGAAGTTTCCCACGCTTCTCCGGGG
GCCACGGAGAGACCTTCCATTGCTTCCGTTGTCGGATCAAGAGAGTGGCCGAGAATTTCG
AGTTATAGAGCGTGCCTTCGTGCACTCCCGCCGAAAGTTAAAATGATTGATTCGCTTTTT
AAGCCAATTTCTGAACAAAAAGATGCTGGCATATTCAGTGAGTTGCTGTTGGAGTTTTTC
GCGAGTTCCGGGAATAAAAAACCAGCTCAAATTATCATATTTAGGAATGGATTGAGCACA
ACAGAGTATAAACAAATTGTGAAGGAAGAAATGGATCAGATACTTAAGGCATGTAATTTT
CTCGAGGAGAAATGGCGTCCGAAATTCACAGTGATCGTCTCACAGAGACGACATCACACT
AAATTCTTCGATAAATCCAATTCGGGAGCTAATGTTTCTCCAGGAACTATTGTTGACAAC
AAAGTTTGTGATCTTCAATGTAATAACTTCTACATGAATGCTCATGCTGCAAGAATAGGG
ACTCCGAGGCCGACACATTACCATGTGCTGTTAGATGAAATAGGTTTCTCGTCGGATGAT
CTGCAAGAAGTTATTCATTCTTTATCATACGTTTTCCAGAGAAGCAACAACGCGATTTCC
GAAGTTGCTCCAGTTCGCTATGCTCGATTAGCAGCTGCTAAGATATCACAAGTAATTAAA
GCTGAGAAAATGACGAACACATCTTGTAACGGTCAAGGGGGTCAACTGCCAAAGCTGCAC
AAAAATGTGTCTTCTTCGATGTTCTTCATTTGA
>Mimulus-guttatus_mgf016487m
ATGCTGCCTCCACCACCGCCTGCCATACCGCCGAATGTTGTACCGCAGAAGGTGGTTCCG
GTTATTAAGCGCCTTCCGATGGTGAGGTCGGGATTCGGTTCGAAAGGGCAGCCCATCCCC
CTTTTGACGAATCACTTCAAAGTTGCTGTTTCTAAGACCGATGGGCACTTTTATCACTAC
AGTGTTGCTATCAAGTACGATGACGACACCCCTGTTGAGGCGAAAGGGTCTGGAAGAAAA
ATACTCGAGAAGGTGTACGACACGTATGCTGCTGAGCTGGATCGTAAGAGATTCGCGTAC
GATGGAGAAAAGACCCTCTTCACAGTCGGCCCACTGCCTCGAAACAAGTTTGAGTTCACG
GTGATCTTGGAAGATGGCCAATCGAATAGGAGCGCTGGGAATGGGAGCCCTGGAGCAGAT
GAGAGTGGCCCGAAAAGATCGAAGAGGCAGCCCCGTTCGAAGACGTACAAAGTGGCCATA
AATTTCGCTGCGAAAATTCCGATGCAAGCCATTGCAGATGCCCTAAGAGGACACGATTCT
GAGCATTTTCAGGACGCTGTGAGAGTGCTCGATATCATGTTGAGGCAGCACGCTTCTCAA
CAAGGCTGCCTTCTAGTACGACAGTCCTTTTTCCATAACGAGCCAAGAAATTTCGTGGAC
TTGGGGGGCGGTGTTGTTGGTTGTCGGGGTTTTCATTCGAGCTTTAGGGCAACACAAGGA
GGGCTTTCGTTGAATATGGATGTTTCGACAACTATGATTGTCAAGCCAGGGCCTGTGCTG
GACTTTCTCCTGGCTAATCAACGAGTCGACAATCCTGGTCAGATTGATTGGAGCAAGGCG
AAAAGGACGTTGAAGAATCTTAGGATCAGGGCCACTAATTCGAACTTGGAGTACAAGATC
AGTGGACTGAGCGACTCGATATGCAGGAAGCAAACGTTTCCTCTAAAACAGAGAAAAGAA
GGTGCAAACGAAGGAGACGAAGTACAAATCACAGTCTACGACTACTTCGTGAACCACCGA
GATATCAAACTGAGATACTCTGGGGATTTTCCATGTATCAATGCAGGCAAACCAAAGCGC
CCCACTTATATCCCCATTGAGCTATGTGAATTAGTTTCTTTGCAACGATATACCAAATCG
TTGTCGAACCTGCAACGAGCTTCGCTTGTGGAAAAATCTAGACAGAAGCCAATGGAGAGA
ATGAGCTCCTTGAAGCAAGCTTTGGCTACTAGCAATTATGGGGCTGACCCTCTTATAGTA
GCTTCTGGAGTTTCGATCTCCACTGAGTTCACAAAGATCCAAGGCCGCGTTCTGCCAGCC
CCAAAGTTGATGGTCGGTAAAGGGGAAGACCTTTGGCCACGTAATGGAAGGTGGAACTTC
AACAGCAAGAGATTGATTGAACCAGTAAAGCTCGATAGATGGGCAGTTGTGAACTTCTCT
GCTAGGTGTAATGTGAAAATGCTGAGCGAAAGCATCGTCAAATGTGGGGGAATGAAAGGA
ATGACTATTAGCCCACCCTTCGATTGCTTCGAAGAGAGCCCAAATAACAGGCGTAGCCCT
GCTGTGGTAAGAGTGGAGCAAATGATGGAGTTGATAAAGTCGAGGCTACCGGGCCCGCCT
CAGCTTCTTCTTTGCATATTGCCGGAGAAGAAAAACTGCGATCTCTATGGTCCATGGAAG
AAGAAGAATCTTTCTGACATGGGGATAGTCACTCAATGCATGGCACCGACGAAACCTGTC
AATGATCAGTACACCACCAATCTGCTCCTAAAGATCAATGCCAAGGAAATTTTCGTTCTT
GGCGGCATTAATTCACTTTTGGGTATCGAAAAATCGCCCTCGATTCCCCTGGTTTCCACA
GTGCCCACCCTTATTGTCGGGATGGACGTTTCACATGGTTCGCCTGGTCGATCAGATGTT
CCATCTATTGCTGCTGTGGTAAGTTCAAGGCAGTGGCCTTTGATATCTAAGTACAGGGCT
GCAGTTCGAACCCAGTCACCAAAACTAGAAATGATCGATTCGTTGTTCAAGAAAGTGGAT
GATGTGGATCAGGGCATTTTCAGGGAGTTATTGCAGGACTTCTTCATAAGTTCCGGCAAA
AGAAAACCCGAGCAGATTATCATCTTTAGGGATGGAGTGAGCGAGTCGCAGTTCAATCAA
GTATTGAATATAGAGCTGGAACAAATCATCGAGGCATGCAAATTTCTCGACGAAACTTGG

TCTCCCAAATTCATGGTTGTGGTGGCACAAAAGAACCATCACACGAAATTTTTCCAAGCA
AACGGACCCGATAATGTTCCACCAGGCACTGTAATTGACAATGGAATTTGCCATCCAAGA
ACAAACGACTTCTACATGTGTGCTCACGCTGGAATGATTGGCACAACTAGGCCAACACAC
TACCATGTGCTATTTGACGAGCTCGGCTTCTCTGCCGATGCTTTACAAGAACTTGTGCAC
TCATTATCCTATGTGTACCAGAGAAGTACTACAGCCATCTCTGTCGTTGCTCCGATCTGC
TACGCCCACTTGGCCGCTGCACAAATGTCTCAATTCATAAAGTTCGACGAAATATCCGAC
AAGTCATCGAGCCACAGTGGCACAGCGGGGCCCGGTGGATTATCAGTGGCCCCACTTCCG
CTGCTCCACCATAAGGTCTCGAGTTCCATGTTCTTCTGCTGA
>Mimulus-guttatus_mgf016601m
ATGTCGGGAATCGGTAGCGAAGGGCGAGGACGTGGCGGCGGTAGGGGACAACATCCTGCC
GGCCGTGGACAACAACCATCGCGCGGCGGCGGTCCAGGCCGTGGTCGGGGACGCGGTGCC
GGTGCTCCGCCGCAGCAGTCGCTTTCGGCTTCTGCCACTCCTTACCAGCAATCTCCGGTG
GCTTCTTCGTCCGTGGTTCCGGTGACCAAACCACCTGCTCCGACGCCTCAGCCACAGGCT
CCGGCGGTGGCGGCGGTGATTCCGCAGCAAGGACAGTTACCACCGGCTTCCACGAAGGCG
GTGAGGCTGCCGGCAAGGCCAGGCCTCGGATCATTCGGACAGAAGACCGTCGTTAAGGCG
AACCATTTTTTGGTCTCGGTTGCTGATAGAGATCTGAATCACTACGATGTTGCAATCTCG
CCTGAGGTTGCATCAAAGAAGGTATGCCGGATGATAATGAATCAGCTTGTCAAAACCTTT
CACTCCTCGCATTTGGGCAAGAGAAAGTTAGCATACGACGGAAGAAAGAGTTGCTACACT
GCAGGGCCACTGCCCTTTGCCTCTAAGGACTTTGTCATCAAGCTTGATGAAGATAGTGGA
TCCAGGAGGGAGCGCGAATTCAAGGTTTCGATCAAATTTGCTTCCAAAGCTGATCTCTAT
CACTTGAAGGAGTTCCTAAAAGGTCAACAACGTGATATCCCACAGGAAACTATACAGTTT
CTTGATGTGGTCCTTAGGGAGAAGCCATCTAATAGCTATGAAGCTGTTGGGAGGTCCTTC
TTTCATCCGGAGTTTGAATCTGGGGAGCTTGGTAATGGCCTGGAATACTGGAAAGGGTTT
TATCAGAGTCTTCGCCCAACACAGATTGGGCTGTCACTAAACATTGATATGTCGGCTAGA
GCATTTTTTGAACCTATTTATGTGTCTGAGTTTGTTTTCAAGTACCTCAACCTCAGGGAT
CCGAATAGGCCTTTATCTGACCAAGATCGCATTAAGGTGAGAAGAGCTCTGAAAGGTGTT
AAGGTTGAGAACAATCATCAGGACCATGTAAAGCATCATAAGATAACAGGGCTTTCGACT
GAACCAACACAAAGGCTTATGTTTTCTGTGGATGAGTCTGGAGCACAAATTTCAGTTTAT
CAGTACTTCCGTCAGAAATACAATATTGTGCTGAAGTATCCACTTCTGCCAGCACTTCAG
GCTGGCAGTGCTGCGAGACCTATTTATCTTCCAATGGAGGTATGCAAAATTGTCGCGGGG
CAAAGATACTCCAAAAAATTGAACGAGAGACAAGTTACTCAACTGCTCCGAGCCACATGC
AAACGTCCAGAGGAGAGAGAGAGAATGTCATTAAAAAGATGGTGGAAAAGAATAACTACAAC
AATGATGAACTTGTGAATGCAGAGTTTGGTATACAAGTTAGGCCTGACCTTCTGTCCATC
GAGGCACGAGTATTGCCGCCTCCAATGCTGAAATATCATGATTCTGGAAAGGAGAAACGC
GTTCAGCCATATGTAGGACAATGGAATATGATGAACAAGAAAATGATCAATGGTGGAACG
GTGGATTTTTGGACGTGTGTCAATTTCTCCCGGAGCAATGATGATGTTGTCACTAGATTT
ATCAATAAATTGCTTGAAATGTGCTGTGGCAAGGGGATGAAATTTGGTCAACATCCTCTA
GTTCCTATTCGTACAAGGCATTCTAACCAAATTGAGCAGTCCCTAAATGCCATCCACTCG
GAGGCTTTGAAAACTGGGAACAAACTTCAGTTGCTTCTTATTATATTGCCCGACGTGACT
GGTTCATATGGTTTAATCAAGAGGGTGTGTGAAACAGAATTAGACATTGTATCACAATGC
TGTCAGCCTAAACAGGTTTTGAAATGCAATATTCAGTACCTCGAAAATGTATCACTGAAG
ATCAATGTGAAGGCTGGCGGGCGAAATACAGTTTTGGAGCAGGCACTGCTTAGGAAAATG
CCTTACATTAGTGACATCCCTACCATAATATTTGGTGCTGATGTCACTCACCCTCAACCA
GGGGAGGATTCCAGTCCTTCTATAGCTGCTGTGGTGGCCTCAATGGATTGGCCAGAAGTC
ACTAAGTACAGAGGTTTGGTGTCTGCACAAGGCCACAGGGAAGAGATTATTCAGGATCTC
TACACTAGTTATCAAGATCCTAACAAGGGCTTAGTCCATGGTGGATTGATTCGTGAACAT
TTGGTTGCATTCTACAAGAACACTAAGTGCAAGCCTAGCAGGCTCATCTTTTACAGGGAT
GGTGTGAGCGAAGGACAGTTTAATCAAGTTCTTCTGTATGAAATTGATGCAATTAGGAAG
GCATGTGCTTCGCTTCAGGCAGATTATCAGCCAAGAATAACTTTTGTTGTGGTTCAAAAG
AGGCATCATACTCGTCTCTTTCCTGCTGACCATAGAAGTCGTAGTACTACAGACAAGAGT
GGTAACATTTTGCCAGGTACTGTGGTTGATACCAAGATTTGCCACCCCAATGAATTCGAT
TTCTATCTTTGCAGCCATGCTGGGATCCAGGGAACTAGTCGTCCAGCACATTACCATGTG
TTGTACGATGAAAATCGCTTCAGTGCAGATGCACTGCAAATTCTCACCAACAGCTTGTGT
TACACATATGCAAGGTGCACTCGCTCAGTTTCCATAGTCCCACCAGCTTACTACGCACAT
CTTGCTGCATTCCGAGCCCGTTACTACATTGAAGCGGGAGAATTCTCCGACAGTGGATCT
GCTGCAGCGGGCCCCGGTGGAGCGATGAGGGAGAGGGTTGGAGAGGTTCGGGCTCTGCCT
GCAATTATGGACAATGTTAAGGATGTCATGTTCTACTGCTGA
>Mimulus-guttatus_mgf019782m
ATGTCGGGAATAGGGAGAGAAGAACGAGGACGATCACGTGCCGCCGGCGGAAGGGGACCG
CAGCAGCCAACCGGCCGCGGCAAAAAACAACCATCGCGCGGCGGCGGTCCAGGCCGTGGT
CGAGGCCGCGGTAGTAGTGCTACGCCGGAGCACTCAATTTCAGCTTTTGTGGCTCTGTTC
GAGCAGTCTCCGATGGAGCAGAAGCTCACGTTTCAGGCTTCGTCTTCGACCACTGTTCCG

GCGCCTCGCCCACAGGTTACGGCGGTTTTGGCGGTGAATCCGCCGCAAGGACCGTCGAAG
AAGGCGTTGACTATCCCGGCAAGGCCAGGTTTCGGATCACTCGGACAGAAGACCATCGTT
AAGGCGAACAATTTTTTGGTTCCGGTGACTAAACCGCCTGCTCCGACGCCTCAGCCACAG
GCTCCGGCGGTGATTCCGCAGCAAGGACAGTTACCACCGGCTTCCACGAAGGCGGTGAGG
CTACCGGCAAGGCCAGGCCTCGGATCATTCGGACAGAAGACCATTGTTAAGGCGAACCAT
TTTTTGGTTGCGGTTGCTGATAGAGATCTGAACCACTACGATGTTGCTATCTCGCCTGAG
GTAACCTCGAAGAAGGTATGCCGGATGATAATGGATGAGCTTGTCAAATCCTTTAACGCT
TCAAATTTGGGCAAGAGACAATTGGCTTACGATGGAAGGAAGAATTGCTACACTGCAGGG
CCATTGCCCTTTGTCTCTAAGGAGTTTGCCGTCACGCTTGATCAAGATAGTGGATTCAGG
AAGGAACGCGAATTCAAGGTTTCCATTAAGTTTGCTTCCAAAGCTGACTTACACTACTTA
AAAGAATTCTTACAAGGCAGACAACTCGATAATAATCCAAATGAAACTATCCAGTGTCTT
GATGTGGTGCTTAGGTCGAAACCATATAATAATAGCTATGAAGCTGTTGGAAGGTCCTTC
TTTCATTGGGACTTCGGATCAGGGAAACTCGGTGATGGCCTGGATTACTGGAAAGGATTT
TACCAGAGTCTTCGCCCAACTCAGATGGGGCTGTCACTGAATATTGATATGTCCGCTAGA
GCTTTTATTGAACCTATTTATGTGTCTGAGTTTGTTTTCAAGTACCTCAACCTCAGGGAT
CGGAATAGGCCTTTATCTGACCAAGATCGGATTAAGGTCAAAAGAGCTCTTAAAGGTGTC
AAGGTTGAGAGCATATATCTGAAGTACACCAAACGTTTCAATATCACAGGCGTGTCGACT
GAACCCACTGAAAGCCTGATGTTTACCATCGATGCATCCGGTGCACAAATATCTGTTTCT
GAGTTCTTCCGTCAGCAGCACAGTATCGTGCTTATGTATCCACGTCTGCCTGCTATTCAG
TCCGGCATTGGGAGACCAATTTATATTCCAATGGAGTTATGCAAAATTGTTGAGGGTCAA
AGATACTCTAGGAAATTGAACGGGAGACAAGTTACTGCCCTGCTCCAAGCCACATGCGAA
CGGCCAGCGAAGAGAGAGGGTGTCATTAGAACGATGGTGGAAAGAAATAATTACAACCAT
GACGAACTTGTCAATAGAGAGTTTAGTATACAAATTAGGCCTGAACTTATGTCCGTAGAG
GCACGGGTTTTGCCTCCTCCAATGCTGAAATATCATGATTCTGGAAGGGAGAACCGTGTT
CAGCCAAATGAAGGACAATGGAATATGATGAACAAGAAAATGATCAATGGTGGAAAGGTG
GATTTTTGGACTTGTGTCAATTTCTCCCGAAGCAAAGATCATGTTGTCAAGAGATTTATC
AATGAACTGGTTACTAAATGCCTCAGCAGAGGGATGGAATTTAGTCCACATCCTCTAGTT
CCTATTCGTACAAAGCCTTCTAACCAGATTGAGAAGTCCCTAATCGGTATCCACTCAGAC
TGTTTGAAAACTAGGAAACAACTTCAGTTGCTTCTTATTATATTGCCCGATGGCAACGTG
ACTGATTCATACGGTTTAATCAAGAGAGTGTGCGAAACCGAGTTGGGCTTTGTGTCACAA
TGCTGTCAGCCCCAACATGTTTTAAAATGCAATAATCATCAGTACTTGGAGAATGTTTCT
CTCAAGATCAACGTTAAGGTTGGAGGGCGAAATACGGTGCTGGAGAAGGCACTCTGTAGA
ACAATACCTTTCGTTAGTGACATGCCTACCATAATATTCGGTGCTGATGTCACTCATCCT
CAACCAGGGGACGAATCCAGTCCTTCTATAGCTGCGGTGGTGGCCTCAATGGATTGGCCA
GAAGTCTCTAAGTACAGAGGCTTGGTTTCTGCACAGGGCCATAGGGACGAGATTATCCAG
GATCTCTACATTAATAAGGACTCAGGAATGATTCGCGAACACTTGGTTGCATTCTACCAG
AACACTAAGTTGAAGCCTAGCAGGCTCATCTTTTACAGGGATGGTGTGAGCGAAGGGCAG
TTTAATCAAGTTCTTCTGTATGAAGTTGATGCAATTAGGAAGGCATGTAATGAACTTCAG
ACAGACTATCAGCCAAGAATAACTTTTGTTGTCGTTCAAAAGAAGCATCACACTCGTCTC
TTTCCTGCTGACCATACAAGTCCTAATACTACAGACAAGAGTGATAACATTTTGCCAGGC
ACTGTGGTGGATACCAAGATTTGCCACCCCAACCAATTGAGTTTCTATCTTTGCAGCCAT
GCTGGGATCCAGGGAACGAGCCATCCAGCACATTACCATGTGTTGTTCGATGAAAATAAA
TTCAGTGCAGATAGTATGCAAATGCTTACCTACAGCTTGTGTTACACATATGCTAGGTGC
ACTCGATCAGTATCCATAGTACCACCAGCTTACTATGCACATCTTGCTGCATTTCGAGCA
CGGTATTACATCGAAGCTGGGGGAGATCTCTCTGACAGTGGATCCGCTGCAGCGGGCCCC
GATGGAGTTATGAGGGAAAGGGTCAGAGAGGTTCGGGCTCTGCCTGCAATTATGGACAAT
GTTAAGGATGTCATGTTCTACTGCTGA
>Mtruncatula_Medtr2g034460
ATGTTGACCTTCTATATATCAGACAATTTAGCTTCATCATCTCCGGCACTTGAAAAAGAG
GATAAAACTTCACCAATTCGGAGACCTGACAGTGGTGGCACATTAGCGGTTCATACAAGC
ACACTCCGTGTCAATCATTTCCCTGTTAAGTTCGATCCAAGGAGTATAATATTTCATTAT
AATGTTGCTGTCAAACCAAAGTTTTCCTCAAAAGTTGGCCAGCCTAAGAAGTTATCTAAA
AATGATCTTTCCATGATTAAAGAGAAATTGTTTTCTGATGATCCTGAGAAGTTTCCTTTG
GACATGACTGCACATGACGGTGCAAACAATATCTTTAGTGCAGTGCAGTTACCGGAAGAG
ACCATTACCGTGGAGATCTCTGAAGGAGAGGACGAAAAAATCACTACATATAGTGTTACT
ATAACACTTCTTAATAAACTCCGACTTCACAAGTTAATGGACTATCTTTGTGGGCATTCA
TTCTCTCTGCCTCGGGATATTTTGCAAGGGATGGACGTGGTAATAAAAGAGAACCCCGTT
AGGCGCACAATTTCTGTGGGAAGATACTTCTATCCCACAAATCCTCCTTTGGTAATGAAG
GAACTTCGCCCTGGAATAATCGCAGTCGGGGGGTTTCATCATAGTTTAAAACCAACATCT
CAGGGTCTGTCCTTATGTGTAGACTACTCAGTGGTGCCTTTCCGAAAGCAAATGTCAGTC
GTGGATTTCTTGCATGAGCGTATTGATAACTTTAACTTGGGTGAATTTGAAAAATTCAGG
AAATACGTTGAGGAAGTGCTTATTGGATTGAAAGTTAGTGTGACACACCGTAAATCCCAA

CAAAAATATATCATTGCAGGATTAACACCTACAGTTACAAGGTATGTCACTTTCCCCATT
GACCATACTAAGGGCTGGAAACTTGAAAAGGAAGTCGGTCTTCTTAGCTTTTTAATGAC
AAATATGACAAGGACATTGTATACAAAGATATTCCTTGTTTAGATTTAGGGAAAGGCAAC
AAGAAGAACTATGTACCGATGGAATTTTGTGTTTAGCTGAAGGCCAAAGGTATCCCAAG
GAGCGTTTAGATGGTATTTCTGCAAAGACATTGACGGCAATGGCTCTAGCTCACCCAAGT
GAGCGGCAGGGTGCTATACAGAAGATGGTGCAATCTAGTGATGGACCTTGCGGTGGTGAT
CTTATTCAGAACTTTGGAATGAGAGTCAGCACGACCATGACAACTATTCTCGGACGTGTA
ATTGGCCCTCCGGAATTGAAGTTGGGTGATCCAAATGGCAAGAATGTCAAAATAACAGTG
GATCTAGATAAATGTCACTGGAACCTTTCTGGAAGATCAATGGTGGAAGGCAAACCAGTT
GAGCGTTGGGGCATTCTTGATTTCACCAGTATTGGACCGTATAATAGAAAATTAAGACGA
AAGGAATTCGTTGAAAAGCTTATAGGTAAATACAAAAAGTTGGGTATCTATATGCAGGAG
CCCATTTGGTATGAAGAATCTTCAATGAAGATACTTACGAGTCATGACTTGCTATCTGAA
TTACTTGAAAAAATCAACAACATTTGTAAATATAACCAAGGTCGCTTACAATTTCTTCTC
TGCGTGATGGCTAATAAAAGTCCAGGTTACAAATACCTCAAGTGGATTTCTGAGACCAAA
GTTGGTATAGTGACACAATGTTGTTTGTCTTATAGTGCTAATCAAGGGGATGACAAATTC
TATACTTATCTTGCTCTCAAAATCAATGCCAAGCTCGGAGGCAGTAATGTAGAGCTCAAT
AACAGGCTCCCTTACTTTGAGGGTGAAGAACATGTTATGTTTATAGGGGCTGATGTCAAT
CACCCTGGTTCGCGAGACAACAAGAGTCCATCAATTGTTGCTGTGGTTGCAACCATTAAC
TGGCCTGCTGCAAATCGTTATGCGGCACGTGTTTGCCCACAATTCAATCGTAGTGAGAAA
ATATTGAACTTTGGGGAGATTTGTGTTGAGCTTGTTAGTTGCTATTGGCAGAAGAATGGA
GTCAGGCCTGAAAAAATTGTTGTTTTTCGTGATGGGGTGAGCGAGTTCCAGTTTGACATG
GTTCTTAATGAAGAGCTACTTGATTTGAAGAGAGCATTCCAAAGATTAAATTATTTCCCA
ACGATCACTCTTATTGTTGCACAAAAACGACATCAAACTCGATTTTTTCCAGACAGTTGG
AGGGATGGGTCTTCTAGCGGCAATATTTTACCGGGAACGGTTGTTGACACAAAAGTTACT
CACCCCTTTGAGTTTGACTTTTACCTTTGTAGTTACTATGGAAGCCTAGGTACAAGCAAG
CCCACCCACTATCATGTTTTATGGGATGAGCACAAGTTTACATCTGATGAATTGCAGAAA
CTTATTTATGAGATGTGCTTCACCTTTGCAAGGTGCACTAAACCTGTGTCTCTAGTCCCT
CCTGTGTATTATGCTGACCTTGCTGCTTATAGAGGACGATTATACCACGAAGCAAAGACT
GGGATGCAACCTAAGAAGTCAAGAACATATTTGTCTTCTAAAGATTCATCATCGATTCCA
CCGACAGCTTCGTTTGAACAGGGATTTTACAGGCTGCATGCTGACCTGGAAAACATTATG
TTCTTCATCTAG
>Mtruncatula_Medtr3g105930
ATGGTGAGTGTACCTCACTTTGAAGAAGCTGTTGAGCCACCAGCTTTACCTCCATCACCT
CCTGATGTGCTGTCTAGTATGGAAGCGGAACCAATAGCTCTCCCAACCTATTCTATTATC
AGTAGGCCGAGTGTCGGAACCACAGGAAAACGCATACATTTGCTTGCAAATTTCTTTAAA
GCTGCTGCCGATGCTACAGATGCAACCTTTTCCCAGTATAATGTATGTCAAGTGATTCTG
TCATTGCAATTTCCCTTATTTGGCTTTGGCTTTGGCTTTGGCTTATTGAAGTTTTTCCAG
GTTGCTGTCACTTCAGAAGATAAAAGAACTGTTGAAAGCAAGGGCATCAGGAGAAAACTG
ATTAATAGGCTTCACCAAACATACTCTTCTGAACTTGGTGGCAAAAGCTTTGCGTACGAT
GGAGAAAGAACTTTGTACACAGTGGGTCCTCTTCCAGATAATAAATTTGAGTTCAATGTA
TTTCTTGAAGAAACATTTGCAAGCAGCACTGAGAGTTTTGGTGCTAATGGAAGCCCTCGC
GAGGAAAATAAAAGGTCAAAACGTTCTTTTCAGTCGAAGACATTCACAGTGGAGATAAGT
TTTGCTGCCAAAATACCCTTGCAGTCCATTGTTCTTTCCCTTAAAGGGATCGAGTCATAT
GCTAATTCTCAGGATGCATTGAGGGTTCTTGATACCGTACTGAGGCAGCAAGCAGCTAAC
AGAGGGTGTCTCTTGGTAAGGCAATCTTTTTTTCATAATGACTTGAGGAATTTCATCGAT
GTTGGAGGCGGAGTAGAAGGAGTTCGGGGTATTCATTCCAGTTTTCGTCTTACAGAGGGA
GGATTGTCTCTTAATATGGATGTGTCCACAACAACGATTGTAAAACCTGGACCTGTAATT
GATTTTCTCCTATCCAACCAGAGTGTGAGGGAACCTCGTTATATTGACTGGGCAAAGGCC
AAGAGAATCCTTAAAAATTTAAGAGTTCGTGCTACACATCGTAACCAAGAATTCAAAATT
TCGGGCATGAGTGAGAAACCCTGCATTCAACAACTTTTTAGTATGAAGATGAAAATTGGA
GAAGACAATAACACGGAGCAGACAGTGGATATTACTGTATATGAGTATTTCGCTAAACAC
CGTGGCATTGAGCTGACCTCTTCAGCTTACTTTCCATGTCTTGATGTTGGGAAGCCAAAT
CGACCCAACTTTTTGCCCCTGGAGCTATGTTCACTTGTTCCCCTTCAGCGGTATACAAAG
GCATTATCTCCTGTGCAAAGAGCATCTTTAGTAGAAAAATCACGCCAAAAGCCTCAAGAA
AAAATCGAAGTTCTGACAAATGCTATTGGAAATTCTGGCTATGATGATGATGCTGTTCTT
GCCGCATGTGGCATTTCTATTGACAAGCAATTTACTCCAGTTGAAGGTAGAGTTCTTGAG
GCACCAAAGTTGAAGGTAGGTAAGAATGAGGACTGCTTTCCCAATAATGGAAGGTGGAAT
TTTAAAACGAAGAAATTTCTACAACCATCACATATCGGTTATTGGGCTGTTGTAAACTTT
TCTAAACAATGTGATACTAGTTACATAACAAGGGAGCTGATTAAATGTGGAATGAGCAAG
GGAATGAATATTGAACGGCCATTTACTTTATTAGAGGAAGAAGCACAAATGAGAAAATCT
AACCCTGTTGCACGGGTTGAAAAGATGTTTTCCTTGCTGCAATCAAAACTTACTGATGAT
CCAAAGTTGATTCTTTGTGTCTTGCCAGAGAGGAAAAACTGTGACATCTATGGGCCTTGG

AAAAGGAAGTGTCTGAGTGATGTTGGGGTTGTCACACAGTGCATTTCCCCTCTCAAGATC
ACTGATCAATACCTTACTAACGTACTTCTTAAAATCAATTCTAAGCTTGGAGGAATAAAT
TCTTTGCTGGCAATAGAGCATTCTGGGCATCTTCCCTTGATTAAAGATACCCCAACAATG
ATTTTAGGGATGGATGTCTCTCATGGATCCCCTGGCCGATCAGATATTCCATCAATAGCT
GCTGTTGTTGGATCTCGATGCTGGCCTCTAATTTCGAGATATAGAGCATCTGTAAGATCA
CAGTCTCCTAAGGTGGAGATGATTGATTCTCTATTCAAGCTTGTGGATAAGATGAATGAT
GATGGTATTTTAGAAAAAAAGGATGATGGTATTATCAGGGAATTGCTTCTAGATTTCTAT
AGTTCAAGTGGTAACCGCAGACCAACTCAAATTATTCTCTTCAGGGATGGAGTTGGTGAA
TCTCAATTTCAACATGTTTTAGATATAGAGCTTAACCAGATAATAAAGGCCTATAAACAT
ATTGATGGGGATGTTCCCAAGTTCACTGTAATTGTGGCACAGAAGAATCACCATACAAAG
CTGTTTCAAGCTAATGCTCTGGAAAAAAACGTTCCTCCTGGGACAGTTGTTGATACAAAC
ATTGTGCATCCAAGAAATTACGATTTCTACATGTGTGCTCATGCTGGAATGATTGGAACG
TCTAGGCCTGTGCATTATCATGTGTTGCTTGATGAAATTGGATTCTCGTCAGATGGCTTG
CAAAACTTGATCAATTCGCTGTCTTATGTGAACCAGAGGAGCACAGCTGCAACCTCAATT
GTGGCACCTATATACTATGCCCACCATGCTGCAGCTCAAATGAGAAAATTTATGAATTTT
GATGATTTATCAGAAGCATCTCCAAGTCCCGATTCAGAGGGAAACATCCCCATTCAAGAG
CTTCCAAAGTTACATTCAGATGTCAGGGACTCCATGTTCTTCTGTTGA
>Mtruncatula_Medtr4g114860
ATGGAAAACTGTTTTTCCCTTCAAAAATATTGCAGCAAACAGGCTCTAAATACTAGTGAA
AATATCAACTTATATCTGAGAGGGAAGATGTTTTTCTGTGCAGATATTGGAGCTCTGAAA
ATAAAAGAGCAGCCCGTGGAAAATACCACAATTGGACCCATGTTCCGGCCTGACAAGGGA
GGGACAGTGTCAATTCGAGACTGCAGACTTCGTGTCAATCATTTTCCGGTTGCTTTCAAT
CCACAAAGCATTATCATGCACTACGATGTGGACGTGAAGGCTTCTGTGCCACCGAGAAAG
GGTTTGCCTCCAAAGAAGATTTCCAAGTCTGACTTGTCTATGATCAGGGACAAATTGTGT
GCTGATCATCCTCAGATACTGCCTTTGTTGAAGACTTCATATGATGGAGAGAAGAACATC
TTCAGTTCTGTGCCATTGCCTGAAGAAACTTTTACCGTGGAGGTCTCAAAAGGAGAAGAT
GAAAGAGCTGTTTCTTATACGGTTACTATAACACTGGTGAACAAACTCGAGCTTCGTAAG
TTGAGAGATTACCTTAGTGGCAATGTGTATTCCATTCCGAGGGATATTTTGCAGGGAATG
GACTTAGTGGTGAAGGAGAATCCGGCAAGGCGCACAGTTTCTTTAGGACGGTGTTTCTTT
CCCACCAATCCTCCCTTAATACAGAGAGATCTTGAACCCGGAATAATTGCAATTGGAGGA
TTTCAACACAGTCTCAAGACCACGGCTCAGGGTTTAGCTTTGTGTCTTGATTATTCAGTT
TTGTCTTTTCGAAAGAAAATGTCAGTCTTGGATTTCCTCCATGATCATATTAGAGGTTTC
AATTTGGCTGAGTTCAGGAAATATAAGAAATTTGTCGAGGAGGTACTCTTGGGATTGAAA
GTAAATGTTACTCACAGAAGAACCAAACAGAAATATACTATTGCTAAGCTAACAGATAAA
GATACTCGCCACATCACTTTCCCTATTTTGGACCAAGAGGGCCAAACCCCCCCTAGAAGC
ACCTCTCTTCTTGCCTACTTTAAAGATAAACATAACTATGATATTCAACACAAAGATATT
CCTGCATTGGATTTTGGGGGAAACAAGACTAATTTCGTGCCTATGGAGCTATGCGTCTTG
GTTGAGGGTCAGCGATTTCCCAAAGAGTATTTGGACAAGAATGCTGCCAAGAACTTGAAA
AATATGTGCTTGGCTAGTCCAAGGGACAGGGAATCTACAATACAAATGATGATGAAGTCT
AGTGATGGACCGTGCGGCGGTGGTATTCTTCAGAATTTTGGAATGAATGTCAACACTTCC
ATGACAAATGTGACCGGACGTGTAATTGGACCTCCAATGTTGAAGTTAGGCGATCCACGT
GGAAAGAGTACTCCTATGAAACTGGATCCAGAGAAATGCCATTGGAATCTTGTTGGAAAG
TCAATGGTAGAAGGAAAAGCTGTTGAATGTTGGGGCATCCTTGATTTACCAGCGATGCA
CCTAATTGGTGCAAATTAAGAGGCAATCAGTTCGTTAACAATCTTATGGATAAGTACAGG
AAGTTGGGGATTGTCATGAATGAACCTGTTTGGCATGAGTATTCTGCAATGTGGAAACTT
GGGGATTACAATTTACTATGTGAGTTACTTGAAAAAATAAATGAGAAGGTTCAAAAAAAG
TGTCGACGGCGGCTACAATTTCTCCTTTGTGTGATGGCCAACAAGGATCCAGGTTACAAG
AGCCTCAAGTGGATTGCTGAGACCAAGGTTGGCATAGTGACACAGTGCTGCTTATCTGGT
AATGCTAATGAAGGGAAAGACCAATATCTCACAAATCTTGCTTTAAAAATCAATGCAAAA
ATTGGAGGCAGTAACGTTGAGCTCATTAATAGGCTCCCACACTTTGAGGATGAAAGTCAT
GTTATGTTTATAGGGGCTGATGTCAATCATCCAGGTTCCCGGGACACAAATAGTCCATCA
ATTGTTGCAGTGGTTGCCACTACTAACTGGCCAGCTGCAAATCGCTATGCAGCACGTGTT
TGCGCTCAAGAGCATTGTACAGAGAAAATTTTGAATTTTGGAGAGATTTGCCTTGACCTT
GTTAGACATTATGAGAAGTTGAACAAAGTCAGGCCCCAAAAAATTGTTATCTTTCGTGAT
GGTGTTAGTGAGAGCCAATTTCACATGGTTCTTGGCGAGGAGTTAAAAGATTTGAAGACC
GTGTTTCAGCACTCAAATTACTTTCCAACTATCACTCTTATTGTAGCTCAAAAGCGCCAT
CAAACTCGATTGTTTCCTGCCGGTGTAAGGGAGGGGGCTCCCAGTGGAAATGTGTTCCCT
GGAACAGTTGTGGACACAAAGGTCGTACATCCTTTTGAATTTGACTTTTACCTGTGTAGT
CACTATGGAAGCCTAGGTACAAGCAAGCCCACTCACTATCATGTCTTGTGGGATGAGCAC
AGGTTTACTTCTGATAATTTGCAGAAGCTCATATATGATATGTGCTTTACCTTTGCAAGG
TGCACTAAACCTGTATCTTTAGTCCCTCCAGTGTACTATGCTGACCTTGCTGCTTACAGA
GGAAGACTATACTATGAAGCAAAGATGTCAACTCAATCTCCATATTCAACTGTATCTTCT

TCATCATCACCTTTAGCTTCTTCATCCATTTCATCCACCGCCTCAATTTCAAATGATCCA
GGGTTTTACAAGCTGCATCCTGATACGGAAAACGGAATGTTCTTCGTCTGA
>Mtruncatula_Medtr5g045600
ATGGAACAACAAGAAAACTCAACCAACAACACTAACCAAAACAAAACAACCACTTTCATC
ACAAAAAATGGAACAAACTCTCATCAACATCATCATTACTATAATCACTATCAACAGCAA
CAAGAACAACAACAGTATCAGAATCATCTTCAACAATATCAAACTCAACTTGGTTTCTAC
AACAACTACTACCAAAACAAGTATCAAAGATATTACCCTGCTCTTCTTCCTTTACCTTCA
CTTCAACAACTACCTTTCATTCCATCTTTCCCTCAAAATCTCAAAACCCATTTGCACAAA
CTTCCATGCAAACTCAATACTTCTCCTTCATCTGAATACAATCTTTCTCAACTTTCACTT
GATCCTGCTCCAAAGGAACTTCAGAAACAAACTAGACCATCCTTTAAAGAAGTTGATGGG
AAGAAGCTTATTTCAACAAGGAAGCCACATGAAGTAATTGTTGCAAGGAGACCAGATTCT
GGTGGACAAGAAGGTCCTGTGATTTCTCTCCTTGCCAACCATTTTTTGGTGAAATTTGAT
TCATCACATAAAATATATCATTACAATGTTGAAATTACTCCTCATCCCTCTAAGGATGTT
GCTAGAGAAATCAAGCATAAATTGGTAAATAACAATGCTGAGATTTTATCGGGCGCTCTG
CCGGCATATGACGGGAGGAAAAATCTTTATAGTCCAATTGAATTCCAAAATGATAAGCTT
GAGTTCTACATAGGCCTCCCGATCCCGACTAGCAAATCGACATCACCTTATGAGAAACGT
GAACAACATAAACTTTTTCGGATAAATATCAAACTAGTCTCGAAGATTGACGGGAAGGGG
TTGACTAATTACTTGAGCAAAGAAGGTGATGAAGGGATTCCGCTTCCGCAGGATTATCTA
CATGCTTTGGATGTAGTTTTGAGGGAAAGTCCGACTGAGAAATGTATACCTGTTGGAAGG
TCATTCTATTCGAGTTCAATGGGGAGAAGCAAAGATATCGGTGGAGGAGCTGTTGGATTG
AGAGGCTTCTTTCAGAGTCTTAGACCAACACAACAAGGACTTGCTCTCAATGTGGATTTC
TCGGTAACTGCTTTCCATGAGAGTATAGGAGTTATTCCGTATTTGCAGAAACGTCTCGAG
TTTCTTAGAGACCTTTCTCAGAGGCAGACAACTCAGCTAACTTGTGAAGAAAGGAAGGAA
GTAGAGAAAACGTTGAAGAATATCAGAGTCTTTGTTTGCCATAGAGAAACTGTTCAGAGA
TACCGTGTCTATGGCTTAACTGAGGAGGCAACTGAAAATCTTTGGTTTCCTGATAGAGAT
GGAAAGAATCTGAGGCTTATGAGTTACTTTAAAGATCACTATAACTACGACATTCAATTC
AGGAAGTGGCCATGCTTGCAAATTAGTAGAAGTAAACCTTGTTATCTCCCTATGGAGCTT
TGTGTGATCTGTGAAGGACAGAAGTTCCTTGGGAAACTGTCGGATGATCAGACGGCAAAA
ATACTCAAGATGGGCTGTCAAAGACCAGGAGAACGGAAAGCCATCATCGAAGGCGTCATG
AGAGGAAATGTTGGTCCTACCAGTGGTGATCAGGAAAAGGAATTTAAACTCCAAGTCTCA
AGAGAAATGACAAAGTTGACTGGGAGAATTCTTTACCCTCCAAAACTAAAGCTTGGAGAT
GGAGGTCATGTGAGAAATCTGACTCCTTCACGTCATGATCGCCAGTGGAACTTTCTAGAT
GGCCATGTCTTTGAAGGAACTACAATTGAAAGGTGGGCACTAATAAGTTTTGGAGGCACA
CCTGAGCAAAAGTCTCATATCCCAAGATTCATAAACCAGTTAACTCAAAGATGTGAACAA
TTAGGCATTTTTCTCAACAAGAACACAATTATTAGTCCACAGTTTGAATCAATCCAAGTT
CTTAACAATGTCACAGTTTTGGAATCTAAGCTCAAAAGAATCCAGAGTATTGCCTCAAAC
AATCTACAGCTTCTTATTTGTATAATGGAGAAAAAACACAAAGGGTATGCAGATTTGAAA
AGAATTGCCGAGACAAGTGTCGGTGTTGTAAGCCAGTGCTGCTTGTATCCGAATCTCATC
AAATTAAGTTCACAGTTTTGGCTAATTTGGCTCTCAAGATCAATGCTAAAGTTGGTGGA
TGCACTGTTGCTTTGTACAACTCGCTTCCTTCTCAATTACCGCGTCTTTTCAACATCGAC
GAGCCGGTGATGTTCATGGGAGCCGACGTCACGCATCCTCATCCGCTCGACGATTCAAGT
CCATCTGTCGCTGCTGTTGTTGGTAGCATGAACTGGCCAACAGCAAACAAATATATTTCA
AGAATAAGGTCTCAAACACACAGGCAAGAAATTATTGCAGATCTCGGTGCAATGGTAGGA
GAATTGCTTGAAGATTTTTATCAAGAAGTGGAAAAACTCCCAAACCGAATAATTTTCTTC
CGAGACGGTGTTAGCGAAACTCAGTTTTACAAAGTTCTGCAAGAGGAACTACAATCAATA
AAACAAGCATGTTCATCAAGGTTTCATGGTTATAAACCTTTTATTACTTTTGTAGTTGTG
CAAAAGAGGCATCACACAAGGTTGTTTCCTGCCGACACCGATCAATCTTCGATGCACAAC
AATTTTCACTTTCAATATGAAAATATTCCACCAGGGACTGTGGTTGATTCAGTGATTACT
CATCCAAAGGAATTTGATTTCTATCTATGTAGTCATTGGGGTGTGAAGGGAACAAGTAGG
CCAACTCATTACCATGTCTTGTTGGATGAAAATAAGTTTACTTCAGATGAACTGCAAAAG
CTTGTTTACAATTTGTGTTTTACTTTTGTTAGATGTACTAAGCCAATTTCATTAGTTCCT
CCTGCATATTATGCACATTTAGCTGCATATAGAGGTAGACTCTACCTTGAGAGATCGGAG
TCCTTAGGTTTGTTCAGAAGTGCTTCTACATTATCAAGAGCTGCTACGCCGAAGACACCA
CCTCTACCTAAACTTAGTGAAAACATCAAGAAGCTTATGTTTTACTGCTAG
>Mtruncatula_Medtr5g094930
ATGAAATGGACCAGTGTTCTATTCTCTAATTTTATTCATTTCACAGCACAAAACACACTT
CACTCTTTTTTCTCATTTCTCTCTCAAACCTTCTTCAAGGAAATGGGTTCACAGGAGAAC
GAAGAATGTTTACCCCCGCCACCTCCCATTGTCCCAGCAGACATTGAACCAATCAAGATT
GAGCCGCAGATAGTTAAGAAAAAACTTCCCACCAAGGTTCCAATGGCAAGACGTGGACTT
GGGTCTAAGGGAGCTAAACTGCCTCTGCTTACTAATCACTTTAAAGTGAATGTTACCAAC
ACTGATGGTTATTTCTTTCAGTACAGTGTTGCTCTTTTTTATGAAGATGGACGCCCTGTT
GAAGGAAAGGGTGCTGGTAGGAAGATACTTGATAGAGTTCAAGAAACTTATGGTTCCGAG

CTTAACGGGAAAGACCTTGCTTATGATGGGGAGAAGACTCTGTTTACTATTGGCTCACTT
GCTCAGAACAAGCTTGAATTTACTGTTGTTCTTGAAGATGTAACTTCAAACAGGAACAAC
GGTAATGCTAGCCCTGATGGACACGGAAGTCCAAATGATACTGATAGGAAGAGGTTGAAA
AAATCACACCGATCAAAGACCTACAAAGTTGAAATCAGCTTTGCCTCAAAGATCCCTCTG
CAGGCCATCGCCAATGCCTTAAAAGGGCATGAAACTGAGAATTATCAAGAAGCGATTAGG
GTTCTTGACATTATCTTGAGGCAACATGCTGCCAAACAGGGCTGTCTGCTTGTCCGCCAA
AACTTTTTCCACAATGATCCGAAAAATTTCACAGATGTAGGAGGTGGTGTTCTTGGATGC
AGAGGGCTGCATTCTAGCTTCAGGACCACACAAAGTGGGCTGTCTTTGAATATAGACGTG
TCAACAACCATGATTGTCCATCCTGGGCCTGTGGTTGATTTCTTGATTGCAAACCAGAAT
GTGAGAGATCCTTTCAGTCTTGACTGGAATAAGGCTAAAAGGACTCTAAAGAATTTGAGG
ATCACTACTAGCCCAACCAACCAAGAGTACAAAATTACTGGTTTAAGTGAAATGCCATGC
AAGGACCAGCTGTTTACTCTGAAGAAGAGGGGTGCTGTTCCTGGAGAAGATGATACCGAG
GAGATCACTGTTTATGATTATTTTGTCAACCGCAGAAAAAATTTCTCTACAGTACTCAGCT
GATCTGCCATGTATTAATGTGGGAAAGCCAAAGCGACCTACATTTGTCCCAGTTGAGCTT
TGCTCATTGGTATCCCTTCAACGATATACCAAAGCCCTGTCCACACTCCAGAGATCCTCC
CTTGTGGAAAAGTCTAGGCAGAAGCCTCAAGAGCGGATGAGGGTCTTGACGGATGCCTTA
AAAACAAGTGACTATGGCTCTGAACCCATGCTACGTAACTGTGGAATTTCAATAACATCT
GGGTTCACACAAGTTGATGGGAGGGTTTTGCAGGCTCCAAGGTTGAAGTTTGGGAATGGC
GAAGACTTCAATCCCAGGAATGGTAGATGGAACTTCAACAACAAGAAAATTGTGCAACCT
GTTAAAATAGAGAAATGGGCTGTGGTGAACTTTTCTGCGCGTTGTGATGTGCGAGGACTT
GTGAGGGATCTGATAAAATGTGGAGGAATGAAAGGAATTCATGTTGAACAGCCTTTTGAT
TGCTTTGAAGAAAATGGCCAGTTTAGGCGTGCACCACCTCTGGTCCGTGTTGAAAAAATG
TTTGAACATGTTCAGTCCAAACTACCTGGTGCTCCCAAGTTTCTTCTTTGTCTCCTTTCT
GAGAGAAAGAACTCTGATCTTTATGGTCCATGGAAGAAAAAGAATCTTGCAGAGTTTGGA
ATTGTTACTCAGTGTATAGCTCCTACCAGGGTGAACGACCAGTATCTGACCAATGTTTTG
CTGAAGATCAATGCAAAGCTTGGTGGCATGAATTCTCTTTTAGGCGTTGAACACTCTCCA
TCCATTCCCATTGTGTCAAAAGCTCCTACTCTAATTTTGGGCATGGATGTTTCTCATGGT
TCACCTGGGCAAACTGAAATTCCCTCAATTGCTGCGGTCGTAAGCTCAAGGCAATGGCCC
TTGATATCTAAATATAGGGCATGCGTCCGTACTCAGGGTGCTAAGGTTGAAATGATTGAC
AATCTGTTCAAGCCAGTGTCAGATACGGAGGATGAAGGAATAATAAGGGAGCTTTTGATT
GATTTTTATAATAGTTCTGGGAACAGAAAACCAGACAATATCATAATTTTCAGGGATGGT
GTCAGCGAGTCTCAGTTCAACCAAGTTTTGAACATTGAACTGAGCCAAATCATTGAGGCC
TGCAAATTTCTTGATGAAAAGTGGAACCCCAAATTTCTGGTTATTGTTGCCCAGAAGAAT
CATCACACAAAGTTCTTCCAGCCTGGATCTCCGGATAATGTGCCTCCTGGTACTGTTGTG
GACAACAAAATCTGTCATCCCCGGAATTATGATTTCTACATGTGTGCTCATGCTGGAATG
ATTGGTACCAGTAGGCCAACTCATTATCATGTCCTTTTGGATGAGATTGGCTTTTCTCCA
GATGATCTTCAAGAGCTTGTGCACTCTCTCTCCTATGTTTATCAGCGGAGCACTACTGCC
ATCTCTGTTGTTGCTCCAATCTGCTATGCCCATTTGGCTGCTTCTCAAGTGGGACAGTTC
ATGAAGTTTGAAGACAAGTCTGAAACTTCATCTAGCCATGGTGGTAGCGGTCGTGATATC
AATGCCTCTCCCATCCCACAGTTGCCCAAATTGATGGATAGTGTCTGCAACTCTATGTTC
TTTGTTTGA
>Mtruncatula_Medtr5g094940
ATGGAACAAAGACCTGAGTGTGATGAAGAAAGAATGAAGACGAACAAAGATTATTGTAAT
ATGAGTTCACAAGAGAACAAAGAATGTTTGCCCCCACCACCTCCCATTGTTCCACCCAAT
GTTAAACCAATAAAGATAGAACAGGAGCATTTAAAGAAGAAGCTTCCTACTAAGGCTCCA
ATGGCTAGGCGTGGCCTAGGGACGAAGGGAGCTAAACTGCCTTTGTTAACAAATCACTTT
GAAGTGAATGTTGCTAACACAAATAGGGTTTTCTTTCAGTACAGTGTTGCTTTGTTTTAT
GAAGACGGACGTCCTGTTGAAGGGAAAGGCGCCGGGAGGAAGATAATTGACAAAGTTCAA
GAAACATATGATTCCGAGCTTAATGGAAAAGACCTTGCTTATGACGGGGAGACCCTGTTC
ACAATCGGCTCTCTAGCTCAGAAAAAGCTCGAGTTCATTGTTGTTGTTGAAGATGTCGCT
TCAAACAGGAACAATGCGAACACCAGCCCTGATAAGAAGAGGATAAGAAAATCATACCGT
TCGAAGACTTATAAAGTTGAAATCAACTTTGCTAAGGAAATTCCTCTGCAGGCCATTGCC
AATGCCTTAAAGGGGCATGAAGCTGAGAATTATCAAGAAGCCATTAGGGTTCTTGATATT
ATCTTGAGACAACATTCTGCCAAACAGGGCTGTCTGCTTGTGCGCCAAAATTTCTTTCAC
AACGACCCAAATAATTTGAATGATGTTGGAGGTGGTGTACTTAGTTGCAAAGGGCTGCAT
TCCAGCTTTAGAACCACACAAAGTGGATTGTCTCTGAACATAGATGTGTCGACAACCATG
ATTGTACGTCCTGGGCCTGTGGTTGACTTCCTAATTGAAAATCAAAATGTGAGAGATCCT
TTCAGTCTTGACTGGAATAAGGCTAAAAGGACCCTCAAGAATTTGAGGATCACAGCTAAA
CCATCTAACCAAGAGTACAAGATAACCGGGCTGAGTGAATTGTCGTGCAAAGACCAGCTG
TTTACCATGAAGAAGAGAGGTGCTGTGGCTGGAGAGGATGATACAGAAGAGATCACGGTT
TATGATTATTTCGTTCATCGGCGAAAAATTGATCTACAGTACTCTGCTGGTCTTCCATGT
ATTAATGTTGGGAAGCCAAAACGACCTACTTATATCCCAATTGAGCTTTGCTCATTGATA

TCCCTGCAACGATACACCAAAGCCCTCTCCACATCTCAACGATCCTCCCTTGTGGAAAAG
TCGAGGCAGAAGCCTGTAGAACGGATGAGGGTCTTGTCTAATGCGTTGAAAGCAAGCAAC
TATGGCTCTGAACCAATGCTACGTAACTGTGGCATTTCAATAACATCCGAGTTCACCCAA
GTTGATGGGAGGGTTTTGCAGGCTCCACGGTTGAAGTTTGGGAATGAAGATTTCAATCCC
AGGAATGGGAGGTGGAACTTCAATAATAAGAAATTTGTAGAACCTGTTAGCTTAGGAAAT
TGGTCTGTGGTGAACTTTTCTGCTCGCTGTGATGTGCGAGGACTTGTGAGGGATCTAATA
AAATGTGGAGGAATGAAAGGAATTCTTGTCGAACAACCTAAAGATGTAATTGAAGAAAAT
CGTCAGTTTAAAGGAGAACCACCTGTGTTTAGAGTTGAGAAGATGTTCGCGGATGTGTTA
AAACTCTCGAAGCGTCCTAGCTTTCTGCTTTGTCTGCTTCCTGAGAGGAAGAACTCTGAT
CTTTATGGTCCATGGAAGAAGAAGAATCTTGCGGAGTTTGGAATTGTTACCCAGTGCATA
GCTCCTACCAGGGTGAACGACCAGTATCTGACCAATGTTTTGCTGAAGATCAATGCAAAG
CTTGGTGGGATGAATTCTTGGTTAGGCGTTGAACACTCTCGATCCATCCCTATTGTGTCG
AAAGTTCCTACTCTCATTTTGGGCATGGATGTTTCTCATGGTTCACCTGGGCAACCTGAC
ATTCCTTCAATTGCTGCGGTCGTAAGCTCGAGGAAATGGCCCCTGATATCTAAATACAGG
GCATGTGTCCGAACTCAGGGTTCGAAGGTTGAGATGATTGACAATCTGTTCAAGCCAGTG
TCTGATAAGGAAGATGAAGGAATTATAAGGGAACTTTTGCTTGATTTCTTTCATAGTTCT
GAAGAGAGACGACCAGAGAATATAATCATTTTCAGGGATGGTGTTAGTGAGTCTCAGTTC
AACGAAGTGTTGAACGTTGAACTCAGCCAAATCATCGAGGCTTGTAAATTTCTTGATGAA
AATTGGAACCCCAAGTTTATGGTGATTGTTGCCCAGAAGAATCATCACACAAAATTCTTC
CAACCTCGATCTCCAGACAATGTGCCACCTGGAACTGTTGTGGACAGCAAAATTTGCCAT
CCTAGGAATTATGATTTTTATATGTGTGCTCATGCTGGAATGATTGGTACAAGCAGACCA
ACACATTATCACGTTCTTTTGGATGAGATTGGTTTTTCTCCAGATGATTTACAGGAGCTT
GTGCACTCTCTATCTTATGTTTATCAGCGGAGCACCACTGCTATTTCTGTCGTTGCTCCA
ATCTGCTATGCACACTTGGCTGCATCTCAAGTTGGACAGTTCATGAAGTTTGAAGATAAG
TCTGAAACTTCCTCGAGCCAAGGTGGTATCAATGCCTCCCTCATCCCACAGTTGCCAAAC
TTACATAAGAGAGTCTGCAATTCCATGTTTTTTGTCTGA
>Mtruncatula_Medtr8g118920
ATGAAGGAAGAAACTGGACTTGAATCTCAACTAGCTTCATGTTTTTCTTCCTCTTGCAAA
AGTCTCGTGTTTCCTTCTAGGCCTGACTATGGCAAACTTGGAACAAAGTGTGTTGTCAAA
GCTAATTACTTCCTTGCAGATATTTCAGTATCTGACTTGAGCCATTACCATGTTGATATA
ACTCCTGAAGTTATATCTAGTAAAACAAGAAAAGCTATCATAGCTAAGTTAGTGAAGTTT
CACCAGAACACTGAATTGGGAAAGAAGCTCCCTGTTTATGATGGTGCAGAGAATCTTTAC
ACTGCCGGTTCGCTTCCTTTTACACACAAAGAGTTCAATATACTATTGATTGAGGATGAT
GAGGGTTTTGGTACTACAAGGGAAAGAAAATTTGAAGTTGCAATCAAGTTTTTAGCTCAT
GTTAGTATGCATCAGTTACATGAGCTTCTCAGTGGAAAAAAAGTGGAAACTCCACAAGAA
GCTATCAATGCCATTGACATTGTATTGAAGGAGCTTGCATCTCATAGCTATGTATCATTT
GGGAGCTTACACTATTCTCCGGATTTAAAAAAACCACATAAGCTGAGTGGTGGCTTGGAA
TCATGGAGCGGTTTCTATCAAAGTATAAGGCCTACTCAGATGGGATTATCGCTTAATGTT
GACATGGCATCCACGGCGTTTATAGAACCGCTCCCTGTGATTGACATTGCAGCTCAAATT
TTAGGAAAAGATGTGCACTCAAAGCCATTGTCAGATGCAGATCGCATCAAGATTAAGAAA
GCCTTGAAAGGTGTGAAAGTAGAAGTTACATATAGAGGAAGTTTTAGAAGGAAGTACAGG
ATAACTGGATTAACATCACAACCTACAAGAGAACTCAGTTTCCCTTTGGGTGAGAAAATG
AATATGATATCAGTAATTGATTACTTTCAAGAAATGTATGGATACAAAATCATGTATCCT
CATTTACCTTGCCTTCAAGTAGGAAGTCAAAAGAAGGTGAACTATTTGCCTATGGAGGCA
TGCAAGATAGTTGGTGGACAGAGATATACAAAAGGGCTTAGTGAGAAGCAGATAACTTCT
ATGCTAAAAGTCTCATGTCAGAGACCACGCGAAAGGGAGAATGATATTCTTCAGACAATT
CATCAAAATGATTATGATTGTAATCCTTATGCAAAGGAGTTTGGCATCAGCATCGGCAAC
GAACTTGCATCAGTCGAGGCTAGGGTTCTTCCTGCTCCTTGGTTGAAATATCATGAAACT
GGAAGAGATAAAAAAATCTTGCCACAAGTTGGACAATGGAACATGACGAACAAGAAAGTT
GTAAATGGAAGCAAAGTAAGATATTGGGCATGTATCAATTTCTCAAGAAGTGTCAAAGAA
AAAACAGCTAGTGCATTTTGCCAACAGTTAGTTCAGACTTGCCAAAGTTTAGGCATGGAA
TTTAGTGAGGAACCAGTTATTCCTGTATATTCAGCAAGGCCAGATATGGTTAAGAAGGCC
TTGAAGTATGTACATTCTTTTTCTCTAAACAAACTTGAGGGAAAAGAGCTAGAGTTGGTT
GTTGCCATTCTTCCAGACAACAATGGCTCTCTCTATGGTGATCTCAAAAAAATCTGCGAA
ACAGATCTCGGGCTGATTTCCCAATGTTGTCTTACAAAATATGTATTCAAGATTAATAGA
CAGTATTTGTCAAATGTAGCACTAAAGATCAATGTCAAGATGGGAGGAAGGAACACAGTA
CTTTTGGATGCTATAAGTTGCAGGATTCCATTGGTTAGTGACGTTCCAACAATAATTTTT
GGAGCTGATGTATCTCATCCTGAATCTGGAGAGGACGTGTGTCCATCCATTGCGGCTGTT
GTAGCCTCCCAAGACTGGCCAGAAGTGACAAAGTATGCAGGATTGGTATGTGCTCAGCCT
CCTCGAGAAGAAATTATTAAAGATCTTTTCAAATGTTGGAACGATCCTCGTCGCGGTATC
GTTTATGGTGGCATGATCAGAGAGCTCTTGCTCTCATTTCAGAAAGCAACTGGAAAGAAA
CCATGTAGGATATTATTTACAGGGATGGGGTAAGTGAAGGACAGTTCTACCAGGTTTTG

CTATATGAGCTCGATGCCATCCGTAAGGCTTGTGCATCTTTGGAGCCTGGTTACCAACCT
CCGGTTACATTTGTCGTGGTTCAAAAACGGCATCACACTCGACTCTTCTCAGACAATCAC
AACGACAGAAACAGCATGGATAGGAGTGGGAATATCTTACCTGGAACTGTGGTGGACACA
AAGATTTGTCATCCTACTGAGTTCGACTTCTACTTGTGTAGTCATGCTGGAGTTCAGGGT
ACAAGTAAACCAGCTCACTATCATGTTATATGGGACGACAACAAATTCAGTGCCGATGAG
ATTCAGTCCTTAACTAATAACTTGTGCTACACATATGCAAGATGTACGCGGTCTGTTTCT
TTAGTGCCTCCTGCGTACTATGCTCATCTGGCTGCTTACAGAGCGCGATTCTACATGGAA
CCTGATGTCCATGAAAATGCAAAATCGCAAGTTACAGGATCGAAAGTCGAGTCAGTTCGA
CCACTACCAGCTCTAAAAGAGAAGGTTAAGAAAGTAATGTTTTACTGTTGA
>Physcomitrella-patens_1885752_locus
ATGTTGGGACCTTTGCATGACCCCCTTACTCCTAGACAGGAACTACCTCCGACGCCGCCG
CCACTATCCAGCAAAGGTGTGAGGTTCCCTTTGCGTCCTTCAAAAGGGAGTAATGGTCTG
AGATGTATTGTCATAGCCAATCATTTCTATGCTGAGCTGCCCGATAAGGATCTGCATCAT
TACGACGTTGCCATCAATCCAGAGCTCCCATCTAAGGGAATCAACAGGGCTGTTATGGAG
CAGCTCGTGAAGCTCTACAGAGAATCTCATTTAGGCACTAGGCTACCTGCTTATGACGGT
CGAAAAAGTCTTTACACCGCCGGACCGCTTCCGTTTCAGAGCAGGGAGTTCGAAATTAGT
CTTACGGACGAGGAGGACGGAAGCAATCAGCTTAGGAGAGCGAGGCACTTCAAGGTGGTT
ATAAAATTTGCTGCCCGGGCAGATTTGCACCATCTTGGAGAATTTCTTGCTGGACGCCAA
GCTGATGCTCCTCAAGAAGCTCTGCAAGTTCTGGACATTGTTTTGAGAGAGCTCCCTACC
CACCGGTATTCTCCCGTTGGGCGCTATTTCTATTCACCCGATCTGGGCACGCGACGGCCA
TTAGGAGATGGTCTTGAAAGCTGGCGGGGCTTTTATCAGAGTATTCGACCAACTCAAATG
GGGTTGTCCCTAAACATTGACATGTCTTCCACAGCGTTCATTGAACCTAAAACTGTTATA
GAGTTTGTAAAGGATCTCCTGAGGAAGGATTTAAACCGTAGTCTGACCGATGCTGATCGC
ATTAAGATCAAGAAAGCCCTCCGTGGGGTGAAGGTGGAAGTCACTCATCGCGGAAGTATG
CGGCGCAAGTATCGCATATCTGGGCTCACTAATCAGGCCACCAGTGAATTGCAATTCCCT
GTGGACGATAATGGGACTATGAAGTCTGTTACTGACTACTTTCGCGAGACTTATAGCTAC
ACAATTCGACATCCAGCTTTACCCTGTCTCCAAGTGGGAAATACTCAGCGTCCCAATTAT
CTTCCAATGGAAGTCTGCAAAATTGTTGAGGGCCAGCGATATTCCAAACGGCTGAATGAG
CGCCAAATTGCTGCACTTCTACAAGTCACTTGTCAGCGACCCAGAGACAGAGAGAGAGAT
ATTATGCAGACTGTTCATCACAATGCGTATCATCAAGACCCATATGCCCAGGAGTTTGGA
ATCAGGATCAGCAATGAATTAGCGCAAGTGGAGGCTCGAATCCTACCAGCCCCACGACTG
AAGTACCATGATACCGGAAGGGAAAAAGAGTGCCTTCCACAAGTTGGGCAGTGGAACATG
ATGAACAAGAAAATGGTGAATGGAGGTATAGTTCAGCACTGGGCTTGTGTCAATTTCTCA
TCAAATGTTCAAGAGAAAATTGCTCGCGACTTCTGCTTAGAGCTGGCTCAGATGTGTCAA
ACATCTGGCATGCAATTTGCTCGAGATCCCATTGTGCCGGTGAAAACTGTCCGTCCTGAT
AATTCAGAAAAAGCTTTGTATCAACTCTGTGAAGATGTGAATAGAAGGACAAAGGGGAAG
GGCTTGGATCTGCTTATTGCAATTCTTCCCGACAACAACGGGTCTTTGTATGGCGATCTT
AAGAAACAGTGTGAGACTGTCCTGGGGGTCGTGTCTCAATGTTGTTTGACGAAACATGTA
TTTAAGATGAGCAAACAGTACTTGGCAAATGTTGCCCTGAAAATCAATGTCAAGGTTGGA
GGCCGAAATACTGTTCTGGTGGACGCTCTTTCTAGAAGAATTCCTCTTGTCAGTGACAAA
CCCACCATTATATTCGGTGCAGATGTCACTCATCCGCACCCCGGAGAAGATTCGAGCCCT
TCTATTGCGGCTGTTGTGGCATCGCAGGATTGGCCTGAGGTCACCAAGTACGCTGGTTTA
GTGTGCGCTCAAACTCACCGTCAAGAGTTGATTGCGGACCTATTCAAAGAATACACTGAC
CCAATGAAGGGGAAAATGTTTGGAGGGATGATTAGGGAACTTCTCATCTCTTTCCGGAGC
GCAACTGGTCAAAAGCCTCTCCGTATCATCTTTTACAGAGATGGAGTAAGCGAGGGCCAG
TTTTCACAGGTTCTTCTTCATGAGCTAGACGCAATCAGGCGAGCCTGTGCTTCGCTTGAA
GAAGGATACCAACCTCCAGTCACGTTTGTAGTGGTTCAGAAAAGGCACCACACTCGTTTG
TTTGCCAGTGATCGTCGCAATACTGATAGGAGTGGCAACATTCTACCAGGTACGGTTGTG
GACTCTACAATCTGTCACCCTACGGAATTCGACTTCTATCTTTGCAGTCACGCTGGAATT
CAGGGTACCAGTCGACCTGCACATTACCATGTATTGTGGGACGAGAATAGTTTTTCTGCT
GACAGTTTGCAGTCATTGACAAACAACCTATGCTATACGTATGCACGGTGCACACGGTCA
GTGTCAATCGTCCCCCCCGCATATTATGCTCATTTGGCCGCTTTCCGAGCGAGATTCTAC
ATGGATCTAGAATCTTCGGATACTGGATCTGCAACCAGTGGAATAGGGGCAAATCGTACA
CAGGTCACCGGATCAACTGCCGCTCGCACTAATCGGGTGGCAGGAAATACTGCCGTGCGT
CCACTTCCTCCTTTGAAGGAAAACGTCAAAAGGGTAATGTTCTACTGTTAG
>Physcomitrella-patens_1888444_locus
ATGGCACAAGGTAAAGGTAATTCTGTTCAACGACGACGACAGAAAGCTGAAGGAGCTGTT
CCTATCAGTGCTGCTGGCGAAGGAGGTGGCGAAGGACCTCGGGCAGGTCCATCACTATCC
GGACCAGTCGGACGTTGTGAGGTTAGAGCGGTGACTGTGCCGAAAAAATCATTAAGACCA
GGATATGGTCGAGCAGGGCGTGGGACACTGCTTGGCGTGAATTACTTCAAGACTTCATTG
TCGAAGAGAGTCGATGTCCATCATTACAATGTTTCAATCGAGCCGGAAACCGTGAGCAAG
AGGATGTGCCGTGAAATAATGAAGAAGCTCCGCGAAACATATGGAAACGAATATTTTGAT

GGTAAACATGGAGCGTACGATGGAGAAAAATCTCTGTTTACGAGTGGGTGTTTACCGTTC
AAGTCAATGAAGTTTTCAGTACTCCTGGACAATTTCGAGGGTTCCTCATGTAGACCTGGT
GACAGTGGAAGACCGAGTTCAGAGCCTCTCTCACAGAGAAGGGCTCCATCCAAAGTTAGG
AAATTTGATGTTTCAATTGAGTTGGCTGCAAAGATTCGGATGGATGCGATAGAGGAAATG
ATGAAGAGAGCGCTGGGTAGGTGTGATCAGGAGCTACAAGATCGAGCGCTGGACGCTCTA
CGTGTTCTGGACGTTATACTCCGTGAAATTGCTTCAAGAAGAGGCTACCTGCTTGTGAGA
GACAATTTCTTCCATCCGAGTTTGGGTCAAGTTTGCGATTTGGGCGATGGTGTGGAAGGT
TGGAGGGGTTATCACTCCAGCGTGAGACCGACGCTGATGGGATTGATGTTAAATTTAGAT
ACCACTATGACGGTGGTACTGAAGCCTACTCTGGTTGATGAGTTTCTCAAGGAAAGGTTC
AATGTAAGAGACCTGTCTGGTTTGCAGAAAAGAGATTGGGCTAAAGCGAAGGACATGTTG
AAAAACGTCAGAATAGAAACGACTCACACGGGAGTGTCCCGAAAGTACAGAATATCGGGC
TTCAGTGATCGGTCCATACGAGAATTAAAGTTTATGAAGGGGACGAAAGATGGGAATGGG
GAGGAGGAAATTTCAGTATATAATTATTTTTTTGATACTTACTCGCGCAAACTAAAAAAC
CTAAATTTTCCAGCGCTGGATCTTGGAAACAGCAGGAAGCCAATATACATGCCCATAGAA
TTGTGCAAGATAGTTAGTGGACAGCGCTATACCAAGCCGCTGTCCAGCAAGCAGAGGATG
GCACAAATCGGTGCAAGCAAGCAGGCGCCGCAAGAACGACAAAAGATCTGTGAGAATGCG
CTCAAAGTTTGCAACTACAGCTCCGACAAGCTGATTGCGGAGTTCGGTTTACAATTTGAT
AACAAGCTTGCGTCCGTTTCTGGCAGAGTGCTACCTGCCCCTCAGCTCGACTTTGGAAAT
GGAAAGACCGAGGAGCCCAAAGAAGGGCGATGGAATTTCAACCACAAGACCTTGAAAAAG
GGAGTTACGATAGCCGCTTGGGCTGTAGCTGTGTTCGACCCCTGCTGCAATGATGGAGAT
CGAATCGCATTCCAGTTGATAGAGAGTTGCAGCCGACGAGGCATGGTGATGAAAAGTCCG
GCGGTGGTGCAGAAACAGCCGAGGAATTCGTTTGGATCTTCTCCTGAAGAAAGAGTGGAG
ACGATGTTTAATGCTTTGAGGCCGTACGCGCCTGTGTTCATTCTTGCTATTCTAGCAGAA
AAAGACAGTCCCATTTATGTTCCTTTCAAGCGGTTGTGTGAAATTAGACTTGGAATAATC
TCGCAGTGCATGGTGAAGCCGCGAACTCTGAACGATCAGTTCCTAGGAAACCTTGCGTTG
AAGATTAATTTGAAGATGGGAGGATTGAACTCGCCATTGAGTCAGCGGATGTTACATTGC
CTTGGTCAGTCGACCATAATCTTCGGCATGGATGTGACCCATGGGTCTCCTGGGGATGTC
GAAATACCCTCAATTGCGGCAGTAGTCGCAACGAAGAATTGGCCCGAGGTATTCCATTAT
TCGACTCAAGTTAAGGTCCAACCAGCCAGGATGGAGATGATTCAAGGGCTGTATGAACCT
GAAGGTGGCATGGTACGAGAATTGCTCATGTCGTTCTATTCGACATGCGCCAAAGGCGTC
AACCCGAAGCCATCCCAAATTATAATTTACAGGGATGGAGTTAGCGATAGCATGTTTGCG
AAATGTTTGGAAGTTGAGTTCGTCGCATTTAAACGAGCTTGTGCAGAACTCGAAGCGGGT
TACAATCCCGGAATAACGTTCATTGTGGCTAAAAAGCGTCACGGCACGCGCTTCTTTCCT
CAGAGTCGAGATGCGTTGCGGAATGGGAACGTTCTACCAGGAACTGTTGTCGACAAGGAT
GCGTGCCACCCTCGGAATTTTGACTTCTTCCTCATCTCTCAGGCCGGCCTCATTGGCACA
GCTCGGCCTACTCACTACACAATACTGGTGAATGAAAACCAGCTTGGGCCTGATGATATT
CAGACCTTGACCAACAAACTGTGCTACACGTTTGGACGCTGTACCTCATCCATCTCGATG
GCGGCTCCAGCCGCATATGCTCATATTCTGGCATCAAGATATCGGAAGTTGATGAGTCCT
TTGGAAGGAGGATCTACTACATCGTCATCGAGCTCTCGAGGAATAGGTGTTTCAAGACCT
TTACCACCAGTCCCGATTTTGCGGATGAAGGCAGATCATTCCATGTTTTTTGTTGA
>Physcomitrella-patens_1901732_locus
ATGTCTGAAGTAGGGCTCCCCCTGCCACCGCCTCCGCCGGTGCCCCCAGGAGCTTTTCCT
GCCATATCCCCAGGCTATGAAAAGGCATTGCCTCCGTCTGGGCTCAACCGTGAACCGAAC
GAAGGTCACACTGGTCATGACGGAACTCCAAAGGGAGCCAGCGGCAGCTCTGCCCCGATT
TCACCAGCGCTGGCTCGAATTCCCTTGCCGAAGAAAGCAACAAGACCAAGCTTTGGAAAG
CTTGGACGTCCTTCTAAATTGTGTATGAATCACTTCAAAACCAGCATAGTGAAATGGGAC
GACGTTTACCAATATAGTGTGTCGATTGAGCCATCTGTGAAGGACAAGAAGCAATGTCGT
GAGATCATGAAGAAGCTCCGGGAGACATATGGGGAGGCTGAGTGCGGTGGAAAGCAGGGT
GCTTACGATGGGGAGAACTGCTTGTTTACAAGCGGTAGCTTGTCTTTCAATACAAAGGAG
TTCCCAGTGTTCCTGGAGGATTCAAAAGGCTCCTCGTATCGACCTGGTGACCGGGATGGA
AAGACCGGCGACATCTTTCCGAAAAGGAGAAAGACCGTGTCCCGGGGAAGAGAGTTTTCG
GTGAAAATTGAATTCGCGGCCACTATTCGGATGAAGGTTATTGATGACATGATGAAGGGA
GTGATGGGCAAGGGTGATTTGGACCAGGAGACACGAGCTCTTGATGCATTGCGCGTGCTG
GATATAGTGCTTCGTGAGAATGCTTCCGAAAGAGGCTATCTTCTTGTTAGGGACAATTTC
TTCCATCCAGAGTTGGGTCCGGTTGGCGACTTGGGAGAAGGAGTGGAGGCTTGGAGAGGT
TATCACTCGAGTATAAAGCCAACTGGATTGGGGTTGACTCTCAACCTTGATGTCACCATG
ACAACTATACTGAAGCCCATTACGGTAGAGAAATTCCTTGCTGAATATTTCGGTGTCAGA
GACCTGAATGGCTTGCAAGCAAGAAACTGGACTAAAGCGAAGTCCATCCTTAAAGGTGTA
AAGGTCGAAACAACACACATGTCTGTGTCACGCGAACATAAAATCTCAGGTTTCAGTGAC
CGTGCCATTCGGGATCTCAAGTTCTCGAGAAGGGTCAAAGATGGTGAAGGCAATATTGGG
GAAGAGGAAATTTCAGTACAACAGTATTACTCTGACGTGTACATGTACACTCTAAGGTTC
CCAGACCTTCCAGCACTTGTTTCCGGGAACAAAAAGAAGGCCACGTTCCTACCATTGGAG

CTGTGTAAGATTATCGCTGGGCAACGGTATACCAAGTCTCTATCCAGCAAGCAAAGGCAA
TTGCAGATCGCCGCTTGCAAGCAGTCTCCGCAGGAGCGTCAGAGGATCTGTGAGAATGCT
ATGGAAGTCAGCAAGTACAGCTCCGACAAGCTGATAGCAGAGTTCGGTCTGAAGTTTGAG
AGCAGTCTGGCCGGTGTCACAGGCAGGATATTAAGACCTCCTCAGCTTGAGTTTGGACAT
GGAAGGACCGAGGAACCAAGGGACGGACGGTGGAACTTCAACCAGAAGGAACTGAAGCAA
GGAGCAAGAATCGATACCTGGGCTGTTGCTATATTTGATGGGCGCTGCAGTGATGGTCAA
CGCATCGCAGAAAGTTTGGTCGACTGTTGCTGCAAGAGAGGAATGCAAATGCGGCAAGCA
GCGATTGTGGAGAAAGAGCCCCCGAGTTCTCAGAGATTTTCTCCTGAACAACGGGTTGAG
AGGATGATTACCGCACTAAAGCAGACAAAGCCTGTATTTATCCTGGTCATATTACCTGAC
AAAGACAGTCCAATATATGTTCCATTCAAGCGGTTCTGTGAGATGAAGATTGGGGTTGTT
TCGCAGTGCATGGTGAAGCCTCGACAGCTTAATGATCAATACCTGGGAAATCTCGCCTTG
AAGATTAATCTCAAGATGGGGGGATTCAATTCGCCGCTTAGCCCCAGAATGGTTTCCTGT
CTTGGCCCGTCGACAATCATCTTTGGGATGGACGTGTCGCATGGGTCGCCTGGAGAATCC
AGTGTCCCTTCGATTGCAGCCGTGGTTGCAACCAAAAATTGGCCGGACGTTTTTCACTAC
TCGACGCAAGTGAGAATTCAGCCCGCCAAAACGGAGATGATAGAAGGGCTCCACGATTCG
AAAGGTGGAATGGTCAAGGAATGCTTGAAAGCATACTACATATCATGCAGAAGTCCAAAC
TATCGCAAACCAACCCAAATAATTGTTTACAGGGATGGAATCAGTGAAAGTCAGTTTGCA
GAATGTTTGGAGGTTGAGTTTACTGCGTTTAAAAGGGCATGTGCAGAACTGGAAGAAGGA
TATAATCCCGGTATCACCTTCATTGTTGCTCAAAAGCGACACAATACTCGTTTCTTTCCT
CAAGGCCCTGACAATCAGATGCGCAATGGAAACGTTCTTCCAGCCCACGCTGAAAGTGCA
GGAACTGTTGTCGACAAGGACGCTTGCCACCCTCACAACTATGACTTTTTCCTCGTCTCT
CAAGCTGGCCTTATTGGTACGTCTCGTCCCACGCACTATCACGTTCTCGTGAATGAAAAT
AAGCTTTCGCCTGATGACATTCAGGGCTTGACAAACAATTTGTGCTACACGTTTGGCCGC
TGCACAACATCAGTTTCTATGGGTAAGCCGCTTCAAGGATTTCGATGTTTTCTCTTGTCT
TATGTTACTACGTAA
>Physcomitrella-patens_1904560_locus
ATGCCGCGGAGACGCAAGACCCCGCGGGAACTTGAGGAGAGTGCCCAAGCTCAGGCTTTA
GCAGAAGCTCAAGCATCGGCATTAGCAGGAGCGGAGGGGTCAGCTCCTTCAGCTGTTGCG
CCGGAACCTGCTTCGACTCCGACTCCAGCTGCTGGTGGAGCTGCTGTTCCAGCCCCTATT
CCAGTGTCAGCACCTGCACAGCACGTTGCTTCTCAACATGTTGAGGGCCAGGGCCATGGT
CAAGCTCACTCCCAGCATTTAGGAGAGGGCTCGCAAGGCCAAGGTTCGGGGCATTCTcag
ggacagaggcaaaggcaaggacaagggcaagggcaaggtcaaggacaggggcaaggtcaa
ggacaggggcaaggtcaaggacaggggcaaggtcaaggacaggggcaagggcaaggacag
gggcaagggcaagggcaaggacgCGATGAACCCAATAGCAGTGGACCTCCTGGGCGGGCG
AGAGGAGGCATGGGAGGAGGCGAGCGAGGTTCTGGAAGGAGGGGTGGCAGGAGCCGCTAT
AATCAGGGACCCCCTGTGCATTTCGCTGCACTGCCTGCAGGCTATGGATCAGGTGTAGGT
CCAGTTGTTGAGCAGTTCGGGTCAGTGGTTATAGGGGGTGTTGGACCTCGTTTCAACTCT
GGTCCAACTCAAGAGCAAGCTGTTTCTAAATACGAGCCGCCACCCACGCCTCCACCGATT
TCTAGCAAACAGTTGAGATTTCCTTTGCGTCCAGACCGAGGAAGGATCGGTCTGAGGTGT
ATTGTGAAGGCAAATCATTTTTTTGTCGAGCTACCTGACAAAGATCTTCATCAGTACGAT
GTGACTATCACTCCAGAGGTCACATCCAGGGGTATCAACAGGGCCGTCATGGAGCAGCTT
GTGAAGCTTTACAGAGAGTCACACTTGAGCTCCAGGCTTCCTGCGTATGATGGTCGGAAG
AGTTTGTATACCGCTGGGCCACTTCCGTTTCAGAGCAAGGAATTCCAGATCAGTCTTTTG
GACGAGGATGATGGAACCAACCAACCTAGACGGGAGAGGCTTTTCAAAGTGGTTATCAAG
TTTGCTGCTCGAGCAGATTTGCATCATCTTGGGCAATTTCTTCTTGGACGCCAAGCTGAT
GCTCCTCAAGAAGCCTTGCAAGTCCTAGATATCGTATTGAGGGAGCTTCCTACCCACCGG
TGCGTTTATTCTCCTGTAGGACGCTCGTTCTATTCTCCCAATCTGGGCACTCGACAGCCG
TTAGGAGATGGTCTTGAAAGCTGGCGGGGCTTTTATCAAAGTATTCGACCAACTCAAATG
GGATTGTCCCTAAACATTGACATGTCTTCCACAGCGTTCATAGAACCCAAGACTGTAATG
GAGTTTATAAGGGATCTCCTCAACAAGGAGTTGACCCGTAGCCTAAGTGATGCCGATCGT
ATGAAGATCAAGAAAGCTCTTCGCGGAGTCAAAGTGGAAGTGACTCATCGCGGAAGTATG
CGTCGCAAATACCGTATATCTGGGCTTACCCATCAGGCTACTAATGAACTCGAATTTCCA
GTTGATGAGAATGGGACATTGAAGTCTGTCACCGACTATTTTCGGGAAACATACGGCTAT
TTTATCCGTCATCCGTCTTTGCCGTGCCTTCAAGTGGGTAATTCTCTGCGTCCCAACTAC
CTTCCCATGGAAGTATGCAAGATCGTTGAAGGCCAGCGATATTCGAAACGGTTGAATGAG
CGCCAAATTACAGCTCTTCTCAAAGTAACTTGCCAGCGACCTCGAGACAGAGAAGCAGAT
ATTATGCAGACAGTACACCACAATGCATACCACCAAGATCCCTATGCTCAAGAGTTTGGG
ATCAGAATTAGTAATGAGCTTGCGCAAGTGGAGGCACGAGTCCTGCCAGCTCCACGGCTG
AAGTACCATGATACTGGCAGGGAAAAGGAGTGTCTTCCACAAGTTGGACAGTGGAATATG
ATGAATAAGAAAATGGTGAACGGCGGTATCGTGAACTACTGGGCATGTATCAATTTTTCA
CGCACTGTCCAAGAGAATGTGGCGAAGAACTTCTGCCAAGAGCTGGCTCAGATGTGCCAC
ACATCCGGGATGCAATTTACAAGAGATCCCGTTGTGCCTTTGCAAAGCTACCGACCCGAG

CACTCGGACCGAGCTCTGTTTCAGCTATGTGATGACGTGCACAAAAAGACTAAGGGCAAG
AGCTTGGATCTCCTTATTGCAATCCTTCCAGACAACAACGGGCCTTTGTATGGTGATTTG
AAGAAGCAATGTGAGACTGTACTGGGTGTGGTTTCTCAGTGTTGTTTGACCAAGCATGTT
TTCAAGATGAGCAAGCAGTACCTGGCAAACGTGGCTCTCAAAATTAATGTCAAGGTTGGG
GGTCGGAACACTGTCCTGGTGGATGCACTTACACGCAAGATTCCTCTCGTCAGTGATATT
CCCACCATAATATTTGGTGCAGATGTTACTCATCCACATCCTGGAGAGGACTCCAGTCCC
TCCATTGCTGCAGTGGTAGCTTCGCAGGATTGGCCCGAGGTCACCAAGTATGCAGGGTTG
GTGTGTGCTCAAGCTCACCGTCAGGAGTTGATTCAGGATCTGTACAAGGAATGGAGGGAC
CCCCAGAAAGGCACGATGACAGGAGGGATGATAAAGGAACTCCTTATCTCTTTCCGGTGT
GCGACCGGTCAAAAGCCACTTCGGATTATCTTTTACAGAGATGGAGTAAGTGAAGGTCAA
TTTTACCAGGTTCTTCTGTATGAACTGGACGCAATCAGGAAAGCTTGCGCTTCCCTTGAA
CCGGATTACCAGCCTCCAGTCACATTTGTGGTCGTTCAGAAAAGACACCACACTCGTTTA
TTTGCCAGCAATCATAATGACAATCGCTCTACTGATAGAAGTGGCAACATTTTACCAGGG
ACTGTAGTGGACTCAAAGATCTGTCATCCTACAGAATTCGATTTCTATCTTTGTAGTCAT
GCTGGGATTCAGGGAACCAGTCGACCTGCGCATTACCATGTGCTGTGGGATGAGAACAAA
TTTTCTGCGGATAGTTTACAGTCGTTGACAAATAATCTGTGCTATACATATGCACGGTGC
ACGCGCTCTGTTTCTATTGTTCCTCCAGCATATTATGCACATCTAGCTGCTTTTCGTGCC
AGGTTCTACATGGATCCAGAAGCTTCAGATACTGGTTCTGTTACTAGTGGGTTGGGAGGG
GCGAATCGCAGTCAGTTTACTGGATCGACTGCTGGCCGCACTCATCGTGCGGCGGGAGGA
AATGCCGTGCGCCCACTTCCACCTCTTAAGGAGAATGTGAAGAGAGTCATGTTCTACTGT
TAA
>Physcomitrella-patens_1910596_locus
ATGGACGGTTCGGAGTCTCGTGATACTAGTGGTCGTCCACGACCACCTCCAGTTTCAGAG
GGAGCTCCAGGGCATCCTAGGGTTGGAGCTAACCAAGTGCGGCCATCTCAACCCGGACCT
AGTGGACAACATTTGCAGGGCCGTAGCGGAGGTAGTGACAGTGTTGCTTTGGCTCTACCT
GTAGCGTGTGTCCCGAGTAAGACAACTAGACCAAGCTTTGGGAGAGCGGGACGGCTCACA
CAGTTGTGTGTGAATCACTTCAAGACCGAACTGGTGAAGTGGGACGACGTGTACCATTAC
AATGTGAGGTCCATTACATCATTTCTCTGCCAGCGATATGTGTCGATGGAACCGGAAGTA
ACGAATAAGAAAATATGTCGTGATATCATGACGAAGCTCCGCGACACATTTGGGGAATCC
GAATGCGGTGGTAAGCAAGGGGCTTACGATGGAGGAAAATCTTTGTTTACGAGTGGGAGT
TTATCGTTCAACTCTAAGGAGTTTCCAGTGTTCCTGGATGACCGCAAAACTCCCTCTTTC
AGACCTGGTTTTCCTAGGGAAGAAGCCATCACGAAGAGGAGGAGGACTGCTGCGAGAGGG
AGGGATTTCATTGTTAAGATTGAATTTGCTGGAAAGATTCGGATGAAAGCAATTCACGGG
ATACTAAAGAGAGTTATGGGTATGGGCGATCTAGAGCAGGAAGTGCGTGCAATAGATGCT
CTACGTGTTCTAGATATAGTGCTTCGGGAGAGTGCTTCAAGAAGAGGGTACCTTCTTGTG
AGGGATAATTTCTTTCATCCGAGCTTGGGTCCGGTTGGCAACTTGGGAGAGGGAGTTGAG
GCTTGGAGGGGTTATCACTCAAGTGTGAGGCCTACTGGGTTGGGCTTGACTTTGAATCTC
GACATGACTATGACAACGATGTTGAAGCCCATCCTTGTAGAGGAATTTCTGATGGAAAGG
TTCAACGTAAGAGACCTGAATTGTTTGCAAGGCAGGGACTGGGTTAAAGCCAATAGCGTG
TTGAAAGGAGTTAGGATCGAAACGATTCACATGGAAGTGTCTCGATCTCACAAGATTGCG
GGGTTCAGTCCGCGGCCCATTAAAGACCTAAAATTCACTAAGAGGATCAAAGACGGCGAA
GGTAATGCGCGAGAGGAGGAGATGTTAGTGGAGCAATACTACTTCGATGTGTACTCGTAC
ACCCTTAAATACCCAGGTCTTCCAGCAATAGATGTTGGGAACAAGAAGAAGCCCACATTC
TTGCCGTTAGAGTTGTGCAAGATAGTTGCGGGACAGCGTTACTCGAAGTCGCTGTCTAGC
AGGCAGAGGACTGCCCAGATTGCTGCATGCAAGCAGGGACCGCAAGAGCGGCAAAGAATC
TGTGAGAACGCGATTACTGTGAGCAACTACAACTCTGACAGAATAATCTCAGAGTTCGGT
CTTCGATTTGAGAACAAGCTCGCTTCCATCGAAGGCAGAATGCTACCTGCCCCTCAGCTC
GAATTCGGAAATGGGAAGACCGAGGAACCTAGAGAGGGGCGGTGGAATTTTAATAACAAG
ACCGTAAGGAAGGGAGTCAAAATAGACCCCTGGGCTGTCGCTGTGTTTGATCCCCGCTGC
AATGACGGAGGTCGCATCGGAGATCAGCTGGTGGAGAGTTGCTGCAGACGTGGTATGATG
ATGCGAAGACCGGCTGTGGTGCAAAAAGAGTTGCCTGATGCATTGAACCGTTCACCCGAG
CAGCGGGTGGAGTGGATGCTTATGTCACTTAAGAAGTATACGCCTGTATTCATTCTGGTC
ATACTATCAGACAAAGACAGCCCAATTTACGCTCCATTCAAGAGGTTTTGTGAGATGAAA
ATAGGAATTATCTCGCAGTGCATGGTTAAGCCTAGGCAGATCAACGATCAGTATCTTGGA
AATCTTGCGCTGAAGATAAATTTGAAGATGGGAGGGTTTAATTCCCCATTGAGCCGGCGG
ATGCTCACCTGCCTTGGTGAGTCAACCATAATATTTGGGATGGATGTGTCGCATGGATCC
CCTGGAGATTTGAGTGTTCCTTCGATTGCAGCTGTGGTTGCCACCAAAAACTGGCCGGAA
GTGTTCCATTATTCGACGCAAGTTAGAACACAGCCACCCAAGATGGAGATGATAACTGGT
CTTTATGAGCCTAAGGGTGGCATGGTGAGAGAATTGCTTTTGACATATTACAACACATGC
GCCAGAGGCACAAATCCTAAACCAAGTCAAATTATCATCTACAGGGATGGAGTCAGCGAA
AGCCAGTTCGCGGAATGTTTGGAGGTAGAATTTATGGCGTTCAAAAGGGCGTGTGCGGAA
CTGGAAGAAGGCTATAATCCTGGGATAACCTTCATTGTCGCTCAGAAACGTCACAACACA

CGTTTCTTTCCTCAGAACCGAGATTCGTTGAAGAATGGAAACGTTCTGCCAGGTACTGTT
GTCGATAAGGATGTGTGCCATCCTCACAACTTTGACTTCTTCCTCGTCTCTCAAGCTGGA
CTCATTGGTACATCTCGTCCGACTCACTATCATGTGCTGGTGAATGAAAACAAACTTGGG
CCGGATGACATCCAGATGTTGACCAACAACCTCTGTTACACGTTTGGACGCTGTTCGACG
TCAATTTCGATGGCGGCTCCTGCGGCATATGCCCATGTTGTGGCAGGAAGGTATCGGAAG
TTGCTTGACACATGGGGAAGAGGATCTGATACCTCTTCCTTGAGGAGTTCCAAAGAAGGA
GGGGTTGACTCAATGCCTTTACCAGAACTTCCGGCTCTCAAGATCAAGCCCGAATATTCG
ATGTTTTTTTGTTGA
>Physcomitrella-patens_1912837_locus
ATGTATGGAAGGCCCTCCAGACCTTTCCAGCAACCGATTCCTCCAGCACCCAGACCTCTG
CCCCAAGGGGGAAGAGGCCCTTCGTACCCAGGGCAAGCATATGGACGTGGCGGAGGTCCT
CCTGCTCATTTTTCTGCATCACCTCCAAGTTACGGACCAGGTGTAGGTGCAGTTGTTGAG
CAGTTTGGGTCGGTGGTTTTGGGTGGTGTGGGGCATAGTTCCAATGTTGGCCAAATTTCA
GAACCCCATGTTTCTAAATATGAACAGCCATCCACACCTCCACCAATTTCTAGTAAACAG
CTGAGGTTTCCTTTGCGTCCTGACAGAGGAAGGATTGGCCAATGGTGTATCGTGAAAGCA
AATCATTTCTTTGCCGAGCCACCTGACAAAGATCTGCATCAATATGATGTTACAATCACC
CCAGAGGTTCCATCCAGAGGTATCAACAGAGCCGTCATGGAGCAGCTTGTTAAACTTTAC
AGAGAGTCTCACTTGGGCACTAGGCTTCCTGCTTACGATGGTAGGAAGAGTCTGTATACG
GCTGGACCCCTTCCATTTCAGAGCAAGGAATTCGAGATCAGACTCTTGGACGAGGATGAT
GGGACCAACCAACCTAGACGGGAGAGGCCCTTCAAAGTTGTAATTAAATTTGCTGCCCGA
GCAGATTTGGATCATCTCAGACGATTTCTTCTTGGGCGCCAAGCTGATGCTCCTCAAGAA
GTCCTACAAGTCTTAGATATTGTATTGAGGGAGCTTCCTACCCATCGGTATTCTCCTGTA
GGACGCTCTTTTTATTCTCCCAATCTGGGAACTCGACAGCCGCTAGGAGATGGTCTGGAA
AGCTGGCGGGGCTTCTATCAAAGTATTCGACCAACTCAAATGGGGCTGTCCCTAAACATT
GACATGTCTTCCACAGCGTTCATAGAACCCAAGACTGTGATGGAGTTTATAGGGGATCTC
CTCAACAAGGACGTGACACGTGGTCTATCCGATGCTGATCGCATGAAGATCAAGAAAGCT
CTTCGTGGAGTTAAAGTGGAAGTGACTCATCGCGGAAGTATGCGTCGCAAGTACCGCATA
TCTGGGCTTACAAATCAGGCCACCAATGAATTGGAGTTCCCAGTTGATGATAATGGTACC
TTGAAGTCTGTTACCGACTATTTTCGGGAGACGTATGGCTATGTTATCCGGCATCCATCT
TTGCCTTGCCTCCAAGTAGGGAATGCTCAGCGTCCCAACTATCTTCCCATGGAAGTCTGC
AAGATCGTTGAGGGCCAGCGGTACTCCAAACGGCTGAATGAGCGCCAAATCACCGCACTT
CTTAAAGTTACTTGTCAGCGACCACGAGACAGAGAACACGCCATTATGAATACGGTACAC
CATAATGCATACCATCAAGATCCTTATGCTCAAGAATTCGGAATTAGAATCAGTAATGAA
CTTGCGCAAGTAGAGGCTCGAGTCCTTCCAGCTCCACGGCTCAAGTACCATGACACTGGC
AGGGAAAAAGAATGTCTTCCACAAGTTGGACAATGGAACATGATGAACAAGAAAATGGTT
AATGGTGGAATCGTCAACAACTGGGCATGCATCAATTTTTCACGCAATGTCCAGGAAAAT
GTTGCTAAGAGTTTCTGTCAAGAGCTGGCTCAGATGTGTCAGACATCTGGAATGCAATTT
ACTAGGGACCCCGTAGTGCCTCTTCAATATTATCACCCTGATAACTATGACCGAGCTTTG
ATTCATCTATGTGATGACGTGTATAAAAAGACGAAGGGCAAGAGCTTGGATCTCCTCATA
GCTATTCTACCAGACAACAACGGGCCTTTATATGGTGATCTGAAGAAGCAATGTGAGACT
GTCCTGGGAGTGGTTTCACAATGTTGTTTGACTAAGCATGTCTTCAAGATGAGCAAGCAG
TATTTAGCAAATGTGGCCCTCAAAATTAATGTGAAGGTCGGAGGTCGGAATACTGTTTTG
GTAGATGCACTTTCACGCAAGATCCCTCTTGTCAGTGATATTCCCACTATAATATTTGGT
GCAGATGTTACCCACCCTCACCCTGGAGAGGATTTCAGTCCTTCCATAGCTGCAGTGGTA
GCTTCGCAGGATTGGCCTGAGGTCACTAAATATGCCGGATTGGTGTGCGCTCAAGCTCAC
CGTCAGGAGTTGATTCAGGATCTGTACAAAGAGTGGAAGGATCCTCAGAAAGGCTTGATG
ACAGGAGGGATGATCAAGGAGCTCCTGATCTCTTTCTGGCGCGCAACAGGTCAAAAGCCT
CTTCGCATCATTTTTTACAGGGATGGAGTTAGTGAAGGGCAGTTTTATCAGGTGCTTCTG
TTCGAGCTGGACGCAATCAGGAAAGCTTGCGCTTCCCTTGAACCGGATTACCAGCCTCCA
GTCACGTTTGTGGTTGTGCAGAAGAGGCACCACACTCGATTATTTGCCAACAATCACAAT
GATAATCGCTCTACGGATAGAAGTGGCAACATTTTACCAGGGACTGTTGTGGACTCGAAG
ATTTGTCACCCTACGGAGTTTGACTTTTATCTTTGCAGTCATGCTGGAATTCAGGGGACT
AGTCGACCTGCACATTATCATGTACTATGGGATGAAAATAAATTTTCTGCGGACAGTTTG
CAGTCGTTGACAAACAATCTTTGCTATACGTATGCACGGTGCACACGTTCAGTATCTATT
GTTCCTCCGGCTTATTATGCGCATTTGGCTGCTTTCCGAGCGCGCTTCTACATGGATCCA
GAAGCTTCCGATACTGGATCTCTAACTAGTGGGATGGGAGGGGCTAATCGGAGCCAGTAC
ACTGGATCAGCAACATCTCGTACCAATCGTGTGGTGGGAGGAAATGCTGTGCGTCCACTT
CCTCCTCTGAAGGAGAACGTGAAGAGAGTCATGTTCTACTGTTAA
>Populus-trichocarpa__POPTR_0001s22120
ATGGCATCATCTTCGAATGCGCCACCGCCCTCGGCTTCGAGTTCGGCTTCGATGGAGGAG
CTGAGTCAAGAGATCGCAAAAAAATTGTCATTTGGAAGTACTAGTGCGACGGGAGGGTCG
GTGCCTGTGTCATCAAAGGCCATTGTGCCTCCGCCCCGGCCACAACTTGGAAGGATAGGA

AGGAAATGTACGATTAGAGCAAATCATTTTGTAGTTGAGGTTTCTGATAGAGATCTCTTT
CACTACGATGTGGCAATAACTCCTGAAATTACATCGAAGAAAGTAAATAGAGATGTAATA
TCTCAGCTTGTTCGTTCTTATCGTGAGTCCCACTTGGGCAATCGGATGCCAGCTTATGAT
GGCAGGAAAAGTCTATACACTGCTGGGGCATTGCCTTTTGAAGCAAAGGAATTTGTCGTG
AAGTTGGTTGAGAAGAATGATCCTGCTAGTTCATCCAGTTCTGAACGTCAATTTAACGTG
GCAATCAAATATGCATCCAAAGTTGATATGAATCATTTAAAGGAATTCCTAAGTGGCAGA
CAAAAGGATGTTCCACAGGAAACCATACAAATCCTTGACATTGTTCTCAGAGCATCACCA
TCAGAAAAGTACGTTACTGTTGGGAGGTCATTCTTCTCACTTGATTTGGGCAAAAAAGGC
GAGCTTGGTAATGGAATAGAATACTGGAGAGGTTATTATCAAAGTCTTCGGCCAACCCAA
ATGGGACTCTCTTTAAATATTGATGTGTCAGCCAGATCCTTTTATGAGCCAATTTTGGTG
ACTGAGTTTGTGGCCAAGTACTTCAATCTCAGAGACTTGTCAAGGCCTCTTTCTGATCAG
GATCGTGTTAAGGTGAAAAGGGCCTTAAGAGGAATCAAAGTGGAAATTTCATACAGGGAC
TATGCCAGAAGCTTCAAGGTTACTGGCATATCTAATCTACCAGTAGACAAGACAATGTTT
ACTCTGGATGATAAGAAAACAAAGGTGTCTGTCCATCAGTATTTCTGGGACAGATACAAT
ATTGGGCTGAAATATACATCTTTGCCTCCCCTTCAAGCCGGAACTGATGCGAAACCCATT
TATTTGCCCATGGAGCTCTGTAAGATTGCTGGAGGACAGCGATACACAAAGAAACTAAAT
GAAAGACAAGTCACTGCACTTTTAAGAGCTACCTGTCAACGCCCCTTCTGCTAGGGAAAAT
AGCATTAAGGAGGCAAATAATCTCTCTTCGACATCTTTGAATGTGCTTGTAAGGAATGAA
TTTGGTATTCAAGTGAAAGAAGAACTAACATCGGTTGATGCCCGAGTTTTACCCCCACCC
ATGCTTAAATATCATGATACAGGGCGAGAAGCTAGAGTGGATCCACACTTAGGACAATGG
AATATGATTAACAAGAAAATGGTGAATGGTGGCAAAATTGATTTCTGGACGTGTGTGAAC
TTTTCTACGAGAGTGCAGAGAGACTTGCCATTTGAATTTTGTTGGCAATTAATGGATATG
TGCAACAGCAAAGGAATGGAATTCCACCCAGATCCCATTATTCAGATACATTCAGCTGAT
TCCAGGCATATTGAGAAGGCTCTTCATGATGTTCATAAGAAATGTACTGCAAAACTTGCA
AATCAGAAGGGGAAACAGCTTCAACTGCTGATCATTATTCTACCTGATTTCAGTGGATCC
TATGGGAAAATCAAACGTATTTGTGAAACTGAGTTGGGAATAGTTTCACAATGCTGCCAG
CCCCAGCAGGCAAAGAAGCTCAGCAAACAATACCTGGAAAATGTTGCTCTCAAAATTAAT
GTGAAGGCTGGTGGACGGAACACTGTGTTAAATGATGCTATTCAAAGAAGAATTCCTAAT
GTTACTGATCTTCCTACTATTATTTTTGGTGCTGATGTGACCCATCCACAGCCAGGGGAA
GACTCTAGCCCGTCGATTGCAGCAGTAGTGGCTTCTATGGACTGGCCAGAGGTAACCAAA
TATAGAGGACTTGTCTCTGCACAGGCTCATCGCGAGGAAATTATTCAGGATCTTTACAAA
AAATATCAGGATCCACAAAAGGGTTTAGTTCACAGCGGAATGATCAGGGAGCTGTTTATT
GCATTCAGAAGATCAACAGGCCAAAAACCTCATAGAATTATATTCTATAGAGATGGTGTA
AGTGAAGGCCAATTCAGCCAAGTTCTGCTACATGAGATGCAGGCGATACGAGAGGCATGT
GGCACCCTGGAAGAAGGATATTGTCCTCCGGTTACCTTTGTTGTAGTGCAGAAACGGCAT
CATACACGCTTCTTTCCTGCTGACCATAGCAAGCGAGATCTGACTGACAGGAGTGGCAAT
ATCCTACCAGGCACGGTCGTGGACACTAAAATTTGCCACCCTACAGAGTTTGATTTCTAC
CTTAACAGTCACGCTGGAATTCAGGGAACTAGCAGACCTACACACTACCATGTATTGTTT
GATGAAAACAACTTCACTGCTGATGGCTTACAAACCCTCACTAACAATTTGTGCTACACG
TATGCAAGATGCACTCGGTCTGTTTCCATAGTGCCCCCTGCATATTACGCCCATTTGGCA
GCTTTTAGGGCTCGGTATTACATTGAGGGTGAAACATCAGACAGTGGTTCTACTGGTGCG
ACTGGGAGGAGTGTGGAGGCCCGATCTCTTCCAGTCGTCAAAGAAAATGTCAAGGATGTT
ATGTTTTACTGTTGA
>Populus-trichocarpa__POPTR_0001s22710
ATGGAGTCAGCTGATGAACAAAATGGAAATGGGTCACAGGAAGCCCTCCCACCTCCCCCT
CCTGATGTTCCACCAAATGTTGTTCCAGTTAAAGCTGAACCTGAGCCAGTCAAGAAAAAA
CCTCTGCGGGTTCCAATAGCCAGGCGTGGCCTTGGATCCAAAGGCCAAAAGATGCCTCTA
TTGACCAATCACTTTAAAGTCAATGTTACTAATACTGAGGGTTACTTCTTTCACTACTGT
GTTTCCCTTGCTTATGAAGATGGCCGCCCTGTTGATGGTAAGGGTGTTGGAAGAAAGGTG
ATTGATAGGGTGCATGAAACTTATGATACCGAGTTTGGAAAGGATTTTGCTTATGACGGT
GAAAAGAGCTTGTTCACTGTTGGTCCCCTTCCTCGCAACAAGCTTGAGTTCACGGTTGTG
CTTGAGGATGTAGTATCTAACAGAAATAATGGAAATGCAAGCCCTGATGGTCATGGAAGT
CCAAATGAGGGTGACCGAAAGAGGTTGCGCCGTCCCTATCACTCCAAGACATTCAAAGTG
GAGATCAGTTTTGCTGCAAAAATCCCCATGCAAGCCATTGCAAATGCCTTGCGTGGTCAG
GAATCAGAGAATTCCCAAGAAGCCTTTAGAGTGCTGGATATTATATTGCGACAGCATGCC
GCCAAGCAGGGCTGCCTCCTTGTGCGCCAATCCTTCTTCCATAATGATCCAAAAAATTTT
GTGGATTTGGGAGGCGGTGTTCTTGGCTGCAGAGGTTTCCACTCAAGTTTTAGAACATCT
CAGGGAGGCTTGTCTCTTAATATTGATGTGTCTACGACCATGATAATACAGCCAGGTCCT
GTGGTAGATTTTCTAATTGCCAACCAAAATGTGAGAGATCCATTTTCACTTGACTGGGCG
AAGGCGAAACGAATGCTCAAAAATCTGAGGGTTAAGGCAAGTCCTTCCAATCAAGAGTAC
AAGATAACTGGGTTGAGTGAGAAGACTTGTAAAGAACAAATGTTTCAGTTGAAACAAAAA
AATGGAGGGGATGGTGGGATCGAGGCTGTTGAAATAACTGTTTATGATTATTTTGTCAAT

CACCGCAAAATCGATTTACGATATTCTGGTGATCTGCCATGCATTAATGTTGGGAAGCCA
AAGCGCCCTACTTATATTCCTCTTGAGCTTTGTTCCCTGGTGTCCCTACAACGCTATACC
AAAGCACTGTCCACACTTCAAAGGTCTTCACTGGTGGAGAAATCACGGCAGAAGCCGCAA
GAAAGGATGACTGTTTTATCTAGTGCTCTGAAGAGCAGCAAGTATGATGCTGAACCTATG
CTACGCTCGTGTGGCATTTCAATCAACCCTAGTTTCACACAAGTGGAAGGCCGTGTTCTG
CCTGCTCCGAAGCTGAAAGTTGGCAACGGGGAAGATTTCTTCCCAAGAAATGGACGTTGG
AATTTCAATAACAAGAAACTTGTGGAGCCATCTAGGATCGAGAAGTGGGCTGTCGTGAAC
TTCTCAGCTCGCTGTGATATACGCAACCTTGTACAAAATCTCACAAAATGTGCAGAGATG
AAAGGAATTCCCATAGAAGATCCTTTTGATGTATTTGAGGAGAATCCACAATCAAGACGT
GCCCCACCAGTGGTTAGAGTGGAGAAAATGTTTGAGCAAATTCAGTCTAGACTTCCTGGG
CAACCAAAGTTCTTACTGTGCTTGCTTCCTGAGAGAAAGAATTCTGATATATATGGCCCA
TGGAAGCGCAAAAATCTTGCTGAATATGGAATTGTCACTCAGTGCATTGCGCCTCAAAGA
GTTAACGACCAATATATTACCAATGTTCTCCTGAAGATCAATGCAAAGCTTGGTGGGTTG
AACTCTATGTTGGCTGTGGAACACGCCCCCTCATTACCTCTTGTGTCGAAGGTTCCCACG
CTTATCCTTGGGATGGACGTGTCCCATGGCTCTCCTGGGCAGTCTGATGTCCCTTCAATT
GCTGCGGTAGTCAGCTCCAGGCAGTGGCCTTTGATTTCTCGCTATCGGGCATGTGTGCGA
ACACAATCCCCAAAGCTTGAGATGATTGATTCATTATTTAAGCGAGTGTCTGAGACTGAG
GATGAAGGAATAATTAGGGAGCTTCTGTTAGACTTTTATGTGACTTCAGGAAAAAGGAAA
CCCGATCAGATCATCATATTTAGAGACGGGGTCAGTGAATCACAATTCAATCAGGTCTTG
AATATCGAATTGGATCAGATAATTGAGGCGTGCAAGTTTCTTGATGAGAAGTGGTCCCCA
ACGTTTGTGGTAATTGTAGCTCAGAAAAACCACCACACTAAATTTTTCCAACCTGGATCT
CCTGATAATGTACCACCTGGTACGATCATTGACAACAAAGTCTGCCATCCAAGAAACAAT
GACTTCTATCTCTGTGCTCATGCTGGGATGATTGGGACTACAAGGCCTACTCACTACCAT
GTTTTGTTAGACGAGGTTGGTTTTTCAGCAGATGATCTGCAGGAACTTGTGCATTCCCTC
TCATATGTATACCAAAGAAGCACGACTGCCATCTCTGTAGTTGCACCAATCTGCTATGCC
CACCTGGCAGCTACTCAAATGGGCCAGTTTATGAAGTTTGAAGATACTTCTGAGACTTCC
TCAAGCCATGGTGGGGTGACCTCTGCAGGAGCTGTTCCTGTCCCACAGTTGCCGAGGTTG
CAGGAGAAAGTTTGCAATTCCATGTTCTTTTGTTGA
>Populus-trichocarpa__POPTR_0006s02680
ATGGAATCCACTGAAGAGCCAGAGGCCCTCCCTCCACCTCCATCCGATGCCCTCCCTCCA
CCTCCACCTGATGCCCTCCCTCCACCTCCACCTGAGATACCGCCAAATGTAGTTCCTGTT
CAATTGACAACAGACTCTGTTCCGGAGGAAACAAAAAAGATATCAAAACCAAAACGTTCC
CCGATTGCCAGGCGTGGATTTGGGTCTAGAGGGCAAAAAATACAACTGCTCTCAAATCAT
TTCAAAGTTTCCATCTCTAATACTGGTGGCCACTTTTTTCATTACTGTGTTTCCTTGTCT
TATGAGGATGGTCGCCCTATTGATGCAAAGGGCATTGGGAGAAGATTAATTGATAAAGTT
CATGAGACCTATGGCTCAGACCTTGCTGGGAAGGACTTTGCATATGATGGAGAGAAGAGC
TTATTTACAATTGGTGCTCTGCCTCGAAACAAAATGGAATTCACTGTTTTGCTCGATAGT
TTCTCATCAAATAGGAATTCTGGAAATGGCAGTCCTGTTGGCAACGGAAGTCCAAACGAG
ACTGATAAAAAGAGGATGAGGCGGGCATTCCAGTCCAAAACATTTAAAGTGGAGATGAGT
TTTGCTGCCAAAATCCCTATGCAGGCTATTGCAGCTGCTTTGCGTGGTCAAGAATCAGAA
AACTCACAGGAAGCCTTAAGAGTCTTAGACATCATTTTAAGACAGCATGCAGCCAAACAG
GGTTGCCTTCTTGTTCGCCAGTCATTCTTTCACGATGATCCAAAGAACTATGTTGATCTG
GGAGGAGGTGTCCTTGGATGCCGAGGATTTCATTCGAGCTTTAGAACCTCGCAGGGTGGA
TTATCCCTAAATATAGATGGTTCGACTACAACGATAATACAGCCTGGGCCTCTTATTGAC
TTTCTCATAGCCAACCAGAATGTGTCAAACCCCTTTCAGATTGACTGGGCAAAGGCTAAG
CGAACATTGAAAAATCTGAGGATAAGGGTGTCACCTACCAATCAAGAGTACAGAATCACT
GGCTTGAGTGAAAATACTTGCAAAGAGCAAATGTTCTCTCTGAAATCAAGAGCATCTGAT
GGAAATGATGTTGAAAGTGTTGACATTACAGTTTACCATTATTTTGTAAATCATCGCAGC
ATAGATTTACGCTACTCTGGAGATTTGCCTTGCATCAATGTTGGCAAGCCTAAAAGGCCC
ACTTACATTCCTGTCGAGCTTTGTTCACTGCTTCCCTTGCAACGCTATATAAAGGCACTA
ACTGTCCTTCAGAGATCACAGTTAGTAGAAAAATCAAGACAAAAACCACAAGAAAAAATT
AGGATCTTAACTGATGTTATGAAAAGCAACAACTATGCTGCAGAACAAATGTTGCGTTCT
TGTGGTATCACCATCAGCAGCCAGTTTACTCAAGTTCAAGGCCGTGTCCTAACTGCTCCA
AAGTTAAAGGCAGGAAATGGCGAGGATGTTATTCCAAGAAATGGGCGGTGGAATTTTAAT
CATAAGAAATTTTTCGAACCTTCTAAAATTGAAAACTGGGCCGTGGTGAACTTTTCTGCT
CGTTGTGATGTGCGTGGTCTAGTCAGAGATTTGATAAGATTTGGAGAAATGAAAGGGATT
CTCATAAGTGACCCGGTGGATGTTGTTGAAGAGAATGGTCAGTTTCGACGGGCACCGCCT
CTTGTTCGAGTGGAGAAGATGTTTGAACAGATACAGAAAGCATTTCCAAATGCACCTCCT
CGCTTTCTCGTGTGTCTTCTTCCTGATAGGAAGAACTCTGACATATATGGTCCTTGGAAA
AGAAAGAATCTTGCAGAATATGGAATTTTCAATCAATGCCTGGCACCCACTAGAGTTAAT
GAGCAGTATATACTTAATGTTCTCCTGAAGATAAATGCTAAGCTTGGTGGTTTGAATTCT
TTGTTGGCCATGGAGCAATCACGAAACATCCCATTCGTCTCGAAGGTTCCTACAATAATA

TTTGGAATGGATGTATCACATGGTTCGCCTGGTCAGTCTGACATGCCCTCCATTGCTGCG
GTTGTCAGTTCTAGAAACTGGCCTCTACTTTCTCGTTATAGAGCTTCTGTGCGTAGTCAG
TCACCAAAAGTTGAGATGGTAGATTCTCTTTTTACACTAACACCGGATAAGAAAGATGAT
TCTGGGATTGTCAGGGAATTGTTGTTGGACTACTATAGGAGTTCTGGCCAAACAAAACCA
GCTCAGATAATCATATTCAGGGATGGAGTTAGCGAGTCACAGTTCAATCAAGTCCTCAAC
ATCGAGCTGGATCAAATCATTGAGGCATGCAAGTTCCTTGATGAAAGCTGGTCACCCAAG
TTCACTGTTATTGTTGCACAGAAAAATCATCACACTAAATTTTTCCAAGATGGATCTCCA
GACAATGTTCCTCCTGGAACCGTTATTGATAATGCTGTTTGTCACCCACAAAGCTATGAT
TTCTACATGTGTGCACATGCAGGGATGATAGGAACAACAAGGCCAACACATTATCACGTT
CTTTTAGATGAGATTGGCTTTTCAGCTGATGATCTACAGGAGTTGATTCACTCTTTGTCT
TATGTGTACCAAAGAAGCACAACAGCAATATCTGTAGTTGCTCCTGTCCGTTATGCTCAC
TTGGCAGCAACTCAGATTTCACAATTCTTGAAGTGTGATGACATGTCAGAGACATCCTCG
AGCCATGGAGGTCTAACTTCTGCTGGGCAAACCCCTGTGCCCGAGCTTCCTGAGCTACAC
CGGAATGTCTGCAGCTCTATGTTTTTCTGCTGA
>Populus-trichocarpa__POPTR_0006s12010
ATGTACGGGAGAGGTCGACGCGGTGGCTCGCCTGCTCCGACAAAAGGAGGAGGGCGTGGC
AGAGGACGCGGAGCTCCACTTCCTTCTCCTATGGCTTCGTCCGAGGCCGACTCTATCTCA
TCCGTGAGCCAGCTCGGTGGTGAGATGGAGCGGCTTAGTGTTCAAACTGAACCACCTGCT
CCTACTCAGGCGCCAGCGGCTATTCCTGCACCGCAACAACAGAAGCAACAGCAGCAGCAA
CTCGTACCGGCTTCCTCTGTGAAGTTCGCTCAAAGACCTGATCACGGTACGGTGGGATCC
CGGTGTCTCATTAGAGCTAACCACTTTCTTGTTGAGCTCGCTGACCGAGACTTGCATCAC
TACGATGTTTCTATAACTCCTGAGGTTGCATCTCGAGGGGTCAACAGAGCAATAATGAGA
GAGCTGCTTGCTTCGAATAGCACACACTTCCAGAGTAGAAAACCTGCTTATGATGGAAGA
AAGGGATTCTACACTGCTGGACCTTTAACATTCACCTCAAAGGATTTTGTGGTTACCCTT
GTAGACAAAGATGATCAAGGATCTGTGAGAAAAGAGAGGAAATTCAAAGTCACTGTCCGG
TTGGCATCCAAAACAGACCTTTATCATCTCAAGGAGTTCCTGCAGGGAAGACAAAGGGGT
GCGCCGCATGATACCATACAAGTACTCGATGTAGTCCTAAGGGAACCACCATCCAACAAG
CAAGTTTGCACCATCGTTGGGAGGTCTTTTTTCACAGCAGGTCTGGGTGGCCAAAATGAG
ATTGGTAATGGTATAGAATGCTGGAAGGGATTCTACCAGTCTCTACGCCCAACGCAGATG
GGAATGTCTCTTAACATAGATGTATCAGTCGCTGCCTTCTACGAGCCGATTCTTGCTGTT
GACTTTGTTGCAAAACTATTAAATTTAGGAGATCCAATTAGAGCAGCAACCAGGCCTTTG
TCAGATAGTGATCGAGCAAAGTTGAAAAAAGCTTTGAGAGGAGTCAGAGTAAAAGTTACC
CATGGAGAGGAGAAGCGTTACAAAATCACTGGAATATCTGCTTCAGCAACAAACCAACTA
AGGTTTGCTGCTGAAGATGGAAAACAAAAATCAGTTGTCCAATACTTCCTGGAAAAATAC
AATATAAGGCTTCGTTTTGCATCCTGGCCTGCTCTTCAATCAGGAAATGATTCAAGACCA
ATATTCCTGCCTATGGAGTGTTGCAAGATTATCGAAGGACAAAGGTACTCAAAGAAGTTG
AATGAGAAGCAGGTGACAGCCTTATTGAGAGAGGCCTGCAGGCGCCCTGTTGAGAGAGAA
CATAGTATTGAGCAGATTGTTCATTTTAATGACGTTGCACAAGATGATCTAGCCAAAGAA
TTTGGAGTCAGTGTCAAAAAGGAGCTGACTTGCATCGATGCCAGAGTCTTGCCACCTCCG
GTGCTCAAGTACCATGATTTGGGAAAAGCTAGAACTGTAAGACCCCGAGTGGGGCAGTGG
AACATGATTAATGCTAAATTGTTCAATGGGGCCACAGTGAACTTTTGGATGTGTGTCAAC
TTCTCAAGTCTTGGTGAGCAAATGGCTGCTAGCTTTTGTCGAGCCCTGGTAGGCATGTGC
AACAACAAGGGGATGGTCATAAATCCTGCACCTGTATTCCCAATACGGTCTGGCCATCCT
AACCAGCTGGAGAAAACATTGGCCGAAGTTCACAGCATGTGTAATAATGAAAGAAAACAG
CTTCAAATCTTAATTATCATTCTCCCTGATGTCAGTGGAAGTTATGGCACAATAAAAAGA
GTATGCGAAACTGAACTTGGGATAGTTTCTCAATGCTGTCAACCCAAGCAAGCAAGAAAG
TGTAGTCCTCAATACTTGGAAAATGTTGCGCTGAAAATTAATGTGAAGGCTGGAGGGCGA
AACACAGTATTAGAGGATGCCCTGAATAGGAGAATACCTCTTCTAAGTGACACCCCAACT
ATAATCTTTGGTGCTGATGTAACCCATCCACAACCAGGGGAGGATTCTAGCCCTTCAATA
GCTGCGATTGTGGCATCAATGGACTGGCCTGAAGTAACCACCTACAGGGGCCTGGTATCT
GCTCAGAAACATCGTCAAGAGATTATTCAAGATTGTGCTGGAATGATCAGGGAACTTATG
ATTGCTTTCAGAAGAACAACTAATCAGAAACCTAGCAGAATAATTTTCTATAGGGATGGT
GTTAGTGAGGGCCAGTTCAGCCAAGTTCTCCTGTATGAGATGGATGCTATCCGAAAGGCT
TGTGCATCTCTGGAACCAAACTATTTGCCACCAGTTACTTTTATTGTAGTGCAGAAGAGA
CACCATACTCGGCTCTTTGCTACAAATCCTAACCAAACAGACAAGAGTGGAAACATCCTT
CCTGGAACGGTTGTTGATACAAAGATATGCCATCCTTCAGAGCACGACTTTTATCTCTGC
AGTCATGCAGGAATTCAGGGGACTAGCAGGCCGGTGCACTATCATGTGTTGTGTGACATG
AACAAATTCACTGCTGATTGCCTGCAGATGTTGACGAACAACCTGTGCTACACGTATGCA
AGGTGCACCCGCTCTGTTTCTGTGGTTCCTCCTGCATACTATGCACACTTGGCAGCATTC
AGGGCAAGATACTACATAGAGGGGGACATTGCATCTGATAGTGGCGGCGGCGGCACAGGA
CCTCCTGTGAGGAGGGAAGCTGCCCCTGTCCGCCCACTTCCAGCCATCAGCCCCAACGTG
AAGAATGTTATGTTTTACTGTTGA

>Populus-trichocarpa__POPTR_0008s01100
ATGGAGTCTTCAGATTCACGGAAAGATTTGCCACCACCTCCGGCTATAATTCCTGCTGAC
GTGGTGAAAACAGAATTGGGTCCAACTTGTGAACAAACAAAGAAAGCAGCAACCCCAAAA
CGAGTTCCCATGGCTCGCCGTGGCTACGGAGCTAAAGGACAGAGAATACAACTACTAACT
AACCATTTCAAAGTGGCAGTGCCCAAGTCAAATGACCACTTCTACCAATACAGTGTTGCT
CTATTTTATGAAGATGGCCACCCGACCGATGGAAAGGGAATCGGAAGAAAGGTTATGGAC
AAAGTCCAGGAGACCTATGATTCTGAGCTGGAAGGTAAGCAGCTTGCTTATGACGGGGAG
AAGACCCTGTTTACGACTGGTTCTCTTCCACACAACAAGCTGGAATTCACTGTTGTGTTG
GAAGATGTCTCCTTAACCAGAGGTGGAGATAACGACAGTTCCAGGGGTAATGGCAGCCCC
AGTGAAAGTGATCAAAAGCGAAGGAAGCGGCCCTACCACTCCAAAACGATTAAAGTACAA
ATCAGCTACGCAACTAAAATTCCAGTTCAAGCAATTGCAGCTGTTCTGCAGGGTCAAGAA
TCTGAGCATTTTCAAGAAGCTGTCAGAGTTCTAGACATTGTGCTAAGACAGAATGCAGCA
AGGCAGGGTTGCCTGTTGGTCCGACAGTCGTTTTTCCATAATAATCCAAGAAACTTCGTT
GAGTTGGGAGGTGGTGTCATGGGATGCCGTGGTTTTCATTCAAGTTTTCGAGCTGCCCAG
GATGGCCTATCCCTGAATATTGATGTATCAACCACCATGATAGTTAAACCTGGACCAGTA
GTGGACTTCCTAATCATGAATCAGAATGTTCGAGATCCTTACCATATTGACTGGACAAAG
GCTAAGAGGATGCTTAAAAATTTGAGGATTAAAACTAATCACTCCAACACTGAGTACAAA
ATCACTGGATTGACTGAAAAATCCTGCAGAGAGCAAACTTTCTCGCTAAACCAAAGAAGT
GGACGGGACGGGGATGGTGAAGTGCAAACTATTGAGGTTACGGTTTATGATTACTTTGTC
AATCATCGCAACATGGGATTGCAGTATTCAGCTGATTTCCCGTGCATTAATGTTGGGAAA
CCAAAGCGCCCATCGTATTTTCCTCTTGAGCTGTGCAATCTGGTTTCGTTACAACGCTAC
ACCAAAGCGTTGTCCAGCCTGCAAAGGGCTTCCCTTGTGGAGAAATCACGGCAGAAGCCC
CAAGAGCGGATGAGATCTTTGACTGATGCTTTGAGAAGCAGCAATTATGATGCTGATCCA
ATGCTTCGTTCTTCTGGAATTTCAATTAGTGCTCAGTTTACTCAAGTTGAAGGTCGTGTT
TTATCTGCTCCCAGGTTAAAAGTGGGAAATGGAGAGGACTTCTTCCCTCGAAATGGGAGA
TGGAATTTCAACAACAAGAAACTAGTGGATCCAGTGAAGATTGAAAAATGGGCTATAGTA
AACTTCTCTGCACGTTGTGACATACGTTATCTGTGCAATAACTTGATAAAGTGTGGAGAC
ATGAAAGGCATTAGCATAAGTAATCCATTTGAGGTATTTGAAGAGAGTCCCCAGTTTAGG
CGAGAATCTGCTCCTGTAAGAGTGGAGAGAATGTTCGAGGCCATTAAGTCTAAGCTTCCA
GGGCCGCCACAATTTCTGTTGTGTATTCTTCCAGAGAGGAAGAACTCTGACATTTATGGT
CCATGGAAAAGGAAAAATCTTTCTGATTTAGGGATCGTCACACAGTGCATTGCACCTACA
AAGGTGAATGATCAGTACCTGACAAATGTGCTACTGAAAATCAATGCCAAGGTAGAATTT
TGCAATGTTATACCGTTTGAGCTTGGTGGAATGAATTCCTTGTTATCAATTGAGCATGCT
CCTTCTATACCTTTGGTGTCTAAGCTTCCCACCTTAATACTTGGGATGGATGTATCACAT
GGCTCTCCTGGTCATTCTGATGTGCCATCTATTGCTGCGGTGGTAAGTTCCAGGCACTGG
CCATTAATTTCTCGCTACAGAGCTTCTGTTCGCACCCAGTCACAGAAGGTTGAAATGATT
GCAAATCTATTCAAGCCTGTTGCTGGTACTCGTGAGGATCAGGGCATTATCAGGGAGTCA
CTTCTGGACTTCTACTCGAGCTCAGGAAAAAGAAAGCCTGATCAGATAATTATTTTCAGG
GATGGAGTGAGCGAGTCACAGTTCATCCAAGTTCTAAACATTGAACTTGAACAAATTATT
GAGGCCTGCAAGTTCTTGGATGAGAACTGGTGCCCGAAATTCATGGTGATTGTTGCCCAG
AAAAATCACCATACCAAGTTCTTCCAGTCGGGATCACCTGATAATGTTCCACCTGGTACT
GTCATTGACAACAAGGTTTGCCATCCCAGAAACAATGACTTCTACATGTGTGCTCATGCT
GGGATGATTGGAACGACTCGGCCTACTCATTACCATGTTCTACATGATGAGCTTGGTTTT
TCTGCAGATGATTTACAAGAGCTTGTGCACTCCTTGTCATATGTGTATCAGAGGAGCACC
ACTGCCATATCTGTAGTTGCTCCAATTTGCTATGCCCACTTAGCTGCAAGCCAGATGACT
CAGTTTATCAAATTCGATGATTTGTCAGACACATCCTCTAGCCATGGTGGGGTTACTGTT
CCAGGTGCTGTTCCAGTTCCAGAATTACCTCGTCTGCATAATAATGTCAGCAGTTCGATG
TTCTTCTGTTAA
>Populus-trichocarpa__POPTR_0008s15860
ATGCCTGTAAGGCAAATGAAAGAGAGCTCGGAGCAGCACTTAGTGATAAAAACCCACATG
CAGAACTCCATGAACCAACCACAAAAACACCACAAAACTGCTCAAAATGGAAAAGGACCA
CCACAACCACTCCAAGAAAGTTCCAACACTAAACCCCAAAACCAGGCCTCACCTCCTGCA
AAGAACAGAGGGAGGAGAAGAGGCAGAGGTGGTAGAAAGTCTGATCAAGGAGATGTATGT
ACGAGGCCTAGTTCAAGGCCTTGTACAGTGGCACATAAACCTGTTCTGAACCCAACAGGT
GATCTCCTGGCAAATGCTTCTAATGGGCATATTGAGAATAGTAAAAATGTTTGTGAAATG
GAGATGGGTTTGGGGTTCCCTACCTCAAGCAAGTCTTTGAGTTTAGCTCCTAGGCCTGGT
TATGGTCAAGTTGGGACTAAGTGTATTGTTAAAGCCAACCATTTCCTTGCAGAGTTACCA
GACAAGGACTTGAACCAGTATGATGTTACCATAACTCCTGAAGTGGCATCAAGAACTATG
AACAGAGATATTATGGCAGAGCTCGTGAGGCTTTACAAAGATTCTGACTTGGGAATGAGA
CTGCCTGCTTATGATGGCAGAAAGAGCTTGTACACGGCTGGTGAGCTTCCTTTTGCTTGG
AAGGAGTTCATAATTAAGCTTATTGATGAAGAGGATGGTATCAATGGTCCCAAGAGAGGG
AGGGAATACAAGGTGGTGATCAAGTTTGTCGCGAGGGCCAATATGTATCATTTAGGTCAA

TTCTTAGCTGGGAAACGTGCTGATGCCCCGCAGGAAGCTCTACAGATTCTTGACATTGTA
CTGAGAGAGCTATCTTCAAAGAGGTATTGCCCTGTCGGAAGATCCTTCTTTTCACCTGAT
ATAAGAGCACCACAACGACTTGGTGATGGCTTAGAGTCCTGGTGTGGATTTTACCAGAGT
ATAAGGCCTACTCAAATGGGACTGTCCTTAAATATTGATATGGCCTCAGCTGCTTTCATC
GAGCCTCTTCCTGTGATAGAGTTCGTTGCCCAGCTTCTAGGCAAAGATATATTATCAAGG
CCATTGTCTGATTCTGATCGAGTTAAGATTAAGAAGGGTCTCAGAGGAGTGAAAGTTGAA
GTTACTCACAGAGGGAGTGTACGAAGAAAGTACCGTGTCTCAGGATTGACATCTCAGCCT
ACAAGAGAACTTGTGTTTCCTGTTGATGATAACTCGACCATGAAGTCAGTTGTTGAATAC
TTCCAGGAGATGTATGGCTTTACCATTCAACATACACATCTACCTTGCCTTCAGGTTGGA
AACCAGAAGAAAGCAAACTATCTACCTATGGAGGCTTGCAAAATTGTGGAGGGGCAGCGT
TATACAAAAAGGTTGAATGAGAGGCAAATTACTGCCCTCTTAAGAGTTACATGCCAAAGA
CCCAGGGATCGAGAAAATGACATTTTACAGACAGTTCAGCATAATGCTTATGATCAAGAT
CCTTATGCAAAGGAATTTGGTATCAAAATCAGTGAAAAGCTAGCTTCTGTTGAGGCTCGA
ATTCTCCCTGCCCCTTGGCTGAAATATCATGAAACTGGAAAAGAAAAGGATTGCCTGCCT
CAAGTTGGGCAATGGAATATGATGAACAAGAAAATGATTAATGGAATGACTGTAAGCCGG
TGGGCATGTATTAACTTCTCTAGGAGTGTGCAAGAGAGTGTTGCTCGTGGCTTTTGCAAT
GAACTTGCCCAAATGTGTCAAGTTTCTGGAATGGAGTTCAATTCAGAGCCTGTGATCCCT
ATTTACAATGCCAGGCCTGAGCATGTAGAGAAAGCTTTGAAGCATGTGTATCATGCATCC
ACGAACAGAACCAAAGGAAAAGAGCTAGAGCTTCTCTTAGCTATTCTACCTGACAACAAT
GGGTCCCTCTATGGTGATCTGAAGCGAATATGTGAAACTGATCTTGGTTTAATAACTCAA
TGTTGTCTCTCAAAACACGTTTTCAAGATCAGTAAGCAGTACTTGGCAAACCTGTCGCTC
AAGATCAATGTAAAGATGGGTGGTAGGAATACCGTCCTTTTAGATGCTATAAGCTGCAGA
ATACCATTAGTTAGCGACATACCGACAATTATTTTTGGAGCAGATGTGACTCACCCAGAG
AATGGAGAGGACTCAAGCCCCTCGATTGCTGCTGTGGTAGCTTCTCAGGACTGGCCTGAA
GTAACAAAATACGCTGGATTGGTATGTGCTCAAGCTCACAGACAGGAACTCATACAGGAC
TTATACAAAACATGGCAAGATCCTGTTCGTGGTACTGTAAGTGGTGGCATGATCAGAGAT
CTCCTGATTTCTTTCAGGAAAGCAACGGGGCAAAAGCCGCTAAGGATCATATTTTATAGG
GATGGTGTAAGTGAAGGGCAATTTTATCAAGTTCTGCTTTATGAGTTGGATGCAATTCGG
AAGGCCTGCGCTTCTTTGGAGCCAAATTATCAGCCACCGGTGACTTTCATTGTTGTACAA
AAACGTCACCACACAAGATTGTTTGCTAACAACCATAGGGACAGGAATAGCACAGACAAG
AGTGGAAACATATTACCTGGCACTGTGGTTGATTCTAAAATCTGTCATCCAACGGAATTT
GACTTTTATCTCTGTAGCCATGCTGGTATTCAGGGAACAAGTAGACCAGCACACTATCAT
GTGTTGTGGGATGAGAACAATTTCACAGCTGATGGAATCCAGTCCTTGACAAACAATCTC
TGTTACACATACGCAAGGTGCACGCGGTCTGTTTCAGTGGTTCCACCCGCATACTATGCA
CATTTAGCTGCTTTTCGTGCTCGATTTTACACGGAGCCAGTAATGCAGGAGAATGGCTCA
GCAGGCAGTGGTGCTTGCCATGGTGCTAAGGGAACGCGGACAGGAGAATCTGGTGTCCGG
CCACTTCCAGCCTTGAAAGAGAATGTAAAGAGAGTAATGTTTTATTGTTAG
>Populus-trichocarpa__POPTR_0009s00660
ATGTCTCGCCGTGGTGGCGGCCGACGATCCGAAACTCGTCGTGACCAGGACTCATCCGCT
CCGTCACCTTCATTTCAGCGCGGTGGTGGTGGTGGAGGCCGTGGCCGTGGTGGAAGAGGC
GTCTCCACTCCTGCTCCGTCTCCAACTCCAGTTCGTGCTCCTACCTCGGCGGCACCCGGC
TACCTCTCCGCAACCGCAGGTCCTTCGGCAAAACCAACTCCTCAAGCGGCTTATACAGCG
GCGGCGGCTTCGAGTTCCTCTTCAGTCGGGGAGCTGAGTCAAGAGACCGCAAAGAAGTTG
ACATTGGGAGGGCTGGTGCCTGTCTCTTCAAAGGCGATAGTGCCTCCGCGACGACCAGAT
TATGGAAAGATAGGGAAGAAATGTGTGATTAGAGCGAATCATTTTGTAGTTGAGGTTTCT
GATAGAGATCTCTTTCACTACGATGTGGCAATAACTCCTGAAATTACATCAAAGAAAGTC
AATAGAGATGTAATATCTCAACTTGTTCGTTCTTACCGTGAGTCTCATCTGGGCAACCGG
ATGCCAGCTTATGATGGCAGGAAAAGTCTATACACTGCTGGGGCATTGCCTTTTGAAGCA
AAGGAGTTTGTTGTGAAGTTGGCTGAGAGGGGTGATCCTGCCAGTTCATCCAGTTCTGTC
AAAAAGGAACGTCAATTTAAGGTGGCCATCAAATATGCATCCAAAGTTGATATGTATCAT
TTGAAGGAGTTCCTTAGTGGTAGACAAGCGGATGCTCCACAGGAAACCATACAAATCCTT
GACATTGTTCTCAGAGCATCACCATCAGAGAAGTACATTACTGTTGGGCGATCGTTTTTC
TCACCTGATTTGGGTCCAAAAGGCGATCTCGGTGATGGAATAGAATATTGGAGGGGGTAT
TATCAAAGTCTTCGGCCAACCCAAATGGGACTATCTTTCAATATTGATGTGTCGGCCAGA
TCCTTTTATGAGCCAATTTTGGTAACTGAGTTTGTAGCGAAGTACTTTAATTTTAGAGAC
TTATCAAGGCCTCTTTCTGATCAGGAGCGTGTTAAGGTGAAAAGGGCCTTGAGAGGAATC
AAAGTACAAATTACTTACAGTGACTACACTAAAAGCTACAAGGTTACTGGCATATCTAAT
CTACCAGTAAACAAAACAATGTTTACTCTGGATGATAAGAAAACAAAGGTGTCAGTCTAT
CAGTATTTTTTGGAGAGATACAATATTGGGCTGAAATATACATCTTTGCCTCCCCTTCAA
GCTGGAACTGATGCAAAACCTATTTATTTGCCCATGGAGCTTTGTCAGATTGCTGGAGGA
CAACGATACACTAAGAAGCTAAATGAAAGACAAGTCACAGCCCTTTTAAGAGCAACCTGT
CAACGGCCTTCTGCTAGGGAAAATAATATTAAGCAGGCAAATAATCTCTCCTTGACATCT

TTATTTCCATCATTGCGAATCTTGATTTTTATGGTAAGGCAAAATGATTACAGTAAGAAT
GCGCTTGTGAGGGATGAATTTGGAATTCAAGTGAAAGAAGAACTCACATTGGTTGATGCT
CGAGTATTACCCCCTCCCATGCTTAAATATCATGATACAGGGCGAGAAGCTAGAGTGGAT
CCGCGCTTTGGACAATGGAACATGATAGACAAGAAAATGGTGAATGGAGGCAGAATCGAT
TTCTGGACCTGTTTGAACTTCTCTACAAGAGTGCACAGAGAGTTGCCATCTGAATTTTGT
TGGCAGTTAATGGATATGTGCAATAACAAAGGAATGGAATTCAACCCAGAACCCATTATT
CCAATACGCTCAGCTGATTCTAGGCAAATTGAGAAGGCTCTTCATGATGTTCATAAGCAA
TGCACTGCAGAACTTGCAAATCAGAAGGGGAAACAGCTTCAGCTGCTGATTATTATTCTA
CCTGATGTCACCGGATCCTATGGGAAAATCAAACGTGTTTGTGAAACTGAGTTAGGAATA
GTTTCACAATGCTGTCAGCCCCAGCAGGCAAAGAAGCTTAGCAAACAATACATGGAAAAT
GTTGCTCTCAAAATTAATGTGAAGGCTGGGGGACGAAACACTGTATTGAATGATGCTTTT
CATAGAAGAATTCCTCTTCTTACTGATGTTCCTACTATTGTTTTTGGTGCTGATGTAACC
CATCCACAAGCGGGAGAAGACGCAGGCCCATCAATTGCAGCAGTAGTTGCTTCTATGGAT
TGGCCAGAGGTAACCAAGTATAGAGGACTTGTCTCTGCACAGGCTCACCGTGAAGAAATT
ATCGAGGATCTTTACAAAAAATACCAGGATCCAAAGAAGGGTTTAGTTCATGGTGGAATG
ATCAGGGAACTGTTAATTGCTTTCAAAAGATCCACCGGCCAAAAACCTTTTAGAATTATA
TTCTACAGAGATGGTGTTAGTGAAGGTCAATTCAGCCAAGTTCTGCTGCATGAGATGCAG
GCAATACGACAGGCATGTGGCTCCCTAGAAGAAGGGTATTGTCCTCGGGTTACCTTTGTA
GTAGTTCAGAAAAGGCATCATACACGCTTTTTCCCTGCTGACCATAGCAGGCGTGATCAG
ACTGACAAGAGTGGCAATATTTTACCAGGTACAGTTGTTGATACTACAATTTGCCACCCT
ACAGAGTTTGACTTCTACCTAAACAGTCATGCTGGAATTCAGGGAACCAGCAGACCTACA
CATTACCACGTGTTGTTTGATGAAAACAACTTCAGTTCTGATGGTTTACAAACCCTCACT
AACAATTTGTGCTACACGTATGCAAGATGCACTCGGTCTGTTTCCATAGTGCCTCCCGCA
TATTATGCTCATTTGGCAGCTTTTAGGGCGCGATATTACATTGAGGGTGAAACATCAGAT
GCTGGTTCATCTGGTGGGACTGCTGAGTTCCGTCCTCTTCCGGTTATCAAAGAAAATGTG
AAGGATGTTATGTTTTACTGTTGA
>Populus-trichocarpa__POPTR_0010s09150
ATGAACAGAGCTATTATGGCAGAGCTCGTGAGGCTTTACAAAGAATCTGATTTAGGAATG
CGACTGCCTGCTTATGATGGCAGAAAGAGCTTGTACACAGCTGGTAAGCTTCCGTTTGCT
TGGAAGGAGTTCGCTATTAAGCTTGTTGATGCACAGGATGGTATCAGTGGTCCCAAGAGA
GAGAGAGAATACAAAGTGGTGATCAAGTTTGTTGCAAGGGCAAACATGTATCATTTGAGT
CAATTCTTAGCTGGAAAACATGCTGATGCTCCGCAGGAAGCTCTACAGATTCTTGACATT
GTACTGAGAGAGCTCTCTACAAAGAGGTATTGCCCTGTTGGAAGATCCTTTTTTTCACCC
GATATAAGAGCACCACAGCGACTAGGTGATGGCTTGGAATCCTGGTGCGGATTTTACCAG
AGTATAAGACCGACTCAAATGGGACTGTCCTTAAACATTGATATGGCCTCGGCAGCATTC
ATTGAGCCTCTTCCTGTGATAGAGTTTGTCGCTCAGCTTCTAGGCAAAGATGTATTGTCA
AGGCCATTGTCTGATTCTGATCGAGTGAAGATTAAAAAAGGTCTAAGAGGAGTGAAAGTT
GAAGTAACTCACAGAGGGAGTGTACGAAGAAAGTATCGTGTCTCGGGCTTGACATCTCAG
CCTACAAGAGAACTTGTGTTTCCTGTTGATGATAACTCGACAATGAAGTCAGTTGTTGAA
TACTTCCAAGAGATGTATGGCTTCACCATTCAACATGCACATCTACCTTGCCTTCAGGTT
GGAAACCAGAAGAAAGCAAATTATCTACCCATGGAGGCTTGCAAAATTGTGGAGGGGCAA
CGGTATACAAAACGGTTGAATGAGAGGCAAATTACTGCCCTTCTAAAAGTTACATGTCAA
AGACCCCGGGATCGGGAAAATGACATTTTGCAGACTGTTCAGAATAATGCTTATGATCAA
GACCCCTACGCGAAGGAGTTTGGGATCAAAATCAGTGAAAAGCTTGCTTCTGTTGAGGCT
CGAATTCTCCCTGCCCCTTGGCTGAAATATCATGAAACTGGAAAAGAAAAGGATTGCTTG
CCTCAAGTAGGGCAATGGAATATGATGAACAAGAAAATGATTAATGGAATGACTGTAAGC
CGGTGGGCATGTATTAACTTCTCAAGGAGTGTGCAAGAGAGTGTTGCTCGTGGCTTTTGC
AATGAACTTGCCCAAATGTGTCAAGTTTCTGGAATGGAGTTCAATTCAGAGCCTGTGATC
CCTATCTACAATGCCAGGCCTGAGCATGTAGAGAAAGCTTTGAAGCACGTCTATCATGCA
TCCACAAACAGAACCAAAGGAAAAGAGCTAGAGCTTCTCTTAGCTATTCTACCTGACAAC
AATGGGTCCCTATATGGTGATCTGAAGCGAATATGCGAAACTGATCTTGGTTTACTAACT
CAATGCTGTCTCTCGAAACATGTGTTCAAGATCAGTAAGCAGTACTTGGCAAATGTGTCC
CTCAAGATCAATGTAAAGATGGGTGGTAGAAATACTGTCCTTTTAGATGCTATCAGCTGC
AGAATACCATTAGTTAGCGACATACCAACCATTATTTTTGGAGCAGATGTGACTCACCCA
GAGAATGGAGAGGACTCAAGCCCCTCAATAGCTGCTGTGGTGGCTTCTCAGGACTGGCCT
GAAGTAACAAAATATGCTGGACTAGTTTGTGCTCAAGCTCACAGACAGGAACTCATACAG
GACTTGTACAAAACATGGCAAGATCCTGTTCGTGGTACTGTTAGCGGTGGCATGATCAGA
GATCTTCTGATTTCTTTCAGGAAAGCAACTGGACAAAAGCCGCTAAGGATCATATTTTAC
AGGCGAGTATTGGATGGTGTTAGTGAAGGACAATTTTATCAAGTTCTGCTTTATGAGTTG
GATGCGATTCGGAAGGCCTGTGCGTCTTTGGAGCCAAATTATCAGCCACCAGTAACTTTC
ATAGTTGTACAAAAACGTCACCACACTAGATTGTTTGCTAACAACCATAGGGACAGGACT
AGCACAGACAAGAGTGGAAACATACTGCCTGGCACTGTGGTTGATTCTAAAATCTGTCAT

CCAACAGAGTTTGACTTCTATCTCTGTAGCCATGCTGGTATTCAGGGGACAAGTAGGCCA
GCGCACTATCATGTTTTGTGGGATGAGAACAACTTCACAGCTGATGGAATCCAGTCTTTG
ACGAACAATCTCTGCTACACATATGCTAGGTGCACTCGCTCCGTTTCAGTAGTTCCTCCG
GCATACTATGCACATTTAGCTGCATTTCGTGCTCGATTTTACACGGAGCCAGTAATGCAC
GAGACTGGCTCAGCAGGCAGTGGTGCTGGCCATGGTGCTAAGGGAACACGAACAGGAGAA
TCTGGTGTCCGGCCACTGCCGGCCTTGAAAGAGAATGTAAAGAGAGTAATGTTTTATTGT
TAG
>Populus-trichocarpa__POPTR_0010s17100
ATGGAAGAGACAGAAGAGTCCAGTGGTAACAAGAAATGCACCACCAAGACCAGGACTTTC
AGAGGAAGGACCAACACTCACAAGCATCATTATCACCAGTATCAGTATCAGTATCAATAT
CACTATCACTATCAGCACCAACTCTTGCAATACTCAAATCAATATGGTTTCTTTAACCAT
AACCAGTACCCGGGTTACTACCCAGCTCTTCTTCCTTTACCTCCACCAATACCTCTCCAA
CTTGCTCTAACACCGCCTCTCCCTCAAAACCATAGCTTTATATCGAAAACCCAGTTGCAG
AAACCTTTATGCAAGCTAAATAACCCCCCTCCCCCTCCTCCTTCCCCCGCTCCTCCTACC
TCCTCTGATACCAAAGGCCCAGCTGTTACAACTTCACCAGCTTTTGAGGGGCTTCAACAA
CAGAAGAATCGGCCTCTTAAAGGAGATGGTGGAAGGAAAGTCATGAGTGCCACCACACAA
TCACTAGTGGTTGCAAGGAGACCAGATTCTGGTGGTGTAGAAGGATCAGTTATTACTCTC
CTTGCCAACCATTTCCCTGTTCAATTCGACTCCTCGCAACGAATTTTCCATTACAATGTG
GAAATTTCTCCTAATCCTTCCAGGGAGGTTGCCCGGATGATCAAGCAAAAACTGGTGAAG
GAAAATTCAGCTGTGCTCTCTGGTGCTCTTCCAGCCTACGATGGCCGAAAAAGTCTTTAC
AGTCCTGTTGAATTCCAAAAGGATAGGCTTGAGTTCTATGTTAGCCTCCCAATCCCAACT
ACTAAGTCATCACTGCCTTTTGGAGAATTCAATTTCTTGCAAGAGAAGCATCAACAGCTC
AAACTATTTCGAATAAATATCAAGCTTGTTTCGAAGTTGGATGGCAAGGAATTGAGTCGC
TACTTGAGCAAGGAAGGCGATGATTGGATCCCTCTGCCTCAGGATTATCTGCATGCTTTG
GATGTTGTTTTGAGAGAGAGTCCAATGGAGAGATGTCTACCTGTGGGAAGATCACTGTAT
TCTAGTTCAATGGGAGGAACTAAAGAAATTGGAGGTGGAGCGGTTGCATTAAGAGGGTTC
TTTCAAAGTCTAAGGCCAACTCAACAAGGGCTAGCTCTCAATGTGGATTTCTCTGTGACT
GCTTTTCATGAAAGTATTGGAGTAATTCCCTACCTGCAAAAGCGTCTAGAATTTCTTCGG
GACCTTCCTCAAAGGAAAAAAAGAAGTTTGGTTGGTGAAGAAAGGAAAGAAGTGGAGAAG
GCCTTAAAGAACATCAGGATCTTTGTTTGTCACAGGGAAACTGTTCAGAGATACCGGGTG
TTTGGCCTAACTGAGGAAGCTACAGAAAATCTTTGGTTTTCTGACAGGGATGGAAAAAAT
CTGAGGTTGCTGAATTACTTCAAGGATCACTACAACTACGATATACAATTCAGAAACTTA
CCATGCTTGCAGATTAGTAGGAGCAAACCATGTTATCTTCCTATGGAACTCTGTATGATT
TGTGAAGGCCAGAAGTTTCTCGGGAAGCTATCTGATGATCAGACTGCAAGGATACTTAAG
ATGGGCTGCCAAAGACCAAAAGAACGAAAAGCCATCATAGATGGAGTCATGCGAGGATCT
GTTGGCCCAACAAGTGGCAGCCAGGGAAGAGAATTCAAACTCCATATTTCAAGAGAAATG
ACACGATTGAGCGGCAGAATTCTTCAACCTCCAAAACTGAGACTTGGTGATGGTGGCCAT
GTAAGAGATCTCATTCCTTCTCGCCATGATTGCCAGTGGAACCTGCTGGATAGCCATGTC
TTTGAAGGAACTAGAATCCAAAGGTGGGCACTGATAAGTTTTGGGGGCACCCTTGATCAG
AAGTCCAGCATTCCAAAATTCATAAACCAGCTATCTCAAAGGTGTGAACAATTAGGCATC
TTCCTTAACAAGAACACAATGATTAAACCTCAATATGAGCCAACTCAGGTGCTAAATAAC
GTCTCCCTTCTGGAATCAAAACTCAAGAAGATCCACAGCGCCGCATCAAACAATCTCCAG
CTGCTTATATGCGTAATGGAGAAGAAACACAAAGGGTACGCAGATTTGAAGCGAATAGCA
GAGACAAGTGTTGGTGTCGTAACCCAATGCTGCTTGTATTTAAATCTTGGCAAGTTGAGC
TCACAATTTCTGGCTAATTTGGCTCTCAAGATCAATGCCAAAGTTGGTGGGTGCACAGTT
GCTTTGTACAATTCGTTACCCTCTCAGATTCCTCGCCTCCTTCGTTCCAATGAACCTGTG
ATCTTCATGGGAGCTGATGTGACTCATCCTCACCCGCTCGACGATATCAGTCCATCTGTT
GCTGCTGTGGTTGGTAGCATGAACTGGCCTGCAGCAAACAAGTATGTCTCAAGAATGAGG
TCGCAAACACATCGACAAGAAATCATCCAAGACCTTGGTGAAATGGTGAAAGAATTACTA
GACGACTTTTACCAAGAATTGAATGAACTTCCCAAAAGAATTATATTCTTTAGGGACGGG
GTAAGCGAAACCCAATTTTATAAGGTCCTTAAAGAGGAGTTGCAAGCCATTAGAGAAGCT
TGTTCTAGATTCCCTGGTTATAGACCTCCCATTACTTTTGCAGTAGTCCAGAAGAGACAT
CACACAAGGTTGTTTCCGAATGAAACTGATCCATCTTCAACTCAGAACCAGTTTTCTGAC
GAAAATATACCCCCTGGGACTGTCGTGGACACTGTGATTACACATCCAAGGGAATTTGAT
TTCTATCTATGCAGCCATTGGGGAGTGAAAGGTACAAGCAGGCCAACACATTACCATGTC
CTGTGGGATGAGAACCAATTCACTTCTGATGAACTACAGAAGTTGGTTTACAATCTGTGC
TACACATTTGTAAGGTGCACCAAGCCAGTTTCTTTAGTGCCTCCAGCTTACTATGCTCAC
TTGGCTGCATATAGAGGCAGACTTTACCTTGAGCGATCAGAGTGCATGGCTTCCATAAGA
AATGCTTCTACAATCTCAAGAGCAGCCCCACCAAAGGCAGCTCCTCTACCAAAGCTCAGT
GAAAATTTAAAGAAGCTAATGTTCTACTGCTGA
>Populus-trichocarpa__POPTR_0012s03410
ATGGTGAGGAAGAGAAGAACCGAAATTCCTCAAAGTGGGGGTGAGAGTTCTGAGTCGCAG

GAAACTGATACTGGTCGGGGTGCCCAGCCCCCAGCTGAGAGGAGTGGTCCACCTCAACAG
GGAGGAGGTGGAGGAGGATACCAAGGTGGAAGGGGTTGGGGTCCCCAGTCTCAGCAAGGA
GGTCGTGGAGGTGGGTATGGTGGACGAGGCCGGGGAGGGATGCAACAACAGCAATATGGT
GGAGCCCCTGAGTACCAGGGCCGGGGAAGAGGGCAACCTCAGCAAGGAGGTCGAGGATAT
GGTGGTGGTCGTCCTGGAGGTGGCCGCGGAGGGCCATCTTCAGGTGGATTTAGACCACCA
GCACCCGAGCTGCACCAAGCTACCCCAGCTCCTTATCCAGCTGTGGTGACCACTCAGCCC
ACGCCATCTGAGGCAAGTTCTTCGATGCGACCACCAGAGCCATCGCTAGCAACTGTATCG
CAGCAGCTGCAGCAACTATCTGTTGAGCAAGAAGGTTCTTCTAGCCAGGCGATTCAGCCG
CTGCCAGCCTCAAGTAAATCAGTGAGGTTCCCTCTTCGACCAGGAAAAGGTAGCACTGGC
ATTAGGTGTATTGTAAAGGCCAATCACTTCTTTGCTGAGCTACCAGATAAGGATTTGCAC
CAATATGATGTCACCATAACACCAGAGGTCACATCAAGGGGTGTTAATCGAGCTGTAATG
GAACAACTGGTTAAACTGTACCGGGAATCTCATCTTGGAAAGCGTCTTCCTGCTTATGAT
GGACGGAAGAGTCTATATACTGCCGGAGCACTTCCTTTTCAGGCGAAAGATTTCAAGATC
ACCCTCATTGACGATGATGATGGATCAGGCGGGCCAAGGAGAGAGAGAGAATTCAAAGTT
ACAATCAAATTGGCTGCTCGTGCTGACCTGCACCACTTGGGACTTTTTTTGCGGGGACAG
CAAGCTGATGCGCCTCAGGAAGCCCTTCAAGTTCTTGATATAGTTCTGCGTGAATTGCCT
ACTGCTAGGTATTGTCCTGTGGGTCGATCATTTTATTCCCCTGACCTAGGAAGGAGACAA
TCACTTGGTGAGGGCTTGGAGAGTTGGCGTGGTTTCTATCAAAGTATTCGTCCTACTCAG
ATGGGACTATCGCTGAACATTGATATGTCATCCACGGCCTTCATTGAGCCACTGCCAGTC
ATTGATTTTGTGACTCAGTTATTAAATCGTGACGTTTCCTCTAGACCGTTGTCTGATTCT
GATCGTGTAAAGATTAAAAAGGCACTAAGAGGCGTCAAAGTTGAAGTTACACACCGTGGA
AATATGCGCAGAAAGTACCGTATTTCTGGCTTAACATCACAGGCAACACGGGAGCTGACT
TTCCCGGTTGATGAAAGAGGAACCCTGAAATCTGTCGTGGAGTACTTCTACGAAACCTAT
GGTTTTGTAATTCAACACCCTCAATGGCCTTGTCTACAAGTAGGAAATCAACAGAGACCT
AATTATTTGCCTATGGAGGTCTGCAAGATTGTTGAGGGTCAGAGGTACTCCAAAAGATTG
AACGAGAGACAGATAACTGCATTGTTGAAGGTGACCTGCCAGCGTCCTCAGGAAAGGGAG
AAAGATATTATGCAGACGGTTTATCACAATGCATACCACAATGATCCATATGCAAAGGAG
TTTGGTATCAAAATCAGTGATAAGCTTGCTTCAGTTGAAGCTCGCATTCTGCCTCCCCCA
TGGCTTAAGTATCATGACACAGGCAGAGAGAAGGATTGTCTTCCTCAAGTTGGGCAGTGG
AATATGATGAATAAGAAAATGGTCAATGGGGGAAGAGTGAATAATTGGATCTGCGTCAAT
TTTTCACGAAATGTGCAAGACAGTGTGGCTCGAGGATTTTGCTATGAACTTGCACAAATG
TGTCAAATTTCTGGCATGGACTTTGCTCTTGAGCCATTGCTGGCTCCTGTCAGTGGTCGT
CCTGAGCATGTAGAAAGGGTTTTGAAAAATCGATACCATGAAGCAATGACCAAGCTCCGG
CCCCATAGCAAGGAACTTGACTTGCTTATTGTGATTCTCCCTGACAACAATGGTTCTCTT
TATGGTGATTTGAAGCGTATTTGTGAGACAGATCTTGGGCTTGTTTCCCAGTGCTGTTTG
ACAAAGCATGTGTTCAAAATGAGCAAGCAATATCTTGCTAATGTGGCTTTGAAGATAAAT
GTGAAGGTTGGAGGAAGGAATACAGTACTTGTGGATGCAATATCTAGACGTATCCCTCTA
GTCAGCGACCGACCTACTATTATTTTTGGTGCTGATGTTACTCATCCTCATCCTGGGGAG
GACTCAAGCCCATCTATTGCAGCCGTTGTGGCTTCGCAAGATTGGCCAGAGGTTACCAAA
TATGCTGGCCTGGTTTGTGCTCAAGCCCACCGACAAGAGCTTATCCAAGATTTATATAAA
ACATGGCAGGATCCTGTACGAGGGACAGTGTCTGGTGGCATGATCAAGGAGCTTCTCATA
TCCTTCCGTAGGGCAACCGGGCAGAAGCCACAGCGAATTATATTCTACAGAGATGGTGTC
AGTGAAGGACAGTTTTATCAAGTTTTGTTGTACGAGCTTGATGCTATTCGTAAGGCATGT
GCTTCTTTAGAGCCCAATTACCAGCCTCCTGTGACATTTGTTGTGGTTCAGAAGCGTCAT
CACACAAGGCTGTTTGCAAATGATCACCGTGACCGCAATGCTGTTGACAGGAGTGGGAAT
ATATTGCCTGGTACTGTTGTGGACTCGAAGATCTGCCATCCTACTGAATTCGACTTCTAT
TTGTGTAGTCACGCTGGGATTCAGGGCACAAGCCGTCCAGCTCATTACCATGTACTTTGG
GATGAGAACAAGTTTACTGCTGATGGGCTGCAGTCCCTGACAAACAATCTTTGCTACACA
TATGCACGATGCACGAGATCTGTTTCCATTGTGCCACCTGCATACTATGCTCATCTTGCT
GCATTTCGAGCTCGTTTCTACATGGAGCCAGAGACATCAGACAGTGAATCAATTGCAAGT
GGCATGGCTGGTGGTCGTGGAGGTGCTGGTGGGGGTCCTCGTCCAACACGTGGACCTGGT
GCTAATGCTGCTGTGAGGCCTTTACCTGCCTTGAAGGAGAATGTCAAGCGGGTTATGTTC
TACTGCTAG
>Populus-trichocarpa__POPTR_0012s11920
ATGCCAAAAGGTTTGGCTTCTTCCTCTTCGGACAATGCTAATAGGGTTAGTCCGGTTCAA
CGACCAGATACAGGTGGCAAACTGGCGGTCCGAACCCCTAGGCTTCTTGTTAATCACTTT
CCTGTTAAGTTCAGTCCTAAGAGTATCATAAGGCATTACGATGTTGATATCAAACAAGAA
GTGCCTCCCAAGCATGGTCGGCCTGGGAAAATTTCAAAGTCCATTTTAACAATGATCAGG
GATAAGTTATTTACTGATGATCCCTCACGGTTTCCTTTAGGTAAGACAGCCTATGATAGG
GAGAAGAATATTTTCAGTGCAGTTCCCTTGCCCACAGGAACATTTAGAGTGGAGGTCTCT
GAGGCAGAAGATGCGAAACCACGTTCTTACCTGTTTACCATAAAGCTTGTGAATGAACTT
CAGCTGCGCAAGCTGAAGGATTACTTGGATGGGACACTTCGTTCAGTACCTCGTGACATA

TTGCAAGGGATGGATGTGGTGGTGAAAGAGCACCCAGCTAGGACGATGATCTCTGTTGGT
CGTGGCTTCCATTCTGTTAGAGCTCACCAAGATTACCTTGGGTATGGAATCATAGCATCT
AAAGGTTGTCAACATAGCCTCAAGCCGACCTCCCAGGGCCTAGCTTTATGCTTGGATTAT
TCTGTTCTGTCATTTCATGAGCCAGTTTCTGTGATAGATTTCTTGACAAAACATATTTGT
GGGTTTAATTTAAATAATTTTAGAAGATGTAGGGGAGATGTGGAAATTGCATTGAAAGGA
CTCAAAGTTAGAGTGACTCACCGTGTCACCAAACAAAAATATGTTATTGTTGGGTTGACG
AGAGATGACACACGAGATATTACATTTCTCAAGAAGATCCAGATGGCAAGGCTTCACAG
AATGTTAGGCTTGTTGATTATTTCAGGCAAAAATATGGCAGGGATATAGTGCATCAAGAT
ATCCCTTGCCTAGAAATGAAAAGCAACATGAGAAACTATGTACCGATGGAGTACTGTGTC
TTGGTTGAAGGGCAAGTATTTCCAAAAGAGCATCTGCAGAGAGACGAAGCCCAGATGTTG
AAGGACATCTCACTAGCCAAGGCCAAGGATAGACAGAAAACAATATGCAGTATGGTACGA
GATGGAGATGGACCTTTTGGCGGAGAGATTATCCGAAATTTTGGAATGGAAGTCAGTGTG
GATATGACCCCGGTGGTAGGTCGCGTGATCGGGCCACCTGAATTGAAGTTAGGTGCTCCA
AATGGAAGGGTGATGAAAGTACCTGTTGACGAGAAGTGTCAATGGAATCTTGTTGGAAAA
GGAGTGGTGGAAGGGAAACCAATTGAGCGTTGGGCTGTTCTTGACTTCAGCTCTGATGAT
TATCAGTGTCCATTAAATGCTGACCATTTCATCCCAAAGCTTATTGCTCGGTGCCTGAAA
CTGGGGATTCGCATGGAAGAGCCTCTCTTTTATGAACCTACTAGCATGCGGTTGTTCTCT
AACTCTAATGTTGATAGGCTTCGTGAACTCCTTGAAAGAGTCAATGGCCGGGCTCGCAAA
ATCTCTAGAGGCCAGTTGCAGTTTCTTCTCTGTGTCATGTCAAAGAAGGATCCTGGTTAC
AAGTATCTCAAATGGATTTGTGAAACCAAAGTTGGTATTGTGACACAATGCTGTCTGTCT
CGTTCTGCGAATAAAGTAAATGATCAGTACCTTGCCAATATTGGTCTCAAGATTAATGCT
AAGCTTGGAGGAAGCAATGCAGAGCTCAGCGACAGACTTCCATACTTTGGGGATGAAAAC
CACATCATGTTTATTGGGGCTGATGTCAACCATCCTGCTGCTCGAAACACAACAAGTCCA
TCCATTGCTGCTGTTGTTGGTACTACAAATTGGCCAGCTGCAAATCGCTATGCAGCTCGA
GTTCGTCCTCAGGACCATCGTTGTGAGAAGATTCTGAATTTTGGTGATATGTGTTTGGAG
CTTGTTGAATTTTATTCTCGGCTCAATAAAGCAAAACCTGAGAAGATTGTGATATTCCGT
GATGGGGTCAGTGAGGGCCAGTTTGATATGGTTCTCAATGACGAGTTAATGGACATCAAG
AGGGCGTTCAGGTCAATCATGTACACCCCAACCATCACACTCATTGTTGCCCAGAAGCGG
CACCAGACTCGTCTTTTTCTTGAGGATGGGGGGCGAATAGGCAATGTGTCTCCGGGTACA
GTTGTGGACACAAAAAATTGTCCATCCTTTCGAGTATGATTTTTACCTCTGCAGCCACTAC
GGAAGCCTTGGGACAAGCAAGCCCACACATTACCATGTTCTATGGGATGAGCATGGCCTC
AGTTCTGATCAGTTGCAGAAGCTCATATATGACATGTGCTTTACATTTGCTCGTTGCACT
AAACCTGTGTCGTTAGTTCCACCTGTCTACTATGCTGACCTTGTTGCTTATAGAGGAAGG
CTCTACCATGAGGCAGTAATGGAAGGGCAGTCTCCATCTTCTGTATCATCTTCATCATCT
TCAAGGACTTCATCATCTCTGTCAGTGGGAGCTTCGTTGGAAGAGAGGTTCTCCTCATCT
TCCACTGTACATTCAAGTTAA
>Populus-trichocarpa__POPTR_0014s15760
ATGATCAGTAGGCGTGGGGTTGGGACTAGTGGGCGTCACATATCTTTGCTCACCAACCAC
TTTAAAGTTTCTGTCAATGTTCCAGATGCAGTATTTTACCAGTATAATGTTTCCATTACC
TCAGAAGATAATAGAGCTGTTGAAAGCAAGGGAATTGGGAGGAAACTGATCGATAGGCTT
TACCAAACTTATTCCTCAGAATTTGCTGGTAAAAGATTTGCTTATGATGGGGAGAAAAGT
TTGTACACTGTGGGCCCTCTTCCACAGAACAAGTCGGAGTTCACAGTGGTGCTTGAGGAA
TCTTTTGCAAAACATGAAAGTGGGAGCCCTGGTGGTGGTGAGAGCCCTCCTGCAGCTGTT
AAGCGATCAAAGCGCTCTTATCGGTCAAAGACTTTTAAGGTAGAAACAAGCTATGCTGCT
AAAATCCCCTTGAAGTCAATAGCCCTTGCCCTCAAAGGAATTGAGATAGACAATAGCACT
CAGGATGCACTCAGAGTGCTGGATATTATCTTAAGGCAGCAAGCAGCTAACAGGGGGTGC
CTTTTGGTTAGGCAGTCGTTCTTTCATGATGATTCAAGGAACTTCAATGATGTGGGAGGT
GGTGTAACTGGTGTTAAGGGTTTCCATTCTAGCTTCCGTACCACTCAGGGTGGCTTGTCT
CTTAACATGGATGTGTCCACTACAATGATCCTAACTCCTGGGCCAGTAATTGATTTTCTG
ATAGTTAATCAAAATGTCCGGGAACCTCGCTATGTTGATTGGGTGAAGGCCAGAAGGATG
TTGAAAAATTTGAGGGTGAAGACGAAGCATAACAACATGGAGTTTAAAATAATTGGTCTG
AGTGAGAAGCCATGCAATCAACAATATTTTCCTATGAAACTGAAAAACAGGGACGGAGCT
AATGTTGAAGCACAGATTGTTGAAGTTACTGTATATGATTATTTCACTAAACACTGTGGC
ATACAACTTGGTTATTCTGCATACCTACCCTGCCTTGATGTTGGTAAACCAAAACGTCCA
AACTACCTGCCACTGGAGCTTTGTTCACTTATTTCACTTCAACGGTATAAAAAAGCTTTA
TCTTCAATGCAAAGAGCATCTTTGGTTGAAAAATCACGACAAAAGCCTCAGGAAAGAATA
AAAACTGTGACTGAAGCCATGAGAAGCTACTGTTATGATGAGGATCCTGTGCTTTCTTCA
TGTGGTATTTCTATAGAAAAACAAATGACCCAGGTTGATGGCCGCATCCTTGAAACCCCA
AAGTTGAAGGTTGGTAATAGTGAGGATTGTATCCCCCCAAATGGGAGATGGAACTTTAAC
AATAAGACACTTTTAAACCCCACCAGCATCAGTAAGTGGGCCATTGTCAACTTTTCTGCT
CGCTGTGATATTAGTCACGTCTCCCGTGAGCTTATCAACTGTGGAAGGAGAAAGGGCATT
AATATTGAACGCCCACATACATTAATTGAAGAGGACCAACAATCTAGAAGAGGCAGCCCT

CTTGCTAGAGTTGAAAGGATGTTTGAGCTGATCAGAGAGAAGCTTCCAGGGCCTCCTGAA
TTTATTCTTTGTGTATTGGCAGAGAGGAAAAACTCAGATATTTATGGACCGTGGAAGAAG
ACAAGTCTCAGTGATTTTGGCATTGTTACACAGTGCATATCCCCGACGAAGATTAATGAC
CAGTATCTTACAAATGTGCTTCTTAAGATCAATTCTAAGCTAGGAGGAATAAATTCTCTG
TTGGCAATTGAGCACTCCTCACATATTCCATTGATAATGGATACTCCTACAATGATTCTG
GGCATGGATGTCTCTCATGGCTCTCCAGGTCGATCAGACATGCCATCAGTGGCTGCGGTT
GTTGGATCTCGATGTTGGCCACTGATTTCTAGGTACAGAGCATCTGTAAGAACGCAATCT
CCTAAGGTGGAGATGATTGATGCTTTGTACAAGCCTTTAGCAAATGGGAATGATGATGGT
ATAATAAGGGAACTGCTCGTGGATTTCTTTCAAACAAGCAAGGGGCACAAACCAAAACAA
ATTATTGTGTTCAGGGACGGTGTCAGCGAGTCACAATTCAATCAGGTGCTGAACATTGAG
CTGGAGCAAATTATCAAGGCCTATCAACATCTTGGTGAGGTTGACATACCGAAGTTCACA
GTAATTGTGGCTCAGAAGAATCACCACACAAAGCTTTTTCAAGCTGGTGGTGGCACTGAA
AATGTTCCTCCTGGGACAGTTGTTGACACAAAGATTGTTCATCCTAGAAACTATGATTTC
TACATGTGTGCTCATGCAGGCATGATTGGAACTTCAAGGCCAGCACACTATCATGTCTTG
CTCGATGAGATTGGTTTCTCTCCAGATGAATTGCTAAATCTTGTCCACTCTCTTTCATAT
GTGTATCAAAGGAGTACCACTGCTGTTTCGATTGTGGCTCCCATATGTTATGCTCACCTG
GCTGCAGCACAGATAGGGCAGTTTATGAAGTTTGAGGATTTTTCTGAAACCTCTTCCGGA
CAGAGAAGCATGACATCAGTTGGAAGCACCCCTGTTCCAGAGCTCCCCAGGTTACACGAA
AATGTCGAGGGTTCAATGTTCTTCTGCTGA
>Populus-trichocarpa__POPTR_0015s05550
ATGGTGAGGAAGAGGAGAACGGAACTTCCACGAAGTGGGGGTGAGAGTTCTGAGTCCCAG
GAAACTGGTGCTGGTCGGGGTGCTCAGCCCCCAGCTGAGAGGAGCGGTCCTCCTCAACAG
GGAGGAGGTGGAGGAGGATACCAAGGTGGAAGGGGTCCCCAGTCTCAGCAGGTAGGCCGT
GGAGGTGGTTACGGTGGCGGCCGAGGAAGGGGAGGAATGCAACAACAGCATTATGGTGGA
GCTCCGGAATACCAGGGACGGGGGAGAGGGCAACCTCAGCATGGAGAACGAGGTTATGGC
AGTGGTCGTAGTGGAGGTGGCCGAGGAGGACCCCCTTCAGGCGGACCATTTAGAGCACCA
GCACCCGAGCTGCACCAAGCTACTCCAGCTCCTTACCCAGCTGGGATGACCCCTCAGCCC
ATGCCATCTGAGGCACGTTCTTCAATGCCCATGCTGTCTGAGGCAAGTTCTTCAATGCAA
CCACTAGAGCCATCACCAGCGGCTGTGTCACAGCAGATGCAACAACTATCAATCCAGCAA
GAAGGCTCTTCTAGCCAGGCAACTCAGCCACCGCCAGCCTCAAGTAAATCAATGAGGTTC
CCTCTTCGGCCTGGGAAGGGAAGCACTGGCATAAGGTGTATTGTAAAGGCTAATCACTTT
TTTGCTGAGCTACCAGACAAGGATTTGCACCAGTATGATGTTAGCATTACACCGGAGGTT
TCATCAAGGGGTGTCAATCGAGCTGTAATGGCACAACTGGTTAAATTGTACCAGGAATCC
CATCTCGGAAAGCGTCTTCCTGCATATGATGGACGTAAGAGTCTTTATACTGCTGGGGCT
CTTCCTTTTCAAGCAAAGGAATTCAAAATCATCCTCATTGACGAGGATGATGGAACAGGC
GGACAAAGGAGAGAGAGAGGGAATTCAAAGTTGTCATCAAATTTGCTGCTCGTGCTGATCTG
CACCATCTAGGACTCTTTTTGCAGGGAAAGCAAGCTGATGCACCTCAGGAAGCCCTTCAA
GTCCTTGATATAGTGCTGCGTGAATTGCCAACTGCTAGGTACTGCCCGGTGGGTCGATCA
TTTTATTCCCCTGATCTAGGAAGGAGACAGTCTCTTGGTGAGGGCTTGGAGAGTTGGCGT
GGTTTCTATCAAAGTATTCGTCCTACACAGATGGGACTCTCACTCAATATTGATATGTCC
TCAACTGCATTCATTGAGCCACTACCAGTTATTGATTTTGTGACTCAATTATTGAATCGA
GATGTTTCCTCCAGACCGTTGTCTGATTCTGATCGTATAAAGATTAAAAAGGCTCTCAGA
GGTGTCAGAGTTGAAGTTACACACCGTGGAAATATGCGCAGAAAGTATCGTATATCTGGT
TTAACATCACAGGCAACACGGGAGCTGACTTTCCCGGTTGATGAAAGAGGAACATTGAAA
TCTGTTGTGGAGTATTTTTATGAAACCTATGGCTTTGTAATTCAACATACTCAATGGCCT
TGTCTACAAGTGGGAAATCAACAGAGACCTAACTATTTGCCTATGGAGGTTTGTAAGATT
GTTGAGGGTCAGAGGTACTCCAAAAGATTGAATGAAAGACAGATCACTGCGTTGTTGAAG
GTGACTTGCCAACGTCCTCAAGAAAGGGAACGAGATATCATGCAGACGGTTTATCACAAT
GCATATCACAACGATCCTTACGCCAAGGAGTTTGGTATAAGAATTAGCGAGAAGCTTGCT
TCAGTTGAAGCTCGCATTCTGCCTCCTCCATGGCTCAAGTATCATGATACAGGCAGGGAG
AAAGATTGTCTTCCTCAAGTTGGGCAGTGGAACATGATGAATAAGAAAATGGTTAATGGA
GGCAGAGTCAATAATTGGATTTGCATCAATTTTTCAAGGACTGTCCAGGACAGTGTGGCC
CGAGGATTTTGCTATGAGCTTGCACAAATGTGCCACATTTCTGGCATGGACTTTGCTCTT
GAGCCATTGCTTCCTCCTGTTGGTGCTCGTCCTGAGCAAGTAGAAAGGGTTTTGAAAACC
CGATACCATGATGCAATGACAAAACTCCAGCCACATAGCAAGGAACTTGACTTGCTCATT
GTGATTCTCCCGGATAACAATGGTTCTCTTTACGGTGATTTGAAGCGAATTTGTGAGACA
GATCTTGGGCTTGTTTCTCAGTGCTGCTTGACAAAGCATGTATTCAAAATGAGCAAGCAA
TATCTGGCCAATGTGGCTCTGAAGATAAATGTGAAGGTTGGAGGAAGGAATACTGTTCTT
GTTGATGCATTATCAAGACGTATTCCTCTAGTCAGCGACCGACCTACTATTATTTTTGGT
GCTGACGTTACTCATCCTCATCCTGGGGAGGACTCAAGCCCATCCATTGCAGCTGTTGTG
GCTTCTCAAGATTGGCCAGAGGTTACTAAGTATGCTGGCCTGGTTTGTGCTCAAGCCCAT
CGCCAAGAGCTTATCCAAGATTTATATAAAACATGGCAGGATCCTGTACGAGGGACAGTG

TCTGGTGGCATGATCAAGGAACTTCTCATATCTTTCCGGAGAGCCACAGGGCAGAAGCCT
CAGAGAATTATATTCTACAGAGATGGTGTCAGTGAAGGGCAGTTCTATCAAGTTTTGTTG
CACGAACTTGATGCAATTCGTAAGGCATGTGCTTCTTTAGAGCCCAACTACCAGCCTCCA
GTGACATTTGTTGTGGTTCAGAAGCGTCATCACACAAGGCTGTTTGCAAATGATCACCGT
GACCGTAATGCTGTTGACCGGAGCGGGAATATATTGCCTGGTACTGTTGTGGACTCAAAA
ATTTGTCACCCAACAGAGTTCGACTTCTATTTGTGTAGTCATGCTGGGATTCAGGGTACA
AGCCGTCCAGCTCATTACCATGTACTTTGGGATGAGAACAAGTTCACTGCTGATGGGTTG
CAGTCTCTTACAAACAATCTTTGCTACACATATGCAAGATGCACGCGTTCTGTTTCCATT
GTGCCACCTGCATACTATGCACATCTTGCTGCATTCCGAGCTCGTTTCTACATGGAACCG
GAGACATCAGACAGTGGATCGCTTACAAGTGGCATGGCTTCTGGACGAGGAGGTGGAGGA
GCAGGTGGTCGAGCCACTCGCGGACCTGCTGCCAATGCTGCTGTGAGGCCCCTGCCTGCC
TTGAAGGAGAATGTCAAGCGGGTTATGTTCTACTGTTAG
>Populus-trichocarpa__POPTR_0015s15780
ATGAAGTCTGGGCAACTGCAGATAGAGCAACTAGATGCTGAATATCGGTCTATTCCAAGA
TTCAATAATTTTTTATGGCAACAATTGGATACTTTGAATTTGTCATATGTTGGAAACATT
AATGGGATAAGCTCTCTTTTCTTTGCAGGTCCTAATGTGCCAAAAGGATTGGCTTCTGTC
ACTGCTAATAGGATTAGTCCAGTTCTACGACCAGATAAAGGTGGCAAACTGGCTGTCCGA
ACTCCTAGACTTCTTGTAAATCATTTTCTTGTCAAGTTCAATCCTAAGAGCATCATACGG
CATTATGATGTTAATATCAAGCAAGAAGTGCTTCCAAAGCATGGCAGGCCTGGGAAAATT
TCAAAGTCCAATTTAGCAATGATCAGGGATAAGTTATTTGCTGATGATCCTTCTTCGTTT
CCTCGTGCTATGACTGCTTATGACGGGGAGAAGAACATTTTCAGTGCAGTTTCCTTGCCC
ACTGGAACATTTAAAGTGCAGGTCTCTGAGGCAGAAGATGCCAAACCACGTTCTTACCTG
TTTACCATAAAGCTTGTGAATGAACTTGAGCTGCACAAGCTGAAGGATTACTTGGACGGG
AAACTTCGCTCAACACCTCGTGACATATTGCAAGGGATGGATGTCGTGGTGAAGGAGCAC
CCAGCTAGGACAATGATCTCTGTTTCCCGTAGCTTCCATTCTGTTAGAGATCATCAAATT
CACCTTGGGCATGGAATCATAGCATCTAGAGGGTGCCAACATAGCCTCAAACCGACCTCC
CAAGGCATAGCTTTATGCTTGGACTATTCTGTTCTGTCATTTCATGAGCCACTTTCTGTG
ATAGAATTCTTGACAAAACATATTTCTGGGTTTAATTTAAATAATTTTAGAAGCTTTAGG
AGGGCTGTGGAAGGTGTGCTGAAGGGACTGAAAGTTAGAGTGACTCACCGTGTCACCAAA
CAAAAATATGTTATTACAGGGTTGACGAGAGATGACGCTCAATATATTACATTTCCTCAA
GAAGATCCAGATGGCAAGGCTTCGCAGAATGTTAGGCTTGTTGAATATTTCAGGCAAAAA
TATCACAGAGATATAGTGCATCAAGATATCCCTTGCCTAGAGATGAAAAGCAAGATGAAG
AACTATGTACCAATGGAGTTCTGTGTCTTGGTTGAGGGGCAAGTATTTCCAAAAGAGTAT
CTGAAGGAAACTGAAGCCAAGATGTTGAAGAAATTCTCACTAGCAAATCCAAAGGATAGA
CAGAAAACAATATGCAGGATGGTGCAGGATGGAGATGGACCTTGTAGTGGAGAGATTATC
CGAAATTTTGGTATCGAAGTCAGCAAGAATATGACCTCATTGATTGGTCGTGCCCTCGAT
GGCAGGGTGATTAAAATACCAGTTGACAAGGAGAAGTGCCAATGGAATCTTGTTGGGAAA
GGAGTGCTGATTGCTCGATGCCAGAGTCTGGGGATTCGCATGGAGGAGCCTCTCTTTTAT
CAACCTACTAGCATGCGGAAGTTCTCTAATGTTGATGTGCTTCGTGAACAACTTGAAACA
GTCAATGAACGGGCTCACAAAAGCTGCGGAGGCCAGTTGCAATTTCTTCTCTGTGTCATG
TCAAGGAAGGATCCTGGTTACAAGTATCTCAAATGGATTTCTGAAACCAAAGTTGGTATT
GTGACACAATGTTGTTTGTCCACTCCTGCAAATGAAGGAAAGGATCAGTACCTTGCTAAT
CTTGGTCTCAAGATTAATGCTAAGCTTGGAGGAAGCAATGCAGAGCTCAGTGGCAGACTC
CCGTACTTTGGGAATGAAAACCGTGTCATGTTTATTGGGGCTGATGTCAATCATCCTGGT
GCTCAAAACAAAACTAGTCCATCCATTGCAGCTGTTGTTGGTACTATAAATTGGCCAGCT
GCAAACCGCTATGCAGCTCGAGTTCGTCCTCAGTATCATCGTAAAGAGCAGATTCTGAAT
TTTGGTGATATGTGCTTGGAGCTTGTTGAATGTTATTCCCGGCTCAATAAAGCGAAACCT
GAGAAGATTGTGATTTTTCGTGATGGGGTCAGTGAGGGCCAGTTTGATATGGTTCTTAAT
GAAGAGTTAACGGACATAATGAAGGCATTCAAGTCAATCAATTACACCCCAACCATAACA
CTCATTGTTGCCCAGAAACGGCACCAGACTCGTCTTTTTCCTGGGGACGAGGGATCTACT
GGCAATGTGTCTCCAGGCACAGTTGTGGACACAACAATTGTTCATCCTTTTGAGTATGAC
TTTTACCTCTGTAGCCACTACGGAAGTCTTGGGACAAGCAAGCCCACGCATTATTATGTT
CTATGGGATGAGCATGGCCTCAGTTCGGACGACTTGCAGAGGCTCATATACAACTTGTGC
TTCACATTTGCTCGTTGCACAAAACCTGTGTCATTAGTCCCACCAGTCTACTATGCTGAC
CTTGTTGCTTATAGAGGAAGGCTTTACCATGAGGCAGTAATGGAAGGGCAGTCTCCATCT
TCATCATCTTCAAGGACTTCATCATCACTTTCAACTGCAGCTTCGCTGGAAGAGAG-
>Populus-trichocarpa__POPTR_0016s02480
ATGGAGTCTAATGAAGAGCCAGAGGCCCTTGCTCCTCCACCTGATGCCCTCCCTCCTCCT
CCACCTGAGATACCACCAAATGTAGTTCCTGTTCAATTGACAACAGGCACTTTTCCTGAG
GAAACAAAAAAGACATCAAAATTAAAACGTTCCCCGATTACCAGGCGCGGAGTTGGGTCT
AGAGGGCAAAAAATACAACTAGTCACAAATCATTTCAAAGTTTCTATCTCTAATACTGGT
GGCCACTTTTTTCACTACAGTGTTTCCTTATATTATGAGGACGGTCGCCCTGTCGATGCA

AAGGGCATTGGGAGAAGATTAATTGACAAAGTTCATGAGACTTATGGGTCAGATCTTGCC
GGGAAGGACTTTGCATACGATGGAGAGAAGAGCTTATTCACAATTGGTGCTCTGCCACGA
AACAAAATGGAATTCACTGTTTTGCTTGATAGTTTCTCATCAAATAGGAATTCTGGAAAT
GGAAGCCCTGTTGGCAATGGAAGTCCTAATGAGACCGATAAAAAGAGGATGAGGCGGGCA
TTCCAGTCCAAAACATTTAAAGTGGAGATGAGTTTTGCTGCCAAAATCCCTATGCAAGCT
ATTGCAGCTGCTTTGCGTGGTCAAGAATCGGAAAACTCTCAGGAAGCCTTAAGAGTCTTG
GACATCATTTTAAGACAGCATGCAGCTAAACAGGGTTGCCTTCTTGTTCGCCAGTCATTC
TTTCACAATAATCCAAAGAACTATGTTGATCTGGGAGGAGGAGTCCTTGGATGCCGAGGA
TTTCATTCGAGCTTTCGAGCCTTGCAGGGTGGATTATCCCTAAATATGGATGGTTCGACT
ACAACGATAATACAGCCTGGGCCACTTATTGACTTTCTCATAGCCAACCAGAATGTGTCA
AACCCTTTTCAGATCGACTGGGCAAAGGCAAAGCGGACAATGAAAAATTTGAGGATAAAG
GTGTCACCTACCAATCAAGAGTACAGAATCACTGGCCTAAGTGAAAATAGTTGCAAAGAG
CAAATGTTTTCGCTGAAATCAAGAGCAGCTGATGGAAATGATGTTGAAAGTTTTGACATT
ACAGTTTATGATTATTTTGTTAATCATCGCAGCATAGATTTACGTTACTCTGGAGATTTG
CCATGCATCAATGTTGGCAAGCCTAAAAGGCCTACTTACATTCCCGTCGAGCTTTGTTCA
CTGCTTTCTTTGCAACGCTACACAAAGGCACTGACTGTCCATCAGAGATCGCAGTTGGTA
GAAAAATCAAGACAAAAACCCCAAGAAAAGATTAGGATCTTAGCTGATGTAATGAAAAGC
AACAACTATGCTGCAGAACCAATGCTGCGTTCTTGTGGCATCACCATCAGCAGCCAGTTT
ACTCAAGTTCAAGGCCGTGTGCTTCCTGCTCCAAAGTTAAAAGCAGGAAATGGTGAGGAT
GTTATTCCAAGAAATGGGCGGTGGAATTTTAATAATAAGAAATTTTTTGAACCTTCTAAA
ATTGAAAACTGGGCAGTGGTGAACTTTTCCGCCCGTTGTGATGTGCGTGGTCTAGTAAGA
GATTTGATAAAATTTGGAGAAATGAAAGGGATTCTCATAAGTGACCCGATGGATGTTCTT
GAAGAGAATGCTCAGTTTCGACGGGCACCACCTCCTGTTCGAGTGGATAAGATGTTTGAA
CAGATACAGACAGCTTTTCCAGATGCTCCTCCTCGCTTTCTTGTGTGTGTCTTCTTCCTGAT
AGGAAGAACTCTGACATATATGGTCCTTGGAAACGAAAGAATCTTGCAGAATATGGAATT
TTCAACCAATGCCTGGCACCCACTAGAGTTAATGATCAGTATATATTGAATGTTCTCCTG
AAGATAAATGCTAAGCTCGGTGGTTTGAATTCTTTGTTGGCTATGGAGCAATCACGAAAC
ATCCCTTTTGTTTCAAAAGTTCCTACAATAATATTTGGGATGGATGTATCACATGGCTCG
CCTGGCCAGTCTGACATCCCATCCATTGCTGCGGTTGTCAGTTCTAGAAACTGGCCTCTA
CTTTCTCGTTATAGAGCTTCTGTGCGTAGTCAGTCACCAAAAGTTGAGATGGTAGATTCT
CTTTTTAAACTAACAGCAGATAAGAAAGATGATTGTGGGATTGTTAGGGAATTGTTGTTA
GACTACTATAAGAGTTCTGGCCAAACAAAGCCAGCTCAGATAATCATATTCAGGGATGGA
GTTAGTGAGTCGCAGTTTAATCAAGTCCTCAACATCGAGCTGGATCAAATCATTGAGGCC
TGCAAGTTCCTTGATGAAAGCTGGTCACCCAAGTTCACTGTAATTGTTGCACAGAAAAAT
CACCACACTAAATTCTTCCAAGATGGATCTCCAGACAATGTTCCTCCTGGAACTGTTATT
GATAATGCTGTTTGTCACCCACAAACCTATGATTTCTACATGTGTGCCCATGCAGGGATG
ATAGGAACAACTAGGCCAACACATTATCATGTTCTTTTAGATGAGATTGGCTTTTCAGCT
GATGATCTACAGGAGTTGATTCACTCTTTGTCTTATGTGTACCAAAGAAGCACAACAGCA
ATATCCCTAGTTGCTCCAGTCCGGTACGCACACTTGGCAGCAACTCAGATTTCACAATTC
TTGAAGTTTGATGACATGTCAGAGACATCTTCGAGCCATGGAGGTCTGACTTCTGCTGGG
CAAGCACCTGTGCCTGAGCTTCCTGAGCTACACCACAATGTCCGCAGCTCTATGTTTTTC
TGCTGA
>Ricinus_communis_29589.t000074
ATGTCTCACCGTGGTGGTGGCCGACGTCAGGAATCTCAGCGCGACCGACAATCCTCTGCT
ACATCCCCGTCGTTCAATCGCGGAGGCGGTGGTGGACGTGGCGGTAGAGGCCGAGGTGGA
GCTGGCTCCTTTTACGCTCAACCTGCTCCTCCTCCAGCAGGATCGGACTTTCCTTCATTA
TCTCGTCCCCCGACTACTTCATCCAGAGCGGCTGCTGCTCCTCAAGCAGCGCCTCCTTCT
TCATCTTCGAGAGCTCCACCGGCTCCGGCTCCGGCTCCTGCTCCTCCTCCTGCAGCGGCG
GCCTCAACTTCCACTCAGATAGAGAAGCTGACGCATGGGGTTGAGCAGTTAACGACGACG
GCCGGAGCTCCTACACCGTCGTCATCAAAGGAGATTAGATTTCCTAATAGGCCAGGGTAC
GGTAGTATAGGGATGAAGTGTGTGGTTAAAGCCAACCATTTTTTGGTTGACGTCGCTGAT
AGAGATCTCCGTCAATACGATGTGAGCATAACTCCTGAACTAACCTCAAAGAAGATAAAC
AGAGATGTTATATCTCAGCTCATTCGCATGTTTCGTCAGTCACATTTGGGTAACCGAAGA
GCAGCTTATGATGGTAGGAAAAGCTTATACACTGCAGGGCCTTTGCCTTTTGAGTCTAAA
GAATTTGTTGTCAAGCTTGTTGAATCAAATAAAAATGCCGGTTCTTCTGTTTCATCCAAG
AAGGAACGTGAATTTAAGGTGGCAATTAAATTTGCATCTAAGCCTGATATTCATCATTTG
AAACAATTCTTAATCGGTAGACAAATGGATTGCCCACAAGAAACGATTCAAGTTCTTGAC
ATTGTTCTTCGGGAAACACCTTCAGAGAAGTATACCCCTGTTGGGAGGTCATTTTTTTCA
CCTGATTTGGGCCAAAAGGGAGAGCTTGGCGATGGTATAGAGTATTGGAGAGGATACTAT
CAGAGTCTTCGCCCAACCCAGATGGGGCTTTCTCTTAACATAGATGTATCAGCTCGATCA
TTCTATGAGCCCATTATTGTGACCGACTTTGTTTCAAAATACTTGAAGTTGAGAGACATG
TCAAGGCCTCTCTCTGATCAAGATCGAATTAAGGTGAAAAAGGCTCTGAAATCAGTGAAA

GTGCAAATACTTCACAGGGAGTATGCTAAAAGCTATAAAGTTACTGGCATATCCAATAAA
CCCCTGAATCAGATATTCTTCAAATTGGATGACAAGAGCACAGATATCTCAGTTGTACAG
TATTTCCGTGAAAAATACAATATTGGGCTAAAATATACGTCCTTGCCTGCCCTTCAAGCT
GGGAGTGATGCAAAACCAATTTACTTGCCTATGGAGCTTTGTAAGATTGTTGATGGTCAG
AGGTATTCAAAGAAACTTAATGAAAGACAAGTAACTGCACTGCTAAGAGCAACCTGTCAA
CGCCCTCATGAACGAGAGGAAAGCATTAAGCAGATGGTCAAGCGGAATAGTTACAACCAA
GATGTGCTTGTAAGGGATGAGTTTGGAATCCAAGTGAAAGAAGAACTTACATTTGTTGAT
GCCCGAGTTTTGCCTGCTCCCATGCTTAATTATCATGAGACTGGAAGGGAATCAAGGGTT
GATCCCCGCTGTGGGCAATGGAATATGATTAACAAGAAAATGGTGAATGGTGGGAGTGTG
AATTTTTGGACATGTGTGAACTTCTCTTTGAACATCAATCGAGATTTGCCTGCTGAGTTC
TGTAGGCAATTGATTCAGATGTGTGTCAGCAAAGGAATGGCATTCAATCCAAACCCTATT
ATTCCAATAAGCTCAGCTCATCCAGGTCAAATTGGGAAGACTCTTAATGATATTAAGAGG
CAATGTGAGGCAAAACTTGTGAAACAGCTGCAGTTGCTGATAATTATTCTACCTGATATC
AGTGGATCTTATGGAATAATTAAGAGAGTCTGTGAAACAGAATTGGGAATTGTTTCTCAG
TGTTGCCAACCACGACAAGCAGCAAAACTGAGTAAACAGTATTTTGAGAATGTTGCTCTC
AAAATCAATGTTAAGGTTGGGGGAAGAAACACTGTGTTGAATGATGCCGTGCAAAGGAGG
ATCCCACTTGTGACTGATTGCCCTACAATTATTTTGGTGCTGATGTAACTCATCCACCA
CCAGGGGAGGACTCTAGTCCGTCGATAGCAGCAGTAGTGGCTTCAATGGACTGGCCTGAG
GTAACCAAGTATAGAGGCATTGTCTCTGCACAGGCGCATCGTGAAGAAATCATCCAGGAT
TTGTATAAATCCTTTCAGGATCCTCAGGGAATATTAAAGCATTCAGGAATGATCAGAGAA
TTGTTTGTTGCATTTAGAAGAGAAACTGGGATGAAACCTAAAAGGATTATATTCTACAGA
GATGGTGTTAGTGAGGGGCAGTTTAGTCAAGTTTTGCTGTATGAGATGGATGCCATAAGA
AAGGCTTGTGCCTCACTGGAGGAGGGATATCTTCCACCGGTTACCTTCGTTGTGGTGCAA
AAAAGGCACCATACACGACTTTTTCCCGTTGATCGTGGTCAGACAGACAGGAGTGGCAAT
ATTCTGCCAGGCACTGTTATTGACACTAAGATATGCCACCAGAGGGAGTTCGACTTCTAC
CTGAACAGCCATGCTGGAATTCAGGGAACCAGCAGACCAACTCATTACCATGTGCTGTAT
GATGAAAATCATTTCACTGCTGATAATCTGCAAGTGCTGACTAATAATCTGTGTTACACG
TTTGCCAGGTGTACACGCTCTGTGTCCATAGTCCCTCCTGCCTATTATGCCCATTTAGCA
GCTTTCCGTGCTCGATATTACATTGAGGGTGAAATGTCAGATGGTGGATCTACCAGTGGA
AAGAGCACAACAGGGCGGAGTAAGGAGGTCCAGCCTCTTCCTGTCATCAAAGATAATGTG
AAAGATGTGATGTTTTATTGCTGA
>Ricinus_communis_29677.t000007
ATGGTGAGGAAGAGGAGAACTGAGGCTCCTGCAAGTGGGGGTGAGAGCTCTGAGCCCCAT
GAAGCGGCAAGTGGAGGATCTCAACGGCCATATGAGAGGAATGCACCACCTCAACAGGGA
CCAGGTGGACCATACCAAGGTGGAAGGAGTTGGGGTCCGCAGTCTCAGCAAGGAGGTCGC
GGCGGTGGTGGACGTGGACGTAGCGGTGGGATGTCTCAACAACAACAGTATGGTGGTGGC
CCTGAATATCAAGGCAGGGGAAGAGGTCCACCTCAGCAAGGTGGTCGAGGAGGCTATGGC
GGTGGTCGCTCCAGTAGTAACCGTGGAGGTCCACCTTCTGTTGGTCCATCCAGACCTCCA
GTTCCCGAGCTGCACCAAGCAACCTTGGCTCCTTACCAAGCTGGGGTGTCCCCTCAGCTG
ATGCCATCTGAGGGGAGCTCTTCTTCTGGTCCACCAGAGCCATCTCCGGTGGTAGTAGCC
CAACAGATGCAGGAGCTCTCTATCCAGCAAGAAGTTTCTTCAAGCCAACCAATCCAGGCA
CCACCACCCTCCAGCAAATCAATGAGGTTCCCACTTAGGCCTGGGAAGGGTAGCACTGGA
ATAAGGTGTATAGTGAAGGCTAACCACTTCTTTGCCGAATTACCCGACAAGGATTTGCAC
CAATATGATGTTACAATAACACCAGAGGTCACATCACGGGGTGTCAACCGTGCTGTAATG
GAACAACTGGTTAAGTTGTACAGAGAGTCTCATCTTGGAAAGCGTCTTCCTGCATATGAT
GGCCGAAAGAGTCTATACACTGCTGGGCCACTTCCCTTTATATCAAAGGAATTCAAAATC
ACTCTGATTGATGAAGATGATGGATCAGGCGGGCAAAGGAGAGAGAGGGAATTTAGGGTT
GTGATCAAATTAGCTGCTCGTGCGGACCTGCATCATTTAGGGCTCTTTTTGCAGGGAAGG
CAAGCAGATGCACCACAGGAAGCCCTTCAAGTTCTTGACATTGTTCTGCGAGAGTTACCT
ACTACTAGGTATTGCCCTGTCGGTCGATCATTTTATTCTCCTGATCTAGGAAGAAGGCAA
CCCCTTGGTGAGGGTTTGGAGAGCTGGCGTGGTTTTTACCAAAGCATTCGACCTACACAG
ATGGGATTGTCTTTAAATATTGATATGTCTTCCACTGCTTTCATTGAACCGCTACCAGTC
ATTGACTTTGTGAATCAACTGTTGAACCGAGATGTTTCTTCTCGACCATTGTCTGATGCT
GACCGCGTGAAGATTAAAAAGGCTCTTAGAGGAGTCAAGGTGGAAGTTACACACCGTGGA
AATATGCGCAGAAAGTACCGCATATCTGGTTTAACATCACAGGCAACACGAGAGTTGACT
TTTCCAGTTGACGAAAGAGGAACAATGAAATCTGTCGTGGAGTACTTCTACGAGACATAT
GGTTTTGTAATTCAACACACGCAATGGCCCTGTCTACAAGTGGGGAATCAGCAGAGGCCG
AATTATCTGCCTATGGAGGTTTGCAAGGTTGTTGAGGGCCAGAGGTACTCCAAGAGACTG
AACGAGAGGCAGATAACTGCCTTGCTGAAGGTGACCTGTCAGCGTCCTCAAGAAAGGGAG
CGTGATATTATGCAGACTGTTCATCATAATGCGTACGGCAATGATCCTTATGCAAAGGAG
TTTGGAATCAAAATTAGTGAGAAGCTTGCTTCAGTTGAAGCTCGCATTTTGCCTGCCCCA
TGGCTTAAATATCATGATACTGGTAGAGAGAAGGATTGCCTGCCACAAGTTGGACAATGG

AACATGATGAATAAGAAAATGGTCAACGGGGGAACAGTGAACAACTGGATCTGCATAAAT
TTTTCTAGAAATGTTCAAGACAGTGTGGCCCGTGGATTTTGCTATGAACTTGCACAAATG
TGTTACATATCTGGAATGGCATTTAATCCTGAACCTGTACTTCCTCCGGTTAGTGCTCGT
CCAGAGCAGGTAGAGAAGGTATTGAAAACTCGTTACCATGATGCCATGACAAAACTCCAA
CAGGGCAAGGAGCTTGATCTGCTTATCGTAATTCTCCCTGACAATAATGGCTCTCTTTAT
GGTGAACTGAAGCGCATTTGTGAGACGGATCTTGGTCTTGTTTCTCAGTGCTGTTTAACA
AAGCATGTGTTCAGGATGAACAAACAATATTTGGCAAATGTGGCATTAAAAATTAATGTT
AAGGTTGGGGGAAGAAACACTGTGCTTGTTGATGCATTATCAAGGCGCATTCCTTTGGTT
AGTGATCGGCCCACTATTATTTTTGGTGCTGATGTCACCCATCCACATCCGGGGGAAGAC
TCGAGCCCATCTATTGCAGCTGTTGTGGCTTCTCAAGATTGGCCAGAAGTAACGAAATAT
GCTGGCTTGGTTTGTGCACAAGCCCATCGGCAAGAGCTTATTCAAGATCTCTTTAAAGAA
TGGCAAGACCCTGTGAGAGGAAGAGTTACTGGTGGCATGATTAAGGAACTCCTTATATCT
TTCCGCAGAGCAACTGGGCAGAAACCTCAGCGTATTATATTTTACAGGGATGGTGTTAGT
GAAGGACAATTCTACCAAGTGCTGCTGTATGAACTTGATGCTATCCGGAAGGCGTGTGCT
TCTTTAGAACCAAACTATCAGCCTCCAGTGACATTTGTTGTGGTTCAGAAGCGTCACCAC
ACTAGGTTGTTTGCAAACAACCATAACGACCGTAATGCTGTTGACAAGAGCGGAAATATA
CTGCCTGGTACTGTCGTGGACTCTAAGATCTGCCATCCAACTGAATTTGACTTCTATCTG
TGTAGCCATGCTGGGATTCAGGGTACCAGCCGTCCAGCTCATTACCATGTATTATGGGAT
GAGAACAAGTTTACTGCCGACGGGTTACAGTCCCTTACAAACAATCTTTGCTACACATAT
GCGAGATGCACGCGCTCTGTTTCCATCGTACCTCCTGCATACTATGCTCATCTTGCAGCA
TTTCGAGCTCGGTTCTACATGGAACCAGAGACGTCAGACAGTGGATCAATGACAAGTGGA
CCTGTCGGTGGACGTGGAGGCATGGGTGGAGGTGCAGGTGCCAGGAGCACACGGGGACCT
GCAGCTAGTGCTGCTGTGCGACCCTTGCCTGCCTTGAAGGAGAATGTTAAGCGGGTCATG
TTTTATTGCTAG
>Ricinus_communis_29684.t000014
ATGGATTCATTTGAACCTGATGGAAATGGGTTGCGTGAAGGAAATGGGATCCACGAGGGA
AATGGATCACAGGAAGGCTTACCGCCTCCCCCACCTGTTGTTCCACCAGATGTGGTTCCA
ATGCGAGCAGAACCTGAACCAGTCAAGAAAAAGGTTGTCAGGGTTCCAATAGCTAGGCGT
GGCCTTGGATCTAAAGGCCAGAAGATATCTCTACTAACTAATCACTTTAAAGTTAATGTT
AACAAAGTTGATGACTATTTCTTCCATTACTGTGTGTCCCTTTCCTATGAGGATGGCCGC
CCGGTTGATGGTAAGGGTGTTGGAAGAAAGGTCATTGATCGAGTGCATGAAACTTATGAC
TCTGAAATGGGTGGAAAGAAATTTGCTTACGATGGAGAGAAAAGCTTATTCACTGTTGGT
GCTCTTCCACGCAACAAACTTGAGTTCACTGTCGTGCTTGAGGATGTCACCTCTAACAGA
AATAACGGAAATGCAAGCCCTGATGGGCATGGTAGTCCCAATGAGGGTGACCGAAAGAGA
ATGCGGCGCCCATATCAGTCCAAAACATTCAAAGTGGAGATCAGTTTTGCTGCAAAAATT
CCCATGCAGGCCATTGCTAATGCACTGCGTGGACAGGAATCTGAGAATTCTCAAGAAGCC
ATAAGGGTCCTGGATATTATATTGCGCCAGCATGCGGCTAAACAGGGCTGCCTTCTTGTA
CGCCAGAATTTCTTTCACAATGATCCAAAGAATTTTGCAGATGTCGGGGGTGGTGTTCTA
GGTTGTCGAGGATTTCATTCTAGTTTTAGAACCACTCAGGGAGGGCTGTCTCTGAATATT
GATGTATCAACTACCATGATAATTCAGCCTGGTCCTGTTGTGGATTTTTTGATTGCAAAC
CAAAATGTGAGAGATCCCTTTCAGCTTGACTGGGCTAAGGCCAAACGAACATTGAAAAAC
TTGAGGATTAAGGCAAGTCCCTCCAACCAAGAATACAAGATAACTGGTTTGAGTGAGATG
CCCTGCAAAGAGCAAACTTTTCAATTAAACCAAAAGGGAAGGGATGATAATGACCCGCTT
GAATTAACAGTTTATGACTACTTTGTTAATCATCGCCGTATAGAATTGCGCTATTCTGGT
GATTTACCATGCATCAATGTAGGGAAACCAAAGCGGCCTACTTTTATTCCTATTGAGCTT
TGTTCTTTGGTGTCCTTGCAACGTTACACAAAAGCACTCAACACCCTTCAAAGGGCTTCT
TTGGTGGAGAAATCAAGACAAAAGCCACAAGAGAGGATGAGTACTTTATCAAATGCTTTG
AAGAGCAGCAATTATGATGCTGAACCTATGCTACGCTCATGTGGGGTCTCAATCAGTACT
AGTTTTGTTCAAGTTGATGGCCGTCAATTGCAGGCGCCAAAGCTAAAAGTAGGCAATGGG
GAGGATTTCTTTCCACGAAATGGGCGCTGGAATTTTAATAACAAGAAACTTGTGGACCCA
AGTAAAATAGAGCGATGGGCTGTTGTGAACTTCTCAGCACGTTGTGATATACGTAACCTT
GTACGTGATTTGACAAAATGTGCAGAGATGAAAGGAATTCCTATAGAGCCTCCTTTTGAT
GTATTTGAGGAGAACCCGCAGTTCAGACGTGCCCCGCCTACAGTTCGAGTTGAGAAAATG
TTTGATTCGATACAGTCTAAGCTTCCTGGTGCTCCGAAGTTCCTTTTATGTCTACTTCCT
GAGAGAAAAAACTCTGACCTATATGGTCCTTGGAAAAAGAAAAATCTTTCTGATTTTGGA
ATTGTCACCCAGTGCATTGCTCCCCAGAGGGTCAATGACCAGTATTTGACAAATGTTCTT
CTGAAGATAAATGCAAAGCTTGGTGGTTTAAACTCTATGCTGGCAGTTGAACATTCTCCC
TCTATACCACTGGTTTCTAAGGTTCCTACCATTATCATTGGTATGGATGTCTCACATGGC
TCTCCTGGGCATTCTGATGTCCCATCAATTGCTGCGGTAGTCAGTTCCAGGCAGTGGCCA
TTAATTTCTCGATATAGGGCATGTGTCCGCACTCAGTCTCCGAAGGTTGAAATGATAGAC
TCTCTGTACAAGCCTGTGTCTGACACTGAGGACGAAGGCATGATGAGGGAGCTTTTGTTG
GACTTCTATTCTAGTTCAGGAAAAAGGAAACCTGAACAGATCATCATATTCAGGGATGGC

GTCAGTGAATCACAATTCAATCAAGTGTTGAACATTGAATTGAATCAGATAATCGAGGCA
TGTAAGCATCTTGATGAAAAGTGGAATCCCAAGTTTGTGGTGATTATTGCACAAAAAAAC
CACCACACTAAGTTCTTCCAGCCTGGACTTCCTGATAATGTTCCACCTGGAACTGTCATT
GATAATAAAGTCTGTCATCCACGGAACAATGATTTCTATCTCTGTGCACATGCTGGGATG
ATTGGAACTACGAGGCCCACCCATTATCATGTTTTATTAGATGAGGTTGGCTTTTCAGCG
GATGAACTTCAGGAACTTGTGCATTCATTGTCCTACGTGTACCAAAGAAGCACAACTGCC
ATTTCTGTAGTTGCACCGGTGTGTTATGCTCACCTGGCTGCCACTCAGATGGGACAATTC
ATGAAGTTTGAGGATGCATCTGAGACATCCTCAAGTCATGGTGGTGTAACTTCTGCTGGA
GCTGTTCCTGTTCCTCAGATGCCCAAATTGTCAGATAAAGTCTCCAGTTCAATGTTCTTT
TGTTGA
>Ricinus_communis_29807.t000009
ATGCCTCTCATGCAAATGAAGGACTTTGAAGAAAGCTATATGGTGGTATCTATGCAATCT
CTTCAAACTTGCAAAAATCTTGAGATTCCCTTTAGTAATGGAGATGATAACTCGGAAACT
GCAAAAGAAAGCTTAGGTAGGAAAAGAGCTAATGGAAGGAGATCAAGAGGAGGCAAAGGA
CTCAAAGTTGAGTCCAAGAAAATATTATTTCAAGATTATAAGCTTGATTCAAGTGAAGAG
TCTTCTCCTTCTTCATGCAAGAGCCTTATGTTTCATCGTAGGCCTGGACATGGACAATTG
GGTACCAAGTGCATAGTTAAAGCTAACCATTTTCTAGCTCAGATGCCTGATTCAGACTTG
AGCCACTATAGTGTTGAAATAAAACCAGAAGTAACATCTCGCAAATTAAGCAAAGCCATC
ATGACACAGTTGGTTAAAATGCATAGGGAAACTGATTTAGGAACGAGACTGCCTGTTTAT
GATGGAGGCAGAAACCTTTACACAGCAAGATCACTTCCTTTTACCTCAAAAGACTTTACT
ATAACCTTGGTTCATGAAGATGAGGCAACAGGCAATATCAAGAAGAGAGATTTTGAAGTG
ACAATAAAATTTGAAGCACTTGCTGGTATGCTGCAATTACGTGAGCTTCTTTCTGGAAAA
CCAGTTGATACCCCTCAAGAAGCAATTACTGTTATTGACATTGTGCTGAGAGAACTTGCA
GCTCAAAGATATGTATCAATTGGAAGGTCATTCTATTCTCCTGATATCAAGAAACCGCAG
CAGCTCGAAGGAGGATTAGAATCATGGAGAGGTTTCTACCAAAGTATAAGACCGACTCAG
ATGGGACTGTCACTAAATATTGACATGTCAGCAACGGCTTTCATTGAACCTCTCCTTGTC
ATCGAATTTGTTGCTCAAATTTTGAATAAAGATGTGTATTCGAGGCCATTGTCAGACGCA
GATCGTGTCAAGGTAAAGAAAGCACTCAGGGGTGTAAAAGTTGAAGTCACTCACAGAAGA
AATGTACGGAGAAAGTATAGAATTTCAGGATTGACGACACAGCCTACAAGAGAGCTAATC
TTCCCGCTTGATGAGCATATGAACATGAAATCAGTTGTTGAGTACTTTCAAGAAATGTAT
GACTATACCATTCAATATCCCCATCTACCTTGCCTGCAAGTTGGAAACCAGAGGAAGGTT
AACTATTTGCCGATGGAGGCTTGCAAGATAGTCAGGGGGCAAAGATATACCAAAGGCCTC
AATGAGAAGCAAATAACTTCTTTGCTAAAAGTTTCATGTCAAAGGCCGCGTGATCAAGAA
ATGGACATTTTGCAGACAATTCACCAAAATGGTTATGAACATGATCCCTATGCAAAGGAG
TTCGGCATCAGCATAGACAGCAAGCTTGCATCGATTGATGCTCGTGTTTTGCCTGCTCCG
TGGCTGAAGTACAGCGATACTGGCAAAGTGAAAGAATATCTGCCTCAAGTTGGTCAGTGG
AATATGATGAATAAGAAAGTGATAAATGGAAGCATTGTAAGATATTGGGCTTGCATCAAC
TTCTCACGAAGCGTTCAAGAAACCACTGCTCGCAGTTTTTGCCAACAGCTGGTCCAGATG
TGCCGAATTTCTGGCATGGATTTTAACGGTGAACCTGTAATTCCAATATATGCAGCAAGA
CCTGATCAGGTAAAGAAGGCCTTGAAATATGTATACCACGCTGCTGCAAAGAAACTTGAA
GGAAAAGAATTAGAGTTACTCATTGCCATTCTTCCAGACAGCAATGGTTCATTGTATGGT
GATTTAAAACGCATTTGTGAAACTGATCTCGGGTTAATTTCTCAATGCTGCCTTACCAAG
CATGTCTTCAAGATTAACAGACAGTATTTGGCAAATGTGTCGTTAAAAATCAATGTTAAG
ATGGGAGGAAGAAATACTGTGCTTTTAGATGCTATAAGTTGGAGGATTCCCTTGGTTAGT
GACATTCCAACCATAATTTTTGGAGCTGATGTAACACATCCAGAGTCTGGAGAGGACATC
AGTCCGTCTATAGCTGCTGTTGTAGCCTCCCAAGACTGGCCAGAAGTTACAAAATATGCT
GGGTTGGTTTGCGCTCAGCCTCATCGGCAAGAGCTTATTCAAGATTTATTCAAAACCTGG
CAAGACCCTCAGCAGGGGACAGTTGCTGGTGGGATGATCAGGGAGCTCCTACTTTCATTC
AAGAAGGCTACCGGGCAAAAGCCATTGAGGATCATATTTTACAGGGATGGTGTCAGTGAA
GGTCAATTCTATCAGGTTCTACTGTATGAACTGGATGCAATTCGAAAGGCTTGCGCATCA
CTTGAACCTAGTTACCAACCTCCGGTAACATTTGTTATTGTCCAAAAGCGGCATCATACT
AGACTTTTCGCAAGCAATCACAATGATAGAAGTAGCATTGACAGGAGTGGAAATATTTTA
CCTGGTACTGTTGTGGACACTAAAATATGCCATCCGACTGAATTTGACTTCTATTTATGT
AGTCATGCAGGAATTCAGGGGACTAGCCGACCTGCACATTACCACGTGCTGTGGGATGAA
AACAACTTCACCGCCGATGAGATTCAATCTCTGACGAACAACCTTTGCTATACGTATGCT
AGGTGTACGCGGTCGGTTTCTGTAGTTCCTCCTGCGTATTATGCTCATCTGGCAGCTTAT
CGAGCTCGATTTTACATGGAACCTGACGCATCAGAAAATCCTAAAATTTGCCGCACGCTC
ACAGCTAATGGATCGTGTGTCCGCCCGCTGCCTGCATTGAAAGAGAAGGTGAAGAATGTG
ATGTTTTATTGCTAG
>Ricinus_communis_29813.t000096
ATGGAAGAGACTAGAGAATCCAATGCTAACAAGAAATGCACCAGCAAGCCTAGAACACTT
AGAGGTAGAACTAATACGCATAAGCATCAGTATCAATATCAGTACCAATATCAGTATCAG

CATCACTTCCTTCAATACTCCAACCAGTTTGGTTTCTTTAATCACAGCAGCAACCTCTAC
CCAAGTTACTATCCAGCTCTTCTTCCTCTTCCTCCTCCAATACCTCTTCAACTTGCTTTA
AATCCTCCTTTCCCTCAAAACCATAGCTTTGGATCAAAAACCCATTTCCAGAAACCTTCA
TGTAAGCTAAATAATCCTCCTCGCCCCACCTCTTCTGCTACCCCGGAACCTCTTCTTTCA
ATTTCATCAGCTCCAGAGCGGCTGCAACCAAGAAAGAGTCTGCCTTTGAAAAGAAATGAC
AGAAGGAAAGGCGTGGGTTCCACTACACAAGCACTGGTAGTTGCAAGGAGACCAGATTCA
GGTGGTGTGGAAGGACCAGTTATCACTCTCCTCGCCAACCATTTCTTGTCCAATTCAAT
CCCTCACAGAAAATTTTCCACTACAATGTGGAAATTTCTCCCAATCCTTCCAGGGAAGTT
GCCAGGATGATCAAACAAAAACTGGTAGATGAAAATTCAGCTGTGCTTTCTGGTGCTTTT
CCGGCCTATGATGGCCGAAAGAATCTTTACAGCCCAGTTGAATTCCAAAATGATAGGTTC
GAAGTCTACATTAGCCTCCCAATCCCAACTAGCAAATCATCATTGCCTCTTGGGGAACTT
AATGACTTTCAGGAGAAGCATCAACAGCTCAAACTATTTAGACTTAATATCAAACTTGTA
TCAAAATTGGATGGGAAAGAGCTGGCTAGCTACTTGAGCAAGGAAAGTGATGACTGGATT
CCACTTCCTCAGGATTATTTACATGCTTTGGATGTTGTATTAAGAGAAAGCCCCATGGAG
AAATGTATACCTGTAGGGAGATCATTCTATTCAAGTTCAATGGGAGGAACTAAAGAAATT
GGAGGAGGGGCTGTTGGATTGAGAGGATTCTTTCAAAGTCTTAGACCTACACAGCAAGGA
CTAGCTCTCAATGTAGACTTCTCTGTGACTGCTTTTCATGAAAGTATCGGTGTGATAGCC
TACCTGCAAAAGCGTCTTGATTTTCTGTGGGACCTTCCTCAAAACAAAAGGAGGAGTTTG
ATTGGTGAAGAAAGGAAAGAAGTAGAGAAAGCCTTAAAGAACATCAGGGTCTTCGTTTGT
CACAGAGAAACTGTTCAGCGATACCGGGTTTATGGCCTAACTGAACAAGCTACAGAAAAT
CTTTGGTTTGCCGACAGAGATGGGAAGAACCTGAGGCTACTGAGTTATTTCAAGGATCAC
TACAACTATGATATAAAATTTAGGAACTTGCCATGCTTGCAAATTAGTAGAAGCAAACCA
TGTTATCTTCCCATGGAACTTTGTATGATCTGTGAAGGCCAGAAGTTTCTTGGGAAGCTC
TCAGATGATCAGACAGCAAGAATACTTAAGATGGGCTGCCAAAGACCAAAAGAACGAAAA
GCCATAATAAATGAAGTCATGAGAGGATCTGTTGGTCCAACAAGTGGCAACAAGGACAGA
GAGTTCAAACTCCATGTTTCAAGAGAAATGACAAAATTAAAGGGGAGAATCCTACAACCT
CCAAAACTCAGACTTGGTAATGGTGGCTCCAAAAGAGATCTAATTCCATCTCGCCATGAT
CGACAGTGGAATCTTCTAGATAGCCATGTCTTGGAAGGAACTAGAATCGAGAGGTGGGCA
CTGATGAGTTTTGGAGGCACCCCTGAACAGAAGTCCAACATTCCAAAATTCATAAACCAG
CTATCTCAAAGATGTGAACAGTTAGGTATCTTTCTTAACAAAAACACAATAATTAGCCCT
CAATATGAACCAACGCAAGTGCTTAATAATGTCTCCCTTCTGGAATCCAAACTCAAGAAA
ATCCACAAAGCTGCCTCAAACAATCTCCAGCTGCTTATATGTATAATGGAGAAGAGACAC
AAAGGATATGCAGATTTGAAGCGAATAGCAGAGACAAGTGTTGGCGTTGTAAGCCAGTGC
TGCTTATTTCCAAATCTTGGGAAGTTGAGTTCACAATTTCTGGCTAATTTGGCTCTGAAG
ATCAATGCCAAAGTTGGAGGATGCACAGTTGCTTTGTTCAATTCGCTACCCTCACAGATT
CCACGCCTTCTTCATTCTGATGACCCTGTGATCTTTATGGGAGCTGATGTTACTCATCCT
CACCCTCTTGATGACTTCAGTCCATCTGTTGCTGCTGTTGTTGGGAGCATGAACTGGCCG
GCAGCAAACAAGTATGCCTCGAGGATGAGGTCACAAACTCATCGACAAGAAATTATCCAG
GACCTTGGTGCAATGGTGAAAGAATTGCTAGATGATTTTTTCCAAGAAGTTGGCAAACTT
CCCAAGCGGATAATATTCTTTAGAGATGGAGTAAGTGAAACCCAGTTTCATAAGGTTCTT
CAAGAGGAGTTGCAAGCGATTAGAGAAGCTTGTTCTAGATTTCCTGGTTATAGACCTCCC
ATTACTTTTGCAGTAGTCCAAAAGAGGCATCACACTAGGTTGTTTCCTTGCGAAACTGAT
CTAGCTTCAATTCAGAACCAGTTCTACGATGAAAATATACCGCCAGGGACAGTTGTGGAT
ACCGTGATTACTCATCCAAAGGAATTCGATTTCTATCTATGCAGCCATTGGGGGGTGAAA
GGAACAAGTAGACCAACTCATTACCATGTCTTATGGGATGAGAACCAATTCACTTCTGAT
GAATTACAGAAGTTGGTTTACAATCTGTGCTATACATTCGTAAGGTGCACCAAGCCAGTT
TCTTTAGTCCCTCCTGCTTACTATGCTCACTTGGCTGCATACAGGGGTAGACTTTACCTT
GAGCGATCCGAGTCCATGACTTCCGCGAGAAATGCTTCTGCAGTCTCAAGAGCTGCACCG
CCAAAGGCAACACCTCTACCAAAACTCAGTGAGAATGTAAAGAATCTTATGTTTTACTGC
TGA
>Ricinus_communis_29828.t000011
ATGGAACCTCCGGAAGAGGCAGAGGCATTGCCTCCACCTCCACCTGTGGTACCGGCTGAT
GTAGTTCCCATTCAACTAAAACCAGAACCAGACAATGTTCCTGAGACAACTGAAAAGGCA
ATAAAACCAAAACGTGTTCCAATGTCCAGGCGTGGTAATGGATCTAGAGGACAAAGAATA
GAACTACTAACTAACCATTTCAAAGTTGGTGTCAACTGTGATGGTGGCCACTTCTCTCAC
TACAGTGTTGCGTTGTTTTATGAGGATGGTCGCCCTGTTGATTCAAAGGGGATTGGGAGA
AAAGTTATTGATAAGGTTCGTGAGACTTATGATTCTGATCTTGCTGGTAAGGACTTTGCT
TATGATGGAGAGAAGAGCTTATTTACTGTTGGTTCTCTTCCACGTAACAAAATGGAATTC
ACTGTTTTGCTTGACGATGTCTCATCAAATAGGATTAATGGCAGTGGCAGTCCTGTTGGT
AATGGAAGTCCTAACGGAAGTGAGAAAAAGAGGATGAAGCGGGTGTTCCATTCTAAAACA
TATAAAGTGGAGATAAGTTTTGCTGCCAAAATCCCAATGCAGGCTATCAAAGCTGCTTTG
CGTGGTCAAGAATCAGAGAACTCCCAAGAAGCCATCAGAGTCTTAGACATTGTTCTGAGG

CAGCATGCAGCAAAACAAGGCTGTCTTCTTGTTCGTCAATCATTCTTTCATGATGATTCG
AGGAACTATGTCGATCTGGATGGTGGTGTACTCGGATGCAGGGGATTTCATTCAAGTTTT
AGAGTCTCACAAGGCGGATTATCACTGAATATTGATGGGTCAACTACAACAATAATACAG
CCTGGGCCATTAATTGACTTTCTTCTAGCCAACCAACATGTGTCAACTCCTTTTCAAATT
GATTGGTCAAAGGCTAAACGTACCCTGAAAAATTTGAGGATAAGGGTATCACCCACCAAT
CAGGAGTACAGAATCACTGGCTTGAGTGAGAATCTTTGCAAGGACCAGATATTCTCAATG
AAATCAAAAGGACTGAATGATGGAAATTGCGATGATGGAATGGTCGATATTACAGTTTAT
GAATATTTCGTTAATCATCGTAATATAGACTTGCGCTACTCTGGTGATTTGCCTTGCATT
AATGTTGGAAGGCCTAAACGACCTACTTTCTTTCCTATTGAGCTGTGTTCATTGCTTCCG
TTGCAACGCTATACAAAGGCATTATCTGTTATTCAGAGGTCAAAGTTGGTTGAAAGTTCA
CGTCAAAAGCCCCAGGAGAAGATGAAGATCTTGGCTGATGTAATGAAAAGCAACAACTAT
GGTGCAGATCCTATATTGCGCTCTTGTGGTATTACTATCAGCAGTCAGTTTACTCAACTG
GAAGGCCGTGTTCTAACTGCTCCAAGGTTGAAAGTGGGAAACGGGGAAGATTTAATTCCA
AGAAATGCACGGTGGACCTTTAATAATAAGAAATTTGCTGAGCCTGCCAGAATTGAAAAC
TGGGCGGTGGTAAACTTTTCAGCTCGTTGTGACATTCGCGGTTTATGTAGAGATTTATGT
AGAGTTGGAGAAATGAAAGGGATTATGATAAGTCCCCCAGAGCATGTTTTTGAAGAGAAT
CCTCAATTTCGACATGCACCACCTCCTATTCGAGTGGAGAAGATGTTTGAACAAATACAG
CCAAGATTCCCTGATAATCCTCCTCGATTTCTTCTGTCTATTTTTCCTGATAGGAAGAAT
TCTGACATATATGGTCCATGGAAAAGGAAGAATCTGGCAGAATTTGGAATTTTCAATCAA
TGCCTTTGTTCACCTAATAGACTCAGTGAGATGTATGTTACGAATGTTCTCATGAAGATA
AATGCCAAGCTTGGTGGTTTAAATACTTTCTTGGCTGTTGAACAATCACGAAACGTCCCT
TTCGTTTCAAAGGTTCCTACAATAATTTTTGGTATGGATGTTTCACATGGTTCTCCTGGA
CAATCTGATGTGCCATCTATTGCTGCGGTGGTAAGTTCTAGGAATTGGCCACTACTATCT
CGTTATAGAGCTTCTGTTCATAGTCAGTCACCAAAGGTTGAGATGATTGATTCCCTTTTT
AAACCAGAGGGAAAAGATGATGATGGAATAATCAGGGAGTTGCTGCTGGACTTCTATAGG
AGTTCTGGTCAGACAAAGCCGGCTCAGATAATCATATTCAGGGATGGAGTCAGTGAGTCA
CAGTTTAATCAAGTCCTCAACATCGAGTTAAATCAAATCATTGAGGCTTGCAAGTTCCTT
GATGAAAGTTGGTCGCCTAAGTTCACTGTGATTGTAGCACAAAAGAATCACCACACTAAA
TTCTTCCAATTACGATCTGCAGAAAATGTCCCTCCAGGAACTGTTGTAGATAATGGTGTT
TGCCACCCACAAAGCAATGATTTCTACATGTGTGCCCACGCAGGGATGATTGGGACAACC
AGGCCAACACATTACCATGTTCTGTTAGATGAAATTGGCTTTTCAGCTGATGATCTACAA
GAACTCATTCATTCTCTATCCTATGTGTATCAGAGAAGCACAAGTGCAGTATCTGTAGTT
GCTCCTGTTCGTTATGCTCATTTGGCAGCAACTCAAATAAGGCTATTCATGAAGTTTGAA
GACATGTCCGAGACTTCTTCAAGCCATGGAGGCCTGACTACTTCAGGGCCTACCCCTGTG
CCCGAGCTACCAGTGCTGCACCAGAAAGTGCGCAGTTCTATGTTTTTCTGCTGA
>Ricinus_communis_29844.t000058
ATGCCTATAAGGCAAATGAAAGAGAGTTCAGAGCAGCACCTAGTGTTAAAAACCCACTTG
CAAAACACTATGAACCAACCTCAGAAACACCATAAAATTGCCCAAAATGGCAAGGGACCA
CCACAATCTCAAGAAACTCACAACAGCAAACCCCAAAACCAGACCTCACCTCCAACAAAA
AATAGAGGAAGAAGAAGAGGAAGAGGTGGTAGAAAATCTGATCAAGGAGATGTCTTTACG
AGACCCAGTTCAAGGCCTTGTACTGTGGTGCATAAGCCAGTGAATCAAGCTGGTGGTCTC
TTGGCAAATGCTCCCAATGGAAATAGTGGAAATATTTGTGAAATGGAGATGGGTTTGGGA
TTCCCTACTTCTAGCAAGTCCTTAACTTATGCTCGTAGGCCTGGTTATGGTCAACTTGGG
ACAAAATGCATTGTTAAGGCGAACCACTTCTTTGCAGAGCTGCTGGACAAGGACTTAAAC
CAGTATGATGTTACGATAACTCCTGAAGTGGCATCAAGAACTACTAACAGAGCTATCATG
GCAGAGCTTGTGAGGCTCTACAAAGAATCTGACTTAGGAATGAGACTGCCTGCTTATGAT
GGCAGAAAGAGTCTTTATACATCTGGTGAGCTTCCCTTTGCTTGGAAGGAGTTCATCATT
AAGCTTGTTGATGAAGATGATGGAGTCAATGGCCCCAAGAGAGAAAGGGAGTACAAAGTG
GTAATCAAGTTTGTTGCGAGGGCCAACATGCATCATCTGGGTCAATTTTTAGCTGGTAAA
CGTGCTGATGCTCCACAGGAAGCTTTGCAAATTCTTGACATTGTACTGAGGGAGCTCTCA
ACAAGGAGGTACTGCCCAGTTGGTAGATCCTTCTTTTCACCAGATATTAGAGCACCACAG
CGACTTGGTGATGGCTTGGAGTCATGGTGTGGGTTTTACCAGAGTATAAGACCTACACAG
ATGGGCCTGTCACTGAATATTGATATGGCTTCAGCTGCATTCATTGAGCCTCTCCCTGTA
ATTGAGCTTGTTGCCCAGCTTCTAGGCAAGGATGTGCTATCAAGGCCATTATCTGATGCT
GATAGAATTAAGATCAAAAAGGCTCTCAGAGGAGTTAAAGTTGAAGTAACTCACAGAGGA
AATGTACGAAGAAAGTATCGTGTCTCAGGATTGACATCTCAACCTACGAGAGAACTTGTA
TTTCCTGTTGATGATAACTCAACTATGAAGTCAGTAGTAGAATATTTCCAAGAAATGTAT
GGCTTTACCATTCAACATACACATCTACCTTGCCTTCAAGTAGGAAACCAGAAGAAAGCC
AACTATCTACCAATGGAGGCTTGCAAAATTGTAGAGGGGCAAAGATATACAAAAAGGCTG
AATGAGAGGCAAATTACTGCCCTGTTGAAAGTTACATGCCAACGACCTAGGGACCGGGAA
AATGACATTTTACAGACAGTTCAGCATAATGCTTATGATCAAGACCCTTATGCAAAGGAA
TTTGGAATTAAAATCAGCGAAAAGCTAGCTTCTGTTGAGGCTCGAATTCTCCCTGCCCCT

TGGCTGAAATATCATGATACTGGAAAGGAAAAGGATTGCTTGCCTCAGGTTGGCCAATGG
AATATGATGAACAAGAAAATGATCAATGGGATGACTGTAAGTAGGTGGGCTTGTATTAAC
TTTTCAAGGAGTGTGCAAGAGAGTGTTGCTCGTGGGTTTTGCAGTGAACTTGCTCAGATG
TGCCAAGTGTCTGGCATGGAATTCAATCCAGAGCCTGTTATCCCAATTTACAGTGCCAGG
CCTGAGCAAGTTGAGAAAGCTTTGAAGCATGTTTATCATGCTTCCATGAACAAAACCAAA
GGAAAAGAGCTGGAGCTTTTATTAGCTATTCTACCTGACAACAATGGCACCCTATATGGT
GATCTCAAAAGGATCTGTGAAACGGATCTTGGTTTAATATCACAATGCTGTCTTACAAAA
CATGTCTTCAAGATCAGCAAGCAGTATCTGGCTAATGTGTCCCTTAAGATTAACGTTAAG
ATGGGTGGCAGAAATACTGTCCTTTTGGACGCCATAAGCTGTAGAATACCATTAGTTAGT
GACATACCAACCATTATATTCGGAGCAGACGTGACTCACCCAGAGAATGGGGAGGATTCA
AGCCCCTCAATTGCAGCTGTAGTAGCTTCACAGGACTGGCCTGAAGTGACAAAATATGCT
GGATTAGTTTGTGCTCAGGCTCACAGGCAAGAACTCATACAGGACTTGTACAAAACATGG
CAAGATCCTGTTCGTGGTACTGTTAGTGGTGGCATGATCAGAGATCTTCTGGTCTCGTTT
CGGAAGGCAACAGGCCAGAAACCGCTAAGGATTATATTTTACAGGGACGGTGTTAGTGAA
GGGCAATTTTATCAAGTTCTGCTTTATGAATTAGATGCAATAAGAAAGGCCTGTGCTTCT
CTAGAACCAAACTATCAACCTCCTGTGACTTTCATTGTTGTACAAAAACGCCACCATACT
CGATTGTTTGCTAACAACCATAGGGATAGGAGTAGCACAGACAAGAGTGGGAACATATTA
CCTGGCACTGTGGTTGATTCTAAAATCTGTCATCCGACAGAATTCGATTTTTACCTCTGT
AGCCATGCTGGTATTCAGGGGACTAGTAGGCCTGCTCACTACCATGTTCTGTGGGATGAG
AACAACTTCACTGCAGATGGAATCCAGTCTTTGACAAACAATCTGTGTTACACATATGCA
AGGTGTACTCGCTCCGTTTCCGTTGTACCTCCGGCATACTATGCGCATTTAGCTGCTTTT
CGTGCCCGGTTTTACATGGAGCCAGAAATGCAGGATAACGGCTCAACAGGTACCAGGGGA
ACACGAGCAGCTGGAGAAACTGGAGTCCGGCCATTGCCAGCCTTGAAGGAAAATGTGAAG
AGAGTAATGTTCTATTGTTAA
>Ricinus_communis_30093.t000002
ATGGAGAGTGGTGGTGGGGGCTTTATGGGAGGAAAGGCGGCTACTGGCGGTGGTCGTGCT
GCTGCTGGAAGAGGGAGAGGGAGAGGAATGAATAATGATAATAGTGGATGCACTGGTGGT
GGCCGCGGTGGTTATCAGTATCGTACTCATTATCATCAACAGCAGCATGGTCAGAGTGGG
GTTTCAAGCCAAGATAACTCCAGTTATGGTGGCCGTGGTGGTGCTGGTGGTTATGGCTGC
CGTGGTGGTGATGGTGCTGGCCGCGGTGGTGGTGGTGCTGGGGGTAGATTCAGCGGTAGT
AGTGGAAGAGGAGGACGAGGACGACGAGGGAGCAGCAGGGGATCTGCCAGTAGCCAAGAT
ATAGCAGCCATTAACCCTGAACATACTGTTCAACAAATGCAGCCATTAAACCTTTCTCGA
CAAGATAACAGCAGTGGCAAAAATAATATCCGGACGATTAGCCTTCGTGTTAATCATTTT
CTGCTAAGCTTCGACCCAGAAAGCATCATCAGGCACTATGATTTTTCCATTAAACCAGAT
GTACCTGCAAGGAATAGTTTGCCTATGAAGGTGCCAAAGACCATTCTATCCATGATACGG
AACAAGTTATTCTCAGATGACCCTACCCGCTTCCCCTTGTCAATGACTGTATATGATGGT
GAGAAGAACATTTTCAGTACAGTTTCATTACCCACAGGTAAATTTAAGGTCGAATTATCA
AAGAATGAAGGAATCAGGATCCGTTCGTTTATGGTTGAACTCCAGCTTGTGAATGAGCTG
AAATGTGACAAGTTGAATGATTATCTTAGAGGAAGGGTGGTTTCGGTTCCTCGTGAAGTA
TTGCAAGCTTTGGATGTGGTCATGAAGGAGAATCCAATGAGGCAAATGATTTATGCTGGT
CGAATTTTCCATCCTATCATGCCTTATCCTGGGGATGATCTCAGACGTGGAATTACAGCT
TCTAGAGGAATCAAACATACCCTGAAGCCAACATCCCAGGGTCTAGCCTTGTGTCTGGAT
TATTCAGTTCTGCCTTTGCTCAAGCAAATGCCGGTCATAGATTTTCTCAAGGAGCATATT
CGAGGCTTTAACTTGAACAATTTCAGAGCTTTCAGGAGAGAAGTGGAGAGAGTTTTGAAA
GAATTGAAAGTTACTGTTAATCACAGAACTACAGGTCAGAAATTCAAAATAGCAGGTTTA
ACCCACGATGACACACAGGATATTTCATTCGAGGTGGATCGTATCTCTGAGAGGAAAGTT
TGGCTTGTTGACTATTTTAAAGAAAAATACAACAAGAATATTACACACAGAAATATACCA
TGCTTAGATTTAGGTAAAAAGAACAGGACAAATTATGTTCCTATGGAGTTCTGCAGCATA
GCTAAGGGGCAGAGGTTTGCAATGGAGGATCTGGATCGAAATCAATCCGAAAAGTTGAGG
AGAATTTCATTAGCTTCGCCAAAGTCTAGAGAGGGCATGATATGCGACATGATACAATCA
AGTGATGGACCCTGTGGTGGAGATATCAGCCAAAATTTTGGGATTGGAACAGATCTGAAT
ATGACAAAAGTTACTGGTCGGGTTCTTGCGCCACCAGAACTGAAGCTTGGCAATTCAGGA
GGAAGACCAACAGCTGTTGATAGAGACAAATGCCATTGGAACCTTTTCAAAAAGTCGGTA
GTTCACAGCAAACCAATTAGGCTGTGGGGAGTGCTCAACTTCGGTAGTAATGATCTTGAA
AAATTCATTCCTGAGCTGATCTCTAACAGCGAAAAACTGGGCATTCACATGGATGAGCCT
CTTTTCTGTTTACATCATCCAATGAATTTATTACACAATGTTGATAATCTCCAACAGCTG
CTTGAAAGTGTTAATAATGAGTGCTATAAGAGGAATGGAGGGGAATATTTACAGATTCTT
GTTTGTGTCCTGCCAAAGGAGGATCCTGGTTACAGCAATCTCAAGTGGATATGTGAGACC
AAAGTTGGCATTGTAACTCAATGTTGTTTATCCGAAAATGCATTTAGACCAAAAGCTCAA
TTTCTTGCAAATCTAGCTTTAAAGATCAATGCGAAGCTTGGAGGCAGTAATGTGGAGCTC
TTCAAGCAGCCTCAATGCTTGCAAAGTAAAGGCCATGTTATGTTTATTGGTGCTGATGTT
AACCATCCTAGCTCTTACAACTCAACAAGTCCTTCAATAGCAGCTGTTGTTGCCACCATG

AATTGGCCTGCAGCAAATCAATATGGCGCACTGATTTGTCCTCAAGATCATCGTGCAGAG
AAAATCCTGAAATTCGGAGACATGTGTTTAGAGCTTGTGAATGCTTATGCCCGGCTAAAT
CAGGTAAGACCAGAGAATATTGTTGTATTTCGTGATGGAGTAAGTGAGAGCCAGTTTGAT
ATGGTTCTCAATGAAGAGTTGAAGGATATCAAGGCGGCATTTGAATCACTTGAATACTTC
CCAACCATCACTCTTATTGTTGCCCAGAAGCGACACACAACTCGTTTGTTTCTAGATAGT
GATGAAGATGAAAATGTACCTCCGGGCACTGTGGTGGATACAGTAATTACAAGTCCTTCT
GGGTCTGATATCTATCTTTGCAGCCATTTTGGTCAAATTGGGACGAGCAAGCCAGCACAC
TACCAGGTGCTGCAGAACGAGATCGAGTTTACTCCTAATGAGTTGCAGGAGTTCATTTAC
AGCATTTGCTTCACCTCTGCTCAGTGCACCAAGCCTGTCTCACTTGTCCCTCCAGTGATC
TATGCTGATCGTGCAGCTTTTAGAGGTCGGCTTTACTACAATGCAATGGAGTTGCATCAG
CCTTCAGCACCTTCAACATCATCATCTTCACTGGCTTCCTTTGATGAGCAGCCTTTCAGG
CTACACCCTAACCTGGAGAACTCTATGTTCTATATTTGA
>Sorghum-bicolor_Sb01g004920
ATGGCTTaccgcggcggcggccgaggaggaggccgcggcggcgACCGCGGGGACCAGCGC
CCGCCGTACGGCCACGGAGGAGCCGGGGGATccggaggcggcggcggaggccgGCCCTTC
ATCTGGCCGCCACCGCCTTCCACGCCGCGCCCGGTGAtgccggcgccgccgccggggccA
TACCCGGTCCCGGTGCCGATGGGCGGCTACCGCGGGCCCATGGTGATGCCGCACCCGGGC
GCGTACGGGGTTCCGATGGCCGTGTACCGGCCGTCCGGGCAAGCGGCCGTGTTCAGGGCG
CCCGCGCCAGCGGCGCCGCAGGTCTCCTTCTCGCCTGCGCCTCCGGCAGCTCCGGTCACC
ATCAGGGCGCCGCCACCATCCTCGAAGACGACGTCGCCGGCTGCTCCGTCGTCTACCACT
ccggcgcctccacgccAGTCCGCGCGGCAGAGCGCCTCCACGCCGGCGTCCGATTCGGCT
CCGGCCGCCTCCTCCGCGCCGTCTCCCGCGGCGGCCCTGGCgaaggaggtggagaagAAG
CTGTTCGTCTCCGAGACCGCGCTGGCGCCGCCTGCGTCGGCGGCGCATGGGACGGCTGCT
GCCCGCGCGGACGAGGCGTCGGACCTCGACCTGGCGCCGGTCTCCAAGAAGGGGCTGGCG
CACCCCGCTCGCCCGGGCCTCGCCACCGTGGGGAAGAAGGTGATGATCCGCGCGAACCAC
TTCCTCGTCAACGTCGCCGACAACAACTTGTTCCACTATGATGAAGTGGACTGCAGATCA
GTGCCGTTCCGTAAAAGGCTCTTGCCAGAACATGGAATCGGAAAAACCATTGGGCAGATG
CGTTGGAAATCTTGCATCTGTTTGGTGGCTATTAATCCTGAGTCAAAGTCAAGACAAACC
AACAGGGAAGTTCTGAATGAGCTTATCAAGTTGCATGGGAAGACAGCTCTGGGTGGCAAA
CTGCCTGCCTATGATGGAAGGAAGAGTCTTTATACTGCTGGTTCACTTCCTTTTGAATCA
GAGGAGTTTGTGGTTACGCTGGTTGATCCAGAAaagaaggacaaagaaaGTTATGTCACA
GTGTCCAGATCCTTTTTCTCTACTACTTTTGGCCACCGAGGCGACATTGGCGAGGGGCTT
GAGTGTTGGAGGGGTTACTACCAAAGCCTGCGCCCGACACAAATGGGGCTTTCACTGAAT
ATAGacatatcagcaacatcCTTTTTTAAGCCTGTGTCAGTTATCAAATTTGTGGAGGAG
TACCTGAACATGCGTGATACATCTCGGCCTTTGTCGGACAGAGATCGTGTGAAGATAAAG
AAAGCATTACGTGGAGTTCGCATTGAAACAACCCACCAACAGGATCAAATCAGAAGATAC
AAGATAACAGGGGTTACTTCCATCCCTATGAGCCAGCTGATATTTCCTGTTGATGATAAA
GGAACAAGAAAGACTGTTGTGCAGTACTTCTGGGACAAATACAACTACAGCTTGAAGCAT
GGTTCTTGGCCTTGTCTTCAGGCTGGCAGTGATTCACGACCTGTATATTTGCCTATGGAG
GTTTGTAAAATTCTGGAAGGGCAGAGATACTCTAAGAAGCTTAATGACAGACAAGTGACC
AACATACTTAGAGCAACATGTAAACGTCCCCAGGAGAGGGAGCAGAGCATACATGATATG
GTTCTGCATAACAAGTATGCAGATGATAGGTTTGCTCAGGAGTTTGGCATCAAAGTTAGC
AGTGATCTAGTGACTGTTCCAGCCCGTGTGCTGCCTCCACCCCTGTTGAAATATCATGAG
TCTGGTAGGGAGAAAACCTGTGCGCCAAGTGTTGGACAATGGAACATGATCAATAAGaaa
atgatCAACGGAGGAACTATTGATAACTGGACTTGTTTGAACTTTTCACGCATGCGACCT
GATGAGGTACAGAGGTTCTGTATGGATCTGATTCATATGTGCAATGCAACTGGAATGGTT
GTCAATCCACGTCCATTTGTTGATGTCAAGTCTGCAGCTCCCAACCatatagagaatgct
ttGAGAGATGTACACAGGAGAGCCACACAAATGCTTGCCCAACaaggagtgggaaatcaG
CTGCAGCTTCTGATCGTAATTCTGCCTGATGTTAGTGGTTCTTATGGTAAAATCAAAAGA
GTCTGTGAGACTGACATTGGAATTGTATCTCAGTGTTGCTTGCCAAAGCATGCTAGCAGA
CCAAACAAACAATATTTAGAAAATGTTGCACTCAAAATCAATGTCAAGGTTGGTGGGCGC
AACACAGTTCTTGAGCGAGCCTTTGTACGCAATGGCATACCCTTTGTGTCAGAAGTCCCA
ACAATCATCTTTGGTGCAGATGTCACACACCCCCCACCAGGAGAGGACTCTGCCTCATCC
ATTGCTGCTGTGGTGGCATCAATGGACTGGCCAGAAATCACCAAGTACAGAGGCTTGGTT
TCTGCTCAACCACACCGACAAGAGATAATAGAAGATTTGTTTACTGTCACTAAGGATCTG
CAGAAGGGGCACAGTGTTAATGGTGGAATGATCAGGGAGCTACTGATTGCTTTCCGCAGG
AAGACAAACAGAAGGCCTGAGAGGATAATATTCTATCGGGACGGTGTAAGTGAAGGTCAA
TTCAGCCATGTACTTCTTCATGAAATGGATGCCATCAGAAAGGCTTGCGCTTCTTTGGAG
GAGGGATATCTACCTCCAGTCACATTTGTGGTTGTCCAGAAAAGGCATCACACAAGGCTT
TTCCCTGAGGTtcatggaaggcgtgataTGACTGATAAAAGTGGAAACATTCTTCCCGga
actgtggtTGACCAACAGATTTGCCATCCTACTGAGTTTGATTTCTACTTGTGTAGTCAT
GCTGGTATTCAGGGAACAAGTCGGCCCACCCATTACCATGTCCTCTATGATGAGAATCAT

TTTACTGCTGATGCACTGCAGTCACTGACCAACAATCTATGCTACACTTATGCTCGTTGC
ACCCGTGCTGTATCAGTGGTTCCACCAGCCTACTATGCCCACCTTGCCGCATTCCGGGCA
CGCTACTATGTGGAAGGAGAAAGCTCAGACGGTGGCTCAACCCCTGGCAGCAGCGGGCAG
ACAGTAGCCCGGGAGGGTCCTGTGGAGGTGCGTCAACTTCCGAAGATCAAGGACAACGTG
AAGGACGTTATGTTCTACTGCTGA
>Sorghum-bicolor_Sb01g011870
AtgtccacgcgcggcggcggaggaggaggccggcGCGGAGGACGGGGTGAACAAGGAGGA
GCCCGTGGAAGCGGGAGCGGCGCTGCCGGTGGTGGCCGAGGGCGCGGCCAGGGCGCCGCT
GCAGACCTCGAAGGATTCCGTAAGGACACGCGCGGCGGGGCTGGGCATGGCGATCGTGGC
GGCGCCACGACGCCAGGCGCCGCACAGAGAGGCGGCCATGGCCAGCCCCCGTCCCCGCAT
CCGGCTGCCGGGCCTGGGCGTGGCGGCTACTCTGGAGTTCATCCTCAGCAGGGGCGCGGC
CAACAGGTGACAGCGCCGGTGTTGCCCTCGGCGCCGACCCCGGCGGAGGTTGAGGCGGTG
AGGCGCCAGGTGGAGAGAAAGGTTGTAGTGGCCGAGGCGCAGGTGGGGCCGCGCCAGGgc
tcgtcgtcgtcctcACAAGCACCGGCGCCGAGACCGGCGATGCAAGGCAAGGTGCCCGAT
CAGTTCGCTCCTGTCGGGAGAGGGAGCCCGTCGTTCCCAGCGCAGTCTCCGTCGTCCTTT
CAGGCGCCTGCGGTGCGACCGGCTATGCAAGGGAAGCCGCCGGGTCAGGTCGCACCGGCA
GCGATCCCATCGTCGTTCCCAGCGCTGGCGCCAGTTCGGGGCCCTACAACCGGACGACCT
GCTATGCAACAAGGGAAGCCGCCAGGTCAGGTGGTCGCACCGGCAGCGAGCCCATCGTCG
TTCCCAGCGCTGGCGCCGGTGCAGGGGCCTACGCCCGGGCGACCAGCCATGCAAGGGAAG
CCGCCCGGTCAAGTCGCACCGGCAGTGCGCCCATCGTCGCTCGCAGCGCCGGTTCAGGGC
CCTGCGCCCGGGCGACCGGCCATGCAAGTGAGGCCGCCGGGTCAGATCGCACCGGCGGCA
GCGAGCCCATCGTCGCTCCCAGCGTCGGCGCCGGTTCAGGGCCCTGTGCCCGCCGGGCGA
CCGGCCATGCAAGTGAGGTCGCCAGGTCAGGTCGCACCAGCAGCGACCCTAACGTCACtc
ccagcgccggcgccgccAGTTCAGGGCCCTGTGCCAGGGCGACCGGCCATGCAAGTTAGG
CCGCCGGCGCCGGGTCAGGTCGTCGCACCGGCATCGGGACCGCAGATGCAAGGGAAGGCG
CCGGCCGGTCAAGTGGCGCTTTCGACGCCTGCAGGTACGCTGCCTCCGGCTTCGAGCAAG
GCGATGGTGTTCCCCGCGCGGCCGGGGTACGGAACCGTCGGGCGGAGGTGCCAGGTGCGC
GCCAATCACGTCCTTGTACAGCTCGCTGACAAGGATATCTACCACTACGATGTGACAATC
ACACCAGAATCGGTGTCAAGGGCAAGAAATAGATGGATCATCAACGAGCTCGTTAGCTTG
CACAAGAAACACTTGGACGGGCGGCTTCCTGTTTATGATGGAAGCAAAAGCCTGTTCACG
GCAGGGCCACTGCCATTCAAATCCAAAGAGTTTGTGCTCAATCTGACAAACCCTGAGAGA
GCAAGCCAAGGCGAGAGAGAGTACAGGGTGGCAATCAAGGATGCTGCAAAAATTGATATG
TACAGCCTTAAAATGTTCTTGGCTGGCAGGAATCGGGATCTGCCACAAAACACTATCCAG
GCTCTGGATATCGCTTTGAGAGAATTCCCAACTTCTAGGTATACATCGATCTCAAAATCG
TTTTTCTCACATGAAGCATTTGGAAATGGTGGGCCTCTAGGAAATGGTGTGGAATGCTGG
AGGGGTTACTACCAGAGCCTACGCCCTACACAGATGGGGTTGTCCCTTAATATAGATGTT
TCTGCAACGTCATTTTTCAAGGCTCAACCTGTTATTGACTTCGCAGTGGATTATCTGAAC
CTCCATGATACTAAAAGGCGTTTGTCTGATCAGGATCGCATAAAActgaagaaagcacTT
AAGGGGGTCCGGGTCGCAACCAAGCATAGACATGATATATCCATGCGCTACAGGATTACG
GGGCTAACCTCGGCTCCATTAAATGATTTGACGTTTGATCAAGATGGCACAAGGGTGTCA
GTTGTGCAGTACTTCAAACAACAATATGACTACTCATTAAAATACACTCACTGGCcatgc
cttcaagctggCAGTGCTAGCAAGCAGATCTATTTACCTATTGAGGTTTGCAGCATAGTT
GAGGGACAACGCTACTCGAGTAAGCTGAATGAGAATCAAGTCAGGAATATCCTGAAGTTG
GCCTGTGAGCGACCGTCAGAGAGGGAGAATAGAACTCTTCAGGTATTCAGTAGGAACAAC
TCCCCTGATGATTCTTATGCAAAAGAATTTGGCCTTAAGGTGATGAACCAACTTACGTTG
GTTGATGCTCGAGTGCTCCCAGCTCCAAGGCTTAAATACCATGACTCTGGAAGAGAGAAG
ATTTGCAACCCATCCATTGGACAATGGAATATGATTAACAAGAGAATGGTTAATGGAGGA
TCTATCAAATATTGGGCATGCATAACTTTCGCCTCTCGTTTGCATCCAAATGATATTGCA
ATGTTTTGTGAGCATCTTGTTGGCATGTGCAATAACATCGGCATGCAAATGAGTACCAGG
CCGTGTGCAGAAATCAAGAAAGCACACCAAGACAATTTAGAAGCCGAAATCAGGGGTATC
CATTTGCGTTCTGCACAAGTGCTTGCTCAACAAGGTCTAACAGATCAACAACTTGAGTTA
CTCATCATAATTTTGCCTGATATGAGTGGTTTTTACGGAAGGATAAAACGACTTTGTGAA
ACTGAGCTTGGTTTAATCACTCAGTGTTGTGCGCCTAAGAATGTAAGGAAAGGAGGAAAT
CAATATCTTGAGAATCTTTCCTTGAAAATCAACGTAAAGGTTGGTGGGAGGAACACAGTA
CTTGATGATGCTTTAAACCGGAGAATACCGCTTCTGACAGATTGTCCtacaatagtcttt
ggaGCTGATGTTACCCACCCATCTCCTGGGGAAAGTTCATCTCCATCTATTGCAGCAGTC
GTTGCATCCATGGACTGGCCACAAGTTACAAAGTACAAATGCTTGGTATCTTCACAAGGT
CATAGGGTTGAAATTATAAATGGTCTTTATACAGAAGTGAGAGATCCACAGAAAGGGAAT
GTCAGAGGTGGAATGATTAGAGATTTGCTTTTGTCGTTCCACAAGTCAACTGGTTACAAG
CCTTCGAGGATTATATTCTATCGTGATGGTGTTAGTGAGGGGCAGTTCAGCCAAGTCTTG
CTTTATGAAATGGATGCATGTGCAAGTTTACAGGAGGGGTACCAACCAAGAGTCACATTC
GTTGTTGTGCAAAAGCGGCATCATACTCGCCTGTTTCCTGAAAATCATCGTGCACGAGAC

CAGACAGACAGGAGTGGAAACATCCTGCCTGGAACTGTTGTCGATACGAAGATCTGCCAC
CCCAGCGAGTTTGATTTTTACCTTTGTAGTCATTCTGGTATTCAGGGAACAAGCCGCCCA
GCTCATTATCATGTTCTTATGGACGAAAATGGTTTCAGTGCTGATGCACTGCAAACCTTG
ACTTACAATCTTTGCTACACCTATGCCCGGTGCACTCGTTCAGTCTCTATAGTTCCTCCG
GCGTACTATGCACACCTGGGTGCGTTCCGTGCCCGTTACTACATTGAGGACGATAATTCT
GATCAGGGCTCATCAACGGGTGCAACTCGGACGTTTGATCCATCTGTCCCAGTGAAGCAA
CTCCCCAAGGTCAAGGAGTACGTTCAGCAGTTCATGTTCTATTGCTAA
>Sorghum-bicolor_Sb01g011880
ATGTCGACGCGCGACGGCTGTGGCGGCCGGCGTGGCGGCCACGAAGCAGCAGGCCGTgga
ggtggcggtggcggtggcggccGAGGGCGGGGCGGCCGTGGCGCCGCGGAGCTCGGTGGC
CGTGGCGGACAAGGGCGGGGCCGGGGCGGCGCGGAGCTTGGTGGTGGCCGAGGTGGCGCC
GTCGGTGCGGCTCGTGGCGGCGGTCGTGGAGGCGGCGAGCAGAGAGGGGGACACCAGTTC
TTCCACGGCGCCCACCAGCCGGtcggtggtggtggtgggcgaggcggcggtggcggACAT
TACCACCACCATGGCGGCGTGCCTGCTCAGCAAGGTGCTCTTCAGGGCCAGCCGCGCTGG
CAAGtggccgcgccgccgccgccgccgccggcggcggcggcgtcgagGCCGAGACCTGCG
GAAGTCGAGGCGCTGAGCGGCGAGGTGGAGAGGAAAGCGGCTGTGACGGCAGTGGCAGAG
GCAGAGGCGCGCGAGGGGCCGTCGGGCACCGCGCGTgagcctgctgccgccgCGGCGAGA
ATCGAGGAGCAGCAAGGGACGTCGCTGGGGGCGGCAGACCAGGGGGCTGCCGCTCAGGGG
AGGCTGCCTCCGGCGTCGAGCAAGGCGCTGGTGTTCCCCCCGCGGCCGGGGTACGGCAcg
ctcggccggaggtgcCGCGTGCGCGCCAACCATTTCCTCGTCCAGGTGGCGGACAAGGAG
ATCTACCACTACGACATCGTGATTACTCCAGAATCAGAGTCAAGGAAAAGAAATAGATGG
ATCGTTAACGAACTCGTTAAACTGCACAAGCAATACTTGGATGGGCGACTGCCTGTTTAT
GATGGTAGGAAGGGCCTTTTCACTGCTGGTCCTCTACCGTTCAAAGCCAAAGAATTTGTT
CTAAAACTAACCAATCCTGAGAGAGCAAACCAGGGGTATGGTGTTCTAATTTGTTTCTCA
TCATGCGCGCGCTTGTTCTTTATATGTGTTTACGTTGTAAATTTTAAGCCTTGCCTTCCT
GCCTGCAGTGAGAAAGAGTACAGGGTGACCATAAAagatgctgcaaaaataGATATGCAC
AGCCTTAGGCAGTTCTTGGCTGGTAGGCAGAGGGAACTGCCACAGGATACTATCCAAGGC
CTTGATATTGCTTTGAGGGAATGTCCAAGTGAAAAGTATGTATCCATCTCAAGATCATTT
TTCTCAAAGGCATTTGGACATGGTGGTGATATTGGCAGTGGTGTGGAATGCTGGAGGGGG
TACTACCAAAGTCTACGTGCTACACAAATGGGACTGTCCTTGAATATTGATATTTCAGCA
ACTGCATTTTACAAGGCTCAACCGATTTTGGACTTTGCATTGGAGTACCTCAATATACGT
GACACCTCAAGGCGTTTGTCTGACCAGGATCGCATAAAATTGAAGAAAGTCCTCAAAGGA
GTACGGGTTGTGGCAACACATCGTCGTGATATAGCCATACGTTACAAGATTACTGGGATA
ACCTCACTTCCCTTGAATGATTTAACGTTTGATCAAGATGGGACAAGGGTTTCAGTTGTT
CAATACTTTAAACACCAATATAATTACTGTTTGAAACACATTCACTGGCcatgccttcaa
gctggCAGTGATAGCAGGCCAACTTATTTACCCATGGAGGTTTGCAATATACTTGAAGGA
CAACGCTATTCTAGAAAGCTAAATGAGCGCCAAGTCACAAGCATCCTGAAGATGGCATGT
GAGCGACCAACTCAAAGGGAGGGTAGCATTTTAGAGGTAGTTAATAGGAACAACTACGGT
AATGATCATTGTGCCAAAGAATTTGGTATTAAGGTGACAAATGAACTTGCCTTGGTTGAT
GCGCGTGTGCTGCCAGCTCCGACACTTAAGTACCATGACTCTGGGCGAGAAAAAGtgtgt
agtccttccatTGGTCAGTGGAACATGAACAACAAGCAAATAAATGGAAGACCTTGTGTT
GATGTTGGGCAAGCTCGCCCAGATAACTTAGAAGCTGCACTCAGAAATACTCATAGGCAG
TCTGCACAAATACTTGCCCAACAAGGTGTGACTAGCCCACAGCTTGATTTACTAATTGTT
GTATTGCCAGATGCAAATGCAAGTTTCTTTTATGGAAGGATAAAGCGTCTTTGTGAAACT
GAACTTGGTATAATAACTCAGTGTTGTATACCTAAGAATGTTCATAAAGGTGGCCGACAA
TATCTCCAAAACCTGGCCCTTAAAATTAATGTTAAGGTTGGTGGTCGTAATACAGTTCTT
GAAGATGCTTTAAATAGGAGGATACATTTGTTAACAGATTTGCCAACAATAATCTTTGGA
GCTGATGTTACCCACCCAGCTCCAGGGGAGGATGCATCCCCGTCTATCGCTGCGGTTGTT
GCATCAATGGATTGGCCAGAAGTTTCAAAGTACAGGTGCTTGGTGTCTTCTCAAGGTCAT
AGGGAAGAGATCATAGCTGATCTTTTCACACAAGTGAAGGATCCACAAAAAGGACTACTT
CATGGTGGAATGATCAGGCATGCTGAACATATTAACTTTGCTGTTCTTCCAATAAATGAt
ctttccttttttttttgaagtCATATCGTTGCTGCAGGGAACTACTTGTATCATTCTAT
AGGGCAAATGGGAGCCGAAAACCGAGCAGGATAATTTTCTATCGAGATGGTGTCAGTGAG
GGGCAGTTTAGCCAGGTCTTGCTTTATGAAGTGGATGCAATTCGAAAGGCTTGTGCCAGC
TTGGAAGAGGGCTACCTTCCTCCAGTTACATTTGTTGTGGTGCAAAAGCGGCATCACACA
CGGCTTTTCCCTGAAGATCATCGTGCACAAGGACAGACAGATCGTAGTGGAAATATTTTG
CCAGGAACTGTTGTTGACACGAAGATATGTCATCCAAGTGAATTCGACTTCTACCTTTGT
AGCCACTCGGGCATTCAGGGAACGAGCCGTCCAACACACTACCATGTTCTGTTTGACGAA
AACAATTTCACCGCTGATGCACTACAGACATTGACCTACAAACTGTGCTACACGTATGCG
CGGTGCACGCGATCAGTCTCTATAGTTCCCCCAGCATACTACGCGCACCTGGCGGCTTTC
CGTGCGCGCCACTACTTGGATGATGGCCTCTCCGACCAGGGATCGTCTTCTGTGGCGAGC
TCTCGCCAGCAAGATCGCGCTGCTCCAGTGAAGCAGCTGCCGAAGGTGATGGAGAGCGTC

AAGCAGTTCATGTTCTACTGCTGA
>Sorghum-bicolor_Sb01g032060
ATGGAGGGTGAGGCGGCGGTGGCCAAGAACGAGAGGAAGGTTggcggcggaggaggaggc
gTTAGCAATGACGGTGGCGCGAATGCGAGGAGGAGGTGCAAGGGCGGCGGCCCCGGCAGG
CATCACCCGATCATCCAGGCCTACCCGGCGCTCCTGCCGTTGCCGCTACACGCCGCCGCC
CACGCGCGCCGCAGCGGCGCCGTcgcgctgccgctgccgctgccgccgcccGTGCTGGTC
TACCTgcaccagccgccgccgccgctgttGTTCCCTAAGGTGCCGGCGTGCTACGGGAAG
CCCAGTGGGCCCCCGCTGCTGAGGGGGCCGCCGTGGAGATCGAggaagccgccgccgccg
ccgcacgccgtcaccgccgcgCTCCTGCCGCTACCGCACGATACCGAGCTGATTCAGCAC
AGAAAGTGTTTCATTCATGAGAACCAAACATCTGAAATGAAAGCAAATCATCTGAGCACC
CATCACAAttcatctatcaccatgCATGGAGTTAACATTGCAACAAGACCTGATGGTGGT
GGGATTGGGGGGACTGCAATTCCTCTCTATGCAAACCATTTCCTTGTGTGTTTTGATCCT
GGGCAGAAGATTTTTCATTATGATGTCGACATATCCCCACACCCGTCAAAAGAAACAGCA
AGAATGATCAAGAACAAGCTAGTTGAAGAAAATTCAGATATCCTCTCAGGCGCCCTTCCA
GCCTTTGATGGCCGCAAGAATCTATTTAGTCCCATTCAGTTTCAACAGGACAGGCTTGAA
TTCTTTGTTAGTCTTCCAGCAGCTGCATCGACACGATTTATAGAAGCTAAAGATAATGCC
CACATGATTGACAAGCAGAATCATAAggttttcagggtgaaccttcgattggtttcAAAG
CTAAGTGGCGAGGAGTTGAACAAGTATTTGAATGAAGACAAGGATGGTATTCCTCTTCCT
CAAGAATACCTTCATGCATTGGATGTCATCCTGCGGGAAGGTGCTATGGAAAACTCTATT
CCCATGGGCCGGTCTTTGTATCCACGTTCAATGGGAGAAGCAAAGGAGATTGGTGGTGGA
GCTGTCATGTTACGAGGTTTCTTCCAGAGCTTGAGACCAACAAAGCAAGGTCTTGCCCTC
AATGTTGACCTCTCACTTACAGCTTTCCATGAAAACACGGGCATAATTGCATACTTGCAG
AAGCGCTGTGACTTTATGAAGGACCTTTCACAGGTGAAGAGTAGGGCTTTGACAGTAGAT
GAGAGGAGGGAGGTGGAAAAAGCATTGAAGAATATCCGAGTGTTCGTGTGCCACCGTGAA
ACTGACCAAAGGTACCATGTGCATGGCTTGACTGAGGAGACAACAGAGAACCTCAAGTTT
CGAGATCGCAGTGGGAAGGATTATACGGTCGTAGATTACTTCAAAGAGCACTACAACCAT
GATATTAAATTCAGGAACCTGCCCTGCTTGCAGATTGGTAAGAGCAAGCCATGCTATGTG
CCAATGGAGCTTTGCATGGTTTGTGAGGGCCAGAAGTTTCTTGGCAAGCTCTCAGATGAA
CAGACCTCCAAAATGCTCAGAATGGGCTGCCAAAGACCAAGCGAAAGAAAGGGAATCATA
AAGGGTGTTgttgaaggagcatttgGTACAAGAAGCAATTCGTATGCTGATCAATTCAAC
CTTCAAGTGTCCAAGGACATGACTCAGCTCTTGGGGAGGGTTCTCTTGCCACCAAAACTG
AAGCTTGGCAATGGGGGGCGCATCAAGGACATAACACCAGACAGATTTGACCGGCAATGG
AGTTTGATGGACAGCCATGTTGCTGAGGGTTCCAAGATCAAGAGCTGGGCCTTGATAAGT
TTTGGTGGCAGCCCAGAGCATCAATCATTCATTCCAAAGTTTATCAACCAGCTATCAAGC
CGCTGTGAGCAACTTGGGATTTTACTCAACAAGAAAACTGTCGTTAGCCCATTGTTTGAG
CGGATTCAAATCCTGAACAATGTGGGCATTTTGGAGAGCAAGCTGAAGAAAATCCAGGAA
GCCGCATCAGGCAATTTACAGCTGCTAATCTGCGTCATGGAGCGGAGGCACCGGGGCTAC
GCTGATCTGAAGCGTATTGCAGAAACATCCATTGGTGTCTTGACACAGTGTTGCCTGTAT
TCCAACCTAAGCAAGCTGAGCTTTCAGTTCTTGGCCAACTTAGCACTGAAGATAAATGCG
AAGGTTGGTGGAAGCAACGTTGCCCTCTACAACAGCTTGCCATGCCAAATTCCTAGGGTG
TTTTCAGACAAGGAGCCAGTGATGTTCATGGGTGCTGACGTGACACACCCACATCCCCTA
GATGACTCAAGCCCGTCCGTGGTCGCTGTAGTTGCGAGCATGAATTGGCCTTCAGCAAAC
AAGTACATCTCCAGGATGAGATCACAGACGCACCGTAAAGAGATCATCGAGCGCCTTGAT
GTAATGACCGGTGAACTGCTCGAGGAGTTTGTGAAAGAAGTCGGCAAGCTCCCTAGCAGA
ATCATATTCTTCAGAGATGGTGTTAGTGAGACGCTgttctacaaggtgttgACAGAGGAG
CTGCAGGCAGTGCGACTGGCATGCTCGAGGTACCCGGGCTACAAGCCAGCGATCACGTTC
GTGGTGGTTCAGAAGAGGCAGCACACCAGGCTCTTCCACAGGGAGAAGAATGGCGGCTCC
ACGCACTACGCCGACCAGAACGTACCACCGGGAACGGTGGTGGACACCGTGATCACGCAC
CCAAGGGAGTTTGATTTCTACCTGTGCAGTCACTGGGGCACCAAGGGGACGAGCAGGCCG
ACGCACTACCGCGTGCTGTGGGATGAGAACAACTTCAAGTCTGACGAGATGCAGCAGCTG
ATACACAACCTTTGCTACACGTTTGCCCGGTGCACCAAGCCTGTTTCTCTCGTCCCACCG
GCATACTACGCACACCTGGCCGCATATAGAGGAAGGCTATACCTTGAGAGATCGGACTCG
ACGGCGACCAGCCGGACAACTCTGTACAGGGCCACGCCATTGCAGACTGCACCGCTCCCT
AAGCTCAGAGATAGTgtgaaggggctcatgtTCTACTGCTGA
>Sorghum-bicolor_Sb02g005150
ATGGCTTCCCGTGGGCGCGGTTTCGGCGGAGGGCGGCAAGGCCCTGGTGGTGGGCGCGGC
AGCGAGGGCAGGGGGCGCGGGATCGGTGGCGGCGGAGGTTACCAACAACCCTACGGTCGC
GGCGGTGATGGAGGCGGTGAATCCGGAGGCCGAGGCGGCGTGGGCCGAGGGCGCGGGGTC
GGCGGTCGCGGCAACGGGGGAGGCCGCGGACCCGGCGGACGAGGTGGCGTCGGCTACCAG
cagccgccaccgccgccgctcGGGAACGTAGAGGTCGGAGGCCGGGGCCGCGGTGGCGTG
GCGGTAGCTGTACCAGTACCAGCCCGCCCCGCGGCGCCTCGTCCTCTTGCGCCGGTCGTC
GCTGCCCCGGCAGTTCCAGCAGCGGCCTCGTCTTCTGCGCCGCCGGCGCAAGCGCCTCGT

GCCCCCGCCGGGGCGGCTGGAGGCGGAGCTGCCTTGGCGGCAGGCATGGGGAGGTTGGCC
GTGGCCGACAACCCCGCTCCTCCCGCGCCTGCTGCAGGTAGATCCGAGGCGCAAGGTCCT
GCGCACcggcctccgcctccgccccTCTCGAGCAAGGGGATCACTCCGCCTGCGCGCCCC
GGGTTCGGCACGTTGGGGAGGAAGCTCATCGTTCGCGCAAACCACTTCGCTGTCCAGGTT
GCCGACAACGACATCTGCCATTATGACGTTTTAATCAATCCTGAACCAAAGGCAAGAAGA
ACCAACAGGGTGATTCTGTCAGAGCTTCTCAAGGTGCATGGTGCGACATCCCTCGCCCAT
AAGATACCTGCCTATGATGGAAGCAAGAGCCTATACACCGCAGGCGAGCTGCCATTCAAA
TCAATGGAGTTTGTTGTCAAGTTGGGGCGCCGAGAAATTGAGTACAAGGTGACAATCCGA
TATGCGGCACAGCCAAACCTGTACCATCTTCAGCAATTCCTCAAGGGTCAGCAGAGGGAC
GCCCCATATGACACAATTCAAGCATTGGATGTTGCCCTGAGAGAGTCTCCTTCTCTCAAT
TATGTCACTCTTTCTCGATCCTTCTTCTCCAAGAAGTTTGATAATGGGGTGGACATTGGT
GGTGGGCTAGAGAGCTGGAGCGGATATTACCAGAGCTTGCGCCCAACTCAAATGGGCCTC
TCATTGAACATTGATATATGCTCGACTTCATTTTACCAATCTATCCCTGTGGTAAAATTT
GTTGATGATTGTCTTGGGCTGACAAACCCTGCCCAACCTTTTTCGGACAGGGATCGTTTG
AAGCTTAAGAAAGCCCTGCGTGGAGTTCGTGTTGAGACTACACACCAGCAGGGGAAAAAA
AGCGCCTACAAGATAACTGGGATTACTCCTGTTCCATTGGCTCAGCTGAGCTTTTCTTGT
AACGAAGGACCTCAGTTGACTGTTGTCCAGTACTTTGCTGAACGGTACAACTACCGGTTG
CGCTATACTGCTTGGCCCTGCCTTCAGTCCGGCAATGATTCTAAGCCGATATATTTACCT
ATGGAGGTGTGCCAAATCATTGAAGGACAGAGGTACCCTAGGAAGCTCAGCGACACACAG
GTGACCAATATACTGAAGGCAACCTGTAAACGTCCTCAGGAGAGGGAGGGGAGCATTATT
CAGATGGTTCACCGCAACAACTATTCAGCTGATAAGATGGCACAGGTGTTTGGGATCACT
GTGGCCAACCAGATGGCTAATGTGCAAGCTCGTGTTCTGCCTGCACCTATGCTGAAATAT
CATGAATCTGGAAGGGAGAAAACGGTTGCACCAAGTTTGGGGCAATGGAATATGATTAAC
AAGAAAATGGTCAATGGTGGAACTGTTCACAGCTGGACTTGTTTGAGCTTTTCGCGGATT
CAGCTTCATATAGTAGACAGAATATGCGAGGACTTGGCTCAGATGTGCAATTCCATTGGC
ATGGATTTTAATCCAAGGCCCGTGACAGAAGTTCAGTCAGCCTCACCCAACCACATAGAA
GCTGCTTTAAGGGATGTGCACATGAGGGCTCCAAATTTGCAGCTGCTTATTGTCGTTCTT
CCAGATGTTTCTGGTCATTATGGGAAAATTAAGAGGATATGCGAGACTGACCTTGGTATA
GTATCTCAGTGCATCAATCCAAAGAAGAATAAAAACAAGCAGTATTTTGAAAATGTCGCC
CTTAAAATCAATGTGAAGGTGGGAGGGCGCAATACAGTGCTTGAGAGAGCCTTTGTGCCT
AATGGAATACCTTTTGTCTCAGATGTGccaacaatcatttttggTGCTGATGTTACCCAT
CCTACAGCAGGAGAAGATTCCTCGGCTTCTATTGCAGCTGTGGTTGCATCCATGGACTGG
CCACAAGTCACAACATATAAAGCACTAGTCTCGGCGCAAGCACATAGGGAAGAGATTATA
CAAAATCTGTTCTGGACTGGTACAGATCCAGAGAAGGGCACTCCAGTGAACGGTGGAATG
ATAAGGGAGTTGCTGACTTCATTCTTTAAGAGGACTGGACGAAAGCCCAAAAGGATTATA
TTTTACAGGGATGGTGTAAGTGAGGGACAATTCAGCCACGTTTTGCTCCATGAAATGGAC
GCAATCAGGAAGGCCTGTGCCTCTATGGAAGATGGTTATCTACCACCAGTGACATTCGTG
GTGGTACAGAAAAGGCACCACACAAGGCTCTTCCCTGAAGTTCATGGAAGGAGAGATCTT
ACTGACAAAAGCGGAAACATTCTTCCTGGAACTGTGGTTGATACTAGCATTTGTCATCCC
AGCGAGTTTGATTTCTACCTCTGTAGCCATGCTGGAATTAAGGGAACAAGCAGGCCaaca
cactatcatgtccTCTATGATGAGAACCGTTTCTCGGCTGATGCTCTGCAGTTtctcaca
aacaaccttTGCTACACATACGCACGCTGCACACGCGCTGTTTCTGTTGTTCCACCAGCC
TACTACGCTCACCTGGCAGCATTCCGCGCAAGGTACTATGACGAACAAGAGAGCACCGAT
GGAACCTCAGTTGTCAGTGGTAGCGCCGCCACAGCCGGCGGTGGCCCACCTGCGTTCCGC
AGGCTCCCCCAGATAAAGGAGAATGTGAAGGAAGTGATGTTCTTCTGCTGA
>Sorghum-bicolor_Sb02g032980
ATGGCGAGTCATCAGCGCGGAGGAGGTCGGGTtggcggcggtggcggccgGGGTCAGGCG
AACCACAACGTCTCTCAGGGCCAGGGAGGCAGGGGCTACGGCGGGCGTGGTGGCCAGTAC
TACGGCGACGACAGCGGAGGCCGTGGCGGCGGACgtggtggcggcggcagGGGCGGTGGT
CGTGGCTTcgacggccgcggcggcgggTACCAGGAAGCGCGTGGCGGGGgccgaggcggc
ggcggcggatACCAGGAAGGGGGCCGCGGGGgccgaggcggcggcggcggttACCAGGAA
GGACGTGGCGGGGGACGAGGTGGCGGCGGCTACTATGAAGGGCACGGCGGTGGCCGAGGT
GGTGGCGGCTACCAGGGCGGTGGTCgaggtggtggtggcggcTACAACGACGGCCGTGGC
GGGGGCCGAGGTGGCCGCGGCTACCAGGGACAGGGAGGCAGCGACTACGGCCGCGATCGT
GGGTTTGGAGGTCTGCAACCTCCACGACCTGATCTGCGCCAAGCCGGTCCGCCGCTCGCG
GATCGCTACGCGGCCGACGCGGCCGCGCTTAGGGAGAAGTTCAAGACGATGGACATCTAC
CGCGACGCGCCCATGTTCCCAGCGCGCCCGGGCTTCGGCGCCATGGGGACGCCGTGCGTC
GTCAGGGCCAACCACTTCTTCGTCGGCCTCGTCGACAAGGGCCTGCACCACTACGACGTg
accatttccccagagACGACGCTAAAGGGCGTGTACAGGCAAGTCATGTCGAAGCTGGTT
TCAGAGAACAGGCAGACCGAGCTTGGCGGCCGCCTACCCGCATACGATGGCAAGAAGTCA
CTGTTCACCGCCGGCGAGCTGCCCTTCAAAAGCAAGGAATTCGTGGTCACTTTGCCTGGC
AGGGTGGAGAGGAGGTACAAGGTGGTCATCAAGCATGCCACGGCGGTCAGCCTGCACCAG

CTGTTCATGCTCATGGCAGGCTACCCTACGGACATCCCCATGCAGGCGCTGCAGGTGCTC
GACATTGTGCTGCGTGACATTGTGCTCAACGAACGCAACTCCATGGAGTACGTTGCAGTT
GGCCGGTCCTTCTTCTCACCACTTGTAAAGCCAGGACCCAAGAATCTTGGCCTGGGTGTG
GAGGGATGGAATGGTTTCTATCAGAGCATCAGGCCGACACAGAAGGGCCTGTCTGTGGTC
GTAGACATGTCTTCAACAGCTTTTGTTCGACCCATGCCACTGATTGAATTTGTGATGGAG
ATTCTGAACAAAGATAGCAGGACCATTAGAAATATTACTCCCATGGAGCTTGTCAAGCTC
AAGAAAGCCCTCAGGGGTGTGAGGATTGAAGTCACACACCGAGGAGATGCACGCCGGAAG
TACCGGATTGCCAGCCTGACAACGAGTCCTCCTTCTTTACAGTTCTTTGAATCGTCCGCT
GGAGTTCAGAAGTCTGTCGCAGATTACTTCAGAGAGGCATACAATCTGGAAATGCACTAC
GATTCTCTCCCATGCCTCCAAGTTGGCAGTGATGAGAGGCCGAACTACCTCCCTATGGAG
GTTTGCAAGATAGTAGCTGGACAGCAATACCGGAAGAAGTTGGATGGCCAACAAGTCCTT
AATCTAATGGACTCAACCTGCCTGCGCCCATCTGACCGTGAGAACAACATTCGTCAGGTT
GTTGAGCAAAATGACTACAATAGAactgaacgtgcaagtgaattCGGTCTGGAGGTTGAC
TATCATCCTACTTCAGTTAATGCTAGAGTTCTGCCAGCCCCCACTCTGAAGTACCGTGGC
ACTGGATCTGAAAGTTTGTGTTGTCCAAAGGATGGGCAGTGGAACATGATTAAAAAGCAA
GTAGTACATGGTGCAAGGGTGGGCAACTGGGCCTGCGTTAACTTTTGTCATAATTTACCT
AGAGATGTTGTTGGTAAATTCTGTTCTGATCTGGTTAAGTGGTCTCGTACTACTGGAGTG
GACATggataacttgagaattCCAATATACGCTGTTCGTCCTGAACAAGTTGAAACTGAT
CTTCATAAGCTATGTCACGATGCTGGGAACAGGCTAAGAGTGCAAAAGATAGATCTTTTG
CTTGCTATACTGCCAGAGAAAAACGGCAACTTATATGGTAATTTCAAAAGGATCTGCGAG
ACAGAGATTGGTATCATGTCGCAGTGttgcctggataaaaatGTTAGAAGTGCAGGTCCT
CCATACTTTGCTAATGTTGCTATTAAGATCAATGCCAAGTTTGGAGGAAGGAACTTAGAA
TTTGCTAATCCCAAAGAAAGCTTACCGGTTGTTTCGATTGAACCAACAATTATATTTGGT
GCCGATGTCACTCACCCTGCTGCTCTGGATGATACTGCCCCTTCCATTGCTTCTGTTGTT
GCCTCCCAAGACTGGCCCACGGTGGCTaactataatggcattgCCCGTGCACAAGGTCAC
CGTAAAGAGCTCATCGATGGCCTGGAAGACATTGTCAAGGAACTCCTACTTGCATTTCAG
GAACGGTCTAAGCAGAGGCCCAAGCAGCTGATCTTCTACAGGGATGGCGTAAGTGAGGGC
CAATTCAAACAAGTGCTGGAACAAGAAATCCCAGAGATAGAGAAGGCATGGAAAGCTCTT
TACAATGAGAAGCCAAAGATCACCTTCATAGTGGTGCAGAAGAGGCACCACACAAGGCTC
TTCCCAAATGATCGCCAATGGACAGACAGGAGTGGAAATATTCTACCTGGCACTGTAGTT
GATAAGAGTATCTGCCACCCAACAGAATTTGATTTCTTCCTGTGCAGCCATGCTGGTATC
AAGGGAACAAGCCGTCCTACGCATTACCATGTGCTGCGAGATGACAATAAGTTCACTGCA
GATGCTCTGCAGTCTCTCACATATAACTTATGTTACTTGTATTCAAGCTGCACTCGCTCT
GTTTCAATCGCTCCTCCCGCATACTACGCCCACAAGCTAGCGTTCCGTGCCcgcttctac
atcaaccaAGGCTATGATACGGCGACAAGTGTCGGCTCTTTTGGTTCATCTGCTCCTCCT
GCTACTGCTGGTCCTGGCCTGAAGCCACTTCCGGAGATCAAGGGTGAACTGAAAAGGCTC
ATGTTCTACTGCTAG
>Sorghum-bicolor_Sb03g011020
ATGGAGTCTCACAATGGCGAGGCTGATGACTTGCCTCCACCACCTCCTTTGAATGCTGGT
GTTGAACCACTTAAAGCTGATGAAACAAAGGTGCCATTGAAACATAGGACTCTGGTCCAG
AGGAATGGCTTTGGCAGAAAGGGGCAGCAGATAAAACTGCTAACAAATCACTTCAAAGTT
TCTCTCATGAATGCTGCAGATTATTTCTATCATTACTACGTCAACTTGAAGTATGAAGAT
GATACACCGGTTGATCGCAAAGGGTCGGGAagaaaagttatagaaaaACTGCAGCAGACT
TATGCTGCTGAACTTgcaaataaagattttgcATATGATGGTGAGAAGAGCCTGTTCACA
ATCGGTGCTCTTCCTCAAGTTAAAAATGAGTTCACtgttgttgttgatgatgtTTCAACT
GGAAAGACTCCTGCAAATGGCAGTCCAGGAAATGACAGTCCTCCTGGAAGTGACAGGAAA
AGGATCAGAAGGCCTTACAATACAAAGACGTACAAGGTCGAACTCTCTTTTGCGGCAAGA
ATTCCTATGAGTGCAATCTCACAGGCCCTCAGAGGTCAGGAATCAGAGCACACGCAGGAA
GCAATTCGAGTGATTGACATTATTCTGAGGCAGCACTCAGCTAAGCAGGGTTGCCTATTA
GTAAGGCAATCATTCTTCCACAATAATCCTTCTAACTTTGTTGACCTGGGTGGTGGTGTA
GTGGGCTGTAGAGGGTTTCATTCTAGTTTTCGTGCAACCCAGAGTGGACTTTCACTCAAC
ATCGATGTGTCCACCACAATGATAGTGAAACCTGGTCCTGTCATTGATTTTCTGCTTGCC
AATCAGAAAGTTAAtcatccaggcatgattGATTGGGCTAAGGCCAAGCGTTCACTGAAG
AACTTGAGGATCAAAACAAGTCCAGCAAACCAAGAACAGAAGATTGTTGGTCTCAGCGAC
AGACCTTGCTGTGAGCAATTATTCACACTGAAACATAAGAATGGTAATGGAGACTCTGAA
GAGATCACTGTTTATGATTACTTCGTAAAGAACCGTGGCATAGAGCTGCAATACTCTGGT
GATCTTCCATGTATCAATGTGGGAAAACCAAAGCGGCCAACATATTTCCAGTTGAGTTA
TGCAGTCTTTTGCCTTTACAAAGGTACACTAAAGCTTTGAGCACACTTCAGAGGTCATCA
CTTGTTGAGAAATCTAGGCAGAAACCACAAGAAAGGATAGGTGTTTTGTCTGATGTACTG
CAAAGAAGCAACTATGATGCAGAGCCCATGCTGAAGGCCTGCGGGATTACAATTGCTAGA
AGTTTTACCGAAGTTGATGGTAGGATACTGCAGCCCCCCAAGCTTAAAGCTGGCAATGGA
GAAGACATTTTTACACGCAATGGTAGATGGAACTTCAACAATAAGAGGCTCATTAAGGGT

AGCAGTGTTGAGAAATGGGCAGTGGTCAACTTTTCTGCACGATGCAATGTCAGGGATCTT
GTCCGTGATCTCATCAAGTGTGGAGGCATGAAGGGGATTATGGTTGAAGCTCCTTTTGAT
GTATTTGAGGAGAATCCTTCGATGAGACGGTCGCCTGCTGTAAGAAGGGTTGAAGACAtg
tttgaacaagtcaaaACCAAGCTTCCTGGAGCTCCGAAGTTTCTTTTGTGTGTTCTAGCT
GAAAGAAAGAATTCTGATATTTATGGGCCTTGGAAGAAGAAATGCCTTGCTGAATTTGGG
ATCGTTACACAGTGCGTGGCACCAACTAGAGTCAATGATCAGTATCTTACAAATGTCCTA
CTAAAGATAAATGCAAAGCTGGGTGGCATGAATTCGTTGCTCCAAATTGAAACATCCCCA
GCAATTCCTCAAGTATCCAAGGTCCCAACTATAATCTTGGGAATGGATGTCTCCCATGGT
TCTCCTGGACATTCTGATGTACCGTCCATTGCTGCTGTTGTTAGTTCTCGTGAATGGCCT
CTTATCTCGAAATATAGAGCTTCTGTCCGCACCCAATCACCTAAGATGGAAATGATTGAC
TCTTTGTTTAAGCCACGGGAAACTGAAGATGATGGTCTGATCCGGGAGTGTCTGattgac
ttctacaccaGTTCTGGGAAGAAGAAGCCTGACCAAGTCATCATCTTCAGGGATGGTGTT
AGTGAAAGTCAGTTTAATCAGGTGCTGAACATTGAGTTGCAACAAATCATCGAGGCTTGC
AAGTTTTTGGATGAAAAATGGAATCCAAAGTTCACGTTGATTATTGCCCAGAAGAATCAC
CACACTAAATTTTTTATTCCTGGAAAGACAGAAAATGTTCCAGCTGGAACTGTTGTGGAC
AACAAAGTTTGTCATCCAAGGAACTTTGACTTCTACATGTGTTCACATGCTGGAATGATC
GGGACTACGAGGCCAACTCATTATCATATACTGCATGATGAGATAGGCTTCAATCCTGAT
GATCTGCAGGAGCTGGTGCATTCGCTCTCTTACGTGTACCAAAGGAGCACAACAGCCATA
TCAGTTGTTGCTCCCATCTGCTATGCACATCTGGCAGCAGCTCAGGTCGGCCAGTTCATT
AAGTTCGATGAGATGTCGGAGACATCCTCCAGCCATGGCGGCCATACTTCGGCGGGCAGC
GTCCCTGTCCAGGAGCTACCTCGTCTACATGAGAAAGTCAGGAGCTCGATGTTCTTTTGC
TGA
>Sorghum-bicolor_Sb04g038420
ATGGGGTCGAGGAGGGGAAGGCAACATCCTGGTGCTGGCCACGCCGCGCAGCCTCCGCAG
CCGGGCGGGAGAGGCGCTGCTCGTGCTCAGCAGCCCCCGCCGCAGCCTGCTGCaggaaga
ggaggaggaggaggtcgTGGAGGGATCCAGCAGCAGGGGCGAGGTGGAGGCCGCACGTCT
CGTGGCGGGAGTGGAGCGTGGCGCCCACGTGGTGCTGGTGCTACACCTCTGCAGGGGGCT
GCCTCGTCATCCAGCCCTTTTGCTCCCGAGCTGCGCCAAGCAATGGAGGAGGCTCCTCGT
GAGCTCGCACCACAAGCGCCACCAGTGCAGGCGGCAGGTCCTTCGCAGCCATCGCCAGAG
GCTCCCCCGCCTGTTCAGCCTAGAGAGACTGTGCCTACTGGGCCCACTGCAGGCCAGGAG
ATTATTGTGCCTACCGCACCTCCACAATCAAGCAAGTCATTCAGGTTTCCTTTGCGCCCA
GGAAAGGGCAGTATTGGCACCAGGTGCCTGGTTAAGGCcaaccatttctttgctGAGCTG
CCAGACAAGGATCTTCATCACTACGATGTCTCAATCACACCGGAAGTTACCTCAAGGGTT
GTTGGTCGAGCCATCATCAAggagctggtcaatctcTACAAGCAATCTTACTTGGGTGGG
AGGCTACCGGCCTATGATGGTAGGAAGAGCCTGTACACAGCCGGCCCACTGCCGTTTACT
TCACAGGAATTTCATATCACTTTATTTGACGATGATGGTGGCCCTGGTTCCGAGAGGCGA
CGTAGGAATTTCAAAGTAGTCATTAAATTTGCTGCACGAGCTGACCTCCACCGCCTCGAG
CTCTTTTTAGCTGGGAGGCATGCAGAAGCTCCTCAAGAGGCATTGCAGGTTCTGGATATT
GTGCTGCGGGAACTGCCTTCATCAAGGCCAAGATACGCACCATTTGGCCGGTCATTCTTT
TCGCCTGACTTGGGCCGAAggcagccccttggtgaTGGATTAGAAAGCTGGCGTGGATTC
TACCAGAGCATTCGTCCTACTCAAATGGGCTTGTCACTCAACATTGATATGTCAGCGACA
GCTTTCATTGAGCCATTGCCTGTAATCGAATTTGTTGCACAGCTGCTTAATTGTGAAATT
CACTCTAGGCCACTCTCAGACGCGGAACGGGTGAAGATCAAGAAAGCCTTGCGAGGAGTT
AAGGTGGAAGTTACTCATCGTGGAAACATGCGGAGAAAGTATCGAATATCTGGGTTAACA
ACTCAGGCGACTCGAGAGTTAACCTTTCCTGTTGATGAAGGGGGTACAATAAAGTCAGTT
GTACAATACTTTCAAGAGACATATGGATTTTCCATTCAACACACCTACCTTCCTTGCCTT
CAAGTTGGCAATCAACAGCGTCCAAATTACTTGCCAATGGAGGTCTGCAAAATAGTGGAG
GGACAGAGGTACTCCAAGAGATTAAACCAGAATCAGATCAGAGCTCTTTTGGAGGAGACA
TGCCAGCACCCACGCGATCGTGAGCGTGATATAATTCGGATGGTTAAACAGAATGCCTAT
GACAAGGATGATTatgcacaagagtttggCATTAAGATTAGCGATCGTCTGGCATCAGTT
GAGGCACGGATTTTGCCAGCTCCACGGCTTAAGTACAATGAGACTGGTCGAGAGAAGGAC
TGCTTACCTAGGGTTGGTCAGTGgaatatgatgaacaagAAAATGGTAGATGgtggtaag
gtcagaagTTGGATATGTGTCAATTTTGCTCGCAATGTGCAAGACAGTGTTGTTCGTGGG
TTCTGCCATGAACTTGCACTGATGTGCCAAGCGTCAGGAATGGATTTCGCTCGGGAGCCT
GTTCTTCCACCTCTATATGCACGTCCTGATCAAGTGGAGCGAGCTCTGAAAGCTAGGTAC
CATGATGCCATGAACGTTCTTGGACCCAAACACAAGGAACTTGATTTACTTATTGGAATA
CTACCTGACAACAATGGCTCACTTTATGGTGATTTGAAGCGTGTCTGTGAAATAGATCTT
GGGATAGTTTCACAGTGCTGTTGCACAAAGCAGGTTTTCAAAATGAACAACAAACAAATT
CTTGCAAATCTTGCTCTGAAGATTAATGTGAAGGTTGGGGGCAGGAACACCGTGCTGGTA
GATGCCGTCTCAAGGGGAATTCCTCTGGTAACTGATCGACCTACAATCATATTTGGTGCT
GATGTGACTCATCCTCATCCCGGTGAGGACAGTAGTCCCTCAATTGCTGCTGTTGTGGCC
TCCCAAGATTGGCCAGAGGTGACAAAGTATGCTGGACTAGTTTGTGCTCAAGCTCATCGG

CAAGAGTTGATAGAGGATTTGTATAAGGTCTGGCAAGATCCACAGAGAGGGACAGTCAGC
GGTGGAATGATAAGGGAGCTACTTGTATCCTTCAAAAAATCAACTGGTGAGAAGCCCcag
cgaataatattttACAGGGATGGTGTCAGTGAAGGACAATTTTATCAAGTTCTGTTGTAT
GAGCTCAATGCTATCCGAAAGGCCTGTGCCTCCCTGGAAGCGGAGTACCAACCAAAGGTG
ACTTTTGTTGTGGTTCAGAAGCGCCATCATACTAGATTATTTGCTCACAACCACAATGAT
CAGAATTCAATTGACAGGAGTGGAAACATACTCCCAGGTACTGTTGTAGATTCGAAGATC
TGTCATCCTACTGAATTTGACTTCTACTTGTGTAGCCATGCTGGCATTAAGGGCACTAGC
CGTCCAGCTCATTATCATGTcttgtgggatgaaaacAACTTCTCTGCTGACGAGTTGCAG
ActcttacaaacaacctCTGTTACACTTATGCAAGGTGCACCCGCTCTGTATCGATCGTT
CCACCAGCGTATTATGCTCACCTGGCTGCCTTCAGGGCTCGTTTTTACATGGAACCAGAG
ACTTCTGACAGTGGATCGGTGGCAAGTGGTCCTGCAGGCCGTGGACCTCAGTCAGCATCC
CATAGCACTCGGGCCCCTGGTGGTGCAGCTGTTAGGCCACTTCCAGCTCTGAAGGATAAC
GTAAAGAGGGTCATGTTCTACTGCTGA
>Sorghum-bicolor_Sb06g025560
ATGCCAGTGCCCATCATGGTGAGGAAGAAGAGAACTGGCcctggtggctctggagAAACT
TCTGGAGAGTCTTCAGGAGGTTCTGGACaaggttcttcacagcgGCCTGAGCGAACTCAA
CAACCTGGGggaggacgtggctgggtgcctcaacAGGGTGGTCGTGGTGGCGGGCAACAC
CAGGGTCGTGGTGGACATTATCAGGGCCGCGGAGGACCTGGGTCACATCACCCTGGTGGT
GGGCCCCCTGAGTATCACCCGCGTGAATACCAGGGACGTGGCGGTGAATATCAGGGACGA
GGTGGTGAGTACCAGGGACGTGGTGGCGCCCGCTCCAGAGGTGGAATGCCACAGCCATAC
TATGGTGGGCATAGGGGAGGTAATGTTGGACGAAATGTTCCTCCAGGTCCTTCTAGAACA
GTTCCCGAGCTGCACCAAGCCCCTTATGTCCAGTATCAAGCCCCGGTGGTTTCACAATCC
CCATCGGGACCTGGCTCATCCTCACAGCCTGTGGCAGAGGTGAGCTCTGGACAAGTCCAG
CAACAGTTTCAGCAACTTGCCATTCGTGGTCAGTCTTCCACGAGCCAAGAAATTCAAGTG
GCACCAGCATCAAGCAAATCGGTTCGATTCCCATTGCGCCCTGGCAAGGGCACTTATGGG
GACAGGTGCATTGTGAAGGCAAATCATTTCTTTGCTGAGCTTCCTGACAAAGACCTTCAC
CAATATGATGTATCTATAACACCTGAGGTTACTTCACGTGGTGTTAATCGTGCTGTCATG
GGAGAGCTTGTAAGACTATATAGAATTTCCCATTTGGGTGGGCGTCTACCTGCGTATGAT
GGAAGAAAGAGCCTTTATACCGCTGGACCATTGCCATTTACTTCTATGACATTTGAAATT
ACGTTGCAAGATGAGGAAGATAGTCTTGGTGGTGGCCAAGGTGGACAAAGGCGCGAGAGA
GTATTTAGGGTGGTGATCAAATTTGCGGCCCGTGCTGATCTCCACCATCTGGCTATGTTT
CTAGCTGGAAGGCAAGCGGATGCTCCTCAAGAAGCTCTTCAAGTGCTTGACATTGTACTA
CGTGAATTGCCTACTGCGAGGTATTCTCCTGTTGGTAGGTCATTTTATTCTCCCAACTTA
GGGAGACGCCAGCAACTTGGTGAGGGTCTGGAAAGTTGGCGTGGCTTTTACCAAAGCATA
AGGCCGACACAGATGGGCCTTTCACTGAATATTGATATGTCCTCTACCGCATTTATCGAG
CCTCTCCCTGTGATCGATTTTGTTGCTCAGCTTCTTAACAGAGATATCTCAGTTAGACCA
TTGTCTGATTCTGATCGTGTGAAGATCAAAAAAGCCCTAAGAGGTGTGAAGGTCGAGGTC
ACTCACAGGGGAAACATGCGCAGAAAATATCGCATTTCTGGCCTAACCTCACAAGCAACA
AGAGAGCTATCATTCCCTGTTGATGATCGCGGTACTGTGAAGACTGTGGTGCAATACTTC
ATGGAGACTTATGGTTTTAGTATCCAGCACACCCACTTTACCGTGCTTGCAAGTGGGCAAT
CAACAAAGACCAAATTATCTTCCAATGGAGGTTTGCAAAATAGTTGAAGGACAGCGCTAC
TCAAAGCGACTCAATGAGAAGCAAATCACTGCTCTACTGAAAGTGACCTGCCAGCGCCCT
CAAGAGCGTGAGCTGGACATCTTACAGACTGTGCATCACAATGCATACTATGAAGACCCA
TATGCACAGGAATTTGGCATAAGAATTGATGAACGTCTTGCAGCAGTTGAAGCTCGTGTT
CTGCCACCACCAAGGCTTAAATACCATGATAGTGGCCGAGAGAAGGATGTTTTGCCCAGA
GTTGGCCAATGGAACATGATGAATAAGAAAATGGTTAATGGTGGCAGAGTCAGCAACTGG
GCATGTATTAACTTCTCTCGGAATGTGCAAGATAGTGCTGCTAGGGGTTTCTGTCACGAG
CTTGCAGTCATGTGCCAAATATCAGGAATGGATTTTGCTCTTGAGCCTGTGCTCGCTCCA
GTGACTGCAAGACCTGAACATGTTGAAAGGGCGTTAAAGGCCCGTTATCAAGATGCAATG
AACGTATTGAGGCCACAGGGCAGGGAACTCGATCTGCTGATTGTAATACTGCCTGacaat
aatggttctcttTATGGGGATCTCAAAAGGATCTGTGAGACTGATCTTGGATTGGTCTCC
CAGTGTTGTCTGACTAAACATGTTTTTAAGATGAGCAAGCAGTATCTTGCAAATGTTGCA
CTCAAAATAAATGTTAAGGTTGGGGGAAGGAATACTGTACTTGTAGATGCTTTGACAAGG
AGAATCCCCCTTGTCAGTGACAGGCCGACCATAATATTTGGTGCTGATGTTACCCATCCA
CATCCTGGAGAAGATTCCAGTCCTTCCATTGCAGCTGTTGTTGCTTCACAAGACTGGCCT
GAGGTCACCAAATATGCTGGACTAGTGAGTGCCCAAGCTCATCGCCAGGAGCTGATACAG
GATCTTTTCAAAGTATGGCAAGACCCACAGAGAAGGACAGTAACTGGTGGCATGATAAAG
GAACTTCTCATTTCTTTCAAGAGGgcaactggacagaagcCCCAAAGGATCATATTCTAC
AGGGATGGTGTCAGTGAGggacagttctatcaagTattgttgtatgaacttGATGCCATC
AGAAAGGCCTGTGCATCCCTGGAGCCCAATTACCAGCCTCCAGTTACTTTTGTCGTGGTC
CAGAAGCGACATCACACTAGGCTGTTTGCTAATAACCACAATGACCAGCGTACAGTTGAT
AGAAGTGGAAACATACTGCCTGGCACCGTGGTTGATTCCAAGATTTGCCATCCTACGGAA

TTTGATTTCTACCTGTGTAGCCATGCCGGCATTCAGGGAACAAGCCGCCCTGCTCATTAC
CATGTCCTGTGGGATGAGAACAAGTTTACAGCTGATGAGCTGCAGACTCTGACAAACAAC
CTGTGCTACACGTACGCTAGGTGCACTCGCTCCGTGTCAATTGTGCCCCCGGCATACTAT
GCTCATCTGGCAGCCTTCCGAGCTCGCTTCTACATGGAGCCAGATACCTCTGACAGTGGG
TCAATGGCCAGTGGGGCCCGTGGGCCTCCACCAGGTGGGGCACGCGGCATCAGAGGGGCC
GGGAGTGTTGCGGTCAGGCCCCTACCCGCTCTCAAGGAAAACGTGAAGCGTGTCATGTTT
TACTGCTGA
>Sorghum-bicolor_Sb06g028510
ATGGAGCACGAGCGCGGCCGTGGCCGCGGGCGGGGACGTGGAGGACGGggaggtggtggc
ggcggcggcggcggaggaggaggacgcggAGGTGCCGGCGGGTACGGGCGGCATGGA
AGCGGCGGCGACGACCGcggcggaggcggatacGGCTCGCGCGGAGGCGAGTACGGCGGT
GGAGGCGGATACGGCCCCCGCGGTGGTGAGTACGGCGGCGGAGGCTACGGAGGGGGCAGT
GGAGGCGGTGGCTATCACCAAGGGCCTCGCGGAGGCGAGTAcggcggtggaggcggcGGA
TACGGGACCCGCGGTGGCGAGTAcggcggcggaggaggaAAAGGGGTGTACGGGCACGAC
GGACGACACTACGGAAGGGGCAATGGAGGCGGTGGCTATCACCAAGGGCCTCgcggaggc
ggaggtggaggcggCCGCGGAGGGCGCGGCCCcggcgcgggcggcggcCAGGCGTACGCG
TCCGGCGGTGGCCGCGGAGGCAACGCGTGGGCGCCGGCGCCGGGTGCTGGGAGAGGTCGT
GGAGTTggcggcggcgcggccgAGTACGCCCCCGTCAGGGGGCCCGCTCCCGCGCCTGCG
GCGAGGGCGGTTGCTGTTGCGCCCAGGGACAAGGAGGCGCCGAGTTCGTCGGGATCCGTT
GAACGCATCACATCCAGTGAATTGGCCAGAGTAGAACCACCAGCATCCACACTAGCTGCG
ACATCTTCAGTTGGCACACGAGTGCCAATGCAGAGACCTGATTCTGGAGGTTCATTATCT
CAAGCAAAGGTCAAACTTTTGGTGaatcacttcattgtcaACTACCGAGAAGTGTCAACT
ATTTTTCACTATGACATAAGCATCAAGCTTGATGAAGCTTCCCCTAAGGCTTCGGGCAAA
GAACTCTCCAAGGCAGAATTTCTTTCTGTCAAGGATGAACTCTTCAGGGAAAGCAGTTTA
CGGCGTCTTTCCTCATGTGTTGCTTATGATGGTGGAAGAAACCTCTACACTTCTGCTGAA
CTGCCAGCAGGTTTATTTCGTGTGAGAGTTCGATCAAAGACCTACATTGTATCAGTAGAT
TTGAAGAAGCAGCTGCCATTAAGTCAACTCTCAGAGTTACCTGTGCCTAGAGAGGTCTTG
CAGGGTCTTGATGTTGTTGTGCGTGAGGCCTCCAGATGGCGCAAGATTATCCTTGGTAGA
GGATTTTACTCACCAAGCAGCAGTGTAGACATTGGGCAGGGTGCTGTAGCTATGAAAGGA
ACCCAGCAGACCCTTAAATACACTCAGCAAGGGCTGATCCTATGTGTTGACTATTCAGTT
ATGCCATTTTACAAAGCTGGGCCGGTGATGGATATTGTTCAGAAATTAGTACCCCACCTT
GATTACCGGACAACACTGAACAGGAGGCAACTGGAAAATCTGATTGAGGAGCTCAAAGGC
CGACGTGTGACTGTGGTTCATCGGAGGACTAATCAGAAGTACACAGTGCAAGGCTTGACA
CCCTTACCTGCCATCCAGATGACCTTTGTGGATGCTGAATCTGGCCAAACGAAGAGGCTT
GTGGATTATTATGCTCAGAAACATGACAAGGTGATTGAGTACCAGATGCTTCCATGCTTG
GATTTGAGCAAGAGCAAGGACAAACCAAATCATGTACCAATTGAGCTCTGCACTCTTCTT
GAAGGACAGAGGTTTCCAAAAGCAAACTTGGATAAGAATTCTGACAGGATACTGAAAGGA
AAGGCTCTAATTCCTCCATCTCATCGGAGGAATGAGATTCAAGACTTGGTGAATGCTTCG
GATGGACCTTGCAGAGGAGAAATTGCACAGCAATTTGGGATTTCCTTGGATTTACGAATG
ACAGAAGTCACGGGTAGGATCCTTCCCCCACCAAATCTCAAACTTGGGGCATCCAATGGC
CACATGTCCAAATTCAGTATGGATCAGAACTGCCAGTGGAATCTTGTGAAGAAGAGACTA
GTAGAGGGCCGGGATCTTCAGTGCTGGGGCATTGTCGACTTCAGTGCTGAGCCGTCTCAC
CCCCGGCAGGAGCCCCTCAATGGAAGGATGTTTGTTGACAAGATCGTGAGGAAGTGCTGT
GAGCTTGGTATCCAAATGAACTCTAATCCTTGCTTCATacacatatcaaagatgGCAGTG
CTCTCCGATCCACATCGACTAAAGGAGGAGCTAAACAAAGCAAAACAGGCTGCAGTGAGC
AAgaagcagaggttgcagcTCCTTTTCTGCCCGATGTCCGAGCAGCACCCAGGGTACAAG
ACACTGAAGCTGATTTGTGACACACAGCTCGGGATCCTGACCCAGTGTTTCCTGAGCGAC
CGCGCAAACAAGCCAAATGGGCAGGACCAGTACATGACCAACCTTGCTCTCAAGATTAAT
GGCAAGCTTGGGGGCAGCAACGTTCAGCTGTTTGACTCGCTTCCACGGGTCGGTGGTGGG
GCACCTTTCATGTTCATCGGTGCTGACGTTAACCACCCGTCACCCGGGAACGTGGAGAGC
CCATCAATCGCAGGCGTGGTTGCATCTatcaacagcggtgccaaCAAGTATGTGTCAAGA
ATCCGTGCACAGCCACACCGCTGCGAGGTGATCCAGCAGCTGggtgagatctgcctggAg
ctcattggagtctttgTGAAGATAAATCGCGTGAAGCCACAGAAGATCATCTACTTCCGT
GACGGCGTGAGTGACGGGCAGTTTGACATGGTCCTGAACGAGGAGCTGGCTGAcctggag
aaggcaatcAAGGTGGACGGCTATGCACCTACCATCACTGTGATCGTGGCCAAGAAGCGG
CACCACACGCGGCTGTTCCCCAAGGACCAGGGCCAGCAGCAGACGAAGACTGGGAACGTG
CCGCCTGGCACGGTGGTGGACACTGGTGTGGTTGACCCGTCTGCATACGACTTCTACCTG
TGCAGCCACACCGGGCTTCTAGGGACGAGCAGGCCGACACACTACTACAGCCTGGTGGAC
GAGCACGGCTTCGGGTCTGACGACCTGcagaagctgatctacaACCTGTGCTTCGTGTTC
GCGCGGTGCACCAAGCCGGTGTCACTGGCGACGCCCGTCTACTATGCTGACCTCGTGGCG
TACCGTGGAAGGGTCTACTACGAGGCAGCCATGATGGTGTCCCAGCGAGGGATGGGGTCG
GCTTCTTCagcttcctcgacctccTCTGCTGGGACTGTTGACTTCACTAACTTCCCGAGG

TTGCACAAGGATGTGGAGGACAACATGTTCTTCATCTGA
>Sorghum-bicolor_Sb09g000530
ATGGTGAGGAAGAAAAGAACTGGTCCAGGAGAGAGTTCTGGGGAGGCTTCTGGAGCGCCT
GGGCAGGGCTCCTCACAGCGTCCTCAGGCAACTCAACAGGGTGCCCGTGGTGGAGGGCAA
CACCAGGGCCGTGGTGGATATCAGGGCCGTGGAGCGCCGCCTTCACAGCACCCAGGTGGT
GGGCTGACTGAGTATCAACCGCGCGACTACCAGGGACGCGGTGGATATCAGGGCCGTGGC
GGTCCACCTTCACAGGTTCCTGGTGGTGGGCCGCCTGAGCCTCAGCCGCGTGCCTACCAG
GGACACGGTGGATACCAGGGCCGTGGCGGGCCACCTTCACAGCATCCTGGTGGTGGGCCA
CCACCTGGGTCTCAACCACGTGACTATCAGGGACGTGGTGGTCCGCGTCCCAGAGGGGGA
ATGCCGCAGCCACACCGTGGCGGGCATGTGGGAGGTAGTGTTGGACCAAGTGTTCCTTCA
GGTCCATCTAGACCAGTTCCCGAGCTGCACCAAGCCCCAGATGTCCAACATCAAGCCCCT
GTGGTGGCAACACCATCACCACAAGGAGCTGGCTCGTCCTCGCAGCCTAGGAAGGCCGAG
GTGAGCACTGGACAAGTCCAGCAACAGCTTCAGCAACTTGTGATTCATGACCAGAGTTCA
GCCAGccaagctggtcaggtgGCACCAGCGTCAAGCAAAGCGGTTAGATTCCCATTGCGC
CCTGGCAAGGGTACGCATGGGTCCAGGTGCATCGTGAAGGCAAATCATTTCATTGCTGAG
CTGCCTAATAAAGACCTTCACCAATATGATGTATCGATAACGCCAGAGGTTACTTCACGC
GGTGTCAATCGTGCTGTCATGGGAGAGCTTGTAAACCTTTATAGACACTCCCATTTGGAT
GGGCGTCTGCCTGCGTACGATGGAAGAAAGAGTCTTTATACAGCTGGAGCATTGCCGTTT
ACTTCGAAGACATTCGAAATTACTCTGCAAGATGAGGAAGACAGTCATGGTGGAGGCCAA
AGGCGCCAGAGGGTATTTCGGGTGGTGATCAAATTTGCTGCTCGCGCTGATCTCCACCAT
CTGGCTATGTTTCTAGCTGGGAGGCAACCAGATGCTCCTCAAGAGGCTCTTCAAGTACTT
GACATTGTGCTGCGCGAATTGCCTACTGCCAGGTATTGTCCTGTTGGTAGATCATTTTAT
TCTCCCAACTTAGGGAGACGTCAGCAACTTGGTGAAGGTTTGGAAACTTGGCGTGGTTTC
TACCAAAGCATAAGGCCCACACAGATGGGTCTTTCTCTGAATATTGATATGTCCTCTACT
GCATTTATTGAGCCCCTCCCAGTGACTGAATTTGTTGCTCAGCTTCTTAACAGAGATATA
TCAGTTAGACCATTGTCTGATTCTGATCGTGTGAAGattaaaaaaGCCCTACGAGGTGTG
AAAGTCGAGGTCACACACCGTGGAAACATGCGTAGGAAATATCGGATATCTGGCCTCACT
TCACAAGCAACAAGGGAGTTATCATTCCCTATTGATGATCGTGGTACTGTTAAGACTGTG
GTGcaatacttcctggagaCTTATGGCTTCAGTATTCAGCACACCACTTTACCTTGCTTG
CAAGTGGGCAATCAGCAAAGACCAAATTATTTGCCTATGGAGGTCTGTAAGATAGTTGAG
GGACAGCGTTACTCAAAACGGCTTAATGAGAAACAGATCACTGCTCTACTGAAGGTGACT
TGCCAGCGTCCCCATGAGCGTGAGAAAGACATCTTGCAGACTGTTCATCATAACGCCTAC
TctgaggatccttatgcCCAGGAATTTGGTATAAGGATTGATGAGCGTCTTGCATCTGTT
GAAGCTCGTGTTCTGCCTCCCCCAAAGCTGAAATACCATGATAGTGGCAGAGAGAGGGAT
GTATTGCCAAGAGTTGGCCAGTGGAATATGATGAATAAGAAAATGGTCAATGGTGGTAGA
GTTAGCAGCTGGGCATGCATTAACTTCTCAAGAACTGTGCAAGATGGCGCTGCCAGGAGT
TTCTGTCATGAACTGGCTTTGATGTGCCAAGTATCAGGAATGGATTTTGCACTTGAACCT
GTGCTGCCCCCATGCTATGCGAGGCCTGAACATGTTGAAAGAGCATTAAAGGGACGCTAT
CAAGATGCCATGAACATACTCAGGCCTCAGGACCGAGAACTTGACTTGCTGATTGTAATA
CTGCCTGacaataatggttctcttTACGGGGATCTCAAAAGGATCTGTGAGACTGATCTT
GGATTGGTCTCCCAATGCTGTCTGACTAAACATGTTTTCAAGGCGAACAAGCAGCAGTAT
CTTGCAAATGTTGCCCTGAAAATAAATGTGAAGGTTGGGGGACGGAATACGGTACTTGTT
GATGCTTTGACAAGGAGAATTCCCCTTGTCAGTGATGTACCAACTATTATCTTTGGTGCT
GATGTGACCCATCCCCATCCTGGGGAagattctagtccttccATTGCAGCTGTTGTTGCT
TCTCAAGACTGGCCTGAGGTTACCAAGTATGCAGGATTAGTGAGTGCTCAAACCCATCGC
CAAGAATTGATACAGGATCTTTTCAAAGTATATCAAGATCCCCAAAGGGGATCTGTCTCT
GGTGGCATGGTCAGGGAActtctcatttctttctgGAGGTCAACTAAACAGAAACCAAAA
AGGATCATATTCTACAGGGATGGTGTCAGTGAGGGACAGTTCTACCAAGTTCTGTTGCAT
GAACTTGATGCCATTAGAAAGGCCTGTGCATCATTGGAGTCCGATTACCAGCCTCCAGTT
ACGTTTGTTGTGGTCCAGAAGCGTCATCACACTAGGttgtttgctaataatcaCAATGAC
CAACGTGCTGTTGATAAAAGTGGAAACATACTGCCTGGTACTGTGGTGGACTCAAAGATC
TGCCATCCAACTGAATTTGATTTCTACCTCTGTAGCCATGCTGGCATTCAGGGAACAAGC
CGCCCTGCCCATTATCATGTCCTGTGGGATGAGAACAAATTTACCGCGGATGGGTTGCAA
ACTCTCACCAACAACCTGTGTTACACGTATGCTAGGTGCACTCGCTCAGTATCAATCGTT
CCTCCTGCATATTATGCTCACCTGGCAGCCTTCCGAGCTCGCTTCTACATGGAGCCGGAT
ACCACTGACAGTGGGTCTATGGCGAGTGGTGCTACGACAAGCCGTGCCCCAGGAGGGGCA
CGCAACACCAGGGCTGGTGTTGGAAATGTTGCCGTGAGGCCATTACCGGCCCTCAAGGAA
AACGTGAAGCGTGTCATGTTCTATTGCTAA
>Sorghum-bicolor_Sb09g030910
ATGGGGTCTCATGATGGTGAGGATGAAGAGTTGCCACCACCCCCGCCCGTGCCACCAGAT
GTGGTTCCCATTAAAGCTGAAGATGTTGTGGGGGAACCACCAGCAAACAAGCCAATAAAG
CCAAAGAGATTACTGATGGACAGGCCTGGTATAGGAAGAAAAGGGCAGCTAGCCCAGCTC

TATTCAAATCACTTTAAAGTCGCCGTGAAGAGCACAGAAGACTTCTTCTTTCACTATTAT
GTGAACCTGAAGTATGAGGATGATCAACCTGTTGAAGGTAAAGGGATTGGCAGAAAGGTG
ATTGATAAACTGCAGCAGACATATCGCGCtgagctttccaacaagGATTTTGCATATGAC
GGAGAAAAGAGCCTGTTTACAGTTGGTGGGCTTCCACAAAAAAACAATGAGTTCACTGTC
GTCTTGGAGGACGCGTCTACTGGAAAGACTGCTGCCAATGGGAGCCCTGGCGGTAATGAC
AGTCCTGGAGGTGGTGACAggaagagagtgaggagGCCATACCAGACGAAAACTTTCAAA
GTGGAGATAAATTTTGCAGCAAAGGTTCCTATGAGTGCTATTGGTCAAGTCATTAGAGGC
GAAGAAACGGAGAACTCCCTGGAGGCACTTCGTGTTCTTGATATCATACTGAGGCAGCAT
TCCGCAGAACAAGGCTGCCTTTTGGTTAAGCAATCGTTTttctacaacaacccttcaaac
tttgttGACTTGGGTGGTGGCGTGATGGGTTGCCGTGGATTTCATTCAAGTTTCCGCGGC
ACACAGAGTGGACTTTCCCTGAACGTTGATGTCTCAACAACAATGATCGTGAAGCCTGGC
CCTGTtattgattttcttctttCTAACCAGAATGTTAATGATCCTAGCAGAATTGATTGG
CAAAAGGCCAAGCGTGCTCTCAAGGGCTTGAGGATTAGAACCACTCCTGCAAATGCAGAA
TTCAAGATTTTTGGTCTCAGCGAGAGGATTTGTAAAGAACAAACGTTTCCGCTGAGGCAG
AGAAATGGTAGCAACGGAGATTGTGATACCATTGATATAACTGTCTATGACTACTATGCG
AAGAAAGGAATTGATCTAAAGTACTCTGGTGATTTGCCTTGTATAAATACAGGGAGGGCA
AagcgtccaacatatttTCCAATCGAGCTATGCTGCCTTGTTCCgcttcaaagatacacc
aaaGCTTTGTCTACGCTACAAAGGTCATCCCTTGTTGAGAAGTCTAGACAGAAGCCTCAA
GAAAGGATGACAGTTCTAAACGATGCACTGCAACGCAGTAACTATGATTCTGACCCcatg
ttgagggcatgtgGCGTTTCAGTTGCTCCAAAATTTACCCAAGTTGAAGGAAGGATCCTT
CAAGCCCCAAAGCTGAAAGCCGGCAATGGTGATGATATCTTCTCACGAAATGGACGGTGG
AATTTCACCAATAGGAAGTTTTATCAAACCTGCTCTGTGGATAAGTGGGCGGTTGTTAAT
TTCTCTGCACGTTGTGATGTTCGGAATCTCATCCGTGACCTGATGAGGAATGCATCTGCA
AAGGGAAttCAAATGGAGGAACCTTTTGATGTGTTTGAAGAGAGCCCCTCTATGAGGCGT
GCACCCGTGTCGAGAAGAGTGGATGATATGTTTGGGCAGATAAAATCAAAACTTCCTGGA
GCTCCTAGGTTCCTCATGTGCCTTCTCCCTGAGAGGAAAAATTGCGAAGTCTATGGTCCT
TGGAAGAGAAAGTGCCTGGCCGAGTTTGGTATTGTCACACAGTGTCTAGCTCCAACAAGA
GTCAATGATCCGTACCTGCTTAATTTGCTGATGAAGATTAATGCAAAGCTTGGTGGTCTG
AATTCGTTGCTGCAAGTTGAAGCATCTCCATCAATACCCCATGTGTCGGAAGTACCCACC
ATCATCTTGGGTATGGATGTGTCTCATGGCCATCCAGGACAAGATAGGCCTTCAGTTGCA
GCCGTGGTTAGTTCTCGTCAATGGCCTCTTATCTCTAAATATAGAGCATCAGTGCACACC
CAATCCGCCAGACTAGAAATGATGTCCTCCTTGTTTAAGCCACGGGGTACTGATGATGAT
GGCCTCATTAGGGAATCACTGATTGACTTCTACACTAGCTCTGGCAAGCGAAAACCAGAC
CACATTATCATTTTCAGGGATGGAGTCAGCGAAAGTCAGTTTACCCAGGTCATCAACATT
GAACTGGATCAGATAATTGAGGCATGTAAGTTTCTTGATGAGAAGTGGTCACCCAAGTTC
ACTGTGATTGTTGCTCAAAAGAACCACCACACCAAGTTTTTCAGACCGGATCACCAGAC
AATGTTCTTCCCGGAACTGTCGTGGACAATAAAGTTTGCCATCCTAAGAACTTTGACTTC
TACATGTGTGCACATGCTGGGATGATTGGAACAACAAGGCCGACCCATTATCATGTTCTG
CACGATGAGATAGGTTTCTCTGCTGACGAAATGCAGGAGTTTGTTCATTCGCTCTCTTAT
GTGTATCAAAGGAGCACAACAGCCATCTCAGTAGTTGCTCCAATCTGCTATGCCCACCTT
GCTGCAGCCCAGGTGGGCACGTTCCTGAAATTCGAGGAGATGTCAGACACGTCCTCCAGC
CAGGGAGGGCATACCTCGGCGGGCAGTGCTCCCGTGCCGGAGCTGCCTCGCCTGCATGAG
AAAGTGAGGAGTTCCATGTTCTTCTGCTGA
>Sorghum-bicolor_Sb10g023230
atggcgccgccgccgccgccgccaccgccgcaggcgcaggcgcggcaccaccaccaccac
caccaggGGCCAGCCAACAAGGGCGGCGGCCACGCGGAGCGGACGAAGCAGCCGCTGCAG
AGCAGCGTGACGCAGCCCAAGGCGGAGCCACcggcggtggtggcggcggccgTGCTGCCG
GAGGGAGGCAAGCGGTgcggaggaggaggaggagggaggcGCCGCggcgggcgcggccgg
gcCAAGGCGCCGCCGGGTGAGTCTCGTGCTGCGCTGGCGGTAGCGGTAgcgccagcgccg
gcgcAGGCGCCTCCGCCGCGCACGGTCATTGGGCCGCCCGTGCCGAGCAAGGGGCTGTCG
TTCTGCCGCCGGCCGGGGTTCGGGACGGTGGGTGCGCGCTGCGTCGTCAAGGCTAACCAC
TTCCTCGCCGAGCTCCCGGACAAGGACCTCACCCAGTATGATGTGAAGATCACGCCGGAG
GTGAGCTCGCGGACCGTGAACCGGGCCATAATGGCGGAGCTGGTCCGCCTCTACCGCGCG
TCCGACCTCGGAATGCGACTCCCGGCCTACGACGGCCGCAAGAATCTCTACACCGCGGGC
ACCCTCCCGTTCGACTCTCGCGAGTTCGTCGTGCGCCTCAccgatgaggacgacggcaCC
GGCGTGCCGCCTCGGGAGAGGGAGTACAGGGTCGCCATCAAGTTCGCCGCGCGCGCCGAC
CTCCACCACCTCAGGCAGTTCATCGCCGGGCGGCAGGCGGACGCGCCCCAGGAGGCCCTA
CAGGTACTCGACATCGTGCTCCGCGAGCTCGCCAACCAGAGGTACGTGTCCATAGGGCGG
TCCTTCTACTCGCCGGACATCAGGAggccgcagcggctaggCGACGGCCTGCAGTCGTGG
TGTGGGTTCTACCAGAGCATCCGGCCGACACAGATGGGATTGTCGCTTAACATCGAcatg
tcgtccacggcgTTCATTGAACCCCTGCCGGTGATCGAGTTCGTGGCCCAGATATTAGGA
AAGGATGTCATATCAAGGCCATTATCCGATGCTAACCGAATCAAGATCAAGAAGGCATTG

CGAGGAGTAAAAGTTGAGGTCACTCACCGGGGGAATGTAAGGCGCAAGTATCGCATTTCT
GGGTTGACAACGCAACCAACTCATGAACTGATTTTCCCAATTGATGAGCAAATGAATATG
AAATCTGTCGTGGAGTACTTCAAGGAAATGTATGGTTTCACCATTCAGCATCCTCATCTT
CCCTGCCTTCAGGTTGGGAACCAAAAGAAGGCGAACTATCTACCGATGGAGGCATGCAAG
ATCGTTGAAGGCCAGAGATACACGAAGAGGCTGAATGAAAAacagatcacatcattgcTG
AAGGTTACATGCCAAAGGCCTAGAGAACAGGAGATGGATATTCTACAGACAGTTCATCAA
AATGGATATGAGCAAGATCCATATGCAAAGGAATTTGGGATCAACATTAGTGAGAAGCTA
ACCTCTGTTGAAGCTCGAGTCCTTCCTGCACCTTGGTTGAAGTACCATGACACTGGAAAA
GAGAAAGAGTGCTTACCACAGGTTGGTCAATGGAACATGGTAAACAAGAAAGTGATAAAT
GGATGCAAGGTGAGCCATTGGGCGTGTATAAACTTCTCAAGGAGTGTTCCAGAAAACACA
GCTAGGGGATTTTGCCAGGAATTGGCGCAAATGTGTCAGATTTCGGGCATGGAATTTAAC
AGTGAGCCAGTGATGCCATTATATTCAGCTAGACCAGACCAAGTAGTGAAGGCACTTAAA
AatgtgtataatattgcaTTGAACAAACTCAAGGGTAAAGAACTTGAACTTCTTTTGGCT
ATCCTCCCTGACAACAATGGTCCGTTATATGGTGACATCAAACGTATTTGTGAAACTGAT
TTGGGATTGATAACACAATGTTGCTTGACCAAACATGTTTTTAAGATCAGCAAGCAGTAC
TTGGCAAATGTCTCACTGAAAATTAATGTTAAGATGGGAGGAAGAAATACTGTGCTCCTG
GATGCAATAAGTTGGAGGATTCCTTTGGTCAGTGACATACCAACTATTATATTTGGTGCG
GATGTAACACATCCTGAAACTGGGGAGGACTCAAGTCCATCAATTGCTGCCGTTGTTGCT
TCTCAAGATTGGCCAGAAGTTACAAAGTATGCTGGATTGGTTTGTGCTCAGGCACACCGG
CAAGAGCTCATTCAGGACCTTTACAAAACATGGCATGATCCTCAGAGAGGCACTGTAACA
GGCGGCATGATCAGggagCTCTTAATATCCTTCAGGAAGGCCACTGGGCAGAAGCCATTG
agaataatattctacaGGGACGGTGTTAGTGAAGGCCAGTTCTACCAAGTTCTCCTTTAC
GAGTTAGATGCCATCCGTAAGGCATGTGCGTCCCTAGAACCAAATTACCAGCCTCCTGTA
ACATTTGTGGTAGTTCAAAAACGTCATCATACAAGACTTTTTGCAAATAATCACAAAGAC
AGAAGTAGCatggacaagagtggaaATATTTTGCCAGGAACCGTTGTTGATTCTAAGATA
TGCCACCCAACGGAGTTTGATTTCTACCTCTGCAGTCATGCTGGAATTCAGGGAACAAGT
AGGCCTGCTCACTACCATGTCCTCTGGGATGAGAACAATTTCACAGCAGACGAAATGCAG
ACATTGACAAACAACCTTTGCTACACTTATGCCCGGTGCACACGCTCGGTTTCTGTTGTC
CCTCCTGCATACTACGCACACCTGGCAGCATTCCGGGCTCGGTTCTACATGGAACCAGAG
ATGTCAGAGAACCAGACGTCAAAGAGCTCCAATAGCACGAACGGAGTCTCGGTGAAGCCC
CTGCCTGCTGTGAAGGAGAAGGTGAAAAGGGTGATGTTCTACTGCTGA
>Sorghum-bicolor_Sb10g031030
ATGGGATCTTGGAGACCGAAACTGCCTGGGTTGGGCGAGGGCTCTCAGGATGCTGAGCCC
GGTGGTGGTGGGAGAGGTCCTGGAAGAGGTTTCCGTGGCCGCGGTGGCTCCTaccatcaa
caactcccACAAGGTGGTCGTGGAACTGGTTACTACCAGCATGGACAGGGCTCCATGTTA
CAACCTCGTGGAGTGATGATGTCGCAGCGGTGGCAGCCTGCTGGCCCTGCTGCAGGATAT
CTGGGCCAAGGTCAAGCTTACAGAGAAGTGCAGGCACCACAGTACTATGGTGGTGGTAGA
GGTGGACGTGGGGCAGGCCCGTCCGCCATAGCTCCCGAGCTGCGCCAAGCAATGGAAACT
TCACATGAACCTGATAACATCTCACCAGAAACAGGCTCTCCAGATCTGTCACCAAGAGCT
TCTACTGTAGAAGTTACGGATCAGCTGAAAGATTTGTCTGTACAGGACGAATCAAGCATG
TGCCAAGACATTGTGCAAGCATTTCCAGTGTCGAGCAATGCATGTAAGTTTCCTCATCGC
CCAGGAAGTGGAAGTATTGGCACCAGGTGTTTGGTGAAGGCAAATCACTTCTTTGCTGAA
TTGCCTGACAAGGATCTTCATCAGTATGATGTTTCAATCACCCCGGAAGTCACATCGCGA
ATTGTGAACCGATCTGTCATGGAAGAGCTGGTGAAGCTGCACAAGATGTCATACTTGGGA
GGGCGGCTTCCAGCCTATGATGGTAGAAAGAGCCTATACACGGCTGGTCCACTGCCATTC
ATTTCAAAAGAATTTCACATCACTCTACTTGAGGAAGACGATGGTTCTGGAGTAGAGAGG
CGTAAGAAAACATACAAGGTAGTGATTAAATTTGCTGCAAGGGCTGATCTCTGTCGTCTG
GAGCAATTTTTAGCTGGAAGGCAGGCAGAGGCTCCTCAAGAAGCCTTGCAAGTTCTTGAT
ATTGTTCTGCGGGAGCTGCCAACAACAAGATATGCACCATTTGGGCGATCATTTTTCTCT
CCTGACCTGGGGAGGAGGCGATCCCTTGGTGAGGGAATAGAATGCTGGCGTGGGTTTTAC
CAGAGCATTCGGCCTACTCAAATGGGCCTGTCATTGAATATTGGTAAAGCTCTGTCCCTC
ATGGATGTATATATGTCGGCAACAGCTTTCTTTGAGCCGTTACCTGTGATAGATTTTGTT
GCACAGCTTTTAAACACCGACATCCACTCAAGGCCTCTCTCAGATGCTGAACGTGTCAAG
ATAAAGAAGGCCTTAAGAGGAGTGAAGGTGGAAGTTACCCACCGTGGTAACATGCGACGG
AAGTATCGAATAGCTGGTTTAACATCTCTGGCAACTCGGGAGTTAACTTTTCCTGTTGAT
CAAGGCGGCACATTGAAATCTGTTGTACAATATTTTCAAGAGACCTATGGCTTTGCCATC
CAGCACACCTACCTGCCCTGTCTGCAAGTCGGCAATCAGCAGCACCCAAAttaccttcca
atggagGTCTGCAAAATAGTAGAGGGACAGAGGTACTCTAAGAGATTAAACCAGGGTCAG
ATAAGAGCTCTTTTAGAGGAGACATGCCAGCGCCCACATGATCGGGAGCGTGACATTATT
CAGATGGTGAATCATAACTCTTACCATGAGGATCCTTATGCTAAAGAGTTTGGCATTAAG
ATCAGTGAGCGTTTGGCTTCAATTGAGGCACGGATTTTACCTGCTCCTCGGCTCAAGTAT
AATGAAACTGGCAGAGAGAAGGACTGCTTGCCTAGAGTTGGGCAGTGgaatatgatgaac

aagGACTTTGCCTTGGAGCCTATTCTTCCGCCTATATATGCACATCCTGATAAAGTGGAG
AGAGCTCTGAAAGCCAGGTTCCATGATGCAATGAGCATGCTCGGACCACAGCGCAAAGAA
CTCGATTTGCTTATTGGGATACTTCCTGATAACAACGGTTCTCTTTATGGTGATTTGAAG
CGTATCTGTGAAATTGACCTTGGATTAGTTTCACAGTGCTGCTGTGCAAAGCAAGTTTTT
AAGATGAACAAACAGATACTGGCAAATCTCGCTCTGAAGATAAATGTCAAGGTTGGAGGA
AGGAACACAGTGCTGGCTGATGCAGTATCAAGACGCATTCCTTTGGTGACTGACAGGCCT
ACCATCATATTTGGTGCTGATGTGACCCATCCTCATCCTGGTGAAGATAGCAGCCCTTCC
ATTGCTGCTGTTGTGGCTTCCCAAGATTGGCCTGAGGTGACAAAGTATGCAGGTTTAGTT
TCTGCTCAATCTCATAGGCAAGAGTTAATAGAGGATCTGTATAAGGTCGTACATGATCCT
CAGAAAGGAACCATTTGTGGTGGCATGATCAGGGAGCTCCTTATATCCTTCAAAAGATCA
ACTGGTCAAAAGCCTCAGAGGATACTATTCTACAGGGATGGTGTGAGTGAAGGGCAGTTC
TACCAAGTTCTACTGCATGAATTGGATGCAATCCGAAAGGCGTGTGCATCGCTGGAAGCA
AATTACCAACCACAGGTGACTTTCATCGTGGTCCAGAAACGGCACCACACGAGGTTGTTC
GCACACAACCACAATGATCAGAATTCAGTCGACAGGAGTGGGAACATACTTCCTGGTACT
GTTGTGGACTCGAAGATCTGCCACCCTACAGAGTTTGACTTCTTCCTGTGCAGCCATGCT
GGCATCAAGGGCACGAGCCGTCCTGCTCACTACCATGTcttgtgggatgaaaacAACTTC
ACAGCTGATGCATTGCAGACCCTTACCAATAACCTTTGCTACACTTACGCGAGGTGCACA
CGTTCTGTATCCATTGTCCCACCAGCATACTACGCTCATCTAGCTGCATTCCGCGCCCGG
TTCTACATGGAGCCGGACAGCTCAGACAGTGGATCACTGGCGAGTGGCTCTCGCGGAGGA
GCGCCGTCCAGCTCTTCTACATCCCGCAGTACCCGTGCCGCCACCAGTGGAGCCGTTAGG
CCCCTTCCTGTGCTCAAGGACAGTGTCAAGAACGTCATGTTCTACTGCTGA
>Selaginella-moellendorffii_15405134_locus
ATGGCCAGGCATCCACAATACTCGCAGCATTACCAAGTAGGAGGAACTAGTGGTGGAAGG
AGTGGAGGTCAGTATAGTCAATCACGTAGTGGCCGTGGAGGAGGAGGAACGGGAGGATAT
CATGGGCAGCAGCAGCCACAGCAGCAGCAACAGCCGCGACAAGCTCCAGCAGTGAGCGCC
GTGGTAGAGCAATTTTCTGGATTGGGTGTGGAAGGTCCAGTTGCTGCGCAACCAGCGCCA
CCGCAGCCGAGCGTCGTAGCTCCTCCAGCGTCTAGCAAGGCCTTGAGATTTCCGCTGAGG
CCCGGACGCGGCCAAACGGGGGTTAAGTGCATTGTCAAGGTGAATCATTTCTTTGCAGAG
CTACCAGACAAGGATCTACACCACTATGATGTGACGATCACTCCCGAGGTGACATCGCGT
GGTGTGAATCGAGCGGTCATGGAGCAGCTGGTTAAGCTTCACAGGGATTCGAGTTTGGGT
CACAGGCTCCCAGTGTATGATGGCCGGAAAAGCCTGTACACTGCTGGGCCTTTGCCCTTC
CATTATAAGGACTTTCAGGTAAGTCTGCCGGAGGAAGATGATGGATGTGGTACACCGAGG
AGAGATCGGCAGTTCAAGGTGGTCATCAAGTTCGCTGCTCGTGCTGATCTGCATCATCTT
GGCCAGTTTCTGGCCGGGAGGCAAGCAGATGCTCCTCAAGAGGCCTTGCAAGTGCTGGAC
ATAGTGCTGCGAGAGCTTCCTACCCATAGGTATTCGCCTGTTGGACGATCGTTTTATTCA
CCAGACTTAGGACGTAGACAACCCCTCGGTGACGGCTTAGAAAGCTGGCGAGGGTTCTAC
CAGAGTATAAGGCCCACGCAAATGGGTCTTTCTCTGAATATTGACATGTCATCTACTGCT
TTCATTGAGCCGCTGCCCGTGGTGGACTTTGTTGGCAAACTCCTTAACAAGGACATAAGC
AGGCCACTTTCGGACGCTGACCGCATCAAGATCAAAAAAGCTCTGAGAGGGGTCAAAGTG
GAAGTTACACATCGCGGAACCATGCGACGAAAATACAGGATTTCTGGCTTAACTTCTCAG
CCTACTCAAGAACTAATGTTTCCTGTTGATGATAGAGGTACAATGAAATCGGTAATGGAG
TATTTCCGAGATACATATCACTACACTATACGAAGCCCATCTTTACCTTGTTTGCAAGTT
GGAAATCAAGAAAGACCAAATTATCTGCCAATGGAGGTCTGCAAGATTGTGGAAGGACAA
AGGTATACTAAACGTCTCAACGAGCGTCAAGTGACAGCCCTTCTAAAAGTAACATGCCAA
CGGCCCAGGGAAAGAGAACTCGATATTTTGCAAACTGTTTATCATAATGCATATAATCAA
GACCCATACGCCCAGGAGTTTGGGATACGTATCAGTGACAGACTGGCTCTCGTGGAAGCT
AGAATACTGCCTGCCCCATGGCTGAAATATCATGAAACGGGTAGAGAAAAGGATTGCTTG
CCTCAAGACGGGACCTGGAATATGATGAACAAGAAAATGGTCGATGGAGGGACAGTGAAC
TACTGGGCATGCGTCAATTTCTCTCGTACTGTTCAAGATAATATAGCTCGGGGATTTTGT
AACGACCTTGCTCAGATGTGTCTTATTTCCGGAATGGCGTTTGCTGCTGAACCTATCATT
CCAGTTCATGCTGCACGCCCAGACCAGGTAGAACGTGCTCTGAAGTCGGTATATCGTGAA
GTTCAAAGCAAGGTCAAAGGAAAGGAATTAGAGTTACTAATTGCCATATTACCCGACAAC
AATGGCTCACTTTATGGCGACTTGAAACGAATCTGCGAAACAGACCTGGGATTGGTTTCT
CAATGCTGCTTGACAAAGCATGTCTTTAAGAGAGGAAAGCAGTATCTTGCTAATGTTGCG
TTGAAAATCAATGTCAAGGTCGGAGGCAGAAACACTGTCTTGGTGGATGCACTATCACGG
AGGCTTCCTTTAGTAAGTGATACACCAACAATTATATTTGGAGCGGATGTTACGCATCCT
CATCCTGGAGAAGATTCGAGTCCCTCGATTGCAGCGGTTGTCGCTTCTCAGGATTGGCCA
GAAGTAACGAAATACGCAGGTCTAGTCTGCGCTCAAGCTCACAGACAAGAGTTGATCCAA
GATTTGTATAAAACATGGGTTGATCCTCAGAAGGGAACCATGAATGGCGGTATGATAAGA
GAGCTTTTAATTTCTTTCCGAAGTGCCTCTGGATACAAACCTGGAAGAATCATCTTCTAC
AGAGATGGTGTGAGCGAAGGACAGTTCTACCAAGTTCTCCTTCACGAGCTGGATGCAATT
AGAAAGGCTTGCGCGTCACTCGAGCCGAACTATCAGCCTCTCGTGACGTTCGTTGTGGTC

CAGAAACGGCACCACACCCGACTATTTGCTAACGATCACGACGATACAAGAACTACTGAT
AAGAGTGGAAATATCCTGCCAGGCACTGTGGTGGATTCGAAGATTTGCCATCCAACGGAG
TTTGACTTCTACCTTTGCAGCCATGCCGGCATTCAGGGAACAAGCAGACCAGCTCACTAT
CACGTACTTTGGGACGAGAACAAGTTCACTGCCGATGGCTTGCAGTCCCTCACCAACAGC
CTGTGCTACACTTACGCTCGCTGCACACGCTCAGTCTCGATAGTACCACCTGCATACTAC
GCTCACCTGGCTGCGTTCCGCGCGAGATTCTACATGGAGCCGGACGCGTCCGAGGCTGGC
TCCGTCCACCGGAACACTGCTCCCCGAGCTGGTAATCGCCAGGATGGCTCCATCCGGCCG
CTGCCCGCGTTGAAGGACAAGGTTAAGAAGGTCATGTTCTACTGCTAG
>Selaginella-moellendorffii_15408087_locus
ATGGCCAGGCATCCACAATACTCGCAGCATTACCAAGTAGGAGGAACTAGTGGTGGAAGG
AGTGGAGGTCAGTATAGTCAATCAAGTAGTGGCCGTGGAGGAGGAGGAACGGGAGGATAT
CATGGGCAGCAGCAGCCGCGACAAGCTCCAGCAGTGAGCGCAGTGGTAGAGCAATTTTCT
GGATTGGGTGTGGAAGTTCCAGTTCCTGCGCAACCAGCGCCACCGCAGCCGAGCGTCGTA
GCTCCTCCAGCGTCTAGCAAGGCCTTGAGATTTCCGCTGAGGCCCGGACGCAGCCAAACG
GGGGTTAAGTGCATTGTCAAGGCGAATCACTTCTTTGCAGAGCTACCAGATAAGGATCTA
CACCACTATGATGTGACGATCACTCCCGAGGTGATATCGCGTGGTGTGAATCGAGCGGTC
ATGGAGCAGCTCGTTAAGTTTCACAGGGATTCGAGTTTGGGTCACAGGCTCCCAGTGTAT
GATGGCCGGAAAAGCCTGTACACTGCTGGGCCTTTACCCTTCCATTATAAGGACTTTCAG
GTAAGTCTGCCGGAGGAAGATGATGGATGTGGTACACCGAGGAGAGATCGGCAGTTCAAG
GTGGTCATCAAGTTCGCTGCTCGTGCTGATCTGCATCATCTTGGCCAGTTTCTGGCCGGG
AGGCAAGCAGATGCTCCTCAAGAGGCCTTGCAAGTGCTGGACATAGTGCTGCGAGAGCTT
CCTACCCATAGGTATTCGCCTGTTGGACGATCGTTTTATTCACCAGACTTAGGACGTACA
CAATCCCTCGGTGACGGCTTAGAAAGCTGGCGAGGGTTCTACCAGAGTATAAGGCCCACG
CAAATGGGTCTTTCTCTGAATATTGACATGTCATTTACTGCTTTCATTGAGCCGCTGCGC
GTGGTGGACTTTGTTGGCAAACTCCTTAACAAGGACGTAAGCAGGCCACTTTCGGACGCT
GACCGCATCAAGATCGAAAAAGCTCTGAGAGGGGTCAAAGTGGAAGTTACACATCGCGGA
ACCATGCGACGAAAATACAGGATTTCTGGCTTAACTTCTCAGCCTACTCAAGAACTAATG
TTTCCTGTTGATGATAGAGGTACAATGAAATCGGTAATGGAGTATTTCCGAGATACATAT
CACTACACTATACGAAGCCCATCTTTACCTTGTTTACAAGTTGGAAATCAAGAAAGACCA
AATTATCTGCCAATGGAGGTCTGCAAGATTGTGGAAGGACAAAGGTATACTAAACGTCTC
AACGAGCGTCAAGTGACAGCCCTTCTAAAAGTAACATGCCAACGGCCCAGGGAAAGAGAA
CTCGATATTTTGCAAACTGTTTATCATAATGCATATAATCAAGACCCATACGCCCAGGAG
TTTGGGATACGTATCAGTGACAGACTGGCTCTCGTGGAAGCTAGAATACTGCCTGCCCCA
CGGCTGAAATATCATGAAACGGGTAGAGAAAAGGATTGCTTGCCTCAAGACGGGACCTGG
CATATGATGAACAAGAAAATGGTCGATGGAGGGACAGTGAACTACTGGGCATGCGTCAAT
TTCTCTCGTACTGTTCAAGATAATATAGCTCGGGGATTTTGTAACGACCTTGCTCAGATG
TGTCTTATTTCCGGAATGGCGTTTGCTGCTGAACCTATCATTCCAGTTCATGCTGCACGC
CCAGACCAGGTAGAACGTGCTCTGAAGTCGGTATATCGTGAAGTTCAAAGCAAGGTCAAA
GGAAAGGAATTAGAGTTACTGATTGCCATATTACCTGACAACAATGGCTCACTTTATGGC
GACTTGAAACGAATTTGCGAAACAGACCTGGGATTGGTTTCTCAATGCTTCTTGACAAAG
CACGTCTTTAAGAGAGAGGAAAGCAGTGTCTTGCTAATGTTGCGTTGAAAATCAATGCCAAG
GTCGGAGGCAGAAACACTGTCTTGGTGGATGCACTATCTCGGAGGCTTCCTTTAGTAAGT
GATACGCCAACAATTATATTTGGAGCGGATGTTACGCATCCTCATCCTGGAGAAGATTCG
AGTCCCTCGATTGCAGCGGTAAGTGATTGGCCAGAAGTCACGAAATACGCAGGTCTAGTG
TGCGCTCAAGCTCACAGACAAGAGTTGATCCAAGATTTGTATAAAACATGGGTTGATCCT
CAGAAGGGAACCATGAATGGCGGTATGATAAGAGAGCTTTTAATTTCTTTCCGAAGTGCC
TCTGGATACAAACCTGGAAGAATCATCTTCTACAGAGATGGTGTGAGCGAAGGACAGTTC
TACCAAGTTCTCCTTCACGAGCTGGATGCAATTAGAAAGGCTTGCGCGTCACTCGAGCCG
AACTATCAGCCTCTCGTGACGTTCGTTGTGGTCCAGAAACGGCACCACACCCGGCTATTT
GCTAACGATCACGACGATACAAGAACCACTGATAAGAGTGGAAATATCCTGCCAGGCACT
GTGGTGGATTCGAAGATTTGCCATCCAACGGAGTTTGACTTCTACCTTTGCAGCCATGGC
GGCATTCAGGGAACAAGCAGACCAGCACACTATCACGTACTTTGGGACGAGAACAAGTTC
ACTGCCGATGGCTTGCAGTCCCTCACCAACAGCCTGTGCTACACTTACGCTCGCTGCACA
CGCTCAGTCTCGATAGTACCACCTGCATACTACGCTCACCTGGCTGCGTTCCGCGCGAGA
TTCTACATGGAGCCGGACGCGTCCGAGGCTGGCTCCGTCCACCGGAACACTGCTCCCCGA
GCTGGTAATTGCCAGGATGGCTCCATCCGGCCGCTGCCCGCGTTGAAGGACAAGGTTAAG
AAGGTCATGTTCTACTGCTAG
>Selaginella-moellendorffii_15412065_locus
ATGCCACCGCGGCGGGGAGGCGGGCGAGGCGGCGGACGAGGAGGAGGCGATGGACGGGCG
CCATCGCAGGAATGGCGTGGTCAAGGAGAGGAATTTCAGCAGGGAAGAGGTAGAGGTAGA
GGCGGGAGAAATCCCGACGGTCGCGGGCAAGGAGGAAGAGAGGGCGGCGGCGGGCGTGGA
GCTGGAGCTGGAGGAGGTGGTCCTTCTCAAGCTCCGATTGAGGCCTTCCCTCCACTAGGC

GGCCGTCGGCAGCAACAAATTCCTCCAGAGACCTCGCAGCGGCCGCGCCAGAGCGCTAGT
GCCGACTCGCTGGTTCCTCGAGTAAAAGATTTGGACGTGAAGGAAACTCCTGCTGGCCCG
AGTGCTCCCGTTCCTAGCAGCAGCACTGGTGATGTTCCTACCTCCTCGAAAGCACTTGCA
CCTCCTAAAAGGCCTGATCGAGGAACTGTTGGACAGAAGGTTACCATCAGAGTGAACCAC
TTCAAGATGAAGGTGAAAGATGGACCCATCTTTCACTACGACGTATCCATTCATCCGGCT
GTTGGCTCGAAAGGGATTGCTCGTGCTCTGGAGCGGCAGCTCGTGTCACAGTATCGGGCT
TCAGAGCTAAACAACCTTCTTCCCGTCTACGATGGAAGCAAGAGCTTGTACACGGCGGGG
CCACTTCCGTTTGAGCAGAAGGACTTCAAAGTTACGTTGCCGGCAGAGGAAGAAGGGCGC
AGGACCCGCGAGTTCAAAGTCACGATCAAGTTTGCCGCGAAGCTGGACCAGTATCAAATG
GACCTCTTCCTCGAAGGCAGAGGTGCGGTCCTACAAGCCCCGTATGAGTTCTTGCAAGCT
TTGGACGTGGCTTTGAGAGAGTGGCCCATGAAGTCGTACGTTCCCAGTGGACGTAACTTC
TTCGATCCGTCATTCGGGAGGCTGGCACTAGAAGGTGGTTTTGAGGCCTGGAAAGGATTT
TACCAGAGTGTTCGTCCAACCATGCAAGGCCTCGTGCTGAACGTCGATTTGTCTGCAGCA
GCATTTTACGAGGCATTGCCAGTTCTCGAGTTCCTCAAGAAGTCACTCCCGTACTTCGAC
CCAAGCCGAGGCTTGTCGGACGGTGATCGAGCCAAGGCCAAAAATCTCTTGAACCGGCTC
AAGGTCGAAGTCACGCACAGGAACATCCCTCGGAGATACAGGATTTCGGGACTGTCTTTG
CGGCCAACAAAGGCGTTGACGTTCACAACTGATTCTGGACAGGAGGTGAAGGTTGTGGAC
TACTTTTGGACGACGTACAAGCATAAGATCCAGTATCCCGAGTTGCCTTGCCTCGAGTTG
CAAGGAAGGAAAACTACTTATCTGCCTATGGAAGTGTGCAAGCTGGCTGCCGGTCAGAAA
TATCAGGGAAAGCTCAACGAGAGGCAGACAACAAACATGCTGAGGTTCACGTGTCAGATT
CCTGCTGTTCGAGAGCAGAACATAAAGACTCTTATGAGCAATGTTCAAGACTTTCAGCGG
AATGATTATGCTGCGGAGTTTGGAATTCAGGTGGCTAAGTCGATGACGTCTCTGCACGCA
AGGGTTCTTCCAACTCCGAGTCTGAGGTATAGCAGCAACCGGATCACGCCATCTGATGGC
GGCTGGAACATGATGCGGTCTAGGTTTCTTCGAGGGGGAGTAATCCGCCGCTGGACTCTC
GTCAACTTCGCGAGATTAGCAAGACAGGACGTGGATGCTTTCATCAGTGAGTTGATCACG
CGGTGCGCGGCGGTTGGAGTTCAGATGGATCCTCCCGTGATCCCTCCTTCCAGCGGTCGG
CTGGAGCAGTATGATACGCTACTCCGGAATGCCGTCAGGAATCACGCCAGCAAAAGCAAG
CCCGGGGAAGGTCTTCAGCTGGTAGTTTGTCTCATGGACGCCAAGCACCAGATTTACGGG
GACCTGAAGAAGCTGTGCGAGACGGAGCTCGGGCTCGTGACGCAAGTGTGCCTTAAGAAA
AATGTGATGAAGGAGTACAACAGTTTGTCACAGTACCTGGCCAACCTCGCGATGAAGATC
AACGTCAAAGTTGGAGGCCAGAACATGGATCTGGCGCAGGATCTGCGACTCATGGTTCCA
TCGATTCTCGGCAATCCAACAATCATCTTTGGAGCTGATGTCTCTCATCCCATGGCTCGC
GACGACACGAGTCCCTCCATCTCCGCGGTGGTGGCGAGCATGGACTGGCCATCGGCGGTC
AAGTATCTCGCTCGCGCGAGATCTCAGAGGGGCAGGGTGGAGATGATCGAGCACCTGCAT
GACATGGTGGTCGACCTCATGAGGGCGTTTTTCACTCACACCAGACTCAAACCGGAGAGG
CTTCTCTTCTTCCGGGATGGTGTGAGCGAGGGCCAGTTCTCCGACGTCCTGAACAACGAA
GTGCAGGCAATCCGGAGGGCGTTCCTTACCCTCCAGCCGAATGGCGACTACTGTCCCCAG
ATAACTTTCGTGGTCGTCCAGAAGCGTCACCACACGAGGTTCTTCCCGGCGGACAGTAAC
GTGGTGAGCAACAACGTGAGACCGGGGACCGTTGTGGACACGGAGATCACCCATCCTCGA
GAGTTCGACTTTTACCTTTGCAGCCACAGGGGTCTCCAGGGAACCAGCCGGCCGACGCAC
TACCACGTCCTGCTCGACCAGAATGGCTTCACGGCCGACCAGCTCCAGACGCTCGTCAAC
AGCTTATGCTACACTTATGCCCGGTGTACCAAGGCGGTCTCGGTGATACCTCCGGCGTAC
TACGCTCACCTGGTGGCCTACCGGTCTCGGCTCCACGTCGACAGTGTTGCCGCTGGAGCT
GGATCTTCTTCTGCTCGCGCTGCGGCCGCGGAGTATAGGCTGCCTGAGGTACTTCCTGAA
GTCCGGGACTACATGTACTACTGCTGA
>Selaginella-moellendorffii_15414178_locus
CTCGCGCGCCGGCCGGGCTATGGCACCGCGGGGACGGGGACGACGGTGCTGGCCAATAAT
TTTTGGGTGCGCTTCGACAAGACGAGGAATGTTTTCCACTACGATGTGGTTGTGACCCCC
GATGTTCCCGCGGGATTCAAGCGAGCCATCATCCAGGCCATGATCGAGCAGTACAAGAAT
GAGAAGATCCAGTGCTTGCCGGTCTACGATGGAGAGAAGAGCCTCTACACGGCGAAATCG
ATCGGCGATAGCCTAGAGCTGGACGTGAAGCTCGGGGATGTCGACGATACAATGTATGTT
GCCAGGAGAGACACAGACTTTCGTGTCACACTGCGGCTCGCCTCGCGGCCGAATTTGGGC
TCGCTCGCGGATTTTCTAGGCGGGAGAACCACTGTTTGCCCACACGACACGATCCAAGTC
ATGGATCTCGTCTTGAGGGAGTGTGCTACGAACTCGAAGAACCTTGTTCCTGGTGGTCGG
TCTTTCTTCTCCCCTAACCTGGGAAACAAGGATCTTGGAGGCGGCCTCGTCGCGTGGCAA
GGCTTCTATATGTCCATCCGTCCAGCACAGGACAATTTGCTCGTCCTTAACATTGACATG
ACCGGGAATGCGTTTATCAAGGAGGGGCAGACTCTGGTGGAGTTTGTGAGCAGGTCTTTT
GGTGCCGATCCTCGCGATCTGGATCGTAACATGCGTCGGCAGGATGCACAGGGCGACACT
TACCGCGTCAAGATGAAGAAGCTGGTGAAAGGCCTCAAGGTGGAAACAAGTCACTGCAAG
ACCAAACGAAAGCTCAAGATTGTGAGCTTGACGCGACAGCCACTGGAAACTTTGAACTTT
AATATGAATGGTATGCAAGTTTCCGTAGTCGATTATTTTCGGCAAACTTATGGTTTAAAC
CTGGCATTTGGGGGGTTTCCTGCTGTGGAGCAAGGATCCGGTGATCGGAAAAAGTATATC

CCGCTCGAGCTCTGCCGACTCGTTAAGGGCCAGAATTTTACCAGAAGGGTGAATGACGAC
CAGAGAAAGGGGCTCTCTGCAATGACATGCTGCCTACCTGAGCAACGCGTGAACGCCACA
CAACAGGCCTGCTTAAATCTGAAAAAACAAAGCGAAGATCACGCCAAAGAGTTCGGAGTG
GAAATCAATCCTAACTGGACTAGAGTTCCAGCTCGAGTTCTTAATCCTCCAAAGGTGAAG
TATGGAAATGGTGAAATTTGTCCCCGCGATGGGACGTGGAACATGATAAACAAGAAAATG
GTCGAAGGGCGGGAGATCAAACATTGGGGCATTATTAGCTGCTCTAACCGTGTGAGAGAA
AATGACCTTCAAAGGATAGCACAGCAGCTCTCGTCAGCATGTCTTTCTTATGGAGGCTTG
GACGTGAATGTCGTAATGCCTCCGCTGGTGGTGACTGTTCCACAGAGGGTGGACGAAACG
ATACGGTCTTCCGTGAAAAAACTTGCGGAGCGGAATATCGAGCTCCAGCTGCTCGTGTGC
ATTCTACCAGATCTGAACACTTCTTCCTTCTTCACAGTGACCATCAAGCGCTTATGCGAG
CTTGAACTTGGTGTCATAACTCAATGCGCGCAGGAGGGTAAAATTAGGAAGTGTGATCCT
CGCTACCTCGCAAACCTTATTTTAAAAATCAATGCCAAGTTCGGAGGGAAAAACGCAGTT
ATATGTGCGCAAGATCTGAAGAAATGCAAGCCGGTAGCAGACTCGCCAACTCTGATAATC
GGTGCGGATGTTTCTCATCCTCGTGCTGGGGAGGAAACAGGGTGCTCAATGGCAGCGGTT
GTGGCGAGTATGGATTGGCCCGGGTTTGCGCAGTATGCGACGGTCGTGAGAAGTCAGCCT
TCGCGACAGGAGATGTTGGACGACCTGTTCTGGGAGAACGTTGACGAGCAGGGCCGTAGT
GTCAGTGGTGGCATTTTCAAGGAGATGTTGATGGCATTCCATCACCGGACGAACTTTATC
CCTGAGCGTATAATTTATTACAGAGACGGAGTAAGCGAAGGACAGTTCGAGGCTGTGCTT
CGTAGCGAATACGAGTCGCTTCAGCGACAGGCCGAGAAGCGGTCCAAGCAACCATCAAAG
GGTCCCAAGATCACGTTCATAGTGGTGCAAAAGAGGCACCATACCTGTTTTTTCCCGGCC
ACGAAGCCGACTGGGAAAAACCAGAACATCAGCCCGGGGACAATCGTCGATAAAGTCGTG
TGCCATCCGACAAACTTCGACTTCTACCTTTGCAGCCACCAGGGAATCAAGGGGACTAGC
CGGCCCGTGCACTACCACGTCCTCAAAGACGAGAACGGGTTTACTGCTAACGAGATCCAG
CAGTTCACGCACGACCTCTGCTACCTCTACTCTCGCTGCACCCGAGCTGTTTCGTATGTT
CCACCGTGCTATTACGCTCACTTGGCAGCACAGCGAGCGCAGGCTTGGGTGGATCCCGAG
>Selaginella-moellendorffii_15415029_locus
ATGAGGAGGCCGGGTTATGGCCAACAGGGAACGCCTGTCCGCCTCACCTGTAACCATTTT
GCCGTGACGCTCTCTCGAGGAATGGATGTAGCGCAATACAACGTGAGTATATCATATGCT
GATGATCCCAACGACAAGGTTCTCGTCGAGAAAGGGGCGAACCGCCGAGTAATGGACAAG
GTCCGGGCCGAGCTTGGAAAAAAACTCATTTTTGACGGAGAGAATACGGCGTACGTGCTC
GGCGATCTCTCTTTCGGCGACAAGGAAATGGAAGTCACGCTCGACAAAGCAATGGGAGCA
TCTTCCTCGTCTGGGGGCCCCGCGAAAAAGAGAAGAGCGGATGCCAGCTCTTACATGGTG
AGGATCAAGTTCTCAACCAAAGTGGACTTGGGCATCCTTATGAGGAAGGAAGATCTTCAT
TTGTCTCGGGCGCAAGATGCATTGAGGGTCCTTGACGTTCTTGTTCGCGAACAAGCCGCC
AGAAGGGAGTACGTTTTACTGCGTGAGAGCTACTTTCATCAGTCCCTTGGTCCAGTAAAA
GATGTTGGAGAAGGGGTGGAAAGCTGGAGTGGCTACCACGCTAGCTTCCGACCTTGTTCT
CTGGGATTATCACTGAACTTGGCAGATCCGTCGACAACAATCGTCATCAAGCCCCAGTTG
GTGCACGAATTTCTTGCGGAGTATTTCGATACATCTCCAGGAGGCATTCGTGCCGATCAC
TTGACCCGGGCGAAGAGAGTGCTGAAGGGAATCGTGGTGCAAGTTTACACTAAGACGAGG
CACAAAATATTCGGTTTTAGCGATGAACCAGCATCCTCTCAAAGGTTTGAATTGAAAGAG
AAAGGAGCCGATGGCACGTTTAGACTGAATTCAACGACAGTTCTTCAGTACTATCAAGCT
AGATACAACGAAACCTTGCAATTCCCCAATTTGCACTGTGTCAACGTAGGGAAAGCCACG
CGAGCAGTTTATGTACCGATGGAGTTCTGTTCGATTCTGCCTGGACAACGGTATAAAAGA
AAACTGAGCGGGAATCAGATTCGCAGGCACCTTGACCAAGCACGACTGCTGCCGAGTGAT
CGTGCTAATGTGATCAACTCGGGCATTACGCAACTACTGTCAAATTCAAGCGTGGAATTG
CAGTCCCTCAACGTCAAGGTTGACAGCAAAATGATGTCAGTTCAAGGACGTGTTCTTCCG
GCGCCATTGCTCAAGTTTGGACACCGGGACGTACCAGTCCAGGCTGGGAGGTGGAACTAC
AACCGCGATGTAAGCCAACCTGCCCTTCCTGTCAAGGAGTGGATTGTTGTTTGCTTTAAC
CGCAGAAAGAATCCTTTCAGCCATCAAGACGTTTCTCGGATTGCTAACCAGTTGAAGGAA
TGTTGCGTGCAGAAGGGCATGGCTGTTGAGAATCCCGGTCTAGTGTTGGTAGAAGATCCA
AGCTTCTCTGAGCATCCAGGTTGGGAAAGGGTGGATATGATGGTTGCCAAGATGCGGAAG
AATAATCCTAATCCCGGTCGTCGTCCGGGGTTCGTTCTCTGTCTTCTTCCGAGCAAGGAA
TCGGATGCCTACGCGCCATTCAAACGACTCTTTTTGACGAAAGAAGGAATTCCGAACCAG
TGCATAGCGCCCCAGAGGAATCCCAACAATCAATACCTGACGAACGTCGTTTTGAAGATG
AATGCAAAGTTAGGGGGCTACAACACGGTTTTAACTAGTGAATTTAAGAAAGAATTGCCA
AAGCTTTCATATGCTCAAACCATGATCTTGGGAATGGACGTGTCGCATGGATCTCCATTT
TCCCACACCCCGTCGGTCGCGGCTATGGTTGGCTCTTTTGACTGGCCAAGGATTACGAGG
TACTCCGCTCGCGTGATGGCGCAATCGGCAAAACAGGAAGCGTTCGCCAACATTCCATCG
ATGCTGGAGAGCCTCCTGAAGAACTTTAAGAACTTCCAGGGCGAGAAGGGTTGCTACCCT
CAGCAATTGATTGTGTTCAGAGATGGAGTGAGTGAATCCCAGTTTGAGAGCGTCCTCACC
GGGGAACTGCAAGACATCATCAAGACTTGCGAAGGTCTCGGCATCAGGCCGAAGATAACT
CTGGTTGTAGCACAGAAAAGGCACCATACGCGTTTCTTGCCGGTGGGGCAGCAGAAAAAG

AATGTCGAACCTGGGACCGTTGTGGACCGCGACGTCGCACATCCTACGAATTTCGACTTC
TTCCTGTGCAGTCACTTCGGCATGCTCGGAACAAGCAGGCCGACACACTACATCGTGCTC
TACGACGAGATTGGTTTCACGCCGGACGAGATCCAAATGACCATCAACAATCTGTGCTAC
ACCTACGTCAAGAGCACGACAGCAGTATCAGTGGTTGCGCCGATCAACTACGCGCATCTG
GCCGCGAAGAAAATGAAGAACTTTATGTCGCTGGACGGCTCCGAGACGGGGTCACTTTCA
TCCGCCGCTACCAGAGAGTCAGCACCGCCGGCCCCGGTTTTGCCAGAGCTCCAGGGCAAC
GTCGCCAACACTATGTTCTTCGTCTAG
>Vitis-vinifera_GSVIVT00000553001
ATGGCAACTATTCCCAGCATGAAACGACCTCTCCTTTGTAGTTCTCTTCTCTTTCTTCTT
TTCTTCTGTCATCACTCTTCTTCCCAAAAGGACTGTCCAAACTGTGGCTCCATACAGGTC
CCATATCCTCTAAGCACAAACCCCAACTGTGGTGACCCAGACTATTCACTCCGCTGTGAT
GGCGACTCCCAGAAACTCTACTTTGATGGCCTTAATGGGAGCTCTTATCTTGTTCTCAGA
ATCATGGCTTCATCTCAAAGGATGGTGGTGCAACCATCACCATGGCTGCCTGGCACATGT
GTTACTCAAGACATGCCAGTGAGTGAGGGCCTCTGGTTAAACCAGACACGCCCCTTCAAG
ATCACCTCATCCAACACAATCTTCCTCTTCAACTGTTCGCCTCGCCTCTTGGTCTCACCT
CTCAATTGCACACCTTCTAGTCTTTGCCACCACTACCTGGAGAGCTCAGGTCATGTTGAC
AGAAAGCGTGCTCTTCAGTGTGCAAGTGGTCTTGATCCCTGCTGCACTTTCGTGGCAGGT
GGTATGCCTTCTGCATACAAGATTCGACTTCACAGTTCGGGTTGTAGAGCATTTAGAAGC
ATCCTTGGTTTGGATCCAGAAAAACCTCCAAGTCAATGGGAAGAAGGATTAGAGATTCAG
TGGGCTCCTGCTCCAGAACCAGTTTGTAAAACACAACTTGATTGCACCAGGGATTCCAAA
TGTTCTCCTGCTGGTGGAAAAGGTCTCTTGCGCTGCCTCTGCAATAGGGGTTACTACTGG
GACCTTGCTCGGGGTACTTGCCTGAAAAAGGAGAAAAACTCCAAATTGGCCATTAGCCTG
AAGGTTTCAATAGGAGTAGTTTCCTTTTTCTCTCTTGCTGTAGCAATTGCTGCAGTCACA
GTGAGGAGATCTGGGAAATTTTCTAACCAGGAAAAGCTCGTCAAGGCAAGAGAAGAAATG
TTGAAGTCAAGCATGGGTGGGAAATCTGCAAGGATGTTTTCCCTGAAAGAGGTAAAGAAA
GCAACAAATGGGTTTTCTAAAGACAGGGTTTTGGGAAGCGGGGGCTTTGGAGAAGTATAC
AAGGGTGAGCTTCATGATGGCACCATTGTGGCTGTCAAGTCAGCCAAAGTTGGCAACCTC
AAAAGCACTCAGCAAGTACTGAATGAAGTTGGGATCCTTTCTCAAGTCAACCACAAGAAC
CTGGTTAAACTACTGGGTTGTTGCGTGGAAGCTGAGCAGCCACTGATGATCTACAATTAC
ATTCCAAACGGGACCCTCCATGAACATTTGCATGGTAAGCGTTCCACTTTTCTGAAATGG
GATACCAGGCTGCGAATTGCTCTACAAACTGCTGAAGCATTGGCTTATCTGCACTCTGCT
GCACACACCCCCATCTACCACAGGGACGTGAAGTCAACAAATATACTTCTAGATGAAGAT
TTCAATGCAAAGGTGGCAGATTTTGGGCTATCCAGATTGGCTGAGCCAGGGCTAAGTCAT
GTGTCAACTTGTGCTCAGGGAACACTCGGGTACTTGGACCCTGAGTACTATCGCAACTAC
CAATTAACAGATAAAAGCGATGTTTACAGTTATGGGATTGTGATGCTTGAACTGCTGACT
TCTCAGAAGGCAATCGACTTCTCCCGGGAGCCAGACGATATAAATCTAGCAATTTATGTG
AGCCAACGAGCCAGTGATGGTGCAGTAATGGGAGTTGTGGACCAGCGGTTGCTTGGACAT
AATCCTTCTGTTGAGGTGATAACAAGCATAAGGCTCTTCTCAGAGCTTGCCCTTGCCTGT
CTGAGGGAGAAGAAGGGGGAGAGGCCTAGCATGAAGGCCGTGGTTCAAGAACTTCAACGC
ATAATCAAATTTGTGGATAAAGAAGAGGTTTTTAGTGAGGTTTCAATAACTCCTGAGGTG
ACATCTCGAGTCCTTAGCCGGAGCTTGATCAAAGAACTTGTTCATCTGTATGGACAGTCA
CACCTTTATCGAAATCCTGCTTATGATGGAAGGAGGGGCATTTATACAGCAGGTCCTTTA
CCATTTACCTCAAAGGAATTCATGATTAAGTTGGAAGAGGGAAATGATGGAACTCATGAA
AGAAAAAAGAAGGAATTCATAGTGAAAATTAGATTTGCTACCAGCACAGACATTCACAAT
CTGCGAGAGTTCTTGCTCAGCAGACAAAGTAATGTGCCATATGAAATCATCCATGCTCTT
GATGTGGTTCTTAAGGATTCTCTATCAAACAACAGATGCACTCTGTCGGGGAAGACATTT
TTCCCACTTGGTTTAGGAGCAAGAAGTGAGATTGGCAATGGGGTACAATGCTGGAATGGA
TTCTACCAGAGTCTTCGGCCCACCCAGATGGGATTGTCCCTGAATATTGATGTGTCCTCC
AAATCCTTTTACGAACCAATTCCTGTCATCGAATTTGCGGCTAAATTTCTAAATTTGGAG
GATCCATCAATAATGGCCCGAATGCCTCTGTCTAATGATGATCGTTTAAAGTTGAAGAAA
GTTCTGAAAGGAATCAAGGTGGAAGTCACTCATGGGGGACAAAGGCGTTACAAAATTTTT
GACATAACAGAGCAACCAACAAACCAACTAAGGTTTACTGAAGATGGACAACAGAAGTCA
GTGATTCAGTATTTTCGTGAGAAATACAATATTGTCCTTCGTTATGCCTCTTGGCCTTCA
CTTCGATCAGGGAAGGATTCAAGACCCATTTATTTACCCATGGAGACATGCACAATTGTT
GCAGGGCAGCGTTATGCTAAGAAGTTGAATGAGAGGCAGGTTGCGTCCATGTTGAGAATG
ACATGCCAGCGGCCCTGGCGGAGACAAGAGATTATACATCAGATTGCTGATCAAGATGAT
TATATAAGGAATGATTTTGTCAAGGAGTTTGGAGTTAATGTGAGTGTGGATATGGCAGCC
ATTGATGCCAGAGTTCTCCCACCCCCTGCTCTCAAGTATCATGATTCTGGAAGAGAGAAA
ACTATTAGACCACGCACAGGACAGTGGAATGCGCAGCATGTGAAATTATATCATGGTGCT
GTAGTGGAGTACTGGATGTGTGTAAACTTCTCTAATTTAAAACAAGAAGTGGTTTTCAAT
TTTTGTCAGCATTTGGTAGACATGTGCTGCCGCAAAGGAATGGATTTTGCTCGTAACCCT
TTGTTTCCTATTCAATCAAGTCCACCTGGGCAGATAGAGGCAAAGCTCTCAGATGTTCAC

CACCAGTGCAGAGTTGAGGGAAAGCAACTCCAAATGTTGATTATTATTCTCCCCGAAGTC
AATGCATACTATGGGAAAATCAAAAGAATATGTGAAACAGAGCTTGGAATGGTTTCTCAG
TGCTGTCAGCCCAGACATGCTAGAACTTGTAATCGGATTTATCTTGAAAATATCGTCTTG
AAGATCAATGTGAAGNNNNNNNNNTCAAAATGCTATATTGGAAGACACTCTGTATGGAAGA
ATACCTCTTCTGACTGACATTCCTACTATTATATTTGGTGCTGATGTTACCCATCCACAA
TCAGGGGAAGATCAAGGCCCTTCAATAGCAGCAGTGGTGGCATCAATGGACTGGCCAACA
GTGGTTACATACAGGGGCCTGGTTTCTGCACAGCCTCATCGATCAGAAATTATCGAGGAC
CTCTTCAGAGTTAAAGAAGATCCAAAGAGAGGCGTAGTTCATGCAGGAATGATAAGGGAA
CTCTTACTTGCTTTCAAGAGTTCAACTGGCCTCAAACCTTTGAGGATTATCTTCTTCAGA
GATGGCGTTAGTGAGGGCATGTTTGAAATGGTGCTGCTGAAAGAAATGGATGCCATCCGA
AAGGCTTGTGCATCTCTAGAGGAAGGATATCTCCCGCCGGTCACTTTTATTGTAGTGCAG
AAAAGGCACAACACCCGGCTGTTTCCTACAAATGAAGACAATATGGATAAGAGTGGAAAC
ATACTCCCTGGAACTGTGGTGGATACGGTGATCTGCCATCCATCTGAGCATGACTTTTAC
CTGTGCAGCCATGCAGGAATTCGGTACCAGCAGACCTGCGCACTATCGTCTGATGCATTG
CAAATGCTGGCAAACGATCTTTGCTACACGTATGCAAGGTGCACCCGGTCTGTTTCCATA
GTGCCTCCTGTTTATTATGCACACTTGGCGGCATTCAGGGCGAAGTTCTACGTGGAAAGA
AGCGGTGCCCAATATGAAGGTGGATCAAGCACAGGGCCTGATGATAGAATAGAGCTGCCG
GAGATAGATCCAACTGTGAAAAGCGTCATGTTCTATTGCTAA
>Vitis-vinifera_GSVIVT00000886001
ATGGTTAGGAAGAGGAGAACTGAACTTCCCACTGCCAGCAGTGGGGAGAGCTCGGAGTCC
ACTCAGGAAGCTAGTGGAGCTAGTGGAGGTAGTGGAGGAGGCCGAGGTTCTCAGCAGCCT
GCAGCAACACCACAACAAGGAGCTACTGGGATGGGATTTCAAGGGGGTAGAGGTTGGGCT
CCTCAATCACAACAAAGCGGGCGCGGTGGGTATGCAGGTGGCCGGGGTCCTCAGCGTGGT
GGAATGGCTCCACAACAGCAATACACTGTCCCGACTGAATATCAAGGTCGGGGTCGTGGA
GGAGGAGCACCACCTCAGCAACCTCCTGCAGCAGCTGCTGCTTATGAGTCTGGAAGCAGA
AGCCGAGCTCGTGTGGGTGGTGGCCGTGGGGTAGAGCCAGTGTCTTCTGGTGGCCCTCCT
TCTAAGCCGTTAAGTTCCGATCTGCACCAAGCTACGCAGGCGAGTTATGCAGCCGGGGGA
ACTCCTCATCGCGTTCCCTCTGAAGCCAGTTCATCACGTCAGGCTGCTGAGTCTTTGACA
CAGCAGCTACAGAAGGTTTCTATCCAGCAGGAGGTGCCTCCCAGCCAGGCAATTCAGCCT
GTCGCTCCCTCCAGCAAATCAATGAGGTTTCCTCTTCGACCAGGCAAGGGAGTTACTGGT
AAGAAGTGTATAGTCAAGGCTAATCACTTCTTTGCTGAACTTCCAGATAAAGATTTACAC
CAGTATGATGTGTCAATTAATCCGGAGGTTACATCAAGGGGGGTGAACCGTGCTGTGATG
GAACAGCTGGTGAAATTGTACCGGGAGTCACATCTTGGAAAGCGACTCCCTGCCTACGAT
GGAAGGAAAAGTCTGTATACTGCTGGGCCGCTTCCTTTCATTTCAAAAGAGTTTATGATA
ACTCTCATAGATGAAGATGATGGAACAGGCGCCCCCAGGAGAGAGAGAGGGAATTCAAAGTT
GTGATCAAACTGGCTGCACGTGCTGATCTACACCATTTAGGACTGTTCTTACAGGGGAGG
CAAGCTGATGCTCCCCAAGAAGCTCTTCAGGTTCTTGACATTGTTTTGCGCGAGTTGCCT
ACAACTAGGTACTGTCCCGTGGGGCGATCATTTTATTCTCCTGATCTAGGGAGAAGGCAG
CCATTGGGTGAGGGTTTGGAAAGTTGGCGTGGTTTCTATCAGAGTATTCGGCCAACTCAG
ATGGGACTGTCACTGAATATTGATATGTCCTCGACTGCTTTTATTGAGCCATTGCCTGTC
ATTGATTTTGTGACACAGCTTCTGAATCGGGATGTTTCTTCCCGACCATTATCTGATGCT
GATCGTGTAAAGATTAAGAAGGCTCTCAGAGGAGTCAAAGTTGAAGTTACTCATCGGGGA
AATATGCGCAGAAAATATCGTATATCTGGCTTAACATCACAAGCAACACGTGAATTGACT
TTTCCTGTTGATGATAGAGGAACCATGAAATCTGTCGTTGAGTACTTCTATGAAACATAT
GGGTTTGTCATTCAGCATTCTCAATGGCCTTGTCTGCAAGTGGGAAATCAACAGAGGCCA
AATTATTTGCCTATGGAGGTTTGCAAGATTGTTGAAGGCCAAAGATATTCCAAGAGGTTG
AATGAGAGGCAGATTACTGCTTTACTGAAGGTGACTTGTCAGCGTCCTCAAGAAAGGGAG
CATGACATAATGCAGACGGTTCACCATAATGCTTATCATGAGGATCCTTATGCTAAGGAG
TTTGGTATCAAAATCAGTGAGAAGCTCGCTTCAGTTGAAGCTCGCATTTTACCTGCTCCA
TGGCTTAAATACCATGATACGGGAAGGGAAAAGGATTGTCTGCCTCAAGTTGGCCAGTGG
AATATGATGAATAAGAAAATGGTCAATGGTGGAACTGTCAACAATTGGATTTGCATAAAT
TTTTCACGTGGAGTACAAGAGAGTGTGGCTCGTGGGTTCTGTCAGGAGCTTGCACAAATG
TGTTATATTTCTGGCATGGCATTTAATCCGGAACCAGTACTTCCACCAATTACTGCACGT
CCTGATCAGGTGGAGAGAGTTTTGAAGGCAAGGTTTCATGAAGCCATGACCAAACTCCAG
CCACAGGGCAAGGAGCTTGATTTGCTTATTGTTATTTTGCCAGATAATAATGGTTCACTC
TATGGTGATTTGAAACGAATCTGTGAGACAGATCTTGGGCTTGTTTCACAGTGTTGCTTA
CATAAACATGTGTATAGAATGAGTAAGCAATATCTGGCAAATGTGGCATTGAAGATTAAT
GTAAAGGTTGGAGGGAGAAATACGGTGCTTGTTGATGCAATATCAAGGCGCATACCTCTG
GTCAGTGATCGGCCTACTATCATTTTTGGGGCTGATGTTACCCATCCTCACCCAGGAGAG
GATTCAAGCCCATCTATTGCTGCTGTTGTCGCCTCTCAAGATTGGCCCGAGATTACAAAA
TATGCTGGTTTGGTTTGTGCTCAGGCCCATCGACAGGAGCTCATCCAAGATCTTTATAAG
ACTTGGCAGGATCCTGTAAGAGGAACTGTGTCTGGTGGAATGATCAAAGAACTGCTTATA

TCTTTCCGTAGAGCAACTGGGCAGAAACCTCAGCGCATAATATTCTATAGGGATGGGGTC
AGTGAAGGGCAGTTCTATCAAGTCTTACTATATGAACTTGATGCTATTCGTAAGGCATGT
GCATCTTTGGAGCCAAACTATCAGCCTCCTGTGACATTTGTTGTGGTTCAGAAACGCCAT
CATACTAGATTGTTTGCGAACAACCATAATGACCGGAATGCAGTTGACAAGAGTGGGAAT
ATATTACCTGGTACTGTTGTAGATTCTAAGATCTGCCATCCTACTGAGTTTGACTTTTAC
CTGTGCAGCCATGCTGGCATTCAGGGCACAAGCCGACCTGCCCATTACCATGTGTTGTGG
GATGAAAACAAGTTCACAGCTGATGGGCTGCAGTCCCTCACAAACAACCTCTGCTACACA
TATGCTAGGTGCACACGCTCTGTTTCCATCGTGCCCCCTGCATACTATGCTCATCTGGCT
GCTTTCCGAGCACGCTTCTATATGGAGCCAGAGACATCAGATAGTGGGTCAATGACAAGC
GGTGCTGCTGCTGGTCGTGGGGGCATGGGTCCTCGAAGCACACGGGTATCAGGTGCTAAT
GCAGCCGTTAGACCCCTCCCGGCCCTCAAGGAGAATGTCAAGAGGGTCATGTTTTACTGT
TAG
>Vitis-vinifera_GSVIVT00001880001
ATGGGTGCCACACAAGCACTGGTGGCTGCAAGAAGACCAGATTCTGGTGGAGTAGAAGGG
CCAGTTATCTCTCTCTTGGCAAACCATTTTCTTGTCCAATTTGATTCTTTGCAGAGAATT
TTCCATTATGATGTTGAGATCTCTCCAAATCCTTCCAAGGAAGTTGCTCGAATGATCAAG
AGAAAACTAGTAGAGGAGAACTCTGTTGAGCTCTCTGGTGCTCTTCCTGCCTTTGATGGA
CGAAAGAATCTCTACAGCCCAGTTGAATTCCAAAATGACAGGCTTGAGCTATTCATTAGC
CTTCCAATTCCCACTAGCAAGTCATTGTCCCCCTCAGGAGACTTGCAAGAGAAGCATCGA
CAGCTTAAGCTCTTCCGCATAAACATCAAGCTTGGAGATGATTGGATACCACTTCCTCAG
GATTATCTTCATGCTCTGGATATTGTTTTGAGGGAGAGTCCCACTGAGAAGTGTTTGCCT
GTGGGGAGATCCCTGTACTCGAGCTCAATGGGAGGGACTAAAGATATTGGAGGAGGAGCG
GTTGGATTAAGAGGGTTCTTTCAGAGTCTTAGACCAACCCAACAAGGACTTGCTCTCAAT
GTGGATTTTTCAGTGACTGCTTTCCATGAGAGCATTGGAATAATACCCTACTTGCAGAAG
CGAGTTGAGTTTCTTCGAGACCTTTCCCAAAGGAAGACGAGAGGTTTGACGGGCGAAGAA
AGGAAAGAAGTGGAGAAGGCATTGAAGAATATTAGGGTCTTTGTACGTCATAGAGCGACT
GTTCAGAGATATCGGGTGCATAGCTTAACTGAGGAAACTACAGAAAATCTCTGGTTTGAA
GACAGGGATGGGAAGATTCTGAGGCTGGTGAATTACTTCAAGGATCACTATGGCTACGAT
ATACAGTTCAGGAACTTGCCATGTTTGCAGATTAGTAGGAGCAAACCATGCTATCTTCCT
ATGGAGCTTTGTATGATCTGTGAAGGCCAAAAATTTCTTGGGAAGCTCTCTGATGATCAA
ACTGCAAGAATACTTAAAATGGGCTGCCAAAGACCACGAGAAAGAAAAGCCATTATTGAT
GGGGTCATGAGAGGAGCAGTTGGGCCAACAAGTGGCAGCCAGGAAAGAGAATTCAAACTC
CATGTTTCAAGAGAAATGACACGATTGAATGGAAGAGTTCTTCAACCTCCTAAGCTAAAA
CTTGGTGAGGGTGGGCATGTAAGAGATCTAATTCCTTCTCGTCATGACCGGCAATGGAAC
CTTTTGGACAGTCATGTGTTTGAAGGGACTTGCATAGAAAGGTGGGCACTGATTAGTTTT
GGTGGCACTCCTGATCAGAAATCCAATATTCCCAGATTCATAATCCAGCTTTCTCAAAGG
TGTGAACAATTGGGCATCCTTCTTAACAAAAACACTATCATGAGCCCCCAGTTTGAACCA
ATCCAACTTCTCAACAATGTCTCCCTTTTGGAATCCAAACTGAAGAAAATCCACACAGCT
GCATTGAATAATCTCCAGCTCCTTATATGCATAATGGAGAGAAAGCACAAAGGGTATGCA
GATTTGAAGCGAATTGCTGAGACCAGCATTGGGGTTGTAAGTCAGTGCTGCTTGTACCAA
AATCTTGGCAAGTCGAGTTCACAGTTTCTGGCAAACCTGGCTCTCAAGATCAATGCCAAA
ATGGGAGGATGCACTGTTGCCTTGTACAATTCACTTCCTTCCCAGATACCACGGCTGCTT
CGGCCTGATGAGCCAGTCATCTTTATGGGTGCTGATGTGACTCATCCTCATCCTCTTGAT
GATTTCAGCCCCTCCATTGCTGCTGTGGTTGGGAGCATGAACTGGCCAGCAGCTAACAAG
TATGTTTCAAGAATGAGGTCCCAGACCCATCGTCAAGAAATCATCCAGGATCTTGGTGCC
ATGGTTGGAGAAATACTGGATGATTTTTATCAGCAAGTATCCAAACTCCCCAAGAGGATC
ATTTTTTTCAGGGATGGAGTGAGCGAAACCCAGTTCTATAAGGTGCTCCAAGAGGAGTTG
CAAGCTATAAGAGTGGCTTGTTGTAGATTTCCCAATTACAGACCTCCCATTACTTTTGCA
GTGGTGCAGAAGAGGCACCACACGAGGTTGTTTCGGAATGAAAGCAACCACCCATCTTCC
ACAGGAAACCAGTTGTTGGAGGAGAACATTCCCCCAGGGACAGTTGTGGATGCTGTGATT
ACACACCCAAGGGAATTTGATTTCTATCTTTGTAGCCATTGGGGGGTGAAGGGAACAAGC
CGACCAACCCACTACCATATATTGTGGGATGAAAACCATTTCACTTCTGATGAAGTACAG
AAGCTGGTTTACAGTCTATGCTACACATTTGTGAGATGTACAAAGCCTGTGTCGTTGGTC
CCACCAGCTTACTATGCCCACCTGGCTGCATACAGAGGCAGACTCTACCTGGAGCGATCA
GAATTCACTACATTTACAAGTAGTACTTGTGCACTCTCCCGAGCTGCCCCTCCTAAGACT
ACTCCTCTACCCAAACTCAGCGAGAATGTTAAGAAACTAATGTTCTATTGCTGA
>Vitis-vinifera_GSVIVT00002671001
ATGGAATCCCAGAAGCCGCCAAATGCTGAACCTGCTAAGAGATCATCATCAATTCCAAAT
CGACTCCCAATGGCAAGACGTGGACTTGGGAGAACCGGCGAAACTATACAACTTGTAACC
AACCATTTCAAAGTTTCCATGCACTCCAATGCGAACACTGGTGCCCACTTCTACCAATAT
AATGTATCTCTGGCTCATGAAGATGGGCACCCGGCTGATGCCAAAGATATTGGCAGAAAA
GTTATGGACAAAGTTCATGAGACTTATCACACTGAGATGGCTGGTATGAGCTTTGCCTAT

GATGGCGAGAAGAGTTTGTTTACAATTGGCTCTCTTCCAAGCAAAAAGCTCCGATTCACG
GTTGTCTTGGAGGATGCATCATCAAATAGGTATAAATGCACATTGTGTTCCCTGATGATG
GGAGTGACCGAAAAAGATCAAGGCGTCCCTACCATTCCAAAACATTCAATTTTTGCAGCC
AAATTCCCAATGGATTCCATTGTAAGGGCATCATACGGCCAGCCATCAAAGCACCTCCAG
GACGCCGCTCGGGTTTTAGACATCATACTAAGGCAGCATGCAGCAAAGAAGGGCTGCCTT
GTTGTCCGACAGTCATTTTTTGACAATCTACCTAGGAATTTCACACCTTTGGGAGGTGGT
GTTCTTGGATGTCGAGGTTTCAATTCAAGCTTCAGAGCTACTCAAGGGGGTCTATTCTTG
AATATGGATGTCTCTACCACACTGGTCATACAGCCTGATCCAGTGAGAGATTTTCTCGTC
TCTAATCAAAATGTTAAAGACATGTATCACATTGACTGGAGCAAGGCCAAAAGGATGCTA
AAAAATCTGAGGGTCAAAACTTTACACTCCAATGCAGAGTGGAAAATCAGTGGATTGAGT
GAGAGGACCTGCAGAAATCAAACCTTCTTAATGAAACAAAGAAATGAAGGGCCGGATGGG
GATGAAGTCCGAAGTGTTGAAGTAACAGTTTATGACTATTTTGTCAAACATCGAAAAATA
AGTTTACAGTATTCTGGGGATTTTCCATGCATCAACGTTGGGAGATCAAAGCACCCCGTC
TATATTCCTCTCGAGCTTTGCACATTGGTTTCCTTGCAACGCTATACCAAGCCATTGTCT
ACTCAGCAGAGATCTTCACTTGTGGAGAAATCACGACAAAAGCCTCAAGAACGGATGCGA
GCATTAAAAAGCAACAAGTATGATGCAAACCCAATGCTCCGTTCTTCTGGAATTTCAATT
AGTACTCAATTTACACAAGTTGAAGGCCGTATCTTGCCGACTCCAAGTTTGAAATCAGGC
AATGGGCAGGACTTGTCACCTCGAAACGGAAGGTGGAACTTCAACAATAAGGAACTAGCT
CAACCAACAAAGATTGACCCTTGGCTTATTGCAAGCTTCTCATCACGCTGTAATATGAAA
ACACTGATTCAGGATCTCATTAAGTGTGCAAAAATGAAAGGAATTTCCATGGGATATCCG
GCTGAAATTTTTACAGAGAATCCTCAATATATGCGACAACCAGCTCCTGTTAGAGTGGAC
AAGATGATTGGCACTATGATGTCACAATTCAGACGTTTGCCCCAGTTTATTCTTTGTATT
CTTCCACAGAAGAAGAACTGCGACATCTATGGTCCATGGAAACGGCAATGCCTTTCAGGT
TGTGGAGTTCCCATACAGTGCATTGCACCATCTACACCAGTAGTGAATGATCAATACCTC
ACTAATGTGTTGTTAAAAATCAATGCCAAGCTTGGGGGTTTGAATTCTTTGTTAACTATG
GGATATTGTCCATCCCTTCATTTGATTTCAACGATACCCACTTTAATCCTTGGAATGGAT
GTGTCCCATGGCTCTCCTGGTCGGCCTGATGTGCCGTCTATTGCTGCGGTGGTAAGTTCC
AGGCATTGGCCATCGATTTCTCAATATAGAGCTACAGTCCGTACACAGTCTCCCAAACTT
GAGATGATAGATTCTCTATTCGAGCCTCTGCCTAATTCCAAAGATAGTGGCATAATCAGG
GGCACCCTGTTGGACTTCTATAAAACTTCAGCAAAAAGGAAGCCTGAACACATAATCATT
TTCAGGGATGGAGTCGGTGAATCGCAATTCAACCAAGTCTTGAACATTGAACTGGAACAA
ATAATTGAGGCCTGCAAACTCCTGGATGAGCAATGGCATCCAAAGTTCATGGTGATTATT
GCACAGAAGAATCACCATATAAGATTCTTCCAGAATGGATCCCCGTCTAATGTTCCTCCC
GGGACAATCGTGGACAACACAATATGTCATCCAAGAAACAATGATTTCTATTTGTGTGCA
CATGCTGGGATGATTGGGACAAGCCGGCCTACGCATTACCATGTATTGCTGGATGAGCTT
GGCTTTTCAGCAGATGACTTGCAGCAACTTGTGCATTCCTTGTGTTACGTATATCAGAGG
AGCACCACGGCTGTATCTCTAGTGGCCCCAGTATGCTATGCTCACCTGGCTGCAGCCCAA
GTTGCCCAGTTTATCAAGTTTGAGGACTTGCCGGAGTCATCCTCTGGTCATGCTGCTGCT
CCAGTACCACAGTTGCCCTCCTTCCATGAGAAGGTCGCTGATACTATGTTCTTTTGCTAA
>Vitis-vinifera_GSVIVT00005949001
ATGGCTGCCACACAAGCAATGGTGACTGCAAGAAGACCAGATTCTGGTGGAATAGAAGGG
CCAGTTATATCTCTTCTTGCAAACCATTTTCTTGTCCAGTTTGATTCTTCGCAGAGAATA
TTCCATTATGATGTTGAGATCTCTCCAAATCCTTCTAAGGAAGTGGCTCGAATGATCAAG
AGAAAACTAGTCGAGGAGAAGTCTGTTGAGCTCTCTGGTGCTCTTCCTGCCTTTGATGGG
CGAAAGAATCTCTACAGCCCAGTCGAATTCCAAAATGACAGGCTTGAACTATTTATTGAA
AAGCATCCACAGATTAAGCTCTTCCGCATAAACATCAAACTTGTATCAAAATTTGATGGG
AAGGAATTGAATAGCTACTTGAGCAAAGAGGGAGATGATTGGATTCCACTTCCTCAGGAT
TATCTTCATGCTCTAGATATTGTTTTGAGGGAGAGCCCAACTGAGAAGTGTGTGCCTGTG
GGTAGATCACTGTACTCGAGTTCAATGGGAGGGACTAAAGAAATTGGAGGAGGGGCGGTT
GGATTAAGAGGGTTCTTTCAGAGTCTTAGACCAACCCAGCAAGGACTTGCTCTCAATGTG
GATTTTTCAGTGACCGCATTCCATGAGAGCATAGGAATAATACCCTACTTGCAGAAGCGT
GTTGAGTTTCTTCGAGACCTTTCCCAGAGGAAGACAAGAGGTTTGACGGGCGAAGAAAGG
AAAGAAGTGGAGAAGGCATTGAAGAATATCAGGGTCTTCGTATGTCATAGAGAAACTGTT
CAGAGATATCGGGTGCATAGCTTAACGGAGGAAACTACGGAAAATCTCTGGTTCAAAGAC
AGGGATGGGAAGATTCTGAGGCTGGTGAATTACTTCAAGGATCACTATAGCTACGATATA
CAGTTCAGGAATTTACCATGCTTGCAGATTACAAGTAGCAAACCATGCTATCTTCCTATG
GAGCTTTGTATGATCTGTGAAGGCCAAAAATTTCTTGGGAAGCTATCTGATGATCAAACT
GCAAGAATACTTAAAATGGGCTGCCAGAGACCAAGAGAACGAAAAGCCATTATTGATGGG
GTCATGAGAGGAGCGGTTGGGCCAACGAGTGGCAGCCAGGAAAGAGAATTCAAACTTGAT
GTTTCAAGAGAAATGACACGATTGAATGGGAGAGTTCTGGAACCTCCTAAGCTAAAACTT
GGTGACGGTGGGCATGTAAGAGATCTAATTCCTTCCCGTCATGATCGGCAGTGGAACCTT
TTGGACAGTCATGTGTTTGAAGGGACTCACATAGAAAGGTGGGCACTGATTAGCTTTGGT

GGCACTCCAGATCAGAAATCTAATATTCCCAGATTCATAATCCAGCTATCTCAAAGGTGT
GAACAATTGGGCATCCTTCTTAACAAAAACACCATCATGAGCCCCCAGTTTGAACCAATC
CAAGTTCTCAACAATGTCTCCCTTTTGGAATCTAAACTCAAGAAAATCCACAGAACTGCA
TTGAATAATCTCCAGCTTCTTATGTGCATAATGGAGAGAAAGCACAAAGGGTATGCAGAT
TTGAAGCGAATTGCTGAGACCAGCATTGGGGTTGTAAGTCAGTGCTGCTTGTACCAAAAT
CTTGGGAAGTTGAGTTCACAGTTCCTGGCAAACCTGGCTCTCAAGATCAATGCCAAAGTC
GGAGGATGCACTGTTGCCTTGTACAATTCATTACCTTCCCAGATCCCGCGCCTCCTTCGC
CCCGATGAGCCAGTCATCTTCATGGGTGCTGATGTGACTCATCCTCATCCGCTTGATGAT
TTCAGCCCCTCTATTGCAGCCGTGGTTGGGAGCATGAACTGGCCATCAGCTAACAAGTAT
GTTTCAAGAATGAGGTCCCAGACCCATCGTCAAGAAATTATCCAGGATCTTGGTGCTATG
GTCGGAGAAATACTGGATGATTTTTATCAGCAAGTTTCCCAACTCCCCAAGAGGATAATT
TTTTTCAGGGATGGAGTCAGCGAAACCCAGTTCTATAAGGTGCTCCAAGAGGAGTTGCAA
GCGATAAGAGTGGCTTGCTCTAGATTTCCCAGTTACAGACCTCCCATTACTTTTGCAGTG
GTTCAGAAGAGGCACCACACGAGATTGTTTCCGAATGAAAGCAATCCATCTTCCATTGGA
AACCAGTTCTCGGACGACAACATCCCCCCAGGGACAGTTGTTGATGCTGTGATTACTCAC
CCAAGGGAATTTGATTTCTATCTTTGTAGCCATTGGGGTGTGAAGGGAACAAGCCGACCA
ACCCATTATCATGTCTTATGGGATGACAACCATTTCACTTCTGATGAACTCCAGAAGCTG
GTTTACAATCTATGCTACACATTTGTGAGATGTACCAAACCGGTTTCGTTGGTGCCCCCA
GCTTACTACGCCCACCTGGCTGCATACCGAGGCAGACTATACCTGGAGCGTTCAGAATTC
ACAGCATTGGCTAGAAGCACTAGCGCACTCTCCCGAGCAGCCCCTCCTAAGACAGCTCCT
CTGCCCAAACTTAGCGAGAATGTTAAGAAACTGATGTTCTATTGCTGA
>Vitis-vinifera_GSVIVT00016822001
ATGACCCACATGCAAATGAAGAACCCAGAGCAGAAGCATGTGATCTCTACTAGGAAGCCT
CTGCAGAATTCCATGAATCAACAAACATCATCAGAAATTATGCCCAGCACTGAAGAAAGC
TTGGAAAATGCAAAAAGGAGTATAGGGAGGAGAAAGGGAAGGAGAAGGGGGAAAGGAGCC
AAAGTGGAGCAGTTGCAGGATCCAAAGCTTGATTTAAGTGTTGGTTCTCATGACTCAAGC
AAAGGTCTGGTGTTCCACCGCAGGCCCGGTTATGGTCAATTGGGAAGGAAATGTGTAGTT
AAAGCCAACCATTTCCTAGCGCAAGTGCCAGATACAGACTTGAGTCAATATAGTGTTACC
ATAACCCCCGAAGTTGCTTCTCGCAAAATCAACAAATCTATCATGGCCCAGTTGGTGAAA
CTTCACAGAGATACTGACCTAGGGATGAGGCTCCCTGTTTATGATGGTAAACGGGTCCTT
TACACTGCTGGACTGCTTCCATTTGTATCAAAAGAGTTCACTGTGAAACTGGTTGAAGAG
GATGAGGGGACAGGGATTACAAAGGAGCGAGAGTTCAAAGTGACAATCAAGTTTGTAGGT
ATCACTAGTATGGTCCAATTGCGAGAGTTTCTTGCTGGGAAACAAGTTGATACTCCCCAT
GAAATAATTAGGATCTTTGATATTGTGTTAAATCAACTAGCAGCCCAAAGGTATGTATCA
GTTGGGAGATGCTTATATTCTCCTGATATTAAAAATCCTCAGCAACTAGGTGGGGGTTTA
CAATCATGGCAAGGCTTCTATAAGAGTATAAGGCCAACTCAGATGGGGTTATCATTGAAC
ATCGATATGTCATCAACTGCATTCATTGAACCATTACCTGTTATTGACTTTGTGGCTCAA
CTTTTGGACAAAGATGTATTTTCAAGGCCATTGTCAGATGCAGACCGTGTCAAGGTTAAG
AAAGCTCTTAGAGGTGTTAAAGTTGAAGTTACGCACAGAGGAAATGTACGAAGGAAATAT
CGGATTTCAGGACTGACATCACAACCTACAAGGGAACTAATTTTCCCAGTTGATGAGCAA
ATGAACATGAAATCAGTTGTTGAGTACTTTCAGGAGATGTATGGATTTACCATTCGATAT
TCTCATCTACCTTGCCTCCAAGTAGGTAACCAGAGGAAAGTGAATTACTTACCAATGGAG
GCTTGTAAGATTATTGGGGGACAGAGATATACCAAAGGGCTGACTGACAAGCAGATAACT
TCCTTGTTAAAAGTAACATGCCAAAGACCCAGGGATCGAGAAACAGACATTTTACAGACC
ATTAACCAAAATGGGTATGAGAAAGATCCCTATGCGAAGGAGTTTGGCATCACTGTAGAT
GAAAAGCTTGCTTCAGTTGAAGCTCGAGTTCTACCTGCACCATGGCTTAAATATCACGAT
ACCGGAAAAGAAAAAGAATATTTGCCGCAAGTTGGTCAATGGAATATGACAAACAAGAAA
ATGATAAATGGAAGCACTATCAATTACTGGGCTTGCATCAACTTCTCACGAAGTGTTCAG
GAGAGTACTGTAAGCGGTTTTTGTCATCAGCTGGTTCAAATGTGCAAAGTTTCTGGCATG
GAATTCAACCATGAGCCAGTAATTCCAATACATTCAGCTAGACCGGATCAGGTTAAGAAG
GCCTTGAAACATGTATATAGTGCTGCTGCAAACAAACTTGGAGGAAAAGAGTTGGAGTTA
CTCATTGCCATTCTTCCAGACAACAATGGCTCTTTGTATGGTGATTTGAAGCGGATTTGT
GACACAGATCTGGGGTTGATTTCTCAGTGCTGTCTTACTAAAAATGTCTACAAGATTAGC
AACCAGTACCTGGCAAACGTGTCACTTAAAATCAATGTTAAGATGGGTGGAAGAAATACT
GTGCTTTTAGATGCTTTGAGTTCAGGAATTCCTTTGGTTAGTGACATTCCAACAATCATA
TTTGGAGCCGATGTAACTCATCCAGAGACTGGAGATGACTCTTGTCCATCAATTGCTGCT
GTAGTAGCCTCCCAAGACTGGCCAGAAGTCACCAAGTATGCTGGATTGGTATGTGCTCAG
GCTCATCGGCAAGAACTTATTCAAGATTTGTATAAAACCTGGAAAGATCCTCAAGGGGGC
ACAGTTACTGGAGGCATGATCAGAGAGCTTTTACTTTCATTTAAGGCAGCCACTGGAAAA
AAACCATTGAGGATAATATTTTACAGGGATGGTGTCAGTGAAGGGCAGTTCTACCAGGTT
CTACTATATGAACTTGATGCCATTCGTAAGGCCTGCGCATCATTGGAACCTAGTTACCAA
CCTCCAGTGACATTTGTTGTCGTCCAAAAACGGCACCACACTAGACTCTTTGCAAGCAAC

CACAATGACAAAAGCAGCACTGATAGGAGTGGGAACATCTTACCTGGTACTGTGGTCGAT
TCGAAGATCTGCCATCCCAGTGAGTTTGACTTTTATCTATGCAGTCATGCAGGAATCCAG
GGGACCAGCCGACCCGCTCATTATCATGTTCTCTGGGATGAGAACAACTTTACAGCAGAT
GAGATCCAATCTTTGACGAACAACCTCTGTTACACGTATGCAAGGTGCACTCGGTCTGTT
TCTCTAGTTCCTCCTGCGTATTATGCTCATCTGGCAGCCTACAGAGCTCGATTCTACATG
GAACCTGATAAGCCTGAGAATGCAATACCAAATTGCATGCGCACATCAAATGAATCACGC
GTCCGGCCCCTGCCAGCATTGAATGAGAAGGTGAAGAATGTGATGTTCTACTGTTAG
>Vitis-vinifera_GSVIVT00017422001
ATGGATTCTGGGGAAGATGGAAATGGGGCACAAGATGCTTTGCCACCTCCCCCACCTGTT
CCGCCAAATGTTGTTCCAATAAAAGCTGATTCACCAGTTAAGAAAAAGGTCGCACGTGTT
CCAATAGCTCGCCGTGGCTTTGCATCCAAGGGGCAAAAAATAGCACTAACTACTAACCAC
TTCAAAGTTAATGTTACTGGTGCTGATGGTCACTTCTTCCATTACAGTGTTTCCCTTTCA
TATGAAGATGGCCGTCCTGTTGATGGTAAGGGAATTGGAAGAAAGGTTATCGATAGAGTT
CATGAGACATATGATAGCGAGTTAGGTGGAAAGGACTTTGCTTATGATGGGGAGAAGAGT
TTGTTCACAGTTGGTCCTCTTCCACGAAACAAACTTGAGTTCACTGTTGTGCTTGAGGAT
GTTTCATCAAATAGGAATAATGGCAATGGAAGTCCTGATCGTGGTAGTCCGAATGAGAGT
GATCGAAAAAGGATGCGGCGTCCTTACCAGTCAAAGACTTTTAAAGTAGAGATTAGCTTT
GCTGCTAAAATACCAATGCAGGCAATTGCCAATGCACTACGTGGTCAAGAATCAGAAAAC
TCTCAAGAAGCACTTAGAGTTTTGGATATCATTTTAAGGCAGCATGCATCAAAACAGGGT
TGCCTCCTTGTTCGTCAATCCTTTTTTCACAATGATCCAAAAAAATTTCATTGATTTGGGA
GGGGGCGTTCTTGGCTGCAGAGGATTCCATTCAAGTTTTCGAACCACCCAAGGAGGCTTA
TCACTGAATATTGGCAAGTTATTGATTTTATTTTATGTATCTACTACCATGATAGTGCAA
CCTGGGCCAGTGGTTGATTTTTTAATTGCCAATCAAAATGCGAGGGATCCTTTTTCCCTG
GACTGGGCTAAGGCCAAGAAAATGCTAAAAAATCTGAGGGTGAAGACAAGCCCCTCAAAC
ACCGAGTACAAAATAACTGGACTGAGTGAGAAGCCTTGCAAGGAGCAGTTGTTTACGCTT
AAGCAAAGAAATGGGAAGGATGAGAATGGCGAGGCCCAAACGATTGAAGTGACTGTTTTT
GATTATTTTGTTAATCATCGCCGCATAGAACTACGTTATTCTGCAGATTTACCTTGCATT
AATGTTGGGAAGCCAAAACGACCGACTTACTTCCCTATAGAGCTTTGTACCTTGGTGTCG
TTACAACGTTATACTAAAGCGTTGTCCACTCTTCAAAGAGCTTCACTGGTGGAAAGATCA
AGGCAAAAACCACAAGAAAGGATAGGAGTTTTGACTAATGCTTTGAGAAGCAACAATTAT
GATGCTGAGCCTATGCTACGTTCCTGTGGCATTTCAATAAGCAGAGACTTGACCCAAATT
GAAGGCCGTGTTCTGGCAGCTCCAAGGTTGAAAGTTGGTAATGGGGAGGATTTCTTTCCA
CGAAATGGGCGGTGGAATTTTAACAATAAGAAACTGGTGGAGCCCACAAAGATAGAACGT
TGGGCTGTGGTCAACTTCTCGGCTCGCTGTGATATTCGAAACCTCGTCCGAGAACTGATC
AAATGTGGAGGAATGAAAGGAATTCACATTGATCCTCCATTTGATGTATTTGAAGAGAAT
CCACAATCTCGACGAGCCCCACCCATTGTTAGGGTGGAGAAAATGTTTGAGGAGATACAG
TCTAAACTCCCTGGAGCTCCTCAGTTCCTTCTCTGTCTACTTCCAGAGAGGAAAAACTCT
GATCTATATGGTCCTTGGAAACGAAAGAATCTTTCTGAATATGGAATTGTGACTCAATGC
ATTGCTCCTACAAGGGTTAATGATCAATATCTTACGAATGTTCTCCTAAAGATTAATGCA
AAACTTGGTGGATTAAATTCTATGCTAGCAGTAGAACATTCCCCTTCTATTCCAATTGTT
TCGAAGGGACCCACCATAATCCTTGGGATGGATGTGTCTCATGGTTCTCCTGGACAATCT
GATGTACCATCTATTGCTGCGGTTGTCAGCTCCAGGCAGTGGCCACTGATTTCGCGCTAT
AGAGCATCAGTTCGTACACAATCTCCAAAGGTTGAGATGATTGATTCTCTGTATAAGCGA
GTATCTGAAACTGAAGATGAAGGCATAATTAGAGAGCTTTTGCTAGACTTTTATGTGAGT
TCAGGCAAAAGAAAACCCGATCAGATTATCATATTCAGGGATGGAGTCAGCGAGTCTCAG
TTCAATCAAGTTCTGAACATCGAACTGGATCAAATTATTGAGGCCTGCAAGTTCCTTGAT
GAGAAGTGGTCTCCCAAATTTGTGGTGATTGTTGCGCAGAAAAACCATCATACCAAGTTT
TTCCAACATGGATCTCCTGATAACGTCCCACCTGGCACAGTCATAGACAACAAAGTTTGT
CATCCACGGAACAATGACTTTTATCTCTGTGCACATGCTGGAATGATTGGTACTACCAGG
CCGACGCATTACCACGTTCTATTGGATGAAGTTGGTTTCTCTTCGGATGATCTTCAGGAG
CTTGTGCATTCTTTATCCTATGTGTACCAAAGGAGCACCACTGCCATTTCCGTAGTTGCT
CCCATATGCTATGCCCACTTAGCAGCTACTCAGATGTCTCAGTTCATGAAGTTTGAAGAC
ACGTCAGAGACATCCTCAAGCCAAGGTGGACTGACGTCAGCAGGGCCTGTTCCAGTGCCT
CAACTCCCCAAATTGCAGGAGAGTGTCTGCAATTCGATGTTCTTTTGCTGA
>Vitis-vinifera_GSVIVT00020067001
ATGCCTATGAGGCAGATGAAAGAGAGCTCAGAGCAACACCTAGTGATCAAAACCCACTTG
CAGAACTCCATGAACCCAGTTCAAAAGCCCCCCAAAACTGCTCAAAATGGGAAAGGCCCC
CCATCCCATGAACCTCAGAACGCCAAACCTCACAGCCAAACTTCGCCTTCATCAAAAAAC
AGAGGGAGGAGAAGAGGAAGAGGTGGCAGAAAATCTGATCAAAGCGATGTTTTCATGCGC
CCCAGTTCAAGGCCCTGCACTGTAGCAGATAAGCCAGTTTTGGCGCACCAGGCTGGACCT
CTTGTTACTGATATTCCTCATGGGTGTGTCGAAAATGGGGGCAACATGTGTGAAATGGAG
ATGGGTTTTCCTTCTTCAAGTAAGTCTTTGACCTTTGCACCTAGGCCCGGGTATGGTCAA

CTTGGAACAAAATGTATTGTGAAGGCCAACCATTTCTTTACTGAGTTACCAGAGAAGGAC
TTGAATCAGTATGATGTGACAATTACTCCTGAAGTGTCCTCAAGAACGGTTAACAGAGCT
ATCATGAATGAGCTGGTGAAACTGTACAAAGAATCTGACTTGGGAATGAGATTGCCTGCT
TATGATGGTAGAAAGAGTCTATACACTGCTGGTGAGCTTCCGTTTGCTTGGAAGGAGTTC
AAGGTTAAGCTAGTTGATGAAGAGGATGGAATCAATGAGAGGGAGTATAAAGTGGTCATC
AAGTTTGTAGCACGGGCAAGCTTGCATCATTTGGGCCAGTTTCTGGCTGGTAAGCGAGCA
GATGCTCCACAGGAAGCTCTTCAAATTCTTGACATTGTATTGAGAGAGCTCTCTACTAGA
AGGTACTGTCCTGTTGGGAGATCCTTCTTTTCTCCTGATATTAGAGCACCTCAACGTCTT
GGAGAAGGTTTGGAGTCATGGTGTGGATTCTACCAGAGTATAAGACCTACTCAGATGGGT
TTGTCACTGAATATTGATATGTCTTCGGCTGCATTCATTGAAGCTCTCCCTGTAATAGAG
TTTGTTGGTCAGCTATTAGGCAAAGATGTGTTATCTAGGCCATTGTCTGATTCTGACCGT
GTGAAGATCAAAAAGGCCCTTAGAGGAGTAAAAGTTGAAGTAACACACAGAGGGAATGTA
CGAAGAAAGTATCGTGTATCAGGACTAACATCCCAACCTACAAGAGAACTAGTTTTTCCT
GTTGATGATAATTCAACCATGAAGTCAGTTGTGGAATACTTCCAAGAAATGTATGGCTTT
ACAATTCAACATGCACACCTTCCTTGCCTTCAAGTAGGAAACCAGAAGAAGGCTAACTAT
TTACCTTTGGAGGCCTGCAAAATTGTAGAGGGGCAGCGGTATACCAAAAGGTTGAATGAG
AGGCAAATTACTGCTCTATTAAAAGTTACATGCCAAAGACCCAGGGATCAGGAAAATGAT
ATTCTGCAGACGGTTCAACATAATGCTTATGATCAAGATCCATATGCAAAGGAGTTTGGG
ATCAAAATCAGTGAAAAATTAGCTTCTGTTGAGGCTCGGATTCTTCCTGCTCCTTGGCTG
AAATATCATGAAACTGGGAAGGAAAAGGATTGTTTGCCTCAAGTCGGTCAGTGGAATATG
ATGAACAAAAAAATGATCAATGGAATGACTGTTAGCCGGTGGGCATGTATCAACTTCTCT
CGGAGTGTGCAAGAGAGTGTTGCTCGTGGGTTTTGTAATGAATTGGCTCAAATGTGTCAA
GTGTCTGGCATGGAATTTAACCCCGAACCTGTTATTCCAATCTACATGGCCAGACCTGAT
CAAGTAGAGAAAGCTCTGAAGCATGTGTATCATGCATCTATGAACAAACTCAAAGGAAAA
GAATTGGAGCTTCTATTAGCTATTTTACCTGACAACAATGGGTCTTTATATGGTGATCTT
AAGAGAATATGTGAAACCGACCTTGGTCTAATATCACAATGCTGTCTCACAAAACATGTC
TTCAAGATTAGCAAGCAGTACTTAGCCAATGTGTCTCTGAAGATTAATGTTAAGATGGGT
GGTAGAAACACTGTCCTTTTGGATGCTATCAGTTGCAGGATACCATTAGTTAGTGACATA
CCAACCATAATATTTGGAGCAGATGTGACGCATCCAGAGAATGGAGAAGACTCCAGCCCT
TCAATTGCTGCTGTAGTAGCTTCTCAGGACTGGCCTGAAGTCACAAAATATGCAGGATTG
GTTTGTGCTCAGGCTCACAGACAGGAACTGATACAAGATTTATACAAGACATGGCATGAT
CCTGTTCGTGGCACAGTTAGTGGTGGCATGATTCGGGATCTTTTGGTTTCCTTTCGGAAG
GCGACAGGGCAGAAGCCACTAAGGATTATATTTTACAGGGATGGTGTTAGTGAAGGGCAA
TTTTATCAAGTGCTACTTTATGAACTAGATGCAATCCGGAAAGCTTGTGCTTCTCTAGAA
CCAAACTATCAACCACCAGTTACTTTTATTGTGGTTCAAAAACGACATCATACCCGATTG
TTCGCTAACAACCACAGGGACCGGAACAGCACTGACAGAAGCGGGAATATTTTGCCTGGC
ACAGTGGTTGATTCTAAAATTTGCCATCCGACTGAATTTGATTTTTATCTCTGCAGCCAT
GCTGGTATTCAGGGGACAAGTAGGCCTGCTCATTACCATGTTTTATGGGATGAGAACAAT
TTCACAGCAGATGGAATTCAATCCTTGACGAACAATCTTTGCTACACATATGCAAGGTGC
ACACGATCGGTGTCTGTTGTTCCTCCAGCATATTATGCACATTTAGCAGCATTTCGAGCC
CGATTCTACATGGAGCCTGACATGCAGGAGAATGGTTCAAATGGAGGTGGCAGTGGTGGT
CATGCCGCCAAGGCTACACGAGCATCTGGAGAGACTGGTGTCCGGCCATTGCCAGCACTT
AAAGAAAATGTGAAAAGAGTAATGTTTTATTGTTAG
>Vitis-vinifera_GSVIVT00023378001
ATGAAGCCTTCGGACACAACGGAGGTCACAGAGGAGGTTCTGCCACCACCTCCATCTGTG
ATTCCTCATGATGGAGTTGGCATACAAGTAAAACCAGAACATACATCTGAGTCGGTGAAG
AAAACAACAAAGCCAAAGCGTGTCCCGATGTCTAGGCGTGGTTTTGGATCTAAAGGACAA
AAAATATCACTCCTAACGAACCATTTTAAAGTTGGTATTACTAATGCTAGTGGCCACTTC
TTTCACTACAGCGTAGCCTTGACATATGAAGATGGCCGGCCTGTTGAAATGAAGGGTGTT
GGAAGAAAAAATAATGGATAAAGTTCACGAGACTTATGACACTGAGCTGTCTGGGAAGGAT
TTTGCTTATGATGGGGAGAAGAGCTTATTCACGGTTGGGGCCCTCCCACACAACAAACTG
GAATTCACTGTTGTGCTTGACAGTGTCTCTTCAAATAGAAATACTAGAAATGGCAGCCCT
GATGTCAATGGAAGTCCAAATGGGGGTGACCGAAAGAGGCCAAGGCGCGCATCCCAGTCT
AAAACATTTAAAGTAGAGATAAGTTTTGCTGCCAAAATCCCAATGCAGGCCATTGCAAGC
GCATTACGTGGTCAAGAATCAGAGAATTCTCAAGAAGCCATCAGAGTCTTAGACATAATT
TTGAGGCAGCATGCAGCAAAACAGGGCTGCCTTCTTGTCCGTCAATCATTCTTTCATGAT
AACTCAAGGAATTTTACAGACTTGGGAGGTGGTGTCCTTGGATGCAGAGGGTTTCATTCC
AGTTTTCGAGCCACACAAGGTGGTTTATCTCTAAATGTTGATGGGTCCACTACTACAATC
ATACAGCCTGGACCGCTTGTTGATTTTCTCATTGCAAACCAAAATGCACGAGATCCATTC
CAGCTCGACTGGTCAAAGGCTAAACGGACTCTCAAAAAATTTAAGGATAAAGGTAAAACCT
TCTAATTCAGAGTACAGAATTGTTGGACTGAGTGAAAGTCCTTGCAAAGAGCAGATGTTC
ACCCTAAAAAATAGGGGAAAAAATGGAAATGATGATGCTGAAAGTATAGAAGTGACGGTT

TATGATTATTTTGTTAACTATCGCCAGATAGAGTTGCGTTATTCTGGAGACTTACCTTGC
ATCAATGTTGGGAAACCAAAAAGGCCTACGTACTTGCCTATTGAGCTTTGTTTTTTGGTT
TCTTTACAACGCTATACGAAGGCATTAACTGTTCATCAAAGGTCCACCTTGGTAGAAAGA
TCAAGACAAAAGCCCCAAGAGAAGATGACAATCTTAACTGATGTCATGAAAAGCAACAAT
TACGAGGCTGATTCATTGCTGCGTTCTTGTGGCATTTCAATCAGTACTCAATTTACTCAA
GTTGAAGGTCGCGTTCTTTCAGCCCCAAGGTTAAAGGCAGGAAATGGAGAAGATCTTATT
GCTAGAAATGGGCGGTGGAGCTTCAACAATAAGAAACTTGCTGAACCTTCAAAAATCAAG
AATTGGGCTGCTGTAAACTTCTCTGCTCGCTGTGATACAAAGGGTCTATGTAGAGATATA
GCCAGGTTTGGAGAAACGAAAGGAATTTTCATAGATCCCCCAATAGATGTTTTTGAAGAG
AATCCTCAATTTAGACGAGCTCCACCTATGGTTCGAGTGGAGAAGATGTTTGAACAAATG
AAGCCACAATTGCCTGATGGTCCTCCCCATTTTATTGTCTGCCTTCTTCCTGATAGGAAG
AACTCTGACATATATGGTCCATGGAAAAGGAAGTGCCTTGCAGAATTTGGAATTTTTAAT
CAATGCCTTGCACCTACTAGAGTTAATGATCAGTATATCATGAATGTACTTTTGAAGATC
AATGCCAAACTTGGTGGTTTGAATTCTCTGTTAGCCATTGAACCATCAAGAAATATACCC
TTGGTTTCCAAGGTTCCTACCATAATCTTTGGAATGGATGTGTCACATGGCTCCCCTGGC
CAATCTGATATTCCATCTGTTGCTGCTGTTGTGAGTTCTAGGTGTTGGCCACTGATCTCT
CGTTACAGAGCTTCTGTTCGTACACAGTCACCGAAAGTTGAAATGATAGATTCGCTTTTT
AAGCCAGTATCAGATGATAAAGATTTAGGCATTGTCAGGGAGCTGCTGTTGGACTTTTAT
GTGAGTTCAGGGCAAACAAAGCCCACTCAAATAATCATTTTCAGGGATGGAGTCAGTGAA
TCGCAGTTCAACCAAGTCTTGAACATTGAGCTAGATCAAATTATTGAGGCTTGCAAGTTC
CTCGATGAGAAGTGGACTCCTAAATTCACAATAATTATTGCCCAGAAAAATCACCACACA
AAATTCTTCCAAGCTGGGTCTCAAGATAATGTGCCTCCTGGAACTGTAATTGACAGCAAA
GTCTGTCATCCAACGCACAACGATTTCTACATGTGCGCACATGCAGGGATGATAGGGACA
ACAAGACCAACACATTACCATGTTCTTCTAGACGAGATAGGCTTTTCAGCTGATGATATG
CAAGAACTCATCCATTCTCTGTCCTATGTGTATCAGAGAAGCACTACTGCCATATCCATC
GTCGCCCCAGTTCGCTATGCACACCTGGCGGCTACACAGGTTTCACAGTTCATGAAGTTC
GATGACTCCTCGGAGACATCTTCGAGCCATGGAAGTTTGACTTCTGTTGGAGGTCCCCCT
GTCCCCGAACTCCCCAGGTTGCATGAAAAGGTTTGCAGTTCTATGTTCTTTTGCTGA
>Vitis-vinifera_GSVIVT00031923001
ATGGCAGAAGCTGGGGACAAGCGTATTCCTATGAGAAGGCCGGACAAAGGTGGCACCAAT
GCTGTCAGATCTGTTTCACTTCGTGTGAATCATTTTCCTGTCAAGTTCAAGTCTAATAGG
CTCATAATGCATTATGATGTTGATATTAAACCAGAGGCTCCACCCAAGGGTCGTGCAGTA
AAGATATCAAAGGCCACTTTGTATATGATACGAGAAAAGCTGTGTGTTGATCACCCCTCA
CAGTTTCCTACATCAAAGATTGCTTATGATGGTGAGAAGAACATTTTTAGTGCTGTTGAG
CTTCCCACTGGGAAATTTAAGGTGGAGATCTCTGGAGGAGAAGAGATGAAGGTTTGTTCG
TTCATTGTCACTATAAATCTGGTGAAGCAACTTGAGCTTCAAAAGTTGAGTGATTACTTA
AGCGGGGTACTCTCCTTTGTTCCCCGTGATATATTACAAGGTATGGATGTGGTAATGAAG
GAGAATCCTGCTAGACATATGATATCTTCTGGTCGGAGCTTTTACCAATTTAAAGACTCA
GGAAAAGACGAGCTTGGATATGGTATTATAGCTTCTAGAGGATTTCAACATAGTCTCAAA
CCCACTGCCCAGGGTCTATCCTTGTGCTTGGACTACTCAGTTGTGCCATTTTTTAATCCA
ATTTCGGTTTTAGAGTTCCTAAAGGAGCATGTTCGTGGCTTCTCCTTACGAGAGTTTAAG
AGATACAGGAGTAAGGTTGAGGCTACCTTAAAGGGATTGAAAGTTAGAGTGACTCACCGT
AATACAGGTCAAAAATTCATTATTGCAGGTTTAACTAGTCAAGACACGCAAAATCTGTCA
TTTCTTGCTGAAGATCCAGAACGCAAAGTTTTGTCAAAGAAAGTAATGCTTGTTGATTAT
TTCTATGAAAAGTATGGCAAGGATATTGTGCACAAGGATATTCCCTGCTTAGATGTGGGA
AAAAACAATAGGAATAACTATGTACCAATGGAATTCTGCACTTTGGTTGAGGGGCAGAGG
TATACAAAAGAGATTTTGGATAAAGATGCTGCTCAGGGGCTGAAACGTGAGCAACTTCCT
ACACCAGTTGTTAGAGAAAGCAAAATATGTGCAATGGTGCAGGCAAACGATGGACCATGC
GGGGGAGGTATCATTGACAGTTTTGGCATTGATGTAAACAAGAACATGACAGCACTTGCA
GGACGAGTCATTGGGCCACCAGAGTTGAAGTTAGGAGACCCTTCCGAGGGCAAGGTGAAC
AAGTTAACTGTGGATAAGGACAAATGCCAGTGGAATTTGGTTGGAAAATTGGTCGTGAAA
GGCATACCAGTTGACCACTGGGCTGTGGTGGACTTCACTGCGTACGAGCAGTACAACAGG
CTGAATACCGGGCAGTTTATTTCAGGATTTATTAGGCGGTGTGGAAAATTAGGAATCCAA
ATGCGGAATCCTCTTTTCTGTGAAACTGCCAACATGTATGCCTTCAGAGAATTCCCTGTG
CTACAGGAGCTGCTCGACAAGGTTTACAAAAAAGCAAGATGCCAATTACAAATTCTTGTG
TGTGTCATGGCTAGGAGGGATGCTGGCTATGGATATCTTAAGTGGTTCTCTGAGACCAGA
TTGGGGATGGTTACTCAGTGTTGTTTGTCCAGCCCCGCCAACAAAGCCAGTGATCAGTAT
CTTGCCAATCTTGCTCTCAAGTTGAATGCTAAGCTAGGGGGCAGCAATGTAGAGCTTATA
GAACGACTTCCACGGTTTGAAGGTGAAGGACATGTGATGTTTATTGGTGCTGATGTCAAT
CACCCAGGCTCTCAGAACACAACTAGTCCATCAATAGCAGCTGTTGTTGCCACAGTGAAT
TGGCCTGCAGCAAACCGCTATGCAGCTCGAATTCGCCCACAAGCCCATCGAATGGAGAAG
ATTCAGAATTTTGGGGCAATGTGCCTGGAGCTTGTTGAGGCTTATGTTCAGGCAAATAAA

GTCAAGCCAGAGAAGATCGTGGTGTTCCGTGATGGTGTAAGTGAGGGCCAATTTGACATG
GTTCTGAACGAAGAATTACTTGATCTCAAGAGAGCAATCCAGGGGGGAAATTACTGCCCG
ACCATCACTCTTATTGTGGCCCGGAAGAGACACCTAACCCGGTTGTTTCCTAAGGTAAAT
GATCGGAGCTTTAATGGGAATGTGCCTCCAGGCACTGTTGTGGACACAACAGTGGTCCAC
CTATCTGAGTTCGACTTCTATCTTTGCAGCCACTATGTAAGCATTCCTTGCACAACTCGA
GCTCAAGATCAAAGAAATTCTCATTCATATTTAAGGTTAGATTATTGA
>Vitis-vinifera_GSVIVT00031924001
ATGGCAGAAGCTGGGGACAAGCGTATTCCTATGAGAAGGCCGGACAAAGGTGGCACCAAT
GCTGTCAGATCTGTTCCACTTCGTGTGAATCATTTTCCTGTCAAGTTCAAGTCTGATGGG
CTCAATATGCATTATGATGTTGATATTAAACCAGAGGCTCCGCCCAAGAAGGGTCGTGCA
GTAAAGATATCAAAGTCCACTTCGTATATGATAAGGGAAAAGCTCTGTGTTGATCACCCC
TCACAGTTTCCTGCATCAGAGATTGCTTATGATGGTGAGAAGAACATTTTTAGTGCTGTT
GAGCTTCCCACTGGGAAATTTAAGGTGGAGATCTCTGGAGGAGAAGAGATGAAGGTTTGT
TCGTTCATTGTCACTATAAATCTGGTGAAGCAACTTGAGCTTCAAAAGTTGAGTGATTAC
TTAAGCGGGGTACTCTCCTTTGTTCCCCGTGATATATTACAAGGTATGGATGTGGTAATG
AAGGAGAATCCTGCTAGACATATGATATCTTCTGGTCGGAGCTTTTACCAATTTAAAGAC
TCAGGAAAAGACGAGCTTGGATACGGTATTATAGCTTCTAGAGGATTTCAACATAGTCTC
AAACCCACTGCCCAGGGTCTATCCTTGTGCTTGGACTACTCGGTTGTGCCATTTTTTAAT
CCAATTTCGGTTTTAGAGTTCCTAAAGGAGCATGTTTGTGACTTCTCTTTACGAGAGTTT
AAGAGATACAGGAGTGAGGTTGAGGCTGCCTTAAAGGGATATAAAGTTAGAGTGACTCAC
CGTAATACGGGTCAAAAATTCATTGTTGCAGGTTTAACTAGTGAAGACACGCGAAACCTG
TCATTTCTTCCTGAAGATCCAGAAGGCAATGTTTTGCCAAAGAAAGTAATGCTTGTTGAT
TATTTCTATGAAAAGTATGGCAAGGATATCGAGAACCAGGATATTCCCTGCTTAGATGTG
GGAAAAAACAATAGGAAGAATTATGTACCAATGGAGTTCTGCATCTTGGTTGAGGGGCAG
AGGTATACAAAAGAGATTTTGGATAAAGAGGCTGCTAAGAGGCTGAAACATGTGCAACTT
CCTACACCAGTTGTCAGAGAAAGCAAAATATGTGAAATGATGCAGGCAAACGATGGACCA
TGCGGGGGAGGTATCATTGACAGTTTTGGCATTGGTGTAAGCAAGAACATGACAGAAGTT
GCAGGACGAGTCATTGAGCCGCCAGAGTTGAAGCTAGGAGGCAAGCTGAACAAGATAACT
GTGGAAAGGGACAGATGCCAGTGGAATTTGGTTGGAAAAATGGTCGTGAAAGGCATACCA
GTTGACCATTGGGCTGTGGTGGACTTCAGTGGGCAGGAGCAGTACAACAGGCAGAATACC
AATCAGTTTATTTCAAGATTTATTAGGCGGTGTGAAAAGTTGGGAATCCAAACGAAGAAT
CCTCTTTTCTGTGAAACTGCCAGCATGCATGCCTTCAGAGTTTTCCCTGTGCTACGGGAG
CTGCTCGACAAGGTTTACAAAAAAGCAAGATGCCAATTACAAATTCTTGTGTGTGTCATG
GCTAGGAAGGATGCTGGCTATGGATATCTTAAGTGGTTTGCTGAGACCAAATTGGGGATG
GTTACTCAGTGTTGTTTGTCCCGCCCCGCCAACAAAGTCAGTGATCACCATCTTGCCAAC
CTTGCTCTCAAGTTGAATGCTAAGCTAGGGGGTAGCAATGTAGAACTTATAAAACGACTT
CCACGGTTTGAAGGTGAAGGGCATGTGATGTTTATTGGTGCTGATGTCAATCACCCTGGC
TCTCAGAACACAACTAGTCCATCAATAGCAGCTGTTGTTGCCACAGTGAATTGGCCTGCA
GCAAACCGCTATGCAGCTCGAATTCGCCCACAAGCCCATCGAATGGAGAAAATTCAGAAT
TTTGGGGCAATGTGCCTGGAGCTTGTTGAGACTTATGTTCAGGCAAATAAAGTCAAGCCA
GAGAAGATCGTGGTGTTCCGTGATGGTGTAAGTGAGGGCCAATTTGACATGGTTCTGAAT
GAAGAATTACTTGATCTCAAGAGAGCAATCCAGGGGGAAAATTACTGCCCGACCATCACT
CTTATTGTGGCCCGCAAGAGACACCTAACACGTTTGTTTCCTAAGGTAAATGATGGGAGC
TTTAATGGGAATGTGCCTCCAGGCACTGTTGTGGACACAACAGTGGTCCACCTATCTGAG
TTCGACTTCTATCTTTGCAGCCACTATGGTACACTTGGGACAAGCAAACCCACGCACTAT
CATGTCCTATACGATGAGCACAGGTTTAGTTCTGACCAGATCCAGAAGCTTACCTATAAC
TTGTGTTTCACCTTTGCTCGGTGTACAAAACCCGTCTCGCTGGTCCCCCCAGTGTACTAT
GCTGACCTCGCTGCCTATAGAGGAAGGTTGTACTATGATGCAATTGTGGCGGAGGCTGGA
GCTTCAGCTGCAACTTCATCATCAGTTGCCTCATCATCATCGTCGTCTGGAGCTTGGCTT
AATGAGAGGCTTTACCGTCTGCATGGTGCTCTGGAGAACATGATGTTTTTCATCTGA
>Vitis-vinifera_GSVIVT00031927001
ATGGAGCCATCAAAACTTTTAGGTAGCTTGACACCAACATCCTCCTTGGAACGCATGGAC
AGAGTTCTCCCTATAAGGCGACCTGACAAAGGTGGCACAAATGCAATCCAATCTACCATG
GTTCGTGTCAATCATTTCCCTGTGAAATTCAACTCTGAGAAAATCATTCTGCACTATGAT
GTTGATATTAAACCAGAGGTGCTACCCAAGCATGGTCGTACCTTGAAGTTATCAAAGTCT
AATCGCTGCATGATAAAAGAGAAGTTGTTCTCTGATGATCCCTCACGATTTCCCTTGTCA
AGAACGGCTTTTGATGGTGAGAAGAATATTTTTAGTGTTGTTGAGCTGCCCACTGGAAAA
TTTAAGGTGGAGTTCTCTGAGAGCGAAGACATGAAGATCTGTTCGTATATATTCACCATA
AAGCTTGTGAATCAACTAGAGCTTCGCAAGTTGAAGGATTATTTAAGTGGGAAACTTTTT
TCCATACCTCGTGAGATATTGCAAGGGATGGATGTGGTGATGAAGGAGAATCCTGCAAGG
CATATGATCTCTGTTGGACGGAGCTTTTACCCAACTCTGTTCTCTCTAGATGATGATCTT
GGACATGGCATTGTAGCTTCTAGAGGATTTTTACATAGCCTCAAGCCCACGGCCCAGGGT

TTAACCTTATGTTTGGACTACTCAGTCTTAGCATTTCGAAAGCCAATTCCGGTTATAGAT
TTCCTTGAGGAGCATGTTAATGGATTTAAGTTGAATGATTTAAGAAGAGTAAGGAAAGAA
GTTGAGGTTGCTCTAAAAGGATTAAAAGTTAGAGTCATTCATCGTCTTTGCAAACAAAAA
TACACTATCTCAGGATTAAGTGGTGAGGACACAAGGTATCTTTCATTTATCGCTGAAGAC
CTAGAGGGCAAATCTCCAGCAAAGAAAGTTGGGATTATTGATTATTTCAGGGAAAAATAT
GGGAAGGATATTAAGTACAAGGATATTCCGTGCTTAGATTTGGGGAAAAACAATAGGAAG
AACTATGTACCCATGGAGTTCTGCATCTTGACTGAGGGACAGAGGTTTCTCAAAGAGAAC
TTGGATAGAAATGGGGCTCAGAAGTTGAAAAATTTGTCACTGGTTGCCCCTAAAGTCAGA
GAGAACAACATATGTGAAATGGTGCGATCAAAGACTGGACCATGTGGTGGAGATATGATC
AATAACTTTGGGATTGAAGTCAACATGAGGATGACTACAGTTGCAGGACGTGTGATTATG
GCACCTGAATTAAAGCTAGGAGGGGCTCATAATGGAAGGATGAGCAAGATTACTGTGGAC
AGGAACAGGTGTCACTGGAATTTTGTAGGAAAATCTGTGGTGGAGGGCAAACACATTGAT
CGGTGGGCTGTACTTGATTTCAGTGCATATGAAGGATTCAACAGACTGAATCCTGACCAC
TTTATTCCCAAATTTATCAGGCGCTGTGCAAGTCTTGGAATTAGAATGGATGAGCCACTT
TTGTATCAGTCGTCTCGAATGAATGCCTTCTCCAATGTTGCCATGCTTCGTGAACTGCTC
TTAGGAGTAGCTGGCAGAGCTCATGATAGTACAAAAAACCAGTTGCAAATTCTTGTATGT
GTGATGGCTAGGAAGGATCCTGGCTACAACTATCTCAAGTGGTTCTGTGAAACCAACATT
GGGATAGTTACTCAATGTTGTTTGTCCAGCCCTGCAAATAAAGCAAATGACCAGTATCTT
GCAAACTTGGCTCTCAAGATGAATGCCAAGCTTGGAGGCAGCAACGTGGAGCTCATTGAC
CGGCTTCCTCATTTTGAGAATGAAGGTTATGTAATGTTTGTAGGTGCTGATGTCAATCAT
CCTGGTGCTTGGAACTCAGCTAGCCCTTCCATAGCTGCTGTTGTTGCCACTGTAAATTGG
CCTGCTGTAAATCGTTATGCTGCTCGTGTACGCCCCCAGCTCCATCGAACTGAGAAGATT
CTAAATTTTGGGGACATGTGCCTGGAGCTTATTGAGACCTATGCTCAGGTGAATAGAGCC
AAGCCGGATAAGATTGTGGTGTTTAGGGATGGGGTGAGCGAGGGCCAATTTGACATGGTG
CTTAATGAAGAATTAGTAGATCTGAAGGGGGCTATCCAGAGGGGAAATTACAACCCAACA
ATCACACTTATTATAACCCAGAAGAGACATCAGACTCGACTGTTTCCAGAAAGCAAGAGG
GAAAGGGGTCAGGACAGGAGCTTCAATGAGAATGTGTCTCCAGGCACGGTTGTAGACACA
ACTGTGGTTCACCCCTTCGAGTTTGATTTTTATCTTTGTAGCCATTATGGTGGTATTGGG
ACGAGCAAGCCAACACACTACCATGTCCTCTATGACGAGCACAGGTTCTCTTCTGACCAA
CTCCAGAAGCTTATCTACAACTTGTGTTTCACCTTTGTGCGGTGTACCAAACCTGTCTCC
CTTGTTCCTCCTGTATACTATGCCGACCTTGCTGCATACAGAGGACGGCTCTACCATGAC
GCCCTGGAGTTGGAGCGCCCAGCTTCAGCTTCTGCAGCCTCTGCAGCTTCATTTGATGAG
AGGTTTTACCGCTTGCACGGGGATCTGGAAAATACGATGTTTTTGTTTGA
>Oryza-sativa_Os01g16850

ATGGCGCTTCAGGATGAGGTCGCCCAAAACCTGAGATGGTCTATGGATAGCGCACGCGAT

ACAGCCTCATGCCCATCGTACCCCTCAGCAATGGACGGCTGGATGAAAACTGGGGTACCG

AAAGGAGGACGGGAGAAAATCTCTTGTCGGGGATTTCACTCCAGCTTTCGACCCACTGAC

AGTGGCCTGTCACTGAATGTTGATGTATCCACGACGATGATCGTCAGACCTGGACCTGTC

ATAGAGTTTCTTCTCTTCAACCAGAATATCAAGAACCCTCATGAAATTGACTGGGGAAAG

TTTTACCTTTTCCAGGACAGCATCTACCCTTCAGCATATTTTTCAGTTGCTAGGACAACT

GCGCAGCCCCCCGGAGGTGCGCCATGTCCGACGCTCGGTCTCGGCGTTCGGTCGCCTCGA

GCACTCGGCGTCAGCAGGACGCAGAGCTCGCCGCAGCAGAAGAGCGCAGCAGCGAGGGCG

GCCAGACTGGCGGCAGCGGAGTTGGCAGCAGCCAGGGCGGAGGCGGAGGCGGCGAAAGAT

GCGGCACGTGCGGCGGAGGTAGAGGTTGAGACCTTGCGCAGCAGCATCAACGGCTCCATC

GCCGGCGACATCACCGCCGACAGGGAGCTTGAGGAGCTGGCAAGAGCAAGGGCACGGGAG

CGAGCAGAGCGGTGGGCAGCAGCCCACCTCCACGGCGGCGGCGGCGGCCCAAGGGACCGC

GCGCCCGCCGACGGGAACCCAGACGGGCTCGGGCGTGCCGGCGGCAGCCCGGAGCCCGCA

CGTGGCCCTCGCAGGCAGCACGGCTCTCTCTCCCCTGACCGGCACCATGGTCACCACGGC

GTCCAGACAGTGGTCAGGGACTTTGGTCCCGGCGGTGGGTGGCCTACCCTCACCAAAACC

AACTACATCGAGTGGGCCGCGGTGATGAGGGCAGTCCGGTACGGTGACGTCGACTACGAC

GAGGATCGGCGAGCACTGGATGCCCTCATCGCTGCAGTCCCGCCCGAGATGCAGTTCTCG

CTTTCCCAGAAGCGGACTGCCAAGGAGGCCTGGGACGCCATCGCTGCGGCACGCATCGGC

AGCGACCGCGCCCGCAAGTCCACACTGCAGGCACTCCGCAAGGAGTGGGAGAACCTGGCC

TTCAAGCCAGGTGAGGATGTTGATGACTTTGCTCTCCGTCTCAACACTCTCTTGCAGAAA

ATGGTGCAGTACGGCGACGACACCTACGACGAGGAGAGAGCTGTCGAGAAGCTCTTCCGT

TGCGTCCCTGAGAAGTACAGGCAGATCGCTCGCTCGATCGAATCTCTGCTGGACCTCTCC

ACGATGTCGATCGAGGAGGCGTTAAGTCGCCTCAAGGTCGTCGATGGTGATGAGCCACAG

CCTCTCTCGGGGCCCATCACCATCGGCGGGAAGCTCCATCTCACTCGGGAACAGTGGGAG

GCCTCTCAAGGTGACGGGAGGAAGGGGGAGTCATCTTCCCCGATAGGCGGCCGTAAGCCG

CGCAAGGCACAGGGAGGTGTCCAGCTACGGTGGGCGCGAAGACGTGCCGAGGGTGGCGCC

CGCAGAGGCGCCCAGGGCGTTGCCACCGGCAACCACAAGCCGGCACGAGACGACGCCTGC

CGCAACTGTGGCAAGCTTGGCCATTGGGCCAAAGACTGTCGACAGCCACGACGTGGCCAG

GCCCACGTCGCACGGGTGGAGGAAGAGCCGGCTCTGCTCCTGGCTCACGCAAGCATCGAG

CTACCTCCAGCGGCACCGGCCGCAGCGGCTTTCCTCCACCTTGATGAGCCGAAGGTACTC

GTCTCCCTCTGCAACGGCTCCAGCAACGACAAGGCTGATGGGTGGTACCTCGACACCGGC

GCCACCCATCACATGACCAGCCGACGGGAGTTCTTCACCGAGTTCGACTCCAGCGTCCGA

GGCTCCGTCAAGTTCGGGGACGCCTCTGGCGTGGAGATCAAGGGTGTTGGCTCCGTCACC

TTCACCGCCAAGTCCGGTGAGCACAGGCTGCTCACCGGAGTCTACTACATCCCCGCGTTG

AGGAATTCTATCATCAGCTTGGGACAGCTGGATGAGAACGGCTCACGCGTGTTGGTCGAG

GACGGACTCATGAGGATTTGGGATCGCCGTCGTCGCCTTCTTGCCAAGGTAACCAGAGGC

ACTAATCGACTCTACATCCTCAGCGCGCAGGTCGCACAACCAGTTTGCCTCGCCGCTCGT

CGGGATGACGAGGCGTGGCAGTGGCACGAGCGCTTCAGGCACCTCCACTTCGAGGCCCTG

AAGCAGCTCAGTGCCAAGGAGATGGTGCGAGGCATGCCGTGCCTTGACCACGTGGAGCAG

CTCTGTGACGTCTGCGTGGTGACGAAGCAGCGGCGGCTCCCCTTTCCCCAGCAGACGAGC

TTCCGAGCCAAGGAGCGGCTCGAGCTCGTGCACGGGGACTTGTGTGGCCCAGTGACACCG

GCCACACCAGGAGGACGACGTTACTTCCTACTGCTCGTCGACGACCTCTCCTGCTACATG

TGGGTGATGGTCCTCGGCAGCAAGGGAGAGGCTGCGGACGCCATCAGGCATGCGCAGGCT

GCTGCAGAGGCAGAGTGCGGCCGCAAGCTGCACGTGCTGCGCACCGACAACGGCGGTGAA

TTCACAGCGGCTGAATTCGCGTCGTACTGCGCTGATGAGGGCATTCAGCGACACTACACC

ACGCCTTACAGCCCGCAACAGAACGACGTCGTCGAGCGGCGCAACCAGACGGTTGTGGGG

ATGGCTCGGGCCCTCCTCAAGCAGAGGGGGATGCCGGCCATCTTCTGGGGGGAGGCGGTG

GTGACGGCCGCCTACATCCTCAACCGCTCGCCTACCAAGGCCCTCGATGGCAGGACACCG

TACGAGGCTTGGCATGGGCGCAAGCCGGCGGTCTCCCACCTGCGGGTCTTCGGCTGCCTC

GCGTTCGCCAAGGAGCTTGGCCACATCGGCAAGCTCGACGACAGGAGCACCCCAGGGGTG

TTCATCGGCTACGCGGAGGGCTCGAAGGCCTACCGCATCCTCGACCCGGAGACACAGCGT

GTGCGCACTGCGCGCGACGTTGTGTTTGATGAAGGGCGAGGGTGGGTATGGGACAAGGCG

GTGGACGATGGTTCGACTCCGACGTACGACGACTTCACCGTCGAGTACGTCCACTTCGAG

GGAGCTGGGGGAGTAGGCAGCTCTTCTTCACCTAGCGTGTCTACCCCAGCCCCCAAATCT

CCACCGACTCCAACACCAACACACCCTCGGGCCACGACTTCGACTACGACGAGCTCTTCG

TCGACTCCACATGCTCCAGCACCAACAGCCACTCCTCCAAGCACGTCTACTCCGACGCCA

GCTCGTGTTGAGCGCAGCCCGGTGGAGTTCGCTACTCCGCTCTCCCACGACGGGGAGCGC

ATCGACGTGTACCACGACGGCGAGCAGCTATGGTACCGTACGATGGAGGATCTTCTCGGC

GACCAGCCGGTGCCGGGACTGGTGCCTCGCGACCTAGAGGCGCAGTTGCACCTTGCGTGC

GATGACGGTGAGCCTCGGTCTTTTGCAGAGGCCGAGAAACACGCGGCATGGCGTGCCGCG

ATGCAGTCGGAGATGGACGCGGTTCAGGAGAACCGCACCTGGGAGCTTGCTGACCTCCCT

CGTGGTCACCGCGCGATCACCCTTAAGTGGGTGTTCAAGCTGAAGAGGGATGAAGCCGGA

GCCATCGTCAAGCACAAGGCTCGCTTGGTGGCACGCGGTTTCGTGCAGCAGGAGGGGATC

GACTACGACGATGCCTTCGCTCCCGTGGCACGGATGGAGTCCGTGCGACTCCTTCTTGCG

CTGGCTGCTCAGGAAGGCTGGGGCGTCCATCACATGGACGTCAAGTCGGCGTTTCTAAAC

GGCGACTTGAAGGAGGAGGTCTACGTGCACCAGCCGCCGGGATTTGTGATCCCCGGCAAG

GAGGGCAAGGTGCTACGCCTGCACAAGGCCCTCTACGGCTTGCGGCAGGCATCGAGGGCG

TGGAATGCCAAGTTGGATTCTACGCTCAAGGGGATGGGCTTCGAGCAAAGCCCGCACGAG

GCGGCCATCTACCGGCGGGGCAATGGAGGAAATGCCTTGCTGGTGGGTGTCTACGTCGAC

GACTTGGTGATCACCGGCACCAAGGATGCGGAGGTCGCGGCGTTCAAGGAGGAGATGAAG

GCCACCTTCCAAATGAGTGATCTGGGGCCTCTCTCCTTCTACCTGGGGATTGAAGTGCAC

CAGGACAACTCCGGGATCACGCTTCGACAGACCGCCTACGCCAAGCGCGTCGTTGAGCTG

GCTGGGCTCACCGATTGCAACCCAGCTCTCACTCCGATGGAGGAGAGACTGAAGCTGAGC

CGCGACAGCACGGCGGAGGAGGTGGATGCTACACAGTACCGACGTCTTGTGGGGAGCCTT

CGCTACCTCACCCACACACGGCCGGACTTGGCCTTCTCCGTCGGCTACGTCAGTCGGTTC

ATGCAGCGACCAACGACGGAGCACCAGCAGGCTGTGAAGAGGATCATCCGCTACGTTGCG

GGGACTCTCGACCACGGTCTCTACTACCCGAAGTGTCCTGGCAAGGCACACTTCGTCGGG

TACAGCGACAGCGACCACGCCGGTGACATCGACACCAGCAAGAGCACGAGCGGGATTCTC

TTCTTCCTCGGCAAGTGCCTCGTTAGCTGGCAGTCAGTCAAGCAGCAGGTGGTGGCCCTG

TCCAGCTGCGAGGCCGAGTACATGGCGGCCTCCGCCGCTTCGACCCAGGCGCTCTGGCTT

GCTCGACTGCTTAGTGATCTCCTCGGCAGAGACACTGGAAAGGTGGAGCTCAGGGTGGAT

AGCAAGTCCGCTCTAGCCCTGGCAAAGAACCCCGTTTTTCACGAACGGACCAAGCACATC

CGGGTGAGGTACCACTTCATCCGAAGCTACTTGGAGGAATGGAGCATCAATGCGAGCTAC

ATCAACACCAAGGACCAGCTTGCGGACTTGCTCACCAAGCCTCTTGGGAGGATCAAACTA

GGAGGGTGCAGCAATAGGCTAGCAGCCAGCTATGGCTATATATATATGTATCCAACCTCC

CTTGGACAGGCCAAGTGTGCACTGAAGAACCTGAGGATAAAAACGACTCACACCGGCTCT

GAATTTAGGATCATCGGTTTGTCTGAAGACACTTGCTATTCGCAGACGTTCCAAATAAAG

AGAAAAAATGGCAACGGTGGCTCGGATACAGTGGAAGAAGTGACAGTCTTTGAATACTAC

AGGAAGAATTGGAAAATAGATTTGAAGGGATCTGCTCACTTTCCCTGTCTAAATGTTGGG

AAGCCAAAGCGGCCAACATATATCCCATTGGAGCTTTGCCATTTGGTGCCATTGCAAAGG

TACAAAAAGGCTTTGTCGACGTTACAGCGGTCCACGTTGGTTGAGAGATCAAGGCAGAAT

CCTCAAGAGAGGATGTTTGTCTTGTCTGGTGTGTTGAGGGACAGTGATTATAACTCTGTG

CCAATGCTGAGGGAGTGTGGCATTTCTATAGCTCAAGAATTTACCCAGGTTGCTGCTAGA

GTCCTTCCGGCACCAAAGCTGAAATCTGGAGATGGCGAAGATATTTTTGCGCGCAATGGG

AGGTGGAACTTCAATAAAAATTGCAATGCCCATCATCTTGCTCAGCGTCTCATCCATTGT

GGGAACTTGAAGGGACTTGTAAGTTCATCCTCCACCCAATCTTGTGCCTTTTCTGCAGAT

TCCGGACCATGGAAGCGTATGTGTCTCGTCAAATACGGTATTGTAACACAATGCTTGGCT

CCTACCAAGATCAACGATCAGTACCTGACTAATGTGCTTCTAAAGATAAATGCAAAGCTT

GGAGGGTTGAATTCGCTGCTGCAAATTGAAAGAAACCAAGCCATTCCTCTCTTGTCGAAG

ACTCCAACCATTATCTTAGGCATGGATGTTTCCCATGGCTCACCGGGACGGGATGATGTA

CCGTCTGTCGCTGCGGTTGTTAGTTCCCTGGAGTGGCCTCTCATATCAAAATATAAAGCC

TCCGTATGCACCCAGTCTCCCAGGCTAGAAATGATCGATTCCTTGTTTAAGCTAGTGGGG

AATGAGGATCATGTTATCATTAGAGATGGGGTTAGTGAAGGCCAGTTTAATCAGGTGCTG

AACATTGAGCTAGCCCAGATAATCAAGGCATGCGAGTTTCTTGCCAATGAGAAAAATGAC

AGTGAATGGTCTCCAAAGTTCACGGTGATAGTTGCGCAGAAGAACCATCACACCAAATTT

TTTCAGACAGATCGATCGAACAAAGTTGTCAATGTTCCTCCTGGTACTGTTGTTGACAAA

GGAATCTGTCATCCCAGGAACTGTGATTTCTACATGTGTGCTCATGCTGGGATGATTGGG

ACTACAAGGCCGACGCATTACCATGTGCTGCATGATGAGAACAATTTCACCCCTGATGAC

TTGCAGGAGCTTGTGCACAACCTCTCATACGTGTACCAGAGGAGCACGACGGCCATCTCA

GGTGTCGCTCCGATCTGCTACGCGCACCTGGCGGCGGCGCAGGTGTCGCAGTTCGTGAGA

CTCGACGACGCGGCGTCGGAGGGCAGCGGTGACGGCGGCGCGCCGCCGCGGCCGGTGCCG

GAGCTCCCGCGCCTGCACCCGGACGTCAGGCAGTCCATGTTCTTCTGCTGA

>Oryza-sativa_Os01g16870

ATGGAGTCCAACAGTGGTGAAATCGAGGAGTTGCCACCTCCCCCACCTTTGCCACCAAAT

GCTGAGCCAATTAAAACTGATGATACAAAAAAGTTATCAAAACCTAAGAGGGCCTTGATG

GCTCGCTCTGGTTGTGGCAAAAAAGGGCAGCCGATACAGCTGCTGACGAACCACTTCAAG

GTTTCACTGAAGGCAGCAGACGAATTTTTCCATCACTACTATGTTAATCTGAAGTATGAG

GATGATAGACCAGTGGATGGAAAGGGCATAGGTAGAAAGGTGCTGGACAAACTCCAGCAG

ACCTATGCGTCTGAGCTGGCAAATAAAGATTTTGCATACGATGGTGAGAAGAGCCTGTTC

ACCATTGGTGCTCTTCCTCAAGTGAACAATGAGTTCACGGTAGTTCTGGAAGACTTCAAC

ACTGGAAAGTCATCTGCAAATGGTGGCAGCCCTGGCAATGACAGTCCAGGAAATGACAGG

AAAAGGGTTAGAAGGCCATATCAGACAAAAACTTTTAAAGTTGAGCTGAACTTTGCAGCA

AAAATTCCTATGAGCGCCATTGCTCAGGCCTTGAGAGGCCAGGAATCTGAAAACACCCAA

GAAGCAATTCGTGTTATTGATATCATATTAAGACAGCATTCCGCAAAACAGGGTTGCCTT

TTGGTTCGCCAATCCTTTTTCCATAACAATCCTTCAAATTTTGTTGACTTGGGTGGTGGT

GTGATGGGCTGTAGGGGATTCCATTCAAGCTTCCGAGCTACACAGAGTGGACTTTCACTC

AATATCGATGTGTCCACAACGATGATTGTGAAACCTGGTCCTGTGGTAGATTTTCTACTT

GCCAACCAGAAGGTTGATCACCCAAACAAAATTGACTGGGCAAAGGCCAAGCGTGCTCTG

AAGAATTTAAGGATAAAAACAAGTCCAGCAAATACTGAATACAAGATTGTTGGTTTGAGT

GAGAGAAACTGCTATGAACAAATGTTCACTTTGAAGCAAAGAAATGGTGATGGTGAACCT

GAAGGTGTGGAAGTATCTGTTTACGAGTACTTTGTGAAGAATCGGGGCATAGAGTTGAGA

TACTCTGGTGATTTTCCCTGTATCAATGTGGGGAAACCAAAACGGCCAACTTATTTTCCA

ATTGAGCTCTGCTCTCTTGTGCCTTTGCAAAGGTATACCAAGGCTTTGAGTACACTACAG

AGGTCTTCGCTTGTTGAGAAGTCCAGGCAGAAACCTGAAGAAAGGATGTCAGTTTTGTCT

GATGTGCTGAAACGCAGCAACTATGATTCAGAGCCTATGTTGAATTCTTGTGGTATTTCA

ATTGCTCGAGGTTTTACACAAGTTGCTGGCAGGGTGCTGCAGGCCCCCAAGCTAAAAGCT

GGAAATGGTGAAGATCTTTTTGCACGTAATGGGCGGTGGAACTTTAATAATAAGAGGCTC

ATTAAGGCTAGCAGTATCGAGAAATGGGCAGTTGTTAACTTCTCTGCACGGTGTAATATT

AGGGATCTTGTCCGAGACATCATCAAGTGTGGTGGCATGAAAGGAATTAAAGTAGAAGAT
CCATTTGATGTCATTGAGGAGGACCCTTCAATGCGACGAGCTCCTGCTGCAAGAAGGGTA
GATGGCATGATTGACAAGATGCAAAAAAAGCTTCCTGGACAACCAAAGTTTCTGCTCTGT
GTTCTTGCTGAGAGGAAGAATTCAGATATTTATGGGCCCTGGAAGCGGAAATGTCTTGCT
GAATTTGGGATTATTACACAATGTGTGGCCCCTACTAGGGTCAATGATCAGTATATTACA
AATGTACTGTTAAAGATAAATGCAAAGCTTGGTGGCTTGAACTCCCTGCTTCAAATTGAA
ACATCCCCTTCCATTCCTCTTGTATCCAAGGTCCCGACAATAATATTGGGAATGGATGTT
TCCCATGGATCACCTGGACAGTCTGATATACCTTCAATTGCTGCGGTCGTTAGTTCTCGT
GAATGGCCTCTTGTATCAAAATACAGGGCTTCAGTCCGTTCTCAGTCACCTAAGTTAGAA
ATGATTGATGGACTATTTAAGCCACAAGGAGCTCAGGAAGATGATGGCCTCATTCGGGAG
TTATTAGTTGACTTCTATACCAGTACTGGTAAACGTAAGCCTGATCAAGTCATTATCTTC
AGGGATGGGGTCAGTGAGAGCCAGTTTACCCAGGTGCTGAACATTGAGCTGGATCAAATA
ATTGAGGCATGCAAGTTTCTTGACGAAAACTGGTCTCCAAAGTTTACACTGATTGTTGCA
CAGAAGAATCATCATACCAAATTTTTTGTGCCTGGATCTCAAAATAACGTTCCTCCTGGT
ACTGTTGTGGACAACGCAGTCTGTCATCCAAGGAACAATGACTTCTACATGTGTGCGCAC
GCTGGAATGATTGGAACTACAAGGCCTACACATTATCATATCCTTCATGATGAGATAGGC
TTCTCTGCTGACGATCTTCAGGAGTTGGTCCACTCCCTTTCCTATGTCTACCAGAGGAGC
ACTACAGCCATATCAGTCGTTGCACCCATTTGCTACGCGCATCTCGCTGCTGCTCAGGTC
AGCCAGTTCATCAAGTTCGACGAGATGTCGGAGACGTCGTCGAGCCATGGTGGCCATACC
TCGGCGGGCAGCGCTCCGGTGCCCGAGCTGCCTCGCCTGCATAACAAAGTCAGGAGCTCC
ATGTTCTTCTGCTGA
>Oryza-sativa_Os02g07310
ATGGAGAGTCAGAGAATGACCTGGCTCTACGATCGTCACCACTCCTTGAAACATAATAAA
GCTGAAAGACAAGCCATTCTATCAACTTATAGATTAGCAAAACGTCCAAATCTCTCTTCA
GAAGGTATGATTGGCGAAAGTTGCATTGTGAGGACAAATTGTTTCAGTGTTCATCTGGAG
TCTTTGGATGATCAGACTATCTATGAATATGATGTATGTGTCACTCCTGAGGTTGGAATC
AATCGTGCTGTTATTAGAGAGTTGGTAAAACAGCAGAAAGATTCTGGTTTGGGTGGCCGT
CTTCCTGCCTATGATGGAAGGAAGAGGCTATACACATCTGGTCCATTGCCATTTGATTCA
CATAGATTTCTTGTTCTGCTGGATAGTATTGAAGACAGTCCTGAAGAGTCAAGGCACTTG
AGAGTGAGAGATTTTGTGGTCACTTTAAAATTTGCTGCAAAAATTTCCTTATGGACTTTA
CGCAAGTTCCGTGGTGGGAAGCCAAACCGAGAAAGTCGAGCGGCCCTGCGAGCACTTGAC

GTTGTTCTAAAGGAATTACCCACTGCAAGGTATACCCAATTTGCTGGTTCATTTTACTCA

CCTAACTTAGGAGAATGCCGACAATTGTGTAAGGTCTTGGAAAGCTGGCGTGGTTTTCAC

CAAAGAATACAAGCTACTCAGATGGGACTTCAACTGAATATCGACGTGTCATCATCAGTG

TTCATCAAGCCTGTACCTGTGGTTGACTATGTTGCACAGCTTCTTAACGAGGACATCTTA

TTAGACAGACCATTATGTAGCACTGAGTTCTTGAAGATTAAGGAGGCTCTAGAAGGTCTG

AAGGTTCAGATTAATGGCATTTTGTTCAACACATATCATGTACAAGACCTTGTCCATCAA

GCAGCAAGTTTTCCAGTTAACTTTAGTATTCAGTACCCATCTTTGCCTTGCTTGAAAGTG

GCTCATTTTGGGGAGACAATATTTCTGCCATTGGAGGTCTGTAAAATTGCTGAGGGACAA

TGTCACCAGAAACAGCTCAATGCAAAACATATGGCTGCTCTTCTTCAGGTAGCTCGACAG

CCCCCTAATGAGCGTGACTACAACATTCTGCAGACTGTGCATCAGAACAAATACCAGGAG

GACCCACATGCTAAAGAGTTTGGCATTAAAATTGAGGAAAAACTTGTATCAATTAAATCT

CGCATCCTACCTGCTCCTTGGCTTAAATTCCATGACAGTGGCGAGACGACAGAATTCTTG

CCACAACTTGGCATATGGAATATGATGCATAAGAAAATGATCAATGGTGGGAGAGTGAAA

AGCTGGGCATGTGTTAACTTTTGCTGGAGTGTACGGGAGTATGCTGCTAGGAATTTCTGT

TATGACCTTGGCTTTATGTGCCGAGAATCTGGGATGGTCTTTTCAGTTAAACCTGTGCTT

CCTCTAGTGATTGCTAAACCTGGATGTGTAGAATCTGCACTCAGGACACTTCATGACGAT

GTCATGGACATACTTAGACCACAGGGCAGAAAACTTGACCTGCTGATTGTAATATTGCCT

AACAACAATGGATCTCTTTATGGTGATGTCAAAAGAATATGTGAGACAGATATTGGACTG

ATCTCTCAATGTTGTCTTGCAAAACATGTTCTTAAGATGAACAAGTGGTATCTTGCAAGC

GTTGCCCTTAAAATCAATGCTAAGATGGGCGGAAGAAATACTGTACTGGTTGATGCTTTA

GAAATGAGACTCCCCCATGTTAGGGATACACCAACTATTGTATTTGGTGCTCATGTCACC

CATCCACATCCAGGAAAAGCTAATAGTTCTTCCATTGCTGCTGTTGTTGCTTCTCAAGAC

TGGCCCGAGGTTACCAAGTATGCTGGTTTAATCAGTGTGCAAGCGTGCCACCAGGAGTCA

ATACAAGGTCTTTTTAAAGTCCAGGATGATCCGGAAAGAGGAACCACAACTAGCGGAATG

ATCAAAGAGCATCTCATGTCTTTCTATCGAGCCACTAAACGGAAGCCCGGAAGGATTATA

TTTTACAGGGATGGTGTGAGCAAAGGACAGCTCCCTCAGGCTTTGATGCACGAACTTGGT

GCCATCAAAATGGCGTGTGCATCTATGGGACCCGATTATAATCCATTAGTTACATACGTG

GTGCTCCAGAAGTGTCGCCATACACGTCTGTTTGCTGACTACTATAATGCGAATACTCAT

GATTCAACTGCAAACATACGGGCTGGTACTGTGGTTGATTCAAATATTTGCCAACCAAAT

CAGTTTGATTTCTACTTGTGTAGCCACCGCAGCACGCAGGGAACTAAGCGGCCCAGGTAT

TATCATGTTCTGTGGGACGAGAACGACTTCTTGGCTGGTTCTTTCCAAGAGCTCACAAAC

TACCTCTGCTACACCTCCGCAACTTGCACCCAATCCATATCAGTTGTGGCCCCTGTGCAC

TACGCTCGTCTTCTGTCATCACGAGCTCGATGTTACATTAAACCACGCTCGATTGGCGAT

TCAACGAGCCACACCTCCTTGCCTTCTGAAGAAGATAGCAGCGCAGCCTCAGAGACTGGC

AGTCTTCTTCCTATAAAGGATAATTTGAAAGGGGCCATGTTTTTCTGCTAG

>Oryza-sativa_Os02g45070

ATGGCCTTCCAGTTGGACAACGGGTACTACTCCCATCAAGCTTTAGCCATGATGAGAAAG

AAAAAAACTGAACCCCGTAATGCTGGGGAAAGTTCTGGAACTCAACAAGCCACTGGAGCT

CCTGGACGGGGTCCTTCACAGCGACCTGAGAGAGCTCAACAGCATGGAGGTGGTGGTTGG

CAACCTGCCAATCCTCAATATGCTCAACAAGCTGGTCGTGGTGGTGGACAACACCAGGGA

CGTGGTGGACGTTACCAGGGTCGTGGAGGGCCAACATCACATCAACCAGGTGGTGGTCCG

GTTGAATATCAAGCACATGAGTACTATGGCCGTGGTGTCCAACGGCAAGGAGGAATGCCA

CAACACAGGAGTGGCAGTGGTGGACATGGAGTTCCTGCCAGTCCATCAAGAACAGTTCCC

GAGCTGCACCAAGCCTCACAAGACCAGTACCAAGCTACGGTGGTTGCACCATCACCATCA

AGAACTGGCCCATCTTCGCTGCCTGTTGAGGCCAGCAGCGAAGAAGTCCAACATCAGTTT

CAGGAACTTGCCATCCAGGGTCAAAGCCCCACTAGCCAGGCCATTCAACCAGCACCACCA

TCGAGCAAATCAGTGAGATTTCCAATGCGCCCTGGCAAGGGTACATTTGGTGATAGGTGC

ATCGTGAAAGCCAACCATTTCTTTGCTGAATTGCCTGACAAAGACCTTCACCAGTATGAT

GTGTCTATAACTCCTGAGGTTCCTTCACGTGGTGTCAATCGTGCTGTCATTGGAGAAATT

GTAACACAATATAGGCAGTCTCATTTGGGTGGCCGTCTTCCAGTCTATGATGGAAGGAAG

AGCTTATACACAGCTGGTCCATTACCATTTACTTCTAGGACCTTTGACGTTATTCTGCAG

GATGAGGAAGAGAGCCTTGCTGTTGGGCAAGGTGCACAGAGGCGTGAGAGACCATTTAAG

GTCGTGATCAAATTTGCTGCACGCGCTGATCTCCACCATTTAGCCATGTTTTTAGCTGGA

AGGCAAGCGGATGCTCCTCAAGAAGCTCTTCAAGTTCTTGACATTGTTCTACGTGAATTG

CCTACTGCAAGGTACTCTCCAGTTGCAAGGTCATTTTATTCGCCTAACTTAGGAAGGCGC

CAACAACTTGGCGAGGGCCTGGAAAGTTGGCGTGGTTTTTACCAAAGCATACGACCCACG

CAGATGGGACTTTCTCTGAATATTGATATGTCATCGACAGCATTCATTGAGCCTCTACCT

GTGATTGACTTTGTTGCACAGCTTTTGAACAGAGACATCTCAGTTAGACCATTATCTGAT

GCTGATCGTGTGAAGATCAAGAAGGCCCTAAGGGGTGTAAAGGTTGAGGTCACACATAGA

GGCAATATGCGCAGGAAGTATCGCATTTCTGGCCTTACCTCGCAAGCAACACGAGAGTTG

TCTTTTCCCATTGATAATCATGGTACTGTGAAGACGGTGGTGCAATACTTCCAGGAGACA

TATGGATTTAACATTAAGCACACAACTTTGCCTTGCTTGCAAGTGGGCAATCAACAAAGG

CCAAATTATCTACCAATGGAGGTCTGTAAGATTGTGGAGGGACAGCGTTACTCAAAAAGA

CTAAATGAGAAGCAGATAACTGCTCTTCTTAAAGTGACCTGCCAGCGCCCTCAAGAGCGT

GAGCTGGACATTTTGCAGACTGTGCATCACAATGCATACCATCAGGATCCATATGCACAG

GAGTTTGGCATAAGGATCGATGAGCGACTTGCATCTGTTGAAGCTCGTGTTCTACCACCC

CCCTGGCTTAAGTACCACGATAGTGGCAGAGAGAAGGATGTCTTGCCAAGAATTGGCCAA

TGGAATATGATGAATAAGAAAATGGTCAATGGTGGTAGAGTTAACAACTGGACATGCATC

AATTTTTCTCGTCATGTCCAAGATAATGCTGCTAGGAGTTTCTGTCGCGAGCTTGCTATT

ATGTGCCAAATATCTGGGATGGACTTCTCAATTGATCCTGTGGTTCCTCTAGTGACTGCA

AGACCTGAACATGTGGAAAGAGCGCTCAAGGCACGCTATCAAGAGGCCATGAATATACTG

AAACCACAGGGCGGGGAGCTTGACCTGCTGATTGCAATATTGCCTGACAATAATGGTTCT

CTTTATGGCGATCTCAAAAGGATATGTGAGACTGATCTTGGATTGGTCTCGCAATGCTGT

CTTACGAAGCATGTTTTTAAGATGAGCAAACAGTATTTAGCAAACGTTGCCCTTAAAATC

AATGTTAAGGTGGGAGGAAGAAATACAGTACTTGTTGATGCTTTGACAAGGAGGATTCCC

CTTGTGAGTGATAGGCCAACGATCATTTTTGGTGCTGATGTTACACATCCCCATCCTGGA

GAAGATTCTAGCCCTTCCATTGCAGCTGTTGTTGCTTCTCAAGACTGGCCTGAGGTCACC

AAATATGCCGGATTAGTGAGTGCACAGGCCCATCGTCAGGAATTGATACAGGATCTTTTT

AAAGTATGGAAAGATCCTCAAAGAGGAACTGTAAGTGGTGGAATGATCAGAGAGCTTCTC

ATTTCTTTCAAGAGGGCAACTGGACAGAAACCCCAGAGGATTATATTTTACAGGGATGGT

GTCAGTGAAGGACAGTTTTATCAGGTTCTGTTTTATGAGCTTGATGCCATTAGAAAGGCA

TGTGCATCTTTGGAAGCCGATTATCAGCCACCCGTTACCTTTGTGGTGGTCCAGAAGCGT

CATCACACAAGGCTGTTTGCTAATAACCACAAGGACCAGCGCACTGTTGACAGAAGTGGA

AATATACTACCAGGCACCGTGGTTGATTCAAAGATATGCCATCCTACTGAGTTTGATTTC

TACCTGTGTAGCCATGCGGGCATTCAGGGAACAAGTCGCCCTGCACATTATCATGTTCTG

TGGGATGAGAACAAGTTCACTGCTGATGGTTTGCAAACTCTGACAAACAACTTGTGCTAC

ACCTATGCAAGGTGCACCCGTTCCGTATCAATCGTTCCTCCTGCATATTATGCCCATCTC

GCGGCATTTCGAGCTCGATTTTACATGGAGCCGGATACTTCAGATAGTGGGTCGATGGCG

AGCGGTGCCCATACCCGTGGCGGTGGCCCCCTTCCTGGTGCACGCAGCACTAAGCCTGCT

GGGAATGTTGCTGTAAGGCCGCTTCCTGATCTGAAGGAAAATGTCAAACGTGTCATGTTC

TACTGCTAA

>Oryza-sativa_Os02g58490

ATGGCCTCACGGAGGCCAACGCACCGCCACCATACCGAGGCTCCAGATCCTGGTGGAAGA

GGGAGAGGGAGAGGCCGTGCCGCCCGATATGCTCAGCCTCAGCCTCAGCCTCAGCAGCAG
CAGCAGCAGCAAGGACGAGGATGCCGTGCTAGAGGTGCCTCGCCGCCGCCGCCGCCGCAG
CAGCAGCAGCAGCAACAGCCAAGGTCTACTCCTACTCGTGCTACCACTGTCACCGTGGCT
TCCTCTTCATCTACCACGGCCACCGCATCATCAAGCCCTCTAGCTCCCGAGCTGCGCCAA
GCAATAATGGAAGCTCCCCGTCCCAGTGAGCTCGCTCAACCCTCACCCACTCCGCCTCAG
GAACAACCTGTGGATGCCGCTACCACGACGCCGCACCATATTCCATCCTCAAGCAAGTCA
ATTAGGTTTCCTTTGCGCCCGGGGAAGGGTACCATTGGCACCAGGTGCATGGTCAAGGCC
AATCATTTCTTCGCTCACCTGCCCAACAAGGATCTTCATCACTACGATGTCTCCATCACT
CCAGAAGTTACATCACGCATTGTTAACCGAGCTGTGATCAAGGAGCTGGTAAATCTGTAC
AAGGCATCTTATCTGGGTGGGAGGCTGCCTGCATATGATGGTAGAAAGAGCTTATACACG
GCTGGCCCATTGCCGTTTACTTCACAGGAGTTTCAGATCACTTTACTTGACGACGATGAT
GGTTCTGGTTCTGAGAGGCGACAGAGGACTTTCCGAGTAGTAATCAAGTTCGCCGCACGG
GCCGACCTTCACCGGCTTGAGTTGTTTTTAGCTGGGAGGCACGCAGAAGCTCCACAGGAG
GCACTGCAAGTTCTTGATATTGTGCTGCGAGAGCTACCATCAGCAAGATATGCGCCATTT
GGACGTTCCTTCTTTTCGCCTTACTTGGGTAGGAGGCAACCCCTTGGTGAGGGATTGGAA
AGCTGGCGTGGATTTTACCAGAGCATTCGGCCTACTCAGATGGGCTTATCACTGAATATT
GATATGTCAGCTACAGCTTTCATTGAGCCGTTACCTGTTATTGATTTTGTTGCACAACTA
TTGAATTCTGACATCCATTCAAGGCCGCTTTCTGATGCCGAACGTGTTAAGATCAAGAAA
GCCTTGAGAGGAGTAAAGGTGGAAGTTACCCACCGTGGCAACATGAGGCGGAAGTACCGG
ATATCTGGTTTGACAATTCAGCCAACTCGTGAGCTAACTTTTCCTGTTGATGAAGGAGGC
ACAGTGAAGTCAGTTGTACAGTACTTTCAAGAGACATATGGCTTTGCCATCCAACACACC
TACCTTCCATGCCTTACAGTTCAGCGATTAAATTACCTGCCTATGGAGGTCTGCAAAATA
GTGGAAGGACAGAGATACTCCAAGAGATTAAATCAGAATCAGATAAGAGCTCTTTTGGAG
GAGACATGCCAACACCCACGTGATCGGGAGCGTGATATTATTAAGATGGTTAAACATAAC
GCTTATCAGGATGATCCTTACGCAAAAGAGTTTGGCATTAAGATAAGTGATCGTCTGGCA
TCAGTAGAGGCACGAATTTTACCGGCTCCACGGCTTAAGTACAATGAGACTGGTCGAGAG
AAGGATTGCTTACCAAGAGTTGGTCAATGGAATATGATGAACAAGAAAATGGTAAATGGT
GGTAAAGTCAGAAGCTGGATGTGCGTCAATTTTGCCCGTAATGTGCAGGAAAGTGTTGTT
CGTGGGTTCTGCCATGAACTTGCTCTGATGTGTCAAGCCTCAGGAATGGATTTCGCTCCA
GAACCTATTCTTCCACCACTAAATGCACATCCAGATCAAGTGGAGCGTGCTCTAAAAGCT
AGGTATCATGATGCAATGAACGTTCTTGGACCCCAGCGCAGGGAACTTGATTTGCTTATT

GGGATACTACCTGATAACAATGGCTCGCTTTATGGTGATTTGAAGCGCGTGTGTGAAATA
GATCTTGGAATAGTTTCACAATGCTGTTGCACGAAGCAGGTGTTCAAAATGAACAAACAA
ATTCTTGCAAATCTTGCTCTGAAGATAAATGTCAAGGTTGGGGGCAGGAACACTGTGCTG
GTGGATGCTGTGTCAAGGCGTATTCCTCTGGTAACCGACAGACCTACAATTATATTTGGT
GCTGATGTTACCCATCCTCATCCTGGAGAGGACAGCAGTCCCTCAATTGCTGCTGTTGTA
GCCTCCCAAGATTGGCCTGAGGTGACAAAGTATGCTGGGTTGGTTTCTGCTCAAGCCCAC
CGACAAGAGCTGATAGAAGATCTATATAAAATCTGGCAGGATCCACAGAGAGGAACAGTT
AGTGGTGGCATGATCCGTGAGCTGCTTATATCCTTCAAAAGATCAACTGGTGAGAAGCCC
CAGCGAATAATATTTTACAGGGATGGCGTTAGTGAAGGCCAATTTTACCAAGTTCTACTT
TATGAATTGAATGCAATCCGAAAAGCATGTGCCTCCCTGGAGACAAATTACCAACCAAAG
GTGACTTTCATTGTGGTTCAGAAACGTCACCACACAAGATTATTTGCACATAATCACAAC
GATCAGAACTCAGTTGACAGGAGCGGGAACATACTCCCTGGTACGGTTGTAGATTCAAAG
ATCTGTCATCCAACTGAGTTTGACTTCTACCTGTGTAGCCATGCTGGCATTAAGGGTACT
AGTCGTCCAGCTCATTATCATGTCTTGTGGGATGAAAACAACTTCACAGCTGATGCATTG
CAGATTCTTACCAACAACCTTTGCTACACCTATGCAAGGTGCACTCGCTCTGTATCAATT
GTTCCACCTGCTTATTATGCTCATCTGGCTGCCTTCCGTGCTCGTTTCTATATGGAACCA
GATACATCTGACAGCAGCTCTGTCGTTAGTGGGCCTGGTGTACGTGGGCCACTTTCTGGC
TCATCAACATCACGTACTCGGGCCCCTGGTGGTGCAGCTGTTAAGCCACTTCCTGCTCTG
AAGGATAGTGTGAAGAGGGTCATGTTCTACTGCTGA

>Oryza-sativa_Os03g33650

ATGGAGGGGGAGCGGGAGGGGGTGGTGGCCAAGAACGAGGACAACGCAGGCGGAGGTGGT
GGTGGATTAGGTACAGGTGGTAATGGTGGTGGCGGCGGCGGCGGCAGCGCGAACGGGCGG
AGGAGGTGGAGGGGCGGGGGCAGCAGCGGGTATAGGCAGCACCCGATCATCCAGGCCTAC
CCGGCGCTCCTGCCGCTGCCGATAAACGGCGCCACCGGCCACGCACACATCAACGGCGCG
GTCTCGCTGCCGCTGCCTCTGCCGCCGCCGGTGCTGCTGTACCTGCAGCCGCCACCGCCA
CCGCCGCTGCTGCCCCTGCTCCCCAAGGTCGCCGCCGCCACGTTCTACGGCAAGCCACCC
AAGGCGGCCGACGCGGCACCGAGGGGCTCAATGTGGAAGCACAGGCCATCGAAAAAGCCG
CCACCGCATGCCATTACCGCCGCGCTGCTGCCGCTCCCGCGGGATGGCAAGGCGCTTCAG
GAGAAAATATTCTTCGCAAATGAAAGAAAAACATCTGAAAAGGAGGTAAATCATGTAGAC
ACCCATGAGAAGTTTACTGTTGCACCACTGGATAATGCAATAGCACGGAGACCTGACATG
GGTGGTGTTGAGGGGGCCGAGATCCCTCTTTCTGCAAACCATTTCCTTGTCCAGTTTGAT

CCTGGCCAGAAGATTTTCCATTACAATGTTGACATATCTCCGCGTCCATCAAAAGAAACA

GCAAGAATGATCAAGAAAAAATTAGTTGAAGAAAATCCAAGTGTTCTCTCAGGTTCCCAA

CCAGCCTTTGATGGCCGCAAGAACTTATATAGTCCTGTTAGATTTCAGGAGGACAGGGTT

GAGTTCTTTGTCAGCCTCCCAGTTGCATTAGCACGATGTTCAGTAGTTAAAGAGGATACT

GGCCACATGCTTGACAAACAGAAACTCAAGACTTTCAAAGTGAATGTCCGGTTGGTTTCG

AAGTTATGTGGTGAGGACTTGAACAAATATCTGAACGAGGACAAGGATGGCATCCCTCTT

CCACAAGATTACCTCCATGCATTGGATGTTGTGTTGCGTGAAGGTGCTATGGAAAGTTCT

ATCCTTGTGGGTCGGTCACTGTATGCACGCTCCATGGGGGAAGCAAGGGACATTGGTGGT

GGTGCTGTTGGATTAAGAGGTTTCTTTCAGAGATTGAGGCCAACCAAGCAAGGCCTTGCC

CTTAATGTTGATCTCTCACTCTCAGCTTTCCACGAGAGCACAGGCATAATTTCATACTTG

CAGAAGCGCTGTGACTTCTTGAAGGACCTTCCACAGAAGAAAACAAGGGCTTTGGCAGAA

GAGGAGCACAGGGAGGTGGAGAAAGCATTGAAAAATATCCGGGTATTTGTGTGCCATCGT

GAGACTAATCAAAGGTACCATGTGCATAGCTTGACTAAGGAGACAACAGAGAACCTCAAG

TTTCGCGACCGAAGTGGGAAGGATCTTATGGTGGTGGATTACTTCAAGGAGCACTATAAC

CATGATATACAATTCAGGAACCTTCCATGCTTGCAGATTGGCAGGAGCAAGCCATGTTAT

GTGCCAATGGAGCTTTGTGTAGTTTGTGAGGGCCAGAAGTTTCTTGGCAAGCTGTCTGAT

GAACAAACTTCTAAGATTCTGAAAATGGGTTGTGAAAGACCGAGTGAAAGAAAGGGAATC

ATAAAGGGTGTTGTCAAAGGTGCATTTCATGCAAGAAGCGATACTTATGCTGATCAGTTT

AGTCTCCAAGTGTCGAAACACATGACGAAACTCTCTGGGAGGGTTCTCTTGCCCCCAAAA

TTAAAGCTTGGCAGCAGTGGGCGCATCAAGGACATAACACCGGACCGATTTGATCGACAG

TGGAGCTTTCTGGATAGCCATGTTGCAGAGGGTTCCAAGATCAAGAGTTGGGCCTTGATA

AGTTTTGGTGGCACCCCGGAGCAGCACTTTTGCATTACAAAGTTTGTGAACCAGCTATCA

AATCGGTGTGAGCAGCTAGGAATTCTGCTAAACAAGAAGACCATTATCAGCCCAATATTT

GAGAGGATTCAACTACTCAATAACGTGGGAATTTTGGAGGGCAAGCTCAAGAAAATTCAA

GAAGCTGCATCAGGCAACTTGCAGTTGCTAATCTGTGTCATGGAGAGGAGGCACCAAGGC

TATGCTGATCTGAAGCGAATTGCAGAAACATCCATTGGTGTTGTGACACAATGTTGCCTT

TACTCCAACTTGAGCAAGCTGACCTCTCAATTCTTGACGAATTTGGCTTTGAAGATCAAT

GCGAAACTCGGTGGCTGCAATATTGCCCTATACAGCAGCTTTCCATGCCAAATTCCTAGA

ATATTTTTGTCGGAGGAGCCGGTGATGTTCATGGGTGCTGATGTCACACACCCGCATCCC

CTTGATGATTCAAGTCCATCAGTGGTTGCTGTAGTTGCAAGCATGAATTGGCCGTCAGCA

AATAAGTACATCTCCAGGATGAGATCACAGACACACCGGAAAGAAATCATTGAGCAACTG

GATGTTATGGCTGGTGAACTGCTTGAAGAGTTTCTAAAAGAAGTGGGGAAGCTCCCAAGC
AGAATCATATTCTTCAGAGATGGTGTGAGCGAGACACAGTTCTACAAGGTGCTGAAGGAG
GAGATGCATGCAGTGCGCACAACTTGTTCGAGGTATCCGGGTTACAAACCCTTGATCACA
TTCATCGTAGTTCAGAAGAGGCATCACACTAGACTCTTCCACAGGGAGAGGAATGGCAGC
TCGTCACACTACTCTGATCAGAACATACCACCAGGAACAGTTGTGGACACTGTGATTACA
CACCCAAGGGAATTTGATTTCTATCTGTGCAGCCACTGGGGCACCAAGGGGACGAGCCGG
CCAACTCATTATCATGTTCTGTGGGATGAGAATAACTTCCGTTCCGACGAAGTGCAGCAG
TTGATACACAATCTTTGCTACACATTTGCTCGGTGCACCAGGCCAGTTTCTCTTGTCCCA
CCGGCTTACTACGCACATCTCGCGGCATATAGAGGCAGGCTGTACCTTGAGAGGTCAGAT
ACAACTATGTACAGGGTCAGTCCGTTGCAGACTGTGCCGCTACCTAAGCTCAGAGACAAT
GTTAAGAGGCTCATGTTCTACTGCTAG

>Oryza-sativa_Os03g47820

ATGTCTTCGCGCGGCGGCGGCGGCGGAGGCCGCCGCGGTGGGCGAGGCGGCGGTGGTGGG
CGGGAAGGAGGCGGAGGTGGAGGTGGAGGTGGCGGGAGGGGTGGCCAGGGTAGGGGCGAT
CTCGGCGTCGTCGGCGAGCGGCAAGGCGGTGGTCGAGGGGCGGGTGAGCGCGGAGGCCGT
CACGACGCGCCGCGCGGCCGTGGCGGCGTCGCTGTGGGCGCCGGGGCTGGGCGGCAGCAG
CAGCAGCCATTCCACGCCCCGGCCCCGCCATCTGGCGGCGGCGGACGAGGCGGCGTGCAG
GTGCAGCCCAATGCGGCGGCGCGTCGACCCGTAGGCGGCGGGCGCGGCGGCGTCGGAGTG
CCGGCGCCTGCGCCGGCGGTTGCGGTGGGGGCTCTGTGCGGCGAGATGAAGGGGAAGATG
GTTGTGTCGGGTGGAGCGCCGCCAGCAGGCCAGGGTTCGTCGTTGGCGGCGGCGCAGGGC
ACGGACAATGTCAAGAGAGAGCCATCTCAGGTCGCCGCACCGGCGCCCGCGCCCCCGCCC
GCGACGCTGCCGCCGTCGTCGAGCAAGGCGGTGACCTTCCCGGCGCGTCCGGACGTCGGC
ACGATCGGGAGGCGGTGCCGCGTCCGCGCCAACCATTTCCTGGTGCAAGTCGCGGACAAG
GACATCTACCACTACGACGTGGTGATCACTCCAGAATCAACTTATCGTGAGAGAAATAGA
TCAATAATCAACAAACTTGTTGCGTTGCACAAACAATTCTTGGATGGTAGGTTACCTGTC
TATGATGGAAGAAAGAGCATATATACTGCAGGTCCACTACCTTTCAAAACCAAAGACTTT
GTTGTCAAGCACATTAATCCTTTGAGGGGTAACCAACGGGAGGAGGAATACAAGGTGACC
ATAAAGCAAGCCTCCAAAACAGATCTGTACAGCCTTAAGCAGTTTTTGGTTGGAAGGCAG
AGGGAGTTGCCACAGGACACTATTCAAGCTCTTGACATTGCTCTTAGGGAATGCCCTACC
TCAGTAAATTTTACTTGTGACAGGTATGTATCTATCTCAAGGTCATTTTTCTCCCAATCA
TTTGGGCATGGTGGCGAGATTGGTAGTGGTACAGAGTGTTGGAGGGGGTATTACCAAAGT

CTGCGCCCCACGCAGATGGGACTGTCGCTAAATATCGATATTTCTGCAACAGCATTTTAC

AAGGCCCAGCCAGTGATGGACTTTGCAGTTCAGTATTTGAACATCCGAGATGTTTCAAGG

CGCTTGTCTGATCAAGATCGTATTAAACTGAAAAAGGCCCTAAAAGGAGTCCAAATTGTG

GCAACTCACTGGAAAGAGAAATCCATACGTTACAAGATCACTGGAATACCCTCAGCTCCA

ATGAATGAATTGATGTTTGATCTAGATGGAAACAGGATATCAGTCGTCCAATACTTTAAG

AAACAGTACAATTACTCCTTAAAACATGTTAATTGGCCATGCCTTCAAGCTGGCAGTGAT

AGCAGACCAAAATATTTGCCTATGGAGGTCTGCAGTATACTTGAAGGACAACGGTATTCG

AAGAAGCTAAATGAGCATCAAGTTACAAACATTCTAAGGATGACATGTGAACGACCCGCA

CAAAGGGAGAGCAGTATCATAGAGATAAAAACATTTTACTTGTATTCTCAGATTGTTAAT

ACGAACTCTTATGGCAATGATGATTGTGCAAAAGAATTTGGCATCAAAGTCGCTAACCAA

CTTGCCGTGGTTGATGCCCGTGTACTGCCTACCCCTAGGCTTAAATATCATGACTCTGGA

AGAGAAAAAGTATGTAATCCTTCCGTTGGACAATGGAACATGATTAACAAGAGGATGGTA

AATGGAGGATGTATCAACCATTGGACCTGCCTATCTTTTGCTTCTCGGATGCATGTAAAT

GACATTAGGATGTTTTGTGAAGACCTGGTTGGCATGTGCAATAATATTGGCATGCAAATG

AATACGAGACCATGTGTGGATATCATACAAGGACAGCAACGCAACATCGAGGGTGCAATC

AGAAATATCCATAGGCAATCTTCAGAAAAGCTTGACCAGCAAGACCTGACAGGGCAGCAA

CTTCAGTTATTAATTGTAATATTGACTGAAATTAGTGGTTCTTATGGAAGGATAAAACGA

ATTTGTGAAACAGAGGTTGGTGTGATAACTCAATGCTGTGCACCCAAGAGTCTTCAGAAA

GGTGGGAAACAGTATCTTGAAAATCTGGCCCTAAAAATGAATGTTAAGGTCGGGGGGCGA

AATACAGTACTTGAAGATGCGTTGCACAAGAAAATACCAATTCTGACAGATCGTCCTACG

ATAGTGTTTGGAGCGGATGTTACACATCCATCTCCTGGGGAGGATGCTTCTCCATCCATT

GCAGCGGTTGTTGCATCCATGGATTGGCCAGAAGTTACAAAGTACAAATGCTTGGTATCT

ACACAAAGTCATAGGGAAGAAATCATATCTAATCTTTACACAGAAGTGAAAGATCCATTG

AAGGGAATTATTAGAGGTGGAATGATTAGGGAGTTGCTCAGGTCTTTCTACCAAGAAACT

GGACAGAAACCTAGCCGGATTATATTTTATCGAGATGGTATCAGCGAGGGACAGTTTAGC

CAAGTGTTGCTTTATGAAATGGATGCAATTCGCAAGGCATGTGCTAGTTTGCAGGAGGGC

TACCTTCCTCCAGTTACATTTGTTGTTGTCCAGAAAAGGCATCACACCCGCCTATTTCCA

GAAAATCGTCGAGATATGATGGATAGAAGCGGAAATATCCTTCCTGGAACTGTTGTTGAC

ACAATGATCTGCCATCCGAGTGAGTTTGACTTTTACCTCTGTAGCCATTCTGGTATCAAG

GGGACGAGCCGTCCAACACATTATCATGTTCTTTTAGATGAAAATGGGTTCAAGGCTGAT

ACGCTGCAAACCCTAACCTACAATCTTAGCTACACCTATGCCCGATGCACCCGAGCAGTC

TCCATAGTTCCTCCAGCATACTATGCGCATCTGGGAGCCTTCCGAGCACGCTACTACATG
GAGGACGAGCATTCTGATCAGGGCTCTTCTTCCTCCGTAACGACGCGAACGGATCGATCG
ACGAAGCCGCTTCCTGAAATCAAGGAGAATGTTAAGCGGTTCATGTTCTACTGCTGA

>Oryza-sativa_Os03g47830

ATGTCTTCCCGCGGCGGCGGGGTAGGGGGCCGGCGCGGTGGCCCCGGCGGCGCCAGCAGT
GTCCGTGGCGGCGAGCGCGGACGCAAACGCGGTCGAGGTGCTTTGGACGCCGTGGAGCCC
CGCGTCCCGCTTCCACGGGGCACGGGATCCGGCCCTGGGGCTGGCCGGGACGGAGCCGCC
GCGCCGGTGCCGGCGCTGCAACCGGCGGAGGCGGATGTGCTCAGCGGCGAGGTGGAGACG
GAGATGGCGGCCGGGATGGAGGCGCGCGAGGGGGCGTCGTCGTCGTCGTCTGCTTCGGCG
CCGGCGGTGGGGGAGGTCGAGCCGCCGTCGCGGGCGGTCGGTGCGCTGCCGCCGACGTCG
AGCAAGGCGGTGGTGCTCCAGGCGAGGCCGGGGTTCGGGACGGTCGGGACGAGCTGCCGC
GTCCGCGCCAACCATTTCGTCGTGCAGCTTGCCGACAAGGAGATCTACCATTACGATGTT
GCCATCGCCCCTGAATTGAGATCTCGAGAAAGGAACAGGAATATAATCAATGAACTTTTA
AGATCACACAAAAAATACTTGGATGGGCGGCGTTCGCCTGCTTATGATGGAAGAAAAGGC
ATGTTTACGGCTGGTGCATTGCCTTTTACAGACAGAGAGTTCGTTGTCAAAATTGCAAAC
GACCCTGAGAGAGGAAACCAGGGGGAGAAAGAGTTCAAAGTGACCATAAAGTGTGCTGGT
GCTGCAAACTTATATATGCACAGCCTTAAGCAATTCTTGGCTGGTACATATCCATCTCAA
GATCGTTTTTCTCACAAGCATTTGGACATAAGGATATTGATTGTGGCGTTGAATGGTGGA
GAGGATATTTCTGCAACAACATTTTACAAGGCTCAGCCAGTAATTGACTTTGCATTGGAC
TATCTGAACATGAACATTCGTGATGCTTATTCAAGGTTTGATCAAGATGGGACAAGGGTA
TCAGTTGTCCAGTACTTCAATCGCCAATACAGTTATTCTTTGAAATATATTAACTGGCCG
TGCCTTCAGGCTGGCAGCGACAGCAGGCCAACATATTTACCTATGGAGGTTTGCCGCATA
GTAAAAGGACAACGCTATTCTAGAAAATTAAATGAATGTCAAGTCACACGCATGTTGAGG
TTGGCACGTGAGACACCAGAAGAAAGGGAGAATAGTATTTTAGAGATTGCTAATGAAAAC
AACTATGGCAATGATTATCATGCCAGAGAATTTGGCATCGGGGTGACAAACCAACTTGCC
TTAGTCGATGCTCGTGTACTCCCTGCTCCAATGCTTAAGTACCATGACTCTGGACAAGAG
AAAGTATGTAATCCTTCCATTGGTCAGTGGAACATGAATAACAAGAGGATGCTCAATGGT
GGATCCATCAATTACTGGGCATGCTTGACTTTTGCTTCCTGCGTACGTCTGGCTGAAGTT
AGGACGTTTTGCAAGGAGTTGGTTCGTGTGTGCAATAGCATTGGCATGCAAATTACCGGG
GAACCATGTGTCCGTATTAGGCAAGAACGCCAAGATCACTTAGATGCTGCTGTCAGAGAT
ATACATCGACAATCTGCAGAATTTCTTTCTCAACAAGGTGTGATCGGGCAACAACTTGAG

TTACTGGTAATAGTACTACCTGATGCAAATGCAACTGTCTTTTATGGAAGGATAAAGCGG
CTTTGTGAAACTGAACTTGGTGTGATAACTCAGTGCTGTTTAGCTAGGAATGTTCAGAAT
GTTGGTGGACGAAATACAGTACTGGAAGATGCTTTGCATAGGAGAATTCCTCTGTTAACA
GATATGCCTACAATGATCTTTGGAGCTGACGTGACCCATCCACCTGCCGGGGAGGATTCA
TCTCCATCAATTGCTGCGGTTGTTGCATCGATGGATTGGCCAGAAGTGTCAAAATACAAA
TGCTCGGTTTCTTCGCAAAGCCATAGGGAAGAGATCATAGCTGATCTCTTCACAGAGGTG
AAAGATTCACAGAACAGACTTGTTTATGGTGGAATGATCAGAGAGTTGATAGAGTCTTTC
CGTAAAGCAAATGGCAGCTACAAACCTGGAAGGATAATATTTTATCGAGACGGTGTTAGT
GAAGGCCAGTTTAGCCAAGTTCTGCTTAGTGAAATGGATGCAATTCGGAAGGCTTGTGCT
AGCATAGAGGAGGGCTACCTCCCTCCAGTTACCTTTGTTGTGGTGCAAAAGAGGCATCAC
ACCCGTCTTTTTCCTGAAGATCATCACGCGAGGGATCAGATGGATCGAAGCAGAAACATC
TTACCTGGAACTGTTGTTGACACTAAGATATGCCATCCCAGTGAATTTGACTTTTACCTT
TGTAGCCATTCTGGCATTCAGGGAACAAGCCACCCCACGCATTACTATGTTCTATTCGAC
GAGAACAATTTCAGCGCCGATGCATTGCAAACATTGACTTACCATTTGTGCTACACATAT
GCACGCTGCACGCGATCAGTCTCCATAGTTCCTCCGGTGTACTATGCGCACCTGGCGGCT
TCCAGAGCGCGGCACTACCTGGAGGAGGGATCACTCCCCGACCACGGATCGTCTTCTGCT
TCTGCAGCCGGCGGTTCTCGGCGGAACGACCGTGGTGTGCCGGTGAAGCCGCTGCCAGAG
ATTAAGGAGAATGTGAAGCAGTTCATGTTTTACTGCTGA

>Oryza-sativa_Os03g57560

ATGCCCCAAGAAACCATTCAATTTGGTCACCGGGGTGACATTGGTGAGGGACTTGAGTGT
TGGAGAGGTTACTATCAGAGCCTGCGCCCAACACAGATGGGCCTTTCGCTGAATATAGAT
ATATCTGCAACATCATTTTTTAAGCCTGTGACAGTGATCCAATTTGTGGAGGAGTTCCTG
AACATACGTGACACCTCAAGACCTTTGTCAGACCGGGATCGTGTGAAGATAAAGAAAGCA
TTACGTGGGGTTCGCATTGAAACAAACCACCAAGAGGACCAAATCAGAAGATACAAAATA
ACAGGGATTACCCCCATTCCTATGAGCCAGCTGATGTACTTATCTTTCCTCTGCTTCTGC
TATAATTATCTTTTTGTTTACATGCTATTTCCTGTTGATGATAATGGGACAAGGAAGACT
GTTGTTCAGTACTTCTGGGATAGGTACAATTACAGACTGAAGTATGCTTCTTGGCCCTGC
CTACAGTCTGGCAGTGATTCTCGTCCTGTATACTTGCCTATGGAGGTGTGCAAGATTGTA
GAAGGGCAGAGGTACTCCAAGAAGCTTAACAACAAACAGGTGACCAACATCCTTAGAGCA
ACCTGTCAACGCCCCCAGCAGAGGGAACAGAGAATTCATGAGATGGTTCTCCACAACAAG
TATACAGATGATAGGTTTGCTCAGGAGTTCGGCATCAAGTTGAAGTATCATGATTCTGGA

AGGGAGAAAACTTGTGCACCCAGTGTTGGACAGTGGAACATGATTAACAAGAAAATGATC
AATGGAGGAACTGTGGATAACTGGACATGTCTGAGTTTTTCACGAATGCGTCCAGAGGAG
GTACAAAGGTTCTGTGGTGACCTGATTCAGATGTGCAATGCCACTGGAATGTCTTTCAAT
CCAAGACCAGTTGTGGATGTCCGGTCATCAAATCCTAACAATATAGAGAATGCTCTGAGG
GATGTTCACAGCAGAACATCAGAACTGCTAGCCAGAGAGGGAAAGGGAGGCCTGCAGCTT
TTAATTGTAATTCTGCTTGAAGTTAGTGGTTCTTATGGGAAAATTAAAAGGGTATGTGAG
AATGACCTTGGCATTGTATCTCAATGTTGTTTGCCAAGGCATGCCAGCAGGCCGAACAAG
CAATATTTGGAAAATGTTGCACTCAAAATCAATGTCAAGAAGTCCCAACAATCATCTTTG
GTGCTGATGTCACACACTCCACCTGGAGAGGACTCTGCATCATCTATTGCTGCGGTTGTG
GCATCAATGGATTGGCCTGAAATCACCAAATACCGAGGTCTGGTCTCTGCTCAATCACAT
AGACAGGAGATAATAGAAGATCTCTTTAGTGTTGGTAAAGATCCAGTGAAGGTTGTAAAT
GGTGGGATGATCAGGGAGTTCCTTATCGCATTCCGCAAGAAGACTGGCAGAAGGCCTGAG
AGGATAATCTTCTATAGAGATGGTGTAAGTGAAGGTCAGTTCAGCCGTGTGCTTCTTCAT
GAAATGGATGCCATCAGAAAGGCTTGTGCATCTTTGGAGGAGGGATATCTACCACCTGTC
ACATTTGTAGTAGTCCAGAAAAGGCATCACACAAGGCTTTTCCCAGAGGTTCATGGGAGG
CGAGACATGACTGACAAGAGCGGAAACATCCTTCCTGGAACTGTCAAGGACCGCCAGATT
TGCCATCCTACAGAGTTCTATTTCTACCTGTGTAGCCATGCTGGCATACAGGGTACTAGC
AGGCCAACTCATTACCATGTCCTTTATGATGAGAACCATTTTACAGCTGATGAACTTCAG
ACCCTGACCAACAATCTTTGCTATATCTATGCACGATGCACCCATGCAGTGTCTGTGGTC
CCACCGGCCTATTATTCTCATCTTGCTGCATCACATGCACACTGCTGCATTAAAGGACAT
AGTTCAGGTAGTGGCTCAACCCCTGGCAATGAGCATGACATAGTTAAAAATTCTGCTCCA
ACACTCCAGATCCTTGTCAAAGTGCTGGACTTCCAAATAGTTCCACTGACAATGAAACTG
AAGTCTTCAGCTGAAGACATTGTAGCTTTAGCTCTATCAAAACATCGCGTCTCTCTGCAT
GATGTCTATGTCTACCATGGTAGGCGAGTTATTGCCAAGAGCTTGACTCTTGAATCTTTA
AAAGCAGACAGGGATTCAACTTTTCTTATAATGCCACGTATGAGGGGTGGATGCAATGAT
ACGATTGGCGGTTTCAAGTGCATTCCTTTAGAACAACATATCAGATCATTAGGTGATTCC
CTTTTTGAAATCATATGGATACCACCAGATCTGCGAGTGAGCGGGTTCTGTTCATATCTT
ATCATACTTGGAAAACCAGCAAGAAAAATCATCTGCCAACTTTTAAAGTTGTTGGAGATT
ATCCATGCTGCCAACAGATTTGCTTCCAGGTTTACAATAGCTGATCTGGTGTTCCTCCCT
GATCTGGGTTGTATAGCTTTTAAGAAAGGAGTAAAAATAAGGTGGAACCTCAGGAGAGAG
GAGTACAAACTCAACATGGGTGATGTAGCTAGCATAATTAGCTGCTGGTTCAGGTTTAAC

CGCAGGAAGCTTGAAGCGCTGGAAGCAGGGATCCATGAGCTTCGTCCTGGCCAAGGTGAC

TCTCCAATGTTTGTTGATATATTAGTTAAGGATCTTAGAAGCCCCACACATGAGACTGGT

CTCAGTGCCAATTATAGAGGTTTCTACAAAAATTGTAGTGCTCTGAGATCTTGTTCGGCC

CACATGAATTTGTTCACCTCTTTGGACATTCGTAAGGACTTCATGGTTGGTTCTGCTGAT

TGGGGTAATTTTGTCAAGGCTTTGGGTGATATCAAACTTCCAGGCTGGTATCGTACTGCT

ATGCGATCACCTGAAATGAGGAAAGTACTCTTCTTTGAGTTTAATGATCCACATACTGGG

GAGTTACGAGGCAAAAGATATCGTGCCTTATCAGTTTTTTCATGGCTTGAATTTGCGCGG

ATATTTATTAAGCACATGAAGAAAGGGCTGTGTACCGATAAACAGGCAACAGCACTTCTC

TGTGTGATATTCTCGAATATTGTACCTGTTGTGGAGAAGAAACTTACTTACAGTTACAGG

CCACCAGCTAAGGAGAAGAGCAATGAAAGCTTTACAGTTGAAGAAATCCTGGACCCATCA

TAG

>Oryza-sativa_Os03g58600

ATGGCCTACCGCGGCGGAGGCCGAGGTGGCCGCGGCGGCGAGCAGCGGCCGCCCTACTCC

GGCCGCGGCGACGTCCCTGGCCGTGGTGGCGGTGGTGGTGGGGGTGGAGCCCCGCCGTAC

CGTCCCGCCTCGGGCTTCGTGTGGCCGCCGCCGGGGATGACGCCGCGGCCGGGGCCTCCT

CAGCCGCAGTACCCGCGGCCGGGGCCGCCGGCCGTCGTCTACGGCGCGCCGATGCCGGCG

GCGCACCACCAGGGCGCGTACCAGCCCGGGGGCGTGTACCGTGCGCCCTCCCCGGGGGTT

CCGGTGATCGGTGGCTACGCGCGCAGCACGCCCGTCACCATCCGGGCGCCTCCGCCGTCG

CACTCGTCGGCTCCGGCTCCGTACCAGCCGGCGGCGGCCGCGCCGGCTCCCTCGTCGTCC

TCCACGGCGCCGTCCGCTACGGCTCTGGCCAAGGAGTTCGAGCAGAAGCTGTTCGTGTCG

GAGACCGCGCTGGCGCCACCCGCGGCGGTGGCGTCGGCCGCGGCCGCGCCGGCTGGCGAG

GCGTCGGTGGAGTCGGACAAGGATCTGGCACCGGTGTCGAAGAAGGGGCTCGCCCACCCT

GCGCGGCCGGGGTTCGGAGCGGCCGGGAAGAAGGTGATGATCCGCGCGAACCACTTCCTC

GTCAATGTCGCCGACAACAACCTCTTCCACTACGATGTTTCAATCAATCCTGAGTCAAAA

TCAAGAGCAACAAATAGGGAAGTACTCAATGAGCTCATCAAGTTGCACGGGAAGACATCT

CTTGGTGGCAAATTGCCTGCCTATGATGGAAGAAAGAGTCTCTATACTGCTGGTTCACTC

CCTTTTGAATCGGAGGAGTTTGTGGTTAAGCTAATTGATCCCGAAAAGAAGGATAAAGAA

AGGGCGGAGAGGGAGTACAAGATCACAATTCGGATTGCTGGCAGAACAGACTTGTACCAC

CTCCAGCAGTTTTTGCTTGGAAGACAGAGGGATATGCCCCAAGAAACCATTCAAGTTCTT

GATGTTGTCCTTAGGGAGTCACCATCTTGGAATTATGTCACAGTGTCCAGATCCTTCTTC

TCTACCCAGTTTGGTCACCGGGGTGACATTGGTGAGGGACTTGAGTGTTGGAGAGGTTAC

TATCAGAGCCTGCGCCCAACACAGATGGGCCTTTCGCTGAATATAGATATATCTGCAACG

TCCTTTTTTAAGCCTGTGACAGTGATCCAATTTGTGGAGGAGTTCCTGAACATACGTGAC

ACCTCAAGACCTTTGTCAGACCGGGATCGTGTGAAGATAAAGAAAGCATTACGTGGGGTT

CGCATTGAAACAAACCACCAAGAGGACCAAATCAGAAGATACAAGATAACAGGGATTACC

CCCATTCCTATGAGCCAGCTGATATTTCCTGTTGATGATAATGGGACAAGGAAGACTGTT

GTTCAGTACTTCTGGGATAGGTACAATTACAGACTGAAGTACGCTTCTTGGCCCTGCCTA

CAGTCTGGCAGTGATTCTCGCCCTGTATACTTACCTATGGAGGTGTGCAAGATTGTAGAA

GGGCAGAGGTACTCCAAGAAGCTTAATGACAAACAAGTGACCAACATCCTTAGAGCAACC

TGTCAACGCCCCCAGCAGAGGGAACAGAGCATTCATGAGATGGTTCTCCACAACAAGTAT

ACAGAGGATAGGTTTGCTCAGGAGTTCGGTATCAAGGTCTGCAATGACCTAGTCTCTGTT

CCAGCCCGTGTGCTGCCTCCACCCATGTTGAAGTATCATGATTCTGGAAGGGAGAAAACT

TGTGCACCCAGTGTTGGACAGTGGAACATGATTAACAAGAAAATGATCAATGGAGGAACT

GTGGATAACTGGACATGTCTGAGTTTTTCACGAATGCGTCCTGAGGAGGTACAAAGGTTC

TGTGGTGACCTGATTCAGATGTGCAATGCCACTGGAATGTCTTTCAATCCAAGACCAGTC

GTGGATGTCCGGTCAACAAATCCTAACAATATAGAGAATGCTCTGAGGGATGTTCACAGG

AGAACATCAGAACTGCTAGCCAGAGAGGGAAAGGGAGGCCTGCAGCTTTTAATTGTAATT

CTGCCTGAAGTTAGTGGTTCTTATGGGAAAATTAAAAGGGTCTGTGAGACTGACCTTGGC

ATTGTATCTCAATGTTGTTTGCCAAGGCATGCCAGCAGGCCGAACAAGCAATATTTGGAA

AATGTTGCACTCAAAATCAATGTCAAGGTCGGAGGGCGCAACACTGTTCTTGAGCGAGCC

TTTATCCGCAATGGCATACCATTTGTGTCAGAAGTCCCAACAATCATCTTTGGCGCTGAT

GTCACACACCCTCCACCTGGAGAGGACTCTGCATCATCTATTGCTGCGGTTGTGGCATCT

ATGGATTGGCCTGAAATCACCAAATACCGAGGTCTGGTCTCTGCTCAACCACATAGACAG

GAGATAATAGAAGATCTCTTTAGTGTTGGTAAAGATCCAGTGAAGGTTGTAAATGGTGGG

ATGATCAGGGAGTTGCTTATCGCATTCCGCAAGAAGACTGGCAGAAGGCCTGAGAGGATA

ATCTTCTATAGAGATGGTGTAAGTGAAGGTCAGTTCAGCCATGTGCTTCTTCATGAAATG

GATGCCATCAGAAAGGCTTGTGCATCTTTGGAGGAGGGATATCTACCACCTGTCACATTT

GTAGTAGTTCAGAAAAGGCATCACACAAGGCTTTTCCCAGAGGTTCATGGGAGGCGAGAC

ATGACTGACAAGAGCGGAAACATCCTTCCTGGAACTGTCGTGGACCGTCAGATTTGCCAT

CCTACAGAGTTCGATTTCTACCTGTGTAGCCATGCTGGCATACAGGGTACTAGCAGGCCA

ACTCATTACCATGTCCTTTACGATGAGAACCATTTTACAGCCGATGCACTTCAGTCCCTG

ACCAACAATCTTTGCTATACCTATGCGCGATGCACCCGGGCAGTGTCTGTGGTCCCACCG

GCCTACTATGCTCATCTTGCTGCATTCCGCGCTCGCTACTACGTGGAAGGAGAGAGTTCG

GATGGTGGCTCGACCCCTGGCAGCAGCGGGCAGGCTGTGGCGCGAGAGGGCCCTGTGGAG

GTGCGCCAGCTTCCCAAGATCAAGGAGAACGTCAAGGACGTCATGTTCTACTGCTGA

>Oryza-sativa_Os04g06770

ATGGACGCTCATGATGGTGAGGCCGATGAGTTGCCGCCGCCTCCTCCCGTACCTGCAAAT

GTGGTGCCTATTAAAGCTGATGACGTTGAGAGCGAGGTGCCAGCAAACAAACCAGCAAAA

CCTAAGCGGTTTCCAATGGCCAGGCCTGGTTTAGGGAGAAAAGGACAGCCAATCCAGCTC

TTGGCGAATCATTACAAAGTTTCTGTGAAGAGTAGTGAAGAATATTTCTTCCACTACAAT

GTTATTCTGAAGTATGAGGATGATCGACCGGTTGATGGGAAAGGGGTAGGTCGAAAGGTG

ATTGATAAACTGCAGCAAACCTACCGTTCTGAGCTTTCAAGCAAGGACTTTGCCTATGAT

GGTGAAAAGAGCCTGTTTACAATTGGTGCTCTTCCACAAGTAACTAATGAGTTCACAGTG

GTGTTGGAGGATGTTTCAACTGGAAAGACTGCTGCCAATGGAAGCCCTGGAGGTAATGAC

AGCCCTGGAGGTAGTGACAGGAAGAGAGTTAGGAGACCATACCAGACCAAGACTTTCAAA

GTGGAGCTGTGCTTTGCAGCAAAGATCCCTATGAATGCAATTGCTCAGGCCATAAAAGGT

CAAGAATCAGAGAACTCCCAAGAAGCTCTCAGAGTTCTTGATATAATATTAAGGCAGCAC

TCTGCTAAACAGGGCTGCCTTTTAGTACGCCAGTCATTTTTCCACAACAATCCTAATAAC

TTTGTTGACCTGGGTGGTGGTGTGATGGGCTGTCGAGGATTCCATTCAAGTTTCCGTGGC

ACACAGAGTGGACTTTCTCTGAATATCGATGTTTCGACAACTATGATTGTTAAACCTGGT

CCTGTTATCGATTTTCTTCTTGCCAACCAGAAAGTTGATCACCCTGACAGAATTGATTGG

CAAAAGGCCAAGCGTGCTCTCAAGAACTTGAGGATAAGAACCACCCCTGTGAATTCAGAA

TTCAAGATCATTGGTTTGAGTGACAGAAATTGCAACGAACAGATGTTTTCGTTGAGGCAG

AGGAATGGGAACAATGGGGATGTTGATGAAGTTGAAGTTACAGTCTATGATTACTTTGTG

AAAAACAAAGGCATAGAGCTGCGCTATTCTGGCAATCTTCCTTGCATAAATGTGGGGAAA

CCAAAGCGTCCAACTTATTTTCCAATAGAGCTATGCTCTCTTATTCCACTACAAAGATAC

ACCAAAGCTTTGTCAACACTGCAAAGGTCCTCCCTTGTTGAGAAATCCAGACAGAAGCCC

CAGGAAAGAATGTCAGTCCTTAATGATGCACTTCGACATAGTAATTATGATTCTGATCCC

ATGTTGAGGGCATCTGGCATTTCAATTGCCCAAAATTTTACTCAAGTTGAAGGAAGGGTC

CTGCAACCCCCAAAGCTGAAAGCTGGCAATGGTGAAGATATATTCCCACGCAATGGTCGG

TGGAACTTCAACAATAAGAAACTGATTCAGACCTGTTCTGTCGATAAGTGGGCAGTAGTT

AATTTCTCTGCACGCTGTGATGTGCGAAATCTTATCCGGGACCTCATAAGAAATGCATCT

GCGAAGGGGATTCAAATGGCCGAACCTTTTGATGTGTTTGAAGAGAGCCCCTCATTGAGG

CGAGCACCTGTATCAAGAAGAGTAGATGATATGTTTGAACAGATAAAATCGAAACTTCCT

GGAGCTCCGAAATTCCTCTTGTGCCTTCTTCCTGAGAGGAAAAATTGTGAAGTTTATGGT

CCTTGGAAGAGGAAGTGTCTTGCTGAATTCGGTATTGTCACACAATGCCTTGCTCCACAA

AGAGTCAATGATCAGTACCTACTCAATTTGCTATTGAAGATAAATGCCAAGCTTGGTGGA

ATAAACTCATTGCTGCAAATTGAAGCATCCCCTTCAATACCTCTTGTATCGAAGACACCT

ACCATCATCTTAGGTATGGATGTGTCACACGGTCAACCAGGACAATCTGATAGACCTTCC

ATTGCTGCGGTGGTTAGCTCTCGCCAATGGCCTCTCATCTCTAAATACAGAGCATCGGTG

CACACTCAGTCACCTAAGCTAGAAATGATGTCTTCCTTGTTTAAGCCACGAGGAACTGAA

GATGATGGCCTCATTCGGGAATCGCTGATTGACTTCTACACTAGTTCTGGAAAACGGAAA

CCGGATCATGTTATCGTTTTCAGGGATGGAGTTAGTGAAAGCCAGTTTACTCAGGTCATT

AACATTGAGCTTGATCAGATCATTGAGGCATGCAAATTTCTCGATGAGAAGTGGTCACCA

AAGTTCACAGTGATTGTTGCTCAGAAGAATCATCATACCAAATTTTTTCAGTCTGGATCT

CCAGATAATGTTCCGCCAGGTACTGTTGTGGACAAACAAGTGTGCCATCCAAGGAATTAT

GACTTCTACATGTGTGCTCATGCTGGAATGATTGGAACGACGAGGCCAACACATTATCAT

GTTCTGCACGATGAGATAGGTTTCTCCCCTGATGATCTGCAGGAGCTAGTGCACTCACTC

TCTTATGTGTATCAGAGAAGCACAACAGCCATATCAGTTGTTGCTCCGATTTGCTATGCC

CATCTTGCTGCTGCTCAGGTGGGCACATTCCTCAAGTTCGAAGATATGTCGGACGCGTCG

TCCAGCCAAGGAGGTCATACGTCTGTCGGAAGTGTACCGGTGCCTGAGCTGCCTCGCCTC

CATGAGAAAGTGAGGAGCTCCATGTTTTTCTGCTGA

>Oryza-sativa_Os04g47870

ATGGTGAAGAAGAAAAGAACTGGGTCTGGCAGCACCGGTGAGAGTTCTGGAGAGGCTCCA

GGAGCTCCTGGCCATGGTTCTTCACAGCGAGCTGAGAGAGGTCCTCAACAGCATGGGGGA

GGACGTGGTTGGGTGCCTCAACATGGTGGCCGTGGTGGTGGGCAATACCAGGGCCGTGGT

GGACATTATCAGGGCCGTGGAGGGCAAGGTTCACACCATCCAGGTGGAGGGCCTCCTGAG

TATCAGGGTCGTGGAGGGCCAGGTTCACATCATCCAGGTGGTGGGCCTCCTGACTATCAG

GGCCGTGGAGGATCAGGTTCACATCACCCAGGTGGTGGGCCTCCCGAGTATCAACCGCGT

GACTATCAAGGACGTGGTGGTCCACGCCCCAGAGGTGGAATGCCACAGCCATACTATGGC

GGACCTAGGGGGGAGTGGCGGACGTAGTGTTCCTTCAGGTTCATCAAGAACAGTTCCCGAG

CTGCACCAAGCCCCACATGTCCAATACCAAGCCCCGATGGTTTCACCAACCCCATCGGGA

GCTGGCTCATCCTCTCAGCCTGCGGCGGAGGTGAGCAGTGGACAAGTCCAACAACAGTTT

CAGCAACTTGCCACCCGTGATCAAAGTTCGACCAGCCAAGCCATTCAAATAGCACCACCG

TCAAGCAAATCAGTTAGATTCCCGTTGCGCCCTGGCAAGGGTACATATGGGGACAGGTGC

ATTGTGAAGGCGAACCATTTCTTTGCTGAACTTCCTGATAAAGACCTTCACCAATACGAC

GTATCTATTACTCCTGAGGTTACTTCACGTGGCGTGAATCGTGCTGTTATGTTTGAGTTA

GTAACGCTGTATAGATATTCCCATTTGGGCGGGCGTCTACCTGCCTATGATGGAAGGAAG

AGTCTTTACACAGCTGGACCATTGCCATTTGCTTCTAGGACATTTGAAATTACTCTTCAA

GATGAGGAAGATAGTCTTGGTGGTGGCCAAGGCACCCAAAGGCGTGAGAGACTATTTAGG

GTGGTGATCAAGTTTGCTGCCCGTGCTGATCTTCACCATTTGGCTATGTTTCTAGCTGGA

AGGCAAGCAGATGCTCCTCAAGAAGCCCTTCAAGTCCTTGACATTGTGTTACGTGAATTG

CCTACCACAAGGTACTCACCAGTTGGTCGGTCATTTTATTCTCCCAATTTAGGGAGACGC

CAGCAACTTGGTGAGGGTTTGGAAAGTTGGCGTGGTTTTTACCAAAGCATAAGGCCTACC

CAGATGGGTCTCTCACTGAATATTGATATGTCATCAACTGCATTTATTGAGCCTCTACCT

GTGATTGACTTTGTTGCTCAGCTTCTGAACAGAGACATCTCAGTTAGACCATTATCTGAT

TCTGATCGTGTGAAGATAAAGAAAGCTCTAAGAGGTGTGAAGGTTGAGGTGACGCATAGA

GGAAACATGCGTAGAAAATATCGTATATCTGGACTCACTTCACAGGCAACAAGGGAGTTA

TCATTCCCTGTCGATGATCGTGGTACTGTGAAGACTGTGGTGCAATATTTTCTGGAGACA

TATGGTTTTAGTATTCAGCACACCACTTTGCCTTGCCTTCAAGTGGGCAATCAGCAAAGG

CCCAATTATCTGCCTATGGAGGTTTGTAAGATCGTTGAGGGACAGCGTTACTCGAAGCGG

CTTAACGAGAAACAGATTACTGCGCTATTGAAAGTGACTTGCCAGCGACCTCAAGAGCGT

GAACTGGATATTTTGCGGACTGTATCTCACAATGCATACCATGAAGATCAGTATGCGCAG

GAATTTGGCATAAAAATTGATGAGCGTCTTGCATCTGTTGAAGCTCGTGTTCTGCCTCCC

CCAAGGCTTAAATACCATGATAGTGGGAGAGAAAAGGATGTATTGCCGAGAGTTGGCCAG

TGGAACATGATGAATAAGAAAATGGTCAATGGTGGGAGAGTCAACAACTGGGCATGTATT

AACTTCTCTAGAAATGTGCAAGATAGTGCTGCCAGGGGCTTCTGTCATGAGCTGGCTATC

ATGTGCCAAATATCTGGAATGGATTTTGCACTGGAACCTGTGCTGCCCCCACTTACTGCT

AGACCTGAACATGTGGAAAGAGCACTGAAGGCACGCTATCAAGATGCAATGAACATGCTC

AGACCGCAGGGCAGGGAACTTGATTTACTGATTGTAATACTGCCTGACAATAATGGTTCT

CTTTATGGGGATCTCAAAAGAATCTGTGAGACTGATCTTGGATTGGTCTCCCAATGTTGT

TTGACAAAACATGTTTTTAAAATGAGCAAGCAGTATCTTGCAAATGTTGCCCTTAAAATA

AACGTTAAGGTGGGGGGAAGGAATACTGTACTTGTGGATGCTTTGACAAGGAGGATTCCC

CTTGTCAGTGACAGACCAACTATCATATTTGGTGCGGATGTTACTCATCCTCATCCTGGA

GAAGATTCCAGTCCTTCCATTGCAGCTGTGGTTGCTTCTCAAGACTGGCCTGAAGTCACT

AAGTATGCTGGATTGGTGAGTGCCCAAGCCCATCGTCAAGAATTGATACAAGATCTTTTC
AAAGTATGGCAAGACCCGCATAGAGGAACTGTTACTGGTGGCATGATCAAGGAGCTTCTC
ATTTCTTTCAAGAGGGCTACTGGACAGAAACCTCAGAGGATAATATTTTACAGGGATGGT
GTCAGCGAGGGGCAGTTTTATCAAGTTTTGTTGTATGAGCTTGATGCCATTAGAAAGGCT
TGTGCATCCCTGGAACCCAACTATCAGCCTCCAGTTACCTTTGTGGTGGTCCAGAAGCGG
CATCACACAAGGTTGTTTGCTAATAATCACAACGACCAGCGTACTGTTGATAGAAGTGGA
AACATTCTGCCTGGAACTGTTGTTGACTCAAAGATTTGCCATCCAACCGAGTTTGATTTC
TACCTGTGTAGCCATGCTGGCATACAGGGAACAAGCCGTCCTGCTCATTATCATGTTCTG
TGGGATGAGAACAAATTTACTGCAGACGAGTTGCAAACCCTCACGAACAACTTGTGCTAC
ACGTATGCAAGGTGCACTCGCTCTGTATCAATTGTGCCTCCTGCGTACTATGCTCATCTG
GCAGCCTTCCGAGCTCGCTTTTACATGGAGCCAGAGACATCTGACAGTGGATCAATGGCG
AGTGGAGCTGCAACGAGCCGTGGCCTTCCACCAGGTGTGCGCAGCGCCAGGGTTGCTGGA
AATGTAGCCGTCAGGCCTCTACCTGCTCTCAAGGAAAACGTGAAGCGTGTCATGTTTTAC
TGCTAA
>Oryza-sativa_Os04g52540
ATGGAGCACGAGCGCGGTGGCGGTGGCCGCGGCCGCGGGAGGGGTCGCGGTGGCGGGCGT
GGCGGCGGTGGCGGCGATGGTCGCGGAGGCGGTTATGGTGGTGCTGGTGGTGGTGGTGTC
GGCGGGCGCGGTGGGCGTGGGCCTCCTGGTGGTGGTGGTGGACGCGGGTACGAGCCCGGC
GGCGGGCGTGGGTACGGTGGCGGCGGCGGCGGTGGTGGACGTGGGTATGGCGGCGGAGGC
GGCGGTGGTGGGTACGAGTCCGGCGGTGGGCGTGGGTATGGCGGCGGTGGACGTGGGTAT
GAATCCGGCGGTGGGCGTGGACCTGGCGGCGGCGGCCGTGGGCACGAGTCCGGCGGTGGC
GGTGGCCGCGGCGGGAACGTGTGGGCGCAGCCGGGGAGAGGGCGCGGAGGAGCCCCCGCC
CCGGCGCCGGCGCCAGCACCAGCAGCGAGGAGGATCCAGGACGAGGGGGCCGCGAGGTCG
TCGGGTACCGTTGAGCGCATTGCTTCTACTGAGGTTGTAAGAGTACAACCACCTGCACCC
CCAGTTGCTGTGTCTCGTAGTGGCACGCGTGTGCCAATGCGAAGACCTGATGGTGGAGGC
TCAGTATCGAAAGCCAAGGTCAAATTGTTGGTGAACCATTTTATAGTTAAGTACCGACAG
GCATCAACTGTTTTTCACTATGACATAGACATCAAGCTTGATATAAGTTCCCCCAAGGCT
TCAGACAAGGAGCTATCCAAGGGAGATTTTCTTACTGTCAAGGACGAGCTCTTCAAGGAT
GAGAGCTTTCGGCGGCTTTCATCAGCTGTTGCTTATGATGGAAAAAGAAATTTATTTACT
TGTGCTGAGCTACCAGATGGTTTGTTTCGTGTCAAAGTCCGTTCACGGACTTACATTGTA
TCTGTGGAGTTCAAGAAGAAGCTTCCTTTGAGCCAACTCTCGGAACTGCCTGTGCCCAGA

GAGGTCTTGCAGGGGCTTGATGTCATTGTGCGTGAGGCCTCTAGCTGGCGCAAGATTATC

ATTGGTCAGGGATTTTACTCGCAGGGCCGCAGTGTGCCCATTGGGCCGGATGTTGTAGCT

CTCAAAGGAACCCAGCAGACCCTGAAATGCACTCAGAAAGGACTGATCCTTTGTGTGGAC

TATTCGGTTATGCCGTTTCGCAAAGCTGGACCTGTGTTGGATCTTGTTCAGAAGTCTGTG

AGATACCTTGACTACAGGACAACACTAAACAAACACCAATTGGACACTTTGAAGAATGAA

CTCAAAGGCCAGCGTGTCACTGTAAATCATAGGAGGACAAAGCAGAAGTACATTGTTAAA

GGTTTGACTGATAAACCTGCAAGTCAGATAACTTTTGTAGATTCTGAATCAGGACAGACC

AAGAAGCTTCTTGATTACTATTCGCAGCAGTATGGCAAGGTTATTGAGTATCAAATGCTT

CCATGCTTGGATTTGAGCAAGAGCAAGGACAAGCAAAACTATGTGCCGATTGAATTGTGT

GATCTTCTTGAAGGGCAGAGATACCCAAAAGCAAGCTTAAATAGGAATTCTGATAAAACA

CTGAAAGAAATGGCTTTGATCCCTGCCTCAAGTAGGAAGGAGGAGATTCTGGAGTTGGTG

AATGCTGACGATGGGCCTTGCAGGGGTGAAATTGCTCAGCAGTTCGGGATTTCTTTGGAT

GTACAAATGATGGAAGTCACTGGTAGGACCCTTCCTCCTCCCAGCCTAAAACTTGGCACC

TCCAGTGGCCAACCCCCCAAATTCAATATTGATCAGCCTAACTGCCAGTGGAACCTTACG

AGGAAAAGACTAGCAGAGGGCGGGGTGCTACAGTGCTGGGGCGTTGTGGACTTCAGTGCA

GATTCTGGGCAGTACGCCCTGAATGGGAACATGTTTATTGACAAGATTGTCAGGAAGTGC

TGCGACCTTGGCGTACAGATGAACCGTAACCCATGCATTGTGCAACTGTTAGATATGGAG

GTGCTATCCGATCCACATCAGCTCTTCGAGGAGCTTAACAAAGCTAAGCAGGCGGCAGCC

AGTAAGAAACAGAAGCTGCAGCTCCTCTTCTGCCCAATGTCTGATCAGCATCCTGGGTAC

AAGACGCTGAAGCTTATCTGCGAGACGCAGCTGGGGATCCAGACCCAGTGCTTCTTGAGC

TTCCTCGCGAACAAACAACAGGGACAGGACCAGTACATGTCCAACCTTGCTCTGAAGATC

AACGGCAAGATTGGAGGAAGCAACATCCAACTGTTTGGTGAATCGCTCCCGCGGATCTCC

GGCGCGCCATACATGTTCATCGGCGCCGACGTGAATCACCCATCGCCGGGGAACGTCGAG

AGCCCGTCGATTGCAGCAGTGGTGGCCTCGGTGGATCAAGGCGCCAGCAAGTACGTGCCA

AGAATCCGCGCTCAGCCTCACCGCTGCGAGGTGATCCAGCACCTCGGCGACATGTGCAAG

GAGCTCATCGGCGTGTTCGAGAAGCGGAACCGCGTGAAGCCCCAGAGGATCATCTACTTC

CGCGACGGCGTCAGCGACGGTCAGTTCGACATGGTGCTGAACGAGGAGCTGGCGGACATG

GAGAAGGCGATCAAGACCAAGGACTACTCCCCGACGATCACCGTGATCGTGGCCAAGAAG

CGGCACCACACCAGGCTGTTCCCCAAGGACCTGAACCAGCAGCAGACCAAGAACGGCAAC

GTGCTCCCCGGCACGGTGGTGGACACCGGCGTGGTCGACCCGGCGGCGTACGACTTCTAC

CTGTGCAGCCACAACGGGCTGATCGGGACGAGCCGGCCGACGCACTACTACAGCCTTCTG

GACGAGCACGGCTTCGCCTCCGACGACCTGCAGAAGCTGGTGTACAACCTCTGCTTCGTC
TTCGCCCGCTGCACCAAGCCGGTGTCGCTGGCCACGCCCGTCTACTACGCCGACCTCGCC
GCCTACCGCGGCAGGCTCTACTACGAGGGCATGATGATGTCGCAGCCGCCACCGTCTTCC
GCGGCGTCGGCGTCGTCGGCATCCTCCTCCGGCGCCGGCGCTTCCGACTTCAGGAGCTTC
CCGGCGCTGCACGAGGATCTGGTGGACAACATGTTCTTCATCTGA
>Oryza-sativa_Os04g52550
ATGGCCGGTCGCGGTGGCCGCGACCCCCGGAGAGGCTACGACGGCGGCTACGGGTACCCG
AGAGGCGGAGGAGGGCAAGGGGGTACTAACCGGGGAAGAGACGGGCAGCGCGGTGGAGGA
AGAAACGGGCCTCGCGGAGGACGCTTTCCCGGTGGAAGAGGAGTCGAGCCTCGCAGAGGC
GGCGATGTTCTGGGAGGAGGCCAAGGCGGTGGGCGGGGGACTACTGCTGGTGCTGGTGGT
CTTGTGCGAGGAGGGTCCGAGCTCGCAGTGGGAGCGGAACTGCGGCGGCGCGTGCCCACC
TGCCACGTGAACGACTTCCCGGAGTTGGGGATTGGGGGGACGCCCGTGCTCGTTCGGCGA
TGGCGCCCGCGCGACCACCGAGACCAGCACGACCACCAGAGCCAGCGTCACCACCACCGC
CACCACCACCACCAGCGCCAGCGTCACCACCACCACCACCAGCGCCAGCAGCGAAGGGGA
TCCAGGACGAGGGATCCTCGAGTTCGTCGTGGTCCGTTGCGCATTCCCTACGGTGAGGAT
GAAAAAGAAGAACCACCGGCAACCCCAATTGCTTCCAGTAACAAGAATAAAAGAGAAGAA
CCACCTACCAAACATAGGCCAATGGCAAGACCACCTGGTGGTGGAGGTCCATTATCTAAA
GGCGAGGTCAAATTGTTGGTGAACCATTTTTCAGTCGACTACCCAAAGGAATCAACCTTT
TTTCACTATGAAATACGAATCAAGCTTGGTGACGGTCCCAACAGAAAGCTCTCAAAGGCA
GAGCTTCTTACAGTCAAGAATGAGCTCTTCGAGCACGAGAGCCTTCAGGAGCTTTCGTCA
GCTGTTGCTTATGATGGAGAGAGAAATTTATACACTTGTGCTGAACTACCAGAAGACTGC
ATAGTCCCTGTGAGCAAATTCCGTGTGAAGGACAGTTCACGGACCTACATTGTATCAGTG
AAGTTGAAGAAGCCGCTGCCTTTAAGCCAACTCTTGGAGCAGCGGCCTGGGCCTAGAGAT
GTCATGCAGGGCCTTGATGTCATTGTGCGTGAGGCATCTAGCTTCGGCAAGATTGTCCTT
GGTCAGGGATTTTACCCGCAGAGCGGCAGTGAGGCCATCAGTGATAGCAATATTGTAGCT
CTCAAAGGAACCCAGCAGAGCCTTAAATGCACTCAGAAAGGGCTGATCCTGTGTGTGGAC
TATTCGGTTTTGCCATGTTGGAAAGCTGGATCTGTGTTGGACCTTGTTAAGACTATGAAG
TTCATGGAATACCCGCTCTTGGAAGACCAATTGAAAAAATTGAACAATGCGCTCAAAGGC
CTGTGTGTTACTGTAAGTCACAGGAAGACTGAGGAGAAGTACACTGTTAAAGGCTTGACA
GATAAACCTGCCGACCAGATAACTTTTAAAGACTCTAAATCAGGACAGACGACGAAGCTT
ATCGAGTACTATAAGGAGACGTATAAGAAAGAGATTGAGCATCCGATGCTTCCATGCTTG

GATTTGAGCAAGAGCAAGTCCAAACAAAACTATGTGCCGATTGAATTTTGTAATATTCCT

GAAGGGGAGAGGTATCCAGTGGCGAGGTTAGATGACAAGAAGTCTGATAATAAGGGCGAA

CAAGAGAAGCCATCTACAAAGACGACACTGAGAAAAATTTCTATAAAGGTTGCCTCTAGT

CGGAAGGAGGAGATTCTGGACTTGGTGGGGAATGCTCAAGATGGGCCTTGCAGGGGAAAA

ATAGCTCAGCGATTCAGGATTTCTTTAGATGCAGCAATGATGGAAGTCACTGGTAGGATT

CTTGCTCCCCCCACCCTAGAACTTGGCACTGGCACCTCCAGAGGCCAGACCTTCAAATTC

ACTATTCATCAGGATGACTGCCAGTGGAACTGGAAGCTTAAAAAATACGATAAACGAGTA

GTAGCACATGGCGGGACTCTTAACTGCTGGGGCGTCGTCGATTTCAGTGAAGGCGACCTA

GAGAGCAAGTTTATTGACAAGGTTGTCAGGAAGTGCTCCGCCCTTGGCATGGTCATGACC

CGTAAACCATGCTATGAGCATGTGTCAAATATGGAAGTGCTATCCGATCCAAAGAGCCTC

AGAGATGCGCTTATCGAAGCGAAGCGTGCCGCGGAGGAGGAAGACAAGAAGCTGCAGCTC

CTCTTCTGCCCGATGCTCAACCGGTGCCATGGGTACAAGACCCTGAAGTTGATGTGCGAG

ACGGAGCTGGGGATCCAGACCCAGTGCTTCTTGAGCACCGCCGCAAAACTCGACGAAAAA

CGACAGGACCAGTACATTACCAACCTTGCTCTGAAGATCAACGGCAAGATCGGGGGTAGC

AACATGCAGCTCGACCCGGACTCGATCCCAGTGGTGTCCGCCAAGGATTTCATGTTCATC

GGTGCGGACGTGAACCACCCTCCGCCTGGGAATGTCAGTAAGGACATCCCGTCCATAGCA

GCCGTGGTGGCCTCCGTTGATAAAGGCGCCAGCAAGTACGTGACAAGGATCCGCGCCCAG

TATCACCGGTGCGAGATGATCCAGAACCTCGGTGATATCTGCAAGGAGCTCATCGGCGCG

TATGAGAAGGTAAACAAGAAGAAGCCTGATAGCATCATCTACTTCCGCGACGGCGTCAGT

GACGGTCAGTTCGACATGGTGCTGAACGAGGAGCTGGCGGACATGGAGAATAAGATCATG

GTGGGTGACTACCCGAAGATCACCGTGATCGTTGCCAAGAAGAGGCACCACACGCGGCTG

TTCCCCAAGGACAGGAACCAGCGGCAGACCAAGAACGGCAACGTGCTCCCCGGCACGGTG

GTGGACACCGACGTGGTCGACCCGACGGCGTACGACTTCTACCTGTGCAGCCACAAGGGG

GAGGTCGGGACGAGCCGGCCGACGCACTACTACAGCCTTCTGGACGAGCACGGCTTCGCC

TCCGACGACCTGCAGAAGCTGGTGTACAACCTCTGCTTCGTCTTCGCCCGCTGCACCAAG

CCGGTGTCGCTGGCCACTCCCGTCTACTACGCCGACCTCGCCGCCTACCGCGGCAGGCTC

TACTACGAGGGCATGATGATGTTGCAGCCGGCGGCGTCAGCGGCATCTGCCTCCGAGGCC

ATGATGCCGGCGGCACAGCCCCAGGCGGCTGCGGCTGCGGCTGCGGCAGCGTCGCCGTCG

TCGTCAGCGGCATCCTCATCTGAGGGCATGACGGCGTCCCAGCCCCAGGCGCCGGCGGCG

GAGGCGGCATCCTCCTCCGCGGGAGCTGCCGACTTCAGGGAGTTGCCTCCGATGCACGGG

GATCTGTTGAACAACATGTTCTTCCTCTGA

>Oryza-sativa_Os06g39640

ATGGCGCCGCCCCGGCACCAGCCGGCAGCTGGGAAGGCGGGCGGCGGGCGCGGGCATGGG

CATGGGCATGGGCATGGTGGTGGTGGTGGTGGGCCGGCGGCGAGGAAGCAGCCGTTGCAG

AGTAGCATGGCGCAGCCCAAGGCGGAGACGGCGGCGGCGACGGCGGCGGTCGCGCCGCCG

GAGGGAGGGAAGAAGTGCGGCGGCGGTGGCGGGAGGCGGCGCGGCGGGCGCGGGCGCGGC

GGCAGGGCGGGTGCGGGGCCGGGGCCGGGGCTGGCGGCGGCGCCGGCCGTGGTGGTGGCG

CCCGCGGCGCGCGCCGTCATTGGGCCGCCGGTGGCGAGCAAGGGGCTGTCGTTCTGCCGG

CGGCCGGGGTTCGGGACGGTGGGCGCCCGGTGCGTCGTGAAGGCGAACCACTTCCTCGCC

GAGCTCCCCGACAAGGATCTCACCCAGTACGACGTGAAGATCACGCCGGAGGTGAGCTCC

CGGAGCGTGAACCGGGCAATCATGTCGGAGCTGGTCCGCCTCTACCACGACTCCGATCTC

GGAGGTCGCCTCCCGGCCTACGACGGCCGCAAGAACCTCTATACCGCCGGGACGCTCCCG

TTCGACGCGCGCGAGTTCGTCGTCCGCCTCACCGACGACGACGACGGCACCGGCGTCCCG

CCACGGGAGAGGGAGTACAGGGTCGCCATCAAGTTCGCCGCGCGCGCCGACCTCCACCAC

CTCCGGCAGTTCATCGCCGGGCGCCAGGCCGACGCGCCGCAGGAGGCTCTCCAGGTGCTC

GACATCGTGCTCCGTGAGCTTGCCAACCGCAGGTATGTCTCGATAGGGCGGTCGTTCTAC

TCGCCGGACATAAGGAAGCCGCAGCGGCTCGGCGATGGCCTACAGTCATGGTGTGGGTTC

TACCAGAGCATCCGGCCGACTCAGATGGGGTTGTCGCTTAACATTGATATGTCATCTACC

GCGTTCATCGAGCCGCTACCAGTGATCGAATTCGTGGCCCAAATACTAGGGAAGGATGTC

ATATCGAGGCCATTGTCGGATGCAAACAGAATCAAGATCAAGAAAGCCTTGCGGGGCGTG

AAAGTTGAAGTCACTCACCGGGGAAATGTGAGGCGGAAGTATCGCATTTCAGGGCTGACA

ACACAACCAACTCATGAACTGATTTTCCCAATCGATGACCAAATGAACATGAAATCTGTC

GTAGAGTATTTCAAGGAAATGTATGGCTTCACCATTCAGCATCCCCATCTTCCCTGCCTT

CAGGTGGGAAACCAAAAGAAGGCAAACTATCTACCAATGGAGGCCTGTAAGATTGTTGAG

GGTCAGAGATATACAAAGAGGTTGAATGAAAAGCAGATCACATCATTACTCAAGGTTACT

TGCCGTAGGCCTAGAGAACAGGAGATGGATATTCTACAGACAGTTCAACAAAATGGATAT

GAGCAAGACCCTTATGCAAAAGAATTTGGAATCAACATTAGTGAGAAGCTAACCTCAGTT

GAAGCTCGGGTCCTTCCTGCACCTTGGCTGAAATATCATGATACTGGGAAGGAAAAGGAG

TGCTTGCCACAAGTTGGTCAGTGGAACATGGTGAACAAGAAAGTGATAAATGGGTGCAAG

GTGAATCATTGGGCTTGTATAAATTTCTCAAGGAGTGTGCAAGAAACTACTGCTCGGGGA

TTCTGCCAGGAGTTGGCACAAATGTGTCAGATATCCGGCATGGAATTCAACAGTGAGCCT

GTGATACCAATATACTCAGCTAGACCAGATCAAGTAGAGAAGGCGCTTAAGCATGTGTAT

AATATGTCATTAAACAAGCTCAAGGGAAAAGAGCTTGAGCTTCTTTTGGCCATCCTCCCT
GACAACAATGGTTCTTTATATGGTGATATAAAACGTATATGTGAAACTGACTTGGGGTTG
ATATCACAATGTTGCTTAACCAAACATGTTTTCAAGATCAGCAAGCAGTACCTGGCAAAT
GTCTCACTTAAAATCAATGTTAAGATGGGAGGAAGAAACACCGTGCTGCTGGATGCAATA
AGTTGGAGGATTCCTTTGGTCAGTGACATACCAACTATTATATTTGGTGCAGATGTCACG
CATCCTGAAACCGGGGAGGACTCTAGCCCATCCATTGCTGCTGTTGTTGCATCTCAAGAC
TGGCCAGAAGTTACAAAGTATGCTGGATTGGTGTGTGCTCAGGCTCATCGGCAAGAGCTC
ATTCAAGATCTTTACAAGACATGGCATGATCCTCAAAGAGGCACCGTAACAGGAGGCATG
ATCAGGGAGCTCTTAATATCCTTCAGGAAGGCCACTGGGCAAAAGCCATTGAGAATAATT
TTCTACAGGGATGGTGTCAGTGAAGGCCAATTCTACCAAGTTCTCCTCTATGAGTTGGAT
GCTATCCGCAAGGCATGTGCATCTCTAGAACCAAATTATCAGCCTCCTGTAACATTCGTG
GTTGTCCAAAAGCGTCACCATACAAGACTCTTCGCAAACAATCACAAAGACAGAAGTAGC
ACGGACAAAAGTGGAAACATTTTGCCTGGAACTGTTGTTGATTCAAAGATCTGCCACCCA
TCAGAGTTTGATTTCTACCTCTGTAGCCATGCTGGAATTCAGGGAACAAGTAGGCCAGCT
CACTACCACGTCCTTTGGGATGAGAACAATTTCACTGCAGACGAAATGCAGACATTGACA
AACAACCTTTGCTACACTTATGCACGGTGCACACGCTCTGTTTCTGTTGTTCCACCTGCA
TACTATGCGCATCTGGCTGCGTTTCGAGCGCGGTTCTACATGGAGCCAGAGATGTCGGAG
AACCAGACGACGTCGAAGAGCTCCACTGGGACGAACGGAACCTCGGTGAAGCCATTGCCT
GCGGTGAAGGAGAAGGTGAAGAGGGTGATGTTCTACTGTTGA

>Oryza-sativa_Os06g51310

ATGGGATCAAGGAGACCAAGACTGCCTGGGTTTGGTGAGGACTGTGAGCCTCGTGGTGGT
GGGAGAGGAGGAGGTGGCCGTGGCCGTGGCAGCTACTACCCACAGGCACAGCAATACCAC
CCACAAGGACATGGTGGCCGTGGAGGAGCAGGCTACTACCATGGTGCTGCTCCTCAACCT
CGCGGCGCAATGGTGGTGCAGCAATGGCGTCCTGCTACTGCTGCTGCTGAGCATTTGGGC
CATCAACAGCCCTACAACAGCAGTGTGAGACCACAGCACTATTATGGTCCGTCTGCCATA
GCTCCCGAGCTGCTCCAAGCAATGGATGCTCCACATGAGCCTCCTGCCAATGTCTCCTCA
CCAGAAGCAGCCTCTCCGGAGGCATCATCACCACGGTCTCTTGCTCTTGAGGTCACAGAG
CAGCTTCAGGACTTGTCTGTGCAGTACCAATTAAGTGAGAGCCAGGAAGAGATTGTCCAA
CATGTTCCTGTGTCCACCAAATCATTTAAATTTCCTCACCGCCCTGGAAGTGGGAGTATT
GGAACCAGATGTTTAGTGAAGGCGAATCACTTCTTTGCTCAACTGCCAGACAAGGATCTT
CATCAGTATGACGTTTCAATCACCCCGGAGCTTACATCACGAATTCGGAGCCGTGCTGTG

ATGGAAGAGTTGGTGAGACTGCACAAGATGTCATACTTGGGAGGACGTCTTCCAGCCTAT

GATGGTAGGAAGAGCCTTTACACGGCCGGTCCATTGCCGTTTACTTCAAAAGAATTTCGC

ATCTCTTTGCTTGAGGAAGATGATGGTTCTGGTTCGGAGAGGCGTCAGAAAACATATAAC

GTGGTGATTAAGTTTGCTGCAAGAGCTGATCTTCACCGTCTTGAGCAATTTCTAGCTGGA

AGGCAGGCAGAGGCTCCCCAGGAGGCCTTGCAAGTTCTTGATATTGTCTTGCGAGAGTTG

CCAACAGCAAGATATGCACCCTTTGGTCGATCCTTCTTCTCTCCTGACCTGGGGAGGAGA

CGATCCCTCGGTGAGGGACTAGAAACCTGGCGTGGGTTTTATCAGAGCATTCGTCCTACT

CAAATGGGCTTGTCACTGAATATTGATATGTCGGCAACTGCTTTCTTTGAGCCGTTACCA

GTCATAGATTTTGTCATACAGCTTTTAAATACTGACATCCGCTCGAGGCCCTTATCAGAT

GCTGAGCGTGTCAAGATCAAGAAGGCCTTAAGAGGAGTGAAGGTAGGAGTTACTCACCGT

GGCAACATGCGCCGGAAGTATCGGATATCTGGTTTGACATCTCAGGCAACTCGGGAACTG

ACTTTTCCTGTTGATCAAGGAGGCACAGTGAAATCTGTTGTACAATATTTTCAAGAGACA

TATGGATTTGCGATCCAGCATACCTATCTTCCTTGTCTGCAAGTTGGCAATCAGCAGCGT

CCAAATTACCTACCAATGGAGGTCTGCAAAATAGTGGAAGGACAGAGGTACTCCAAGAGA

CTGAACCAGAATCAGATAAGGGCTCTTTTAGAGGAGACATGTCAGCGCCCACACGATAGG

GAGCGTGACATAATTCAGATGGTGAATCACAACTCCTACCATGAAGATCCTTATGCAAAG

GAGTTTGGCATTAAGATCAGCGAGCGTCTGGCCTTGGTTGAGGCACGGATTTTACCTGCC

CCTCGGCTCAAGTATAATGAGACTGGCAGAGAGAAGGATTGCTTGCCTAGAGTTGGTCAG

TGGAATATGATGAACAAGAAAATGGTAAATGGTGGTAGAGTCAGGAGCTGGATATGTGTC

AATTTTGCTCGAAATGTGCAAGAGAGTGTTGCTAGTGGATTTTGTCGTGAACTGGCCCGC

ATGTGCCAGGCCTCAGGAATGGACTTTGCTTTGGAGCCTGTTCTTCCATCTATGTATGCA

CGTCCTGATCAAGTGGAACGAGCTCTGAAAGCCAGGTTCCATGATGCAATGAACATACTT

GGGCCACAGCACAAGGAGCTCGATTTACTTATCGGACTGCTTCCTGATAACAATGGTTCT

CTTTATGGTGATTTGAAGCGTATATGCGAAATTGACCTTGGATTGGTTTCCCAGTGCTGT

TGCACAAAGCAAGTGTTTAAAATGAACAAACAAATCCTAGCAAATCTTGCTCTGAAGATA

AATGTGAAGGTTGGGGGAAGGAACACTGTACTGGTTGATGCAGTGTCGAGACGCATTCCG

TTGGTAACTGACAGGCCTACTATTATATTCGGTGCTGATGTCACCCATCCTCACCCTGGC

GAAGATAGCAGCCCATCCATTGCTGCTGTTGTGGCCTCCCAAGATTGGCCTGAAGTGACA

AAGTATGCTGGTTTAGTTTCTGCTCAATCTCACAGACAAGAGTTAATAGATGATCTGTAT

AACATCACGCATGATCCTCATAGAGGGCCCATCTGCGGTGGAATGGTCAGGGAACTTCTT

ATATCCTTCAAAAGATCAACTGGTCAAAAGCCTCAACGGATAATATTCTATAGGGATGGT

GTTAGTGAAGGGCAATTTTACCAGGTTCTATTGCATGAGCTTGATGCAATCCGAAAGGCT

TGTGCATCACTCGAAGCAAATTACCAACCGCAGGTGACTTTCATTGTGGTTCAGAAGCGC

CACCACACGAGATTATTTGCACACAACCACAATGATCAGAATTCAGTCGACAGGAGTGGG

AACATATTGCCTGGTACTGTTGTTGACTCCAAGATTTGCCATCCTACGGAGTTTGACTTC

TTCTTGTGCAGCCATGCTGGAATCAAGGGCACAAGCCGTCCCGCTCATTACCACGTCCTG

TGGGATGAAAACAACTTCACAGCTGATGCGTTGCAGACCCTCACCAACAACCTCTGTTAC

ACTTATGCGAGGTGCACACGATCCGTATCTATTGTTCCACCAGCATACTATGCTCATCTG

GCGGCGTTCAGAGCCCGTTTCTACATGGAGTCAGATAGCTCGGACAGTGGTTCAATGGCG

AGTGGTCGTGGAGGAGGTTCGTCTACATCGCGCAGCACTCGTGCTGCAGGTGGTGGAGCC

GTCAGGCCCCTTCCTGCACTCAAGGACAGCGTCAAGAATGTCATGTTCTACTGTTAG

>Oryza-sativa_Os07g09020

ATGGCGTCACGCGGCGGCGACGGCCTCGTCGGAGGAGGGCGCGGGCCCCTCGGTGGGCGG

GACGGTAGAGGGCGCGGCCCGGCCGGTGGGCGAGGCGGCGGAAGGGGAGGCGGCCACCCG

CAGCAGCAGCAGCAGCAGCAGCCGGGCTACGGCCGCGGCGACGGGGGCGGCCGTGGTCCT

GCCCCTGCTGCTGGTGGTGTCGTTGGTCGAGGAACTGGAGGCGGCGGCGGCGGCGGGCGC

GGGGACGGCGGACGCGGCCGTGGCCGTGGCGGCGGCGGCGGAGATGGCGTTCGCCCTGCT

ATGGCCGCGGCTCCGGCGGCGTCGACTCCTGGTCCGGTTGCTGTTGCTGCTCGGAGTACT

CCTCCTCCTACTCCCGCGGTCCAGATCCCCGCGGTGGCGTCGTCGTCGTCAGCGCAGCCA

GCGGCGGCGGCGCAGCCTCCTCCGGCAGCCGCGGCGGTTTCTGCCCTCGCGAGGGACGTT

GGGAGGCAGCTGGCTGTCGTGGCGGGCGGCGGCCGCCCCGCTCCCCCCGCGGCGCCGCCG

GCGCCCATCCCGGTGTCGAGCAAGGGCGTCGCGCCACCGTCGCGGCCGGGGTTCGGGACG

GTGGGGGAGAGGATCGTGGTGCGCGCGAACCATTTCCTCGTCCGCGTCTCCGACAACGAC

ATGATCTACCTCTACGACGTGAGTTTGAGTCCCCCACCAAAGACCAGGCGCATCAACAGG

GTAGTGATGTCTGAGCTGGCCAGGTTGCACCGTGAGTCGCATCTCGGTGGCATAAGCTTC

GCGTACGACGGAAGCAAGGCCCTGTACACTGCAGGAAAACTGCCGTTCGACTCCATGGAC

TTCAAGATCAAGCTGGGCAAAGAGCTCAGGGAAATCGAGTACAAGGTGACAATTCGACGT

GCTGGCCAAGCAGATCTGCACCACCTGCACGAGTTCATCGCCGGCCGGCAGAGGGACTCT

CAGCAGCAAACGATCCAAGCACTGGACGTTGTACTGAGGGAGTCACCTTCTCTGAACTAT

GTCATCGTTTCTCGGTCGTTCTACTCCACTATGTTCGGCCGACAAGACATTGGTGATGGG

CTGGAATGCTGGAAAGGATACTATCAGAGCCTGCGCCCAACTCAGATGGGACTCTCATTG

AACATAGACATATCCTCTACCCCATTCTTCAAACCTATCAGCGTGGTAGAATATGTCAAG

AATTGTCTGGGCACACCTACTAATGCTAATGGCCCTGACCCTAGGCGGCCTCTTTCTGAC

ATTGATCGCCTGAAGGTTAAGAAAGCACTACGGGGAGTTCGTGTTGAAACAACACACCAG

GGGAAGAGCAGCAAGTACAAGATCACTACGATTACATCTGAGCCACTGAGTCAGCTGAAC

TTTTCTATGGATGGAACTACCCAGACTGTTATTCAGTACTTCTCGCAGCGGTACAAATAC

AGGCTGCAGTACACGTCTTGGCCCTGTCTGCAATCCGGCAACCCTTCTAACCCTATATAT

TTGCCAATGGAGGTATGCACCATTGTAGAAGGGCAAAGATACTCCAAGAAGCTCAATGAC

AAACAAGTGACTGGCCTCCTGAGAGCAACATGCCAGCCTCCCCAGAAAAGGGAGCAGAAA

ATCATTGAGATGGTTCAACACAACAACTACCCGGCTGATAAGGTGGTGAGTGATTTTAGA

ATTAATATTTCCAATCAGATGGCCACTATGCCAGCTCGCGTGCTGCCTGCGCCCACGCTG

AGATACCATGACTCTGGAAAGGAGAAAACTTGTAATCCCAGAGTTGGGCAATGGAATATG

ATCAATAAGAAAATGGTTGGTGGAGCTGTGGTTCAAAAGTGGACTTGCGTGAATTTTTCA

CGCATGCATATTGATGCCGTGCACAGACTATGCGGCGAACTAGTTTATACATGCAATGCT

ATTGGCATGGTTTTCAATGAAATGCCAGAGATAGAGGTGGGGTCAGCTGCTCCTAATAAC

ATCGAAGCTGCCCTGAGCAACATTCACACAAGGGCTCCTCAACTCCAGCTGCTCATTGTG

ATTCTCCCAGATGTTAATGGGTATTATGGAAGAATTAAGAGGGTGTGTGAGACTGAACTT

GGGATAGTATCCCAGTGCCTCAAGCCAGGCCGCAAGCTCTTGAGCTTAGACAGGCAGTTC

CTGGAAAATGTCTCACTCAAAATCAATGTCAAGGCTGGAGGACGCAACTCAGTTCTTCAG

AGACCTCTTGTACCCGGTGGGCTTGAAAACACAACAATAATTTTTGGTGCCGATGTCACC

CATCCTGCTTCTGGAGAGGACTCATCGGCGTCGATCGCAGCTGTGGTGGCCTCCATGGAC

TGGCCTGAGATCACCAAGTACAAAGCCCTCGTCTCTGCCCAGCCACCTCGGCAGGAGATT

ATACAAGATCTCTTCACCATGACTGAAGTTGCGCAGAATGCTGATGCTCCAGCACAGAAG

GCTGAAGGTTCGAAGAAGAATTTCATATGCGGCGGAATGTTCAGGGAGTTGCTTATGTCA

TTCTACAGTAAGAATGCTAAACGTAAGCCTCAAAGGATAATATTTTACAGGGATGGTGTA

AGTGATGGACAATTCCTTCATGTTCTGCTCTATGAGATGGACGCAATCAAGAAGGCTATT

GCATCTTTGGACCCAGCATACAGGCCCCTGGTGACATTTGTGGTTGTCCAGAAGAGGCAC

CACACAAGGCTCTTCCCTGAGGTGCATGGAAGGCAAGATCTGACGGACAGAAGTGGAAAT

GTTCGTCCAGGAACCGTGGTTGACACTAACATTTGCCACCCTAGTGAGTTCGACTTCTAC

CTGTGCAGCCATGCCGGAATCCAGGGAACAAGCAGACCAACCCACTACCACGTTCTCCAT

GACGAGAACCGTTTTAGTGCCGATCAGCTGCAGATGCTCACTTACAATTTGTGTTACACC

TACGCTCGATGCACCCGGTCTGTCTCTGTTGTCCCTCCAGCCTACTACGCTCACCTGGCA

GCATTCCGGGCGAGGTACTACGATGAGCCCCCCGCCATGGACGGAGCTTCGTCGGTCGGC

AGCGGCGGCAACCAGGCGGCGGCCGGCGGCCAGCCACCGGCGGTGCGCCGCCTCCCGCAG

ATCAAGGAGAACGTCAAGGACGTGATGTTCTACTGCTGA

>Oryza-sativa_Os07g28850

ATGGCGAGCCGAGGAGGAGGCCAGCACCAGCGCCACCAGCAGCAGCAGCAGCAGCCCGGC

GGGTACGGACGCGGCGGCGGCGGCGGCCGCGGGCGCGGCCGGGACGGGGCGCCGTACTCG

GGTGGTCGTGGGCGCGGTCAGGACGGATCGTACCCTGGCGGCCGCGGTGGGGGCTATGGC

GGTGGTGGTGGTGGAGGTGGGCCGCCGTACTATGGCGGAGGAGGTGGTGGTGGTGGAGGA

GGAGGAGGCCAAGGGCGTGGGTACTATGATGATGGCGGCGATGGCCGCGGGTACCAGCGG

GGCATGGAGGGAGGCGGAGGCCGTGGAGGTTATCGCGGGGACGGCGATGGTGGCTACGGA

CGAGGCGGTGGCGGTTATCACGGGGACGGTGAGCGTGGCTACGGCCGAGGCGGTGGCGGT

GGCGGTGGAGGCGGCGGCGGCTATCGTGGGGATGACGAGGGTCGTAGCAGCTACGGCCGA

GCCCGTGGCGGTGGCGGTGGCGGTGGCGGCTATCATGGGGACGGCGAGGCTGGATACGGC

CGTGGACGCGGCGGCAGGGACTACGATGGCGGCCGCGGCGGCGGAGGAAGAAGAGGAGGA

CGCGGCGGCGGTGGTTCGAGCTACCACCAGCAGCCGCCCCCCGATCTGCCCCAAGCTCCG

GAGCCGCGCCTCGCCGCGCAATACGCCCGCGAGATCGACATTGCCGCGCTCCGGGCGCAG

TTCAAGGGGCTGACCACCACCACCCCCGGCGCCGCGTCGTCGCAGTTCCCGGCGCGGCCT

GGGTTCGGCGCCGCCGGAGAGGAGTGCCTCGTGAAGGTCAACCACTTCTTCGTCGGCCTC

AAGAACGACAACTTCCACCACTACGACGTGGCGATCGCGCCGGATCCGGTGCTGAAGGGC

TTGTTCCGCACGATCATCTCGAAGCTGGTGACGGAGCGGCGGCACACCGACTTCGGCGGC

CGCCTCCCCGTCTACGACGGCCGCGCCAACCTCTACACCGCCGGCGAGCTCCCGTTCAGG

AGCAGGGAGCTCGAGGTGGAGCTGTCCGGCAGCAGGAAGTTCAAGGTGGCCATCCGGCAC

GTCGCGCCGGTCAGCCTGCAGGACCTGCGGATGGTCATGGCCGGCTGCCCCGCCGGCATC

CCGTCGCAGGCGCTGCAGCTGCTCGACATCGTGCTGCGCGACATGGTGCTCGCCGAGCGC

AACGACATGGGGTACGTTGCGTTTGGTCGGTCTTACTTCTCACCGGGGCTCGGATCGAGG

GAACTCGACAAGGGCATCTTTGCGTGGAAGGGGTTCTACCAGAGTTGCCGGGTCACGCAG

CAGGGCCTTTCTCTGAACATAGACATGTCTTCGACTGCTTTCATTGAACCTGGTCGGGTG

CTGAATTTTGTAGAAAAAGCTATTGGACGTCGAATCACTAATGCTATTACTGTGGGATAT

TTTTTGAACAATTATGGGAATGAATTGATGAGGACCCTTAAGGGTGTTAAGGTTGAAGTC

ACTCACCGAGGAAATCTACGCAAGAAGTACCGCATTGCTGGCTTCACCGAGCAGTCTGCA

GATGTTCAGACGTTCACATCATCTGATGGTATCAAGACTGTCAAGGAGTATTTCAACAAA

AAATACAATCTGAAGTTAGCTTTTGGTTATCTTCCATGCCTGCAAGTTGGCAGCAAGGAG

AGACCGAATTACCTGCCCATGGAGCTTTGCAATATAGTTCCTGGACAACGATACAAGAAC

CGGCTCAGTCCGACACAGGTTTCCAATCTGATTAACATAACCAACGATCGTCCTTGTGAC

CGTGAGAGCTCCATTCGTCAGACTGTTAGCAGCAACCAGTATAACAGTACGGAACGCGCA

GATGAGTTTGGCATAGAAGTTGACTCTTATCCTACTACTTTAAAGGCTAGAGTTTTGAAA

GCTCCAATGCTGAAGTACCATGATTCTGGAAGGGTGAGAGTATGCACGCCAGAGGATGGG

GCGTGGAACATGAAAGACAAGAAAGTAGTTAACGGTGCTACAATTAAAAGCTGGGCATGT

GTCAACTTGTGCGAGGGTTTGGATAATCGTGTTGTTGAAGCATTCTGCCTTCAATTGGTC

AGAACGTCCAAAATAACTGGACTGGACTTTGCGAATGTGAGCCTTCCAATATTGAAAGCT

GATCCTCATAATGTTAAAACTGATCTTCCTATGCGCTATCAGGAAGCATGCAGCTGGTCG

AGGGATAACAAGATTGACCTCCTACTTGTTGTAATGACAGATGATAAAAATAATGCCAGC

TTATATGGTGACGTTAAAAGAATCTGTGAAACAGAAATCGGTGTATTGTCACAGTGTTGT

CGAGCGAAGCAAGTCTACAAGGAGAGGAATGTTCAGTACTGCGCAAATGTTGCTCTTAAG

ATCAATGCCAAGGCTGGAGGAAGGAACTCGGTATTTCTTAATGTAGAAGCAAGTTTACCG

GTTGTTTCAAAGAGCCCAACTATTATATTTGGTGCTGATGTTACCCATCCTGGGTCCTTT

GATGAAAGTACCCCTTCCATTGCTTCGGTTGTTGCTTCCGCAGACTGGCCTGAGGTGACC

AAGTATAATTCTGTTGTTCGTATGCAAGCTTCTCGTAAGGAGATTATACAAGATCTTGAT

AGCATTGTTAGGGAACTTCTCAATGCATTCAAAAGGGACTCCAAGATGGAGCCGAAGCAG

CTCATTTTCTACAGGGACGGCGTAAGCGAGGGTCAGTTCCAGCAAGTTGTAGAGAGCGAA

ATACCGGAGATAGAAAAGGCTTGGAAGTCTCTGTATGCTGGCAAGCCACGAATTACCTTC

ATAGTGGTGCAGAAGAGGCATCATACAAGGCTGTTCCCCAACAATTACAATGATCCACGC

GGCATGGATGGGACTGGAAATGTTCGTCCAGGCACAGTAGTTGATACAGTGATCTGTCAC

CCTCGAGAGTTTGATTTCTTCCTGTGCAGCCAAGCCGGGATCAAAGGGACAAGCCGTCCT

AGCCATTACCATGTGCTGCGCGACGACAACAACTTCACCGCAGATCAGCTTCAGTCTGTC

ACAAACAACCTGTGCTACTTATATACAAGCTGCACTCGCTCGGTGTCTATTCCACCTCCT

GTTTACTACGCTCATAAGCTCGCATTCCGCGCTCGTTTCTACCTCACCCAAGTTCCCGTC

GCCGGTGGAGATCCAGGTGCTGCTAAGTTCCAGTGGGTACTTCCAGAGATTAAGGAAGAG

GTGAAAAAGTCCATGTTCTTTTGCTAG

>Zea-mays_GRMZM2G007791_T01 cds:_protein_coding

ATGGAGTACGAGCGCGGTGGCGGAGGCGGCCGCGGCCGCGGGCGGGGCCGTGGAGGCGGC

GGAGGAGGAGGGCGCGGAGGTGGCGGCGGATACGGGCGGCAGCACGGAGGCGGCGGCGAC

GCCCGCGGAGGCCGAGACGAGTACGGCGGCGGAGGAGGAGGGTACGGATACGACGAAGGA

GAAGGCTATGGCGGGGGTCGTAGAGGCGGCGGCGGAGGGTACGGGTACGACGTAGGGGGC
CGTAGAGGCGGCGGCGGCTACCACCAAGGGCCTCGCGGAGGCTATGGCACGGGCGGCGGC
GGCTACCACCAAGGGCCTCGCGGAGGCTATGGCACGGGCGGCCGCGGCGGAAACGCGTGG
GCGCCGGCTCCTGGCGCGGGGAGAGGTCGCGGAGTTGGCGGTGGCGCGGCCGAGTACGCC
CCCGTCAGGGGGCCAGCGCCCGCGCAGGCGCCGGTGGCGAGTCCGCTTGCGCCCAAGGAC
AAGGAGGCGCCGCGTTCGTCGGGATCCGTCGAACGCATCGCATCCAGCGAAATGGCCAGA
GTAGAACCAATAGGATCAACACTAGCTGCCACATCTTCTGTTAATACACGTGTGCCAATG
CAGAGACCTGATTCTGGAGGTTCATTATCTCAAGCAACGGTTAAACTTTTGGTGAATCAC
TTTATTGTCAGCTACCGAAAGGTGACAACTATTTTTCATTATGACATAAACATCAAGCTT
GATGAAGCTTCGTCTAATGCTTCGGGCAAAGAGCTATCCAAGGCAGAATTTCTCTCTGTC
AAGGATGAGCTCTTCAGGGAAAGCAGTTTACGGCGTCTTTCCTCATGTGTTGCTTATGAT
GGTGGAAGAAATCTGTATACTTCTGCTGAACTGCCTGCAGGTTTATTTCGTGTGAGAGTT
CGATCAAAGACCTACATTGTATCAGTAGATTTGAAGAAGCAGCTGCCATTAAGTCAACTC
TCAGATTTACCTGTGCCTAGAGAGGTCTTGCAGGGCCTTGATGTTGTTGTGCGTGAGGCC
TCAAGATGGAACAAGATTATCCTTGGTAGAGGATTTTACTCACCAAGCAGCAGTATAGAC
ATTGGGCAGGGTGCTGTAGCTATGAAAGGAACCCAGCAGTCCCTTAAATCCACTCAGCAA
GGGTTGATCCTGTGTGTTGACTATTCTGTCATGCCGTTTTACAAAGCTGGACCGGTGATG
GATCTTGTTCAGAAATTAGTGAGGTACCTTGATTATCGGACAACTTTGAACAAGAGGCAA
ATGGAAAATCTGGTTGATGAGCTTAAAGGCCGACGTGTAACTGTGATTCATCGGAGGACT
AATCAGAAGTACACAGTGCAAGGTTTGACACCCTTACCTGCCAGCCAGATGACCTTTGTG
GATGCTGAATCCGGCCAAACACGGAGGCTCGTGGATTATTATGCTCAGAAACATGGCAAG
GTGATTGAGTATCAGATGCTGCCATGCTTGGATTTGAGCAAGAGCAAGGACAAACCGAAT
CATGTTCCAATTGAGCTCTGCACTCTTCTTGAAGGACAGAGGTTTCCAAAAGCAAACTTG
AATCAGAATTCTGAGAGGATACTAAAAGGAAGTGCTCTAATCCGTGCATCTGACCGGAGG
AAGGAGATTCAAAACTTGGTGAATGCTTCGGATGGACCGTGCAGAGGAGAAATTGCACAG
CAATTTGGGATTTCCTTGGATGTACGAATGACAGAAGTCACGGGTAGGATCCTTCCTCCA
CCAAACCTCAAACTTGGGGCATCCAATGGGCAGACCTCCAAATTGAGTATCGATCATGGC
TGCCAGTGGAATCTTGTGAAGAAGAGACTAGTAGAGGGCCGGGTTCTTCAGTGCTGGGGC
ATCGTCGACTTCAGTGCTGAGCCGTCTGGCTCTGGCGCCCGTCAGGAGCCCCTCGATACA
AGGATGTTTGTTGAGAAGATTGTGAGGAAGTGCTGTGAGCTTGGTATCCGTATGAACCCT
AATCCATGCTTCGTGCACATAACAAGGATGGCAGTGCTCTTCGATCCACATGGACTACAT

GAAGAGCTAAACAAAGCAAAACAAGCTGCAGTGAGCAAGAAGCAGAGGTTGCAGCTCCTT

TTCTGCCCGATGTCCGAGCAGCATTCGGGGTACAAGACACTGAAGCTGATTTGTGACACA

CAGCTGGGGATCCTGACCCAGTGTTTACTGAGCGACCGCGCAAACAATCGAAAGGGACAG

GACCAGTACATGACGAATCTTGCTCTAAAGATCAACGGCAAGCTTGGGGGCAGCAACGTT

CAGCTGTTTGACTCGCTCCCACGGGTCGGTGGTGGGGTACCTTTCATGTTCATCGGTGCT

GACGTTAACCACCCGTCCCCCGGTAACGTGGAGAGCCCATCGATCGCAGCCGTGGTCGCC

TCTGTCAACTCTGGTGTCAACAAGTATGTGACCAGAATCCGTGCCCAGCCGCACCGCTGC

GAGGTGATCCAGCAGCTTGGTGAGATCTGCCGGGAGCTCATTGGAGTCTTTGAGAAGCAG

AACCGCGTGAAGCCGCAGAAGATCATCTACTTCCGTGATGGCGTGAGCGACGGGCAGTTC

GATATGGTCCTGAACGAGGAGCTGGCTGACCTGGAGAAGGCGATCAAGGTGAATGGCTAT

GCGCCAACCATCACCGTGGTCGTGGCCAAGAAGCGGCACCACACTCGGCTGTTCCCCAGG

GACGAACAGCAGCCGCAGACGAAGACCGGGAACGTGCCGCCTGGCACGGTGGTGGACACG

GGCGTGGTGGACCCGTCCGCGTACGACTTCTACCTGTGCAGCCACACTGGGATTCTGGGG

ACGAGCAGGCCGACGCACTACTACACCCTGGTGGACGAGCACGGCTTCGGCTCCGACGAC

CTGCAGAAGCTGATCTACAACCTGTGCTTCGTGTTCGCGCGGTGCACCAAGCCGGTGTCG

CTGGCGACGCCCGTCTACTATGCCGACCTCGCGGCCTACCGTGGCAGGCTCTACTACGAG

GCTGCCATGATGGCGTCCCAGGCCCAGCGAGGGGGGTCCTTTGACGTCACTAACTTCCCG

AGGCTGCACAAGGATGTGGAGGACAACATGTTCTTCATCTGA

>Zea-mays_GRMZM2G031147_T01 cds:_protein_coding

ATGGAGTACGAGCGCGGTGGCGGAGGCGGCCGCGGCCGCGGGCGGGGCCGTGGAGGCGGC

GGAGGAGGAGGGCGCGGAGGTGGCGGCGGATACGGGCGGCAGCACGGAGGCGGCGGCGAC

GCCCGCGGAGGCCGAGACGAGTACGGCGGCGGAGGAGGAGGGTACGGATACGACGAAGGA

GAAGGCTATGGCGGGGGTCGTAGAGGCGGCGGCGGAGGGTACGGGTACGACGTAGGGGGC

CGTAGAGGCGGCGGCGGCTACCACCAAGGGCCTCGCGGAGGCTATGGCACGGGCGGCGGC

GGCTACCACCAAGGGCCTCGCGGAGGCTATGGCACGGGCGGCCGCGGCGGAAACGCGTGG

GCGCCGGCTCCTGGCGCGGGGAGAGGTCGCGGAGTTGGCGGTGGCGCGGCCGAGTACGCC

CCCGTCAGGGGGCCAGCGCCCGCGCAGGCGCCGGTGGCGAGTCCGCTTGCGCCCAAGGAC

AAGGAGGCGCCGCGTTCGTCGGGATCCGTCGAACGCATCGCATCCAGCGAAATGGCCAGA

GTAGAACCAATAGGATCAACACTAGCTGCCACATCTTCTGTTAATACACGTGTGCCAATG

CAGAGACCTGATTCTGGAGGTTCATTATCTCAAGCAACGGTTAAACTTTTGGTGAATCAC

TTTATTGTCAGCTACCGAAAGGTGACAACTATTTTTCATTATGACATAAACATCAAGCTT

GATGAAGCTTCGTCTAATGCTTCGGGCAAAGAGCTATCCAAGGCAGAATTTCTCTCTGTC

AAGGATGAGCTCTTCAGGGAAAGCAGTTTACGGCGTCTTTCCTCATGTGTTGCTTATGAT

GGTGGAAGAAATCTGTATACTTCTGCTGAACTGCCTGCAGGTTTATTTCGTGTGAGAGTT

CGATCAAAGACCTACATTGTATCAGTAGATTTGAAGAAGCAGCTGCCATTAAGTCAACTC

TCAGATTTACCTGTGCCTAGAGAGGTCTTGCAGGGCCTTGATGTTGTTGTGCGTGAGGCC

TCAAGATGGAACAAGATTATCCTTGGTAGAGGATTTTACTCACCAAGCAGCAGTATAGAC

ATTGGGCAGGGTGCTGTAGCTATGAAAGGAACCCAGCAGTCCCTTAAATCCACTCAGCAA

GGGTTGATCCTGTGTGTTGACTATTCTGTCATGCCGTTTTACAAAGCTGGACCGGTGATG

GATCTTGTTCAGAAATTAGTGAGGTACCTTGATTATCGGACAACTTTGAACAAGAGGCAA

ATGGAAAATCTGGTTGATGAGCTTAAAGGCCGACGTGTAACTGTGATTCATCGGAGGACT

AATCAGAAGTACACAGTGCAAGGTTTGACACCCTTACCTGCCAGCCAGATGACCTTTGTG

GATGCTGAATCCGGCCAAACACGGAGGCTCGTGGATTATTATGCTCAGAAACATGGCAAG

GTGATTGAGTATCAGATGCTGCCATGCTTGGATTTGAGCAAGAGCAAGGACAAACCGAAT

CATGTTCCAATTGAGCTCTGCACTCTTCTTGAAGGACAGAGGTTTCCAAAAGCAAACTTG

AATCAGAATTCTGAGAGGATACTAAAAGGAAGTGCTCTAATCCGTGCATCTGACCGGAGG

AAGGAGATTCAAAACTTGGTGAATGCTTCGGATGGACCGTGCAGAGGAGAAATTGCACAG

CAATTTGGGATTTCCTTGGATGTACGAATGACAGAAGTCACGGGTAGGATCCTTCCTCCA

CCAAACCTCAAACTTGGGGCATCCAATGGGCAGACCTCCAAATTGAGTATCGATCATGGC

TGCCAGTGGAATCTTGTGAAGAAGAGACTAGTAGAGGGCCGGGTTCTTCAGTGCTGGGGC

ATCGTCGACTTCAGTGCTGAGCCGTCTGGCTCTGGCGCCCGTCAGGAGCCCCTCGATACA

AGGATGTTTGTTGAGAAGATTGTGAGGAAGTGCTGTGAGCTTGGTATCCGTATGAACCCT

AATCCATGCTTCGTGCACATAACAAGGATGGCAGTGCTCTTCGATCCACATGGACTACAT

GAAGAGCTAAACAAAGCAAAACAAGCTGCAGTGAGCAAGAAGCAGAGGTTGCAGCTCCTT

TTCTGCCCGATGTCCGAGCAGCATTCGGGGTACAAGACACTGAAGCTGATTTGTGACACA

CAGCTGGGGATCCTGACCCAGTGTTTACTGAGCGACCGCGCAAACAATCGAAAGGGACAG

GACCAGTACATGACGAATCTTGCTCTAAAGATCAACGGCAAGCTTGGGGGCAGCAACGTT

CAGCTGTTTGACTCGCTCCCACGGGTCGGTGGTGGGGTACCTTTCATGTTCATCGGTGCT

GACGTTAACCACCCGTCCCCCGGTAACGTGGAGAGCCCATCGATCGCAGCCGTGGTCGCC

TCTGTCAACTCTGGTGTCAACAAGTATGTGACCAGAATCCGTGCCCAGCCGCACCGCTGC

GAGGTGATCCAGCAGCTTGGTGAGATCTGCCGGGAGCTCATTGGAGTCTTTGAGAAGCAG

AACCGCGTGAAGCCGCAGAAGATCATCTACTTCCGTGATGGCGTGAGCGACGGGCAGTTC

GATATGGTCCTGAACGAGGAGCTGGCTGACCTGGAGAAGGCGATCAAGGTGAATGGCTAT
GCGCCAACCATCACCGTGGTCGTGGCCAAGAAGCGGCACCACACTCGGCTGTTCCCCAGG
GACGAACAGCAGCCGCAGACGAAGACCGGGAACGTGCCGCCTGGCACGGTGGTGGACACG
GGCGTGGTGGACCCGTCCGCGTACGACTTCTACCTGTGCAGCCACACTGGGATTCTGGGG
ACGAGCAGGCCGACGCACTACTACACCCTGGTGGACGAGCACGGCTTCGGCTCCGACGAC
CTGCAGAAGCTGATCTACAACCTGTGCTTCGTGTTCGCGCGGTGCACCAAGCCGGTGTCG
CTGGCGACGCCCGTCTACTATGCCGACCTCGCGGCCTACCGTGGCAGGCTCTACTACGAG
GCTGCCATGATGGCGTCCCAGGCCCAGCGAGGGGGGTCCTTTGACGTCACTAACTTCCCG
AGGCTGCACAAGGATGTGGAGGACAACATGTTCTTCATCTGA
>Zea-mays_GRMZM2G039455_T01 cds:_protein_coding
ATGCTAGTGCCCATCATGGTGAGGAAGAAGAGAACTGGCCCTGGCGGCTCTGGAGAAACT
TCTGGAGAGTCTTCAGGAGCTTCTGGACAAGGTTCCTCACAGCGGCCTGAACGGACTCAA
CAACCTGGGGCAGGACGTGGCTGGGTGCCTCAGCAGGGTGGCCGTGGTGGCGGGCAACAC
CAGGGTCGTGGTGGACATTATCAAGGCCGTGGAGGGCCAGGTCCACATCACCCTGGTGGA
CTGCCTGAGTATCACCAGCGTGAATACCAGGGACGAGGTGGTGAGTACCAGGGACAGTAC
CAGGGGCGTGGTGGTGCCCGCTCCAGAGGTGGAATTTCACAGCCATACTATGGTGGGCAT
AGGGGAGGTAGTGTTGGACGAAATGTTCCTCCAGGTCCATCCAGAACAGTTCCCGAGCTG
CACCAAGCCCCATACGTCCAGTATCAAGCCCCGGTGATTTCACCATCCCCATCGGGACCT
GGCTCATCCTCACAGCCTATGGCAGAGGTGAGCTCTGGACAAGTCCAGCAACAGTTTGAG
CAACTTGCCATTCATGGTCAGAGTTCCATGAGTCAAGAAGTTCAAGTGGCACCAGCATCA
AGCAAATCGGTTCGATTCCCATTACGCCCCGGCAAGGGCACTTATGGGGACAGGTGCATT
GTGAAGGCGAATCATTTTTTTGCTGAGCTTCCTGACAAAGACCTTCACCAATATGATGTA
ACTATAACACCTGAAGTTACTTCACGTGGCGTTAATCGTGCTGTCATGGGAGAGCTTGTA
ACACTATATAGACAATCCCATTTGGGCGGGCGTCTACCTGCGTACGATGGAAGAAAGAGC
CTTTATACCGCTGGACCATTGCCTTTTACTTCTATGACATTTGAAATTACCTTGCAAGAT
GAGGAAGATAGTGTTGGCGGTGGCCAGGGCGGACAAAGGCGCGAGAGAGTATTTAGGGTG
GTGATCAAATTTGCGGCCCGTGCTGATCTCCATCATCTGGCTATGTTTCTAGCTGGAAGG
CAAGCAGACGCTCCTCAAGAAGCTCTTCAAGTGCTTGACATTGTACTACGTGAATTGCCT
ACTGCGAGGTATTCTCCTGTTGGTAGGTCATTTTATTCTCCCAACTTAGGGAGACGTCAG
CAACTTGGTGAGGGTTTGGAAAGTTGGCGCGGTTTTTACCAAAGCATAAGGCCGACACAG
ATGGGCCTTTCACTGAATATTGATATGTCCTCTACTGCATTTATCGAGCCTCTCCCTGTG

ATTGATTTTGTTGCTCAGCTTCTTAATAGAGATATTTCAGTTAGGCCATTGTCTGATTCT

GATCGCGTGAAGATCAAAAAAGCCTTAAGAGGTGTGAAGGTTGAGGTCACTCACAGGGGA

AACATGCGCAGAAAGTATCGCATTTCTGGCCTCACCTCACAAGCAACAAGAGAGCTATCA

TTCCCTGTTGATGATCGTGGTACTGTGAAGACTGTGGTCCAATACTTCATGGAGACTTAT

GGTTTTAGCATCCAGCACACCACTTTACCGTGCTTGCAAGTGGGCAATCAACAAAGACCA

AATTATCTGCCTATGGAGGTTTGCAAGATAGTTGAAGGACAGCGTTACTCAAAGCGACTC

AATGAGAAACAAATCACTGCTTTACTGAAAGTGACCTGCCAGCGCCCTCAAGAGCGTGAG

CTGGACATTTTACAGACTGTGCATCACAATGCGTACTATGAAGACCCGTATGCACAGGAA

TTTGGTATAAGAATTGATGAACGCCTTGCTGCAGTTGAAGCTCGTGTTCTGCCACCACCA

AGGCTTAAATACCATGATAGTGGCCGAGAGAAGGATGTTTTGCCCAGAGTTGGCCAATGG

AACATGATGAATAAGAAAATGGTAAATGGTGGCAGAGTCAGCAACTGGGCATGTATTAAC

TTCTCTCGGAATGTGCAAGATAGTGCCGCTAGGGGTTTCTGTCATGAACTGGCAATCATG

TGCCAAATATCAGGAATGGATTTTTCCCTTGAGCCTGTGCTGCCTCCAGTGACTGCAAGG

CCAGAACATGTTGAAAGAGCGTTGAAGGCACGTTATCAAGATGCAATGAACATACTGAGG

CCACAGGGGAGGGAACTTGATCTGCTGATTGTAATACTGCCTGACATTAATGGTTCCTTA

TATGGGGATCTCAAAAGGATCTGTGAGACTGATCTCGGATTGGTCTCCCAGTGTTGTCTG

ACTAAACATGTTTTTAAGATGAGCAAGCAGTATCTTGCAAATGTTGCACTCAAAATAAAT

GTTAAGGTTGGTGGAAGGAATACTGTACTTGTAGATGCTTTGACAAGGAGAATCCCCCTT

GTCAGTGACAGACCGACCATAATATTTGGTGCTGATGTTACCCATCCACATCCTGGAGAA

GATTCCAGTCCTTCCATTGCAGCTGTGGTTGCTTCGCAAGACTGGCCTGAGGTCACCAAA

TATGCTGGACTAGTGAGTGCCCAAGCCCATCGCCAGGAGCTGATACAGGATCTTTTCAAA

GTATGGCAAGATCCACAGAGAAGGACAGTAACTGGTGGCATGATAAAGGAACTTCTCATT

TCTTTCAAGAGAGCAACTGGACAGAAGCCCCAGAGGATCATATTCTACAGGGATGGTGTC

AGTGAGGGACAGTTCTATCAAGTATTGTTGTATGAACTTGATGCCATCAGAAAGGCATGT

GCATCCTTGGAGCCCAACTACCAGCCTCCAGTTACTTTTGTCGTGGTGCAGAAACGACAT

CACACTAGGCTGTTTGCTAATAACCACAACGATCAGCGTACAGTTGATAGAAGCGGAAAC

ATACTGCCTGGCACCGTGGTTGATTCGAAGATTTGCCATCCTACTGAATTTGATTTCTAC

CTGTGTAGCCATGCTGGCATTCAGGGAACAAGCCGCCCTGCTCATTACCATGTCCTGTGG

GACGAGAACAAGTTCACAGCTGATGAGCTGCAGACTCTGACAAACAACCTATGCTACACG

TACGCTAGGTGCACCCGCTCCGTGTCAATTGTGCCCCCGGCATACTATGCTCATCTGGCA

GCCTTCCGAGCTCGCTTCTACATGGAGCCAGATACCTCTGACAGTGGCTCAATGGCCAGT

GGTGCCCGTGGCCCTCCACCAGGTGCGGCACGCAGCATGAGAGGAGCGGGGAGTGTTGCG

GTCAGGCCCCTACCTGCTCTCAAGGAAAACGTGAAGCGTGTCATGTTTTACTGCTGA

>Zea-mays_GRMZM2G059033_T01 cds:PUTATIVE_protein_coding

ATGGCCTCTCGTGGGCGCGGTGGTGGCGGAGGGAAAGGCCCTAGCGGTGGGCGGGGCGGT

GAAGGCAGGGGGCGGGGGATCCGTGGCGGGGGAGGTTACCATCACGGCGACGATGGAGGC

GGCGAACGCGGAGGCAGAGGCGACCCGGGCCGAGGGCGCGGGGTCGGCGGTCGTGGCCGC

GGTCCCGGAGGCCGAGGTGTCGACGACCACCAGCAGCAGCCGCCGCCGCTCGGGAACACA

GAGGGCGGAGGCCAGGGCCGCGGCGGCATGGCGGTAGCGGTACCGCCAGCCCGCCCCGCC

GCGCCCTGTCCTCTAGCGCCGGACGCCGCTGCCCCGGCATTTCCCGCAGCAGCGGCCTCG

TCTTCTGCGCAAGCGCCTCGAGCCCCCACCGAGGCGGCTGGAGCTGCCTTGGCGGATGGC

ATGGGGAAGTTGGCCGTGGCGGACGACCGGCCCGCTGCTCCTCCCGTGCCCGCAGGTAAA

CCCGTCGCGCAAGGTCCCGCGCACCAGCCCCAGGAGGCTCCGCCACTCTCGAGGAAGAGG

ATCGTGCCGCCGCCCCGCCCCGGTTTAGGCACGTCTGGGAGAAAGCTGGCGGTTCGCGCG

AACCACTTCTTTGTCGAGGTGTCCGTGAACGACATCTTCCATTACGACGTTTTAATCAAT

CCTGAACCAAAAGCAAGAAAAACCAACAGGATGCTGCTCTCAGAGCTTGTCAAGATACAT

GGCGCGACATCTCTCGCCCACAAGACACCTGCATATGATGGAAGCAAGAGCCTGTACACT

GCAGGCGAGCTGCCGTTCAAATCAATGGAGTTTGTTGTCAAGTTGGGGAAGGCGGGCCGA

GAAGTTGACTACAAGGTGACAATCCGATACGCGGCACGGCCTAACGTGTACCAACTTAAG

CAATTGATCAATTCTCAGCTAAGGAACACGCCATTTGACGCAATCCAAGCACTGGATGTT

GTCTTGAGAGAGTCGCCTTCTCTCAACTATGTAACTCTTTCTCGATCCTTCTTCTCCAAG

AAGTTCGGTGATGACGACATTGGCGGTGGGCTAGAGTGCTGGAGAGGATATTACCAGAGC

TTGCGTCCAACTCAAATGGGCCTCTCATTGAACATTGATACATGCTCGACTTCATTTTAC

CAACCTATCGATGTGGTAAAATTTGTTGCCGATTGTCTCCAGCTGACAAACCCTGGCCAA

CCTTTTTTGGACAGGGATCGTTTAAAGCTTAAGAGAGCCCTGCGCGGAGTTCTTGTTGAG

ACTGAACACCAGCAGGGAAAGAGAAGCATCTACAGGATAACTGGGATTACTTCTGTTCCA

TTGGCTCAACTGAGCTTTTCTTGTAACGAAGGCCCTCAGCTGACTGTTGTTGAGTACTTT

GCACAACGGTACAATGTCCAGCTGCGCTACACTGCTTGGCCCTGCCTGCAGTCGGGCAAT

GATTCTAAGCCGATATATTTACCAATGGAGGTGTGCAAAATCATTGAAGGGCAGAAGTAC

CCTAGGAAGCTCAGCGACACACAGGTGGCCAACATACTGAAGGCAACCTGTAAACGTCCT

CAGGATAGGGAGGAGAACATTATTAAGATGGTTCGCCACAACAACTATTCTGCTGATAAG

ATGGCACAGGTGTTTGGGATCACTGTGGCCAACCAGATGGCTAATGTGCAAGCCCGTGTT

CTGCCTCCACCTATGCTGAAATACCACGAATCTGGAAAGGAGAAAACCGTTGCACCAAGC

TTGGGGCAATGGAATATGATTAACAAGAAAATGGTCAATGGTGGAACCATTCACAGCTGG

ACTTGTCTGAGCTTTTCGCGGATCCCGCTTCGTTTGGTAGACGAAATATGCCATGAATTG

GTTCAAAAGTGCAATTCCATTGGCATGAGTTTCAATCCAAGGCCGGTGACAGAAGTTCAG

AAAGACTCACACAACAACATAGAAGCTGCTTTAAGGGATGTTCACAGGAGGGCTCCGAAT

CTGCAGCTGCTTATTGTGATTCTTCCAGATGTTACTGGTTATTATGGGGAAATTAAGAGG

ATGTGCGAGACTGACCTTGGTATAGTATCTCAGTGCATCAATCCGAAGAAGAATAGAAAC

AAGCAGTATTTTGAAAATCTTGCCCTTAAAATCAATGTGAAGGCTGGAGGGCGCAATACA

GTGCTTGAGAGAGCCTCTGTGCCTAATGGGATACCTTTTGTCTCAGATGTGCCAACAATC

ATTTTTGGTGCTGATGTTACCCATCCTACAGCAGGAGAAGAATCCTCGGCTTCTGTTGGA

GCTGTGGTTGCATCCATGGACTGGCCACAGGTCACAACATATAAAGCTCTGGTCTCGGCA

CAAGCACACAGGGAAGAGATTATACAAAATCTCGGCGGAATGATAAGGGAGTTGCTGATT

TCGTTCTATAAGAGGACTGGCAAAAAGCCCAAAAGGATTATATTTTACAGGGATGGAATA

AGTGAAGGACAATTCAACCATGTTTTGCTCCTTGAAATGGACGCGATAAGGAAGGCTTGT

GCCTCTCTGGAAGATGGGTATCTACCCCCAGTGACATTTGTCGTAATACAGAAAAGGCAC

CACACAAGGCTCTTCCCTGGAGTTCATGGAAGGAGAGATGTTACTGACAGAAGTGGGAAC

ATTCTTCCTGGAACTGTGGTTGATACCGAGATTTGTCATCCGCGGGAGTTTGATTTCTAC

CTTTGTAGCCATGCTGGAATTCAGGGAACCAGCAGGCCAATACACTATCATGTCCTCTAC

GATGAAAACCGTTTCTCGGCTGATGGGCTGCAGATACTCACAAACAGCCTGTGCTACACA

TACGCACGATGCACGCGCGCTGTCTCAGTTGTTCCACCAGCCTACTACGCTCACCTGGCA

GCATTCCGCGGGAGGTACTACGACGAACAAGGTAGCAGCCCCGCCCCCGATGGAACCTCA

GTTGTCGGTGGCGATGCCGCTGCAGCCGGTGATGATCCACCTGCGTGCCGCAGGCTCCCC

CAGATCAAGGAGAATGTGAAGGAAGTGATGTTCTTCTGCTAG

>Zea-mays_GRMZM2G077801_T01 cds:_protein_coding

ATGGCTGCCAAAATGGCTGGAGCTGTCCAAGTGCTCAAGGATGACACTGTTAAACGCACA

CCCATGGCACGACCTAGTAACGGCCGTGAAGGAAAGCCCATTAGGTTGCTGTCGAACCAC

TTCTCAGTGAAGCTTAGAGGAGTTGATGCTGTTTTCTACCAATACAGTGTCTGCATCAAA

TCTGAGGATGATAAGGTGGTTGATAGCAAGGGTATTGGCCGAAAGGTCATAGATAAACTA

TTGCAAACATACTGTTCTGAGCTTGATGGGAAGGATTTTGCATATGATGGAGAGAAATGT

CTATTTACTGTGGGACCTCTTCCACAGAATAACTTTGAGTTCACTGTTATCTTGGAGGAA

ACATCTTCAAGGGCTGCTGGTGGAAGTCCAGTGCATGAAAGCCCTACTCAAGCTAACAAA

AAGCGAGTCAAGCGATCACATCTGGCAAAAAAGTTCAGTGTAGACATAAGTTATGCCGCA

AAGATTCCTCTTCAGTCGGTTGCTTTGGCTCTTCGAGGAAGCGAGTCAGAACATGGTCAA

GATGTTCTGAGAGTCCTTGACGTTGTTTTAAGGCAACAGCAGGCTAAGAGAGGTTGTCTA

CTTGTTAGACAGTCATTTTTCAGTGATGATAGTCGAAACCTTGTTGATTTAACTGGTGGA

GTTAGTGGTTGTCGTGGACTCCACTCTAGTTTCCGTACTACAATTGGTGGTCTTTCACTA

AATATGGATGTTTCAACCACTATGGTTGTAACTCCTGGACCAGTTATTGATTTTCTCGTC

ACAAATCAAAATGTAAGAGACATCAGAGATATTGACTGGCCCAGGGCCAAGAAAATGCTT

AAAAATCTCAGAGTTAAAGCTAAGCACAACAACATGGAGTTCAAGATTATTGGCCTTAGT

GATCAACCATGCTCTAGACAGATGTTCCCAATGAAAGTTCGAAATGGAAACATCGAAATT

AAATCTGTTGATATCACTGTTCAGGATTATTTTAAATCCAAGCAAGTTGAGCTAACAATG

CCTTATCTGCCATGTCTTGATGTGGGAAAACCAAAACGCCCTAATTATCTCCCAATTGAG

TTATGCCACATGGTATCACTTCAACGTTATACAAAGGCACTGTCTTCTCAACAAAGGGCA

ATGTTGGTTGAAAAGTCACGACAGAAACCTCAAGAAAGAATGCGAGTTGTTACAGATGCT

GTAAAAAGTAATATGTATGATGATGATCCAATCTTATCTTCATGTGGTATTGAAATTGAG

AAACAACTTACTCGTGTTGACGCTCGTGTTCTCTCTGCACCAGCGCTAGTTGTGGGCAAC

AGTGAAGATTGCATCCCAAACAGGGGTAGGTGGAACTACAATAATAAGAGGCTATTCGAT

CCAGTCAAGATTGAGCGTTGGGCCATTGTTAATTTCTCTGCTCGTTGTGACATGAGCCGA

ATCTCAAGAGAACTGATAAACTGTGGACGCAGCAAAGGCATTTTCATTGAATGTCCTCAC

AGTTTGGTGGATGAGGACAGCCAGTCTAGGAGATGTTCACCTGTGGAAAGGGTTGAAAAG

ATGTTTGAAAAAGTCAAAGCAAGCCTTCCTGGTCCTCCAGAGTTTCTCCTTTGTCTTTTA

CCAGAGAGGAAGAATTGTGATATTTACGGGCCATGGAAGAAGAAAAATCTTCATGAAATG

GGTATTGTCACTCAATGCATTGCTCCAAGTAATAAGATGAATGATCAATATTTCACCAAT

GTTCTTCTAAAAATTAATGCTAAGCTTGGTGGAATGAACTCCAAACTGGCACTGGAACAT

CGTCAAATGATACCAGTTGTGACTCAAATACCAACATTAATTCTTGGCATGGATGTTTCA

CATGGTTCTCCAGGTCGAGCAGATATACCATCAATTGCTGCGGTTGCCCACAACAACTCG

CGTAACAGCAGCCTGCTACGCAAGCTGCGCCTCCCTCTTAGGCATATGCCCCCTACTGGC

GGCAACAACAAAGGGAAGGGCAAAGGAAGATGCGCCAACGACCACTCCTCCAGCGACAAC

CATGACCTTGGCACCGAGAGCATCAACACCCCATGGCTATCGTTCGACAAGCCGTGCACG

GGCATGATCACCATGTGGCACAGACCACGTCCCCCTCTACAACAACATGTGCACCCTCAT

CAACATGCCCTGATGGCTGTCTCGCCCCACCTGGCGCCTAAATGGGAGGGGTTTAGTTTA

TTAGCGTGTTGTGGCGGTTGA

>Zea-mays_GRMZM2G079080_T02 cds:_protein_coding

ATGCTCGAGGTCGTCCTGGACATGACGCCGCCGCCGCCGCCGCCGCAGGCGCGGCTCCAC

CAGGGGTCATCAGCCAAGGGCGGGCACGCGGAGCGGAGGAAGCAACCGCTGCAGAGCAGC

GTGACACAGCCCAAGGCAGAGCCCGCGGCGGCGGCGGCCGTGCTGCCGGTGCCGGAGGGA

GGCAAGAGGTGCAGAGGGGGCGGGAGGCGCCGCGGCAGGGCCAAGGCGCCCGGTGAGCCT

CGCGCCGCGCTACTAGCGCCAGCGCAGGCGCAGACGCAGGCGCCTCCGCCGCGCACGGTC

ATTGGGCCGCCCGTGCCGAGCAAGGGGCTGTCATTCTGCCGCCGGCCAGGGTTCGGGACG

GTGGGCGCGCGCTGCGTCGTCAAGGCCAACCACTTCCTCGCCGAGCTCCCGGACAAGGAC

CTCACCCAGTACGATGTGAAGATCACGCCGGAGGTGAGCTCGCGGACCGTGAACCGGGCC

ATAATGGCGGAGCTGGTCCGCCTCTACCGCGCGTCCGACCTGGGGATGCGACTCCCGGCC

TACGACGGCCGCAAGAACCTCTACACCGCCGGGACACTACCGTTCGACGCTCGCGAGTTC

GTCGTGCGCCTCGCCGATGAGGACGACGGCTCCGGCGTCCCGCCTCGGGAGAGGGAGTAC

AGGGTCGCCATCAAGTTCGCCGCGCGCGCCGACCTCCACCACCTCAGGCAGTTCATCGCC

GGGCGGCAGGCGGACGCGCCGCAGGAGGCCCTACAGGTACTCGACATCGTGCTCCGCGAG

CTCGCCAACCAGAGGTACGTGTCCATAGGGCGGTCCTTCTACTCGCCGGACATCAGGAAG

CCGCAGCGGCTCGGCGACGGCCTGCAGTCGTGGCGTGGGTTCTACCAGAGCATCCGGCCG

ACCCAGATGGGATTGTCGCTTAACATCGACATGTCGTCCACGGCATTTATTGAACCCCTG

CCGGTGATCGAGTTCGTGGCCCAGATATTAGGAAAGGATGTCATATCAAGGCCATTGTCC

GATGCTAACCGAATCAAGATCAAGAAGGCATTGCGGGGTGTAAAAGTTGAGGTCACTCAC

CGGGGGAATGTACGGCGCAAGTATCGCATTTCAGGCCTCACAACACAGCCAACTCATGAA

TTGATTTTCCCGATTGATGAACAAATGAATATGAAATCTGTCGTGGAATACTTCAAGGAA

ATGTATGGTTTCACCATTCAGCATCCTCATCTTCCCTGCCTTCAGGTTGGAAACCAAAAG

AAGGCAAACTATCTACCCATGGAGGCCTGCAAGATCATTGAAGGCCAGAGATACACAAAG

AGGCTGAATGAAAAACAGATCACATCGCTGCTAAAGGTTACATGCCAAAGGCCTAGAGAG

CAAGAGATGGATATTCTACAGACAGTTCATCAAAATGATTATGAGCAAGATCCATATGCG

AAGGAATTTGGGATCAACATTAGTGAGAAGCTAACCTCTGTTGAAGCCCGAGTCCTTCCT

GCACCTTGGTTGAAGTATCATGACACTGGAAAAGAGAAAGAGTGCTTACCACAGGTTGGT

CAGTGGAACATGGTAAACAAGAAAGTGATAAATGGATGCAAGGTGAGCCATTGGGCATGT

ATAAACTTCTCAAGGAGTGTTCCAGAAACCACAGCTCGGGGATTTTGCCAGGAATTGGCA

CAAATGTGTCAAATTTCGGGCATGGAATTTAACAGTGAGCCTGTGATGCCAATATATTCA

GCTAGACCAGATCAAGTTGTGAAGGCACTTAAAAACGTGTATAATATTGCATTGAACAAA

CTTAAGGGTAAAGATCTTGAACTTCTTTTGGCTATCCTCCCTGACAACAATGGGCAGTTA
TATGGTGACATCAAACGTATTTGTGAAACTGATTTGGGGTTGATATCACAATGTTGCTTA
ACCAAGCATGTTTTTAAGATCAGCAAGCAGTACTTGGCAAATGTCTCACTGAAAATTAAT
GTTAAGATGGGAGGAAGAAACACTGTGCTCCTGGACGCAATAAGTTGGAGGATTCCGTTG
GTCAGTGACATCCCAACTATTATATTTGGTGCAGATGTAACACATCCTGAAACCGGGGAG
GACTCAAGTCCATCGATTGCTGCCGTTGTTGCTTCTCAAGATTGGCCAGAAGTTACAAAG
TATGCTGGATTGGTTTGTGCTCAGGCACACCGGCAAGAGCTCATTCAAGACCTTTACAAA
ACATGGCACGATCCTCAGAGAGGCACTGTAACAGGCGGCATGATCAGGGAGCTCTTAATA
TCCTTCAGGAAGGCCACTGGACAGAAGCCATTGAGAATAATATTCTACAGGGACGGTGTT
AGTGAAGGTCAGTTCTATCAAGTTCTCCTTTACGAGTTAGATGCCATCCGGAAGGCATGT
GCATCCCTAGAACCAAATTACCAGCCTCCTGTAACATTTGTGGTGGTTCAAAAACGTCAT
CATACAAGACTATTTGCAAATAATCACAAAGACAGAAGTAGCATGGACAAGAGTGGAAAT
ATTTTGCCAGGAACCGTTGTTGATTCTAAGATATGCCACCCAACGGAGTTTGATTTCTAC
CTCTGTAGTCATGCTGGAATCCAGGGAACGAGTAGGCCTGCTCACTACCATGTCCTCTGG
GATGAGAACAATTTCACAGCAGACGAAATGCAGACATTGACAAACAACCTTTGCTACACT
TATGCCCGGTGCACACGCTCGGTTTCTGTTGTCCCTCCTGCATACTACGCACACTTGGCA
GCATTCCGGGCTCGGTTCTACATGGAACCAGAGATGTTGGACAACCAGACGTCCAAGACC
TCCAATGGCACGAGCGGAGTCTCGGTGAAGCCCCTGCCTGCTGTGAAGGAGAAGGTGAAA
AGGATGATGTTCTACTGCTGA

>Zea-mays_GRMZM2G105250_T01 cds:_protein_coding

ATGGCGAGCCATCAGCGCGGAGGAGGTCGGGTTGGCGGCGGCGGCTGGGGTCAGGCGAAC
CCCAACGTTATTCAGGGCCAGGCGGGGCGGGGCTACGGCGGCGGTCGAGGTGGTCAGTAC
TACGGCGACGACAGCCGCGGTCGAGGTGGTCAGTACTACGGCGACGACAGCGGCGGACGT
GGCGGCGGCAGGGGCGGTGGTCGTGGCTTCGACGGCCGCGACGGCGGCGGATACCAGGAA
GGTCGTGGCGGGGGCCGCGGCGGCGGCGGATACCAGGAAGGGCGTGGCGGGGGCCGCGGC
GGCGGCAGATACCAGGAAGGACGTGGTGGTGGCCGGGGTGGCGGCGGCTACTACTATGAA
GGACGTGGCGGGGACCGTGGTGGCGGCGGCTACTATGGAGGACATGGTGGGGGCTACCAG
GAAGGCCGTGGCGGGGGCCGAGGTGGTCGCGGCTACCAGGGACAGGGAGGCAGCGACTAC
GGCCGCGAGCGCGGGCATGGCGGCCTACAAACCCCACGTCCTGCTCTGCGCCAAGCCGGT
CCGCCCCTCGCGGACCGCTACGCGGCCGACGCAGCCGCGTTGAGGGACAAGTTCAAGACG
ATGGACATTCACCGCGACGAGCCCATGTTTCCAGCGCGCCCGGGCTTCGGCGCCGTGGGG

ACGCCGTGCGTCGTCAAGGCCAACCACTTCTTTGTCGGCCTCGTCGAAAAGGGCCTGCAC

CACTACGACGTGGCCATCTCACCAGAGACGACGCTAAGGGGCATATACAGGCAAGTCATG

TCGAAGCTGGTTTCCGAGAACCGGCAGACCGAGCTTGGCGGCCGCCTGCCTGCATACGAC

GGGCAGAAGTCACTGTTCACCGCCGGAGAGCTGCCCTTCAAGACCAAGGAATTCGTTGTC

ACCTTGTCTGGCAGGATGGAAAGGAGGTACAAGGTGGTGATCAAGCATGCCACGGCGGTC

AGCCTCGACCAGCTGTTGATGCTCATGGCAGGGTACCCAACGGACATCCCGGCGCAGGCG

CTGCAGGTGCTTGACATTGTGCTGCGTGACATTGTGCTCAACGAACGCAACACCATGGAG

TACGTTGCAGTTGGCCGGTCCTTCTTCTCACCACTCATAGACTCAATGGGACCCAAGAAC

CTTGGCTTGGGTGTGGAGGGATGGAAGGGTTTCTATCAGACCATCAGGCCGACACAAAAA

GGCTTATCTGTGATCATAGACATTTCTTCATCAGCTTTCATTCGACCCCTGCCACTGATT

GACTTTGTTATGGAGATTCTGAACAAAGATAACAGGACCTTTAGAAGTATTACTTCCATG

GATCTTGTCAAGCTCAAGAAAGCCCTCAAGGGTATGAGGATTGAAGTCACACACCGAGGA

GACATACGCCGGAAGTACCGAATTGCCAGCTTGACAAACAGTCCTCCATCTTCACAGTTC

TTTGAATCATCTGCTGGAGTTCAGAAGTCCGTAGCAGATTACTTCAGAGAGGCATACCAT

CTGGAAATGCACTATGATTTTCTCCCATGCCTCCAAGTTGGCAGTGATCAGAGGCCGAAT

TACCTCCCTATGGAGGTTTGCAAGATAGTAGCTGGACAGCAATACCGGAAGAAGTTGGAA

GGCCAACAGGTCTCTAAACTAATGGACTCCACCTGCCAGCGCCCATCTCTCCGCGAGGAC

AACATTTGTCAGATTGTTGAGCAAAATGACTACAATAAAACTGAGCGTGCAAGTGAATTT

GGCATGGAGGTTGATTATCGTCCTACTTCAGTGCAGGCTAGAGTTCTGCCAGCTCCCACT

CTGAAGTACCGTGGCACTGGATCTGACAGTTTGTGTTGTCCAAAGGATGGTCAGTGGAAC

ATGATAAAAAAGCAAGTAGTAGATGGTGCAAGGGTGGGCAACTGGGCCTGCGTTAACTTT

TGTCAAGAGTTGCGTGCAGATGGTGTTGGTAAATTTTGCTCTGATCTGGTCAAGTGGTCT

CGCACTACTGGAGTGGACATGGATAACTTGAGACTTCCAATATATACCGCTCGTCCTGAA

CAAGCTGAAACTGATCTTCGTAGGCTCTATCAGGATGCTCGGAACAAGCTAAGAGGGCAA

AAGTTTGATCTTTTGCTTGCTATACTGCCAGAGAAAAATGGCAGCTTATATGGTAATTTC

AAAAGGATCTGCGAGACAGAGATTGGTATCATGTCGCAGTGTTGTTTGGATAAAAATGTT

AGAAGTGCAGGCCCTGCATATTTTGCTAATGTTGCTATTAAGATCAATGCCAAGTTTGGA

GGAAGAAACTTAGAATTTGCTAATCCCAAAGAAAGCTTACCGGGTGTTACAATTGAACCA

ACAATTATATTTGGTGCTGATGTCACTCACCCTGCCGCTCTAGATGATACCGCCCCTTCC

ATTGCTTCTGTTGTTGCCTCCCAAGACTGGCCCAAGGTGGCTAACTATAATGGGATTGTC

CGTGCACAAGGTCATCGTAAAGAGCTCATCAATGGCCTGGAAGACATTGTCAAGGAACTC

CTACTTGCATTCGAGGAGAGGTCTAAGCGGAGACCCAAGCAGCTGATCTTCTACAGGGAT
GGCGTAAGTGAGGGCCAATTCAAACAAGTGCTGGAACAAGAAATCCCTGAGATAGAGAAG
GCATGGAAAGCTCTTTACAATGAGAAGCCAAAGATCACCTTCCTAGTGGTGCAGAAGAGG
CACCACACAAGGCTCTTCCCAAACAACCCAAATGATCGCCAATGGGCGGACAAGAGTGGA
AATATTCTACCTGGCACTGTTGTTGACAAGGATATCTGCCACCCAACAGAATTTGATTTC
TTCCTGTGCAGCCATGCTGGGATCAAGGGAACTAGCCGTCCTGCGCATTACCATGTCCTG
AGAGACGACAACAACTTCACTGCAGACGCGCTGCAGTCTCTCACATATAACCTATGCTTC
TTGTATTCAAGCTGCACTCGCTCTGTGTCAATCGCTCCCCCAGCATACTACGCCCACAAG
TTAGCGTTCCGCGCCCGCTTCTACGTCAACCAAGACTCTGATGCGGCGACAAGTGTCGGC
TCTTATGGTTCATCAGCTCCCTCTGCTGCTGCTGCTGCTGCTGGTCCGAAGCCGCTTCCG
GAGATCAAGGGTGAACTGAGAAGGCTCATGTTCTACTGCTAG
>Zea-mays_GRMZM2G108281_T03 cds:PUTATIVE_protein_coding
ATGCTCGAGGTCCTGGACATGGCGCCGCCGCCACCACCGCCGCCGCCGCCGCCGCATGCG
CGGCACCATCAGGGGGCAGCCAAGGGCGGCCACGCGGAGCGGAGGAAGCAACCGCTGCAG
AGCAGCGTGACGCAGCCCAAGGCGGAACCGGCGGCGGCGGCGGCGGCGGCCGTGCTGCCG
GAGGGAGGCAAGAGGTGCGGAGGAGGCGGCGGGAGGCGCCGCGGCGGGCGCGGCCGGGCC
AAGGCACCACCTGGTGAGCCTCGCGTCGCGCTAGCGCTGGCGCCTCCGCCGCGCACGGTC
ATTGGCCCGCCCGTGCCGAGCAAGGGGCTGTCGTTCTGCCGCCGCCCCGGATTCGGGACC
GTGGGCGCGCGCTGCGTCGTCAAGGCCAACCACTTCCTCGCCGAGCTCCCGGACAAGGAC
CTCATCCAGTATGATGTGAAGATCACGCCGGAGGTGAGCTCGCGGACCGTGAACCGGGCC
ATAATGGCTGAGCTGGTCCGCCTCTACCGCTCGTCCGACCTGGGGATGCGGCTCCCGGCC
TACGACGGCCGCAAGAATCTCTACACCGCCGGGACCCTCCCGTTCGACGCTCGCGAGTTC
GTCGTGCGGCTCACCGATGAGGACGACGGCACCGGCGTCCCGCCTCGGGAGAGGGAGTAC
AGGGTCGCCATCAAGTTCGCCGCGCGCGCCGACCTCCACCACCTCAGGCAGTTCATCGCC
GGACGGCAGGCGGACGCGCCGCAGGAAGCCCTACAGGTACTCGACATCGTGCTCCGCGAG
CTCGCCAACCAGAGGTACGTGTCCATAGGGCGGTCCTTCTACTCGCCGGACATCAGGAAG
CCGCAGCGGCTCGGCGACGGCCTGCAGTCATGGTGTGGGTTCTACCAGAGCATCCGGCCG
ACCCAGATGGGATTGTCACTCAACATCGACATGTCGTCCACGGCGTTTATTGAACCCCTG
CCGGTGATCGAGTTCGTGGCCCAGATATTAGGAAAGGATGTCATATCAAGGCCATTGGCC
GATGCTAACCGAATCAAGATCAAGAAGGCATTACGGGGCGTAAAAGTTGAGGTCACGCAC
CGGGGGAATGTAAGGCGCAAGTATCGCATATCTGGGCTCACAACACAGCCAACTCATGAA

CTGATTTTCCCAATTGATGAACAAATGAATATGAAATCTGTCGTGGAGTACTTCAAGGAA
ATGTATGGTTTCACCATTCAGCATCCTCATCTTCCTTGCCTTCAGGTTGGAAACCAAAAG
AAGGCGAACTATTTACCAATGGAGGCCTGCAAGATCGTTGAAGGCCAGAGATACACGAAG
AGGTTGAATGAAAAACAGATCACATCGTTGCTAAAGGTTACATGCCAAAGGCCTCGAGAA
CAAGAGATGGATATTTTACAGACAGTTCATCAAAATGGATATGAGCAAGATCCATATGCG
AAGGAATTTGGGATCAACATTAGTGAGAAGCTAACCTATGTTGAAGCCCGAGTCCTTCCT
GCACCTTGGCTGAAGTATCATGACACTGGAAAAGAGAAAGAGTGCTTACCACAGGTTGGT
CAGTGGAACATGGTAAACAAGAAAGTGATAAACGGATGCAAGGTGAGCCACTGGGCATGT
ATAAACTTCTCAAGGAGTGTTCCAGAAGCCACAGCTCGGGGATTTTGCCAGGAATTGGCA
CAAATGTGTCAAATTTCGGGCATGGAATTTAACAGTGAGCCCGTGATGCCAATATATTCA
GCTAGACCAGATCAAGTAGTGAAGGCACTTAAAAGTGTGTATAATATTGCACTGAACAAA
CTCAAGGGTAAAGAACTTGAACTTCTTCTGGCTATACTCCCCGACAACAATGGTCCGTTA
TATGGTGACATCAAACGTATTTGTGAAACTGATTTGGGATTGATATCACAATGTTGCTTA
ACCAAGCATGTTTTTAAGATCAGCAAACAGTACTTGGCAAATGTCTCACTGAAAATTAAT
GTTAAGATGGGAGGAAGAAACACTGTGCTCCTGGACGCAATAAGTTGGAGCATTCCTTTG
GTCAGTGACATCCCAACTATTATATTTGGTGCAGATGTAACACACCCTGAAACCGGGGAG
GACTCAAGTCCATCAATCGCTGCCGTTGTTGCTTCTCAAGATTGGCCAGAAGTTACAAAG
TATGCTGGATTGGTTTGTGCTCAGGCACACCGGCAAGAGCTCATTCAGGACCTTTACAAA
ACATGGCACGATCCTCAGAGAGGCACTGTAACAGGCGGCATGATCAGGGAGCTGTTAATA
TCCTTCAGGAAGGCCACTGGGCAGAAGCCATTGAGAATAATATTCTACAGGGACGGTGTT
AGTGAAGGCCAGTTCTATCAAGTTCTCCTTTACGAGTTAGATGCCATCCGTAAGGCATGC
GCATCCCTAGAACCAAATTACCAGCCTCCTGTAACATTTGTGGTGGTTCAAAAACGTCAT
CATACGAGACTATTTACAAACAATCACAAAGACAGAAGTAGCATGGACAAGAGTGGAAAT
ATTTTGCCAGGAACTGTTGTTGATTCTAAGATATGCCACCCAACAGAGTTTGATTTCTAC
CTCTGTAGTCATGCTGGAATCCAGGGAACAAGTAGGCCCGCTCACTACCATGTCCTCTGG
GATGAGAACAATTTCACAGCAGACGAAATGCAAACACTGACAAACAACCTTTGCTACACT
TATGCCCGGTGCACACGCTCGGTTTCTGTTGTCCCTCCTGCATACTACGCACACCTGGCA
GCATTCCGGGCGCGGTTCTACATGGAACCAGAGATGTCGGAGAACCAGACGTCGAAGAGC
TCCAATGGCACGAACGGAGGCTTGGTGAAGCCCCTGCCTGCTGTGAAGGAGAAGGTGAAA
AGGGTGATGTTCTACTGCTGA

>Zea-mays_GRMZM2G141818_T02 cds:_protein_coding

ATGGGCTCTCATGATGGCGAGGATGAAGAGTTGCCACCCCCCCCTCCGGTGCCACCAGAT

GTGATTCCCATTAAAGCTGAAGATGCTGTGGGTGAATCACCAGCAAACCATATATTAAAG

CCAAAGAGATTACTGATGGACAGGCCTGGTATAGGAAGAAAAGGGCAGCCGACCCAGCTC

TATTCAAATCACTTTAAAGTCGCTGTGAAGAGTACAGAAGACGTCTTCTTTCACTACTAT

GTAAACCTGAAGTATGAGGATGATCGACCCGTTGATGGTAAAGGGATCGGCAGAAAGGTG

ATTGATAAACTGCAGCAGACATATCGTGCAGAGCTTTCTAACAAGGACTTTGCATATGAT

GGAGAAAAGAGCCTGTTTACAGTTGGTGGTCTTCCACAAAAAAAGAATGAGTTCACCGTT

GTCTTGGAGGACGTATCTACTGGAAAGACTGCTGCCAATGGGAGCCCTGGAGGTAATGAC

AGTCCTGGAGGTGGTGATAGGAAGAGAGTGAGGAGGCCATACCAGACGAAAACTTTCAAA

GTGGAGATAAATTTTGCAGCAGAGGTTCCTATGAGTGCTATTGGTCAAGTCATTAGAGGC

GAAGAATCTGAGAACTCCCTGGAGGCGCTTCGTGTTCTTGATATCATACTGAGGCAGCAT

TCCGCAGAACAAGGCTGCCTTTTGGTTAAGCAATCATTTTTCTACAACAACCCTTCATGC

TTTGTTGACTTGGGTGGTGGTGTGATGGGTTGTCGTGGATTTCATTCAAGCTTCCGTGGC

ACACAGAGTGGACTTTCCCTCAATGTTGATGTCTCAACAACAATGATCGTGAAACCTGGC

CCTGTTATTGATTTTCTTCTTTCTAACCAGAATGTTAATGATCCTAGCAGAATTGATTGG

CAAAAGGCCAAGCGTGCTCTCAAGGGCTTGAGGATTAGAACCACTCCTGCAAATTCAGAA

TTCAAGATTTTTGGTCTCAGCGAGAGGATCTGCAAAGAACAAACGTTTCCGCTGAGGCAG

AGAAATGGTAGCAACGGAGATTGTGATACCATTGAAATAACTGTCTATGACTACTATGCA

AAGAAAGGAATCGATCTAAAGTATTCTGGTGATTTCCCCTGTATAAATACAGGGAAGGCA

AAGCGCCCAACATATTTTCCAATCGAGCTATGCTCGCTTGTTCCGCTTCAAAGATACACC

AAAGCTTTGTCTACGCTACAAAGGTCATCCCTTGTGGAGAAGTCTAGACAGAAGCCTGAA

GAAAGGATGACCGTTCTAAATGATGCACTGCAACGCAGTAACTACGATTCTGACCCCATG

TTGAGGGCATGTGGTGTTTCAGTTGCTCCAAAATTTACCCAAGTTGAAGGAAGGATCCTT

CAAGCCCCAAAGCTGAAAGCCGGCAATGGTGATGATATCTTTTCACGAAATGGACGGTGG

AATTTCACTAATAGGAAGTTTTATGAAACCTGCTCTGTGAATAAGTGGGCGGTCGTTAAT

TTCTCTGCACGTTGTGATGTTCGGAATCTTATCCGTGACCTGATGAGGAATGCATCTGCA

AAGGGAATTCAAATGGAGGAACCTTTTGATGTGTTTGAAGAGAGTCCCTCTATGAGGCGT

GCACCTGTGTCAAGAAGGGTGGATGATATGTTTGGGCAGATAAAATCAAAACTTCCTGGA

GCTCCTAGGTTCCTCTTGTGCCTTCTCCCTGAGAGGAAAAATTGTGAAATCTATGGTCCT

TGGAAGAGAAAGTGCCTGGCCGAGTTTGGTATTGTCACACAGTGTCTAGCTCCATTAAGA

GTCAATGATCCGTACCTGCTTAATTTGCTGATGAAGATCAATGCAAAGCTTGGTGGTCTG

AACTCGTTGCTGCAAGTTGAAGCATCTTCGTCAATACCACATGTGTCGCAAGTACCCACC

ATCATCTTAGGTATGGATGTTTCACATGGTCATCCAGGACAAGATAGACCTTCGGTTGCA

GCGGTGGTTAGTTCTCGTCAATGGCCTCTTATCTCTAGATATAGAGCATCAGTGCACACC

CAATCTGCCAGACTAGAAATGATGTCCTCGTTGTTTAAGCCGCGGGGTACTGATGATGAT

GGCCTCATCCGGGAATCACTGATCGACTTCTACACTAGCTCTGGAAAGCGAAAACCAGAA

CACATAATTATTTCAGGGATGGAGTCAGTGAAAGTCAGTTTACCCAGGTCATCAACATT

GAGCTGGATCAGATCATCGAGGCATGTAAGTTTCTGGATGAGAAGTGGTCACCCAAGTTC

ACTGTGATTGTTGCTCAAAAGAACCACCACACCAAGTTCTTTCAGACGGCATCACCAGAC

AATGTTCTTCCTGGAACTGTGGTGGATAGTAAAGTTTGCCATCCTAAGAACTTCGACTTC

TACATGTGTGCACATGCTGGGATGATTGGAACAACAAGGCCGACCCACTATCATGTTCTG

CACGACGAGATAGGTTTCAGTGCCGACGAGATGCAGGAGTTTGTTCATTCGCTCTCTTAC

GTGTACCAGAGGAGCACGACAGCCATCTCAGTGGTTGCTCCAGTGTGCTACGCCCACCTC

GCTGCAGCCCAGGTGAGCACGTTCCTGAGATTGGAGGAGATGTCAGACGCGTCCTCCAGC

CAGGGAGGAGGGCATACCTCGGCTGGCAGTGCTCCTGTGCCGGAGCTGCCTCGCCTGCAT

GACAAAGTCAGGAGCTCCATGTTCTTCTGCTAG

>Zea-mays_GRMZM2G153859_T04 cds:PUTATIVE_protein_coding

ATGGAGTCTCACAATGGCGAGGCCAATGACTTGCCTCCACCACCTCCTCTGATTGCTGGT

GTTGAACCACTTAAAGCTGATGAAACAAAGATGCCATTGAAACCTAGGAGTCTGGTCCAG

AGAAATGGATTTGGCAGAAAGGGGCAGCCAATAAAGCTGATAACAAATCACTTCAAAGTT

TCTCTTGTGAATGCTGAAGAATTTTTCTACCATTACTATGTCAATTTGAAGTATGAAGAT

GATACACCGGTTGATCGCAAAGGGTCAGGAAGGAAAGTGATTGAAAAACTGCAGCAAACT

TATGCTGCTGAACTTGCAAATAAAGATTTTGCCTATGATGGTGAGAAGAGCCTGTTCACA

ATTGGTGCTCTTCCTCAAGTTAAAAATGAGTTTACTGTCGTGGTTGAAGATTTTTCAACT

GGAAAGACTCCTGCAAACGGCAGTCCAGGAAATGACAGTCCTCCCGGAAGTGACAGGAAA

AGGGTCAGAAGGCCTTACAATACAAAGACCTATAAGGTCGAGCTCTCTTTTGCAGCAAAA

ATTCCTATGAGTGCAATCTCACAGGCCTTAAGAGGTCAGGAATCAGAGCACACTCAGGAA

GCAATTCGAGTGATTGACATTATTCTGAGGCAGCACTCAGCTAAGCAGGGTTGCCTATTA

GTAAGGCAATCATTCTTCCACAACAATCCTTCCAATTTTGTTGACCTGGGTGGTGGTGTA

GTGGGCTGTAGAGGTTTTCATTCTAGTTTTCGAGCAACCCAGAGTGGACTTTCACTCAAT

ATCGATGTGTCGACTACAATGATAGTGAAACCTGGTCCTGTCATTGATTTTCTGCTTGAC

AATCAGAAAGTTGGTGATCCAAGCATGATTGATTGGGCTAAGGCCAAGCGTGCACTGAAG

AACTTGAGGATAAAAATAAGTCCAGCGAACCAAGAACAGAAGATTGTTGGTCTCAGCGAA
AGAACTTGTCGTGAGCAATTATTCACACTGAAACATAAAAATGGTAACAATGGTGACTCT
GAAGAGATCACTGTTTATGATTACTTCGTAAAGCAGCGTGGCATAGTGCTGCAATACTCT
GGTGATCTTCCTTGCATCAATGTGGGAAAACTAAAGCGGCCAACATATTTTCCAATTGAG
TTATGCAGTCTTGTGCCTTTACAAAGATACACTAAAGCTTTGAACACACTTCAGAGGTCA
TCACTCGTGGAGAAATCTAGGCAGAAACCGCAGGAAAGGATGTCTGTTTTATCTGATGTG
CTGCAAAGAAGCAACTATGATGCAGAGCCCATGTTGAAGGCATGCGGGATTACAATTGCT
AGAAATTTCACAGAAGTTGATGGTAGGGTATTGCAGCCACCTAAGCTTAAAGCTGGGAAT
GGTGAAGACATTTTTACACGCAATGGTAGATGGAACTTCAACAATAAGAGGCTCATTAGA
GCTTGTAGTGTCGAGAAATGGGCGGTGGTAAACTTTTCTGCACGATGCAATGTCAGGGAT
CTTGTCCGGGATCTCATCAAGTGTGGAGGCATGAAGGGCATTATGGTTGATGCTCCTTTT
GCTGTATTTGATGAGAATCCTTCAATGAGACGGTCACCTGCTATAAGAAGGGTTGAAGAC
ATGTTTGAACAAGTGAAAACTAAGCTTCCTGGAGCACCAAAGTTTCTTTTGTGTGTTCTA
GCTGAAAGGAAGAATTCTGATATTTATGGGCCTTGGAAGAAGAAATGCCTTGCTGAATTT
GGGATCGTTACACAATGTGTGGCACCAACTAGAGTGAACGACCAGTATCTTACAAATGTC
CTACTTAAGATAAATGCAAAGCTGGGTGGCATGAATTCGTTGCTCCAAATTGAAACATCC
CCAGCAATTCCTCTTGTATCCAAGGTCCCAACTATAATCTTGGGAATGGATGTGTCACAC
GGTTCTCCTGGACATTCTGATGTACCATCTATTGCTGCTGTTGTTAGTTCTCGTGAATGG
CCTCTTATCTCGAAATACAGAGCTTCTGTCCGCACCCAATCACCTAAAATGGAAATGATT
GACTCATTGTTTAAGCCACGGGAAGCTGAAGATGATGGTCTGATCCGGGAGTGTCTGATT
GACTTCTACACCAGTTCTGGGAAGAGAAAGCCTGACCAAGTTATCATATTCAGGGACGGT
GTTAGCGAAAGTCAGTTTAATCAGGTGCTGAACATTGAGTTGCAACAAATCATCGAGGCT
TGCAAATTTCTTGATGAGAAATGGAATCCCAAGTTCACGTTGATTATTGCCCAGAAGAAT
CATCACACTAAATTTTTCATTCCTGGAAAGCCAGATAATGTCCCACCAGGAACTGTGGTG
GACAACAAAGTCTGCCATCCAAAGAACTTCGATTTCTACATGTGTGCGCATGCTGGAATG
ATCGGGACTACGAGGCCAACTCACTACCACATCCTGCATGATGAGATAGGCTTCAGTCCT
GATGATCTGCAGGAGCTGGTGCATTCGCTCTCTTATGTGTACCAAAGGAGCACAACAGCC
ATATCAGTCGTTGCTCCCATCTGCTACGCACATCTGGCAGCTGCTCAGGTTGGCCAGTTC
ATAAAGTTCGATGAGATGTCGGAGACGTCCTCCAGTCATGGCGGGCATACTTCGGCGGGC
AGCGTTCCGGTCCAGGAGCTGCCGCGCCTGCATGAGAAAGAAGGCTCGAGCTGGTACGAT
GTTAAATGTTAG

>Zea-mays_GRMZM2G165242_T02 cds:_protein_coding

ATGGAGTCTCACAATGGCGAGGCTGATGATTTGCCTCCACCACCCCCTTTGGCTGCTGGT

GTTGAACCACTTAAAGCCGATGAAACAAAGATGCCATTGAAACCTAGGAGTCTGGTCCAG

AGGAATGGCTTTGGCAGAAAGGGGCAGCAGATAAAGCTGATAACAAACCACTTCAAAGTT

TCTCTCATGAAGGCTGAAGATTTTTTCTATCATTACTATGTCAATTTGAAGTATGAAGAT

GATACACCGGTTGATCGGAAAGGGTCGGGAAGAAAAGTGATCGAAAAACTGCAGCAAACT

TATGCTGCTGAACTTGCAAATAAAGATTTTGCATATGATGGTGAGAAGAGCCTGTTTACA

ATTGGTGCCCTTCCTCAAGTTAAAATGGAATTCACTGTTGTGGATGAAGACGTTTCAACT

GGAAAGACTCCTGCAAATGGCAGTCCAGGCAATGATAGTCCTCCTGGAAGTGACAGGAAA

AGGGTCCGAAGGCCTTACAATACAAAGACATATAAGGTTGAACTATCTTTTGCGGCAAAA

ATCCCTATGAGTGCAATCTCACAGGCTTTGAGGGGTCAGGAGTCAGAGCACACTCAGGAA

GCAATTCGAGTGATTGACATTATTCTGAGGCAGCACTCAGCTAAGCAGGGTTGCCTATTA

GTAAGGCAATCGTTCTTCCACAATAATCCTTCCAATTTTGTTGACCTGGGTGGTGGTGTA

GTGGGATGTAGAGGGTTCCATTCTAGTTTTCGTGCAACCCAGAGTGGACTTTCACTCAAT

ATCGATGTGTCCACCACAATGATAGTGAAACCTGGTCCTGTCATTGACTTTCTGATTGCC

AATCAGAAAGTTAATGATCCAAGCATGATTGATTGGGCAAAGGCCAAGCGCTCACTGAAG

AACTTAAGGATAAAAACAAGTCCGGCGAACCAAGAACAGAAGATTGTTGGTCTCAGCGAC

AGGCCTTGCCGTGAGCAATTATTCACACTGAAACATAAAAATGGTGAATCTGAAGAGATC

ACTGTTTTTGATTACTTTGTAAAGAACCGTGGCATAAAGCTGGAATATTCTGGTGATCTT

CCTTGTATCAATGTGGGAAAACCAAAGCGTCCAACTTATTTTCCAGTTGAGTTATGCAGT

CTTCTTCCTTTGCAACGGTACACTAAAGCATTGAGCACACTTCAAAGATCATCACTCGTT

GAGAAATCTAGGCAGAAACCACAAGAAAGGATGTCTGTTTTGTCTGATGTGCTGCAAAGA

AGCAACTATGATGCAGAACCCATGCTGAAGGCTTGTGGGATTACAATTGCTAGAAATTTC

ATAGAAGTTGATGGTAGGGTACTGCAGCCCCCTAAGCTTAAAGCTGGGAACGGAGAAGAC

ATTTTTACGCGCAATGGCAGATGGAATTTCAATAATAAGAAGCTCATTAGAGCTAGCAGT

GTCGAGAAATGGGCAGTAGTAAACTTTTCTGCACGATGCAATGTCCGGGATCTTGTCCGT

GATCTCATCAAGTGTGGAGGCATGAAGGGCATTATGGTTGATGCACCATTTGCTGTATTT

GATGAGAATCCTTCAATGAGACGGTCACCTGCTGTAAGAAGGGTTGAAGACATGTTTGAA

CAAGTGAAAACTAAGCTTCCTGGAGCACCCAAGTTTCTTTTGTGTGTTCTAGCGGAAAGA

AAGAATTCCGATATTTATGGGCCTTGGAAGAAGAAATGCCTTGCTGAATTTGGGATCGTT

ACACAGTGTGTGGCACCAACTAGAGTCAATGATCAGTATCTTACAAATGTCTTGTTAAAG

ATTAACGCAAAGTTGGGTGGCTTGAATTCGTTGCTCCAAATTGAAACATCCCCAGCAATT
CCTCTCGTATCCAAGGTCCCAACTATAATCTTGGGTATGGACGTATCACACGGATCTCCT
GGACATTCTGATATACCGTCTGTTGCTGCTGTTGTTAGTTCTCGTGAATGGCCTCTTATC
TCAAAATACAGAGCATCTGTCCGCACCCAATCACCAAAAATGGAAATGATTGACTCGTTG
TTTAAGCCACGGGAAACTGATGACGATGGTCTGATTCGGGAGTGTCTGATTGACTTCTAC
ACCAGTTCAGGGAAGAGAAAGCCCGACCAAGTCATCATCTTCAGGGACGGTGTTAGTGAA
AGTCAGTTTAATCAGGTGCTGAACATTGAGTTGCAACAAATCATTGAGGCTTGCAAATTT
CTTGATGAGAAATGGAATCCCAAGTTCACATTGATCATTGCCCAGAAGAATCACCACACT
AAATTTTTCATTCCTGGAAAGCCAGATAATGTTCCAGCTGGCACTGTTGTTGACAACAAA
GTCTGTCATCCAAGGAACTTCGATTTCTACATGTGTTCACATGCTGGAATGATTGGAACT
ACCAGGCCAACTCACTATCACATCCTGCATGACGAGATAGGCTTCAATCCTGATGACCTG
CAGGAGCTGGTGCACTCGCTCTCTTATGTGTACCAAAGGAGCACAACAGCCATATCAGTT
GGTAAGTTTATCTTGGCACCGTTTTTCGTTTATGTCAAACTCTTATCACCTGCATCTGTG
GGAATTTGA

>Zea-mays_GRMZM2G317927_T01 cds:_protein_coding

ATGGTGAGGAAGAAAAGGACTGGCCCTGGAGAGAGTTCTGGGGAGACTTCTGGAGCGCCT
GGTCAGGGCTCCTCACAGCGTCCTCAGGCAACACAACAGGGTGCCCGAGGTGGAGGACAA
CACCAGGTGCGTGGTGGATATCCGGGCCATGGAGTGCCGCCTTCAGAGCACCCAGGTGGT
GGGCCGCCTGAGTATCAACCACGTGGCTATCAGGGACGTGGCGTTCCACCTTTACTTCCT
GGTGGTGGGCCGCCTGAGCCTCAACCGCGTGGCTACCAGGGACATGGTGGATACCAGGGC
CGTGGCGGGCCACCTTCACAGCATCCTGGTGGTGGGCCATCACCTGGGTCTCAGCCTCGT
GGCTACCAGGGACGTGGTGGTCTGCGTCCCAGAGGGGGAGTGCCGCAGCCATACCGTGGC
GGGCATGTGGGAGGTAGTGTTGGACCAATTGTTCCTTCAGGTCCGTCTAGACCAGTTCCC
GAGCTGCACCAAGCCCCAGATGTCCAACATCAAGCCCCTGTGGTGGCAGCACCATCACCA
CCGGGAGCTGGCTCGTCCTCGCAGCCTGGGATGGCCGAGGTGAGCACTGGACAAGTCCAG
CAACTTGTGATTCATGACCAAAGTTCAGCCAGCCAAGTTAGTCAGGTAGCACCAGCATCA
AGCAAAGCTGTTAGATTCCCATTGCGCCCTGGCAAGGGTACGCATGGGTCCAGGTGCATC
GTGAAGGCAAATCATTTCTTTGCTGAGCTGCCTGATAAAGACCTCCACCAATACGATGTA
TCAATAACACCTGTGGTTCCTTCACGTGGTGTCAATCGTGCTGTCATGAAAGAGCTTGTA
AACCTTCATAGACACTCCCATTTGGATGGGCGTCTGCCTGCATATGATGGAAGGAAGAGT
CTTTATACAGCTGGAGCATTGCCATTTACTTCGAAGACATTCGAAATTACTCTGCAAGAT

GAAGAAAACAGTCTTGGTGGAGGACAAAGACACCAAAGGGGCCAGAGGGTCTTTCAGGTG
GTGATCAAATTTGCTGCTCGCGCTGATCTCCACCATTTGGCTATGTTTCTAGCTGGGAGG
CAACCAGATGCTCCTCAAGAGGCTATTCAAGTACTTGACATTGTACTACGTGAATTTCCT
ACTGCCAGGTATTGTCCTGTTGGTAGATCATTTTATTCTCCCAATTTAGGGAGACGCCAG
CAACTTGGTGAAGGTTTGGAAACTTGGCGTGGTTTCTATCAAAGCATAAGGCCCACACAG
ATGGGTCTTTCTCTGAATATTGATATGTCCTCTACTGCATTTATTGAGCCCCTCCCTGTG
ATTGATTTTGTTGCTCAGCTTCTTGACAGAGATATCTCAGTTAGACCATTGTCTGATTCT
GATCGTGTGAAGATTAAAAAAGCCCTACGAGGTGTGAAAGTCGAGGTCACACACCGTGGA
AACATGCGTAGGAAGTATCGGATATCTGGCCTCACTTCACAAGCAACAAGGGAGTTATCA
TTCCCTATTGATGATCGTGGTACTGTTAAGACTGTGGTGCAATACTTCCTGGAGACTTAT
GGCTTTAATATTCAGCACACCACTTTACCTTGTTTGCAAGTGGGCAATCAGCAAAGAATA
AATTATCTGCCTATGGAGGTCTGTAAGATAGTTGAGGGACAGCGTTACTCAAAACGACTC
AATGAGAAACAGATCACTGCTCTACTGAAGGTGACTTGCCAGCGTCCCCAAGAGCGTGAG
AAAGCCATCTTGCAGACTGTGCATCACAATGCCTACTCCGAGGATCCTTATGCCCAGGAA
TTTGGTATAAAGATTGATGAGCGTCTTGCATCCGTTGAAGCTCGTGTTCTGCCTCCTCCA
AGGCTGAAATACCATGATAGTGGCAGAGAGAGGGATGTATTGCCAAGAGTTGGGCAGTGG
AATATGATGAATAAGAAAATGGTCAATGGTGGTAGAGTTAGCAGCTGGGCATGCATTAAC
TTCTCACGAAATGTGCAAGATGGTGCTGCTAGGAGTTTCTGTCATGATCTCGCTTTGATG
TGCCAAGTATCAGGAATGGATTTTGCACTTGAACCTGTGCTGCCCCCTGTCTATGCGAGG
CCTGAACATGTTGAAAGAGCACTAAAGAGACTTTATCAAGATGCCATGAGCATACTCAGG
CCTCAGGGCCGAGAGCTTGACTTGCTGATGGTAATACTGCCTGACAATAATGGCTCTCTT
TACGGGGATCTTAAAAGGATATGTGAGACTGATCTTGGATTGGTCTCCCAATGCTGTCTG
ACTAAACATGTTTTCAAGGCGAACAAGCATCAGTATCTTGCAAATGTTGCCCTGAAAATA
AATGTTAAGGTTGGGGGAAGGAATACTGTACTTGTTGATGCTTTGGCAAGGAGAATCCCC
CTTGTCAGTGACGTAGCTACTATTATCTTTGGTGCTGATGTGACCCATCCCCATCCTGGG
GAAGATTCTAGTCCTTCCATTGCAGCTGTGGTTGCTTCTCAAGACTGGCCTGAGGTTACG
AAGTATGCAGGATTAGTGAGTGCTCAAACCCATCGCCAAGAATTGATACAGGATCTTTTC
AACGTACGGCAAGATCCCCAAAGGGGGGCTGTCTCTGGTGGCATGATTAGGGAACTTCTC
ATTTCTTTCTGGAGGGCGACTGGACAAAAACCCAAGAGGATCATATTCTACAGGGATGGT
GTCAGTGAGGGACAGTTCTACCAAGTTCTGTTGTACGAACTTGATGCCATTAGAAAGGCC
TGTGCATCATTGGAGTCTGATTACCAGCCTCCAGTTACCTTTGTCGTGGTCCAGAAGCGT

CATCACACCAGGTTGTTTGTTAATAATCACAATGATCAGCGTGCTGCCGATAGAAGTGGC

AACATACTGCCGGGCACTGTGGTGGACTCGAAGATTTGCCATCCAACCGAGTTTGATTTC

TACCTGTGCAGCCATGCTGGCATTCAGGGAACAAGCCGTCCTGCCCATTATCATGTTCTG

TGGGATGAGAACAAATTTACGGCTGATGGGTTGCAAACTCTCACCAACAACTTGTGTTAC

ACGTATGCCAGGTGCACACGCTCAGTATCAATCGTTCCTCCTGCATACTATGCTCATCTG

GCAGCCTTCCGAGCTCGGTTTTACATGGAGCCAGATACAAGTGACAGCGGATCTGTGGCG

AGCGGTGCTACGACAAGCCGTGGCCCTCCACCAGGGGCGCGCAACACCAGGGCTGGTGCT

GCGAATGTTGCTGTGAGGCCATTACCTGCTCTCAAGGAAAACGTGAAGCGCGTCATGTTC

TACTGCTAA

>Zea-mays_GRMZM2G347402_T01 cds:_protein_coding

ATGGCTGCCAAAATGGCTGGAGCTGTCCAAGTGCTCAAGGATGACACTGTTAAACGCACA

CCCATGGCACGACCTAGTAACGGCCGTGAAGGAAAGCCCATTAGGTTGCTGTCGAACCAC

TTCTCAGTGAAGCTTAGAGGAGTTGATGCTGTTTTCTACCAATACAGTGTCTGCATCAAA

TCTGAGGATGATAAGGTGGTTGATAGCAAGGGTATTGGCCGAAAGGTCATAGATAAACTA

TTGCAAACATACTGTTCTGAGCTTGATGGGAAGGATTTTGCATATGATGGAGAGAAATGT

CTATTTACTGTGGGACCTCTTCCACAGAATAACTTTGAGTTCACTGTTATCTTGGAGGAA

ACATCTTCAAGGGCTGCTGGTGGAAGTCCAGTGCATGAAAGCCCTACTCAAGCTAACAAA

AAGCGAGTCAAGCGATCACATCTGGCAAAAAAGTTCAGTGTAGACATAAGTTATGCCGCA

AAGATTCCTCTTCAGTCGGTTGCTTTGGCTCTTCGAGGAAGCGAGTCAGAACATGGTCAA

GATGTTCTGAGAGTCCTTGACGTTGTTTTAAGGCAACAGCAGGCTAAGAGAGGTTGTCTA

CTTGTTAGACAGTCATTTTTCAGTGATGATAGTCGAAACCTTGTTGATTTAACTGGTGGA

GTTAGTGGTTGTCGTGGACTCCACTCTAGTTTCCGTACTACAATTGGTGGTCTTTCACTA

AATATGGATGTTTCAACCACTATGGTTGTAACTCCTGGACCAGTTATTGATTTTCTCGTC

ACAAATCAAAATGTAAGAGACATCAGAGATATTGACTGGCCCAGGGCCAAGAAAATGCTT

AAAAATCTCAGAGTTAAAGCTAAGCACAACAACATGGAGTTCAAGATTATTGGCCTTAGT

GATCAACCATGCTCTAGACAGATGTTCCCAATGAAAGTTCGAAATGGAAACATCGAAATT

AAATCTGTTGATATCACTGTTCAGGATTATTTTAAATCCAAGCAAGTTGAGCTAACAATG

CCTTATCTGCCATGTCTTGATGTGGGAAAACCAAAACGCCCTAATTATCTCCCAATTGAG

TTATGCCACATGGTATCACTTCAACGTTATACAAAGGCACTGTCTTCTCAACAAAGGGCA

ATGTTGGTTGAAAAGTCACGACAGAAACCTCAAGAAAGAATGCGAGTTGTTACAGATGCT

GTAAAAAGTAATATGTATGATGATGATCCAATCTTATCTTCATGTGGTATTGAAATTGAG

AAACAACTTACTCGTGTTGACGCTCGTGTTCTCTCTGCACCAGCGCTAGTTGTGGGCAAC

AGTGAAGATTGCATCCCAAACAGGGGTAGGTGGAACTACAATAATAAGAGGCTATTCGAT

CCAGTCAAGATTGAGCGTTGGGCCATTGTTAATTTCTCTGCTCGTTGTGACATGAGCCGA

ATCTCAAGAGAACTGATAAACTGTGGACGCAGCAAAGGCATTTTCATTGAATGTCCTCAC

AGTTTGGTGGATGAGGACAGCCAGTCTAGGAGATGTTCACCTGTGGAAAGGGTTGAAAAG

ATGTTTGAAAAAGTCAAAGCAAGCCTTCCTGGTCCTCCAGAGTTTCTCCTTTGTCTTTTA

CCAGAGAGGAAGAATTGTGATATTTACGGGCCATGGAAGAAGAAAAATCTTCATGAAATG

GGTATTGTCACTCAATGCATTGCTCCAAGTAATAAGATGAATGATCAATATTTCACCAAT

GTTCTTCTAAAAATTAATGCTAAGCTTGGTGGAATGAACTCCAAACTGGCACTGGAACAT

CGTCAAATGATACCAGTTGTGACTCAAATACCAACATTAATTCTTGGCATGGATGTTTCA

CATGGTTCTCCAGGTCGAGCAGATATACCATCAATTGCTGCGGTTGCCCACAACAACTCG

CGTAACAGCAGCCTGCTACGCAAGCTGCGCCTCCCTCTTAGGCATATGCCCCCTACTGGC

GGCAACAACAAAGGGAAGGGCAAAGGAAGATGCGCCAACGACCACTCCTCCAGCGACAAC

CATGACCTTGGCACCGAGAGCATCAACACCCCATGGCTATCGTTCGACAAGCCGTGCACG

GGCATGATCACCATGTGGCACAGACCACGTCCCCCTCTACAACAACATGTGCACCCTCAT

CAACATGCCCTGATGGCTGTCTCGCCCCACCTGGCGCCTAAATGGGAGGGGTTTAGTTTA

TTAGCGTGTTGTGGCGGTTGA

>Zea-mays_GRMZM2G354867_T01 cds:_protein_coding

ATGCAGAGACCTGACTGTGGTGGCGCATTATCTCAAGCAAAGGTCAAACTCTTGGTGAAT

CACTTTATTGTCAACTACCAAAAGGTGTCAACTATTTTTCACTATGACATAAACATCAAG

CTTGATGAAGCTTCCTCTAAGGCTTCAGGCAAAGAACTCTCGAAGGCAGAATTTCTTTCT

GTCAAGGATGAGCTCTTCAGGGAAAGCAGTTTACGGCGTCTTTCCTCATGTGTTGCTTAT

GATGGTGGAAGAAATCTCTTCACTTCTGCTGAACTGCCAGCAGGTTTATTTCGTGTGAGA

GTTCGATCAAAGGCCTACATTGTATCAGTAGATTTGAAGAAGCAGCTGCCATTAAGTCAA

CTCTCAGATTTACCTGTACCTAGAGAGGTCTTGCAGGGTCTTGATGTTGTTGTGCGTGAG

GCCTCCAGATGGCGCAAGGTTATCCTTGGTAGAGGATTTTACTCACCAAGCAGCAGTATA

GACATTGGGCAGGGTGTTGTAGCTATGAAAGGAACCCAGCAGACACTTAAATACACTCAA

CAAGGGTTGAACCTGTGTGTTGATTATTCAGTTATGCCATTTTACAAAGCTGGACCGGTG

ATGGACCTTGTTCACAAAATAGTGGGGTACCTTGATTATCGAACAACTCTGAACAAGAGG

CAAATGGAAAATCTGGTTGATGAGCTTAAAGGCCGACGTGTAACTGTGATTCATCGGAGG

ACTAATCAGAAGTACACAGTGCAAGGCTTGACACCCTTACCTGCCAGCCAGATGACCTTT

GTGGATGCTGAATCCGGACAAACAAAGTGTCTTGTGGAGTATTATGCTCAGAAACATGGC

ATTGTGATTGAGTATCAGATGCTGCCATGCTTGGATTTGAGCAAGAGCAAGGACAAACCG

AATCATGTCCCAATTGAGCTCTGCACTCTTCTTGAAGGACAGAGGTTTCCAAAAGCAAAC

TTGGATAAGAATTCTGGCAGGATACTAAAAGGAAAGGCTCTAATTCCTGCATCCAATCGG

AGGAAAGAGATTCTAGACTTGGTGAATGCTTCGGATGGACCTTGCAGAGGAGAAATTGCA

CAGCGATTTGGGATTTCCTTGGATTTACGAATGACAGAAGTCACGGGTAGGATCCTTCCC

CCACCAAACCTCAAACTCGGGGCATCCAATGGCCAGACCTCCAAATTCAGTATCGATCAG

AACTGCCAGTGGAACCTTGTGAAGAAGAGACTCGTAGAGGGCCGGGATCTTCAGTGTTGG

GGCATTGTCGACTTCAGTGCTGAGCCGTCTGACCCCCAGCAGGAGCCCCTCAATGGAAGG

ATGTTTATTGAGAAGATTGTGAGGAAGTGCTGTGAGCTTGGTATCCGTATGAACTCCAAC

CCATGCTTCGTACACAAATCTAAGATGGCAGTGCTCTCCGATCCGCATCGACTACAGGAG

GAGCTAAACAAGGCAAAACAGGCTGCAGTGAGCAAGAAGCAGAGGTTGCAGCTCCTTTTC

TGCCCGATGTCCGAGCAGCATCCAGGGTACAAGACACTGAAGCTGATTTGCGATACACAG

CTTGGGATCATGACCCAGTGTTTCCTGGGCGACCGCGCAAACAAGCCGAATGGGCAGGAC

CAGTACATGACCAACCTTGCCCTCAAGATAAACGGCAAGCTTGGGGGCAGCAACGTCCAG

CTGTTCGACTCGCTCCCACGGGTCGGTGGGGCACCTTTCATGTTCATCGGTGCTGACGTC

AACCACCCGTCACCGGGGAACGTGGAGAGCCCATCGATTGCAGCCGTGGTTGCGTCTATC

AACTCCGGTGTCAGCAAGTACGTGACAAGAATCCGTGCCCAGCCGCACCGCTGTGAGGTG

ATCCAGCAGCTCGGCGAGATCTGCCTGGAGCTCATCGGAGTCTTCGAGAAGCGAAACCGC

GTGAAGCCGCAGAAGATCATCTACTTCCGCGACGGCGTGAGCGACGGGCAGTTCGACATG

GTCCTGAACGAGGAGCTGGCGGACCTGGAGAAGGCGATCAAGGTGGGCGGCTACGCGCCG

ACCGTCACCGTGATCGTGGCCAAGAAGCGGCACCACACGCGCCTGTTCCCCAAGGACCCC

AGCCAGCCGCAGACGAAGAACGGGAACGTGCCGCCCGGCACGGTGGTGGACACGGGCGTG

GTGGACCCGTCCGCGTACGACTTCTACCTGTGCAGCCACGCCGGGATCCTGGGCACGAGC

AGGCCGACGCACTACTACAGCCTGGTGGACGAGCACGGCTTCCGGTCCGACGACCTGCAG

AAGCTGGTCTACAACCTCTGCTTCGTGTTCGCGCGGTGCACCAAGCCCGTGTCGCTGGCG

ACGCCCGTCTACTACGCCGACCTCGCGGCGTACCGTGGCAGGCTCTACTACGAGGCGGCC

ATGATGCCGTCCCACCAGCGAGGGACGGGGTCGGCGTCCTCGGGCTCCTCCGCTGGGACT

TTTGGCGTCACTAACTTCCCGAGGCTGCACAAGGATGTGGAGAACAACATGTTCTTCATC

TGA

>Zea-mays_GRMZM2G359875_T01 cds:_protein_coding

ATGGTGAGGAAGAAGAGAACTGGCCCTGGTGGCTCTGGAGAAACTTCTGGAGAGTCTTCA
GGAGCCTCTGGACAAGGTTCCTCACAGCAGCCTGAGCGAACTCAACAACCTGGGGGAGGA
CGTGGCTGGGTGCCTCAACAGGGTGGCCATGGTGGTGGGCAACACCAGGGTCGTGATCGA
CATTATCAGGGACGTGGAGGACCAGGGCCACATCACCTTGGTAGTGGGGCACCTGAGTAT
CACCCGCGTGAATACCAGGGACGTGGTGGTGAATATCAGGGACATGGTGGTGAGTACCAG
GGACGGGGTGGTGACTACCAGGGACGTGGTGGTGGCCGCTCCAGAGGTGGAATGCCACAG
CCATACTATGGTGGGCATAGGGGAGGTAATGTTGGACGCAATGTTCCTCCAGGTCCGTCC
AGGACAGTTCCCGAGCTGCACCAAGCCCCATATGTCCAGTATCCAGCCCCGGTGGTTTCG
CCCTCCCCATCGGGACCTGGCTCATCCTCACAGCCTATGGCAGAGGTGAGCTCTGGACAA
GTCCAGCAACAGTTTCAGCAACTTGCCGATCGTGGTCAGAGTTCCACGAGCCAAGAAATT
CAAGTGGCACCAGCATCAAGCAAATCGGTTCGATTCCCGTTACGGCCCGGCAAGGGCACT
TATGGGGACAGGTGCATTGTGAAGGCAAATCATTTTTTTGCTGAGCTTCCTGACAAAGAC
CTTCACCAATATGATGTATCTATAACACCTGAGGTTACTTCACGTGGCGTCAATCGTGCT
GTCATGGGTGAGCTTGTAACAATATATAGACAATCCCATTTGGGTGGGCGTCTACCTGCA
TACGATGGAAGAAAGAGCCTGTATACTGCTGGACCATTGCCATTTACTTCTATGGCATTT
GAAATTACCTTGCAAGATGAGGAAGATAGTCTTGGCGGTCGCCAAGGTGGACATAGGCGT
GAGAGAGTATTTAGGGTGGTGATCAAATTTGCAGCCCGTGCTGATCTCCACCATCTGGCT
ATGTTTCTAGCTGGAAGGCAAGCAGATGCCCCTCAGGAAGCTCTTCAAGTGCTTGACATT
GTACTACGTGAATTGCCTACCGCGAGGTATTCTCCTGTCGGTAGGTCATTTTACTCTCCC
AACTTAGGGAGACGTCAAAAACTTGGTGAGGGATTGGAAAGTTGGCGTGGTTTTTACCAA
AGCATAAGGCCGACACAGATGGGCCTTTCACTGAATATTGATATGTCCTCTACTGCATTT
ATCGAGCCTCTCCCTGTGATCGATTTTGTTGCTCAGCTTCTTAACAGAGATATCTCAGTT
AGGCCATTGTCTGATTCTGATCGCGTGAAGATTAAAAAAGCCCTAAGAGGTGTGAAGGTT
GAGGTGACTCACAGGGGAAACATGCGCAGAAAATATCGCATTTCTGGCCTCACCTCACAA
GCAACAAGAGAGCTATCATTCCCTGTTGATGATCGTGGTACTGTGAAGACTGTGGTGCAA
TACTTCATGGAGACTTATGGTTTTAGTATCCAGCACACCACTTTACCATGCTTGCAAGTG
GGTAATCAACAAAGACCAAATTATCTGCCTATGGAGGTTTGCAAGATAGTTGAAGGACAG
CGTTACTCAAAGCGACTCAATGAGAAACAAATCACTGCTCTACTGAAAGTGACCTGCCAG
CGCCCTCAAGAGCGCGAGCTGGACATCTTACAGACTGTGCATCACAATGCATACTATGAA
GACCCCTATGCACTGGAATTTGGTATAAGAATTGATGAACGTCTTGCTGCAGTTGAAGCT
CGTGTTCTGCCACCACCAAGACTTAAATACCATGATAGTGGCCGAGAGAAGGATGTTTTG

CCCAGAGTTGGCCAATGGAACATGATGAATAAGAAAATGGTTAATGGTGGCAGAGTGAGC
AACTGGGCATGTATTAACTTCTCTCGGAATGTGCAAGATAGTGCCGCTAGGGGTTTCTCT
CATGAGTTGGCAGTCATGTGCCAAATATCAGGAATGGATTTTGCTCTTGAGCCTGTGCTG
CCTCCAGTGACTGCAAGGCCAGAACATGTTGAGAGAGCGTTAAAGGCACGTTATCAAGAT
GCAATGAACATACTGAGGCCACAGGGAAGGGAACTTGATCTGCTGATCGTAATACTGCCT
GACAACAATGGTTCTCTTTATGGGGATCTCAAAAGGATCTGTGAGACTGAACTCGGATTG
GTCTCCCAGTGTTGTCTGACTAAACATGTTTTTAAGATGAGCAAGCAGTACCTTGCAAAT
GTTGCACTCAAAATAAATGTTAAGGTTGGGGGAAGGAATACTGTACTTTTAGATGCTTTG
TCAAGGAGAATCCCCCTTGTCAGTGACAGACCGACCATAATATTTGGTGCTGATGTTACC
CATCCACATCCTGGAGAAGATTCCAGTCCTTCCATTGCAGCCGTTGTTGCTTCGCAAGAC
TGGCCCGAGGTCACGAAATACGCTGGACTAGTGAGTGCGCAAGCCCATCGCCAGGAGCTG
ATACAGGATCTTTTCAAAGTATGGCAGGACCCGCAGAGAAGGACGGTAACTGGCGGCATG
ATAAAGGAACTTCTCATTTCTTTCAAGAGGGCAACTGGACAGAAGCCCCAGAGGATCATA
TTCTACAGGGATGGTGTCAGTGAGGGACAGTTCTATCAAGTATTGCTGTACGAACTTGAT
GCCATTAGAAAGGCCTGTGCGTCCCTGGAGCCCAACTACCAGCCTCCAGTTACTTTTGTC
GTGGTACAGAAGCGCCATCACACTAGGCTGTTTGCGAACAACCACAGTGATCAGCGCACA
GTCGATAGAAGCGGAAACATACTGCCTGGCACCGTGGTCGATTCGAAGATTTGCCATCCT
ACTGAGTTTGACTTCTACCTGTGTAGCCATGCTGGCATTCAGGGAACGAGCCGCCCTGCT
CACTACCATGTCCTGTGGGACGAGAACAAGTTCACAGCTGACGAGCTGCAGACCCTGACG
AACAACCTGTGCTACACGTACGCTAGGTGCACCCGCTCCGTGTCCATCGTGCCCCCGGCG
TACTACGCTCATCTGGCAGCCTTCCGAGCTCGCTTCTACATGGAGCCAGACACCTCTGAC
AGCGGGTCACTGGCCAGCGGTGCCCGTGGCCCCCCACCCGGTGCGGCACGCAGCAGCACG
AGAGGGGCCGGGAGTGTCGAGGTCAGGCCCCTACCTGCTCTCAAGGAGAACGTGAAGCGT
GTCATGTTTTACTGCTGA
>Zea-mays_GRMZM2G361518_T01 cds:_protein_coding
ATGTATATTACTCAACCAAAAAAAAAGCGCATTGAGTTTGTCAGTTTCACTGCCCACGGC
ATGGGATCTTGGAGACAAAAACTGCCTGGGTTAGGCGAGGGCTCAGGCTCAGGCTCACAG
GCTGCTGAGGCCGGCGGTGGTGGGAGAGGTTTCCGTGGCCGTGGTGGCTTCTACCATCAA
CAGCAGTCCCCACAAGGTGGTCGTGGTCGTGGAACTGGCTACTACAGTGGTGGTGGTGGT
GGTAGAGGTGGACGTGGGGCAGTCCCGTCCGCCATAGCTCCCGAGCTGCGCCAAGCAATG
CAAACTTCAAATGAACCTGATAACATCCCAGCGCCAGAGGCAGGCTCCCAGTCCCAGGAT

GCGCCGTCACCAAGCGAAGAGGCTGTGGATCAGCTGAAAGGCTTGTCTGTACAGGACATT

GTGCAGGCGTTTCCAGTGTCGAGCAAGTTCCCTCACCGCCCAGGAAATGGAAGCGTTGGC

ACCAGGTGTTTGGTGAAGGCAAATCACTTCTTTGCTGAATTATTACCTGCCAAGAAGGAT

CTTCATCAGTATGATGTTTCAGTTACCCCGGAAGTGACATCGCGAATTGTGAACCGTTCT

GTCATGGAGGAGCTGGTGAGGCTGCACAAGCTGTCATATTTGGGAGGTCGGCTTCCAGCC

TATGATGGTAGAAAGAGCCTGTACACGGCTGGACCCCTGCCATTCACTTCGAAAGAATTT

CACATCACTCTACTCGAGGAAGACGATGGTTCTGGAGTAGAGAGGCGTAAGAAGACATAC

AAGGTAGTGATTAAATTTGCCGCAAGGGCTGACCTCCGGCGTCTGGAGCAGTTTTTAGCT

GGAAGGCAGGCAGAGGCTCCTCAAGAAGCCTTGCAAGTTCTTGATATTGTTCTGCGGGAG

CTGCCGACAACAAGATATGCACCGTTTGGGCGATCGTTTTTCTCTCCTGACCTGGGGAGG

AGGCGTTCCCTTGGTGAGGGAATAGAAAGCTGGCGTGGGTTTTACCAGAGCATTCGCCCT

ACTCAAATGGGCTTGTCATTGAATATTGATATGTCTGCAACTGCTTTCTTTGAGCCATTA

CCTGTCATAGATTTTGTTGCACAGCTTTTAAACACCGACGACATCTACTCGAGGCCCCTC

TTAGACGCTGAACGTGTCAAGATAAAGAAGGCCTTAAGAGGCGTGAAGGTGGAAGTTACG

CACCGTGGTAACATGCGACGCAAGTATCGAATAGCTGGTTTAACATCTCAGGAAACTCGG

GAGCTAACTTTTCCTGTTGATCAAGGTGGCACAGTGAAGTCAGTTGTACAGTATTTTCAA

GAGACCTATGGCTTTGCCATCCAGCACACCTACCTGCCCTGTCTGCAGGTTGGCAACCAG

CAGCACCCAAATTACCTTCCAATGGAGGTCTGCAAGATAGTGGAGGGACAGAGGTACTCT

AAGAGATTAAACCAGAGTCAGATAAGAGCTCTTTTAGAGGAGACATGCCAACGCCCACAT

GATCGTGAGCGCGACATTATTCAGATGATGAATCATAACTCTTACCATGAGGATCCTTAT

GCTAAAGAGTTTGGCATTAAGATCAGCGAGCGTTTGGCTTCGATTGAGGCACGGATTTTA

CCTGCTCCTCGGCTCAAATATAATGAAACCGGCAGAGAGAAGGACTGTTTGCCTAGAGTT

GGGCAGTGGAATATGATGAACAAGAAAATGGTAAACGGTGGCAGAGTGAGGAGCTGGACC

TGTGTGAATTTTGCTCGGAATGTGCAAGAGAATGTTGCTATTGGATTCTGCCGTGAACTT

GCTCGGATGTGCCAGGCCTCGGGAATGGACTTCGCGCTGGAGCCTATCCTTCCGCCTATA

TACGCGCATCCTGATAAAGTGGAGAGAGCTCTGAAAGCCAGGTTCCATGACGCGATGAAC

TTGCTCGGACCACAGCGCAGAGAACAACTCGACTTGCTGCTCATTGGAATACTCCCTGAT

AACAACGGTTCTCTTTATGGTGATCTGAAGCGTATCTGTGAAATCGACCTTGGATTAGTT

TCCCAGTGCTGCTGCGCAAAGCAAGTCTTTAAGATGAACAAGCAGATACTGGCAAACCTT

GCGCTGAAGATAAATGTCAAGGTCGGAGGAAGGAACACGGTGCTGGCTGATGCGGTGTCA

AGACGCATTCCCTTGGTGACTGACAGGCCTACCATCATATTCGGTGCCGATGTGACCCAT

CCTCATCCTGGAGAAGATAGCAGCCCTTCCATTGCTGCTGTTGTGGCTTCCCAAGACTGG
CCTGAGGTGACAAAGTATGCTGGTCTAGTTTCTGCTCAGTCTCATAGGCAAGAGCTGATA
GAGGATCTCTACAACGTCACACACGATCCTCAGAAAGGAACCGTTTGTGGTGGCATGGTC
AGGGAGCTTCTTATATCCTTCAAAAAATCAACTGGTCAAAAGCCTCAGAGGATACTATTC
TACAGGGATGGTGTGAGTGAAGGGCAGTTCTACCAAGTTCTACTGCATGAACTGGATGCT
ATCCGAAAGGCGTGTGCATCGCTGGAAGCAAACTACCAACCGCAGGTGACTTTCATCGTC
GTCCAGAAACGCCACCATACCAGGCTGTTCGCGCACAACCACAACGACCAGAATTCGGTC
GACAGGAGCGGCAACATACTTCCTGGAACTGTCGTGGACTCGAAGATCTGCCACCCTACA
GAGTTCGACTTCTTCCTGTGCAGCCATGCTGGCATCAAGGGCACCAGCCGTCCTGCTCAC
TACCATGTCTTGTGGGACGAGAACAACTTCACAGCCGACGCACTGCAGACCCTCACCAAC
AACCTTTGCTACACCTACGCGAGGTGCACGCGCTCTGTGTCCATTGTCCCGCCGGCGTAC
TACGCTCACCTGGCCGCATTCCGCGCCCGGTTCTACATGGAGCCTGACAGCTCAGACAGC
GGGTCGCTGGCGAGTGGCGCCCGTGGAGGCGGAGCGCCCTCCAGCTCGTCAACGTCCCGC
AGCACCCGCGCCACCGCCGGCGGAGCCGTTAGACCCCTCCCCGCGCTCAAGGACAGCGTC
AAGAAGGTCATGTTCTACTGCTGA

>Zea-mays_GRMZM2G366277_T02 cds:_protein_coding

ATGGCCAGAGTAGAACCAATAGGATCAACACTAGCTGCCACATCTTCTGTTAATACACGT
GTGCCAATGCAGAGACCTGATTCTGGAGGTTCATTATCTCAAGCAACGGTTAAACTTTTG
GTGAATCACTTTATTGTCAGCTACCGAAAGGTGACAACTATTTTTCATTATGACATAAAC
ATCAAGCTTGATGAAGCTTCGTCTAATGCTTCGGGCAAAGAGCTATCCAAGGCAGAATTT
CTCTCTGTCAAGGATGAGCTCTTCAGGGAAAGCAGTTTACGGCGTCTTTCCTCATGTGTT
GCTTATGATGGTGGAAGAAATCTGTATACTTCTGCTGAACTGCCTGCAGGTTTATTTCGT
GTGAGAGTTCGATCAAAGACCTACATTGTATCAGTAGATTTGAAGAAGCAGCTGCCATTA
AGTCAACTCTCAGATTTACCTGTGCCTAGAGAGGTCTTGCAGGGCCTTGATGTTGTTGTG
CGTGAGGCCTCAAGATGGAACAAGATTATCCTTGGTAGAGGATTTTACTCACCAAGCAGC
AGTATAGACATTGGGCAGGGTGCTGTAGCTATGAAAGGAACCCAGCAGTCCCTTAAATCC
ACTCAGCAAGGGTTGATCCTGTGTGTTGACTATTCTGTCATGCCGTTTTACAAAGCTGGA
CCGGTGATGGATCTTGTTCAGAAATTAGTGAGGTACCTTGATTATCGGACAACTTTGAAC
AAGAGGCAAATGGAAAATCTGGTTGATGAGCTTAAAGGCCGACGTGTAACTGTGATTCAT
CGGAGGACTAATCAGAAGTACACAGTGCAAGGTTTGACACCCTTACCTGCCAGCCAGATG
ACCTTTGTGGATGCTGAATCCGGCCAAACACGGAGGCTCGTGGATTATTATGCTCAGAAA

CATGGCAAGGTGATTGAGTATCAGATGCTGCCATGCTTGGATTTGAGCAAGAGCAAGGAC

AAACCGAATCATGTTCCAATTGAGCTCTGCACTCTTCTTGAAGGACAGAGGTTTCCAAAA

GCAAACTTGAATCAGAATTCTGAGAGGATACTAAAAGGAAGTGCTCTAATCCGTGCATCT

GACCGGAGGAAGGAGATTCAAAACTTGGTGAATGCTTCGGATGGACCGTGCAGAGGAGAA

ATTGCACAGCAATTTGGGATTTCCTTGGATGTACGAATGACAGAAGTCACGGGTAGGATC

CTTCCTCCACCAAACCTCAAACTTGGGGCATCCAATGGGCAGACCTCCAAATTGAGTATC

GATCATGGCTGCCAGTGGAATCTTGTGAAGAAGAGACTAGTAGAGGGCCGGGTTCTTCAG

TGCTGGGGCATCGTCGACTTCAGTGCTGAGCCGTCTGGCTCTGGCGCCCGTCAGGAGCCC

CTCGATACAAGGATGTTTGTTGAGAAGATTGTGAGGAAGTGCTGTGAGCTTGGTATCCGT

ATGAACCCTAATCCATGCTTCGTGCACATAACAAGGATGGCAGTGCTCTTCGATCCACAT

GGACTACATGAAGAGCTAAACAAAGCAAAACAAGCTGCAGTGAGCAAGAAGCAGAGGTTG

CAGCTCCTTTTCTGCCCGATGTCCGAGCAGCATTCGGGGTACAAGACACTGAAGCTGATT

TGTGACACACAGCTGGGGATCCTGACCCAGTGTTTACTGAGCGACCGCGCAAACAATCGA

AAGGGACAGGACCAGTACATGACGAATCTTGCTCTAAAGATCAACGGCAAGCTTGGGGGC

AGCAACGTTCAGCTGTTTGACTCGCTCCCACGGGTCGGTGGTGGGGTACCTTTCATGTTC

ATCGGTGCTGACGTTAACCACCCGTCCCCCGGTAACGTGGAGAGCCCATCGATCGCAGCC

GTGGTCGCCTCTGTCAACTCTGGTGTCAACAAGTATGTGACCAGAATCCGTGCCCAGCCG

CACCGCTGCGAGGTGATCCAGCAGCTTGGTGAGATCTGCCGGGAGCTCATTGGAGTCTTT

GAGAAGCAGAACCGCGTGAAGCCGCAGAAGATCATCTACTTCCGTGATGGCGTGAGCGAC

GGGCAGTTCGATATGGTCCTGAACGAGGAGCTGGCTGACCTGGAGAAGGCGATCAAGGTG

AATGGCTATGCGCCAACCATCACCGTGGTCGTGGCCAAGAAGCGGCACCACACTCGGCTG

TTCCCCAGGGACGAACAGCAGCCGCAGACGAAGACCGGGAACGTGCCGCCTGGCACGGTG

GTGGACACGGGCGTGGTGGACCCGTCCGCGTACGACTTCTACCTGTGCAGCCACACTGGG

ATTCTGGGGACGAGCAGGCCGACGCACTACTACACCCTGGTGGACGAGCACGGCTTCGGC

TCCGACGACCTGCAGAAGCTGATCTACAACCTGTGCTTCGTGTTCGCGCGGTGCACCAAG

CCGGTGTCGCTGGCGACGCCCGTCTACTATGCCGACCTCGCGGCCTACCGTGGCAGGCTC

TACTACGAGGCTGCCATGATGGCGTCCCAGGCCCAGCGAGGGGGGTCCTTTGACGTCACT

AACTTCCCGAGGCTGCACAAGGATGTGGAGGACAACATGTTCTTCATCTGA

>Zea-mays_GRMZM2G411082_T01 cds:_protein_coding

ATGTATATTACTCAACCAAAAAAAAAGCGCATTGAGTTTGTCAGTTTCACTGCCCACGGC

ATGGGATCTTGGAGACAAAAACTGCCTGGGTTAGGCGAGGGCTCAGGCTCAGGCTCACAG

GCTGCTGAGGCCGGCGGTGGTGGGAGAGGTTTCCGTGGCCGTGGTGGCTTCTACCATCAA
CAGCAGTCCCCACAAGGTGGTCGTGGTCGTGGAACTGGCTACTACAGTGGTGGTGGTGGT
GGTAGAGGTGGACGTGGGGCAGTCCCGTCCGCCATAGCTCCCGAGCTGCGCCAAGCAATG
CAAACTTCAAATGAACCTGATAACATCCCAGCGCCAGAGGCAGGCTCCCAGTCCCAGGAT
GCGCCGTCACCAAGCGAAGAGGCTGTGGATCAGCTGAAAGGCTTGTCTGTACAGGACATT
GTGCAGGCGTTTCCAGTGTCGAGCAAGTTCCCTCACCGCCCAGGAAATGGAAGCGTTGGC
ACCAGGTGTTTGGTGAAGGCAAATCACTTCTTTGCTGAATTATTACCTGCCAAGAAGGAT
CTTCATCAGTATGATGTTTCAGTTACCCCGGAAGTGACATCGCGAATTGTGAACCGTTCT
GTCATGGAGGAGCTGGTGAGGCTGCACAAGCTGTCATATTTGGGAGGTCGGCTTCCAGCC
TATGATGGTAGAAAGAGCCTGTACACGGCTGGACCCCTGCCATTCACTTCGAAAGAATTT
CACATCACTCTACTCGAGGAAGACGATGGTTCTGGAGTAGAGAGGCGTAAGAAGACATAC
AAGGTAGTGATTAAATTTGCCGCAAGGGCTGACCTCCGGCGTCTGGAGCAGTTTTTAGCT
GGAAGGCAGGCAGAGGCTCCTCAAGAAGCCTTGCAAGTTCTTGATATTGTTCTGCGGGAG
CTGCCGACAACAAGATATGCACCGTTTGGGCGATCGTTTTTCTCTCCTGACCTGGGGAGG
AGGCGTTCCCTTGGTGAGGGAATAGAAAGCTGGCGTGGGTTTTACCAGAGCATTCGCCCT
ACTCAAATGGGCTTGTCATTGAATATTGATATGTCTGCAACTGCTTTCTTTGAGCCATTA
CCTGTCATAGATTTTGTTGCACAGCTTTTAAACACCGACGACATCTACTCGAGGCCCCTC
TTAGACGCTGAACGTGTCAAGATAAAGAAGGCCTTAAGAGGCGTGAAGGTGGAAGTTACG
CACCGTGGTAACATGCGACGCAAGTATCGAATAGCTGGTTTAACATCTCAGGAAACTCGG
GAGCTAACTTTTCCTGTTGATCAAGGTGGCACAGTGAAGTCAGTTGTACAGTATTTTCAA
GAGACCTATGGCTTTGCCATCCAGCACACCTACCTGCCCTGTCTGCAGGTTGGCAACCAG
CAGCACCCAAATTACCTTCCAATGGAGGTCTGCAAGATAGTGGAGGGACAGAGGTACTCT
AAGAGATTAAACCAGAGTCAGATAAGAGCTCTTTTAGAGGAGACATGCCAACGCCCACAT
GATCGTGAGCGCGACATTATTCAGATGATGAATCATAACTCTTACCATGAGGATCCTTAT
GCTAAAGAGTTTGGCATTAAGATCAGCGAGCGTTTGGCTTCGATTGAGGCACGGATTTTA
CCTGCTCCTCGGCTCAAATATAATGAAACCGGCAGAGAGAAGGACTGTTTGCCTAGAGTT
GGGCAGTGGAATATGATGAACAAGAAAATGGTAAACGGTGGCAGAGTGAGGAGCTGGACC
TGTGTGAATTTTGCTCGGAATGTGCAAGAGAATGTTGCTATTGGATTCTGCCGTGAACTT
GCTCGGATGTGCCAGGCCTCGGGAATGGACTTCGCGCTGGAGCCTATCCTTCCGCCTATA
TACGCGCATCCTGATAAAGTGGAGAGAGCTCTGAAAGCCAGGTTCCATGACGCGATGAAC
TTGCTCGGACCACAGCGCAGAGAACAACTCGACTTGCTGCTCATTGGAATACTCCCTGAT

AACAACGGTTCTCTTTATGGTGATCTGAAGCGTATCTGTGAAATCGACCTTGGATTAGTT

TCCCAGTGCTGCTGCGCAAAGCAAGTCTTTAAGATGAACAAGCAGATACTGGCAAACCTT

GCGCTGAAGATAAATGTCAAGGTCGGAGGAAGGAACACGGTGCTGGCTGATGCGGTGTCA

AGACGCATTCCCTTGGTGACTGACAGGCCTACCATCATATTCGGTGCCGATGTGACCCAT

CCTCATCCTGGAGAAGATAGCAGCCCTTCCATTGCTGCTGTTGTGGCTTCCCAAGACTGG

CCTGAGGTGACAAAGTATGCTGGTCTAGTTTCTGCTCAGTCTCATAGGCAAGAGCTGATA

GAGGATCTCTACAACGTCACACACGATCCTCAGAAAGGAACCGTTTGTGGTGGCATGGTC

AGGGAGCTTCTTATATCCTTCAAAAAATCAACTGGTCAAAAGCCTCAGAGGATACTATTC

TACAGGGATGGTGTGAGTGAAGGGCAGTTCTACCAAGTTCTACTGCATGAACTGGATGCT

ATCCGAAAGGCGTGTGCATCGCTGGAAGCAAACTACCAACCGCAGGTGACTTTCATCGTC

GTCCAGAAACGCCACCATACCAGGCTGTTCGCGCACAACCACAACGACCAGAATTCGGTC

GACAGGAGCGGCAACATACTTCCTGGAACTGTCGTGGACTCGAAGATCTGCCACCCTACA

GAGTTCGACTTCTTCCTGTGCAGCCATGCTGGCATCAAGGGCACCAGCCGTCCTGCTCAC

TACCATGTCTTGTGGGACGAGAACAACTTCACAGCCGACGCACTGCAGACCCTCACCAAC

AACCTTTGCTACACCTACGCGAGGTGCACGCGCTCTGTGTCCATTGTCCCGCCGGCGTAC

TACGCTCACCTGGCCGCATTCCGCGCCCGGTTCTACATGGAGCCTGACAGCTCAGACAGC

GGGTCGCTGGCGAGTGGCGCCCGTGGAGGCGGAGCGCCCTCCAGCTCGTCAACGTCCCGC

AGCACCCGCGCCACCGCCGGCGGAGCCGTTAGACCCCTCCCCGCGCTCAAGGACAGCGTC

AAGAAGGTCATGTTCTACTGCTGA

>Zea-mays_GRMZM2G419182_T03 cds:_protein_coding

ATGGAGTCTCACAATGGCGAGGCTGATGATTTGCCTCCACCACCCCCTTTGGCTGCTGGT

GTTGAACCACTTAAAGCCGATGAAACAAAGATGCCATTGAAACCTAGGAGTCTGGTCCAG

AGGAATGGCTTTGGCAGAAAGGGGCAGCAGATAAAGCTGATAACAAACCACTTCAAAGTT

TCTCTCATGAAGGCTGAAGATTTTTTCTATCATTACTATGTCAATTTGAAGTATGAAGAT

GATACACCGGTTGATCGGAAAGGGTCGGGAAGAAAAGTGATCGAAAAACTGCAGCAAACT

TATGCTGCTGAACTTGCAAATAAAGATTTTGCATATGATGGTGAGAAGAGCCTGTTTACA

ATTGGTGCCCTTCCTCAAGTTAAAATGGAATTCACTGTTGTGGATGAAGACGTTTCAACT

GGAAAGACTCCTGCAAATGGCAGTCCAGGCAATGATAGTCCTCCTGGAAGTGACAGGAAA

AGGGTCCGAAGGCCTTACAATACAAAGACATATAAGGTTGAACTATCTTTTGCGGCAAAA

ATCCCTATGAGTGCAATCTCACAGGCTTTGAGGGGTCAGGAGTCAGAGCACACTCAGGAA

GCAATTCGAGTGATTGACATTATTCTGAGGCAGCACTCAGCTAAGCAGGGTTGCCTATTA

GTAAGGCAATCGTTCTTCCACAATAATCCTTCCAATTTTGTTGACCTGGGTGGTGGTGTA

GTGGGATGTAGAGGGTTCCATTCTAGTTTTCGTGCAACCCAGAGTGGACTTTCACTCAAT

ATCGATGTGTCCACCACAATGATAGTGAAACCTGGTCCTGTCATTGACTTTCTGATTGCC

AATCAGAAAGTTAATGATCCAAGCATGATTGATTGGGCAAAGGCCAAGCGCTCACTGAAG

AACTTAAGGATAAAAACAAGTCCGGCGAACCAAGAACAGAAGATTGTTGGTCTCAGCGAC

AGGCCCTTGCCATTCACACTGAAACATAAAAATGGTGAATCTGAAGAGATCACTGTTTTT

GATTACTTTGTAAAGAACCGTGGCATAAAGCTGGAATATTCTGGTGATCTTCCTTGTATC

AATGTGGGAAAACCAAAGCGTCCAACTTATTTTCCAGTTGAGTTATGCAGTCTTCTTCCT

TTGCAACGGTACACTAAAGCATTGAGCACACTTCAAAGATCATCACTCGTTGAGAAATCT

AGGCAGAAACCACAAGAAAGGATGTCTGTTTTGTCTGATGTGCTGCAAAGAAGCAACTAT

GATGCAGAACCCATGCTGAAGGCTTGTGGGATTACAATTGCTAGAAATTTCATAGAAGTT

GATGGTAGGGTACTGCAGCCCCCTAAGCTTAAAGCTGGGAACGGAGAAGACATTTTTACG

CGCAATGGCAGATGGAATTTCAATAATAAGAAGCTCATTAGAGCTAGCAGTGTCGAGAAA

TGGGCAGTAGTAAACTTTTCTGCACGATGCAATGTCCGGGATCTTGTCCGTGATCTCATC

AAGTGTGGAGGCATGAAGGGCATTATGGTTGATGCACCATTTGCTGTATTTGATGAGAAT

CCTTCAATGAGACGGTCACCTGCTGTAAGAAGGGTTGAAGACATGTTTGAACAAGTGAAA

ACTAAGCTTCCTGGAGCACCCAAGTTTCTTTTCATTATGCCATTGACCTTTACAGGGCCT

TGGAAGAAGAAATGCCTTGCTGAATTTGGGATCGTTACACAGTGTGTGGCACCAACTAGA

GTCAATGATCAGTATCTTACAAATGTCTTGTTAAAGATTAACGCAAAGTTGGGTGGCTTG

AATTCGTTGCTCCAAATTGAAACATCCCCAGCAATTCCTCTCGTATCCAAGGTCCCAACT

ATAATCTTGGGTATGGACGTATCACACGGATCTCCTGGACATTCTGATATACCGTCTGTT

GCTGCTGTTGTTAGTTCTCGTGAATGGCCTCTTATCTCAAAATACAGAGCATCTGTCCGC

ACCCAATCACCAAAAATGGAAATGATTGACTCGTTGTTTAAGCCACGGGAAACTGATGAC

GATGGTCTGATTCGGGAGTGTCTGATTGACTTCTACACCAGTTCAGGGAAGAGAAAGCCC

GACCAAGTCATCATCTTCAGTTTGGCTTGCAAATTTCTTGATGAGAAATGGAATCCCAAG

TTCACATTGATCATTGCCCAGAAGAATCACCACACTAAATTTTTCATTCCTGGAAAGCCA

GATAATGTTCCAGCTGGCACTGTTGTTGACAACAAAGTCTGTCATCCAAGGAACTTCGAT

TTCTACATGTGTTCACATGCTGGAATGATTGGAACTACCAGGCCAACTCACTATCACATC

CTGCATGACGAGATAGGCTTCAATCCTGATGACCTGCAGGAGCTGGTGCACTCGCTCTCT

TATGTGTAA

>Zea-mays_GRMZM2G441583_T01 cds:_protein_coding

ATGGTGAGGAAGAAAAGGACTGGCCCTGGAGAGAGTTCTGGGGAGACTTCTGGAGCGCCT
GGTCAGGGCTCCTCACAGCGTCCTCAGGCAACACAACAGGGTGCCCGAGGTGGAGGACAA
CACCAGGTGCGTGGTGGATATCCGGGCCATGGAGTGCCGCCTTCAGAGCACCCAGGTGGT
GGGCCGCCTGAGTATCAACCACGTGGCTATCAGGGACGTGGCGTTCCACCTTTACTTCCT
GGTGGTGGGCCGCCTGAGCCTCAACCGCGTGGCTACCAGGGACATGGTGGATACCAGGGC
CGTGGCGGGCCACCTTCACAGCATCCTGGTGGTGGGCCATCACCTGGGTCTCAGCCTCGT
GGCTACCAGGGACGTGGTGGTCTGCGTCCCAGAGGGGGAGTGCCGCAGCCATACCGTGGC
GGGCATGTGGGAGGTAGTGTTGGACCAATTGTTCCTTCAGGTCCGTCTAGACCAGTTCCC
GAGCTGCACCAAGCCCCAGATGTCCAACATCAAGCCCCTGTGGTGGCAGCACCATCACCA
CCGGGAGCTGGCTCGTCCTCGCAGCCTGGGATGGCCGAGGTGAGCACTGGACAAGTCCAG
CAACTTGTGATTCATGACCAAAGTTCAGCCAGCCAAGTTAGTCAGGTAGCACCAGCATCA
AGCAAAGCTGTTAGATTCCCATTGCGCCCTGGCAAGGGTACGCATGGGTCCAGGTGCATC
GTGAAGGCAAATCATTTCTTTGCTGAGCTGCCTGATAAAGACCTCCACCAATACGATGTA
TCAATAACACCTGTGGTTCCTTCACGTGGTGTCAATCGTGCTGTCATGAAAGAGCTTGTA
AACCTTCATAGACACTCCCATTTGGATGGGCGTCTGCCTGCATATGATGGAAGGAAGAGT
CTTTATACAGCTGGAGCATTGCCATTTACTTCGAAGACATTCGAAATTACTCTGCAAGAT
GAAGAAAACAGTCTTGGTGGAGGACAAAGACACCAAAGGGGCCAGAGGGTCTTTCAGGTG
GTGATCAAATTTGCTGCTCGCGCTGATCTCCACCATTTGGCTATGTTTCTAGCTGGGAGG
CAACCAGATGCTCCTCAAGAGGCTATTCAAGTACTTGACATTGTACTACGTGAATTTCCT
ACTGCCAGGTATTGTCCTGTTGGTAGATCATTTTATTCTCCCAATTTAGGGAGACGCCAG
CAACTTGGTGAAGGTTTGGAAACTTGGCGTGGTTTCTATCAAAGCATAAGGCCCACACAG
ATGGGTCTTTCTCTGAATATTGATATGTCCTCTACTGCATTTATTGAGCCCCTCCCTGTG
ATTGATTTTGTTGCTCAGCTTCTTGACAGAGATATCTCAGTTAGACCATTGTCTGATTCT
GATCGTGTGAAGATTAAAAAAGCCCTACGAGGTGTGAAAGTCGAGGTCACACACCGTGGA
AACATGCGTAGGAAGTATCGGATATCTGGCCTCACTTCACAAGCAACAAGGGAGTTATCA
TTCCCTATTGATGATCGTGGTACTGTTAAGACTGTGGTGCAATACTTCCTGGAGACTTAT
GGCTTTAATATTCAGCACACCACTTTACCTTGTTTGCAAGTGGGCAATCAGCAAAGAATA
AATTATCTGCCTATGGAGGTCTGTAAGATAGTTGAGGGACAGCGTTACTCAAAACGACTC
AATGAGAAACAGATCACTGCTCTACTGAAGGTGACTTGCCAGCGTCCCCAAGAGCGTGAG
AAAGCCATCTTGCAGACTGTGCATCACAATGCCTACTCCGAGGATCCTTATGCCCAGGAA
TTTGGTATAAAGATTGATGAGCGTCTTGCATCCGTTGAAGCTCGTGTTCTGCCTCCTCCA

AGGCTGAAATACCATGATAGTGGCAGAGAGAGGGATGTATTGCCAAGAGTTGGGCAGTGG

AATATGATGAATAAGAAAATGGTCAATGGTGGTAGAGTTAGCAGCTGGGCATGCATTAAC

TTCTCACGAAATGTGCAAGATGGTGCTGCTAGGAGTTTCTGTCATGATCTCGCTTTGATG

TGCCAAGTATCAGGAATGGATTTTGCACTTGAACCTGTGCTGCCCCCTGTCTATGCGAGG

CCTGAACATGTTGAAAGAGCACTAAAGAGACTTTATCAAGATGCCATGAGCATACTCAGG

CCTCAGGGCCGAGAGCTTGACTTGCTGATGGTAATACTGCCTGACAATAATGGCTCTCTT

TACGGGGATCTTAAAAGGATATGTGAGACTGATCTTGGATTGGTCTCCCAATGCTGTCTG

ACTAAACATGTTTTCAAGGCGAACAAGCATCAGTATCTTGCAAATGTTGCCCTGAAAATA

AATGTTAAGGTTGGGGGAAGGAATACTGTACTTGTTGATGCTTTGGCAAGGAGAATCCCC

CTTGTCAGTGACGTAGCTACTATTATCTTTGGTGCTGATGTGACCCATCCCCATCCTGGG

GAAGATTCTAGTCCTTCCATTGCAGCTGTGGTTGCTTCTCAAGACTGGCCTGAGGTTACG

AAGTATGCAGGATTAGTGAGTGCTCAAACCCATCGCCAAGAATTGATACAGGATCTTTTC

AACGTACGGCAAGATCCCCAAAGGGGGGCTGTCTCTGGTGGCATGATTAGGGAACTTCTC

ATTTCTTTCTGGAGGGCGACTGGACAAAAACCCAAGAGGATCATATTCTACAGGGATGGT

GTCAGTGAGGGACAGTTCTACCAAGTTCTGTTGTACGAACTTGATGCCATTAGAAAGGCC

TGTGCATCATTGGAGTCTGATTACCAGCCTCCAGTTACCTTTGTCGTGGTCCAGAAGCGT

CATCACACCAGGTTGTTTGTTAATAATCACAATGATCAGCGTGCTGCCGATAGAAGTGGC

AACATACTGCCGGGCACTGTGGTGGACTCGAAGATTTGCCATCCAACCGAGTTTGATTTC

TACCTGTGCAGCCATGCTGGCATTCAGGGAACAAGCCGTCCTGCCCATTATCATGTTCTG

TGGGATGAGAACAAATTTACGGCTGATGGGTTGCAAACTCTCACCAACAACTTGTGTTAC

ACGTATGCCAGGTGCACACGCTCAGTATCAATCGTTCCTCCTGCATACTATGCTCATCTG

GCAGCCTTCCGAGCTCGGTTTTACATGGAGCCAGATACAAGTGACAGCGGATCTGTGGCG

AGCGGTGCTACGACAAGCCGTGGCCCTCCACCAGGGGCGCGCAACACCAGGGCTGGTGCT

GCGAATGTTGCTGTGAGGCCATTACCTGCTCTCAAGGAAAACGTGAAGCGCGTCATGTTC

TACTGCTAA
>Lactuca-sativa_CL344
CTGCTCATTGTCATTCTGCCAGACAACAATGGATCCCTCTATGGTGACCTGAAACGAATCTGTGAGACTG
ATCTTGGCGTTGTTTCTCAGTGCTGTCTGACTAAACATGTTTTTAAGATGAGCAAACAGTACCTTGCAAAC
GTGGCTTTGAAGATTAACGTGAAGGTTGGAGGAAGGAACACGGTCCTTCTGGATGCAATTTCAAGGCGG
ATACCTAATGTCAGCGATGTACCAACTATCATCTTTGGTGCTGATGTCACCCATCCACACCCCGGGGAAG
ATTCAAGCCCCTCTATTGCAGCTGTTGTTGCCTCTCAAGACTGGCCTGAGATAACAAAGTATGCTGGGTTG
GTTTGTGCACAAGCCCACCGTCAAGAACTGATCCAGGATCTGTACAAGGAGTGGCATGACCCACAAAGA
GGCAAAATGTCTGGTGGCATGGTCAAGGAACTGCTGATATCTTTTCGCAGAGCAACTGGGCAGAAGCCA
AAGCGGATTATTTTTTACAGGTATTTTGGATATAGGGATGGAGTGAGCGAGGGGCAGTTCTATCAAGTTT
TACTTTATGAGCTTGATGCGATTCGAAAGGCATGTGCATCGTTGGAGCCAGATTATCAGCCTCCAGTGAC
ATTTGTGGTGGTTCAAAAACGTCATCACACGCGATTGTTTGCTAACAACCATCGTGACCGCAATGCTACC
GACAGGAGTGGGAATATACTGCCAGGCACCGTTGTCGACTCCAAGATATGTCATCCAACCGAGTTTGATT
TCTATCTATGCAGCCATGCTGGGATTCAAGGGACAAGCCGTCCGGCCCATTACCATGTGCTGCGGGATGA

GAACAAGTTCGCAGCTGATGCTTTGCAATCACTCACAAACAATCTCTGTTACACGTATGCAAGGTGCACT
CGTTCTGTGTCCATTGTGCCACCTGCATACTATGCTCATCTGGCTGCCTTCCGTGCACGGTTCTATATGGA
G
>Pinus-radiata_Contig2618
CTTCTTGTTGTTATTTTGCCTGATAACAATGGTTCTTTGTATGGTGACCTGAAGCGTATATGTGAGACTGA
CCTTGGTTTGGTTTCCCAGTGCTGTCTGACGAAACATGTCTTTAAAATGAGCAAACAATATCTGGCAAAT
GTTGCCCTTAAAATTAATGTGAAGGTTGGTGGAAGAAACACTGTCTTAGTTGACGCACTGTCAAGGAGGA
TACCTTTGGTCAGCGACAAACCTACAATAATCTTTGGAGCAGATGTTACCCATCCACACCCTGGAGAAGA
TTCGAGCCCGTCAATAGCTGCTGTTGTAGCGTCTCAAGATTGGCCGGAAGTCACTAAATATGCAGGCTTA
GTTTGTGCTCAGGCACATCGTCAGGAACTCATTCAGGATCTTTACAAAGTCTGGCAGGATCCTGTGCGAG
GGTCTGCAACTGGAGGCATGATAAAGGAATTGTTAATTTCCTTCAGAAGGAATACAGGACACAAGCCGG
AGCGTATAATATTTTACAGAGATGGAGTAAGCGAGGGTCAGTTTTATCAAGTTCTATTGTATGAGTTAGA
TGGCTATTCGAAA
>Citrus-sinensis_CL450
CTCCTAGTTCGCCAATCGTTCTTTCACAATGATCCAAAAAATTTTGCTGATGTCGGAGGGGGAGTTCTTGG
CTGCAGAGGATTCCATTCAAGTTTTAGGACCACTCAGGGAGGCTTGTCTTTGAATATTGATGTATCAACTA
CCATGATAATTCAGCCGGGTCCAGTAGTTGATTTTCTTATTGCCAACCAAAATGTTAGAGATCCCTTTTCA
ATTGACTGGGCCAAGGCTAAACGGACACTAAAAAATCTGAGGATTAAGACGATTACCTCCAATCAAGAG
TACAAGATAACTGGACTGAGTGAGAAACTGTGTAAAGAGCAAATGTTTTCACTGAAGCAGAAGAATGTT
AAGGATGATGATGGTGAAGTGCAGGAACTGGAAATTACTGTTTACGACTATTTTGTTAATAATCGCAATA
TAGATTTGCGATATTCTGGAGATCTACCATGCATCAATGTTGGTAAACCAAAGCGGCCGACCTATATTCC
TCTTGAGCTTTGTGAATTGGTGTCCTTACAACGTTACACGAAAGCCCTGACCAATCTACAGAGAGCATCA
CTAGTGGAGAAGTCAAGGCAGAAGTTCCTTCTTTGTTTGCTTCCTGAGAGGAAAAACTCTGATTTATACG
GTCCTTGGAAACGAAAGAATCTTGCCGACTTTGGAATTGTCACTCAGTGTATGGCTCCTATGAGGGTCAA
TGACCAGTATCTTACAAATGTTCTCCTTAAGATTAATGCTAAGCTTGGTGGATTAAATTCATTGTTGGCTG
TTGAACATTCCCCTTCAATTCCTATTGTTTCGAAGGTTCCCACCATCATCCTTGGGATGGATGTATCCCAT
GGGTCTCCTGGACATTCAGATATACCATCTATTGCTGCGGTAGTCAGTTCCAGGCACTGGCCCTTAATATC
TCGCTACAGAGCAGCTGTGCGTACACAGTCTCCAAAAGTCGAAATGATTGATTCTTTGTTCAAGAAAGTA
TCTGACACTGAGGATGAAGGGATAATAAGAGAGCTTTTGTTGGACTTCTACACTAGCTCAGGGAAAAGG
AAACCTGAACAGATCATCATATTCAGGGATGGTGTCAGTGAATCACAGTTTAATCAAGTCTTGAACGTTG
AACTGAATCAGATTATAGAGGCGTGCAAGTTTCTGGATGAGAAGTGGTCCCCAAAGTTTGCGGTGATTGT
TGCACAGAAGAATCACCATACAAAATTTTTCCAGTCGGGATCTCCTGACAATGTTCCTCCTGGAACTGTT
GTTGACAACAAAGTCTGTCATCCAAGAAACTATGACTTCTACCTTTGTGCCCATGCTGGAATGATTGGTA
CTTCAAGGCCAACACATTACCACGTTCTATTTGATGAAATTGGCTTTTCAAGTGATGAGCTGCAGGAACT
AGTGCACTCACTTTCTTATGTGTACCAGAGAAGCACTACAGCCATTTCTGTAGTTGCTCCAATTTGCTATG
CCCACTTGGCAGCAAGCCAGGTAGGATCATTCATGAAG
>Citrus-sinensis_CL2621
TGTCCTGTGGGTCGTTCATTCTATTCTCCTGATCTGGGGAGAAGGCAACCACTGGGGGAGGGATTGGAAA
GTTGGCGTGGTTTCTACCAAAGTATTCGTCCAACTCAGATGGGCCTATCCCTAAATATTGATATGTCCTCT
ACTGCCTTTATTGAGCCTTTGCCAGTGATTGATTTCGTACAGCAGTTGCTGAACCGAGATGTTTCCTCAAG
ACCATTATCTGATGCTGATCGTGTTAAGATCAAGAAGGCTCTCAGAGGAGTTAGGGTTGAAGTTACGCAT
CGAGGAAATATGCGTAGGAAGTATCGTATATCTGGCTTGACATCGCAAACAACAGGAGAGCTCACTTTTC
CGGTTGATGAAAGTGGTACCCTGAAATCTGTTGTTGAATACTTCTACGAAACCTATGGTTTTGTTATTCAA
CATACTCAGTGGCCCTGCCTACAAGTGGGAAATCAGCAGAGACCAAATTATTTGCCTATGGAGGTTTGCA
AGATTGTTGAGGGTCAGAGGTACTCCAAGAGATTAAATGAGAGGCAGATTACTGCTTTGCTGAAGGTGA
CCTGTCAACGTCTGCTTATTGTCATTCTCCCTGATAATAATGGCTCTCTTTATGGTGATCTTAAACGGATTT
GTGAGACAGACCTTGGGCTTGTCTCCCACTGCTGTTTGACTAAGCATGTTTCAAAATGAGCAAGCAATA
CATGGCCGATGTGGCTTTGAAGATAAATGTGAAGGTTGGAGGGAGGAATACTGTGCTTGTTGATGCATAT
TCAGGCGTATACTCTATT
>Citrus-sinensis_CL5705
ACAGTTGTGGGAAGGTCATTTTTCTCAACTGATCTTGGACCAGTGGGTCAGCTTGGTGATGGCGTTGAAT
ATTGGCGTGGATATTTTCAGAGTCTTCGCCCAACCCAGATGGGGCTATCTCTTAATATTGATGTTTCAGCC
CGCTCCTTTTATGAGCCAGTTACTGAGTTTGTTCAGTATTATTGCAGAGACTTATCACGTCCTCTTTCTGAT
CAAGTGCGTCTGAAGGTGAAAAAGGCATTGAAGGGAATAAAGGTAGTGCTCACGCATATGGAGTATAAC
AAAAGTTACAAGATCACTGGAATATCCAGTGAACCGATGAGCCGACTAATGTTTACTGATGACAATGCG
ACAAGGTTGTCAGTGGTTCAATATTTTCGTCAAAGATATAATATTGGGCTTCAATTCACGTCATTGCCTGC
TCTTGTAGCTGGAAGTGAAGCGAGGCCTATTTATTTGCCAATGGAGCTTTCTAGGATTGCCGCGGGACAG
AGATATACTAAAAGATTAAATGAAGGACAGTAACTGCTCTTTTAGAGCACCTGTCAGCGTCC
>Pinus-taeda_contig1654
AGGGGCATGGATGTATCACATGGTTCTCCGGGGCATGCAGATTCACCTTCAATTTCAGCTGTTGTTGCCTC
TCGGGAATGGCCCTTGATATCCAGGTATAGAGCTTCAGTGAGAACACAGTCACCTAAGGTTGAGATGATT
GAGGCTCTGCACAAACAATCGCCCACAGGAAAGGATGTCGGAATGATCAAAGAGCTACTTCTAGATTTCT

ACCAAACATGCAATCCTCCACCAAACGTAGAAAGAAAGCCGCAACAAATGATCATTTTCAGAGATGGAG
TCAGTGAATCGCAATTTGACCAGGTCTTGAATGTTGAGTTGCAGGCTATACTCAAGGCATGTAATGACAT
AGAGGATGGTTATAGGCCCAAAGTTACATTGATTGTTGCGCAGAAGAATCATCACACGAAGCTGTTCCCA
ACTGGTCAAGGCAATGTGCAACCAGGGACTATTGTAGATGCTCAGATTTGTCATCCTAGAAACTTTGATT
TCTACTTGTGCCCTCAGGCTGGCCCAATAGGAACTTCACGGCCTACTCATTACCATGTATTACTTGATGAG
AATAGTTTTACTGTGGATGATCTTCAGATTTTGGTCCATGCATTATCTTATGTG
>Pinus-taeda_contig11958
CTTCTTGTTGTTATTTTGCCTGATAACAATGGTTCTTTGTATGGCGACTTGAAGCGTATATGTGAGACTGA
CCTTGGTTTGGTTTCCCAGTGCTGTCTGACGAAACATGTCTTTAAAATGAGCAAACAATATCTTGCAAATG
TTGCTCTTAAGATTAATGTAAAGGTTGGTGGAAGAAACACTGTCTTAGTTGACGCACTGTCAAGGAGGAT
ACCTTTGGTCAGTGACAAACCTACAATAATCTTTGGAGCAGATGTTACCCATCCACACCCTGGAGAGGAT
TCTAGCCCATCAATAGCTGCTGTTGTAGCATCTCAAGATTGGCCTGAAGTCACTAAATATGCAGGCTTAG
TTTGTGCTCAGGCACATCGTCAGGAACTCATTCAGGATCTTTACAAAGTCTGGCAAGATCCTGTGCGAGG
GTCTGCAACTGGAGGCATGATAAAGGAATTGTTGATTTCCTTCAGAAGGAATACAGGACACAAGCCGGA
GCGTATAATATTTTACAGAGATGGAGTAAGCGAGGGCCAGTTTTATCAAGTTCTGTTGTATGAGTTAGAT
GCTATTCGAAAGGCCTGTGCATCTCTAGAACCAAATTATCAACCTCCAGTTACTTTTGTGGTGGTTCAAAA
ACGTCACCATACTAGACTCTTTGCAAACAACCATCAAGATCGAAATGCGACAGACAGGAGTGGAAACAT
ATTGCCAGGCACTGTAGTGGATTCCAAAATTTGCCATCCAACAGAATTTGACTTTTACCTCTGCAGCCATG
CTGGTATTCAGGGAACTAGTAGGCCTGCCCATTATCATGTTCTTTGGGATGAAAACAAATTCACAGCTGA
TGGATTGCAGTCCTTAACCAACAATCTCTGTTACACATATGCGAGGTGCACACGGTCAGTTTCTATAGTAC
CCCCAGCGTATTATGCCCATCTGGCTGCATTTCGGGCTCGGTTTTATATGGAG
>Pinus-taeda_contig12732
AAGGGGTTGAAAATTAAAGTTACCCACAGACTGTGTTCTCAGAAATTCACAATAGTAGGACTGACAAAA
CAGGTGACTACGGAAATCAAATTTCTCATGACCGAGGACAATGGCACTCAGCGAATGGTTACTATGGTGG
AATTTTTCAGGATAAAGCATGGATGTAAAATTGCTTTCCAGCTGCTGCCTTGCCTGGACATCAGTAAAAG
CAAAGAAAAACCGAATTATGTTCCCATGGAGTTTTGTATGATCTGTGAAGGCCAAAGGTTTCCTCGAGAT
AATTTGAACGGTTACCAGTCAAAGAATTTTAGTCGAATTGCCTGCCAAATTTTAATAATTGCTATGGAGG
AGAAACATCCAGGGTATAATACTCTGAAGCTTATTGCAGAAACAGATGTCGGATTGGTGACGCAGTGTTG
TTTGTTTGATCATGTCAAAAAATGCTCAGATCGGGCAACACAATCTCAGGCATCACAATATCTGGCTAAT
TTGGCATTGAAGATAAATGCCAAAGTGGGGGGAAGTAACGCGGCGCTTCTTCATAGCTTGTCTCGCCAGT
TACCGAGATTTGGCAGCGACCATGTCATGTACATTGGCGCAGATGTAAATCATCCTGGATCGGGGGATAG
TACGAGTCCTTCCATTGCTGCAGTGGTTGGAAGCATCAACTGGCCCTGGTGCAATCGCTATAATGCGAGA
GTCAGCTATCAGAGTCACAGGGTGGAACACATCCAACAACTCGAGGAGATGTCTAAGGAATTACTGGAC
GATTATTTCAAAGCAAACAAGAAGCTGCCTGATAGGATACTCTTCTTCAGAGATGGTGTGAGTGAGAGTC
AATTCGATATGGTTCTCAACCAAGAATTAGAAGCCCTCAGAGAAGCAGTAGCCCACTTCAAAAACTACA
ATCCTCCTGTTTCCTTCATAGTAGCTCAGAAGAGACATCACACCAGACTGTTCCTTGGTGATGGAGAGCA
ACGGACGAAGTCTGGCAATGTTCCTCCTGGCACTGTGGTGGACACAGTCATTGTTCATCCACGACAGTTT
GATTTCTATCTGTGCAGCCATAATGGGCTTCTGGGCACTAGCAAGCCCACTCATTACCATGTTCTCTGGGA
TGATAACCGCTTCAGTTCTGATGAGCTGGAAACCTTGATTAACAATCTTTGTTATACATTGCAAGATGCA
CAAAGCCAGTGTCATTGGCCCCCCCTGTGTATTATGCAGATCTTGCGGCTTATCGGGGTCGATTATACTTG
CAG
>Pinus-taeda_contig9005
ACAGACCTTGGTATAGTTTCTCAATGCTGTTTAGTAAAGCATGTATTGAAGAGGAGCAAACAATACATGG
CGAATGTAGCCTTAAAGATAAATGTTAAGGCCGGAGGCAGGAACACTGTACTTGTTGATGCTCTTAGAAG
GAGAATATCTCTGGTGAGTGATGTACCAACAATAATATTTGGTGCAGATGTAACTCATCCTCATCCTGGA
GAAGACACTAGCCCATCCATAGCAGCCGTTGTGGCTTCTCAAGATTGGCCAGAAGTCACAAAATATGTTG
GTCTAGTTTCTGCTCAGGCACATCGTCAAGAGATGATTGAAGACCTTTTTAAGGTTGTAAAAGATCCTAA
AAGAGGTGATGTCGTTCGAAGCATGATAAGAGATCTTTTGCGTTCCTTCTACACTGCAACAAGTCAGAAA
CCTATGAGGATCATCTTCTACAGAGATGGTGTAAGTGAGGGGCAGTTTTACCAAGTCCTGCTGGATGAGT
TAGAGGCCATACGCAGGGCTTGCATGTCAATTGACAAGGATTATCAGCCTCCTGTGACTTTTATAGTTGTT
CAAAAACGTCATCACACACGTTTATTTGCTCATAATCATCGTGACAGGAAGAGTGTTGATAAAAGAAGTG
GTAACATATTGCCAGGTACCGTTGTTGATTCCACAATTTGTCATCCAACAGAGTTTGATTTCTACCTTTGC
AGTCATGCTGGTATTCAGGGAACAAGCAGGCCAGCTCATTATCATGTTCTGTGGGATGATAATAGATTCA
GTGCAGATGCTTTGCAGTCTTTGACAAATAATCTCTGTTACACATATGCTCGGTGTACTCGCTCCGTTTCA
ATCGTTCCTCCAGCATATTATGCGCATCTGGCTGCATTTCGAGCTCGCTTCTACATAGAG
>Aquilegia-vulgaris_AGO1901
TTCCTTCTCTGTATTTTGCCAGAGCGGAAAAATTGTGATATATATGGTCCATGGAAGAAGAAAAATCTCC
ATGAAATGGGAATCTTCACACAGTGCCTCTCCCCAACGAAGATTAATGATCAGTATCTGACCAATGTACT
TCTAAAGATCAACTCCAAACTTGGAGGTATGAATTCCTTATTGGAGGTTGAGCGTATTCCAACTCTTCCGC
GTTTGAAGGAAACTCCTACCATGATCTTGGGGATGGATGTATCTCATGGGTCTCCTGGTCAATCAGATGTT
CCATCTATTGCTGCTGTTGTTGGTTCAAGATCTTGGCCGCTGATCTCAAGATACAGGGCATCTGTAAGAAC
CCAATCACCAAAGGTGGAGATGATTGATTCTTTGTTCAAACTTCTACCAAATGGGGAGGATGATGGCATT

ATAAGGGAATTACTGGTTGAGTTTTACAAGTCAAGTCAAGGACGCAAGCCTGAGCAGATCATAATCTTCA
GGGATGGAGTAAGCGAGTCTCAGTTTCCTCAAGTTCTGAACATTGAGCTGGAGCAAATCGTTAAGGCAGT
TGAGCATTTAGGTTATACCAGAATTCCGAAGATTACATTGATTGTGGCTCAAAAGAACCATCACACAAAG
CTTTTCCAATCCAACGACCCAAACAATGTTCCTCCTGGAACTGTGGTAGATACGCAGATTGTGCATCCTA
GGAACTATGACTTTTACATGTGTGCTCACAATGGAGCAATTGGGACATCCCGTCCAGCACACTACCATGT
CTTGCTTGATGAGATTGGCTTCTCTGCTGATGAATTGCAGAATCTTGTGCACTCGCTTTCCTATGTGTATC
AGCGCAGCACAACTGCTATTTCAATTGTGGCACCTATCTGCTATGCTCACCTTGCTGCTGCACAGATGGG
GCAGTTCATCAAG
>Aquilegia-vulgaris_AGO1902
CTTGGAGGCATTAACTCTTTATTATCGGTTGAGAAAACTCTTCCACTTCTTAAGGACATTCCAACCATGAT
CTTGGGGATGGACGTATCCCATGGTTCACCTGGTCAATCAGACATGCCATCCATTGCCGCTGTTGTTGGTT
CGAGATCTTGGCCACTAATATCAAGATACAGGGCATCAGTGAGGACACAGTCTCCGAAGGTTGAGATGA
TTGATTCCTTGTTCAAGCTTCTACCAAATGGAAAGGATGATGGCATTATAAAAGAACTACTGGTTGAATT
TTATGAGTCAAGTCGAGGACGCAAGCCTGAACAGATTATAATCTTTAGGGATGGAGTTGGCGAGTCTCAA
TTCAACCAAGTTCTCAACATTGAACTGGAGCAAATCATAAAGGCACTTGAGCTTTTAGGTGTCACTAAGC
TTCCAAAGTTTACATTGATTGTGGCTCAAAAGAACCACCACACGAAGCTTTTTCAAGCAAATGACCCAAA
CAATGTTCCTCCTGGAACTGTGGTGGATACTCGAATTGTGCATCCAAGGAATTATGACTTTTACATGTGCG
CACAGAATGGAATGATTGGAACCTCCCGCCCAGCTCATTACCATGTCCTGCTTGACGAGATTGGCTTTTCT
CCTGATGATTTGCAAAACCTTGTGCATTCTCTTTCCTATGTATATCAACGCAGCACGACTGCCATCTCAAT
CGTGGCACCTATCTGTTACGCTCACCTTGCTGCCGCACAGATTGGACAGTTCATAAAG
>Aquilegia-vulgaris_AGO1903
TTTCTTCTTTGTCTTATTCCAGAGAGGAATGGAACTATATATGGTCCATGGAAGAGGAAGTGTCTTGTGGA
GTTTGGCATCATCAATCAATGCCTTGCACCTATGAGAGTTAATGATCAACTTCTTGCAAATGTTCTCCTAA
AGATTAATGCAAAGCTTGGTGGTTTGAATTCTTTGTTAGCGGTGGAACACTCTCGTAACATTCCTCTGATT
TCCAATGTTCCCACTATGATACTTGGTATGGATGTATCACATGGTTCCCCAGGCCAGTCAGATATACCATC
TGTTGCTGCGGTTGTCAGCTCAAGGCAGTGGCCCTCGATTTCTCGCTATCGAGCATCTGTGAGAACTCAGT
CACCGAAGCTTGAAATGGTAGATTCTTTATTCAAGCCAGTATCAGACACTGTTGATGAAGGCCTAGTTCG
GGAGCTATTGATGGATTTCTATACTAGCTCAAATAAGAGGAAGCCTGATCAGATAGTCATTTTCAGGGAT
GGTGTAAGTGAGTCCCAGTTCAACCAGGTTTTGAACATTGAATTGGATCAAATCATTGAGGCTTGTAAGT
TCCTTGATGAGAAATGGCGTCCAAAGTTCACTCTGATTGTTGCACAAAAAAACCACCACACTAAGTTCTT
CCAGAAAGGGTCCCCTGACAATGTCCCACCTGGAACTGTGATTGATAGCCAGATTTGTCATCCTAGACAC
AATGATTTCTATATGTGTGCTCATGCTGGAATGATTGGGACAACTAGGCCTACACATTATCATGTCCTTTT
CGATGAAATTGGCTTTACAGCTGATGATCTTCAGGAGCTTGTCCATTCATTATCTTATGTATACCAACGAA
GCTCCACGGCCATTTCTACTGTTGCCCCTGTTTTCTATGCTCACCTTGCAGCTGCTCAGATAGGAAAGTTT
ATGAAG
>Aquilegia-vulgaris_AGO1904
CTTCTGATTGTTATATTACCTGATAACAACGGCCCTTTGTATGGTGAGCTGAAGCGAATATGTGAGACAG
ACCTAGGACTTGTATCACAGTGTTGTCTGACCAAACATGTGTTTAAGATGAATAAGCAGTATCTGGCAAA
CGTAGCCCTAAAGATAAATGTAAAGGTTGGGGGGCGAAACACAGTGCTCCTTGATGCGCTTTCTAGACGC
ATGCCTGTGGTCAGTGATGAACCAACAATCATCTTTGGTGCTGACGTAACCCATCCCCATCCTGGAGAGG
ATTCGAGCCCCTCTATCGCAGCTGTTGTTGCATCCCAAGATTGGCCTGAGGTCACAAAGTATGCTGGTTTG
GTTTGTGCTCAAGCTCATCGCCAGGAATTGATCCAAGATCTGTATAAAGTCTGGAACGATCCGCAAAAGG
GTACAATGACAGGAGGCATGATCAAGGATCTTTTGAGATCTTTTCATAAAGCTACTGGACGAAAACCTTC
ACGAATCATATTCTACAGGGATGGAGTCAGTGAAGGACAATTTTATCAAGTCTTGCTATATGAACTGGAT
GCGATTCGTAAGGCATGTGCTTCTCTGGAGCCAAATTATCAGCCCAGGGTGACATTTGTCGTTGTTCAAA
AGCGCCATCATACAAGGTTGTTTGCTAATAATCATGCTGACCGGCGCACAATTGATAGGAGCGGAAATAT
ATTACCAGGTACGGTGGTGGACTCCAAGATTTGCCATCCAACTGAATTTGACTTTTATTTGTGTAGCCATG
CTGGAATACAGGGCACAAGCCGTCCTGCCCATTATCATGTATTATGGGATGAGAACAACTTTACTGCCGA
TGGACTCCAGTCACTCACGAATAACCTCTGTTATACGTATGCAAGGTGCACCCGTTCTGTTTCTATAGTCC
CCCCAGCATACTACGCTCATTTGGCAGCTTTCCGTGCACGTTTTTACATGGAG
>Aquilegia-vulgaris_AGO1905
CTACTCTTAGTTATCTTACCTGACAGTAATGGTTCTCTATATGGTGATCTCAAGCGGATATGTGAAACAGA
TCTGGGTTTAATAACACAATGTTGTCTTACTAAGCATGTCTTCAAGATCAACAAACAGTACTTAGCTAATG
TGGCCTTGAAAATAAATGTTAAGATGGGAGGAAGGAACACTGTTCTTTTGGATGCTATCAGCTGCAGGAT
ACCATTGGTTAGTGACATACCAACAATAATATTTGGAGCAGATGTCACCCACCCGGAGAATGGAGAAGA
TTCAAGCCCTTCAATTGCGGCTGTAGTTGCTTCTCAGGACTGGCCTGAGGTCACAAAATATGCTGGTTTAG
TTTGTGCCCAAGCTCACAGACAGGAACTCATACAAGACTTGTTCAAGACCTGGCACGATCCTGTGCGCGG
CACTGTTAGTGGTGGGATGATAAGGGATCTTTTGATTTCATTTCGAAAGGCAACGGGTCAAAAACCGCAA
AGGATTATATTCTACAGGGATGGTGTAAGTGAAGGGCAATTTTATCAAGTCCTACTCTATGAGTTAGATG
CAATTAGGAAGGCTTGTGCTTCTTTAGAACCAAATTACCAACCACCAGTGACTTTTGTCATAGTACAGAA
AAGACATCATACTAGATTGTTTGCTAATAACCACAAGGACCGCAGCAGTATTGACAAGAGCGGGAATAT
TTTACCTGGAACTGTGGTTGACTCAAAAATATGTCATCCGACTGAGTTTGATTTTTATCTCTGCAGCCATG

CTGGGATACAGGGCACAAGCAGACCTGCTCATTACCATGTTTTATGGGACGAAAACCACTTTACAGCTGA
CGGAATTCAATCCCTGACAAACAATTTGTGTTACACGTATGCAAGGTGTACGCGCTCTGTCTCAGTTGTTC
CACCCGCTTATTATGCACACTTAGCAGCATTTCGAGCTAGATTTTACATGGAG
>Aquilegia-vulgaris_AGO1906
TTACTAATTGTTATTTTGCCAGACAATAATGGTTCTCTTTATGGTGATTTGAAACGAATATGTGAGACAGA
TCTTGGTCTTGTTTCACAGTGTTGTTTAACAAAGCATGTTTTTAGGATGAGTAAGCAGTATATGGCAAATG
TAGCTCTTAAGATTAATGTGAAGGTTGGTGGAAGGAACACAGTGCTTGTTGATGCGTTGTCAAGGCGCAT
ACCTTTGGTTAGTGATCGACCTACTATCATTTTTGGTGCTGATGTTACTCATCCTCATCCTGGAGAGGACT
CAAGCCCGTCAATTGCAGCTGTTGTCGCTTCTCAAGATTGGCCAGAAGTTACTAAATATGCTGGTTTGGTA
TGTGCTCAAGCCCATCGCCAAGAACTCATTCAAGATCTTTACAAAACCTGGCATGATCCTGTTAAAGGAA
CTATGCATGGGGGCATGGTGAAGGAACTTTTGATATCCTTCCGCAGAGCAACTGGACAGAAGCCTGAGC
GCATTATCTTTTACAGGGATGGGGTCAGCGAGGGACAGTTTTACCAGGTCTTGCTGTATGAGCTGGATGC
AATTAGAAAGGCATGTGCCTCCCTGGAGCCGAATTACCAGCCACCTGTTACTTTTGTTGTGGTTCAGAAG
CGTCACCACACACGACTGTTTGCGAACAACCATGCAGATCGTCGTGCAGTGGACAAAAGTGGCAACATTT
TGCCAGGAACGGTGGTAGACTCAAAGATCTGTCATCCGACAGAGTTTGACTTTTATCTTTGCAGTCACGC
TGGGATACAGGGCACTAGTCGTCCAGCCCATTATCATGTTCTCTGGGATGAGAACAAATTTACTGCCGAT
GGACTCCAGACTTTAACCAATAATCTCTGTTACACATATGCGCGATGCACCCGCTCAGTTTCCATTGTACC
TCCGGCATACTATGCCCACTTGGCTGCCTTTCGTGCACGTTTTTACATGGAG
>Aquilegia-vulgaris_AGO1907
CTGCTGATTGTTATTTTACCCGACAATAATGGTTCTCTTTATGGTGAAGTGAAGCGAATCTGTGAGACACA
ACTTGGGATAATCTCACAGTGCATCTTAGCTAGAACTGTTGAAAAAATGAATGTGCATACTTTGGCTAAC
ATTGTTCTAAAGATAAACACCAAGGTTGGGGGGATAAACGTTGTGCTTAGGGATCCTATACCAATGGTCA
GTGATAGGCCAACAATAATTTTTGGGGCTGATGTAACTCATCCTAATCCTGGGGAAAGTGGGAGCCCTTC
GATAGCAGCAGTTGTGGCTTCTCAAAACTGGCCAAATGTCACAAATTATATTCCTATACTGTCAGCACAA
CTTGGTCGTGAGGAAAAGATCCTAGATCTGGAAAGAATGGCCAAGGAACATTTTCACGCTTTTGAGAAG
AACAATCAGCGGAGGCCTGAAAGGATCATATTCTACAGGGATGGTGTTAGTGACGGACAATTTAATCAA
GTCCGCGAGTATGAGCTAGAAGCAATTCGGGAGGCATGGAGGAATGAATTCAAGGATTCTGTTGTGCCTC
CTATAACATTTGTGGTGGTCCAAAAACGACATCATACCAAGTTATTTCCCTGGAACCATGATGATTTCTCG
TCTGTAGACAGGAGCGGAAATATATATCCTGGAACTGTGGTGGATTCAGATATCTGCCACCCAACACAAT
TCAACTTCTACTTGTGTAGCCATGCTGGTATTCAGGCCACGAGCCGTCCTGCTCATTACCATGTTCTATGT
GATGATAATCAGTTCACGGCAGATCAACTTCAAACCCTCACCAATAACCTGTGTTACATATATGCAAGGT
GCACACGTTCGGTCTCCTATGTGTCACCAGCATATTATGCTCATCTGGCTGCTTTTCGTGCACGATTCTAC
TTTGAG
>Amborella trichopoda_AGO24601
CAAGTCGGGAATCATCAGCGCCCAAATTACCTCCCAATGGAGGTATGCCAAATCGTTGAGGGCCAACGAT
ACTCAAAGCGATTGAACGAAAGACAAATCACTGCCCTTCTGAAAGTGACCTGCCAGCGACTGCTGATTGT
AATCTTGCCTGATAATAATGGTTCCCTTTATGGGGATCTAAAGAGAATTTGCGAGACGGATTTGGGCTTG
GTCTCCCAATGCTGCTTAACTAAGCATGTATTTAAAATGAGCAAGCAGTATCTTGCTAATGTTGCTTTGAA
AATCAATGTTAAGGTCGGTGGGAGGAATACTGTACTTGTGGATGCGATTGCAAGGAGAATTCCACTTGTA
AGTGACGTTCCAACTATAATATTTGGGGCTGATGTCACTCATCCTCACCCAGGAGAGGATTCCAGCGCAT
CCATTGCTGCTGTCGTTGGTTCTCAGGACTGGCCAGAGGTGACTAAATATGCTGGATTGGTCTGTGCTCAA
GCTCACAGACAAGAATTGATCCAAGATCTTTATAAGGTCACCCAGGATCCTCAACGTGGAACTGTGACGG
GTGGCATGATCAAGGAGTTGCTCATTTCATTCAGGAGAGCAACTGGGCAAAAGCCTCAGAGGATTATTTT
CTACAGGGATGGAGTTAGCGAGGGCCAGTTTTACCAGGTTTTGTTTCATGAATTGGATGCTATTCGCAAG
GCATGTTCCTCCCTTGAGCCAAACTATCAGCCCCCTGTGACATTTGTTGTGGTTCAAAAGCGGCATCACAC
TCGGCTATTTGCAAACAATCATAAGGATCATCATCTGACTGACAAGAGTGGAAATATATTACCCGGAACT
GTGGTGGATTCAAAGATCTGCCATCCCACTGAATTTGATTTTTACTTATGCAGTCATGCTGGCATCCAGGG
TACTAGCCGCCCTGCCCATTACCATGTTTTGTTTGATGAGAATCACTTCACAGCCGATGGCCTCCAGTCGC
TCACTAATAATCTGTGCTATACGTATGCACGTTGCACCCGCTCTGTCTCTATTGTGCCGCCTGCTTACTAT
GCTCATCTAGCAGCCTTCAGGGCTCGCTTCTACATGGAG

Supplementary File 2. List of Pfam-derived domains of all AGO proteins used in this study.

SEQ ID	aln_s	aln_e	env_s	env_e	env_l	domain	type	h_s	h_e	h_l	score	E-value	sig	clan
Aguilegia_AGO1901	2	309	1	309	308	Piwi	Family	2	304	304	336.9	6.6e-101	1	CL0219
Aguilegia_AGO1902	1	254	1	254	253	Piwi	Family	58	304	304	284	8.4e-85	1	CL0219
Aguilegia_AGO1903	2	308	1	308	307	Piwi	Family	2	304	304	344.3	3.7e-103	1	CL0219
Aguilegia_AGO1904	2	321	1	322	321	Piwi	Family	2	303	304	384.6	2e-115	1	CL0219
Aguilegia_AGO1905	2	321	1	322	321	Piwi	Family	2	303	304	383	6.1e-115	1	CL0219
Aguilegia_AGO1906	2	321	1	322	321	Piwi	Family	2	303	304	380.2	4.2e-114	1	CL0219
Aguilegia_AGO1907	2	303	1	306	305	Piwi	Family	2	301	304	335.4	1.8e-100	1	CL0219
Amborella_AGO24601	1	42	1	43	42	PAZ	Family	91	133	134	33.7	1.6e-08	1	No_clan
Amborella_AGO24601	45	364	44	365	321	Piwi	Family	2	303	304	373.2	5.7e-112	1	CL0219
Arabidopsis-lyrata_16036850_locus	239	290	238	290	52	DUF1785	Domain	2	52	52	60.7	5.1e-17	1	No_clan
Arabidopsis-lyrata_16036850_locus	292	430	291	431	140	PAZ	Family	2	133	134	114.1	2.3e-33	1	No_clan
Arabidopsis-lyrata_16036850_locus	577	885	577	885	308	Piwi	Family	1	304	304	356	9.6e-107	1	CL0219
Arabidopsis-lyrata_16039685_locus	322	374	322	374	52	DUF1785	Domain	1	52	52	47.1	8.9e-13	1	No_clan
Arabidopsis-lyrata_16039685_locus	376	511	375	514	139	PAZ	Family	2	131	134	94.2	3.1e-27	1	No_clan
Arabidopsis-lyrata_16039685_locus	676	972	674	974	300	Piwi	Family	2	302	304	278.9	3e-83	1	CL0219
Arabidopsis-lyrata_16042271_locus	314	365	314	366	52	DUF1785	Domain	1	51	52	62.2	1.7e-17	1	No_clan
Arabidopsis-lyrata_16042271_locus	376	505	374	506	132	PAZ	Family	3	133	134	101.4	1.9e-29	1	No_clan
Arabidopsis-lyrata_16042271_locus	649	946	648	949	301	Piwi	Family	2	301	304	310.8	5.9e-93	1	CL0219
Arabidopsis-lyrata_16047041_locus	183	234	182	234	52	DUF1785	Domain	2	52	52	67.8	3e-19	1	No_clan
Arabidopsis-lyrata_16047041_locus	237	372	235	373	138	PAZ	Family	3	133	134	82.9	9.7e-24	1	No_clan
Arabidopsis-lyrata_16047041_locus	522	808	521	809	288	Piwi	Family	2	303	304	281.2	6e-84	1	CL0219
Arabidopsis-lyrata_16051341_locus	413	461	412	462	50	DUF1785	Domain	2	51	52	43	1.7e-11	1	No_clan
Arabidopsis-lyrata_16051341_locus	464	593	463	601	138	PAZ	Family	2	125	134	92.5	1.1e-26	1	No_clan
Arabidopsis-lyrata_16051341_locus	764	1059	762	1060	298	Piwi	Family	2	303	304	289.7	1.5e-86	1	CL0219
Arabidopsis-lyrata_16055324_locus	207	257	206	257	51	DUF1785	Domain	2	52	52	58.8	2e-16	1	No_clan
Arabidopsis-lyrata_16055324_locus	259	394	258	395	137	PAZ	Family	2	133	134	115.1	1.1e-33	1	No_clan
Arabidopsis-lyrata_16055324_locus	541	848	541	850	309	Piwi	Family	1	301	304	356.4	7.7e-107	1	CL0219
Arabidopsis-lyrata_16062541_locus	220	271	219	271	52	DUF1785	Domain	2	52	52	65.2	2e-18	1	No_clan
Arabidopsis-lyrata_16062541_locus	273	408	272	409	137	PAZ	Family	2	133	134	101.1	2.4e-29	1	No_clan
Arabidopsis-lyrata_16062541_locus	557	863	556	863	307	Piwi	Family	2	304	304	336.7	7.7e-101	1	CL0219
Arabidopsis-lyrata_16062911_locus	303	360	303	360	57	DUF1785	Domain	1	52	52	78.9	1e-22	1	No_clan
Arabidopsis-lyrata_16062911_locus	371	495	363	496	133	PAZ	Family	8	133	134	89.7	8.1e-26	1	No_clan
Arabidopsis-lyrata_16062911_locus	643	961	642	962	320	Piwi	Family	2	303	304	351.5	2.4e-105	1	CL0219
Arabidopsis-lyrata_16063851_locus	338	390	338	390	52	DUF1785	Domain	1	52	52	82.4	8.8e-24	1	No_clan
Arabidopsis-lyrata_16063851_locus	392	527	391	528	137	PAZ	Family	2	133	134	118	1.5e-34	1	No_clan
Arabidopsis-lyrata_16063851_locus	681	1000	680	1001	321	Piwi	Family	2	303	304	372.8	7.5e-112	1	CL0219
Arabidopsis-lyrata_16064878_locus	279	331	279	331	52	DUF1785	Domain	1	52	52	74.7	2.1e-21	1	No_clan
Arabidopsis-lyrata_16064878_locus	333	468	332	469	137	PAZ	Family	2	133	134	113.8	2.9e-33	1	No_clan
Arabidopsis-lyrata_16064878_locus	621	940	620	941	321	Piwi	Family	2	303	304	379.8	5.6e-114	1	CL0219
Arabidopsis-thaliana_AT1G31280.1	315	367	315	367	52	DUF1785	Domain	1	52	52	44.3	7.1e-12	1	No_clan
Arabidopsis-thaliana_AT1G31280.1	369	502	368	505	137	PAZ	Family	2	131	134	94.2	3.3e-27	1	No_clan
Arabidopsis-thaliana_AT1G31280.1	667	963	665	965	300	Piwi	Family	2	302	304	280.2	1.2e-83	1	CL0219
Arabidopsis-thaliana_AT1G31290.1	488	538	488	538	50	DUF1785	Domain	1	52	52	39.2	2.7e-10	1	No_clan
Arabidopsis-thaliana_AT1G31290.1	540	675	539	679	140	PAZ	Family	2	130	134	94.8	2.1e-27	1	No_clan
Arabidopsis-thaliana_AT1G31290.1	842	1144	841	1145	304	Piwi	Family	2	303	304	284.4	6.5e-85	1	CL0219
Arabidopsis-thaliana_AT1G48410.2	336	388	336	388	52	DUF1785	Domain	1	52	52	82.3	9.5e-24	1	No_clan
Arabidopsis-thaliana_AT1G48410.2	390	525	389	526	137	PAZ	Family	2	133	134	117.9	1.5e-34	1	No_clan
Arabidopsis-thaliana_AT1G48410.2	679	998	678	999	321	Piwi	Family	2	303	304	373.9	3.5e-112	1	CL0219
Arabidopsis-thaliana_AT1G69440.1	315	366	315	367	52	DUF1785	Domain	1	51	52	62.6	1.3e-17	1	No_clan
Arabidopsis-thaliana_AT1G69440.1	376	506	375	507	132	PAZ	Family	2	133	134	105.5	1.1e-30	1	No_clan
Arabidopsis-thaliana_AT1G69440.1	650	947	649	950	301	Piwi	Family	2	301	304	313.5	9e-94	1	CL0219
Arabidopsis-thaliana_AT2G27040.1	239	290	238	290	52	DUF1785	Domain	2	52	52	61.4	3.1e-17	1	No_clan
Arabidopsis-thaliana_AT2G27040.1	292	430	291	431	140	PAZ	Family	2	133	134	116.1	5.7e-34	1	No_clan
Arabidopsis-thaliana_AT2G27040.1	577	885	577	885	308	Piwi	Family	1	304	304	358.9	1.3e-107	1	CL0219
Arabidopsis-thaliana_AT2G27880.1	301	358	301	358	57	DUF1785	Domain	1	52	52	77.9	2.2e-22	1	No_clan
Arabidopsis-thaliana_AT2G27880.1	370	493	361	494	133	PAZ	Family	9	133	134	89	1.4e-25	1	No_clan
Arabidopsis-thaliana_AT2G27880.1	639	957	638	958	320	Piwi	Family	2	303	304	351.4	2.5e-105	1	CL0219
Arabidopsis-thaliana_AT2G32940.1	207	257	206	257	51	DUF1785	Domain	2	52	52	57.9	3.9e-16	1	No_clan
Arabidopsis-thaliana_AT2G32940.1	259	393	258	395	137	PAZ	Family	2	132	134	108.8	9.9e-32	1	No_clan
Arabidopsis-thaliana_AT2G32940.1	541	851	541	851	310	Piwi	Family	1	304	304	361.3	2.3e-108	1	CL0219
Arabidopsis-thaliana_AT5G21030.1	194	245	193	245	52	DUF1785	Domain	2	52	52	64.9	2.5e-18	1	No_clan
Arabidopsis-thaliana_AT5G21030.1	247	383	246	384	138	PAZ	Family	2	133	134	89	1.3e-25	1	No_clan

Carica-papaya_.TU.supercontig_26.59	294	429	293	430	137	PAZ	Family	2	133	134	108.9	9e-32	1	No_clan
Carica-papaya_.TU.supercontig_26.59	577	884	576	884	308	Piwi	Family	2	304	304	346.9	6e-104	1	CL0219
Carica-papaya_.TU.supercontig_44.130	286	338	286	338	52	DUF1785	Domain	1	52	52	74.1	3.5e-21	1	No_clan
Carica-papaya_.TU.supercontig_44.130	340	475	339	476	137	PAZ	Family	2	133	134	119.9	3.7e-35	1	No_clan
Carica-papaya_.TU.supercontig_44.130	628	947	627	948	321	Piwi	Family	2	303	304	377.9	2.1e-113	1	CL0219
Carica-papaya_.TU.supercontig_47.31	241	290	240	290	50	DUF1785	Domain	2	52	52	67.2	4.9e-19	1	No_clan
Carica-papaya_.TU.supercontig_47.31	294	426	292	427	135	PAZ	Family	4	133	134	74.6	3.6e-21	1	No_clan
Carica-papaya_.TU.supercontig_47.31	582	715	581	717	136	Piwi	Family	2	136	304	105.6	1.7e-30	1	CL0219
Carica-papaya_.TU.supercontig_47.31	721	851	717	852	135	Piwi	Family	178	303	304	167.4	2.7e-49	1	CL0219
Carica-papaya_.TU.supercontig_75.90	235	287	235	287	52	DUF1785	Domain	1	52	52	75.2	1.5e-21	1	No_clan
Carica-papaya_.TU.supercontig_75.90	289	392	288	428	140	PAZ	Family	2	101	134	77.8	3.7e-22	1	No_clan
Carica-papaya_.TU.supercontig_75.90	583	881	582	882	300	Piwi	Family	2	303	304	323.3	8.8e-97	1	CL0219
Chlamydomonas-reinhardtii_Au9.Cre01.g071850	312	340	312	341	29	DUF1785	Domain	1	29	52	18.1	0.0011	0	No_clan
Chlamydomonas-reinhardtii_Au9.Cre01.g071850	356	433	343	441	98	PAZ	Family	48	126	134	47.2	1.1e-12	1	No_clan
Chlamydomonas-reinhardtii_Au9.Cre01.g071850	611	927	610	928	318	Piwi	Family	2	303	304	297.6	6e-89	1	CL0219
Chlamydomonas-reinhardtii_Au9.Cre04.g214250	330	431	314	439	125	PAZ	Family	25	126	134	53.4	1.3e-14	1	No_clan
Chlamydomonas-reinhardtii_Au9.Cre04.g214250	613	929	612	930	318	Piwi	Family	2	303	304	280.8	7.9e-84	1	CL0219
Cucumis-sativus_Cucsa.082260	227	279	227	279	52	DUF1785	Domain	1	52	52	75.7	1.1e-21	1	No_clan
Cucumis-sativus_Cucsa.082260	281	416	280	417	137	PAZ	Family	2	133	134	116.2	5.2e-34	1	No_clan
Cucumis-sativus_Cucsa.082260	569	887	568	889	321	Piwi	Family	2	302	304	369.6	7e-111	1	CL0219
Cucumis-sativus_Cucsa.112480	344	395	343	395	52	DUF1785	Domain	2	52	52	78.3	1.6e-22	1	No_clan
Cucumis-sativus_Cucsa.112480	397	532	396	533	137	PAZ	Family	2	133	134	122.4	6.4e-36	1	No_clan
Cucumis-sativus_Cucsa.112480	687	1006	686	1007	321	Piwi	Family	2	303	304	375.7	1e-112	1	CL0219
Cucumis-sativus_Cucsa.152920	233	284	232	284	52	DUF1785	Domain	2	52	52	67.9	3e-19	1	No_clan
Cucumis-sativus_Cucsa.152920	286	421	285	422	137	PAZ	Family	2	133	134	101.9	1.3e-29	1	No_clan
Cucumis-sativus_Cucsa.152920	569	876	568	876	308	Piwi	Family	2	304	304	345.9	1.2e-103	1	CL0219
Cucumis-sativus_Cucsa.185140	220	271	219	271	52	DUF1785	Domain	2	52	52	61.5	2.8e-17	1	No_clan
Cucumis-sativus_Cucsa.185140	273	410	272	411	139	PAZ	Family	2	133	134	103.1	5.7e-30	1	No_clan
Cucumis-sativus_Cucsa.185140	557	865	557	865	308	Piwi	Family	1	304	304	331.8	2.4e-99	1	CL0219
Cucumis-sativus_Cucsa.200260	152	204	152	204	52	DUF1785	Domain	1	52	52	78.4	1.5e-22	1	No_clan
Cucumis-sativus_Cucsa.200260	222	340	209	341	132	PAZ	Family	15	133	134	97.8	2.6e-28	1	No_clan
Cucumis-sativus_Cucsa.200260	495	810	494	811	317	Piwi	Family	2	303	304	354.2	3.5e-106	1	CL0219
Cucumis-sativus_Cucsa.254700	281	333	281	333	52	DUF1785	Domain	1	52	52	74.9	1.9e-21	1	No_clan
Cucumis-sativus_Cucsa.254700	335	470	334	471	137	PAZ	Family	2	133	134	119.8	4.1e-35	1	No_clan
Cucumis-sativus_Cucsa.254700	623	942	622	943	321	Piwi	Family	2	303	304	378.8	1.1e-113	1	CL0219
Cucumis-sativus_Cucsa.284770	322	374	322	374	52	DUF1785	Domain	1	52	52	69.5	9.4e-20	1	No_clan
Cucumis-sativus_Cucsa.284770	384	513	382	514	132	PAZ	Family	3	133	134	98.4	1.6e-28	1	No_clan
Cucumis-sativus_Cucsa.284770	671	977	670	980	310	Piwi	Family	2	301	304	302	2.7e-90	1	CL0219
Glycine-max_Glyma02g00510	267	319	267	319	52	DUF1785	Domain	1	52	52	74.1	3.5e-21	1	No_clan
Glycine-max_Glyma02g00510	321	456	320	457	137	PAZ	Family	2	133	134	115	1.2e-33	1	No_clan
Glycine-max_Glyma02g00510	609	928	608	929	321	Piwi	Family	2	303	304	378.7	1.2e-113	1	CL0219
Glycine-max_Glyma02g12430	64	116	64	116	52	DUF1785	Domain	1	52	52	73.4	5.7e-21	1	No_clan
Glycine-max_Glyma02g12430	125	254	124	256	132	PAZ	Family	2	132	134	99.8	5.9e-29	1	No_clan
Glycine-max_Glyma02g12430	413	720	411	723	312	Piwi	Family	2	301	304	299.7	1.4e-89	1	CL0219
Glycine-max_Glyma02g44260	225	276	224	276	52	DUF1785	Domain	2	52	52	68.6	1.7e-19	1	No_clan
Glycine-max_Glyma02g44260	278	413	277	414	137	PAZ	Family	2	133	134	97.2	3.8e-28	1	No_clan
Glycine-max_Glyma02g44260	561	868	560	868	308	Piwi	Family	2	304	304	350.4	5e-105	1	CL0219
Glycine-max_Glyma06g23920	211	263	211	263	52	DUF1785	Domain	1	52	52	69.3	1.1e-19	1	No_clan
Glycine-max_Glyma06g23920	265	400	264	401	137	PAZ	Family	2	133	134	119.8	4.1e-35	1	No_clan
Glycine-max_Glyma06g23920	553	872	552	873	321	Piwi	Family	2	303	304	363.5	5.1e-109	1	CL0219
Glycine-max_Glyma06g47230	192	243	191	243	52	DUF1785	Domain	2	52	52	65.7	1.4e-18	1	No_clan
Glycine-max_Glyma06g47230	245	379	244	380	136	PAZ	Family	2	133	134	87.1	4.9e-25	1	No_clan
Glycine-max_Glyma06g47230	530	840	529	840	311	Piwi	Family	2	304	304	351.7	2e-105	1	CL0219
Glycine-max_Glyma09g29720	352	404	352	404	52	DUF1785	Domain	1	52	52	83.4	4.3e-24	1	No_clan
Glycine-max_Glyma09g29720	406	541	405	542	137	PAZ	Family	2	133	134	124.1	1.9e-36	1	No_clan
Glycine-max_Glyma09g29720	694	1013	693	1014	321	Piwi	Family	2	303	304	376.5	5.8e-113	1	CL0219
Glycine-max_Glyma10g38770	266	318	266	318	52	DUF1785	Domain	1	52	52	74.1	3.5e-21	1	No_clan
Glycine-max_Glyma10g38770	320	455	319	456	137	PAZ	Family	2	133	134	118.8	8.3e-35	1	No_clan
Glycine-max_Glyma10g38770	608	927	607	928	321	Piwi	Family	2	303	304	376.8	4.5e-113	1	CL0219
Glycine-max_Glyma12g08860	226	278	226	278	52	DUF1785	Domain	1	52	52	78	2.1e-22	1	No_clan
Glycine-max_Glyma12g08860	280	413	279	414	135	PAZ	Family	2	133	134	102.4	9.3e-30	1	No_clan
Glycine-max_Glyma12g08860	567	885	565	886	321	Piwi	Family	2	303	304	352.4	1.2e-105	1	CL0219
Glycine-max_Glyma13g26240	232	283	231	283	52	DUF1785	Domain	2	52	52	58.5	2.4e-16	1	No_clan

Arabidopsis-thaliana_AT5G21030.1	529	706	528	708	180	Piwi	Family	2	177	304	125.9	1.2e-36	1	CL0219
Arabidopsis-thaliana_AT5G21030.1	707	810	705	811	106	Piwi	Family	197	303	304	124.9	2.4e-36	1	CL0219
Arabidopsis-thaliana_AT5G21150.1	214	265	213	265	52	DUF1785	Domain	2	52	52	65.2	2e-18	1	No_clan
Arabidopsis-thaliana_AT5G21150.1	267	402	266	403	137	PAZ	Family	2	133	134	100.6	3.5e-29	1	No_clan
Arabidopsis-thaliana_AT5G21150.1	551	857	550	857	307	Piwi	Family	2	304	304	331.8	2.4e-99	1	CL0219
Arabidopsis-thaliana_AT5G43810.1	284	336	284	336	52	DUF1785	Domain	1	52	52	75.3	1.5e-21	1	No_clan
Arabidopsis-thaliana_AT5G43810.1	338	473	337	474	137	PAZ	Family	2	133	134	113.8	2.9e-33	1	No_clan
Arabidopsis-thaliana_AT5G43810.1	626	945	625	946	321	Piwi	Family	2	303	304	380.5	3.4e-114	1	CL0219
Brachypodium-distachyon_Bradi1g16060	345	396	345	397	52	DUF1785	Domain	1	51	52	64.7	2.9e-18	1	No_clan
Brachypodium-distachyon_Bradi1g16060	408	536	405	537	132	PAZ	Family	4	133	134	99.1	1e-28	1	No_clan
Brachypodium-distachyon_Bradi1g16060	694	997	693	1001	308	Piwi	Family	2	300	304	310.8	5.6e-93	1	CL0219
Brachypodium-distachyon_Bradi1g28260	366	418	366	418	52	DUF1785	Domain	1	52	52	77.2	3.5e-22	1	No_clan
Brachypodium-distachyon_Bradi1g28260	420	552	419	553	134	PAZ	Family	2	133	134	109.2	7.7e-32	1	No_clan
Brachypodium-distachyon_Bradi1g28260	746	1034	740	1037	297	Piwi	Family	15	300	304	340.5	5.2e-102	1	CL0219
Brachypodium-distachyon_Bradi1g29580	332	382	330	382	52	DUF1785	Domain	3	52	52	76.9	4.5e-22	1	No_clan
Brachypodium-distachyon_Bradi1g29580	384	519	383	520	137	PAZ	Family	2	133	134	112.7	6.2e-33	1	No_clan
Brachypodium-distachyon_Bradi1g29580	614	933	613	934	321	Piwi	Family	2	303	304	359.2	1.1e-107	1	CL0219
Brachypodium-distachyon_Bradi1g36910	182	234	182	234	52	DUF1785	Domain	1	52	52	75	1.7e-21	1	No_clan
Brachypodium-distachyon_Bradi1g36910	236	371	235	372	137	PAZ	Family	2	133	134	120.4	2.5e-35	1	No_clan
Brachypodium-distachyon_Bradi1g36910	524	843	523	844	321	Piwi	Family	2	303	304	375.8	9.5e-113	1	CL0219
Brachypodium-distachyon_Bradi2g10370	239	290	238	290	52	DUF1785	Domain	2	52	52	71	3e-20	1	No_clan
Brachypodium-distachyon_Bradi2g10370	292	429	291	430	139	PAZ	Family	2	133	134	106.7	4.5e-31	1	No_clan
Brachypodium-distachyon_Bradi2g10370	577	883	576	883	307	Piwi	Family	2	304	304	346.4	8.3e-104	1	CL0219
Brachypodium-distachyon_Bradi2g14150	231	282	230	282	52	DUF1785	Domain	2	52	52	67.9	2.9e-19	1	No_clan
Brachypodium-distachyon_Bradi2g14150	284	419	283	420	137	PAZ	Family	2	133	134	102.2	1.1e-29	1	No_clan
Brachypodium-distachyon_Bradi2g14150	593	886	582	886	304	Piwi	Family	16	304	304	323.5	7.7e-97	1	CL0219
Brachypodium-distachyon_Bradi3g51080	339	390	338	390	52	DUF1785	Domain	2	52	52	80.1	4.6e-23	1	No_clan
Brachypodium-distachyon_Bradi3g51080	392	527	391	528	137	PAZ	Family	2	133	134	120.2	2.9e-35	1	No_clan
Brachypodium-distachyon_Bradi3g51080	646	966	645	967	322	Piwi	Family	2	303	304	377.1	3.7e-113	1	CL0219
Brachypodium-distachyon_Bradi4g08590	207	258	206	258	52	DUF1785	Domain	2	52	52	61.3	3.4e-17	1	No_clan
Brachypodium-distachyon_Bradi4g08590	281	408	260	409	149	PAZ	Family	9	133	134	92.9	7.9e-27	1	No_clan
Brachypodium-distachyon_Bradi4g08590	520	828	519	828	309	Piwi	Family	2	304	304	336.6	8e-101	1	CL0219
Brachypodium-distachyon_Bradi5g18540	382	433	381	433	52	DUF1785	Domain	2	52	52	80.6	3.3e-23	1	No_clan
Brachypodium-distachyon_Bradi5g18540	435	570	434	571	137	PAZ	Family	2	133	134	116.2	5e-34	1	No_clan
Brachypodium-distachyon_Bradi5g18540	725	1044	724	1045	321	Piwi	Family	2	303	304	379.5	6.8e-114	1	CL0219
Brachypodium-distachyon_Bradi5g21800	374	406	365	406	41	DUF1785	Domain	20	52	52	24.1	1.4e-05	1	No_clan
Brachypodium-distachyon_Bradi5g21800	413	537	408	542	134	PAZ	Family	3	128	134	88.4	2e-25	1	No_clan
Brachypodium-distachyon_Bradi5g21800	693	982	686	983	297	Piwi	Family	9	303	304	301.4	4.1e-90	1	CL0219
Brachypodium-distachyon_Bradi5g21810	219	275	218	277	59	DUF1785	Domain	2	49	52	17.9	0.0012	0	No_clan
Brachypodium-distachyon_Bradi5g21810	303	377	292	382	90	PAZ	Family	16	80	134	31.9	5.9e-08	1	No_clan
Brachypodium-distachyon_Bradi5g21810	568	865	565	866	301	Piwi	Family	3	303	304	292.7	1.9e-87	1	CL0219
Citrus-sinensis_CL2621	1	53	1	53	52	DUF1785	Domain	1	52	52	86	6.3e-25	1	No_clan
Citrus-sinensis_CL2621	55	190	54	191	137	PAZ	Family	2	133	134	125.1	9.1e-37	1	No_clan
Citrus-sinensis_CL2621	193	257	192	263	71	Piwi	Family	2	68	304	52	3.8e-14	1	CL0219
Lactuca-sativa_CL344	2	326	1	327	326	Piwi	Family	2	303	304	371.6	1.7e-111	1	CL0219
Citrus-sinensis_CL450	2	53	1	53	52	DUF1785	Domain	2	52	52	70.1	6.2e-20	1	No_clan
Citrus-sinensis_CL450	55	194	54	195	141	PAZ	Family	2	133	134	104.2	2.7e-30	1	No_clan
Citrus-sinensis_CL450	197	504	196	504	308	Piwi	Family	2	304	304	349.9	7.3e-105	1	CL0219
Citrus-sinensis_CL5705	1	53	1	53	52	DUF1785	Domain	1	52	52	83.7	3.4e-24	1	No_clan
Citrus-sinensis_CL5705	67	179	52	184	132	PAZ	Family	15	128	134	96.5	6.1e-28	1	No_clan
Pinus-taeda_contig11958	2	321	1	322	321	Piwi	Family	2	303	304	383.9	3.2e-115	1	CL0219
Pinus-taeda_contig12732	1	103	1	110	109	PAZ	Family	30	127	134	67.7	4.9e-19	1	No_clan
Pinus-taeda_contig12732	110	418	109	421	312	Piwi	Family	2	301	304	312.5	1.8e-93	1	CL0219
Pinus-taeda_contig1654	2	204	1	205	204	Piwi	Family	84	274	304	195.5	7.5e-58	1	CL0219
Pinus-radiata_Contig2618	2	187	1	191	190	Piwi	Family	2	174	304	182.7	5.9e-54	1	CL0219
Pinus-taeda_contig9005	1	300	1	301	300	Piwi	Family	23	303	304	356.6	6.3e-107	1	CL0219
Carica-papaya_.TU.supercontig_1.68	181	233	181	233	52	DUF1785	Domain	1	52	52	71.3	2.5e-20	1	No_clan
Carica-papaya_.TU.supercontig_1.68	242	372	241	373	132	PAZ	Family	2	133	134	101.2	2.3e-29	1	No_clan
Carica-papaya_.TU.supercontig_1.68	530	836	528	839	311	Piwi	Family	2	301	304	305.8	2e-91	1	CL0219
Carica-papaya_.TU.supercontig_135.40	313	364	313	365	52	DUF1785	Domain	1	51	52	48.1	4.3e-13	1	No_clan
Carica-papaya_.TU.supercontig_135.40	367	494	366	504	138	PAZ	Family	2	123	134	91.5	2.2e-26	1	No_clan
Carica-papaya_.TU.supercontig_135.40	665	961	665	965	300	Piwi	Family	1	300	304	305.4	2.5e-91	1	CL0219
Carica-papaya_.TU.supercontig_26.59	241	292	240	292	52	DUF1785	Domain	2	52	52	67.1	5.2e-19	1	No_clan

Glycine-max_Glyma13g26240	285	423	284	424	140	PAZ	Family	2	133	134	96.3	7.5e-28	1	No_clan
Glycine-max_Glyma13g26240	570	876	569	877	308	Piwi	Family	2	303	304	318.1	3.4e-95	1	CL0219
Glycine-max_Glyma14g04510	225	276	224	276	52	DUF1785	Domain	2	52	52	68.7	1.7e-19	1	No_clan
Glycine-max_Glyma14g04510	278	413	277	414	137	PAZ	Family	2	133	134	96.1	8.5e-28	1	No_clan
Glycine-max_Glyma14g04510	561	868	560	868	308	Piwi	Family	2	304	304	350.6	4.3e-105	1	CL0219
Glycine-max_Glyma15g13260	252	304	252	305	53	DUF1785	Domain	1	51	52	48.3	3.9e-13	1	No_clan
Glycine-max_Glyma15g13260	308	441	306	444	138	PAZ	Family	3	131	134	89.6	8.4e-26	1	No_clan
Glycine-max_Glyma15g13260	605	899	603	903	300	Piwi	Family	2	300	304	277.8	6.5e-83	1	CL0219
Glycine-max_Glyma16g34300	339	391	339	391	52	DUF1785	Domain	1	52	52	83.4	4.2e-24	1	No_clan
Glycine-max_Glyma16g34300	393	528	392	529	137	PAZ	Family	2	133	134	122.8	4.6e-36	1	No_clan
Glycine-max_Glyma16g34300	681	1000	680	1001	321	Piwi	Family	2	303	304	375.8	9.5e-113	1	CL0219
Glycine-max_Glyma17g12850	205	257	205	257	52	DUF1785	Domain	1	52	52	67.1	5.2e-19	1	No_clan
Glycine-max_Glyma17g12850	259	394	258	395	137	PAZ	Family	2	133	134	115.5	8.8e-34	1	No_clan
Glycine-max_Glyma17g12850	547	866	546	867	321	Piwi	Family	2	303	304	365.1	1.7e-109	1	CL0219
Glycine-max_Glyma20g02820	279	331	279	332	53	DUF1785	Domain	1	51	52	41.9	3.9e-11	1	No_clan
Glycine-max_Glyma20g02820	341	462	334	470	136	PAZ	Family	8	125	134	84.8	2.6e-24	1	No_clan
Glycine-max_Glyma20g02820	634	931	632	932	300	Piwi	Family	2	303	304	288.1	4.8e-86	1	CL0219
Glycine-max_Glyma20g12070	231	282	230	282	52	DUF1785	Domain	2	52	52	66.9	6e-19	1	No_clan
Glycine-max_Glyma20g12070	284	421	283	422	139	PAZ	Family	2	133	134	98.7	1.3e-28	1	No_clan
Glycine-max_Glyma20g12070	569	876	568	876	308	Piwi	Family	2	304	304	288.8	2.9e-86	1	CL0219
Glycine-max_Glyma20g28970	220	272	220	272	52	DUF1785	Domain	1	52	52	74.1	3.3e-21	1	No_clan
Glycine-max_Glyma20g28970	274	409	273	410	137	PAZ	Family	2	133	134	118.8	8e-35	1	No_clan
Glycine-max_Glyma20g28970	562	881	561	882	321	Piwi	Family	2	303	304	378.4	1.5e-113	1	CL0219
Zea-mays_GRMZM2G007791_T01	348	396	346	396	50	DUF1785	Domain	3	52	52	48.6	3.1e-13	1	No_clan
Zea-mays_GRMZM2G007791_T01	403	529	399	533	134	PAZ	Family	4	129	134	89.3	1e-25	1	No_clan
Zea-mays_GRMZM2G007791_T01	702	999	699	1000	301	Piwi	Family	4	303	304	298.6	2.9e-89	1	CL0219
Zea-mays_GRMZM2G031147_T01	348	396	346	396	50	DUF1785	Domain	3	52	52	48.6	3.1e-13	1	No_clan
Zea-mays_GRMZM2G031147_T01	403	529	399	533	134	PAZ	Family	4	129	134	89.3	1e-25	1	No_clan
Zea-mays_GRMZM2G031147_T01	702	999	699	1000	301	Piwi	Family	4	303	304	298.6	2.9e-89	1	CL0219
Zea-mays_GRMZM2G039455_T01	366	417	365	417	52	DUF1785	Domain	2	52	52	81.1	2.1e-23	1	No_clan
Zea-mays_GRMZM2G039455_T01	419	554	418	555	137	PAZ	Family	2	133	134	116.8	3.4e-34	1	No_clan
Zea-mays_GRMZM2G039455_T01	709	1028	708	1029	321	Piwi	Family	2	303	304	376.6	5.2e-113	1	CL0219
Zea-mays_GRMZM2G059033_T01	332	382	331	382	51	DUF1785	Domain	2	52	52	75.6	1.1e-21	1	No_clan
Zea-mays_GRMZM2G059033_T01	385	518	383	519	136	PAZ	Family	3	133	134	93.6	5.1e-27	1	No_clan
Zea-mays_GRMZM2G059033_T01	664	967	663	969	306	Piwi	Family	2	302	304	337.8	3.4e-101	1	CL0219
Zea-mays_GRMZM2G077801_T01	201	252	200	252	52	DUF1785	Domain	2	52	52	61.1	3.9e-17	1	No_clan
Zea-mays_GRMZM2G077801_T01	254	388	253	389	136	PAZ	Family	2	133	134	107	3.6e-31	1	No_clan
Zea-mays_GRMZM2G077801_T01	536	644	535	654	119	Piwi	Family	2	113	304	83.6	8.6e-24	1	CL0219
Zea-mays_GRMZM2G079080_T02	267	319	267	319	52	DUF1785	Domain	1	52	52	78	2e-22	1	No_clan
Zea-mays_GRMZM2G079080_T02	321	456	320	457	137	PAZ	Family	2	133	134	124.5	1.4e-36	1	No_clan
Zea-mays_GRMZM2G079080_T02	609	928	608	929	321	Piwi	Family	2	303	304	377	4.1e-113	1	CL0219
Zea-mays_GRMZM2G105250_T01	362	416	362	416	54	DUF1785	Domain	1	52	52	64.2	4.1e-18	1	No_clan
Zea-mays_GRMZM2G105250_T01	418	553	417	554	137	PAZ	Family	2	133	134	106.2	6.2e-31	1	No_clan
Zea-mays_GRMZM2G105250_T01	705	1008	704	1010	306	Piwi	Family	2	302	304	327.5	4.8e-98	1	CL0219
Zea-mays_GRMZM2G108281_T03	267	319	267	319	52	DUF1785	Domain	1	52	52	74.9	1.9e-21	1	No_clan
Zea-mays_GRMZM2G108281_T03	321	456	320	457	137	PAZ	Family	2	133	134	125.1	8.9e-37	1	No_clan
Zea-mays_GRMZM2G108281_T03	609	928	608	929	321	Piwi	Family	2	303	304	377.5	2.9e-113	1	CL0219
Zea-mays_GRMZM2G141818_T02	228	279	227	279	52	DUF1785	Domain	2	52	52	67.1	5.1e-19	1	No_clan
Zea-mays_GRMZM2G141818_T02	281	417	280	418	138	PAZ	Family	2	133	134	102.6	8.4e-30	1	No_clan
Zea-mays_GRMZM2G141818_T02	565	869	564	870	306	Piwi	Family	2	303	304	330.1	7.5e-99	1	CL0219
Zea-mays_GRMZM2G153859_T04	220	271	219	271	52	DUF1785	Domain	2	52	52	73.5	5e-21	1	No_clan
Zea-mays_GRMZM2G153859_T04	273	408	272	409	137	PAZ	Family	2	133	134	100.7	3.2e-29	1	No_clan
Zea-mays_GRMZM2G153859_T04	556	862	555	862	307	Piwi	Family	2	304	304	352.1	1.5e-105	1	CL0219
Zea-mays_GRMZM2G165242_T02	220	271	219	271	52	DUF1785	Domain	2	52	52	73.6	4.7e-21	1	No_clan
Zea-mays_GRMZM2G165242_T02	273	405	272	406	134	PAZ	Family	2	133	134	104.6	1.9e-30	1	No_clan
Zea-mays_GRMZM2G165242_T02	553	840	552	852	300	Piwi	Family	2	285	304	322.9	1.2e-96	1	CL0219
Zea-mays_GRMZM2G317927_T01	385	437	385	437	52	DUF1785	Domain	1	52	52	80.8	2.8e-23	1	No_clan
Zea-mays_GRMZM2G317927_T01	439	574	438	575	137	PAZ	Family	2	133	134	116.1	5.7e-34	1	No_clan
Zea-mays_GRMZM2G317927_T01	729	1049	728	1050	322	Piwi	Family	2	303	304	369.7	6.8e-111	1	CL0219
Zea-mays_GRMZM2G347402_T01	201	252	200	252	52	DUF1785	Domain	2	52	52	61.1	3.9e-17	1	No_clan
Zea-mays_GRMZM2G347402_T01	254	388	253	389	136	PAZ	Family	2	133	134	107	3.6e-31	1	No_clan
Zea-mays_GRMZM2G347402_T01	536	644	535	654	119	Piwi	Family	2	113	304	83.6	8.6e-24	1	CL0219
Zea-mays_GRMZM2G354867_T01	149	197	147	197	50	DUF1785	Domain	3	52	52	45.9	2.2e-12	1	No_clan

Zea-mays_GRMZM2G354867_T01	149	197	147	197	50	DUF1785	Domain	3	52	52	45.9	2.2e-12	1	No_clan
Zea-mays_GRMZM2G354867_T01	210	326	198	334	136	PAZ	Family	10	125	134	88.2	2.3e-25	1	No_clan
Zea-mays_GRMZM2G354867_T01	500	797	497	798	301	Piwi	Family	3	303	304	296.9	9.9e-89	1	CL0219
Zea-mays_GRMZM2G359875_T01	372	423	371	423	52	DUF1785	Domain	2	52	52	80.6	3.2e-23	1	No_clan
Zea-mays_GRMZM2G359875_T01	425	560	424	561	137	PAZ	Family	2	133	134	116.8	3.5e-34	1	No_clan
Zea-mays_GRMZM2G359875_T01	715	1034	714	1035	321	Piwi	Family	2	303	304	379	9.8e-114	1	CL0219
Zea-mays_GRMZM2G361518_T01	308	359	307	359	52	DUF1785	Domain	2	52	52	79	1e-22	1	No_clan
Zea-mays_GRMZM2G361518_T01	361	497	360	498	138	PAZ	Family	2	133	134	115.6	7.8e-34	1	No_clan
Zea-mays_GRMZM2G361518_T01	654	973	653	974	321	Piwi	Family	2	303	304	363.9	3.9e-109	1	CL0219
Zea-mays_GRMZM2G366277_T02	171	219	169	219	50	DUF1785	Domain	3	52	52	48.9	2.5e-13	1	No_clan
Zea-mays_GRMZM2G366277_T02	226	352	222	357	135	PAZ	Family	4	129	134	89.9	7e-26	1	No_clan
Zea-mays_GRMZM2G366277_T02	525	822	522	823	301	Piwi	Family	4	303	304	299.2	2e-89	1	CL0219
Zea-mays_GRMZM2G411082_T01	308	359	307	359	52	DUF1785	Domain	2	52	52	79	1e-22	1	No_clan
Zea-mays_GRMZM2G411082_T01	361	497	360	498	138	PAZ	Family	2	133	134	115.6	7.8e-34	1	No_clan
Zea-mays_GRMZM2G411082_T01	654	973	653	974	321	Piwi	Family	2	303	304	363.9	3.9e-109	1	CL0219
Zea-mays_GRMZM2G419182_T03	220	271	219	271	52	DUF1785	Domain	2	52	52	73.8	4.3e-21	1	No_clan
Zea-mays_GRMZM2G419182_T03	273	402	272	403	131	PAZ	Family	2	133	134	95.2	1.6e-27	1	No_clan
Zea-mays_GRMZM2G419182_T03	559	706	549	708	159	Piwi	Family	15	156	304	112.8	1.2e-32	1	CL0219
Zea-mays_GRMZM2G419182_T03	709	801	707	802	95	Piwi	Family	179	274	304	120.3	5.9e-35	1	CL0219
Zea-mays_GRMZM2G441583_T01	385	437	385	437	52	DUF1785	Domain	1	52	52	80.8	2.8e-23	1	No_clan
Zea-mays_GRMZM2G441583_T01	439	574	438	575	137	PAZ	Family	2	133	134	116.1	5.7e-34	1	No_clan
Zea-mays_GRMZM2G441583_T01	729	1049	728	1050	322	Piwi	Family	2	303	304	369.7	6.8e-111	1	CL0219
Oryza-sativa_Os01g16850	36	77	24	77	53	DUF1785	Domain	13	52	52	46.2	1.7e-12	1	No_clan
Oryza-sativa_Os01g16850	1704	1818	1695	1820	125	PAZ	Family	25	132	134	88.3	2.2e-25	1	No_clan
Oryza-sativa_Os01g16850	1922	2199	1916	2200	284	Piwi	Family	15	303	304	284.9	4.6e-85	1	CL0219
Oryza-sativa_Os01g16870	221	272	220	272	52	DUF1785	Domain	2	52	52	73.4	5.5e-21	1	No_clan
Oryza-sativa_Os01g16870	274	410	273	411	138	PAZ	Family	2	133	134	109.5	6.2e-32	1	No_clan
Oryza-sativa_Os01g16870	558	865	557	865	308	Piwi	Family	2	304	304	342.7	1.1e-102	1	CL0219
Oryza-sativa_Os02g07310	195	244	192	244	52	DUF1785	Domain	4	52	52	53.7	8.1e-15	1	No_clan
Oryza-sativa_Os02g07310	307	360	306	361	55	PAZ	Family	79	133	134	29.7	2.7e-07	1	No_clan
Oryza-sativa_Os02g07310	515	834	514	834	320	Piwi	Family	2	304	304	291.8	3.5e-87	1	CL0219
Oryza-sativa_Os02g45070	367	418	366	418	52	DUF1785	Domain	2	52	52	79	9.8e-23	1	No_clan
Oryza-sativa_Os02g45070	420	555	419	556	137	PAZ	Family	2	133	134	120.6	2.3e-35	1	No_clan
Oryza-sativa_Os02g45070	710	1029	709	1030	321	Piwi	Family	2	303	304	373.3	5.5e-112	1	CL0219
Oryza-sativa_Os02g58490	299	350	298	350	52	DUF1785	Domain	2	52	52	79.8	5.5e-23	1	No_clan
Oryza-sativa_Os02g58490	352	484	351	485	134	PAZ	Family	2	133	134	107.2	3.1e-31	1	No_clan
Oryza-sativa_Os02g58490	639	958	638	959	321	Piwi	Family	2	303	304	368.4	1.7e-110	1	CL0219
Oryza-sativa_Os03g33650	361	412	361	413	52	DUF1785	Domain	1	51	52	59.7	1e-16	1	No_clan
Oryza-sativa_Os03g33650	424	550	421	553	132	PAZ	Family	4	131	134	99.9	5.8e-29	1	No_clan
Oryza-sativa_Os03g33650	710	1014	709	1017	308	Piwi	Family	2	301	304	320	9.4e-96	1	CL0219
Oryza-sativa_Os03g47820	350	402	350	402	52	DUF1785	Domain	1	52	52	79.8	5.6e-23	1	No_clan
Oryza-sativa_Os03g47820	403	537	403	538	135	PAZ	Family	1	133	134	112.2	9e-33	1	No_clan
Oryza-sativa_Os03g47820	704	1020	702	1021	319	Piwi	Family	2	303	304	339.3	1.2e-101	1	CL0219
Oryza-sativa_Os03g47830	309	382	304	385	81	PAZ	Family	56	131	134	77	6.7e-22	1	No_clan
Oryza-sativa_Os03g47830	542	847	541	848	307	Piwi	Family	2	303	304	302.7	1.7e-90	1	CL0219
Oryza-sativa_Os03g57560	7	49	3	49	46	DUF1785	Domain	11	52	52	64.2	4.1e-18	1	No_clan
Oryza-sativa_Os03g57560	51	203	50	204	154	PAZ	Family	2	133	134	95.5	1.3e-27	1	No_clan
Oryza-sativa_Os03g57560	342	637	339	638	299	Piwi	Family	3	303	304	278.8	3.1e-83	1	CL0219
Oryza-sativa_Os03g58600	353	405	353	405	52	DUF1785	Domain	1	52	52	80.3	3.9e-23	1	No_clan
Oryza-sativa_Os03g58600	407	542	406	543	137	PAZ	Family	2	133	134	109	8.9e-32	1	No_clan
Oryza-sativa_Os03g58600	697	1015	696	1016	320	Piwi	Family	2	303	304	344.1	4e-103	1	CL0219
Oryza-sativa_Os04g06770	228	279	227	279	52	DUF1785	Domain	2	52	52	69.9	6.8e-20	1	No_clan
Oryza-sativa_Os04g06770	281	418	280	419	139	PAZ	Family	2	133	134	108.3	1.5e-31	1	No_clan
Oryza-sativa_Os04g06770	566	872	565	872	307	Piwi	Family	2	304	304	342.5	1.3e-102	1	CL0219
Oryza-sativa_Os04g47870	387	438	386	438	52	DUF1785	Domain	2	52	52	81.1	2.2e-23	1	No_clan
Oryza-sativa_Os04g47870	440	575	439	576	137	PAZ	Family	2	133	134	118	1.4e-34	1	No_clan
Oryza-sativa_Os04g47870	730	1049	729	1050	321	Piwi	Family	2	303	304	379.5	6.9e-114	1	CL0219
Oryza-sativa_Os04g52540	340	389	339	389	50	DUF1785	Domain	2	52	52	44.1	8.1e-12	1	No_clan
Oryza-sativa_Os04g52540	397	525	391	527	136	PAZ	Family	5	132	134	100.3	4.3e-29	1	No_clan
Oryza-sativa_Os04g52540	690	988	687	989	302	Piwi	Family	3	303	304	301.4	4.1e-90	1	CL0219
Oryza-sativa_Os04g52550	358	409	358	409	51	DUF1785	Domain	1	52	52	42.9	1.8e-11	1	No_clan
Oryza-sativa_Os04g52550	411	535	410	543	133	PAZ	Family	2	125	134	97.8	2.6e-28	1	No_clan
Oryza-sativa_Os04g52550	729	1022	720	1023	303	Piwi	Family	11	303	304	295	3.8e-88	1	CL0219

Oryza-sativa_Os06g39640	273	325	273	325	52	DUF1785	Domain	1	52	52	74.9	1.9e-21	1	No_clan
Oryza-sativa_Os06g39640	327	462	326	463	137	PAZ	Family	2	133	134	125.3	7.9e-37	1	No_clan
Oryza-sativa_Os06g39640	615	934	614	935	321	Piwi	Family	2	303	304	375.4	1.3e-112	1	CL0219
Oryza-sativa_Os06g51310	327	378	326	378	52	DUF1785	Domain	2	52	52	77.8	2.3e-22	1	No_clan
Oryza-sativa_Os06g51310	380	515	379	516	137	PAZ	Family	2	133	134	117.9	1.6e-34	1	No_clan
Oryza-sativa_Os06g51310	671	989	669	990	321	Piwi	Family	3	303	304	364.1	3.4e-109	1	CL0219
Oryza-sativa_Os07g09020	342	392	341	392	51	DUF1785	Domain	2	52	52	77	4.2e-22	1	No_clan
Oryza-sativa_Os07g09020	422	533	404	533	129	PAZ	Family	24	134	134	103.8	3.5e-30	1	No_clan
Oryza-sativa_Os07g09020	678	1006	677	1009	332	Piwi	Family	2	301	304	315.2	2.6e-94	1	CL0219
Oryza-sativa_Os07g28850	406	457	406	457	51	DUF1785	Domain	1	52	52	67.1	5.3e-19	1	No_clan
Oryza-sativa_Os07g28850	489	593	475	597	122	PAZ	Family	25	130	134	99.8	5.9e-29	1	No_clan
Oryza-sativa_Os07g28850	749	1054	747	1056	309	Piwi	Family	2	302	304	347.6	3.7e-104	1	CL0219
Manihot-esculenta_cassava1330.valid.m1	291	343	291	343	52	DUF1785	Domain	1	52	52	70.8	3.5e-20	1	No_clan
Manihot-esculenta_cassava1330.valid.m1	352	482	351	483	132	PAZ	Family	2	133	134	98.6	1.4e-28	1	No_clan
Manihot-esculenta_cassava1330.valid.m1	642	949	640	952	312	Piwi	Family	2	301	304	304.8	3.9e-91	1	CL0219
Manihot-esculenta_cassava13333.valid.m1	280	328	277	329	52	DUF1785	Domain	4	51	52	40.2	1.3e-10	1	No_clan
Manihot-esculenta_cassava13333.valid.m1	332	457	330	467	137	PAZ	Family	3	122	134	90.1	5.9e-26	1	No_clan
Manihot-esculenta_cassava13333.valid.m1	621	901	601	904	303	Piwi	Family	13	300	304	279	2.8e-83	1	CL0219
Manihot-esculenta_cassava13489.valid.m1	292	344	292	344	52	DUF1785	Domain	1	52	52	74.4	2.6e-21	1	No_clan
Manihot-esculenta_cassava13489.valid.m1	348	478	346	479	133	PAZ	Family	4	133	134	88.7	1.6e-25	1	No_clan
Manihot-esculenta_cassava13489.valid.m1	634	949	632	950	318	Piwi	Family	2	303	304	336.8	7.2e-101	1	CL0219
Manihot-esculenta_cassava2314.valid.m1	292	344	292	344	52	DUF1785	Domain	1	52	52	68.3	2.1e-19	1	No_clan
Manihot-esculenta_cassava2314.valid.m1	753	791	753	804	51	DUF3734	Family	2	40	108	15.8	0.009	0	No_clan
Manihot-esculenta_cassava2314.valid.m1	353	483	352	484	132	PAZ	Family	2	133	134	96.9	4.7e-28	1	No_clan
Manihot-esculenta_cassava2314.valid.m1	641	947	639	950	311	Piwi	Family	2	301	304	306.7	1e-91	1	CL0219
Manihot-esculenta_cassava23918.valid.m1	293	345	293	345	52	DUF1785	Domain	1	52	52	76.2	7.4e-22	1	No_clan
Manihot-esculenta_cassava23918.valid.m1	351	481	347	482	135	PAZ	Family	6	133	134	106.3	5.9e-31	1	No_clan
Manihot-esculenta_cassava23918.valid.m1	637	952	635	953	318	Piwi	Family	2	303	304	352.8	9.6e-106	1	CL0219
Manihot-esculenta_cassava27530.m1	179	231	179	231	52	DUF1785	Domain	1	52	52	81.2	2e-23	1	No_clan
Manihot-esculenta_cassava27530.m1	234	368	232	369	137	PAZ	Family	3	133	134	117.3	2.3e-34	1	No_clan
Manihot-esculenta_cassava27530.m1	521	840	520	841	321	Piwi	Family	2	303	304	369.9	5.9e-111	1	CL0219
Manihot-esculenta_cassava35367.valid.m1	287	339	287	339	52	DUF1785	Domain	1	52	52	74.8	2e-21	1	No_clan
Manihot-esculenta_cassava35367.valid.m1	341	476	340	477	137	PAZ	Family	2	133	134	119.7	4.3e-35	1	No_clan
Manihot-esculenta_cassava35367.valid.m1	629	948	628	949	321	Piwi	Family	2	303	304	379.6	6.3e-114	1	CL0219
Manihot-esculenta_cassava43463.valid.m1	227	278	226	278	52	DUF1785	Domain	2	52	52	67.4	4.2e-19	1	No_clan
Manihot-esculenta_cassava43463.valid.m1	280	415	279	416	137	PAZ	Family	2	133	134	100.4	4e-29	1	No_clan
Manihot-esculenta_cassava43463.valid.m1	563	870	562	870	308	Piwi	Family	2	304	304	347.9	3e-104	1	CL0219
Manihot-esculenta_cassava586.valid.m1	284	336	284	336	52	DUF1785	Domain	1	52	52	74.8	2e-21	1	No_clan
Manihot-esculenta_cassava586.valid.m1	338	473	337	474	137	PAZ	Family	2	133	134	116.8	3.4e-34	1	No_clan
Manihot-esculenta_cassava586.valid.m1	626	944	625	946	321	Piwi	Family	2	302	304	379.3	7.8e-114	1	CL0219
Manihot-esculenta_cassava7327.valid.m1	296	347	296	348	52	DUF1785	Domain	1	51	52	49	2.3e-13	1	No_clan
Manihot-esculenta_cassava7327.valid.m1	355	485	349	487	138	PAZ	Family	6	132	134	92.4	1.2e-26	1	No_clan
Manihot-esculenta_cassava7327.valid.m1	650	947	649	948	299	Piwi	Family	2	303	304	300.8	6.2e-90	1	CL0219
Manihot-esculenta_cassava7471.valid.m1	227	278	226	278	52	DUF1785	Domain	2	52	52	67.6	3.6e-19	1	No_clan
Manihot-esculenta_cassava7471.valid.m1	280	413	279	415	136	PAZ	Family	2	132	134	102.2	1.1e-29	1	No_clan
Manihot-esculenta_cassava7471.valid.m1	562	869	561	869	308	Piwi	Family	2	304	304	344.9	2.4e-103	1	CL0219
Mimulus-guttatus__mgf000736m	315	367	315	367	52	DUF1785	Domain	1	52	52	52.6	1.7e-14	1	No_clan
Mimulus-guttatus__mgf000736m	368	503	368	507	139	PAZ	Family	1	130	134	90	6.2e-26	1	No_clan
Mimulus-guttatus__mgf000736m	663	961	662	963	301	Piwi	Family	2	302	304	296.8	1e-88	1	CL0219
Mimulus-guttatus__mgf005010m	369	420	368	420	52	DUF1785	Domain	2	52	52	82.3	9.5e-24	1	No_clan
Mimulus-guttatus__mgf005010m	422	557	421	558	137	PAZ	Family	2	133	134	126	4.7e-37	1	No_clan
Mimulus-guttatus__mgf005010m	712	1031	711	1032	321	Piwi	Family	2	303	304	377.6	2.7e-113	1	CL0219
Mimulus-guttatus__mgf006816m	302	354	302	354	52	DUF1785	Domain	1	52	52	68.9	1.5e-19	1	No_clan
Mimulus-guttatus__mgf006816m	363	493	362	494	132	PAZ	Family	2	133	134	102.1	1.2e-29	1	No_clan
Mimulus-guttatus__mgf006816m	651	950	649	954	305	Piwi	Family	2	300	304	308.1	3.7e-92	1	CL0219
Mimulus-guttatus__mgf009177m	251	302	250	302	52	DUF1785	Domain	2	52	52	68	2.7e-19	1	No_clan
Mimulus-guttatus__mgf009177m	307	442	303	443	140	PAZ	Family	5	133	134	98.5	1.5e-28	1	No_clan
Mimulus-guttatus__mgf009177m	590	896	589	897	308	Piwi	Family	2	303	304	334.7	3e-100	1	CL0219
Mimulus-guttatus__mgf010559m	216	267	215	267	52	DUF1785	Domain	2	52	52	55.8	1.8e-15	1	No_clan
Mimulus-guttatus__mgf010559m	269	408	268	409	141	PAZ	Family	2	133	134	103.8	3.6e-30	1	No_clan
Mimulus-guttatus__mgf010559m	556	863	555	863	308	Piwi	Family	2	304	304	347.3	4.5e-104	1	CL0219
Mimulus-guttatus__mgf011041m	251	303	251	303	52	DUF1785	Domain	1	52	52	44.3	6.9e-12	1	No_clan
Mimulus-guttatus__mgf011041m	324	442	304	444	140	PAZ	Family	19	132	134	76.7	8.4e-22	1	No_clan

Mimulus-guttatus__mgf011041m	602	896	601	900	299	Piwi	Family	2	300	304	278	5.5e-83	1	CL0219
Mimulus-guttatus__mgf011360m	266	318	266	318	52	DUF1785	Domain	1	52	52	77.5	3e-22	1	No_clan
Mimulus-guttatus__mgf011360m	320	456	319	459	140	PAZ	Family	2	129	134	97.9	2.3e-28	1	No_clan
Mimulus-guttatus__mgf011360m	590	909	589	910	321	Piwi	Family	2	303	304	380.4	3.8e-114	1	CL0219
Mimulus-guttatus__mgf015766m	199	249	198	250	52	DUF1785	Domain	2	51	52	40.2	1.3e-10	1	No_clan
Mimulus-guttatus__mgf015766m	252	383	251	384	133	PAZ	Family	2	133	134	91.6	2.1e-26	1	No_clan
Mimulus-guttatus__mgf015766m	531	840	531	840	309	Piwi	Family	1	304	304	271.2	6.5e-81	1	CL0219
Mimulus-guttatus__mgf016487m	205	256	204	256	52	DUF1785	Domain	2	52	52	72.2	1.3e-20	1	No_clan
Mimulus-guttatus__mgf016487m	258	395	257	396	139	PAZ	Family	2	133	134	99.5	7.5e-29	1	No_clan
Mimulus-guttatus__mgf016487m	543	854	542	854	312	Piwi	Family	2	304	304	325.2	2.4e-97	1	CL0219
Mimulus-guttatus__mgf016601m	255	305	255	305	50	DUF1785	Domain	2	52	52	74.9	1.9e-21	1	No_clan
Mimulus-guttatus__mgf016601m	310	441	307	442	135	PAZ	Family	5	133	134	102.3	1e-29	1	No_clan
Mimulus-guttatus__mgf016601m	592	910	590	911	321	Piwi	Family	2	303	304	351.2	2.8e-105	1	CL0219
Mimulus-guttatus__mgf019782m	315	365	315	365	50	DUF1785	Domain	2	52	52	74.2	3e-21	1	No_clan
Mimulus-guttatus__mgf019782m	370	499	367	501	134	PAZ	Family	5	132	134	84.7	2.7e-24	1	No_clan
Mimulus-guttatus__mgf019782m	651	964	650	965	315	Piwi	Family	2	303	304	350.3	5.3e-105	1	CL0219
Medicago-truncatula_Medtr2g034460	184	236	184	237	53	DUF1785	Domain	1	51	52	43.1	1.6e-11	1	No_clan
Medicago-truncatula_Medtr2g034460	241	367	238	376	138	PAZ	Family	4	124	134	81.6	2.5e-23	1	No_clan
Medicago-truncatula_Medtr2g034460	539	833	537	837	300	Piwi	Family	2	300	304	278.6	3.8e-83	1	CL0219
Medicago-truncatula_Medtr3g105930	245	296	244	296	52	DUF1785	Domain	2	52	52	60.5	6.2e-17	1	No_clan
Medicago-truncatula_Medtr3g105930	298	436	297	437	140	PAZ	Family	2	133	134	97	4.5e-28	1	No_clan
Medicago-truncatula_Medtr3g105930	584	898	583	899	316	Piwi	Family	2	303	304	324.9	3e-97	1	CL0219
Medicago-truncatula_Medtr4g114860	213	265	213	266	53	DUF1785	Domain	1	51	52	41	7.2e-11	1	No_clan
Medicago-truncatula_Medtr4g114860	280	403	267	405	138	PAZ	Family	13	132	134	77.7	4e-22	1	No_clan
Medicago-truncatula_Medtr4g114860	568	865	566	866	300	Piwi	Family	2	303	304	299.3	1.9e-89	1	CL0219
Medicago-truncatula_Medtr5g045600	316	368	316	368	52	DUF1785	Domain	1	52	52	72.9	8.1e-21	1	No_clan
Medicago-truncatula_Medtr5g045600	378	507	376	508	132	PAZ	Family	3	133	134	98.7	1.3e-28	1	No_clan
Medicago-truncatula_Medtr5g045600	665	974	663	977	314	Piwi	Family	2	301	304	308.6	2.6e-92	1	CL0219
Medicago-truncatula_Medtr5g094930	257	308	256	308	52	DUF1785	Domain	2	52	52	64.2	4.3e-18	1	No_clan
Medicago-truncatula_Medtr5g094930	310	447	309	448	139	PAZ	Family	2	133	134	102.2	1.1e-29	1	No_clan
Medicago-truncatula_Medtr5g094930	595	902	594	902	308	Piwi	Family	2	304	304	346	1.1e-103	1	CL0219
Medicago-truncatula_Medtr5g094940	233	284	232	284	52	DUF1785	Domain	2	52	52	64.1	4.6e-18	1	No_clan
Medicago-truncatula_Medtr5g094940	286	423	285	424	139	PAZ	Family	2	133	134	98.4	1.6e-28	1	No_clan
Medicago-truncatula_Medtr5g094940	569	876	568	876	308	Piwi	Family	2	304	304	338.9	1.6e-101	1	CL0219
Medicago-truncatula_Medtr8g118920	184	230	178	230	52	DUF1785	Domain	7	52	52	64.2	4.1e-18	1	No_clan
Medicago-truncatula_Medtr8g118920	232	367	231	368	137	PAZ	Family	2	133	134	119.9	3.7e-35	1	No_clan
Medicago-truncatula_Medtr8g118920	520	839	519	840	321	Piwi	Family	2	303	304	368	2.1e-110	1	CL0219
Physcomitrella-patens_1885752_locus	185	236	184	236	52	DUF1785	Domain	2	52	52	78.3	1.6e-22	1	No_clan
Physcomitrella-patens_1885752_locus	238	372	237	373	136	PAZ	Family	2	133	134	123.5	2.9e-36	1	No_clan
Physcomitrella-patens_1885752_locus	525	840	524	842	318	Piwi	Family	2	302	304	370.6	3.5e-111	1	CL0219
Physcomitrella-patens_1888444_locus	238	289	237	289	52	DUF1785	Domain	2	52	52	59.6	1.1e-16	1	No_clan
Physcomitrella-patens_1888444_locus	309	420	292	428	136	PAZ	Family	17	125	134	82.2	1.6e-23	1	No_clan
Physcomitrella-patens_1888444_locus	573	878	573	879	306	Piwi	Family	1	303	304	275.9	2.4e-82	1	CL0219
Physcomitrella-patens_1901732_locus	255	306	254	306	52	DUF1785	Domain	2	52	52	56	1.5e-15	1	No_clan
Physcomitrella-patens_1901732_locus	327	442	308	448	140	PAZ	Family	21	127	134	82.2	1.7e-23	1	No_clan
Physcomitrella-patens_1901732_locus	593	892	593	893	300	Piwi	Family	1	290	304	269.7	1.9e-80	1	CL0219
Physcomitrella-patens_1904560_locus	405	456	404	456	52	DUF1785	Domain	2	52	52	82.6	7.3e-24	1	No_clan
Physcomitrella-patens_1904560_locus	458	592	457	593	136	PAZ	Family	2	133	134	115.7	7.1e-34	1	No_clan
Physcomitrella-patens_1904560_locus	745	1063	744	1065	321	Piwi	Family	2	302	304	374.7	2e-112	1	CL0219
Physcomitrella-patens_1910596_locus	259	310	258	310	52	DUF1785	Domain	2	52	52	55.1	2.8e-15	1	No_clan
Physcomitrella-patens_1910596_locus	332	445	313	451	138	PAZ	Family	19	126	134	78	3.2e-22	1	No_clan
Physcomitrella-patens_1910596_locus	597	902	597	903	306	Piwi	Family	1	303	304	297.8	5.3e-89	1	CL0219
Physcomitrella-patens_1912837_locus	259	310	258	310	52	DUF1785	Domain	2	52	52	82.9	6.2e-24	1	No_clan
Physcomitrella-patens_1912837_locus	312	446	311	447	136	PAZ	Family	2	133	134	119.9	3.7e-35	1	No_clan
Physcomitrella-patens_1912837_locus	599	917	598	919	321	Piwi	Family	2	302	304	370.9	3e-111	1	CL0219
Populus-trichocarpa_POPTR_0001s22120	205	257	205	257	52	DUF1785	Domain	1	52	52	80.1	4.4e-23	1	No_clan
Populus-trichocarpa_POPTR_0001s22120	262	393	259	394	135	PAZ	Family	5	133	134	94.6	2.4e-27	1	No_clan
Populus-trichocarpa_POPTR_0001s22120	550	868	548	869	321	Piwi	Family	2	303	304	356.3	8.2e-107	1	CL0219
Populus-trichocarpa_POPTR_0001s22710	227	278	226	278	52	DUF1785	Domain	2	52	52	70.9	3.4e-20	1	No_clan
Populus-trichocarpa_POPTR_0001s22710	280	417	279	418	139	PAZ	Family	2	133	134	104.8	1.7e-30	1	No_clan
Populus-trichocarpa_POPTR_0001s22710	565	872	564	872	308	Piwi	Family	2	304	304	343.6	5.8e-103	1	CL0219
Populus-trichocarpa_POPTR_0006s02680	244	295	243	295	52	DUF1785	Domain	2	52	52	65.3	1.9e-18	1	No_clan
Populus-trichocarpa_POPTR_0006s02680	297	434	296	435	139	PAZ	Family	2	133	134	98.1	2e-28	1	No_clan
[illegible]	[illegible]	[illegible]	[illegible]	[illegible]	[illegible]	[illegible]	[illegible]	[illegible]	[illegible]	[illegible]	[illegible]	[illegible]	[illegible]	[illegible]

Populus-trichocarpa_POPTR_0006s02680	583	891	582	891	309	Piwi	Family	2	304	304	335.5	1.8e-100	1	CL0219
Populus-trichocarpa_POPTR_0006s12010	245	296	244	296	52	DUF1785	Domain	2	52	52	78.2	1.7e-22	1	No_clan
Populus-trichocarpa_POPTR_0006s12010	317	434	306	435	129	PAZ	Family	14	133	134	103	6e-30	1	No_clan
Populus-trichocarpa_POPTR_0006s12010	584	886	582	887	305	Piwi	Family	2	303	304	356	9.8e-107	1	CL0219
Populus-trichocarpa_POPTR_0008s01100	226	277	225	277	52	DUF1785	Domain	2	52	52	70.6	4e-20	1	No_clan
Populus-trichocarpa_POPTR_0008s01100	279	418	278	419	141	PAZ	Family	2	133	134	103.7	3.8e-30	1	No_clan
Populus-trichocarpa_POPTR_0008s01100	566	884	565	884	319	Piwi	Family	2	304	304	323.9	5.9e-97	1	CL0219
Populus-trichocarpa_POPTR_0008s15860	290	342	290	342	52	DUF1785	Domain	1	52	52	75.5	1.2e-21	1	No_clan
Populus-trichocarpa_POPTR_0008s15860	344	479	343	480	137	PAZ	Family	2	133	134	116.7	3.6e-34	1	No_clan
Populus-trichocarpa_POPTR_0008s15860	632	950	631	952	321	Piwi	Family	2	302	304	378.6	1.3e-113	1	CL0219
Populus-trichocarpa_POPTR_0009s00660	273	325	273	325	52	DUF1785	Domain	1	52	52	79	9.8e-23	1	No_clan
Populus-trichocarpa_POPTR_0009s00660	330	461	327	462	135	PAZ	Family	5	133	134	98.5	1.5e-28	1	No_clan
Populus-trichocarpa_POPTR_0009s00660	636	954	635	955	320	Piwi	Family	2	303	304	353.4	6e-106	1	CL0219
Populus-trichocarpa_POPTR_0010s09150	111	163	111	163	52	DUF1785	Domain	1	52	52	75.9	9.5e-22	1	No_clan
Populus-trichocarpa_POPTR_0010s09150	165	300	164	301	137	PAZ	Family	2	133	134	117.4	2.2e-34	1	No_clan
Populus-trichocarpa_POPTR_0010s09150	453	774	452	776	324	Piwi	Family	2	302	304	369.6	7.2e-111	1	CL0219
Populus-trichocarpa_POPTR_0010s17100	334	385	333	385	52	DUF1785	Domain	2	52	52	68.7	1.7e-19	1	No_clan
Populus-trichocarpa_POPTR_0010s17100	394	523	393	525	132	PAZ	Family	2	132	134	97.6	2.9e-28	1	No_clan
Populus-trichocarpa_POPTR_0010s17100	682	988	680	991	311	Piwi	Family	2	301	304	312.4	1.8e-93	1	CL0219
Populus-trichocarpa_POPTR_0012s03410	345	397	345	397	52	DUF1785	Domain	1	52	52	81.1	2.2e-23	1	No_clan
Populus-trichocarpa_POPTR_0012s03410	399	534	398	535	137	PAZ	Family	2	133	134	123.6	2.6e-36	1	No_clan
Populus-trichocarpa_POPTR_0012s03410	689	1008	688	1009	321	Piwi	Family	2	303	304	380.7	2.9e-114	1	CL0219
Populus-trichocarpa_POPTR_0012s11920	177	228	177	228	51	DUF1785	Domain	1	52	52	39.1	2.9e-10	1	No_clan
Populus-trichocarpa_POPTR_0012s11920	238	362	229	366	137	PAZ	Family	9	130	134	79.8	9.3e-23	1	No_clan
Populus-trichocarpa_POPTR_0012s11920	529	820	527	824	297	Piwi	Family	2	300	304	311.1	4.8e-93	1	CL0219
Populus-trichocarpa_POPTR_0014s15760	182	233	181	233	52	DUF1785	Domain	2	52	52	62	2e-17	1	No_clan
Populus-trichocarpa_POPTR_0014s15760	235	374	234	375	141	PAZ	Family	2	133	134	99	1.1e-28	1	No_clan
Populus-trichocarpa_POPTR_0014s15760	521	830	521	830	309	Piwi	Family	1	304	304	340.4	5.4e-102	1	CL0219
Populus-trichocarpa_POPTR_0015s05550	355	407	355	407	52	DUF1785	Domain	1	52	52	81.1	2.2e-23	1	No_clan
Populus-trichocarpa_POPTR_0015s05550	409	544	408	545	137	PAZ	Family	2	133	134	121.5	1.2e-35	1	No_clan
Populus-trichocarpa_POPTR_0015s05550	699	1018	698	1019	321	Piwi	Family	2	303	304	377.1	3.7e-113	1	CL0219
Populus-trichocarpa_POPTR_0015s15780	226	277	226	277	51	DUF1785	Domain	1	52	52	39.3	2.5e-10	1	No_clan
Populus-trichocarpa_POPTR_0015s15780	280	411	278	415	137	PAZ	Family	3	130	134	83.2	7.9e-24	1	No_clan
Populus-trichocarpa_POPTR_0015s15780	536	827	534	831	297	Piwi	Family	2	300	304	299.6	1.5e-89	1	CL0219
Populus-trichocarpa_POPTR_0016s02480	235	286	234	286	52	DUF1785	Domain	2	52	52	60.2	7.6e-17	1	No_clan
Populus-trichocarpa_POPTR_0016s02480	288	425	287	426	139	PAZ	Family	2	133	134	95.8	1e-27	1	No_clan
Populus-trichocarpa_POPTR_0016s02480	574	882	573	882	309	Piwi	Family	2	304	304	335.5	1.7e-100	1	CL0219
Ricinus_communis_29589.t000074	292	344	292	344	52	DUF1785	Domain	1	52	52	81.5	1.7e-23	1	No_clan
Ricinus_communis_29589.t000074	348	480	346	481	135	PAZ	Family	4	133	134	105.4	1.1e-30	1	No_clan
Ricinus_communis_29589.t000074	633	948	632	949	317	Piwi	Family	2	303	304	340.4	5.5e-102	1	CL0219
Ricinus_communis_29677.t000007	345	397	345	397	52	DUF1785	Domain	1	52	52	83.4	4.2e-24	1	No_clan
Ricinus_communis_29677.t000007	399	534	398	535	137	PAZ	Family	2	133	134	123	4e-36	1	No_clan
Ricinus_communis_29677.t000007	688	1007	687	1008	321	Piwi	Family	2	303	304	377.9	2.1e-113	1	CL0219
Ricinus_communis_29684.t000014	239	290	238	290	52	DUF1785	Domain	2	52	52	68.2	2.4e-19	1	No_clan
Ricinus_communis_29684.t000014	292	427	291	428	137	PAZ	Family	2	133	134	96.9	4.6e-28	1	No_clan
Ricinus_communis_29684.t000014	575	882	574	882	308	Piwi	Family	2	304	304	355.5	1.4e-106	1	CL0219
Ricinus_communis_29807.t000009	245	297	245	297	52	DUF1785	Domain	1	52	52	80.4	3.5e-23	1	No_clan
Ricinus_communis_29807.t000009	300	434	298	435	137	PAZ	Family	3	133	134	117.1	2.7e-34	1	No_clan
Ricinus_communis_29807.t000009	587	906	586	907	321	Piwi	Family	2	303	304	369.1	1e-110	1	CL0219
Ricinus_communis_29813.t000096	323	375	323	375	52	DUF1785	Domain	1	52	52	71.1	2.9e-20	1	No_clan
Ricinus_communis_29813.t000096	385	513	383	515	132	PAZ	Family	3	132	134	96.8	5.1e-28	1	No_clan
Ricinus_communis_29813.t000096	672	978	670	981	311	Piwi	Family	2	301	304	304	6.6e-91	1	CL0219
Ricinus_communis_29828.t000011	230	281	229	281	52	DUF1785	Domain	2	52	52	62.4	1.6e-17	1	No_clan
Ricinus_communis_29828.t000011	283	422	282	423	141	PAZ	Family	2	133	134	95.2	1.6e-27	1	No_clan
Ricinus_communis_29828.t000011	571	877	570	878	308	Piwi	Family	2	303	304	333.2	8.8e-100	1	CL0219
Ricinus_communis_29844.t000058	285	337	285	337	52	DUF1785	Domain	1	52	52	75.5	1.2e-21	1	No_clan
Ricinus_communis_29844.t000058	339	474	338	475	137	PAZ	Family	2	133	134	118.9	7.7e-35	1	No_clan
Ricinus_communis_29844.t000058	627	946	626	947	321	Piwi	Family	2	303	304	380.5	3.4e-114	1	CL0219
Ricinus_communis_30093.t000002	310	344	297	349	52	DUF1785	Domain	14	47	52	26.4	2.6e-06	1	No_clan
Ricinus_communis_30093.t000002	351	480	350	485	135	PAZ	Family	2	129	134	97.7	2.7e-28	1	No_clan
Ricinus_communis_30093.t000002	640	933	639	934	295	Piwi	Family	2	303	304	292.9	1.6e-87	1	CL0219
Sorghum-bicolor_Sb01g004920	359	411	359	411	52	DUF1785	Domain	1	52	52	79.8	5.7e-23	1	No_clan
Sorghum-bicolor_Sb01g004920	413	548	412	549	137	PAZ	Family	2	133	134	105.8	8.2e-31	1	No_clan
[illegible]	[illegible]	[illegible]	[illegible]	[illegible]	[illegible]	[illegible]	[illegible]	[illegible]	[illegible]	[illegible]	[illegible]	[illegible]	[illegible]	[illegible]

Sorghum-bicolor_Sb01g004920	704	1024	703	1025	322	Piwi	Family	2	303	304	344.6	3e-103	1	CL0219
Sorghum-bicolor_Sb01g011870	556	608	555	608	53	DUF1785	Domain	2	52	52	75.8	9.7e-22	1	No_clan
Sorghum-bicolor_Sb01g011870	610	742	609	744	135	PAZ	Family	2	132	134	108.9	9.2e-32	1	No_clan
Sorghum-bicolor_Sb01g011870	901	1215	900	1216	316	Piwi	Family	2	303	304	322.5	1.5e-96	1	CL0219
Sorghum-bicolor_Sb01g011880	394	446	394	446	52	DUF1785	Domain	1	52	52	79.2	8.8e-23	1	No_clan
Sorghum-bicolor_Sb01g011880	448	580	447	582	135	PAZ	Family	2	132	134	105.2	1.3e-30	1	No_clan
Sorghum-bicolor_Sb01g011880	698	1045	697	1047	350	Piwi	Family	2	302	304	341.3	2.9e-102	1	CL0219
Sorghum-bicolor_Sb01g032060	340	391	340	392	52	DUF1785	Domain	1	51	52	63.2	8.3e-18	1	No_clan
Sorghum-bicolor_Sb01g032060	403	531	400	532	132	PAZ	Family	4	133	134	106.3	6e-31	1	No_clan
Sorghum-bicolor_Sb01g032060	689	993	688	996	308	Piwi	Family	2	301	304	305.8	2e-91	1	CL0219
Sorghum-bicolor_Sb02g005150	343	394	342	394	52	DUF1785	Domain	2	52	52	71.4	2.4e-20	1	No_clan
Sorghum-bicolor_Sb02g005150	397	530	395	531	136	PAZ	Family	3	133	134	99.3	8.8e-29	1	No_clan
Sorghum-bicolor_Sb02g005150	676	992	675	995	320	Piwi	Family	2	301	304	331.7	2.5e-99	1	CL0219
Sorghum-bicolor_Sb02g032980	358	411	358	411	53	DUF1785	Domain	1	52	52	66.4	8.5e-19	1	No_clan
Sorghum-bicolor_Sb02g032980	413	547	412	549	137	PAZ	Family	2	132	134	102.8	7.3e-30	1	No_clan
Sorghum-bicolor_Sb02g032980	700	1001	699	1002	303	Piwi	Family	2	303	304	334.9	2.6e-100	1	CL0219
Sorghum-bicolor_Sb03g011020	220	271	219	271	52	DUF1785	Domain	2	52	52	73.6	5e-21	1	No_clan
Sorghum-bicolor_Sb03g011020	273	407	272	408	136	PAZ	Family	2	133	134	105	1.5e-30	1	No_clan
Sorghum-bicolor_Sb03g011020	555	861	554	861	307	Piwi	Family	2	304	304	346.4	8.6e-104	1	CL0219
Sorghum-bicolor_Sb04g038420	314	365	313	365	52	DUF1785	Domain	2	52	52	82.9	5.9e-24	1	No_clan
Sorghum-bicolor_Sb04g038420	367	502	366	503	137	PAZ	Family	2	133	134	120.2	2.9e-35	1	No_clan
Sorghum-bicolor_Sb04g038420	657	977	656	978	322	Piwi	Family	2	303	304	370.9	2.9e-111	1	CL0219
Sorghum-bicolor_Sb06g025560	370	421	369	421	52	DUF1785	Domain	2	52	52	81.1	2.2e-23	1	No_clan
Sorghum-bicolor_Sb06g025560	423	558	422	559	137	PAZ	Family	2	133	134	116.8	3.4e-34	1	No_clan
Sorghum-bicolor_Sb06g025560	713	1032	712	1033	321	Piwi	Family	2	303	304	379.6	6.7e-114	1	CL0219
Sorghum-bicolor_Sb06g028510	398	446	396	446	50	DUF1785	Domain	3	52	52	46.9	1e-12	1	No_clan
Sorghum-bicolor_Sb06g028510	461	581	448	584	136	PAZ	Family	12	131	134	79.4	1.2e-22	1	No_clan
Sorghum-bicolor_Sb06g028510	749	1047	746	1048	302	Piwi	Family	3	303	304	296.7	1.2e-88	1	CL0219
Sorghum-bicolor_Sb09g000530	393	445	393	445	52	DUF1785	Domain	1	52	52	80.8	2.8e-23	1	No_clan
Sorghum-bicolor_Sb09g000530	447	582	446	583	137	PAZ	Family	2	133	134	117.4	2.2e-34	1	No_clan
Sorghum-bicolor_Sb09g000530	737	1057	736	1058	322	Piwi	Family	2	303	304	372.8	7.6e-112	1	CL0219
Sorghum-bicolor_Sb09g030910	228	279	227	279	52	DUF1785	Domain	2	52	52	68.8	1.6e-19	1	No_clan
Sorghum-bicolor_Sb09g030910	281	417	280	418	138	PAZ	Family	2	133	134	99.3	8.3e-29	1	No_clan
Sorghum-bicolor_Sb09g030910	565	870	564	870	306	Piwi	Family	2	304	304	333.8	5.7e-100	1	CL0219
Sorghum-bicolor_Sb10g023230	276	328	276	328	52	DUF1785	Domain	1	52	52	75.2	1.5e-21	1	No_clan
Sorghum-bicolor_Sb10g023230	330	465	329	466	137	PAZ	Family	2	133	134	125.8	5.4e-37	1	No_clan
Sorghum-bicolor_Sb10g023230	618	937	617	938	321	Piwi	Family	2	303	304	378.4	1.5e-113	1	CL0219
Sorghum-bicolor_Sb10g031030	333	374	332	379	47	DUF1785	Domain	2	42	52	62.1	1.8e-17	1	No_clan
Sorghum-bicolor_Sb10g031030	395	530	394	531	137	PAZ	Family	2	133	134	115.9	6.4e-34	1	No_clan
Sorghum-bicolor_Sb10g031030	644	963	643	964	321	Piwi	Family	2	303	304	363.8	4.2e-109	1	CL0219
Selaginella-moellendorffii_15405134_locus	253	304	252	304	52	DUF1785	Domain	2	52	52	83.7	3.3e-24	1	No_clan
Selaginella-moellendorffii_15405134_locus	306	440	305	441	136	PAZ	Family	2	133	134	120.7	2.1e-35	1	No_clan
Selaginella-moellendorffii_15405134_locus	593	912	592	913	321	Piwi	Family	2	303	304	367.1	4e-110	1	CL0219
Selaginella-moellendorffii_15408087_locus	247	298	246	298	52	DUF1785	Domain	2	52	52	79.3	7.8e-23	1	No_clan
Selaginella-moellendorffii_15408087_locus	300	434	299	435	136	PAZ	Family	2	133	134	120.5	2.4e-35	1	No_clan
Selaginella-moellendorffii_15408087_locus	587	903	586	904	318	Piwi	Family	2	303	304	348.6	1.8e-104	1	CL0219
Selaginella-moellendorffii_15412065_locus	294	345	294	345	51	DUF1785	Domain	1	52	52	71.8	1.7e-20	1	No_clan
Selaginella-moellendorffii_15412065_locus	348	479	346	480	134	PAZ	Family	3	133	134	93.4	5.7e-27	1	No_clan
Selaginella-moellendorffii_15412065_locus	627	930	626	934	308	Piwi	Family	2	300	304	314.6	4e-94	1	CL0219
Selaginella-moellendorffii_15414178_locus	159	207	156	208	52	DUF1785	Domain	4	51	52	58	3.6e-16	1	No_clan
Selaginella-moellendorffii_15414178_locus	230	344	224	351	127	PAZ	Family	10	126	134	80.6	5.2e-23	1	No_clan
Selaginella-moellendorffii_15414178_locus	498	814	497	817	320	Piwi	Family	2	300	304	302.2	2.4e-90	1	CL0219
Selaginella-moellendorffii_15415029_locus	167	217	165	218	53	DUF1785	Domain	3	51	52	34.4	8.2e-09	1	No_clan
Selaginella-moellendorffii_15415029_locus	238	348	223	356	133	PAZ	Family	19	125	134	80.3	6.1e-23	1	No_clan
Selaginella-moellendorffii_15415029_locus	512	809	511	810	299	Piwi	Family	2	303	304	306.1	1.6e-91	1	CL0219
Vitis-vinifera_GSVIVT00000553001	755	806	754	806	52	DUF1785	Domain	2	52	52	66.6	7.4e-19	1	No_clan
Vitis-vinifera_GSVIVT00000553001	833	943	808	944	136	PAZ	Family	19	133	134	102.7	7.6e-30	1	No_clan
Vitis-vinifera_GSVIVT00000553001	1093	1396	1092	1399	307	Piwi	Family	2	301	304	269	3e-80	1	CL0219
Vitis-vinifera_GSVIVT00000886001	365	417	365	417	52	DUF1785	Domain	1	52	52	83.4	4.3e-24	1	No_clan
Vitis-vinifera_GSVIVT00000886001	419	554	418	555	137	PAZ	Family	2	133	134	123.9	2.2e-36	1	No_clan
Vitis-vinifera_GSVIVT00000886001	709	1028	708	1029	321	Piwi	Family	2	303	304	377.9	2.2e-113	1	CL0219
Vitis-vinifera_GSVIVT00001880001	160	211	159	211	52	DUF1785	Domain	2	52	52	67.6	3.6e-19	1	No_clan
Vitis-vinifera_GSVIVT00001880001	221	349	219	351	132	PAZ	Family	3	132	134	100.4	3.8e-29	1	No_clan
Vitis-vinifera_GSVIVT00001880001	508	815	506	818	312	Piwi	Family	2	301	304	305.6	2.2e-91	1	CL0219
Vitis-vinifera_GSVIVT00002671001	201	252	200	252	52	DUF1785	Domain	2	52	52	60.6	5.4e-17	1	No_clan
Vitis-vinifera_GSVIVT00002671001	254	393	253	394	141	PAZ	Family	2	133	134	95.9	9.8e-28	1	No_clan
Vitis-vinifera_GSVIVT00002671001	536	846	536	846	310	Piwi	Family	1	304	304	344.8	2.5e-103	1	CL0219
Vitis-vinifera_GSVIVT00005949001	158	210	158	210	52	DUF1785	Domain	1	52	52	67.2	4.8e-19	1	No_clan
Vitis-vinifera_GSVIVT00005949001	220	348	218	350	132	PAZ	Family	3	132	134	97.2	3.9e-28	1	No_clan
Vitis-vinifera_GSVIVT00005949001	507	813	505	816	311	Piwi	Family	2	301	304	306.5	1.2e-91	1	CL0219
Vitis-vinifera_GSVIVT00016822001	239	291	239	291	52	DUF1785	Domain	1	52	52	68.9	1.4e-19	1	No_clan
Vitis-vinifera_GSVIVT00016822001	293	428	292	429	137	PAZ	Family	2	133	134	113.5	3.5e-33	1	No_clan
Vitis-vinifera_GSVIVT00016822001	581	900	580	901	321	Piwi	Family	2	303	304	363.1	6.6e-109	1	CL0219
Vitis-vinifera_GSVIVT00017422001	223	264	222	280	58	DUF1785	Domain	2	42	52	52.2	2.3e-14	1	No_clan
Vitis-vinifera_GSVIVT00017422001	283	421	282	423	141	PAZ	Family	2	132	134	101.1	2.5e-29	1	No_clan
Vitis-vinifera_GSVIVT00017422001	570	877	569	877	308	Piwi	Family	2	304	304	344.5	3.2e-103	1	CL0219
Vitis-vinifera_GSVIVT00020067001	283	335	283	335	52	DUF1785	Domain	1	52	52	74.1	3.3e-21	1	No_clan
Vitis-vinifera_GSVIVT00020067001	337	472	336	473	137	PAZ	Family	2	133	134	115.8	6.9e-34	1	No_clan
Vitis-vinifera_GSVIVT00020067001	625	944	624	945	321	Piwi	Family	2	303	304	379.4	7.3e-114	1	CL0219
Vitis-vinifera_GSVIVT00023378001	232	283	231	283	52	DUF1785	Domain	2	52	52	65.9	1.3e-18	1	No_clan
Vitis-vinifera_GSVIVT00023378001	285	422	284	424	140	PAZ	Family	2	132	134	100.6	3.4e-29	1	No_clan
Vitis-vinifera_GSVIVT00023378001	571	879	571	879	308	Piwi	Family	1	304	304	341.1	3.3e-102	1	CL0219
Vitis-vinifera_GSVIVT00031923001	168	220	168	220	52	DUF1785	Domain	1	52	52	51.9	2.9e-14	1	No_clan
Vitis-vinifera_GSVIVT00031923001	232	354	221	360	139	PAZ	Family	11	128	134	85.2	1.9e-24	1	No_clan
Vitis-vinifera_GSVIVT00031923001	519	754	517	761	244	Piwi	Family	2	242	304	200.9	1.6e-59	1	CL0219
Vitis-vinifera_GSVIVT00031924001	169	221	169	221	52	DUF1785	Domain	1	52	52	51.7	3.3e-14	1	No_clan
Vitis-vinifera_GSVIVT00031924001	236	348	223	360	137	PAZ	Family	14	121	134	81.8	2.2e-23	1	No_clan
Vitis-vinifera_GSVIVT00031924001	516	811	514	813	299	Piwi	Family	2	302	304	289.7	1.5e-86	1	CL0219
Vitis-vinifera_GSVIVT00031927001	183	235	183	235	52	DUF1785	Domain	1	52	52	53.1	1.2e-14	1	No_clan
Vitis-vinifera_GSVIVT00031927001	238	370	236	374	138	PAZ	Family	3	129	134	82.1	1.8e-23	1	No_clan
Vitis-vinifera_GSVIVT00031927001	538	836	536	839	303	Piwi	Family	2	300	304	303.2	1.2e-90	1	CL0219

Supplementary File 3. Perl script code designed to eliminate multiple DNA sequences inter-domain regions based on the Pfam batch domain search output table.

```
#! /usr/bin/perl
use Getopt::Long;
## Contribution done by Isaac Rodriguez-Arevalo.
## Bachelor in Genomic Sciences.
## Center for Genomic Sciences (CCG-UNAM), Institute of Biotechnology (IBt-UNAM).
## Laboratory of Reproductive Development and Apomixis.
## Department of Genetic Engineering-Langebio. Cinvestav-Irapuato, Mexico

## Cut and paste protein domains from a fasta file using Pfam search results into a new fasta file.


my %opts=();
GetOptions(\%opts, 'i|file=s', 'f|fasta=s', 'o|outfile1=s', 'd|outfile2=s', 'h|?|help'=>\$help);

#if(!$opts{'i'} || !$opts{'f'}){
# print "\n\n\nscript for cut and paste Pfam domains into new archives from a fasta file containing the
sequences\n";
# print "for help write -h/--help/-?\n\n";
#}

if($help){
 print "
 -i = file that contains a tab-delimited domain coordinates taken from Pfam domain search script (check Pfam
website for more description about it).
 -f = fasta files that contain the sequences
 -o = the output file with concatenated sequences of the domains
 -d = name of the outfile for the sequence of each domain
\n\n\n

 If you encounter any bugs or errors in the code, please notify Isaac Rodríguez at isaac [dot] rodriarev [at] gmail
[dot] com
 \n";
}
elsif(!$opts{'i'} || !$opts{'f'}){
	print "\n\n\nscript for cut and paste Pfam domains into new archives from a fasta file containing the
sequences\n";
 print "for help write -h/--help/-?\n\n";
}

## -i = file that contains a tab-delimited domain coordinates taken from Pfam domain search script (check Pfam
website for more description about it).
## -f = fasta files that contain the sequences to be processed

open(INFILE, "$opts{i}") || die "Error I can not open the infile \n";
open(FASTAFILE, "$opts{f}") || die "Error I can not open the fasta file\n";
open(OUT2, ">$opts{o}") || die "Error I can not create the outfile \n";
open(OUT, ">$opts{d}") || die "Error i can not create the oufile \n";


## The site where to start to cut would be the fourth column of the file, and where to end would be the fifth
column.
```

```
## The name of the sequence would be the first column
## The name of the domain is in the 7th column


#Las localizaciones en el arreglo de las regiones de inicio y terminio son las siguientes:
## Para la secuencia start = $data[3]
## Para la secuencia ending = $data[4]

$sum=0;
while(<INFILE>){
 chomp($_);
 $_=~ s/\s+/\t/g;
 @data=split(/\t/,$_);
 $data[3]=~ s/\'//g;
 $data[0]=~ s/\'//g;
 $data[4]=~ s/\'//g;
 $data[7]=~ s/\'//g;

 if($data[1]=~ /SED ID/){
  next;
 }
 else{
  chomp($data[1]);
  $info[$sum][0]=$data[0];
  $info[$sum][1]=$data[3];
  $info[$sum][2]=$data[4];
  $info[$sum][3]=$data[7]; ## el nombre del archivo
  $sum++;
 }
}

#### This is part where it comes out a hash with the names of the new archives, the file name will be the name
of the domaing.
for($i=0; $i<$sum; $i++){
 $flag=0;
 for($j=0; $j<$cont; $j++){
  if($name[$j] eq $info[$i][3]){
   $flag=1;
   last;
  }
 }
 if(!$flag){
  $name[$cont]=$info[$i][3];
  open(OFILE, ">$name[$cont]") || die "Error I can not create the file $name[$cont]\n";
  close(OFILE);
  $cont++;
 }
}

## Rearreglo del archivo fasta y corte de la secuencia.
## Rearrangement of fasta files and cut-and-paste of the sequence-domains.
## Gets the headers and the sequences from the fasta file and saves it into an array

$cont=0;
$flag=0;
while(<FASTAFILE>){
```

```
 chomp($_);
 if($_=~ />/){
  $_=~ s/\>//;
  $final[$cont][1]=$sequence;
  $cont++;
  $final[$cont][0]=$_;
  $sequence=0;
 }
 else{
  $sequence.=$_;
 }
}
$final[$cont][1]=$sequence;

## In the for loop, for each domain, it gets its sequence in nucleotides from the whole string,
## because the info gotten from Pfam was in aminoacids, the for loop
## takes it into account and write the sequences into different files
for($i=1; $i<=$cont; $i++){
 $final[$i][1]=~ s/\s+//g;
 $domains=0;
 for($j=0; $j<$sum; $j++){
  if($final[$i][0]=~ "$info[$j][0]"){
   $start=$info[$j][1];
   $end=$info[$j][2];
   $inicio=1;
   $c=3;
   $sequence=0;
   for($rond=1; $rond<=$end; $rond++){
    $letter=substr($final[$i][1],$inicio,3);
    $inicio=$inicio+3;
    if($rond>=$start){
     $sequence.=$letter;
    }
   }
   $sequence=~ s/^0//;
   print OUT ">".$final[$i][0]."\n";
   print OUT $sequence."\n";
   open(OFILE, ">>$info[$j][3]") || die "Error I can not create the $info[$j][3]\n";
   print OFILE ">".$final[$i][0]."|".$info[$j][3]."\n";
   print OFILE $sequence."\n";
   close OFILE;
   $domains.=$sequence;
  }
 }
 print OUT2 ">".$final[$i][0]."\n";
 $domains=~ s/^0//;
 print OUT2 $domains."\n";
}
```

Supplementary File 4. Fasta files containing the trimmed sequences using the script described in Supplementary File 3.

>Alyrata_16039685_locus
ATCACTGTTGGTAAAAGCTTTTTCACTCGTGAAACTGAGCGAGATGAAGACTTTGGCTTC
GGGGTTGCAGCTGCGAAAGGGTATCGCCACACTCTGAAGCCCACAGCACAAGGTTTGTCT
TTGTGTTTGGACTACTCAGTGTTGGCGTTCCGCAAAGCAATGTCGGTCATTGAATACCTG
AAGTTGTACTTTAACTGGTCCGATATGCGTCAGTTTAGGAATTGTAGGCGTGATGTGGAA
AAGGAACTGACTGGTTTGAAAGTCACTGTGAATCATCGGAAGAACAAGCAGAAACTCACC
ATTGTAGGGCTGAGTATGCAAGACACAAAAGACATCAAATTCGATCTTATTGATCAAGAG
GGAAACGAGCCACCAAGGAAAACGTCCATTGTTGAGTATTTCAGGATAAAGTATGGAAGA
GACATTGTTCACAAAGATATTCCTTGCTTGGATTTGGGGAAAAACGGTAGGCAAAATTTT
GTCCCCATGGAGTTCTGCGACTTGGTTGAGGGACAGATTTATCCAAAGGACGACTTGGAT
AAAGATTCAGCTTTGTGGTTAAAAAAGTTGTCGCTAGTCACTCTTGTTCTGTGTGCTATG
TCTCGGAAGGACGATGGCTACAAGACTCTGAAATGGATAGCCGAGACCAAACTTGGTCTT
GTGACTCAGTGTTTCTTGACCGGTTCTGCCACTAAAGGAGGTGATCAGTACTGGGCAAAT
CTTGCCCTCAAGATGAATGCAAAGGTTGGTGGAAGCAACGTTGAGTTGATGGATACGTTC
TCTTTTTTCCAAAAAGAGGATGAGGTCATGTTCATTGGTGCCGATGTCAATCATCCTGCT
GCTCGGGACAAGATGAGCCCGTCTATTGTTGCTGTTGTGGGTACTCTAAACTGGCCTGCA
GCTAACCGTTATGCAGCTAGAGTCATTGCCCAGCCTCACCGTAAAGAGGAGATACAAGGA
TTTGGCGATGCTTGCCTGGAGCTTGTCAAAGCTCATGTTCAGGCCACCGGGAAACGACCT
AACAAGATTGTGATATTCCGTGATGGTGTCAGCGACGCTCAGTTCGATATGGTTCTCAAT
GTGGAGTTGCTTGATGTTAAGCTGACTTTTGAGAAGAATGGTTACAATCCAAAGATAACG
GTAATCGTAGCCCAGAAACGCCATCAAACCCGTTTCTTCCCTGCCACAAGCAATGATGGA
AGTGATAAAGGCAATGTGCCTTCAGGTACGGTCGTTGATACTAAAGTCATTCACCCATAT
GAGTATGATTTCTACATCTGCAGTCACCATGGAGGGATCGGGACAAGCAAACCGACTCAT
TATTACACTCTTTGGGACGAACTTGGATTCACTTCAGATCAGGTTCAGAAGCTCATCTTT
GAGATGTGCTTCACTTTCACTCGCTGCACCAAACCTGTCTCTCTTGTTCCTCCGGTGTAT
TATGCTGACATGGTTGCCTTTAGAGGAAGGATGTACCACGAG------------------

>Vvinifera_GSVIVT00017422001
CTCCTTGTTCGTCAATCCTTTTTTCACAATGATCCAAAAAATTTCATTGATTTGGGAGGG
GGCGTTCTTGGCTGCAGAGGATTCCATTCAAGTTTTCGAACCACCCAAGGAGGCTTATCA
CTGAATATTGGCAAGttattgattttatttTATGTATCTACTACCATGATAGTGCAAGGG
CCAGTGGTTGATTTTTTAATTGCCAATCAAAATGCGAGGGATCCTTTTTCCCTGGACTGG
GCTAAGGCCAAGAAAATGCTAAAAAATCTGAGGGTGAAGACAAGCCCCTCAAACACCGAG
TACAAAATAACTGGACTGAGTGAGAAGCCTTGCAAGGAGCAGTTGTTTACGCTTAAGCAA
AGAAATGGGAAGGATGAGAATGGCGAGGCCCAAACGATTGAAGTGACTGTTTTTGATTAT
TTTGTTAATCATCGCCGCATAGAACTACGTTATTCTGCAGATTTACCTTGCATTAATGTT
GGGAAGCCAAAACGACCGACTTACTTCCCTATAGAGCTTTGTACCTTGGTGTCGTTACAA
CGTTATACTAAAGCGTTGTCCACTCTTCAAAGAGCTTCACTGGTGGAAAGATCAAGGCAA
AAATTCCTTCTCTGTCTACTTCCAGAGAGGAAAAACTCTGATCTATATGGTCCTTGGAAA
CGAAAGAATCTTTCTGAATATGGAATTGTGACTCAATGCATTGCTCCTACAAGGGTTAAT
GATCAATATCTTACGAATGTTCTCCTAAAGATTAATGCAAAACTTGGTGGATTAAATTCT
ATGCTAGCAGTAGAACATTCCCCTTCTATTCCAATTGTTTCGAAGGGACCCACCATAATC
CTTGGGATGGATGTGTCTCATGGTTCTCCTGGACAATCTGATGTACCATCTATTGCTGCG
GTTGTCAGCTCCAGGCAGTGGCCACTGATTTCGCGCTATAGAGCATCAGTTCGTACACAA

TCTCCAAAGGTTGAGATGATTGATTCTCTGTATAAGCGAGTATCTGAAACTGAAGATGAA
GGCATAATTAGAGAGCTTTTGCTAGACTTTTATGTGAGTTCAGGCAAAAGAAAACCCGAT
CAGATTATCATATTCAGGGATGGAGTCAGCGAGTCTCAGTTCAATCAAGTTCTGAACATC
GAACTGGATCAAATTATTGAGGCCTGCAAGTTCCTTGATGAGAAGTGGTCTCCCAAATTT
GTGGTGATTGTTGCGCAGAAAAACCATCATACCAAGTTTTTCCAACATGGATCTCCTGAT
AACGTCCCACCTGGCACAGTCATAGACAACAAAGTTTGTCATCCACGGAACAATGACTTT
TATCTCTGTGCACATGCTGGAATGATTGGTACTACCAGGCCGACGCATTACCACGTTCTA
TTGGATGAAGTTGGTTTCTCTTCGGATGATCTTCAGGAGCTTGTGCATTCTTTATCCTAT
GTGTACCAAAGGAGCACCACTGCCATTTCCGTAGTTGCTCCCATATGCTATGCCCACTTA
GCAGCTACTCAGATGTCTCAGTTCATGAAG------------------------------

>AGO1902_Aquilegia
CTTGGAGGCATTAACTCTTTATTATCGGTTGAGAAAACTCTTCCACTTCTTAAGGACATT
CCAACCATGATCTTGGGGATGGACGTATCCCATGGTTCACCTGGTCAATCAGACATGCCA
TCCATTGCCGCTGTTGTTGGTTCGAGATCTTGGCCACTAATATCAAGATACAGGGCATCA
GTGAGGACACAGTCTCCGAAGGTTGAGATGATTGATTCCTTGTTCAAGCTTCTACCAAAT
GGAAAGGATGATGGCATTATAAAAGAACTACTGGTTGAATTTTATGAGTCAAGTCGAGGA
CGCAAGCCTGAACAGATTATAATCTTTAGGGATGGAGTTGGCGAGTCTCAATTCAACCAA
GTTCTCAACATTGAACTGGAGCAAATCATAAAGGCACTTGAGCTTTTAGGTGTCACTAAG
CTTCCAAAGTTTACATTGATTGTGGCTCAAAAGAACCACCACACGAAGCTTTTTCAAGCA
AATGACCCAAACAATGTTCCTCCTGGAACTGTGGTGGATACTCGAATTGTGCATCCAAGG
AATTATGACTTTTACATGTGCGCACAGAATGGAATGATTGGAACCTCCCGCCCAGCTCAT
TACCATGTCCTGCTTGACGAGATTGGCTTTTCTCCTGATGATTTGCAAAACCTTGTGCAT
TCTCTTTCCTATGTATATCAACGCAGCACGACTGCCATCTCAATCGTGGCACCTATCTGT
TACGCTCACCTTGCTGCCGCACAGATTGGACAGTTCATAAAG------------------

>GRMZM2G105250_T01 cds:_protein_coding
GTTGCAGTTGGCCGGTCCTTCTTCTCACCACTCATAGACtcaatgGGACCCAAGAACCTT
GGCTTGGGTGTGGAGGGATGGAAGGGTTTCTATCAGACCATCAGGCCGACACAAAAAGGC
TTATCTGTGATCATAGACATTTCTTCATCAGCTTTCATTCGACCCCTGCCACTGATTGAC
TTTGTTATGGAGATTCTGAACAAAGATAACAGGACCTTTAGAAGTATTACTTCCATGGAT

CTTGTCAAGCTCAAGAAAGCCCTCAAGGGTATGAGGATTGAAGTCACACACCGAGGAGAC
ATACGCCGGAAGTACCGAATTGCCAGCTTGACAAACAGTCCTCCATCTTCACAGTTCTTT
GAATCATCTGCTGGAGTTCAGAAGTCCGTAGCAGATTACTTCAGAGAGGCATACCATCTG
GAAATGCACTATGATTTTCTCCCATGCCTCCAAGTTGGCAGTGATCAGAGGCCGAATTAC
CTCCCTATGGAGGTTTGCAAGATAGTAGCTGGACAGCAATACCGGAAGAAGTTGGAAGGC
CAACAGGTCTCTAAACTAATGGACTCCACCTGCCAGCGCCTTTTGCTTGCTATACTGCCA
GAGAAAAATGGCAGCTTATATGGTAATTTCAAAAGGATCTGCGAGACAGAGATTGGTATC
ATGTCGCAGTGTTGTTTGGATAAAAATGTTAGAAGTGCAGGCCCTGCATATTTTGCTAAT
GTTGCTATTAAGATCAATGCCAAGTTTGGAGGAAGAAACTTAGAATTTGCTAATCCCAAA
GAAAGCTTACCGGGTGTTACAATTGAACCAACAATTATATTTGGTGCTGATGTCACTCAC
CCTGCCGCTCTAGATGATACCGCCCCTTCCATTGCTTCTGTTGTTGCCTCCCAAGACTGG
CCCAAGGTGGCTAACTATAATGGGATTGTCCGTGCACAAGGTCATCGTAAAGAGCTCATC
AATGGCCTGGAAGACATTGTCAAGGAACTCCTACTTGCATTCGAGGAGAGGTCTAAGCGG
AGACCCAAGCAGCTGATCTTCTACAGGGATGGCGTAAGTGAGGGCCAATTCAAACAAGTG
CTGGAACAAGAAATCCCTGAGATAGAGAAGGCATGGAAAGCTCTTTACAATGAGAAGCCA
AAGATCACCTTCCTAGTGGTGCAGAAGAGGCACCACACAAGGCTCTTCCCAAACAACCCA
AATGATCGCCAATGGGCGGACAAGAGTGGAAATATTCTACCTGGCACTGTTGTTGACAAG
GATATCTGCCACCCAACAGAATTTGATTTCTTCCTGTGCAGCCATGCTGGGATCAAGGGA
ACTAGCCGTCCTGCGCATTACCATGTCCTGAGAGACGACAACAACTTCACTGCAGACGCG
CTGCAGTCTCTCACATATAACCTATGCTTCTTGTATTCAAGCTGCACTCGCTCTGTGTCA
ATCGCTCCCCCAGCATACTACGCCCACAAGTTAGCGTTCCGCGCCCGCTTCTACGTCAAC

>Creinhardtii_Au9.Cre04.g214250
ACCACACCCGCCTTCCCGGGGCAGGCCTACCACTACGATGTGGAGATACGCTCCGTTGAA
gaggcggcaGGAGGAGGAGGAGGCGGCGGCCGTGGCGGCGGCCGTGGCGGCGGCGGCGGC
GGCGGCGGCGGGCGCGGGGGCCGGCGTGACCTGGCGCCTGCTAGTGCTACTGGCGACGTG
CCGGGTGGCGCCGCCGCCGCCGCCGCCGCTGAGGACCTGCCGCCGCGGCTGGCGCACCGC
GTGATGGCGGCGGCGGCGGCGGCGCACGGCTGGCCGGCGGGGGCGTGGCGGTTTGGCGAC
ccggcgcaactggcggcggccgacCCACGCCGGCTGAGGGCGGCTGCCAGGTCGCTGGTG
GGGTTCAAGGTGGAGTTCCCCATGCCCGGCGGCCGCGCTCGGCGCAAGATGCTGACGGGG
CTGAGCGAGCAGGGAGCAGACCGCACCATGTTCATGAACGAGAAGGAGGGGCGTGAGATG
TCAGTGGCCGAGTACTTCCGCTCCACTGGCCGCCCGCTGCGCCACCCCGGCCTGCCCTGC
GCCAACGTGGGCGACCGCCGCCGCGCAGTCTACATTCCGCTGGAGCTGTGCACTGTGGTT
GCTGGCCAGCGGCGCATGAAGCTGGACGCCACGCAGTCGGCCGGCATGATCACCGCCGCC
AAACAGGATCCGCTGGTGCTGGTGGTGCTGCCGGTCAAGCCCTCGGACGAGTACCGGGAG
GTGAAGCGCGTGTCGGACATCGAGTTGGGCATACCCAGCCAGGTGGTGGTGGGGGGCAAG
GCGCGTGTGGGCTACCGCgagcagAGGGGCGGCGGCCCGCAGTACTGCGCCAACGTGGCC
ATGAAGATTAACAACAAGCTGGGCGGGGTCAACGTGCAGCTCAGCGGCGGACTGCGCTAC
ATGCCCGTGCTGGGCGGCGCGGGCTCCGTGCCCTTCATGGTGTTGGGCGCCGACGTGACG
CACCCCACCGGCGCCGCCGCGCGCGCCGACAGCCGCGACCCCTCCGTGGCGGCTGTGGTG
GGCAGCCTGGACGCCAGCCTGGGCCGCTGGGCCAGCCGCGTGCTGCTGCAGGCGGGGCGC
CAGGAGGTGATCACGGGCATGTGCGGCGCCACAAAGGAGCTGCTGCTGGAGTTCTACAGG
GCAAACAAGCAGGTCAAGCCGCAGCGGCTGGTCATGTACCGCGACGGCGTGTCGGAGGGC
CAGTTCGAGCAGGTCCTAGCGGAGGAGTTCACGGCCCTGCGCCGCGCCTGCCGCGAGCTG
GAGGAGGGCTACCGGCCCGCCATCACCTTCGTGGTGGTGCAGAAGCGCCACAACACCCGC
CTGCTGCCCAACGACCGCGCGTCGGCCGACCCCAAGGGCAACGTGGTCCCCGGCACGGTG
GTGGACAGTGGCATCACCGCCCCGGACGGCTTCGACTTCTACCTCAACAGCCACTCCGGA
CTGCAGGGCACCAACAAGCCCGCCCACTACCACGTGCTGGTGGACGAGATCGGGTTCGGC
GCCGACGGCATGCAGCTGCTCACCTACTGGCTGTGCTACCTCTACCAGCGCACCACCAAG

TCGGTCAGCTACTGCCCGGCCGCCTACTACGCCGACCGCGCCGCCTTCAGGGGCCGCACA
CTGCTGGCC---

>AT5G21150.1
CTCCTTGTTCGCCAGTCCTTTTTCCACAATGACGTAAAGAACTTTGTACCTATTGGTGGA
GGTGTCAGTGGTTGCAGAGGGTTCCATTCAAGTTTCAGAACTACTCAGGGAGGCTTATCC
CTGAATATTGACACTTCAACTACGATGATAGTACAACCTGGACCTGTAGTTGATTTCCTG
CTTGCTAACCAGAACAAGAAAGATCCATACGGAATGGACTGGAACAAGGCTCGACGTGTC
CTCAAGAATCTGAGAGTTCAAATTACTCTTTCCAATAGAGAATACAAGATAAGTGGACTA
AGTGAACACAGCTGCAAAGATCAACTATTTACATGGAGGAAACCTAACGACAAGGGAGAA
TTTGAGGAGGTTGAGATCACAGTGCTCAATTACTATAAAGAGCGTAACATTGAAGTGCGT
TATTCAGGTGACTTCCCTTGCATCAATGTTGGTAAGCCGAAGCGTCCCACTTACTTCCCC
ATTGAGTTCTGTAATCTTGTGTCTCTACAGCGATACACAAAATCGCTTACCAATTTTCAG
AGGGCTGCCCTAGTTGAAAAGTCTAGGCAGAAGTTCCTTCTTTGCATACTCGCTGAAAGG
AAAAACTCTGATGTTTATGGCCCTTGGAAAAAAAAGAATCTTGTTGATCTTGGAATTGTG
ACTCAGTGCATTGCTCCCACCAGACTGAACGATCAGTATCTCACCAATGTTCTCCTGAAG
ATAAATGCCAAGCTTGGTGGATTGAATTCGTTGTTAGCTATGGAGCGCTCACCAGCAATG
CCAAAAGTAACGCAAGTTCCTACCATCATTGTTGGGATGGATGTATCCCATGGTTCCCCT
GGCCAGTCTGATATACCATCAATTGCTGCTGTTGTGAGCTCAAGACAATGGCCACTCATC
TCAAAATATAAGGCATGTGTACGCACACAATCACGCAAAATGGAAATGATTGATAATCTC
TTCAAACCCGTCAATGGCAAAGACGAAGGAATGTTCAGGGAACTCTTGTTAGACTTTTAC
TACAGTTCAGAGAATAGGAAACCAGAGCACATCATTATTTTCAGGGATGGTGTAAGCGAG
TCTCAGTTCAATCAAGTTCTTAATATTGAATTGGATCAGATGATGCAGGCATGCAAGTTT
CTTGATGATACGTGGCATCCGAAGTTTACAGTGATAGTTGCCCAGAAGAACCACCACACA
AAGTTCTTCCAGTCTCGAGGCCCTGATAATGTTCCTCCAGGAACAATCATTGACAGCCAG
ATCTGTCACCCACGCAACTTTGATTTCTATCTCTGCGCCCATGCTGGCATGATTGGAACT
ACAAGGCCAACACATTACCATGTTCTGTATGACGAGATTGGGTTTGCCACAGACGACCTC
CAAGAACTTGTGCATTCTCTGTCCTATGTCTACCAGAGGAGCACCACTGCGATCTCAGTC
GTTGCACCTGTATGTTACGCTCATTTGGCAGCTGCACAGATGGGAACTGTGATGAAG---

>Mtruncatula_Medtr5g094930
CTGCTTGTCCGCCAAAACTTTTTCCACAATGATCCGAAAAATTTCACAGATGTAGGAGGT
GGTGTTCTTGGATGCAGAGGGCTGCATTCTAGCTTCAGGACCACACAAAGTGGGCTGTCT
TTGAATATAGACGTGTCAACAACCATGATTGTCCATCCTGGGCCTGTGGTTGATTTCTTG
ATTGCAAACCAGAATGTGAGAGATCCTTTCAGTCTTGACTGGAATAAGGCTAAAAGGACT
CTAAAGAATTTGAGGATCACTACTAGCCCAACCAACCAAGAGTACAAAATTACTGGTTTA
AGTGAAATGCCATGCAAGGACCAGCTGTTTACTCTGAAGAAGAGGGGTGCTGTTCCTGGA
GAAGATGATACCGAGGAGATCACTGTTTATGATTATTTTGTCAACCGCAGAAAAATTTCT
CTACAGTACTCAGCTGATCTGCCATGTATTAATGTGGGAAAGCCAAAGCGACCTACATTT
GTCCCAGTTGAGCTTTGCTCATTGGTATCCCTTCAACGATATACCAAAGCCCTGTCCACA
CTCCAGAGATCCTCCCTTGTGGAAAAGTCTAGGCAGAAGTTTCTTCTTTGTCTCCTTTCT
GAGAGAAAGAACTCTGATCTTTATGGTCCATGGAAGAAAAAGAATCTTGCAGAGTTTGGA
ATTGTTACTCAGTGTATAGCTCCTACCAGGGTGAACGACCAGTATCTGACCAATGTTTTG
CTGAAGATCAATGCAAAGCTTGGTGGCATGAATTCTCTTTTAGGCGTTGAACACTCTCCA
TCCATTCCCATTGTGTCAAAAGCTCCTACTCTAATTTTGGGCATGGATGTTTCTCATGGT

TCACCTGGGCAAACTGAAATTCCCTCAATTGCTGCGGTCGTAAGCTCAAGGCAATGGCCC
TTGATATCTAAATATAGGGCATGCGTCCGTACTCAGGGTGCTAAGGTTGAAATGATTGAC
AATCTGTTCAAGCCAGTGTCAGATACGGAGGATGAAGGAATAATAAGGGAGCTTTTGATT
GATTTTTATAATAGTTCTGGGAACAGAAAACCAGACAATATCATAATTTTCAGGGATGGT
GTCAGCGAGTCTCAGTTCAACCAAGTTTTGAACATTGAACTGAGCCAAATCATTGAGGCC
TGCAAATTTCTTGATGAAAAGTGGAACCCCAAATTTCTGGTTATTGTTGCCCAGAAGAAT
CATCACACAAAGTTCTTCCAGCCTGGATCTCCGGATAATGTGCCTCCTGGTACTGTTGTG
GACAACAAAATCTGTCATCCCCGGAATTATGATTTCTACATGTGTGCTCATGCTGGAATG
ATTGGTACCAGTAGGCCAACTCATTATCATGTCCTTTTGGATGAGATTGGCTTTTCTCCA
GATGATCTTCAAGAGCTTGTGCACTCTCTCTCCTATGTTTATCAGCGGAGCACTACTGCC
ATCTCTGTTGTTGCTCCAATCTGCTATGCCCATTTGGCTGCTTCTCAAGTGGGACAGTTC
ATGAAG--

>LOC_Os04g06770
CTTTTAGTACGCCAGTCATTTTTCCACAACAATCCTAATAACTTTGTTGACCTGGGTGGT
GGTGTGATGGGCTGTCGAGGATTCCATTCAAGTTTCCGTGGCACACAGAGTGGACTTTCT
CTGAATATCGATGTTTCGACAACTATGATTGTTAAACCTGGTCCTGTTATCGATTTTCTT
CTTGCCAACCAGAAAGTTGATCACCCTGACAGAATTGATTGGCAAAAGGCCAAGCGTGCT
CTCAAGAACTTGAGGATAAGAACCACCCCTGTGAATTCAGAATTCAAGATCATTGGTTTG
AGTGACAGAAATTGCAACGAACAGATGTTTTCGTTGAGGCAGAGGAATGGGAACAATGGG
GATGTTGATGAAGTTGAAGTTACAGTCTATGATTACTTTGTGAAAAACAAAGGCATAGAG
CTGCGCTATTCTGGCAATCTTCCTTGCATAAATGTGGGGAAACCAAAGCGTCCAACTTAT
TTTCCAATAGAGCTATGCTCTCTTATTCCACTACAAAGATACACCAAAGCTTTGTCAACA
CTGCAAAGGTCCTCCCTTGTTGAGAAATCCAGACAGAAGTTCCTCTTGTGCCTTCTTCCT
GAGAGGAAAAATTGTGAAGTTTATGGTCCTTGGAAGAGGAAGTGTCTTGCTGAATTCGGT
ATTGTCACACAATGCCTTGCTCCACAAAGAGTCAATGATCAGTACCTACTCAATTTGCTA
TTGAAGATAAATGCCAAGCTTGGTGGAATAAACTCATTGCTGCAAATTGAAGCATCCCCT
TCAATACCTCTTGTATCGAAGACACCTACCATCATCTTAGGTATGGATGTGTCACACGGT
CAACCAGGACAATCTGATAGACCTTCCATTGCTGCGGTGGTTAGCTCTCGCCAATGGCCT
CTCATCTCTAAATACAGAGCATCGGTGCACACTCAGTCACCTAAGCTAGAAATGATGTCT
TCCTTGTTTAAGCCACGAGGAACTGAAGATGATGGCCTCATTCGGGAATCGCTGATTGAC
TTCTACACTAGTTCTGGAAAACGGAAACCGGATCATGTTATCGTTTTCAGGGATGGAGTT
AGTGAAAGCCAGTTTACTCAGGTCATTAACATTGAGCTTGATCAGATCATTGAGGCATGC
AAATTTCTCGATGAGAAGTGGTCACCAAAGTTCACAGTGATTGTTGCTCAGAAGAATCAT
CATACCAAATTTTTTCAGTCTGGATCTCCAGATAATGTTCCGCCAGGTACTGTTGTGGAC
AAACAAGTGTGCCATCCAAGGAATTATGACTTCTACATGTGTGCTCATGCTGGAATGATT
GGAACGACGAGGCCAACACATTATCATGTTCTGCACGATGAGATAGGTTTCTCCCCTGAT
GATCTGCAGGAGCTAGTGCACTCACTCTCTTATGTGTATCAGAGAAGCACAACAGCCATA
TCAGTTGTTGCTCCGATTTGCTATGCCCATCTTGCTGCTGCTCAGGTGGGCACATTCCTC
AAG---

>GRMZM2G317927_T01 cds:_protein_coding
ATGGTGAGGAAGAAAAGGACTGGCCCTGGAGAGAGTTCTGGGGAGACTTCTGGAGCGCCT
GGTCAGGGCTCCTCACAGCGTCCTCAGGCAACACAACAGGGTGCCCGAGGTGGAGGACAA

CACCAGGTGCGTGGTGGATATCCGGGCCATGGAGTGCCGCCTTCAGAGCACCCAGGTGGT
GGGCCGCCTGAGTATCAACCACGTGGCTATCAGGGACGTGGCGTTCCACCTTTACTTCCT
GGTGGTGGGCCGCCTGAGCCTCAACCGCGTGGCTACCAGGGACATGGTGGATACGGCTAC
CAGGGACATGGTGGATACCAGGGCCGTGGCGGGCCACCTTCACAGCATCCTGGTGGTGGG
CCATCACCTGGGTCTCAGCCTCGTGGCTACCAGGGACGTGGTGGTCTGCGTCCCAGAGGG
GGAGTGCCGCAGCCATACCGTGGCGGGCATGTGGGAGGTAGTGTTGGACCAATTGTTCCT
TCAGGTCCGTCTAGACCAGTTCCCGAGCTGCACCAAGCCCCAGATGTCCAACATCAAGCC
CCTGTGGTGGCAGCACCATCACCACCGGGAGCTGGCTCGTCCTCGCAGCCTGGGATGGCC
GAGGTGAGCTGTCCTGTTGGTAGATCATTTTATTCTCCCAATTTAGGGAGACGCCAGCAA
CTTGGTGAAGGTTTGGAAACTTGGCGTGGTTTCTATCAAAGCATAAGGCCCACACAGATG
GGTCTTTCTCTGAATATTGATATGTCCTCTACTGCATTTATTGAGCCCCTCCCTGTGATT
GATTTTGTTGCTCAGCTTCTTGACAGAGATATCTCAGTTAGACCATTGTCTGATTCTGAT
CGTGTGAAGATTAAAAAAGCCCTACGAGGTGTGAAAGTCGAGGTCACACACCGTGGAAAC
ATGCGTAGGAAGTATCGGATATCTGGCCTCACTTCACAAGCAACAAGGGAGTTATCATTC
CCTATTGATGATCGTGGTACTGTTAAGACTGTGGTGCAATACTTCCTGGAGACTTATGGC
TTTAATATTCAGCACACCACTTTACCTTGTTTGCAAGTGGGCAATCAGCAAAGAATAAAT
TATCTGCCTATGGAGGTCTGTAAGATAGTTGAGGGACAGCGTTACTCAAAACGACTCAAT
GAGAAACAGATCACTGCTCTACTGAAGGTGACTTGCCAGCGTTTGCTGATGGTAATACTG
CCTGACAATAATGGCTCTCTTTACGGGGATCTTAAAAGGATATGTGAGACTGATCTTGGA
TTGGTCTCCCAATGCTGTCTGACTAAACATGTTTTCAAGGCGAACAAGCATCAGTATCTT
GCAAATGTTGCCCTGAAAATAAATGTTAAGGTTGGGGGAAGGAATACTGTACTTGTTGAT
GCTTTGGCAAGGAGAATCCCCCTTGTCAGTGACGTAGCTACTATTATCTTTGGTGCTGAT
GTGACCCATCCCCATCCTGGGGAAGATTCTAGTCCTTCCATTGCAGCTGTGGTTGCTTCT
CAAGACTGGCCTGAGGTTACGAAGTATGCAGGATTAGTGAGTGCTCAAACCCATCGCCAA
GAATTGATACAGGATCTTTTCAACGTACGGCAAGATCCCCAAAGGGGGGCTGTCTCTGGT
GGCATGATTAGGGAACTTCTCATTTCTTTCTGGAGGGCGACTGGACAAAAACCCAAGAGG
ATCATATTCTACAGGGATGGTGTCAGTGAGGGACAGTTCTACCAAGTTCTGTTGTACGAA
CTTGATGCCATTAGAAAGGCCTGTGCATCATTGGAGTCTGATTACCAGCCTCCAGTTACC
TTTGTCGTGGTCCAGAAGCGTCATCACACCAGGTTGTTTGTTAATAATCACAATGATCAG
CGTGCTGCCGATAGAAGTGGCAACATACTGCCGGGCACTGTGGTGGACTCGAAGATTTGC
CATCCAACCGAGTTTGATTTCTACCTGTGCAGCCATGCTGGCATTCAGGGAACAAGCCGT
CCTGCCCATTATCATGTTCTGTGGGATGAGAACAAATTTACGGCTGATGGGTTGCAAACT
CTCACCAACAACTTGTGTTACACGTATGCCAGGTGCACACGCTCAGTATCAATCGTTCCT
CCTGCATACTATGCTCATCTGGCAGCCTTCCGAGCTCGGTTTTACATGGAG---------

>LOC_Os07g28850
GTTGCGTTTGGTCGGTCTTACTTCTCACCGGGGCTCGGATCGAGGGAACTCGACAAGGGC
ATCTTTGCGTGGAAGGGGTTCTACCAGAGTTGCCGGGTCACGCAGCAGGGCCTTTCTCTG
AACATAGACATGTCTTCGACTGCTTTCATTGAACCTGCTattactgtgggatatTTTTTG
AACAATTATGGGAATGAATTGATGAGGACCCTTAAGGGTGTTAAGGTTGAAGTCACTCAC
CGAGGAAATCTACGCAAGAAGTACCGCATTGCTGGCTTCACCGAGCAGTCTGCAGATGTT
CAGACGTTCACATCATCTGATGGTATCAAGACTGTCAAGGAGTATTTCAACAAAAAATAC
AATCTGAAGTTAGCTTTTGGTTATCTTCCATGCCTGCAAGTTGGCAGCAAGGAGAGACCG
AATTACCTGCCCATGGAGCTTTGCAATATAGTTCCTGGACAACGATACAAGAACCGGCTC
AGTCCGACACAGGTTTCCAATCTGATTAACATAACCAACGATCGTCTCCTACTTGTTGTA
ATGACAGATGATAAAAATAATGCCAGCTTATATGGTGACGTTAAAAGAATCTGTGAAACA
GAAATCGGTGTATTGTCACAGTGTTGTCGAGCGAAGCAAGTCTACAAGGAGAGGAATgtt
CAGTACTGCGCAAATGTTGCTCTTAAGATCAATGCCAAGGCTGGAGGAAGGAACTCGGTA
TTTCTTAATGTAGAAGCAAGTTTACCGGTTGTTTCAAAGAGCCCAACTATTATATTTGGT
GCTGATGTTACCCATCCTGGGTCCTTTGATGAAAGTACCCCTTCCATTGCTTCGGTTGTT
GCTTCCGCAGACTGGCCTGAGGTGACCAAGTATAATTCTGTTGTTCGTATGCAAGCTTCT
CGTAAGGAGATTATACAAGATCTTGATAGCATTGTTAGGGAACTTCTCAATGCATTCAAA
AGGGACTCCAAGATGGAGCCGAAGCAGCTCATTTTCTACAGGGACGGCGTAAGCGAGGGT
CAGTTCCAGCAAGTTGTAGAGAGCGAAATACCGGAGATAGAAAAGGCTTGGAAGTCTCTG
TATGCTGGCAAGCCACGAATTACCTTCATAGTGGTGCAGAAGAGGCATCATACAAGGCTG
TTCCCCAACAATtacaatgatCCACGCGGCATGGATGGGACTGGAAATGTTCGTCCAGGC
ACAGTAGTTGATACAGTGATCTGTCACCCTCGAGAGTTTGATTTCTTCCTGTGCAGCCAA
GCCGGGATCAAAGGGACAAGCCGTCCTAGCCATTACCATGTGCTGCGCGACGACAACAAC
TTCACCGCAGATCAGCTTCAGTCTGTCACAAACAACCTGTGCTACTTATATACAAGCTGC
ACTCGCTCGGTGTCTATTCCACCTCCTGTTTACTACGCTCATAAGCTCGCATTCCGCGCT

CGTTTCTACCTCACC---

>Sbicolor_Sb01g032060
ATTCCCATGGGCCGGTCTTTGTATCCACGTTCAATGGGAGAAGCAAAGGAGATTGGTGGT
GGAGCTGTCATGTTACGAGGTTTCTTCCAGAGCTTGAGACCAACAAAGCAAGGTCTTGCC
CTCAATGTTGACCTCTCACTTACAGCTTTCCATGAAAACCAGAAGCGCTGTGACTTTATG
AAGGACCTTTCACAGGTGAAGAGTAGGGCTTTGACAGTAGATGAGAGGAGGGAGGTGGAA
AAAGCATTGAAGAATATCCGAGTGTTCGTGTGCCACCGTGAAACTGACCAAAGGTACCAT
GTGCATGGCTTGACTGAGGAGACAACAGAGAACCTCAAGTTTCGAGATCGCAGTGGGAAG
GATTATACGGTCGTAGATTACTTCAAAGAGCACTACAACCATGATATTAAATTCAGGAAC
CTGCCCTGCTTGCAGATTGGTAAGAGCAAGCCATGCTATGTGCCAATGGAGCTTTGCATG
GTTTGTGAGGGCCAGAAGTTTCTTGGCAAGCTCTCAGATGAACAGACCTCCAAAATGCTC
AGAATGGGCTGCCAAAGACTGCTAATCTGCGTCATGGAGCGGAGGCACCGGGGCTACGCT
GATCTGAAGCGTATTGCAGAAACATCCATTGGTGTCTTGACACAGTGTTGCCTGTATTCC
AACCTAAGCAAGCTGAGCTTTCAGTTCTTGGCCAACTTAGCACTGAAGATAAATGCGAAG
GTTGGTGGAAGCAACGTTGCCCTCTACAACAGCTTGCCATGCCAAATTCCTAGGGTGTTT
TCAGACAAGGAGCCAGTGATGTTCATGGGTGCTGACGTGACACACCCACATCCCCTAGAT
GACTCAAGCCCGTCCGTGGTCGCTGTAGTTGCGAGCATGAATTGGCCTTCAGCAAACAAG
TACATCTCCAGGATGAGATCACAGACGCACCGTAAAGAGATCATCGAGCGCCTTGATGTA
ATGACCGGTGAACTGCTCGAGGAGTTTGTGAAAGAAGTCGGCAAGCTCCCTAGCAGAATC
ATATTCTTCAGAGATGGTGTTAGTGAGACGCTGTTCTACAAGGTGTTGACAGAGGAGCTG
CAGGCAGTGCGACTGGCATGCTCGAGGTACCCGGGCTACAAGCCAGCGATCACGTTCGTG
GTGGTTCAGAAGAGGCAGCACACCAGGCTCTTCCACAGGGAGAAGAATGGCGGCTCCACG
CACTACGCCGACCAGAACGTACCACCGGGAACGGTGGTGGACACCGTGATCACGCACCCA
AGGGAGTTTGATTTCTACCTGTGCAGTCACTGGGGCACCAAGGGGACGAGCAGGCCGACG
CACTACCGCGTGCTGTGGGATGAGAACAACTTCAAGTCTGACGAGATGCAGCAGCTGATA
CACAACCTTTGCTACACGTTTGCCCGGTGCACCAAGCCTGTTTCTCTCGTCCCACCGGCA
TACTACGCACACCTGGCCGCATATAGAGGAAGGCTATACCTTGAG---------------

>Ptrichocarpa_POPTR_0012s03410
ACCGAAATTCCTCAAAGTGGGGGTGAGAGTTCTGAGTCGCAGGAAACTGATACTGGTCGG
GGTGCCCAGCCCCCAGCTGAGAGGAGTGGTCCACCTCAACAGGGAGGAGGTGGAGGAGGA
TACCAAGGTGGAAGGGGTTGGGGTCCCCAGTCTCAGCAAGGAGGTCGTGGAGGTGGGTAT
GGTGGACGAGGCCGGGGAGGGATGCAACAACAGCAATATGGTGGAGCCCCTGAGTACCAG
GGCCGGGGAAGAGGGCAACCTCAGCAAGGAGGTCGAGGATATGGTGGTGGTCGTCCTGGA
GGTGGCCGCGGAGGGCCATCTTCAGGTGGATTTAGACCACCAGCACCCGAGCTGCACCAA
GCTACCCCAGCTCCTTATCCAGCTGTGGTGACCACTCAGCCCACGCCATCTGAGGCAAGT
TCTTCGATGCGACCACCAGAGCCATCGCTAGCATGTCCTGTGGGTCGATCATTTTATTCC
CCTGACCTAGGAAGGAGACAATCACTTGGTGAGGGCTTGGAGAGTTGGCGTGGTTTCTAT
CAAAGTATTCGTCCTACTCAGATGGGACTATCGCTGAACATTGATATGTCATCCACGGCC
TTCATTGAGCCACTGCCAGTCATTGATTTTGTGACTCAGTTATTAAATCGTGACGTTTCC
TCTAGACCGTTGTCTGATTCTGATCGTGTAAAGATTAAAAAGGCACTAAGAGGCGTCAAA

GTTGAAGTTACACACCGTGGAAATATGCGCAGAAAGTACCGTATTTCTGGCTTAACATCA
CAGGCAACACGGGAGCTGACTTTCCCGGTTGATGAAAGAGGAACCCTGAAATCTGTCGTG
GAGTACTTCTACGAAACCTATGGTTTTGTAATTCAACACCCTCAATGGCCTTGTCTACAA
GTAGGAAATCAACAGAGACCTAATTATTTGCCTATGGAGGTCTGCAAGATTGTTGAGGGT
CAGAGGTACTCCAAAAGATTGAACGAGAGACAGATAACTGCATTGTTGAAGGTGACCTGC
CAGCGTTTGCTTATTGTGATTCTCCCTGACAACAATGGTTCTCTTTATGGTGATTTGAAG
CGTATTTGTGAGACAGATCTTGGGCTTGTTTCCCAGTGCTGTTTGACAAAGCATGTGTTC
AAAATGAGCAAGCAATATCTTGCTAATGTGGCTTTGAAGATAAATGTGAAGGTTGGAGGA
AGGAATACAGTACTTGTGGATGCAATATCTAGACGTATCCCTCTAGTCAGCGACCGACCT
ACTATTATTTTTGGTGCTGATGTTACTCATCCTCATCCTGGGGAGGACTCAAGCCCATCT
ATTGCAGCCGTTGTGGCTTCGCAAGATTGGCCAGAGGTTACCAAATATGCTGGCCTGGTT
TGTGCTCAAGCCCACCGACAAGAGCTTATCCAAGATTTATATAAAACATGGCAGGATCCT
GTACGAGGGACAGTGTCTGGTGGCATGATCAAGGAGCTTCTCATATCCTTCCGTAGGGCA
ACCGGGCAGAAGCCACAGCGAATTATATTCTACAGAGATGGTGTCAGTGAAGGACAGTTT
TATCAAGTTTTGTTGTACGAGCTTGATGCTATTCGTAAGGCATGTGCTTCTTTAGAGCCC
AATTACCAGCCTCCTGTGACATTTGTTGTGGTTCAGAAGCGTCATCACACAAGGCTGTTT
GCAAATGATCACCGTGACCGCAATGCTGTTGACAGGAGTGGGAATATATTGCCTGGTACT
GTTGTGGACTCGAAGATCTGCCATCCTACTGAATTCGACTTCTATTTGTGTAGTCACGCT
GGGATTCAGGGCACAAGCCGTCCAGCTCATTACCATGTACTTTGGGATGAGAACAAGTTT
ACTGCTGATGGGCTGCAGTCCCTGACAAACAATCTTTGCTACACATATGCACGATGCACG
AGATCTGTTTCCATTGTGCCACCTGCATACTATGCTCATCTTGCTGCATTTCGAGCTCGT
TTCTACATGGAG--

>Sbicolor_Sb09g000530
ATGGTGAGGAAGAAAAGAACTGGTCCAGGAGAGAGTTCTGGGGAGGCTTCTGGAGCGCCT
GGGCAGGGCTCCTCACAGCGTCCTCAGGCAACTCAACAGGGTGCCCGTGGTGGAGGGCAA
CACCAGGGCCGTGGTGGATATCAGGGCCGTGGAGCGCCGCCTTCACAGCACCCAGGTGGT
GGGCTGACTGAGTATCAACCGCGCGACTACCAGGGACGCGGTGGAtatcagggccgtggc
GGTCCACCTTCACAGGTTCCTGGTGGTGGGCCGCCTGAGCCTCAGCCGCGTGAGCCTCAG
CCGCGTGCCTACCAGGGACACGGTGGATACCAGGGCCGTGGCGGGCCACCTTCACAGCAT
CCTGGTGGTGGGCCACCACCTGGGTCTCAACCACGTGACTATCAGGGACGTGGTGGTCCG
CGTCCCAGAGGGGGAATGCCGCAGCCACACCGTGGCGGGCATGTGGGAGGTAGTGTTGGA
CCAAGTGTTCCTTCAGGTCCATCTAGACCAGTTCCCGAGCTGCACCAAGCCCCAGATGTC
CAACATCAAGCCCCTGTGGTGGCAACACCATCACCACAAGGAGCTGGCTCGTCCTCGCAG
CCTAGGAAGGCCGAGGTGAGCTGTCCTGTTGGTAGATCATTTTATTCTCCCAACTTAGGG
AGACGTCAGCAACTTGGTGAAGGTTTGGAAACTTGGCGTGGTTTCTACCAAAGCATAAGG
CCCACACAGATGGGTCTTTCTCTGAATATTGATATGTCCTCTACTGCATTTATTGAGCCC
CTCCCAGTGACTGAATTTGTTGCTCAGCTTCTTAACAGAGATATATCAGTTAGACCATTG
TCTGATTCTGATCGTGTGAAGATTAAAAAAGCCCTACGAGGTGTGAAAGTCGAGGTCACA
CACCGTGGAAACATGCGTAGGAAATATCGGATATCTGGCCTCACTTCACAAGCAACAAGG
GAGTTATCATTCCCTATTGATGATCGTGGTACTGTTAAGACTGTGGTGCAATACTTCCTG
GAGACTTATGGCTTCAGTATTCAGCACACCACTTTACCTTGCTTGCAAGTGGGCAATCAG
CAAAGACCAAATTATTTGCCTATGGAGGTCTGTAAGATAGTTGAGGGACAGCGTTACTCA
AAACGGCTTAATGAGAAACAGATCACTGCTCTACTGAAGGTGACTTGCCAGCGTTTGCTG
ATTGTAATACTGCCTGACAATAATGGTTCTCTTTACGGGGATCTCAAAAGGATCTGTGAG
ACTGATCTTGGATTGGTCTCCCAATGCTGTCTGACTAAACATGTTTTCAAGGCGAACAAG
CAGCAGTATCTTGCAAATGTTGCCCTGAAAATAAATGTGAAGGTTGGGGGACGGAATACG
GTACTTGTTGATGCTTTGACAAGGAGAATTCCCCTTGTCAGTGATGTACCAACTATTATC
TTTGGTGCTGATGTGACCCATCCCCATCCTGGGGAAGATTCTAGTCCTTCCATTGCAGCT
GTTGTTGCTTCTCAAGACTGGCCTGAGGTTACCAAGTATGCAGGATTAGTGAGTGCTCAA
ACCCATCGCCAAGAATTGATACAGGATCTTTTCAAAGTATATCAAGATCCCCAAAGGGGA
TCTGTCTCTGGTGGCATGGTCAGGGAACTTCTCATTTCTTTCTGGAGGTCAACTAAACAG
AAACCAAAAAGGATCATATTCTACAGGGATGGTGTCAGTGAGGGACAGTTCTACCAAGTT
CTGTTGCATGAACTTGATGCCATTAGAAAGGCCTGTGCATCATTGGAGTCCGATTACCAG
CCTCCAGTTACGTTTGTTGTGGTCCAGAAGCGTCATCACACTAGGTTGTTTGCTAATAAT
CACAATGACCAACGTGCTGTTGATAAAAGTGGAAACATACTGCCTGGTACTGTGGTGGAC
TCAAAGATCTGCCATCCAACTGAATTTGATTTCTACCTCTGTAGCCATGCTGGCATTCAG
GGAACAAGCCGCCCTGCCCATTATCATGTCCTGTGGGATGAGAACAAATTTACCGCGGAT

GGGTTGCAAACTCTCACCAACAACCTGTGTTACACGTATGCTAGGTGCACTCGCTCAGTA
TCAATCGTTCCTCCTGCATATTATGCTCACCTGGCAGCCTTCCGAGCTCGCTTCTACATG
GAG---

>GRMZM2G059033_T01 cds:PUTATIVE_protein_coding

GTAACTCTTTCTCGATCCTTCTTCTCCAAGAAGTTCGGTGATGACGACATTGGCGGTGGG
CTAGAGTGCTGGAGAGGATATTACCAGAGCTTGCGTCCAACTCAAATGGGCCTCTCATTG
AACATTGATACATGCTCGACTTCATTTTACCAACCTATCGATGTGGTAAAATTTGTTGCC
GATTGTCTCCAGCTGACAAACCCTGGCCAACCTTTTTTGGACAGGGATCGTTTAAAGCTT
AAGAGAGCCCTGCGCGGAGTTCTTGTTGAGACTGAACACCAGCAGGGAAAGAGAAGCATC
TACAGGATAACTGGGATTACTTCTGTTCCATTGGCTCAACTGAGCTTTTCTTGTAACGAA
GGCCCTCAGCTGACTGTTGTTGAGTACTTTGCACAACGGTACAATGTCCAGCTGCGCTAC
ACTGCTTGGCCCTGCCTGCAGTCGGGCAATGATTCTAAGCCGATATATTTACCAATGGAG
GTGTGCAAAATCATTGAAGGGCAGAAGTACCCTAGGAAGCTCAGCGACACACAGGTGGCC
AACATACTGAAGGCAACCTGTAAACGTCTGCTTATTGTGATTCTTCCAGATGTTACTGGT
TATTATGGGGAAATTAAGAGGATGTGCGAGACTGACCTTGGTATAGTATCTCAGTGCATC
AATCCGAAGAAGAATAGAAACAAGCAGTATTTTGAAAATCTTGCCCTTAAAATCAATGTG
AAGGCTGGAGGGCGCAATACAGTGCTTGAGAGAGCCTCTGTGCCTAATGGGATACCTTTT
GTCTCAGATGTGCCAACAATCATTTTTGGTGCTGATGTTACCCATCCTACAGCAGGAGAA
GAATCCTCGGCTTCTGTTGGAGCTGTGGTTGCATCCATGGACTGGCCACAGGTCACAACA
TATAAAGCTCTGGTCTCGGCACAAGCACACAGGGAAGAGATTATACAAAATCTCGGCGGA
ATGATAAGGGAGTTGCTGATTTCGTTCTATAAGAGGACTGGCAAAAAGCCCAAAAGGATT
ATATTTTACAGGGATGGAATAAGTGAAGGACAATTCAACCATGTTTTGCTCCTTGAAATG
GACGCGATAAGGAAGGCTTGTGCCTCTCTGGAAGATGGGTATCTACCCCCAGTGACATTT
GTCGTAATACAGAAAAGGCACCACACAAGGCTCTTCCCTGGAGTTCATGGAAGGAGAGAT
GTTACTGACAGAAGTGGGAACATTCTTCCTGGAACTGTGGTTGATACCGAGATTTGTCAT
CCGCGGGAGTTTGATTTCTACCTTTGTAGCCATGCTGGAATTCAGGGAACCAGCAGGCCA
ATACACTATCATGTCCTCTACGATGAAAACCGTTTCTCGGCTGATGGGCTGCAGATACTC
ACAAACAGCCTGTGCTACACATACGCACGATGCACGCGCGCTGTCTCAGTTGTTCCACCA
GCCTACTACGCTCACCTGGCAGCATTCCGCGGGAGGTACTACGACGAA-----------

>AGO1907_Aquilegia
CTGCTGATTGTTATTTTACCCGACAATAATGGTTCTCTTTATGGTGAAGTGAAGCGAATC
TGTGAGACACAACTTGGGATAATCTCACAGTGCATCTTAGCTAGAACTGTTGAAAAAATG
AATGTGCATACTTTGGCTAACATTGTTCTAAAGATAAACACCAAGGTTGGGGGGATAAAC
GTTGTGCTTAGGGATCCTATACCAATGGTCAGTGATAGGCCAACAATAATTTTTGGGGCT
GATGTAACTCATCCTAATCCTGGGGAAAGTGGGAGCCCTTCGATAGCAGCAGTTGTGGCT
TCTCAAAACTGGCCAAATGTCACAAATTATATTCCTATACTGTCAGCACAACTTGGTCGT
GAGGAAAAGATCCTAGATCTGGAAAGAATGGCCAAGGAACATTTTCACGCTTTTGAGAAG
AACAATCAGCGGAGGCCTGAAAGGATCATATTCTACAGGGATGGTGTTAGTGACGGACAA
TTTAATCAAGTCCGCGAGTATGAGCTAGAAGCAATTCGGGAGGCATGGAGGAATGAATTC
aagGATTCTGTTGTGCCTCCTATAACATTTGTGGTGGTCCAAAAACGACATCATACCAAG
TTATTTCCCTGGAACCATGATGATTTCTCGTCTGTAGACAGGAGCGGAAATATATATCCT
GGAACTGTGGTGGATTCAGATATCTGCCACCCAACACAATTCAACTTCTACTTGTGTAGC
CATGCTGGTATTCAGGCCACGAGCCGTCCTGCTCATTACCATGTTCTATGTGATGATAAT
CAGTTCACGGCAGATCAACTTCAAACCCTCACCAATAACCTGTGTTACATATATGCAAGG
TGCACACGTTCGGTCTCCTATGTGTCACCAGCATATTATGCTCATCTGGCTGCTTTTCGT
GCACGATTCTACTTTGAG--

>Sbicolor_Sb03g011020
CTATTAGTAAGGCAATCATTCTTCCACAATAATCCTTCTAACTTTGTTGACCTGGGTGGT
GGTGTAGTGGGCTGTAGAGGGTTTCATTCTAGTTTTCGTGCAACCCAGAGTGGACTTTCA
CTCAACATCGATGTGTCCACCACAATGATAGTGAAACCTGGTCCTGTCATTGATTTTCTG
CTTGCCAATCAGAAAGTTAATCATCCAGGCATGATTGATTGGGCTAAGGCCAAGCGTTCA
CTGAAGAACTTGAGGATCAAAACAAGTCCAGCAAACCAAGAACAGAAGATTGTTGGTCTC
AGCGACAGACCTTGCTGTGAGCAATTATTCACACTGAAACATAAGAATGGTAATGGAGAC
TCTGAAGAGATCACTGTTTATGATTACTTCGTAAAGAACCGTGGCATAGAGCTGCAATAC
TCTGGTGATCTTCCATGTATCAATGTGGGAAAACCAAAGCGGCCAACATATTTTCCAGTT
GAGTTATGCAGTCTTTTGCCTTTACAAAGGTACACTAAAGCTTTGAGCACACTTCAGAGG
TCATCACTTGTTGAGAAATCTAGGCAGAAATTTCTTTTGTGTGTTCTAGCTGAAAGAAAG
AATTCTGATATTTATGGGCCTTGGAAGAAGAAATGCCTTGCTGAATTTGGGATCGTTACA
CAGTGCGTGGCACCAACTAGAGTCAATGATCAGTATCTTACAAATGTCCTACTAAAGATA
AATGCAAAGCTGGGTGGCATGAATTCGTTGCTCCAAATTGAAACATCCCCAGCAATTCCT
CAAGTATCCAAGGTCCCAACTATAATCTTGGGAATGGATGTCTCCCATGGTTCTCCTGGA
CATTCTGATGTACCGTCCATTGCTGCTGTTGTTAGTTCTCGTGAATGGCCTCTTATCTCG
AAATATAGAGCTTCTGTCCGCACCCAATCACCTAAGATGGAAATGATTGACTCTTTGTTT
AAGCCACGGGAAACTGAAGATGATGGTCTGATCCGGGAGTGTCTGATTGACTTCTACACC
AGTTCTGGGAAGAAGAAGCCTGACCAAGTCATCATCTTCAGGGATGGTGTTAGTGAAAGT
CAGTTTAATCAGGTGCTGAACATTGAGTTGCAACAAATCATCGAGGCTTGCAAGTTTTTG
GATGAAAAATGGAATCCAAAGTTCACGTTGATTATTGCCCAGAAGAATCACCACACTAAA
TTTTTTATTCCTGGAAAGACAGAAAATGTTCCAGCTGGAACTGTTGTGGACAACAAAGTT
TGTCATCCAAGGAACTTTGACTTCTACATGTGTTCACATGCTGGAATGATCGGGACTACG
AGGCCAACTCATTATCATATACTGCATGATGAGATAGGCTTCAATCCTGATGATCTGCAG
GAGCTGGTGCATTCGCTCTCTTACGTGTACCAAAGGAGCACAACAGCCATATCAGTTGTT
GCTCCCATCTGCTATGCACATCTGGCAGCAGCTCAGGTCGGCCAGTTCATTAAG------

>Gmax_Glyma06g47230
CTCCTTGTTCGCCAATCCTTCTTTCATGACAATCGAAGAACCTGTACTGATATAGGAGGT
GGTGTGCAAGGGTGCCGCGGTTTTCATTCAAGCTTTCGAGTCACACAAGGAGGTTTATCT
CTCAACATGGATGTGACAACCACAATGATTGTAAAGCCTGGCCCTGTGGTGGACTTCCTT
CTCCAGAATCAGAGTGTTCAGAACCCAAACTACATTGATTGGACCAAGGCAAAGAGAATG
CTGAAGAACCTAAGAATCAGGGCTAATGGTGTTGAGTTCAAAATCTCTGGATTGAGTGAT
AACACTTGCAGGAATCAGAAATTTCTTTTAAGGCAAAAGGGTACGAATGGTGAAGTGCAG
GAAAGGGAAATCACAGTTCATGACTACTTTACACGCCAAAAACTGATTGGCCTGAACTAT
TCTGCTGACATGCCATGCATCAATGTTGGCAAACCAAAACGCCCGTCTTATTTTCCAATA
GAGCTATGTGAAATGGTCTCGTTGCAGCGTTACACCAAGGCTCTAACAAATTTGCAAAGG
GCTCAGCTAGTGGAGAAGACAAGGCAAAAGTTTCTGTTGTGTATTCTTCCAGAGAAAAAG

AATTCTGACATTTATGGTCCTTGGAAGAAGAAAAGTCTTGTTGAAGAAGGGATTGTAACA
CAATGTATTGCACCAACAAAGATTAATGATCAATACATTACTAATGTACTTTTGAAAATT
AATGCAAAGTATGGTGGGATGAATTCGTATCTGTCAGTTGAGCTTTGTAATTCCATTCCA
TTTGTTTCAGCTGTACCAACCTTGATTCTTGGTATGGACGTTTCTCATGGATCTCCTGGT
CGATCAGATGTGCCCTCTATTGCTGCGGTTGTGAGCTCAAGATGTTGGCCTCAAATTTCA
CGTTATAGAGCTTCAGTTCGCACACAATCATCAAAAGTTGAGATGATTCAATCTCTATTC
AAGCCTGTGGCTAATACTAATAAGGATGAAGGTATCATCAGGGAGGTGCTGTTGGACTTT
GAAATTACTTCATTCAAAAGAAAGCCTCAACAAATTATAATTTTCAGGGATGGAGTGAGT
GAATCACAGTTCAACCAGGTGCTCAATATTGAGTTAAGTCAAATCATTGAGGCATGCAAA
CACCTTGATGAAAAATGGGATCCAAAGTTCACTTTGATTATTGCCCAGAAGAATCATCAT
ACTAGGTTCTTTCAGGCCAATGCTagagatCAAACTAATGTTCCACCAGGAACCGTTATT
GACAACACTGTCTGTCATCCCAAAAACAATGACTTCTACTTGTGTGCTCAAGCTGGGATG
ATTGGGACAACTCGACCTACTCACTACCATGTTTTACACGATGAAATCGGATTCTCAGCT
GATGAAGTGCAAGAATTAGTGCATTCATTGTCTTATACGTATCAGAGGAGCACAACTGCT
GTATCATTAGTCGCTCCCATTTGCTATGCCCACTTGGCAGCAGCCCAGATGGCACAATTT
ATGAAG--

>AGO1904_Aquilegia
CTTCTGATTGTTATATTACCTGATAACAACGGCCCTTTGTATGGTGAGCTGAAGCGAATA
TGTGAGACAGACCTAGGACTTGTATCACAGTGTTGTCTGACCAAACATGTGTTTAAGATG
AATAAGCAGTATCTGGCAAACGTAGCCCTAAAGATAAATGTAAAGGTTGGGGGGCGAAAC
ACAGTGCTCCTTGATGCGCTTTCTAGACGCATGCCTGTGGTCAGTGATGAACCAACAATC
ATCTTTGGTGCTGACGTAACCCATCCCCATCCTGGAGAGGATTCGAGCCCCTCTATCGCA
GCTGTTGTTGCATCCCAAGATTGGCCTGAGGTCACAAAGTATGCTGGTTTGGTTTGTGCT
CAAGCTCATCGCCAGGAATTGATCCAAGATCTGTATAAAGTCTGGAACGATCCGCAAAAG
GGTACAATGACAGGAGGCATGATCAAGGATCTTTTGAGATCTTTTCATAAAGCTACTGGA
CGAAAACCTTCACGAATCATATTCTACAGGGATGGAGTCAGTGAAGGACAATTTTATCAA
GTCTTGCTATATGAACTGGATGCGATTCGTAAGGCATGTGCTTCTCTGGAGCCAAATTAT
CAGCCCAGGGTGACATTTGTCGTTGTTCAAAAGCGCCATCATACAAGGTTGTTTGCTAAT
AATCATGCTGACCGGCGCACAATTGATAGGAGCGGAAATATATTACCAGGTACGGTGGTG
GACTCCAAGATTTGCCATCCAACTGAATTTGACTTTTATTTGTGTAGCCATGCTGGAATA
CAGGGCACAAGCCGTCCTGCCCATTATCATGTATTATGGGATGAGAACAACTTTACTGCC
GATGGACTCCAGTCACTCACGAATAACCTCTGTTATACGTATGCAAGGTGCACCCGTTCT
GTTTCTATAGTCCCCCCAGCATACTACGCTCATTTGGCAGCTTTCCGTGCACGTTTTTAC
ATGGAG--

>Sbicolor_Sb10g023230
GTGTCCATAGGGCGGTCCTTCTACTCGCCGGACATCAGGAGGCCGCAGCGGCTAGGCGAC
GGCCTGCAGTCGTGGTGTGGGTTCTACCAGAGCATCCGGCCGACACAGATGGGATTGTCG
CTTAACATCGACATGTCGTCCACGGCGTTCATTGAACCCCTGCCGGTGATCGAGTTCGTG
GCCCAGATATTAGGAAAGGATGTCATATCAAGGCCATTATCCGATGCTAACCGAATCAAG
ATCAAGAAGGCATTGCGAGGAGTAAAAGTTGAGGTCACTCACCGGGGGAATGTAAGGCGC
AAGTATCGCATTTCTGGGTTGACAACGCAACCAACTCATGAACTGATTTTCCCAATTGAT
GAGCAAATGAATATGAAATCTGTCGTGGAGTACTTCAAGGAAATGTATGGTTTCACCATT
CAGCATCCTCATCTTCCCTGCCTTCAGGTTGGGAACCAAAAGAAGGCGAACTATCTACCG
ATGGAGGCATGCAAGATCGTTGAAGGCCAGAGATACACGAAGAGGCTGAATGAAAAACAG
ATCACATCATTGCTGAAGGTTACATGCCAAAGGCTTCTTTTGGCTATCCTCCCTGACAAC
AATGGTCCGTTATATGGTGACATCAAACGTATTTGTGAAACTGATTTGGGATTGATAACA
CAATGTTGCTTGACCAAACATGTTTTTAAGATCAGCAAGCAGTACTTGGCAAATGTCTCA
CTGAAAATTAATGTTAAGATGGGAGGAAGAAATACTGTGCTCCTGGATGCAATAAGTTGG
AGGATTCCTTTGGTCAGTGACATACCAACTATTATATTTGGTGCGGATGTAACACATCCT
GAAACTGGGGAGGACTCAAGTCCATCAATTGCTGCCGTTGTTGCTTCTCAAGATTGGCCA
GAAGTTACAAAGTATGCTGGATTGGTTTGTGCTCAGGCACACCGGCAAGAGCTCATTCAG
GACCTTTACAAAACATGGCATGATCCTCAGAGAGGCACTGTAACAGGCGGCATGATCAGG
GAGCTCTTAATATCCTTCAGGAAGGCCACTGGGCAGAAGCCATTGAGAATAATATTCTAC
AGGGACGGTGTTAGTGAAGGCCAGTTCTACCAAGTTCTCCTTTACGAGTTAGATGCCATC
CGTAAGGCATGTGCGTCCCTAGAACCAAATTACCAGCCTCCTGTAACATTTGTGGTAGTT
CAAAAACGTCATCATACAAGACTTTTTGCAAATAATCACAAAGACAGAAGTAGCATGGAC
AAGAGTGGAAATATTTTGCCAGGAACCGTTGTTGATTCTAAGATATGCCACCCAACGGAG
TTTGATTTCTACCTCTGCAGTCATGCTGGAATTCAGGGAACAAGTAGGCCTGCTCACTAC
CATGTCCTCTGGGATGAGAACAATTTCACAGCAGACGAAATGCAGACATTGACAAACAAC
CTTTGCTACACTTATGCCCGGTGCACACGCTCGGTTTCTGTTGTCCCTCCTGCATACTAC
GCACACCTGGCAGCATTCCGGGCTCGGTTCTACATGGAA---------------------

>Mguttatus_mgf016487m
CTTCTAGTACGACAGTCCTTTTTCCATAACGAGCCAAGAAATTTCGTGGACTTGGGGGGC
GGTGTTGTTGGTTGTCGGGGTTTTCATTCGAGCTTTAGGGCAACACAAGGAGGGCTTTCG
TTGAATATGGATGTTTCGACAACTATGATTGTCAAGCCAGGGCCTGTGCTGGACTTTCTC
CTGGCTAATCAACGAGTCGACAATCCTGGTCAGATTGATTGGAGCAAGGCGAAAAGGACG
TTGAAGAATCTTAGGATCAGGGCCACTAATTCGAACTTGGAGTACAAGATCAGTGGACTG
AGCGACTCGATATGCAGGAAGCAAACGTTTCCTCTAAAACAGAGAAAAGAAGGTgcaAAC
GAAGGAGACGAAGTACAAATCACAGTCTACGACTACTTCGTGAACCACCGAGATATCAAA
CTGAGATACTCTGGGGATTTTCCATGTATCAATGCAGGCAAACCAAAGCGCCCCACTTAT
ATCCCCATTGAGCTATGTGAATTAGTTTCTTTGCAACGATATACCAAATCGTTGTCGAAC
CTGCAACGAGCTTCGCTTGTGGAAAAATCTAGACAGAAGCTTCTTCTTTGCATATTGCCG
GAGAAGAAAAACTGCGATCTCTATGGTCCATGGAAGAAGAAGAATCTTTCTGACATGGGG
ATAGTCACTCAATGCATGGCACCGACGAAACCTGTCAATGATCAGTACACCACCAATCTG
CTCCTAAAGATCAATGCCAAGgaaattTTCGTTCTTGGCGGCATTAATTCACTTTTGGGT
ATCGAAAAATCGCCCTCGATTCCCCTGGTTTCCACAGTGCCCACCCTTATTGTCGGGATG
GACGTTTCACATGGTTCGCCTGGTCGATCAGATGTTCCATCTATTGCTGCTGTGGTAAGT
TCAAGGCAGTGGCCTTTGATATCTAAGTACAGGGCTGCAGTTCGAACCCAGTCACCAAAA
CTAGAAATGATCGATTCGTTGTTCAAGAAAGTGGATGATGTGGATCAGGGCATTTTCAGG
GAGTTATTGCAGGACTTCTTCATAAGTTCCGGCAAAAGAAAACCCGAGCAGATTATCATC
TTTAGGGATGGAGTGAGCGAGTCGCAGTTCAATCAAGTATTGAATATAGAGCTGGAACAA
ATCATCGAGGCATGCAAATTTCTCGACGAAACTTGGTCTCCCAAATTCATGGTTGTGGTG

GCACAAAAGAACCATCACACGAAATTTTTCCAAGCAAACGGACCCGATAATGTTCCACCA
GGCACTGTAATTGACAATGGAATTTGCCATCCAAGAACAAACGACTTCTACATGTGTGCT
CACGCTGGAATGATTGGCACAACTAGGCCAACACACTACCATGTGCTATTTGACGAGCTC
GGCTTCTCTGCCGATGCTTTACAAGAACTTGTGCACTCATTATCCTATGTGTACCAGAGA
AGTACTACAGCCATCTCTGTCGTTGCTCCGATCTGCTACGCCCACTTGGCCGCTGCACAA
ATGTCTCAATTCATAAAG--

>LOC_Os01g16850
TGCCCATCGTACCCCTCAGCAatggacggctggatgaaaactggggtaccgaaaggaGGA
CGGGAGAAAATCTCTTGTCGGGGATTTCACTCCAGCTTTCGACCCACTGACAGTGGCCTG
TCACTGAATGTTGATGTATCCACGACGATGATCGTCAGACCTagagcaagggcacgggag
cgagcagagcggtgggcagcagcccacctccacggcggcggcggcggcccaagggaccgc
gcgcccgccgacgggaacccagacgggctcgggcgtgccggcggcagcccggagcccgca
cgtggccctcgcaggcagcacggctctctcccctgaccggcaccatggtcaccacggc
gtccagacagtggacgccTGCCGCAACTGTGGCAAGCTTggccattgggccaaagactgt
cgacagcgagccaaggagcggctcgagctcgtgcacggggacttgtgtggcccagtgaca
ccggccacaccaggaggacgacgttacttcctactgctcgtcgacgacctctcctgctac
atgtgggtgatggtcctcggcagcaagggagaggctgcggacgccatcaggcatgcgcag
gctgctgcagaggcagagtgcggccgcaagctgcacgtgctgcgcaccgacaacggcggt
gaattcacagcggctgaattcgcgtcgtactgcgctgatgagggcattcagcgacactac
accacgccttacagcccgcaacagaacgacgtcgtcgagcggcgcaaccagacggttgtg
gggatggctgcgtcgtactgcgctgatgagggcattcagcgacactacaccacgccttac
agcccgcaacagaacgacgtcgtcgagcggcgcaaccagacggttgtggggatggctcgg
gccctcctcaagcagagggggatgccggccatcttctgggggggaggcggtggtgacggcc
gcctacatcctcaaccgctcgcctaccaaggccctcgatggcaggacaccgtacgaggct
tggcatgggcgcaagccggcggtctcccacctgcgggtcttcggctgcctcgcgttcgcc
aaggagcttggccacatcggcaagctcgacgacaggagcaccccaggggtgttcatcggc
tacgcggagggctcgaaggcctaccgcatcctcgacccggagacacagcgtgtgcgcact
gcgcgcgacgttgtgtttgatgaagggcgagggtgggtatgggacaaggcggtggacgat
ggttcgactccgacgtacgacgacttcaccgtcgagtacgtccacttcgagggagctggg
ggagtaggcagctcttcttcacctagcgtgtctaccccagcccccaaatctccaccgact
ccaacaccaacacaccctcgggccacgacttcgactacgacgagctcttcgtcgactcca
catgctccagcaccaacagccactcctccaagcacgtctactccgacgccagctcgtgtt
gagcgcagcccggtggagttcgctactccgctctcccacgacggggagcgcatcgacgtg
taccacgacggcgagcagctatggtaccgtacgatggaggatcttctcggcgaccagccg
gtgccgggactggtgcctcgcgacctagaggcgcagttgcaccttgcgtgcgatgacggt
gagcctcggtcttttgcagaggccgagaaacacgcggcatggcgtgccgcgatgcagtcg
gagatggacgcggttcaggagaaccgcacctgggagcttgctgacctccctcgtggtcac
cgcgcgatcacccttaagtgggtgttcagcccgcaacagaacgacgtcgtcgagcggcgc
aaccagacggttgtggggatggctcgggccctcctcaagcagagggggatgccggccatc
ttctgggggggaggcggtggtgacggccgcctacatcctcaaccgctcgcctaccaaggcc
ctcgatggcaggacaccgtacgaggcttggcatgggcgcaagccggcggtctcccacctg
cgggtcttcggctgcctcgcgttcgccaaggagcttggccacatcggcaagctcgacgac
aggagcaccccaggggtgttcatcggctacgcggagggctcgaaggcctaccgcatcctc
gacccggagacacagcgtgtgcgcactgcgcgcgacgttgtgtttgatgaagggcgaggg
tgggtatgggacaaggcggtggacgatggttcgactccgacgtacgacgacttcaccgtc
gagtacgtccacttcgagggagctgggggagtaggcagctcttcttcacctagcgtgtct
accccagcccccaaatctccaccgactccaacaccaacacaccctcgggccacgacttcg
actacgacgagctcttcgtcgactccacatgctccagcaccaacagccactcctccaagc
acgtctactccgacgccagctcgtgttgagcgcagcccggtggagttcgctactccgctc

tcccacgacggggagcgcatcgacgtgtaccacgacggcgagcagctatggtaccgtacg
atggaggatcttctcggcgaccagccggtgccgggactggtgcctcgcgacctagaggcg
cagttgcaccttgcgtgcgggtgggtatgggacaaggcggtggacgatggttcgactccg
acgtacgacgacttcaccgtcgagtacgtccacttcgagggagctgggggagtaggcagc
tcttcttcacctagcgtgtctaccccagcccccaaatctccaccgactccaacaccaaca
caccctcgggccacgacttcgactacgacgagctcttcgtcgactccacatgctccagca
ccaacagccactcctccaagcacgtctactccgacgccagctcgtgttgagcgcagcccg
gtggagttcgctactccgctctcccacgacggggagcgcatcgacgtgtaccacgacggc
gagcagctatggtaccgtacgatggaggatcttctcggcgaccagccggtgccgggactg
gtgcctcgcgacctagaggcgcagttgtacgtccacttcgagggagctgggggagtaggc
agctcttcttcacctagcgtgtctaccccagcccccaaatctccaccgactccaacacca
acacaccctcgggccacgacttcgactacgacgagctcttcgtcgactccacatgctcca
gcaccaacagccactcctccaagcacgtctactccgacgccagctcgtgttgagcgcagc
ccggtggagttcgctactccgaaccgcacctgggagcttgctgacctccctcgtggtcac
cgcgcgatcacccttaagtgggtgttcaagctgaagagggatgaagccggagccatcgtc
aagcacaaggctcgcttggtggcacgcggtttcgtgcagcaggaggggatcgactacgac
gatgccttcgctcccgtggcacggatggagtccgtgcgactccttcttgcgctggctgct
caggaaggctggggcgtccatcacatggacgtcaagtcggcgtttctaaacggcgacttg
aaggaggaggtctacgtgcaccagccgccgggatttgtgatccccggcaaggagggcaag
gtgctacgcctgcacaaggccctctacggcttgcggcaggcatcgagggcgtggaatgcc
aagttggattctacgctcaaggggatgggcttcgagcaaagcccgcacgaggcggccatc
taccggcggggcaatggaggaaatgccttgctggtgggtgtctacgtcgacgacttggtg
atcaccggcaccaaggatgcggaggtcgcggcgttcaaggaggagatgaaggccaccttc
caaatgagtgatctggggcctctctccttctacctggggattgaagtgcaccaggacaac
tccgggatcacgcttcgacagaccgcctacgccaagcgcgtcgttgagctggctgggctc
accgattgcaacccagctctcactccgatggaggagagatatatgtatccaacctccctt
ggaCAGGCCAAGTGTGCACTGAAGAACCTGAGGATAAAAACGACTCACACCGGCTCTGAA
TTTAGGATCATCGGTTTGTCTGAAGACACTTGCTATTCGCAGACGTTCCAAATAAAGAGA
AAAAATGGCAACGGTGGCTCGGATACAGTGGAAGAAGTGACAGTCTTTGAATACTACAGG
AAGAATTGGAAAATAGATTTGAAGGGATCTGCTCACTTTCCCTGTCTAAATGTTGGGAAG
CCAAAGCGGCCAACATATATCCCATTGGAGCTTTGCCATTTGGTGCCATTGCAAAGGTAC
AAAAAGGCTTTGTCGACGTTACAGCGGTCCACGTTGGTTGAGAGATCAAGGCAGAATgcc
TTTTCTGCAGATTCCGGACCATGGAAGCGTATGTGTCTCGTCAAATACGGTATTGTAACA
CAATGCTTGGCTCCTACCAAGATCAACGATCAGTACCTGACTAATGTGCTTCTAAAGATA
AATGCAAAGCTTGGAGGGTTGAATTCGCTGCTGCAAATTGAAAGAAACCAAGCCATTCCT
CTCTTGTCGAAGACTCCAACCATTATCTTAGGCATGGATGTTTCCCATGGCTCACCGGGA
CGGGATGATGTACCGTCTGTCGCTGCGGTTGTTAGTTCCCTGGAGTGGCCTCTCATATCA
AAATATAAAGCCTCCGTATGCACCCAGTCTCCCAGGCTAGAAATGATCGATTCCTTGTTT
AAGCTAGTGGGGAATGAGGATCATGTTATCATTAGAGATGGGGTTAGTGAAGGCCAGTTT
AATCAGGTGCTGAACATTGAGCTAGCCCAGATAATCAAGGCATGCGAGTTTCTTgccaat
gagaaaaatGACAGTGAATGGTCTCCAAAGTTCACGGTGATAGTTGCGCAGAAGAACCAT
CACACCAAATTTTTTCAGACAGATCGATCGAACaaagttgtcAATGTTCCTCCTGGTACT
GTTGTTGACAAAGGAATCTGTCATCCCAGGAACTGTGATTTCTACATGTGTGCTCATGCT
GGGATGATTGGGACTACAAGGCCGACGCATTACCATGTGCTGCATGATGAGAACAATTTC
ACCCCTGATGACTTGCAGGAGCTTGTGCACAACCTCTCATACGTGTACCAGAGGAGCACG
ACGGCCATCTCAGGTGTCGCTCCGATCTGCTACGCGCACCTGGCGGCGGCGCAGGTGTCG
CAGTTCGTGAGA
>Smoellindorffii_15405134_locus
TCGCCTGTTGGACGATCGTTTTATTCACCAGACTTAGGACGTAGACAACCCCTCGGTGAC
GGCTTAGAAAGCTGGCGAGGGTTCTACCAGAGTATAAGGCCCACGCAAATGGGTCTTTCT
CTGAATATTGACATGTCATCTACTGCTTTCATTGAGCCGCTGCCCGTGGTGGACTTTGTT
GGCAAACTCCTTAACAAGGACATAAGCAGGCCACTTTCGGACGCTGACCGCATCAAGATC
AAAAAAGCTCTGAGAGGGGTCAAAGTGGAAGTTACACATCGCGGAACCATGCGACGAAAA
TACAGGATTTCTGGCTTAACTTCTCAGCCTACTCAAGAACTAATGTTTCCTGTTGATGAT
AGAGGTACAATGAAATCGGTAATGGAGTATTTCCGAGATACATATCACTACACTATACGA
AGCCCATCTTTACCTTGTTTGCAAGTTGGAAATCAAGAAAGACCAAATTATCTGCCAATG

GAGGTCTGCAAGATTGTGGAAGGACAAAGGTATACTAAACGTCTCAACGAGCGTCAAGTG
ACAGCCCTTCTAAAAGTAACATGCCAACGGTTACTAATTGCCATATTACCCGACAACAAT
GGCTCACTTTATGGCGACTTGAAACGAATCTGCGAAACAGACCTGGGATTGGTTTCTCAA
TGCTGCTTGACAAAGCATGTCTTTAAGAGAGGAAAGCAGTATCTTGCTAATGTTGCGTTG
AAAATCAATGTCAAGGTCGGAGGCAGAAACACTGTCTTGGTGGATGCACTATCACGGAGG
CTTCCTTTAGTAAGTGATACACCAACAATTATATTTGGAGCGGATGTTACGCATCCTCAT
CCTGGAGAAGATTCGAGTCCCTCGATTGCAGCGGTTGTCGCTTCTCAGGATTGGCCAGAA
GTAACGAAATACGCAGGTCTAGTCTGCGCTCAAGCTCACAGACAAGAGTTGATCCAAGAT
TTGTATAAAACATGGGTTGATCCTCAGAAGGGAACCATGAATGGCGGTATGATAAGAGAG
CTTTTAATTTCTTTCCGAAGTGCCTCTGGATACAAACCTGGAAGAATCATCTTCTACAGA
GATGGTGTGAGCGAAGGACAGTTCTACCAAGTTCTCCTTCACGAGCTGGATGCAATTAGA
AAGGCTTGCGCGTCACTCGAGCCGAACTATCAGCCTCTCGTGACGTTCGTTGTGGTCCAG
AAACGGCACCACACCCGACTATTTGCTAACGATCACGACGATACAAGAACTACTGATAAG
AGTGGAAATATCCTGCCAGGCACTGTGGTGGATTCGAAGATTTGCCATCCAACGGAGTTT
GACTTCTACCTTTGCAGCCATGCCGGCATTCAGGGAACAAGCAGACCAGCTCACTATCAC
GTACTTTGGGACGAGAACAAGTTCACTGCCGATGGCTTGCAGTCCCTCACCAACAGCCTG
TGCTACACTTACGCTCGCTGCACACGCTCAGTCTCGATAGTACCACCTGCATACTACGCT
CACCTGGCTGCGTTCCGCGCGAGATTCTACATGGAG------------------------

>AGO24601_Amborella trichopoda
CAAGTCGGGAATCATCAGCGCCCAAATTACCTCCCAATGGAGGTATGCCAAATCGTTGAG
GGCCAACGATACTCAAAGCGATTGAACGAAAGACAAATCACTGCCCTTCTGAAAGTGACC
TGCCAGCGACTGCTGATTGTAATCTTGCCTGATAATAATGGTTCCCTTTATGGGGATCTA
AAGAGAATTTGCGAGACGGATTTGGGCTTGGTCTCCCAATGCTGCTTAACTAAGCATGTA
TTTAAAATGAGCAAGCAGTATCTTGCTAATGTTGCTTTGAAAATCAATGTTAAGGTCGGT
GGGAGGAATACTGTACTTGTGGATGCGATTGCAAGGAGAATTCCACTTGTAAGTGACGTT
CCAACTATAATATTTGGGGCTGATGTCACTCATCCTCACCCAGGAGAGGATTCCAGCGCA
TCCATTGCTGCTGTCGTTGGTTCTCAGGACTGGCCAGAGGTGACTAAATATGCTGGATTG
GTCTGTGCTCAAGCTCACAGACAAGAATTGATCCAAGATCTTTATAAGGTCACCCAGGAT
CCTCAACGTGGAACTGTGACGGGTGGCATGATCAAGGAGTTGCTCATTTCATTCAGGAGA
GCAACTGGGCAAAAGCCTCAGAGGATTATTTTCTACAGGGATGGAGTTAGCGAGGGCCAG
TTTTACCAGGTTTTGTTTCATGAATTGGATGCTATTCGCAAGGCATGTTCCTCCCTTGAG
CCAAACTATCAGCCCCCTGTGACATTTGTTGTGGTTCAAAAGCGGCATCACACTCGGCTA
TTTGCAAACAATCATAAGGATCATCATCTGACTGACAAGAGTGGAAATATATTACCCGGA
ACTGTGGTGGATTCAAAGATCTGCCATCCCACTGAATTTGATTTTTACTTATGCAGTCAT
GCTGGCATCCAGGGTACTAGCCGCCCTGCCCATTACCATGTTTTGTTTGATGAGAATCAC
TTCACAGCCGATGGCCTCCAGTCGCTCACTAATAATCTGTGCTATACGTATGCACGTTGC
ACCCGCTCTGTCTCTATTGTGCCGCCTGCTTACTATGCTCATCTAGCAGCCTTCAGGGCT
CGCTTCTACATGGAG---

>Contig2618_radiata
CTTCTTGTTGTTATTTTGCCTGATAACAATGGTTCTTTGTATGGTGACCTGAAGCGTATA
TGTGAGACTGACCTTGGTTTGGTTTCCCAGTGCTGTCTGACGAAACATGTCTTTAAAATG
AGCAAACAATATCTGGCAAATGTTGCCCTTAAAATTAATGTGAAGGTTGGTGGAAGAAAC
ACTGTCTTAGTTGACGCACTGTCAAGGAGGATACCTTTGGTCAGCGACAAACCTACAATA
ATCTTTGGAGCAGATGTTACCCATCCACACCCTGGAGAAGATTCGAGCCCGTCAATAGCT
GCTGTTGTAGCGTCTCAAGATTGGCCGGAAGTCACTAAATATGCAGGCTTAGTTTGTGCT
CAGGCACATCGTCAGGAACTCATTCAGGATCTTTACAAAGTCTGGCAGGATCCTGTGCGA
GGGTCTGCAACTGGAGGCATGATAAAGGAATTGTTAATTTCCTTCAGAAGGAATACAGGA
CACAAGCCGGAGCGTATAATATTTTACAGAGATGGAGTAAGCGAGGGTCAGTTTTATCAA
GTTCTATTGTATGAGTTAGATGGCTATTCGAAA---------------------------

>Gmax_Glyma10g38770
TGCCCTATTGGGAGGTCCTTCTTTTCACCTGATATTAGAACACCGCAACGGCTTGGAGAG
GGATTAGAATCATGGTGTGGATTTTACCAGAGTATAAGGCCTACACAAATGGGCCTTTCC
CTTAATATTGATATGGCGTCTGCTGCGTTTATTGAGCCTCTTCCAGTAGTGGAATTTGTT
GGCCAGCTATTAGCAAAAGATGTGCTGTCAAGGCCATTGTCAGATGCTGATCGCATTAAG
ATTAAGAAAGCCCTTAGAGGAGTTAAAGTTGAAGTAACACACAGAGGAAGTGTGAGAAGA
AAATATCGTGTTTCTGGATTGACTTCTCAACCAACCAGAGAACTTGTGTTTCCTGTTGAT
GAGAACTCAACTATGAAATCAGTAGTTGAATACTTCCAAGAGATGTATGGTTTCACTATT
CAATATACTCACCTTCCTTGCCTTCAAGTAGGAAACCAAAAGAAGGCTAACTATTTACCT
ATGGAGGCCTGCAAAATTGTTGAGGGGCAACGTTATACAAAAAGATTGAATGAGAAGCAA
ATTACAGCTCTGTTGAAAGTTACTTGCCAGAGACTTTTGTTAGCAATATTGCCAGACAAT
AACGGGTCTCTCTATGGTGATCTCAAGCGAATTTGTGAAACTGACCTTGGTTTAATTTCA
CAATGCTGTCTGACAAAGCATGTCTTCAAAATCACTAAACAGTACTTGGCTAATGTGTCT
CTGAAGATCAATGTGAAGATGGGAGGTAGAAACACTGTACTTCTTGATGCTGTAAGCAGC
AGAATACCATTGGTTAGTGACATGCCAACCATAATTTTCGGAGCAGATGTAACCCACCCT
GAAAATGGAGAAGAATTGAGCCCTTCAATAGCAGCTGTAGTCGCATCCCAGGACTGGCCC
GAAGTGACAAAATATGCCGGTTTAGTATGTGCTCAAGCTCATAGGCAGGAACTTATACAA
GATTTGTACAAAACTTGGCAAGACCCTGTTCGTGGCACAGTTAGTGGTGGCATGATCCGA
GATTTACTGGTTTCCTTCAGAAAGGCAACAGGACAAAAGCCACTACGAATTATATTTTAC

AGGGATGGTGTAAGTGAAGGACAATTTTACCAAGTTTTACTTTATGAGTTAGATGCAATT
CGGAAGGCATGTGCTTCCTTAGAACCAAACTACCAGCCTCCAGTAACTTTCATAGTTGTG
CAAAAAAGACATCATACCCGGTTATTTGCAAACAACTACAGGGACAGAAGCAGTACAGAT
CGGAGTGGGAATATATTGCCTGGGACTGTTGTTGATACCAAAATCTGCCATCCAACAGAA
TTTGATTTTTATCTCTGCAGCCATGCTGGCATCCAGGGTACTAGTCGGCCAGCTCATTAT
CATGTCCTGTGGGATGAAAACAACTTCACACCTGATGGAATTCAGTCTCTGACAAACAAC
CTTTGTTATACATATGCCAGGTGTACACGCTCAGTATCAGTTGTTCCTCCAGCATATTAT
GCACATTTAGCAGCGTTTCGAGCACGTTTCTATATGGAA--------------------

>Gmax_Glyma20g12070
TTACTTGTACGCCAATCCTTTTTCCACAATAATCCAAATAATTTTGCTGATGTAGGAGGT
GGTGTCCTAGGCTGTAGAGGATTCCACTCAAGCTTTAGAACTACACAGAGTGGCCTGTCT
CTTAACATAGATGTGTCAACTACAATGATAATTTCTCCTGGGCCTGTGGTGGATTTCTTA
ATTTCCAATCAAAATGTGAGAGATCCTTTTCAACTTGACTGGGCTAAGGCCAAAAGGACC
CTAAAAAATCTGAGGATTAAAACTAGCCCATCCAATCAAGAATTCAAAATTTCTGGGCTC
AGTGAACTCCCATGCAGAGAGCAGACTTTTACTTTGAAAGGTAAAGGTGGGGGGGATGGT
GAAgatggtAATGAGGAAATCACTGTATATGATTATTTTGTTAAGGTTCGTAAGATAGAT
CTCCGATACTCTGCTGACCTTCCATGTATCAATGTTGGCAAGCCTAAACGACCAACATTT
TTCCCCATTGAGGTTTGTGAATTGGTATCATTGCAACGATATACAAAAGCTCTGTCCACG
CTTCAAAGGGCTTCATTAGTGGAGAAGTCGAGGCAGAAGTTCCTTCTCTGTTTGCTTCCT
GATCGGAAAAATTGTGATATTTATGGTCCATGGAAAAAGAAGAATCTTGCTGATTTTGGA
ATCATAAATCAGTGTATGTGTCCTTTAAGGGTCAATGACCAGTACCTGACTAATGTTATG
TTGAAGATCAATGCCAAGCTTGGTGGGTTGAATTCATTGTTAGGCGTTGAACATTCTCCT
TCTCTTCCTGTTGTTTCCAAAGCTCCCACCCTCATTCTGGGAATGGACGTGTCACATGGC
TCACCTGGGCAGACTGACATTCCTTCAATTGCTGCGGTGGTCAGCTCTAGACACTGGCCT
CTGATATCAAAGTATAGGGCATGTGTTCGTACGCAATCTGCAAAGATGGAAATGATTGAT
AATTTGTTCAAGCTAGTATCTGAAAAGGAAGATGAAGGCATCATAAGGGAACTTTTGCTT
GATTTCTATACAACTTCTGGGAGGAGAAAACCGGAAAATATAATCATATTCAGGGATGGG
GTTAGTGAGTCACAATTCAATCAAGTTTTGAATATTGAACTCGATCGAATCATTGAGGCT
TGCAAATTTCTCGATGAAAATTGGGAGCCAAAATTTGTGGTAATTGTTGCTCAGAAGAAC
CACCACACTAGATTTTTCCAGCCTGGCTCTCCCGACAATGTCCCACCTGGCaagtgttct
gggtctttacagcatttagggagtTGTGAAAAAGGTGCCCCTTCACCTGCCCTCAATTAC
TGTGGAACTAGTAGGCCTACCCATTATCATGTGCTGCTTGATCAGGTTGGTTTCTCTCCG
GATCAGCTGCAGGAGCTTGTCCATTCATTATCATATGTGTATCAGAGGAGCACTACTGCC
ATTTCTGTTGTTGCTCCAATATGCTATGCGCACTTGGCTGCTACTCAGTTGGGGCAGTTC
ATGAAA--

>GRMZM2G153859_T04 cds:PUTATIVE_protein_coding
CTATTAGTAAGGCAATCATTCTTCCACAACAATCCTTCCAATTTTGTTGACCTGGGTGGT
GGTGTAGTGGGCTGTAGAGGTTTTCATTCTAGTTTTCGAGCAACCCAGAGTGGACTTTCA
CTCAATATCGATGTGTCGACTACAATGATAGTGAAACCTGGTCCTGTCATTGATTTTCTG
CTTGACAATCAGAAAGTTGGTGATCCAAGCATGATTGATTGGGCTAAGGCCAAGCGTGCA
CTGAAGAACTTGAGGATAAAAATAAGTCCAGCGAACCAAGAACAGAAGATTGTTGGTCTC
AGCGAAAGAACTTGTCGTGAGCAATTATTCACACTGAAACATAAAAATGGTAACAATGGT

GACTCTGAAGAGATCACTGTTTATGATTACTTCGTAAAGCAGCGTGGCATAGTGCTGCAA
TACTCTGGTGATCTTCCTTGCATCAATGTGGGAAAACTAAAGCGGCCAACATATTTTCCA
ATTGAGTTATGCAGTCTTGTGCCTTTACAAAGATACACTAAAGCTTTGAACACACTTCAG
AGGTCATCACTCGTGGAGAAATCTAGGCAGAAATTTCTTTTGTGTGTTCTAGCTGAAAGG
AAGAATTCTGATATTTATGGGCCTTGGAAGAAGAAATGCCTTGCTGAATTTGGGATCGTT
ACACAATGTGTGGCACCAACTAGAGTGAACGACCAGTATCTTACAAATGTCCTACTTAAG
ATAAATGCAAAGCTGGGTGGCATGAATTCGTTGCTCCAAATTGAAACATCCCCAGCAATT
CCTCTTGTATCCAAGGTCCCAACTATAATCTTGGGAATGGATGTGTCACACGGTTCTCCT
GGACATTCTGATGTACCATCTATTGCTGCTGTTGTTAGTTCTCGTGAATGGCCTCTTATC
TCGAAATACAGAGCTTCTGTCCGCACCCAATCACCTAAAATGGAAATGATTGACTCATTG
TTTAAGCCACGGGAAGCTGAAGATGATGGTCTGATCCGGGAGTGTCTGATTGACTTCTAC
ACCAGTTCTGGGAAGAGAAAGCCTGACCAAGTTATCATATTCAGGGACGGTGTTAGCGAA
AGTCAGTTTAATCAGGTGCTGAACATTGAGTTGCAACAAATCATCGAGGCTTGCAAATTT
CTTGATGAGAAATGGAATCCCAAGTTCACGTTGATTATTGCCCAGAAGAATCATCACACT
AAATTTTTCATTCCTGGAAAGCCAGATAATGTCCCACCAGGAACTGTGGTGGACAACAAA
GTCTGCCATCCAAAGAACTTCGATTTCTACATGTGTGCGCATGCTGGAATGATCGGGACT
ACGAGGCCAACTCACTACCACATCCTGCATGATGAGATAGGCTTCAGTCCTGATGATCTG
CAGGAGCTGGTGCATTCGCTCTCTTATGTGTACCAAAGGAGCACAACAGCCATATCAGTC
GTTGCTCCCATCTGCTACGCACATCTGGCAGCTGCTCAGGTTGGCCAGTTCATAAAG---

>LOC_Os06g39640
GTCTCGATAGGGCGGTCGTTCTACTCGCCGGACATAAGGAAGCCGCAGCGGCTCGGCGAT
GGCCTACAGTCATGGTGTGGGTTCTACCAGAGCATCCGGCCGACTCAGATGGGGTTGTCG
CTTAACATTGATATGTCATCTACCGCGTTCATCGAGCCGCTACCAGTGATCGAATTCGTG
GCCCAAATACTAGGGAAGGATGTCATATCGAGGCCATTGTCGGATGCAAACAGAATCAAG
ATCAAGAAAGCCTTGCGGGGCGTGAAAGTTGAAGTCACTCACCGGGGAAATGTGAGGCGG
AAGTATCGCATTTCAGGGCTGACAACACAACCAACTCATGAACTGATTTTCCCAATCGAT
GACCAAATGAACATGAAATCTGTCGTAGAGTATTTCAAGGAAATGTATGGCTTCACCATT
CAGCATCCCCATCTTCCCTGCCTTCAGGTGGGAAACCAAAAGAAGGCAAACTATCTACCA
ATGGAGGCCTGTAAGATTGTTGAGGGTCAGAGATATACAAAGAGGTTGAATGAAAAGCAG
ATCACATCATTACTCAAGGTTACTTGCCGTAGGCTTCTTTTGGCCATCCTCCCTGACAAC
AATGGTTCTTTATATGGTGATATAAAACGTATATGTGAAACTGACTTGGGGTTGATATCA
CAATGTTGCTTAACCAAACATGTTTTCAAGATCAGCAAGCAGTACCTGGCAAATGTCTCA
CTTAAAATCAATGTTAAGATGGGAGGAAGAAACACCGTGCTGCTGGATGCAATAAGTTGG
AGGATTCCTTTGGTCAGTGACATACCAACTATTATATTTGGTGCAGATGTCACGCATCCT
GAAACCGGGGAGGACTCTAGCCCATCCATTGCTGCTGTTGTTGCATCTCAAGACTGGCCA
GAAGTTACAAAGTATGCTGGATTGGTGTGTGCTCAGGCTCATCGGCAAGAGCTCATTCAA
GATCTTTACAAGACATGGCATGATCCTCAAAGAGGCACCGTAACAGGAGGCATGATCAGG
GAGCTCTTAATATCCTTCAGGAAGGCCACTGGGCAAAAGCCATTGAGAATAATTTTCTAC
AGGGATGGTGTCAGTGAAGGCCAATTCTACCAAGTTCTCCTCTATGAGTTGGATGCTATC
CGCAAGGCATGTGCATCTCTAGAACCAAATTATCAGCCTCCTGTAACATTCGTGGTTGTC
CAAAAGCGTCACCATACAAGACTCTTCGCAAACAATCACAAAGACAGAAGTAGCACGGAC
AAAAGTGGAAACATTTTGCCTGGAACTGTTGTTGATTCAAAGATCTGCCACCCATCAGAG
TTTGATTTCTACCTCTGTAGCCATGCTGGAATTCAGGGAACAAGTAGGCCAGCTCACTAC
CACGTCCTTTGGGATGAGAACAATTTCACTGCAGACGAAATGCAGACATTGACAAACAAC
CTTTGCTACACTTATGCACGGTGCACACGCTCTGTTTCTGTTGTTCCACCTGCATACTAT
GCGCATCTGGCTGCGTTTCGAGCGCGGTTCTACATGGAG--------------------

>Mguttatus_mgf000736m
ATCACAGTCGGTCGAAGTTTTTCTCTCCCGAATTCCGGAGCCGTGACGACTTTCGTAAC
GGACTCGCTGCATACAGAGGGTTGCAACAGAGTTTGAAGCCTACTTTGCAGGGTCTTGCT
CTGTGTTTGGACTACTCTGTTTTAGCATTTCGAAAGCCGTGGCCAGTGATGGAGTTTCTC
AAAGAGCATTTTAAAGGGTTTCAAGTGCAAGATGTAAAAAAGTCACGACGAGCTGTCAAC
GATGCTTTGAGAGGGTTGAAAGTGACGGTTACACACCGTCGCACGAAGCAAAAGTATACT
GTTGCAGGGCTAAGTGAAGAGGACACCTGTGATATTTACTTTGATCTTGTTGATGTGGAA
GGCAAGAACCCTCCTCAAAGAACTAGCCTTGTTAAGTATTTCAAGGAAAAATGGGGTAAG
GATATTGTGTACCAGAATATACCTTGCTTGGAACTTGGCAAAGGTAGTAAGTCGAACAAA
GTACCGATGGAGTTCTGTGTGTTAGTCGAAGGGCAGAGGTACCCGAAGGAGAATCTGGAC
AGAAACACGGCTGTGCTTTTGAAAAACATATCACTTGCGTTGATTGTTTGTGTAATGACC
AAAAAGGACCCGGGCTACAAGAATCTCAAGTGGGTTTCCGAGACAAAACTCGGTGTGGTG
ACTCAGTGTTGTCTTTCCGGTTCTGCCAGCAAACCGCAGGGGCAGGACCAGTATTTTGCC
AACCTCTGTCTGAAAATCAATGCTAAATTAGGTGGCAATAATTTCGAAGTGGCGGGAAAC
TTTACCCATTTCGATGCGAGAGATCATGTGATGTTTATAGGAGCTGACGTGAACCATCCT
GCACCTATGAACTCGTCTTGCCCCTCCATAGCTGCAGTTGTGGCGACCGTTAACTGGCCA
GCTGCAAATAAATATGCAGCCAGAGTTAGCCCACAGAAACACCGCTGTGAAAAGATCGAA
AATTTCGGAGCAATGTGTTTGGATTTGGTCAACACATACGCCAAATTTAATAAAGTCAAG
CCGAAGAGAATCGTGGTGTTTCGTGATGGTGTTAGTGAGGGGCAGTTCGAAATGGTCCTA
AGTCAGGAGCTGTTGGATTTGAAGAACTCGATTTATGACGGTGATTATCAGCCGTCGATC
ACTCTCGTTGTTGCTCAGAAGCGTCACCAGACGAGGCTTTTTATCGATAATGGAAACAGC
GGGGGTAGAATCGGAAATGTACCTCCTGGAACTGTTGTGGATACGAGGATTGTGCATCCG
TTCGATTTTGACTTTTATCTGTGCAGCCACTATGGAGCTCTTGGAACGAGCAAGCCTACT
CATTACTATGTGCTTTGGGATGAGAATTCGTTTACTTCTGACCAATTGCAGAAGCTTATA
AACGACATGTGCTACACATTTGTTCGGTGCACTAAACCCGTTTCGCTTGTGCCACCTGTC
TACTATGCTGACCTGGTTGCTTACAGGGGAAGAATGTTCCAAGAG---------------

>Smoellindorffii_15414178_locus
GTTCCTGGTGGTCGGTCTTTCTTCTCCCCTAACCTGGGAAACAAGGATCTTGGAGGCGGC
CTCGTCGCGTGGCAAGGCTTCTATATGTCCATCCGTCCAGCACAGGACAATTTGCTCGTC
CTTaacATTGACATGACCGGGAATGCGTTTATCAAGGAGCCTCGCGATCTGgatcgtaac
atgcgtcggcaggatGCACAGGGCGACACTTACCGCGTCAAGATGAAGAAGCTGGTGAAA
GGCCTCAAGGTGGAAACAAGTCACTGCAAGACCAAACGAAAGCTCAAGATTGTGAGCTTG
ACGCGACAGCCACTGGAAACTTTGAACTTTAATATGAATGGTATGCAAGTTTCCGTAGTC
GATTATTTTCGGCAAACTTATGGTTTAAACCTGGCATTTGGGGGGTTTCCTGCTGTGGAG
CAAGGATCCGGTGATCGGAAAAAGTATATCCCGCTCGAGCTCTGCCGACTCGTTAAGGGC
CAGAATTTTACCAGAAGGGTGAATGACGACCAGAGAAAGGGGCTCTCTGCAATGACATGC
TGCCTGCTCGTGTGCATTCTACCAGATCTGAACACTTCTTCCTTCTTCACAGTGACCATC
AAGCGCTTATGCGAGCTTGAACTTGGTGTCATAACTCAATGCGCGCAGGAGGGTAAAATT
AGGAAGTGTGATCCTCGCTACCTCGCAAACCTTATTTTAAAAATCAATGCCAAGTTCGGA
GGGAAAAACGCAGTTATATGTGCGCAAGATCTGAAGAAATGCAAGCCGGTAGCAGACTCG
CCAACTCTGATAATCGGTGCGGATGTTTCTCATCCTCGTGCTGGGGAGGAAACAGGGTGC
TCAATGGCAGCGGTTGTGGCGAGTATGGATTGGCCCGGGTTTGCGCAGTATGCGACGGTC
GTGAGAAGTCAGCCTTCGCGACAGGAGATGTTGGACGACCTGTTCTGGGAGAACGTTGAC

GAGCAGGGCCGTAGTGTCAGTGGTGGCATTTTCAAGGAGATGTTGATGGCATTCCATCAC
CGGACGAACTTTATCCCTGAGCGTATAATTTATTACAGAGACGGAGTAAGCGAAGGACAG
TTCGAGGCTGTGCTTCGTAGCGAATACGAGTCGCTTCAGCGACAGGCCGAGaagcggTCC
AAGCAACCATCAAAGGGTCCCAAGATCACGTTCATAGTGGTGCAAAAGAGGCACCATACC
TGTTTTTTCCCGGCCACGAAGCCGACTGGGAAAAACCAGAACATCAGCCCGGGGACAATC
GTCGATAAAGTCGTGTGCCATCCGACAAACTTCGACTTCTACCTTTGCAGCCACCAGGGA
ATCAAGGGGACTAGCCGGCCCGTGCACTACCACGTCCTCAAAGACGAGAACGGGTTTACT
GCTAACGAGATCCAGCAGTTCACGCACGACCTCTGCTACCTCTACTCTCGCTGCACCCGA
GCTGTTTCGTATGTTCCACCGTGCTATTACGCTCACTTGGCAGCACAGCGAGCGCAGGCT
TGGGTG--

>Mesculenta_cassava586.valid.m1
TGCCCCATTGGAAGATCCTTCTTTTCACCGGATATTAGAGCACCACAACGACTTGGTGAT
GGCTTGGAGTCCTGGTGTGGGTTTTACCAGAGTATAAGGCCTACACAAATGGGCCTATCC
TTGAATATTGATATGGCTTCAGCTGCATTCATTGAGCCTCTCCCAGTAATTGAGTTTGTT
GCACAGCTTCTGGGCAAGGATGTATTATCAAGGCCATTATGTGATTCCGATAGAATCAAG
ATTAAGAAGGCCCTCAGAGGAGTAAAAGTTGAAGTAACTCATAGAGGGAATATACGGAGA
AAGTATCGTGTCTCAGGATTGACATCTCAACCTACAAGAGAACTTGTATTTCCTGTTGAT
GATAACTCAACTATGAAGTCAGTAGTTGAATATTTCCAAGAGATGTATGGATTCACCATT
CAACATACGCATCTACCTTGCCTTCAAGTAGGAAACCAGAAGAAGGCGAACTATCTACCT
ATGGAGGCTTGCAAAATTGTGGAGGGCCAACGATATACGAAAAGGTTGAATGAGAGACAA
ATTACTGCCCTGTTGAAAGTTACATGCCAAAGGCTTTTATTAGCTATTCTACCTGACAAT
AATGGATCCCTATATGGGGATCTTAAGCGGATATGTGAAACTGATCTTGGTTTAATATCA
CAATGCTGTCTTACTAAACATGTTTTCAAAATCAGCAAGCAGTATTTGGCTAATGTATCA
CTGAAGATTAATGTTAAGATGGGTGGTAGAAACACTGTCCTTTTAGATGCCATTAGCTGC
AGAATACCATTGGTTAGTGACATACCAACTATTATATTTGGAGCAGATGTAACTCACCCA
GAGAATGGGGAGGACTCAAGCCCCTCCATTGCAGCTGTGGTAGCTTCTCAGGACTGGCCT
GAAGTGACAAAATATGCTGGATTGGTTTGTGCTCAAGCTCACAGACAAGAACTCATACAA
GACTTGTACAAAACATGGCAAGATCCAGTTCGTGGAACTGTTAGTGGTGGCATGATCAGA
GATCTTCTAGTTTCCTTTCGGAAGGCAACAGGACAAAAGCCACTAAGGATTATATTTTAC
AGGGATGGTGTTAGTGAAGGGCAATTTTATCAAGTTTTGCTTTATGAATTGGATGCAATC
CGGAAGGCTTGTGCTTCTCTAGAACCAAACTATCAACCACCTGTGACTTTCATAGTTGTA
CAAAAACGACACCATACTCGATTATTTGCTAACAACCACAGGGATAGAAGTAGCATAGAT
AAGAGTGGAAACATATTACCCGGCACTGTGGTTGATTCGAAAATCTGCCATCCAACAGAG
TTTGATTTTTATCTCTGCAGTCATGCAGGTATTCAGGGGACAAGTCGGCCTGCTCACTAC
CATGTTCTGTGGGATGAGAACAACTTCACTGCTGACGGAATCCAGTCATTGACGAACAAT
TTATGTTACACATATGCAAGATGCACTCGCTCTGTTTCTGTTGTTCCTCCTGCATATTAC
GCACATTTAGCTGCTTTTCGTGCCCGATTTACACGGAG----------------------

>Csativus_Cucsa.254700
TGTCCTATAGGGAGATCTTTCTTTTCTCCTGATATTAGATCACCTCAGCGGCTCGGTGAT
GGGCTAGAATCATGGTGTGGATTTTATCAGAGTATTAGACCTACTCAAATGGGACTGTCT
CTGAACATAGATATGGCTTCAGCTGCGTTCATTGAGCCTCTCCCTGTCCTTGAGTTTGTT
GCTCAGCTTCTAGGGAAAGATGTTTTGTCACGGCCACTGTCCGACTCCGATCGAGTAAAG

ATTAAAAAGGCTCTGAGAGGGGTGAAAGTTGAAGTAACACACCGTGGAAATGTTAGGCGG
AAGTATCGAGTTTCGGGTCTGACATCACAGCCTACAAGAGAATTAGTATTTCCTGTTGAT
GACAACTCAACCATGAAGTCAGTTGTTGAATACTTCCAGGAGATGTATGGCTTCACCATT
CAGCATGCACATCTCCCTTGCCTTCAAGTAGGAAACCAGAAGAAGGCAAATTATTTGCCA
ATGGAGGCCTGCAAAATTGTGGGGGGGCAAAGATATACAAAGAGATTGAATGAAAAGCAA
ATAACAGCACTTCTAAAAGTCACGTGTCAAAGATTATTATTAGCTATTTTACCTGACAAT
AACGGATCGCTTTATGGTGATCTTAAGCGAATTTGTGAAACTGATCTTGGTTTAATATCA
CAATGCTGTCTTACAAAGCACGTGTTCAAGATAAGCAAGCAATACCTTGCTAATGTGTCA
CTGAAGATCAATGTCAAGATGGGTGGAAGAAACACCGTTCTTCTAGATGCTATCAGCTGC
AGGATACCACTTGTGAGTGACATACCAACAATTATATTTGGAGCAGACGTGACCCATCCA
GAGAATGGCGAAGATTCCAGCCCTTCAATAGCTGCTGTAGTAGCCTCTCAGGATTGGCCT
GAAGTGACAAAATATGCAGGGCTAGTATGTGCTCAAGCTCATAGACAAGAACTTATACAA
GACTTGTACAAAACTTGGCAGGATCCTGTCCGTGGCACTGTCAGTGGTGGCATGATCAGG
GATCTTCTGATTTCGTTTAGGAAAGCAACAGGGCAGAAGCCTCTCAGGATAATATTTTAC
AGGGACGGCGTGAGCGAAGGACAATTTTATCAAGTATTACTTTATGAGTTGGATGCAATC
AGGAAGGCATGTGCTTCTTTAGAACCGAATTACCAACCACCTGTAACATTCATTGTCGTA
CAAAAGCGACACCACACCCGATTGTTTGCCAACAACTATAGAGATAGAAGTAGCACAGAC
AAGAGTGGGAACATTTTACCTGGAACTGTAGTCGACTCCAAAATATGCCACCCAACAGAA
TTTGATTTCTATCTCTGTAGTCATGCTGGAATTCAGGGAACGAGTCGGCCAGCTCACTAC
CATGTTCTTTGGGATGAAAACAATTTCACTGCAGATGGAATTCAGTCGTTAACAAACAAT
CTTTGCTACACGTATGCAAGATGTACACGTTCAGTTTCTGTCGTCCCTCCAGCATACTAT
GCACATTTAGCTGCATTCCGAGCTCGATTCTACATGGAA--------------------

>Alyrata_16064878_locus
TGTCCGGTTGGAAGATCTTTCTTTTCGCCTGATATTAGAACACCGCAGCGACTCGGTGAA
GGGTTAGAGTCATGGTGTGGGTTTTACCAGAGTATTAGACCAACTCAAATGGGTTTATCA
CTAAATATCGATATGGCTTCAGCTGCGTTCATCGAGCCTCTTCCGGTAATAGAGTTTGTA
GCACAGCTTCTTGGAAAGGATGTCTTGTCGAAGCCATTGTCTGATTCTGATCGGGTTAAG
ATTAAGAAGGGTCTCAGAGGAGTGAAAGTAGAGGTTACTCACAGAGCGAATGTAAGGAGG
AAATACCGTGTTGCGGGTTTAACAACTCAACCTACAAGAGAGCTAATGTTTCCAGTTGAT
GAGAACTGTACGATGAAGTCGGTTATTGAGTACTTCCAAGAGATGTATGGATTCACGATC
CAGCACACGCATTTGCCGTGTCTCCAAGTTGGAAACCAAAAGAAAGCAAGCTATTTGCCA
ATGGAGGCCTGCAAAATTGTTGAGGGACAACGATACACGAAAAGGTTGAATGAGAAGCAG
ATTACTGCTCTCTTGAAAGTTACATGTCAAAGGCTTCTGTTGGCAATTTTACCTGATAAC
AACGGTTCACTTTATGGTGATCTTAAGAGAATCTGCGAAACCGAGCTTGGTTTGATATCT
CAATGTTGTCTCACAAAACATGTGTTCAAGATTAGCAAACAGTATCTGGCAAATGTATCC
CTTAAAATCAATGTCAAGATGGGAGGAAGGAACACAGTTCTTTTAGACGCCATAAGCTGT
AGAATTCCACTGGTTAGCGATATACCGACAATTATATTTGGCGCAGACGTGACTCACCCA
GAGAACGGGGAAGAGTCAAGCCCTTCAATCGCTGCTGTTGTTGCTTCTCAAGACTGGCCT
GAAGTCACAAAATATGCGGGTTTAGTTTGTGCTCAAGCTCACAGGCAAGAACTTATACAA
GATTTGTATAAAACATGGCAAGATCCTGTACGCGGTACTGTTAGTGGCGGTATGATCAGG
GACCTTCTGATCTCATTTAGGAAAGCAACAGGGCAGAAACCGCTTCGAATTATCTTTTAC
CGTGATGGAGTAAGCGAAGGGCAATTCTATCAAGTTTTACTCTATGAGTTGGATGCAATT
CGAAAGGCTTGTGCGTCGCTTGAACCGAATTATCAGCCACCGGTGACATTCATTGTTGTA
CAGAAGCGTCACCACACTCGTTTGTTTGCTAATAATCACCGAGACAAAAGCAGTACTGAC
CGAAGCGGAAATATCTTACCTGGTACTGTAGTTGACACCAAAATATGTCATCCAACTGAA
TTCGACTTCTACCTTTGTAGCCATGCGGGTATTCAGGGAACAAGCAGGCCTGCACATTAC
CATGTTCTTTGGGACGAGAACAATTTCACAGCTGATGGTATTCAATCTTTGACTAATAAT
CTCTGTTATACCTATGCACGATGCACTCGATCAGTCTCGATAGTTCCTCCAGCGTATTAT
GCGCATCTTGCAGCATTTCGAGCACGTTTCTACATGGAA---------------------

>Csativus_Cucsa.200260
ACAGTTGTTGGAAGGTCATTTTTCTCGCATGAACTGGGGCAGCCAGGTGAACTTGGTAAT
GGTGTTGAGTATTGGAGGGGATATTACCAAAGTCTACGACCTGTACAAATGGGTTTATCT
TTAAATATAGATGTGTCAGCTAGATCATTTTATGAACCAGAATATGTTGTTAAACACTTC
AACCTAAGGAATCTCTCAAAGCCAATGTCTGATCAGGATTGTCGCAAGATCAAAAAGGTA
CTTAGAGGAGTGAAGGTTGGATTAACGTGTAGGGAGCATGCCAGGACCTACAAGATTACT
GGGATATCATCAGAGCCTGTTAATAGATTAATGTTTACTCTTGACGATCAGAAAACCCGA
ATCTCTGTTGCACAGTACTTTCATGAAAAATATGGCGTGGCACTCAAATATCCATTTCTA
CCAGCTATACAAGCTGGTAATGATGCCAAGCCGGTTTATTTGCCTATGGAGGTTTGTAAG
ATTGTTGCTGGGCAGAGATATACCAAAAAATTGAATGAACGCCAAGTAACTGAGATGCTA
AGAGCGACTTGTCAGAGATTGCTCATAATTATTTTACCTGATATTTCTGGTTCCTATGGG
AAGATCAAAAGGATATGTGAGACTGAGCTTGGAATTGTTTCACAATGTTGCCAACCTAGG
CAAGCACAAAAGCTGAACAAGCAGTACTTTGAAAATGTGGCCCTTAAAATTAACGTTAAG
GTTGGGGGAAGAAACAATGCCCTAAATGATGCTATTCAGCGGAAAATTCCACTTGTTTCA
GATCGGCCTACAATAATCTTTGGAGCAGATGTAACACACCCACAACCTGGAGAGGACTCC
AGTCCTTCAATAGCAGCTGTTGTTGCCTCAATGGACTGGCCCGAGGTAACGAAGTATAGA
GGAATTGTTTCAGCTCAGGGCCACCGGGATGAAATTATACAAGATTTGTACAGAGAAGAT
AAAGATCCTCAAAAAGGGTTGGTGTGTGCAGGAATGATCAGGGAGCTGTTCATTGCTTTT
AGAAGGTCAACAAATCTGAAACCGCACAGAATAATATTTTACAGAGATGGTGTAAGTGAA
GGACAGTTCTCACAAGTTTTATTTTATGAGGTGGATGCAATTAGGAAGGCATGTGCCTCT
CTTGAAGAGGGTTACCAGCCTCCGATTACCTTTGTTGTGGTGCAAAAGAGACATCATACC
CGTCTTTTCCCTATCAGCGGTGCGGATACTGATCGGAGTGGAAACATCCTTCCAGGTACT
GTTGTTGACACCAATATTTGCCATCCAACTGAATTTGATTTCTATTTAAACAGCCATGCC
GGCATTCAGGGGACGAGTAGGCCAACACATTATCACGTTTTGTATGATGAAAACAAATTC
ACTGCCGATGCAATGCAGATGCTTACTAATAATCTTTGCTACACGTATGCAAGGTGCACT
CGGTCGGTTTCCATTGTTCCACCAGCATACTATGCACATCTTGCAGCCTTTCGTGCCCGT
TATTACATAGAG--

>Sbicolor_Sb10g031030
GCACCATTTGGGCGATCATTTTTCTCTCCTGACCTGGGGAGGAGGCGATCCCTTGGTGAG
GGAATAGAATGCTGGCGTGGGTTTTACCAGAGCATTCGGCCTACTCAAATGGGCCTGTCA
TTGAATATTGGTAAAgctctgtccTTACCTGTGATAGATTTTGTTGCACAGCTTTTAAAC
ACCGACATCCACTCAAGGCCTCTCTCAGATGCTGAACGTGTCAAGATAAAGAAGGCCTTA
AGAGGAGTGAAGGTGGAAGTTACCCACCGTGGTAACATGCGACGGAAGTATCGAATAGCT
GGTTTAACATCTCTGGCAACTCGGGAGTTAACTTTTCCTGTTGATCAAGGCGGCACATTG
AAATCTGTTGTACAATATTTTCAAGAGACCTATGGCTTTGCCATCCAGCACACCTACCTG
CCCTGTCTGCAAGTCGGCAATCAGCAGCACCCAAATTACCTTCCAATGGAGGTCTGCAAA
ATAGTAGAGGGACAGAGGTACTCTAAGAGATTAAACCAGGGTCAGATAAGAGCTCTTTTA
GAGGAGACATGCCAGCGCTTGCTTATTGGGATACTTCCTGATAACAACGGTTCTCTTTAT
GGTGATTTGAAGCGTATCTGTGAAATTGACCTTGGATTAGTTTCACAGTGCTGCTGTGCA
AAGCAAGTTTTTAAGATGAACAAACAGATACTGGCAAATCTCGCTCTGAAGATAAATGTC
AAGGTTGGAGGAAGGAACACAGTGCTGGCTGATGCAGTATCAAGACGCATTCCTTTGGTG
ACTGACAGGCCTACCATCATATTTGGTGCTGATGTGACCCATCCTCATCCTGGTGAAGAT

AGCAGCCCTTCCATTGCTGCTGTTGTGGCTTCCCAAGATTGGCCTGAGGTGACAAAGTAT
GCAGGTTTAGTTTCTGCTCAATCTCATAGGCAAGAGTTAATAGAGGATCTGTATAAGGTC
GTACATGATCCTCAGAAAGGAACCATTTGTGGTGGCATGATCAGGGAGCTCCTTATATCC
TTCAAAAGATCAACTGGTCAAAAGCCTCAGAGGATACTATTCTACAGGGATGGTGTGAGT
GAAGGGCAGTTCTACCAAGTTCTACTGCATGAATTGGATGCAATCCGAAAGGCGTGTGCA
TCGCTGGAAGCAAATTACCAACCACAGGTGACTTTCATCGTGGTCCAGAAACGGCACCAC
ACGAGGTTGTTCGCACACAACCACAATGATCAGAATTCAGTCGACAGGAGTGGGAACATA
CTTCCTGGTACTGTTGTGGACTCGAAGATCTGCCACCCTACAGAGTTTGACTTCTTCCTG
TGCAGCCATGCTGGCATCAAGGGCACGAGCCGTCCTGCTCACTACCATGTCTTGTGGGAT
GAAAACAACTTCACAGCTGATGCATTGCAGACCCTTACCAATAACCTTTGCTACACTTAC
GCGAGGTGCACACGTTCTGTATCCATTGTCCCACCAGCATACTACGCTCATCTAGCTGCA
TTCCGCGCCCGGTTCTACATGGAG------------------------------------

>Rcommunis_29677.t000007
ATGGTGAGGAAGAGGAGAACTGAGGCTCCTGCAAGTGGGGGTGAGAGCTCTGAGCCCCAT
GAAGCGGCAAGTGGAGGATCTCAACGGCCATATGAGAGGAATGCACCACCTCAACAGGGA
CCAGGTGGAccaTACCAAGGTGGAAGGagttggGGTCCGCAGTCTCAGCAAGGAGGTCGC
GGCGGTGGTGGACGTGGACGTAGCGGTGGGATGTCTCAACAACAACAGTATGGTGGTGGC
CCTGAATATCAAGGCAGGGGAAGAGGTCCACCTCAGCAAGGTGGTCGAGGAGGCTATGGC
GGTGGTCGCTCCAGTAGTAACCGTGGAGGTCCACCTTCTGTTGGTCCATCCAGACCTCCA
GTTCCCGAGCTGCACCAAGCAACCTTGGCTCCTTACCAAGCTGGGGTGTCCCCTCAGCTG
ATGCCATCTGAGGGGAGCTCTTCTTCTGGTCCACCAGAGCCATCTCCGGTGTGCCCTGTC
GGTCGATCATTTTATTCTCCTGATCTAGGAAGAAGGCAACCCCTTGGTGAGGGTTTGGAG
AGCTGGCGTGGTTTTTACCAAAGCATTCGACCTACACAGATGGGATTGTCTTTAAATATT
GATATGTCTTCCACTGCTTTCATTGAACCGCTACCAGTCATTGACTTTGTGAATCAACTG
TTGAACCGAGATGTTTCTTCTCGACCATTGTCTGATGCTGACCGCGTGAAGATTAAAAAG
GCTCTTAGAGGAGTCAAGGTGGAAGTTACACACCGTGGAAATATGCGCAGAAAGTACCGC
ATATCTGGTTTAACATCACAGGCAACACGAGAGTTGACTTTTCCAGTTGACGAAAGAGGA
ACAATGAAATCTGTCGTGGAGTACTTCTACGAGACATATGGTTTTGTAATTCAACACACG
CAATGGCCCTGTCTACAAGTGGGGAATCAGCAGAGGCCGAATTATCTGCCTATGGAGGTT
TGCAAGGTTGTTGAGGGCCAGAGGTACTCCAAGAGACTGAACGAGAGGCAGATAACTGCC
TTGCTGAAGGTGACCTGTCAGCGTCTGCTTATCGTAATTCTCCCTGACAATAATGGCTCT
CTTTATGGTGAACTGAAGCGCATTTGTGAGACGGATCTTGGTCTTGTTTCTCAGTGCTGT
TTAACAAAGCATGTGTTCAGGATGAACAAACAATATTTGGCAAATGTGGCATTAAAAATT
AATGTTAAGGTTGGGGGAAGAAACACTGTGCTTGTTGATGCATTATCAAGGCGCATTCCT
TTGGTTAGTGATCGGCCCACTATTATTTTTGGTGCTGATGTCACCCATCCACATCCGGGG
GAAGACTCGAGCCCATCTATTGCAGCTGTTGTGGCTTCTCAAGATTGGCCAGAAGTAACG
AAATATGCTGGCTTGGTTTGTGCACAAGCCCATCGGCAAGAGCTTATTCAAGATCTCTTT
AAAGAATGGCAAGACCCTGTGAGAGGAAGAGTTACTGGTGGCATGATTAAGGAACTCCTT
ATATCTTTCCGCAGAGCAACTGGGCAGAAACCTCAGCGTATTATATTTTACAGGGATGGT
GTTAGTGAAGGACAATTCTACCAAGTGCTGCTGTATGAACTTGATGCTATCCGGAAGGCG
TGTGCTTCTTTAGAACCAAACTATCAGCCTCCAGTGACATTTGTTGTGGTTCAGAAGCGT
CACCACACTAGGTTGTTTGCAAACAACCATAACGACCGTAATGCTGTTGACAAGAGCGGA
AATATACTGCCTGGTACTGTCGTGGACTCTAAGATCTGCCATCCAACTGAATTTGACTTC
TATCTGTGTAGCCATGCTGGGATTCAGGGTACCAGCCGTCCAGCTCATTACCATGTATTA
TGGGATGAGAACAAGTTTACTGCCGACGGGTTACAGTCCCTTACAAACAATCTTTGCTAC
ACATATGCGAGATGCACGCGCTCTGTTTCCATCGTACCTCCTGCATACTATGCTCATCTT
GCAGCATTTCGAGCTCGGTTCTACATGGAA------------------------------

>Ptrichocarpa_POPTR_0006s12010
ACCATCGTTGGGAGGTCTTTTTCACAGCAGGTCTGGGTGGCCAAAATGAGATTGGTAAT
GGTATAGAATGCTGGAAGGGATTCTACCAGTCTCTACGCCCAACGCAGATGGGAATGTCT

CTTAACATAGATGTATCAGTCGCTGCCTTCTACGAGCCGCTATTAAATTTAGGAGATCCA
attagagcaGCAACCAGGCCTTTGTCAGATAGTGATCGAGCAAAGTTGAAAAAAGCTTTG
AGAGGAGTCAGAGTAAAAGTTACCCATGGAGAGGAGAAGCGTTACAAAATCACTGGAATA
TCTGCTTCAGCAACAAACCAACTAAGGTTTGCTGCTGAAGATGGAAAACAAAAATCAGTT
GTCCAATACTTCCTGGAAAAATACAATATAAGGCTTCGTTTTGCATCCTGGCCTGCTCTT
CAATCAGGAAATGATTCAAGACCAATATTCCTGCCTATGGAGTGTTGCAAGATTATCGAA
GGACAAAGGTACTCAAAGAAGTTGAATGAGAAGCAGGTGACAGCCTTATTGAGAGAGGCC
TGCAGGCGCCAAATCTTAATTATCATTCTCCCTGATGTCAGTGGAAGTTATGGCACAATA
AAAAGAGTATGCGAAACTGAACTTGGGATAGTTTCTCAATGCTGTCAACCCAAGCAAGCA
AGAAAGTGTAGTCCTCAATACTTGGAAAATGTTGCGCTGAAAATTAATGTGAAGGCTGGA
GGGCGAAACACAGTATTAGAGGATGCCCTGAATAGGAGAATACCTCTTCTAAGTGACACC
CCAACTATAATCTTTGGTGCTGATGTAACCCATCCACAACCAGGGGAGGATTCTAGCCCT
TCAATAGCTGCGATTGTGGCATCAATGGACTGGCCTGAAGTAACCACCTACAGGGGCCTG
GTATCTGCTCAGAAACATCGTCAAGAGATTATTCAAGATTGTGCTGGAATGATCAGGGAA
CTTATGATTGCTTTCAGAAGAACAACTAATCAGAAACCTAGCAGAATAATTTTCTATAGG
GATGGTGTTAGTGAGGGCCAGTTCAGCCAAGTTCTCCTGTATGAGATGGATGCTATCCGA
AAGGCTTGTGCATCTCTGGAACCAAACTATTTGCCACCAGTTACTTTTATTGTAGTGCAG
AAGAGACACCATACTCGGCTCTTTGCTACAAATCCTAACCAAACAGACAAGAGTGGAAAC
ATCCTTCCTGGAACGGTTGTTGATACAAAGATATGCCATCCTTCAGAGCACGACTTTTAT
CTCTGCAGTCATGCAGGAATTCAGGGGACTAGCAGGCCGGTGCACTATCATGTGTTGTGT
GACATGAACAAATTCACTGCTGATTGCCTGCAGATGTTGACGAACAACCTGTGCTACACG
TATGCAAGGTGCACCCGCTCTGTTTCTGTGGTTCCTCCTGCATACTATGCACACTTGGCA
GCATTCAGGGCAAGATACTACATAGAG---------------------------------

>Alyrata_16047041_locus
CTCCTAGTTCGCCAATCTTTCTTCCACAATGATGCAAAGTACTTTACGAATATCGGTGAA
GGTGTTGATTGTTGCAAAGGATTCCATTCAAGCTTCAGAACTACTCAGGGAGGCTTGTCC
CTCAATATTGATGTTTCGACTACGATGATAGTAAAACCTGGTCCTGTTGTTGGTTTTCTT
ATTGGAAACCAAGGTGTGAAAGATCCATTCTCTATTAACTGGAAAAAGGCTAAAAGTACT
CTCAAGAATCTTAGAGTTAAAGTCATCCCCTCAAATCAAGAATACAAGATAACCGGACTA
AGTGGACTACACTGCAAAGATCAAATGTTTACTTGGAAGAAAAAGAACCAAAACGGGGAA
GTTGAGGAGGTTGAGATTACAGTGTTTGATTACTTCACCAAAATCCGTGACATCAAACTG
CATTATTCAGGTGGCTTACCATGTATCAATGTTGGTAAGCCAAATCGTCCTACCTACTTT
CCCATTGAGCTCTGTGAGCTTGTATCTCTACAACGCTATACTAAAGCTCTTACAAGTTTT
CAGAGGAGTAACCTTGTCAAAGAATCAAGGCAAAACTTCCTTCTATGCATACTCGAAAAG
AAAAACTCTGATGTTTATGGTCCTTGGAAAAAGAAAAATCTTGTTCAAGTTGGAATTGTG
AATCAGTGTATTGCTCCTCCTCAAAACGTTAATGATCATTATCTCACAAATGTTCTCCTC
AAGATAAATGCCAAGCTTGGTGGATTGAATTCAGTGTTGGATATGGAGCGGTCACGGGCA
ATGCCTTTGGTAATGAAAGTTCCTACCATCATTATTGGGATGGATGTATCTCATGGTTCT
CCTGGACAGTCTGATGTACCATCCATTGCCGCGGTTGTGAGCTCCAGAGAATGGCCACTG
ATCTCAAAATACAGGGCTTGTGTGCGTACACAGTCGCGTAAAGTTGAAATGATCGATAAC
CTCTTTAAACCAGTCTCTGACAAAGATGATGAAGGTATCATGAGGGAGCTCTTGCTTGAC
TTTTACTCAAGTTCTGCAGTCAAACCCAATCACATTATTATTTTCAGGGATGGTGTGAGT
GAATCTCAGTTTAACCAAGTTCTTAATATTGAACTGGATCAGATGAAGCAAAAAAACCAC
CACACCAAGTTCTTCCAGACCAGGAGCCCTGATAATGTTCCTCCAGGAACAATAATTGAT
AGCAACATCTGTCACCAACACAACAACGATTTCTATCTTTGTGCTCATGCTGGAATGATT
GGAACTACAAGGCCAACACATTACCATGTGCTGTATGACGAGATTGGATTTGACACAGAT
CAACTCCAAGAACTTGTGCATTCCCTATCCTATGTCTACCAGCGGAGCACAACTGCAATC
TCTCTTGTTGCGCCGATATGTTATGCTCATTTGGCGGCTGCACAGATGGGAACTGCGATG

```
AAG---------------------------------------------------------
------------------------------------------------------------
------------------------------------------------------------
------------------------------------------------------------
------------------------------------------------------------
------------------------------------------------------------
------------------------------------------------------------
------------------------------------------------------------
------------------------------------------------------------
------------------------------------------------------------
------------------------------------------------------------
------------------------------------------------------------
------------------------------------------------------------
------------------------------------------------------------
------------------------------------------------------------
------------------------------------------------------------
------------------------------------------------------------
------------------------------------------------------------
------------------------------------------------------------
------------------------------------------------------------
------------------------------------------------------------
------------------------------------------------------------
------------------------------------------------------------
------------------------------------------------------------
------------------------------------------------------------
------------------------------------------------------------
------------------------------------------------------------
------------------------------------------------------------
------------------------------------------------------------
------------------------------------------------------------
------------------------------------------------------------
------------------------------------------------------------
------------------------------------------------------------
------------------------------------------------------------
------------------------------------------------------------
------------------------------------------------------------
------------------------------------------------------------
------------------------------------------------------------
------------------------------------------------------------
------------------------------------------------------------
------------------------------------------------------------
------------------------------------------------------------
------------------------------------------------------------
------------------------------------------------------------
------------------------------------------------------------
------------------------------------------------------------
------------------------------------------------------------
------------------------------------------------------------
------------------------------------------------------------
------------------------------------------------------------
------------------------------------------------------------
------------------------------------------------------------
------------------------------------------------------------
------------------------------------------------------------
------------------------------------------------------------
------------------------------------------------------------
```


>GRMZM2G031147_T01 cds:_protein_coding
ATTATCCTTGGTAGAGGATTTTACTCACCAAGCAGCAGTATAGACATTGGGCAGGGTGCT
GTAGCTATGAAAGGAACCCAGCAGTCCCTTAAATCCACTCAGCAAGGGTTGATCCTGTGT
GTTGACTATTCTGTCATGCCGTTTTACAAAGCTGTGATGGATCTTGTTCAGAAATTAGTG
AGGTACCTTGATTATCGGACAACTTTGAACAAGAGGCAAATGGAAAATCTGGTTGATGAG
CTTAAAGGCCGACGTGTAACTGTGATTCATCGGAGGACTAATCAGAAGTACACAGTGCAA
GGTTTGACACCCTTACCTGCCAGCCAGATGACCTTTGTGGATGCTGAATCCGGCCAAACA
CGGAGGCTCGTGGATTATTATGCTCAGAAACATGGCAAGGTGATTGAGTATCAGATGCTG
CCATGCTTGGATTTGAGCAAGAGCAAGGACAAACCGAATCATGTTCCAATTGAGCTCTGC
ACTCTTCTTGAAGGACAGAGGTTTCCAAAAGCAAACTTGAATCAGAATTCTGAGAGGATA
CTAAAAGGAAGTGCTCTACTCCTTTTCTGCCCGATGTCCGAGCAGCATTCGGGGTACAAG
ACACTGAAGCTGATTTGTGACACACAGCTGGGGATCCTGACCCAGTGTTTACTGAGCGAC
CGCGCAAACAATCGAAAGGGACAGGACCAGTACATGACGAATCTTGCTCTAAAGATCAAC
GGCAAGCTTGGGGGCAGCAACGTTCAGCTGTTTGACTCGCTCCCACGGGTCGGTGGTGGG
GTACCTTTCATGTTCATCGGTGCTGACGTTAACCACCCGTCCCCCGGTAACGTGGAGAGC
CCATCGATCGCAGCCGTGGTCGCCTCTGTCAACTCTGGTGTCAACAAGTATGTGACCAGA
ATCCGTGCCCAGCCGCACCGCTGCGAGGTGATCCAGCAGCTTGGTGAGATCTGCCGGGAG
CTCATTGGAGTCTTTGAGAAGCAGAACCGCGTGAAGCCGCAGAAGATCATCTACTTCCGT
GATGGCGTGAGCGACGGGCAGTTCGATATGGTCCTGAACGAGGAGCTGGCTGACCTGGAG
AAGGCGATCAAGGTGAATGGCTATGCGCCAACCATCACCGTGGTCGTGGCCAAGAAGCGG
CACCACACTCGGCTGTTCCCCAGGGACGAACAGCAGCCGCAGACGAAGACCGGGAACGTG
CCGCCTGGCACGGTGGTGGACACGGGCGTGGTGGACCCGTCCGCGTACGACTTCTACCTG
TGCAGCCACACTGGGATTCTGGGGACGAGCAGGCCGACGCACTACTACACCCTGGTGGAC
GAGCACGGCTTCGGCTCCGACGACCTGCAGAAGCTGATCTACAACCTGTGCTTCGTGTTC
GCGCGGTGCACCAAGCCGGTGTCGCTGGCGACGCCCGTCTACTATGCCGACCTCGCGGCC
TACCGTGGCAGGCTCTACTACGAG------------------------------------

>Gmax_Glyma15g13260
GTTTCTGTAGGACGACACTTCTATCCCACAAATCCTCCTGTCATAATGAAGGATCTTCAC
CACGGGATAATTGCTATTGGAGGGTTTCAGCATAGTTTGAAGCCTACATCTCAGGGTCTG
TCCTTATGTGTAGACTACTCAGTATTGGCTTTTCGAAAGCAAATGTCAGTCTTGGATTTC
TTGCATGAGCGTATTGATAACTTTAAATTGGATGAATTCGAAAAATTCAGAAAATTCATT
GAGGAGGCACTTATTGGATTGAAAGTTAATGTGACTCATCGTAAATGCAATCGGAAATAC
ATTATTTCAAGATTAACACCTATGATTACTAGGTATGTCACTTTTCCCATTGACAATACT
GGTGGATGGAACTCAAATGATGTTAGTCTTATTACCTTTTTTAAAGAAAAATATGGCAAG
GATATCGTGTACAAAGATATTCCTTGTCTAGATTTAGGGAAAGACAGGAAGAAGAACTAT
GTACCCATGGAATTCTGTGTTTTGGTTGAGGGCCAAAGATATCCCAAAGAGCGTCTGGAT
GGTATTTCTGCGAATACATTGAAAGCAATGTCACTAGCTCAATTTCTTCTCTGTGTAATG
GCTAAAAAAAGTCCCGGTTATAAGTACCTAAAGTGGATTTCTGAGACCAAACTTGGTATA
CTGACACAATGCTGCTTGTCTAATAGTGCTAATGAAGGGGAGGACAAATTTTATACTAAT

CTGGCTCTCAAGATCAATGCCAAGCTTGGCGGCAGTAACGTGGAGCTCAGTAATGGACTA
CCTTACTTTGAGGATGAAGGAGATGTTATGTTTTTAGGGGCTGATGTCAACCATCCTGGT
TATCAAGACACCAGGAGTCCATCAATTGCAGCTGTGGTTGCTACTGTTAATTGGCCTGCT
GCAAATCGTTATGCAGCACGTGTTTTCCCACAATACAATCGAAGTGAGAAAATATTGAAC
TTTGGGGATGTTTGTCTTGAGCTTGTTGCATGCTATAGGAGGATGAATGGAGTTAGGCCA
GAAAGAATTGTTATTTTTCGTGATGGGGTGAGCGAATACCAGTTTGACATGGTTCTTAAT
GAAGAGCTACTAGATTTGAAGGGAGTATTTCAAAGAGTAAATTACTTTCCAACAATCACT
CTTATTGTAACACAAAAACGACATCATACTCGATTTTTTCCAGAGGGCTGGAGAGATGGA
TCTTCCAGTGGCAATGTTTTGCCGGGAACAGTTGTCGACACAAAAGTTATACACCCTTAT
GAGTTTGACTTCTACCTTTGTAGTTACTATGGAAACCTAGGTACAAGCAAGCCTACTCAT
TACCATGTCTTATGGGACGAGCACAAGTTTACATCTGATTTATTGCAGAAGCTAATATAC
GAGATGTGTTTCACCTTTGCAAAGTGCACTAAACCTGTATCATTAGTCCCTCCTGTGTAT
TATGCTGACCTTGCTGCTTATAGAGGACGATTATACCATGAA------------------

>Rcommunis_29589.t000074
ACCCCTGTTGGGAGGTCATTTTTTTCACCTGATTTGGGCCAAAAGGGAGAGCTTGGCGAT
GGTATAGAGTATTGGAGAGGATACTATCAGAGTCTTCGCCCAACCCAGATGGGGCTTTCT
CTTAACATAGATGTATCAGCTCGATCATTCTATGAGCCCATTGTGACCGACTTTGTTTCA
AAATACTTGAAGTTGAGAGACATGTCAAGGCCTCTCTCTGATCAAGATCGAATTAAGGTG
AAAAAGGCTCTGAAATCAGTGAAAGTGCAAATACTTCACAGGGAGTATGCTAAAAGCTAT
AAAGTTACTGGCATATCCAATAAACCCCTGAATCAGATATTCTTCAAATTGGATGACAAG
AGCACAGATATCTCAGTTGTACAGTATTTCCGTGAAAAATACAATATTGGGCTAAAATAT
ACGTCCTTGCCTGCCCTTCAAGCTGGGAGTGATGCAAAACCAATTTACTTGCCTATGGAG
CTTTGTAAGATTGTTGATGGTCAGAGGTATTCAAAGAAACTTAATGAAAGACAAGTAACT
GCACTGCTAAGAGCAACCTGTCAACGCTTGCTGATAATTATTCTACCTGATATCAGTGGA
TCTTATGGAATAATTAAGAGAGTCTGTGAAACAGAATTGGGAATTGTTTCTCAGTGTTGC
CAACCACGACAAGCAGCAAAACTGAGTAAACAGTATTTTGAGAATGTTGCTCTCAAAATC
AATGTTAAGGTTGGGGGAAGAAACACTGTGTTGAATGATGCCGTGCAAAGGAGGATCCCA
CTTGTGACTGATTGCCCTACAATTATTTTTGGTGCTGATGTAACTCATCCACCACCAGGG
GAGGACTCTAGTCCGTCGATAGCAGCAGTAGTGGCTTCAATGGACTGGCCTGAGGTAACC
AAGTATAGAGGCATTGTCTCTGCACAGGCGCATCGTGAAGAAATCATCCAGGATTTGTAT
AAATCCTTTCAGGATCCTCAGGGAATATTAAAGCATTCAGGAATGATCAGAGAATTGTTT
GTTGCATTTAGAAGAGAAACTGGGATGAAACCTAAAAGGATTATATTCTACAGAGATGGT
GTTAGTGAGGGGCAGTTTAGTCAAGTTTTGCTGTATGAGATGGATGCCATAAGAAAGGCT
TGTGCCTCACTGGAGGAGGGATATCTTCCACCGGTTACCTTCGTTGTGGTGCAAAAAAGG
CACCATACACGACTTTTTCCCGTTGATCGTGGTCAGACAGACAGGAGTGGCAATATTCTG
CCAGGCACTGTTATTGACACTAAGATATGCCACCAGAGGGAGTTCGACTTCTACCTGAAC
AGCCATGCTGGAATTCAGGGAACCAGCAGACCAACTCATTACCATGTGCTGTATGATGAA
AATCATTTCACTGCTGATAATCTGCAAGTGCTGACTAATAATCTGTGTTACACGTTTGCC
AGGTGTACACGCTCTGTGTCCATAGTCCCTCCTGCCTATTATGCCCATTTAGCAGCTTTC
CGTGCTCGATATTACATTGAG---------------------------------------

>Gmax_Glyma20g28970

TGCCCCATTGGGAGGTCCTTCTTTTCACCTGATATTAGAACACCGCAACGGCTTGGAGAG
GGATTAGAGTCATGGTGTGGATTTTACCAGAGTATAAGGCCTACACAAATGGGCCTTTCC
CTTAATATTGATATGGCATCTGCTGCGTTTATTGAGCCTCTGCCAGTAGTGGAATTTGTT
GGTCAGCTATTAGGAAAAGATGTGCTGTCAAGGCCATTGTCAGATGCTGATCGCATTAAG
ATTAAGAAAGCCCTTAGAGGAGTTAAAGTTGAAGTAACACACAGAGGAAGTGTGAGAAGA
AAGTATCGTGTTTCTGGGTTGACTTCTCAACCTACCAGAGAACTTGTGTTTCCTGTTGAT
GAGAACTCAACTATGAAATCAGTAGTTGAATACTTCCAAGAGATGTATGGTTTCACTATT
CAATATACTCACCTTCCTTGCCTTCAAGTAGGAAACCAAAAGAAGGCGAACTATTTACCT
ATGGAGGCCTGCAAAATTGTTGAGGGGCAACGTTATACAAAAAGATTGAATGAGAAGCAA
ATTACGGCTCTATTGAAAGTTACTTGCCAGAGGCTTTTGTTAGCAATATTGCCAGACAAT
AATGGGTCTCTCTATGGTGATCTCAAGCGAATTTGTGAAACTGACCTTGGCTTAATTTCA
CAATGCTGTCTGACAAAGCATGTCTTCAAAATCACTAAACAGTACCTGGCTAATGTGTCT
CTGAAGATCAATGTGAAGATGGGAGGTAGAAACACTGTACTTCTTGATGCTGTAAGCTGC
AGAATACCATTGGTTAGTGACATACCAACCATAATTTTTGGAGCAGATGTAACCCACCCA
GAAAATGGAGAAGACTCGAGCCCTTCAATAGCAGCTGTAGTAGCATCCCAGGACTGGCCC
GAAGTGACAAAATATGCCGGTTTGGTATGCGCCCAAGCTCATAGGCAGGAACTTATACAA
GATTTGTACAAAACTTGGCAAGACCCTGTTCGTGGCACAGTTAGTGGTGGCATGATCCGA
GATTTACTGGTTTCCTTTAGAAAGGCAACAGGACAAAAGCCACTACGAATTATATTTTAC
AGGGACGGTGTAAGTGAAGGGCAATTTTACCAAGTTTTACTTTATGAGTTGGATGCGATT
CGGAAGGCATGTGCTTCCTTAGAACCAAACTACCAGCCTCCAGTAACATTCATAGTTGTG
CAAAAAAGACATCATACCCGGTTATTTGCAAACAACTACAGGGACAGAAGTAGTACAGAT
AGGAGTGGGAATATATTGCCTGGGACTGTCGTTGATTCCAAAATCTGCCATCCAACAGAA
TTTGATTTTTATCTCTGCAGCCATGCTGGCATCCAGGGTACTAGTCGGCCAGCTCATTAT
CATGTCCTGTGGGATGAAAACAACTTCACAGCAGATGGAATTCAGTCTCTGACAAACAAT
CTTTGTTATACATATGCCAGGTGTACGCGCTCAGTATCAGTTGTTCCTCCAGCATATTAT
GCACATTTAGCAGCGTTCCGAGCACGTTTCTATATGGAG---------------------

>Rcommunis_30093.t000002
ATTTATGCTGGTCGAATTTTCCATCCTATCATGCCTTATCCTGGGGATGATCTCAGACGT
GGAATTACAGCTTCTAGAGGAATCAAACATACCCTGAAGCCAACATCCCAGGGTCTAGCC
TTGTGTCTGGATTATTCAGTTCTGCCTTTGCTCAAGCAAATGCCGGTCATAGATTTTCTC
AAGGAGCATATTCGAGGCTTTAACTTGAACAATTTCAGAGCTTTCAGGAGAGAAGTGGAG
AGAGTTTTGAAAGAATTGAAAGTTACTGTTAATCACAGAACTACAGGTCAGAAATTCAAA
ATAGCAGGTTTAACCCACGATGACACACAGGATATTTCATTCGAGGTGGATCGTATCTCT
GAGAGGAAAGTTTGGCTTGTTGACTATTTTAAAGAAAAATACAACAAGAATATTACACAC
AGAAATATACCATGCTTAGATTTAGGTAAAAAGAACAGGACAAATTATGTTCCTATGGAG
TTCTGCAGCATAGCTAAGGGGCAGAGGTTTGCAATGGAGGATCTGGATCGAAATCAATCC
GAAAAGTTGAGGAGAATTTCATTAGCTATTCTTGTTTGTGTCCTGCCAAAGGAGGATCCT
GGTTACAGCAATCTCAAGTGGATATGTGAGACCAAAGTTGGCATTGTAACTCAATGTTGT
TTATCCGAAAATGCATTTAGACCAAAAGCTCAATTTCTTGCAAATCTAGCTTTAAAGATC
AATGCGAAGCTTGGAGGCAGTAATGTGGAGCTCTTCAAGCAGCCTCAATGCTTGCAAAGT
AAAGGCCATGTTATGTTTATTGGTGCTGATGTTAACCATCCTAGCTCTTACAACTCAACA
AGTCCTTCAATAGCAGCTGTTGTTGCCACCATGAATTGGCCTGCAGCAAATCAATATGGC
GCACTGATTTGTCCTCAAGATCATCGTGCAGAGAAAATCCTGAAATTCGGAGACATGTGT
TTAGAGCTTGTGAATGCTTATGCCCGGCTAAATCAGGTAAGACCAGAGAATATTGTTGTA
TTTCGTGATGGAGTAAGTGAGAGCCAGTTTGATATGGTTCTCAATGAAGAGTTGAAGGAT
ATCAAGGCGGCATTTGAATCACTTGAATACTTCCCAACCATCACTCTTATTGTTGCCCAG
AAGCGACACACAACTCGTTTGTTTCTAGATAGTGATGAAGATGAAAATGTACCTCCGGGC
ACTGTGGTGGATACAGTAATTACAAGTCCTTCTGGGTCTGATATCTATCTTTGCAGCCAT
TTTGGTCAAATTGGGACGAGCAAGCCAGCACACTACCAGGTGCTGCAGAACGAGATCGAG

TTTACTCCTAATGAGTTGCAGGAGTTCATTTACAGCATTTGCTTCACCTCTGCTCAGTGC
ACCAAGCCTGTCTCACTTGTCCCTCCAGTGATCTATGCTGATCGTGCAGCTTTTAGAGGT
CGGCTTTACTACAAT---

>Mguttatus_mgf011041m
ATCTCTATCGATCGTCACTTTTACTCTTCCAGTTTTAACGTGGAAGATGATTTTAAGCAC
GGTGTTGCAGCGTATCGAGGCTTCCAATCAACTTTAAGACCGACGTCGCAGGGTCTGGCC
TTGTGCCTCGACAGCTCGGTTTTGGCGTTTCGTAAGCCGTTGGCAGTTATGGATTTCCTT
AAAGAGAATATTCCTGAATTCgacggaatgtacCTCGGTTTCAATTTGAGGCGGAGAGTT
GCACATGCGTTGAAAGGATTGACGGTTAGAGTGACTCACCGTGTTACGAAACAGCGGTTC
ACCATAGCTGGTTTGACTGCCAAAAACACCCGTGATCTTTGGTTCGATTTTGTCGACCCG
AAAGGGAGGGACCCGACTGTTAACGTCAGCCTTGTGCAGTACTTCAGGCATAAGTATGGC
AAGGACATTGTGTACCAGGATATTCCTTGCTTGATTCTTGGCAGAAATAATCGGACGAAT
CACGTACCTATGGAATTCTGTATCTTGTCTGTCGGACAACGCTACAGAAAAGAGCTTTTG
GATGAAGTTTCGCAGGATAAATACGATGCAAAATGCCTGGCTATAATAATATGCGTAATG
GCGGAAAAACATCACGGCTACAAGTACCTTAAGTTCGTGTCGGAAACACGAATTGGTGTA
GTGACTCAGTGTTGCCTGTCTGGTCACGCATTCAGAGGAGACGAAAAGTTTCTCGGAAAT
CTGTGTCTCAAGATTAATGCAAAGCTCGGAGGAAGTAATGTTGAGTTGACTCAGAGACTT
GCTGATTTCGAGGAGGAAGATCATGTTATGTTCATTGGAGCCGATGTGAACCATCCAGTC
TCGAAGAAATCAACAACTCCATCCATAGCAGCAGTTGTCTCTACAGTGAACTGGCCTGCA
GTGAACCGCTATGCCGCAAGAGTTTGCCCTCAAGACCACAGAACCGAGAAGATTCTCGAA
TTCGGTTCCATGTGCCGCGATCTCGTCAACACTTATTTCCAGGTCAACAAAGTCAAACCG
AAGAAGATTGTTGTTTTCGAGACGGTGTGAGCGATGGGCAATTCGATATGGTACTGAAC
GAAGAATTATCCGACTTAAAAAGGTCTGTCTGCGATGAGAATTACAAGCCAACAATCACT
CTTGTTCTAGCTCAGAAGAGGCACCAGACTCGTCTCTTTCTCGAAAATGTTCTGGATGGT
GGGGCCACTGGGAATGTGCCACCTGGCACCGTTGTAGACACGAAGATTGTCCATCCGTTT
GAGTTCGATTTTTACCTCTGCAGTCACTATGGAAGGATTGGGACTAGCAAGGCGGTGAGG
TACTGTGTACTGTGGGACGAAAATTCGTTCACGTCCGATGAATTACAGAAGCTTATATAT
AATTTATGCTTCACGTTTGCACGTAGTACGAGGCCTGTTTCGCTGGTGCCACCTGTATAC
TACGCTGACCTGGTCGCTTACAGGGGTCGTATTTTTCAGGAG-----------------

>Sbicolor_Sb06g028510
ATTATCCTTGGTAGAGGATTTTACTCACCAAGCAGCAGTGTAGACATTGGGCAGGGTGCT
GTAGCTATGAAAGGAACCCAGCAGACCCTTAAATACACTCAGCAAGGGCTGATCCTATGT
GTTGACTATTCAGTTATGCCATTTTACAAAGCTCCGGTGATGGATATTGTTCAGAAATTA
GTACCCCACCTTGATTACCGGACAACACTGAACAGGAGGCAACTGGAAAATCTGATTGAG
GAGCTCAAAGGCCGACGTGTGACTGTGGTTCATCGGAGGACTAATCAGAAGTACACAGTG
CAAGGCTTGACACCCTTACCTGCCATCCAGATGACCTTTGTGGATGCTGAATCTGGCCAA
ACGAAGAGGCTTGTGGATTATTATGCTCAGAAACATGACAAGGTGATTGAGTACCAGATG
CTTCCATGCTTGGATTTGAGCAAGAGCAAGGACAAACCAAATCATGTACCAATTGAGCTC
TGCACTCTTCTTGAAGGACAGAGGTTTCCAAAAGCAAACTTGGATAAGAATTCTGACAGG
ATACTGAAAGGAAAGGCTCTAATTCAGCTCCTTTTCTGCCCGATGTCCGAGCAGCACCCA

GGGTACAAGACACTGAAGCTGATTTGTGACACACAGCTCGGGATCCTGACCCAGTGTTTC
CTGAGCGACCGCGCAAACAAGCCAAATGGGCAGGACCAGTACATGACCAACCTTGCTCTC
AAGATTAATGGCAAGCTTGGGGGCAGCAACGTTCAGCTGTTTGACTCGCTTCCACGGGTC
GGTGGTGGGGCACCTTTCATGTTCATCGGTGCTGACGTTAACCACCCGTCACCCGGGAAC
GTGGAGAGCCCATCAATCGCAGGCGTGGTTGCATCTATCAACAGCGGTGCCAACAAGTAT
GTGTCAAGAATCCGTGCACAGCCACACCGCTGCGAGGTGATCCAGCAGCTGGGTGAGATC
TGCCTGGAGCTCATTGGAGTCTTTGTGAAGATAAATCGCGTGAAGCCACAGAAGATCATC
TACTTCCGTGACGGCGTGAGTGACGGGCAGTTTGACATGGTCCTGAACGAGGAGCTGGCT
GACCTGGAGAAGGCAATCAAGGTGGACGGCTATGCACCTACCATCACTGTGATCGTGGCC
AAGAAGCGGCACCACACGCGGCTGTTCCCCAAGGACCAGGGCCAGCAGCAGACGAAGACT
GGGAACGTGCCGCCTGGCACGGTGGTGGACACTGGTGTGGTTGACCCGTCTGCATACGAC
TTCTACCTGTGCAGCCACACCGGGCTTCTAGGGACGAGCAGGCCGACACACTACTACAGC
CTGGTGGACGAGCACGGCTTCGGGTCTGACGACCTGCAGAAGCTGATCTACAACCTGTGC
TTCGTGTTCGCGCGGTGCACCAAGCCGGTGTCACTGGCGACGCCCGTCTACTATGCTGAC
CTCGTGGCGTACCGTGGAAGGGTCTACTACGAG---------------------------

>Vvinifera_GSVIVT00031927001
ATCTCTGTTGGACGGAGCTTTTACCCAACTCTGTTCTCTCTAGATGATGATCTTGGACAT
GGCATTGTAGCTTCTAGAGGATTTTTACATAGCCTCAAGCCCACGGCCCAGGGTTTAACC
TTATGTTTGGACTACTCAGTCTTAGCATTTCGAAAGCCAATTCCGGTTATAGATTTCCTT
GAGGAGCATGTTAATGGATTTAAGTTGAATGATTTAAGAAGAGTAAGGAAAGAAGTTGAG
GTTGCTCTAAAAGGATTAAAAGTTAGAGTCATTCATCGTCTTTGCAAACAAAAATACACT
ATCTCAGGATTAAGTGGTGAGGACACAAGGTATCTTTCATTTATCGCTGAAGACCTAGAG
GGCAAATCTCCAGCAAAGAAAGTTGGGATTATTGATTATTTCAGGGAAAAATATGGGAAG
GATATTAAGTACAAGGATATTCCGTGCTTAGATTTGGGGAAAAACAATAGGAAGAACTAT
GTACCCATGGAGTTCTGCATCTTGACTGAGGGACAGAGGTTTCTCAAAGAGAACTTGGAT
AGAAATGGGGCTCAGAAGTTGAAAAATTTGTCACTGCAAATTCTTGTATGTGTGATGGCT
AGGAAGGATCCTGGCTACAACTATCTCAAGTGGTTCTGTGAAACCAACATTGGGATAGTT
ACTCAATGTTGTTTGTCCAGCCCTGCAAATAAAGCAAATGACCAGTATCTTGCAAACTTG
GCTCTCAAGATGAATGCCAAGCTTGGAGGCAGCAACGTGGAGCTCATTGACCGGCTTCCT
CATTTTGAGAATGAAGGTTATGTAATGTTTGTAGGTGCTGATGTCAATCATCCTGGTGCT
TGGAACTCAGCTAGCCCTTCCATAGCTGCTGTTGTTGCCACTGTAAATTGGCCTGCTGTA
AATCGTTATGCTGCTCGTGTACGCCCCCAGCTCCATCGAACTGAGAAGATTCTAAATTTT
GGGGACATGTGCCTGGAGCTTATTGAGACCTATGCTCAGGTGAATAGAGCCAAGCCGGAT
AAGATTGTGGTGTTTAGGGATGGGGTGAGCGAGGGCCAATTTGACATGGTGCTTAATGAA
GAATTAGTAGATCTGAAGGGGGCTATCCAGAGGGGAAATTACAACCCAACAATCACACTT
ATTATAACCCAGAAGAGACATCAGACTCGACTGTTTCCAGAAAGCAAGAGGGAAaggggt
CAGGACAGGAGCTTCAATGAGAATGTGTCTCCAGGCACGGTTGTAGACACAACTGTGGTT
CACCCCTTCGAGTTTGATTTTTATCTTTGTAGCCATTATGGTGGTATTGGGACGAGCAAG
CCAACACACTACCATGTCCTCTATGACGAGCACAGGTTCTCTTCTGACCAACTCCAGAAG
CTTATCTACAACTTGTGTTTCACCTTTGTGCGGTGTACCAAACCTGTCTCCCTTGTTCCT
CCTGTATACTATGCCGACCTTGCTGCATACAGAGGACGGCTCTACCAT------------

>Ptrichocarpa_POPTR_0016s02480
CTTCTTGTTCGCCAGTCATTCTTTCACAATAATCCAAAGAACTATGTTGATCTGGGAGGA
GGAGTCCTTGGATGCCGAGGATTTCATTCGAGCTTTCGAGCCTTGCAGGGTGGATTATCC
CTAAATATGGATGGTTCGACTACAACGATAATACAGCCTGGGCCACTTATTGACTTTCTC
ATAGCCAACCAGAATGTGTCAAACCCTTTTCAGATCGACTGGGCAAAGGCAAAGCGGACA
ATGAAAAATTTGAGGATAAAGGTGTCACCTACCAATCAAGAGTACAGAATCACTGGCCTA
AGTGAAAATAGTTGCAAAGAGCAAATGTTTTCGCTGAAATCAAGAGCAGCTGATGGAAAT
GATGTTGAAAGTTTTGACATTACAGTTTATGATTATTTTGTTAATCATCGCAGCATAGAT
TTACGTTACTCTGGAGATTTGCCATGCATCAATGTTGGCAAGCCTAAAAGGCCTACTTAC
ATTCCCGTCGAGCTTTGTTCACTGCTTTCTTTGCAACGCTACACAAAGGCACTGACTGTC
CATCAGAGATCGCAGTTGGTAGAAAAATCAAGACAAAAATTTCTTGTGTGTCTTCTTCCT
GATAGGAAGAACTCTGACATATATGGTCCTTGGAAACGAAAGAATCTTGCAGAATATGGA
ATTTTCAACCAATGCCTGGCACCCACTAGAGTTAATGATCAGTATATATTGAATGTTCTC
CTGAAGATAAATGCTAAGCTCGGTGGTTTGAATTCTTTGTTGGCTATGGAGCAATCACGA
AACATCCCTTTTGTTTCAAAAGTTCCTACAATAATATTTGGGATGGATGTATCACATGGC
TCGCCTGGCCAGTCTGACATCCCATCCATTGCTGCGGTTGTCAGTTCTAGAAACTGGCCT
CTACTTTCTCGTTATAGAGCTTCTGTGCGTAGTCAGTCACCAAAAGTTGAGATGGTAGAT
TCTCTTTTTAAACTAACAGCAGATAAGAAAGATGATTGTGGGATTGTTAGGGAATTGTTG
TTAGACTACTATAAGAGTTCTGGCCAAACAAAGCCAGCTCAGATAATCATATTCAGGGAT
GGAGTTAGTGAGTCGCAGTTTAATCAAGTCCTCAACATCGAGCTGGATCAAATCATTGAG
GCCTGCAAGTTCCTTGATGAAAGCTGGTCACCCAAGTTCACTGTAATTGTTGCACAGAAA
AATCACCACACTAAATTCTTCCAAGATGGATCTCCAGACAATGTTCCTCCTGGAACTGTT
ATTGATAATGCTGTTTGTCACCCACAAACCTATGATTTCTACATGTGTGCCCATGCAGGG
ATGATAGGAACAACTAGGCCAACACATTATCATGTTCTTTTAGATGAGATTGGCTTTTCA
GCTGATGATCTACAGGAGTTGATTCACTCTTTGTCTTATGTGTACCAAAGAAGCACAACA
GCAATATCCCTAGTTGCTCCAGTCCGGTACGCACACTTGGCAGCAACTCAGATTTCACAA
TTCTTGAAG---

>contig11958_taeda
CTTCTTGTTGTTATTTTGCCTGATAACAATGGTTCTTTGTATGGCGACTTGAAGCGTATA
TGTGAGACTGACCTTGGTTTGGTTTCCCAGTGCTGTCTGACGAAACATGTCTTTAAAATG
AGCAAACAATATCTTGCAAATGTTGCTCTTAAGATTAATGTAAAGGTTGGTGGAAGAAAC
ACTGTCTTAGTTGACGCACTGTCAAGGAGGATACCTTTGGTCAGTGACAAACCTACAATA
ATCTTTGGAGCAGATGTTACCCATCCACACCCTGGAGAGGATTCTAGCCCATCAATAGCT
GCTGTTGTAGCATCTCAAGATTGGCCTGAAGTCACTAAATATGCAGGCTTAGTTTGTGCT
CAGGCACATCGTCAGGAACTCATTCAGGATCTTTACAAAGTCTGGCAAGATCCTGTGCGA
GGGTCTGCAACTGGAGGCATGATAAAGGAATTGTTGATTTCCTTCAGAAGGAATACAGGA
CACAAGCCGGAGCGTATAATATTTTACAGAGATGGAGTAAGCGAGGGCCAGTTTTATCAA
GTTCTGTTGTATGAGTTAGATGCTATTCGAAAGGCCTGTGCATCTCTAGAACCAAATTAT
CAACCTCCAGTTACTTTTGTGGTGGTTCAAAAACGTCACCATACTAGACTCTTTGCAAAC
AACCATCAAGATCGAAATGCGACAGACAGGAGTGGAAACATATTGCCAGGCACTGTAGTG
GATTCCAAAATTTGCCATCCAACAGAATTTGACTTTTACCTCTGCAGCCATGCTGGTATT
CAGGGAACTAGTAGGCCTGCCCATTATCATGTTCTTTGGGATGAAAACAAATTCACAGCT
GATGGATTGCAGTCCTTAACCAACAATCTCTGTTACACATATGCGAGGTGCACACGGTCA
GTTTCTATAGTACCCCCAGCGTATTATGCCCATCTGGCTGCATTTCGGGCTCGGTTTTAT
ATGGAG--

>Gmax_Glyma14g04510
TTGCTTGTGCGGCAATCGTTCTTTCACAACAATCCCAAGAATTTTGCTGATGTAGGAGGA
GGTGTACTTGGTTGCAGGGGCTTCCACTCTAGTTTTAGAACTACTCAAAGTGGACTGTCT
TTGAACATAGATGTCTCAACCACCATGATAATTACCCCTGGGCCTGTGGTTGACTTCCTA
ATATCCAATCAAAATGTGAGAGACCCCTTTTCACTTGATTGGGCAAAGGCCAAGAGGACA
TTAAAAAACCTGAGGATTAAATCGAGCCCATCTAATCAAGAATTCAAAATCACTGGGCTT
AGTGAACTCCCTTGCAAAGATCAAATGTTTACATTGAAGAAAAAAGGTGGTGATGATGAC
ACTGAGGAAGAAGTGACAGTATATGATTATTTTGTTAATATCCGCAAGATAGATCTTCGG
TATTCTGGGGATCTCCCATGTATCAACGTTGGGAAACCAAAAAGGCCAACTTACATTCCT
CTTGAGCTGTGTTCTTTGGTATCACTTCAACGTTATACAAAAGCACTATCCACACTTCAA
AGGTCTTCATTGGTGGAGAAGTCCAGGCAGAAGTTTCTTCTTTGTCTGCTTCCTGAGAGG
AAAAATTCTGATCTTTATGGTCCATGGAAGAAGAAGAATCTTGCTGAGTTTGGAATTGTG
ACTCAGTGTATAGCTCCGACAAGGGTCAATGACCAATATTTGACTAATGTTCTGTTGAAA
ATCAATGCTAAGCTTGGTGGTCTGAACTCAATGTTAGGCGTTGAGCATTCTCCTTCTATT
CCTATTGTTTCTAGAGCGCCAACCATCATTATTGGCATGGATGTGTCTCATGGTTCGCCA
GGGCAAACAGATATTCCTTCAATTGCTGCGGTGGTCAGCTCTAGAGAATGGCCTCTAATA
TCAAAGTATAGGGCCAGTGTCCGTACGCAGTCTCCAAAGATGGAAATGATTGATAATTTG
TTCAAGAAAGTTTCCGACAAGGAGGATGAAGGCATAATGAGGGAGCTTCTACTTGATTTC
TATACAAGTTCTGGAAATAGAAAGCCCGATAATATAATCATATTCAGGGATGGTGTTAGT
GAGTCCCAGTTCAATCAAGTTTTGAACATTGAACTTGATCAAATTATCGAGGCTTGCAAG
TTTTTAGATGAAAAGTGGAATCCCAAGTTTTTGGTGATTGTTGCTCAAAAGAACCATCAT
ACTAAATTCTTTCAACCTGGAGCTCCTGACAATGTTCCTCCTGGAACGGTAATTGATAAC
AAAATTTGTCATCCTCGGAATTATGATTTCTACATGTGTGCACATGCTGGAATGATTGGT
ACTAGCAGGCCTACACACTACCATGTTCTCTTAGACGAGATTGGCTTTTCACCTGATGAT
CTACAGGAGCTTGTGCATTCATTATCATATGTGTATCAGAGGAGCACAACTGCCATTTCT
GTCGTTGCTCCAATATGCTATGCTCATCTGGCTGCAACTCAGATGGGTCAATTTATGAAA

>Csativus_Cucsa.185140
CTTTTGGTAAGGCAGTCGTTCTTTCATGATGACTCAAGGAACTTTGCTGATGTAGGAGGA
GGGGTAACAGGAGTACGGGGATTCCATTCTAGCTTTAGGTTGGCACAGGATGGATTATCA
TTGAATATGGATGTTTCTACCACAATGATCCTGAAGCCTGGGCCAGTTATTGATTTCCTA
ATAGCAAATCAGAATGTACGGGAACCACGCTATATTGATTGGGGGAAGGCAAAAAAAATG
TTAAAGAATTTGAGAGTCAAGGCAAGACATCGGAACATGGAATTTAAAATCATTGGTTTA
AGTGAAAAGCCTTGTAACCAACAATTTTTTTCCATGAAACTGAAGAATAATGGCagcact
GATGGGGAGATGGTTGATATTACTGTTTATGAATACTTTGTCAGACACTGTGGCATTGAA
CTGACTCATTCTGCTTATTTGCCATGTCTAGATGTTGGGAAACCTAAACGACCAACTTAT

ATACCTTTGGAGTTGTGCTCACTTGTTTCACTTCAACGGTACACAAAAGCTTTGTCTTCA
ATGCAAAGAGCATCTTTGGTAGAGAAATCAAGGCAGAAGTTTATTCTTTGTGTCCTCCCA
GAAAAGAAAAATTCGAATATTTACGGACCCTGGAAGAAAAAGTGTTTGTGCGACTTTGGG
ATTTTTACACAGTGCATTTCCCCCACTAAAATTAATGATCAGTACATTACTAATGTACTT
CTTAAGATTAACTCCAAGCTGGGAGGTATAAACTCATTGTTGGCCATTGAGCATGCATCA
TGTGTTCCATTGATAAAAGATACTCCAACGTTGATCTTGGGAATGGATGTGTCTCATGGG
TCTCCTGGACGATCAGATGTTCCATCCATTGCTGCAGTTGTTGGATCCAGATCCTGGCCT
TTGATATCAAGGTATAGAGCAGCTGTACGGACCCAGTCGCCTAAGGTGGAAATGATTGAT
GCTCTATTTAAGCCTCTGGAGAATGGCAAGGATGATGGTATCATTAGAGAATTGCTTTTA
GATTTCTATAGCACCAGCAAGGGCCGCAAACCAACTCAGATTGTTGTCTTTAGAGACGGA
GTTAGTGAATCTCAATTTAATCAAGTTTTGAACATTGAGTTGGATCAAATAGTCAAGGCT
TACCAACATCTTGGAGAGGTTAACATTCCAAAGTTCACGGTTATTATAGCACAAAAAAAT
CACCATACAAGATTTTTTCTACCTGGTGCCTCTGAAAATGTTCCACCTGGAACGGTTGTT
GACACGAAAGTTGTACATCCAAAAAATTACGACTTCTACATGTGTGCTCATGCGGGAATG
ATCGGCACGTCAAGGCCAGCACACTACCATGTCCTGCTCGATGAAATAGGTTTTTCTCCT
GATGATCTTCAAAATTTTGTTCATTCACTTTCATACGTGTATCAAAGGAGCACAACCGCA
CTTTCAATTGCTGCACCAATATGTTATGCGCATCTTGCTGCAGCACAGATGAGCCAGTTT
ATTAAG--

>Cpapaya_evm.TU.supercontig_47.31
ATTATTGTGGGAAGATCGTTTTTCTCACCTGACATGAGGGGCAACCTTGGAGATGGCGTG
GACTTTTGGTGGGGACATTACCAAAGCTTGAGACCAACCCAGATGGGTTTATCCCTTAAT
ATTGATTTGTCTGCCACGGCATTCTATCAGCCACTGGTGACTGATTTTTTAAAAGAGAAT
TTGAAAATCAGAGACCTTTCGAGGCCATTGTCTGATCAGGATCGGGTGAAGGTCAGAAGG
GCCTTGAAATTGTTAAAGGTATCACTTACCTGTGGCCGATATCCTAGGGCTTATAAAATT
TTTGGCATATCAGATCAGCCATTAAGCGAACTAACGTTTTATCTTGATGAAGAGAGGCCA
AATATGTCCGTGGTCCAATATTTTTATGAAAAATACAAAACTAAGCTCAAATTTACTTCT
TTGCCTGCAATTCAAGCTGGAAGTGTATCAAGTCCTGCATTTCTGCCTATGGAGGTTTGC
CAAATTGCTGATAGACAGAGATATACAAAGAAGTTAAACGCTAGACAGATAACTAACCTG
TTAAGAGCAACCTGTCGGCGTTTATTGATTGTAATTTTACCCGATGTGACTGACTCATAT
GGAAAAATTAAAAGAGTGTGTGAAACAGAATTAGGAATTGTCTCACAATGCTGTCAACCT
GGACAAGCAAAGAAACTCAGCAAGCAATACTTTGAAAATATCGCTCTCAAGATAAATGTG
AAGGCTGGGGGACGAAACACTGTATTAGATGATGCTATTGAGGGAAGAATTCCTTTTCTT
ACTGATCGTCCTACCATCATTCTTGGAGCTGATGTGACCCACCCACAACCTGGCAATGAT
TCTTTTCCTTCAATAGCAGCAGTGGTGGCTTCCATGGACTGGCCTGAAGCAACCAAGTAT
CGAGGAATTGTATCTGCTCAGTCACACCGAGAAGAAATTATACAAGATCTTTATAAAATA
ATTCACGATCCTCCTGAAAGAGGTCGTGCTTGTGCCTCCCTGGAGGAAGGATATCTTCCG
CCAGTTACATTTATTGTGGTGCAGAAAAGGCATCATACACGTCTTTTCCCTGCTGACCAC
ACAAGGCACAACCTGACAGACAAGAGTGGCAATATTCTACCAGGTACCGTGGTTGACACC
AAGATTTGCCATCCCAGAGAATTCGATTTTTATCTGAATAGCCATGCTGGAATTCAGGGC
ACTAGTCGTCCTGTGCACTACCATGTTCTGTGGGATGAGAACAAATTCACTGCTGATGCA
TTGCAAGTGCTCACTAATAATTTGTGTTACACCTATGCAAGGTGCACACGGGCTGTTTCA
ATTGTGCCTCCTGCTTATTATGCGCATCTAGCTGCTTTTCGAGCTCGGTATTATTTAGAA

>Cpapaya_evm.TU.supercontig_26.59
CTTCTTGTTAGGCAGTCTTTCTTTCATAATGATCCGAAGAACTTCGCGGATGTGGGTGGT
GGTGTCTTAGGTTGTAGAGGATTTCATTCAAGCTTCAGGGCCTCTCAGGGAGGCTTGTCT
TTAAATATTGATGTGTCAACTACAATGATAATACGGCCTGGGCCTGTGGTGGATTTCTTA
ATTGAGAATCAAAATGTCAGAGATCCCTACTCCATTGATTGGTCAAAGGCTAAAAGAACA
CTGAAAAATTTGAGGATCAAGGCCAATCCCTCAAATCAGGAATACAAAATAACTGGTTTG
AGTGAGAGACCATGCAAGGAGCAAACGTTTGTATTGAAGAATaggAAGGATGATAATGGG
GAAGAAGCTGAAGTGACTGTGTATGATTACTTTGTTAATACTCGTGGTATAGAGTTGAGG
TACTCTGGTGATTTGCCTTGCATTAATGTTGGAAAGCCGAAACGCCCCACTTTTATCCCA
CTTGAGCTTTGCTCTTTGGTGCCCTTGCAGCGATACACAAAAGCACTTTCAACTCTCCAA
AGAGCTTCGCTAGTGGAGAAATCAAGGCAGAAGTTCCTTCTTTGCTTACTTCCTGAGAGA
AAGAACTGTGACATTTATGGTCCTTGGAAACGGAAGAATCTTGCTGAATTTGGTATAGTC
ACTCAATGCATTGCTCCCACAAGGGTCAATGACCAATATCTCACTAATGTTTTACTGAAG
ATCAATGCAAAGCTTGGTGGATTGAATTCAATATTAGCAATTGAACATTCTCCTGCAATC
CCTATTGTTTCAAAGGCTCCTACTATCATCCTTGGGATGGATGTTTCACACGGCTCTCCT
GGGCAATCTGATGTTCCATCAATTGCAGCAGTTGTCAGCTCTAGGCAGTGGCCATTGATC
TCACGATATAGAGCTTCTGTGCGTACACAGTCCCCCAAGGTTGAAATGATTGATTCTCTT
TTCAAGCGAGTGTCGGAGACTGAGGATGATGGTATCATGAGGGAGCTGCTGCTGGACTTC
TATACGAGTTCTGGGAAAAGAAAACCTGATCAGATCATCATATTCAGGGATGGAGTAAGT
GAGTCACAATTCAATCAAGTTCTTAACATCGAACTTGATCAAATTATTGAGGCCTGTAAA
TTTCTTGATGAATCCTGGTCCCCAAAGTTCTTGGTGATCATTGCACAGAAGAACCATCAT
ACGAAATTTTTCCAGTCTGGCTCCCCAGATAATGTTCCACCTGGAACTGTTATTGACAGT
AAAATTTGTCATCCACGAAACAACGATTTTTATCTATGTGCTCATGCTGGCATGATTGGA
ACTACAAGGCCTACACACTATCATGTTCTGTTAGATGAGATTGGCTTCTCTGCAGATGAT
CTCCAAGAGCTAGTTCATTCACTATCTTATGTATATCAAAGAAGCACAACTGCCATTTCT
GTAGTTGCACCTGTATGCTATGCTCATCTGGCAGCCACCCAGATGGGACAGTTTATTAAG

>LOC_Os03g58600
GTCACAGTGTCCAGATCCTTCTTCTCTACCCAGTTTGGTCACCGGGGTGACATTGGTGAG
GGACTTGAGTGTTGGAGAGGTTACTATCAGAGCCTGCGCCCAACACAGATGGGCCTTTCG
CTGAATATAGATATATCTGCAACGTCCTTTTTTAAGCCTGTGACAGTGATCCAATTTGTG
GAGGAGTTCCTGAACATACGTGACACCTCAAGACCTTTGTCAGACCGGGATCGTGTGAAG
ATAAAGAAAGCATTACGTGGGGTTCGCATTGAAACAAACCACCAAGAGGACCAAATCAGA
AGATACAAGATAACAGGGATTACCCCCATTCCTATGAGCCAGCTGATATTTCCTGTTGAT
GATAATGGGACAAGGAAGACTGTTGTTCAGTACTTCTGGGATAGGTACAATTACAGACTG
AAGTACGCTTCTTGGCCCTGCCTACAGTCTGGCAGTGATTCTCGCCCTGTATACTTACCT
ATGGAGGTGTGCAAGATTGTAGAAGGGCAGAGGTACTCCAAGAAGCTTAATGACAAACAA
GTGACCAACATCCTTAGAGCAACCTGTCAACGCCTTTTAATTGTAATTCTGCCTGAAGTT
AGTGGTTCTTATGGGAAAATTAAAAGGGTCTGTGAGACTGACCTTGGCATTGTATCTCAA
TGTTGTTTGCCAAGGCATGCCAGCAGGCCGAACAAGCAATATTTGGAAAATGTTGCACTC
AAAATCAATGTCAAGGTCGGAGGGCGCAACACTGTTCTTGAGCGAGCCTTTATCCGCAAT
GGCATACCATTTGTGTCAGAAGTCCCAACAATCATCTTTGGCGCTGATGTCACACACCCT
CCACCTGGAGAGGACTCTGCATCATCTATTGCTGCGGTTGTGGCATCTATGGATTGGCCT
GAAATCACCAAATACCGAGGTCTGGTCTCTGCTCAACCACATAGACAGGAGATAATAGAA
GATCTCTTTAGTGTTGGTAAAGATCCAGTGAAGGTTGTAAATGGTGGGATGATCAGGGAG
TTGCTTATCGCATTCCGCAAGAAGACTGGCAGAAGGCCTGAGAGGATAATCTTCTATAGA

GATGGTGTAAGTGAAGGTCAGTTCAGCCATGTGCTTCTTCATGAAATGGATGCCATCAGA
AAGGCTTGTGCATCTTTGGAGGAGGGATATCTACCACCTGTCACATTTGTAGTAGTTCAG
AAAAGGCATCACACAAGGCTTTTCCCAGAGGTTCATGGGAGGCGAGACATGACTGACAAG
AGCGGAAACATCCTTCCTGGAACTGTCGTGGACCGTCAGATTTGCCATCCTACAGAGTTC
GATTTCTACCTGTGTAGCCATGCTGGCATACAGGGTACTAGCAGGCCAACTCATTACCAT
GTCCTTTACGATGAGAACCATTTTACAGCCGATGCACTTCAGTCCCTGACCAACAATCTT
TGCTATACCTATGCGCGATGCACCCGGGCAGTGTCTGTGGTCCCACCGGCCTACTATGCT
CATCTTGCTGCATTCCGCGCTCGCTACTACGTGGAA------------------------

>Ppatens_1904560_locus
gagGAGAGTGCCCAAGCTCAGGCTTTAGCAGAAGCTCAAGCATCGGCATTAGCAGGAGCG
GAGGGGTCAGCTCCTTCAGCTGTTGCGCCGGAACCTGCTtcgACTCCGACTCCAGCTGCT
GGTGGAGCTGCTgttccagcccctattccagtgtcagcacctgcacagcacgttgcttct
caacatgttgagggccagggccatggtcaagctcactcccagcatttaggagagggctcg
caaggccaaggttcggggcattctcagggaCAGAGGCAAAGGCAAGGACAAGGGCAAGGG
CAAGGTCAAGGACAGGGGCAAGGTCAAGGACAGGGGCAAGGTCAAGGACAGGGGCAAGGT
CAAGGACAGGGGCAAGGGCAAGGACAGGGGCAAGGGCAAGGGCAAGGACGCGATGAACCC
AATAGCAGTGGACCTTCTCCTGTAGGACGCTCGTTCTATTCTCCCAATCTGGGCACTCGA
CAGCCGTTAGGAGATGGTCTTGAAAGCTGGCGGGGCTTTTATCAAAGTATTCGACCAACT
CAAATGGGATTGTCCCTAAACATTGACATGTCTTCCACAGCGTTCATAGAACCCAAGACT
GTAATGGAGTTTATAAGGGATCTCCTCAACAAGGAGTTGACCCGTAGCCTAAGTGATGCC
GATCGTATGAAGATCAAGAAAGCTCTTCGCGGAGTCAAAGTGGAAGTGACTCATCGCGGA
AGTATGCGTCGCAAATACCGTATATCTGGGCTTACCCATCAGGCTACTAATGAACTCGAA
TTTCCAGTTGATGAGAATGGGACATTGAAGTCTGTCACCGACTATTTTCGGGAAACATAC
GGCTATTTTATCCGTCATCCGTCTTTGCCGTGCCTTCAAGTGGGTAATTCTCTGCGTCCC
AACTACCTTCCCATGGAAGTATGCAAGATCGTTGAAGGCCAGCGATATTCGAAACGGTTG
AATGAGCGCCAAATTACAGCTCTTCTCAAAGTAACTTGCCAGCGACTCCTTATTGCAATC
CTTCCAGACAACAACGGGCCTTTGTATGGTGATTTGAAGAAGCAATGTGAGACTGTACTG
GGTGTGGTTTCTCAGTGTTGTTTGACCAAGCATGTTTTCAAGATGAGCAAGCAGTACCTG
GCAAACGTGGCTCTCAAAATTAATGTCAAGGTTGGGGGTCGGAACACTGTCCTGGTGGAT
GCACTTACACGCAAGATTCCTCTCGTCAGTGATATTCCCACCATAATATTTGGTGCAGAT
GTTACTCATCCACATCCTGGAGAGGACTCCAGTCCCTCCATTGCTGCAGTGGTAGCTTCG
CAGGATTGGCCCGAGGTCACCAAGTATGCAGGGTTGGTGTGTGCTCAAGCTCACCGTCAG
GAGTTGATTCAGGATCTGTACAAGGAATGGAGGGACCCCCAGAAAGGCACGATGACAGGA
GGGATGATAAAGGAACTCCTTATCTCTTTCCGGTGTGCGACCGGTCAAAAGCCACTTCGG
ATTATCTTTTACAGAGATGGAGTAAGTGAAGGTCAATTTTACCAGGTTCTTCTGTATGAA
CTGGACGCAATCAGGAAAGCTTGCGCTTCCCTTGAACCGGATTACCAGCCTCCAGTCACA
TTTGTGGTCGTTCAGAAAAGACACCACACTCGTTTATTTGCCAGCAATCATAATGACAAT
CGCTCTACTGATAGAAGTGGCAACATTTTACCAGGGACTGTAGTGGACTCAAAGATCTGT
CATCCTACAGAATTCGATTTCTATCTTTGTAGTCATGCTGGGATTCAGGGAACCAGTCGA
CCTGCGCATTACCATGTGCTGTGGGATGAGAACAAATTTTCTGCGGATAGTTTACAGTCG
TTGACAAATAATCTGTGCTATACATATGCACGGTGCACGCGCTCTGTTTCTATTGTTCCT
CCAGCATATTATGCACATCTAGCTGCTTTTCGTGCCAGGTTCTACATGGAT---------

>AT1G48410.2
ACGGATGCTCCATCTGAAGGAGGTGAAGGCTCTGGGTCTCGTGAAGCTGGTCCAGTCTCA
GGTGGTGGACGTGGTTCACAGCGAGGTGGTTTCCAGCAGGGAGGAGGACAACACCAAGGT
GGAAGGGGTTATACTCCTCAACCTCAACAGGGAGGTCGTGGTGGTCGTGGATATGGGCAA
CCACCACAACAGCAACAACAGTATGGAGGACCACAAGAGTACCAAGGAAGAGGAAGAGGA
GGACCTCCTCATCAAGGAGGTCGAGGAGGGTATGGCGGTGGCCGTGGAGGTGGACCTTCT
TCTGGACCACCGCAGAGACAATCAGTTCCCGAGCTGCATCAAGCTACCTCACCTACTTAT

CAAGCGGTGTCTTCTCAGCCTACACTGTCTGAGGTGAGTCCTACCCAGGTACCAGAACCT
ACTGTTCTGATTCCGGTGGGCCGGTCCTTTTATTCCCCTGATATAGGAAAAAAACAATCA
TTGGGGGATGGCTTGGAGAGCTGGCGTGGATTCTACCAAAGCATTCGTCCTACACAGATG
GGCTTATCACTCAATATTGATATGTCATCGACAGCCTTCATAGAGGCAAACCCTGTGATT
CAGTTTGTCTGTGATTTGCTTAACCGGGATATTTCTTCTCGACCTTTATCTGATGCTGAT
CGTGTTAAGATAAAAAAGGCTCTTAGAGGTGTCAAAGTTGAAGTGACTCATCGAGGAAAC
ATGCGCCGGAAGTACCGCATTTCCGGTTTGACTGCTGTGGCCACTCGGGAATTGACATTC
CCAGTAGATGAAAGAAATACTCAGAAATCTGTTGTAGAATACTTCCACGAAACATATGGT
TTTCGCATTCAGCACACTCAACTACCATGCTTGCAAGTTGGGAATTCTAATAGGCCTAAT
TACTTACCAATGGAGGTATGCAAGATTGTTGAAGGCCAGCGGTATTCCAAAAGATTGAAT
GAGAGACAGATCACTGCTTTGCTGAAGGTTACCTGTCAGCGCCTGCTTATTGTCATTCTG
CCCGATAATAATGGATCATTATACGGTGATTTGAAACGCATATGTGAGACTGAACTCGGC
ATAGTCTCTCAATGTTGCCTGACAAAGCATGTCTTTAAGATGAGCAAACAATACATGGCT
AATGTTGCGCTGAAGATTAATGTGAAGGTTGGAGGAAGAAACACAGTGCTTGTTGATGCT
CTATCTAGGCGGATTCCTCTAGTCAGTGATCGACCCACCATTATATTTGGTGCTGATGTT
ACCCACCCTCACCCTGGAGAGGATTCAAGCCCATCTATTGCTGCTGTTGTGGCATCTCAG
GATTGGCCTGAAATCACTAAATATGCTGGATTAGTTTGCGCTCAAGCGCATAGGCAGGAG
CTCATTCAGGATCTGTTCAAAGAGTGGAAGGATCCTCAGAAAGGTGTGGTGACTGGTGGC
ATGATAAAGGAGTTGCTCATAGCCTTCCGTAGATCAACTGGGCATAAACCACTAAGGATC
ATCTTCTACAGGGATGGAGTCAGTGAGGGACAATTTTACCAAGTTTTGCTCTATGAACTT
GATGCCATCCGCAAGGCCTGTGCTTCGCTGGAAGCAGGTTATCAACCACCAGTGACATTT
GTGGTGGTGCAGAAGCGTCATCACACGAGGCTGTTTGCTCAGAACCACAATGATCGCCAT
TCGGTGGACAGAAGTGGGAATATTTTACCTGGCACTGTTGTGGACTCTAAAATCTGCCAC
CCTACAGAGTTTGACTTTTACCTCTGTAGTCATGCTGGTATTCAGGGCACTTCTCGACCT
GCTCATTACCACGTTCTTTGGGATGAGAACAACTTTACTGCAGATGGACTTCAATCTCTG
ACCAATAACTTATGTTACACGTATGCAAGATGCACACGCTCAGTTTCAATTGTTCCCCCT
GCATATTATGCACATCTAGCAGCTTTTAGGGCTCGATTCTACATGGAG------------

>Cpapaya_evm.TU.supercontig_1.68
ATATCTGTAGGGAGATCTTTCTATTCAAGTTCAATGGGAGGTGCTAAGGAAATTGGGGGA
GGAGCTGTTGGTTTGAGAGGGTTCTTTCAGAGTCTTCGACCAACCCAGCAGGGCCTTGCT
CTCAATGTGGATTTCTCTGTTACTGCTTTCCATGAGAGCCAGAAGCGTCTCGAGTTTCTC
CAAGATCTTCCTCAAAGGAAAACGAGGAGGTTGAGTGGTGAAGAAAGGAAAGAAGTAGAG
AAGGCATTGAGGAACATCCGGGTTTTTGTTTGCCATAGAGAAACTGTTCAGAGGTACCGG
GTTTACGGCTTAACTGAGGAAGCTACCGAAAATCTTTGGTTTGCAGACAGGGATGGGAAG
AATCTGAGACTGCTAACTTACTTCAAAGATCATTACAACTATGATATACAATTCAGGAAC
TTGCCATGCTTGCAGATTAGTAGAAGCAAGCCATGTTATCTTCCTATGGAGCTTTGTATG
ATATGTGAAGGCCAAAAGTTTCTTGGTAAGCTTTCAGATGATCAGACTGCAAGAATACTT
AAGATGGCCTGCCAAAGACAGCTGCTGATGTGCATAATGGAGAGAAAGCACAAGGGATAT
GCAGATTTGAAGCGAATAGCAGAGACAAGTGTGGGAGTAGTAACCCAATGTTGTTTGTAT
CCAAACCTTAGCAAGTTGAGTTCACAATTCCTGGCTAACTTAGCTCTCAAAATGAATGCT
AAGGTTGGGGGCTGCACAGTTGCTTTGTACAACTCGCTACCGTCTCAAATTCCACGTATC
CTTCAGCTCGATGAGCCTGTGATGTTTATGGGTGCTGATGTTACTCATCCTCATCCTCTC
GATGATTTCAGCCCATCTGTTGCTGCTGTTGTAGGAAGCATGAACTGGCCTGCAGCCAAC
AAGTATGTTTCAAGAATGAGGTCCCAAACCCATAGACAAGAAATTATCCAGGATCTTGCT
TCGATGGTAGGGGAGTTACTAGATGATTTCCTCCAAGAAATAACCCAACTTCCCAAAAGA
ATCATATTCTTCCGGGATGGAGTAAGTGAAACCCAATTTAACAAGGTGCTTAAAGAGGAG
CTACAAGCTATTAGAGAAGCATGTTCAAGATTTCCTGGTTATAGACCTCCCATTACTTTT
TCTGTAGTTCAAAAGAGGCATCACACAAGGTTGTTTCCCTATGATCTTGATTCATCTTCC
TCCCAAAATCAGTTCTTTGAAGAAAACATTCCTCCGGGAACTGTGGTAGACACCGTGATT
ACTCACCCGAGAGAATTTGATTTCTACCTGTGTAGCCACTGGGGAGTAAAAGGAACCAGC
CGTCCAACACATTACCATGTCCTGTGGGATGAGAATCATTTCACTTCTGATGAACTCCAA
AAGCTGGTTTACAATCTGTGCTACACATTTGTGAGGTGTACGAAGCCAGTTTCTTTAGTT
CCCCCTGCCTACTATGCTCACTTGGCTGCATATAGAGGCAGACTTTACCTTGAG------

>Csativus_Cucsa.112480
ATGGGGAGGAAGAAGAGAACCGATGCTGGTGGTGAAAGTTCTGAGTCTCAAGATGGTGGT
GGTCGTGGCTCCCAAAGATCAGCTGAAAGGAGAGACACCCCACAGCAACATGGTGGTAGT
GGGTACCAGCGTGGGAGAGGTTGGGGTTCTCAAGGGGGACGTGGAGGCCAAGGTGGTGGA
GGGCGTGGCCGAGGAACGTCTCAACATCAGCACTATGGAGGACCTCCCGATCACCAAGGC
AGGGGTAGAGGTGGGCCATATCATGGAGGACATAACAACTATGGTGGTGGTGGTGGTAAT
CGTGGTGGCATGGGTGGTGGTGGCATTGGGGGAGGACCTTCTTCTGGTGGACCATCCAGG
TCACTAGTTCCCGAGCTGCACCAAGCAACCCCAATGTATCAAGGAGGGATGACTCAGCCA
GTCTCATCTGGGGCGAGTTCTTCCTCCCATCCATCTgacacttcaTCATGTCCAGTGGCC
CGATCATTTTACTCTCCAGACCTTGGCAGACGTCAGACACTGGGTGAGGGGCTGGAAAGT
TGGCGTGGTTTCTATCAGAGTATCCGCCCTACTCAAATGGGACTCTCCCTTAATATTGAT
ATGTCATCTACTGCTTTCATAGAGCCTTTGCATGTCATTGAATTTGTCACTCAACTTCTC
AATCGTGATGTGTCGTCCAGACCATTATCTGATGCTGACCGTGTGAAGATAAAGAAGGCT
CTTCGAGGTGTCAAGGTTGAAGTGACACATCGTGGGAATATGCGCAGAAAATATCGCATT
TCTGGTCTGACATCACAAGCAACACGCGAGCTAACTTTCCCTGTCGATGAGAGAGGTACT
ATGAAGTCAGTGGTTGAGTACTTCTACGAAACGTATGGGTTTGTTATCCAACATACCCAG
TGGCCTTGTCTTCAAGTAGGGAATCAGCAGAGACCTAATTATCTACCGATGGAAGTATGT
AAGATTGTTGAAGGCCAGAGGTATTCCAAAAGGCTTAATGAAAGACAAATAACTGCTCTG
CTCAAGGTTACATGCCAACGGTTGCTTATTGTTGTATTACCAGATAACAACGGATCCCTT
TATGGTGATTTAAAGCGTATCTGTGAAACTGATCTTGGCCTCGTTTCCCAGTGCTGTTTG
ACTAAACATGTTTTCAAAATGAGTAAACAGTATTTGGCCAATGTGGCCTTAAAAATAAAT
GTTAAGGTTGGGGGAAGGAATACGGTTCTTGTTGATGCATTATCTAGGCGCATACCTTTG
GTTAGCGACCGGCCTACCATTATATTTGGCGCCGATGTAACTCATCCTCACCCAGGAGAA
GACTCCAGTCCATCCATTGCTGCTGTTGTGGCTTCTCAAGATTGGCCGGAAGTTACAAAA
TATGCTGGTTTGGTTAGTGCTCAAGCCCACAGGCAAGAACTCATTCAAGATCTTTTCAAA
ACATGGCAGGATCCTGTTAGGGGGACAGTAACGGGTGGAATGATCAAGGAACTCCTCATC
TCCTTCCGTCGAGCAACTGGACAGAAACCTCAACGCATTATATTTTACAGGGATGGGGTT
AGCGAAGGACAGTTCTACCAAGTTTTGTTGCATGAGCTGGATGCCATTAGAAAGGCATGT
GCTTCTTTGGAACCAAACTACCAACCTCCAGTGACATTTGTGGTGGTACAGAAGCGTCAT
CACACTAGGTTGTTTGCTAATAACCATTCTGATCGCCATACAGTCGATAAGAGTGGGAAC
ATATTACCTGGCACGGTTGTCGATTCTAAAATTTGTCACCCCACGGAGTTCGATTTTTAC
CTGTGCAGTCATGCTGGAATCCAGGGTACAAGTCGTCCTGCTCATTACCATGTTCTATGG
GATGAGAACAAGTTCACTGCTGATGGATTGCAGACCCTAACGAATAATCTTTGTTACACA
TATGCTAGGTGTACTCGCTCCGTTTCTATTGTGCCCCCAGCGTACTACGCTCATCTTGCT
GCTTTCCGAGCTCGTTTCTATATGGAA--------------------------------

>Rcommunis_29813.t000096
ATACCTGTAGGGAGATCATTCTATTCAAGTTCAATGGGAGGAACTAAAGAAATTGGAGGA
GGGGCTGTTGGATTGAGAGGATTCTTTCAAAGTCTTAGACCTACACAGCAAGGACTAGCT
CTCAATGTAGACTTCTCTGTGACTGCTTTTCATGAAAGTCAAAAGCGTCTTGATTTTCTG
TGGGACCTTCCTCAAAACAAAAGGAGGAGTTTGATTGGTGAAGAAAGGAAAGAAGTAGAG
AAAGCCTTAAAGAACATCAGGGTCTTCGTTTGTCACAGAGAAACTGTTCAGCGATACCGG
GTTTATGGCCTAACTGAACAAGCTACAGAAAATCTTTGGTTTGCCGACAGAGATGGGAAG
AACCTGAGGCTACTGAGTTATTTCAAGGATCACTACAACTATGATATAAAATTTAGGAAC
TTGCCATGCTTGCAAATTAGTAGAAGCAAACCATGTTATCTTCCCATGGAACTTTGTATG
ATCTGTGAAGGCCAGAAGTTTCTTGGGAAGCTCTCAGATGATCAGACAGCAAGAATACTT
AAGATGGGCTGCCAAAGACAGCTGCTTATATGTATAATGGAGAAGAGACACAAAGGATAT
GCAGATTTGAAGCGAATAGCAGAGACAAGTGTTGGCGTTGTAAGCCAGTGCTGCTTATTT
CCAAATCTTGGGAAGTTGAGTTCACAATTTCTGGCTAATTTGGCTCTGAAGATCAATGCC
AAAGTTGGAGGATGCACAGTTGCTTTGTTCAATTCGCTACCCTCACAGATTCCACGCCTT
CTTCATTCTGATGACCCTGTGATCTTTATGGGAGCTGATGTTACTCATCCTCACCCTCTT
GATGACTTCAGTCCATCTGTTGCTGCTGTTGTTGGGAGCATGAACTGGCCGGCAGCAAAC
AAGTATGCCTCGAGGATGAGGTCACAAACTCATCGACAAGAAATTATCCAGGACCTTGGT

GCAATGGTGAAAGAATTGCTAGATGATTTTTTCCAAGAAGTTGGCAAACTTCCCAAGCGG
ATAATATTCTTTAGAGATGGAGTAAGTGAAACCCAGTTTCATAAGGTTCTTCAAGAGGAG
TTGCAAGCGATTAGAGAAGCTTGTTCTAGATTTCCTGGTTATAGACCTCCCATTACTTTT
GCAGTAGTCCAAAAGAGGCATCACACTAGGTTGTTTCCTTGCGAAACTGATCTAGCTTCA
ATTCAGAACCAGTTCTACGATGAAAATATACCGCCAGGGACAGTTGTGGATACCGTGATT
ACTCATCCAAAGGAATTCGATTTCTATCTATGCAGCCATTGGGGGGTGAAAGGAACAAGT
AGACCAACTCATTACCATGTCTTATGGGATGAGAACCAATTCACTTCTGATGAATTACAG
AAGTTGGTTTACAATCTGTGCTATACATTCGTAAGGTGCACCAAGCCAGTTTCTTTAGTC
CCTCCTGCTTACTATGCTCACTTGGCTGCATACAGGGGTAGACTTTACCTTGAG------

>CL344_lactuca
CTGCTCATTGTCATTCTGCCAGACAACAATGGATCCCTCTATGGTGACCTGAAACGAATC
TGTGAGACTGATCTTGGCGTTGTTTCTCAGTGCTGTCTGACTAAACATGTTTTTAAGATG
AGCAAACAGTACCTTGCAAACGTGGCTTTGAAGATTAACGTGAAGGTTGGAGGAAGGAAC
ACGGTCCTTCTGGATGCAATTTCAAGGCGGATACCTAATGTCAGCGATGTACCAACTATC
ATCTTTGGTGCTGATGTCACCCATCCACACCCCGGGGAAGATTCAAGCCCCTCTATTGCA
GCTGTTGTTGCCTCTCAAGACTGGCCTGAGATAACAAAGTATGCTGGGTTGGTTTGTGCA
CAAGCCCACCGTCAAGAACTGATCCAGGATCTGTACAAGGAGTGGCATGACCCACAAAGA
GGCAAAATGTCTGGTGGCATGGTCAAGGAACTGCTGATATCTTTTCGCAGAGCAACTGGG
CAGAAGCCAAAGCGGATTATTTTTtacaggtattttggaTATAGGGATGGAGTGAGCGAG
GGGCAGTTCTATCAAGTTTTACTTTATGAGCTTGATGCGATTCGAAAGGCATGTGCATCG
TTGGAGCCAGATTATCAGCCTCCAGTGACATTTGTGGTGGTTCAAAAACGTCATCACACG
CGATTGTTTGCTAACAACCATCGTGACCGCAATGCTACCGACAGGAGTGGGAATATACTG
CCAGGCACCGTTGTCGACTCCAAGATATGTCATCCAACCGAGTTTGATTTCTATCTATGC
AGCCATGCTGGGATTCAAGGGACAAGCCGTCCGGCCCATTACCATGTGCTGCGGGATGAG
AACAAGTTCGCAGCTGATGCTTTGCAATCACTCACAAACAATCTCTGTTACACGTATGCA
AGGTGCACTCGTTCTGTGTCCATTGTGCCACCTGCATACTATGCTCATCTGGCTGCCTTC
CGTGCACGGTTCTATATGGAG---------------------------------------

>Alyrata_16042271_locus
ACGTCGATTGGAAGATCGTTTTACTCGAGTTCTATGGGTGGTTCCAAGGAGATTGGAGGA
GGAGCTGTTGGACTCAGAGGGTTTTTCCAGAGTCTTAGGCAGACTCAACAAGGTTTAGCA
CTTAACATGGATCTCTCAATCACAGCTTTCCATGAAAGCCAGAAGCGGCTCGAGTTTCTC
AAGGACCTTTCTAGGAACAAAGATACAGAATTGAATCTAGAGGAAAAGAGAGAAGTGGAG

AAAGCACTTAAGAACATAAGAATCTTTGTTTGCCATAGAGAAACAGTTCAAAGGTATCGG
GTTTACGGGTTAACAGAGGAGATTACCGATAATATATGGTTTCCGGATAGAGATGGGAAG
AACTTAAGGCTTATGAGCTACTTTAAAGATCATTATGGTTATGAGATTCAGTATAAGAAC
TTACCGTGTCTGCAAATCAGTAGGGCAAGACCTTGCTACCTTCCTATGGAACTATGTATG
ATCTGTGAAGGTCAAAAGTTTCTTGGGAAGCTTTCGGATGATCAAGCTGCAAAGATCATG
AAAATGGGCTGCCAAAAACTGATTATCTGTGTAATGGAGAAAAAACATAAAGGGTACGGA
GATCTTAAGCGAATAGCAGAGACAAGAATTGGTGTTGTGACGCAATGCTGCTTATACCCT
AACATCACTAAGCTCAGTTCTCAGTTCGTTTCAAACTTAGCTCTCAAGATAAACGCCAAG
ATCGGCGGATCCATGACCGAGCTCTACAACTCAATACCTTCTCACATCCCAAGACTACTT
AGACCCGATGAGCCGGTTATCTTCATGGGAGCTGATGTAACGCATCCTCATCCATTCGAT
GACTGTAGCCCTTCAGTAGCGGCTGTGGTCGGGAGCATAAACTGGCCAGAAGCTAACAGA
TACGTCTCGAGAATGAGATCTCAGACTCATAGGCAAGAGATCATTCAAGATCTTGACTTG
ATGGTCAAGGAACTTCTTGACGATTTCTACAAAGCGGTAAACAAGCTTCCGAATCGAATC
ATATTCTTCAGAGACGGTGTGAGCGAGACACAGTTCAAGAAAATCCTCCAAGAAGAGCTT
CAATCCATCAAAATCGCTTGCTCTAAGTTCCAGGATTACAATCCAAGCATCACATTCGCC
GTGGTCCAGAAAAGACACCACACAAGGCTGTTCCGGTGCAATCCAGACAATGAGAACATC
CCTCCTGGCACAGTGGTTGATACAGTGATAACTCATCCGAAAGAGTTTGATTTCTATCTC
TGTAGCCACTTAGGAGTAAAAGGCACGAGCAGGCCAACGCATTACCACATCCTATGGGAC
GAGAACGAGTTTACTTCAGACGAATTGCAGAGACTTGTGTATAACTTGTGTTACACTTTC
GTGAGGTGCACGAAACCTATTTCGATTGTACCACCGGCTTATTATGCACACCTTGCTGCG
TACAGAGGAAGGCTCTACATCGAG------------------------------------

>Sbicolor_Sb02g032980
GTTGCAGTTGGCCGGTCCTTCTTCTCACCACTTGTAAAGCCAGGACCCAAGAATCTTGGC
CTGGGTGTGGAGGGATGGAATGGTTTCTATCAGAGCATCAGGCCGACACAGAAGGGCCTG
TCTGTGGTCGTAGACATGTCTTCAACAGCTTTTGTTCGACCCATGCCACTGATTGAATTT
GTGATGGAGATTCTGAACAAAGATAGCAGGACCATTAGAAATATTACTCCCATGGAGCTT
GTCAAGCTCAAGAAAGCCCTCAGGGGTGTGAGGATTGAAGTCACACACCGAGGAGATGCA
CGCCGGAAGTACCGGATTGCCAGCCTGACAACGAGTCCTCCTTCTTTACAGTTCTTTGAA
TCGTCCGCTGGAGTTCAGAAGTCTGTCGCAGATTACTTCAGAGAGGCATACAATCTGGAA
ATGCACTACGATTCTCTCCCATGCCTCCAAGTTGGCAGTGATGAGAGGCCGAACTACCTC
CCTATGGAGGTTTGCAAGATAGTAGCTGGACAGCAATACCGGAAGAAGTTGGATGGCCAA
CAAGTCCTTAATCTAATGGACTCAACCTGCCTGCGCCTTTTGCTTGCTATACTGCCAGAG
AAAAACGGCAACTTATATGGTAATTTCAAAAGGATCTGCGAGACAGAGATTGGTATCATG
TCGCAGTGTTGCCTGGATAAAAATGTTAGAAGTGCAGGTCCTCCATACTTTGCTAATGTT
GCTATTAAGATCAATGCCAAGTTTGGAGGAAGGAACTTAGAATTTGCTAATCCCAAAGAA
AGCTTACCGGTTGTTTCGATTGAACCAACAATTATATTTGGTGCCGATGTCACTCACCCT
GCTGCTCTGGATGATACTGCCCCTTCCATTGCTTCTGTTGTTGCCTCCCAAGACTGGCCC
ACGGTGGCTAACTATAATGGCATTGCCCGTGCACAAGGTCACCGTAAAGAGCTCATCGAT
GGCCTGGAAGACATTGTCAAGGAACTCCTACTTGCATTTCAGGAACGGTCTAAGCAGAGG
CCCAAGCAGCTGATCTTCTACAGGGATGGCGTAAGTGAGGGCCAATTCAAACAAGTGCTG
GAACAAGAAATCCCAGAGATAGAGAAGGCATGGAAAGCTCTTTACAATGAGAAGCCAAAG
ATCACCTTCATAGTGGTGCAGAAGAGGCACCACACAAGGCTCTTCCCAAATGATCGCCAA
TGGACAGACAGGAGTGGAAATATTCTACCTGGCACTGTAGTTGATAAGAGTATCTGCCAC
CCAACAGAATTTGATTTCTTCCTGTGCAGCCATGCTGGTATCAAGGGAACAAGCCGTCCT
ACGCATTACCATGTGCTGCGAGATGACAATAAGTTCACTGCAGATGCTCTGCAGTCTCTC
ACATATAACTTATGTTACTTGTATTCAAGCTGCACTCGCTCTGTTTCAATCGCTCCTCCC
GCATACTACGCCCACAAGCTAGCGTTCCGTGCCCGCTTCTACATCAAC------------

```
-------------------------------------------------------------
-------------------------------------------------------------
-------------------------------------------------------------
-------------------------------------------------------------
-------------------------------------------------------------
-------------------------------------------------------------
-------------------------------------------------------------
-------------------------------------------------------------
-------------------------------------------------------------
-------------------------------------------------------------
-------------------------------------------------------------
-------------------------------------------------------------
-------------------------------------------------------------
-------------------------------------------------------------
-------------------------------------------------------------
-------------------------------------------------------------
-------------------------------------------------------------
-------------------------------------------------------------
-------------------------------------------------------------
-------------------------------------------------------------
-------------------------------------------------------------
-------------------------------------------------------------
-------------------------------------------------------------
-------------------------------------------------------------
-------------------------------------------------------------
-------------------------------------------------------------
-------------------------------------------------------------
-------------------------------------------------------------
-------------------------------------------------------------
-------------------------------------------------------------
-------------------------------------------------------------
-------------------------------------------------------------
-------------------------------------------------------------
-------------------------------------------------------------
-------------------------------------------------------------
-------------------------------------------------------------
-------------------------------------------------------------
-------------------------------------------------------------
-------------------------------------------------------------
-------------------------------------------------------------
-------------------------------------------------------------
-------------------------------------------------------------
-------------------------------------------------------------
-------------------------------------------------------------
-------------------------------------------------------------
-------------------------------------------------------------
-------------------------------------------------------------
-------------------------------------------------------------
-------------------------------------------------------------
-------------------------------------------------------------
-------------------------------------------------------------
-------------------------------------------------------------
-------------------------------------------------------------
-------------------------------------------------------------
-------------------------------------------------------------
-------------------------------------------------------------
```


>Mesculenta_cassava27530.m1
GTATCAATTGGAAGGTCCTTCTATTCTCCTGATATAAAGAAACCACAGAAGCTGGATGGA
GGTTTAGAATCATGGCGGGGCTTCTACCAAAGTATAAGACCAACTCAAATGGGGCTATCA
CTGAATATTGATATGTCGGCTACGGCTTTCATTGAACCACTCCTTGTTATTGATTTTGTT
GCTCAAATTTTGTGCAAGGACGTGTATAAAAGGCCATTATCAGATGCAGATCGCGTTAAG
GTCAAGAAAGCTCTTAGAGGTGTCAAAGTTGAAGTCACTCACAGAGGAAATGTACGAAGA
AAATACCGAATTTCCGGGTTGACAACACAGCCTACAAGAGAGCTAATCTTCCCACTCGAT
GGGCATATGAACATGAAATCAGTTGTTGAGTACTTTCAAGAGATGTACGGCTATACCATT
CAGTATCCTCATCTACCTTGCCTGCAAGTAGGAAACCAGAGGAAGGTGAACTATTTGCCG
ATGGAGGCTTGCAAGATAGTCCAGGGACAAAGATATACAAAAGGGCTAAATGAGAAGCAG
ATCACTTCTTTGCTAAAAGTTTCATGTCAAAGATTGCTTATTGCAATTCTTCCTGACAGC
AATGGCTCATTGTATGGTGATTTAAAAAGAATTTGTGAAACTGATCTCGGGTTGATTTCC
CAGTGCTGCCTTACCAAACATGTCTTCAAAATTAACAGACAGTACTTGGCAAATGTGTCA
CTCAAAATCAACGTTAAGATGGGAGGAAGAAATACTGTGCTTTTGGATGCTTTGAGTTGG
AGAATTCCCTTGGTCAGCGACATCCCAACAATAATTTTTGGGGCTGATGTAACACATCCA
GAGTCAGGAGAGGACACTAGTCCATCTATAGCTGCTGTTGTAGCCTCCCAAGACTGGCCT
GAAGTTACAAAATATGCCGGATTAGTATGCGCTCAGCCTCACCGGCAAGAACTTATTCAA
GATTTATTCAAAACATGGCAGGACCCTCAACAGGGAACTGTCTCTGGAGGCATGATCAGG
GAGCTCTTACTTTCATTTAAGAAAGCCACTGGGCAAAAGCCATTGAGGATAATATTTTAC
AGGGATGGTGTAAGCGAAGGACAGTTCTACCAGGTTCTGTTGTATGAACTTGACGCGATT
CGAAAGGCTTGTGCATCGCTAGAACCCAGTTACCAACCTCCAGTGACATTTGTGGTTGTC
CAAAAGCGGCACCATACTAGACTCTTCACGAGCAATCACAACGACAGAAATAGCACTGAC
AAGAGTGGAAATATTTTACCTGGTACTGTAGTGGATTCTAAAATATGTCATCCGACTGAA
TTCGACTTCTATTTATGTAGTCATGCAGGAATCCAGGGCACCAGCCGGCCTGCCCATTAT
CATGTGCTGTGGGATGAGAACAACTTCACTGCAGATGAGATTCAATCTCTGACAAACAAC
CTCTGCTATACGTATGCAAGGTGCACACGCTCGGTTTCTGTAGTTCCTCCTGCATATTAT
GCTCATTTGGCAGCTTATCGAGCTCGGTTTTACATGGAA---------------------

>Ptrichocarpa_POPTR_0001s22120
GTTACTGTTGGGAGGTCATTCTTCTCACTTGATTTGGGCAAAAAAGGCGAGCTTGGTAAT
GGAATAGAATACTGGAGAGGTTATTATCAAAGTCTTCGGCCAACCCAAATGGGACTCTCT
TTAAATATTGATGTGTCAGCCAGATCCTTTTATGAGCCATTGGTGACTGAGTTTGTGGCC
AAGTACTTCAATCTCAGAGACTTGTCAAGGCCTCTTTCTGATCAGGATCGTGTTAAGGTG
AAAAGGGCCTTAAGAGGAATCAAAGTGGAAATTTCATACAGGGACTATGCCAGAAGCTTC
AAGGTTACTGGCATATCTAATCTACCAGTAGACAAGACAATGTTTACTCTGGATGATAAG
AAAACAAAGGTGTCTGTCCATCAGTATTTCTGGGACAGATACAATATTGGGCTGAAATAT
ACATCTTTGCCTCCCCTTCAAGCCGGAACTGATGCGAAACCCATTTATTTGCCCATGGAG
CTCTGTAAGATTGCTGGAGGACAGCGATACACAAAGAAACTAAATGAAAGACAAGTCACT
GCACTTTTAAGAGCTACCTGTCAACGCCAACTGCTGATCATTATTCTACCTGATTTCAGT
GGATCCTATGGGAAAATCAAACGTATTTGTGAAACTGAGTTGGGAATAGTTTCACAATGC
TGCCAGCCCCAGCAGGCAAAGAAGCTCAGCAAACAATACCTGGAAAATGTTGCTCTCAAA
ATTAATGTGAAGGCTGGTGGACGGAACACTGTGTTAAATGATGCTATTCAAAGAAGAATT
CCTAATGTTACTGATCTTCCTACTATTATTTTTGGTGCTGATGTGACCCATCCACAGCCA

GGGGAAGACTCTAGCCCGTCGATTGCAGCAGTAGTGGCTTCTATGGACTGGCCAGAGGTA
ACCAAATATAGAGGACTTGTCTCTGCACAGGCTCATCGCGAGGAAATTATTCAGGATCTT
TACAAAAAATATCAGGATCCACAAAAGGGTTTAGTTCACAGCGGAATGATCAGGGAGCTG
TTTATTGCATTCAGAAGATCAACAGGCCAAAAACCTCATAGAATTATATTCTATAGAGAT
GGTGTAAGTGAAGGCCAATTCAGCCAAGTTCTGCTACATGAGATGCAGGCGATACGAGAG
GCATGTGGCACCCTGGAAGAAGGATATTGTCCTCCGGTTACCTTTGTTGTAGTGCAGAAA
CGGCATCATACACGCTTCTTTCCTGCTGACCATAGCAAGCGAGATCTGACTGACAGGAGT
GGCAATATCCTACCAGGCACGGTCGTGGACACTAAAATTTGCCACCCTACAGAGTTTGAT
TTCTACCTTAACAGTCACGCTGGAATTCAGGGAACTAGCAGACCTACACACTACCATGTA
TTGTTTGATGAAAACAACTTCACTGCTGATGGCTTACAAACCCTCACTAACAATTTGTGC
TACACGTATGCAAGATGCACTCGGTCTGTTTCCATAGTGCCCCCTGCATATTACGCCCAT
TTGGCAGCTTTTAGGGCTCGGTATTACATTGAG---------------------------

>Bdistachyon_Bradi1g36910
GTGCCGATAGGGCGCTCGTTCTACTCGCCAGACATACGGAAGCCACAGCGTCTCGGCGAC
GGTCTGCAGTCCTGGTGTGGGTTCTACCAGAGCATTCGGCCAACTCAGATGGGCTTGTCG
CTTAACATCGATATGTCGTCCACTGCATTCATCGAGCCGCTGCCGGTGATCGAGTTCGTG
GCCCAGATTTTAGGGAAGGATGTCATGTCAAGGCCATTGTCTGATGCAAACAGAATTAAG
ATCAAGAAAGCACTGCGGGGTGTGAAAGTTGAAGTTACTCACCGGGAAAATGTAAGGCGG
AAGTACCGTATTTCAGGGGTGACAGCACAACCAACACACGAACTGATTTTCCCAATTGAC
GATCAAATGAATATGAAATCAGTTGTAGAGTATTTCAAGGAAATGTATGGGTTCACAATT
CAGCATGCCCATCTTCCTTGCCTTATGGTGGGAAACCAAAAGAAGGCAAACTATCTACCA
ATGGAGGCTTGCAAGATTGTTGAGGGCCAGAGATACACAAAGAGGTTGAATGAAAAGCAG
ATCACCTCGCTGCTAAAGGTTACATGCCAAAGGCTTCTTTTGGCCATTCTCCCTGACAAC
AATGGTGCTTTATATGGTGATATCAAACGTATCTGTGAAACTGATTTGGGATTGATATCA
CAATGTTGCTTAACTAAGCATGTTTTTAAGATTAGCAAGCAGTACTTGGCAAATGTTTCG
CTTAAAATCAATGTTAAGATGGGAGGAAGAAACACTGTGCTTGTGGATGCGCTAAGTTGG
AGGATTCCATTGGTCAGTGACATACCAACTATTATATTTGGTGCGGATGTAACACATCCT
GAAACCGGGGAGGACTCTAGTCCATCCATCGCTGCAGTTGTGGCTTCTCAAGACTGGCCA
GAAGTTACGAAGTATGCTGGATTGGTGTGCGCGCAGGCGCATCGGCAAGAGCTCATTCAG
GACCTTTATAAGACATGGCATGACCCTCAGAGAGGCACGGTCACAGGAGGCATGGTCAGG
GAACTCTTAATATCCTTCAGGAAGGCCACTGGGCAGAAGCCATTAAGAATAATTTTCTAC
CGTGATGGTGTTAGCGAAGGCCAATTCTACCAAGTTCTCCTGTATGAGTTAGATGCTATC
CGTAAGGCATGTGCATCCCTGGAACCAAATTACCAACCTCCTGTAACATTCGTGGTCGTT
CAAAAGCGTCACCATACAAGGCTGTTTGCAAACAATCACAAGGACAGAAGTAGCATGGAC
AAGAGTGGAAATATTTTGCCTGGAACTGTTGTTGATTCGAAGATATGCCATCCAACGGAG
TTTGATTTCTACCTCTGTAGTCATGCTGGAATCCAGGGAACAAGTAGGCCAGCTCACTAC
CATGTCCTCTGGGATGAGAACAATTTCTCAGCAGACGAGATGCAGACGTTGACAAACAAC
CTTTGCTACACGTACGCACGGTGCACACGCTCTGTTTCTGTCGTCCCTCCTGCATACTAT
GCGCATTTGGCTGCATTCCGGGCACGGTTCTACATGGAG---------------------

>Ppatens_1885752_locus
TCTCCCGTTGGGCGCTATTTCTATTCACCCGATCTGGGCACGCGACGGCCATTAGGAGAT
GGTCTTGAAAGCTGGCGGGGCTTTTATCAGAGTATTCGACCAACTCAAATGGGGTTGTCC

CTAAACATTGACATGTCTTCCACAGCGTTCATTGAACCTAAAACTGTTATAGAGTTTGTA
AAGGATCTCCTGAGGAAGGATTTAAACCGTAGTCTGACCGATGCTGATCGCATTAAGATC
AAGAAAGCCCTCCGTGGGGTGAAGGTGGAAGTCACTCATCGCGGAAGTATGCGGCGCAAG
TATCGCATATCTGGGCTCACTAATCAGGCCACCAGTGAATTGCAATTCCCTGTGGACGAT
AATGGGACTATGAAGTCTGTTACTGACTACTTTCGCGAGACTTATAGCTACACAATTCGA
CATCCAGCTTTACCCTGTCTCCAAGTGGGAAATACTCAGCGTCCCAATTATCTTCCAATG
GAAGTCTGCAAAATTGTTGAGGGCCAGCGATATTCCAAACGGCTGAATGAGCGCCAAATT
GCTGCACTTCTACAAGTCACTTGTCAGCGACTGCTTATTGCAATTCTTCCCGACAACAAC
GGGTCTTTGTATGGCGATCTTAAGAAACAGTGTGAGACTGTCCTGGGGGTCGTGTCTCAA
TGTTGTTTGACGAAACATGTATTTAAGATGAGCAAACAGTACTTGGCAAATGTTGCCCTG
AAAATCAATGTCAAGGTTGGAGGCCGAAATACTGTTCTGGTGGACGCTCTTTCTAGAAGA
ATTCCTCTTGTCAGTGACAAACCCACCATTATATTCGGTGCAGATGTCACTCATCCGCAC
CCCGGAGAAGATTCGAGCCCTTCTATTGCGGCTGTTGTGGCATCGCAGGATTGGCCTGAG
GTCACCAAGTACGCTGGTTTAGTGTGCGCTCAAACTCACCGTCAAGAGTTGATTGCGGAC
CTATTCAAAGAATACACTGACCCAATGAAGGGGAAAATGTTTGGAGGGATGATTAGGGAA
CTTCTCATCTCTTTCCGGAGCGCAACTGGTCAAAAGCCTCTCCGTATCATCTTTTACAGA
GATGGAGTAAGCGAGGGCCAGTTTTCACAGGTTCTTCTTCATGAGCTAGACGCAATCAGG
CGAGCCTGTGCTTCGCTTGAAGAAGGATACCAACCTCCAGTCACGTTTGTAGTGGTTCAG
AAAAGGCACCACACTCGTTTGTTTGCCAGTGATCGTCGCAATACTGATAGGAGTGGCAAC
ATTCTACCAGGTACGGTTGTGGACTCTACAATCTGTCACCCTACGGAATTCGACTTCTAT
CTTTGCAGTCACGCTGGAATTCAGGGTACCAGTCGACCTGCACATTACCATGTATTGTGG
GACGAGAATAGTTTTTCTGCTGACAGTTTGCAGTCATTGACAAACAACCTATGCTATACG
TATGCACGGTGCACACGGTCAGTGTCAATCGTCCCCCCCGCATATTATGCTCATTTGGCC
GCTTTCCGAGCGAGATTCTACATGGAT--------------------------------

>Alyrata_16062911_locus
GTCTCTGTTGGGAGGTCTTTTTCCACACTAGTTTGGGAAAGGACaccAGAGATGGTAGG
GGTGAGCTTGGAGATGGTATTGAGTACTGGAGAGGTTTTTCCAAAGTCTAAGGCTGACT
CAGATGGGTTTGTCTCTTAACATCGACGTTTCAGCAAGATCATTCTATGAACCAGTCACT
GAGTTTATTAGCAAGTTTCTGAATATAAGAGACTTAAACAGACCACTTAGAGACTCAGAT
CGACTTAAGGTGAAGAAAGTTTTGAGGACACTAAAAGTTAAGTTGCTACATTGGAACAGC
ACAAAAAGTGCCAAGATTAGTGGGATTTCCAGCTGTCCCATCAGTCAGCTAAGGTTCACT
TTAGAGGACAAATCAGAGAAGACGGTTATACAATATTTTGCTGAAAAATATAATTATAGA
GTGAAATACCCGGCTCTACCTGCTATTCAAACTGGGAGTGACACAAGACCCGTCTACCTA
CCTATGGAACTCTGCCAAATTGACGAAGGGCAAAGATACACCAAAAGGCTTAATGAGAAG
CAAGTGACTGCGTTGCTAAGAGCTACCTGCCAACGACTGTTGATTGTAATATTGCCTGAT
GTGACTGGATCATATGGACAAATCAAAAGGATCTGTGAAACAGAATTGGGGATCGTCTCT
CAGTGTTGCCAACCAAGACAAGCTTCTAAACTCAATAAGCAGTACATGGAAAATGTTGCC
TTGAAGATTAATGTCAAGACTGGGGGAAGGAACACTGTTCTTAATGATGCTATTAGAAGA
AACATACCTCTTATTACTGATCGCCCAACCATAATCATGGGTGCTGATGTGACTCATCCG
CAACCTGGAGAGGACTCGAGTCCTTCTATTGCTGCTGTTGTGGCCTCTATGGACTGGCCT
GAGATAACTAAATACCGAGGACTGGTTTCTGCTCAAGCTCATAGGGAAGAAATTATCCAG
GACCTGTATAAGCTGGTTCAGGATCCACAACGTGGGCTAGTCCACTCTGGTTTGATAAGG
GAACATTTCATAGCATTCAGGAGAGCTACGGGCCAGATACCTCAAAGGATTATCTTCTAT
CGCGACGGAGTAAGCGAAGGGCAGTTTAGTCAGGTTCTGCTGCATGAGATGACAGCTATA
CGAAAGGCTTGTAACTCTCTCCAAGAGAACTATGTTCCTCGTGTCACTTTCGTGATTGTC
CAGAAACGTCACCACACTCGTTTGTTCCCTGAGCAACACGGGAATCGTGATACGACTGAT
AAAAGTGGCAATATTCAACCAGGTACTGTCGTTGACACTACAATCTGTCACCCGAATGAG
TTCGACTTCTATTTGAACAGCCACGCTGGTATTCAGGGAACAAGCAGGCCGGCTCATTAC
CATGTTCTTCTCGACGAGAACGGTTTCACTGCTGATCAGTTGCAAATGCTCACCAACAAC

CTCTGCTACACGTTTGCGAGGTGCACAAGATCTGTGTCAATTGTGCCACCAGCCTACTAC
GCTCACTTGGCTGCTTTCCGTGCCCGCTACTACATGGAG---------------------

>Cpapaya_evm.TU.supercontig_75.90
ATATCAGTTGGGAGATTCCTCTATTCTCCTGAGCTCAAGAAGCCGCAGCAGCTGGGCGGA
GGACTGGAGTCATGGCGCGGATTTTACCAGAGTATACGGCCTACCCAGATGGGATTATCA
TTGAATATTGACATGTCAACGACAGCATTCATTGAACCTCTTCCGGTTATTGAATTTGTT
GCCAAATTACTGGGTAAAGACGTGTATTCAAGGCCGTTATCTGACGCAGATAGAGTTAAG
GTGAAGAAAGCTCTTAGAGGTGTAAAGGTTGAAGTAACACATAGAAGACATGTTCGAAGA
AAATACCGGATTTCTGGGCTGACATCTCAGCCAACAAGGGAGCTAATTTTCCCTCTTGAT
GAGCACATGAACATGAAATCAGTTGTTGAGTATTTCCATGAAATGTATGGTTATACCATT
CAATATGCCCATTTACCTTGCCTTCAAGTCGGAAATCCAAGAAAAATCAACTATnnnnnn
nnnnnnnnnnnnnnnnnnnnnnnnnnnnnnnnnnnnnnnnnnnnnnnnnnnnnnnnnnna
GGGCTGAATGAGAAGCAAATTACTTCGCTTCTAAAAGTTTCATTACTGATTGCTATTCTT
CCAGACAATAATGGTTCTTTGTATGGTGATTTGAAACGAATTTGTGAAACAGATCTCGGG
TTGATTTCTCAGTGCTGTCTCACAAAACATGTTTTCAAGATTAGCAGACAGTATCTAGCA
AACTTGTCACTTAAAATCAACGTCAAGATGGGAGGAAGAAACACCGTGCTTTTAGATGCT
TTAAGCTGGAGAATTCCATTGGTCAGTGACATTCCAACAATTATTTTCGGAGCTGATGTA
ACTCATCCGGAGACAGGAGAGGACTCGAATCCATCAATTGCTGCTGTTGTAGCGTCCCAA
GACTGGCCGGAGGTTACGAAATATGCAGGATTGGTATGTGCTCAGCCTCATCGACAAGAA
CTTATTCAAGATTTATACAAAACTTGGCAGGATCCTCAAGGAGGCACAGTTAGTGGAGGG
ATGATTAGGGATGGTGTAAGTGAAGGACAATTCTACCAGGTTTTGCTATTTGAACTTGAT
GCCATTCGCAAGGCTTGTGCGTCACTAGAACCCGGTTATCAACCTCCGGTAACATTTGTT
ATAGTCCAAAAGCGCCACCATACTAGACTCTTTGCAAGCAATCACAACGACAGAAGTCGT
ACTGACAAGAGTGGGAATATCTTGCCAGGCACGGTTGTGGACTCTAAGATTTGCCATCCA
ACTGAGTTTGACTTCTATCTATGCAGCCATGCAGGAATTCAGGGAACAAGTAGACCTGCT
CATTATCATGTTCTGTGGGATGAGAACAATTTTACTGCTGATGAGATTCAGACTCTGACA
AACAATCTCTGCTATACGTATGCTAGATGCACTCGATCGGTATCAGTAGTGCCTCCTGCA
TATTATGCTCATTTAGCGGCTTATCGAGCTCGATTCTACATGGAA--------------

>Vvinifera_GSVIVT00002671001
CTTGTTGTCCGACAGTCATTTTTTGACAATCTACCTAGGAATTTCACACCTTTGGGAGGT
GGTGTTCTTGGATGTCGAGGTTTCAATTCAAGCTTCAGAGCTACTCAAGGGGGTCTATTC
TTGAATATGGATGTCTCTACCACACTGGTCATACAGCCTGATCCAGTGAGAGATTTTCTC
GTCTCTAATCAAAATGTTAAAGACATGTATCACATTGACTGGAGCAAGGCCAAAAGGATG
CTAAAAAATCTGAGGGTCAAAACTTTACACTCCAATGCAGAGTGGAAAATCAGTGGATTG
AGTGAGAGGACCTGCAGAAATCAAACCTTCTTAATGAAACAAAGAAATGAAGGGCCGgat
gggGATGAAGTCCGAAGTGTTGAAGTAACAGTTTATGACTATTTTGTCAAACATCGAAAA
ATAAGTTTACAGTATTCTGGGGATTTTCCATGCATCAACGTTGGGAGATCAAAGCACCCC
GTCTATATTCCTCTCGAGCTTTGCACATTGGTTTCCTTGCAACGCTATACCAAGCCATTG
TCTACTCAGCAGAGATCTTCACTTGTGGAGAAATCACGACAAAAGTTTATTCTTTGTATT
CTTCCACAGAAGAAGAACTGCGACATCTATGGTCCATGGAAACGGCAATGCCTTTCAGGT
TGTGGAGTTCCCATACAGTGCATTGCACCATCTACACCAgtaGTGAATGATCAATACCTC

ACTAATGTGTTGTTAAAAATCAATGCCAAGCTTGGGGGTTTGAATTCTTTGTTAACTATG
GGATATTGTCCATCCCTTCATTTGATTTCAACGATACCCACTTTAATCCTTGGAATGGAT
GTGTCCCATGGCTCTCCTGGTCGGCCTGATGTGCCGTCTATTGCTGCGGTGGTAAGTTCC
AGGCATTGGCCATCGATTTCTCAATATAGAGCTACAGTCCGTACACAGTCTCCCAAACTT
GAGATGATAGATTCTCTATTCGAGCCTCTGCCTAATTCCAAAGATAGTGGCATAATCAGG
GGCACCCTGTTGGACTTCTATAAAACTTCAGCAAAAAGGAAGCCTGAACACATAATCATT
TTCAGGGATGGAGTCGGTGAATCGCAATTCAACCAAGTCTTGAACATTGAACTGGAACAA
ATAATTGAGGCCTGCAAACTCCTGGATGAGCAATGGCATCCAAAGTTCATGGTGATTATT
GCACAGAAGAATCACCATATAAGATTCTTCCAGAATGGATCCCCGTCTAATGTTCCTCCC
GGGACAATCGTGGACAACACAATATGTCATCCAAGAAACAATGATTTCTATTTGTGTGCA
CATGCTGGGATGATTGGGACAAGCCGGCCTACGCATTACCATGTATTGCTGGATGAGCTT
GGCTTTTCAGCAGATGACTTGCAGCAACTTGTGCATTCCTTGTGTTACGTATATCAGAGG
AGCACCACGGCTGTATCTCTAGTGGCCCCAGTATGCTATGCTCACCTGGCTGCAGCCCAA
GTTGCCCAGTTTATCAAG--

>Sbicolor_Sb01g011870
ACATCGATCTCAAAATCGTTTTTCTCACATgaaGCATTTGGAAATGGTGGGCCTCTAGGA
AATGGTGTGGAATGCTGGAGGGGTTACTACCAGAGCCTACGCCCTACACAGATGGGGTTG
TCCCTTAATATAGATGTTTCTGCAACGTCATTTTTCAAGGCTCAACCTGTTATTGACTTC
GCAGTGGATTATCTGAACCTCCATGATACTAAAAGGCGTTTGTCTGATCAGGATCGCATA
AAACTGAAGAAAGCACTTAAGGGGGTCCGGGTCGCAACCAAGCATAGACATGATATATCC
ATGCGCTACAGGATTACGGGGCTAACCTCGGCTCCATTAAATGATTTGACGTTTGATCAA
GATGGCACAAGGGTGTCAGTTGTGCAGTACTTCAAACAACAATATGACTACTCATTAAAA
TACACTCACTGGCCATGCCTTCAAGCTGGCAGTGCTAGCAAGCAGATCTATTTACCTATT
GAGGTTTGCAGCATAGTTGAGGGACAACGCTACTCGAGTAAGCTGAATGAGAATCAAGTC
AGGAATATCCTGAAGTTGGCCTGTGAGCGATTACTCATCATAATTTTGCCTGATATGAGT
GGTTTTTACGGAAGGATAAAACGACTTTGTGAAACTGAGCTTGGTTTAATCACTCAGTGT
TGTGCGCCTAAGAATGTAAGGAAAGGAGGAAATCAATATCTTGAGAATCTTTCCTTGAAA
ATCAACGTAAAGGTTGGTGGGAGGAACACAGTACTTGATGATGCTTTAAACCGGAGAATA
CCGCTTCTGACAGATTGTCCTACAATAGTCTTTGGAGCTGATGTTACCCACCCATCTCCT
GGGGAAAGTTCATCTCCATCTATTGCAGCAGTCGTTGCATCCATGGACTGGCCACAAGTT
ACAAAGTACAAATGCTTGGTATCTTCACAAGGTCATAGGGTTGAAATTATAAATGGTCTT
TATACAGAAGTGAGAGATCCACAGAAAGGGAATGTCAGAGGTGGAATGATTAGAGATTTG
CTTTTGTCGTTCCACAAGTCAACTGGTTACAAGCCTTCGAGGATTATATTCTATCGTGAT
GGTGTTAGTGAGGGGCAGTTCAGCCAAGTCTTGCTTTATGAAATGGATGCATGTGCAAGT
TTACAGGAGGGGTACCAACCAAGAGTCACATTCGTTGTTGTGCAAAAGCGGCATCATACT
CGCCTGTTTCCTGAAAATCATCGTGCACGAGACCAGACAGACAGGAGTGGAAACATCCTG
CCTGGAACTGTTGTCGATACGAAGATCTGCCACCCCAGCGAGTTTGATTTTTACCTTTGT
AGTCATTCTGGTATTCAGGGAACAAGCCGCCCAGCTCATTATCATGTTCTTATGGACGAA
AATGGTTTCAGTGCTGATGCACTGCAAACCTTGACTTACAATCTTTGCTACACCTATGCC
CGGTGCACTCGTTCAGTCTCTATAGTTCCTCCGGCGTACTATGCACACCTGGGTGCGTTC
CGTGCCCGTTACTACATTGAG---------------------------------------

>Mesculenta_cassava13489.valid.m1

ACAACTGTCGGGAGGTCATTCTTCTCAACTAATTTGGGGCCAAGGGGTGAGCTTGGTGAT
GGTATAGAATATTGGAGAGGATACTATCAAAGCCTTCGCCCAGCCCAGATGGGACTGTCT
TTTAATATTGATGTGTCAGCCAGATCCTTCTTTGAGCCAATGGTGACTGACTTTCTAGCT
AAATACTTTAGGTTGAGAGACATGTCAAGTCCTCTGTTGGAACAAGATCGTATTAAGGTG
AAAAGGGCCTTAAGAGGAGTAAAAGTAGAGCTAAGTCATTGGGATTATGCTAAAAGCTGC
AAGATCATTGATTTATCCAATCAGCCCCTGAATCAAATATTCTTCACTTTTGGAGATAAG
AGTGTGTCAGTGGTTCAGTATTTCCGTGATCGATACAATATTGGTCTTAAATATACAACA
TTGCCTGCTATTCAAGCTGGGAGTGATTCAAAGCCAATTTTCTTGCCTATGGAGGTTTGT
AGAATTGTTGAAGGACAGAGATACTCAATGAAATTGAATGATAGGCAAGTAACTGAATTG
TTAAAAGCAACATGTCAACGCCAGTTGTTGATAATCATTCTACCTGATGTCATGGGTTCA
TATGGGAAAATTAAAAGGATATGCGAAACAGAATTGGGTATTGTATCTCAGTGCTGTCAA
CCAAGTCAGGCTGCAAAGCTACGTAGACCGTATTTTGAAAATGTTTCCCTCAAAATCAAT
GTTAAGGTTGGTGGTCGAAATACTGTGTTAAATGATGCTATTCAAAGGAGTATTCCTCTA
GTGACTGATATTCCTACCATTATTTTTGGTGCTGATGTAACTCATCCGCCTCCTGGGGAG
GATACTAATCCGTCAATAGCAGCAGTAGTGGCTTCAATGGACTGGCCAGAGGTAACAAAG
TATAGAGGAAATGTCTCTGCACAGGCTCATCGTGAAGAAATTATCCAGGATCTCTATAAA
TCATATCACGATCCGGTTAGAGGATTAATTCATTCCGGAATGATCAGGGAATTATGTATA
GCATTTAGGAGAGCAACTGGGCATAAACCCAACAGGATGATATTCTACAGAGATGGTGTT
AGTGAGGGCCAATTTAGCCAGGTTTTGCTACATGAGATGGATGCCATACGAAAGGCTTGT
TGCTCACTTGAGGAGGGATACCTTCCACGTGTTACCTTCATTGTGGTGCAGAAAAGGCAT
CATACACGGCTTTTTCCTGTTCAGGGTGGTGAGACAGATCACAGTGGCAATATTCGACCA
GGCACAGTTATTGATACCAAGATTTGCCACCAGAATGAATTTGATTTCTACCTCAACAGC
CATGCTGGAATTCAGGGAACAAGCAGGCCTGCACACTATCATGTGTTGTATGATGAGAAT
TGTTTCACAGCTGATAAGTTGCAAGTTCTGACTAATAACATGTGTTACACGTATGCAAGG
TGTACTCGCTCTGTTTCCGTAGTTCCTCCTGCCTATTATGCCCACTTAGCTGCTTTTCGG
GCTCGATATTACATTGAG--

>Ppatens_1912837_locus
TCTCCTGTAGGACGCTCTTTTTATTCTCCCAATCTGGGAACTCGACAGCCGCTAGGAGAT
GGTCTGGAAAGCTGGCGGGGCTTCTATCAAAGTATTCGACCAACTCAAATGGGGCTGTCC
CTAAACATTGACATGTCTTCCACAGCGTTCATAGAACCCAAGACTGTGATGGAGTTTATA
GGGGATCTCCTCAACAAGGACGTGACACGTGGTCTATCCGATGCTGATCGCATGAAGATC
AAGAAAGCTCTTCGTGGAGTTAAAGTGGAAGTGACTCATCGCGGAAGTATGCGTCGCAAG
TACCGCATATCTGGGCTTACAAATCAGGCCACCAATGAATTGGAGTTCCCAGTTGATGAT
AATGGTACCTTGAAGTCTGTTACCGACTATTTTCGGGAGACGTATGGCTATGTTATCCGG
CATCCATCTTTGCCTTGCCTCCAAGTAGGGAATGCTCAGCGTCCCAACTATCTTCCCATG
GAAGTCTGCAAGATCGTTGAGGGCCAGCGGTACTCCAAACGGCTGAATGAGCGCCAAATC
ACCGCACTTCTTAAAGTTACTTGTCAGCGACTCCTCATAGCTATTCTACCAGACAACAAC
GGGCCTTTATATGGTGATCTGAAGAAGCAATGTGAGACTGTCCTGGGAGTGGTTTCACAA
TGTTGTTTGACTAAGCATGTCTTCAAGATGAGCAAGCAGTATTTAGCAAATGTGGCCCTC
AAAATTAATGTGAAGGTCGGAGGTCGGAATACTGTTTTGGTAGATGCACTTTCACGCAAG
ATCCCTCTTGTCAGTGATATTCCCACTATAATATTTGGTGCAGATGTTACCCACCCTCAC
CCTGGAGAGGATTTCAGTCCTTCCATAGCTGCAGTGGTAGCTTCGCAGGATTGGCCTGAG
GTCACTAAATATGCCGGATTGGTGTGCGCTCAAGCTCACCGTCAGGAGTTGATTCAGGAT
CTGTACAAAGAGTGGAAGGATCCTCAGAAAGGCTTGATGACAGGAGGGATGATCAAGGAG
CTCCTGATCTCTTTCTGGCGCGCAACAGGTCAAAAGCCTCTTCGCATCATTTTTTACAGG
GATGGAGTTAGTGAAGGGCAGTTTTATCAGGTGCTTCTGTTCGAGCTGGACGCAATCAGG
AAAGCTTGCGCTTCCCTTGAACCGGATTACCAGCCTCCAGTCACGTTTGTGGTTGTGCAG
AAGAGGCACCACACTCGATTATTTGCCAACAATCACAATGATAATCGCTCTACGGATAGA
AGTGGCAACATTTTACCAGGGACTGTTGTGGACTCGAAGATTTGTCACCCTACGGAGTTT

GACTTTTATCTTTGCAGTCATGCTGGAATTCAGGGGACTAGTCGACCTGCACATTATCAT
GTACTATGGGATGAAAATAAATTTTCTGCGGACAGTTTGCAGTCGTTGACAAACAATCTT
TGCTATACGTATGCACGGTGCACACGTTCAGTATCTATTGTTCCTCCGGCTTATTATGCG
CATTTGGCTGCTTTCCGAGCGCGCTTCTACATGGAT------------------------

>Smoellindorffii_15412065_locus
GTTCCCAGTGGACGTAACTTCTTCGATCCGTCATTCGGGAGGCTGGCACTAGAAGGTGGT
TTTGAGGCCTGGAAAGGATTTACCAGAGTGTTCGTCCAACCATGCAAGGCCTCGTGCTG
AACGTCGATTTGTCTGCAGCAGCATTTTACGAGGCATTGCCAGTTCTCGAGTTCCTCAAG
AAGTCACTCCCGTACTTCGACCCAAGCCGAGGCTTGTCGGACGGTGATCGAGCCAAGGCC
AAAAATCTCTTGAACCGGCTCAAGGTCGAAGTCACGCACAGGAACATCCCTCGGAGATAC
AGGATTTCGGGACTGTCTTTGCGGCCAACAAAGGCGTTGACGTTCACAACTGATTCTGGA
CAGGAGGTGAAGGTTGTGGACTACTTTTGGACGACGTACAAGCATAAGATCCAGTATCCC
GAGTTGCCTTGCCTCGAGTTGCAAGGAAGGAAAACTACTTATCTGCCTATGGAAGTGTGC
AAGCTGGCTGCCGGTCAGAAATATCAGGGAAAGCTCAACGAGAGGCAGACAACAAACATG
CTGAGGTTCACGTGTCAGATTCAGCTGGTAGTTTGTCTCATGGACGCCAAGCACCAGATT
TACGGGGACCTGAAGAAGCTGTGCGAGACGGAGCTCGGGCTCGTGACGCAAGTGTGCCTT
AAGAAAAATGTGATGAAGGAGTACAACAGTttgtcaCAGTACCTGGCCAACCTCGCGATG
AAGATCAACGTCAAAGTTGGAGGCCAGAACATGGATCTGGCGCAGGATCTGCGACTCATG
GTTCCATCGATTCTCGGCAATCCAACAATCATCTTTGGAGCTGATGTCTCTCATCCCATG
GCTCGCGACGACACGAGTCCCTCCATCTCCGCGGTGGTGGCGAGCATGGACTGGCCATCG
GCGGTCAAGTATCTCGCTCGCGCGAGATCTCAGAGGGGCAGGGTGGAGATGATCGAGCAC
CTGCATGACATGGTGGTCGACCTCATGAGGGCGTTTTTCACTCACACCAGACTCAAACCG
GAGAGGCTTCTCTTCTTCCGGGATGGTGTGAGCGAGGGCCAGTTCTCCGACGTCCTGAAC
AACGAAGTGCAGGCAATCCGGAGGGCGTTCCTTACCCTCCAGCCGAATggcgacTACTGT
CCCCAGATAACTTTCGTGGTCGTCCAGAAGCGTCACCACACGAGGTTCTTCCCGGCGGAC
AGTAACGTGGTGAGCAACAACGTGAGACCGGGGACCGTTGTGGACACGGAGATCACCCAT
CCTCGAGAGTTCGACTTTTACCTTTGCAGCCACAGGGGTCTCCAGGGAACCAGCCGGCCG
ACGCACTACCACGTCCTGCTCGACCAGAATGGCTTCACGGCCGACCAGCTCCAGACGCTC
GTCAACAGCTTATGCTACACTTATGCCCGGTGTACCAAGGCGGTCTCGGTGATACCTCCG
GCGTACTACGCTCACCTGGTGGCCTACCGGTCTCGGCTCCACGTCGAC------------

>Sbicolor_Sb09g030910
CTTTTGGTTAAGCAATCGTTTTTCTACAACAACCCTTCAAACTTTGTTGACTTGGGTGGT
GGCGTGATGGGTTGCCGTGGATTTCATTCAAGTTTCCGCGGCACACAGAGTGGACTTTCC
CTGAACGTTGATGTCTCAACAACAATGATCGTGAAGCCTGGCCCTGTTATTGATTTTCTT
CTTTCTAACCAGAATGTTAATGATCCTAGCAGAATTGATTGGCAAAAGGCCAAGCGTGCT
CTCAAGGGCTTGAGGATTAGAACCACTCCTGCAAATGCAGAATTCAAGATTTTTGGTCTC
AGCGAGAGGATTTGTAAAGAACAAACGTTTCCGCTGAGGCAGAGAAATGGTAGCAACGGA
GATTGTGATACCATTGATATAACTGTCTATGACTACTATGCGAAGAAAGGAATTGATCTA
AAGTACTCTGGTGATTTGCCTTGTATAAATACAGGGAGGGCAAAGCGTCCAACATATTTT
CCAATCGAGCTATGCTGCCTTGTTCCGCTTCAAAGATACACCAAAGCTTTGTCTACGCTA
CAAAGGTCATCCCTTGTTGAGAAGTCTAGACAGAAGTTCCTCATGTGCCTTCTCCCTGAG

AGGAAAAATTGCGAAGTCTATGGTCCTTGGAAGAGAAAGTGCCTGGCCGAGTTTGGTATT
GTCACACAGTGTCTAGCTCCAACAAGAGTCAATGATCCGTACCTGCTTAATTTGCTGATG
AAGATTAATGCAAAGCTTGGTGGTCTGAATTCGTTGCTGCAAGTTGAAGCATCTCCATCA
ATACCCCATGTGTCGGAAGTACCCACCATCATCTTGGGTATGGATGTGTCTCATGGCCAT
CCAGGACAAGATAGGCCTTCAGTTGCAGCCGTGGTTAGTTCTCGTCAATGGCCTCTTATC
TCTAAATATAGAGCATCAGTGCACACCCAATCCGCCAGACTAGAAATGATGTCCTCCTTG
TTTAAGCCACGGGGTACTGATGATGATGGCCTCATTAGGGAATCACTGATTGACTTCTAC
ACTAGCTCTGGCAAGCGAAAACCAGACCACATTATCATTTTCAGGGATGGAGTCAGCGAA
AGTCAGTTTACCCAGGTCATCAACATTGAACTGGATCAGATAATTGAGGCATGTAAGTTT
CTTGATGAGAAGTGGTCACCCAAGTTCACTGTGATTGTTGCTCAAAAGAACCACCACACC
AAGTTTTTTCAGACCGGATCACCAGACAATGTTCTTCCCGGAACTGTCGTGGACAATAAA
GTTTGCCATCCTAAGAACTTTGACTTCTACATGTGTGCACATGCTGGGATGATTGGAACA
ACAAGGCCGACCCATTATCATGTTCTGCACGATGAGATAGGTTTCTCTGCTGACGAAATG
CAGGAGTTTGTTCATTCGCTCTCTTATGTGTATCAAAGGAGCACAACAGCCATCTCAGTA
GTTGCTCCAATCTGCTATGCCCACCTTGCTGCAGCCCAGGTGGGCACGTTCCTGAAA---

>Mesculenta_cassava43463.valid.m1
CTCCTTGTTCGCCAGAACTTTTTCACAATGATCCAAGGAATTTCGCAGATGTTGGGGGT
GGTGTTCTGGGTTGTAAAGGGTTTCACTCGAGTTTTAGAACCACTCAGGGGGGCTTATCT
CTGAATATTGATGTATCTACAACCATGATAATTCAGCCTGGGCCGGTTGTGGATTTTCTA
ATTGCCAACCAAAATGCAAGAGACCCCTTTCAACTTGACTGGGCTAAGGCAAAACGAACT
CTTAAAAACCTGAGGATCAAAGCTAGCCCCTCGAACCAGGAGTACAAGATAACTGGTCTG
AGTGACAAACCATGCAGAGAGCAAATGTTTCAACTTAAACAAAAAAGGGATGGGGATGGT
GAACCTCTGGAATTAACTGTTTATGACTATTTTGTTAATCATCGTCATATAGATTTGCGC
TATTCTGCTGATTTGCCTTGCATTAACGTTGGGAAGCCAAAGCGCCCTACTTATATTCCG
ATTGAGCTTTGTACTTTGGTGTCCTTGCAACGATACACGAAAGCACTCAATACTCTCCAA
AGAGCTTCCCTGGTGGAGAAATCAAGGCAAAAATTCCTATTGTGTTTACTCCCGGAGAGG
AAAAACTCAGATATATATGGCCCTTGGAAGAAGAAAAATCTTGCTGAGTTTGGAATTGTC
ACCCAGTGCCTGGCTCCTCAAAGGGTCAATGACCAGTATCTGACTAATCTTCTGTTGAAG
ATCAATGCAAAGCTTGGTGGGTTAAATTCTATGTTATCAGTTGAACATACTCCTTCTATT
CCGGTTGTTTCTAAGGTTCCTACAATCATACTTGGGATGGATGTATCACATGGCTCTCCT
GGGCATTCTGATTGCCCGTCCATTGCTGCGGTGGTCAGTTCCAGGAATTGGCCATTGATT
TCTCGTTACAGGGCATCAGTTCGCACTCAGTCTCCAAAGGTGGAAATGATAGACTCTCTA
TACAAACGAGTGTCAGAAACTGAGGATGAAGGAATGATTAGGGAGCTTCTGTTGGATTTT
TATACCAGTTCAGGTAAAAGGAAACCAGATCAGATCATCATATTCAGGGATGGTGTAAGT
GAATCTCAATTTAATCAAGTATTGAATATTGAACTGGATCAGATAATTGAGGCATGCAAG
TTTCTTGATGAGAAATGGAACCCCAAGTTTGTGGTGATTGTAGCTCAGAAGAACCACCAT
ACAAAATTCTTCCAAAAGGGATCTCCTGATAATGTTCCACCTGGTACTGTCATAGACAAT
AAAGTCTGTCATCCAAGGAACAATGATTTCTATCTCTGTGCTCATGCAGGAATGATTGGT
ACTACAAGACCCACACATTACCATGTTTTATTAGATGAGGTTGGCTTTTCAGCAGACGAT
CTTCAGGACCTAGTGCATTCTCTATCATATGTTTATCAAAGAAGTACAACTGCCATTTCT
GTAGTTGCGCCAATATGCTATGCTCACCTGGCTGCCACCCAGATGGGATCGTTCATGAAG

>Vvinifera_GSVIVT00031924001
ATATCTTCTGGTCGGAGCTTTTACCAATTTAAAGACTCAGGAAAAGACGAGCTTGGATAC
GGTATTATAGCTTCTAGAGGATTTCAACATAGTCTCAAACCCACTGCCCAGGGTCTATCC
TTGTGCTTGGACTACTCGGTTGTGCCATTTTTTAATCCATCGGTTTTAGAGTTCCTAAAG
GAGCATGTTTGTGACTTCTCTTTACGAGAGTTTAAGAGATACAGGAGTGAGGTTGAGGCT
GCCTTAAAGGGATATAAAGTTAGAGTGACTCACCGTAATACGGGTCAAAAATTCATTGTT
GCAGGTTTAACTAGTGAAGACACGCGAAACCTGTCATTTCTTCCTGAAGATCCAGAAGGC
AATGTTTTGCCAAAGAAAGTAATGCTTGTTGATTATTTCTATGAAAAGTATGGCAAGGAT
ATCGAGAACCAGGATATTCCCTGCTTAGATGTGGGAAAAAACAATAGGAAGAATTATGTA
CCAATGGAGTTCTGCATCTTGGTTGAGGGGCAGAGGTATACAAAAGAGATTTTGGATAAA
GAGGCTGCTAAGAGGCTGAAACATGTGCAACTTCAAATTCTTGTGTGTGTCATGGCTAGG
AAGGATGCTGGCTATGGATATCTTAAGTGGTTTGCTGAGACCAAATTGGGGATGGTTACT
CAGTGTTGTTTGTCCCGCCCCGCCAACAAAGTCAGTGATCACCATCTTGCCAACCTTGCT
CTCAAGTTGAATGCTAAGCTAGGGGGTAGCAATGTAGAACTTATAAAACGACTTCCACGG
TTTGAAGGTGAAGGGCATGTGATGTTTATTGGTGCTGATGTCAATCACCCTGGCTCTCAG
AACACAACTAGTCCATCAATAGCAGCTGTTGTTGCCACAGTGAATTGGCCTGCAGCAAAC
CGCTATGCAGCTCGAATTCGCCCACAAGCCCATCGAATGGAGAAAATTCAGAATTTTGGG
GCAATGTGCCTGGAGCTTGTTGAGACTTATGTTCAGGCAAATAAAGTCAAGCCAGAGAAG
ATCGTGGTGTTCCGTGATGGTGTAAGTGAGGGCCAATTTGACATGGTTCTGAATGAAGAA
TTACTTGATCTCAAGAGAGCAATCCAGGGGGAAAATTACTGCCCGACCATCACTCTTATT
GTGGCCCGCAAGAGACACCTAACACGTTTGTTTCCTAAGGTAAATGATGGGAGCTTTAAT
GGGAATGTGCCTCCAGGCACTGTTGTGGACACAACAGTGGTCCACCTATCTGAGTTCGAC
TTCTATCTTTGCAGCCACTATGGTACACTTGGGACAAGCAAACCCACGCACTATCATGTC
CTATACGATGAGCACAGGTTTAGTTCTGACCAGATCCAGAAGCTTACCTATAACTTGTGT
TTCACCTTTGCTCGGTGTACAAAACCCGTCTCGCTGGTCCCCCCAGTGTACTATGCTGAC
CTCGCTGCCTATAGAGGAAGGTTGTACTATGAT---------------------------

>Ptrichocarpa_POPTR_0015s15780
ATCTCTGTTTCCCGTAGCTTCCATTCTGTTAGAGATCATCAAATTCACCTTGGGCATGGA
ATCATAGCATCTAGAGGGTGCCAACATAGCCTCAAACCGACCTCCCAAGGCATAGCTTTA
TGCTTGGACTATTCTGTTCTGTCATTTCATGAGCCACTTTCTGTGATAGAATTCTTGACA
AAACATATTTCTGGGTTTAATTTAAATAATTTTAGAAGCTTTAGGAGGGCTGTGGAAGGT
GTGCTGAAGGGACTGAAAGTTAGAGTGACTCACCGTGTCACCAAACAAAAATATGTTATT
ACAGGGTTGACGAGAGATGACGCTCAATATATTACATTTCCTCAAGAAGATCCAGATGGC
AAGGCTTCGCAGAATGTTAGGCTTGTTGAATATTTCAGGCAAAAATATCACAGAGATATA
GTGCATCAAGATATCCCTTGCCTAGAGATGAAAAGCAAGATGAAGAACTATGTACCAATG
GAGTTCTGTGTCTTGGTTGAGGGGCAAGTATTTCCAAAAGAGTATCTGAAGGAAACTGAA
GCCAAGATGTTGAAGAAATTCTCACTAGCACAATTTCTTCTCTGTGTCATGTCAAGGAAG
GATCCTGGTTACAAGTATCTCAAATGGATTTCTGAAACCAAAGTTGGTATTGTGACACAA
TGTTGTTTGTCCACTCCTGCAAATGAAGGAAAGGATCAGTACCTTGCTAATCTTGGTCTC
AAGATTAATGCTAAGCTTGGAGGAAGCAATGCAGAGCTCAGTGGCAGACTCCCGTACTTT
GGGAATGAAAACCGTGTCATGTTTATTGGGGCTGATGTCAATCATCCTGGTGCTCAAAAC
AAAACTAGTCCATCCATTGCAGCTGTTGTTGGTACTATAAATTGGCCAGCTGCAAACCGC
TATGCAGCTCGAGTTCGTCCTCAGTATCATCGTAAAGAGCAGATTCTGAATTTTGGTGAT
ATGTGCTTGGAGCTTGTTGAATGTTATTCCCGGCTCAATAAAGCGAAACCTGAGAAGATT
GTGATTTTTCGTGATGGGGTCAGTGAGGGCCAGTTTGATATGGTTCTTAATGAAGAGTTA
ACGGACATAATGAAGGCATTCAAGTCAATCAATTACACCCCAACCATAACACTCATTGTT
GCCCAGAAACGGCACCAGACTCGTCTTTTTCCTGGGGACGAGGGATCTACTGGCAATGTG

TCTCCAGGCACAGTTGTGGACACAACAATTGTTCATCCTTTTGAGTATGACTTTTACCTC
TGTAGCCACTACGGAAGTCTTGGGACAAGCAAGCCCACGCATTATTATGTTCTATGGGAT
GAGCATGGCCTCAGTTCGGACGACTTGCAGAGGCTCATATACAACTTGTGCTTCACATTT
GCTCGTTGCACAAAACCTGTGTCATTAGTCCCACCAGTCTACTATGCTGACCTTGTTGCT
TATAGAGGAAGGCTTTACCATGAG----------------------------------

>LOC_Os02g45070
atgatgagaaagaaaaaaactgaacccCGTAATGCTGGGGAAAGTTCTGGAACTCAACAA
GCCACTGGAGCTCCTGGACGGGGTCCTTCACAGCGACCTGAGAGAGCTCAACAGCATGGA
GGTGGTGGTTGGCAACCTGCCAATCCTCAATATGCTCAACAAGCTGGTCGTGGTGGTGGA
CAACACCAGGGACGTGGTGGACGTTACCAGGGTCGTGGAGGGCCAACATCACATCAACCA
GGTGGTGGTCCGGTTGAATATCAAGCACATGAGTACTATGGCCGTGGTGTCCAACGGCAA
GGAGGAATGCCACAACACAGGAGTGGCAGTGGTGGACATGGAGTTCCTGCCAGTCCATCA
AGAACAGTTCCCGAGCTGCACCAAGCCTCACAAGACCAGTACCAAGCTACGGTGGTTGCA
CCATCACCATCAAGAACTGGCCCATCTTCGCTGCCTGTTGAGGCCAGCAGCTCTCCAGTT
GCAAGGTCATTTTATTCGCCTAACTTAGGAAGGCGCCAACAACTTGGCGAGGGCCTGGAA
AGTTGGCGTGGTTTTTACCAAAGCATACGACCCACGCAGATGGGACTTTCTCTGAATATT
GATATGTCATCGACAGCATTCATTGAGCCTCTACCTGTGATTGACTTTGTTGCACAGCTT
TTGAACAGAGACATCTCAGTTAGACCATTATCTGATGCTGATCGTGTGAAGATCAAGAAG
GCCCTAAGGGGTGTAAAGGTTGAGGTCACACATAGAGGCAATATGCGCAGGAAGTATCGC
ATTTCTGGCCTTACCTCGCAAGCAACACGAGAGTTGTCTTTTCCCATTGATAATCATGGT
ACTGTGAAGACGGTGGTGCAATACTTCCAGGAGACATATGGATTTAACATTAAGCACACA
ACTTTGCCTTGCTTGCAAGTGGGCAATCAACAAAGGCCAAATTATCTACCAATGGAGGTC
TGTAAGATTGTGGAGGGACAGCGTTACTCAAAAAGACTAAATGAGAAGCAGATAACTGCT
CTTCTTAAAGTGACCTGCCAGCGCCTGCTGATTGCAATATTGCCTGACAATAATGGTTCT
CTTTATGGCGATCTCAAAAGGATATGTGAGACTGATCTTGGATTGGTCTCGCAATGCTGT
CTTACGAAGCATGTTTTTAAGATGAGCAAACAGTATTTAGCAAACGTTGCCCTTAAAATC
AATGTTAAGGTGGGAGGAAGAAATACAGTACTTGTTGATGCTTTGACAAGGAGGATTCCC
CTTGTGAGTGATAGGCCAACGATCATTTTTGGTGCTGATGTTACACATCCCCATCCTGGA
GAAGATTCTAGCCCTTCCATTGCAGCTGTTGTTGCTTCTCAAGACTGGCCTGAGGTCACC
AAATATGCCGGATTAGTGAGTGCACAGGCCCATCGTCAGGAATTGATACAGGATCTTTTT
AAAGTATGGAAAGATCCTCAAAGAGGAACTGTAAGTGGTGGAATGATCAGAGAGCTTCTC
ATTTCTTTCAAGAGGGCAACTGGACAGAAACCCCAGAGGATTATATTTACAGGGATGGT
GTCAGTGAAGGACAGTTTTATCAGGTTCTGTTTTATGAGCTTGATGCCATTAGAAAGGCA
TGTGCATCTTTGGAAGCCGATTATCAGCCACCCGTTACCTTTGTGGTGGTCCAGAAGCGT
CATCACACAAGGCTGTTTGCTAATAACCACAAGGACCAGCGCACTGTTGACAGAAGTGGA
AATATACTACCAGGCACCGTGGTTGATTCAAAGATATGCCATCCTACTGAGTTTGATTTC
TACCTGTGTAGCCATGCGGGCATTCAGGGAACAAGTCGCCCTGCACATTATCATGTTCTG
TGGGATGAGAACAAGTTCACTGCTGATGGTTTGCAAACTCTGACAAACAACTTGTGCTAC
ACCTATGCAAGGTGCACCCGTTCCGTATCAATCGTTCCTCCTGCATATTATGCCCATCTC
GCGGCATTTCGAGCTCGATTTTACATGGAG------------------------------

>AT2G27040.1
TTGCTTGTTCGACAGTCTTTTTTCCACAATGATCCAACCAACTGTGAACCAGTTGGTGGT
AACATCTTAGGATGTAGGGGATTTCACTCCAGTTTCAGAACAACGCAGGGTGGCATGTCA
CTTAATATGGATGTTACAACCACCATGATCATCAAGCCTGGTCCAGTGGTTGATTTCCTA
ATTGCTAACCAAAATGCTAGGGACCCTTATTCGATTGACTGGTCTAAGGCTAAACGAACC
CTTAAGAACCTAAGGGTAAAGGTCAGCCCCTCAGGCCAAGAATTCAAGATAACCGGATTG
AGTGACAAGCCTTGCAGGGAACAAACGTTTGAATTGAAGAAAAGGAACCCAAATGAAAAT
GGAGAGTTCGAAACTACTGAAGTTACAGTTGCTGACTACTTCCGCGATACAAGGCATATT
GATTTGCAATATTCTGCGGATTTGCCTTGCATCAATGTTGGGAAGCCAAAGCGACCCACT

TACATTCCTCTCGAGCTCTGCGCGTTGGTTCCACTTCAGAGGTACACAAAAGCACTTACC
ACGTTCCAAAGATCTGCCCTTGTTGAGAAATCCAGACAGAAATTCATACTATGTGTGCTC
CCTGACAAAAAGAACAGTGATCTCTATGGTCCATGGAAGAAAAAAAACTTAACTGAATTT
GGCATTGTTACTCAATGCATGGCTCCAACGCGGCAACCTAATGATCAGTATCTTACTAAC
TTACTTCTGAAGATTAATGCAAAGCTTGGAGGCCTGAACTCAATGTTAAGTGTAGAGCGT
ACACCTGCGTTCACTGTGATTTCTAAGGTTCCAACCATTATCCTTGGGATGGATGTTTCA
CATGGATCTCCTGGACAGTCTGATGTCCCGTCCATCGCTGCTGTGGTGAGTTCTAGGGAG
TGGCCACTGATATCCAAATATAGAGCATCTGTTCGGACACAGCCTTCTAAGGCTGAGATG
ATTGAGTCCCTTGTCAAGAAAAATGGAACTGAAGACGATGGCATTATCAAGGAGTTGCTG
GTAGATTTCTACACCAGCTCGAATAAGAGAAAACCAGAGCATATCATAATTTTCAGGGAT
GGTGTGAGTGAATCTCAATTCAATCAGGTTCTGAATATTGAACTTGATCAGATCATCGAG
GCTTGCAAGCTCTTAGACGCAAATTGGAACCCAAAGTTCCTTTTGTTGGTGGCTCAAAAG
AATCATCATACCAAGTTCTTCCAGCCAACGTCTCCTGAAAATGTTCCTCCAGGGACAATC
ATTGACAACAAAATATGTCACCCAAAGAACAATGATTTCTACCTCTGTGCTCACGCTGGA
ATGATTGGAACTACCCGCCCAACTCACTACCACGTCCTGTATGATGAGATTGGTTTTTCA
GCTGACGAACTTCAGGAACTTGTCCACTCGCTCTCCTATGTGTACCAAAGAAGCACCAGT
GCCATTTCTGTTGTTGCGCCGATCTGCTATGCTCACTTGGCAGCTGCTCAGCTTGGGACG
TTCATGAAG---

>LOC_Os04g52540
ATTATCATTGGTCAGGGATTTTACTCGCAGGGCCGCAGTGTGCCCATTGGGCCGGATGTT
GTAGCTCTCAAAGGAACCCAGCAGACCCTGAAATGCACTCAGAAAGGACTGATCCTTTGT
GTGGACTATTCGGTTATGCCGTTTCGCAAAGCTCCTGTGTTGGATCTTGTTCAGAAGTCT
GTGAGATACCTTGACTACAGGACAACACTAAACAAACACCAATTGGACACTTTGAAGAAT
GAACTCAAAGGCCAGCGTGTCACTGTAAATCATAGGAGGACAAAGCAGAAGTACATTGTT
AAAGGTTTGACTGATAAACCTGCAAGTCAGATAACTTTTGTAGATTCTGAATCAGGACAG
ACCAAGAAGCTTCTTGATTACTATTCGCAGCAGTATGGCAAGGTTATTGAGTATCAAATG
CTTCCATGCTTGGATTTGAGCAAGAGCAAGGACAAGCAAAACTATGTGCCGATTGAATTG
TGTGATCTTCTTGAAGGGCAGAGATACCCAAAAGCAAGCTTAAATAGGAATTCTGATAAA
ACACTGAAAGAAATGGCTTTGATCCAGCTCCTCTTCTGCCCAATGTCTGATCAGCATCCT
GGGTACAAGACGCTGAAGCTTATCTGCGAGACGCAGCTGGGGATCCAGACCCAGTGCTTC
TTGAGCTTCCTCGCGAACAAACAACAGGGACAGGACCAGTACATGTCCAACCTTGCTCTG
AAGATCAACGGCAAGATTGGAGGAAGCAACATCCAACTGTTTGGTGAATCGCTCCCGCGG
ATCTCCGGCGCGCCATACATGTTCATCGGCGCCGACGTGAATCACCCATCGCCGGGGAAC
GTCGAGAGCCCGTCGATTGCAGCAGTGGTGGCCTCGGTGGATCAAGGCGCCAGCAAGTAC
GTGCCAAGAATCCGCGCTCAGCCTCACCGCTGCGAGGTGATCCAGCACCTCGGCGACATG
TGCAAGGAGCTCATCGGCGTGTTCGAGAAGCGGAACCGCGTGAAGCCCCAGAGGATCATC
TACTTCCGCGACGGCGTCAGCGACGGTCAGTTCGACATGGTGCTGAACGAGGAGCTGGCG
GACATGGAGAAGGCGATCAAGACCAAGGACTACTCCCCGACGATCACCGTGATCGTGGCC
AAGAAGCGGCACCACACCAGGCTGTTCCCCAAGGACCTGAACCAGCAGCAGACCAAGAAC
GGCAACGTGCTCCCCGGCACGGTGGTGGACACCGGCGTGGTCGACCCGGCGGCGTACGAC
TTCTACCTGTGCAGCCACAACGGGCTGATCGGGACGAGCCGGCCGACGCACTACTACAGC
CTTCTGGACGAGCACGGCTTCGCCTCCGACGACCTGCAGAAGCTGGTGTACAACCTCTGC
TTCGTCTTCGCCCGCTGCACCAAGCCGGTGTCGCTGGCCACGCCCGTCTACTACGCCGAC
CTCGCCGCCTACCGCGGCAGGCTCTACTACGAG-------------------------

>Mguttatus_mgf006816m
ATACCTTCCGGAAGGTCGTTCTACTCGAGCTCAGTGGGAGGTGCGAAAGAAATCGGAGGT
GGGGCTATTGCACTTAGAGGATTCTTTCAAAGCTTGAGACCTACACAACAAGGGCTTGCT
CTCAACGTCGACTTCTCGGTTACTGCCTTCCACGAGAGCCAGAAACGGCTCGACTTTATG
CGGGACCTATCTTGCAAAAGGGCTTTGAGTTTGACGAGCGAGGAGAAGGATGAAGTGGAG
AAAGCACTCAAGAATATGAGGGTTTTTGTCTGCCATAGAGAAACCATGCAGAGATACCGA
GTTTACGGGTTGACTGAAAAGGTTACGGAAGATCTTTGGTTTCCCGATAGAGATGGGACG
AGTATGAGACTTACGAGCTATTTCAAGGAGCAATATGGGTACGATATACAGTATATGAAT
TTGCCTTGCTTGCAGATTAGTAGAAGGAAGCCGTGTTATTTGCCTATGGAGCTTTGTGTG
ATTTGTGAAGGGCAGAAGTTTCTCGGAAAACTCTCGGATGATCAAACGGCGAAAATACTC
AAAATGGGCTGTCAAAGGCAATTGCTCGTTTGTGTTATGGAGAGGAAACACAAAGGGTAC
GCGGACTTAAAAAGAATTGCCGAAACTAGGATCGGAATAGTGAGCCAATGCTGTCTGTAC
TCGAATCTTGAAAAAATGAGCTCGCAGTTTCTCGCAAATTTAGCTCTCAAGATAAACGCG
AAAGTCGGTGGATGCACTGTCGCCCTCTACAACACACTCCCTTCTCAAATCCCGAGGCTT
TTCAGACAAGAAGATCCCGTTATTTTCATGGGCGCTGATGTCACTCATCCTCATCCATTG
GACGATTCTACCCCTTCCGTCGCCGCTGTTGTAGGCAGTGTGAACTGGCCCGCTTCGAAT
AAATACGTCTCGAGAATGAGGTCGCAAACTCACCGGCAAGAAATAATCGAAGATCTTTGT
AATATGGTTACGGAAATATTGGAGGATTTCTTACACGAGCTCTCAAAACTCCCGACTAGG
ATTGTATTCTTTCGAGATGGAGTAAGCGAAACGCAGTTCCACAAAGTGATGCATGAGGAG
CTGAAAGCTATTAAAGAGGCTTGCTCGAGATTTTCCGATTATGCCCCTCCGATCACTTTC
GCTGTGGTGCAGAAGCGCCACCACACCAGATTATTCCCCACCGAGAATCAGTTACCGCCG
GACGAGAATGTTTCGCCGGGGACAGTGGTCGACAGTGTGATCGTTCATCCGAGGGAGTTC
GATTTCTATCTTTGTAGCCATTGGGGTGTGAAGGGGACGAGCAGGCCGATTCATTACCAT
GTATTATGGGACGAAAACAAATTTACGTCCGACGAAGTGCAGAAGTTGGTCTACAATCTT
TGCTACACGTTTGTGAGGTGTACGAAGCCTGTATCTATTGTGCCTCCCGTTTATTATGCG
CATCTTGCTGCATATAGAGGCAGCTTGTATCTCGAC------------------------

>AT1G31290.1
ATCACTGTTGGTAAAAGATTCTTTAGTACTCGGCTAGAGATTGACTTTGGATACGGAGTT
GGAGCTGCGAAAGGGTTTCACCACACTCTCAAGCCCACAGTACAAGGTTTATCATTATGT
TTGAACTCCTCTTTGTTGGCGTTCCGCAAAGCAATTTCAGTCATCGAATACCTGAAGTTG
TACTTTGGGTGGAGAAATATACGTCAGTTTAAGAATTGTAGGcctGATGACGTGGTACAA
GAATTGATTGGTTTGAAAGTCACTGTTGATCATCGAAAGACCAAACAGAAATTCATCATT
ATGGGGTTGAGTAAGGACGACACAAAAGATATCAAATTCGATTTTATTGATCATGCTGGA
AACCAGCCTCCAAGGAAAATATCCATTGTTGAGTATTTCAAGGAAAAGTATGGAAGAGAC
ATTGATCACAAGGATATTCCTTGCTTGAATTTGGGGAAAAAGGGTCGGGAAAATTTTGTA
CCCATGGAGTTCTGTAACTTAGTCGAGGGGCAGATTTTTCCAAAAGAGAAATTGTATAGA
GATTCAGCCGCGTGGTTAAAGGAGCTTTCACTAGTCCTTGTTCTGTGTGCTATGACTGGG
AAGCACGATGGATACAAGACTCTGAAATGGATAGCCGAGACCAAACTTGGTCTAGTGACT
CAGTGTTTCTTGACCATATCTGCCATTAAAGGAGAAaccgtttctGATCAGTACTTGGCA
AATCTCGCCCTCAAGATAAACGCAAAGGTTGGTGGAACGAACGTGGAGTTGGTGGATAAT
ATTTTCTCTTTCTTCAAAAAAGAAGATAAGGTCATGTTCATTGGTGCTGATGTCAATCAT
CCCGCTGCTCACGACAATATGAGTCCATCCATTGTTGCTGTTGTAGGCACTCTTAACTGG
CCTGAAGCTAACCGCTACGCAGCTAGAGTCAAAGCTCAGAGTCACCGTAAAGAAGAGATA
CAAGGGTTTGGTGAAACTTGCTGGGAGCTTATCGAAGCTCATTCTCAGGCCCCCGAGAAA
CGACCTAACAAGATTGTGATATTCCGTGATGGTGTCAGCGATGGTCAGTTCGATATGGTT

CTCAATGTGGAGTTACAGAATGTTAAGGACGTTTTTGCCAAGGTTGGCTATAATCCGCAG
ATAACTGTAATTGTGGCACAGAAACGTCATCAAACCCGTTTCTTCCCCGCCACAACTAGC
AAAGATGGAAGGGCTAAGGGCAATGTGCCTTCAGGTACGGTCGTTGATACCACGATCATT
CACCCGTTTGAGTATGATTTCTACCTCTGTAGTCAACATGGAGCGATAGGTACAAGCAAA
CCAACTCATTATTATGTTCTTTCGGACGAAATCGGGTTCAACTCGAATCAGATTCAGAAA
CTCATCTTTGACTTGTGCTTCACGTTTACTCGCTGCACCAAACCGGTCGCTCTGGTTCCT
CCGGTTTCTTATGCTGACAAGGCTGCTTCTAGAGGAAGGGTGTACTACGAG---------

>Vvinifera_GSVIVT00020067001
TGTCCTGTTGGGAGATCCTTCTTTTCTCCTGATATTAGAGCACCTCAACGTCTTGGAGAA
GGTTTGGAGTCATGGTGTGGATTCTACCAGAGTATAAGACCTACTCAGATGGGTTTGTCA
CTGAATATTGATATGTCTTCGGCTGCATTCATTGAAGCTCTCCCTGTAATAGAGTTTGTT
GGTCAGCTATTAGGCAAAGATGTGTTATCTAGGCCATTGTCTGATTCTGACCGTGTGAAG
ATCAAAAAGGCCCTTAGAGGAGTAAAAGTTGAAGTAACACACAGAGGGAATGTACGAAGA
AAGTATCGTGTATCAGGACTAACATCCCAACCTACAAGAGAACTAGTTTTTCCTGTTGAT
GATAATTCAACCATGAAGTCAGTTGTGGAATACTTCCAAGAAATGTATGGCTTTACAATT
CAACATGCACACCTTCCTTGCCTTCAAGTAGGAAACCAGAAGAAGGCTAACTATTTACCT
TTGGAGGCCTGCAAAATTGTAGAGGGGCAGCGGTATACCAAAAGGTTGAATGAGAGGCAA
ATTACTGCTCTATTAAAAGTTACATGCCAAAGACTTCTATTAGCTATTTTACCTGACAAC
AATGGGTCTTTATATGGTGATCTTAAGAGAATATGTGAAACCGACCTTGGTCTAATATCA
CAATGCTGTCTCACAAAACATGTCTTCAAGATTAGCAAGCAGTACTTAGCCAATGTGTCT
CTGAAGATTAATGTTAAGATGGGTGGTAGAAACACTGTCCTTTTGGATGCTATCAGTTGC
AGGATACCATTAGTTAGTGACATACCAACCATAATATTTGGAGCAGATGTGACGCATCCA
GAGAATGGAGAAGACTCCAGCCCTTCAATTGCTGCTGTAGTAGCTTCTCAGGACTGGCCT
GAAGTCACAAAATATGCAGGATTGGTTTGTGCTCAGGCTCACAGACAGGAACTGATACAA
GATTTATACAAGACATGGCATGATCCTGTTCGTGGCACAGTTAGTGGTGGCATGATTCGG
GATCTTTTGGTTTCCTTTCGGAAGGCGACAGGGCAGAAGCCACTAAGGATTATATTTTAC
AGGGATGGTGTTAGTGAAGGGCAATTTTATCAAGTGCTACTTTATGAACTAGATGCAATC
CGGAAAGCTTGTGCTTCTCTAGAACCAAACTATCAACCACCAGTTACTTTTATTGTGGTT
CAAAAACGACATCATACCCGATTGTTCGCTAACAACCACAGGGACCGGAACAGCACTGAC
AGAAGCGGGAATATTTTGCCTGGCACAGTGGTTGATTCTAAAATTTGCCATCCGACTGAA
TTTGATTTTTATCTCTGCAGCCATGCTGGTATTCAGGGGACAAGTAGGCCTGCTCATTAC
CATGTTTTATGGGATGAGAACAATTTCACAGCAGATGGAATTCAATCCTTGACGAACAAT
CTTTGCTACACATATGCAAGGTGCACACGATCGGTGTCTGTTGTTCCTCCAGCATATTAT
GCACATTTAGCAGCATTTCGAGCCCGATTCTACATGGAG---------------------

>Smoellindorffii_15408087_locus
TCGCCTGTTGGACGATCGTTTTATTCACCAGACTTAGGACGTACACAATCCCTCGGTGAC
GGCTTAGAAAGCTGGCGAGGGTTCTACCAGAGTATAAGGCCCACGCAAATGGGTCTTTCT
CTGAATATTGACATGTCATTTACTGCTTTCATTGAGCCGCTGCGCGTGGTGGACTTTGTT
GGCAAACTCCTTAACAAGGACGTAAGCAGGCCACTTTCGGACGCTGACCGCATCAAGATC
GAAAAAGCTCTGAGAGGGGTCAAAGTGGAAGTTACACATCGCGGAACCATGCGACGAAAA
TACAGGATTTCTGGCTTAACTTCTCAGCCTACTCAAGAACTAATGTTTCCTGTTGATGAT

AGAGGTACAATGAAATCGGTAATGGAGTATTTCCGAGATACATATCACTACACTATACGA
AGCCCATCTTTACCTTGTTTACAAGTTGGAAATCAAGAAAGACCAAATTATCTGCCAATG
GAGGTCTGCAAGATTGTGGAAGGACAAAGGTATACTAAACGTCTCAACGAGCGTCAAGTG
ACAGCCCTTCTAAAAGTAACATGCCAACGGTTACTGATTGCCATATTACCTGACAACAAT
GGCTCACTTTATGGCGACTTGAAACGAATTTGCGAAACAGACCTGGGATTGGTTTCTCAA
TGCTTCTTGACAAAGCACGTCTTTAAGAGAGGAAAGCAGTGTCTTGCTAATGTTGCGTTG
AAAATCAATGCCAAGGTCGGAGGCAGAAACACTGTCTTGGTGGATGCACTATCTCGGAGG
CTTCCTTTAGTAAGTGATACGCCAACAATTATATTTGGAGCGGATGTTACGCATCCTCAT
CCTGGAGAAGATTCGAGTCCCTCGATTGCAGCGGTAAGTGATTGGCCAGAAGTCACGAAA
TACGCAGGTCTAGTGTGCGCTCAAGCTCACAGACAAGAGTTGATCCAAGATTTGTATAAA
ACATGGGTTGATCCTCAGAAGGGAACCATGAATGGCGGTATGATAAGAGAGCTTTTAATT
TCTTTCCGAAGTGCCTCTGGATACAAACCTGGAAGAATCATCTTCTACAGAGATGGTGTG
AGCGAAGGACAGTTCTACCAAGTTCTCCTTCACGAGCTGGATGCAATTAGAAAGGCTTGC
GCGTCACTCGAGCCGAACTATCAGCCTCTCGTGACGTTCGTTGTGGTCCAGAAACGGCAC
CACACCCGGCTATTTGCTAACGATCACGACGATACAAGAACCACTGATAAGAGTGGAAAT
ATCCTGCCAGGCACTGTGGTGGATTCGAAGATTTGCCATCCAACGGAGTTTGACTTCTAC
CTTTGCAGCCATGGCGGCATTCAGGGAACAAGCAGACCAGCACACTATCACGTACTTTGG
GACGAGAACAAGTTCACTGCCGATGGCTTGCAGTCCCTCACCAACAGCCTGTGCTACACT
TACGCTCGCTGCACACGCTCAGTCTCGATAGTACCACCTGCATACTACGCTCACCTGGCT
GCGTTCCGCGCGAGATTCTACATGGAG---------------------------------

>Mguttatus_mgf016601m
GCTGTTGGGAGGTCCTTCTTTCATCCGGAGTTTGAATCTGGGGAGCTTGGTAATGGCCTG
GAATACTGGAAAGGGTTTTATCAGAGTCTTCGCCCAACACAGATTGGGCTGTCACTAAAC
ATTGATATGTCGGCTAGAGCATTTTTTGAACCTTATGTGTCTGAGTTTGTTTTCAAGTAC
CTCAACCTCAGGGATCCGAATAGGCCTTTATCTGACCAAGATCGCATTAAGGTGAGAAGA
GCTCTGAAAGGTGTTAAGGTTGAGAACAATCATCAGGACCATGTAAAGCATCATAAGATA
ACAGGGCTTTCGACTGAACCAACACAAAGGCTTATGTTTTCTGTGGATGAGTCTGGAGCA
CAAATTTCAGTTTATCAGTACTTCCGTCAGAAATACAATATTGTGCTGAAGTATCCACTT
CTGCCAGCACTTCAGGCTGGCAGTGCTGCGAGACCTATTTATCTTCCAATGGAGGTATGC
AAAATTGTCGCGGGGCAAAGATACTCCAAAAAATTGAACGAGAGACAAGTTACTCAACTG
CTCCGAGCCACATGCAAACGTCAGTTGCTTCTTATTATATTGCCCGACGTGACTGGTTCA
TATGGTTTAATCAAGAGGGTGTGTGAAACAGAATTAGACATTGTATCACAATGCTGTCAG
CCTAAACAGGTTTTGAAATGCAATATTCAGTACCTCGAAAATGTATCACTGAAGATCAAT
GTGAAGGCTGGCGGGCGAAATACAGTTTTGGAGCAGGCACTGCTTAGGAAAATGCCTTAC
ATTAGTGACATCCCTACCATAATATTTGGTGCTGATGTCACTCACCCTCAACCAGGGGAG
GATTCCAGTCCTTCTATAGCTGCTGTGGTGGCCTCAATGGATTGGCCAGAAGTCACTAAG
TACAGAGGTTTGGTGTCTGCACAAGGCCACAGGGAAGAGATTATTCAGGATCTCTACACT
AGTTATCAAGATCCTAACAAGGGCTTAGTCCATGGTGGATTGATTCGTGAACATTTGGTT
GCATTCTACAAGAACACTAAGTGCAAGCCTAGCAGGCTCATCTTTTACAGGGATGGTGTG
AGCGAAGGACAGTTTAATCAAGTTCTTCTGTATGAAATTGATGCAATTAGGAAGGCATGT
GCTTCGCTTCAGGCAGATTATCAGCCAAGAATAACTTTTGTTGTGGTTCAAAAGAGGCAT
CATACTCGTCTCTTTCCTGCTGACCATAGAAGTCGTAGTACTACAGACAAGAGTGGTAAC
ATTTTGCCAGGTACTGTGGTTGATACCAAGATTTGCCACCCCAATGAATTCGATTTCTAT
CTTTGCAGCCATGCTGGGATCCAGGGAACTAGTCGTCCAGCACATTACCATGTGTTGTAC
GATGAAAATCGCTTCAGTGCAGATGCACTGCAAATTCTCACCAACAGCTTGTGTTACACA
TATGCAAGGTGCACTCGCTCAGTTTCCATAGTCCCACCAGCTTACTACGCACATCTTGCT
GCATTCCGAGCCCGTTACTACATTGAA--------------------------------

>AT2G27880.1
GTCTCTGTTGGGAGGTCTTTTTTCCACACTAGTTTGGGAAAGGACgcaAGAGATGGTAGG
GGTGAGCTTGGAGATGGTATTGAGTACTGGAGAGGTTATTTCCAAAGTCTAAGGCTGACT
CAGATGGGTTTGTCTCTGAACATTGACGTTTCAGCAAGATCATTTTATGAACCGGTCACT
GACTTTATTAGCAAGTTTCTGAATATAAGGGACTTAAACAGGCCACTTAGAGACTCAGAT
CGACTTAAGGTGAAGAAAGTTTTGAGGACACTGAAAGTTAAGTTGCTTCACTGGAACGGC
ACAAAAAGTGCCAAAATTAGTGGGATTTCTAGTCTACCCATCAGGGAGCTAAGGTTCACT
CTGGAGGACAAATCAGAGAAGACGGTTGTTCAATATTTTGCTGAAAAATATAATTATAGA
GTGAAATACCAGGCTCTACCTGCTATTCAAACAGGGAGTGACACAAGACCCGTCTACCTA
CCAATGGAGCTCTGCCAAATTGACGAAGGGCAAAGATACACCAAAAGGCTCAATGAGAAG
CAAGTGACTGCATTGCTAAAAGCTACCTGCCAACGACTGTTGATTGTAATATTGCCTGAT
GTGACTGGATCATATGGAAAAATAAAAAGGATCTGTGAAACAGAATTGGGGATTGTCTCT
CAGTGTTGCCAACCTAGACAAGTTAATAAACTCAACAAGCAGTACATGGAAAATGTTGCC
TTGAAGATCAATGTCAAGACTGGGGGAAGGAACACTGTTCTTAATGATGCTATTAGAAGA
AACATACCTCTTATTACTGATCGTCCAACCATCATCATGGGTGCTGATGTGACTCACCCA
CAGCCTGGAGAGGACTCAAGTCCTTCTATTGCTGCTGTTGTGGCCTCTATGGACTGGCCT
GAGATAAACAAATACCGAGGATTGGTTTCTGCTCAAGCTCATAGGGAAGAAATTATTCAG
GACCTGTATAAGCTGGTTCAGGATCCACAACGTGGGCTAGTCCACTCTGGTTTGATAAGG
GAACATTTCATAGCATTCAGGAGAGCTACAGGCCAGATACCTCAAAGGATCATCTTCTAT
CGTGACGGAGTAAGCGAAGGGCAGTTTAGTCAGGTTCTGCTACATGAGATGACTGCTATC
CGCAAGGCTTGTAACTCTCTCCAAGAGAATTATGTTCCTCGTGTTACTTTCGTGATTGTC
CAGAAACGTCACCACACACGTTTGTTCCCTGAGCAACACGGGAATCGTGATATGACTGAT
AAGAGTGGCAATATTCAACCAGGTACTGTCGTGGACACTAAAATCTGTCACCCTAATGAA
TTCGACTTCTATTTGAACAGCCATGCTGGTATTCAGGGAACAAGCAGGCCGGCACATTAC
CATGTACTTCTCGATGAGAACGGTTTCACCGCTGATCAGTTGCAAATGCTCACAAACAAC
CTCTGCTACACGTATGCGAGGTGTACAAAATCTGTGTCAATTGTGCCACCAGCCTACTAC
GCTCACTTGGCTGCATTCCGTGCCCGCTACTACATGGAG---------------------

>Mtruncatula_Medtr5g094940
CTGCTTGTGCGCCAAAATTTCTTTCACAACGACCCAAATAATTTGAATGATGTTGGAGGT
GGTGTACTTAGTTGCAAAGGGCTGCATTCCAGCTTTAGAACCACACAAAGTGGATTGTCT
CTGAACATAGATGTGTCGACAACCATGATTGTACGTCCTGGGCCTGTGGTTGACTTCCTA
ATTGAAAATCAAAATGTGAGAGATCCTTTCAGTCTTGACTGGAATAAGGCTAAAAGGACC
CTCAAGAATTTGAGGATCACAGCTAAACCATCTAACCAAGAGTACAAGATAACCGGGCTG
AGTGAATTGTCGTGCAAAGACCAGCTGTTTACCATGAAGAAGAGAGGTGCTGTGGCTGGA
GAGGATGATACAGAAGAGATCACGGTTTATGATTATTTCGTTCATCGGCGAAAAATTGAT
CTACAGTACTCTGCTGGTCTTCCATGTATTAATGTTGGGAAGCCAAAACGACCTACTTAT
ATCCCAATTGAGCTTTGCTCATTGATATCCCTGCAACGATACACCAAAGCCCTCTCCACA
TCTCAACGATCCTCCCTTGTGGAAAAGTCGAGGCAGAAGTTTCTGCTTTGTCTGCTTCCT
GAGAGGAAGAACTCTGATCTTTATGGTCCATGGAAGAAGAAGAATCTTGCGGAGTTTGGA
ATTGTTACCCAGTGCATAGCTCCTACCAGGGTGAACGACCAGTATCTGACCAATGTTTTG
CTGAAGATCAATGCAAAGCTTGGTGGGATGAATTCTTGGTTAGGCGTTGAACACTCTCGA
TCCATCCCTATTGTGTCGAAAGTTCCTACTCTCATTTTGGGCATGGATGTTTCTCATGGT
TCACCTGGGCAACCTGACATTCCTTCAATTGCTGCGGTCGTAAGCTCGAGGAAATGGCCC
CTGATATCTAAATACAGGGCATGTGTCCGAACTCAGGGTTCGAAGGTTGAGATGATTGAC

AATCTGTTCAAGCCAGTGTCTGATAAGGAAGATGAAGGAATTATAAGGGAACTTTTGCTT
GATTTCTTTCATAGTTCTGAAGAGAGACGACCAGAGAATATAATCATTTTCAGGGATGGT
GTTAGTGAGTCTCAGTTCAACGAAGTGTTGAACGTTGAACTCAGCCAAATCATCGAGGCT
TGTAAATTTCTTGATGAAAATTGGAACCCCAAGTTTATGGTGATTGTTGCCCAGAAGAAT
CATCACACAAAATTCTTCCAACCTCGATCTCCAGACAATGTGCCACCTGGAACTGTTGTG
GACAGCAAAATTTGCCATCCTAGGAATTATGATTTTTATATGTGTGCTCATGCTGGAATG
ATTGGTACAAGCAGACCAACACATTATCACGTTCTTTTGGATGAGATTGGTTTTTCTCCA
GATGATTTACAGGAGCTTGTGCACTCTCTATCTTATGTTTATCAGCGGAGCACCACTGCT
ATTTCTGTCGTTGCTCCAATCTGCTATGCACACTTGGCTGCATCTCAAGTTGGACAGTTC
ATGAAG--

>Bdistachyon_Bradi5g21810
cgccttgacggcACGATCCTTGACATCCATACAACTCTAGGCGAGAAACAGTTGGTGCAT
TTGGAACGTCATCTCAAGGGCCTCTACGTTACCCTGAATTACCAGaaatcccctgaagga
aaaagtgatGGGACCACAGCTCGGAAGTACAAGGTTCACGGCCTGACAAAGCAGCTTGCC
CACCAGATCACCTTTCCGGATTTCAAATCAGGGGACCAGCGGAAGCTTCTTGAGTATTAT
CGTCAGCAGTATGGGAAGAGGtatcccAAAGAACAGCTCCTCTTCTGCCCGATGTCTGAG
CAGCATCCGGGGTACAAGACACTGAAGCAGATCTGCGAGACGAAGCTGGGGATCCAGACT
CAGTGTTTGTTGAGCGAAGCCGCGAATAAAGACAACgtccgggacCGGGACCAGTACATG
TCCAACCTTGCTCTCAAGATCAACAGCAAGCTCGGGGGCAGCAATGTCCAGCTACTATCT
GACGGGCTTCCAAAGATGGCTGGCAGCCATTTCATGTTCATCGGCGCGGACGTGAACCAT
CCATCTCCCAACGACAACTTGAGTCACTCGATAGCAGCTGTGGTCGCGTCCATGGATTGC
CCTGGCGCCAGCAAGTACGTGCCTAGAATCCGTGCCCAGAAGAACCGCTGTGAGGAGATC
GTGGAGCTCGGTCAGATGTGCAAAGAGCTCATCCAAGTCTACGAGAAGAAGAACGGTGTC
AAGCCACAGAAGATCATTTACTTCCGCGATGGCGTGAGCGATAATCAGTTCGAGATGGTC
CTGAAACAGGAGCTGAAGCAGCTGGAGAACATGCTGAAGGCGCTCAAGGAGGGCTACTCG
CCGACAATCACAGCGATCGTGGCCAAGAAGCGGCACCACACACGGCTGTTCCCCAAGGAC
GAAGACCGCAACGTGCTCCCTGGCACGGTGGTCGACACCGATGTGGTCAACACGGCAGAC
CAAGACTTCTTCCTGTGCAGCCACGACGGGCTGCACGGGACGAGCCGGCCAACGCACTAC
CACAGGCTCAAGGACGACCACGGCTTCGAGCCCGTCGACCTGCAGAAGCTGGTGTACAAC
ATGTGCTTCCTGTTCGCGCGCTGCACCAAGCCGGTGTCGCTCACGACGCCCGTCAAGTAC
GCCGACCTTGCGGCGTACCGCGGCAGGGACTACTACGAC--------------------

>LOC_Os02g07310
ACCCAATTTGCTGGTTCATTTTACTCACCTAACTTAGGAGAATGCCGACAATTGTGTAAG
GTCTTGGAAAGCTGGCGTGGTTTTCACCAAAGAATACAAGCTACTCAGATGGGACTTCAA
CTGAATATCGACGTGTCATCATCAGTGTTCATCAAGCCTGTTAACTTTAGTATTCAGTAC
CCATCTTTGCCTTGCTTGAAAGTGGCTCATTTTGGGGAGACAATATTTCTGCCATTGGAG

GTCTGTAAAATTGCTGAGGGACAATGTCACCAGAAACAGCTCAATGCAAAACATATGGCT
GCTCTTCTTCAGGTAGCTCGACAGCCCCTGCTGATTGTAATATTGCCTAACAACAATGGA
TCTCTTTATGGTGATGTCAAAAGAATATGTGAGACAGATATTGGACTGATCTCTCAATGT
TGTCTTGCAAAACATGTTCTTAAGATGAACAAGTGGTATCTTGCAAGCGTTGCCCTTAAA
ATCAATGCTAAGATGGGCGGAAGAAATACTGTACTGGTTGATGCTTTAGAAATGAGACTC
CCCCATGTTAGGGATACACCAACTATTGTATTTGGTGCTCATGTCACCCATCCACATCCA
GGAAAAGCTAATAGTTCTTCCATTGCTGCTGTTGTTGCTTCTCAAGACTGGCCCGAGGTT
ACCAAGTATGCTGGTTTAATCAGTGTGCAAGCGTGCCACCAGGAGTCAATACAAGGTCTT
TTTAAAGTCCAGGATGATCCGGAAAGAGGAACCACAACTAGCGGAATGATCAAAGAGCAT
CTCATGTCTTTCTATCGAGCCACTAAACGGAAGCCCGGAAGGATTATATTTTACAGGGAT
GGTGTGAGCAAAGGACAGCTCCCTCAGGCTTTGATGCACGAACTTGGTGCCATCAAAATG
GCGTGTGCATCTATGGGACCCGATTATAATCCATTAGTTACATACGTGGTGCTCCAGAAG
TGTCGCCATACACGTCTGTTTGCTGACTACTATAATGCGAATACTCATGATTCAACTGCA
AACATACGGGCTGGTACTGTGGTTGATTCAAATATTTGCCAACCAAATCAGTTTGATTTC
TACTTGTGTAGCCACCGCAGCACGCAGGGAACTAAGCGGCCCAGGTATTATCATGTTCTG
TGGGACGAGAACGACTTCTTGGCTGGTTCTTTCCAAGAGCTCACAAACTACCTCTGCTAC
ACCTCCGCAACTTGCACCCAATCCATATCAGTTGTGGCCCCTGTGCACTACGCTCGTCTT
CTGTCATCACGAGCTCGATGTTACATTAAA------------------------------

>Mtruncatula_Medtr4g114860
GTTTCTTTAGGACGGTGTTTCTTTCCCACCAATCCTCCCTTAATACAGAGAGATCTTGAA
CCCGGAATAATTGCAATTGGAGGATTTCAACACAGTCTCAAGACCACGGCTCAGGGTTTA
GCTTTGTGTCTTGATTATTCAGTTTTGTCTTTTCGAAAGAAAATGTCAGTCTTGGATTTC
CTCCATGATCATATTAGAGGTTTCAATTTGGCTGAGTTCAGGAAATATAAGAAATTTGTC
GAGGAGGTACTCTTGGGATTGAAAGTAAATGTTACTCACAGAAGAACCAAACAGAAATAT
ACTATTGCTAAGCTAACAGATAAAGATACTCGCCACATCACTTTCCCTATTTTGGACCAA
GAGGGCCAAACCCCCCCTAGAAGCACCTCTCTTCTTGCCTACTTTAAAGATAAACATAAC
TATGATATTCAACACAAAGATATTCCTGCATTGGATTTTGGGGGAAACAAGACTAATTTC
GTGCCTATGGAGCTATGCGTCTTGGTTGAGGGTCAGCGATTTCCCAAAGAGTATTTGGAC
AAGAATGCTGCCAAGAACTTGAAAAATATGTGCTTGGCTCAATTTCTCCTTTGTGTGATG
GCCAACAAGGATCCAGGTTACAAGAGCCTCAAGTGGATTGCTGAGACCAAGGTTGGCATA
GTGACACAGTGCTGCTTATCTGGTAATGCTAATGAAGGGAAAGACCAATATCTCACAAAT
CTTGCTTTAAAAATCAATGCAAAAATTGGAGGCAGTAACGTTGAGCTCATTAATAGGCTC
CCACACTTTGAGGATGAAAGTCATGTTATGTTTATAGGGGCTGATGTCAATCATCCAGGT
TCCCGGGACACAAATAGTCCATCAATTGTTGCAGTGGTTGCCACTACTAACTGGCCAGCT
GCAAATCGCTATGCAGCACGTGTTTGCGCTCAAGAGCATTGTACAGAGAAAATTTTGAAT
TTTGGAGAGATTTGCCTTGACCTTGTTAGACATTATGAGAAGTTGAACAAAGTCAGGCCC
CAAAAAATTGTTATCTTTCGTGATGGTGTTAGTGAGAGCCAATTTCACATGGTTCTTGGC
GAGGAGTTAAAAGATTTGAAGACCGTGTTTCAGCACTCAAATTACTTTCCAACTATCACT
CTTATTGTAGCTCAAAAGCGCCATCAAACTCGATTGTTTCCTGCCGGTGTAAGGGAGGGG
GCTCCCAGTGGAAATGTGTTCCCTGGAACAGTTGTGGACACAAAGGTCGTACATCCTTTT
GAATTTGACTTTTACCTGTGTAGTCACTATGGAAGCCTAGGTACAAGCAAGCCCACTCAC
TATCATGTCTTGTGGGATGAGCACAGGTTTACTTCTGATAATTTGCAGAAGCTCATATAT
GATATGTGCTTTACCTTTGCAAGGTGCACTAAACCTGTATCTTTAGTCCCTCCAGTGTAC
TATGCTGACCTTGCTGCTTACAGAGGAAGACTATACTATGAA------------------

>Mguttatus_mgf009177m
TTGTTGGTTCGCCAGTCGTTTTTCCACAACGACCCGAAGAATTTCTCTGATGTTGGAGGT
GGGGTCCTCGGCTGCCGAGGCTTCCACTCGAGTTTTAGGACAACCCAGAGTGGATTGTCT
TTGAACATTGATGTATCTACAACAATGATAATCCAGCCTGGTGATGTAGCGAACTTCCTT
GTTGCTAACCAAAATTGCAGGGACCCCTTTTCGGTCGATTGGGCAAAGGCTAAACGAACG
CTGAAGAATTTGAGGATCACCGTAAGTCCGACTAACCAGGAGTTCAAAATAACTGGTCTA
AGCGAGAAGTCTTGCAGAGAGCAAACTTTCACATTGAAGCAGAAAAGCAAGGATGGCGAT
GGTGAATTTCAAACAACAGAAGTGACTGTGTACGACTACTTTGTGAACCAGCGGAACATC
GACTTGCGATTCTCTGCTGACTTACCATGCATTAATGTCGGAAAGCCAAAGCGCCCGACT
TACTTCCCAGTCGAGCTATGCTCTCTTGTGTCACTGCAACGCTACACGAAAGCTCTATCT
ACACTTCAAAGAGCTTCATTGGTCGAGAAATCACGCCAAAAGTTCCTACTCTGTTTGCTT
CCCGAGCGTAAAAACTGTGCACTTTATGGCCCGTGGAAGCGCAAAAACTTGTCCGAATTC
GGAGTTGTTACCCAATGCCTGGCTCCACAGCGAGTTAACGATCAGTATCTGACAAACCTT
CTGCTGAAGATAAATGCAAAGCTTGGTGGGTTGAACTCCGTGTTAGCTAGTGAACTTTCG
CCTACAATTCCGATGGTGTCAAAGCTGCCGACATTGATTCTTGGTATGGACGTCTCTCAT
GGTTCGCCTGGACAGTCTGATATTCCATCGATTGCTGCGGTTGTTAGCTCTAGGCAATGG
CCTTCGGTTTCTCGTTACAGAGCATGTGTTCGCACTCAATCTCCAAAGATGGAAATGATT
GATTCCCTGTTCAAACGTGTTTCCGATTCTGAAGATGATGGAATTATGAGGGAGGCTCTG
CTGGATTTCTATGTTAGTTCTGGGAAACGAAAGCCCGACCAAATCATCATTTTCAGGGAT
GGTGTGAGTGAATCTCAATTCAATCAAGTCCTCAATATTGAACTGAGCCAAATTATAGAG
GCTTGCAAATTCCTTGACGAAAAATGGAATCCCAAGTTTGTCGTAATCATTGCACAGAAG
AATCACCACACTAAGTTTTTCCAGCCGAATTCTCCCGATAATGTGCAGCCAGGGACTGTG
ATTGATAACAAAGTTTGTCATCCGAGGAACAACGACTTCTACCTGTGTGCCCATGCGGGC
ATGATCGGAACAACGAGGCCTACACATTACCATGTTCTGCTCGACGAGATGGGTTTTTCT
ACCGATGATTTACAGGAGCTTGTTCACTCACTTTCTTACGTATACCAGCGCAGCACCACT
GCTATATCGATTGTTGCTCCAATCTGCTACGCGCACTTGGCAGCGACTCAGTTGGGGCAG
TGGATGAAG---

>Bdistachyon_Bradi3g51080
gtgGGCATTACCAGGATAGCATCACAGCAACCACCTGGGGGTCCTGTTAAGTATCAAGCA
CATGGGTATTATGGCCACGGTGGCCCAAACCAAAGAGGAATGTCACAACCATACCATGAC
GGGCGTAGGAGTGGCGGTGGTGGAAGAGGAGTTCCTATTACTCCATCAATAACACTTCCC
GAACTGCACCAAGCCCCACAAGTTCAGCACCAAGTTCCTGTGGTTACTCCTTCACCGCGG
GAAACTGGCTCATCCTCACTAGGTGTTGATATGAACTCCCCAGTTGCCAGGTCATTTTAT
TCACCTAACTTAGGGAGGCGCCAGCAACTCGGTGATGGCTTGGAAAGTTGGCGTGGCTTT
TACCAGAGCATACGGCCCACACAGATGGGACTTTCACTGAATATTGATATGTCATCTACA
GCATTCATCGAGCCTTTGCCTGTGATTGATTTTGTTGCACAACTTTTGAACAGAAACGTA
TCAGTTAGACCATTATCAGATGCTGATCGGGTGAAGATCAAGAAGGCTCTACGAGGTGTA
AAGGTTGAGGTCACACATAGAGGCAATATGCGCAGAAAGTATCGTATATCTGGCCTTACC
TCGCAAGCAACAAGAGAGTTAACTTTCCCTATAGATAATCATGGTACTGTGAAGACAGTA
GTGCGATACTTCCAGGAGACATACGGTTTTAACATTCAGCATACTACTTTGCCTTGCTTG
CAAGTGGGCAATCCACAGAGGCCAAATTACCTTCCCATGGAGGTCTGTAAGATTATCGAG
GGACAGCGTTACTCAAAACGACTGAATGAGAAGCAGATAACTGCTCTTCTTAAAGTGACC

TGCCAGCGCCTGCTTATTGTAATTTTACCTGACAATAATGGTTCTCTTTATGGTGATCTC
AAAAGAATATGTGAGACAGATCTTGGATTGGTCTCTCAATGCTGTCTCACGAAACATGTT
TTTAAGATGAACCAGCAGTATCTTGCAAATGTCGCCCTTAAAATCAATGTTAAGGTGGGA
GGAAGAAATACTGTACTTGTCAATGCTTTGTCAAGGAGAATTCCCCTTGTCAGTGACAGA
CCAACCATTATATTCGGTGCAGATGTTACCCATCCTCATCCTGGAGAGGATTCTAGCCCT
TCCATTGCAGCTGTTGTTGCTTCTCAAGATTGGCCTGAGATCACCAAGTATGCTGGATTA
GTGAGTGCACAAACCCGTCGCCAGGAGTTGATACAAGATCTTTTTAAAGTGCAGCAAGAT
CCTCAGAGAGGGAGTATAGCTGGTGGAATGGTTAGAGAACTTCTCATTTCTTTTAAGAGA
TCAACTGGACAGAAACCCCAGAGGATCATATTCTATAGAGATGGTGTTAGTGAAGGGCAG
TTCTATCAGGTTCTGTTGTATGAGCTTGATGCCATTAGAAAGGCTTGTGCATCATTGGAG
CCCAATTATCAGCCACCTGTTACCTTTGTGGTGGTCCAGAAGCGTCATCATACACGGCTC
TTTGCTAATAACCACAATGATCAGCACAGTGTTGATAGAAAAAGTGGAAACATATTACCT
GGAACTGTGGTTGATTCAAAGATATGTCATCCTACCGAGTTTGATTTCTACCTTTGTAGC
CATGCTGGCATTCAGGGAACAAGCCGCCCTGCGCATTACCATGTCCTGTGGGATGAAAAC
AAATTTACTGCCGACGGTTTGCAGACTCTCACGAACAACTTGTGCTACACCTATGCAAGA
TGCACTCGTTCTGTATCAATTGTGCCTCCTGCATACTATGCTCATCTTGCGGCTTTTCGA
GCTCGGTTCTACTTGGAA--

>GRMZM2G007791_T01 cds:_protein_coding
ATTATCCTTGGTAGAGGATTTTACTCACCAAGCAGCAGTATAGACATTGGGCAGGGTGCT
GTAGCTATGAAAGGAACCCAGCAGTCCCTTAAATCCACTCAGCAAGGGTTGATCCTGTGT
GTTGACTATTCTGTCATGCCGTTTTACAAAGCTGTGATGGATCTTGTTCAGAAATTAGTG
AGGTACCTTGATTATCGGACAACTTTGAACAAGAGGCAAATGGAAAATCTGGTTGATGAG
CTTAAAGGCCGACGTGTAACTGTGATTCATCGGAGGACTAATCAGAAGTACACAGTGCAA
GGTTTGACACCCTTACCTGCCAGCCAGATGACCTTTGTGGATGCTGAATCCGGCCAAACA
CGGAGGCTCGTGGATTATTATGCTCAGAAACATGGCAAGGTGATTGAGTATCAGATGCTG
CCATGCTTGGATTTGAGCAAGAGCAAGGACAAACCGAATCATGTTCCAATTGAGCTCTGC
ACTCTTCTTGAAGGACAGAGGTTTCCAAAAGCAAACTTGAATCAGAATTCTGAGAGGATA
CTAAAAGGAAGTGCTCTACTCCTTTTCTGCCCGATGTCCGAGCAGCATTCGGGGTACAAG
ACACTGAAGCTGATTTGTGACACACAGCTGGGGATCCTGACCCAGTGTTTACTGAGCGAC
CGCGCAAACAATCGAAAGGGACAGGACCAGTACATGACGAATCTTGCTCTAAAGATCAAC
GGCAAGCTTGGGGGCAGCAACGTTCAGCTGTTTGACTCGCTCCCACGGGTCGGTGGTGGG
GTACCTTTCATGTTCATCGGTGCTGACGTTAACCACCCGTCCCCCGGTAACGTGGAGAGC
CCATCGATCGCAGCCGTGGTCGCCTCTGTCAACTCTGGTGTCAACAAGTATGTGACCAGA
ATCCGTGCCCAGCCGCACCGCTGCGAGGTGATCCAGCAGCTTGGTGAGATCTGCCGGGAG
CTCATTGGAGTCTTTGAGAAGCAGAACCGCGTGAAGCCGCAGAAGATCATCTACTTCCGT
GATGGCGTGAGCGACGGGCAGTTCGATATGGTCCTGAACGAGGAGCTGGCTGACCTGGAG
AAGGCGATCAAGGTGAATGGCTATGCGCCAACCATCACCGTGGTCGTGGCCAAGAAGCGG
CACCACACTCGGCTGTTCCCCAGGGACGAACAGCAGCCGCAGACGAAGACCGGGAACGTG
CCGCCTGGCACGGTGGTGGACACGGGCGTGGTGGACCCGTCCGCGTACGACTTCTACCTG
TGCAGCCACACTGGGATTCTGGGGACGAGCAGGCCGACGCACTACTACACCCTGGTGGAC
GAGCACGGCTTCGGCTCCGACGACCTGCAGAAGCTGATCTACAACCTGTGCTTCGTGTTC
GCGCGGTGCACCAAGCCGGTGTCGCTGGCGACGCCCGTCTACTATGCCGACCTCGCGGCC
TACCGTGGCAGGCTCTACTACGAG------------------------------------

>Ptrichocarpa_POPTR_0014s15760
CTTTTGGTTAGGCAGTCGTTCTTTCATGATGATTCAAGGAACTTCAATGATGTGGGAGGT
GGTGTAACTGGTGTTAAGGGTTTCCATTCTAGCTTCCGTACCACTCAGGGTGGCTTGTCT

CTTAACATGGATGTGTCCACTACAATGATCCTAACTCCTGGGCCAGTAATTGATTTTCTG
ATAGTTAATCAAAATGTCCGGGAACCTCGCTATGTTGATTGGGTGAAGGCCAGAAGGATG
TTGAAAAATTTGAGGGTGAAGACGAAGCATAACAACATGGAGTTTAAAATAATTGGTCTG
AGTGAGAAGCCATGCAATCAACAATATTTTCCTATGAAACTGAAAAACAGGGACggagct
AATGTTGAAGCACAGATTGTTGAAGTTACTGTATATGATTATTTCACTAAACACTGTGGC
ATACAACTTGGTTATTCTGCATACCTACCCTGCCTTGATGTTGGTAAACCAAAACGTCCA
AACTACCTGCCACTGGAGCTTTGTTCACTTATTTCACTTCAACGGTATAAAAAAGCTTTA
TCTTCAATGCAAAGAGCATCTTTGGTTGAAAAATCACGACAAAAGTTTATTCTTTGTGTA
TTGGCAGAGAGGAAAAACTCAGATATTTATGGACCGTGGAAGAAGACAAGTCTCAGTGAT
TTTGGCATTGTTACACAGTGCATATCCCCGACGAAGATTAATGACCAGTATCTTACAAAT
GTGCTTCTTAAGATCAATTCTAAGCTAGGAGGAATAAATTCTCTGTTGGCAATTGAGCAC
TCCTCACATATTCCATTGATAATGGATACTCCTACAATGATTCTGGGCATGGATGTCTCT
CATGGCTCTCCAGGTCGATCAGACATGCCATCAGTGGCTGCGGTTGTTGGATCTCGATGT
TGGCCACTGATTTCTAGGTACAGAGCATCTGTAAGAACGCAATCTCCTAAGGTGGAGATG
ATTGATGCTTTGTACAAGCCTTTAGCAAATGGGAATGATGATGGTATAATAAGGGAACTG
CTCGTGGATTTCTTTCAAACAAGCAAGGGGCACAAACCAAAACAAATTATTGTGTTCAGG
GACGGTGTCAGCGAGTCACAATTCAATCAGGTGCTGAACATTGAGCTGGAGCAAATTATC
AAGGCCTATCAACATCTTGGTGAGGTTGACATACCGAAGTTCACAGTAATTGTGGCTCAG
AAGAATCACCACACAAAGCTTTTTCAAGCTGGTGGTGGCACTGAAAATGTTCCTCCTGGG
ACAGTTGTTGACACAAAGATTGTTCATCCTAGAAACTATGATTTCTACATGTGTGCTCAT
GCAGGCATGATTGGAACTTCAAGGCCAGCACACTATCATGTCTTGCTCGATGAGATTGGT
TTCTCTCCAGATGAATTGCTAAATCTTGTCCACTCTCTTTCATATGTGTATCAAAGGAGT
ACCACTGCTGTTTCGATTGTGGCTCCCATATGTTATGCTCACCTGGCTGCAGCACAGATA
GGGCAGTTTATGAAG--

>Ptrichocarpa_POPTR_0006s02680
CTTCTTGTTCGCCAGTCATTCTTTCACGATGATCCAAAGAACTATGTTGATCTGGGAGGA
GGTGTCCTTGGATGCCGAGGATTTCATTCGAGCTTTAGAACCTCGCAGGGTGGATTATCC
CTAAATATAGATGGTTCGACTACAACGATAATACAGCCTGGGCCTCTTATTGACTTTCTC
ATAGCCAACCAGAATGTGTCAAACCCCTTTCAGATTGACTGGGCAAAGGCTAAGCGAACA
TTGAAAAATCTGAGGATAAGGGTGTCACCTACCAATCAAGAGTACAGAATCACTGGCTTG
AGTGAAAATACTTGCAAAGAGCAAATGTTCTCTCTGAAATCAAGAGCATCTGATGGAAAT
GATGTTGAAAGTGTTGACATTACAGTTTACCATTATTTTGTAAATCATCGCAGCATAGAT
TTACGCTACTCTGGAGATTTGCCTTGCATCAATGTTGGCAAGCCTAAAAGGCCCACTTAC
ATTCCTGTCGAGCTTTGTTCACTGCTTCCCTTGCAACGCTATATAAAGGCACTAACTGTC
CTTCAGAGATCACAGTTAGTAGAAAAATCAAGACAAAAATTTCTCGTGTGTCTTCTTCCT
GATAGGAAGAACTCTGACATATATGGTCCTTGGAAAAGAAAGAATCTTGCAGAATATGGA
ATTTTCAATCAATGCCTGGCACCCACTAGAGTTAATGAGCAGTATATACTTAATGTTCTC
CTGAAGATAAATGCTAAGCTTGGTGGTTTGAATTCTTTGTTGGCCATGGAGCAATCACGA
AACATCCCATTCGTCTCGAAGGTTCCTACAATAATATTTGGAATGGATGTATCACATGGT
TCGCCTGGTCAGTCTGACATGCCCTCCATTGCTGCGGTTGTCAGTTCTAGAAACTGGCCT
CTACTTTCTCGTTATAGAGCTTCTGTGCGTAGTCAGTCACCAAAAGTTGAGATGGTAGAT
TCTCTTTTTACACTAACACCGGATAAGAAAGATGATTCTGGGATTGTCAGGGAATTGTTG
TTGGACTACTATAGGAGTTCTGGCCAAACAAAACCAGCTCAGATAATCATATTCAGGGAT
GGAGTTAGCGAGTCACAGTTCAATCAAGTCCTCAACATCGAGCTGGATCAAATCATTGAG
GCATGCAAGTTCCTTGATGAAAGCTGGTCACCCAAGTTCACTGTTATTGTTGCACAGAAA
AATCATCACACTAAATTTTTCCAAGATGGATCTCCAGACAATGTTCCTCCTGGAACCGTT
ATTGATAATGCTGTTTGTCACCCACAAAGCTATGATTTCTACATGTGTGCACATGCAGGG
ATGATAGGAACAACAAGGCCAACACATTATCACGTTCTTTTAGATGAGATTGGCTTTTCA
GCTGATGATCTACAGGAGTTGATTCACTCTTTGTCTTATGTGTACCAAAGAAGCACAACA

GCAATATCTGTAGTTGCTCCTGTCCGTTATGCTCACTTGGCAGCAACTCAGATTTCACAA
TTCTTGAAG---

>AGO1903_Aquilegia
TTTCTTCTTTGTCTTATTCCAGAGAGGAATGGAACTATATATGGTCCATGGAAGAGGAAG
TGTCTTGTGGAGTTTGGCATCATCAATCAATGCCTTGCACCTATGAGAGTTAATGATCAA
CTTCTTGCAAATGTTCTCCTAAAGATTAATGCAAAGCTTGGTGGTTTGAATTCTTTGTTA
GCGGTGGAACACTCTCGTAACATTCCTCTGATTTCCAATGTTCCCACTATGATACTTGGT
ATGGATGTATCACATGGTTCCCCAGGCCAGTCAGATATACCATCTGTTGCTGCGGTTGTC
AGCTCAAGGCAGTGGCCCTCGATTTCTCGCTATCGAGCATCTGTGAGAACTCAGTCACCG
AAGCTTGAAATGGTAGATTCTTTATTCAAGCCAGTATCAGACACTGTTGATGAAGGCCTA
GTTCGGGAGCTATTGATGGATTTCTATACTAGCTCAAATAAGAGGAAGCCTGATCAGATA
GTCATTTTCAGGGATGGTGTAAGTGAGTCCCAGTTCAACCAGGTTTTGAACATTGAATTG
GATCAAATCATTGAGGCTTGTAAGTTCCTTGATGAGAAATGGCGTCCAAAGTTCACTCTG
ATTGTTGCACAAAAAAACCACCACACTAAGTTCTTCCAGAAAGGGTCCCCTGACAATGTC
CCACCTGGAACTGTGATTGATAGCCAGATTTGTCATCCTAGACACAATGATTTCTATATG
TGTGCTCATGCTGGAATGATTGGGACAACTAGGCCTACACATTATCATGTCCTTTTCGAT
GAAATTGGCTTTACAGCTGATGATCTTCAGGAGCTTGTCCATTCATTATCTTATGTATAC
CAACGAAGCTCCACGGCCATTTCTACTGTTGCCCCTGTTTTCTATGCTCACCTTGCAGCT
GCTCAGATAGGAAAGTTTATGAAG------------------------------------

>Mguttatus_mgf019782m
GCTGTTGGAAGGTCCTTCTTTCATTGGGACTTCGGATCAGGGAAACTCGGTGATGGCCTG
GATTACTGGAAAGGATTTTACCAGAGTCTTCGCCCAACTCAGATGGGGCTGTCACTGAAT
ATTGATATGTCCGCTAGAGCTTTTATTGAACCTTATGTGTCTGAGTTTGTTTTCAAGTAC
CTCAACCTCAGGGATCGGAATAGGCCTTTATCTGACCAAGATCGGATTAAGGTCAAAAGA
GCTCTTAAAGGTGTCAAGGTTGAGAGCATATATCTGAAGTACACCAAACGTTTCAATATC
ACAGGCGTGTCGACTGAACCCACTGAAAGCCTGATGTTTACCATCGATGCATCCGGTGCA
CAAATATCTGTTTCTGAGTTCTTCCGTCAGCAGCACAGTATCGTGCTTATGTATCCACGT
CTGCCTGCTATTCAGTCCGGCATTGGGAGACCAATTTATATTCCAATGGAGTTATGCAAA
ATTGTTGAGGGTCAAAGATACTCTAGGAAATTGAACGGGAGACAAGTTACTGCCCTGCTC
CAAGCCACATGCGAACGGTTGCTTCTTATTATATTGCCCGATGGCAACGTGACTGATTCA
TACGGTTTAATCAAGAGAGTGTGCGAAACCGAGTTGGGCTTTGTGTCACAATGCTGTCAG
CCCCAACATGTTTTAAAATGCAATAATCATCAGTACTTGGAGAATGTTTCTCTCAAGATC

AACGTTAAGGTTGGAGGGCGAAATACGGTGCTGGAGAAGGCACTCTGTAGAACAATACCT
TTCGTTAGTGACATGCCTACCATAATATTCGGTGCTGATGTCACTCATCCTCAACCAGGG
GACGAATCCAGTCCTTCTATAGCTGCGGTGGTGGCCTCAATGGATTGGCCAGAAGTCTCT
AAGTACAGAGGCTTGGTTTCTGCACAGGGCCATAGGGACGAGATTATCCAGGATCTCTAC
attAATAAGGACTCAGGAATGATTCGCGAACACTTGGTTGCATTCTACCAGAACACTAAG
TTGAAGCCTAGCAGGCTCATCTTTTACAGGGATGGTGTGAGCGAAGGGCAGTTTAATCAA
GTTCTTCTGTATGAAGTTGATGCAATTAGGAAGGCATGTAATGAACTTCAGACAGACTAT
CAGCCAAGAATAACTTTTGTTGTCGTTCAAAAGAAGCATCACACTCGTCTCTTTCCTGCT
GACCATACAAGTCCTAATACTACAGACAAGAGTGATAACATTTTGCCAGGCACTGTGGTG
GATACCAAGATTTGCCACCCCAACCAATTGAGTTTCTATCTTTGCAGCCATGCTGGGATC
CAGGGAACGAGCCATCCAGCACATTACCATGTGTTGTTCGATGAAAATAAATTCAGTGCA
GATAGTATGCAAATGCTTACCTACAGCTTGTGTTACACATATGCTAGGTGCACTCGATCA
GTATCCATAGTACCACCAGCTTACTATGCACATCTTGCTGCATTTCGAGCACGGTATTAC
ATCGAA--

>AT5G21030.1
CTCCTAGTTCGGCAATCTTTCTTCCACAATGACGCAAAGTACTTTGCGAATATCGGTGAA
GGTGTAGATTGTTGCAAAGGATTTCATTCAAGCTTCAGAACTACTCAGGGAGGCTTGTCC
CTCAATATTGATGTTTCGACTGCTATGATAGTAAAACCTGGTCCTGTTGTTGATTTTCTT
ATTGCAAATCAAGGTGTGAACGATCCATTCTCTATTAACTGGAAAAAGGCTAAAAATACT
CTGAAAAATCTTAGAGTTAAAGTCCTCCCTTCAAATCAAGAATACAAGATAACCGGACTA
AGTGGACTACACTGCAAAGATCAAACGTTTACTTGGAAGAAAAGGAACCAAAACAGGGAA
TTTGAGGAGGTTGAGATTACAGTGTCCGATTACTTCACTAGGATCCGTGAAATCGAACTG
CGTTACTCGGGTGGCTTACCTTGTATCAATGTTGGTAAGCCAAATCGTCCTACCTACTTT
CCCATTGAGCTCTGTGAGCTTGTATCTCTACAACGCTATACTAAAGCGCTAACCAAATTC
CAGAGGAGTAACCTTATCAAAGAATCAAGGCAGAATTTCCTTCTATGCATACTCGAAAAG
AAAAACTCTGATGTTTATGAAAAAtcttgttcaatgtggaattgtGAATGTATTGTTCCT
CCTCAAAACTTAAATGATCAGTATCTCACAAATCTTCTACTAAAGATAAATGCCAAGCTT
GGTGGATTGAATTCAGTTTTGGATATGGAGCTGTCAGGAACAATGCCTCTGGTAATGAGA
GTTCCTACCATCATTATTGGAATGGATGTATCTCATGGTTCTCCTGGACAGTCTGATcat
ATACCATCCATTGCCGCGGTTGTGAGCTCCAGAGAGTGGCCACTGATCTCAAAATACAGG
GCTTGTGTGCGTACACAGTCGCCTAAAGTTGAAATGATCGATAGCCTCTTTAAACCCGTC
TCTGACAAAGATGATCAAGGTATCATGAGAGAGCTCTTGCTTGACTTTCACTCAAGTTCT
GGAAAGAAACCGAATCACATTATCATTTTCAGGGATGGTGTCAGTGAATCTCAGTTTAAC
CAAGTTCTTAATATTGAACTGGATCAGATGATGCAAATAATGATGCAAATAAACCACCAC
ACGAAGTTCTTCCAAACCGAAAGCCCTAATAATGTTCTTCCAGGAACAATAATTGACAGC
AACATCTGTCACCAACACAACAACGATTTCTATCTTTGTGCTCATGCTGGAAAGATTGGA
ACTACAAGGCCAACACATTACCATGTGCTCTACGACGAGATTGGATTTGACACAGATCAA
CTCCAAGAACTTGTGCATTCACTATCCTATGTCTACCAGCGGAGCACAACTGCCATCTCT
CTTGTTGCGCCGATATGTTATGCTCATTTGGCGGCTGCACAGATGGCAACTGCAATGAAG

>Alyrata_16055324_locus

CTTTTGGTCAGGCAGGCTTTTTCCATAGCGATGGCCACCCCATGGAAGTTGGAGGAGGT
GTAAAAGGTATTCGAGGTTTCCATTCGAGCTTTCGTCCAACCCATGGAGGACTCTCGCTT
AACATTGATGTGTCAACAACAATAATCTTAGAACCGGGTCCAGTTCTTGAATTCCTGAAA
GCCAATCAGAGTGTTGAGACACCACGACAAATCGACTGGGTTAAGGCTGCGAAAATGCTT
AAACATATGAGGGTAAAGGCAACACACCGTAACATGGAATTTAAAATTATAGGTCTAAGT
CAGAAACCGTGCAATCAGCAACTGTTTTCGATGAAGATTAAAGATGGTGAACGTGAAGGA
CAGACTAGAGATATTACAGTGTATGACTATTTCAAGCAAACTTACACAGAACCTATTTCT
TCTGCGTACTTGCCATGCCTTGATGTTGGCAAGCCAAATCGCCCCAACTATCTTCCACTG
GAGTTTTGTAATCTTGTATCTCTGCAACGTTATACAAAAGCATTGTCCGGAAGGCAAAGA
GCTCTACTTGTTGAAAAATCAAGACAAAAATTTATCCTCTGTGTCCTGCCAGAACGGAAA
ACTTCTGATATCTATGGTCCCTGGAAGAAGATATGCCTCACTGAAGAAGGGATCCACACA
CAATGCATCTGCCCTGTCAAGATCAGTGACCAATATCTCACCAATGTACTTCTGAAGATT
AATTCAAAGCTTGGAGGCATCAATTCTTTGTTGGGAATAGAGTACTCTTACAACATTCCA
TTGATAAACAAAATTCCCACCTTGATTTTGGGTATGGATGTATCTCATGGGTCTCCAGGT
CGGGCAGATGTTCCTTCAGTAGCAGCGGTTGTTGGTTCAAAATGCTGGCCCTTAATCTCA
AGGTATAGGGCAGCTGCAAGAACTCAGTCACCACGTCTGGAGATGATTGATTCGCTCTTC
CAACCTATCGAGAACACTGAGAAAGGAGATAACGGTATTATGAACGAATTATTTGTAGAG
TTCTACCGGACAAGCAGGTCACGGAAGCCTAAGCAGATTATCATTTTCAGGGATGGTGTG
AGTGAATCACAGTTCAACCAAGTCTTGAACATCGAGGTGGACCAAATTATAAAGGCGTAT
CAACGTCTTGGTGAATCTGATGTGCCAAAATTCACTGTCATTGTGGCTCAGAAGAACCAC
CACACCAAGTTGTTTAAAGCTAAGGGTCCTGAAAATGTTCCTGCAGGAACTGTTGTGGAC
ACCAAGATCGTACACCCGACAAACTACGATTTTTACATGTGTGCTCATGCAGGAATAATA
GGAACTTCAAGACCGGCTCATTACCATGTATTACTTGATGAGATTGGTTTCTCCCCCGAT
GACTTGCAGAATCTCATCCATTCTCTCTCCTATGTCAACCAACGTAGCACAACTGCAACT
TCGATTGTGGCTCCAGTGCGATACGCTCATCTTGCAGCAGCTCAATTTGCTAAGTTTGAA

>GRMZM2G359875_T01 cds:_protein_coding
ATGGTGAGGAAGAAGAGAACTGGCCCTGGTGGCTCTGGAGAAACTTCTGGAGAGTCTTCA
GGAGCCTCTGGACAAGGTTCCTCACAGCAGCCTGAGCGAACTCAACAACCTGGGGGAGGA
CGTGGCTGGGTGCCTCAACAGGGTGGCCATGGTGGTGGGCAACACCAGGGTCGTGATCGA
CATTATCAGGGACGTGGAGGACCAGGGCCACATCACCTTGGTAGTGGGGCACCTGAGTAT
CACCCGCGTGAATACCAGGGACGTGGTGGTGAATATCAGGGACATGGTGGTGAGTACCAG
GGACGGGGTGGTGACTACCAGGGACGTGGTGGTGGCCGCTCCAGAGGTGGAATGCCACAG
CCATACTATGGTGGGCATAGGGGAGGTAATGTTGGACGCAATGTTCCTCCAGGTCCGTCC
AGGACAGTTCCCGAGCTGCACCAAGCCCCATATGTCCAGTATCCAGCCCCGGTGGTTTCG
CCCTCCCCATCGGGACCTGGCTCATCCTCACAGCCTATGGCAGAGGTGAGCTCTCCTGTC
GGTAGGTCATTTTACTCTCCCAACTTAGGGAGACGTCAAAAACTTGGTGAGGGATTGGAA
AGTTGGCGTGGTTTTTACCAAAGCATAAGGCCGACACAGATGGGCCTTTCACTGAATATT
GATATGTCCTCTACTGCATTTATCGAGCCTCTCCCTGTGATCGATTTTGTTGCTCAGCTT
CTTAACAGAGATATCTCAGTTAGGCCATTGTCTGATTCTGATCGCGTGAAGATTAAAAAA
GCCCTAAGAGGTGTGAAGGTTGAGGTGACTCACAGGGGAAACATGCGCAGAAAATATCGC
ATTTCTGGCCTCACCTCACAAGCAACAAGAGAGCTATCATTCCCTGTTGATGATCGTGGT
ACTGTGAAGACTGTGGTGCAATACTTCATGGAGACTTATGGTTTTAGTATCCAGCACACC
ACTTTACCATGCTTGCAAGTGGGTAATCAACAAAGACCAAATTATCTGCCTATGGAGGTT
TGCAAGATAGTTGAAGGACAGCGTTACTCAAAGCGACTCAATGAGAAACAAATCACTGCT
CTACTGAAAGTGACCTGCCAGCGCCTGCTGATCGTAATACTGCCTGACAACAATGGTTCT
CTTTATGGGGATCTCAAAAGGATCTGTGAGACTGAACTCGGATTGGTCTCCCAGTGTTGT
CTGACTAAACATGTTTTTAAGATGAGCAAGCAGTACCTTGCAAATGTTGCACTCAAAATA
AATGTTAAGGTTGGGGGAAGGAATACTGTACTTTTAGATGCTTTGTCAAGGAGAATCCCC

CTTGTCAGTGACAGACCGACCATAATATTTGGTGCTGATGTTACCCATCCACATCCTGGA
GAAGATTCCAGTCCTTCCATTGCAGCCGTTGTTGCTTCGCAAGACTGGCCCGAGGTCACG
AAATACGCTGGACTAGTGAGTGCGCAAGCCCATCGCCAGGAGCTGATACAGGATCTTTTC
AAAGTATGGCAGGACCCGCAGAGAAGGACGGTAACTGGCGGCATGATAAAGGAACTTCTC
ATTTCTTTCAAGAGGGCAACTGGACAGAAGCCCCAGAGGATCATATTCTACAGGGATGGT
GTCAGTGAGGGACAGTTCTATCAAGTATTGCTGTACGAACTTGATGCCATTAGAAAGGCC
TGTGCGTCCCTGGAGCCCAACTACCAGCCTCCAGTTACTTTTGTCGTGGTACAGAAGCGC
CATCACACTAGGCTGTTTGCGAACAACCACAGTGATCAGCGCACAGTCGATAGAAGCGGA
AACATACTGCCTGGCACCGTGGTCGATTCGAAGATTTGCCATCCTACTGAGTTTGACTTC
TACCTGTGTAGCCATGCTGGCATTCAGGGAACGAGCCGCCCTGCTCACTACCATGTCCTG
TGGGACGAGAACAAGTTCACAGCTGACGAGCTGCAGACCCTGACGAACAACCTGTGCTAC
ACGTACGCTAGGTGCACCCGCTCCGTGTCCATCGTGCCCCCGGCGTACTACGCTCATCTG
GCAGCCTTCCGAGCTCGCTTCTACATGGAG------------------------------

>Alyrata_16051341_locus
ATGACTGTTGGTAAAAGCTTTTTCACTCGTGAGCCAGATGAAGACTTTCGCTTCGGGGTT
GTAGCTGCGAAAGGGTATCGCCACACTCTAAAGCCCACAGCACAAGGTTTGTCTTTGTGT
TTGGACTACTCGGTTTTGGCATTCCGCAATGCAATGTCGGTCATTGACTACCTGAAGTTG
TACTTTGGCTGGTCCGATATGCGTCAGTTTAGGAATTGTAGGCGTGATGTGGAAAAGGAA
CTGACTGGTTTGAAAGTCACTGTTAACCATCGGAAGAACAAGCAGAAACTCACCATTGTA
GGGTTGAGTGAGTACAACACAAAAGATATCACATTCGATCTTATTGATCATGCGGGAGAC
GAGCCTCCAAGGAAAGTATCCATTGTTAAGTATTTCATGGAAAAGTATGGAAAAGACATC
CGTTACAAGGATATTCCTTGCCTAAGTTTGGGGAAAAAAGGTCGGCAAAATTATGTACCC
ATGGAGTTTTGTAACTTGGTCGAGGGGCAGATTTATCCAAAAGAAAAATTGAAGGGTAAT
TCAGCTTCCCGGTTAAAACACTTGTCCCTTACTCTTGTTCTGTGTGCTATGTCTGGGAAA
GTCGATGGCTACAAGACTCTGAAATGGCTAGCCGAGACCAAACTTGGTCTGGTGACTCAG
TGTTTCTTGACCGGTTCTGCGAATAGGGGAGGTGATCAGTACTTGGCAAATCTCGCCCTC
AAGATAAACGCAAAGGTTGGTGGAACGAACGTGGAGCTGGTGGACAATTATTTCTCTTTC
TTCAATAAAGAAGATGAGGTCATGTTCATTGGTGCTGATGTCAATCATCCCGCTGCTCAC
GACAAGATGAGTCCATCCATTGTTGCTGTTGTAGGCACTCTTAACTGGCCTGAAGCTAAC
CGCTACGCAGCTAGAGTCAAAGCTCAGACTCACCGTAAAGAAGAGATACAAGGGTTTGGT
GAAACTTGCTTAGAGCTTGTCAATGCTCATTCTAATGCCACCAAGAAACGACCTAACAAG
ATTGTGATATTCCGTGATGGTGTCAGTGATGGTCAGTTCGATATGGTTCTCAATGTGGAG
TTACAGAATGTTAAGGACACTTTTAAAAAGATTGAATATAATCCGTTGATAACGGTAATT
GTGGCACAGAAGCGTCATCAAACCCGTTTCTTCCCTGCCACAAGCAATGATAAGGACAAT
GTGCTTTCAGGTACGGTCGTTGATACAAAGATCATTCACCCATTTGAGTATGATTTCTAT
CTCTGTAGTCACCATGGAGCGATTGGGACAAGCAAACCAACTCATTACTATGTTCTGTAC
GACGAAATCGGGTTCAAGTCGGATCAGATTCAGAAGCTCATATTTGACGTGTGCTTCACG
TTTACTCGCTGCACCAAACCTGTCGCTCTGGTTCCTCCAGTTTCTTATGCTGACAAGGCT
GCTTCTAGAGGAAGGTTGTACTACGAG---------------------------------

>GRMZM2G165242_T02 cds:_protein_coding
CTATTAGTAAGGCAATCGTTCTTCCACAATAATCCTTCCAATTTTGTTGACCTGGGTGGT
GGTGTAGTGGGATGTAGAGGGTTCCATTCTAGTTTTCGTGCAACCCAGAGTGGACTTTCA
CTCAATATCGATGTGTCCACCACAATGATAGTGAAACCTGGTCCTGTCATTGACTTTCTG
ATTGCCAATCAGAAAGTTAATGATCCAAGCATGATTGATTGGGCAAAGGCCAAGCGCTCA
CTGAAGAACTTAAGGATAAAAACAAGTCCGGCGAACCAAGAACAGAAGATTGTTGGTCTC
AGCGACAGGCCTTGCCGTGAGCAATTATTCACACTGAAACATAAAAATGGTGAATCTGAA
GAGATCACTGTTTTTGATTACTTTGTAAAGAACCGTGGCATAAAGCTGGAATATTCTGGT
GATCTTCCTTGTATCAATGTGGGAAAACCAAAGCGTCCAACTTATTTTCCAGTTGAGTTA
TGCAGTCTTCTTCCTTTGCAACGGTACACTAAAGCATTGAGCACACTTCAAAGATCATCA
CTCGTTGAGAAATCTAGGCAGAAATTTCTTTTGTGTGTTCTAGCGGAAAGAAAGAATTCC

GATATTTATGGGCCTTGGAAGAAGAAATGCCTTGCTGAATTTGGGATCGTTACACAGTGT
GTGGCACCAACTAGAGTCAATGATCAGTATCTTACAAATGTCTTGTTAAAGATTAACGCA
AAGTTGGGTGGCTTGAATTCGTTGCTCCAAATTGAAACATCCCCAGCAATTCCTCTCGTA
TCCAAGGTCCCAACTATAATCTTGGGTATGGACGTATCACACGGATCTCCTGGACATTCT
GATATACCGTCTGTTGCTGCTGTTGTTAGTTCTCGTGAATGGCCTCTTATCTCAAAATAC
AGAGCATCTGTCCGCACCCAATCACCAAAAATGGAAATGATTGACTCGTTGTTTAAGCCA
CGGGAAACTGATGACGATGGTCTGATTCGGGAGTGTCTGATTGACTTCTACACCAGTTCA
GGGAAGAGAAAGCCCGACCAAGTCATCATCTTCAGGGACGGTGTTAGTGAAAGTCAGTTT
AATCAGGTGCTGAACATTGAGTTGCAACAAATCATTGAGGCTTGCAAATTTCTTGATGAG
AAATGGAATCCCAAGTTCACATTGATCATTGCCCAGAAGAATCACCACACTAAATTTTTC
ATTCCTGGAAAGCCAGATAATGTTCCAGCTGGCACTGTTGTTGACAACAAAGTCTGTCAT
CCAAGGAACTTCGATTTCTACATGTGTTCACATGCTGGAATGATTGGAACTACCAGGCCA
ACTCACTATCACATCCTGCATGACGAGATAGGCTTCAATCCTGATGACCTGCAGGAGCTG
GTGCACTCGCTCTCTTATGTGTACCAAAGGAGCACAACAGCCATATCAGTTGGTAAGTTT
ATCTTGgcaccgtttttcgttTATGTC--------------------------------

>Mguttatus_mgf010559m
CTTTTGGTTGGTCAATCATTCTTTCATGATGACTCGCGGACGTTCACTGATGTTGGAGGG
GGGGTGACAGGTGTGAGAGGTTTCCACTCCAGCTTTCGTCCAACTCTGAGTGGTTTGTCT
CTGAATATGGATGCATCGACAACCTTGATCTTGACGCCTGGACCAGTCGTGGACTTCCTG
CTTGTTAATCAGAATGTGAAGGAAACTCGGTATATTGACTGGGCAAAGGCCAAAAAGATG
CTCAAGAATATGAGGGTTAAGGCGAGGCACAATAATATGGAATTCAAAATTGCAGGTTTG
AGTGAGAAACCTTGCAATCAGCAGTTATTTTCGATAAAAGTTAATAGTGGTggtagtccc
GGAGGTGATGGAGAAACCGTGGAGATTACTGTTTACGACTATTTTGTTAAACACCGTAAC
ATAGAACTTATATCTTCATCATATATGCCATGCATTGATGTTGGGAAACCGAAAAGACCG
ATCTATCTGCCTATAGAGCTCTGTTCTCTAGTCTCTCTTCAGAGATACACAAAAGCACTG
TCGGTAACACAAAGAGCATCCTTGGTTGAAAAATCAAGGCAGAAGTTTTTGCTTTGTGTT
TTGCCCGAACGAAAAAATTGTGATCTTTATGGACCTTGGAAGAGAAAATGTCTATGTAAC
TTGGGTATCGTCACCCAATGCGTCTCTCCTATCAAAATCAACGACCAGTACCTGACAAAT
GTACTTCTCAAAATGAATTCTAAGTTAGGGGGGATCAACTCATTGTTGGCAATAGAGAAT
TCCCGTCGAATTCCACTCATTACGGATAAGCCGACCATGATCTTGGGAATGGATGTATCG
CATGGTTCTCCTGGTCGATCTGATATCCCTTCAATTGCTGCGGTTGTTGGATCTCGGAGT
TGGCCATTGATATCAAGGTATAGAGCAGCTGTACGAACCCAATCTTCAAGGGTGGAGATG
ATTGAAAGTTTGTTCAAGCCTCTAGCAAATGGGGAGGATGACGGCATTATGAGGGAACTG
CTTAAGGATTTCTACGAAACCAGTAATGGACGCAAACCAACTCAGATTATTATTTTCAGG
GATGGTGTGAGTGAATCCCAGTTTACACAAGTCATCGACATTGAACTCAATCAAATTATC
AAGGCTTATCAGCATCTAGGCGAGACCGAGATTCCGAAATTCACAGTGATAGTGGCCCAG
AAGAATCACCATACGAGACTTTTCCAAGCTACAGCTGCTGAGAATGTTCCACCCGGTACT
GTTGTGGACACAAACATTGTTCATCCTACAAATTACGATTTCTATATGTGCTCTCAGGCA
GGGAAAATAGGAACTTCTCGACCTGCACATTATCATGTATTGCTCGACGAGATTGGTTTC
TCCCCAGACGATATGCAGAATCTCATCCATTCCCTATCATATGTATATCAGAGGAGCACT
ACTGCCATCTCCATTGTTGCACCGGTATGTTATGCTCATCTTGCAGCCCAACAAATGAGT
CAGTTTATAAAA--

>GRMZM2G361518_T01 cds:_protein_coding
GCACCGTTTGGGCGATCGTTTTTCTCTCCTGACCTGGGGAGGAGGCGTTCCCTTGGTGAG
GGAATAGAAAGCTGGCGTGGGTTTTACCAGAGCATTCGCCCTACTCAAATGGGCTTGTCA
TTGAATATTGATATGTCTGCAACTGCTTTCTTTGAGCCATTACCTGTCATAGATTTTGTT
GCACAGCTTTTAAACACCGACGACATCTACTCGAGGCCCCTCTTAGACGCTGAACGTGTC
AAGATAAAGAAGGCCTTAAGAGGCGTGAAGGTGGAAGTTACGCACCGTGGTAACATGCGA
CGCAAGTATCGAATAGCTGGTTTAACATCTCAGGAAACTCGGGAGCTAACTTTTCCTGTT
GATCAAGGTGGCACAGTGAAGTCAGTTGTACAGTATTTTCAAGAGACCTATGGCTTTGCC
ATCCAGCACACCTACCTGCCCTGTCTGCAGGTTGGCAACCAGCAGCACCCAAATTACCTT
CCAATGGAGGTCTGCAAGATAGTGGAGGGACAGAGGTACTCTAAGAGATTAAACCAGAGT
CAGATAAGAGCTCTTTTAGAGGAGACATGCCAACGCCTGCTCATTGGAATACTCCCTGAT
AACAACGGTTCTCTTTATGGTGATCTGAAGCGTATCTGTGAAATCGACCTTGGATTAGTT
TCCCAGTGCTGCTGCGCAAAGCAAGTCTTTAAGATGAACAAGCAGATACTGGCAAACCTT
GCGCTGAAGATAAATGTCAAGGTCGGAGGAAGGAACACGGTGCTGGCTGATGCGGTGTCA
AGACGCATTCCCTTGGTGACTGACAGGCCTACCATCATATTCGGTGCCGATGTGACCCAT
CCTCATCCTGGAGAAGATAGCAGCCCTTCCATTGCTGCTGTTGTGGCTTCCCAAGACTGG
CCTGAGGTGACAAAGTATGCTGGTCTAGTTTCTGCTCAGTCTCATAGGCAAGAGCTGATA
GAGGATCTCTACAACGTCACACACGATCCTCAGAAAGGAACCGTTTGTGGTGGCATGGTC
AGGGAGCTTCTTATATCCTTCAAAAAATCAACTGGTCAAAAGCCTCAGAGGATACTATTC
TACAGGGATGGTGTGAGTGAAGGGCAGTTCTACCAAGTTCTACTGCATGAACTGGATGCT
ATCCGAAAGGCGTGTGCATCGCTGGAAGCAAACTACCAACCGCAGGTGACTTTCATCGTC
GTCCAGAAACGCCACCATACCAGGCTGTTCGCGCACAACCACAACGACCAGAATTCGGTC
GACAGGAGCGGCAACATACTTCCTGGAACTGTCGTGGACTCGAAGATCTGCCACCCTACA
GAGTTCGACTTCTTCCTGTGCAGCCATGCTGGCATCAAGGGCACCAGCCGTCCTGCTCAC
TACCATGTCTTGTGGGACGAGAACAACTTCACAGCCGACGCACTGCAGACCCTCACCAAC
AACCTTTGCTACACCTACGCGAGGTGCACGCGCTCTGTGTCCATTGTCCCGCCGGCGTAC
TACGCTCACCTGGCCGCATTCCGCGCCCGGTTCTACATGGAG------------------

>Bdistachyon_Bradi2g14150
CTTTTAGTCCGCCAGTCATTTTTCCACAACAATCCTCGTAATTTTGTTGACCTGGGCGGT
GGTGTGTTGGGGTGCCGAGGATTTCACTCAAGTTTCCGTGGTACACAGAGTGGACTCTCC
CTGAACATTGATGTTTCCACTACAATGATTGTTCAACCTGGTCCTGTTATTGATTTCCTT
AAAGCTAACCAGAAAGTTGATCAGCCTGCCAGAATTGACTGGTCAAAAGCCAAGCGTGCT
CTTAAGAACTTGAGGATAAAAACAATTCCCGCAAATACAGAATTCAAAATTGTTGGTTTG
AGTGACAGAAATTGCAATGAACAGACGTTTGAATGGAGGCAGAGGAATGGTAGTGGAGGT
ATCGATACTGTTGAAATAACAGTCTATGAGTACTTCGTTAAGATTAGAGGCATAGAACTG
CAGTATGGTAGTCTTCCCTGTATCAATGTAGGGAGGCCAAAGCGTCCAACGTATTTTCCT
GCGGAGCTTTGCATGCTTCTTCCACTGGAAAGATACACCAAAGCTCTGTCTACTCTGCAA
AGGTCCTCGCTTGTTGAGAAATCCAGACAGAAGgattcaaatATATATTCTTTCCTTCAA
CCAGGCCCTTGGAAGAAGAAGTGTCTTGCTGATCTTGGTATTGTTACACAATGTCTAGCT
CCTCCAgcaAGAGTAAATGATCAGTACATAGATAATGTGCTGTTGAAGATAAATGCTAAG
CTTGGTGGGCTGAACTCATTGCTGAGAATTGAAGTAGAACGCACAATACCTCTTGTGTCA
AAGGTGCCTACTATCATCTTGGGCATCGACGTGTCACATGGTCCACCTGGGCAATCTGAT
AGACCTTCCATTGCCGCGGTGGTTAGCTCTCGAGAGTGGCCTTACATCTCTAAATATAGA
GCAACAGTGAACACTCAGTCACCCAAACTAGAGATGGTGTCCTCCTTGTTTAAACCACAG
GGGCCTGAAGATGATGATGGCCTCATTCGGGTATCACTTATTGACTTCTACAACACTAGT
GGGAAGCGCAAACCAGATCACGTTATTATTTTCAGGGATGGAGTTAGTGAAAGCCAATTT
ACTCAGGTCATAAACATCGAGCTTGAAAAGATCATTGAGGCATGCAAGTTCCTCGATGAG
AAGTGGTCGCCTAAGTTCACAGTCATTGTTGCTCAGAAGAACCATCATACCAAATTTTTC

CAGACTGGATCCCCGGATAATGTTCCTCCCGGCACTGTGGTGGACAAACAAGTGTGTCAT
CCCAAGAATTTCGACTTCTACATGTGTGCACATGCTGGCATGATCGGAACATCAAGGCCA
ACACATTATCATGTTCTGCACGATGAGATCGGCTTCACAGCTGACGAACTTGAGGAATTT
GTGCATTCACTCTCCTATGTGTACCAGAGGAGCACAACGGCGGTATCAGTCGTTGCTCCA
ATTTGCTATGCGCATCTGGCGGCTGCCCAGGTCGGCACATTCCTGAAG------------

>LOC_Os03g33650
ATCCTTGTGGGTCGGTCACTGTATGCACGCTCCATGGGGGAAGCAAGGGACATTGGTGGT
GGTGCTGTTGGATTAAGAGGTTTCTTTCAGAGATTGAGGCCAACCAAGCAAGGCCTTGCC
CTTAATGTTGATCTCTCACTCTCAGCTTTCCACGAGAGCCAGAAGCGCTGTGACTTCTTG
AAGGACCTTCCACAGAAGAAAACAAGGGCTTTGGCAGAAGAGGAGCACAGGGAGGTGGAG
AAAGCATTGAAAAATATCCGGGTATTTGTGTGCCATCGTGAGACTAATCAAAGGTACCAT
GTGCATAGCTTGACTAAGGAGACAACAGAGAACCTCAAGTTTCGCGACCGAAGTGGGAAG
GATCTTATGGTGGTGGATTACTTCAAGGAGCACTATAACCATGATATACAATTCAGGAAC
CTTCCATGCTTGCAGATTGGCAGGAGCAAGCCATGTTATGTGCCAATGGAGCTTTGTGTA
GTTTGTGAGGGCCAGAAGTTTCTTGGCAAGCTGTCTGATGAACAAACTTCTAAGATTCTG
AAAATGGGTTGTGAAAGATTGCTAATCTGTGTCATGGAGAGGAGGCACCAAGGCTATGCT
GATCTGAAGCGAATTGCAGAAACATCCATTGGTGTTGTGACACAATGTTGCCTTTACTCC
AACTTGAGCAAGCTGACCTCTCAATTCTTGACGAATTTGGCTTTGAAGATCAATGCGAAA
CTCGGTGGCTGCAATATTGCCCTATACAGCAGCTTTCCATGCCAAATTCCTAGAATATTT
TTGTCGGAGGAGCCGGTGATGTTCATGGGTGCTGATGTCACACACCCGCATCCCCTTGAT
GATTCAAGTCCATCAGTGGTTGCTGTAGTTGCAAGCATGAATTGGCCGTCAGCAAATAAG
TACATCTCCAGGATGAGATCACAGACACACCGGAAAGAAATCATTGAGCAACTGGATGTT
ATGGCTGGTGAACTGCTTGAAGAGTTTCTAAAAGAAGTGGGGAAGCTCCCAAGCAGAATC
ATATTCTTCAGAGATGGTGTGAGCGAGACACAGTTCTACAAGGTGCTGAAGGAGGAGATG
CATGCAGTGCGCACAACTTGTTCGAGGTATCCGGGTTACAAACCCTTGATCACATTCATC
GTAGTTCAGAAGAGGCATCACACTAGACTCTTCCACAGGGAGAGGAATGGCAGCTCGTCA
CACTACTCTGATCAGAACATACCACCAGGAACAGTTGTGGACACTGTGATTACACACCCA
AGGGAATTTGATTTCTATCTGTGCAGCCACTGGGGCACCAAGGGGACGAGCCGGCCAACT
CATTATCATGTTCTGTGGGATGAGAATAACTTCCGTTCCGACGAAGTGCAGCAGTTGATA
CACAATCTTTGCTACACATTTGCTCGGTGCACCAGGCCAGTTTCTCTTGTCCCACCGGCT
TACTACGCACATCTCGCGGCATATAGAGGCAGGCTGTACCTTGAG---------------

>LOC_Os01g16870
CTTTTGGTTCGCCAATCCTTTTTCCATAACAATCCTTCAAATTTTGTTGACTTGGGTGGT
GGTGTGATGGGCTGTAGGGGATTCCATTCAAGCTTCCGAGCTACACAGAGTGGACTTTCA
CTCAATATCGATGTGTCCACAACGATGATTGTGAAACCTGGTCCTGTGGTAGATTTTCTA
CTTGCCAACCAGAAGGTTGATCACCCAAACAAAATTGACTGGGCAAAGGCCAAGCGTGCT
CTGAAGAATTTAAGGATAAAAACAAGTCCAGCAAATACTGAATACAAGATTGTTGGTTTG
AGTGAGAGAAACTGCTATGAACAAATGTTCACTTTGAAGCAAAGAAATGGTGATGGTGAA
CCTGAAGGTGTGGAAGTATCTGTTTACGAGTACTTTGTGAAGAATCGGGGCATAGAGTTG
AGATACTCTGGTGATTTTCCCTGTATCAATGTGGGGAAACCAAAACGGCCAACTTATTTT

CCAATTGAGCTCTGCTCTCTTGTGCCTTTGCAAAGGTATACCAAGGCTTTGAGTACACTA
CAGAGGTCTTCGCTTGTTGAGAAGTCCAGGCAGAAATTTCTGCTCTGTGTTCTTGCTGAG
AGGAAGAATTCAGATATTTATGGGCCCTGGAAGCGGAAATGTCTTGCTGAATTTGGGATT
ATTACACAATGTGTGGCCCCTACTAGGGTCAATGATCAGTATATTACAAATGTACTGTTA
AAGATAAATGCAAAGCTTGGTGGCTTGAACTCCCTGCTTCAAATTGAAACATCCCCTTCC
ATTCCTCTTGTATCCAAGGTCCCGACAATAATATTGGGAATGGATGTTTCCCATGGATCA
CCTGGACAGTCTGATATACCTTCAATTGCTGCGGTCGTTAGTTCTCGTGAATGGCCTCTT
GTATCAAAATACAGGGCTTCAGTCCGTTCTCAGTCACCTAAGTTAGAAATGATTGATGGA
CTATTTAAGCCACAAGGAGCTCAGGAAGATGATGGCCTCATTCGGGAGTTATTAGTTGAC
TTCTATACCAGTACTGGTAAACGTAAGCCTGATCAAGTCATTATCTTCAGGGATGGGGTC
AGTGAGAGCCAGTTTACCCAGGTGCTGAACATTGAGCTGGATCAAATAATTGAGGCATGC
AAGTTTCTTGACGAAAACTGGTCTCCAAAGTTTACACTGATTGTTGCACAGAAGAATCAT
CATACCAAATTTTTTGTGCCTGGATCTCAAAATAACGTTCCTCCTGGTACTGTTGTGGAC
AACGCAGTCTGTCATCCAAGGAACAATGACTTCTACATGTGTGCGCACGCTGGAATGATT
GGAACTACAAGGCCTACACATTATCATATCCTTCATGATGAGATAGGCTTCTCTGCTGAC
GATCTTCAGGAGTTGGTCCACTCCCTTTCCTATGTCTACCAGAGGAGCACTACAGCCATA
TCAGTCGTTGCACCCATTTGCTACGCGCATCTCGCTGCTGCTCAGGTCAGCCAGTTCATC
AAG---

>LOC_Os04g52550
ATTGTCCTTGGTCAGGGATTTTACCCGCAGAGCGGCAGTgaggccatcagtGATAGCAAT
ATTGTAGCTCTCAAAGGAACCCAGCAGAGCCTTAAATGCACTCAGAAAGGGCTGATCCTG
TGTGTGGACTATTCGGTTTTGCCATGTTGGAAAGCTGGATCTGTGTTGGACCTTGTTAAG
ACTATGaagTTCATGGAATACCCGCTCTTGGAAGACCAATTGAAAAAATTGAACAATGCG
CTCAAAGGCCTGTGTGTTACTGTAAGTCACAGGAAGACTGAGGAGAAGTACACTGTTAAA
GGCTTGACAGATAAACCTGCCGACCAGATAACTTTTAAAGACTCTAAATCAGGACAGACG
ACGAAGCTTATCGAGTACTATAAGGAGACGTATAAGAAAGAGATTGAGCATCCGATGCTT
CCATGCTTGGATTTGAGCAAGAGCAAGTCCAAACAAAACTATGTGCCGATTGAATTTTGT
AATATTCCTGAAGGGGAGAGGTATCCAGTGGCGAGGTTAGATGACAAGAAGTCTGATAAT
AAGGGCGAACAAGAGAAGCTCCTCTTCTGCCCGATGCTCAACCGGTGCCATGGGTACAAG
ACCCTGAAGTTGATGTGCGAGACGGAGCTGGGGATCCAGACCCAGTGCTTCTTGAGCACC
GCCGCAAAACTCgacgaaAAACGACAGGACCAGTACATTACCAACCTTGCTCTGAAGATC
AACGGCAAGATCGGGGGTAGCAACATGCAGCTCGACCCGGACTCGATCCCAGTGGTGTCC
GCCAAGGATTTCATGTTCATCGGTGCGGACGTGAACCACCCTCCGCCTGGGAATGTCAGT
AAGgacatcCCGTCCATAGCAGCCGTGGTGGCCTCCGTTGATAAAGGCGCCAGCAAGTAC
GTGACAAGGATCCGCGCCCAGTATCACCGGTGCGAGATGATCCAGAACCTCGGTGATATC
TGCAAGGAGCTCATCGGCGCGTATGAGAAGGTAAACAAGAAGAAGCCTGATAGCATCATC
TACTTCCGCGACGGCGTCAGTGACGGTCAGTTCGACATGGTGCTGAACGAGGAGCTGGCG
GACATGGAGAATAAGATCATGGTGGGTGACTACCCGAAGATCACCGTGATCGTTGCCAAG
AAGAGGCACCACACGCGGCTGTTCCCCAAGGACAGGAACCAGCGGCAGACCAAGAACGGC
AACGTGCTCCCCGGCACGGTGGTGGACACCGACGTGGTCGACCCGACGGCGTACGACTTC
TACCTGTGCAGCCACAAGGGGGAGGTCGGGACGAGCCGGCCGACGCACTACTACAGCCTT
CTGGACGAGCACGGCTTCGCCTCCGACGACCTGCAGAAGCTGGTGTACAACCTCTGCTTC
GTCTTCGCCCGCTGCACCAAGCCGGTGTCGCTGGCCACTCCCGTCTACTACGCCGACCTC
GCCGCCTACCGCGGCAGGCTCTACTACGAG------------------------------

>AT2G32940.1
CTTTTGGTCAGACAGGCATTTTTCCATAGCGATGGGCACCCCATGAAAGTTGGAGGAGGT
GTTATAGGTATTCGAGGTTTACATTCGAGCTTTCGTCCAACTCATGGAGGACTCTCGCTT
AACATTGATGTGTCAACAACAATGATCTTAGAACCTGGTCCAGTTATTGAATTCCTGAAA
GCCAATCAGAGTGTTGAGACACCACGGCAAATCGACTGGATTAAGGCTGCGAAAATGCTT
AAACATATGAGGGTTAAGGCAACACACCGTAACATGGAATTTAAAATTATAGGTCTAAGT
TCGAAACCGTGCAATCAGCAACTGTTTTCAATGAAGATTAAAGATGGTGAACGTGAGgta
CCTATTAGAGAGATTACTGTGTATGACTATTTCAAGCAAACCTACACAGAGCCTATTTCT
TCTGCGTACTTTCCATGCCTTGATGTTGGCAAGCCAGATCGGCCCAACTATCTTCCACTG
GAGTTTTGTAATCTTGTATCTCTGCAACGTTATACAAAACCATTGTCAGGGAGGCAAAGA
GTTTTACTTGTTGAAAGTTCAAGACAAAAATTTATCCTCTGTATTCTGCCAGAACGGAAA
ACTTCTGATATCTATGGTCCCTGGAAGAAGATATGCCTCACTGAAGAAGGGATCCACACA
CAATGCATCTGCCCAATCAAGATCAGTGACCAATATCTCACCAATGTACTTCTGAAGATA
AATTCGAAGCTTGGAGGCATCAATTCTCTGTTGGGAATAGAGTACTCTTACAACATTCCA
TTGATAAACAAAATTCCCACCTTGATTTTGGGTATGGATGTATCTCATGGGCCTCCAGGT
CGGGCAGATGTTCCTTCAGTAGCAGCGGTTGTTGGTTCAAAATGCTGGCCCTTAATCTCA
AGGTATAGGGCAGCTGTAAGAACTCAGTCACCACGTCTGGAGATGATTGATTCGCTCTTC
CAACCTATCGAGAACACTGAGAAAGGAGATAACGGTATCATGAACGAATTATTTGTAGAG
TTCTATCGGACAAGCAGGGCACGGAAACCTAAGCAGATTATCATTTTCAGGGATGGTGTG
AGTGAATCACAGTTCGAGCAAGTCTTGAAGATCGAAGTGGACCAAATTATAAAGGCGTAT
CAACGTCTTGGTGAATCTGATGTGCCAAAATTCACTGTCATTGTGGCTCAGAAGAACCAC
CACACCAAGTTGTTTCAAGCTAAGGGTCCTGAAAATGTTCCTGCAGGAACTGTCGTGGAC
ACCAAGATCGTACACCCGACAAACTACGATTTTTACATGTGTGCTCATGCAGGAAAAATA
GGAACTTCAAGACCGGCTCATTACCATGTATTACTTGATGAGATTGGTTTCTCCCCCGAT
GACTTGCAGAATCTCATCCATTCTCTCTCCTATGTCAACCAACGTAGCACAACTGCAACT
TCGATCGTGGCTCCAGTACGATACGCTCATCTTGCAGCAgctcaagttGCGCAATTCACA
AAG---

>LOC_Os06g51310
GCACCCTTTGGTCGATCCTTCTTCTCTCCTGACCTGGGGAGGAGACGATCCCTCGGTGAG
GGACTAGAAACCTGGCGTGGGTTTTATCAGAGCATTCGTCCTACTCAAATGGGCTTGTCA
CTGAATATTGATATGTCGGCAACTGCTTTCTTTGAGCCGTTACCAGTCATAGATTTTGTC
ATACAGCTTTTAAATACTGACATCCGCTCGAGGCCCTTATCAGATGCTGAGCGTGTCAAG
ATCAAGAAGGCCTTAAGAGGAGTGAAGGTAGGAGTTACTCACCGTGGCAACATGCGCCGG
AAGTATCGGATATCTGGTTTGACATCTCAGGCAACTCGGGAACTGACTTTTCCTGTTGAT
CAAGGAGGCACAGTGAAATCTGTTGTACAATATTTTCAAGAGACATATGGATTTGCGATC
CAGCATACCTATCTTCCTTGTCTGCAAGTTGGCAATCAGCAGCGTCCAAATTACCTACCA
ATGGAGGTCTGCAAAATAGTGGAAGGACAGAGGTACTCCAAGAGACTGAACCAGAATCAG
ATAAGGGCTCTTTTAGAGGAGACATGTCAGCGCTTACTTATCGGACTGCTTCCTGATAAC
AATGGTTCTCTTTATGGTGATTTGAAGCGTATATGCGAAATTGACCTTGGATTGGTTTCC
CAGTGCTGTTGCACAAAGCAAGTGTTTAAAATGAACAAACAAATCCTAGCAAATCTTGCT
CTGAAGATAAATGTGAAGGTTGGGGGAAGGAACACTGTACTGGTTGATGCAGTGTCGAGA
CGCATTCCGTTGGTAACTGACAGGCCTACTATTATATTCGGTGCTGATGTCACCCATCCT
CACCCTGGCGAAGATAGCAGCCCATCCATTGCTGCTGTTGTGGCCTCCCAAGATTGGCCT
GAAGTGACAAAGTATGCTGGTTTAGTTTCTGCTCAATCTCACAGACAAGAGTTAATAGAT
GATCTGTATAACATCACGCATGATCCTCATAGAGGGCCCATCTGCGGTGGAATGGTCAGG
GAACTTCTTATATCCTTCAAAAGATCAACTGGTCAAAAGCCTCAACGGATAATATTCTAT

AGGGATGGTGTTAGTGAAGGGCAATTTTACCAGGTTCTATTGCATGAGCTTGATGCAATC
CGAAAGGCTTGTGCATCACTCGAAGCAAATTACCAACCGCAGGTGACTTTCATTGTGGTT
CAGAAGCGCCACCACACGAGATTATTTGCACACAACCACAATGATCAGAATTCAGTCGAC
AGGAGTGGGAACATATTGCCTGGTACTGTTGTTGACTCCAAGATTTGCCATCCTACGGAG
TTTGACTTCTTCTTGTGCAGCCATGCTGGAATCAAGGGCACAAGCCGTCCCGCTCATTAC
CACGTCCTGTGGGATGAAAACAACTTCACAGCTGATGCGTTGCAGACCCTCACCAACAAC
CTCTGTTACACTTATGCGAGGTGCACACGATCCGTATCTATTGTTCCACCAGCATACTAT
GCTCATCTGGCGGCGTTCAGAGCCCGTTTCTACATGGAG---------------------

>Bdistachyon_Bradi1g29580
GCATCATATGGTCGATCCTTCTTCTCGCCTGACCTGGGGAGGAGGCGATCCCTTGGTGAA
GGAATAGAAAGCTGGCGTGGGTTTTATCAGAGCATTCGTCCTACTCAGATGGGATTATCA
CTAAATATCGACATGTCAGCGACATCTTTCTTCGAGCCACTACCTGTCATTGATTTTGTT
GCACAGCTTCTAAACACTGATGTCTACTCAAGGCCCCTGTCAGATGCTGATCGTGTCAAG
ATCAAGAAGGCCTTAAGAGGAGTGAAGGTGGAAGTTACTCATCGTGGCAACATACGGCGG
AAGTACCGTATATCTGGTTTAACATCTCAGGCAACTCGGGAGTTAAGTTTTCCTGTTGAT
CAAGGGGGCATGGTGAAGTCTGTTGTACAATATTTTCAGGAGACATATGGTTTTGCTATC
CAACACACCTACCTTCCCTGTCTGCAAGTTGGTAATCAGCAGCGTCCAAATTATCTCCCC
ATGGAGGTCTGCAAAATAGTGGAGGGACAGAGATATTCCAAGAGACTGAACCAGAGTCAG
ATAAGAGTTCTCTTAGAGGAGACATGTCAGCGCTTGCTTATTGGAATACTTCCTGATAAC
AATGGCTCACTTTATGGTGACTTGAAGCGTGTCTGTGAAATCGACCTTGGGCTAGTTTCC
CAGTGCTGTTTAACGAAACAAGTGTTTAAAATGAACAAACAGATCCTGGCAAATCTTGCG
CTGAAGATAAATGTCAAGGTTGGGGGAAGGAACACTGTACTGGCTGATGCATTGTCAAGG
CGCATTCCTTTGGTTACTGACAGGCCTACGATTATATTTGGTGCCGATGTCACCCATCCT
CATCCTGGTGAAGACAGCAGCCCTTCCATTGCTGCAGTTGTGGCCTCCCAGGATTGGCCT
GAGGTGACAAAATATGCTGGCCTAGTTTCTGCTCAATCTCACAGGCAAGAATTGATAGAG
GATCTATATAAAGTCACACATGATCCACAGAGAGGAACCATCCATGGTGGCATGATCAGG
GAGCTTCTTATATCCTTTAAAAGGTCAACTGGAGAAAAGCCTCAGCGAATTATATTCTAT
AGGGATGGTGTCAGTGAAGGCCAGTTTTACCAAGTTCTACTGCATGAGCTTGATGCGATC
CGAAAGGCATGTGCATCGCTGGAAGCAAATTACCAACCGCAGGTTACTTTCGTCGTGGTT
CAGAAGCGCCACCACACCAGGTTATTTGCACACAACCACAATGACCAAAACTCTGTTGAC
AGGAGCGGCAACATACTTCCTGGTACTGTTGTTGACTCCAAGATCTGCCATCCTACAGAG
TTTGACTTCTTCCTGTGCAGCCATGCTGGCATTAAGGGCACAAGCCGTCCTGCTCATTAC
CATGTACTGTGGGATGAAAACAACTTCACCGCCGATGGGCTGCAGACCCTCACTAACAAC
CTCTGCTACACTTATGCAAGGTGCACGCGTTCTGTATCTATTGTTCCTCCTGCATATTAT
GCTCATCTGGCTGCCTTCCGTGCCCGTTTCTACATGGAG---------------------

>GRMZM2G354867_T01 cds:_protein_coding
GTTATCCTTGGTAGAGGATTTTACTCACCAAGCAGCAGTATAGACATTGGGCAGGGTGTT
GTAGCTATGAAAGGAACCCAGCAGACACTTAAATACACTCAACAAGGGTTGAACCTGTGT
GTTGATTATTCAGTTATGCCATTTTACAAAGCTGGACCGGTGATGGACCTTGTTCACAAA
ATAGTGGGGTACCTTGATTATCGAACAACTCTGAACAAGAGGCAAATGGAAAATCTGGTT
GATGAGCTTAAAGGCCGACGTGTAACTGTGATTCATCGGAGGACTAATCAGAAGTACACA
GTGCAAGGCTTGACACCCTTACCTGCCAGCCAGATGACCTTTGTGGATGCTGAATCCGGA

CAAACAAAGTGTCTTGTGGAGTATTATGCTCAGAAACATGGCATTGTGATTGAGTATCAG
ATGCTGCCATGCTTGGATTTGAGCAAGAGCAAGGACAAACCGAATCATGTCCCAATTGAG
CTCTGCACTCTTCTTGAAGGACAGAGGTTTCCAAAAGCAAACTTGGATAAGAATTCTGGC
AGGATACTAAAAGGAAAGGCTCTACAGCTCCTTTTCTGCCCGATGTCCGAGCAGCATCCA
GGGTACAAGACACTGAAGCTGATTTGCGATACACAGCTTGGGATCATGACCCAGTGTTTC
CTGGGCGACCGCGCAAACAAGCCGAATGGGCAGGACCAGTACATGACCAACCTTGCCCTC
AAGATAAACGGCAAGCTTGGGGGCAGCAACGTCCAGCTGTTCGACTCGCTCCCACGGGTC
GGTGGGGCACCTTTCATGTTCATCGGTGCTGACGTCAACCACCCGTCACCGGGGAACGTG
GAGAGCCCATCGATTGCAGCCGTGGTTGCGTCTATCAACTCCGGTGTCAGCAAGTACGTG
ACAAGAATCCGTGCCCAGCCGCACCGCTGTGAGGTGATCCAGCAGCTCGGCGAGATCTGC
CTGGAGCTCATCGGAGTCTTCGAGAAGCGAAACCGCGTGAAGCCGCAGAAGATCATCTAC
TTCCGCGACGGCGTGAGCGACGGGCAGTTCGACATGGTCCTGAACGAGGAGCTGGCGGAC
CTGGAGAAGGCGATCAAGGTGGGCGGCTACGCGCCGACCGTCACCGTGATCGTGGCCAAG
AAGCGGCACCACACGCGCCTGTTCCCCAAGGACCCCAGCCAGCCGCAGACGAAGAACGGG
AACGTGCCGCCCGGCACGGTGGTGGACACGGGCGTGGTGGACCCGTCCGCGTACGACTTC
TACCTGTGCAGCCACGCCGGGATCCTGGGCACGAGCAGGCCGACGCACTACTACAGCCTG
GTGGACGAGCACGGCTTCCGGTCCGACGACCTGCAGAAGCTGGTCTACAACCTCTGCTTC
GTGTTCGCGCGGTGCACCAAGCCCGTGTCGCTGGCGACGCCCGTCTACTACGCCGACCTC
GCGGCGTACCGTGGCAGGCTCTACTACGAG------------------------------

>Bdistachyon_Bradi5g18540
ATGGTTAGGAAGAAGAGAACTGGTCCTGCCAGCCCTGGAGAGAGTTCTGGGGAGGCTTCG
GGAGCTTCTGGGCAGGGTTCCTCACAGCGAACTGAAAGAGCTCCTCAACAGCATGGTGGT
GGACGTGGTTGGGTACCTCCACAGGGTGGCCGTGGTGGTGGGCCACACCAGGGGCGTGGT
GGACCCTATCAGGGCCCTGGTGGGCCAGCTCCACCGGAATTTCAACAGCGTGATTATCAG
GGACGTGGACATGCAGGTGGTGGCCCACCTGAATATCAACAGCGTGACTATCAGGGACGT
GGACAGCGTGACTATCAGGGACGTGGACATCCAGGTGGTGGTCCACCTGAATATCAGCCG
CGTGACTATCAGGGACGTGGTGGTCCAAGGCCCAGAggtGGTGGAATGCCGCAGCCATAC
TATGGTGGGCATAGGGGAGGTAGTGGTGGACGCAATGTTCCCCCAGGTCCATCAAGAACA
GTTCCCGAGCTGCACCAAGCCCCTCATGTCCAATATCAAGCCCCGATGGTTTCCCCATCC
GCATCGGGAGCTGGCTCATCCTCTCAGCCCGTGGTGGAGGTGAGCTCTCCAGTTGGCCGA
TCATTTTATTCTCCAAACTTAGGGAGACGTCAGAAACTTGGTGAAGGTTTGGAAAGTTGG
CGTGGATTTTACCAAAGTATAAGACCTACACAGATGGGACTATCGCTTAATATTGATATG
TCTTCTACTGCATTTATTGAGCCTCTCCCTGTGATTGAATTTGTTGCTCAGCTTCTGTGT
AGAGACATCTCAGTTAGACCGCTGTCTGATTCAGATCGTGTGAAGATTAAAAAGGCGTTG
CGAGGTGTAAAGGTGGAGGTGACGCATCGAGGGAACATGCGTAGGAAATATCGTATATCT
GGTCTAACGTCACAAGCTACACGAGAGCTATCATTCCCTGTCGATGAACGTGGTACTGTA
AAAACCGTGGTGCAATACTTCCTGGAGACATATGGTTTTAATATTCAGCACACCACTCTG
CCTTGCTTGCAAGTGGGCAATCAGCAGAGGCCGAATTATCTTCCTATGGAGGTTTGTAAG
ATTGTTGAAGGACAACGTTACTCGAAGCGCCTGAATGAGAAACAGATAACTGCTCTACTG
AAAGTCACTTGCCAACGCCTGCTAATTGTTATACTGCCTGACAATAATGGTTCTCTTTAT
GGGGATCTCAAAAGGATTTGCGAGACTGATCTGGGATTGGTATCTCAATGTTGTCTCACA
AAGCACGTCTTTAAGATGAGCAAGCAGTATCTTGCCAATGTAGCACTTAAAATTAATGTT
AAGGTAGGAGGAAGGAATACTGTACTTGTTGATGCTTTGACAAGGAGGATTCCCCTTGTT
AGTGACAGACCAACCATAATATTTGGTGCTGATGTTACACATCCTCATCCTGGTGAAGAT
TCCAGTCCTTCCATTGCCGCAGTGGTTGCTTCCCAAGACTGGCCTGAGGTCACCAAGTAT
GCAGGATTAGTGAGCGCACAAGCCCATAGACAAGAGTTGATACAGGATCTATTTAAAGTA
TGGCAGGATCCCCAAAGAGGAACTGTGACTGGTGGCATGATCAAGGAGCTTCTCATTTCT
TTCAAGAGGGCAACTGGGCAAAAGCCACAGCGAATCATATTTACAGGGATGGTGTCAGC

GAAGGACAGTTCTATCAAGTTCTGTTGTATGAGCTTGATGCCATTAGAAAGGCCTGTGCA
TCCTTGGAGCCAAATTATCAGCCTCCAGTTACCTTCGTAGTTGTCCAGAAGCGTCATCAT
ACCAGGCTTTTTGCTAATAACCACAACGATCAGCGTACTGTGGATAGAAGCGGGAATATA
CTGCCTGGCACTGTTGTTGATTCCAAGATTTGCCATCCGACAGAGTTTGATTTCTACTTG
TGTAGCCATGCTGGCATTCAGGGAACAAGCCGCCCTGCTCATTATCATGTTCTGTGGGAT
GAGAACAAATTTACTGCTGATGAGTTGCAAACTCTCACAAACAACTTGTGCTACACGTAC
GCAAGGTGCACCCGCTCTGTATCAATAGTGCCGCCGGCATATTATGCACATTTGGCGGCC
TTCCGAGCAAGATTTTACATGGAG------------------------------------

>Ptrichocarpa_POPTR_0012s11920
ATCTCTGTTGGTCGTGGCTTCCATTCTGTTAGAGCTCACCAAGATTACCTTGGGTATGGA
ATCATAGCATCTAAAGGTTGTCAACATAGCCTCAAGCCGACCTCCCAGGGCCTAGCTTTA
TGCTTGGATTATTCTGTTCTGTCATTTCATGAGCCAGTTTCTGTGATAGATTTCTTGACA
AAACATATTTGTGGGTTTAATTTAAATAATTTTAGAAGATGTAGGGGAGATGTGGAAATT
GCATTGAAAGGACTCAAAGTTAGAGTGACTCACCGTGTCACCAAACAAAAATATGTTATT
GTTGGGTTGACGAGAGATGACACACGAGATATTACATTTTCTCAAGAAGATCCAGATGGC
AAGGCTTCACAGAATGTTAGGCTTGTTGATTATTTCAGGCAAAAATATGGCAGGGATATA
GTGCATCAAGATATCCCTTGCCTAGAAATGAAAAGCAACATGAGAAACTATGTACCGATG
GAGTACTGTGTCTTGGTTGAAGGGCAAGTATTTCCAAAAGAGCATCTGCAGAGAGACGAA
GCCCAGATGTTGAAGGACATCTCACTAGCCCAGTTTCTTCTCTGTGTCATGTCAAAGAAG
GATCCTGGTTACAAGTATCTCAAATGGATTTGTGAAACCAAAGTTGGTATTGTGACACAA
TGCTGTCTGTCTCGTTCTGCGAATAAAGTAAATGATCAGTACCTTGCCAATATTGGTCTC
AAGATTAATGCTAAGCTTGGAGGAAGCAATGCAGAGCTCAGCGACAGACTTCCATACTTT
GGGGATGAAAACCACATCATGTTTATTGGGGCTGATGTCAACCATCCTGCTGCTCGAAAC
ACAACAAGTCCATCCATTGCTGCTGTTGTTGGTACTACAAATTGGCCAGCTGCAAATCGC
TATGCAGCTCGAGTTCGTCCTCAGGACCATCGTTGTGAGAAGATTCTGAATTTTGGTGAT
ATGTGTTTGGAGCTTGTTGAATTTTATTCTCGGCTCAATAAAGCAAAACCTGAGAAGATT
GTGATATTCCGTGATGGGGTCAGTGAGGGCCAGTTTGATATGGTTCTCAATGACGAGTTA
ATGGACATCAAGAGGGCGTTCAGGTCAATCATGTACACCCCAACCATCACACTCATTGTT
GCCCAGAAGCGGCACCAGACTCGTCTTTTTCTTGAGGATGGGGGGCGAATAGGCAATGTG
TCTCCGGGTACAGTTGTGGACACAAAAATTGTCCATCCTTTCGAGTATGATTTTTACCTC
TGCAGCCACTACGGAAGCCTTGGGACAAGCAAGCCCACACATTACCATGTTCTATGGGAT
GAGCATGGCCTCAGTTCTGATCAGTTGCAGAAGCTCATATATGACATGTGCTTTACATTT
GCTCGTTGCACTAAACCTGTGTCGTTAGTTCCACCTGTCTACTATGCTGACCTTGTTGCT
TATAGAGGAAGGCTCTACCATGAG------------------------------------

>Ptrichocarpa_POPTR_0008s01100
CTGTTGGTCCGACAGTCGTTTTTCCATAATAATCCAAGAAACTTCGTTGAGTTGGGAGGT
GGTGTCATGGGATGCCGTGGTTTTCATTCAAGTTTTCGAGCTGCCCAGGATGGCCTATCC
CTGAATATTGATGTATCAACCACCATGATAGTTAAACCTGGACCAGTAGTGGACTTCCTA
ATCATGAATCAGAATGTTCGAGATCCTTACCATATTGACTGGACAAAGGCTAAGAGGATG
CTTAAAAATTTGAGGATTAAAACTAATCACTCCAACACTGAGTACAAAATCACTGGATTG
ACTGAAAAATCCTGCAGAGAGCAAACTTTCTCGCTAAACCAAAGAAGTGGACGGGACGGG
GATGGTGAAGTGCAAACTATTGAGGTTACGGTTTATGATTACTTTGTCAATCATCGCAAC
ATGGGATTGCAGTATTCAGCTGATTTCCCGTGCATTAATGTTGGGAAACCAAAGCGCCCA
TCGTATTTTCCTCTTGAGCTGTGCAATCTGGTTTCGTTACAACGCTACACCAAAGCGTTG
TCCAGCCTGCAAAGGGCTTCCCTTGTGGAGAAATCACGGCAGAAGTTTCTGTTGTGTATT
CTTCCAGAGAGGAAGAACTCTGACATTTATGGTCCATGGAAAAGGAAAAATCTTTCTGAT
TTAGGGATCGTCACACAGTGCATTGCACCTACAAAGGTGAATGATCAGTACCTGACAAAT
GTGCTACTGAAAATCAATGCCAAGgtagaattttgcaatgttataccgTTTGAGCTTGGT
GGAATGAATTCCTTGTTATCAATTGAGCATGCTCCTTCTATACCTTTGGTGTCTAAGCTT
CCCACCTTAATACTTGGGATGGATGTATCACATGGCTCTCCTGGTCATTCTGATGTGCCA
TCTATTGCTGCGGTGGTAAGTTCCAGGCACTGGCCATTAATTTCTCGCTACAGAGCTTCT

GTTCGCACCCAGTCACAGAAGGTTGAAATGATTGCAAATCTATTCAAGCCTGTTGCTGGT
ACTCGTGAGGATCAGGGCATTATCAGGGAGTCACTTCTGGACTTCTACTCGAGCTCAGGA
AAAAGAAAGCCTGATCAGATAATTATTTTCAGGGATGGAGTGAGCGAGTCACAGTTCATC
CAAGTTCTAAACATTGAACTTGAACAAATTATTGAGGCCTGCAAGTTCTTGGATGAGAAC
TGGTGCCCGAAATTCATGGTGATTGTTGCCCAGAAAAATCACCATACCAAGTTCTTCCAG
TCGGGATCACCTGATAATGTTCCACCTGGTACTGTCATTGACAACAAGGTTTGCCATCCC
AGAAACAATGACTTCTACATGTGTGCTCATGCTGGGATGATTGGAACGACTCGGCCTACT
CATTACCATGTTCTACATGATGAGCTTGGTTTTTCTGCAGATGATTTACAAGAGCTTGTG
CACTCCTTGTCATATGTGTATCAGAGGAGCACCACTGCCATATCTGTAGTTGCTCCAATT
TGCTATGCCCACTTAGCTGCAAGCCAGATGACTCAGTTTATCAAA---------------

>Gmax_Glyma16g34300
ATGGTCAGAAAGAGAAGAACTGAACTACCAAGTGGGGGTGAAAGCTCTGAGGCTCAACAC
CCTTCTGAAAGGAGTGCACCACCGCCCCAACAACAAGCTGCTGCTGCTGCCCCAGGAGGG
GCTGGACCCCAAGGAGGCAGAGGCTGGGGTCCCCAAGGAGGAAGAGGAGGGGGCCGCAGC
CGTGGGATGCCCCAACAGCAATATGGTGCCCCTCCTGATTATCAAGGTAGGGGAAGGGGA
GGGCCTTCTCAGCAAGGAGGCCGTGGAGGATATGGCAGTGGCCGAAGTGGTGGTGGTGGT
GGTGGTATGGGCAGTGGCCGTGGCGTTGGTCCTTCATATGGTGGCCCATCGAGGCCACCG
GCACCCGAGCTGCACCAAGCAACCTCAGTTCAGTCATATCAAACTGGGGTGAGTTCTCAG
CCTGCATCATCTGAGGCCAGTTCATCCCTGCCGCCAGAGCCTATTGATTTGTGTCCTGTT
GGAAGATCATTTTATTCACCTGATTTGGGCAGAAGACAGCCTTTAGGTGAGGGATTGGAA
AGCTGGCGTGGTTTCTACCAGAGTATTCGGCCTACACAGATGGGGCTATCACTGAACATT
GATATGTCTTCCACTGCATTTATTGAGCCATTGCCAGTAATTGATTTCGTAACTCAACTG
CTGAACAGAGATGTATCCGCCCGGCCACTTTCTGATGCTGATCGTGTGAAGATCAAGAAA
GCTCTCCGGGGTATCAAAGTTGAAGTGACACATCGTGGAAACATGAGAAGAAAATATCGT
ATCTCTGGTCTGACTTCACAGGCAACCAGAGAATTGACATTCCCGGTAGATGAAAGGGGA
ACCATGAAATCTGTTGTGGAGTACTTCTATGAGACATATGGGTTTGTCATTCAACATACT
CAGTGGCCTTGTCTGCAAGTTGGCAATGCACAGAGACCAAACTATTTGCCAATGGAGGTT
TGCAAGATAGTGGAGGGTCAAAGGTACTCGAAAAGGCTTAATGAGAGGCAAATCACTAAT
TTGCTGAGAGTTACATGCCAGCGTTTGCTCATCGTTATCTTGCCCGATAATAATGGATCT
CTTTATGGTGATCTCAAACGGATATGTGAGACTGACCTAGGACTTGTTTCACAATGTTGC
TTAACTAAGCATGTCTTCAAAATGAGCAAGCAGTACCTTGCAAATGTTGCTTTGAAAATT
AATGTCAAAGTTGGAGGGAGAAACACTGTACTGGTTGATGCGCTCTCACGACGCATTCCC
TTGGTCAGTGACAGACCTACAATTATTTTTGGAGCTGATGTGACTCATCCACATCCTGGA
GAGGATTCAAGCCCATCAATTGCAGCAGTTGTGGCTTCGCAAGACTATCCTGAAATTACA
AAGTATGCTGGTTTAGTTTGTGCCCAAGTTCATAGGCAGGAACTCATTCAGGATCTTTTC
AAACAATGGCAAGATCCAGTCAGAGGAACAGTGACTGGTGGAATGATCAAGGAACTTCTT
ATATCTTTTAGGAGAGCTACAGGACAAAAGCCACAACGCATCATATTTTATAGGGATGGT
GTGAGTGAGGGACAGTTTTATCAGGTTCTACTGTTTGAGCTTGATGCTATTCGAAAGGCA
TGTGCATCCCTGGAACCCAATTATCAGCCTCCTGTGACTTTTGTGGTGGTTCAAAAGCGT
CACCACACAAGGCTCTTTGCTAGCAACCATCATGATAAGAGTTCTGTTGACAAGAGTGGC
AACATATTGCCTGGCACTGTTGTTGACTCCAAAATCTGCCATCCCACCGAATTTGACTTT
TATCTCTGCAGCCATGCTGGAATACAGGGTACAAGCCGTCCTGCTCACTACCATGTGTTG
TGGGATGAAAACAATTTTACTGCTGATGCCTTGCAAACACTCACCAACAATCTTTGCTAC
ACATATGCTCGGTGCACCCGGTCTGTTTCAATCGTGCCTCCTGCATACTATGCTCACCTT
GCTGCATTCCGCGCAAGGTTTACATGGAA-------------------------------

>Vvinifera_GSVIVT00023378001
CTTCTTGTCCGTCAATCATTCTTTCATGATAACTCAAGGAATTTTACAGACTTGGGAGGT
GGTGTCCTTGGATGCAGAGGGTTTCATTCCAGTTTTCGAGCCACACAAGGTGGTTTATCT
CTAAATGTTGATGGGTCCACTACTACAATCATACAGCCTGGACCGCTTGTTGATTTTCTC
ATTGCAAACCAAAATGCACGAGATCCATTCCAGCTCGACTGGTCAAAGGCTAAACGGACT

CTCAAAAATTTAAGGATAAAGGTAAAACCTTCTAATTCAGAGTACAGAATTGTTGGACTG
AGTGAAAGTCCTTGCAAAGAGCAGATGTTCACCCTAAAAAATAGGGGAAAAAATGGAAAT
GATGATGCTGAAAGTATAGAAGTGACGGTTTATGATTATTTTGTTAACTATCGCCAGATA
GAGTTGCGTTATTCTGGAGACTTACCTTGCATCAATGTTGGGAAACCAAAAAGGCCTACG
TACTTGCCTATTGAGCTTTGTTTTTTGGTTTCTTTACAACGCTATACGAAGGCATTAACT
GTTCATCAAAGGTCCACCTTGGTAGAAAGATCAAGACAAAAGTTTATTGTCTGCCTTCTT
CCTGATAGGAAGAACTCTGACATATATGGTCCATGGAAAAGGAAGTGCCTTGCAGAATTT
GGAATTTTTAATCAATGCCTTGCACCTACTAGAGTTAATGATCAGTATATCATGAATGTA
CTTTTGAAGATCAATGCCAAACTTGGTGGTTTGAATTCTCTGTTAGCCATTGAACCATCA
AGAAATATACCCTTGGTTTCCAAGGTTCCTACCATAATCTTTGGAATGGATGTGTCACAT
GGCTCCCCTGGCCAATCTGATATTCCATCTGTTGCTGCTGTTGTGAGTTCTAGGTGTTGG
CCACTGATCTCTCGTTACAGAGCTTCTGTTCGTACACAGTCACCGAAAGTTGAAATGATA
GATTCGCTTTTTAAGCCAGTATCAGATGATAAAGATTTAGGCATTGTCAGGGAGCTGCTG
TTGGACTTTTATGTGAGTTCAGGGCAAACAAAGCCCACTCAAATAATCATTTTCAGGGAT
GGAGTCAGTGAATCGCAGTTCAACCAAGTCTTGAACATTGAGCTAGATCAAATTATTGAG
GCTTGCAAGTTCCTCGATGAGAAGTGGACTCCTAAATTCACAATAATTATTGCCCAGAAA
AATCACCACACAAAATTCTTCCAAGCTGGGTCTCAAGATAATGTGCCTCCTGGAACTGTA
ATTGACAGCAAAGTCTGTCATCCAACGCACAACGATTTCTACATGTGCGCACATGCAGGG
ATGATAGGGACAACAAGACCAACACATTACCATGTTCTTCTAGACGAGATAGGCTTTTCA
GCTGATGATATGCAAGAACTCATCCATTCTCTGTCCTATGTGTATCAGAGAAGCACTACT
GCCATATCCATCGTCGCCCCAGTTCGCTATGCACACCTGGCGGCTACACAGGTTTCACAG
TTCATGAAG---

>GRMZM2G411082_T01 cds:_protein_coding
GCACCGTTTGGGCGATCGTTTTTCTCTCCTGACCTGGGGAGGAGGCGTTCCCTTGGTGAG
GGAATAGAAAGCTGGCGTGGGTTTTACCAGAGCATTCGCCCTACTCAAATGGGCTTGTCA
TTGAATATTGATATGTCTGCAACTGCTTTCTTTGAGCCATTACCTGTCATAGATTTTGTT
GCACAGCTTTTAAACACCGACGACATCTACTCGAGGCCCCTCTTAGACGCTGAACGTGTC
AAGATAAAGAAGGCCTTAAGAGGCGTGAAGGTGGAAGTTACGCACCGTGGTAACATGCGA
CGCAAGTATCGAATAGCTGGTTTAACATCTCAGGAAACTCGGGAGCTAACTTTTCCTGTT
GATCAAGGTGGCACAGTGAAGTCAGTTGTACAGTATTTTCAAGAGACCTATGGCTTTGCC
ATCCAGCACACCTACCTGCCCTGTCTGCAGGTTGGCAACCAGCAGCACCCAAATTACCTT
CCAATGGAGGTCTGCAAGATAGTGGAGGGACAGAGGTACTCTAAGAGATTAAACCAGAGT
CAGATAAGAGCTCTTTTAGAGGAGACATGCCAACGCCTGCTCATTGGAATACTCCCTGAT
AACAACGGTTCTCTTTATGGTGATCTGAAGCGTATCTGTGAAATCGACCTTGGATTAGTT
TCCCAGTGCTGCTGCGCAAAGCAAGTCTTTAAGATGAACAAGCAGATACTGGCAAACCTT
GCGCTGAAGATAAATGTCAAGGTCGGAGGAAGGAACACGGTGCTGGCTGATGCGGTGTCA
AGACGCATTCCCTTGGTGACTGACAGGCCTACCATCATATTCGGTGCCGATGTGACCCAT
CCTCATCCTGGAGAAGATAGCAGCCCTTCCATTGCTGCTGTTGTGGCTTCCCAAGACTGG
CCTGAGGTGACAAAGTATGCTGGTCTAGTTTCTGCTCAGTCTCATAGGCAAGAGCTGATA
GAGGATCTCTACAACGTCACACACGATCCTCAGAAAGGAACCGTTTGTGGTGGCATGGTC
AGGGAGCTTCTTATATCCTTCAAAAAATCAACTGGTCAAAAGCCTCAGAGGATACTATTC
TACAGGGATGGTGTGAGTGAAGGGCAGTTCTACCAAGTTCTACTGCATGAACTGGATGCT
ATCCGAAAGGCGTGTGCATCGCTGGAAGCAAACTACCAACCGCAGGTGACTTTCATCGTC
GTCCAGAAACGCCACCATACCAGGCTGTTCGCGCACAACCACAACGACCAGAATTCGGTC
GACAGGAGCGGCAACATACTTCCTGGAACTGTCGTGGACTCGAAGATCTGCCACCCTACA
GAGTTCGACTTCTTCCTGTGCAGCCATGCTGGCATCAAGGGCACCAGCCGTCCTGCTCAC
TACCATGTCTTGTGGGACGAGAACAACTTCACAGCCGACGCACTGCAGACCCTCACCAAC
AACCTTTGCTACACCTACGCGAGGTGCACGCGCTCTGTGTCCATTGTCCCGCCGGCGTAC
TACGCTCACCTGGCCGCATTCCGCGCCCGGTTCTACATGGAG-----------------

>Gmax_Glyma02g44260
TTGCTTGTGAGGCAATCGTTCTTCCACAATGATCCCAAGAATTTTGCTGATGTAGGAGGA
GGTGTACTTGGATGCAGGGGTTTCCATTCTAGCTTTAGAACTACACAAAGTGGACTGTCT
TTGAACATAGATGTCTCAACCACGATGATAATTACCCCTGGGCCTGTGGTGGACTTCCTA
ATATCCAATCAAAATGTGAGAGACCCCTTTTCACTTGACTGGGCCAAGGCCAAGAGGACA
TTAAAAAATCTGAGGATTAAAGCGAGCCCATCTAATCAAGAATTCAAAATAACTGGGATT
AGTGAATTCCCTTGCAAAGATCAAACGTTTACCTTGAAGAGAAAAGGTGGTGATGATGTT
GCTGAGGAAGAAGTGACAGTATATGATTATTTTGTTAATATCCGCAAGATAGATCTTCGA
TATTCTGGGGATCTCCCATGTATTAACGTTGGAAAGCCAAAAAGGCCAACTTATATCCCT
CTTGAGCTTTGCTCTTTGGTATCCCTGCAACGTTATACAAAAGCACTATCCACTCTTCAA
AGGGCTTCATTGGTGGAGAAGTCCAGGCAGAAGTTTCTTCTTTGTCTGCTTCCTGAGAGG
AAAAATTCTGATCTTTATGGTCCATGGAAGAAGAAGAATCTTGCTGAGTTTGGAATCGTG
ACTCAGTGTATAGCTCCGACCAGGGTCAATGACCAATATTTGACTAATGTTCTGTTGAAA
ATCAATGCTAAGCTTGGTGGCCTGAACTCAATACTAGGTGTTGAGCATTCTCCTTCTATT
CCTATTGTTTCTAGAGCACCAACCATTATTATTGGCATGGATGTGTCTCATGGTTCGCCA
GGGCAAACAGATATTCCTTCAATTGCCGCGGTGGTCAGCTCCCGAGAATGGCCACTAATA
TCAAAGTATAGGGCTAGCGTCCGTACCCAGTCTCCAAAGATGGAAATGATTGATAATTTG
TTCAAGAAGGTTTCTGACAAGGAGGATGAAGGCATAATGAGGGAGCTTCTACTTGATTTC
TATACAAGTTCTGGGAATAGAAAGCCCGATAATATAATCATATTCAGGGACGGTGTTAGT
GAGTCCCAGTTCAATCAAGTTTTGAACATTGAACTTGATCAAATTATCGAGGCTTGCAAG
TTTTTAGATGAAAAGTGGAACCCCAAGTTTTTGGTGATTGTTGCTCAAAAGAACCATCAT
ACTAAATTCTTTCAACCTGGAGCTCCTGACAATGTTCCTCCTGGAACTGTAATTGATAAC
AAAATTTGCCATCCTCGGAATTATGATTTCTATATGTGTGCACATGCTGGAATGATTGGT
ACTAGCAGGCCCACACACTACCATGTTCTGTTAGACGAGATTGGCTTTTCACCTGATGAT
CTACAAGAGCTTGTACATTCATTATCATATGTGTATCAGAGGAGCACCACTGCCATTTCT
GTCGTTGCTCCAATTTGCTATGCTCATCTGGCTGCTACTCAGATGGGTCAGTTTATGAAA

>AGO1906_Aquilegia
TTACTAATTGTTATTTTGCCAGACAATAATGGTTCTCTTTATGGTGATTTGAAACGAATA
TGTGAGACAGATCTTGGTCTTGTTTCACAGTGTTGTTTAACAAAGCATGTTTTTAGGATG
AGTAAGCAGTATATGGCAAATGTAGCTCTTAAGATTAATGTGAAGGTTGGTGGAAGGAAC
ACAGTGCTTGTTGATGCGTTGTCAAGGCGCATACCTTTGGTTAGTGATCGACCTACTATC
ATTTTTGGTGCTGATGTTACTCATCCTCATCCTGGAGAGGACTCAAGCCCGTCAATTGCA
GCTGTTGTCGCTTCTCAAGATTGGCCAGAAGTTACTAAATATGCTGGTTTGGTATGTGCT
CAAGCCCATCGCCAAGAACTCATTCAAGATCTTTACAAAACCTGGCATGATCCTGTTAAA
GGAACTATGCATGGGGGCATGGTGAAGGAACTTTTGATATCCTTCCGCAGAGCAACTGGA
CAGAAGCCTGAGCGCATTATCTTTTACAGGGATGGGGTCAGCGAGGGACAGTTTTACCAG
GTCTTGCTGTATGAGCTGGATGCAATTAGAAAGGCATGTGCCTCCCTGGAGCCGAATTAC
CAGCCACCTGTTACTTTTGTTGTGGTTCAGAAGCGTCACCACACACGACTGTTTGCGAAC
AACCATGCAGATCGTCGTGCAGTGGACAAAAGTGGCAACATTTTGCCAGGAACGGTGGTA
GACTCAAAGATCTGTCATCCGACAGAGTTTGACTTTTATCTTTGCAGTCACGCTGGGATA
CAGGGCACTAGTCGTCCAGCCCATTATCATGTTCTCTGGGATGAGAACAAATTTACTGCC

GATGGACTCCAGACTTTAACCAATAATCTCTGTTACACATATGCGCGATGCACCCGCTCA
GTTTCCATTGTACCTCCGGCATACTATGCCCACTTGGCTGCCTTTCGTGCACGTTTTTAC
ATGGAG--

>Ptrichocarpa_POPTR_0010s09150
TGCCCTGTTGGAAGATCCTTTTTTTCACCCGATATAAGAGCACCACAGCGACTAGGTGAT
GGCTTGGAATCCTGGTGCGGATTTTACCAGAGTATAAGACCGACTCAAATGGGACTGTCC
TTAAACATTGATATGGCCTCGGCAGCATTCATTGAGCCTCTTCCTGTGATAGAGTTTGTC
GCTCAGCTTCTAGGCAAAGATGTATTGTCAAGGCCATTGTCTGATTCTGATCGAGTGAAG
ATTAAAAAAGGTCTAAGAGGAGTGAAAGTTGAAGTAACTCACAGAGGGAGTGTACGAAGA
AAGTATCGTGTCTCGGGCTTGACATCTCAGCCTACAAGAGAACTTGTGTTTCCTGTTGAT
GATAACTCGACAATGAAGTCAGTTGTTGAATACTTCCAAGAGATGTATGGCTTCACCATT
CAACATGCACATCTACCTTGCCTTCAGGTTGGAAACCAGAAGAAAGCAAATTATCTACCC
ATGGAGGCTTGCAAAATTGTGGAGGGGCAACGGTATACAAAACGGTTGAATGAGAGGCAA
ATTACTGCCCTTCTAAAAGTTACATGTCAAAGACTTCTCTTAGCTATTCTACCTGACAAC
AATGGGTCCCTATATGGTGATCTGAAGCGAATATGCGAAACTGATCTTGGTTTACTAACT
CAATGCTGTCTCTCGAAACATGTGTTCAAGATCAGTAAGCAGTACTTGGCAAATGTGTCC
CTCAAGATCAATGTAAAGATGGGTGGTAGAAATACTGTCCTTTTAGATGCTATCAGCTGC
AGAATACCATTAGTTAGCGACATACCAACCATTATTTTTGGAGCAGATGTGACTCACCCA
GAGAATGGAGAGGACTCAAGCCCCTCAATAGCTGCTGTGGTGGCTTCTCAGGACTGGCCT
GAAGTAACAAAATATGCTGGACTAGTTTGTGCTCAAGCTCACAGACAGGAACTCATACAG
GACTTGTACAAAACATGGCAAGATCCTGTTCGTGGTACTGTTAGCGGTGGCATGATCAGA
GATCTTCTGATTTCTTTCAGGAAAGCAACTGGACAAAAGCCGCTAAGGATCATATTTTAC
AGGcgagtattgGATGGTGTTAGTGAAGGACAATTTTATCAAGTTCTGCTTTATGAGTTG
GATGCGATTCGGAAGGCCTGTGCGTCTTTGGAGCCAAATTATCAGCCACCAGTAACTTTC
ATAGTTGTACAAAAACGTCACCACACTAGATTGTTTGCTAACAACCATAGGGACAGGACT
AGCACAGACAAGAGTGGAAACATACTGCCTGGCACTGTGGTTGATTCTAAAATCTGTCAT
CCAACAGAGTTTGACTTCTATCTCTGTAGCCATGCTGGTATTCAGGGGACAAGTAGGCCA
GCGCACTATCATGTTTTGTGGGATGAGAACAACTTCACAGCTGATGGAATCCAGTCTTTG
ACGAACAATCTCTGCTACACATATGCTAGGTGCACTCGCTCCGTTTCAGTAGTTCCTCCG
GCATACTATGCACATTTAGCTGCATTTCGTGCTCGATTTTACACGGAG------------

>Mesculenta_cassava7327.valid.m1
GTTTCTGTCGGCCGCAGCTTTCACTCAGTTGAAGCTAATGAAGAAGATGACCTTGGATTT
GGCATCACAGCTTCCAGAGGGTTTCAACATAGCCTTAAGCCCACCTTCCAGGGTCTAGCC

ATGTGCTTGGACTATTCTGTCCTGGCATTTCGCAAGCGACTTCCAGTAATAAATTTTCTT
ATGGAGCATATTCCAGGGTTTAACTTAAATGATTTCGGAAGCTTTAGAAAAGATGTTGAA
AATGCACTGAAGGGACTGAAAGTTACAGTGACTCACCGTGTTACCAAACAAAAATATACT
ATAGCTGGACTTACAAAGGATAATGCACTGTATCTTTCATTTTTGTCTGAAGACCCAAAT
GGCAAAACCCCACCTAAGAAAGTACATCTTGTTGAATATTTCAGGCAAAAATACCAGGAT
ATAAAATTCAAAGACATTCCTTGCCTTGATTTGGGCACAAACAACCGAAAAAATTATGTA
CCCATGGAGTTCTGTGTCCTAGTTGAGGGTCAGATTTATCCAAAGGAGCATTTGGATAGA
AATGCAGCCCTCTTTTTGAAGAAGATGTCGCTGGCATTTATTCTTTGTGTGATGTCTAGG
AAGGATTCTGGTTACAAGTATCTTAAGTGGATTTCTGAGACCAGAGTTGGTGTAGTGACT
CAATGTTGCTTGTCTGATCTTGCTAACAATGGGAATGACCAGTATCTTGCTAATCTTGCT
CTGAAGATAAATGCAAAGCTTGGAGGTAGCAATGTGGAGCTCATTGATCGACTTCCATTG
TTTGAAGGTGAGGGCCATGTTATGTTTGTTGGGGCTGATGTTAATCATCCTGGCTCCCGG
AACAAGACAAGTCCATCTATAGCTGCTGTTGTTGCCACTGTAAATTGGCCTGCTGCAAAC
CGCTATGCAGCTCGTGTTCGTCCCCAGGAACATCGTAAGGAGAAGATTATCAATTTTGGA
GATATGTGTGTTGAGCTTGTTGAAACTTATGTTCGGCTGAATAGAGTCAAACCAGGTAAT
ATTGTAATATTCCGTGACGGGGTAAGTGAGGGCCAGTTTGATATGGTTCTCAATGAAGAG
TTAATCGATCTCAAGAGAGCATTTAGATCAATCAATTATGCACCAACTGTTACACTTATT
GTGGCCCAAAAGCGGCACCAGACTCGTTTGTTTCCTGCAGGCAGGAGTGGTGGGAACTCA
AATGGGAATGTGTCTCCTGGCACAGTTGTGGACTCTAAAATTGTGCATCCATTTGAGTAT
GATTTCTATCTTTGTAGCCATTACGGAAGCCTTGGGACGAGCAAGCCCACACACTATCAT
GTACTGTGGGATGAACATGGTTTCAGTTCTGATCAATTGCAGAAGCTCATATATAATATG
TGCTACACATTTGCTCGATGCACAAAACCTGTGTCCTTGGTTCCACCAGTGTACTATGCT
GACCTTGTTGCTTACAGGGGAAGGCTGTATTATGAA------------------------

>Csativus_Cucsa.082260
ATATCAGTTGGACGATCCTTTTACTCTCCTTGTATTAAGAAGCCTCAGCATGTTGGGGGT
GGTCTGCAAGCATGGCGAGGCTTCTATCAAAGCATCCGGCCTACTCAAATGGGATTGTCT
CTAAATATTGACATGTCATCCACTGCATTCATTGAAGCAATCCCGGTCATTGATTTTGTC
GCTCAACTCTTAAACAAAGACGTGTACTCTAGAACATTGTCTGATGCTGATCGTATCAAG
GTCAAAAAAGTACTTAGAGGTGTAAAAGTTGAAGTTACACATAGAGGAAACGTACGAAGG
AAATATCGGATTTCAGGGCTAACATCACAGCCTACAAGAGAGCTAATTTTCCCTCTTGAT
GAGCAGATGAACATGAAATCTGTAGTTGAGTACTTCCAAGAGATGTATGGGTATACCATT
CAGTATACACATTTGCCTTGCCTTCAAGTAGGAAACCAGAAGAAGGTGAATTATCTACCC
ATGGAGGCATGTAAGATACTCAAAGGACAGAGATACACAAAAGGACTTAACGAAAAGCAA
ATAACTTCCTTGTTGAAGGTTTCATGCCAAAGATTGCTGATAGCTATTCTTCCTGACAAC
AATGGCTCATTATATGGTGATCTTAAAAGGATCTGTGAAACAGAATTGGGATTGATATCC
CAGTGTTGTCTAACAAAACATGTCTTCAAGAAGAGCAGACAGTACTTGGCGAACGTGTCT
CTTAAGATCAATGTCAAGATGGGTGGAAGAAACACTGTTCTTTTAGATGCTTTACGGGCA
AGAATTCCTCTAGTCAGTGACATACCAACCATTATTTTTGGAGCTGACGTCACACATCCA
GAATCTGGAGAGGATTCTCTTCCATCAATAGCAGCTGTTGTGGCTTCCCAAGATTGGCCA
GAAGTTACAAAATATGCTGGATTGGTATGTGCGCAGCCGCACAGGGAGGAACTAATTCAA
GATTTATTCAAAACGTGGAAGGATCCTCATCGGGGAACAGTTGCCGGGGGTATGATAAGG
GAGCTCTTGCTTTCATTTAAGAAGGCCACTGGACAAAAACCATTAAGGATAATATTCTAT
AGGGATGGGGTCAGTGAGGGTCAGTTCTACCAGGTTCTACTGCACGAACTCGATGCCATA
CGCAAGGCCTGTGCTTCACTAGAACCCAGTTACCAACCTCCAGTAACTTTTATCATAGTC
CAAAAGCGACACCACACCAGACTCTTTGCATCCAATCACAACGATAGGAGTAGCACTGAC
AAGAGTGGTAACATTTTACCAGGTACTGTCGTGGATTCAAAAATATGTCATCCAACTGAG
TTCGACTTTTATCTATGCAGTCACGCAGGAATCCAGGGAACAAGTCGTCCTGCACATTAT
CACGTTCTTTGGGATGAGAACAATTTCAGTGCGGATGAAATTCAATCTCTGACTAATAAT

CTCTGTTACACGTATGCTCGGTGCACAAGATCGGTCTCAGTAGTGCCTCCAGCATACTAC
GCTCATTTAGCAGCATACAGAGCTCGATTCTACGTAGAA---------------------

>Gmax_Glyma20g02820
GTTTCCTTGGGGCGGTGCTTCTTCCCCATGAACCCTCCTTTGAGGAAGAAAGATCTTAAC
CATGGCATAATTGCGATTGGAGGGTTTCAGCAGAGTCTTAAGTCTACTTCTCAGGGATTG
TCCTTGTGCCTGGACTATTCGGTTTTGTCCTTTCGGAAGAAGTTGGTGTTGGATTTTCTG
CACGAGCATATTAGGGACTTCAATTTAAGGGAGTTTGGGCGGTTCAGGAGACAAGTTGAG
CATGTACTTATTGGGTTGAAGGTTAATGTTAAACACCGGAAGACAAAGCAGAAGTACACT
ATTACTAGGTTGACACCCAAGGTTACGAGACATATCACATTCCCTATTTTGGATCCCGAG
GGCCGGAATCCCCCAAAGGAAGCTACTCTGGTTGGTTACTTTCTAGAGAAGTATGGTGTG
AACATTGAATACAAGGACATTCCTGCCTTGGATTTTGGAGGCAACAAGACGAATTTTGTG
CCTATGGAGTTGTGTGAGTTGGTTGAGGGGCAGAGATATCCCAAAGAGAATTTGGACAAA
TATGCTGCCAAGGACTTAAAAGACATGTCAGTGCAATTTCTTCTGTGTGTGATGTCCGAC
AAGCATCAAGGTTACAAGTGCCTCAAATGGATTGCTGAGACCAAGGTTGGCATAGTGACA
CAATGCTGCTTGTCTGGTATTGCTAATGAAGGGAAGGACCAATATCTTACAAATCTTGCC
CTCAAGATCAATGCCAAAATTGGAGGAAGTAATGTGGAGCTCATCAATAGGCTACCACAC
TTTGAGGGTGAAGGTCATGTTATGTTCATAGGGGCTGATGTCAATCATCCAGCTTCCCGG
GACATCAACAGTCCATCAATTGCTGCTGTAGTTGCCACTGTTAATTGGCCTGCTGCAAAT
CGCTATGCAGCACGTGTTTGTGCTCAAGGTCATCGGGTTGAGAAAATTTTGAATTTTGGG
AGAATTTGCTATGAACTTGTTTCGTATTACGATAGGCTGAACAAAGTCAGGCCTGAAAAA
ATTGTTGTCTTTCGTGATGGCGTGAGCGAAAGTCAATTCCATATGGTTCTCACAGAGGAG
TTACAAGATTTGAAATCGGTGTTTAGTGATGCAAATTACTTCCCAACCATCACTATTATT
GTCGCACAAAAGCGACATCAAACTCGATTTTTTCCTGTGGGTCCAAAGGATGGGATTCAA
AATGGCAATGTGTTTCCAGGTACAGTTGTGGACACAAAAGTAGTACATCCTTTTGAATTT
GACTTTTACCTTTGTAGTCACTATGGAAGCTTGGGTACTAGTAAGCCCACTCACTATCAT
GTCTTATGGGATGAGCATAAATTTAACTCTGATGATTTGCAGAAACTGATATATGACATG
TGCTTTACCTTTGCAAGGTGCACTAAACCTGTATCTTTAGTCCCTCCAGTGTACTATGCT
GATCTCACTGCATATAGAGGACGGTTATACTATGAA------------------------

>Rcommunis_29828.t000011
CTTCTTGTTCGTCAATCATTCTTTCATGATGATTCGAGGAACTATGTCGATCTGGATGGT
GGTGTACTCGGATGCAGGGGATTTCATTCAAGTTTTAGAGTCTCACAAGGCGGATTATCA
CTGAATATTGATGGGTCAACTACAACAATAATACAGCCTGGGCCATTAATTGACTTTCTT
CTAGCCAACCAACATGTGTCAACTCCTTTTCAAATTGATTGGTCAAAGGCTAAACGTACC
CTGAAAAAATTTGAGGATAAGGGTATCACCCACCAATCAGGAGTACAGAATCACTGGCTTG
AGTGAGAATCTTTGCAAGGACCAGATATTCTCAATGAAATCAAAAGGACTGAATGATGGA
AATTGCGATGATggaatgGTCGATATTACAGTTTATGAATATTTCGTTAATCATCGTAAT
ATAGACTTGCGCTACTCTGGTGATTTGCCTTGCATTAATGTTGGAAGGCCTAAACGACCT
ACTTTCTTTCCTATTGAGCTGTGTTCATTGCTTCCGTTGCAACGCTATACAAAGGCATTA
TCTGTTATTCAGAGGTCAAAGTTGGTTGAAAGTTCACGTCAAAAGTTTCTTCTGTCTATT
TTTCCTGATAGGAAGAATTCTGACATATATGGTCCATGGAAAAGGAAGAATCTGGCAGAA
TTTGGAATTTTCAATCAATGCCTTTGTTCACCTAATAGACTCAGTGAGATGTATGTTACG

AATGTTCTCATGAAGATAAATGCCAAGCTTGGTGGTTTAAATACTTTCTTGGCTGTTGAA
CAATCACGAAACGTCCCTTTCGTTTCAAAGGTTCCTACAATAATTTTTGGTATGGATGTT
TCACATGGTTCTCCTGGACAATCTGATGTGCCATCTATTGCTGCGGTGGTAAGTTCTAGG
AATTGGCCACTACTATCTCGTTATAGAGCTTCTGTTCATAGTCAGTCACCAAAGGTTGAG
ATGATTGATTCCCTTTTTAAACCAGAGGGAAAAGATGATGATGGAATAATCAGGGAGTTG
CTGCTGGACTTCTATAGGAGTTCTGGTCAGACAAAGCCGGCTCAGATAATCATATTCAGG
GATGGAGTCAGTGAGTCACAGTTTAATCAAGTCCTCAACATCGAGTTAAATCAAATCATT
GAGGCTTGCAAGTTCCTTGATGAAAGTTGGTCGCCTAAGTTCACTGTGATTGTAGCACAA
AAGAATCACCACACTAAATTCTTCCAATTACGATCTGCAGAAAATGTCCCTCCAGGAACT
GTTGTAGATAATGGTGTTTGCCACCCACAAAGCAATGATTTCTACATGTGTGCCCACGCA
GGGATGATTGGGACAACCAGGCCAACACATTACCATGTTCTGTTAGATGAAATTGGCTTT
TCAGCTGATGATCTACAAGAACTCATTCATTCTCTATCCTATGTGTATCAGAGAAGCACA
AGTGCAGTATCTGTAGTTGCTCCTGTTCGTTATGCTCATTTGGCAGCAACTCAAATAAGG
CTATTCATGAAG--

>Ppatens_1888444_locus
CTGCTTGTGAGAGACAATTTCTTCCATCCGAGTTTGGGTCAAGTTTGCGATTTGGGCGAT
GGTGTGGAAGGTTGGAGGGGTTATCACTCCAGCGTGAGACCGACGCTGATGGGATTGATG
TTAAATTTAGATACCACTATGACGGTGGTACTGAAGCCTGTTGATGAGTTTCTCAAGGAA
AGGTTCAATGTAAGAGACCTGTCTGGTTTGCAGAAAAGAGATTGGGCTAAAGCGAAGGAC
ATGTTGAAAAACGTCAGAATAGAAACGACTCACACGGGAGTGTCCCGAAAGTACAGAATA
TCGGGCTTCAGTGATCGGTCCATACGAGAATTAAAGTTTATGAAGGGGACGAAAGATGGG
AATGGGGAGGAGGAAATTTCAGTATATAATTATTTTTTTGATACTTACTCGCGCAAACTA
AAAAACCTAAATTTTCCAGCGCTGGATCTTGGAAACAGCAGGAAGCCAATATACATGCCC
ATAGAATTGTGCAAGATAGTTAGTGGACAGCGCTATACCAAGCCGCTGTCCAGCAAGCAG
AGGATGGCACAAATCGGTGCAAGCAAGCAGTTCATTCTTGCTATTCTAGCAGAAAAAGAC
AGTCCCATTTATGTTCCTTTCAAGCGGTTGTGTGAAATTAGACTTGGAATAATCTCGCAG
TGCATGGTGAAGCCGCGAACTCTGAACGATCAGTTCCTAGGAAACCTTGCGTTGAAGATT
AATTTGAAGATGGGAGGATTGAACTCGCCATTGAGTCAGCGGATGTTACATTGCCTTGGT
CAGTCGACCATAATCTTCGGCATGGATGTGACCCATGGGTCTCCTGGGGATGTCGAAATA
CCCTCAATTGCGGCAGTAGTCGCAACGAAGAATTGGCCCGAGGTATTCCATTATTCGACT
CAAGTTAAGGTCCAACCAGCCAGGATGGAGATGATTCAAGGGCTGTATGAACCTGAAGGT
GGCATGGTACGAGAATTGCTCATGTCGTTCTATTCGACATGCGCCAAAGGCGTCAACCCG
AAGCCATCCCAAATTATAATTTACAGGGATGGAGTTAGCGATAGCATGTTTGCGAAATGT
TTGGAAGTTGAGTTCGTCGCATTTAAACGAGCTTGTGCAGAACTCGAAGCGGGTTACAAT
CCCGGAATAACGTTCATTGTGGCTAAAAAGCGTCACGGCACGCGCTTCTTTCCTCAGAGT
CGAGATGCGTTGCGGAATGGGAACGTTCTACCAGGAACTGTTGTCGACAAGGATGCGTGC
CACCCTCGGAATTTTGACTTCTTCCTCATCTCTCAGGCCGGCCTCATTGGCACAGCTCGG
CCTACTCACTACACAATACTGGTGAATGAAAACCAGCTTGGGCCTGATGATATTCAGACC
TTGACCAACAAACTGTGCTACACGTTTGGACGCTGTACCTCATCCATCTCGATGGCGGCT
CCAGCCGCATATGCTCATATTCTGGCATCAAGATATCGGAAGTTGATGAGT---------

>Csativus_Cucsa.284770

ATACCTGTGGGAAGATCTCTGTATTCCAGTTCAATGTGGGGAGGCAAAGAAATTGGGGGA
GGAGTTGTTGGCTTAAGAGGCTTTTTCCAAAGCCTTAGACCCACACAACAAGGCTTAGCT
ATGAATGTAGATTGTTCTGTCACTGCTTTCCATGAAAGCCAAAAACGTCTTGATTTTCTT
AGAGATCTTTCTCAGAGGAAGACAAGAGGTTTAACCATTGAAGAAAAGAAGGAAGTGGAG
AAGGCTTTAATGCATATCAGAGTATTTGTTTGTCACAGAGAGAGTGTTCAAAGATATAGA
GTTTATGGCTTGACAGATGAACCTACTGAGAGTCTCTGGTTTGCTGATAGAAATGGGAAG
AATCTAAGATTGGTGGGTTACTTCAAGGATCATTATAACTATGACATTCAATTCAGGAAT
TTGCCTTGTTTGCAGATTAGTAGGAGTAAGCCATGTTATCTTCCTATGGAGCTTTGTATG
ATTTGTGAAGGGCAAAAGTTTCTTGGGAAGCTCACTGATGAACAGACTACAAGAATGCTT
AAGATGGGTTGCCAGAGGCTCTTGATATGTGTGATGGAGAGAAAACACAAAGGCTATGCA
GATTTGAAACGCATTGCCGAGACCAGTATCGGCGTTGTAAGTCAATGTTGCCTTTATCCT
AATCTTGCCAAGTTGAGCTCTCAATTTCTGGCTAATTTGGCTCTTAAGATTAATGCCAAG
GTTGGTGGTTGCACTGTTGCTTTATATAATTCGTTACATTCACAAGTACCTCGACTGCTC
CGAATCGATAAGCCTGTGATCTTCATGGGTGCAGATGTAACTCATCCTCATCCCTTGGAT
GATTTTAGCCCCTCCATCGCTGCTGTGGTTGGTAGCATGAATTGGCCAGCAGCAAACAAG
TACGTCTCCAGAATGAGATCACAAACACACAGACAAGAAATTATTGTGGATCTTGGAACA
ATGGTCGAAGAGCTGCTTGAGGAATTCTATCAAGAAGTGAATGAATTGCCATCACGAATC
ATATTCTTCAGAGACGGTGTAAGTGAAACCCAGTTCTACAAGGTGCTTCAAGAGGAATTG
CAAGCTATAAAAACAGCCTGTTCTAGGTTTCTCAATTATAAACCTCCTATAACTTTTGCT
GTTGTTCAGAAGAGACATCACACAAGATTGTTCCCATTCAAAGTCGATCCATCTTCTAAT
CAAACTGAGGTTCTCGATGAAAATATTCCTCCCGGGACAGTCGTTGATACCGTAATTACG
CACCCGAAGGAATTCGATTTCTATCTCTGCAGCCATTGGGGGGTGAAGGGAACAAGCAGG
CCTACACATTATCATATTCTGTGTGATGAGAATCAATTCACTTCTGATGAACTACAAAAG
CTAGTTTACAACTTATGCTACACATACAACAGGTGCACAAAGCCTGTTTCATTAGTACCT
CCAGCTTATTATGCTCATCTTGCTGCTTATAGAGGCAGACTTTACCTTGAA---------

>Vvinifera_GSVIVT00016822001
GTATCAGTTGGGAGATGCTTATATTCTCCTGATATTAAAAATCCTCAGCAACTAGGTGGG
GGTTTACAATCATGGCAAGGCTTCTATAAGAGTATAAGGCCAACTCAGATGGGGTTATCA
TTGAACATCGATATGTCATCAACTGCATTCATTGAACCATTACCTGTTATTGACTTTGTG
GCTCAACTTTTGGACAAAGATGTATTTTCAAGGCCATTGTCAGATGCAGACCGTGTCAAG
GTTAAGAAAGCTCTTAGAGGTGTTAAAGTTGAAGTTACGCACAGAGGAAATGTACGAAGG
AAATATCGGATTTCAGGACTGACATCACAACCTACAAGGGAACTAATTTTCCCAGTTGAT
GAGCAAATGAACATGAAATCAGTTGTTGAGTACTTTCAGGAGATGTATGGATTTACCATT
CGATATTCTCATCTACCTTGCCTCCAAGTAGGTAACCAGAGGAAAGTGAATTACTTACCA
ATGGAGGCTTGTAAGATTATTGGGGGACAGAGATATACCAAAGGGCTGACTGACAAGCAG
ATAACTTCCTTGTTAAAAGTAACATGCCAAAGATTACTCATTGCCATTCTTCCAGACAAC
AATGGCTCTTTGTATGGTGATTTGAAGCGGATTTGTGACACAGATCTGGGGTTGATTTCT
CAGTGCTGTCTTACTAAAAATGTCTACAAGATTAGCAACCAGTACCTGGCAAACGTGTCA
CTTAAAATCAATGTTAAGATGGGTGGAAGAAATACTGTGCTTTTAGATGCTTTGAGTTCA
GGAATTCCTTTGGTTAGTGACATTCCAACAATCATATTTGGAGCCGATGTAACTCATCCA
GAGACTGGAGATGACTCTTGTCCATCAATTGCTGCTGTAGTAGCCTCCCAAGACTGGCCA
GAAGTCACCAAGTATGCTGGATTGGTATGTGCTCAGGCTCATCGGCAAGAACTTATTCAA
GATTTGTATAAAACCTGGAAAGATCCTCAAGGGGGCACAGTTACTGGAGGCATGATCAGA
GAGCTTTTACTTTCATTTAAGGCAGCCACTGGAAAAAAACCATTGAGGATAATATTTTAC
AGGGATGGTGTCAGTGAAGGGCAGTTCTACCAGGTTCTACTATATGAACTTGATGCCATT
CGTAAGGCCTGCGCATCATTGGAACCTAGTTACCAACCTCCAGTGACATTTGTTGTCGTC
CAAAAACGGCACCACACTAGACTCTTTGCAAGCAACCACAATGACAAAAGCAGCACTGAT
AGGAGTGGGAACATCTTACCTGGTACTGTGGTCGATTCGAAGATCTGCCATCCCAGTGAG

TTTGACTTTTATCTATGCAGTCATGCAGGAATCCAGGGGACCAGCCGACCCGCTCATTAT
CATGTTCTCTGGGATGAGAACAACTTTACAGCAGATGAGATCCAATCTTTGACGAACAAC
CTCTGTTACACGTATGCAAGGTGCACTCGGTCTGTTTCTCTAGTTCCTCCTGCGTATTAT
GCTCATCTGGCAGCCTACAGAGCTCGATTCTACATGGAA---------------------

>Ptrichocarpa_POPTR_0010s17100
CTACCTGTGGGAAGATCACTGTATTCTAGTTCAATGGGAGGAACTAAAGAAATTGGAGGT
GGAGCGGTTGCATTAAGAGGGTTCTTTCAAAGTCTAAGGCCAACTCAACAAGGGCTAGCT
CTCAATGTGGATTTCTCTGTGACTGCTTTTCATGAAAGTCAAAAGCGTCTAGAATTTCTT
CGGGACCTTCCTCAAAGGAAAAAAAGAAGTTTGGTTGGTGAAGAAAGGAAAGAAGTGGAG
AAGGCCTTAAAGAACATCAGGATCTTTGTTTGTCACAGGGAAACTGTTCAGAGATACCGG
GTGTTTGGCCTAACTGAGGAAGCTACAGAAAATCTTTGGTTTTCTGACAGGGATGGAAAA
AATCTGAGGTTGCTGAATTACTTCAAGGATCACTACAACTACGATATACAATTCAGAAAC
TTACCATGCTTGCAGATTAGTAGGAGCAAACCATGTTATCTTCCTATGGAACTCTGTATG
ATTTGTGAAGGCCAGAAGTTTCTCGGGAAGCTATCTGATGATCAGACTGCAAGGATACTT
AAGATGGGCTGCCAAAGACAGCTGCTTATATGCGTAATGGAGAAGAAACACAAAGGGTAC
GCAGATTTGAAGCGAATAGCAGAGACAAGTGTTGGTGTCGTAACCCAATGCTGCTTGTAT
TTAAATCTTGGCAAGTTGAGCTCACAATTTCTGGCTAATTTGGCTCTCAAGATCAATGCC
AAAGTTGGTGGGTGCACAGTTGCTTTGTACAATTCGTTACCCTCTCAGATTCCTCGCCTC
CTTCGTTCCAATGAACCTGTGATCTTCATGGGAGCTGATGTGACTCATCCTCACCCGCTC
GACGATATCAGTCCATCTGTTGCTGCTGTGGTTGGTAGCATGAACTGGCCTGCAGCAAAC
AAGTATGTCTCAAGAATGAGGTCGCAAACACATCGACAAGAAATCATCCAAGACCTTGGT
GAAATGGTGAAAGAATTACTAGACGACTTTTACCAAGAATTGAATGAACTTCCCAAAAGA
ATTATATTCTTTAGGGACGGGGTAAGCGAAACCCAATTTTATAAGGTCCTTAAAGAGGAG
TTGCAAGCCATTAGAGAAGCTTGTTCTAGATTCCCTGGTTATAGACCTCCCATTACTTTT
GCAGTAGTCCAGAAGAGACATCACACAAGGTTGTTTCCGAATGAAACTGATCCATCTTCA
ACTCAGAACCAGTTTTCTGACGAAAATATACCCCCTGGGACTGTCGTGGACACTGTGATT
ACACATCCAAGGGAATTTGATTTCTATCTATGCAGCCATTGGGGAGTGAAAGGTACAAGC
AGGCCAACACATTACCATGTCCTGTGGGATGAGAACCAATTCACTTCTGATGAACTACAG
AAGTTGGTTTACAATCTGTGCTACACATTTGTAAGGTGCACCAAGCCAGTTTCTTTAGTG
CCTCCAGCTTACTATGCTCACTTGGCTGCATATAGAGGCAGACTTTACCTTGAG------

>contig9005_taeda
ACAGACCTTGGTATAGTTTCTCAATGCTGTTTAGTAAAGCATGTATTGAAGAGGAGCAAA
CAATACATGGCGAATGTAGCCTTAAAGATAAATGTTAAGGCCGGAGGCAGGAACACTGTA
CTTGTTGATGCTCTTAGAAGGAGAATATCTCTGGTGAGTGATGTACCAACAATAATATTT
GGTGCAGATGTAACTCATCCTCATCCTGGAGAAGACACTAGCCCATCCATAGCAGCCGTT
GTGGCTTCTCAAGATTGGCCAGAAGTCACAAAATATGTTGGTCTAGTTTCTGCTCAGGCA
CATCGTCAAGAGATGATTGAAGACCTTTTTAAGGTTGTAAAAGATCCTAAAAGAGGTGAT
GTCGTTCGAAGCATGATAAGAGATCTTTTGCGTTCCTTCTACACTGCAACAAGTCAGAAA
CCTATGAGGATCATCTTCTACAGAGATGGTGTAAGTGAGGGGCAGTTTTACCAAGTCCTG
CTGGATGAGTTAGAGGCCATACGCAGGGCTTGCATGTCAATTGACAAGGATTATCAGCCT
CCTGTGACTTTTATAGTTGTTCAAAAACGTCATCACACACGTTTATTTGCTCATAATCAT

CGTGACAGGAAGAGTGTTGATAAAAGAAGTGGTAACATATTGCCAGGTACCGTTGTTGAT
TCCACAATTTGTCATCCAACAGAGTTTGATTTCTACCTTTGCAGTCATGCTGGTATTCAG
GGAACAAGCAGGCCAGCTCATTATCATGTTCTGTGGGATGATAATAGATTCAGTGCAGAT
GCTTTGCAGTCTTTGACAAATAATCTCTGTTACACATATGCTCGGTGTACTCGCTCCGTT
TCAATCGTTCCTCCAGCATATTATGCGCATCTGGCTGCATTTCGAGCTCGCTTCTACATA
GAG---

>GRMZM2G079080_T02 cds:_protein_coding
GTGTCCATAGGGCGGTCCTTCTACTCGCCGGACATCAGGAAGCCGCAGCGGCTCGGCGAC
GGCCTGCAGTCGTGGCGTGGGTTCTACCAGAGCATCCGGCCGACCCAGATGGGATTGTCG
CTTAACATCGACATGTCGTCCACGGCATTTATTGAACCCCTGCCGGTGATCGAGTTCGTG
GCCCAGATATTAGGAAAGGATGTCATATCAAGGCCATTGTCCGATGCTAACCGAATCAAG
ATCAAGAAGGCATTGCGGGGTGTAAAAGTTGAGGTCACTCACCGGGGGAATGTACGGCGC
AAGTATCGCATTTCAGGCCTCACAACACAGCCAACTCATGAATTGATTTTCCCGATTGAT
GAACAAATGAATATGAAATCTGTCGTGGAATACTTCAAGGAAATGTATGGTTTCACCATT
CAGCATCCTCATCTTCCCTGCCTTCAGGTTGGAAACCAAAAGAAGGCAAACTATCTACCC
ATGGAGGCCTGCAAGATCATTGAAGGCCAGAGATACACAAAGAGGCTGAATGAAAAACAG
ATCACATCGCTGCTAAAGGTTACATGCCAAAGGCTTCTTTTGGCTATCCTCCCTGACAAC
AATGGGCAGTTATATGGTGACATCAAACGTATTTGTGAAACTGATTTGGGGTTGATATCA
CAATGTTGCTTAACCAAGCATGTTTTTAAGATCAGCAAGCAGTACTTGGCAAATGTCTCA
CTGAAAATTAATGTTAAGATGGGAGGAAGAAACACTGTGCTCCTGGACGCAATAAGTTGG
AGGATTCCGTTGGTCAGTGACATCCCAACTATTATATTTGGTGCAGATGTAACACATCCT
GAAACCGGGGAGGACTCAAGTCCATCGATTGCTGCCGTTGTTGCTTCTCAAGATTGGCCA
GAAGTTACAAAGTATGCTGGATTGGTTTGTGCTCAGGCACACCGGCAAGAGCTCATTCAA
GACCTTTACAAAACATGGCACGATCCTCAGAGAGGCACTGTAACAGGCGGCATGATCAGG
GAGCTCTTAATATCCTTCAGGAAGGCCACTGGACAGAAGCCATTGAGAATAATATTCTAC
AGGGACGGTGTTAGTGAAGGTCAGTTCTATCAAGTTCTCCTTTACGAGTTAGATGCCATC
CGGAAGGCATGTGCATCCCTAGAACCAAATTACCAGCCTCCTGTAACATTTGTGGTGGTT
CAAAAACGTCATCATACAAGACTATTTGCAAATAATCACAAAGACAGAAGTAGCATGGAC
AAGAGTGGAAATATTTTGCCAGGAACCGTTGTTGATTCTAAGATATGCCACCCAACGGAG
TTTGATTTCTACCTCTGTAGTCATGCTGGAATCCAGGGAACGAGTAGGCCTGCTCACTAC
CATGTCCTCTGGGATGAGAACAATTTCACAGCAGACGAAATGCAGACATTGACAAACAAC
CTTTGCTACACTTATGCCCGGTGCACACGCTCGGTTTCTGTTGTCCCTCCTGCATACTAC
GCACACTTGGCAGCATTCCGGGCTCGGTTCTACATGGAA---------------------

>Bdistachyon_Bradi2g10370
CTTTTAGTCCGCCAATCCTTTTTCCATAACAATCCATCACAATTTGTGGATCTGGGAGGC
GGTGTAATGGGCTGCAGGGGATTTCACTCAAGCTTCCGAGCCACAAAGAGCGGGCTTTCT
CTTAATATTGATGTATCTACAACAATGATTGTGAAACCTGGCGCTGTCGTTGATTTTCTA
ATTGCCAACCAGAAGGTAGATCACCCCAATAAAATTGATTGGGCAAAGGCTAAGCGTGCA
CTGAAGAATCTAAGGATAAAAACAAGTCCAGCAAACACAGAATTTAAGATTGTTGGACTG
AGTGAAAGGAACTGTTATGAACAAATGTTTTCACTGAAGCAAAGGAATaatGGGAATGGT
GACTCTGAAGCCATAGAAATATCTGTTTATGATTACTTTGTGAAGAATCGGGGCATAGAG
CTAAGATATTCTGGTGATTTCCCCTGTATCAATGTGGGGAAACCAAAGCGCCCAACATAT
TTTCCTATTGAGCTCTGCTCGCTTGTGCCTTTGCAAAGATATACCAAGTCTTTAAGTACT
CTCCAAAGGTCCTCTCTTGTCGAGAAGTCGAGGCAGAAGTTTTTGTTGTGTATCCTTGCT
GAGAGGAAGAATTCAGATGTTTATGGGCCTTGGAAGCGAAAATGTCTAGCTGAATTTGGG
ATTGTCACACAATGTGTGGCCCCAACTAGGGTCAATGACCAATATCTGACAAATGTCCTG
CTAAAAATAAATGCAAAGCTTGGTGGGATGAATTCTTTGCTGCAAATTGAACTGTCCCCT
GCAATCCCTCTTGTATCAAAGGTCCCAACTATGATTTTGGGCATGGATGTGTCCCATGGG
TCCCCTGGACAGGCTGATACACCATCTATTGCTGCAGTCGTTAGTTCTCGTGAATGGCCT
CTTGTCTCAAAGTACAGGGCCTCAGTGCGTTCTCAGTCACCAAAATCAGAAATGATTGAT
TCACTGTTTAAGCCACAAGGAACTGAAGACGATGGCCTTATTAGGGAGTGCCTAATAGAC
TTCTATACCAGTTCTGGAAAAAGAAAACCTGATCAGATTATAATCTTCAGGGATGGTGTT
AGTGAGAGCCAATTTACTCAGGTGCTAAACAAGGAGTTAGATCAAATAAATGAGGCATGC
AAATTCTTGGATGAAAGTTGGTCCCCCAAATTCACACTAATTGTTGCGCAGAAAAACCAC
CACACAAAGTTTTTCATCCCTGGATCACCGGATAATGTCCCTCCCGGCACTGTTGTTGAT
AACGTAGTTTGCCATCCAAAGAACTATGATTTCTACATGTGCGCACATGCAGGAATGATA
GGAACAACAAGGCCAACACATTACCATATCCTGCATGATGATATACACTTTACCGCAGAT
GATCTGCAGGATCTCGTGCACTCGCTCTCTTATGTGTACCAAAGAAGCACAACGGCCATA
TCAGTAGTCTCTCCAATCTGCTATGCCCATCTTGCTGCTGCCCAAGTGTCACAGTTCGTC
AAG---

>Sbicolor_Sb02g005150
GTCACTCTTTCTCGATCCTTCTTCTCCAAGAAGTTTGATAATGGGGTGGACATTGGTGGT
GGGCTAGAGAGCTGGAGCGGATATTACCAGAGCTTGCGCCCAACTCAAATGGGCCTCTCA
TTGAACATTGATATATGCTCGACTTCATTTTACCAATCTATCCCTGTGGTAAAATTTGTT
GATGATTGTCTTGGGCTGACAAACCCTGCCCAACCTTTTTCGGACAGGGATCGTTTGAAG
CTTAAGAAAGCCCTGCGTGGAGTTCGTGTTGAGACTACACACCAGCAGGGGAAAAAAAGC
GCCTACAAGATAACTGGGATTACTCCTGTTCCATTGGCTCAGCTGAGCTTTTCTTGTAAC
GAAGGACCTCAGTTGACTGTTGTCCAGTACTTTGCTGAACGGTACAACTACCGGTTGCGC
TATACTGCTTGGCCCTGCCTTCAGTCCGGCAATGATTCTAAGCCGATATATTTACCTATG
GAGGTGTGCCAAATCATTGAAGGACAGAGGTACCCTAGGAAGCTCAGCGACACACAGGTG
ACCAATATACTGAAGGCAACCTGTAAACGTCTGCTTATTGTCGTTCTTCCAGATGTTTCT
GGTCATTATGGGAAAATTAAGAGGATATGCGAGACTGACCTTGGTATAGTATCTCAGTGC
ATCAATCCAAAGAAGAATAAAAACAAGCAGTATTTTGAAAATGTCGCCCTTAAAATCAAT
GTGAAGGTGGGAGGGCGCAATACAGTGCTTGAGAGAGCCTTTGTGCCTAATGGAATACCT
TTTGTCTCAGATGTGCCAACAATCATTTTTGGTGCTGATGTTACCCATCCTACAGCAGGA
GAAGATTCCTCGGCTTCTATTGCAGCTGTGGTTGCATCCATGGACTGGCCACAAGTCACA
ACATATAAAGCACTAGTCTCGGCGCAAGCACATAGGGAAGAGATTATACAAAATCTGTTC
TGGACTGGTACAGATCCAGAGAAGGGCACTCCAGTGAACGGTGGAATGATAAGGGAGTTG
CTGACTTCATTCTTTAAGAGGACTGGACGAAAGCCCAAAAGGATTATATTTTACAGGGAT
GGTGTAAGTGAGGGACAATTCAGCCACGTTTTGCTCCATGAAATGGACGCAATCAGGAAG
GCCTGTGCCTCTATGGAAGATGGTTATCTACCACCAGTGACATTCGTGGTGGTACAGAAA

```
AGGCACCACACAAGGCTCTTCCCTGAAGTTCATGGAAGGAGAGATCTTACTGACAAAAGC
GGAAACATTCTTCCTGGAACTGTGGTTGATACTAGCATTTGTCATCCCAGCGAGTTTGAT
TTCTACCTCTGTAGCCATGCTGGAATTAAGGGAACAAGCAGGCCAACACACTATCATGTC
CTCTATGATGAGAACCGTTTCTCGGCTGATGCTCTGCAGTTTCTCACAAACAACCTTTGC
TACACATACGCACGCTGCACACGCGCTGTTTCTGTTGTTCCACCAGCCTACTACGCTCAC
CTGGCAGCATTCCGCGCAAGGTACTATGACGAA---------------------------
------------------------------------------------------------
------------------------------------------------------------
------------------------------------------------------------
------------------------------------------------------------
------------------------------------------------------------
------------------------------------------------------------
------------------------------------------------------------
------------------------------------------------------------
------------------------------------------------------------
------------------------------------------------------------
------------------------------------------------------------
------------------------------------------------------------
------------------------------------------------------------
------------------------------------------------------------
------------------------------------------------------------
------------------------------------------------------------
------------------------------------------------------------
------------------------------------------------------------
------------------------------------------------------------
------------------------------------------------------------
------------------------------------------------------------
------------------------------------------------------------
------------------------------------------------------------
------------------------------------------------------------
------------------------------------------------------------
------------------------------------------------------------
------------------------------------------------------------
------------------------------------------------------------
------------------------------------------------------------
------------------------------------------------------------
------------------------------------------------------------
------------------------------------------------------------
------------------------------------------------------------
------------------------------------------------------------
------------------------------------------------------------
------------------------------------------------------------
------------------------------------------------------------
------------------------------------------------------------
------------------------------------------------------------
------------------------------------------------------------
------------------------------------------------------------
------------------------------------------------------------
------------------------------------------------------------
------------------------------------------------------------
------------------------------------------------------------
------------------------------------------------------------
------------------------------------------------------------
------------------------------------------------------------
------------------------------------------------------------
------------------------------------------------------------
```


>Mesculenta_cassava1330.valid.m1
ATACCTGTGGGGAGATCATTCTATTCAAGTTCAATGGGAGGAACAAAGGAAATTGGAGGA
GGAGCCATTGGATTGAGAGGATTCTTTCAAAGTCTCAGACCCACCCAACAAGGATTAGCT
CTCAATGTGGATTTTTCAGTGACTGCTTTTCATGAAAGTCAAAAGCGTCTCAAGTTTCTT
AGGGACCTCCCACAGAACAAAACAAGGAGTTTGATTGGTGAAGAAAGGAAGGAAGTGGAG
AAGGCTTTGAAGAACATCAGGGTCTTTGTTTGCCACAGAGAAACAGTCCAGAGGTACCGA
GTTTTCGGCTTAACTGAAGAAGCTACGGACAATCTTTGGTTCGCGGACAGGGATGGGAAG
AATCTGAGGCTGGTGAGTTACTTCAAGGACCACTACAATTATGATATAAAATTCCGGAAC
TTGCCATGTTTGCAGATCAGTAGAAGTAAACCATGTTATCTTCCTATGGAGCTTTGTATG
ATATGTGAAGGCCAGAAGTTTCTTGGTAAGCTGTCAGATGATCAGACTGCAAAAATACTC
AAGATGGGCTGCCAAAGGCAGCTGCTTATTTGTATAATGGAGAAGAGACACAGAGGATAT
GCAGATTTGAAGCGGATAGCAGAGACAAATGTAGGTGTTGTAAGCCAGTGCTGCTTGTTT
ACAAATCTTGGCAAGTTGAGTTCACAATTTCTTTCCAATTTGTCTCTCAAGATCAATGCC
AAACTTGGAGGATGTACAGTTGCTTTGTACAATTCATTACCCTCTCAGATTCCCCGCCTT
CTTCATTCCGACGAGCCCGTGATCTTCATGGGAGCTGACGTCACTCATCCTCACCCTCTC
GATGACTTCAGTCCATCAGTTGCTGCTGTTGTTGGTAGCATGAACTGGCCAGCAGCAAAT
AAGTATGCATCTCGAATGAGGTCGCAAACGCATCGCCAAGAAATCATCCAGGATCTTGGT
TCAATGGTGAAAGAATTGCTAGATGATTTTTACAAAGAAGCAAACAAACTTCCCAAAAGG
ATAATGTTCTTCAGGGATGGAGTAAGTGAAACTCAGTTTCATAAGGTTCTCCAAGAAGAG
CTGAAATCAATTCGAGAAGCTTGTTCTAGATTCCCTTGTTATAAACCTCCCATTACCTTT
GCAGTTGTCCAAAAGAGGCATCATACAAGGTTGTTTCCCTGCGAAACAGATCTAtctTCT
TCTATTCAGGACCAGTTTTACAATGAAAATATCCCACCAGGGACAGTTGTGGACACTGTG
ATTACACATCCAAAGGAATTTGATTTTTATTTGTGCAGCCATTGGGGGGTGAAAGGAACA
AGCAGGCCAACACATTACCATGTGTTGTGGGATGAGAACCAATTCACTTCTGATGAATTA
CAGAAGTTGGTATACAATCTCTGCTACACATTTGTAAGATGCACCAAGCCAGTTTCTTTG
GTGCCTCCTGCTTACTACGCCCACTTAGCTGCATACAGGGGCAGGCTTTACATTGAA---

>Mesculenta_cassava2314.valid.m1
ATACCTGTAGGGAGATCATTCTATTCAAGTTTAATGGGCGGAACAAAGGAAATTGGAGGA
GGAGCTGTTGGATTGAGAGGATTCTTTCAAAGTCTTAGACCCACACAACAAGGACTAGCT
CTCAATGTAGATTTCTCCGTGACCGCTTTTCATGAAAGTCAAAAGCGTCTCAAGTTTCTG
AGGGACCTTCCTCAAAACAAAACAAGGAGTTTGATTATTGAAGAAAGGAAAGAAGTGGAG
ATAACTTTGAAGAATATCAGGGTCTTTGTTTGCCACAGAGAAACTGTTCAGAGATACCGG
GTTTATGGCTTGACTGAAGAAGCTACGGATAATATCTGGTTCGCCGACAGGGACGGGAAG
AAACTGAGGCTGGTGAGTTACTTCAAGGATCACTACAATTATGACATAAAATTCAGGAAC
TTGCCATGTTTGCAGATTAGTAGAAGTAAACCATGTTATCTTCCCATGGAGCTTTGTATG

ATTTGTGAAGGCCAGAAGTTTCTTGGGAAACTGTCCGATGATCAGACAGCAAAAATTCTC
AAGATGGGCTGCCAAAGACAGCTGCTTATTTGTATAATGGAGAAGAGACACAAAGGATAT
GCAGATTTGAAGCGAATAGCAGAAACAAATGTTGGTGTTGTAAGCCAGTGCTGCTTGTTT
CCGAACCTCGGCAAGTTGAATCCACAATTTCTAGCTAATTTGGCTCTCAAGATCAATGCT
AAAGTTGGAGGATGCACAGTCGCTTTGTACAATTCGTTACCCTCGCAGATTCCACGCCTA
TTTCATTCTGATGAACCTGTGATCTTCATGGGAGCTGATGTAACTCATCCTCACCCTCTT
GATGACTTCAGTCCATCAGTTGCTGCTGTTGTTGGTAGCATGAACTGGCCAGAAACAAAC
AAGTATGCATCACGAATGAGGTCGCAAACGCATCGACAAGAAATTATTCAGGACCTTGGT
GCAATGGTGAAAGAATTGCTAGATGAATTTTATCAAGAAGCAAGAAAACTTCCCAAGAGG
ATAATATTCTTCAGGGATGGAGTAAGCGAAACCCAGTTCTACAAGGTGCTTCAAGAGGAG
TTGCGGGCGATTCAAGAAGCATGTTCGAGATACCCAGGTTATAGACCTCTCATTACATTT
GCAGTTGTCCAAAAGAGACACCATACAAGGTTGTTTCACTGTGAAACAGATGAATCTTCC
ATTCAAAACCAGTTTTACGGCGAAAACATACCCCCAGGAACAGTTGTGGATACTGTTATT
ACTCATCCAAAGGAATTTGATTTTTATCTATGCAGCCATTGGGGAGTAAAAGGAACAAGT
AGGCCAACTCATTACCATGTCTTGTGGGATGAGAACCAATTCACTTCTGATGAATTACAG
AAGCTAGTTTACAATCTCTGCTACACATTTGTAAGGTGCACAAAGCCAATTTCTTTGGTG
CCTCCTGCTTACTATGCACACTTAGCTGCATACAGGGGCAGGCTTTACCTCGAA------

>GRMZM2G441583_T01 cds:_protein_coding
ATGGTGAGGAAGAAAAGGACTGGCCCTGGAGAGAGTTCTGGGGAGACTTCTGGAGCGCCT
GGTCAGGGCTCCTCACAGCGTCCTCAGGCAACACAACAGGGTGCCCGAGGTGGAGGACAA
CACCAGGTGCGTGGTGGATATCCGGGCCATGGAGTGCCGCCTTCAGAGCACCCAGGTGGT
GGGCCGCCTGAGTATCAACCACGTGGCTATCAGGGACGTGGCGTTCCACCTTTACTTCCT
GGTGGTGGGCCGCCTGAGCCTCAACCGCGTGGCTACCAGGGACATGGTGGATACGGCTAC
CAGGGACATGGTGGATACCAGGGCCGTGGCGGGCCACCTTCACAGCATCCTGGTGGTGGG
CCATCACCTGGGTCTCAGCCTCGTGGCTACCAGGGACGTGGTGGTCTGCGTCCCAGAGGG
GGAGTGCCGCAGCCATACCGTGGCGGGCATGTGGGAGGTAGTGTTGGACCAATTGTTCCT
TCAGGTCCGTCTAGACCAGTTCCCGAGCTGCACCAAGCCCCAGATGTCCAACATCAAGCC
CCTGTGGTGGCAGCACCATCACCACCGGGAGCTGGCTCGTCCTCGCAGCCTGGGATGGCC
GAGGTGAGCTGTCCTGTTGGTAGATCATTTTATTCTCCCAATTTAGGGAGACGCCAGCAA
CTTGGTGAAGGTTTGGAAACTTGGCGTGGTTTCTATCAAAGCATAAGGCCCACACAGATG
GGTCTTTCTCTGAATATTGATATGTCCTCTACTGCATTTATTGAGCCCCTCCCTGTGATT
GATTTTGTTGCTCAGCTTCTTGACAGAGATATCTCAGTTAGACCATTGTCTGATTCTGAT
CGTGTGAAGATTAAAAAAGCCCTACGAGGTGTGAAAGTCGAGGTCACACACCGTGGAAAC
ATGCGTAGGAAGTATCGGATATCTGGCCTCACTTCACAAGCAACAAGGGAGTTATCATTC
CCTATTGATGATCGTGGTACTGTTAAGACTGTGGTGCAATACTTCCTGGAGACTTATGGC
TTTAATATTCAGCACACCACTTTACCTTGTTTGCAAGTGGGCAATCAGCAAAGAATAAAT
TATCTGCCTATGGAGGTCTGTAAGATAGTTGAGGGACAGCGTTACTCAAAACGACTCAAT
GAGAAACAGATCACTGCTCTACTGAAGGTGACTTGCCAGCGTTTGCTGATGGTAATACTG
CCTGACAATAATGGCTCTCTTTACGGGGATCTTAAAAGGATATGTGAGACTGATCTTGGA
TTGGTCTCCCAATGCTGTCTGACTAAACATGTTTTCAAGGCGAACAAGCATCAGTATCTT
GCAAATGTTGCCCTGAAAATAAATGTTAAGGTTGGGGGAAGGAATACTGTACTTGTTGAT
GCTTTGGCAAGGAGAATCCCCCTTGTCAGTGACGTAGCTACTATTATCTTTGGTGCTGAT
GTGACCCATCCCCATCCTGGGGAAGATTCTAGTCCTTCCATTGCAGCTGTGGTTGCTTCT
CAAGACTGGCCTGAGGTTACGAAGTATGCAGGATTAGTGAGTGCTCAAACCCATCGCCAA
GAATTGATACAGGATCTTTTCAACGTACGGCAAGATCCCCAAAGGGGGGCTGTCTCTGGT
GGCATGATTAGGGAACTTCTCATTTCTTTCTGGAGGGCGACTGGACAAAAACCCAAGAGG
ATCATATTCTACAGGGATGGTGTCAGTGAGGGACAGTTCTACCAAGTTCTGTTGTACGAA
CTTGATGCCATTAGAAAGGCCTGTGCATCATTGGAGTCTGATTACCAGCCTCCAGTTACC

TTTGTCGTGGTCCAGAAGCGTCATCACACCAGGTTGTTTGTTAATAATCACAATGATCAG
CGTGCTGCCGATAGAAGTGGCAACATACTGCCGGGCACTGTGGTGGACTCGAAGATTTGC
CATCCAACCGAGTTTGATTTCTACCTGTGCAGCCATGCTGGCATTCAGGGAACAAGCCGT
CCTGCCCATTATCATGTTCTGTGGGATGAGAACAAATTTACGGCTGATGGGTTGCAAACT
CTCACCAACAACTTGTGTTACACGTATGCCAGGTGCACACGCTCAGTATCAATCGTTCCT
CCTGCATACTATGCTCATCTGGCAGCCTTCCGAGCTCGGTTTTACATGGAG---------

>Gmax_Glyma06g23920
GTGTCCATTGGGAGGTTTCTGTATTCTCCTGATGTAAGAAAACCACAGCAGTTGGGTGGT
GGTCTTGAATCATGGAGGGGCTTCTATCAGAGTATAAGGCCTACTCAGATGGGATTGTCA
CTTAACATTGATATGTCATCAATGGCATTTATCGAACCACTACCTGTGATTGACTTTGTT
GCTCAAATTTTGGGAAAAGATGTTCACTCAAAGCCGTTGTTGGATGCAGATCGTGTCAAG
ATCAAAAAGGCCCTAAGAGGTGTAAAAGTTGAAGTTACACATAGAGGAAATTTTCGAAGG
AAGTATAGAATATCAGGACTGACTTCACAGCCTACAAGGGAGCTTATTTTCCCTCTTGAT
GACCAAATGAACATGAAATCAGTAGTTGATTATTTTCAAGAAATGTATGGATTTACAATC
AAATATTCTCATCTACCTTGCCTTCAAGTAGGAAGTCAAAGGAAGGTGAACTATTTGCCA
ATGGAGGCATGCAAGATAGTTGGGGGCCAGAGATATACAAAAGGGCTAAATGAAAAGCAG
ATAACTTCTCTGCTGAAGGTCTCATGTCAAAGGTTGCTGATTGCCCTTCTTCCAGACAAT
AATGGCTCTTTGTATGGTGATCTCAAAAGAATCTGCGAAACAGATCTGGGGTTGATTTCT
CAATGCTGCCTTACAAAACATGTATTCAAGATCAATAGACAGTACCTGGCTAACGTGGCA
CTAAAAATCAATGTCAAGATGGGAGGAAGAAACACAGTACTTTTGGATGCTTTAAGTTGG
AGGATTCCACTGGTTAGTGATATTCCAACAATAATATTTGGAGCTGATGTAACTCATCCA
GAATCAGGAGAGGATTCTTGTCCATCCATTGCTGCTGTTGTAGCCTCCCAGGACTGGCCT
GAAGTAACAAAGTATGCAGGGTTGGTTTGTGCTCAGCCTCATCGAGAAGAACTAATTCAA
GATCTTTTCCGATGTTGGAAAGATCCACAGAGGGGTGTAATGTACGGTGGTATGATCAGG
GAGCTTTTACTATCATTTAAGAAGGCAACTGGACAAAAACCATTGAGAATAATCTTTTAC
AGGGATGGGGTAAGTGAGGGACAGTTCTACCAGGTTTTGCTATATGAGCTTGATGCCATA
CGCAAGGCTTGTGCATCTTTGGAACCTAGTTACCAACCTCCAGTAACATTTGTTATTGTT
CAAAAACGGCATCACACTAGACTCTTCGCAAACAATCATGATGACAGAAATAGCACTGAT
AAGAGTGGGAATATCTTACCTGGTACTGTGGTAGATTCTAAGATCTGTCATCCTTCGGAA
TTCGACTTCTACTTGTGCAGTCATGCAGGAATTCAGGGTACAAGTAGACCAGCACACTAT
CACGTCCTCTGGGACGAGAACAATTTCACTGCAGATGAGATTCAGTCTCTAACCAACAAT
TTGTGCTACACTTATGCAAGATGTACGCGGTCTGTTTCAGTAGTGCCTCCAGCATACTAT
GCTCATCTAGCAGCCTACAGGGCTCGATTCTACATGGAA---------------------

>CL5705_sinensis
ACAGTTGTGGGAAGGTCATTTTTCTCAACTGATCTTGGACCAGTGGGTCAGCTTGGTGAT
GGCGTTGAATATTGGCGTGGATATTTTCAGAGTCTTCGCCCAACCCAGATGGGGCTATCT
CTTAATATTGATGTTTCAGCCCGCTCCTTTTATGAGCCAGAGCCAGTTACTGAGTTTGTT
CAGTATTATTGCAGAGACTTATCACGTCCTCTTTCTGATCAAGTGCGTCTGAAGGTGAAA
AAGGCATTGAAGGGAATAAAGGTAGTGCTCACGCATATGGAGTATAACAAAAGTTACAAG
ATCACTGGAATATCCAGTGAACCGATGAGCCGACTAATGTTTACTGATGACAATGCGACA
AGGTTGTCAGTGGTTCAATATTTTCGTCAAAGATATAATATTGGGCTTCAATTCACGTCA
TTGCCTGCTCTTGTAGCTGGAAGTGAAGCGAGGCCTATTTATTTGCCAATGGAGCTTTCT
AGGATTGCCGCGGGACAGAGATATACTAAAAGATTAAATGAAAGGACAGTAACTGCTCTT
TTAGAGCACCTGTCAGCG--

>Vvinifera_GSVIVT00001880001
TTGCCTGTGGGGAGATCCCTGTACTCGAGCTCAATGGGAGGGACTAAAGATATTGGAGGA
GGAGCGGTTGGATTAAGAGGGTTCTTTCAGAGTCTTAGACCAACCCAACAAGGACTTGCT
CTCAATGTGGATTTTTCAGTGACTGCTTTCCATGAGAGCCAGAAGCGAGTTGAGTTTCTT
CGAGACCTTTCCCAAAGGAAGACGAGAGGTTTGACGGGCGAAGAAAGGAAAGAAGTGGAG
AAGGCATTGAAGAATATTAGGGTCTTTGTACGTCATAGAGCGACTGTTCAGAGATATCGG
GTGCATAGCTTAACTGAGGAAACTACAGAAAATCTCTGGTTTGAAGACAGGGATGGGAAG
ATTCTGAGGCTGGTGAATTACTTCAAGGATCACTATGGCTACGATATACAGTTCAGGAAC
TTGCCATGTTTGCAGATTAGTAGGAGCAAACCATGCTATCTTCCTATGGAGCTTTGTATG
ATCTGTGAAGGCCAAAAATTTCTTGGGAAGCTCTCTGATGATCAAACTGCAAGAATACTT
AAAATGGGCTGCCAAAGACAGCTCCTTATATGCATAATGGAGAGAAAGCACAAAGGGTAT
GCAGATTTGAAGCGAATTGCTGAGACCAGCATTGGGGTTGTAAGTCAGTGCTGCTTGTAC
CAAAATCTTGGCAAGTCGAGTTCACAGTTTCTGGCAAACCTGGCTCTCAAGATCAATGCC
AAAATGGGAGGATGCACTGTTGCCTTGTACAATTCACTTCCTTCCCAGATACCACGGCTG
CTTCGGCCTGATGAGCCAGTCATCTTTATGGGTGCTGATGTGACTCATCCTCATCCTCTT
GATGATTTCAGCCCCTCCATTGCTGCTGTGGTTGGGAGCATGAACTGGCCAGCAGCTAAC
AAGTATGTTTCAAGAATGAGGTCCCAGACCCATCGTCAAGAAATCATCCAGGATCTTGGT
GCCATGGTTGGAGAAATACTGGATGATTTTTATCAGCAAGTATCCAAACTCCCCAAGAGG
ATCATTTTTTTCAGGGATGGAGTGAGCGAAACCCAGTTCTATAAGGTGCTCCAAGAGGAG
TTGCAAGCTATAAGAGTGGCTTGTTGTAGATTTCCCAATTACAGACCTCCCATTACTTTT
GCAGTGGTGCAGAAGAGGCACCACACGAGGTTGTTTCGGAATGAAAGCAACcacCCATCT
TCCACAGGAAACCAGTTGTTGGAGGAGAACATTCCCCCAGGGACAGTTGTGGATGCTGTG
ATTACACACCCAAGGGAATTTGATTTCTATCTTTGTAGCCATTGGGGGGTGAAGGGAACA
AGCCGACCAACCCACTACCATATATTGTGGGATGAAAACCATTTCACTTCTGATGAAGTA
CAGAAGCTGGTTTACAGTCTATGCTACACATTTGTGAGATGTACAAAGCCTGTGTCGTTG
GTCCCACCAGCTTACTATGCCCACCTGGCTGCATACAGAGGCAGACTCTACCTGGAG---

>Mesculenta_cassava35367.valid.m1
TGTCCCATAGGAAGATCCTTCTTTTCACCAGATATTAGAGCACCACAACGGCTTGGTGAT
GGCTTGGAGTCTTGGTGTGGGTTTTACCAGAGTATAAGGCCTACACAAATGGGCCTCTCT
TTAAATATTGATATGGCTTCTGCTGCATTCATCGAGCCTCTCCCTGTAATTGAGTTTGTT
GCCCAGCTTCTAGGCAAGGATGTGTTATCCAGACCATTGTCTGATTCTGATAGAATCAAG
ATTAAAAAGGCCCTCAGGGGAGTGAAAGTTGAAGTAACTCATAGAGGACATGTACGGAGA
AAGTATCGTGTCTCAGGATTGACATCTCAACCTACAAGAGAACTTGTATTTCCTGTTGAT

GATAACTCCACTATGAAGTCAGTAGTTGAGTACTTCCAAGAGATGTATGGCTTCACCATT
CAGCATTCTCATCTCCCTTGCCTTCAAGTAGGAAACCAGAAGAAGGCAAACTATCTCCCG
ATGGAGGCATGCAAAATTGTAGAAGGTCAACGATACACTAAAAGGTTGAATGAGAGACAA
ATTACTGCACTGTTGAAAGTTACATGCCAAAGACTTTTATTAGCTATTCTGCCCGACAAC
AACGGATCCCTTTATGGTGACCTTAAGCGGATATGTGAAACTGATCTTGGTTTAATATCA
CAATGCTGTCTTACAAAACATGTCTTCAAGATCAGCAAGCAGTATTTGGCTAATGTGTCA
CTGAAGATTAATGTTAAGATGGGTGGTAGAAACACTGTCCTTTTAGACGCCATTAGCTGC
AGGATACCATTAGTTAGTGACATACCAACCATTATATTTGGAGCAGATGTGACTCACCCA
GAGAATGGGGAGGACTCAAGCCCTTCAATTGCAGCAGTAGTAGCTTCTCAAGACTGGCCT
GAAGTGACAAAATATGCTGGATTAGTTTGTGCTCAAGCTCACAGACAAGAACTCATACAA
GACTTGTATAAAACATGGCAAGATCCAGTTCGTGGAACTGTTAGTGGGGGCATGATCAGA
GATCTTCTGGTTTCCTTTAGGAAGGCAACAGGACAGAAGCCGCTGAGGATTATATTCTAC
AGGGATGGTGTTAGCGAAGGGCAATTCTATCAAGTTCTGCTTTATGAATTAGATGCAATC
CGGAAGGCTTGTGCCTCTTTAGAACCAAACTATCAACCACCTGTGACTTTCATAGTTGTA
CAGAAACGGCATCATACTCGATTATTTGCTAACAACCACAGGGATAGGAGTAGCACGGAC
AAAAGTGGGAACATATTACCTGGTACTGTGGTTGACTCTAAAATCTGTCACCCTACAGAA
TTTGATTTTTATCTCTGTAGCCATGCTGGTATTCAGGGAACAAGTCGGCCTGCTCACTAC
CATGTTTTGTGGGATGAAAACAACTTCACTGCTGATGGAATCCAGTCATTGACAAACAAT
CTATGCTATACATATGCAAGATGCACTCGCTCTGTTTCTGTCGTTCCTCCAGCATATTAT
GCACATTTGGCTGCTTTTCGCGCTCGATTTTACATGGAG--------------------

>GRMZM2G039455_T01 cds:_protein_coding
ATGGTGAGGAAGAAGAGAACTGGCCCTGGCGGCTCTGGAGAAACTTCTGGAGAGTCTTCA
GGAGCTTCTGGACAAGGTTCCTCACAGCGGCCTGAACGGACTCAACAACCTGGGGCAGGA
CGTGGCTGGGTGCCTCAGCAGGGTGGCCGTGGTGGCGGGCAACACCAGGGTCGTGGTGGA
CATTATCAAGGCCGTGGAGGGCCAGGTCCACATCACCCTGGTGGACTGCCTGAGTATCAC
CAGcgtgaataccagGGACGAGGTGGTGAGTACCAGGGACAGTACCAGGGGCGTGGTGGT
GCCCGCTCCAGAGGTGGAATTTCACAGCCATACTATGGTGGGCATAGGGGAGGTAGTGTT
GGACGAAATGTTCCTCCAGGTCCATCCAGAACAGTTCCCGAGCTGCACCAAGCCCCATAC
GTCCAGTATCAAGCCCCGGTGATTTCACCATCCCCATCGGGACCTGGCTCATCCTCACAG
CCTATGGCAGAGGTGAGCTCTCCTGTTGGTAGGTCATTTTATTCTCCCAACTTAGGGAGA
CGTCAGCAACTTGGTGAGGGTTTGGAAAGTTGGCGCGGTTTTTACCAAAGCATAAGGCCG
ACACAGATGGGCCTTTCACTGAATATTGATATGTCCTCTACTGCATTTATCGAGCCTCTC
CCTGTGATTGATTTTGTTGCTCAGCTTCTTAATAGAGATATTTCAGTTAGGCCATTGTCT
GATTCTGATCGCGTGAAGATCAAAAAAGCCTTAAGAGGTGTGAAGGTTGAGGTCACTCAC
AGGGGAAACATGCGCAGAAAGTATCGCATTTCTGGCCTCACCTCACAAGCAACAAGAGAG
CTATCATTCCCTGTTGATGATCGTGGTACTGTGAAGACTGTGGTCCAATACTTCATGGAG
ACTTATGGTTTTAGCATCCAGCACACCACTTTACCGTGCTTGCAAGTGGGCAATCAACAA
AGACCAAATTATCTGCCTATGGAGGTTTGCAAGATAGTTGAAGGACAGCGTTACTCAAAG
CGACTCAATGAGAAACAAATCACTGCTTTACTGAAAGTGACCTGCCAGCGCCTGCTGATT
GTAATACTGCCTGACATTAATGGTTCCTTATATGGGGATCTCAAAAGGATCTGTGAGACT
GATCTCGGATTGGTCTCCCAGTGTTGTCTGACTAAACATGTTTTTAAGATGAGCAAGCAG
TATCTTGCAAATGTTGCACTCAAAATAAATGTTAAGGTTGGTGGAAGGAATACTGTACTT
GTAGATGCTTTGACAAGGAGAATCCCCCTTGTCAGTGACAGACCGACCATAATATTTGGT
GCTGATGTTACCCATCCACATCCTGGAGAAGATTCCAGTCCTTCCATTGCAGCTGTGGTT
GCTTCGCAAGACTGGCCTGAGGTCACCAAATATGCTGGACTAGTGAGTGCCCAAGCCCAT
CGCCAGGAGCTGATACAGGATCTTTTCAAAGTATGGCAAGATCCACAGAGAAGGACAGTA
ACTGGTGGCATGATAAAGGAACTTCTCATTTCTTTCAAGAGAGCAACTGGACAGAAGCCC
CAGAGGATCATATTCTACAGGGATGGTGTCAGTGAGGGACAGTTCTATCAAGTATTGTTG
TATGAACTTGATGCCATCAGAAAGGCATGTGCATCCTTGGAGCCCAACTACCAGCCTCCA

GTTACTTTTGTCGTGGTGCAGAAACGACATCACACTAGGCTGTTTGCTAATAACCACAAC
GATCAGCGTACAGTTGATAGAAGCGGAAACATACTGCCTGGCACCGTGGTTGATTCGAAG
ATTTGCCATCCTACTGAATTTGATTTCTACCTGTGTAGCCATGCTGGCATTCAGGGAACA
AGCCGCCCTGCTCATTACCATGTCCTGTGGGACGAGAACAAGTTCACAGCTGATGAGCTG
CAGACTCTGACAAACAACCTATGCTACACGTACGCTAGGTGCACCCGCTCCGTGTCAATT
GTGCCCCCGGCATACTATGCTCATCTGGCAGCCTTCCGAGCTCGCTTCTACATGGAG---

>Vvinifera_GSVIVT00000886001
CTTCCCACTGCCAGCAGTGGGGAGAGCTCGGAGTCCACTCAGGAAGCTAGTGGAGCTAGT
GGAGGTAGTGGAGGAGGCCGAGGTTCTCAGCAGCCTGCAGCAACACCACAACAAGGAGCT
ACTGGGATGGGATTTCAAGGGGGTAGAGGTTGGGCTCCTCAATCACAACAAAGCGGGCGC
GGTGGGTATGCAGGTGGCCGGGGTcctcagcgtGGTGGAATGGCTCCACAACAGCAATAC
ACTGTCCCGACTGAATATCAAGGTCGGGGTCGTGGAGGAGGAGCACCACCTCAGCAAcct
cctgcagcagctgctgcttatgagtctggaagcagaagccgagctCGTGTGGGTGGTGGC
CGTGGGGTAGAGCCAGTGTCTTCTGGTGGCCCTCCTTCTAAGCCGTTAAGTTCCGATCTG
CACCAAGCTACGCAGGCGAGTTATGCAGCCGGGGGAACTCCTCATCGCGTTCCCTCTGAA
GCCAGTTCATCACGTCAGGCTGCTGAGTCTTTGACATGTCCCGTGGGGCGATCATTTTAT
TCTCCTGATCTAGGGAGAAGGCAGCCATTGGGTGAGGGTTTGGAAAGTTGGCGTGGTTTC
TATCAGAGTATTCGGCCAACTCAGATGGGACTGTCACTGAATATTGATATGTCCTCGACT
GCTTTTATTGAGCCATTGCCTGTCATTGATTTTGTGACACAGCTTCTGAATCGGGATGTT
TCTTCCCGACCATTATCTGATGCTGATCGTGTAAAGATTAAGAAGGCTCTCAGAGGAGTC
AAAGTTGAAGTTACTCATCGGGGAAATATGCGCAGAAAATATCGTATATCTGGCTTAACA
TCACAAGCAACACGTGAATTGACTTTTCCTGTTGATGATAGAGGAACCATGAAATCTGTC
GTTGAGTACTTCTATGAAACATATGGGTTTGTCATTCAGCATTCTCAATGGCCTTGTCTG
CAAGTGGGAAATCAACAGAGGCCAAATTATTTGCCTATGGAGGTTTGCAAGATTGTTGAA
GGCCAAAGATATTCCAAGAGGTTGAATGAGAGGCAGATTACTGCTTTACTGAAGGTGACT
TGTCAGCGTTTGCTTATTGTTATTTTGCCAGATAATAATGGTTCACTCTATGGTGATTTG
AAACGAATCTGTGAGACAGATCTTGGGCTTGTTTCACAGTGTTGCTTACATAAACATGTG
TATAGAATGAGTAAGCAATATCTGGCAAATGTGGCATTGAAGATTAATGTAAAGGTTGGA
GGGAGAAATACGGTGCTTGTTGATGCAATATCAAGGCGCATACCTCTGGTCAGTGATCGG
CCTACTATCATTTTTGGGGCTGATGTTACCCATCCTCACCCAGGAGAGGATTCAAGCCCA
TCTATTGCTGCTGTTGTCGCCTCTCAAGATTGGCCCGAGATTACAAAATATGCTGGTTTG
GTTTGTGCTCAGGCCCATCGACAGGAGCTCATCCAAGATCTTTATAAGACTTGGCAGGAT
CCTGTAAGAGGAACTGTGTCTGGTGGAATGATCAAAGAACTGCTTATATCTTTCCGTAGA
GCAACTGGGCAGAAACCTCAGCGCATAATATTCTATAGGGATGGGGTCAGTGAAGGGCAG
TTCTATCAAGTCTTACTATATGAACTTGATGCTATTCGTAAGGCATGTGCATCTTTGGAG
CCAAACTATCAGCCTCCTGTGACATTTGTTGTGGTTCAGAAACGCCATCATACTAGATTG
TTTGCGAACAACCATAATGACCGGAATGCAGTTGACAAGAGTGGGAATATATTACCTGGT
ACTGTTGTAGATTCTAAGATCTGCCATCCTACTGAGTTTGACTTTTACCTGTGCAGCCAT
GCTGGCATTCAGGGCACAAGCCGACCTGCCCATTACCATGTGTTGTGGGATGAAAACAAG
TTCACAGCTGATGGGCTGCAGTCCCTCACAAACAACCTCTGCTACACATATGCTAGGTGC
ACACGCTCTGTTTCCATCGTGCCCCCTGCATACTATGCTCATCTGGCTGCTTTCCGAGCA
CGCTTCTATATGGAG--

>AT5G43810.1
TGTCCCGTTGGAAGATCTTTCTTTTCGCCTGATATTAAAACACCGCAGCGACTCGGTGAA
GGGTTAGAGTCATGGTGTGGGTTTTACCAGAGTATTAGACCAACTCAAATGGGTTTATCA
CTAAATATCGATATGGCTTCAGCTGCATTCATCGAGCCTCTTCCAGTGATAGAGTTTGTA
GCACAGCTTCTTGGAAAGGATGTCTTGTCGAAGCCATTGTCGGATTCTGATCGCGTCAAG
ATTAAGAAGGGTCTTAGAGGAGTGAAAGTAGAGGTTACTCACAGAGCGAATGTAAGAAGG
AAATACCGTGTTGCGGGTTTAACAACTCAACCAACAAGAGAGCTAATGTTTCCAGTAGAT
GAGAACTGTACTATGAAGTCAGTTATTGAGTATTTCCAAGAGATGTATGGATTCACGATC
CAGCACACGCATTTGCCATGTCTCCAAGTTGGAAACCAAAAGAAGGCAAGCTATTTGCCG
ATGGAGGCATGCAAAATTGTCGAGGGACAACGGTACACGAAAAGGTTGAATGAGAAGCAG
ATTACTGCTCTCTTGAAAGTTACATGCCAAAGGCTTCTGCTGGCAATATTACCTGATAAC
AACGGTTCACTTTATGGTGATCTTAAGAGAATCTGTGAAACCGAGCTTGGTTTGATATCT
CAATGTTGTCTCACAAAACATGTGTTCAAGATTAGCAAACAGTATCTGGCAAATGTATCC
CTTAAAATCAACGTAAAGATGGGAGGAAGGAACACAGTTCTAGTAGACGCCATAAGCTGT
AGAATACCACTGGTTAGCGATATACCGACAATCATTTTTGGCGCAGACGTGACTCACCCA
GAGAACGGGGAAGAGTCAAGCCCTTCAATCGCTGCTGTTGTTGCTTCTCAAGACTGGCCT
GAAGTGACAAAATATGCGGGTTTAGTTTGTGCTCAAGCTCACAGGCAAGAACTTATACAA

GATTTGTATAAAACATGGCAAGATCCTGTTCGCGGTACTGTTAGTGGCGGTATGATCAGG
GACCTTCTTATCTCATTTAGAAAAGCAACAGGGCAAAAACCGCTTCGAATTATCTTTTAT
CGTGATGGAGTAAGCGAAGGGCAATTCTATCAAGTTTTACTCTATGAGTTGGATGCAATT
CGAAAGGCTTGTGCATCGCTTGAACCGAATTATCAGCCACCGGTGACATTCATAGTTGTA
CAGAAGCGTCACCACACTCGTTTGTTTGCTAATAATCACCGAGACAAAAACAGTACTGAC
CGAAGCGGAAATATCTTACCAGGTACTGTAGTTGACACTAAAATATGTCATCCAACTGAA
TTCGACTTCTACCTTTGTAGCCATGCGGGTATTCAGGGAACAAGCAGGCCTGCACATTAC
CATGTTCTTTGGGACGAGAACAATTTCACAGCAGATGGTATTCAATCTCTGACTAACAAT
CTCTGTTATACCTATGCGCGGTGCACTCGGTCGGTCTCTATAGTTCCTCCAGCGTATTAT
GCTCATCTTGCAGCATTTCGAGCACGTTTCTACCTGGAA--------------------

>Smoellindorffii_15415029_locus
GTTTTACTGCGTGAGAGCTACTTTCATCAGTCCCTTGGTCCAGTAAAAGATGTTGGAGAA
GGGGTGGAAAGCTGGAGTGGCTACCACGCTAGCTTCCGACCTTGTTCTCTGGGATTATCA
CTGAACTTGGCAGATCCGTCGACAACAATCGTCATCAAGCCCgaaTTTCTTGCGGAGTAT
TTCGATacatctccaGGAGGCATTCGTGCCGATCACTTGACCCGGGCGAAGAGAGTGCTG
AAGGGAATCGTGGTGCAAGTTTACACTAAGACGAGGCACAAAATATTCGGTTTTAGCGAT
GAACCAGCATCCTCTCAAAGGTTTGAATTGAAAGAGAAAGGAGCCGATGGCACGTTTAGA
CTGAATTCAACGACAGTTCTTCAGTACTATCAAGCTAGATACAACGAAACCTTGCAATTC
CCCAATTTGCACTGTGTCAACGTAGGGAAAGCCACGCGAGCAGTTTATGTACCGATGGAG
TTCTGTTCGATTCTGCCTGGACAACGGTATAAAAGAAAACTGAGCGGGAATCAGATTCGC
AGGCACCTTGACCAAGCACGACTGTTCGTTCTCTGTCTTCTTCCGAGCAAGGAATCGGAT
GCCTACGCGCCATTCAAACGACTCTTTTTGACGAAAGAAGGAATTCCGAACCAGTGCATA
GCGCCCCAGAGGAATCCCAACAATCAATACCTGACGAACGTCGTTTTGAAGATGAATGCA
AAGTTAGGGGGCTACAACACGGTTTTAACTAGTGAATTTAAGAAAGAATTGCCAAAGCTT
TCATATGCTCAAACCATGATCTTGGGAATGGACGTGTCGCATGGATCTCCATTTTCCCAC
ACCCCGTCGGTCGCGGCTATGGTTGGCTCTTTTGACTGGCCAAGGATTACGAGGTACTCC
GCTCGCGTGATGGCGCAATCGGCAAAACAGGAAGCGTTCGCCAACATTCCATCGATGCTG
GAGAGCCTCCTGAAGAACTTTaagaacTTCCAGGGCGAGAAGGGTTGCTACCCTCAGCAA
TTGATTGTGTTCAGAGATGGAGTGAGTGAATCCCAGTTTGAGAGCGTCCTCACCGGGGAA
CTGCAAGACATCATCAAGACTTGCGAAGGTCTCGGCATCAGGCCGAAGATAACTCTGGTT
GTAGCACAGAAAAGGCACCATACGCGTTTCTTGCCGGTGGGGCAGCAGAAAAAGAATGTC
GAACCTGGGACCGTTGTGGACCGCGACGTCGCACATCCTACGAATTTCGACTTCTTCCTG
TGCAGTCACTTCGGCATGCTCGGAACAAGCAGGCCGACACACTACATCGTGCTCTACGAC
GAGATTGGTTTCACGCCGGACGAGATCCAAATGACCATCAACAATCTGTGCTACACCTAC
GTCAAGAGCACGACAGCAGTATCAGTGGTTGCGCCGATCAACTACGCGCATCTGGCCGCG
AAGAAAATGAAGAACTTTATGTCG------------------------------------

>Gmax_Glyma09g29720
ATGGTCAGAAAGAGAAGAACTGAACTACCCAGTGGGGGTGAAAGCTCTGAGGCTCAACGC
CCTGCTGAAAGGAGTGCACCACCCCAACAACAGGCTGCTGCTGCTGCCCCAGGAGGGGCT
GGACCCCAAGGAGGCAGAGGTTGGGGTCCCCAAGGAGGACGAGGAGGCTATGGTGGGGGC
CGCAGTCGTGGGATGCCCCAACAGCAATATGGTGCCCCTCCTGAATATCAAGGTAGGGGA

AGGGGAGGGCCTTCTCAGCAAGGAGGCCGTGGAGGGTATGGCGGTGGCCGAAGTGGTGGT
GGTATGGGCAGTGGCCGTGGCGTAGGTCCTTCATATGGTGGCCCATCCAGGCCACCGGCA
CCCGAGCTGCACCAAGCAACCTCAGTTCAATTCTATCAAACTGGGGTGAGTTCTCAGCCT
GCATTATCTGAGGCCAGTTCATCACTGCCGCCGCCGGAACCTGTTGATTGTCCTGTAGGA
AGATCATTTTATTCACCTGATTTGGGTAGAAGACAGCCTTTAGGTGAGGGATTGGAAAGC
TGGCGTGGTTTCTACCAGAGTATTCGGCCTACACAGATGGGGCTATCCCTGAACATTGAT
ATGTCTTCCACTGCATTTATTGAGCCATTGCCGGTAATTGACTTCGTAAATCAACTGCTG
AACAGAGATGTATCTGCCCGGCCATTATCTGATGCTGATCGTGTTAAGATCAAGAAAGCT
CTTCGAGGTATCAAAGTTGAAGTAACACATCGTGGAAACATGAGAAGGAAATATCGTATC
TCTGGTCTGACTTCACAGGCAACCAGAGAATTGACATTCCCAGTAGATGAAAGGGGAACC
ATGAAATCTGTTGTGGAGTACTTCTATGAGACATATGGGTTTGTCATTCAACATACTCAG
TGGCCTTGTCTGCAAGTTGGCAATACACAGAGACCTAACTATTTGCCAATGGAGGTTTGC
AAGATAGTGGAAGGTCAAAGGTACTCAAAAAGGCTTAATGAGAGGCAAATCACCGCTTTG
CTGAAAGTTACATGCCAGCGTTTACTCATTGTTATCTTGCCGGATAATAATGGATCACTA
TATGGTGACCTCAAACGTATTTGTGAGACAGATCTAGGACTTGTTTCACAATGTTGCTTA
ACTAAGCATGTCTTCAAAATGAGCAAGCAGTACCTTGCAAATGTTGCTTTGAAAATTAAT
GTCAAAGTTGGAGGGAGAAACACTGTACTGGTTGATGCGCTCTCACGACGCATTCCCTTG
GTCAGTGACAGACCTACAATTATTTTTGGAGCTGATGTGACTCATCCACATCCTGGAGAG
GATTCAAGTCCATCAATTGCAGCAGTTGTGGCTTCGCAAGACTATCCTGAAATTACAAAG
TATGCTGGTTTAGTTTGTGCCCAAGCTCATAGGCAGGAACTCATCCAGGATCTTTTCAAA
CAATGGCAAGATCCAGTCAGAGGAACAGTGACTGGTGGAATGATCAAGGAACTTCTTATA
TCTTTTAGGAGAGCTACAGGACAAAAGCCACAACGCATCATATTTTATAGGGATGGTGTT
AGTGAGGGTCAATTTTATCAGGTTCTACTGTTTGAGCTTGATGCTATTCGAAAGGCATGT
GCATCCCTGGAACCCAACTATCAGCCTCCTGTGACTTTTGTGGTGGTTCAAAAGCGTCAC
CACACAAGGCTCTTTGCCAGCAACCATCACGATAAGAGTTCTTTTGACAGGAGTGGCAAC
ATATTGCCTGGTACTGTTGTTGACTCCAAAATCTGCCATCCCACCGAATTTGACTTTTAT
CTCTGCAGCCATGCTGGAATACAGGGTACAAGCCGTCCTGCTCACTACCATGTGTTGTGG
GATGAAAACAATTTTACTGCTGATGCCTTGCAAACACTCACCAATAATCTTTGCTACACA
TATGCTCGGTGCACCCGATCTGTTTCAATTGTGCCTCCTGCATACTATGCTCACCTTGCT
GCATTCCGTGCAAGGTTTTACATGGAA--------------------------------

>GRMZM2G347402_T01 cds:_protein_coding
CTACTTGTTAGACAGTCATTTTTCAGTGATGATAGTCGAAACCTTGTTGATTTAACTGGT
GGAGTTAGTGGTTGTCGTGGACTCCACTCTAGTTTCCGTACTACAATTGGTGGTCTTTCA
CTAAATATGGATGTTTCAACCACTATGGTTGTAACTCCTGGACCAGTTATTGATTTTCTC
GTCACAAATCAAAATGTAAGAGACATCAGAGATATTGACTGGCCCAGGGCCAAGAAAATG
CTTAAAAATCTCAGAGTTAAAGCTAAGCACAACAACATGGAGTTCAAGATTATTGGCCTT
AGTGATCAACCATGCTCTAGACAGATGTTCCCAATGAAAGTTCGAAATGGAAACATCGAA
ATTAAATCTGTTGATATCACTGTTCAGGATTATTTAAATCCAAGCAAGTTGAGCTAACA
ATGCCTTATCTGCCATGTCTTGATGTGGGAAAACCAAAACGCCCTAATTATCTCCCAATT
GAGTTATGCCACATGGTATCACTTCAACGTTATACAAAGGCACTGTCTTCTCAACAAAGG
GCAATGTTGGTTGAAAAGTCACGACAGAAATTTCTCCTTTGTCTTTTACCAGAGAGGAAG
AATTGTGATATTTACGGGCCATGGAAGAAGAAAAATCTTCATGAAATGGGTATTGTCACT
CAATGCATTGCTCCAAGTAATAAGATGAATGATCAATATTTCACCAATGTTCTTCTAAAA
ATTAATGCTAAGCTTGGTGGAATGAACTCCAAACTGGCACTGGAACATCGTCAAATGATA
CCAGTTGTGACTCAAATACCAACATTAATTCTTGGCATGGATGTTTCACATGGTTCTCCA
GGTCGAGCAGATATACCATCAATTGCTGCGGTTGCCCACAACAACTCGCGTAACAGCAGC
CTGCTACGCAAGCTGCGCCTCCCTCTTAGG------------------------------

>GRMZM2G108281_T03 cds:PUTATIVE_protein_coding
GTGTCCATAGGGCGGTCCTTCTACTCGCCGGACATCAGGAAGCCGCAGCGGCTCGGCGAC
GGCCTGCAGTCATGGTGTGGGTTCTACCAGAGCATCCGGCCGACCCAGATGGGATTGTCA
CTCAACATCGACATGTCGTCCACGGCGTTTATTGAACCCCTGCCGGTGATCGAGTTCGTG
GCCCAGATATTAGGAAAGGATGTCATATCAAGGCCATTGGCCGATGCTAACCGAATCAAG
ATCAAGAAGGCATTACGGGGCGTAAAAGTTGAGGTCACGCACCGGGGGAATGTAAGGCGC
AAGTATCGCATATCTGGGCTCACAACACAGCCAACTCATGAACTGATTTTCCCAATTGAT
GAACAAATGAATATGAAATCTGTCGTGGAGTACTTCAAGGAAATGTATGGTTTCACCATT
CAGCATCCTCATCTTCCTTGCCTTCAGGTTGGAAACCAAAAGAAGGCGAACTATTTACCA
ATGGAGGCCTGCAAGATCGTTGAAGGCCAGAGATACACGAAGAGGTTGAATGAAAAACAG
ATCACATCGTTGCTAAAGGTTACATGCCAAAGGCTTCTTCTGGCTATACTCCCCGACAAC
AATGGTCCGTTATATGGTGACATCAAACGTATTTGTGAAACTGATTTGGGATTGATATCA
CAATGTTGCTTAACCAAGCATGTTTTTAAGATCAGCAAACAGTACTTGGCAAATGTCTCA
CTGAAAATTAATGTTAAGATGGGAGGAAGAAACACTGTGCTCCTGGACGCAATAAGTTGG
AGCATTCCTTTGGTCAGTGACATCCCAACTATTATATTTGGTGCAGATGTAACACACCCT
GAAACCGGGGAGGACTCAAGTCCATCAATCGCTGCCGTTGTTGCTTCTCAAGATTGGCCA
GAAGTTACAAAGTATGCTGGATTGGTTTGTGCTCAGGCACACCGGCAAGAGCTCATTCAG
GACCTTTACAAAACATGGCACGATCCTCAGAGAGGCACTGTAACAGGCGGCATGATCAGG
GAGCTGTTAATATCCTTCAGGAAGGCCACTGGGCAGAAGCCATTGAGAATAATATTCTAC
AGGGACGGTGTTAGTGAAGGCCAGTTCTATCAAGTTCTCCTTTACGAGTTAGATGCCATC
CGTAAGGCATGCGCATCCCTAGAACCAAATTACCAGCCTCCTGTAACATTTGTGGTGGTT
CAAAAACGTCATCATACGAGACTATTTACAAACAATCACAAAGACAGAAGTAGCATGGAC
AAGAGTGGAAATATTTTGCCAGGAACTGTTGTTGATTCTAAGATATGCCACCCAACAGAG
TTTGATTTCTACCTCTGTAGTCATGCTGGAATCCAGGGAACAAGTAGGCCCGCTCACTAC
CATGTCCTCTGGGATGAGAACAATTTCACAGCAGACGAAATGCAAACACTGACAAACAAC
CTTTGCTACACTTATGCCCGGTGCACACGCTCGGTTTCTGTTGTCCCTCCTGCATACTAC
GCACACCTGGCAGCATTCCGGGCGCGGTTCTACATGGAA----------------------
----

>Sbicolor_Sb06g025560
ATGGTGAGGAAGAAGAGAACTGGCCCTGGTGGCTCTGGAGAAACTTCTGGAGAGTCTTCA
GGAGGTTCTGGACAAGGTTCTTCACAGCGGCCTGAGCGAACTCAACAACCTGGGGGAGGA
CGTGGCTGGGTGCCTCAACAGGGTGGTCGTGGTGGCGGGCAACACCAGGGTCGTGGTGGA
CATTATCAGGGCCGCGGAGGACCTGGGTCACATCACCCTGGTGGTGGGCCCCCTGAGTAT
CACCCGCGTGAATACCAGGGACGTGGCGGTGAATATCAGGGACGAGGTGGTGAGTACCAG
GGACGTGGTGGCGCCCGCTCCAGAGGTGGAATGCCACAGCCATACTATGGTGGGCATAGG
GGAGGTAATGTTGGACGAAATGTTCCTCCAGGTCCTTCTAGAACAGTTCCCGAGCTGCAC
CAAGCCCCTTATGTCCAGTATCAAGCCCCGGTGGTTTCACAATCCCCATCGGGACCTGGC
TCATCCTCACAGCCTGTGGCAGAGGTGAGCTCTCCTGTTGGTAGGTCATTTTATTCTCCC
AACTTAGGGAGACGCCAGCAACTTGGTGAGGGTCTGGAAAGTTGGCGTGGCTTTTACCAA
AGCATAAGGCCGACACAGATGGGCCTTTCACTGAATATTGATATGTCCTCTACCGCATTT
ATCGAGCCTCTCCCTGTGATCGATTTTGTTGCTCAGCTTCTTAACAGAGATATCTCAGTT
AGACCATTGTCTGATTCTGATCGTGTGAAGATCAAAAAAGCCCTAAGAGGTGTGAAGGTC
GAGGTCACTCACAGGGGAAACATGCGCAGAAAATATCGCATTTCTGGCCTAACCTCACAA

GCAACAAGAGAGCTATCATTCCCTGTTGATGATCGCGGTACTGTGAAGACTGTGGTGCAA
TACTTCATGGAGACTTATGGTTTTAGTATCCAGCACACCACTTTACCGTGCTTGCAAGTG
GGCAATCAACAAAGACCAAATTATCTTCCAATGGAGGTTTGCAAAATAGTTGAAGGACAG
CGCTACTCAAAGCGACTCAATGAGAAGCAAATCACTGCTCTACTGAAAGTGACCTGCCAG
CGCCTGCTGATTGTAATACTGCCTGACAATAATGGTTCTCTTTATGGGGATCTCAAAAGG
ATCTGTGAGACTGATCTTGGATTGGTCTCCCAGTGTTGTCTGACTAAACATGTTTTTAAG
ATGAGCAAGCAGTATCTTGCAAATGTTGCACTCAAAATAAATGTTAAGGTTGGGGGAAGG
AATACTGTACTTGTAGATGCTTTGACAAGGAGAATCCCCCTTGTCAGTGACAGGCCGACC
ATAATATTTGGTGCTGATGTTACCCATCCACATCCTGGAGAAGATTCCAGTCCTTCCATT
GCAGCTGTTGTTGCTTCACAAGACTGGCCTGAGGTCACCAAATATGCTGGACTAGTGAGT
GCCCAAGCTCATCGCCAGGAGCTGATACAGGATCTTTTCAAAGTATGGCAAGACCCACAG
AGAAGGACAGTAACTGGTGGCATGATAAAGGAACTTCTCATTTCTTTCAAGAGGGCAACT
GGACAGAAGCCCCAAAGGATCATATTCTACAGGGATGGTGTCAGTGAGGGACAGTTCTAT
CAAGTATTGTTGTATGAACTTGATGCCATCAGAAAGGCCTGTGCATCCCTGGAGCCCAAT
TACCAGCCTCCAGTTACTTTTGTCGTGGTCCAGAAGCGACATCACACTAGGCTGTTTGCT
AATAACCACAATGACCAGCGTACAGTTGATAGAAGTGGAAACATACTGCCTGGCACCGTG
GTTGATTCCAAGATTTGCCATCCTACGGAATTTGATTTCTACCTGTGTAGCCATGCCGGC
ATTCAGGGAACAAGCCGCCCTGCTCATTACCATGTCCTGTGGGATGAGAACAAGTTTACA
GCTGATGAGCTGCAGACTCTGACAAACAACCTGTGCTACACGTACGCTAGGTGCACTCGC
TCCGTGTCAATTGTGCCCCCGGCATACTATGCTCATCTGGCAGCCTTCCGAGCTCGCTTC
TACATGGAG--

>Rcommunis_29844.t000058
TGCCCAGTTGGTAGATCCTTCTTTTCACCAGATATTAGAGCACCACAGCGACTTGGTGAT
GGCTTGGAGTCATGGTGTGGGTTTTACCAGAGTATAAGACCTACACAGATGGGCCTGTCA
CTGAATATTGATATGGCTTCAGCTGCATTCATTGAGCCTCTCCCTGTAATTGAGCTTGTT
GCCCAGCTTCTAGGCAAGGATGTGCTATCAAGGCCATTATCTGATGCTGATAGAATTAAG
ATCAAAAAGGCTCTCAGAGGAGTTAAAGTTGAAGTAACTCACAGAGGAAATGTACGAAGA
AAGTATCGTGTCTCAGGATTGACATCTCAACCTACGAGAGAACTTGTATTTCCTGTTGAT
GATAACTCAACTATGAAGTCAGTAGTAGAATATTTCCAAGAAATGTATGGCTTTACCATT
CAACATACACATCTACCTTGCCTTCAAGTAGGAAACCAGAAGAAAGCCAACTATCTACCA
ATGGAGGCTTGCAAAATTGTAGAGGGGCAAAGATATACAAAAAGGCTGAATGAGAGGCAA
ATTACTGCCCTGTTGAAAGTTACATGCCAACGACTTTTATTAGCTATTCTACCTGACAAC
AATGGCACCCTATATGGTGATCTCAAAAGGATCTGTGAAACGGATCTTGGTTTAATATCA
CAATGCTGTCTTACAAAACATGTCTTCAAGATCAGCAAGCAGTATCTGGCTAATGTGTCC
CTTAAGATTAACGTTAAGATGGGTGGCAGAAATACTGTCCTTTTGGACGCCATAAGCTGT
AGAATACCATTAGTTAGTGACATACCAACCATTATATTCGGAGCAGACGTGACTCACCCA
GAGAATGGGGAGGATTCAAGCCCCTCAATTGCAGCTGTAGTAGCTTCACAGGACTGGCCT
GAAGTGACAAAATATGCTGGATTAGTTTGTGCTCAGGCTCACAGGCAAGAACTCATACAG
GACTTGTACAAAACATGGCAAGATCCTGTTCGTGGTACTGTTAGTGGTGGCATGATCAGA
GATCTTCTGGTCTCGTTTCGGAAGGCAACAGGCCAGAAACCGCTAAGGATTATATTTTAC
AGGGACGGTGTTAGTGAAGGGCAATTTTATCAAGTTCTGCTTTATGAATTAGATGCAATA
AGAAAGGCCTGTGCTTCTCTAGAACCAAACTATCAACCTCCTGTGACTTTCATTGTTGTA
CAAAAACGCCACCATACTCGATTGTTTGCTAACAACCATAGGGATAGGAGTAGCACAGAC
AAGAGTGGGAACATATTACCTGGCACTGTGGTTGATTCTAAAATCTGTCATCCGACAGAA
TTCGATTTTTACCTCTGTAGCCATGCTGGTATTCAGGGGACTAGTAGGCCTGCTCACTAC
CATGTTCTGTGGGATGAGAACAACTTCACTGCAGATGGAATCCAGTCTTTGACAAACAAT
CTGTGTTACACATATGCAAGGTGTACTCGCTCCGTTTCCGTTGTACCTCCGGCATACTAT
GCGCATTTAGCTGCTTTTCGTGCCCGGTTTTACATGGAG---------------------

>Mguttatus_mgf011360m
TGTCCTGTCGGAAGATCGTTTTTTTCTCCGGATATAAGGAAACCGCAAAGACTAGGTGAC
GGTTTGGAGGCGTGGTGTGGATTCTATCAGAGCATACGGCCCACTCAGATGGGATTGTCA

CTTAACATAGATATGGCTTCGGCTGCATTTATTGAGGCTCTTCCGGTTATCGAATTCGTC
GCTCAATTATTGGGGAAAGATGTTTTGTCGAGGCCGTTATCCGATTCGGATCGCATTAAG
GTTAAAAAAGCACTTAGAGGAGTAAAAGTTGAAGTGACTCATAGAAGAGATGTTCGAAGA
AAATATCGAGTTTGCGGCATTACGACTCAGCCCACTCGTGAGCTAGTATTTCCGGTGGAT
GACGACTCGAACATGAAATCCGTGGTCGAGTATTTCCAAGAAATGTACGGTTTCACAATA
CAGCACACTCATCTGCCTTGCCTACAAGTAGGGAATCAGAAGAAGGCAAACTATTTACCC
ATGGAGGTCagatgttgttggcttgtaAAAATTGTGGAGGGACAGAGATATACTAAAAGG
CATGTGATAACTGTTCAACACAATGCTTATGAAGAAGATCCGCTTTTATTAGCTATTTTG
CCCGACAACAACGGCTCTCTATACGGTGATTTGAAGCGAATATGTGAAACCGATCTTGGT
ATAATATCCCAATGTTGTCTCACGAAACACGTTTTCAAGATCAACAAGCAATATCTTGCT
AATGTGTCCTTAAAAATAAATGTCAAGATGGGTGGAAGAAATACGGTTTTATTGGATGCA
ATAAGCTGTAGAATACCGCTAGTGAGTGATATACCTACGATTATATTCGGAGCTGATGTA
ACGCATCCCGAAAACGGAGAGGAGTCGAGTCCATCTATAGCTGCTGTTGTAGCTTCTCAA
GATTGGCCCGAGGTGACAAAGTACGCGGGGTTGGTTTGCGCGCAAGCGCATAGACAAGAA
TTGATACAAGATTTGTACAAGACTTGGCAAGATCCTGTTCGTGGGACCGTGAGTGGCGGC
ATGATCAGGGATCTTTTGGTTTCGTTTAGAAAAGCAACTGGACAAAAGCCACAGAGGATA
ATATTTTACAGGGATGGTGTGAGTGAAGGGCAATTTTACCAAGTGCTACTATTTGAGTTG
GATGCTATTCGCAAGGCTTGCGCCTCGTTGGAGCCAAACTATCAACCACCGGTGACTTTT
ATCGTAGTTCAAAAACGGCATCACACAAGACTTTTTGCTAACAATCATAGGGATAAAAGC
AGCACCGACAAGAGCGGAAACGTATTACCTGGTACGGTGGTCGATTCGAAAATCTGTCAT
CCTACAGAATTCGACTTTTATCTTTGTAGTCATGCTGGAATTCAGGGAACGAGTCGACCT
GCTCACTACCATGTACTCTGGGATGAGAACAATTTCACTGCCGACGGAATTCAGTCACTG
ACGAACAATCTATGTTATACATACGCCAGGTGTACACGCTCCGTTTCAGTTGTTCCCCCA
GCTTACTATGCACATTTGGCTGCATTCCGGGCGAGATTCTATTTGGAA------------

>Mesculenta_cassava7471.valid.m1
CTCCTTGTTCGCCAGAACTTTTTTCATAATGATCCAAGGAATTTCGCAGATGTTGGGGGT
GGTGTTCTCGGCTGTAGAGGGTTTCATTCAAGTTTTAGAACCACTCAGGGCGGCTTGTCT
CTGAATATTGATGTATCTACAACCATGATAATTCAGCCTGGTCCTGTTGTGGACTTTCTA
ATTGCCAACCAAAATGCGAGAGACCCCTTTCAACTTGACTGGGCAAAGGCAAAACGAACT
TTAAAAAATCTGAGGATCAAGGCTAGGCCGTCCAATCAAGAGTACAGGATAACTGGTCTG
AGTGAGAAACCTTGCAGCGAGCAAACCTTTCAACTAAAGCAAAAAAGTGGGGATGGCGAA
CCTCTTGAGTTAACTGTTTACGAATATTTTGTTAATCACCGCCGTATAGAATTGAGGTAT
TCTGCGGATTTGCCATGCATCAACGTTGGCAAGCCAAAGCGTCCTACTTATATTCCAATT
GAACTTTGTGATTTGGTTTCCCTGCAACGTTATACAAAAGCACTTAATACGCACCAAAGA
GCTTCTTTGGTGGAGAGATCAAGACAAAAATTTCTTTTGTGCTTACTCCCAGAGAGGAAA
AACTCTGACATATATGGTCCTTGGAAGAAGAAAAATCTTTCTGAGTTTGGAATTTTCAAT
CAGTGCTTGGCTCCGCAGAGGGTCAATGACCAGTATCTTACAAATCTTCTCTTGAAGATC
AATGCAAAGCTTGGCGGGTTAAATACTTTGTTAGCAGTGGAGCAAACACCTTCTATCCCT
TTGGTTTCTAAGGTTCCTACAATTATCCTTGGGATGGATGTGTCACATGGCTCTCCTGGG
CATTCTGATGTCCCATCGATTGCTGCGGTGGTCAGTTCCAGGCAGTGGCCATTGATTTCT
CGTTACAGGGCATCTGTCCGTACACAGTCTCCAAAGGTTGAAATGATCGACTCTCTGTTT
AAAAGGGTATCTGAGACCGATGATGAAGGAATGATTAGGGAGCTTCTGTTGGACTTCTAT
ACTAGTTCAGGGAAAAGAAAACCTGATCAGATCATCATATTCAGGGATGGTGTCAGTGAA
TCTCAATTCAATCAAGTATTAAATATTGAACTGGATCAGATAATTGAGGCATGCAAGTTT
CTTGATGAGAAATGGAACCCCAAGTTTGTTGTCATTGTTGCCCAGAAAAATCACCATACA
AAATTCTTCCAGCCAGGAGCTCCTGAAAATGTTCCACCTGGTACTGTCATAGACAACAAA
GTCTGTCATCCTAGGAACTATGATTTCTATCTCTGTGCACATGCAGGAATGATTGGAACT
ACAAGGCCCACACATTACCATGTTTTATTAGATGAGGTTGGGTTTTCAGCAGATGATCTT
CAGGAACTAGTGCATTCTCTATCATATGTGTATCAAAGAAGCACAACTGCCATATCTGTA

GTTGCGCCAATATGCTACGCACACCTGGCTGCAACCCAGATGGGAACATTCATGAAG---

>Creinhardtii_Au9.Cre01.g071850
TTCCCCATGCCCGGCGGCCGCGCGCGGCGCAAGGCGCTGGTGGGTCTGAGCGAGCAGGGC
GCCGACCGCACCATGTTCATGAACGAGAAGGAGGGGCGTGAGATGTCGGTGGCCGAGTAC
TTCCGCTCCACGGGCCGCCCGCTGCGCCACCCCGGCCTGCCCTGCGCCAACGTGGGCGAC
CGCCGCCGCGCCGTGTTCATTCCCGTGGAGCTGTGCACTGTGGTTGCTGGCCAGCGGCGC
ATGAAGCTGGACGCCACGCAGTCGGCCGGCATGATCACCGCCGCCAAACAGGACCCGCTG
GTGCTGGTGGTGCTGCCGGAGAAGACCGCGGACGAGTACCGGGAGGTGAAGCGCGTGTCG
GACATTGAGCTGGGCATACCCAGCCAGGTGGTGGTGGCGTCCAAGGCGCGTGTGGGCTAC
CGCgcgcacAAGGGCGGCGGCCCGCAGTACTGCGCCAATGTGGCCATGAAGATCAACAAC
AAGCTGGGCGGAGTCAACGTGCAGCTCAGCGGCGGACTGCGCAACATGCCGGTGCTGGGC
GGCGCGGGCGCCGTGCCCTTCATGGTGCTGGGCGCCGACGTGACGCACCCCACCGGCGCC
GCGGCGCGCGCCGACAGCCGCGACCCCTCCGTGGCGGCCGTGGTGGCCAGCCTGGACGCC
AGCCTGGGCCGCTGGGCCAGCCGCGTGCTGCTGCAGGCGGGGCGGCAGGAGGTGATCACG
GGCATGTGCGGCGCCACAAAGGAGCTGCTGCTGGAGTTCTACAGGGCAAACAAGCAGGTC
AAGCCGCAGCGGCTGGTCATGTACCGCGACGGCGTGTCGGAGGGCCAGTTCGAGCAGGTC
CTGGCTGAGGAGTACACGGCCCTGCGCCGCGCCTGCCGCGAGCTGGAGGAGGGCTACCGG
CCCGCCATCACCTTCGTGGTGGTGCAGAAGCGCCACAACACCCGCCTGCTGCCCAGCGAC
CGAGCCGCGTCCGACCAGAAGGGCAACGTGGTTCCGGGCACTGTGGTGGACAGCGGCATC
ACCGCCCCGGACGGCTTCGACTTCTACCTCAACAGCCACGCCGGGCTGCAGGGCACCAAC
AAGCCCGCCCACTACCACGTGCTGGTGGACGAGATCGGCTTCGGCGCCGACGGCATCCAG
CTGCTCACCTACTGGCTGTGCTACCTCTACCAGCGCACAACAAAGTCGGTCAGCTACTGC
CCGCCCGCCTACTACGCCGACCGCGCCGCCTTCCGCGGCCGCACGCTGCTGGCC------

>Sbicolor_Sb01g004920
GTCACAGTGTCCAGATCCTTTTTCTCTACTACTTTTGGCCACCGAGGCGACATTGGCGAG
GGGCTTGAGTGTTGGAGGGGTTACTACCAAAGCCTGCGCCCGACACAAATGGGGCTTTCA
CTGAATATAGACATATCAGCAACATCCTTTTTTAAGCCTGTGTCAGTTATCAAATTTGTG
GAGGAGTACCTGAACATGCGTGATACATCTCGGCCTTTGTCGGACAGAGATCGTGTGAAG
ATAAAGAAAGCATTACGTGGAGTTCGCATTGAAACAACCCACCAACAGGATCAAATCAGA
AGATACAAGATAACAGGGGTTACTTCCATCCCTATGAGCCAGCTGATATTTCCTGTTGAT
GATAAAGGAACAAGAAAGACTGTTGTGCAGTACTTCTGGGACAAATACAACTACAGCTTG
AAGCATGGTTCTTGGCCTTGTCTTCAGGCTGGCAGTGATTCACGACCTGTATATTTGCCT
ATGGAGGTTTGTAAAATTCTGGAAGGGCAGAGATACTCTAAGAAGCTTAATGACAGACAA
GTGACCAACATACTTAGAGCAACATGTAAACGTCTTCTGATCGTAATTCTGCCTGATGTT
AGTGGTTCTTATGGTAAAATCAAAAGAGTCTGTGAGACTGACATTGGAATTGTATCTCAG
TGTTGCTTGCCAAAGCATGCTAGCAGACCAAACAAACAATATTTAGAAAATGTTGCACTC

AAAATCAATGTCAAGGTTGGTGGGCGCAACACAGTTCTTGAGCGAGCCTTTGTACGCAAT
GGCATACCCTTTGTGTCAGAAGTCCCAACAATCATCTTTGGTGCAGATGTCACACACCCC
CCACCAGGAGAGGACTCTGCCTCATCCATTGCTGCTGTGGTGGCATCAATGGACTGGCCA
GAAATCACCAAGTACAGAGGCTTGGTTTCTGCTCAACCACACCGACAAGAGATAATAGAA
GATTTGTTTACTGTCACTAAGGATCTGCAGAAGGGGCACAGTGTTAATGGTGGAATGATC
AGGGAGCTACTGATTGCTTTCCGCAGGAAGACAAACAGAAGGCCTGAGAGGATAATATTC
TATCGGGACGGTGTAAGTGAAGGTCAATTCAGCCATGTACTTCTTCATGAAATGGATGCC
ATCAGAAAGGCTTGCGCTTCTTTGGAGGAGGGATATCTACCTCCAGTCACATTTGTGGTT
GTCCAGAAAAGGCATCACACAAGGCTTTTCCCTGAGGTTCATGGAAGGCGTGATATGACT
GATAAAAGTGGAAACATTCTTCCCGGAACTGTGGTTGACCAACAGATTTGCCATCCTACT
GAGTTTGATTTCTACTTGTGTAGTCATGCTGGTATTCAGGGAACAAGTCGGCCCACCCAT
TACCATGTCCTCTATGATGAGAATCATTTTACTGCTGATGCACTGCAGTCACTGACCAAC
AATCTATGCTACACTTATGCTCGTTGCACCCGTGCTGTATCAGTGGTTCCACCAGCCTAC
TATGCCCACCTTGCCGCATTCCGGGCACGCTACTATGTGGAA------------------

>Sbicolor_Sb04g038420
GCACCATTTGGCCGGTCATTCTTTTCGCCTGACTTGGGCCGAAGGCAGCCCCTTGGTGAT
GGATTAGAAAGCTGGCGTGGATTCTACCAGAGCATTCGTCCTACTCAAATGGGCTTGTCA
CTCAACATTGATATGTCAGCGACAGCTTTCATTGAGCCATTGCCTGTAATCGAATTTGTT
GCACAGCTGCTTAATTGTGAAATTCACTCTAGGCCACTCTCAGACGCGGAACGGGTGAAG
ATCAAGAAAGCCTTGCGAGGAGTTAAGGTGGAAGTTACTCATCGTGGAAACATGCGGAGA
AAGTATCGAATATCTGGGTTAACAACTCAGGCGACTCGAGAGTTAACCTTTCCTGTTGAT
GAAGGGGGTACAATAAAGTCAGTTGTACAATACTTTCAAGAGACATATGGATTTTCCATT
CAACACACCTACCTTCCTTGCCTTCAAGTTGGCAATCAACAGCGTCCAAATTACTTGCCA
ATGGAGGTCTGCAAAATAGTGGAGGGACAGAGGTACTCCAAGAGATTAAACCAGAATCAG
ATCAGAGCTCTTTTGGAGGAGACATGCCAGCACTTACTTATTGGAATACTACCTGACAAC
AATGGCTCACTTTATGGTGATTTGAAGCGTGTCTGTGAAATAGATCTTGGGATAGTTTCA
CAGTGCTGTTGCACAAAGCAGGTTTTCAAAATGAACaacAAACAAATTCTTGCAAATCTT
GCTCTGAAGATTAATGTGAAGGTTGGGGGCAGGAACACCGTGCTGGTAGATGCCGTCTCA
AGGGGAATTCCTCTGGTAACTGATCGACCTACAATCATATTTGGTGCTGATGTGACTCAT
CCTCATCCCGGTGAGGACAGTAGTCCCTCAATTGCTGCTGTTGTGGCCTCCCAAGATTGG
CCAGAGGTGACAAAGTATGCTGGACTAGTTTGTGCTCAAGCTCATCGGCAAGAGTTGATA
GAGGATTTGTATAAGGTCTGGCAAGATCCACAGAGAGGGACAGTCAGCGGTGGAATGATA
AGGGAGCTACTTGTATCCTTCAAAAAATCAACTGGTGAGAAGCCCCAGCGAATAATATTT
TACAGGGATGGTGTCAGTGAAGGACAATTTTATCAAGTTCTGTTGTATGAGCTCAATGCT
ATCCGAAAGGCCTGTGCCTCCCTGGAAGCGGAGTACCAACCAAAGGTGACTTTTGTTGTG
GTTCAGAAGCGCCATCATACTAGATTATTTGCTCACAACCACAATGATCAGAATTCAATT
GACAGGAGTGGAAACATACTCCCAGGTACTGTTGTAGATTCGAAGATCTGTCATCCTACT
GAATTTGACTTCTACTTGTGTAGCCATGCTGGCATTAAGGGCACTAGCCGTCCAGCTCAT
TATCATGTCTTGTGGGATGAAAACAACTTCTCTGCTGACGAGTTGCAGACTCTTACAAAC
AACCTCTGTTACACTTATGCAAGGTGCACCCGCTCTGTATCGATCGTTCCACCAGCGTAT
TATGCTCACCTGGCTGCCTTCAGGGCTCGTTTTACATGGAA------------------

>AT1G69440.1

ACATCGATCGGAAGATCGTTTTACTCGAGTTCTATGGGTGGATCTAAGGAGATTGGAGGA
GGAGCTGTTGGACTCAGAGGGTTTTTCCAGAGTCTTAGACATACTCAGCAAGGTTTAGCA
CTTAACATGGATCTCTCAATCACAGCTTTCCATGAAAGTCAGAAGCGGCTCGAGTTTCTT
ACGGACCTTCCTAGGAACAAAGGTAGAGAATTGAGTTTAGAGGAAAAGAGAGAAGTGGAG
AAAGCACTTAAGAACATAAGAGTCTTTGTTTGCCATAGAGAAACAGTTCAAAGGTATCGA
GTTTATGGGTTAACAGAGGAAATTACGGAGAATATATGGTTTCCTGATAGAGAAGGGAAA
TATCTAAGGCTTATGAGTTACTTCAAAGATCATTATGGTTATGAGATTCAGTTCAAGAAC
TTGCCGTGTCTGCAAATCAGTAGGGCAAGACCTTGTTACCTTCCTATGGAGCTATGCATG
ATTTGTGAAGGTCAAAAGTTTCTTGGAAAGCTATCAGATGATCAAGCTGCAAAGATCATG
AAAATGGGCTGCCAAAAACTGATTATTTGTGTAATGGAGAAAAAACATAAAGGGTACGGA
GATCTAAAGAGGATATCAGAGACAAGAATTGGTGTTGTGACACAATGCTGCTTATACCCT
AACATCACTAAGCTCAGTTCTCAGTTCGTTTCGAACTTAGCTCTCAAGATAAACGCCAAG
ATCGGTGGATCCATGACCGAGCTCTACAACTCGATACCTTCTCACATCCCTAGACTGCTT
AGACCCGATGAGCCGGTTATCTTCATGGGAGCTGATGTAACGCATCCTCATCCATTCGAT
GATTGTAGCCCTTCAGTAGCGGCTGTGGTAGGGAGCATAAACTGGCCAGAAGCTAACCGA
TACGTCTCAAGAATGAGGTCTCAGACTCATAGGCAAGAGATCATACAAGATCTTGACTTG
ATGGTCAAGGAACTTCTTGATGATTTTTACAAAGCGGTAAAAAAGCTTCCGAATCGAATC
ATATTCTTCAGGGACGGTGTTAGCGAGACACAGTTCAAGAAAGTTCTCCAAGAAGAGCTT
CAATCGATAAAAACTGCTTGTTCGAAGTTCCAAGATTACAATCCAAGCATCACATTCGCC
GTGGTTCAGAAAAGACACCACACAAGGCTGTTCCGGTGCGATCCAGACCATGAGAACATA
CCTCCTGGTACAGTGGTTGATACAGTGATAACTCATCCGAAAGAGTTTGATTTTTATCTC
TGTAGCCATTTAGGAGTGAAGGGCACGAGCAGGCCAACGCATTACCATATTCTATGGGAC
GAGAACGAGTTCACTTCAGACGAATTGCAGAGACTTGTGTATAATTTGTGTTACACTTTC
GTGAGGTGCACGAAACCTATATCGATTGTGCCACCGGCTTATTATGCTCACCTTGCTGCG
TACAGAGGAAGGCTATACATCGAG------------------------------------

>Sbicolor_Sb01g011880
GTATCCATCTCAAGATCATTTTTCTCAAAGGCATTTGGACATGGTGGTGATATTGGCAGT
GGTGTGGAATGCTGGAGGGGGTACTACCAAAGTCTACGTGCTACACAAATGGGACTGTCC
TTGAATATTGATATTTCAGCAACTGCATTTTACAAGGCTCAACCGATTTTGGACTTTGCA
TTGGAGTACCTCAATATACGTGACACCTCAAGGCGTTTGTCTGACCAGGATCGCATAAAA
TTGAAGAAAGTCCTCAAAGGAGTACGGGTTGTGGCAACACATCGTCGTGATATAGCCATA
CGTTACAAGATTACTGGGATAACCTCACTTCCCTTGAATGATTTAACGTTTGATCAAGAT
GGGACAAGGGTTTCAGTTGTTCAATACTTTAAACACCAATATAATTACTGTTTGAAACAC
ATTCACTGGCCATGCCTTCAAGCTGGCAGTGATAGCAGGCCAACTTATTTACCCATGGAG
GTTTGCAATATACTTGAAGGACAACGCTATTCTAGAAAGCTAAATGAGCGCCAAGTCACA
AGCATCCTGAAGATGGCATGTGAGCGATTACTAATTGTTGTATTGCCAGATGCAAATGCA
AGTTTCTTTTATGGAAGGATAAAGCGTCTTTGTGAAACTGAACTTGGTATAATAACTCAG
TGTTGTATACCTAAGAATGTTCATAAAGGTGGCCGACAATATCTCCAAAACCTGGCCCTT
AAAATTAATGTTAAGGTTGGTGGTCGTAATACAGTTCTTGAAGATGCTTTAAATAGGAGG
ATACATTTGTTAACAGATTTGCCAACAATAATCTTTGGAGCTGATGTTACCCACCCAGCT
CCAGGGGAGGATGCATCCCCGTCTATCGCTGCGGTTGTTGCATCAATGGATTGGCCAGAA
GTTTCAAAGTACAGGTGCTTGGTGTCTTCTCAAGGTCATAGGGAAGAGATCATAGCTGAT
CTTTTCACACAAGTGAAGGATCCACAAAAAGGACTACTTCATGGTGGAATGATCAGGcat
gctgaacatattaactttgctgttcttccaataaatgatctttccttttttttttttgaag
tcatatcgttgctgcAGGGAACTACTTGTATCATTCTATAGGGCAAATGGGAGCCGAAAA
CCGAGCAGGATAATTTTCTATCGAGATGGTGTCAGTGAGGGGCAGTTTAGCCAGGTCTTG
CTTTATGAAGTGGATGCAATTCGAAAGGCTTGTGCCAGCTTGGAAGAGGGCTACCTTCCT
CCAGTTACATTTGTTGTGGTGCAAAAGCGGCATCACACACGGCTTTTCCCTGAAGATCAT

CGTGCACAAGGACAGACAGATCGTAGTGGAAATATTTTGCCAGGAACTGTTGTTGACACG
AAGATATGTCATCCAAGTGAATTCGACTTCTACCTTTGTAGCCACTCGGGCATTCAGGGA
ACGAGCCGTCCAACACACTACCATGTTCTGTTTGACGAAAACAATTTCACCGCTGATGCA
CTACAGACATTGACCTACAAACTGTGCTACACGTATGCGCGGTGCACGCGATCAGTCTCT
ATAGTTCCCCCAGCATACTACGCGCACCTGGCGGCTTTCCGTGCGCGCCACTACTTGGAT

>Alyrata_16063851_locus
ACGGATGCTCCATCTGCAGGAGGTGAAGCCTCTGGGTCTCGTGAAGCTGGTCCAGTCTCA
GGTGGTGGACGTGGTTCACAAAGAGGTGGTTTCCAGCAGGGAGGTGGAGGAGGACAACAG
CAAGGTGGAAGAGGTTATACTCCTCAGTCTCAACAGGGAGGTCGTGGTGGTCGTGGATAT
GGGCAACCACCACAACAGCAACAGCAGTATGGTGGTCCACAAGAGTACCAAGGAAGAGGA
AGAGGAGGACCTCCTCATCAAGGAGGTCGAGGAGGGTATGGCGGTGGCCGTGGAGGTGGA
CCTTCTTCTGGACCACCGCAGAGACAATCAGTTCCCGAGCTGCATCAAGCTACCTCACCT
ACTTATCAAGCGGTGTCTTCTCAGCCTACACTGTCTGAGGTGAGTCCTACCCAGGTGCCG
GAACCTACTGTTCTGACTCCGGTGGGCCGGTCCTTTTATTCCCCTGATATAGGAAGAAAG
CAATCTTTGGGGGATGGCTTGGAGAGCTGGCGTGGATTCTACCAAAGCATTCGTCCTACA
CAGATGGGCTTATCACTCAATATTGATATGTCATCGACAGCATTCATAGAGGCATCCCCT
GTGATTAAGTTTGTCTGTGATTTGCTTAACCGGGATATTTCATCTCGACCTTTATCTGAT
GCTGATCGTGTTAAGATAAAAAAGGCTCTTAGAGGTGTCAAGGTTGAAGTGACTCATCGA
GGAAACATGCGCCGGAAGTACCGTATTTCTGGCTTGACTGCTGTGGCCACTCGGGAATTG
ACATTCCCAGTAGATGAACGAAATACTCAGAAATCTGTTGTAGAATACTTCCACGAAACA
TATGGTTTTCGCATTCAGCACACTCAACTACCATGCTTGCAAGTTGGGAATTCTAACAGG
CCAAATTACTTACCAATGGAGGTATGCAAGATTGTTGAAGGCCAGCGATATTCGAAAAGA
TTGAATGAGAGACAGATCACTGCTTTGCTGAAGGTTACCTGTCAGCGCCTGCTTATTGTC
ATTCTTCCAGACAATAACGGATCATTATATGGTGATTTAAAACGCATATGTGAGACTGAA
CTTGGCATTGTCTCTCAATGCTGCCTGACAAAACATGTCTTTAAGATGAGCAAACAATAC
ATGGCTAATGTTGCGCTGAAGATTAATGTGAAGGTTGGAGGAAGGAATACAGTGCTTGTT
GATGCTTTATCAAGGCGGATTCCACTAGTCAGTGATCGACCCACCATTATATTTGGTGCT
GATGTTACCCATCCTCACCCTGGAGAGGATTCAAGCCCATCTATTGCTGCTGTTGTGGCA
TCCCAGGATTGGCCTGAAATCACTAAATATGCTGGATTAGTTTGCGCTCAAGCGCATAGG
CAGGAGCTCATTCAGGATCTGTTCAAAGAGTGGAAGGATCCTCAGAAAGGGGTGGTGACT
GGTGGCATGATAAAGGAGTTACTCATAGCCTTCCGTAGATCAACTGGGCATAAACCGCTA
AGGATCATCTTCTACAGGGATGGAGTCAGTGAAGGACAATTTTACCAAGTTTTACTCTAT
GAACTTGATGCTATCCGCAAGGCCTGTGCTTCGCTGGAAGCTGGTTATCAGCCACCAGTG
ACATTTGTGGTGGTGCAGAAGCGCCATCACACGAGGCTGTTTGCTCACAACCACAATGAT
CGTCATTCGGTGGACAGAAGTGGGAATATCTTACCTGGCACTGTTGTGGACTCTAAAATC
TGTCACCCTACCGAGTTTGACTTTTACCTCTGTAGTCATGCTGGTATACAGGGAACTTCT
CGACCAGCTCATTACCATGTTCTTTGGGATGAGAACAACTTTACGGCAGATGGACTTCAA
TCTCTGACCAATAACTTATGTTACACGTATGCAAGATGCACACGATCAGTTTCAATTGTT
CCCCCTGCATATTATGCACATCTAGCAGCTTTTAGGGCTCGATTCTACATGGAG------

>Ptrichocarpa_POPTR_0001s22710
CTCCTTGTGCGCCAATCCTTCTTCCATAATGATCCAAAAAATTTTGTGGATTTGGGAGGC
GGTGTTCTTGGCTGCAGAGGTTTCCACTCAAGTTTTAGAACATCTCAGGGAGGCTTGTCT
CTTAATATTGATGTGTCTACGACCATGATAATACAGCCAGGTCCTGTGGTAGATTTTCTA
ATTGCCAACCAAAATGTGAGAGATCCATTTTCACTTGACTGGGCGAAGGCGAAACGAATG
CTCAAAAATCTGAGGGTTAAGGCAAGTCCTTCCAATCAAGAGTACAAGATAACTGGGTTG
AGTGAGAAGACTTGTAAAGAACAAATGTTTCAGTTGAAACAAAAAAATGGAGGGGATGGT
GGGATCGAGGCTGTTGAAATAACTGTTTATGATTATTTTGTCAATCACCGCAAAATCGAT
TTACGATATTCTGGTGATCTGCCATGCATTAATGTTGGGAAGCCAAAGCGCCCTACTTAT
ATTCCTCTTGAGCTTTGTTCCCTGGTGTCCCTACAACGCTATACCAAAGCACTGTCCACA
CTTCAAAGGTCTTCACTGGTGGAGAAATCACGGCAGAAGTTCTTACTGTGCTTGCTTCCT

GAGAGAAAGAATTCTGATATATATGGCCCATGGAAGCGCAAAAATCTTGCTGAATATGGA
ATTGTCACTCAGTGCATTGCGCCTCAAAGAGTTAACGACCAATATATTACCAATGTTCTC
CTGAAGATCAATGCAAAGCTTGGTGGGTTGAACTCTATGTTGGCTGTGGAACACGCCCCC
TCATTACCTCTTGTGTCGAAGGTTCCCACGCTTATCCTTGGGATGGACGTGTCCCATGGC
TCTCCTGGGCAGTCTGATGTCCCTTCAATTGCTGCGGTAGTCAGCTCCAGGCAGTGGCCT
TTGATTTCTCGCTATCGGGCATGTGTGCGAACACAATCCCCAAAGCTTGAGATGATTGAT
TCATTATTTAAGCGAGTGTCTGAGACTGAGGATGAAGGAATAATTAGGGAGCTTCTGTTA
GACTTTTATGTGACTTCAGGAAAAAGGAAACCCGATCAGATCATCATATTTAGAGACGGG
GTCAGTGAATCACAATTCAATCAGGTCTTGAATATCGAATTGGATCAGATAATTGAGGCG
TGCAAGTTTCTTGATGAGAAGTGGTCCCCAACGTTTGTGGTAATTGTAGCTCAGAAAAAC
CACCACACTAAATTTTTCCAACCTGGATCTCCTGATAATGTACCACCTGGTACGATCATT
GACAACAAAGTCTGCCATCCAAGAAACAATGACTTCTATCTCTGTGCTCATGCTGGGATG
ATTGGGACTACAAGGCCTACTCACTACCATGTTTTGTTAGACGAGGTTGGTTTTTCAGCA
GATGATCTGCAGGAACTTGTGCATTCCCTCTCATATGTATACCAAAGAAGCACGACTGCC
ATCTCTGTAGTTGCACCAATCTGCTATGCCCACCTGGCAGCTACTCAAATGGGCCAGTTT
ATGAAG--

>Cpapaya_evm.TU.supercontig_44.130
TGCCCTATTGGAAGGTCATTCTTCTCTCCTGATATTAGAAACCCGCAACGCCTTGGTGAG
GGTTTGGAGTCATGGTGTGGCTTTTATCAGAGTATTAGGCCTACACAGATGGGCTTGTCC
TTAAATATTGATATGGCTTCAGCTGCATTTATTGAGGCTCTCCCTGTGGTAGAGTTTGTA
GCCCAGCTTTTGGGAAAGGATGTTTTATCAAGGCCATTATCTGATTCTGATCGTGTGAAG
ATTAAGAAGGCTCTTAGGGGAGTAAAAGTGGAGGTTACACACCGAGGAAACGTGAGAAGG
AAGTACCGTGTTGCAGGATTGACATCCCAGCCAACAAGAGAACTAGTGTTTCCCGTTGAT
GACAACTCTACCATGAAGTCAGTTGTTGAGTACTTCCAAGAAATGTATGGCTTCACCATT
CAACATACACATCTGCCTTGTCTTCAAGTAGGAAATCAAAAGAAGGCAAACTATTTGCCT
ATGGAGGCCTGCAAAATTGTTGAGGGACAGAGATATACAAAAAGGTTGAATGAGAAGCAA
ATTACTTCCCTTTTGAGAGTTACTTGCCAGAGACTTCTATTAGCTATCTTACCTGACAAT
AATGGGTCCCTGTATGGTGATCTTAAGCGAATCTGTGAAACTGATCTAGGTTTAATATCA
CAATGCTGTCTCACAAAACATGTCTTCAAGATCAGCAAGCAATACCTGGCTAATGTGTCC
CTAAAAATTAATGTTAAGATGGGTGGTAGAAACACTGTTCTTTTGGATGCTATCAGTTGC
AGAATTCCTTTAGTTAGTGATATACCAACTATAATATTTGGAGCAGATGTAACCCACCCA
GAGAATGGGGAAGACTCCAGCCCGTCGATAGCTGCTGTAGTAGCTTCTCAAGATTGGCCG
GAGGTTACTAAATATGCTGGGTTAGTTTGTGCTCAAGCTCACAGACAGGAACTCATACAA
GATTTGTACAAAACGTGGCAGGATCCTGTTCGTGGCAATGTTAGCGGTGGCATGATCAGG
GACCTTCTTGTTTCCTTTAGGAAGGCAACAGGGCAGAAACCATTAAGGATTATATTTTAC
AGGGATGGTGTAAGTGAAGGGCAATTTTATCAAGTTCTACTTTATGAGTTAGATGCAATT
CGAAAGGCTTGTGCCTCTCTAGAACCAGATTATCAACCACCAGTTACTTTCATTGTTGTA
CAAAAGCGGCACCATACTCGATTATTTGCAAGCAACCACAGAGACAGGAGCAGCACAGAC
AAGAGTGGGAACATTCTGCCTGGCACCGTGGTTGATTCAAAAATCTGTCATCCATCAGAA
TTTGATTTTTACCTCTGTAGCCACGCTGGTATTCAGGGGACTAGTAGACCAGCTCACTAC
CATGTGCTATGGGATGAGAACAATTTTACAGCAGATGGGATGCAGTCTTTGACAAACAAC
CTCTGTTATACCTATGCAAGATGCACCCGCTCTGTCTCCGTTGTCCCTCCAGCATATTAT
GCACATTTAGCTGCTTTTCGTGCTCGATTCTACATGGAA---------------------

>AGO1905_Aquilegia
CTACTCTTAGTTATCTTACCTGACAGTAATGGTTCTCTATATGGTGATCTCAAGCGGATA
TGTGAAACAGATCTGGGTTTAATAACACAATGTTGTCTTACTAAGCATGTCTTCAAGATC
AACAAACAGTACTTAGCTAATGTGGCCTTGAAAATAAATGTTAAGATGGGAGGAAGGAAC
ACTGTTCTTTTGGATGCTATCAGCTGCAGGATACCATTGGTTAGTGACATACCAACAATA
ATATTTGGAGCAGATGTCACCCACCCGGAGAATGGAGAAGATTCAAGCCCTTCAATTGCG
GCTGTAGTTGCTTCTCAGGACTGGCCTGAGGTCACAAAATATGCTGGTTTAGTTTGTGCC
CAAGCTCACAGACAGGAACTCATACAAGACTTGTTCAAGACCTGGCACGATCCTGTGCGC
GGCACTGTTAGTGGTGGGATGATAAGGGATCTTTTGATTTCATTTCGAAAGGCAACGGGT
CAAAAACCGCAAAGGATTATATTCTACAGGGATGGTGTAAGTGAAGGGCAATTTTATCAA
GTCCTACTCTATGAGTTAGATGCAATTAGGAAGGCTTGTGCTTCTTTAGAACCAAATTAC
CAACCACCAGTGACTTTTGTCATAGTACAGAAAAGACATCATACTAGATTGTTTGCTAAT
AACCACAAGGACCGCAGCAGTATTGACAAGAGCGGGAATATTTTACCTGGAACTGTGGTT
GACTCAAAAATATGTCATCCGACTGAGTTTGATTTTTATCTCTGCAGCCATGCTGGGATA
CAGGGCACAAGCAGACCTGCTCATTACCATGTTTTATGGGACGAAAACCACTTTACAGCT
GACGGAATTCAATCCCTGACAAACAATTTGTGTTACACGTATGCAAGGTGTACGCGCTCT
GTCTCAGTTGTTCCACCCGCTTATTATGCACACTTAGCAGCATTTCGAGCTAGATTTTAC
ATGGAG--

>LOC_Os07g09020
GTCATCGTTTCTCGGTCGTTCTACTCCACTATGTTCGGCCGACAAGACATTGGTGATGGG
CTGGAATGCTGGAAAGGATACTATCAGAGCCTGCGCCCAACTCAGATGGGACTCTCATTG
AACATAGACATATCCTCTACCCCATTCTTCAAACCTGGCACAcctactaatgctaatggc
cctGACCCTAGGCGGCCTCTTTCTGACATTGATCGCCTGAAGGTTAAGAAAGCACTACGG
GGAGTTCGTGTTGAAACAACACACCAGGGGAAGAGCAGCAAGTACAAGATCACTACGATT
ACATCTGAGCCACTGAGTCAGCTGAACTTTTCTATGGATGGAACTACCCAGACTGTTATT
CAGTACTTCTCGCAGCGGTACAAATACAGGCTGCAGTACACGTCTTGGCCCTGTCTGCAA
TCCGGCAACCCTTCTAACCCTATATATTTGCCAATGGAGGTATGCACCATTGTAGAAGGG
CAAAGATACTCCAAGAAGCTCAATGACAAACAAGTGACTGGCCTCCTGAGAGCAACATGC
CAGCCTCTGCTCATTGTGATTCTCCCAGATGTTAATGGGTATTATGGAAGAATTAAGAGG
GTGTGTGAGACTGAACTTGGGATAGTATCCCAGTGCCTCAAGCCAGGCCGCaagCTCTTG
AGCTTAGACAGGCAGTTCCTGGAAAATGTCTCACTCAAAATCAATGTCAAGGCTGGAGGA
CGCAACTCAGTTCTTCAGAGACCTCTTGTACCCGGTGGGCTTGAAAACACAACAATAATT
TTTGGTGCCGATGTCACCCATCCTGCTTCTGGAGAGGACTCATCGGCGTCGATCGCAGCT
GTGGTGGCCTCCATGGACTGGCCTGAGATCACCAAGTACAAAGCCCTCGTCTCTGCCCAG
CCACCTCGGCAGGAGATTATACAAGATCTCTTCACCATGACTGAAgttgcgcagaatgct
gatgctccagcacagaaggctgaaggttcgAAGAAGAATTTCATATGCGGCGGAATGTTC
AGGGAGTTGCTTATGTCATTCTACAGTAAGAATGCTAAACGTAAGCCTCAAAGGATAATA
TTTTACAGGGATGGTGTAAGTGATGGACAATTCCTTCATGTTCTGCTCTATGAGATGGAC
GCAATCAAGAAGGCTATTGCATCTTTGGACCCAGCATACAGGCCCCTGGTGACATTTGTG

GTTGTCCAGAAGAGGCACCACACAAGGCTCTTCCCTGAGGTGCATGGAAGGCAAGATCTG
ACGGACAGAAGTGGAAATGTTCGTCCAGGAACCGTGGTTGACACTAACATTTGCCACCCT
AGTGAGTTCGACTTCTACCTGTGCAGCCATGCCGGAATCCAGGGAACAAGCAGACCAACC
CACTACCACGTTCTCCATGACGAGAACCGTTTTAGTGCCGATCAGCTGCAGATGCTCACT
TACAATTTGTGTTACACCTACGCTCGATGCACCCGGTCTGTCTCTGTTGTCCCTCCAGCC
TACTACGCTCACCTGGCAGCATTCCGGGCGAGGTACTACGATGAG---------------

>Vvinifera_GSVIVT00031923001
ATATCTTCTGGTCGGAGCTTTTACCAATTTAAAGACTCAGGAAAAGACGAGCTTGGATAT
GGTATTATAGCTTCTAGAGGATTTCAACATAGTCTCAAACCCACTGCCCAGGGTCTATCC
TTGTGCTTGGACTACTCAGTTGTGCCATTTTTTAATCCAATTTCGGTTTTAGAGTTCCTA
AAGGAGCATGTTCGTGGCTTCTCCTTACGAGAGTTTAAGAGATACAGGAGTAAGGTTGAG
GCTACCTTAAAGGGATTGAAAGTTAGAGTGACTCACCGTAATACAGGTCAAAAATTCATT
ATTGCAGGTTTAACTAGTCAAGACACGCAAAATCTGTCATTTCTTGCTGAAGATCCAGAA
CGCAAAGTTTTGTCAAAGAAAGTAATGCTTGTTGATTATTTCTATGAAAAGTATGGCAAG
GATATTGTGCACAAGGATATTCCCTGCTTAGATGTGGAAAAAACAATAGGAATAACTAT
GTACCAATGGAATTCTGCACTTTGGTTGAGGGGCAGAGGTATACAAAAGAGATTTTGGAT
AAAGATGCTGCTCAGGGGCTGAAACGTGAGCAACTTCCTCAAATTCTTGTGTGTGTCATG
GCTAGGAGGGATGCTGGCTATGGATATCTTAAGTGGTTCTCTGAGACCAGATTGGGGATG
GTTACTCAGTGTTGTTTGTCCAGCCCCGCCAACAAAGCCAGTGATCAGTATCTTGCCAAT
CTTGCTCTCAAGTTGAATGCTAAGCTAGGGGGCAGCAATGTAGAGCTTATAGAACGACTT
CCACGGTTTGAAGGTGAAGGACATGTGATGTTTATTGGTGCTGATGTCAATCACCCAGGC
TCTCAGAACACAACTAGTCCATCAATAGCAGCTGTTGTTGCCACAGTGAATTGGCCTGCA
GCAAACCGCTATGCAGCTCGAATTCGCCCACAAGCCCATCGAATGGAGAAGATTCAGAAT
TTTGGGGCAATGTGCCTGGAGCTTGTTGAGGCTTATGTTCAGGCAAATAAAGTCAAGCCA
GAGAAGATCGTGGTGTTCCGTGATGGTGTAAGTGAGGGCCAATTTGACATGGTTCTGAAC
GAAGAATTACTTGATCTCAAGAGAGCAATCCAGGGGGGAAATTACTGCCCGACCATCACT
CTTATTGTGGCCCGGAAGAGACACCTAACCCGGTTGTTTCCTAAGGTAAATGATCGGAGC
TTTAATGGGAATGTGCCTCCAGGCACTGTTGTGGACACAACAGTGGTCCACCTATCTGAG
TTCGACTTCTATCTTTGCAGCCACTATGTAAGCATTCCTTGCACAACTCGAGCT------

>Mguttatus_mgf015766m
CTTGCCGTTCACCAATCGTTCTTTTTGAACGATACGAAGAATTTAATCGACTTAGGTGGC
GGCATCCATGGATGCAGAGGATTCTTTTCTAGCTTTAAAGCTCTTCAAGGTGGAATGTAT
TTGAACCATGATGTATGTATGACCACAATTGTACAATCGGGTTCGGTGGTGGATTTTCTT
ATTCGCAATCAAAATGTGAAAACTCCGTTCGAAATCGACTGGAATAAGGCTAGAGGGACG
CTAAAAAACTTGAGGTTTAAAGTAAATCACTTGAAATTCGAGTTCAAAATCACGGGATTT
AGCGACAGGCCTTGCAAAGAACAGAAATTCTCTCTGAAGCTGggaGAAGCAAACGTTGAG
ACTACGGTTTACGATTATTTTGTGAAGACGCGTGGAATTAAGCTGAGTTTTAGTGCAAAC
TTACCATGCATCAACGCTGGGAAGCCTCGTAAACCGAAATTCTTTCCAATAGAGCTTTGT

TCGTTGGTTCCTTTGCAACGTTACAAGAACGAGTTAACCGGTTATCAAACTTCTGCAATG
ATTAAAAAATCAAGCCAAAAATTCATTCTTTGCCTCCTTTCCGATAAGAAATTCTGCGAC
TTATACGGTCCGTGGAAAAAGAAGACCATTGTAGAATTCGGAATTCTCGATCAATGCGTT
TCGAATAATAAAATCGACGAGAAGTATCTCATGAATCTCATGCTCAAGATAAACGCAAAG
CTCGGTGGTTTTAATCATACTATTCTTTCGGAAATAACGAGAAGTATCCCATTTGTTAGT
AAGATTCCTACAATGATATTTGGAATGGAAGTTTCCCACGCTTCTCCGGGGGCCACGGAG
AGACCTTCCATTGCTTCCGTTGTCGGATCAAGAGAGTGGCCGAGAATTTCGAGTTATAGA
GCGTGCCTTCGTGCACTCCCGCCGAAAGTTAAAATGATTGATTCGCTTTTTAAGCCAATT
TCTGAACAAAAAGATGCTGGCATATTCAGTGAGTTGCTGTTGGAGTTTTTCGCGAGTTCC
GGGAATAAAAAACCAGCTCAAATTATCATATTTAGGAATGGATTGAGCACAACAGAGTAT
AAACAAATTGTGAAGGAAGAAATGGATCAGATACTTAAGGCATGTAATTTTCTCGAGGAG
AAATGGCGTCCGAAATTCACAGTGATCGTCTCACAGAGACGACATCACACTAAATTCTTC
GATAAATCCaatTCGGGAGCTAATGTTTCTCCAGGAACTATTGTTGACAACAAAGTTTGT
GATCTTCAATGTAATAACTTCTACATGAATGCTCATGCTGCAAGAATAGGGACTCCGAGG
CCGACACATTACCATGTGCTGTTAGATGAAATAGGTTTCTCGTCGGATGATCTGCAAGAA
GTTATTCATTCTTTATCATACGTTTTCCAGAGAAGCAACAACGCGATTTCCGAAGTTGCT
CCAGTTCGCTATGCTCGATTAGCAGCTGCTAAGATATCACAAGTAATTAAA---------

>CL450_sinensis
CTCCTAGTTCGCCAATCGTTCTTTCACAATGATCCAAAAAATTTTGCTGATGTCGGAGGG
GGAGTTCTTGGCTGCAGAGGATTCCATTCAAGTTTTAGGACCACTCAGGGAGGCTTGTCT
TTGAATATTGATGTATCAACTACCATGATAATTCAGCCGGGTCCAGTAGTTGATTTTCTT
ATTGCCAACCAAAATGTTAGAGATCCCTTTTCAATTGACTGGGCCAAGGCTAAACGGACA
CTAAAAAATCTGAGGATTAAGACGATTACCTCCAATCAAGAGTACAAGATAACTGGACTG
AGTGAGAAACTGTGTAAAGAGCAAATGTTTTCACTGAAGCAGAAGAATGTTAAGGATGAT
GATGGTGAAGTGCAGGAACTGGAAATTACTGTTTACGACTATTTTGTTAATAATCGCAAT
ATAGATTTGCGATATTCTGGAGATCTACCATGCATCAATGTTGGTAAACCAAAGCGGCCG
ACCTATATTCCTCTTGAGCTTTGTGAATTGGTGTCCTTACAACGTTACACGAAAGCCCTG
ACCAATCTACAGAGAGCATCACTAGTGGAGAAGTCAAGGCAGAAGTTCCTTCTTTGTTTG
CTTCCTGAGAGGAAAAACTCTGATTTATACGGTCCTTGGAAACGAAAGAATCTTGCCGAC
TTTGGAATTGTCACTCAGTGTATGGCTCCTATGAGGGTCAATGACCAGTATCTTACAAAT
GTTCTCCTTAAGATTAATGCTAAGCTTGGTGGATTAAATTCATTGTTGGCTGTTGAACAT
TCCCCTTCAATTCCTATTGTTTCGAAGGTTCCCACCATCATCCTTGGGATGGATGTATCC
CATGGGTCTCCTGGACATTCAGATATACCATCTATTGCTGCGGTAGTCAGTTCCAGGCAC
TGGCCCTTAATATCTCGCTACAGAGCAGCTGTGCGTACACAGTCTCCAAAAGTCGAAATG
ATTGATTCTTTGTTCAAGAAAGTATCTGACACTGAGGATGAAGGGATAATAAGAGAGCTT
TTGTTGGACTTCTACACTAGCTCAGGGAAAAGGAAACCTGAACAGATCATCATATTCAGG
GATGGTGTCAGTGAATCACAGTTTAATCAAGTCTTGAACGTTGAACTGAATCAGATTATA
GAGGCGTGCAAGTTTCTGGATGAGAAGTGGTCCCCAAAGTTTGCGGTGATTGTTGCACAG
AAGAATCACCATACAAAATTTTTCCAGTCGGGATCTCCTGACAATGTTCCTCCTGGAACT
GTTGTTGACAACAAAGTCTGTCATCCAAGAAACTATGACTTCTACCTTTGTGCCCATGCT
GGAATGATTGGTACTTCAAGGCCAACACATTACCACGTTCTATTTGATGAAATTGGCTTT
TCAAGTGATGAGCTGCAGGAACTAGTGCACTCACTTTCTTATGTGTACCAGAGAAGCACT
ACAGCCATTTCTGTAGTTGCTCCAATTTGCTATGCCCACTTGGCAGCAAGCCAGGTAGGA
TCATTCATGAAG--

>contig12732_taeda
AAGGGGTTGAAAATTAAAGTTACCCACAGACTGTGTTCTCAGAAATTCACAATAGTAGGA
CTGACAAAACAGGTGACTACGGAAATCAAATTTCTCATGACCGAGGACAATGGCACTCAG
CGAATGGTTACTATGGTGGAATTTTTCAGGATAAAGCATGGATGTAAAATTGCTTTCCAG
CTGCTGCCTTGCCTGGACATCAGTAAAAGCAAAGAAAAACCGAATTATGTTCCCATGGAG
TTTTGTATGATCTGTGAAGGCCAAAGGTTTCCTCGAGATAATTTGAACGGTTACCAGTCA
AAGAATTTTAGTCGAATTGCCtgcCAAATTCAAATTTTAATAATTGCTATGGAGGAGAAA
CATCCAGGGTATAATACTCTGAAGCTTATTGCAGAAACAGATGTCGGATTGGTGACGCAG
TGTTGTTTGTTTGATCATGTCAAAAAATGCTCAgatcgggcaacacaatctcaggcaTCA
CAATATCTGGCTAATTTGGCATTGAAGATAAATGCCAAAGTGGGGGGAAGTAACGCGGCG
CTTCTTCATAGCTTGTCTCGCCAGTTACCGAGATTTGGCAGCGACCATGTCATGTACATT
GGCGCAGATGTAAATCATCCTGGATCGGGGGATAGTACGAGTCCTTCCATTGCTGCAGTG
GTTGGAAGCATCAACTGGCCCTGGTGCAATCGCTATAATGCGAGAGTCAGCTATCAGAGT
CACAGGGTGGAACACATCCAACAACTCGAGGAGATGTCTAAGGAATTACTGGACGATTAT
TTCAAAGCAAACAAGAAGCTGCCTGATAGGATACTCTTCTTCAGAGATGGTGTGAGTGAG
AGTCAATTCGATATGGTTCTCAACCAAGAATTAGAAGCCCTCAGAGAAGCAGTAGCCCAC
TTCAAAAACTACAATCCTCCTGTTTCCTTCATAGTAGCTCAGAAGAGACATCACACCAGA
CTGTTCCTTGGTGATGGAGAGCAACGGACGAAGTCTGGCAATGTTCCTCCTGGCACTGTG
GTGGACACAGTCATTGTTCATCCACGACAGTTTGATTTCTATCTGTGCAGCCATAATGGG
CTTCTGGGCACTAGCAAGCCCACTCATTACCATGTTCTCTGGGATGATAACCGCTTCAGT
TCTGATGAGCTGGAAACCTTGATTAACAATCTTTGTTATACATTTGCAAGATGCACAAAG
CCAGTGTCATTGGCCCCCCCTGTGTATTATGCAGATCTTGCGGCTTATCGGGGTCGATTA
TACTTGCAG---

>Gmax_Glyma02g12430
ATACCTGTAGGGAGGTCATTCTATTCAAGTTCAATGGGAAGAAGCAAAGACATTGGTGGA
GGAGCTGTTGGATTGAGAGGCTTCTTTCAGAGTCTTAGACCAACACAACAAGGACTTGCT
CTCAACGTGGATTTCTCAGTAACTGCTTTCCATGAGAGCCAGAAGCGCCTCGAGTTTCTT
CGAGACCTGTCTCAAAGAAAGACAGCTCAATTAACCGGTGAAGAGAGGAAGGAAGTGGAG
AAGGCATTGAAGAACATCAGGGTCTTCGTTTGCCATAGAGAAACTGTTCAACGATATCGT
GTCTATGGCTTGACTGAGGAGGTTACTGAAAATCTTTGGTTTGCTGACAGAGATGGGAAG
AATCTGAGGTTGGTGAATTACTTTAAAGATCAGTATAACTATGACATACAATTCAGAAAA
CTGCCATGCTTGCAAATTAGTAGGAGTAAGCCTTGTTATCTCCCTATGGAGCTTTGTGTG
ATCTGTGAAGGCCAGAAGTTCCTTGGGAAACTGTCTGATGATCAAACAGCAAGAATACTC
AAAATGGGCTGCCAAAGACAGCTTCTTATTTGCATAATGGAGAGAAAACACAAAGGGTAT
GCAGACTTGAAACGAATTGCTGAGACAAGTGTTGGTGTTGTGAGCCAATGCTGCCTGTAC
CCCAATCTCAACAAGTTGAGTTCACAATTTTTGGCCAATTTGGCCCTCAAAATCAACGCC
AAAGTTGGTGGATGCACAGTTGCCTTGTACAACTCATTGCCATCGCAGTTACCGCGCCTC
TTTCATATTGACGAGCCGGTGATATTCATGGGTGCAGATGTGACACATCCTCACCCTCTA
GATGATGTCAGTCCATCTGTCGCTGCTGTCGTCGGCAGCATGAATTGGCCAACAGCAAAC
AAGTACATTTCAAGAATAAGGTCTCAAACACATAGACAAGAAATCATCCTGGACCTAGGC
GCAATGGTGGGGGAGTTGCTTGATGATTTTTACCAGGAGGTAGAGAAACTCCCTAACAGA
ATCATTTTCTTCCGAGACGGGGTCAGTGAAACTCAGTTTTACAAAGTGCTGGAAGAGGAA

CTTCAATCCATCAGGTGTGCATGCTCAAGGTTTCCTGGCTACAAACCTACCATTACTTTT
GCAGTTGTGCAAAAGAGGCATCACACAAGGTTGTTTCCCTTTGAAACTGACCAGTCTTCA
ACTCAAaaaAACAATTTTCTATATGAAAACATTCCTCCAGGGACTGTGGTTGATTCTGTG
ATCACTCATCCAAAGGAGTTTGATTTCTATCTTTGTAGCCATTGGGGTGTTAAAGGAACA
AGTAGGCCAACTCACTACCATGTCTTGTGGGATGAAAACCAGTTTACTTCTGATGAACTT
CAGAAACTGGTTTACAACTTGTGCTACACTTTTGTTAGGTGTACCAAGCCAATTTCTTTG
GTGCCTCCTGCATATTATGCACATTTGGCTGCATATAGAGGCAGACTCTACCTTGAG---

>Ptrichocarpa_POPTR_0008s15860
TGCCCTGTCGGAAGATCCTTCTTTTCACCTGATATAAGAGCACCACAACGACTTGGTGAT
GGCTTAGAGTCCTGGTGTGGATTTTACCAGAGTATAAGGCCTACTCAAATGGGACTGTCC
TTAAATATTGATATGGCCTCAGCTGCTTTCATCGAGCCTCTTCCTGTGATAGAGTTCGTT
GCCCAGCTTCTAGGCAAAGATATATTATCAAGGCCATTGTCTGATTCTGATCGAGTTAAG
ATTAAGAAGGGTCTCAGAGGAGTGAAAGTTGAAGTTACTCACAGAGGGAGTGTACGAAGA
AAGTACCGTGTCTCAGGATTGACATCTCAGCCTACAAGAGAACTTGTGTTTCCTGTTGAT
GATAACTCGACCATGAAGTCAGTTGTTGAATACTTCCAGGAGATGTATGGCTTTACCATT
CAACATACACATCTACCTTGCCTTCAGGTTGGAAACCAGAAGAAAGCAAACTATCTACCT
ATGGAGGCTTGCAAAATTGTGGAGGGGCAGCGTTATACAAAAAGGTTGAATGAGAGGCAA
ATTACTGCCCTCTTAAGAGTTACATGCCAAAGACTTCTCTTAGCTATTCTACCTGACAAC
AATGGGTCCCTCTATGGTGATCTGAAGCGAATATGTGAAACTGATCTTGGTTTAATAACT
CAATGTTGTCTCTCAAAACACGTTTTCAAGATCAGTAAGCAGTACTTGGCAAACCTGTCG
CTCAAGATCAATGTAAAGATGGGTGGTAGGAATACCGTCCTTTTAGATGCTATAAGCTGC
AGAATACCATTAGTTAGCGACATACCGACAATTATTTTGGAGCAGATGTGACTCACCCA
GAGAATGGAGAGGACTCAAGCCCCTCGATTGCTGCTGTGGTAGCTTCTCAGGACTGGCCT
GAAGTAACAAAATACGCTGGATTGGTATGTGCTCAAGCTCACAGACAGGAACTCATACAG
GACTTATACAAAACATGGCAAGATCCTGTTCGTGGTACTGTAAGTGGTGGCATGATCAGA
GATCTCCTGATTTCTTTCAGGAAAGCAACGGGGCAAAAGCCGCTAAGGATCATATTTTAT
AGGGATGGTGTAAGTGAAGGGCAATTTTATCAAGTTCTGCTTTATGAGTTGGATGCAATT
CGGAAGGCCTGCGCTTCTTTGGAGCCAAATTATCAGCCACCGGTGACTTTCATTGTTGTA
CAAAAACGTCACCACACAAGATTGTTTGCTAACAACCATAGGGACAGGAATAGCACAGAC
AAGAGTGGAAACATATTACCTGGCACTGTGGTTGATTCTAAAATCTGTCATCCAACGGAA
TTTGACTTTTATCTCTGTAGCCATGCTGGTATTCAGGGAACAAGTAGACCAGCACACTAT
CATGTGTTGTGGGATGAGAACAATTTCACAGCTGATGGAATCCAGTCCTTGACAAACAAT
CTCTGTTACACATACGCAAGGTGCACGCGGTCTGTTTCAGTGGTTCCACCCGCATACTAT
GCACATTTAGCTGCTTTTCGTGCTCGATTTTACACGGAG---------------------

>Mtruncatula_Medtr5g045600
ATACCTGTTGGAAGGTCATTCTATTCGAGTTCAATGGGGAGAAGCAAAGATATCGGTGGA
GGAGCTGTTGGATTGAGAGGCTTCTTTCAGAGTCTTAGACCAACACAACAAGGACTTGCT
CTCAATGTGGATTTCTCGGTAACTGCTTTCCATGAGAGTCAGAAACGTCTCGAGTTTCTT
AGAGACCTTTCTCAGAGGCAGACAACTCAGCTAACTTGTGAAGAAAGGAAGGAAGTAGAG
AAAACGTTGAAGAATATCAGAGTCTTTGTTTGCCATAGAGAAACTGTTCAGAGATACCGT
GTCTATGGCTTAACTGAGGAGGCAACTGAAAATCTTTGGTTTCCTGATAGAGATGGAAAG

AATCTGAGGCTTATGAGTTACTTTAAAGATCACTATAACTACGACATTCAATTCAGGAAG
TGGCCATGCTTGCAAATTAGTAGAAGTAAACCTTGTTATCTCCCTATGGAGCTTTGTGTG
ATCTGTGAAGGACAGAAGTTCCTTGGGAAACTGTCGGATGATCAGACGGCAAAAATACTC
AAGATGGGCTGTCAAAGACAGCTTCTTATTTGTATAATGGAGAAAAAACACAAAGGGTAT
GCAGATTTGAAAAGAATTGCCGAGACAAGTGTCGGTGTTGTAAGCCAGTGCTGCTTGTAT
CCGAATCTCATCAAATTAAGTTCACAGTTTTTGGCTAATTTGGCTCTCAAGATCAATGCT
AAAGTTGGTGGATGCACTGTTGCTTTGTACAACTCGCTTCCTTCTCAATTACCGCGTCTT
TTCAACATCGACGAGCCGGTGATGTTCATGGGAGCCGACGTCACGCATCCTCATCCGCTC
GACGATTCAAGTCCATCTGTCGCTGCTGTTGTTGGTAGCATGAACTGGCCAACAGCAAAC
AAATATATTTCAAGAATAAGGTCTCAAACACACAGGCAAGAAATTATTGCAGATCTCGGT
GCAATGGTAGGAGAATTGCTTGAAGATTTTTATCAAGAAGTGGAAAAACTCCCAAACCGA
ATAATTTTCTTCCGAGACGGTGTTAGCGAAACTCAGTTTTACAAAGTTCTGCAAGAGGAA
CTACAATCAATAAAACAAGCATGTTCATCAAGGTTTCATGGTTATAAACCTTTTATTACT
TTTGTAGTTGTGCAAAAGAGGCATCACACAAGGTTGTTTCCTGCCGACACCGATCAAtct
tcgatgcacaacaattttCACTTTCAATATGAAAATATTCCACCAGGGACTGTGGTTGAT
TCAGTGATTACTCATCCAAAGGAATTTGATTTCTATCTATGTAGTCATTGGGGTGTGAAG
GGAACAAGTAGGCCAACTCATTACCATGTCTTGTTGGATGAAAATAAGTTTACTTCAGAT
GAACTGCAAAAGCTTGTTTACAATTTGTGTTTTACTTTTGTTAGATGTACTAAGCCAATT
TCATTAGTTCCTCCTGCATATTATGCACATTTAGCTGCATATAGAGGTAGACTCTACCTT
GAG---

>LOC_Os03g47820
GTATCTATCTCAAGGTCATTTTTCTCCCAATCATTTGGGCATGGTGGCGAGATTGGTAGT
GGTACAGAGTGTTGGAGGGGGTATTACCAAAGTCTGCGCCCCACGCAGATGGGACTGTCG
CTAAATATCGATATTTCTGCAACAGCATTTTACAAGGCCCAGCCAGTGATGGACTTTGCA
GTTCAGTATTTGAACATCCGAGATGTTTCAAGGCGCTTGTCTGATCAAGATCGTATTAAA
CTGAAAAAGGCCCTAAAAGGAGTCCAAATTGTGGCAACTCACTGGAAAGAGAAATCCATA
CGTTACAAGATCACTGGAATACCCTCAGCTCCAATGAATGAATTGATGTTTGATCTAGAT
GGAAACAGGATATCAGTCGTCCAATACTTTAAGAAACAGTACAATTACTCCTTAAAACAT
GTTAATTGGCCATGCCTTCAAGCTGGCAGTGATAGCAGACCAAAATATTTGCCTATGGAG
GTCTGCAGTATACTTGAAGGACAACGGTATTCGAAGAAGCTAAATGAGCATCAAGTTACA
AACATTCTAAGGATGACATGTGAACGACAGTTATTAATTGTAATATTGACTGAAATTAGT
GGTTCTTATGGAAGGATAAAACGAATTTGTGAAACAGAGGTTGGTGTGATAACTCAATGC
TGTGCACCCAAGAGTCTTCAGAAAGGTGGGAAACAGTATCTTGAAAATCTGGCCCTAAAA
ATGAATGTTAAGGTCGGGGGGCGAAATACAGTACTTGAAGATGCGTTGCACAAGAAAATA
CCAATTCTGACAGATCGTCCTACGATAGTGTTTGGAGCGGATGTTACACATCCATCTCCT
GGGGAGGATGCTTCTCCATCCATTGCAGCGGTTGTTGCATCCATGGATTGGCCAGAAGTT
ACAAAGTACAAATGCTTGGTATCTACACAAAGTCATAGGGAAGAAATCATATCTAATCTT
TACACAGAAGTGAAAGATCCATTGAAGGGAATTATTAGAGGTGGAATGATTAGGGAGTTG
CTCAGGTCTTTCTACCAAGAAACTGGACAGAAACCTAGCCGGATTATATTTTATCGAGAT
GGTATCAGCGAGGGACAGTTTAGCCAAGTGTTGCTTTATGAAATGGATGCAATTCGCAAG
GCATGTGCTAGTTTGCAGGAGGGCTACCTTCCTCCAGTTACATTTGTTGTTGTCCAGAAA
AGGCATCACACCCGCCTATTTCCAGAAAATCGTCGAGATATGATGGATAGAAGCGGAAAT
ATCCTTCCTGGAACTGTTGTTGACACAATGATCTGCCATCCGAGTGAGTTTGACTTTTAC
CTCTGTAGCCATTCTGGTATCAAGGGGACGAGCCGTCCAACACATTATCATGTTCTTTTA
GATGAAAATGGGTTCAAGGCTGATACGCTGCAAACCCTAACCTACAATCTTAGCTACACC
TATGCCCGATGCACCCGAGCAGTCTCCATAGTTCCTCCAGCATACTATGCGCATCTGGGA
GCCTTCCGAGCACGCTACTACATGGAG---------------------------------

>Alyrata_16062541_locus
CTCCTTGTTCGCCAGTCTTTTTCCACAATGACGTAAAGAACTTTGTACCTATTGGTGGA
GGTGTCAGTGGTTGCAGAGGGTTCCATTCAAGTTTCAGAACTACTCAGGGAGGCTTATCC
CTGAATATTGACACTTCAACTACGATGATTGTACAACCTGGACCTGTTGTTGATTTCCTG
CTTGCAAACCAGAACAAGAAAGATCCATACGGTGTGGACTGGAACAAGGCTCGTCGTGTT
CTCAAGAATCTGAGAGTTCAAGTTACTCTTTCAAATAGAGAATACAAGATAAGCGGGCTA
AGTGAACACAGCTGCAAAGATCAAATGTTTACATGGAGGAAACCTAACGACAAGGGGGAA
TTTGAGGAGGTTGAGATCACAGTGCTCAATTACTATAAAGAGCGTAATATTGAAGTGCGA
TATTCAGGTGACTTCCCTTGCATCAATGTTGGTAAGCCGAAGCGTCCCACTTACTTCCCC
ATTGAGTTCTGTAATCTTGTGTCTCTTCAGCGATACACAAAATCGCTTACCAATTTTCAG
AGGGCTGCACTAGTAGAAAAGTCTAGGCAGAAGTTCCTACTTTGCATACTCTCAGAAAGG
AAAAACTCTGATGTTTATGGCCCTTGGAAAAAAAAGAATCTTGTTGATCTTGGAATTGTG
ACTCAGTGCATTGCTCCCACAAGAGTGAACGATCAGTATCTCACCAATGTTCTCCTGAAG
ATAAATGCCAAGCTTGGTGGATTGAATTCGCTGTTAGCAATAGAGCGCTCACCAGCAATG
CCAAAAGTAACGCAAGTTCCTACTATCATTGTTGGGATGGATGTATCCCATGGTTCCCCT
GGCCAGTCTGATATACCATCAATTGCAGCGGTTGTGAGCTCCAGACAATGGCCACTCATC
TCAAAATATAAGGCATGTGTACGCACACAATCGCGCAAAATGGAAATGATTGATAATCTC
TTCAAACCCGTCTCTGGCAAAGACGAAGGAATGTTCAGGGAGCTGTTGCTCGACTTTTAC
TACAGTTCTGAGAAGAGGAAACCAGAGCACATCATTATATTCAGGGATGGTGTGAGTGAG
TCTCAGTTCAATCAAGTTCTTAATATTGAATTGGATCAGATGATGCAGGCATGCAAGTTT
CTTGACGAACACTGGAATCCAAAGTTTACAGTGATCGTTGCCCAGAAGAACCACCACACC
AAGTTCTTCCAGTCGAGTCGCCCTGATAATGTTCCTCCAGGAACAATAATTGACAGCCAG
ATCTGTCACCCACGCAACTTTGATTTCTATCTCTGCGCCCATGCCGGCATGATTGGAACT
ACAAGGCCAACACATTACCATGTGCTGTATGACGAGATTGGATTTGCCACAGATGACCTT
CAAGAACTTGTGCATTCTCTATCCTATGTCTACCAGAGGAGCACCACTGCGATCTCAGTC
GTTGCACCAGTTTGTTATGCGCATTTGGCAGCTGCACAGATGGGAACTGTGATGAAG---

>Bdistachyon_Bradi4g08590
CTGCTTGTAAGACAATCATTTTTCAGCGATGACAATAGGAACTTAGTTGATTTAACTGGT
GGTGTCAGTGGATGTCGTGGACTTCACTCTAGTTTCCGTACTACTATGGGTGGTTTGTCT
TTAAATATGGATGTTTCAACTACGATGATTGTGACTCCTCCTGTTGTTCACTTCCTCCTT
ACAAATCAAAATGTTAGAGATGTTCAAGATCTTGACTGGCCCAAGATTTGTAGAACTTGT
CACAAAATTctagttactttttgCAGGCCAAAAAAATGTTGAAGAATCTGAGAGTTAAA
GCTACGCACAACAACATGGAATTCAAGATCATTGGTCTTAGTGATCAACCATGTTCTAGA
CAGACATTTCCAATGAAAGTTCGGAATGGATGCACTGAAAGTCAGACTGTTGATATTACT
GTTGAGGAATATTTTAAATCCAAGGAGGTATTTTTGGCAAAGCCTTATCTGCCATGTCTT
GATGTGGGAAAACCAAAACGCCCGAATTATCTCCCAATTGAGCTAGCGAACATGGTATCA
CTTCAACGTTATACGAAGGCACTGTCTTCTCAGCAAAGAGCAACGTTGGTTGAAAAATCA
CGACAGAAATTTCTGCTGTGTGTTTTACCAGAGAGGAAGAATTGTGATATATACGGCCCT
TGGAAGAAGAAAAATCTTCATGAGATGGGTATTGTCACTCAATGCATTGTTCCTAGTAAT
AAGATGAATGATCAATACTTCACCAATGTTCTTCTAAAGATCAATGCTAAGCTGGGCGGA
ATGAACTCCAAACTGGCACTGGAACATAGCCATATGATTCCAATTGTCAATAAGAAACCG
ACATTAATTTTGGGAATGGATGTTTCACATGGTTCTCCAGGTCGATCAGATATACCATCA
ATTGCTGCTGTTGTTGGGTCTAGATGTTGGCCACTGATATCACGGTACAGGCATCTGTC

CGGACCCAGTCTCCGAAGGTAGAGATGATTGATTCACTTTTTAAGCCACTGGAGGATGGA
AAGGATGATGGTATAATAAGGGAACTTCTGTTAGACTTCTACCAAACCAGTCAACAAAGA
AAGCCAACACAGATAATCATTTTCAGGGATGGTGTGAGCGAGTCTCAATTTAGCCAAGTA
CTGAATCTTGAGGTTAATCAAATAATAAAGGCTTACCAGAATATGGGTCAGGGGGATCCT
CCAAAGGTTACAGTTATCATTGCTCAAAAGAATCATCACACAAAACTCTTCCAAGCTGAA
GCATCAGATAATGTTCCACCTGGGACTGTTGTGGACTCTGGTATTGTTCATCCAAAACAG
TATGATTTCTACATGTGTGCTCATGCGGGACCTATAGGTACCTCAAGGCCCACCCATTAT
CATGTCTTGCTTGATGAGATTGGCTTCTCACCAGATGACCTCCAGAAGCTAGTTCTTTCG
CTTTCATATGTGTACCAGAGGAGCACTACTGCGATATCTGTCGTGGCACCTATCTGTTAT
GCTCACCTTGCAGCAGCGCAGATGAGCCAGTTCATGAAA---------------------

>LOC_Os02g58490
GCGCCATTTGGACGTTCCTTCTTTTCGCCTTACTTGGGTAGGAGGCAACCCCTTGGTGAG
GGATTGGAAAGCTGGCGTGGATTTTACCAGAGCATTCGGCCTACTCAGATGGGCTTATCA
CTGAATATTGATATGTCAGCTACAGCTTTCATTGAGCCGTTACCTGTTATTGATTTTGTT
GCACAACTATTGAATTCTGACATCCATTCAAGGCCGCTTTCTGATGCCGAACGTGTTAAG
ATCAAGAAAGCCTTGAGAGGAGTAAAGGTGGAAGTTACCCACCGTGGCAACATGAGGCGG
AAGTACCGGATATCTGGTTTGACAATTCAGCCAACTCGTGAGCTAACTTTTCCTGTTGAT
GAAGGAGGCACAGTGAAGTCAGTTGTACAGTACTTTCAAGAGACATATGGCTTTGCCATC
CAACACACCTACCTTCCATGCCTTACAGTTCAGCGATTAAATTACCTGCCTATGGAGGTC
TGCAAAATAGTGGAAGGACAGAGATACTCCAAGAGATTAAATCAGAATCAGATAAGAGCT
CTTTTGGAGGAGACATGCCAACACTTGCTTATTGGGATACTACCTGATAACAATGGCTCG
CTTTATGGTGATTTGAAGCGCGTGTGTGAAATAGATCTTGGAATAGTTTCACAATGCTGT
TGCACGAAGCAGGTGTTCAAAATGAACAAACAAATTCTTGCAAATCTTGCTCTGAAGATA
AATGTCAAGGTTGGGGGCAGGAACACTGTGCTGGTGGATGCTGTGTCAAGGCGTATTCCT
CTGGTAACCGACAGACCTACAATTATATTTGGTGCTGATGTTACCCATCCTCATCCTGGA
GAGGACAGCAGTCCCTCAATTGCTGCTGTTGTAGCCTCCCAAGATTGGCCTGAGGTGACA
AAGTATGCTGGGTTGGTTTCTGCTCAAGCCCACCGACAAGAGCTGATAGAAGATCTATAT
AAAATCTGGCAGGATCCACAGAGAGGAACAGTTAGTGGTGGCATGATCCGTGAGCTGCTT
ATATCCTTCAAAAGATCAACTGGTGAGAAGCCCCAGCGAATAATATTTACAGGGATGGC
GTTAGTGAAGGCCAATTTTACCAAGTTCTACTTTATGAATTGAATGCAATCCGAAAAGCA
TGTGCCTCCCTGGAGACAAATTACCAACCAAAGGTGACTTTCATTGTGGTTCAGAAACGT
CACCACACAAGATTATTTGCACATAATCACAACGATCAGAACTCAGTTGACAGGAGCGGG
AACATACTCCCTGGTACGGTTGTAGATTCAAAGATCTGTCATCCAACTGAGTTTGACTTC
TACCTGTGTAGCCATGCTGGCATTAAGGGTACTAGTCGTCCAGCTCATTATCATGTCTTG
TGGGATGAAAACAACTTCACAGCTGATGCATTGCAGATTCTTACCAACAACCTTTGCTAC
ACCTATGCAAGGTGCACTCGCTCTGTATCAATTGTTCCACCTGCTTATTATGCTCATCTG
GCTGCCTTCCGTGCTCGTTTCTATATGGAA------------------------------

>AT1G31280.1
ATCACTGTTGGTAAAAGCTTTTTCACTCGTGAAACTGAGCCAGATGAAGATTTTCGTTTC
GGGGTTATAGCTGCGAAAGGGTATCGCCACACTCTGAAGCCCACAGCACAAGGTTTGTCT
TTGTGTTTGGATTACTCGGTGTTGGCGTTCCGCAAAGCAATGTCGGTCATTGAATACCTG
AAGTTGTACTTTAACTGGTCTGATATGCGTCAGTTTAGGAGGCGTGATGTGGAAGAGGAA

TTGATTGGTTTGAAAGTCACTGTCAATCATCGGAAGAACAAGCAGAAACTCACCATTGTA
GGGCTGAGTATGCAAAACACAAAAGACATCAAATTTGATCTTATTGATCAAGAGGGAAAC
GAGCCGCCAAGGAAGACGTCCATTGTTGAGTATTTCAGGATAAAGTATGGAAGACACATT
GTTCACAAGGATATACCTTGCTTGGATTTGGGAAAAAACGGTAGGCAAAATTTTGTGCCC
ATGGAATTCTGTGACTTGGTTGAGGGACAGATATATCCAAAGGATAACTTGGATAAAGAT
TCAGCTTTGTGGCTAAAAAAGTTGTCACTGGTCACTCTTGTTCTGTGTGCTATGTCTCGG
AAAGACGATGGCTATAAGACTCTGAAATGGATAGCCGAGACCAAACTTGGTCTGGTGACT
CAGTGTTTCTTGACTGGTCCTGCCACTAAAGGAGGTGATCAGTACCGGGCAAATCTTGCC
CTCAAGATGAACGCAAAGGTTGGTGGAAGCAATGTCGAGCTTATGGATACTTTCTCTTTC
TTCAAAAAAGAGGATGAGGTCATGTTCATTGGTGCTGATGTCAATCATCCCGCTGCTCGG
GACAAGATGAGCCCGTCCATTGTTGCTGTTGTGGGAACTCTTAACTGGCCTGAAGCAAAT
CGCTATGCAGCTAGAGTCATTGCCCAGCCTCACCGCAAAGAGGAAATACAAGGATTTGGC
GACGCTTGTTTGGAGCTTGTCAAAGCTCATGTTCAGGCCACAGGGAAACGGCCTAACAAG
ATTGTGATATTCCGTGATGGTGTCAGCGATGCTCAGTTCGATATGGTTCTCAATGTGGAG
TTGCTTGATGTTAAGCTAACTTTTGAGAAGAATGGTTACAATCCAAAGATAACGGTAATC
GTAGCCCAGAAACGTCATCAAACCCGTTTCTTCCCAGCCACAAATAATGATGGAAGTGAT
AAGGGCAATGTGCCTTCAGGTACGGTTGTTGATACTAAAGTTATTCACCCGTATGAGTAT
GATTTCTACCTCTGCAGTCACCACGGAGGGATAGGGACAAGCAAACCGACTCATTACTAC
ACTCTTTGGGACGAACTTGGATTCACTTCGGATCAGGTGCAGAAGCTCATCTTCGAGATG
TGCTTCACTTTCACTCGCTGCACCAAACCCGTCTCTCTTGTTCCGCCGGTGTATTATGCT
GACATGGTTGCTTTTAGAGGAAGGATGTACCACGAG----------------------

>AGO1901_Aquilegia
TTCCTTCTCTGTATTTTGCCAGAGCGGAAAAATTGTGATATATATGGTCCATGGAAGAAG
AAAAATCTCCATGAAATGGGAATCTTCACACAGTGCCTCTCCCCAACGAAGATTAATGAT
CAGTATCTGACCAATGTACTTCTAAAGATCAACTCCAAACTTGGAGGTATGAATTCCTTA
TTGGAGGTTGAGCGTATTCCAACTCTTCCGCGTTTGAAGGAAACTCCTACCATGATCTTG
GGGATGGATGTATCTCATGGGTCTCCTGGTCAATCAGATGTTCCATCTATTGCTGCTGTT
GTTGGTTCAAGATCTTGGCCGCTGATCTCAAGATACAGGGCATCTGTAAGAACCCAATCA
CCAAAGGTGGAGATGATTGATTCTTTGTTCAAACTTCTACCAAATGGGGAGGATGATGGC
ATTATAAGGGAATTACTGGTTGAGTTTTACAAGTCAAGTCAAGGACGCAAGCCTGAGCAG
ATCATAATCTTCAGGGATGGAGTAAGCGAGTCTCAGTTTCCTCAAGTTCTGAACATTGAG
CTGGAGCAAATCGTTAAGGCAGTTGAGCATTTAGGTTATACCAGAATTCCGAAGATTACA
TTGATTGTGGCTCAAAAGAACCATCACACAAAGCTTTTCCAATCCAACGACCCAAACAAT
GTTCCTCCTGGAACTGTGGTAGATACGCAGATTGTGCATCCTAGGAACTATGACTTTTAC
ATGTGTGCTCACAATGGAGCAATTGGGACATCCCGTCCAGCACACTACCATGTCTTGCTT
GATGAGATTGGCTTCTCTGCTGATGAATTGCAGAATCTTGTGCACTCGCTTTCCTATGTG
TATCAGCGCAGCACAACTGCTATTTCAATTGTGGCACCTATCTGCTATGCTCACCTTGCT
GCTGCACAGATGGGGCAGTTCATCAAG---------------------------------

>Bdistachyon_Bradi1g16060
GTTCTGGTAGGCCGGTCTTTGTATCCACGCTCCATGGGAGAAGCAAAGGATATTGGTGGT
GGGGCTGTTGGTTTAAGAGGTTTCTTTCAGAGCTTGAGGCCAACCAAGCAAGGTCTTGCA
CTTAATGTTGATCTCTCACTCACAGCATTCCATGAGAGCCAGAAGCGGTGTGACTTCTTG
AAAGACCTTCCGCATGACAAAAATAGGGCTTTGGCACAAGAGGAGAGGAGGGACGTCGAG
AAAGCACTGAGAAACATTAGGGTTTTTGTGTGCCACCGTGAGACCAACCAAAGGTACCAC
GTGCATAGCTTGACCGAGGAGACAACAGAAAATCTCAAGTTTCGAGACCGAAGTGGGAAA
GACCTTATGGTGATGGATTACTTCAAGGAGCAATACAATCATGATATCCAATTCAGGAAC
CTGCCATGCTTGCAGATCGGTAGAAGCAAACCATGTTATGTGCCAATGGAGCTTTGTGTA
GTTTGTGAGGGTCAGAAGTTTCTTGGCAAGCTCTCAGATGAACAAACATCCAAGATTCTC
AAGATGGGTTGCCAAAGATTGCTAATATGTGTCATGGAACGGAGGCACCGAGGCTATGCT
GATTTGAAGCGAATTGCTGAAACATCCATTGGTGTTGTGACACAGTGTTGCCTTTACCCC
AACTTAAGCAAGCTGACCGTGCAATTTGTGGCCAATTTAGCCCTGAAGATGAATGCTAAG
CTTGGTGGATGCAATGTTTCCCTCTACAACAGCTTGCCATGCCAAATTCCTAGAATATTT
TCAGATGATGAACCAGTGATGTTCATGGGTGCTGATGTTACACACCCACATCCACTTGAC
GACTCGAGCCCATCAGTGGTTGCTGTAGTTGCAAGCATGAATTGGCCTTCAGCAAATAAG
TACATCTCCAGAATGAGGTCGCAGACGCACCGGAAAGAAATAATTGAACACCTGGATGTG
ATGGCTGGTGAACTACTTGAAGAGTTCCTGAAAGAAGTTGGCAAGCTCCCAGCTCGAATC
ATATTTTTTCGAGATGGTGTGAGCGAGACACAGTTCGACAAGGTCCTGAAGGAGGAGATG
CATGCTGTGCGAATGACATGTTCGAGGTACCCAGGCTACAAGCCCTTAATCACGTTCATC
GTTGTACAGAAGAGGCATCACACGAGGCTCTTCCACAGGGAGAAAAATGGCGGCTCGACA
CACTATTCTGATCAGAACATACCACCTGGAACGGTTGTTGACACCGTGATCACGCACCCA
AGGGAATTCGACTTCTACCTGTGCAGCCATTGGGGCACAAAGGGGACAAGCCGTCCGACT
CATTACCACATCTTGTTGGATGAGAACAAGTTTGGGTCCGACGAATTGCAGCAGCTGATA
CATAATCTTTGCTACACTTTTGTGCGGTGCACCAGGCCAGTCTCCCTTGTTCCTCCGGCA
TACTATGCGCATCTTGCTGCCTATAGAGGCAAGCTGTACCTTGAG---------------

>contig1654_taeda
aggGGCATGGATGTATCACATGGTTCTCCGGGGCATGCAGATTCACCTTCAATTTCAGCT
GTTGTTGCCTCTCGGGAATGGCCCTTGATATCCAGGTATAGAGCTTCAGTGAGAACACAG
TCACCTAAGGTTGAGATGATTGAGGCTCTGCACAAACAATCGCCCACAGGAAAGGATGTC
GGAATGATCAAAGAGCTACTTCTAGATTTCTACCAAACATGCAATCCTccaccaaacgta
GAAAGAAAGCCGCAACAAATGATCATTTTCAGAGATGGAGTCAGTGAATCGCAATTTGAC
CAGGTCTTGAATGTTGAGTTGCAGGCTATACTCAAGGCATGTAATGACATAGAGGATGGT
TATAGGCCCAAAGTTACATTGATTGTTGCGCAGAAGAATCATCACACGAAGCTGTTCCCA
ACTGGTCAAGGCAATGTGCAACCAGGGACTATTGTAGATGCTCAGATTTGTCATCCTAGA
AACTTTGATTTCTACTTGTGCCCTCAGGCTGGCCCAATAGGAACTTCACGGCCTACTCAT
TACCATGTATTACTTGATGAGAATAGTTTTACTGTGGATGATCTTCAGATTTTGGTCCAT
GCATTATCTTATGTG---

>Ppatens_1910596_locus
CTTCTTGTGAGGGATAATTTCTTTCATCCGAGCTTGGGTCCGGTTGGCAACTTGGGAGAG
GGAGTTGAGGCTTGGAGGGGTTATCACTCAAGTGTGAGGCCTACTGGGTTGGGCTTGACT
TTGAATCTCGACATGACTATGACAACGATGTTGAAGCCCGTAGAGGAATTTCTGATGGAA
AGGTTCAACGTAAGAGACCTGAATTGTTTGCAAGGCAGGGACTGGGTTAAAGCCAATAGC
GTGTTGAAAGGAGTTAGGATCGAAACGATTCACATGGAAGTGTCTCGATCTCACAAGATT
GCGGGGTTCAGTCCGCGGCCCATTAAAGACCTAAAATTCACTAAGAGGATCAAAGACGGC
GAAGGTAATGCGCGAGAGGAGGAGATGTTAGTGGAGCAATACTACTTCGATGTGTACTCG
TACACCCTTAAATACCCAGGTCTTCCAGCAATAGATGTTGGGAACAAGAAGAAGCCCACA
TTCTTGCCGTTAGAGTTGTGCAAGATAGTTGCGGGACAGCGTTACTCGAAGTCGCTGTCT
AGCAGGCAGAGGACTGCCCAGATTGCTGCATGCAAGTTCATTCTGGTCATACTATCAGAC
AAAGACAGCCCAATTTACGCTCCATTCAAGAGGTTTTGTGAGATGAAAATAGGAATTATC
TCGCAGTGCATGGTTAAGCCTAGGCAGATCAACGATCAGTATCTTGGAAATCTTGCGCTG
AAGATAAATTTGAAGATGGGAGGGTTTAATTCCCCATTGAGCCGGCGGATGCTCACCTGC
CTTGGTGAGTCAACCATAATATTTGGGATGGATGTGTCGCATGGATCCCCTGGAGATTTG
AGTGTTCCTTCGATTGCAGCTGTGGTTGCCACCAAAAACTGGCCGGAAGTGTTCCATTAT
TCGACGCAAGTTAGAACACAGCCACCCAAGATGGAGATGATAACTGGTCTTTATGAGCCT
AAGGGTGGCATGGTGAGAGAATTGCTTTTGACATATTACAACACATGCGCCAGAGGCACA
AATCCTAAACCAAGTCAAATTATCATCTACAGGGATGGAGTCAGCGAAAGCCAGTTCGCG
GAATGTTTGGAGGTAGAATTTATGGCGTTCAAAAGGGCGTGTGCGGAACTGGAAGAAGGC
TATAATCCTGGGATAACCTTCATTGTCGCTCAGAAACGTCACAACACACGTTTCTTTCCT
CAGAACCGAGATTCGTTGAAGAATGGAAACGTTCTGCCAGGTACTGTTGTCGATAAGGAT
GTGTGCCATCCTCACAACTTTGACTTCTTCCTCGTCTCTCAAGCTGGACTCATTGGTACA
TCTCGTCCGACTCACTATCATGTGCTGGTGAATGAAAACAAACTTGGGCCGGATGACATC
CAGATGTTGACCAACAACCTCTGTTACACGTTTGGACGCTGTTCGACGTCAATTTCGATG
GCGGCTCCTGCGGCATATGCCCATGTTGTGGCAGGAAGGTATCGGAAGTTGCTTGAC---

>Gmax_Glyma13g26240
CTCTTGGTAAGGCAATCTTTTTTTCATGATGACTCAAGAAATTTCAATGATGTTGGGGCT
GGTGTAACAGCAGTCTCAGGTTTTCATTCCAGTTTTCGTTCCACACAGCGAGGATTGTCT

CTCAATATTGACGTGTCAACAACTATTATCATAAAACCTGGACCTGTAATTGATTTTCTG
TTATCAAATCAGCAAGTGAAGGAACCCCGTTATATTGACTGGGAAAAGGCGAAAAAAATG
CTTAAAAATTTAAGGGTGCAAGCAACTCATCATAACCAGGAATTTAAAATTTCTGGGTTG
AGTGAGAAACCATGCATTCAGCAACTTTTTAGTATGAAGGTTAAAAATGATGACaacAAT
AGCAGAGGGCAGACAGTGGATATTACTGTTTATGAATATTTTGCCAAACACTGTGGCATA
GAGTTGACCTCATCTGCTTATCTTCCATGTCTTGATGTTGGGAAACCAAAACGTCCCGTC
TATCTTCCCTTGGAGCTATGTTCACTTGTTTCCCTCCAACGGTATACAAAGGTTTTATCT
CTGATGCAAAGAGCATCTTTAGTTGAAAAATCACGCCAAAAGTTGATTCTTTGTGTCTTG
CCAGAGAGGAAAATTTGTGATATATATGGGCCTTGGAAAAAGAAGTGTCTGAGTGAAATT
GGTGTTGTCACACAGTGCATTGCCCCTGTCAAGATCACTAATCAATACCTTACTAATGTT
CTTCTTAAAATCAATTCTAAGCTTGGAGGAATAAATTCTCTGTTGGCAATAGAGCATTCT
GGGCATCTTCCCCTGATTAAAGATACCCCAACGATGATTTTGGGGATGGATGTCTCTCAC
AATTCACTTGGTCGATTAGATAGTCCATCAATAGCAGCTGTTGTTGGATCTCGACATTGG
CCACTGATTTCAAGGTATAGAGCGTCTGTGAGAATGCAGGCATCCAAGGTGGAGATGATT
GATGCTCTATACAAGCCATTGGAAAATGGGAGTGATGATGGTATTATCAGAGAATTGCTT
TTAGATTTCTATGATTCTAGTAATGGACGCAAGCCAACTCAATTTATTGTATTCAGGGAT
GGAGTTAGTGAATCACAATTCGAACAAGTTTTAACTATCGAGCTTAACCAGATAATCAAG
GCCTATCAACATCTTGGTGAGGTTAATGTTCCCCAGTTCACTGTGATTGTGGCACAAAAA
AAGCATCATATTAAGCTGTTTCTGCCTAATGGCCCAGAAAATGTTCCTCCTGGGACGGTT
GTGGATACAACAATTACGCATCCAAGAAATTATGATTTTTACATGTGTGCTCATGCAGGG
ATGCTTGGAACATCCAGGCCTGTACATTATCATGTGCTACTTGATGAGATTGGTTTCTCG
GCAGATGGCTTGCAAAATTTGATCCATTCGTTGTCCTATGTGAACCAAAGGAGCACAATT
GCAACCTCAGTTGTGGCACCCATATGCTATGCTCACCATGCTGCAGCTCAGATGGGACAA
CTTTTGAAT---

>GRMZM2G366277_T02 cds:_protein_coding
ATTATCCTTGGTAGAGGATTTTACTCACCAAGCAGCAGTATAGACATTGGGCAGGGTGCT
GTAGCTATGAAAGGAACCCAGCAGTCCCTTAAATCCACTCAGCAAGGGTTGATCCTGTGT
GTTGACTATTCTGTCATGCCGTTTTACAAAGCTGTGATGGATCTTGTTCAGAAATTAGTG
AGGTACCTTGATTATCGGACAACTTTGAACAAGAGGCAAATGGAAAATCTGGTTGATGAG
CTTAAAGGCCGACGTGTAACTGTGATTCATCGGAGGACTAATCAGAAGTACACAGTGCAA
GGTTTGACACCCTTACCTGCCAGCCAGATGACCTTTGTGGATGCTGAATCCGGCCAAACA
CGGAGGCTCGTGGATTATTATGCTCAGAAACATGGCAAGGTGATTGAGTATCAGATGCTG
CCATGCTTGGATTTGAGCAAGAGCAAGGACAAACCGAATCATGTTCCAATTGAGCTCTGC
ACTCTTCTTGAAGGACAGAGGTTTCCAAAAGCAAACTTGAATCAGAATTCTGAGAGGATA
CTAAAAGGAAGTGCTCTAATCCTCCTTTTCTGCCCGATGTCCGAGCAGCATTCGGGGTAC
AAGACACTGAAGCTGATTTGTGACACACAGCTGGGGATCCTGACCCAGTGTTTACTGAGC
GACCGCGCAAACAATCGAAAGGGACAGGACCAGTACATGACGAATCTTGCTCTAAAGATC
AACGGCAAGCTTGGGGGCAGCAACGTTCAGCTGTTTGACTCGCTCCCACGGGTCGGTGGT
GGGGTACCTTTCATGTTCATCGGTGCTGACGTTAACCACCCGTCCCCCGGTAACGTGGAG
AGCCCATCGATCGCAGCCGTGGTCGCCTCTGTCAACTCTGGTGTCAACAAGTATGTGACC
AGAATCCGTGCCCAGCCGCACCGCTGCGAGGTGATCCAGCAGCTTGGTGAGATCTGCCGG
GAGCTCATTGGAGTCTTTGAGAAGCAGAACCGCGTGAAGCCGCAGAAGATCATCTACTTC
CGTGATGGCGTGAGCGACGGGCAGTTCGATATGGTCCTGAACGAGGAGCTGGCTGACCTG
GAGAAGGCGATCAAGGTGAATGGCTATGCGCCAACCATCACCGTGGTCGTGGCCAAGAAG
CGGCACCACACTCGGCTGTTCCCCAGGGACGAACAGCAGCCGCAGACGAAGACCGGGAAC
GTGCCGCCTGGCACGGTGGTGGACACGGGCGTGGTGGACCCGTCCGCGTACGACTTCTAC
CTGTGCAGCCACACTGGGATTCTGGGGACGAGCAGGCCGACGCACTACTACACCCTGGTG
GACGAGCACGGCTTCGGCTCCGACGACCTGCAGAAGCTGATCTACAACCTGTGCTTCGTG
TTCGCGCGGTGCACCAAGCCGGTGTCGCTGGCGACGCCCGTCTACTATGCCGACCTCGCG

GCCTACCGTGGCAGGCTCTACTACGAG---------------------------------

>GRMZM2G141818_T02 cds:_protein_coding
CTTTTGGTTAAGCAATCATTTTTCTACAACAACCCTTCATGCTTTGTTGACTTGGGTGGT
GGTGTGATGGGTTGTCGTGGATTTCATTCAAGCTTCCGTGGCACACAGAGTGGACTTTCC
CTCAATGTTGATGTCTCAACAACAATGATCGTGAAACCTGGCCCTGTTATTGATTTTCTT
CTTTCTAACCAGAATGTTAATGATCCTAGCAGAATTGATTGGCAAAAGGCCAAGCGTGCT
CTCAAGGGCTTGAGGATTAGAACCACTCCTGCAAATTCAGAATTCAAGATTTTTGGTCTC
AGCGAGAGGATCTGCAAAGAACAAACGTTTCCGCTGAGGCAGAGAAATGGTAGCAACGGA
GATTGTGATACCATTGAAATAACTGTCTATGACTACTATGCAAAGAAAGGAATCGATCTA
AAGTATTCTGGTGATTTCCCCTGTATAAATACAGGGAAGGCAAAGCGCCCAACATATTTT
CCAATCGAGCTATGCTCGCTTGTTCCGCTTCAAAGATACACCAAAGCTTTGTCTACGCTA
CAAAGGTCATCCCTTGTGGAGAAGTCTAGACAGAAGTTCCTCTTGTGCCTTCTCCCTGAG
AGGAAAAATTGTGAAATCTATGGTCCTTGGAAGAGAAAGTGCCTGGCCGAGTTTGGTATT
GTCACACAGTGTCTAGCTCCATTAAGAGTCAATGATCCGTACCTGCTTAATTTGCTGATG
AAGATCAATGCAAAGCTTGGTGGTCTGAACTCGTTGCTGCAAGTTGAAGCATCTTCGTCA
ATACCACATGTGTCGCAAGTACCCACCATCATCTTAGGTATGGATGTTTCACATGGTCAT
CCAGGACAAGATAGACCTTCGGTTGCAGCGGTGGTTAGTTCTCGTCAATGGCCTCTTATC
TCTAGATATAGAGCATCAGTGCACACCCAATCTGCCAGACTAGAAATGATGTCCTCGTTG
TTTAAGCCGCGGGGTACTGATGATGATGGCCTCATCCGGGAATCACTGATCGACTTCTAC
ACTAGCTCTGGAAAGCGAAAACCAGAACACATAATTATTTTCAGGGATGGAGTCAGTGAA
AGTCAGTTTACCCAGGTCATCAACATTGAGCTGGATCAGATCATCGAGGCATGTAAGTTT
CTGGATGAGAAGTGGTCACCCAAGTTCACTGTGATTGTTGCTCAAAAGAACCACCACACC
AAGTTCTTTCAGACGGCATCACCAGACAATGTTCTTCCTGGAACTGTGGTGGATAGTAAA
GTTTGCCATCCTAAGAACTTCGACTTCTACATGTGTGCACATGCTGGGATGATTGGAACA
ACAAGGCCGACCCACTATCATGTTCTGCACGACGAGATAGGTTTCAGTGCCGACGAGATG
CAGGAGTTTGTTCATTCGCTCTCTTACGTGTACCAGAGGAGCACGACAGCCATCTCAGTG
GTTGCTCCAGTGTGCTACGCCCACCTCGCTGCAGCCCAGGTGAGCACGTTCCTGAGA---

>Ptrichocarpa_POPTR_0015s05550
GGCCGAGGAAGGGGAGGAATGCAACAACAGCATTATGGTGGAGCTCCGGAATACCAGGGA
CGGGGGAGAGGGCAACCTCAGCATGGAGAACGAGGTTATGGCAGTGGTCGTAGTGGAGGT
GGCCGAGGAGGACCCCCTTCAGGCGGACCATTTAGAGCACCAGCACCCGAGCTGCACCAA
GCTACTCCAGCTCCTTACCCAGCTGGGATGACCCCTCAGCCCATGCCATCTGAGGCACGT
TCTTCAATGCCCATGCTGTCTGAGGCAAGTTCTTGCCCGGTGGGTCGATCATTTTATTCC
CCTGATCTAGGAAGGAGACAGTCTCTTGGTGAGGGCTTGGAGAGTTGGCGTGGTTTCTAT
CAAAGTATTCGTCCTACACAGATGGGACTCTCACTCAATATTGATATGTCCTCAACTGCA
TTCATTGAGCCACTACCAGTTATTGATTTTGTGACTCAATTATTGAATCGAGATGTTTCC
TCCAGACCGTTGTCTGATTCTGATCGTATAAAGATTAAAAAGGCTCTCAGAGGTGTCAGA
GTTGAAGTTACACACCGTGGAAATATGCGCAGAAAGTATCGTATATCTGGTTTAACATCA
CAGGCAACACGGGAGCTGACTTTCCCGGTTGATGAAAGAGGAACATTGAAATCTGTTGTG
GAGTATTTTTATGAAACCTATGGCTTTGTAATTCAACATACTCAATGGCCTTGTCTACAA

GTGGGAAATCAACAGAGACCTAACTATTTGCCTATGGAGGTTTGTAAGATTGTTGAGGGT
CAGAGGTACTCCAAAAGATTGAATGAAAGACAGATCACTGCGTTGTTGAAGGTGACTTGC
CAACGTTTGCTCATTGTGATTCTCCCGGATAACAATGGTTCTCTTTACGGTGATTTGAAG
CGAATTTGTGAGACAGATCTTGGGCTTGTTTCTCAGTGCTGCTTGACAAAGCATGTATTC
AAAATGAGCAAGCAATATCTGGCCAATGTGGCTCTGAAGATAAATGTGAAGGTTGGAGGA
AGGAATACTGTTCTTGTTGATGCATTATCAAGACGTATTCCTCTAGTCAGCGACCGACCT
ACTATTATTTTTGGTGCTGACGTTACTCATCCTCATCCTGGGGAGGACTCAAGCCCATCC
ATTGCAGCTGTTGTGGCTTCTCAAGATTGGCCAGAGGTTACTAAGTATGCTGGCCTGGTT
TGTGCTCAAGCCCATCGCCAAGAGCTTATCCAAGATTTATATAAAACATGGCAGGATCCT
GTACGAGGGACAGTGTCTGGTGGCATGATCAAGGAACTTCTCATATCTTTCCGGAGAGCC
ACAGGGCAGAAGCCTCAGAGAATTATATTCTACAGAGATGGTGTCAGTGAAGGGCAGTTC
TATCAAGTTTTGTTGCACGAACTTGATGCAATTCGTAAGGCATGTGCTTCTTTAGAGCCC
AACTACCAGCCTCCAGTGACATTTGTTGTGGTTCAGAAGCGTCATCACACAAGGCTGTTT
GCAAATGATCACCGTGACCGTAATGCTGTTGACCGGAGCGGGAATATATTGCCTGGTACT
GTTGTGGACTCAAAAATTTGTCACCCAACAGAGTTCGACTTCTATTTGTGTAGTCATGCT
GGGATTCAGGGTACAAGCCGTCCAGCTCATTACCATGTACTTTGGGATGAGAACAAGTTC
ACTGCTGATGGGTTGCAGTCTCTTACAAACAATCTTTGCTACACATATGCAAGATGCACG
CGTTCTGTTTCCATTGTGCCACCTGCATACTATGCACATCTTGCTGCATTCCGAGCTCGT
TTCTACATGGAA--

>Vvinifera_GSVIVT00000553001
tgtagttctcttctctttcttcttttcttctgtCATCACTCTTCTTCCCAAAAGGACTGT
CCAAACTGTGGCTCCatacaggtcccatatcctctaagcacaaaccccaactgtggtgac
ccagactattcactccgctgtgatggcgactcccagaaactctacTTTGATGGCCTTAAT
GGGAGCTCTTATcttgttctcagaatcatggcttcatctcaaaggatggtggtgcaaCCA
TCACCATGGCTGCCTGGCACATGTGTTACTCAAGACATGCCAGTGAGTGAGGGCCTCTGG
TTAAACCAGACACGCCCCttcaagatcacctcatccaacacaatcttcctcttcaactgt
TCGCCTCGCCTCTTGGTCTCAcctctcaattgcacaccttctagtctttgccaccactac
ctggagagctcaggtcatgttgacagaaagcgtgctcttcagtgtgcaagtggtcttttt
tctaaagacagggttttgggaagcgggggctttggagaagtatacaagggtgagcttcat
gatggcaccattgtggctgtcaagtcagccaaagttggcaacctcaaaagcactcagcaa
gtactgaatgaagttgggatcctttctcaagtcaaccacaagaacctggttaaactactg
ggttgttgcgtggaagctgagcagccactgatgatctacaattacattccaaacgggacc
ctccatgaacatttgcatggtaagcgttccacttttctgaaatgggataccaggctgcga
attgctctacaaactgctgaagcattggcttatctgcactctgctgcacacacccccatc
taccacagggacgtgaagtcaacaaatatacttctagatgaagatttcaatgcaaaggtg
gcagattttgggctatccagattggctgagccagggctaagtcatgtgtcaacttgtgct
cagggaacactcgggtacttggaccctgagtactatcgcaactaccaattaacagataaa
agcgatgtttacagttatgggattgtgatgcttgaactgctgacttctcagaaggcaatc
gacttctcccgggagccagacgatataaatctagcaatttatgtgagccaacgagccagt
gatggtgcagtaatgggagttgtggaccagcggttgcttggacataatccttctgttgag
gtgataacaagcataaggctcttctcagagcttgcccttgcctgtctgagggagaagaag
ggggagaggcctagcatgaaggccgtggttcaagaacttACTCTGTCGGGGAAGACATTT
TTCCCACTTGGTTTAGGAGCAAGAAGTGAGATTGGCAATGGGGTACAATGCTGGAATGGA
TTCTACCAGAGTCTTCGGCCCACCCAGATGGGATTGTCCCTGAATATTGATGTGTCCTCC
AAATCCTTTTACGAACCACCTGTCATCGAATTTGCGGCTAAATTTCTAAATTTGGAGGAT
CCATCAataatggcccgaATGCCTCTGTCTAATGATGATCGTTTAAAGTTGAAGAAAGTT
CTGAAAGGAATCAAGGTGGAAGTCACTCATGGGGGACAAAGGCGTTACAAAATTTTTGAC
ATAACAGAGCAACCAACAAACCAACTAAGGTTTACTGAAGATGGACAACAGAAGTCAGTG
ATTCAGTATTTTCGTGAGAAATACAATATTGTCCTTCGTTATGCCTCTTGGCCTTCACTT
CGATCAGGGAAGGATTCAAGACCCATTTATTTACCCATGGAGACATGCACAATTGTTGCA
GGGCAGCGTTATGCTAAGAAGTTGAATGAGAGGCAGGTTGCGTCCATGTTGAGAATGACA
TGCCAGCGGATGTTGATTATTATTCTCCCCGAAGTCAATGCATACTATGGGAAAATCAAA
AGAATATGTGAAACAGAGCTTGGAATGGTTTCTCAGTGCTGTCAGCCCAGACATGCTAGA
ACTTGTAATCGGATTTATCTTGAAAATATCGTCTTGAAGATCAATGTGAAGNNNNNNNNT

CAAAATGCTATATTGGAAGACACTCTGTATGGAAGAATACCTCTTCTGACTGACATTCCT
ACTATTATATTTGGTGCTGATGTTACCCATCCACAATCAGGGGAAGATCAAGGCCCTTCA
ATAGCAGCAGTGGTGGCATCAATGGACTGGCCAACAGTGGTTACATACAGGGGCCTGGTT
TCTGCACAGCCTCATCGATCAGAAATTATCGAGGACCTCTTCAGAGTTAAAGAAGATCCA
AAGAGAGGCGTAGTTCATGCAGGAATGATAAGGGAACTCTTACTTGCTTTCAAGAGTTCA
ACTGGCCTCAAACCTTTGAGGATTATCTTCTTCAGAGATGGCGTTAGTGAGGGCATGTTT
GAAATGGTGCTGCTGAAAGAAATGGATGCCATCCGAAAGGCTTGTGCATCTCTAGAGGAA
GGATATCTCCCGCCGGTCACTTTTATTGTAGTGCAGAAAAGGCACAACACCCGGCTGTTT
CCTACAAATGAAGACAATATGGATAAGAGTGGAAACATACTCCCTGGAACTGTGGTGGAT
ACGGTGATCTGCCATCCATCTGAGCATGACTTTTACCTGTGCAGCCATGCAGGAATTCGG
tacCAGCAGACCTGCGCACTATCGTCTGATGCATTGCAAATGCTGGCAAACGATCTTTGC
TACACGTATGCAAGGTGCACCCGGTCTGTTTCCATAGTGCCTCCTGTTTATTATGCACAC
TTGGCGGCATTCAGGGCGAAGTTCTACGTGGAA---------------------------

>Cpapaya_evm.TU.supercontig_135.40

ATTTCTGCTGGTCGTGGCTTTCATTTCATTAACCCTGATCCAGATGATGATCTTGGATTT
GGTCTTACTGCATCCAGAGGGTATCGACATAGCCTCAAGCCCACCTCACAAGGTCTAGCA
CTCTGTGTGGACTATTCCGTGTTGGCATTTTGGAAAAAGATGCCAGTTATTGAGTTTCTG
AAGCAGCATATTCATAGGTTTTCTTTAAACAATTTTGATGTCTCTAGGAATGCTGTTGCA
AATGCTTTGACAAACTTAAAAGTTACAGTAACCCACCGTAAAACCAAACAAAAGTACACT
ATTGTTTGTTTGACCAAGGAGAGAACAAAGAATATTAAATTCAATATGGAAGACCCAGAT
GGCAAATGTCCAAAAAGACAAGTTAGTATAGTTGATTATTTCAGAGAGAAATACGGCAGG
GATATTGTGCATAAAGATATTCCTTGCCTTGATCTAGGAAAAAACAATAGGGCGAGCTAT
GTACCAATGGAATTCTGCGTCTTAGTGGAGGGACAGATTTATCCGAAAGAGCAGCTGCAT
ACTGATGCAGCCTGGAGGTTGAAGAACATGTCACTGTTCATTCTCTGTGTGATGTCGAGA
AAGGATCCTGGCTACAAGTATCTCAAGTGGATCTCTGAGACGAGAACTGGTGTGGTGACA
CAATGTTGTTTGTCCACTAGTGCCAACAAAGTGAATGATCAGTATCTTGCTAATCTTGCT
CTTAAGATCAATGCTAAGCTTGGAGGTAGTAACGTAGAGTTGGTTGATGCCCTTGCTCAT
TTCAAAAGAGAAGACCATGTTATGTTCGTGGGAGCTGACGTCAATCATCCTGCTGCACGG
AACACAACAAGTCCATCGATAGCAGCTGTAGTTGCCACTATAAATTGGCCAGCAGCTAAC
CAATACGCAGCAAGGATTCGTGCTCAAAACCATCGTGAAGAGAGGATTGTTAATTACGGG
AGTATGTGTCTGGATCTTGCTGAAACTTATGCTCGGCTAAATAAAGGAGTGAAACCTAAA
AAAGTTGTGGTCTTCCGCGATGGGGTGAGTGAGGGGCAGTTTGATATGGTTCTTAACGAA
GAGTTACTTGACATGAAAGACGCATTTCAAAAAGTCAGTTATTTCCCAAATATAACTATT
GTTGTGGCACAGAAGCGTCATCAAACTCGTTTCTTTCCAGAGAGTGAGAAAGATGGGGGT
CTTACCGGTAATATACCTCCTGGTACTGTTGTGGATACAAAAATCATTCACCCTTTTGAG
TTCGACTTTTATCTTTGTAGTCACTATGGGAGTATTGGGACAAGCAAGCCTACACACTAC
CATGTGTTGTGGGATGAGAATGGATTTTCTTCTGATCAGTTGCAGAAGCTCATTTATGAC
ATGTGCTTCACATTTGCCCGATGCACTAAATCTGTATCCTTGATACCTCCAGTGTACTAT
GCAGATCTTGTTGCTTACAGGGGGCGGCTGTATCATGAA---------------------

>Mtruncatula_Medtr2g034460
ATTTCTGTGGGAAGATACTTCTATCCCACAAATCCTCCTTTGGTAATGAAGGAACTTCGC
CCTGGAATAATCGCAGTCGGGGGGTTTCATCATAGTTTAAAACCAACATCTCAGGGTCTG
TCCTTATGTGTAGACTACTCAGTGGTGCCTTTCCGAAAGCAAATGTCAGTCGTGGATTTC
TTGCATGAGCGTATTGATAACTTTAACTTGGGTGAATTTGAAAAATTCAGGAAATACGTT
GAGGAAGTGCTTATTGGATTGAAAGTTAGTGTGACACACCGTAAATCCCAACAAAAATAT
ATCATTGCAGGATTAACACCTACAGTTACAAGGTATGTCACTTTCCCCATTGACCATACT
AAGGGCTGGAAACTTGAAAAGGAAGTCGGTCTTCTTAGCTTTTTTAATGACAAATATGAC
AAGGACATTGTATACAAAGATATTCCTTGTTTAGATTTAGGGAAAGGCAACAAGAAGAAC
TATGTACCGATGGAATTTTGTGTTTTAGCTGAAGGCCAAAGGTATCCCAAGGAGCGTTTA
GATGGTATTTCTGCAAAGACATTGACGGCAATGGCTCTACAATTTCTTCTCTGCGTGATG
GCTAATAAAAGTCCAGGTTACAAATACCTCAAGTGGATTTCTGAGACCAAAGTTGGTATA
GTGACACAATGTTGTTTGTCTTATAGTGCTAATCAAGGGGATGACAAATTCTATACTTAT
CTTGCTCTCAAAATCAATGCCAAGCTCGGAGGCAGTAATGTAGAGCTCAATAACAGGCTC
CCTTACTTTGAGGGTGAAGAACATGTTATGTTTATAGGGGCTGATGTCAATCACCCTGGT
TCGCGAGACAACAAGAGTCCATCAATTGTTGCTGTGGTTGCAACCATTAACTGGCCTGCT
GCAAATCGTTATGCGGCACGTGTTTGCCCACAATTCAATCGTAGTGAGAAAATATTGAAC
TTTGGGGAGATTTGTGTTGAGCTTGTTAGTTGCTATTGGCAGAAGAATGGAGTCAGGCCT
GAAAAAATTGTTGTTTTTCGTGATGGGGTGAGCGAGTTCCAGTTTGACATGGTTCTTAAT
GAAGAGCTACTTGATTTGAAGAGAGCATTCCAAAGATTAAATTATTTCCCAACGATCACT
CTTATTGTTGCACAAAAACGACATCAAACTCGATTTTTTCCAGACAGTTGGAGGGATGGG
TCTTCTAGCGGCAATATTTTACCGGGAACGGTTGTTGACACAAAAGTTACTCACCCCTTT
GAGTTTGACTTTTACCTTTGTAGTTACTATGGAAGCCTAGGTACAAGCAAGCCCACCCAC

TATCATGTTTTATGGGATGAGCACAAGTTTACATCTGATGAATTGCAGAAACTTATTTAT
GAGATGTGCTTCACCTTTGCAAGGTGCACTAAACCTGTGTCTCTAGTCCCTCCTGTGTAT
TATGCTGACCTTGCTGCTTATAGAGGACGATTATACCACGAA------------------

>GRMZM2G077801_T01 cds:_protein_coding
CTACTTGTTAGACAGTCATTTTTCAGTGATGATAGTCGAAACCTTGTTGATTTAACTGGT
GGAGTTAGTGGTTGTCGTGGACTCCACTCTAGTTTCCGTACTACAATTGGTGGTCTTTCA
CTAAATATGGATGTTTCAACCACTATGGTTGTAACTCCTGGACCAGTTATTGATTTTCTC
GTCACAAATCAAAATGTAAGAGACATCAGAGATATTGACTGGCCCAGGGCCAAGAAAATG
CTTAAAAATCTCAGAGTTAAAGCTAAGCACAACAACATGGAGTTCAAGATTATTGGCCTT
AGTGATCAACCATGCTCTAGACAGATGTTCCCAATGAAAGTTCGAAATGGAAACATCGAA
ATTAAATCTGTTGATATCACTGTTCAGGATTATTTTAAATCCAAGCAAGTTGAGCTAACA
ATGCCTTATCTGCCATGTCTTGATGTGGGAAAACCAAAACGCCCTAATTATCTCCCAATT
GAGTTATGCCACATGGTATCACTTCAACGTTATACAAAGGCACTGTCTTCTCAACAAAGG
GCAATGTTGGTTGAAAAGTCACGACAGAAATTTCTCCTTTGTCTTTTACCAGAGAGGAAG
AATTGTGATATTTACGGGCCATGGAAGAAGAAAAATCTTCATGAAATGGGTATTGTCACT
CAATGCATTGCTCCAAGTAATAAGATGAATGATCAATATTTCACCAATGTTCTTCTAAAA
ATTAATGCTAAGCTTGGTGGAATGAACTCCAAACTGGCACTGGAACATCGTCAAATGATA
CCAGTTGTGACTCAAATACCAACATTAATTCTTGGCATGGATGTTTCACATGGTTCTCCA
GGTCGAGCAGATATACCATCAATTGCTGCGGTTGCCCACAACAACTCGCGTAACAGCAGC
CTGCTACGCAAGCTGCGCCTCCCTCTTAGG------------------------------

>Ptrichocarpa_POPTR_0009s00660
ATTACTGTTGGGCGATCGTTTTTCTCACCTGATTTGGGTCCAAAAGGCGATCTCGGTGAT
GGAATAGAATATTGGAGGGGGTATTATCAAAGTCTTCGGCCAACCCAAATGGGACTATCT
TTCAATATTGATGTGTCGGCCAGATCCTTTTATGAGCCATTGGTAACTGAGTTTGTAGCG
AAGTACTTTAATTTTAGAGACTTATCAAGGCCTCTTTCTGATCAGGAGCGTGTTAAGGTG
AAAAGGGCCTTGAGAGGAATCAAAGTACAAATTACTTACAGTGACTACACTAAAAGCTAC
AAGGTTACTGGCATATCTAATCTACCAGTAAACAAAACAATGTTTACTCTGGATGATAAG
AAAACAAAGGTGTCAGTCTATCAGTATTTTTTGGAGAGATACAATATTGGGCTGAAATAT
ACATCTTTGCCTCCCCTTCAAGCTGGAACTGATGCAAAACCTATTTATTTGCCCATGGAG
CTTTGTCAGATTGCTGGAGGACAACGATACACTAAGAAGCTAAATGAAAGACAAGTCACA
GCCCTTTTAAGAGCAACCTGTCAACGGCTGCTGATTATTATTCTACCTGATGTCACCGGA

TCCTATGGGAAAATCAAACGTGTTTGTGAAACTGAGTTAGGAATAGTTTCACAATGCTGT
CAGCCCCAGCAGGCAAAGAAGCTTAGCAAACAATACATGGAAAATGTTGCTCTCAAAATT
AATGTGAAGGCTGGGGGACGAAACACTGTATTGAATGATGCTTTTCATAGAAGAATTCCT
CTTCTTACTGATGTTCCTACTATTGTTTTTGGTGCTGATGTAACCCATCCACAAGCGGGA
GAAGACGCAGGCCCATCAATTGCAGCAGTAGTTGCTTCTATGGATTGGCCAGAGGTAACC
AAGTATAGAGGACTTGTCTCTGCACAGGCTCACCGTGAAGAAATTATCGAGGATCTTTAC
AAAAAATACCAGGATCCAAAGAAGGGTTTAGTTCATGGTGGAATGATCAGGGAACTGTTA
ATTGCTTTCAAAAGATCCACCGGCCAAAAACCTTTTAGAATTATATTCTACAGAGATGGT
GTTAGTGAAGGTCAATTCAGCCAAGTTCTGCTGCATGAGATGCAGGCAATACGACAGGCA
TGTGGCTCCCTAGAAGAAGGGTATTGTCCTCGGGTTACCTTTGTAGTAGTTCAGAAAAGG
CATCATACACGCTTTTTCCCTGCTGACCATAGCAGGCGTGATCAGACTGACAAGAGTGGC
AATATTTTACCAGGTACAGTTGTTGATACTACAATTTGCCACCCTACAGAGTTTGACTTC
TACCTAAACAGTCATGCTGGAATTCAGGGAACCAGCAGACCTACACATTACCACGTGTTG
TTTGATGAAAACAACTTCAGTTCTGATGGTTTACAAACCCTCACTAACAATTTGTGCTAC
ACGTATGCAAGATGCACTCGGTCTGTTTCCATAGTGCCTCCCGCATATTATGCTCATTTG
GCAGCTTTTAGGGCGCGATATTACATTGAG------------------------------

>Mesculenta_cassava23918.valid.m1
ACAACTGTTGGGAGGTCATTTTTCTCACCTGATCTGGGGCCAAGGGGTGAGCTTGGTGAT
GGTATAGAATATTGGAGAGGATATTATCAAAGCCTTCGCCCAACCCAGATGGGACTGTCT
TTTAATGTTGATGTGTCAGCCAGATCCTTCTTTGAGCCAATGGTGACTGACTTTGTAGCT
AAGTACTTTAGGTTGAGAGATTTGTCAAGGCCTCTGTCTGAACAAGATCGCATAAAGGTG
AAGAAGTCTTTAAAAGGAGTAAAAGTAGAGCTACATCACAGGGAGTATCCTAAAAGCTAC
AAGATCACTAGCTTATCTAATCAGCCAATGAATCAAACTTTCTTCACTCTCGATGATAAG
AATTCAAAAGTATCAGTTGTTCAATACTTTCGTGAGAGATACAACATTATGCTCAAATAT
ACGTCCTTGCCTGCCCTTCAAGCCGGCAGTGATTCAAAGCCCGTTTATTTGCCTATGGAG
CTTTGTAGGATTGTTGAAGGACAGAGGTACACAAAGAAATTGAACGAAAGGCAAGTTACT
CAGCTGTTAAGGGCAACTTGTCAACGCCAGTTGTTGATAATCATTTTACCAGATTTCACT
GGGTCTTATGGGAAGATCAAACGAATCTGTGAAACAGAATTTGGAATTGTCTCTCAGTGC
TGTCAACCAAAGCAGGCAGCAAAGCTAAGTAAACAGTATTTTGAAAATGTTGCCCTCAAA
ATCAATGTTAAGGTTGGCGGTAGAAATACTGTGTTGAATGATGCTATTCAAAGGAGAATT
CCCCTTGTGACTGATCTTCCTACAATTATTTTTGGTGCTGATGTAACTCATCCAGCTCCT
GGGGAGGACTCTAATCCATCGATAGCTGCAGTAGTAGCTTCAATGGACTGGCCAGAGGTA
ACAAAGTATAGAGGCCTTGTCTCTGCACAAGCTCATCGAGAAGAAATTATCCAGGATCTC
TATAAATCATATCATAATCCAGATAAGGGATTAGTTCATTTAGGAATGATAAGGGAATTA
CTAATATCCTTCAGAAAATCAACTGGGCATAAACCTGGCAGGATTATATTCTACAGAGAT
GGTGTTAGTGAGGGCCAATTTAGCCAGGTCTTGCTACATGAGATGGATGCCATACGAAAG
GCTTGTTCTTCACTTGAGGAGGGATACCTTCCACGTGTTACCTTTGTTGTAGTTCAGAAG
AGACATCATACACGACTTTTTCCTGTTGATCGTGGTCAGACAGACAGGAGTGGCAATATT
CTGCCAGGCACTGTTATTGATACTAAGATATGCCACCCGAAGGAGTTCGATTTCTACCTC
AACAGCCATGCTGGAATTCAGGGAACAAGCAGACCTACACACTACCATGTGTTGTATGAT
GAGAATGGGTTCACTGCCGATGGGTTGCAAATTCTGACTAACAATCTTTGTTATACGTAT
GCGAGGTGTACCCGTTCTGTCTCAATAGTCCCTCCTGCCTATTATGCGCATTTAGCTGCT
TTTCGGGCTCGATATTACATAGAG------------------------------------

>Mguttatus_mgf005010m
CAGCCTcaaccgccctaccagccgcaaCACCCACAACAGCCACAGCAGCCACAAcagcca
caaCAGCAAGGAGGAGGAGGAAGAGGCTGGACACCTCAACGTGGAGGATATGGTGGCCGT
GGTGGTGGAGGCGGCAGAATGGCTCCCCCGCAACACTATGGTGGGCCTCCCGAGTATCAA
CAGCAAGGTCGGGGCGGTCAGCAATATCAGCGAGGAGGGGGTGCACCACGACGTGGCGCT
TATTCAGGTGGCCGAGGCGAGGCACCATTTGCTGGTGGGCCCTCTAGGCCACCCGCTCCC
GAGCTGCACCAAGCTGCTACCCAGTCCCCGTATCAAGCGACAATGACTCAGCCAATTCAG
TATGGGagtcctgcggagacactcagagggGCTAGCTCTTCTTCTCCAGTTGGTCGATCT
TTCTATTCTCCTGATTTAGGTAAAAGGCAGCCTCTTGGTGAAGGACTGGAAAGTTGGCGT
GGATTCTACCAGAGTATTCGTCCTACCCAGATGGGTTTGTCACTGAATATTGATATGTCA
TCCACTGCGTTCATTGAGCCTCTCCCGGTGATTGATTTTGTCACCCAGCTTCTGAACAGA
GATGTATCAGCTAGACCATTATCTGATGCTGACAGAGTCAAGATTAAGAAAGCCCTTAGG
GGAGTAAAGGTGGAAGTTACTCACAGAGGAAACATGCGAAGGAAATACCGTATCTCTGGT
TTGACATCACAAGCAACTCGTGAACTAACATTTCCTGTTGACGAGAGAGGCACTATGAAA
TCTGTTGTTGAGTATTTTCAAGAAACCTATGGCTTTGTTATCCAACACACTCAGTGGCCT
TGTCTGCAAGTTGGGAATACACAGAGGCCAAACTATCTTCCCATGGAGGTGTGCAAGATA
GTAGAGGGCCAGAGGTATTCTAAGAGGTTAAATGAGAGACAGATTACTGCACTACTCAAG
GTCACTTGTCAGCGTTTGTTAATTGTTATATTGCCAGACAACAATGGCTCTCTTTACGGT
GATCTAAAACGGATATGCGAAACGGATCTTGGAATCGTCTCACAGTGCTGTCTTCAGAAG
CATGTGTACAGGATGAGCAAACAGTATCTTGCCAACGTGTCTCTGAAAATTAATGTCAAA
GTTGGTGGGAGGAATACAGTGCTGGTGGATGCGCTCTCCAGGCGTATTCCTCTTGTCAGT
GATCGACCAACTATCATATTTGGTGCAGATGTTACACATCCCCATCCTGGAGAGGACTCC
AGCCCATCCATTGCTGCTGTTGTTGCTTCCCAGGATTGGCCTGAAGTCACAAAGTATGCA
GGCTTGGTTTGTGCTCAGGCACATAGGCAGGAACTCATCCAGGATCTCTATAAAACCTGG
CAGGATCCGGTTAAGGGAACCATGCATGGTGGCATGATCAAGGAACTACTTATTTCATTC
CGCAGAGCAACTGGGCAGAAACCACAGCGAATTATCTTCTATAGGGATGGTGTCAGTGAA
GGCCAGTTTTATCAAGTTTTGCTTTATGAACTCGATGCTATACGAAAGGCATGTGCCTCG
TTGGAGCCAAACTATCAGCCTACTGTTACCTTTGTTGTGGTCCAGAAACGTCATCATACT
CGATTGTTTGCTAATAACCACCATGACCGACATGCAGTTGATAGAAGTGGAAACATTCTT
CCTGGTACTGTTGTTGACTCCAAAATCTGTCACCCAACTGAGTTTGACTTCTACCTCTGC
AGCCATGCTGGTATACAGGGTACTAGCCGTCCAGCACATTACCATGTTCTCTGGGATGAG
AACAAATTTACAGCTGATGCTCTTCAAAGTCTTACTAATAACCTTTGCTACACATACGCC
AGGTGTACTCGCTCTGTTTCCATTGTGCCTCCTGCATATTATGCACATCTGGCAGCTTTT
AGAGCTCGATTCTACATGGAG--------------------------------------

>LOC_Os03g47830
ATGAACATTCGTGATGCTtattcaAGGTTTGATCAAGATGGGACAAGGGTATCAGTTGTC
CAGTACTTCAATCGCCAATACAGTTATTCTTTGAAATATATTAACTGGCCGTGCCTTCAG
GCTGGCAGCGACAGCAGGCCAACATATTTACCTATGGAGGTTTGCCGCATAGTAAAAGGA
CAACGCTATTCTAGAAAATTAAATGAATGTCAAGTCACACGCATGTTGAGGTTGGCACGT
GAGACATTACTGGTAATAGTACTACCTGATGCAAATGCAACTGTCTTTTATGGAAGGATA
AAGCGGCTTTGTGAAACTGAACTTGGTGTGATAACTCAGTGCTGTTTAGCTAGGAATGTT
CAGAATGTTGGTGGACGAAATACAGTACTGGAAGATGCTTTGCATAGGAGAATTCCTCTG
TTAACAGATATGCCTACAATGATCTTTGGAGCTGACGTGACCCATCCACCTGCCGGGGAG
GATTCATCTCCATCAATTGCTGCGGTTGTTGCATCGATGGATTGGCCAGAAGTGTCAAAA
TACAAATGCTCGGTTTCTTCGCAAAGCCATAGGGAAGAGATCATAGCTGATCTCTTCACA
GAGGTGAAAGATTCACAGAACAGACTTGTTTATGGTGGAATGATCAGAGAGTTGATAGAG
TCTTTCCGTAAAGCAAATGGCAGCTACAAACCTGGAAGGATAATATTTTATCGAGACGGT
GTTAGTGAAGGCCAGTTTAGCCAAGTTCTGCTTAGTGAAATGGATGCAATTCGGAAGGCT
TGTGCTAGCATAGAGGAGGGCTACCTCCCTCCAGTTACCTTTGTTGTGGTGCAAAAGAGG
CATCACACCCGTCTTTTTCCTGAAGATCATCACGCGAGGGATCAGATGGATCGAAGCAGA
AACATCTTACCTGGAACTGTTGTTGACACTAAGATATGCCATCCCAGTGAATTTGACTTT
TACCTTTGTAGCCATTCTGGCATTCAGGGAACAAGCCACCCCACGCATTACTATGTTCTA
TTCGACGAGAACAATTTCAGCGCCGATGCATTGCAAACATTGACTTACCATTTGTGCTAC
ACATATGCACGCTGCACGCGATCAGTCTCCATAGTTCCTCCGGTGTACTATGCGCACCTG
GCGGCTTCCAGAGCGCGGCACTACCTGGAG-----------------------------

>Mtruncatula_Medtr8g118920
GTATCATTTGGGAGCTTACACTATTCTCCGGATTTAAAAAAACCACATAAGCTGAGTGGT
GGCTTGGAATCATGGAGCGGTTTCTATCAAAGTATAAGGCCTACTCAGATGGGATTATCG
CTTAATGTTGACATGGCATCCACGGCGTTTATAGAACCGCTCCCTGTGATTGACATTGCA
GCTCAAATTTTAGGAAAAGATGTGCACTCAAAGCCATTGTCAGATGCAGATCGCATCAAG
ATTAAGAAAGCCTTGAAAGGTGTGAAAGTAGAAGTTACATATAGAGGAAGTTTTAGAAGG
AAGTACAGGATAACTGGATTAACATCACAACCTACAAGAGAACTCAGTTTCCCTTTGGGT
GAGAAAATGAATATGATATCAGTAATTGATTACTTTCAAGAAATGTATGGATACAAAATC
ATGTATCCTCATTTACCTTGCCTTCAAGTAGGAAGTCAAAAGAAGGTGAACTATTTGCCT
ATGGAGGCATGCAAGATAGTTGGTGGACAGAGATATACAAAAGGGCTTAGTGAGAAGCAG
ATAACTTCTATGCTAAAAGTCTCATGTCAGAGATTGGTTGTTGCCATTCTTCCAGACAAC
AATGGCTCTCTCTATGGTGATCTCAAAAAAATCTGCGAAACAGATCTCGGGCTGATTTCC
CAATGTTGTCTTACAAAATATGTATTCAAGATTAATAGACAGTATTTGTCAAATGTAGCA
CTAAAGATCAATGTCAAGATGGGAGGAAGGAACACAGTACTTTTGGATGCTATAAGTTGC
AGGATTCCATTGGTTAGTGACGTTCCAACAATAATTTTTGGAGCTGATGTATCTCATCCT
GAATCTGGAGAGGACGTGTGTCCATCCATTGCGGCTGTTGTAGCCTCCCAAGACTGGCCA
GAAGTGACAAAGTATGCAGGATTGGTATGTGCTCAGCCTCCTCGAGAAGAAATTATTAAA
GATCTTTTCAAATGTTGGAACGATCCTCGTCGCGGTATCGTTTATGGTGGCATGATCAGA
GAGCTCTTGCTCTCATTTCAGAAAGCAACTGGAAAGAAACCATGTAGGATATTATTTTAC
AGGGATGGGGTAAGTGAAGGACAGTTCTACCAGGTTTTGCTATATGAGCTCGATGCCATC
CGTAAGGCTTGTGCATCTTTGGAGCCTGGTTACCAACCTCCGGTTACATTTGTCGTGGTT
CAAAAACGGCATCACACTCGACTCTTCTCAGACAATCACAACGACAGAAACAGCATGGAT
AGGAGTGGGAATATCTTACCTGGAACTGTGGTGGACACAAAGATTTGTCATCCTACTGAG
TTCGACTTCTACTTGTGTAGTCATGCTGGAGTTCAGGGTACAAGTAAACCAGCTCACTAT
CATGTTATATGGGACGACAACAAATTCAGTGCCGATGAGATTCAGTCCTTAACTAATAAC
TTGTGCTACACATATGCAAGATGTACGCGGTCTGTTTCTTTAGTGCCTCCTGCGTACTAT
GCTCATCTGGCTGCTTACAGAGCGCGATTCTACATGGAA---------------------

>CL2621_sinensis
TGTCCTGTGGGTCGTTCATTCTATTCTCCTGATCTGGGGAGAAGGCAACCACTGGGGGAG
GGATTGGAAAGTTGGCGTGGTTTCTACCAAAGTATTCGTCCAACTCAGATGGGCCTATCC
CTAAATATTGATATGTCCTCTACTGCCTTTATTGAGCCTTTGCCAGTGATTGATTTCGTA
CAGCAGTTGCTGAACCGAGATGTTTCCTCAAGACCATTATCTGATGCTGATCGTGTTAAG
ATCAAGAAGGCTCTCAGAGGAGTTAGGGTTGAAGTTACGCATCGAGGAAATATGCGTAGG
AAGTATCGTATATCTGGCTTGACATCGCAAACAACAGGAGAGCTCACTTTTCCGGTTGAT
GAAAGTGGTACCCTGAAATCTGTTGTTGAATACTTCTACGAAACCTATGGTTTTGTTATT
CAACATACTCAGTGGCCCTGCCTACAAGTGGGAAATCAGCAGAGACCAAATTATTTGCCT

ATGGAGGTTTGCAAGATTGTTGAGGGTCAGAGGTACTCCAAGAGATTAAATGAGAGGCAG
ATTACTGCTTTGCTGAAGGTGACCTGTCAACGTCTGCTTATTGTCATTCTCCCTGATAAT
AATGGCTCTCTTTATGGTGATCTTAAACGGATTTGTGAGACAGACCTTGGGCTTGTCTCC
CACTGCTGTTTGACTAAGCATGTTTTCAAAATGAGCAAGCAATACATGGCCGATGTGGCT
TTGAAGATAAATGTGAAGGTTGGAGGGAGGAATACTGTGCTTGTTGATGCATATTCAGGC
gtatactct---

>LOC_Os04g47870
ATGGTGAAGAAGAAAAGAACTGGGTCTGGCAGCACCGGTGAGAGTTCTGGAGAGGCTCCA
GGAGCTCCTGGCCATGGTTCTTCACAGCGAGCTGAGAGAGGTCCTCAACAGCATGGGGGA
GGACGTGGTTGGGTGCCTCAACATGGTGGCCGTGGTGGTGGGCAATACCAGGGCCGTGGT
GGACATTATCAGGGCCGTGGAGGGCAAGGTTCACACCATCCAGGTGGAGGGCCTCCTGAG
TATCAGGGTCGTGGAGGGCCAGGTTCACATCATCCAGGTGGTGGGCCTCCTGACTATCAG
GGCCGTGGAGGATCAGGTTCAGGAGGGCCAGGTTCACATCATCCAGGTGGTGGGCCTCCT
GACTATCAGGGCCGTGGAGGATCAGGTTCACATCACCCAGGTGGTGGGCCTCCCGAGTAT
CAACCGCGTGACTATCAAGGACGTGGTGGTCCACGCCCCAGAGGTGGAATGCCACAGCCA
TACTATGGCGGACCTAGGGGGAGTGGCGGACGTAGTGTTCCTTCAGGTTCATCAAGAACA
GTTCCCGAGCTGCACCAAGCCCCACATGTCCAATACCAAGCCCCGATGGTTTCACCAACC
CCATCGGGAGCTGGCTCATCCTCTCAGCCTGCGGCGGAGGTGAGCTCACCAGTTGGTCGG
TCATTTTATTCTCCCAATTTAGGGAGACGCCAGCAACTTGGTGAGGGTTTGGAAAGTTGG
CGTGGTTTTTACCAAAGCATAAGGCCTACCCAGATGGGTCTCTCACTGAATATTGATATG
TCATCAACTGCATTTATTGAGCCTCTACCTGTGATTGACTTTGTTGCTCAGCTTCTGAAC
AGAGACATCTCAGTTAGACCATTATCTGATTCTGATCGTGTGAAGATAAAGAAAGCTCTA
AGAGGTGTGAAGGTTGAGGTGACGCATAGAGGAAACATGCGTAGAAAATATCGTATATCT
GGACTCACTTCACAGGCAACAAGGGAGTTATCATTCCCTGTCGATGATCGTGGTACTGTG
AAGACTGTGGTGCAATATTTTCTGGAGACATATGGTTTTAGTATTCAGCACACCACTTTG
CCTTGCCTTCAAGTGGGCAATCAGCAAAGGCCCAATTATCTGCCTATGGAGGTTTGTAAG
ATCGTTGAGGGACAGCGTTACTCGAAGCGGCTTAACGAGAAACAGATTACTGCGCTATTG
AAAGTGACTTGCCAGCGATTACTGATTGTAATACTGCCTGACAATAATGGTTCTCTTTAT
GGGGATCTCAAAAGAATCTGTGAGACTGATCTTGGATTGGTCTCCCAATGTTGTTTGACA
AAACATGTTTTTAAAATGAGCAAGCAGTATCTTGCAAATGTTGCCCTTAAAATAAACGTT
AAGGTGGGGGGAAGGAATACTGTACTTGTGGATGCTTTGACAAGGAGGATTCCCCTTGTC
AGTGACAGACCAACTATCATATTTGGTGCGGATGTTACTCATCCTCATCCTGGAGAAGAT
TCCAGTCCTTCCATTGCAGCTGTGGTTGCTTCTCAAGACTGGCCTGAAGTCACTAAGTAT
GCTGGATTGGTGAGTGCCCAAGCCCATCGTCAAGAATTGATACAAGATCTTTTCAAAGTA
TGGCAAGACCCGCATAGAGGAACTGTTACTGGTGGCATGATCAAGGAGCTTCTCATTTCT
TTCAAGAGGGCTACTGGACAGAAACCTCAGAGGATAATATTTTACAGGGATGGTGTCAGC
GAGGGGCAGTTTTATCAAGTTTTGTTGTATGAGCTTGATGCCATTAGAAAGGCTTGTGCA

TCCCTGGAACCCAACTATCAGCCTCCAGTTACCTTTGTGGTGGTCCAGAAGCGGCATCAC
ACAAGGTTGTTTGCTAATAATCACAACGACCAGCGTACTGTTGATAGAAGTGGAAACATT
CTGCCTGGAACTGTTGTTGACTCAAAGATTTGCCATCCAACCGAGTTTGATTTCTACCTG
TGTAGCCATGCTGGCATACAGGGAACAAGCCGTCCTGCTCATTATCATGTTCTGTGGGAT
GAGAACAAATTTACTGCAGACGAGTTGCAAACCCTCACGAACAACTTGTGCTACACGTAT
GCAAGGTGCACTCGCTCTGTATCAATTGTGCCTCCTGCGTACTATGCTCATCTGGCAGCC
TTCCGAGCTCGCTTTTACATGGAG------------------------------------

>GRMZM2G419182_T03 cds:_protein_coding
CTATTAGTAAGGCAATCGTTCTTCCACAATAATCCTTCCAATTTTGTTGACCTGGGTGGT
GGTGTAGTGGGATGTAGAGGGTTCCATTCTAGTTTTCGTGCAACCCAGAGTGGACTTTCA
CTCAATATCGATGTGTCCACCACAATGATAGTGAAACCTGGTCCTGTCATTGACTTTCTG
ATTGCCAATCAGAAAGTTAATGATCCAAGCATGATTGATTGGGCAAAGGCCAAGCGCTCA
CTGAAGAACTTAAGGATAAAAACAAGTCCGGCGAACCAAGAACAGAAGATTGTTGGTCTC
AGCGACAGGCCCTTGccaTTCACACTGAAACATAAAAATGGTGAATCTGAAGAGATCACT
GTTTTTGATTACTTTGTAAAGAACCGTGGCATAAAGCTGGAATATTCTGGTGATCTTCCT
TGTATCAATGTGGGAAAACCAAAGCGTCCAACTTATTTTCCAGTTGAGTTATGCAGTCTT
CTTCCTTTGCAACGGTACACTAAAGCATTGAGCACACTTCAAAGATCATCACTCGTTGAG
AAATCTAGGCAGAAATTTCTTttcattatgccattgACCTTTACAGGGCCTTGGAAGAAG
AAATGCCTTGCTGAATTTGGGATCGTTACACAGTGTGTGGCACCAACTAGAGTCAATGAT
CAGTATCTTACAAATGTCTTGTTAAAGATTAACGCAAAGTTGGGTGGCTTGAATTCGTTG
CTCCAAATTGAAACATCCCCAGCAATTCCTCTCGTATCCAAGGTCCCAACTATAATCTTG
GGTATGGACGTATCACACGGATCTCCTGGACATTCTGATATACCGTCTGTTGCTGCTGTT
GTTAGTTCTCGTGAATGGCCTCTTATCTCAAAATACAGAGCATCTGTCCGCACCCAATCA
CCAAAAATGGAAATGATTGACTCGTTGTTTAAGCCACGGGAAACTGATGACGATGGTCTG
ATTCGGGAGTGTCTGATTGACTTCTACACCAGTTCAGGGAAGAGAAAGCCCGACCAAGTC
ATCATCTTCagtttgagtttgGCTTGCAAATTTCTTGATGAGAAATGGAATCCCAAGTTC
ACATTGATCATTGCCCAGAAGAATCACCACACTAAATTTTTCATTCCTGGAAAGCCAGAT
AATGTTCCAGCTGGCACTGTTGTTGACAACAAAGTCTGTCATCCAAGGAACTTCGATTTC
TACATGTGTTCACATGCTGGAATGATTGGAACTACCAGGCCAACTCACTATCACATCCTG
CATGACGAGATAGGCTTCAATCCTGATGACCTGCAGGAGCTGGTGCACTCGCTCTCTTAT
GTG---

>Gmax_Glyma17g12850
GTGTCAATTGGGAGGTTTCTATATTCTCCGGATTTAAGAAAACCACAGCAGCTAGGTGGT
GGCCTGGAATCGTGGTGCGGCTTCTATCAGAGTATAAGGCCAACTCAGATGGGATTGTCA
CTTAATATTGACATGTCATCAATGGCGTTTATTGAACCACTTCCTGTAATTGACTTTGTT
GCTCAAATTTTGGGAAAAGATGTGCTCTCAAAGCCATTGTCAGATGCAGATCGTGTCAAG
ATTAAGAAGGCCTTAAGAGGTGTGAAAGTTGAGGTTACACATAGAGGAAGTTTTCGAAGG
AAGTACAGGATTACAGGATTGACATCACAGCCTACAAGGGAGCTTAATTTCCCCGTCGAT
GAGAAAATGAACATGAAATCAGTAGTTGATTACTTTCAAGAAATGTATGGATATACAATC
ATATATTCTCATCTACCCTGCCTTCAAGTAGGAAGCCAAAAGAAGGTGAACTATTTGCCT
ATGGAGGCATGCAAGATAGTTGGGGGTCAGAGATATACAAAAGGGCTTAACGAAAAGCAG
ATAACTTCTCTGCTAAAGGTCTCATGCCAGAGATTGTTGATTGCCATTCTTCCAGACAAC
AATGGCTCTCTGTATGGCGATCTCAAAAGAATCTGTGAAACCGATCTGGGGTTGATTTCT
CAGTGCTGTCTTACAAAACACGTATTCAAGATCAATAGGCAGTATTTGGCAAATGTGGCA
CTAAAGATCAATGTCAAGATGGGAGGAAGGAACACAGTACTTTTGGATGCCCTAAGTTGG
AGGATCCCATTGGTTAGTGACATTCCAACAATAATTTTTGGAGCAGATGTAACACATCCA
GAATCTGGAGAGGACCCTTGTCCATCCATTGCTGCTGTTGTAGCCTCCCAGGACTGGCCG
GAAGTAACAAAGTACGCAGGATTGGTATGCGCTCAGCCTCATCGTGAGGAACTCATTCAA
GATCTTTTTAAATGTTGGAAGGATCCTCATCATGGTATAGTTTATGGTGGCATGATCAGA
GAGCTGTTACTCTCTTTTAAGAAGGCAACCGGACAAAAACCATTGAGGATAATATTTTAC

AGGGATGGGGTAAGTGAAGGACAGTTCTACCAGGTTTTGTTGTATGAGCTTGATGCCATC
CGTAAGGCTTGTGCATCTTTGGAACCTAGTTACCAACCTCCGGTAACATTTGTTGTGGTT
CAAAAGCGACATCACACTAGACTCTTCTCAAACAATCATGACGACAGAAATAGCACTGAT
AAGAGTGGGAATATCTTACCTGGTACTGTGGTGGATTCTAAGATCTGTCATCCTACGGAA
TTCGACTTCTATTTATGCAGTCATGCGGGAATTCAGGGTACAAGTAGACCAGCTCATTAT
CATGTTCTGTGGGACGAGAACAATTTCACTGCTGATGAGATCCAATCTCTGACCAACAAC
TTGTGCTACACCTATGCAAGATGTACACGATCAGTTTCTGTAGTGCCTCCTGCGTACTAT
GCTCATTTGGCAGCTTACAGAGCTCGATTCTACATGGAA--------------------

>Bdistachyon_Bradi1g28260
GTCGTGGTAGGCCGGTCCTTCTTCTCCGCAAGCATTGACGACCCCAGGCATCTTGGCCTG
GGTATTGAAGGATGGAAGGGGTTCTACCAGAGTATCAGGCCTACGCAGAGCGGATTGTCT
CTGAACATAGATATGTCTTCGACAGCTTTCGTTAAAGCTCAGTCAGTGATTAAGTTTGTT
CAAGATATTCTTAAGAAACCTGATCTCCGCCATGTTACTGGTCCTGATTGTCAGAAGATT
AAGAAAGCCCTCAAGGGTGTGAGGGTTGAAGTGACACATCGAGGAGATGTACGCAGAAAG
TACTGCATTTCTGGCTTAGCTGGTACTGCTCGAGATCTGAGGTTCCAATCATCAACTGGC
GTGTCCAAGACAGTCATGGATTATTTAGAGAGACATACAAGCTGCAACTGCGTTATGAT
TTTCTCCCATGCCTCGATGTTGGTACAACACAGAAACCAAACTATCTTCCGATGGAGGTT
TGCAACATAGTTCCAGGACAGCGGTACCAGAAGAAGCTGGATGAAAATCAGGTTTCTAAC
ATGATGCAAATAACTTGCCAACAAATGTCATTTTTGACCACAGGTGATATTAAAAGAATT
TGCGAAACAGACATCGGTGTGATGTCACAGTGTTGTCTAAGGAAGAATGTCTTAAAGTCG
AGTCCTCAATTTTTTGCAAATGTTGCTATTAAGATCAATGCCAAGTGTGGGGGAAGGAAC
TCAGTATTTGCCAATAGACAAGCAAGTTTACCGGTGGTTTCAGCCAAGCCAACGATTATC
TTCGGTGCAGATGTTACTCATCCAAGTGCCCTAGATGATGCTACCCCTTCCATCGCTTCT
GTTGTTGCCTCTAAAGACTGGCCTGAGGTGACTAAGTATCATGGTGTGGTTCGTGCACAA
GGTCACCGCGAAGAGCTCATCCAAGGTCTTGAGGACATTGTTAGGGAACTCCTTCGTTCA
TTCGAAAAAGAATCTAACCGTAGGCCTGAGCAGCTGATATTCTACAGGGATGGTGTAAGC
GAGGGTCAGTTCAAGCAGGTTCTGGAGAAGGAAATCCCAGAGATAGAGAAGGCATGGAAG
GCAATATACAACGAGGAGCCACAGATCACCTTCATAGTGGTGCAGAAGAGGCACCACACA
AGACTGTTCCCGAACAATCACAGTGATATGAGCAGCAAGGACAGCAGTGGCAATGTTCTG
CCAGGGACAGTTGTTGATAGACAGGTCTGCCACCCAACAGAGTTTGATTTCTTCCTGTGC
AGCCATGCTGGGATCAAGGGAACAAGCCGTCCAACACATTACCATGTGCTGCGAGATGAC
AACAAGTTCACCGCTGATGCACTGCAGTCGCTTACGAACAACCTCTGCTATACGTATGCA
AGCTGCACTCGCTCGGTGTCGATTGCTCCTCCCGTCTATTATGCTCATAAGCTTGCTTTC
CGTGCTCGGTTCTACCAA--

>Mtruncatula_Medtr3g105930
CTCTTGGTAAGGCAATCTTTTTTTCATAATGACTTGAGGAATTTCATCGATGTTGGAGGC
GGAGTAGAAGGAGTTCGGGGTATTCATTCCAGTTTTCGTCTTACAGAGGGAGGATTGTCT
CTTAATATGGATGTGTCCACAACAACGATTGTAAAACCTGGACCTGTAATTGATTTTCTC
CTATCCAACCAGAGTGTGAGGGAACCTCGTTATATTGACTGGGCAAAGGCCAAGAGAATC
CTTAAAAATTTAAGAGTTCGTGCTACACATCGTAACCAAGAATTCAAAATTTCGGGCATG
AGTGAGAAACCCTGCATTCAACAACTTTTTAGTATGAAGATGAAAATTGGAGAAgacAAT

AACACGGAGCAGACAGTGGATATTACTGTATATGAGTATTTCGCTAAACACCGTGGCATT
GAGCTGACCTCTTCAGCTTACTTTCCATGTCTTGATGTTGGGAAGCCAAATCGACCCAAC
TTTTTGCCCCTGGAGCTATGTTCACTTGTTCCCCTTCAGCGGTATACAAAGGCATTATCT
CCTGTGCAAAGAGCATCTTTAGTAGAAAAATCACGCCAAAAGTTGATTCTTTGTGTCTTG
CCAGAGAGGAAAAACTGTGACATCTATGGGCCTTGGAAAAGGAAGTGTCTGAGTGATGTT
GGGGTTGTCACACAGTGCATTTCCCCTCTCAAGATCACTGATCAATACCTTACTAACGTA
CTTCTTAAAATCAATTCTAAGCTTGGAGGAATAAATTCTTTGCTGGCAATAGAGCATTCT
GGGCATCTTCCCTTGATTAAAGATACCCCAACAATGATTTTAGGGATGGATGTCTCTCAT
GGATCCCCTGGCCGATCAGATATTCCATCAATAGCTGCTGTTGTTGGATCTCGATGCTGG
CCTCTAATTTCGAGATATAGAGCATCTGTAAGATCACAGTCTCCTAAGGTGGAGATGATT
GATTCTCTATTCAAGCTTGTGGATaagatgaatgatgatggtattttaGAAAAAAAGGAT
GATGGTATTATCAGGGAATTGCTTCTAGATTTCTATAGTTCAAGTGGTAACCGCAGACCA
ACTCAAATTATTCTCTTCAGGGATGGAGTTGGTGAATCTCAATTTCAACATGTTTTAGAT
ATAGAGCTTAACCAGATAATAAAGGCCTATAAACATATTGATGGGGATGTTCCCAAGTTC
ACTGTAATTGTGGCACAGAAGAATCACCATACAAAGCTGTTTCAAGCTAATGCTCTGGAA
AAAAACGTTCCTCCTGGGACAGTTGTTGATACAAACATTGTGCATCCAAGAAATTACGAT
TTCTACATGTGTGCTCATGCTGGAATGATTGGAACGTCTAGGCCTGTGCATTATCATGTG
TTGCTTGATGAAATTGGATTCTCGTCAGATGGCTTGCAAAACTTGATCAATTCGCTGTCT
TATGTGAACCAGAGGAGCACAGCTGCAACCTCAATTGTGGCACCTATATACTATGCCCAC
CATGCTGCAGCTCAAATGAGAAAATTTATGAAT---------------------------

>Mesculenta_cassava13333.valid.m1
ATTTTTGCTAGTCGAAGTTTTCATCCTATCGAACCTGATACACAAGTTGACCTTCACCGT
GGCATCATTGCTTCTAGAGGGATTAAACATAGCTTGAAGCCAACCTTCCAGGGTCTAGCC
TTGTGTATGGACTATTCCGTTGTGCCATTTCGCAAGCAAATGCCCGTTATAGATTATCTA
CAGGAACACATTCCTGGCTTCAATCCAAACAATTTCAGAAGATTCAAGAAGCAAGTCAAG
GAAGCTTTGGAGGGATTGAATGTTACTGTGACTCACAGAACTACGAATCAGAAATACAAA
ATTGCTGGATTAACCGATCAGAACACACGGGATATTTCATTTGACGTGGAAAACCCGAGT
GACCAGaactctcaagggAAAGTTAACCTTGTTTCTTATTTTAGAAAAAAATACAACAAG
GATATTATCCACATGGATATACCATGCTTAGCTTTGGGGAAAAGCAACAGGAAAAATTAC
GTTCCTATGGAGTTCTGCATCATAGCTGGGTGGCAGAGATATGCAAAGGAGCTTTTGGAC
GTAAATCAATTAGGAGAGTTAAGGAAGATTTCACTTGTTTGTGTGTTGCCTaaggagcct
aaggagccgaaggagCCTAAGGAGGGTCCTGCTGGTTACAATAATCTCAAGTGGATAAGT
GAGAAAAAAGTTGGCATCCTAACTCAATGTTGTTTAGCCAAAAATTGCAGTAGACCTAAA
GATCAGTTTCTCGCAAATCTTGCTCTAAACATCAATGCGAAGCTTGGGGGCAGTAATGTA
GAGCTCATCAAGCAGCCCAATTTCTTGCAAATTAAGGGCCATTTTATGTTTGTTGGTGCT
GATGTTAATCATCCTCGCTCTTACAACACAACAAGTCCTTCAATTGCAGCTGTTGTCGGC
ACCATGAATTGGCCAGCAGCAAATAAATATAGTGCACGGATTTGTCCTCAGGATCATAGG
ACAGAGAAGATTCTGAAGTTTGGAAGCATGTGCATGGAGCTTGTAAATACATATACCAGC
CTAAATCAGGCAATGCCAAAGAATATTATCCTATTCCGTGATGGTGTAAGTGATGGGCAG
TTTGATATGGTTCTGAATGAAGAACTCACGGATCTCAAGATGACATTTCAAGCATCGAAT
TACTCCCCCAGAATTACTGTTGTTGTTGCACAGAAGCGACACACAACTCGGATGTTTCTA
GATGGCAACCAGGATGGAAATGTGCCCCCAGGAACTGTGGTAGACACAAAAATCACACAT
CCTTGTGGATTTGACTTCTACATTTGCAGCCATTATGGTAGTATAGGAACAAGCAAGCCA
GCCCACTACCATGTGTTGTGGGATGAGAATGGTTTTACTTCTGATGAATTGCAGGAGCTT
ATTCATGGCATGTGCTTCACCTGTGCCCAGTGCACCAAGTCTGTCTCACTGGTCCCTCCA
GTGTTGTATGCCGACCGTGCTGCTTATAGAGGTCGGGTTTACCAT---------------

>Csativus_Cucsa.152920
CTACTTGTCAGACAGTCATTTTTCCATAATGACCCAAACTCTTGTACTGATGTAGGCGGT
GGAGTTCTTGGCTGTAGAGGCTTCCACTCTAGTTTTAGAACAACACAGAGTGGCCTCTCT
TTGAATATTGATGTTTCCACAACCATGATTATACAGCCTGGTCCTGTTGTGGACTTTTTA
ATTGCAAACCAGAATGTCAGAGATCCTTTCTCACTTGACTGGACCAAGGCTAAACGGACA
CTCAAGAATTTGAGGATTAAAGCAAGCCCCTCCAATGCAGAATACAAGATAACTGGATTA
AGTGAAAAGCCTTGTAAAGAGCAAACGTTTACTTTGAAACAGAAAGGTGGAAATGATGAA
GACTGCATTGAAATCACTGTTTATGATTATTTTGTCAAGCATCGGAACATCGAACTTCGA
TATTCATCAGATCTTCCTTGTATAAATGTGGGGAAGCCCAAGCGTCCCACTTTTATCCCT
GTTGAGTTGTGCTCTTTGGTATCACTACAACGATACACAAAAGCACTGTCCACATTTCAA
AGAGCCTCACTTGTTGAGAAATCGAGGCAAAAGTTTCTTCTTTGTCTACTACCTGAGAGA
AAGAATTCTGATCTTTATGGTCCTTGGAAGAAAAAAAATCTTGCAGAATTTGGAATTGTT
ACTCAGTGTATTGCTCCTACACGGGTAAATGACCAATACCTCACAAATGTACTCCTTAAG
ATCAACGCCAAGCTTGGTGGGCTTAATTCCTTGTTAGCAGTGGAGCATTCTCCATCCATT
CCTATGGTTTCAAAAGTTCCAACAATTATCCTGGGTATGGATGTTTCACATGGTTCTCCA
GGACAGTCAGATATTCCGTCTATTGCTGCGGTGGTCAGTTCTAGACAGTGGCCATTGATC
TCTCGTTACCGAGCTGCAGTACGTACCCAATCTCCAAAAGTGGAGATGATTGATTCTTTG
TACAAGCGTATTTCTGATACTGAAGATGATGGAATAATGAGGGAGCTTCTACTTGACTTC
TATACTAGTTCAGGAAAAAGAAAGCCAGATCAGATTATCATATTCAGGGATGGTGTCAGT
GAATCCCAATTTAACCAAGTGCTAAATGTTGAATTAGATCAGATTATCCAGTCTTGCAAG
TTCCTTGATGAAAATTGGAACCCCAAGTTTGTGGTGATCGTGGCCCAGAAGAACCATCAC
ACTAAGTTCTTTCAGCCTGGATCTCCTGACAACGTTCCTCCCGGAACTATTATTGACAAC
AAAATTTGTCATCCAAGAAACAACGATTTCTATCTCTGTGCTCATGCTGGAATGATTGGT
ACTACAAGGCCAACTCATTATCACGTTCTGTTAGATGAAGTTGGTTTTTCTGCAGATGAC
CTGCAAGAACTAGTGCACTCTCTATCCTACGTATACCAAAGAAGTACTACAGCGATTTCT
GTTGTTGCTCCCGTCTGCTACGCTCATTTGGCAGCCACCCAGATTGGGCAGTTTATAAAG

>Gmax_Glyma02g00510
TGCCCAATTGGGAGGTCCTTCTTTTCACCTGATATTAGAACACCACAACGGCTTGGAGAG
GGTTTGGAATCGTGGTGTGGATTTTACCAAAGCATAAGGCCTACTCAGATGGGTCTTTCA
CTCAATATTGATATGGCTTCTGCTGCGTTCATTGAACCTCTTCCAGTTGTGGAATATGTT
GGCCAGCTATTAGGAAAAGATATTCTGTCGAGGCAATTGTCTGATGCTGATCGCATTAAA
ATTAAGAAAGCCCTTAGAGGAGTTAAAGTCGAAGTAACCCACCGAGGGAGTGTTAGAAGA
AAGTATCGAGTTTCTGGTTTGACTTGTCAACCAACCAGAGAACTTGTGTTTCCTGTTGAT
GAGAATTCAACTATGAAGTCAGTAGTTGAATACTTTCAAGAGATGTATGGTTTCACAATT
AAATATACTCACCTTCCTTGCCTTCAAGTAGGAAATCAAAAGAAGGCAAATTATTTACCT
ATGGAGGCCTGCAAAATTGTTGAGGGACAACGATATACAAAAAGGTTGAATGAGAAGCAA
ATCACTGCTCTCCTGAAAGTTACTTGTCAAAGACTTTTGTTAGCAATATTGCCGGACAAC
AATGGTTCTCTCTATGGTGATCTCAAGCGTATTTGTGAAACCGACCTTGGTTTAATTTCT
CAATGTTGTTTGACAAAGCATGTCTTTAAGATCACAAAGCAGTATTTGGCTAATGTGTCT
CTGAAAATCAATGTCAAGATGGGAGGTAGAAACACCGTTCTTGTTGATGCTGTAAGCTGT
AGAATACCATTGGTTAGTGACATACCAACTATAATATTTGGAGCAGATGTTACCCATCCT
GAAAATGGAGAAGACTCCAGCCCTTCAATAGCAGCCGTTGTAGCATCTCAAGACTGGCCT
GAAGTGACGAAATATGCCGGTTTAGTGTGTGCTCAAGCTCATAGACAGGAACTCATACAA

GATTTGTACAAAATGTGGCACGACCCTGTTCGTGGCTTAGTCAGTGGTGGCATGATCCGA
GACTTGTTGATTTCCTTTAGAAAGGCAACAGGGCAGAAGCCTCTAAGAATTATCTTTTAT
AGGGATGGTGTAAGTGAAGGGCAGTTTTACCAAGTTCTACTTTATGAGTTGGATGCAATT
CGGAAGGCATGTGCTTCTTTAGAACCAAACTATCAACCTCCAGTAACTTTCATAGTTGTA
CAAAAAAGACACCATACCCGGTTGTTTGCAAATAACCACAGGGACAGGAACAGTACAGAT
AAGAGTGGAAATATATTGCCTGGGACTGTTGTTGATTCCAAAATCTGTCATCCAACAGAA
TTTGATTTTTATCTGTGCAGTCATGCTGGCATCCAGGGTACGAGTCGTCCAGCTCATTAT
CATGTTCTGTGGGATGAAAACAACTTCACAGCAGATGGAATTCAGTCTTTGACAAACAAT
CTCTGTTATACATATGCCAGGTGTACACGCTCCGTATCTGTTGTTCCTCCAGCGTATTAT
GCACATTTGGCAGCTTTTCGAGCGCGGTTCTATATGGAG--------------------

>Bdistachyon_Bradi5g21800
CCTGTCCTGGATGTTGTCAGGCAGTTCATAAGAAGGAACCTTGACTACAGGACAAATCTG
ACCGACAGTGAATGGGATAATTTGTCATCTGAACTGAAAGGCCAACGAGTTACTGTGAAC
CACCGGATGACTAACCAGAAGTACACTATTCGAGGCTTAACAAAGGACCCTGCCAGAATG
ATCACCTTTGAAGATTCTGAATCAGGGCAGCAAAAGAGGCTTGTTGATTATTTTGCTCAG
CAGTATGGCAAGGTGACTGAGTATGAGATGCTTCCATGCTTGGATTTGTCCAAGACGAAA
AAGAACTATGTGCCGATTGAGCTTTGTGATTTCCTTGAAGGACAGAGGTATCCAAAGGCA
AATTTGCCAAGGAATATTGATACAAGCCTGAAAAGGATGGCTTTACTGCTCTTCTGCCCG
ATGTTTGAGCAGCATCCGGGGTACAAGACACTGAAGCTGATCTGCGAGACGAAACTGGGG
ATCCAGACCCAGTGTTTCTTGAGCAACGTCGCGAACAATCCCCGGGGCCAGGACCAGTAC
ATGTCCAACCTTGCTCTCAAAATCAACAGCAAGATCGGTGGGAGCAACGTCCAGCTCTTT
GATCTGCTCCCAAGGGACACGGGCGCTCCTTTCATGCTCATCGGCGCGGACGTGAACCAC
CCGTCGCCCGGTAATCTGGAGAGCCCGTCTATAGCAGCCGTGGTGGCGTCCATGGACAAA
GGAGCCACCAAGTATGCGTCCAGGATTCGCGCGCAGCCACACCGCTGCGAGGTGATCAAG
CACCTCGGCGAGATCTGCCAAGAGCTCATCAGTGTCTTTGAGAACAAGAACAAGGTCAAG
CCACATAAGATCATTTACTTCCGCGATGGCGTGAGCGATGGGCAGTTCGACATGGTCCTG
AATGAGGAGCTGGCGGACATGGAGAAGAGGATCAAGGTGAACGGCTACTCGCCGACGATC
ACCGTCATCGTGGCCAAGAAGCGGCACCACACGCGGCTGTTCCCCAAGGATAAGCAGGCG
GGTAAGGGCAACGTGCTCCCTGGCACGGTGGTGGACACCAAAGTGGTCGACCCCTCGGCA
TACGACTTCTACCTGTGCAGCCACAACGGGCTGATCGGGACGAGCCGGCCGACGCACTAC
TACAGCCTCATGGACGAGCATAACTACAGCTCGGACGACCTCCAAAAGCTGATCTACAAC
CTCTGCTTCGTCTTCGCCCGCTGCACCAAGCCTGTGTCGCTGGCCACGCCCGTCTACTAT
GCCGACCTGGCGGCGTACCGCGGCAGGCTCTACTACGAG---------------------

>Ppatens_1901732_locus
CTTCTTGTTAGGGACAATTTCTTCCATCCAGAGTTGGGTCCGGTTGGCGACTTGGGAGAA
GGAGTGGAGGCTTGGAGAGGTTATCACTCGAGTATAAAGCCAACTGGATTGGGGTTGACT
CTCAACCTTGATGTCACCATGACAACTATACTGAAGCCCACGGTAGAGAAATTCCTTGCT
GAATATTTCGGTGTCAGAGACCTGAATGGCTTGCAAGCAAGAAACTGGACTAAAGCGAAG

TCCATCCTTAAAGGTGTAAAGGTCGAAACAACACACATGTCTGTGTCACGCGAACATAAA
ATCTCAGGTTTCAGTGACCGTGCCATTCGGGATCTCAAGTTCTCGAGAAGGGTCAAAGAT
GGTGAAGGCAATATTGGGGAAGAGGAAATTTCAGTACAACAGTATTACTCTGACGTGTAC
ATGTACACTCTAAGGTTCCCAGACCTTCCAGCACTTGTTTCCGGGAACAAAAAGAAGGCC
ACGTTCCTACCATTGGAGCTGTGTAAGATTATCGCTGGGCAACGGTATACCAAGTCTCTA
TCCAGCAAGCAAAGGCAATTGCAGATCGCCGCTTGCAAGCAGTTTATCCTGGTCATATTA
CCTGACAAAGACAGTCCAATATATGTTCCATTCAAGCGGTTCTGTGAGATGAAGATTGGG
GTTGTTTCGCAGTGCATGGTGAAGCCTCGACAGCTTAATGATCAATACCTGGGAAATCTC
GCCTTGAAGATTAATCTCAAGATGGGGGGATTCAATTCGCCGCTTAGCCCCAGAATGGTT
TCCTGTCTTGGCCCGTCGACAATCATCTTTGGGATGGACGTGTCGCATGGGTCGCCTGGA
GAATCCAGTGTCCCTTCGATTGCAGCCGTGGTTGCAACCAAAAATTGGCCGGACGTTTTT
CACTACTCGACGCAAGTGAGAATTCAGCCCGCCAAAACGGAGATGATAGAAGGGCTCCAC
GATTCGAAAGGTGGAATGGTCAAGGAATGCTTGAAAGCATACTACATATCATGCAGAAGT
CCAAACTATCGCAAACCAACCCAAATAATTGTTTACAGGGATGGAATCAGTGAAAGTCAG
TTTGCAGAATGTTTGGAGGTTGAGTTTACTGCGTTTAAAAGGGCATGTGCAGAACTGGAA
GAAGGATATAATCCCGGTATCACCTTCATTGTTGCTCAAAAGCGACACAATACTCGTTTC
TTTCCTCAAGGCCCTGACAATCAGATGCGCAATGGAAACGTTcttccagcccacGCTGAA
AGTGCAGGAACTGTTGTCGACAAGGACGCTTGCCACCCTCACAACTATGACTTTTTCCTC
GTCTCTCAAGCTGGCCTTATTGGTACGTCTCGTCCCACGCACTATCACGTTCTCGTGAAT
GAAAATAAGCTTTCGCCTGATGACATTCAGGGCTTGACAAACAATTTGTGCTACACGTTT
GGCCGCTGCACAACATCAGTTTCTATGGGTAAGCCGCTTCAAGGA--------------

>LOC_Os03g57560
CAAGAAACCATTCAATTTGGTCACCGGGGTGACATTGGTGAGGGACTTGAGTGTTGGAGA
GGTTACTATCAGAGCCTGCGCCCAACACAGATGGGCCTTTCGCTGAATATAGATATATCT
GCAACATCATTTTTTAAGCCTGTGACAGTGATCCAATTTGTGGAGGAGTTCCTGAACATA
CGTGACACCTCAAGACCTTTGTCAGACCGGGATCGTGTGAAGATAAAGAAAGCATTACGT
GGGGTTCGCATTGAAACAAACCACCAAGAGGACCAAATCAGAAGATACAAAATAACAGGG
ATTACCCCCATTCCTATGAGCCAGCTGatgtacttatctttcctctgcttctgctataat
tatctttttgtttacatgCTATTTCCTGTTGATGATAATGGGACAAGGAAGACTGTTGTT
CAGTACTTCTGGGATAGGTACAATTACAGACTGAAGTATGCTTCTTGGCCCTGCCTACAG
TCTGGCAGTGATTCTCGTCCTGTATACTTGCCTATGGAGGTGTGCAAGATTGTAGAAGGG
CAGAGGTACTCCAAGAAGCTTAACAACAAACAGGTGACCAACATCCTTAGAGCAACCTGT
CAACGCCAGCTTTTAATTGTAATTCTGCTTGAAGTTAGTGGTTCTTATGGGAAAATTAAA
AGGGTATGTGAGAATGACCTTGGCATTGTATCTCAATGTTGTTTGCCAAGGCATGCCAGC
AGGCCGAACAAGCAATATTTGGAAAATGTTGCACTCAAAATCAATGTCAAGAAGTCCCAA
CAATCATCTTTGGTGCTGATGTCACACACTCCACCTGGAGAGGACTCTGCATCATCTATT
GCTGCGGTTGTGGCATCAATGGATTGGCCTGAAATCACCAAATACCGAGGTCTGGTCTCT
GCTCAATCACATAGACAGGAGATAATAGAAGATCTCTTTAGTGTTGGTAAAGATCCAGTG
AAGGTTGTAAATGGTGGGATGATCAGGGAGTTCCTTATCGCATTCCGCAAGAAGACTGGC
AGAAGGCCTGAGAGGATAATCTTCTATAGAGATGGTGTAAGTGAAGGTCAGTTCAGCCGT
GTGCTTCTTCATGAAATGGATGCCATCAGAAAGGCTTGTGCATCTTTGGAGGAGGGATAT
CTACCACCTGTCACATTTGTAGTAGTCCAGAAAAGGCATCACACAAGGCTTTTCCCAGAG
GTTCATGGGAGGCGAGACATGACTGACAAGAGCGGAAACATCCTTCCTGGAACTGTCAAG
GACCGCCAGATTTGCCATCCTACAGAGTTCTATTTCTACCTGTGTAGCCATGCTGGCATA
CAGGGTACTAGCAGGCCAACTCATTACCATGTCCTTTATGATGAGAACCATTTTACAGCT
GATGAACTTCAGACCCTGACCAACAATCTTTGCTATATCTATGCACGATGCACCCATGCA
GTGTCTGTGGTCCCACCGGCCTATTATTCTCATCTTGCTGCATCACATGCACACTGCTGC
ATTAAA--

>Rcommunis_29807.t000009
GTATCAATTGGAAGGTCATTCTATTCTCCTGATATCAAGAAACCGCAGCAGCTCGAAGGA
GGATTAGAATCATGGAGAGGTTTCTACCAAAGTATAAGACCGACTCAGATGGGACTGTCA
CTAAATATTGACATGTCAGCAACGGCTTTCATTGAACCTCTCCTTGTCATCGAATTTGTT
GCTCAAATTTTGAATAAAGATGTGTATTCGAGGCCATTGTCAGACGCAGATCGTGTCAAG
GTAAAGAAAGCACTCAGGGGTGTAAAAGTTGAAGTCACTCACAGAAGAAATGTACGGAGA
AAGTATAGAATTTCAGGATTGACGACACAGCCTACAAGAGAGCTAATCTTCCCGCTTGAT
GAGCATATGAACATGAAATCAGTTGTTGAGTACTTTCAAGAAATGTATGACTATACCATT
CAATATCCCCATCTACCTTGCCTGCAAGTTGGAAACCAGAGGAAGGTTAACTATTTGCCG
ATGGAGGCTTGCAAGATAGTCAGGGGGCAAAGATATACCAAAGGCCTCAATGAGAAGCAA
ATAACTTCTTTGCTAAAAGTTTCATGTCAAAGGTTACTCATTGCCATTCTTCCAGACAGC
AATGGTTCATTGTATGGTGATTTAAAACGCATTTGTGAAACTGATCTCGGGTTAATTTCT
CAATGCTGCCTTACCAAGCATGTCTTCAAGATTAACAGACAGTATTTGGCAAATGTGTCG
TTAAAAATCAATGTTAAGATGGGAGGAAGAAATACTGTGCTTTTAGATGCTATAAGTTGG
AGGATTCCCTTGGTTAGTGACATTCCAACCATAATTTTTGGAGCTGATGTAACACATCCA
GAGTCTGGAGAGGACATCAGTCCGTCTATAGCTGCTGTTGTAGCCTCCCAAGACTGGCCA
GAAGTTACAAAATATGCTGGGTTGGTTTGCGCTCAGCCTCATCGGCAAGAGCTTATTCAA
GATTTATTCAAAACCTGGCAAGACCCTCAGCAGGGGACAGTTGCTGGTGGGATGATCAGG
GAGCTCCTACTTTCATTCAAGAAGGCTACCGGGCAAAAGCCATTGAGGATCATATTTTAC
AGGGATGGTGTCAGTGAAGGTCAATTCTATCAGGTTCTACTGTATGAACTGGATGCAATT
CGAAAGGCTTGCGCATCACTTGAACCTAGTTACCAACCTCCGGTAACATTTGTTATTGTC
CAAAAGCGGCATCATACTAGACTTTTCGCAAGCAATCACAATGATAGAAGTAGCATTGAC
AGGAGTGGAAATATTTTACCTGGTACTGTTGTGGACACTAAAATATGCCATCCGACTGAA
TTTGACTTCTATTTATGTAGTCATGCAGGAATTCAGGGGACTAGCCGACCTGCACATTAC
CACGTGCTGTGGGATGAAAACAACTTCACCGCCGATGAGATTCAATCTCTGACGAACAAC
CTTTGCTATACGTATGCTAGGTGTACGCGGTCGGTTTCTGTAGTTCCTCCTGCGTATTAT
GCTCATCTGGCAGCTTATCGAGCTCGATTTTACATGGAA---------------------

>Gmax_Glyma12g08860
GTTGTTGTGGGAAGATCGTTCTTCTCACCTTCTTTGGGGAAACCTGGATCGCTTGGTAGC
GGAACGGAGTACTGGAGGGGCTATTACCAGAGCCTTCGCCCAACTCAGATGGGCCTGTCT
CTTAACATTAATGTGTCGGCGAGGGCTTTTTATGAGCCTATTCCTGTGATTGATTTCATT
GAAAGTCATTTTAGGGCCAATCCTTCCAGGCCTTTGCCTGATCAGGATCGAATCAAGCTT
AAGAGAGTACTGAGAGGAGTGAAGGTAGAAGTGACTCATGGAAAGAATCTTAGACGTTAC
AAGATCACTGGAGTCACAAAAGAACAACTCAGAAAGTTAATGTTTACTCTTGATGACAAT
AGAACAAAAAGCTCAGTTGTTCAATATTTTCATGAGAAATACAATATTGTGTTGAAGCAT
ACGCTTCTTCCTGCTCTTCAAGCTGGTAGTGACATTAAACCAATTTTTCTGCCTATGGAG
CTTTGTCAAATTGTGGCTGGACAAAGATATACAAAGAGATTGAATGAGGAGCAAGTAACT
AATCTTTTAAGGGCATCTTGTCAGCGTCAATTGTTGATCATAATTTTGCCAGATTTCGAG
GGGTCCTATGAAAAAATAAAGCGTATTTGTGAAACTGAGCTAGGAATAGTGTCTCAGTGT
TGTCAGCCGAGGCATGTTTGCCAGATGAAGCCACAATATCTTGAAAATGTGGCCCTCAAG
ATAAATGTGAAGGTTGGTGGCAGTAACACAGTATTGAATGATGCAATTGCTAGAATAATT
CCTCGTGTGTCTGACAGACCTACATTAATCTTGGGTGCGGATGTAACACATCCACAGCCA

GGGGAAGATTCTAGTCCTTCTATTGCTGCAGTAGTTGCATCTATGGATTGGCCTTATGTA
ACAAGGTACAGAGGAGTTGTTTCTGCTCAGACTCACCGTGAAGAAATCATCCAAGATCTT
TATAATACATGTGAAGATCCTGTGAAGGGGAAGGTGCATTCGGGAATTATCAGGGAGTTA
CTTCGCGCTTTCCGTTTGTCTACTAATCAGAAGCCAGAGAGGATTATATTCTACAGGGAT
GGAGTAAGTGAGGGCCAATTCAGCCAGGTTTTGCTGTACGAGATGGATGCAATACGGCGG
GCTTGTGCTTCACTACAAGAAGGCTATTTACCCCGTGTTACTTTTGTGGTGGTCCAAAAA
CGACACCACACAAGGTTATTTCCTGTAGATCATGGAAGTCATGATCAGACAAATAAAAGT
GGAAATATAATGCCAGGGACTGTCGTAGACACACACATATGCCACCCTCGGGAGTTTGAT
TTTTACCTCAACAGTCATGCTGGAATGCAAGGAACTAGTCGACCAACACATTATCATGTG
CTGTTCGATGAAAACAACTTCACTGCTGACGGGTTGCAAATGTTTACTAATAATTTGTGT
TATACGTATGCAAGGTGTACTCGATCAGTCTCAATAGTTCCACCTGTGTATTATGCACAT
TTGGCTGCCTTCAGGGCTCGCTGTTACATTGAA------------------------

>Rcommunis_29684.t000014
CTTCTTGTACGCCAGAATTTCTTTCACAATGATCCAAAGAATTTTGCAGATGTCGGGGGT
GGTGTTCTAGGTTGTCGAGGATTTCATTCTAGTTTTAGAACCACTCAGGGAGGGCTGTCT
CTGAATATTGATGTATCAACTACCATGATAATTCAGCCTGGTCCTGTTGTGGATTTTTTG
ATTGCAAACCAAAATGTGAGAGATCCCTTTCAGCTTGACTGGGCTAAGGCCAAACGAACA
TTGAAAAACTTGAGGATTAAGGCAAGTCCCTCCAACCAAGAATACAAGATAACTGGTTTG
AGTGAGATGCCCTGCAAAGAGCAAACTTTTCAATTAAACCAAAAGGGAAGGGATGATAAT
GACCCGCTTGAATTAACAGTTTATGACTACTTTGTTAATCATCGCCGTATAGAATTGCGC
TATTCTGGTGATTTACCATGCATCAATGTAGGGAAACCAAAGCGGCCTACTTTTATTCCT
ATTGAGCTTTGTTCTTTGGTGTCCTTGCAACGTTACACAAAAGCACTCAACACCCTTCAA
AGGGCTTCTTTGGTGGAGAAATCAAGACAAAAGTTCCTTTTATGTCTACTTCCTGAGAGA
AAAAACTCTGACCTATATGGTCCTTGGAAAAAGAAAAATCTTTCTGATTTTGGAATTGTC
ACCCAGTGCATTGCTCCCCAGAGGGTCAATGACCAGTATTTGACAAATGTTCTTCTGAAG
ATAAATGCAAAGCTTGGTGGTTTAAACTCTATGCTGGCAGTTGAACATTCTCCCTCTATA
CCACTGGTTTCTAAGGTTCCTACCATTATCATTGGTATGGATGTCTCACATGGCTCTCCT
GGGCATTCTGATGTCCCATCAATTGCTGCGGTAGTCAGTTCCAGGCAGTGGCCATTAATT
TCTCGATATAGGGCATGTGTCCGCACTCAGTCTCCGAAGGTTGAAATGATAGACTCTCTG
TACAAGCCTGTGTCTGACACTGAGGACGAAGGCATGATGAGGGAGCTTTTGTTGGACTTC
TATTCTAGTTCAGGAAAAAGGAAACCTGAACAGATCATCATATTCAGGGATGGCGTCAGT
GAATCACAATTCAATCAAGTGTTGAACATTGAATTGAATCAGATAATCGAGGCATGTAAG
CATCTTGATGAAAAGTGGAATCCCAAGTTTGTGGTGATTATTGCACAAAAAAACCACCAC
ACTAAGTTCTTCCAGCCTGGACTTCCTGATAATGTTCCACCTGGAACTGTCATTGATAAT
AAAGTCTGTCATCCACGGAACAATGATTTCTATCTCTGTGCACATGCTGGGATGATTGGA
ACTACGAGGCCCACCCATTATCATGTTTTATTAGATGAGGTTGGCTTTTCAGCGGATGAA
CTTCAGGAACTTGTGCATTCATTGTCCTACGTGTACCAAAGAAGCACAACTGCCATTTCT
GTAGTTGCACCGGTGTGTTATGCTCACCTGGCTGCCACTCAGATGGGACAATTCATGAAG

>Alyrata_16036850_locus
TTGCTTGTTCGACAGTCTTTTTTCCACAATGATCCAAGTAACTGTGAACAAGTTGGTGGT
AACATCTTAGGCTGTAGGGGATTTCACTCCAGTTTCAGAACAACGCAGGGTGGCATGTCA

CTTAATATGGATGTTACAACCACCATGATCATCAAGCCTGGTCCAGTGGTTGATTTCCTA
ATTGCTAACCAAAATGCGAGGGATCCTTATTCGATTGACTGGTCTAAGGCTAAAAGAACC
CTTAAGAACCTAAGGGTTAAGGTCAGCCCCTCAAACCAAGAATTCAGGATCACCGGATTG
AGTGACAAGCCTTGCAGGGAACAAACGTTTGAATTGAAGAAAAGGAACCCAAATGAAAAT
GGAGAGTTCGATACTACTGAAGTGACAGTTGCTGACTACTTCCGCGAAATAAGGCATATT
GATTTGCAATATTCTGCGGATCTTCCGTGCATCAATGTTGGGAAGCCAAAGCGACCCACT
TACATTCCTCTTGAGCTCTGCGCCTTGATTCCACTTCAGAGGTACACAAAAGCACTTAAC
ACATTCCAAAGATCTGCCCTTGTTGAGAAATCCAGACAGAAATTCATACTATGTGTGCTC
CCAGAGAAAAAGAACTGCGATCTCTACGGTCCATGGAAGAAGAAAAACTTAACTGAATTC
GGCATTGTTACTCAATGCATGGCCCCGACGCGGCAACCTAATGATCAGTATCTTACAAAC
TTACTTTTGAAGATTAATGCTAAGCTTGGAGGCCTGAACTCAATGTTAAGTGTAGAGCGT
ACACCTGCCTTCACTGTGATTTCCAAGGTTCCAACCATTATCCTTGGGATGGATGTTTCA
CATGGATCTCCTGGACAGTCTGATGTCCCGTCCATTGCTGCTGTGGTGAGTTCGAGGGAG
TGGCCACTAATCTCCAAATATAGAGCATCTGTTCGGACACAGCCTTCTAAGGCTGAGATG
ATTGAGTCCCTTTTCAAGAAAAATGGAACTGAAGACGATGGCATCATCAAGGAGTTGCTG
GTTGATTTCTACACCAGCTCGAATAAGAGAAAGCCAGAACATATCATAATTTTCAGGGAT
GGTGTGAGTGAATCTCAATTCAATCAGGTTCTGAATATCGAACTCGATCAGATCATCGAG
GCTTGCAAGCTCCTTGATGCAAATTGGAATCCAAAATTCCTCTTGTTGGTGGCTCAAAAG
AATCATCATACAAAGTTCTTCCAGACATCGTCTCCTGATAATGTTCCCCCAGGGACAATC
ATTGACAACAAAATATGTCACCCAAAGAACAATGATTTCTACCTCTGTGCTCACGCTGGA
ATGATTGGAACTACCCGTCCAACGCACTACCATGTCCTGTATGATGAGATTGGTTTTTCA
CCTGATGAACTTCAGGAACTTGTCCACTCGCTCTCCTATGTGTACCAAAGAAGCACCACT
GCCATTTCAGTTGTTGCGCCGATCTGCTATGCTCACTTGGCAGCTGCTCAGCTTGGGACG
TTCATGAAG---

>Vvinifera_GSVIVT00005949001
GTGCCTGTGGGTAGATCACTGTACTCGAGTTCAATGGGAGGGACTAAAGAAATTGGAGGA
GGGGCGGTTGGATTAAGAGGGTTCTTTCAGAGTCTTAGACCAACCCAGCAAGGACTTGCT
CTCAATGTGGATTTTTCAGTGACCGCATTCCATGAGAGCCAGAAGCGTGTTGAGTTTCTT
CGAGACCTTTCCCAGAGGAAGACAAGAGGTTTGACGGGCGAAGAAAGGAAAGAAGTGGAG
AAGGCATTGAAGAATATCAGGGTCTTCGTATGTCATAGAGAAACTGTTCAGAGATATCGG
GTGCATAGCTTAACGGAGGAAACTACGGAAAATCTCTGGTTCAAAGACAGGGATGGGAAG
ATTCTGAGGCTGGTGAATTACTTCAAGGATCACTATAGCTACGATATACAGTTCAGGAAT
TTACCATGCTTGCAGATTACAAGTAGCAAACCATGCTATCTTCCTATGGAGCTTTGTATG
ATCTGTGAAGGCCAAAAATTTCTTGGGAAGCTATCTGATGATCAAACTGCAAGAATACTT
AAAATGGGCTGCCAGAGACAGCTTCTTATGTGCATAATGGAGAGAAAGCACAAAGGGTAT
GCAGATTTGAAGCGAATTGCTGAGACCAGCATTGGGGTTGTAAGTCAGTGCTGCTTGTAC
CAAAATCTTGGGAAGTTGAGTTCACAGTTCCTGGCAAACCTGGCTCTCAAGATCAATGCC
AAAGTCGGAGGATGCACTGTTGCCTTGTACAATTCATTACCTTCCCAGATCCCGCGCCTC
CTTCGCCCCGATGAGCCAGTCATCTTCATGGGTGCTGATGTGACTCATCCTCATCCGCTT
GATGATTTCAGCCCCTCTATTGCAGCCGTGGTTGGGAGCATGAACTGGCCATCAGCTAAC
AAGTATGTTTCAAGAATGAGGTCCCAGACCCATCGTCAAGAAATTATCCAGGATCTTGGT
GCTATGGTCGGAGAAATACTGGATGATTTTTATCAGCAAGTTTCCCAACTCCCCAAGAGG
ATAATTTTTTTCAGGGATGGAGTCAGCGAAACCCAGTTCTATAAGGTGCTCCAAGAGGAG
TTGCAAGCGATAAGAGTGGCTTGCTCTAGATTTCCCAGTTACAGACCTCCCATTACTTTT
GCAGTGGTTCAGAAGAGGCACCACACGAGATTGTTTCCGAATGAAAGCAATCCATCTTCC
ATTGGAAACCAGTTCTCGGACGACAACATCCCCCCAGGGACAGTTGTTGATGCTGTGATT
ACTCACCCAAGGGAATTTGATTTCTATCTTTGTAGCCATTGGGGTGTGAAGGGAACAAGC
CGACCAACCCATTATCATGTCTTATGGGATGACAACCATTTCACTTCTGATGAACTCCAG
AAGCTGGTTTACAATCTATGCTACACATTTGTGAGATGTACCAAACCGGTTTCGTTGGTG
CCCCCAGCTTACTACGCCCACCTGGCTGCATACCGAGGCAGACTATACCTGGAG------

Supplementary File 5. Final alignment used for BI and ML phylogeny reconstruction after manual and automated sequence edition.

>Alyrata_16039685_locus
TCACTGTTGG TAAAAGCTTT TTCACTGAAA CTGAGACTTT GGGGTTGCAG CTGCGAAAGG
GTATCGCCAC ACTCTGAAGC CCACAGCACA AGGTTGTCTT TGTGTTTGGA CACTCAGTGT
TGGCGTTCCG CAAAGCTCGG TATTGAATAC TGTACTTTAA AATTGGATGT GGAAAAGGAA
CTGACTGGTT TGAAAGTCAC TGTGAATCAT CGAAACTCAC CATTGTAGGG CTGAGAAGAC
ACAAAAGACA TAAATTCGAT CTTATTGAAG GAAAACGTCC ATTGTTGAGT ATTTCAGGAT
AAAGTATGGA AGAGACATTG TTCACAAATA TTCCTTGCTT GGATTTGGGG AAAAACGGAG
GCAAAATTTT TCCCCATGGA GTTTGCGACT TGGTTGAGGG ACAGATTTAT CCAAAGTTGG
ATAAAGATTC AGCTTTGTGG TTAAAAAACT AGTCTTGTTC TGTGTGCTAT -----TCTCG
GAAGGACGAT GGCTACAACT CTGAAATGGA TAGCCGAGAC CAAACTTGGT CTTGTGACTC
AGTGTTTCTT GACGGTTCTG CCACTAAGGT GACAGTACTG GGCAAATCTT GCCCTCAAGA
TGAATGCAAA GTTGGTGGAA GCAACGTTGA GTTGATACGT TCTCTTT--- --TCCAAAAA
GAGAGGTCAT GTTCATTGGT GCCGATGTCA ATCATCCGCT CGGGAAGCCC GTCTATTGTT
GCTGTTGTGG GTACTCTAAA CTGGCCTGCA GCTAACCGTT ATGCAGCTAG AGTCATTGCC
CAGCCTCACC GTAAAGAGGA GATACAAGGA TTT------- ----GGCGAT GCTTGCCTGG
AGCTTGTCAA AGCTCATGTT CAGGCCACCG GAACGACCTA ACAAGATTGT GATATTCCGT
GATGGTGTCA GCGACGCTCA GTTCGATATG GTTCTCAATG TGGAGTTGCT TGATGTTAAG
CTGACTTTGA G-----AAGA ATGGTACAAT CCAAAGATAA CGGTAATCGT AGCCCAGAAA
CGCCATCAAA CCCGTTTCTT CCCCCACAAG CAAAAAGGCA ATGTGCCTTC AGGTACGGTC
GTTGATACTA AAGTCATTCA CCCATATGAG TATGATTTCT ACATCTGCAG TCACCATGGA
GGGATCGGGA CAAGCAAACC GACTCATTAT TACACTCTTT GGGACGAACT TGGATTCACT
TCAGATCAGG TTCAGAAGCT CATCTTTGAG ATGTGCTTCA CTTTCACTCG CTGCACCAAA
CCTGTCTCTC TTGTTCCTCC GGTGTATTAT GCTGACATGG TTGCCTTTAG AGGAAGGATG
TACCACGAG
>Vvinifera_GSVIVT00017422001
TCCTTGTTCG TCAATCCTTT TTTCATGATC CAAAGATTTG GGCGTTCTTG GCTGCAGAGG
ATTCCATTCA AGTTTTCGAA CCACCCAAGG AGGTTATCAC TGAATATTGG Ctattgattt
tatttTATGT ATCTACCCAG TGTTGATTTT CCAATCAAAA GACTGAAGGC CAAGAAAATG
CTAAAAAATC TGAGGGTGAA GACAAGCCCC TCGAGTACAA AATAACTGGA CTGAGAGCCT
TGCAAGGAGC ATTGTTTACG CTTAAGCAAT TGAAGTGACT GTTTTTGATT ATTTTGTTAA
TCATCGCCGC ATAGAACTAC GTTATTCATT TACCTTGCAT TAATGTTGGG AAGCCAAACG
ACCGACTTAC TCCCTATAGA GCTTGTACCT TGGTGTCGTT ACAACGTTAT ACTAAATTGT
CCACTCTTCA AAGAGCTTCA CTGGTGGAAG GCATTCCTTC TCTGTCTACT CCAGAAGGAA
AAACTCTGAT CTATATGGCT TGGAAACGAA AGAATCTTTC TGAATATGGA ATTGTGACTC
AATGCATTGC TCCACAAGGG TTAATGA--- --CAATATCT TACGAATGTT CTCCTAAAGA
TTAATGCAAA ATTGGTGGAT TAAATTCTAT GCTGAACATT CCCCTTCATT CCTTGTTTCG
AACCCACCAT AATCCTTGGG ATGGATGTGT CTCATGGCCT GGACAGTACC ATCTATTGCT
GCGGTTGTCA GCTCCAGGCA GTGGCCACTG ATTTCGCGCT ATAGAGCATC AGTTCGTACA
CAATCTCCAA AGGTTGAGAT GATTGATTCT CTGTATAAGA AGATGAAGGC ATAATTAGAG
AGCTTTTGCT AGACTTTTAT GTGAGTTCAG GGAAAACCCG ATCAGATTAT CATATTCAGG
GATGGAGTCA GCGAGTCTCA GTTCAATCAA GTTCTGAACA TCGAACTGGA TCAAATTATT
GAGGCTGCAA GTTCCTGATG AGAATGGTCT CCCAAATTTG TGGTGATTGT TGCGCAGAAA
AACCATCATA CCAAGTTTTT CCAGATCTCC TGA------A ACGTCCCACC TGGCACAGTC
ATAGACAACA AAGTTTGTCA TCCACGGAAC AATGACTTTT ATCTCTGTGC ACATGCTGGA
ATGATTGGTA CTACCAGGCC GACGCATTAC CACGTTCTAT TGGATGAAGT TGGTTTCTCT
TCGGATGATC TTCAGGAGCT TGTGCATTCT TTATCCTATG TGTACCAAAG GAGCACCACT
GCCATTTCCG TAGTTGCTCC CATATGCTAT GCCCACTTAG CAGCTACTCA GATGTCTCAG
TTCATGAAG
>AGO1902_Aquilegia
---------- ---------- ---------- ---------- ---------- ----------
---------- ---------- ---------- ---------- ---------- ----------
---------- ---------- ---------- ---------- ---------- ----------

---------- ---------- ---------- ---------- ---------- ----------
---------- ---------- ---------- ---------- ---------- ----------
---------- ---------- ---------- ---------- ---------- ----------
---------- ---------- ---------- ---------- ---------- ----------
---------- ---------- ---------- ---------- ---------- ----------
---------- ---------- ---------- ---------- ---------- ----------
---------- ---------- ---------- ---------- ---------- ----------
---------- -TTGGAGGCA TTAACTCTTT ATTGAGAAAA CT-----CTT CCTTCTTAAG
GACCAACCAT GATCTTGGGG ATGGACGTAT CCCATGGCCT GGTCAATGCC ATCCATTGCC
GCTGTTGTTG GTTCGAGATC TTGGCCACTA ATATCAAGAT ACAGGGCATC AGTGAGGACA
CAGTCTCCGA AGGTTGAGAT GATTGATTCC TTGTTCAAAA GGATGATGGC ATTATAAAAG
AACTACTGGT TGAATTTTAT GAGTCAAGTC GGCAAGCCTG AACAGATTAT AATCTTTAGG
GATGGAGTTG GCGAGTCTCA ATTCAACCAA GTTCTCAACA TTGAACTGGA GCAAATCATA
AAGGCCTTGA GCTTTTGGTG TCACAAGCTT CCAAAGTTTA CATTGATTGT GGCTCAAAAG
AACCACCACA CGAAGCTTTT TCAATGACCC AAA------A ATGTTCCTCC TGGAACTGTG
GTGGATACTC GAATTGTGCA TCCAAGGAAT TATGACTTTT ACATGTGCGC ACAGAATGGA
ATGATTGGAA CCTCCCGCCC AGCTCATTAC CATGTCCTGC TTGACGAGAT TGGCTTTTCT
CCTGATGATT TGCAAAACCT TGTGCATTCT CTTTCCTATG TATATCAACG CAGCACGACT
GCCATCTCAA TCGTGGCACC TATCTGTTAC GCTCACCTTG CTGCCGCACA GATTGGACAG
TTCATAAAG
>GRMZM2G105250_T01 cds:_protein_coding
TTGCAGTTGG CCGGTCCTTC TTCTCACTCA TAGAAACCTT GGTGTGGAGG GATGGAAGGG
TTTCTATCAG ACCATCAGGC CGACACAAAA AGGTTATCTG TGATCATAGA CTTTCTTCAT
CAGCTTTCAT TCGACCCCAC TATTGACTTT AGATTCTGAA GATCTAAGCT CAAGAAAGCC
CTCAAGGGTA TGAGGATTGA AGTCACACAC CGAAGTACCG AATTGCCAGC TTGACGTCCT
CCATCTTCAC ATTCTTTGAA TCATCTGCGT TCAGAAGTCC GTAGCAGATT ACTTCAGAGA
GGCATACCAT CTGGAAATGC ACTATGATTC TCCCATGCCT CCAAGTTGGC AGTGATCAAG
GCCGAATTAC TCCCTATGGA GGTTGCAAGA TAGTAGCTGG ACAGCAATAC CGGAAGTTGG
AAGGCCAACA GGTCTCTAAA CTAATGGATG CCACTTTTGC TTGCTATACT CCAGAAAAAA
TGGCAGC--- TTATATGGAT TTCAAAAGGA TCTGCGAGAC AGAGATTGGT ATCATGTCGC
AGTGTTGTTT GGAAAAAATG TTAGAAGGGC CCGCATATTT TGCTAATGTT GCTATTAAGA
TCAATGCCAA GTTGGAGGAA GAAACTTAGA ATT---GCTA ATCCCAATTA CCGTGTTACA
ATCCAACAAT TATATTTGGT GCTGATGTCA CTCACCCGCT CTAGAGCCCC TTCCATTGCT
TCTGTTGTTG CCTCCCAAGA CTGGCCCAAG GTGGCTAACT ATAATGGGAT TGTCCGTGCA
CAAGGTCATC GTAAAGAGCT CATCAATGGC CTG------- ----GAAGAC ATTGTCAAGG
AACTCCTACT TGCATTCGAG GAGAGGTCTA AGGAGACCCA AGCAGCTGAT CTTCTACAGG
GATGGCGTAA GTGAGGGCCA ATTCAAACAA GTGCTGGAAC AAGAAATCCC TGAGATAGAG
AAGGCTGGAA AGCTCTTACA ATGA---AAG CCAAAGATCA CCTTCCTAGT GGTGCAGAAG
AGGCACCACA CAAGGCTCTT CCCACCCAAA TGAAGTGGAA ATATTCTACC TGGCACTGTT
GTTGACAAGG ATATCTGCCA CCCAACAGAA TTTGATTTCT TCCTGTGCAG CCATGCTGGG
ATCAAGGGAA CTAGCCGTCC TGCGCATTAC CATGTCCTGA GAGACGACAA CAACTTCACT
GCAGACGCGC TGCAGTCTCT CACATATAAC CTATGCTTCT TGTATTCAAG CTGCACTCGC
TCTGTGTCAA TCGCTCCCCC AGCATACTAC GCCCACAAGT TAGCGTTCCG CGCCCGCTTC
TACGTCAAC
>Creinhardtii_Au9.Cre04.g214250
CCTTCCCGGG GCAGGCCTAC CACTAGGAGA TACGgaggcg GGCGGCGGCG GCGGGCGCGG
GGGCCGGCGT GACCTGGCGC CTGCTAGTGC TACGGCGACG TGCCGGGTGG CCCGCCGCCG
CCGCCGCCGC TGAGGACCGC CATGGCGGCG CGGCGCACGG CTGAGGCGGC TGCCAGGTCG
CTGGTGGGGT TCAAGGTGGA GTTCCCCATG CCCGCAAGAT GCTGACGGGG CTGAGAGGGA
GCAGACCGCA CATGTTCATG AACGAGAACG TGAGATGTCA GTGGCCGAGT ACTTCCGCTC
CACT---GGC CGCCCGCTGC GCCACCCGCC TGCCCTGCGC CAACGTGGGC GACCGCCGCG
CGCAGTCTAC TTCCGCTGGA GCTTGCACTG TGGTTGCTGG CCAGCGGCGC ATGAAGCTGG
ACGCCACGCA GTCGGCCGGC ATGATCACCA GGACTGGTGC TGGTGGTGCT CCG--GTCAA
GCCCTCGGAC GAGTACCGAG GTGAAGCGCG TGTCGGACAT CGAGTTGGGC ATACCCAGCC
AGGTGGTGGT GGGAAGGCGC GTGTGGGGGC GGCAGTACTG CGCCAACGTG GCCATGAAGA
TTAACAACAA GTGGGCGGGG TCAACGTGCA GCTGGCGGAC TGCGCTAATG CCTGCTGGGC

GGCCCTTCAT GGTGTTGGGC GCCGACGTGA CGCACCCGCG CGCGCGACCC CTCCGTGGCG
GCTGTGGTGG GCAGCCTGGA C---GCCAGC CTGGGCCGCT GGGCCAGCCG CGTGCTGCTG
CAGGCGGGGC GCCAGGAGGT GATCACGGGC ATG------- ----TGCGGC GCCACAAAGG
AGCTGCTGCT GGAGTTCTAC AGGGCAAACA ATCAAGCCGC AGCGGCTGGT CATGTACCGC
GACGGCGTGT CGGAGGGCCA GTTCGAGCAG GTCCTAGCGG AGGAGTTCAC GGCCCTGCGC
CGCGCTGCCG CGAGCTGAGG AGGGTACCGG CCCGCCATCA CCTTCGTGGT GGTGCAGAAG
CGCCACAACA CCCGCCTGCT GCCACCGCGC GTCAAGGGCA ACGTGGTCCC CGGCACGGTG
GTGGACAGTG GCATCACCGC CCCGGACGGC TTCGACTTCT ACCTCAACAG CCACTCCGGA
CTGCAGGGCA CCAACAAGCC CGCCCACTAC CACGTGCTGG TGGACGAGAT CGGGTTCGGC
GCCGACGGCA TGCAGCTGCT CACCTACTGG CTGTGCTACC TCTACCAGCG CACCACCAAG
TCGGTCAGCT ACTGCCCGGC CGCCTACTAC GCCGACCGCG CCGCCTTCAG GGGCCGCACA
CTGCTGGCC
>AT5G21150.1
TCCTTGTTCG CCAGTCCTTT TTCCATGACG TAAACCTATT GGTGTCAGTG GTTGCAGAGG
GTTCCATTCA AGTTTCAGAA CTACTCAGGG AGGTTATCCC TGAATATTGA CCTTCAACTA
CGATGATAGT ACAACCCCTG TGTTGATTTC CTAACCAGAA GACTGAAGGC TCGACGTGTC
CTCAAGAATC TGAGAGTTCA AATTACTCTT TCGAATACAA GATAAGTGGA CTAAGACAGC
TGCAAAGATC ACTATTTACA TGGAGGAAGT TGAGATCACA GTGCTCAATT ACTATAAAGA
GCGT---AAC ATTGAAGTGC GTTATTCACT TCCCTTGCAT CAATGTTGGT AAGCCGAACG
TCCCACTTAC TCCCCATTGA GTTTGTAATC TTGTGTCTCT ACAGCGATAC ACAAAACTTA
CCAATTTTCA GAGGGCTGCC CTAGTTGAAG GCATTCCTTC TTTGCATACT GCTGAAGGAA
AAACTCTGAT GTTTATGGCT TGGAAAAAAA AGAATCTTGT TGATCTTGGA ATTGTGACTC
AGTGCATTGC TCCACCAGAC TGAACGA--- --CAGTATCT CACCAATGTT CTCCTGAAGA
TAAATGCCAA GTTGGTGGAT TGAATTCGTT GTTGAGCGCT CACCAGCATG CCAAGTAACG
CACCTACCAT CATTGTTGGG ATGGATGTAT CCCATGGCCT GGCCAATACC ATCAATTGCT
GCTGTTGTGA GCTCAAGACA ATGGCCACTC ATCTCAAAAT ATAAGGCATG TGTACGCACA
CAATCACGCA AAATGGAAAT GATTGATAAT CTCTTCAAAA AGACGAAGGA ATGTTCAGGG
AACTCTTGTT AGACTTTTAC TACAGTTCAG AGGAAACCAG AGCACATCAT TATTTTCAGG
GATGGTGTAA GCGAGTCTCA GTTCAATCAA GTTCTTAATA TTGAATTGGA TCAGATGATG
CAGGCTGCAA GTTTCTGATG ATACTGGCAT CCGAAGTTTA CAGTGATAGT TGCCCAGAAG
AACCACCACA CAAAGTTCTT CCAGAGGCCC TGA------A ATGTTCCTCC AGGAACAATC
ATTGACAGCC AGATCTGTCA CCCACGCAAC TTTGATTTCT ATCTCTGCGC CCATGCTGGC
ATGATTGGAA CTACAAGGCC AACACATTAC CATGTTCTGT ATGACGAGAT TGGGTTTGCC
ACAGACGACC TCCAAGAACT TGTGCATTCT CTGTCCTATG TCTACCAGAG GAGCACCACT
GCGATCTCAG TCGTTGCACC TGTATGTTAC GCTCATTTGG CAGCTGCACA GATGGGAACT
GTGATGAAG
>Mtruncatula_Medtr5g094930
TGCTTGTCCG CCAAAACTTT TTCCATGATC CGAAGATGTA GGTGTTCTTG GATGCAGAGG
GCTGCATTCT AGCTTCAGGA CCACACAAAG TGGCTGTCTT TGAATATAGA CTGTCAACAA
CCATGATTGT CCATCCCCTG TGTTGATTTC CAAACCAGAA GACTGAAGGC TAAAAGGACT
CTAAAGAATT TGAGGATCAC TACTAGCCCA ACGAGTACAA AATTACTGGT TTAAGTGCCA
TGCAAGGACC ACTGTTTACT CTGAAGAAGA GGAGATCACT GTTTATGATT ATTTTGTCAA
CCGCAGAAAA ATTTCTCTAC AGTACTCATC TGCCATGTAT TAATGTGGGA AAGCCAAACG
ACCTACATTT TCCCAGTTGA GCTTGCTCAT TGGTATCCCT TCAACGATAT ACCAAACTGT
CCACACTCCA GAGATCCTCC CTTGTGGAAG GCATTTCTTC TTTGTCTCCT TCTGAAGAAA
GAACTCTGAT CTTTATGGCA TGGAAGAAAA AGAATCTTGC AGAGTTTGGA ATTGTTACTC
AGTGTATAGC TCCACCAGGG TGAACGA--- --CAGTATCT GACCAATGTT TTGCTGAAGA
TCAATGCAAA GTTGGTGGCA TGAATTCTCT TTTGAACACT CTCCATCATT CCTTGTGTCA
AACCTACTCT AATTTTGGGC ATGGATGTTT CTCATGGCCT GGGCAATTCC CTCAATTGCT
GCGGTCGTAA GCTCAAGGCA ATGGCCCTTG ATATCTAAAT ATAGGGCATG CGTCCGTACT
CAGGGTGCTA AGGTTGAAAT GATTGACAAT CTGTTCAAGA GGATGAAGGA ATAATAAGGG
AGCTTTTGAT TGATTTTTAT AATAGTTCTG GGAAAACCAG ACAATATCAT AATTTTCAGG
GATGGTGTCA GCGAGTCTCA GTTCAACCAA GTTTTGAACA TTGAACTGAG CCAAATCATT
GAGGCTGCAA ATTTCTGATG AAAATGGAAC CCCAAATTTC TGGTTATTGT TGCCCAGAAG
AATCATCACA CAAAGTTCTT CCAGATCTCC GGA------A ATGTGCCTCC TGGTACTGTT
GTGGACAACA AAATCTGTCA TCCCCGGAAT TATGATTTCT ACATGTGTGC TCATGCTGGA

ATGATTGGTA CCAGTAGGCC AACTCATTAT CATGTCCTTT TGGATGAGAT TGGCTTTTCT
CCAGATGATC TTCAAGAGCT TGTGCACTCT CTCTCCTATG TTTATCAGCG GAGCACTACT
GCCATCTCTG TTGTTGCTCC AATCTGCTAT GCCCATTTGG CTGCTTCTCA AGTGGGACAG
TTCATGAAG
>LOC_Os04g06770
TTTTAGTACG CCAGTCATTT TTCCACAATC CTAAGACCTG GGTGTGATGG GCTGTCGAGG
ATTCCATTCA AGTTTCCGTG GCACACAGAG TGGCTTTCTC TGAATATCGA TTTTCGACAA
CTATGATTGT TAAACCCCTG TATCGATTTT CCAACCAGAA GATTGAAGGC CAAGCGTGCT
CTCAAGAACT TGAGGATAAG AACCACCCCT GTGAATTCAA GATCATTGGT TTGAGGAAAT
TGCAACGAAC AATGTTTTCG TTGAGGCAGT TGAAGTTACA GTCTATGATT ACTTTGTGAA
AAACAAAGGC ATAGAGCTGC GCTATTCATC TTCCTTGCAT AAATGTGGGG AAACCAAACG
TCCAACTTAT TTCCAATAGA GCTTGCTCTC TTATTCCACT ACAAAGATAC ACCAAATTGT
CAACACTGCA AAGGTCCTCC CTTGTTGAAG ACATTCCTCT TGTGCCTTCT CCTGAAGGAA
AAATTGTGAA GTTTATGGCT TGGAAGAGGA AGTGTCTTGC TGAATTCGGT ATTGTCACAC
AATGCCTTGC TCCCAAAGAG TCAATGA--- --CAGTACCT ACTCAATTTG CTATTGAAGA
TAAATGCCAA GTTGGTGGAA TAAACTCATT GCTGAAGCAT CCCCTTCATA CCTTGTATCG
AACCTACCAT CATCTTAGGT ATGGATGTGT CACACGGCCA GGACAAGACC TTCCATTGCT
GCGGTGGTTA GCTCTCGCCA ATGGCCTCTC ATCTCTAAAT ACAGAGCATC GGTGCACACT
CAGTCACCTA AGCTAGAAAT GATGTCTTCC TTGTTTAAGA AGATGATGGC CTCATTCGGG
AATCGCTGAT TGACTTCTAC ACTAGTTCTG GGGAAACCGG ATCATGTTAT CGTTTTCAGG
GATGGAGTTA GTGAAAGCCA GTTTACTCAG GTCATTAACA TTGAGCTTGA TCAGATCATT
GAGGCTGCAA ATTTCTGATG AGAATGGTCA CCAAAGTTCA CAGTGATTGT TGCTCAGAAG
AATCATCATA CCAAATTTTT TCAGATCTCC AGA------A ATGTTCCGCC AGGTACTGTT
GTGGACAAAC AAGTGTGCCA TCCAAGGAAT TATGACTTCT ACATGTGTGC TCATGCTGGA
ATGATTGGAA CGACGAGGCC AACACATTAT CATGTTCTGC ACGATGAGAT AGGTTTCTCC
CCTGATGATC TGCAGGAGCT AGTGCACTCA CTCTCTTATG TGTATCAGAG AAGCACAACA
GCCATATCAG TTGTTGCTCC GATTTGCTAT GCCCATCTTG CTGCTGCTCA GGTGGGCACA
TTCCTCAAG
>GRMZM2G317927_T01 cds:_protein_coding
GTCCTGTTGG TAGATCATTT TATTCCAATT TAGGCAACTT GGTTTGGAAA CTTGGCGTGG
TTTCTATCAA AGCATAAGGC CCACACAGAT GGGCTTTCTC TGAATATTGA TTGTCCTCTA
CTGCATTTAT TGAGCCCCTG TATTGATTTT AGCTTCTTGA GATCGAAGAT TAAAAAAGCC
CTACGAGGTG TGAAAGTCGA GGTCACACAC CGAAGTATCG GATATCTGGC CTCACAAGCA
ACAAGGGAGT TTCATTCCCT ATTGATGAAC TGTTAAGACT GTGGTGCAAT ACTTCCTGGA
GACTTATGGC TTTAATATTC AGCACACCTT TACCTTGTTT GCAAGTGGGC AATCAGCAAG
AATAAATTAT TGCCTATGGA GGTTGTAAGA TAGTTGAGGG ACAGCGTTAC TCAAAACTCA
ATGAGAAACA GATCACTGCT CTACTGAATG CCATTGCTGA TGGTAATACT CCTGAAATAA
TGGCTCT--- CTTTACGGAT CTTAAAAGGA TATGTGAGAC TGATCTTGGA TTGGTCTCCC
AATGCTGTCT GACAAACATG TTTTCAAAAC AACAGTATCT TGCAAATGTT GCCCTGAAAA
TAAATGTTAA GTTGGGGGAA GGAATACTGT ACTGATGCTT TGGCAAGATC CCTTGTCAGT
GAGCTACTAT TATCTTTGGT GCTGATGTGA CCCATCCCCT GGGGAAGTCC TTCCATTGCA
GCTGTGGTTG CTTCTCAAGA CTGGCCTGAG GTTACGAAGT ATGCAGGATT AGTGAGTGCT
CAAACCCATC GCCAAGAATT GATACAGGAT CTTTTCAAGT CTCTGGTGGC ATGATTAGGG
AACTTCTCAT TTCTTTCTGG AGGGCGACTG GAAAAACCCA AGAGGATCAT ATTCTACAGG
GATGGTGTCA GTGAGGGACA GTTCTACCAA GTTCTGTTGT ACGAACTTGA TGCCATTAGA
AAGGCTGTGC ATCATTGAGT CTGATACCAG CCTCCAGTTA CCTTTGTCGT GGTCCAGAAG
CGTCATCACA CCAGGTTGTT TGTATCACAA TGAAGTGGCA ACATACTGCC GGGCACTGTG
GTGGACTCGA AGATTTGCCA TCCAACCGAG TTTGATTTCT ACCTGTGCAG CCATGCTGGC
ATTCAGGGAA CAAGCCGTCC TGCCCATTAT CATGTTCTGT GGGATGAGAA CAAATTTACG
GCTGATGGGT TGCAAACTCT CACCAACAAC TTGTGTTACA CGTATGCCAG GTGCACACGC
TCAGTATCAA TCGTTCCTCC TGCATACTAT GCTCATCTGG CAGCCTTCCG AGCTCGGTTT
TACATGGAG
>LOC_Os07g28850
TTGCGTTTGG TCGGTCTTAC TTCTCGGGGC TCGGGAACTC GGCATCTTTG CGTGGAAGGG
GTTCTACCAG AGTTGCCGGG TCACGCAGCA GGGCTTTCTC TGAACATAGA CTGTCTTCGA
CTGCTTTCAT TGAACCatta cgtgggatat ACAATTATGG -----GAATT GATGAGGACC

CTTAAGGGTG TTAAGGTTGA AGTCACTCAC CGAAGTACCG CATTGCTGGC TTCACAGTCT
GCAGATGTTC AACGTTCACA TCATCTGA-- -ATCAAGACT GTCAAGGAGT ATTTCAACAA
AAAATACAAT CTGAAGTTAG CTTTTGGATC TTCCATGCCT GCAAGTTGGC AGCAAGGAAG
ACCGAATTAC TGCCCATGGA GCTTGCAATA TAGTTCCTGG ACAACGATAC AAGAACCTCA
GTCCGACACA GGTTTCCAAT CTGATTAAAA CGACTCCTAC TTGTTGTAAT ACAGAAAAAA
TAATGCCAGC TTATATGGAC GTTAAAAGAA TCTGTGAAAC AGAAATCGGT GTATTGTCAC
AGTGTTGTCG AGCAAGCAAG TCTACAAAAT gtCAGTACTG CGCAAATGTT GCTCTTAAGA
TCAATGCCAA GCTGGAGGAA GGAACTCGGT ATTAATGTAG AAGCAAGTTA CCTTGTTTCA
AACCAACTAT TATATTTGGT GCTGATGTTA CCCATCCTCC TTTGAACCCC TTCCATTGCT
TCGGTTGTTG CTTCCGCAGA CTGGCCTGAG GTGACCAAGT ATAATTCTGT TGTTCGTATG
CAAGCTTCTC GTAAGGAGAT TATACAAGAT CTT------- ----GATAGC ATTGTTAGGG
AACTTCTCAA TGCATTCAAA AGGGACTCCA ATGGAGCCGA AGCAGCTCAT TTTCTACAGG
GACGGCGTAA GCGAGGGTCA GTTCCAGCAA GTTGTAGAGA GCGAAATACC GGAGATAGAA
AAGGCTGGAA GTCTCTTATG CTGG---AAG CCACGAATTA CCTTCATAGT GGTGCAGAAG
AGGCATCATA CAAGGCTGTT CCCATtacaa tgaACTGGAA ATGTTCGTCC AGGCACAGTA
GTTGATACAG TGATCTGTCA CCCTCGAGAG TTTGATTTCT TCCTGTGCAG CCAAGCCGGG
ATCAAAGGGA CAAGCCGTCC TAGCCATTAC CATGTGCTGC GCGACGACAA CAACTTCACC
GCAGATCAGC TTCAGTCTGT CACAAACAAC CTGTGCTACT TATATACAAG CTGCACTCGC
TCGGTGTCTA TTCCACCTCC TGTTTACTAC GCTCATAAGC TCGCATTCCG CGCTCGTTTC
TACCTCACC
>Sbicolor_Sb01g032060
TTCCCATGGG CCGGTCTTTG TATCCTTCAA TGGGGAGATT GGAGCTGTCA TGTTACGAGG
TTTCTTCCAG AGCTTGAGAC CAACAAAGCA AGGCTTGCCC TCAATGTTGA CTCTCACTTA
CAGCTTTCCA TGAAAAAAGC GTGTGACTTT ACCTTTCACA AGGAGGAGGT GGAAAAAGCA
TTGAAGAATA TCCGAGTGTT CGTGTGCCAC CGAGGTACCA TGTGCATGGC TTGACAGACA
ACAGAGAACC TAAGTTTCGA GATCGCAGAA GGATTATACG GTCGTAGATT ACTTCAAAGA
GCACTACAAC CATGATATTA AATTCAGACC TGCCCTGCTT GCAGATTGGT AAGAGC--AA
GCCATGCTAT TGCCAATGGA GCTTGCATGG TTTGTGAGGG CCAGAAGTTT CTTGGCCTCT
CAGATGAACA GACCTCCAAA ATGCTCAGTG CCACTGCTAA TCTGCGTCAT -----GAGCG
GAGGCACCGG GGCTACGCAT CTGAAGCGTA TTGCAGAAAC ATCCATTGGT GTCTTGACAC
AGTGTTGCCT GTATCCAACC TAAGCAAAGC TTCAGTTCTT GGCCAACTTA GCACTGAAGA
TAAATGCGAA GTTGGTGGAA GCAACGTTGC CCTAACAGCT TGCCATGATT CCTTTCAGAC
AACCAGTGAT GTTCATGGGT GCTGACGTGA CACACCCCCC CTAGAAGCCC GTCCGTGGTC
GCTGTAGTTG CGAGCATGAA TTGGCCTTCA GCAAACAAGT ACATCTCCAG GATGAGATCA
CAGACGCACC GTAAAGAGAT CATCGAGCGC CTT------- ----GATGTA ATGACCGGTG
AACTGCTCGA GGAGTTTGTG AAAGAAGTCG GAGCTCCCTA GCAGAATCAT ATTCTTCAGA
GATGGTGTTA GTGAGACGCT GTTCTACAAG GTGTTGACAG AGGAGCTGCA GGCAGTGCGA
CTGGCTGCTC GAGGTA---C CGGGTACAAG CCAGCGATCA CGTTCGTGGT GGTTCAGAAG
AGGCAGCACA CCAGGCTCTT CCAGGGAGAA GAAGACCAGA ACGTACCACC GGGAACGGTG
GTGGACACCG TGATCACGCA CCCAAGGGAG TTTGATTTCT ACCTGTGCAG TCACTGGGGC
ACCAAGGGGA CGAGCAGGCC GACGCACTAC CGCGTGCTGT GGGATGAGAA CAACTTCAAG
TCTGACGAGA TGCAGCAGCT GATACACAAC CTTTGCTACA CGTTTGCCCG GTGCACCAAG
CCTGTTTCTC TCGTCCCACC GGCATACTAC GCACACCTGG CCGCATATAG AGGAAGGCTA
TACCTTGAG
>Ptrichocarpa_POPTR_0012s03410
GTCCTGTGGG TCGATCATTT TATTCTGACC TAGGTCACTT GGCTTGGAGA GTTGGCGTGG
TTTCTATCAA AGTATTCGTC CTACTCAGAT GGGCTATCGC TGAACATTGA TTGTCATCCA
CGGCCTTCAT TGAGCCCCAG TATTGATTTT AGTTATTAAA GATCGAAGAT TAAAAAGGCA
CTAAGAGGCG TCAAAGTTGA AGTTACACAC CGAAGTACCG TATTTCTGGC TTAACAGGCA
ACACGGGAGC TACTTTCCCG GTTGATGAAC CCTGAAATCT GTCGTGGAGT ACTTCTACGA
AACCTATGGT TTTGTAATTC AACACCCAAT GGCCTTGTCT ACAAGTAGGA AATCAACAAG
ACCTAATTAT TGCCTATGGA GGTTGCAAGA TTGTTGAGGG TCAGAGGTAC TCCAAATTGA
ACGAGAGACA GATAACTGCA TTGTTGAATG CCATTGCTTA TTGTGATTCT CCTGAAACAA
TGGTTCT--- CTTTATGGAT TTGAAGCGTA TTTGTGAGAC AGATCTTGGG CTTGTTTCCC
AGTGCTGTTT GACAAGCATG TGTTCAAAGC AACAATATCT TGCTAATGTG GCTTTGAAGA
TAAATGTGAA GTTGGAGGAA GGAATACAGT ACTGATGCAA TATCTAGATC CCTAGTCAGC

GACCTACTAT TATTTTTGGT GCTGATGTTA CTCATCCCCT GGGGAAGCCC ATCTATTGCA
GCCGTTGTGG CTTCGCAAGA TTGGCCAGAG GTTACCAAAT ATGCTGGCCT GGTTTGTGCT
CAAGCCCACC GACAAGAGCT TATCCAAGAT TTATATAAGT GTCTGGTGGC ATGATCAAGG
AGCTTCTCAT ATCCTTCCGT AGGGCAACCG GAGAAGCCAC AGCGAATTAT ATTCTACAGA
GATGGTGTCA GTGAAGGACA GTTTTATCAA GTTTTGTTGT ACGAGCTTGA TGCTATTCGT
AAGGCTGTGC TTCTTTGAGC CCAATACCAG CCTCCTGTGA CATTTGTTGT GGTTCAGAAG
CGTCATCACA CAAGGCTGTT TGCATCACCG TGAAGTGGGA ATATATTGCC TGGTACTGTT
GTGGACTCGA AGATCTGCCA TCCTACTGAA TTCGACTTCT ATTTGTGTAG TCACGCTGGG
ATTCAGGGCA CAAGCCGTCC AGCTCATTAC CATGTACTTT GGGATGAGAA CAAGTTTACT
GCTGATGGGC TGCAGTCCCT GACAAACAAT CTTTGCTACA CATATGCACG ATGCACGAGA
TCTGTTTCCA TTGTGCCACC TGCATACTAT GCTCATCTTG CTGCATTTCG AGCTCGTTTC
TACATGGAG
>Sbicolor_Sb09g000530
GTCCTGTTGG TAGATCATTT TATTCCAACT TAGGCAACTT GGTTTGGAAA CTTGGCGTGG
TTTCTACCAA AGCATAAGGC CCACACAGAT GGGCTTTCTC TGAATATTGA TTGTCCTCTA
CTGCATTTAT TGAGCCCCAG TACTGAATTT AGCTTCTTAA GATCGAAGAT TAAAAAAGCC
CTACGAGGTG TGAAAGTCGA GGTCACACAC CGAAATATCG GATATCTGGC CTCACAAGCA
ACAAGGGAGT TTCATTCCCT ATTGATGAAC TGTTAAGACT GTGGTGCAAT ACTTCCTGGA
GACTTATGGC TTCAGTATTC AGCACACCTT TACCTTGCTT GCAAGTGGGC AATCAGCAAG
ACCAAATTAT TGCCTATGGA GGTTGTAAGA TAGTTGAGGG ACAGCGTTAC TCAAAACTTA
ATGAGAAACA GATCACTGCT CTACTGAATG CCATTGCTGA TTGTAATACT CCTGAAATAA
TGGTTCT--- CTTTACGGAT CTCAAAAGGA TCTGTGAGAC TGATCTTGGA TTGGTCTCCC
AATGCTGTCT GACAAACATG TTTTCAAAAC AACAGTATCT TGCAAATGTT GCCCTGAAAA
TAAATGTGAA GTTGGGGGAC GGAATACGGT ACTGATGCTT TGACAAGATT CCTTGTCAGT
GACCAACTAT TATCTTTGGT GCTGATGTGA CCCATCCCCT GGGGAAGTCC TTCCATTGCA
GCTGTTGTTG CTTCTCAAGA CTGGCCTGAG GTTACCAAGT ATGCAGGATT AGTGAGTGCT
CAAACCCATC GCCAAGAATT GATACAGGAT CTTTTCAAGT CTCTGGTGGC ATGGTCAGGG
AACTTCTCAT TTCTTTCTGG AGGTCAACTA AAGAAACCAA AAAGGATCAT ATTCTACAGG
GATGGTGTCA GTGAGGGACA GTTCTACCAA GTTCTGTTGC ATGAACTTGA TGCCATTAGA
AAGGCTGTGC ATCATTGAGT CCGATACCAG CCTCCAGTTA CGTTTGTTGT GGTCCAGAAG
CGTCATCACA CTAGGTTGTT TGCATCACAA TGAAGTGGAA ACATACTGCC TGGTACTGTG
GTGGACTCAA AGATCTGCCA TCCAACTGAA TTTGATTTCT ACCTCTGTAG CCATGCTGGC
ATTCAGGGAA CAAGCCGCCC TGCCCATTAT CATGTCCTGT GGGATGAGAA CAAATTTACC
GCGGATGGGT TGCAAACTCT CACCAACAAC CTGTGTTACA CGTATGCTAG GTGCACTCGC
TCAGTATCAA TCGTTCCTCC TGCATATTAT GCTCACCTGG CAGCCTTCCG AGCTCGCTTC
TACATGGAG
>GRMZM2G059033_T01 cds:PUTATIVE_protein_coding
TAACTCTTTC TCGATCCTTC TTCTCGAAGT TCGGGACATT GGGCTAGAGT GCTGGAGAGG
ATATTACCAG AGCTTGCGTC CAACTCAAAT GGGCTCTCAT TGAACATTGA TCATGCTCGA
CTTCATTTTA CCAACCGATG TGTAAAATTT ATTGTCTCCA GATCGAAGCT TAAGAGAGCC
CTGCGCGGAG TTCTTGTTGA GACTGAACAC CAATCTACAG GATAACTGGG ATTACTTCCA
TTGGCTCAAC TAGCTTTTCT TGTAACGACC TCAGCTGACT GTTGTTGAGT ACTTTGCACA
ACGGTACAAT GTCCAGCTGC GCTACACCTT GGCCCTGCCT GCAGTCGGGC AATGATTCAA
GCCGATATAT TACCAATGGA GGTTGCAAAA TCATTGAAGG GCAGAAGTAC CCTAGGCTCA
GCGACACACA GGTGGCCAAC ATACTGAATG TAACTGCTTA TTGTGATTCT CCAGAGTTAC
TGGTTAT--- ---TATGGAA ATTAAGAGGA TGTGCGAGAC TGACCTTGGT ATAGTATCTC
AGTGCATCAA TCCAAGAAG- -----AAAAC AACAGTATTT TGAAAATCTT GCCCTTAAAA
TCAATGTGAA GCTGGAGGGC GCAATACAGT GCTAGAGCCT CTGTGCCATA CCTTGTCTCA
GACCAACAAT CATTTTTGGT GCTGATGTTA CCCATCCGCA GGAGATCGGC TTCTGTTGGA
GCTGTGGTTG CATCCATGGA CTGGCCACAG GTCACAACAT ATAAAGCTCT GGTCTCGGCA
CAAGCACACA GGGAAGAGAT TATACAAAAT CTC------- ----GGCGGA ATGATAAGGG
AGTTGCTGAT TTCGTTCTAT AAGAGGACTG GAAAAGCCCA AAAGGATTAT ATTTTACAGG
GATGGAATAA GTGAAGGACA ATTCAACCAT GTTTTGCTCC TTGAAATGGA CGCGATAAGG
AAGGCTGTGC CTCTCTGAAG ATGGTATCTA CCCCCAGTGA CATTTGTCGT AATACAGAAA
AGGCACCACA CAAGGCTCTT CCCTTCATGG AAGAGTGGGA ACATTCTTCC TGGAACTGTG
GTTGATACCG AGATTTGTCA TCCGCGGGAG TTTGATTTCT ACCTTTGTAG CCATGCTGGA

ATTCAGGGAA CCAGCAGGCC AATACACTAT CATGTCCTCT ACGATGAAAA CCGTTTCTCG
GCTGATGGGC TGCAGATACT CACAAACAGC CTGTGCTACA CATACGCACG ATGCACGCGC
GCTGTCTCAG TTGTTCCACC AGCCTACTAC GCTCACCTGG CAGCATTCCG CGGGAGGTAC
TACGACGAA
>AGO1907_Aquilegia
---------- ---------- ---------- ---------- ---------- ----------
---------- ---------- ---------- ---------- ---------- ----------
---------- ---------- ---------- ---------- ---------- ----------
---------- ---------- ---------- ---------- ---------- ----------
---------- ---------- ---------- ---------- ---------- ----------
---------- ---------- ---------- ---------- ---------- ----------
---------- ---------- ---------- ---------- ---------- ----------
---------- ---------- ---------- ---CTGCTGA TTGTTATTTT CCCGAAATAA
TGGTTCT--- CTTTATGGAA GTGAAGCGAA TCTGTGAGAC ACAACTTGGG ATAATCTCAC
AGTGCATCTT AGCAGAACTG TTGAAAAAAT GTCATACTTT GGCTAACATT GTTCTAAAGA
TAAACACCAA GTTGGGGGGA TAAACGTTGT GCT------- -----AGATA CCTGGTCAGT
GACCAACAAT AATTTTTGGG GCTGATGTAA CTCATCCCCT GGGGAAGCCC TTCGATAGCA
GCAGTTGTGG CTTCTCAAAA CTGGCCAAAT GTCACAAATT ATATTCCTAT ACTGTCAGCA
CAACTTGGTC GTGAGGAAAA GATCCTAGAT CTG------- ----GAAAGA ATGGCCAAGG
AACATTTTCA CGCTTTTGAG AAGAACAATC AGGAGGCCTG AAAGGATCAT ATTCTACAGG
GATGGTGTTA GTGACGGACA ATTTAATCAA GTCCGCGAGT ATGAGCTAGA AGCAATTCGG
GAGGCAGGAA TGAATTaagG ATTCGTTGTG CCTCCTATAA CATTTGTGGT GGTCCAAAAA
CGACATCATA CCAAGTTATT TCCACCATGA TGAAGCGGAA ATATATATCC TGGAACTGTG
GTGGATTCAG ATATCTGCCA CCCAACACAA TTCAACTTCT ACTTGTGTAG CCATGCTGGT
ATTCAGGCCA CGAGCCGTCC TGCTCATTAC CATGTTCTAT GTGATGATAA TCAGTTCACG
GCAGATCAAC TTCAAACCCT CACCAATAAC CTGTGTTACA TATATGCAAG GTGCACACGT
TCGGTCTCCT ATGTGTCACC AGCATATTAT GCTCATCTGG CTGCTTTTCG TGCACGATTC
TACTTTGAG
>Sbicolor_Sb03g011020
TATTAGTAAG GCAATCATTC TTCCATAATC CTTCGACCTG GGTGTAGTGG GCTGTAGAGG
GTTTCATTCT AGTTTTCGTG CAACCCAGAG TGGCTTTCAC TCAACATCGA TTGTCCACCA
CAATGATAGT GAAACCCCTG TATTGATTTT CCAATCAGAA GATTGAAGGC CAAGCGTTCA
CTGAAGAACT TGAGGATCAA AACAAGTCCA GCGAACAGAA GATTGTTGGT CTCAGGACCT
TGCTGTGAGC ATTATTCACA CTGAAACAGA AGAGATCACT GTTTATGATT ACTTCGTAAA
GAACCGTGGC ATAGAGCTGC AATACTCATC TTCCATGTAT CAATGTGGGA AAACCAAACG
GCCAACATAT TTCCAGTTGA GTTTGCAGTC TTTTGCCTTT ACAAAGGTAC ACTAAATTGA
GCACACTTCA GAGGTCATCA CTTGTTGAAG GCATTTCTTT TGTGTGTTCT GCTGAAGAAA
GAATTCTGAT ATTTATGGCT TGGAAGAAGA AATGCCTTGC TGAATTTGGG ATCGTTACAC
AGTGCGTGGC ACCACTAGAG TCAATGA--- --CAGTATCT TACAAATGTC CTACTAAAGA
TAAATGCAAA GTGGGTGGCA TGAATTCGTT GCTGAAACAT CCCCAGCATT CCAAGTATCC
AACCAACTAT AATCTTGGGA ATGGATGTCT CCCATGGCCT GGACAGTACC GTCCATTGCT
GCTGTTGTTA GTTCTCGTGA ATGGCCTCTT ATCTCGAAAT ATAGAGCTTC TGTCCGCACC
CAATCACCTA AGATGGAAAT GATTGACTCT TTGTTTAAGA AGATGATGGT CTGATCCGGG
AGTGTCTGAT TGACTTCTAC ACCAGTTCTG GAGAAGCCTG ACCAAGTCAT CATCTTCAGG
GATGGTGTTA GTGAAAGTCA GTTTAATCAG GTGCTGAACA TTGAGTTGCA ACAAATCATC
GAGGCTGCAA GTTTTTGATG AAAATGGAAT CCAAAGTTCA CGTTGATTAT TGCCCAGAAG
AATCACCACA CTAAATTTTT TATGAAAGAC AGA------A ATGTTCCAGC TGGAACTGTT
GTGGACAACA AAGTTTGTCA TCCAAGGAAC TTTGACTTCT ACATGTGTTC ACATGCTGGA
ATGATCGGGA CTACGAGGCC AACTCATTAT CATATACTGC ATGATGAGAT AGGCTTCAAT
CCTGATGATC TGCAGGAGCT GGTGCATTCG CTCTCTTACG TGTACCAAAG GAGCACAACA
GCCATATCAG TTGTTGCTCC CATCTGCTAT GCACATCTGG CAGCAGCTCA GGTCGGCCAG
TTCATTAAG
>Gmax_Glyma06g47230
TCCTTGTTCG CCAATCCTTC TTTCACAATC GAAGGATATA GGTGTGCAAG GGTGCCGCGG
TTTTCATTCA AGCTTTCGAG TCACACAAGG AGGTTATCTC TCAACATGGA TTGACAACCA
CAATGATTGT AAAGCCCCTG TGTGGACTTC AGAATCAGAG GATTGAAGGC AAAGAGAATG

CTGAAGAACC TAAGAATCAG GGCTAATGGT GTGAGTTCAA AATCTCTGGA TTGAGACACT
TGCAGGAATC AAAATTTCTT TTAAGGCAAG GGAAATCACA GTTCATGACT ACTTTACACG
CCAAAAACTG ATTGGCCTGA ACTATTCACA TGCCATGCAT CAATGTTGGC AAACCAAACG
CCCGTCTTAT TTCCAATAGA GCTTGTGAAA TGGTCTCGTT GCAGCGTTAC ACCAAGCTAA
CAAATTTGCA AAGGGCTCAG CTAGTGGAAG GCATTTCTGT TGTGTATTCT CCAGAAAAAA
GAATTCTGAC ATTTATGGCT TGGAAGAAGA AAAGTCTTGT TGAAGAAGGG ATTGTAACAC
AATGTATTGC ACCACAAAGA TTAATGA--- --CAATACAT TACTAATGTA CTTTTGAAAA
TTAATGCAAA GATGGTGGGA TGAATTCGTA TCTGAGCTTT GTAATTCATT CCTTGTTTCA
GCCCAACCTT GATTCTTGGT ATGGACGTTT CTCATGGCCT GGTCGGTGCC CTCTATTGCT
GCGGTTGTGA GCTCAAGATG TTGGCCTCAA ATTTCACGTT ATAGAGCTTC AGTTCGCACA
CAATCATCAA AAGTTGAGAT GATTCAATCT CTATTCAAAA GGATGAAGGT ATCATCAGGG
AGGTGCTGTT GGACTTTGAA ATTACTTCAT TGAAAGCCTC AACAAATTAT AATTTTCAGG
GATGGAGTGA GTGAATCACA GTTCAACCAG GTGCTCAATA TTGAGTTAAG TCAAATCATT
GAGGCTGCAA ACACCTGATG AAAATGGGAT CCAAAGTTCA CTTTGATTAT TGCCCAGAAG
AATCATCATA CTAGGTTCTT TCAATGCTag agaCAAACTA ATGTTCCACC AGGAACCGTT
ATTGACAACA CTGTCTGTCA TCCCAAAAAC AATGACTTCT ACTTGTGTGC TCAAGCTGGG
ATGATTGGGA CAACTCGACC TACTCACTAC CATGTTTTAC ACGATGAAAT CGGATTCTCA
GCTGATGAAG TGCAAGAATT AGTGCATTCA TTGTCTTATA CGTATCAGAG GAGCACAACT
GCTGTATCAT TAGTCGCTCC CATTTGCTAT GCCCACTTGG CAGCAGCCCA GATGGCACAA
TTTATGAAG
>AGO1904_Aquilegia
---------- ---------- ---------- ---------- ---------- ----------
---------- ---------- ---------- ---------- ---------- ----------
---------- ---------- ---------- ---------- ---------- ----------
---------- ---------- ---------- ---------- ---------- ----------
---------- ---------- ---------- ---------- ---------- ----------
---------- ---------- ---------- ---------- ---------- ----------
---------- ---------- ---------- ---------- ---------- ----------
---------- ---------- ---------- ---CTTCTGA TTGTTATATT CCTGAAACAA
CGGCCCT--- TTGTATGGAG CTGAAGCGAA TATGTGAGAC AGACCTAGGA CTTGTATCAC
AGTGTTGTCT GACAAACATG TGTTTAAAAT AACAGTATCT GGCAAACGTA GCCCTAAAGA
TAAATGTAAA GTTGGGGGGC GAAACACAGT GCTGATGCGC TTTCTAGATG CCTGGTCAGT
GACCAACAAT CATCTTTGGT GCTGACGTAA CCCATCCCCT GGAGAAGCCC CTCTATCGCA
GCTGTTGTTG CATCCCAAGA TTGGCCTGAG GTCACAAAGT ATGCTGGTTT GGTTTGTGCT
CAAGCTCATC GCCAGGAATT GATCCAAGAT CTGTATAAAT GACAGGAGGC ATGATCAAGG
ATCTTTTGAG ATCTTTTCAT AAAGCTACTG GGAAAACCTT CACGAATCAT ATTCTACAGG
GATGGAGTCA GTGAAGGACA ATTTTATCAA GTCTTGCTAT ATGAACTGGA TGCGATTCGT
AAGGCTGTGC TTCTCTGAGC CAAATATCAG CCCAGGGTGA CATTTGTCGT TGTTCAAAAG
CGCCATCATA CAAGGTTGTT TGCATCATGC TGAAGCGGAA ATATATTACC AGGTACGGTG
GTGGACTCCA AGATTTGCCA TCCAACTGAA TTTGACTTTT ATTTGTGTAG CCATGCTGGA
ATACAGGGCA CAAGCCGTCC TGCCCATTAT CATGTATTAT GGGATGAGAA CAACTTTACT
GCCGATGGAC TCCAGTCACT CACGAATAAC CTCTGTTATA CGTATGCAAG GTGCACCCGT
TCTGTTTCTA TAGTCCCCCC AGCATACTAC GCTCATTTGG CAGCTTTCCG TGCACGTTTT
TACATGGAG
>Sbicolor_Sb10g023230
TGTCCATAGG GCGGTCCTTC TACTCGGACA TCAGCGGCTA GGCCTGCAGT CGTGGTGTGG
GTTCTACCAG AGCATCCGGC CGACACAGAT GGGTTGTCGC TTAACATCGA CTGTCGTCCA
CGGCGTTCAT TGAACCCCGG TATCGAGTTC AGATATTAGG AACCGAAGAT CAAGAAGGCA
TTGCGAGGAG TAAAAGTTGA GGTCACTCAC CGAAGTATCG CATTTCTGGG TTGACAACCA
ACTCATGAAC TATTTTCCCA ATTGATGAAA TATGAAATCT GTCGTGGAGT ACTTCAAGGA
AATGTATGGT TTCACCATTC AGCATCCATC TTCCCTGCCT TCAGGTTGGG AACCAAAAAA
GGCGAACTAT TACCGATGGA GGCTGCAAGA TCGTTGAAGG CCAGAGATAC ACGAAGCTGA
ATGAAAAACA GATCACATCA TTGCTGAATG CCACTTCTTT TGGCTATCCT CCTGAAACAA
TGGTCCG--- TTATATGGAC ATCAAACGTA TTTGTGAAAC TGATTTGGGA TTGATAACAC
AATGTTGCTT GACAAACATG TTTTTAAAGC AACAGTACTT GGCAAATGTC TCACTGAAAA
TTAATGTTAA GTGGGAGGAA GAAATACTGT GCTGATGCAA TAAGTTGATT CCTGGTCAGT

GACCAACTAT TATATTTGGT GCGGATGTAA CACATCCACT GGGGAAGTCC ATCAATTGCT
GCCGTTGTTG CTTCTCAAGA TTGGCCAGAA GTTACAAAGT ATGCTGGATT GGTTTGTGCT
CAGGCACACC GGCAAGAGCT CATTCAGGAC CTTTACAAGT AACAGGCGGC ATGATCAGGG
AGCTCTTAAT ATCCTTCAGG AAGGCCACTG GAGAAGCCAT TGAGAATAAT ATTCTACAGG
GACGGTGTTA GTGAAGGCCA GTTCTACCAA GTTCTCCTTT ACGAGTTAGA TGCCATCCGT
AAGGCTGTGC GTCCCTGAAC CAAATACCAG CCTCCTGTAA CATTTGTGGT AGTTCAAAAA
CGTCATCATA CAAGACTTTT TGCATCACAA AGAAGTGGAA ATATTTTGCC AGGAACCGTT
GTTGATTCTA AGATATGCCA CCCAACGGAG TTTGATTTCT ACCTCTGCAG TCATGCTGGA
ATTCAGGGAA CAAGTAGGCC TGCTCACTAC CATGTCCTCT GGGATGAGAA CAATTTCACA
GCAGACGAAA TGCAGACATT GACAAACAAC CTTTGCTACA CTTATGCCCG GTGCACACGC
TCGGTTTCTG TTGTCCCTCC TGCATACTAC GCACACCTGG CAGCATTCCG GGCTCGGTTC
TACATGGAA
>Mguttatus_mgf016487m
TTCTAGTACG ACAGTCCTTT TTCCACGAGC CAAGGACTTG GGTGTTGTTG GTTGTCGGGG
TTTTCATTCG AGCTTTAGGG CAACACAAGG AGGCTTTCGT TGAATATGGA TTTTCGACAA
CTATGATTGT CAAGCCCCTG TCTGGACTTT CTAATCAACG GATTGAAGGC GAAAAGGACG
TTGAAGAATC TTAGGATCAG GGCCACTAAT TCGAGTACAA GATCAGTGGA CTGAGCGATA
TGCAGGAAGC AACGTTTCCT CTAAAACAGT ACAAATCACA GTCTACGACT ACTTCGTGAA
CCACCGAGAT ATCAAACTGA GATACTCATT TTCCATGTAT CAATGCAGGC AAACCAAACG
CCCCACTTAT TCCCCATTGA GCTTGTGAAT TAGTTTCTTT GCAACGATAT ACCAAATTGT
CGAACCTGCA ACGAGCTTCG CTTGTGGAAG ACACTTCTTC TTTGCATATT CCGGAAAGAA
AAACTGCGAT CTCTATGGCA TGGAAGAAGA AGAATCTTTC TGACATGGGG ATAGTCACTC
AATGCATGGC ACCAAACCTG TCAATGA--- --CAGTACAC CACCAATCTG CTCCTAAAGA
TCAATGCCAA GTTGGCGGCA TTAATTCACT TTTGAAAAAT CGCCCTCATT CCTGGTTTCC
ACCCCACCCT TATTGTCGGG ATGGACGTTT CACATGGCCT GGTCGGTTCC ATCTATTGCT
GCTGTGGTAA GTTCAAGGCA GTGGCCTTTG ATATCTAAGT ACAGGGCTGC AGTTCGAACC
CAGTCACCAA AACTAGAAAT GATCGATTCG TTGTTCAAGT GGATCAGGGC ATTTTCAGGG
AGTTATTGCA GGACTTCTTC ATAAGTTCCG GGAAAACCCG AGCAGATTAT CATCTTTAGG
GATGGAGTGA GCGAGTCGCA GTTCAATCAA GTATTGAATA TAGAGCTGGA ACAAATCATC
GAGGCTGCAA ATTTCTGACG AAACTGGTCT CCCAAATTCA TGGTTGTGGT GGCACAAAAG
AACCATCACA CGAAATTTTT CCAACGGACC CGA------A ATGTTCCACC AGGCACTGTA
ATTGACAATG GAATTTGCCA TCCAAGAACA AACGACTTCT ACATGTGTGC TCACGCTGGA
ATGATTGGCA CAACTAGGCC AACACACTAC CATGTGCTAT TTGACGAGCT CGGCTTCTCT
GCCGATGCTT TACAAGAACT TGTGCACTCA TTATCCTATG TGTACCAGAG AAGTACTACA
GCCATCTCTG TCGTTGCTCC GATCTGCTAC GCCCACTTGG CCGCTGCACA AATGTCTCAA
TTCATAAAG
>LOC_Os01g16850
GCCCATCGTA CCCCTCAGCA atggactgga tgaaccgaaa GAGAAAATCT CTTGTCGGGG
ATTTCACTCC AGCTTTCGAC CCACTGACAG TGGCTGTCAC TGAATGTTGA TTATCCACGA
CGATGATCGT CAGACCccag tgtggagttc gcgaccagcc aagtgCAGGC CAAGTGTGCA
CTGAAGAACC TGAGGATAAA AACGACTCAC ACGAATTTAG GATCATCGGT TTGTCACACT
TGCTATTCGC AACGTTCCAA ATAAAGAGGA AGAAGTGACA GTCTTTGAAT ACTACAGGAA
GAATTGGAAA ATAGATTTGA AGGGATCACT TTCCCTGTCT AAATGTTGGG AAGCCAAACG
GCCAACATAT TCCCATTGGA GCTTGCCATT TGGTGCCATT GCAAAGGTAC AAAAAGTTGT
CGACGTTACA GCGGTCCACG TTGGTTGAAG GCAgccTTT- ---------- ----------
-TCTGCAGAT TCC---GGCA TGGAAGCGTA TGTGTCTCGT CAAATACGGT ATTGTAACAC
AATGCTTG-- -GC---CCTA CC---AAAAC GACAGTACCT GACTAATGTG CTTCTAAAGA
TAAATGCAAA GTTGGAGGGT TGAATTCGCT GCTGAAAGAA ACCAAGCATT CCTCTTGTCG
AACCAACCAT TATCTTAGGC ATGGATGTTT CCCATGGCCG GGACGGTACC GTCTGTCGCT
GCGGTTGTTA GTTCCCTGGA GTGGCCTCTC ATATCAAAAT ATAAAGCCTC CGTATGCACC
CAGTCTCCCA GGCTAGAAAT GATCGATTCC TTGTTTAA-- ---------- ----------
---------- ---------- ---------- ---------G ATCATGTTAT CATT---AGA
GATGGGGTTA GTGAAGGCCA GTTTAATCAG GTGCTGAACA TTGAGCTAGC CCAGATAATC
AAGGCTGCGA GTTTCTGACA GTGATGGTCT CCAAAGTTCA CGGTGATAGT TGCGCAGAAG
AACCATCACA CCAAATTTTT TCAATCGATC GAAgttgtcA ATGTTCCTCC TGGTACTGTT
GTTGACAAAG GAATCTGTCA TCCCAGGAAC TGTGATTTCT ACATGTGTGC TCATGCTGGG

ATGATTGGGA CTACAAGGCC GACGCATTAC CATGTGCTGC ATGATGAGAA CAATTTCACC
CCTGATGACT TGCAGGAGCT TGTGCACAAC CTCTCATACG TGTACCAGAG GAGCACGACG
GCCATCTCAG GTGTCGCTCC GATCTGCTAC GCGCACCTGG CGGCGGCGCA GGTGTCGCAG
TTCGTGAGA
>Smoellindorffii_15405134_locus
CGCCTGTTGG ACGATCGTTT TATTCAGACT TAGGCCCCTC GGCTTAGAAA GCTGGCGAGG
GTTCTACCAG AGTATAAGGC CCACGCAAAT GGGCTTTCTC TGAATATTGA CTGTCATCTA
CTGCTTTCAT TGAGCCCCCG TGTGGACTTT AACTCCTTAA GACCGAAGAT CAAAAAAGCT
CTGAGAGGGG TCAAAGTGGA AGTTACACAT CGAAATACAG GATTTCTGGC TTAACAGCCT
ACTCAAGAAC TATGTTTCCT GTTGATGAAC AATGAAATCG GTAATGGAGT ATTTCCGAGA
TACATATCAC TACACTATAC GAAGCCCCTT TACCTTGTTT GCAAGTTGGA AATCAAGAAG
ACCAAATTAT TGCCAATGGA GGTTGCAAGA TTGTGGAAGG ACAAAGGTAT ACTAAACTCA
ACGAGCGTCA AGTGACAGCC CTTCTAAATG CCATTACTAA TTGCCATATT CCCGAAACAA
TGGCTCA--- CTTTATGGAC TTGAAACGAA TCTGCGAAAC AGACCTGGGA TTGGTTTCTC
AATGCTGCTT GACAAGCATG TCTTTAAGGA AACAGTATCT TGCTAATGTT GCGTTGAAAA
TCAATGTCAA GTCGGAGGCA GAAACACTGT CTTGATGCAC TATCACGCTT CCTAGTAAGT
GACCAACAAT TATATTTGGA GCGGATGTTA CGCATCCCCT GGAGAAGTCC CTCGATTGCA
GCGGTTGTCG CTTCTCAGGA TTGGCCAGAA GTAACGAAAT ACGCAGGTCT AGTCTGCGCT
CAAGCTCACA GACAAGAGTT GATCCAAGAT TTGTATAAAT GAATGGCGGT ATGATAAGAG
AGCTTTTAAT TTCTTTCCGA AGTGCCTCTG GACAAACCTG GAAGAATCAT CTTCTACAGA
GATGGTGTGA GCGAAGGACA GTTCTACCAA GTTCTCCTTC ACGAGCTGGA TGCAATTAGA
AAGGCTGCGC GTCACTGAGC CGAATATCAG CCTCTCGTGA CGTTCGTTGT GGTCCAGAAA
CGGCACCACA CCCGACTATT TGCATCACGA CGAAGTGGAA ATATCCTGCC AGGCACTGTG
GTGGATTCGA AGATTTGCCA TCCAACGGAG TTTGACTTCT ACCTTTGCAG CCATGCCGGC
ATTCAGGGAA CAAGCAGACC AGCTCACTAT CACGTACTTT GGGACGAGAA CAAGTTCACT
GCCGATGGCT TGCAGTCCCT CACCAACAGC CTGTGCTACA CTTACGCTCG CTGCACACGC
TCAGTCTCGA TAGTACCACC TGCATACTAC GCTCACCTGG CTGCGTTCCG CGCGAGATTC
TACATGGAG
>AGO24601_Amborella trichopoda
---------- ---------- ---------- ---------- ---------- ----------
---------- ---------- ---------- ---------- ---------- ----------
---------- ---------- ---------- ---------- ---------- ----------
---------- ---------- ---------- ---------- ---------- ----------
---------- ---------- ---------- ---------- ---------- ----------
---------- ---------- ---------- ---------- -CAAGTCGGG AATCATCACG
CCCAAATTAC TCCCAATGGA GGTTGCCAAA TCGTTGAGGG CCAACGATAC TCAAAGTTGA
ACGAAAGACA AATCACTGCC CTTCTGAATG CCACTGCTGA TTGTAATCTT CCTGAAATAA
TGGTTCC--- CTTTATGGAT CTAAAGAGAA TTTGCGAGAC GGATTTGGGC TTGGTCTCCC
AATGCTGCTT AACAAGCATG TATTTAAAGC AACAGTATCT TGCTAATGTT GCTTTGAAAA
TCAATGTTAA GTCGGTGGGA GGAATACTGT ACTGATGCGA TTGCAAGATT CCTTGTAAGT
GACCAACTAT AATATTTGGG GCTGATGTCA CTCATCCCCA GGAGAAGCGC ATCCATTGCT
GCTGTCGTTG GTTCTCAGGA CTGGCCAGAG GTGACTAAAT ATGCTGGATT GGTCTGTGCT
CAAGCTCACA GACAAGAATT GATCCAAGAT CTTTATAAGT GACGGGTGGC ATGATCAAGG
AGTTGCTCAT TTCATTCAGG AGAGCAACTG GAAAAGCCTC AGAGGATTAT TTTCTACAGG
GATGGAGTTA GCGAGGGCCA GTTTTACCAG GTTTTGTTTC ATGAATTGGA TGCTATTCGC
AAGGCTGTTC CTCCCTGAGC CAAATATCAG CCCCCTGTGA CATTTGTTGT GGTTCAAAAG
CGGCATCACA CTCGGCTATT TGCATCATAA GGAAGTGGAA ATATATTACC CGGAACTGTG
GTGGATTCAA AGATCTGCCA TCCCACTGAA TTTGATTTTT ACTTATGCAG TCATGCTGGC
ATCCAGGGTA CTAGCCGCCC TGCCCATTAC CATGTTTTGT TTGATGAGAA TCACTTCACA
GCCGATGGCC TCCAGTCGCT CACTAATAAT CTGTGCTATA CGTATGCACG TTGCACCCGC
TCTGTCTCTA TTGTGCCGCC TGCTTACTAT GCTCATCTAG CAGCCTTCAG GGCTCGCTTC
TACATGGAG
>Contig2618_radiata
---------- ---------- ---------- ---------- ---------- ----------
---------- ---------- ---------- ---------- ---------- ----------
---------- ---------- ---------- ---------- ---------- ----------

---------- ---------- ---------- ---------- ---------- ----------
---------- ---------- ---------- ---------- ---------- ----------
---------- ---------- ---------- ---------- ---------- ----------
---------- ---------- ---------- ---------- ---------- ----------
---------- ---------- ---------- ---CTTCTTG TTGTTATTTT CCTGAAACAA
TGGTTCT--- TTGTATGGAC CTGAAGCGTA TATGTGAGAC TGACCTTGGT TTGGTTTCCC
AGTGCTGTCT GACAAACATG TCTTTAAAGC AACAATATCT GGCAAATGTT GCCCTTAAAA
TTAATGTGAA GTTGGTGGAA GAAACACTGT CTTGACGCAC TGTCAAGATA CCTGGTCAGC
GACCTACAAT AATCTTTGGA GCAGATGTTA CCCATCCCCT GGAGAAGCCC GTCAATAGCT
GCTGTTGTAG CGTCTCAAGA TTGGCCGGAA GTCACTAAAT ATGCAGGCTT AGTTTGTGCT
CAGGCACATC GTCAGGAACT CATTCAGGAT CTTTACAAGC AACTGGAGGC ATGATAAAGG
AATTGTTAAT TTCCTTCAGA AGGAATACAG GACAAGCCGG AGCGTATAAT ATTTTACAGA
GATGGAGTAA GCGAGGGTCA GTTTTATCAA GTTCTATTGT ATGAGTTAGA T---------
---------- ---------- ---------- ---------- ---------- ----------
---------- ---------- ---------- ---------- ---------- -GGC------
---------- ---------- ---------- ---------- ---------- ----------
---------- ---------- ---------- ---------- ---------- ----------
---------- ---------- ---------- ---------- ---------- ----------
---------- ---------- ---------- ---------- ---------- ----------
TATTCGAAA
>Gmax_Glyma10g38770
GCCCTATTGG GAGGTCCTTC TTTTCTGATA TTAGCGGCTT GGATTAGAAT CATGGTGTGG
ATTTTACCAG AGTATAAGGC CTACACAAAT GGGCTTTCCC TTAATATTGA TTGGCGTCTG
CTGCGTTTAT TGAGCCCCAG TGTGGAATTT AGCTATTAGC GATCGAAGAT TAAGAAAGCC
CTTAGAGGAG TTAAAGTTGA AGTAACACAC AGAAATATCG TGTTTCTGGA TTGACAACCA
ACCAGAGAAC TGTGTTTCCT GTTGATGAAC TATGAAATCA GTAGTTGAAT ACTTCCAAGA
GATGTATGGT TTCACTATTC AATATACACC TTCCTTGCCT TCAAGTAGGA AACCAAAAAA
GGCTAACTAT TACCTATGGA GGCTGCAAAA TTGTTGAGGG GCAACGTTAT ACAAAATTGA
ATGAGAAGCA AATTACAGCT CTGTTGAATG CCACTTTTGT TAGCAATATT CCAGAAATAA
CGGGTCT--- CTCTATGGAT CTCAAGCGAA TTTGTGAAAC TGACCTTGGT TTAATTTCAC
AATGCTGTCT GACAAGCATG TCTTCAAACT AACAGTACTT GGCTAATGTG TCTCTGAAGA
TCAATGTGAA GTGGGAGGTA GAAACACTGT ACTGATGCTG TAAGCAGATA CCTGGTTAGT
GACCAACCAT AATTTTCGGA GCAGATGTAA CCCACCCAAT GGAGAAGCCC TTCAATAGCA
GCTGTAGTCG CATCCCAGGA CTGGCCCGAA GTGACAAAAT ATGCCGGTTT AGTATGTGCT
CAAGCTCATA GGCAGGAACT TATACAAGAT TTGTACAAGT TAGTGGTGGC ATGATCCGAG
ATTTACTGGT TTCCTTCAGA AAGGCAACAG GAAAAGCCAC TACGAATTAT ATTTTACAGG
GATGGTGTAA GTGAAGGACA ATTTTACCAA GTTTTACTTT ATGAGTTAGA TGCAATTCGG
AAGGCTGTGC TTCCTTGAAC CAAATACCAG CCTCCAGTAA CTTTCATAGT TGTGCAAAAA
AGACATCATA CCCGGTTATT TGCACTACAG GGAAGTGGGA ATATATTGCC TGGGACTGTT
GTTGATACCA AAATCTGCCA TCCAACAGAA TTTGATTTTT ATCTCTGCAG CCATGCTGGC
ATCCAGGGTA CTAGTCGGCC AGCTCATTAT CATGTCCTGT GGGATGAAAA CAACTTCACA
CCTGATGGAA TTCAGTCTCT GACAAACAAC CTTTGTTATA CATATGCCAG GTGTACACGC
TCAGTATCAG TTGTTCCTCC AGCATATTAT GCACATTTAG CAGCGTTTCG AGCACGTTTC
TATATGGAA
>Gmax_Glyma20g12070
TACTTGTACG CCAATCCTTT TTCCATAATC CAAAGATGTA GGTGTCCTAG GCTGTAGAGG
ATTCCACTCA AGCTTTAGAA CTACACAGAG TGGCTGTCTC TTAACATAGA TTGTCAACTA
CAATGATAAT TTCTCCCCTG TGTGGATTTC CCAATCAAAA GACTGAAGGC CAAAAGGACC
CTAAAAAATC TGAGGATTAA AACTAGCCCA TCGAATTCAA AATTTCTGGG CTCAGTCCCA
TGCAGAGAGC AACTTTTACT TTGAAAGGGA GGAAATCACT GTATATGATT ATTTTGTTAA
GGTTCGTAAG ATAGATCTCC GATACTCACC TTCCATGTAT CAATGTTGGC AAGCCTAACG
ACCAACATTT TCCCCATTGA GGTTGTGAAT TGGTATCATT GCAACGATAT ACAAAACTGT
CCACGCTTCA AAGGGCTTCA TTAGTGGAAG GCATTCCTTC TCTGTTTGCT CCTGACGGAA
AAATTGTGAT ATTTATGGCA TGGAAAAAGA AGAATCTTGC TGATTTTGGA ATCATAAATC
AGTGTATGTG TCCTTAAGGG TCAATGA--- --CAGTACCT GACTAATGTT ATGTTGAAGA
TCAATGCCAA GTTGGTGGGT TGAATTCATT GTTGAACATT CTCCTTCCTT CCTTGTTTCC

AACCCACCCT CATTCTGGGA ATGGACGTGT CACATGGCCT GGGCAATTCC TTCAATTGCT
GCGGTGGTCA GCTCTAGACA CTGGCCTCTG ATATCAAAGT ATAGGGCATG TGTTCGTACG
CAATCTGCAA AGATGGAAAT GATTGATAAT TTGTTCAAGA AGATGAAGGC ATCATAAGGG
AACTTTTGCT TGATTTCTAT ACAACTTCTG GGAAAACCGG AAAATATAAT CATATTCAGG
GATGGGGTTA GTGAGTCACA ATTCAATCAA GTTTTGAATA TTGAACTCGA TCGAATCATT
GAGGCTGCAA ATTTCTGATG AAAATGGGAG CCAAAATTTG TGGTAATTGT TGCTCAGAAG
AACCACCACA CTAGATTTTT CCAGCTCTCC CGA------A ATGTCCCACC TGGCaagtgt
tctgggtctt tacagcattt agggagtTGT GAAAAAGGTG CCCCTTCACC TGCCCTCAAT
TACTGTGGAA CTAGTAGGCC TACCCATTAT CATGTGCTGC TTGATCAGGT TGGTTTCTCT
CCGGATCAGC TGCAGGAGCT TGTCCATTCA TTATCATATG TGTATCAGAG GAGCACTACT
GCCATTTCTG TTGTTGCTCC AATATGCTAT GCGCACTTGG CTGCTACTCA GTTGGGGCAG
TTCATGAAA
>GRMZM2G153859_T04 cds:PUTATIVE_protein_coding
TATTAGTAAG GCAATCATTC TTCCACAATC CTTCGACCTG GGTGTAGTGG GCTGTAGAGG
TTTTCATTCT AGTTTTCGAG CAACCCAGAG TGGCTTTCAC TCAATATCGA TTGTCGACTA
CAATGATAGT GAAACCCCTG TATTGATTTT ACAATCAGAA GATTGAAGGC CAAGCGTGCA
CTGAAGAACT TGAGGATAAA AATAAGTCCA GCGAACAGAA GATTGTTGGT CTCAGGAACT
TGTCGTGAGC ATTATTCACA CTGAAACAGA AGAGATCACT GTTTATGATT ACTTCGTAAA
GCAGCGTGGC ATAGTGCTGC AATACTCATC TTCCTTGCAT CAATGTGGGA AAACTAAACG
GCCAACATAT TTCCAATTGA GTTTGCAGTC TTGTGCCTTT ACAAAGATAC ACTAAATTGA
ACACACTTCA GAGGTCATCA CTCGTGGAAG GCATTTCTTT TGTGTGTTCT GCTGAAGGAA
GAATTCTGAT ATTTATGGCT TGGAAGAAGA AATGCCTTGC TGAATTTGGG ATCGTTACAC
AATGTGTGGC ACCACTAGAG TGAACGA--- --CAGTATCT TACAAATGTC CTACTTAAGA
TAAATGCAAA GTGGGTGGCA TGAATTCGTT GCTGAAACAT CCCCAGCATT CCTTGTATCC
AACCAACTAT AATCTTGGGA ATGGATGTGT CACACGGCCT GGACAGTACC ATCTATTGCT
GCTGTTGTTA GTTCTCGTGA ATGGCCTCTT ATCTCGAAAT ACAGAGCTTC TGTCCGCACC
CAATCACCTA AAATGGAAAT GATTGACTCA TTGTTTAAGA AGATGATGGT CTGATCCGGG
AGTGTCTGAT TGACTTCTAC ACCAGTTCTG GGAAAGCCTG ACCAAGTTAT CATATTCAGG
GACGGTGTTA GCGAAAGTCA GTTTAATCAG GTGCTGAACA TTGAGTTGCA ACAAATCATC
GAGGCTGCAA ATTTCTGATG AGAATGGAAT CCCAAGTTCA CGTTGATTAT TGCCCAGAAG
AATCATCACA CTAAATTTTT CATGAAAGCC AGA------A ATGTCCCACC AGGAACTGTG
GTGGACAACA AAGTCTGCCA TCCAAAGAAC TTCGATTTCT ACATGTGTGC GCATGCTGGA
ATGATCGGGA CTACGAGGCC AACTCACTAC CACATCCTGC ATGATGAGAT AGGCTTCAGT
CCTGATGATC TGCAGGAGCT GGTGCATTCG CTCTCTTATG TGTACCAAAG GAGCACAACA
GCCATATCAG TCGTTGCTCC CATCTGCTAC GCACATCTGG CAGCTGCTCA GGTTGGCCAG
TTCATAAAG
>LOC_Os06g39640
TCTCGATAGG GCGGTCGTTC TACTCGGACA TAAGCGGCTC GGCCTACAGT CATGGTGTGG
GTTCTACCAG AGCATCCGGC CGACTCAGAT GGGTTGTCGC TTAACATTGA TTGTCATCTA
CCGCGTTCAT CGAGCCCCAG TATCGAATTC AAATACTAGG AACAGAAGAT CAAGAAAGCC
TTGCGGGGCG TGAAAGTTGA AGTCACTCAC CGAAGTATCG CATTTCAGGG CTGACAACCA
ACTCATGAAC TATTTTCCCA ATCGATGAAA CATGAAATCT GTCGTAGAGT ATTTCAAGGA
AATGTATGGC TTCACCATTC AGCATCCATC TTCCCTGCCT TCAGGTGGGA AACCAAAAAA
GGCAAACTAT TACCAATGGA GGCTGTAAGA TTGTTGAGGG TCAGAGATAT ACAAAGTTGA
ATGAAAAGCA GATCACATCA TTACTCAATG CCGCTTCTTT TGGCCATCCT CCTGAAACAA
TGGTTCT--- TTATATGGAT ATAAAACGTA TATGTGAAAC TGACTTGGGG TTGATATCAC
AATGTTGCTT AACAAACATG TTTTCAAAGC AACAGTACCT GGCAAATGTC TCACTTAAAA
TCAATGTTAA GTGGGAGGAA GAAACACCGT GCTGATGCAA TAAGTTGATT CCTGGTCAGT
GACCAACTAT TATATTTGGT GCAGATGTCA CGCATCCACC GGGGAAGCCC ATCCATTGCT
GCTGTTGTTG CATCTCAAGA CTGGCCAGAA GTTACAAAGT ATGCTGGATT GGTGTGTGCT
CAGGCTCATC GGCAAGAGCT CATTCAAGAT CTTTACAAGT AACAGGAGGC ATGATCAGGG
AGCTCTTAAT ATCCTTCAGG AAGGCCACTG GAAAAGCCAT TGAGAATAAT TTTCTACAGG
GATGGTGTCA GTGAAGGCCA ATTCTACCAA GTTCTCCTCT ATGAGTTGGA TGCTATCCGC
AAGGCTGTGC ATCTCTGAAC CAAATATCAG CCTCCTGTAA CATTCGTGGT TGTCCAAAAG
CGTCACCATA CAAGACTCTT CGCATCACAA AGAAGTGGAA ACATTTTGCC TGGAACTGTT
GTTGATTCAA AGATCTGCCA CCCATCAGAG TTTGATTTCT ACCTCTGTAG CCATGCTGGA

ATTCAGGGAA CAAGTAGGCC AGCTCACTAC CACGTCCTTT GGGATGAGAA CAATTTCACT
GCAGACGAAA TGCAGACATT GACAAACAAC CTTTGCTACA CTTATGCACG GTGCACACGC
TCTGTTTCTG TTGTTCCACC TGCATACTAT GCGCATCTGG CTGCGTTTCG AGCGCGGTTC
TACATGGAG
>Mguttatus_mgf000736m
TCACAGTCGG TCGAAGTTTT TTCTCCGAAT TCCGGACTTT GGACTCGCTG CATACAGAGG
GTTGCAACAG AGTTTGAAGC CTACTTTGCA GGGCTTGCTC TGTGTTTGGA CACTCTGTTT
TAGCATTTCG AAAGCCCCAG TATGGAGTTT AGCATTTTAA CGACGGCTGT CAACGATGCT
TTGAGAGGGT TGAAAGTGAC GGTTACACAC CGAAGTATAC TGTTGCAGGG CTAAGAGGAC
ACCTGTGATA TTACTTTGAT CTTGTTGACA AAGAACTAGC CTTGTTAAGT ATTTCAAGGA
AAAATGGGGT AAGGATATTG TGTACCAATA TACCTTGCTT GGAACTTGGC AAAGGTAGAA
GTCGAACAAA TACCGATGGA GTTTGTGTGT TAGTCGAAGG GCAGAGGTAC CCGAAGAATC
TGGACAGAAA CACGGCTGTG CTTTTGAATC ACTTTGATTG TTTGTGTAAT -----ACCAA
AAAGGACCCG GGCTACAAAT CTCAAGTGGG TTTCCGAGAC AAAACTCGGT GTGGTGACTC
AGTGTTGTCT TTCGGTTCTG CCAGCAACAG GACAGTATTT TGCCAACCTC TGTCTGAAAA
TCAATGCTAA ATAGGTGGCA ATAATTTCGA AGTGGAAACT TTACCCA--- --TCGATGCG
AGCATGTGAT GTTTATAGGA GCTGACGTGA ACCATCCCCT ATGAATGCCC CTCCATAGCT
GCAGTTGTGG CGACCGTTAA CTGGCCAGCT GCAAATAAAT ATGCAGCCAG AGTTAGCCCA
CAGAAACACC GCTGTGAAAA GATCGAAAAT TTC------- ----GGAGCA ATGTGTTTGG
ATTTGGTCAA CACATACGCC AAATTTAATA ATCAAGCCGA AGAGAATCGT GGTGTTTCGT
GATGGTGTTA GTGAGGGGCA GTTCGAAATG GTCCTAAGTC AGGAGCTGTT GGATTTGAAG
AACTCTAT-- ------GACG GTGATATCAG CCGTCGATCA CTCTCGTTGT TGCTCAGAAG
CGTCACCAGA CGAGGCTTTT TATATAATGG AAAATCGGAA ATGTACCTCC TGGAACTGTT
GTGGATACGA GGATTGTGCA TCCGTTCGAT TTTGACTTTT ATCTGTGCAG CCACTATGGA
GCTCTTGGAA CGAGCAAGCC TACTCATTAC TATGTGCTTT GGGATGAGAA TTCGTTTACT
TCTGACCAAT TGCAGAAGCT TATAAACGAC ATGTGCTACA CATTTGTTCG GTGCACTAAA
CCCGTTTCGC TTGTGCCACC TGTCTACTAT GCTGACCTGG TTGCTTACAG GGGAAGAATG
TTCCAAGAG
>Smoellindorffii_15414178_locus
TTCCTGGTGG TCGGTCTTTC TTCTCTAACC TGGGGATCTT GGCCTCGTCG CGTGGCAAGG
CTTCTATATG TCCATCCGTC CAGCACAGGA CAACTCGTCC TTaacATTGA CTGACCGGGA
ATGCGTTTAT CAAGGACGCG A--------- gtaacatgcg TACCGAAGAT GAAGAAGCTG
GTGAAAGGCC TCAAGGTGGA AACAAGTCAC TGAAGCTCAA GATTGTGAGC TTGACAGCCA
CTGGAAACTT TAACTTTAAT ATGAAT--AT GCAAGTTTCC GTAGTCGATT ATTTTCGGCA
AACTTATGGT TTAAACCTGG CATTTGGGGT TTCCTGCTGT GGAGCAAGGA TCCGGTGACG
GAAAAAGTAT TCCCGCTCGA GCTTGCCGAC TCGTTAAGGG CCAGAATTTT ACCAGAGTGA
ATGACGACCA GAGAAAGGGG CTCTCTGCTG C--CTGCTCG TGTGCATTCT CCAGACTGAA
CACTTCTTCC TTCTTCACCC ATCAAGCGCT TATGCGAGCT TGAACTTGGT GTCATAACTC
AATGCGCGCA GGAGGTAAAA TTAGGAAGAT CCCGCTACCT CGCAAACCTT ATTTTAAAAA
TCAATGCCAA GTCGGAGGGA AAAACGCAGT TATCAAGATC TGAAGAAAAG CC--GTAGCA
GACCAACTCT GATAATCGGT GCGGATGTTT CTCATCCGCT GGGGAGGGTG CTCAATGGCA
GCGGTTGTGG CGAGTATGGA TTGGCCCGGG TTTGCGCAGT ATGCGACGGT CGTGAGAAGT
CAGCCTTCGC GACAGGAGAT GTTGGACGAC CTGTTCTGGT CAGTGGTGGC ATTTTCAAGG
AGATGTTGAT GGCATTCCAT CACCGGACGA ATTATCCCTG AGCGTATAAT TTATTACAGA
GACGGAGTAA GCGAAGGACA GTTCGAGGCT GTGCTTCGTA GCGAATACGA GTCGCTTCAG
CGACAGCCGA GaagcgTCCA AGCAAAGGGT CCCAAGATCA CGTTCATAGT GGTGCAAAAG
AGGCACCATA CCTGTTTTTT CCCCGAAGCC GAC------A ACATCAGCCC GGGGACAATC
GTCGATAAAG TCGTGTGCCA TCCGACAAAC TTCGACTTCT ACCTTTGCAG CCACCAGGGA
ATCAAGGGGA CTAGCCGGCC CGTGCACTAC CACGTCCTCA AAGACGAGAA CGGGTTTACT
GCTAACGAGA TCCAGCAGTT CACGCACGAC CTCTGCTACC TCTACTCTCG CTGCACCCGA
GCTGTTTCGT ATGTTCCACC GTGCTATTAC GCTCACTTGG CAGCACAGCG AGCGCAGGCT
TGGGTG---
>Mesculenta_cassava586.valid.m1
GCCCCATTGG AAGATCCTTC TTTTCGGATA TTAGCGACTT GGCTTGGAGT CCTGGTGTGG
GTTTTACCAG AGTATAAGGC CTACACAAAT GGGCTATCCT TGAATATTGA TTGGCTTCAG
CTGCATTCAT TGAGCCCCAG TATTGAGTTT AGCTTCTGGG GATAGAAGAT TAAGAAGGCC

CTCAGAGGAG TAAAAGTTGA AGTAACTCAT AGAAGTATCG TGTCTCAGGA TTGACAACCT
ACAAGAGAAC TGTATTTCCT GTTGATGAAC TATGAAGTCA GTAGTTGAAT ATTTCCAAGA
GATGTATGGA TTCACCATTC AACATACATC TACCTTGCCT TCAAGTAGGA AACCAGAAAA
GGCGAACTAT TACCTATGGA GGCTGCAAAA TTGTGGAGGG CCAACGATAT ACGAAATTGA
ATGAGAGACA AATTACTGCC CTGTTGAATG CCACTTTTAT TAGCTATTCT CCTGAAATAA
TGGATCC--- CTATATGGAT CTTAAGCGGA TATGTGAAAC TGATCTTGGT TTAATATCAC
AATGCTGTCT TACAAACATG TTTTCAAAGC AACAGTATTT GGCTAATGTA TCACTGAAGA
TTAATGTTAA GTGGGTGGTA GAAACACTGT CCTGATGCCA TTAGCTGATA CCTGGTTAGT
GACCAACTAT TATATTTGGA GCAGATGTAA CTCACCCAAT GGGGAAGCCC CTCCATTGCA
GCTGTGGTAG CTTCTCAGGA CTGGCCTGAA GTGACAAAAT ATGCTGGATT GGTTTGTGCT
CAAGCTCACA GACAAGAACT CATACAAGAC TTGTACAAGT TAGTGGTGGC ATGATCAGAG
ATCTTCTAGT TTCCTTTCGG AAGGCAACAG GAAAAGCCAC TAAGGATTAT ATTTTACAGG
GATGGTGTTA GTGAAGGGCA ATTTTATCAA GTTTTGCTTT ATGAATTGGA TGCAATCCGG
AAGGCTGTGC TTCTCTGAAC CAAATATCAA CCACCTGTGA CTTTCATAGT TGTACAAAAA
CGACACCATA CTCGATTATT TGCACCACAG GGAAGTGGAA ACATATTACC CGGCACTGTG
GTTGATTCGA AAATCTGCCA TCCAACAGAG TTTGATTTTT ATCTCTGCAG TCATGCAGGT
ATTCAGGGGA CAAGTCGGCC TGCTCACTAC CATGTTCTGT GGGATGAGAA CAACTTCACT
GCTGACGGAA TCCAGTCATT GACGAACAAT TTATGTTACA CATATGCAAG ATGCACTCGC
TCTGTTTCTG TTGTTCCTCC TGCATATTAC GCACATTTAG CTGCTTTTCG TGCCCGATTT
TACACGGAG
>Csativus_Cucsa.254700
GTCCTATAGG GAGATCTTTC TTTTCTGATA TTAGCGGCTC GGGCTAGAAT CATGGTGTGG
ATTTTATCAG AGTATTAGAC CTACTCAAAT GGGCTGTCTC TGAACATAGA TTGGCTTCAG
CTGCGTTCAT TGAGCCCCTG TCTTGAGTTT AGCTTCTAGG GATCGAAGAT TAAAAAGGCT
CTGAGAGGGG TGAAAGTTGA AGTAACACAC CGAAGTATCG AGTTTCGGGT CTGACAGCCT
ACAAGAGAAT TGTATTTCCT GTTGATGAAC CATGAAGTCA GTTGTTGAAT ACTTCCAGGA
GATGTATGGC TTCACCATTC AGCATGCATC TCCCTTGCCT TCAAGTAGGA AACCAGAAAA
GGCAAATTAT TGCCAATGGA GGCTGCAAAA TTGTGGGGGG GCAAAGATAT ACAAAGTTGA
ATGAAAAGCA AATAACAGCA CTTCTAAATG TCATTATTAT TAGCTATTTT CCTGAAATAA
CGGATCG--- CTTTATGGAT CTTAAGCGAA TTTGTGAAAC TGATCTTGGT TTAATATCAC
AATGCTGTCT TACAAGCACG TGTTCAAAGC AACAATACCT TGCTAATGTG TCACTGAAGA
TCAATGTCAA GTGGGTGGAA GAAACACCGT TCTGATGCTA TCAGCTGATA CCTTGTGAGT
GACCAACAAT TATATTTGGA GCAGACGTGA CCCATCCAAT GGCGAAGCCC TTCAATAGCT
GCTGTAGTAG CCTCTCAGGA TTGGCCTGAA GTGACAAAAT ATGCAGGGCT AGTATGTGCT
CAAGCTCATA GACAAGAACT TATACAAGAC TTGTACAAGT CAGTGGTGGC ATGATCAGGG
ATCTTCTGAT TTCGTTTAGG AAAGCAACAG GAGAAGCCTC TCAGGATAAT ATTTTACAGG
GACGGCGTGA GCGAAGGACA ATTTTATCAA GTATTACTTT ATGAGTTGGA TGCAATCAGG
AAGGCTGTGC TTCTTTGAAC CGAATACCAA CCACCTGTAA CATTCATTGT CGTACAAAAG
CGACACCACA CCCGATTGTT TGCACTATAG AGAAGTGGGA ACATTTTACC TGGAACTGTA
GTCGACTCCA AAATATGCCA CCCAACAGAA TTTGATTTCT ATCTCTGTAG TCATGCTGGA
ATTCAGGGAA CGAGTCGGCC AGCTCACTAC CATGTTCTTT GGGATGAAAA CAATTTCACT
GCAGATGGAA TTCAGTCGTT AACAAACAAT CTTTGCTACA CGTATGCAAG ATGTACACGT
TCAGTTTCTG TCGTCCCTCC AGCATACTAT GCACATTTAG CTGCATTCCG AGCTCGATTC
TACATGGAA
>Alyrata_16064878_locus
GTCCGGTTGG AAGATCTTTC TTTTCTGATA TTAGCGACTC GGGTTAGAGT CATGGTGTGG
GTTTTACCAG AGTATTAGAC CAACTCAAAT GGGTTATCAC TAAATATCGA TTGGCTTCAG
CTGCGTTCAT CGAGCCCCGG TATAGAGTTT AGCTTCTTGG GATCGAAGAT TAAGAAGGGT
CTCAGAGGAG TGAAAGTAGA GGTTACTCAC AGAAATACCG TGTTGCGGGT TTAACAACCT
ACAAGAGAGC TATGTTTCCA GTTGATGAAC GATGAAGTCG GTTATTGAGT ACTTCCAAGA
GATGTATGGA TTCACGATCC AGCACACATT TGCCGTGTCT CCAAGTTGGA AACCAAAAAA
AGCAAGCTAT TGCCAATGGA GGCTGCAAAA TTGTTGAGGG ACAACGATAC ACGAAATTGA
ATGAGAAGCA GATTACTGCT CTCTTGAATG TCACTTCTGT TGGCAATTTT CCTGAAACAA
CGGTTCA--- CTTTATGGAT CTTAAGAGAA TCTGCGAAAC CGAGCTTGGT TTGATATCTC
AATGTTGTCT CACAAACATG TGTTCAAAGC AACAGTATCT GGCAAATGTA TCCCTTAAAA
TCAATGTCAA GTGGGAGGAA GGAACACAGT TCTGACGCCA TAAGCTGATT CCTGGTTAGC

GACCGACAAT TATATTTGGC GCAGACGTGA CTCACCCAAC GGGGAAGCCC TTCAATCGCT
GCTGTTGTTG CTTCTCAAGA CTGGCCTGAA GTCACAAAAT ATGCGGGTTT AGTTTGTGCT
CAAGCTCACA GGCAAGAACT TATACAAGAT TTGTATAAGT TAGTGGCGGT ATGATCAGGG
ACCTTCTGAT CTCATTTAGG AAAGCAACAG GAGAAACCGC TTCGAATTAT CTTTTACCGT
GATGGAGTAA GCGAAGGGCA ATTCTATCAA GTTTTACTCT ATGAGTTGGA TGCAATTCGA
AAGGCTGTGC GTCGCTGAAC CGAATATCAG CCACCGGTGA CATTCATTGT TGTACAGAAG
CGTCACCACA CTCGTTTGTT TGCATCACCG AGAAGCGGAA ATATCTTACC TGGTACTGTA
GTTGACACCA AAATATGTCA TCCAACTGAA TTCGACTTCT ACCTTTGTAG CCATGCGGGT
ATTCAGGGAA CAAGCAGGCC TGCACATTAC CATGTTCTTT GGGACGAGAA CAATTTCACA
GCTGATGGTA TTCAATCTTT GACTAATAAT CTCTGTTATA CCTATGCACG ATGCACTCGA
TCAGTCTCGA TAGTTCCTCC AGCGTATTAT GCGCATCTTG CAGCATTTCG AGCACGTTTC
TACATGGAA
>Csativus_Cucsa.200260
CAGTTGTTGG AAGGTCATTT TTCTCTGAAC TGGGGAACTT GGTGTTGAGT ATTGGAGGGG
ATATTACCAA AGTCTACGAC CTGTACAAAT GGGTTATCTT TAAATATAGA TTGTCAGCTA
GATCATTTTA TGAACC---- ----GAATAT AACACTTCAA GATTGAAGAT CAAAAAGGTA
CTTAGAGGAG TGAAGGTTGG ATTAACGTGT AGACCTACAA GATTACTGGG ATATCAGCCT
GTTAATAGAT TATGTTTACT CTTGACGAAC CCGAATCTCT GTTGCACAGT ACTTTCATGA
AAAATATGGC GTGGCACTCA AATATCCTTC TACCAGCTAT ACAAGCTGGT AATGATGCAA
GCCGGTTTAT TGCCTATGGA GGTTGTAAGA TTGTTGCTGG GCAGAGATAT ACCAAATTGA
ATGAACGCCA AGTAACTGAG ATGCTAAGTG TCATTGCTCA TAATTATTTT CCTGAATTTC
TGGTTCC--- ---TATGGAG ATCAAAAGGA TATGTGAGAC TGAGCTTGGA ATTGTTTCAC
AATGTTGCCA ACCAGGCAAG CACAAAAAAC AACAGTACTT TGAAAATGTG GCCCTTAAAA
TTAACGTTAA GTTGGGGGAA GAAACAATGC CCTGATGCTA TTCAGCGATT CCTTGTTTCA
GACCTACAAT AATCTTTGGA GCAGATGTAA CACACCCCCT GGAGAAGTCC TTCAATAGCA
GCTGTTGTTG CCTCAATGGA CTGGCCCGAG GTAACGAAGT ATAGAGGAAT TGTTTCAGCT
CAGGGCCACC GGGATGAAAT TATACAAGAT TTGTACAGGT GTGTGCAGGA ATGATCAGGG
AGCTGTTCAT TGCTTTTAGA AGGTCAACAA ATGAAACCGC ACAGAATAAT ATTTTACAGA
GATGGTGTAA GTGAAGGACA GTTCTCACAA GTTTTATTTT ATGAGGTGGA TGCAATTAGG
AAGGCTGTGC CTCTCTGAAG AGGGTACCAG CCTCCGATTA CCTTTGTTGT GGTGCAAAAG
AGACATCATA CCCGTCTTTT CCCGC----- ---AGTGGAA ACATCCTTCC AGGTACTGTT
GTTGACACCA ATATTTGCCA TCCAACTGAA TTTGATTTCT ATTTAAACAG CCATGCCGGC
ATTCAGGGGA CGAGTAGGCC AACACATTAT CACGTTTTGT ATGATGAAAA CAAATTCACT
GCCGATGCAA TGCAGATGCT TACTAATAAT CTTTGCTACA CGTATGCAAG GTGCACTCGG
TCGGTTTCCA TTGTTCCACC AGCATACTAT GCACATCTTG CAGCCTTTCG TGCCCGTTAT
TACATAGAG
>Sbicolor_Sb10g031030
CACCATTTGG GCGATCATTT TTCTCTGACC TGGGTCCCTT GGAATAGAAT GCTGGCGTGG
GTTTTACCAG AGCATTCGGC CTACTCAAAT GGGCTGTCAT TGAATATTGG TAAgctctgt
cc-------- ------CCTG TATAGATTTT AGCTTTTAAA GAACGAAGAT AAAGAAGGCC
TTAAGAGGAG TGAAGGTGGA AGTTACCCAC CGAAGTATCG AATAGCTGGT TTAACTGGCA
ACTCGGGAGT TACTTTTCCT GTTGATCAAC ATTGAAATCT GTTGTACAAT ATTTTCAAGA
GACCTATGGC TTTGCCATCC AGCACACACC TGCCCTGTCT GCAAGTCGGC AATCAGCACA
CCCAAATTAC TTCCAATGGA GGTTGCAAAA TAGTAGAGGG ACAGAGGTAC TCTAAGTTAA
ACCAGGGTCA GATAAGAGCT CTTTTAGATG CCATTGCTTA TTGGGATACT CCTGAAACAA
CGGTTCT--- CTTTATGGAT TTGAAGCGTA TCTGTGAAAT TGACCTTGGA TTAGTTTCAC
AGTGCTGCTG TGCAAGCAAG TTTTTAAAAC AACAGATACT GGCAAATCTC GCTCTGAAGA
TAAATGTCAA GTTGGAGGAA GGAACACAGT GCTGATGCAG TATCAAGATT CCTGGTGACT
GACCTACCAT CATATTTGGT GCTGATGTGA CCCATCCCCT GGTGAAGCCC TTCCATTGCT
GCTGTTGTGG CTTCCCAAGA TTGGCCTGAG GTGACAAAGT ATGCAGGTTT AGTTTCTGCT
CAATCTCATA GGCAAGAGTT AATAGAGGAT CTGTATAAAT TTGTGGTGGC ATGATCAGGG
AGCTCCTTAT ATCCTTCAAA AGATCAACTG GAAAAGCCTC AGAGGATACT ATTCTACAGG
GATGGTGTGA GTGAAGGGCA GTTCTACCAA GTTCTACTGC ATGAATTGGA TGCAATCCGA
AAGGCTGTGC ATCGCTGAAG CAAATACCAA CCACAGGTGA CTTTCATCGT GGTCCAGAAA
CGGCACCACA CGAGGTTGTT CGCACCACAA TGAAGTGGGA ACATACTTCC TGGTACTGTT
GTGGACTCGA AGATCTGCCA CCCTACAGAG TTTGACTTCT TCCTGTGCAG CCATGCTGGC

ATCAAGGGCA CGAGCCGTCC TGCTCACTAC CATGTCTTGT GGGATGAAAA CAACTTCACA
GCTGATGCAT TGCAGACCCT TACCAATAAC CTTTGCTACA CTTACGCGAG GTGCACACGT
TCTGTATCCA TTGTCCCACC AGCATACTAC GCTCATCTAG CTGCATTCCG CGCCCGGTTC
TACATGGAG
>Rcommunis_29677.t000007
GCCCTGTCGG TCGATCATTT TATTCTGATC TAGGCCCCTT GGTTTGGAGA GCTGGCGTGG
TTTTTACCAA AGCATTCGAC CTACACAGAT GGGTTGTCTT TAAATATTGA TTGTCTTCCA
CTGCTTTCAT TGAACCCCAG TATTGACTTT AACTGTTGAA GACCGAAGAT TAAAAAGGCT
CTTAGAGGAG TCAAGGTGGA AGTTACACAC CGAAGTACCG CATATCTGGT TTAACAGGCA
ACACGAGAGT TACTTTTCCA GTTGACGAAC AATGAAATCT GTCGTGGAGT ACTTCTACGA
GACATATGGT TTTGTAATTC AACACACAAT GGCCCTGTCT ACAAGTGGGG AATCAGCAAG
GCCGAATTAT TGCCTATGGA GGTTGCAAGG TTGTTGAGGG CCAGAGGTAC TCCAAGCTGA
ACGAGAGGCA GATAACTGCC TTGCTGAATG TCACTGCTTA TCGTAATTCT CCTGAAATAA
TGGCTCT--- CTTTATGGAA CTGAAGCGCA TTTGTGAGAC GGATCTTGGT CTTGTTTCTC
AGTGCTGTTT AACAAGCATG TGTTCAGAAC AACAATATTT GGCAAATGTG GCATTAAAAA
TTAATGTTAA GTTGGGGGAA GAAACACTGT GCTGATGCAT TATCAAGATT CCTGGTTAGT
GACCCACTAT TATTTTTGGT GCTGATGTCA CCCATCCCCG GGGGAAGCCC ATCTATTGCA
GCTGTTGTGG CTTCTCAAGA TTGGCCAGAA GTAACGAAAT ATGCTGGCTT GGTTTGTGCA
CAAGCCCATC GGCAAGAGCT TATTCAAGAT CTCTTTAAGT TACTGGTGGC ATGATTAAGG
AACTCCTTAT ATCTTTCCGC AGAGCAACTG GAGAAACCTC AGCGTATTAT ATTTTACAGG
GATGGTGTTA GTGAAGGACA ATTCTACCAA GTGCTGCTGT ATGAACTTGA TGCTATCCGG
AAGGCTGTGC TTCTTTGAAC CAAATATCAG CCTCCAGTGA CATTTGTTGT GGTTCAGAAG
CGTCACCACA CTAGGTTGTT TGCACCATAA CGAAGCGGAA ATATACTGCC TGGTACTGTC
GTGGACTCTA AGATCTGCCA TCCAACTGAA TTTGACTTCT ATCTGTGTAG CCATGCTGGG
ATTCAGGGTA CCAGCCGTCC AGCTCATTAC CATGTATTAT GGGATGAGAA CAAGTTTACT
GCCGACGGGT TACAGTCCCT TACAAACAAT CTTTGCTACA CATATGCGAG ATGCACGCGC
TCTGTTTCCA TCGTACCTCC TGCATACTAT GCTCATCTTG CAGCATTTCG AGCTCGGTTC
TACATGGAA
>Ptrichocarpa_POPTR_0006s12010
CCATCGTTGG GAGGTCTTTT TTCACAGGTC TGGGGAGATT GGTATAGAAT GCTGGAAGGG
ATTCTACCAG TCTCTACGCC CAACGCAGAT GGGATGTCTC TTAACATAGA TTATCAGTCG
CTGCCTTCTA CGAGCCTTAA A--------- ATCCAattag GATCGAAGTT GAAAAAAGCT
TTGAGAGGAG TCAGAGTAAA AGTTACCCAT GGCGTTACAA AATCACTGGA ATATCCAGCA
ACAAACCAAC TAGGTTTGCT GCTGAAGAAA ACAAAAATCA GTTGTCCAAT ACTTCCTGGA
AAAATACAAT ATAAGGCTTC GTTTTGCCCT GGCCTGCTCT TCAATCAGGA AATGATTCAG
ACCAATATTC TGCCTATGGA GTGTGCAAGA TTATCGAAGG ACAAAGGTAC TCAAAGTTGA
ATGAGAAGCA GGTGACAGCC TTATTGAGTG CAGATCTTAA TTATCATTCT CCTGAGTCAG
TGGAAGT--- ---TATGGCA ATAAAAAGAG TATGCGAAAC TGAACTTGGG ATAGTTTCTC
AATGCTGTCA ACCAAGCAAG CAAGAAAAGT CCCAATACTT GGAAAATGTT GCGCTGAAAA
TTAATGTGAA GCTGGAGGGC GAAACACAGT ATTGATGCCC TGAATAGATA CCTTCTAAGT
GACCAACTAT AATCTTTGGT GCTGATGTAA CCCATCCCCA GGGGAAGCCC TTCAATAGCT
GCGATTGTGG CATCAATGGA CTGGCCTGAA GTAACCACCT ACAGGGGCCT GGTATCTGCT
CAGAAACATC GTCAAGAGAT TATTCAAGAT TGT------- ----GCTGGA ATGATCAGGG
AACTTATGAT TGCTTTCAGA AGAACAACTA AAGAAACCTA GCAGAATAAT TTTCTATAGG
GATGGTGTTA GTGAGGGCCA GTTCAGCCAA GTTCTCCTGT ATGAGATGGA TGCTATCCGA
AAGGCTGTGC ATCTCTGAAC CAAATATTTG CCACCAGTTA CTTTTATTGT AGTGCAGAAG
AGACACCATA CTCGGCTCTT TGCCAAATCC TAAAGTGGAA ACATCCTTCC TGGAACGGTT
GTTGATACAA AGATATGCCA TCCTTCAGAG CACGACTTTT ATCTCTGCAG TCATGCAGGA
ATTCAGGGGA CTAGCAGGCC GGTGCACTAT CATGTGTTGT GTGACATGAA CAAATTCACT
GCTGATTGCC TGCAGATGTT GACGAACAAC CTGTGCTACA CGTATGCAAG GTGCACCCGC
TCTGTTTCTG TGGTTCCTCC TGCATACTAT GCACACTTGG CAGCATTCAG GGCAAGATAC
TACATAGAG
>Alyrata_16047041_locus
TCCTAGTTCG CCAATCTTTC TTCCATGATG CAAAAATATC GGTGTTGATT GTTGCAAAGG
ATTCCATTCA AGCTTCAGAA CTACTCAGGG AGGTTGTCCC TCAATATTGA TTTTCGACTA
CGATGATAGT AAAACCCCTG TGTTGGTTTT GAAACCAAGG AACTGAAGGC TAAAAGTACT

CTCAAGAATC TTAGAGTTAA AGTCATCCCC TCGAATACAA GATAACCGGA CTAAGTACAC
TGCAAAGATC AATGTTTACT TGGAAGAAGT TGAGATTACA GTGTTTGATT ACTTCACCAA
AATCCGTGAC ATCAAACTGC ATTATTCGCT TACCATGTAT CAATGTTGGT AAGCCAAACG
TCCTACCTAC TTCCCATTGA GCTTGTGAGC TTGTATCTCT ACAACGCTAT ACTAAACTTA
CAAGTTTTCA GAGGAGTAAC CTTGTCAAAG GCATTCCTTC TATGCATACT ---GAAAGAA
AAACTCTGAT GTTTATGGCT TGGAAAAAGA AAAATCTTGT TCAAGTTGGA ATTGTGAATC
AGTGTATTGC TCCCAAAACG TTAATGA--- --CATTATCT CACAAATGTT CTCCTCAAGA
TAAATGCCAA GTTGGTGGAT TGAATTCAGT GTTGATATGG AGCGGTCATG CCTGGTAATG
AACCTACCAT CATTATTGGG ATGGATGTAT CTCATGGCCT GGACAGTACC ATCCATTGCC
GCGGTTGTGA GCTCCAGAGA ATGGCCACTG ATCTCAAAAT ACAGGGCTTG TGTGCGTACA
CAGTCGCGTA AAGTTGAAAT GATCGATAAC CTCTTTAAGA TGATGAAGGT ATCATGAGGG
AGCTCTTGCT TGACTTTTAC TCAAGTTCTG CTCAAACCCA ATCACATTAT TATTTTCAGG
GATGGTGTGA GTGAATCTCA GTTTAACCAA GTTCTTAATA TTGAACTGGA TCAGATGAAG
---------- ---------- ---------- ---------- ---------- ----CAAAAA
AACCACCACA CCAAGTTCTT CCAGGAGCCC TGA------A ATGTTCCTCC AGGAACAATA
ATTGATAGCA ACATCTGTCA CCAACACAAC AACGATTTCT ATCTTTGTGC TCATGCTGGA
ATGATTGGAA CTACAAGGCC AACACATTAC CATGTGCTGT ATGACGAGAT TGGATTTGAC
ACAGATCAAC TCCAAGAACT TGTGCATTCC CTATCCTATG TCTACCAGCG GAGCACAACT
GCAATCTCTC TTGTTGCGCC GATATGTTAT GCTCATTTGG CGGCTGCACA GATGGGAACT
GCGATGAAG
>GRMZM2G031147_T01 cds:_protein_coding
TTATCCTTGG TAGAGGATTT TACTCAAGCA GCAGGACATT GGTGCTGTAG CTATGAAAGG
AACCCAGCAG TCCCTTAAAT CCACTCAGCA AGGTTGATCC TGTGTGTTGA CATTCTGTCA
TGCCGTTTTA CAAAGC---G TATGGATCTT AATTAGTGAG -----AATCT GGTTGATGAG
CTTAAAGGCC GACGTGTAAC TGTGATTCAT CGAAGTACAC AGTGCAAGGT TTGACTACCT
GCCAGCCAGA TACCTTTGTG GATGCTGACA AACACGGAGG CTCGTGGATT ATTATGCTCA
GAAACATGGC AAGGTGATTG AGTATCATGC TGCCATGCTT GGATTTGAGC AAGAGCAAAA
ACCGAATCAT TTCCAATTGA GCTTGCACTC TTCTTGAAGG ACAGAGGTTT CCAAAAAACT
TGAATCAGAA TTCTGAGAGG ATACTAAAGC TCTCTCCTTT TCTGCCCGAT -----TCCGA
GCAGCATTCG GGGTACAACA CTGAAGCTGA TTTGTGACAC ACAGCTGGGG ATCCTGACCC
AGTGTTTACT GAGGACCGCG CAAACAACAG GACAGTACAT GACGAATCTT GCTCTAAAGA
TCAACGGCAA GTTGGGGGCA GCAACGTTCA GCTGACTCGC TCCCACG--- --TCGGTGGT
GGCCTTTCAT GTTCATCGGT GCTGACGTTA ACCACCCCCC GGTAAAGCCC ATCGATCGCA
GCCGTGGTCG CCTCTGTCAA C---TCTGGT GTCAACAAGT ATGTGACCAG AATCCGTGCC
CAGCCGCACC GCTGCGAGGT GATCCAGCAG CTT------- ----GGTGAG ATCTGCCGGG
AGCTCATTGG AGTCTTTGAG AAGCAGAACC GTGAAGCCGC AGAAGATCAT CTACTTCCGT
GATGGCGTGA GCGACGGGCA GTTCGATATG GTCCTGAACG AGGAGCTGGC TGACCTGGAG
AAGGCATCAA G---GT---A ATGGTATGCG CCAACCATCA CCGTGGTCGT GGCCAAGAAG
CGGCACCACA CTCGGCTGTT CCCACGAACA GCAACCGGGA ACGTGCCGCC TGGCACGGTG
GTGGACACGG GCGTGGTGGA CCCGTCCGCG TACGACTTCT ACCTGTGCAG CCACACTGGG
ATTCTGGGGA CGAGCAGGCC GACGCACTAC TACACCCTGG TGGACGAGCA CGGCTTCGGC
TCCGACGACC TGCAGAAGCT GATCTACAAC CTGTGCTTCG TGTTCGCGCG GTGCACCAAG
CCGGTGTCGC TGGCGACGCC CGTCTACTAT GCCGACCTCG CGGCCTACCG TGGCAGGCTC
TACTACGAG
>Gmax_Glyma15g13260
TTTCTGTAGG ACGACACTTC TATCCAAATC CT--GATCTT GGGATAATTG CTATTGGAGG
GTTTCAGCAT AGTTTGAAGC CTACATCTCA GGGCTGTCCT TATGTGTAGA CACTCAGTAT
TGGCTTTTCG AAAGCATCAG TTTGGATTTC AGCGTATTGA AAATTTTCAT TGAGGAGGCA
CTTATTGGAT TGAAAGTTAA TGTGACTCAT CGAAATACAT TATTTCAAGA TTAACTGATT
ACTAGGTATG TACTTTTCCC ATTGACAAAA TGATGTTAGT CTTATTACCT TTTTTAAAGA
AAAATATGGC AAGGATATCG TGTACAAATA TTCCTTGTCT AGATTTAGGG AAAGACAGAA
GAAGAACTAT TACCCATGGA ATTTGTGTTT TGGTTGAGGG CCAAAGATAT CCCAAACTGG
ATGGTATTTC TGCGAATACA TTGAAAGCGC TCATTTCTTC TCTGTGTAAT -----GCTAA
AAAAAGTCCC GGTTATAAAC CTAAAGTGGA TTTCTGAGAC CAAACTTGGT ATACTGACAC
AATGCTGCTT GTCAATAGTG CTAATGAGAG GAAAATTTTA TACTAATCTG GCTCTCAAGA
TCAATGCCAA GTTGGCGGCA GTAACGTGGA GCTAATGGAC TACCTTA--- --TTGAGGAT

GAGATGTTAT GTTTTTAGGG GCTGATGTCA ACCATCCTAT CAAGAAGTCC ATCAATTGCA
GCTGTGGTTG CTACTGTTAA TTGGCCTGCT GCAAATCGTT ATGCAGCACG TGTTTTCCCA
CAATACAATC GAAGTGAGAA AATATTGAAC TTT------- ----GGGGAT GTTTGTCTTG
AGCTTGTTGC ATGCTATAGG AGGATGAATG GTTAGGCCAG AAAGAATTGT TATTTTTCGT
GATGGGGTGA GCGAATACCA GTTTGACATG GTTCTTAATG AAGAGCTACT AGATTTGAAG
GGAGTTTTCA AAGAGT---- --AATACTTT CCAACAATCA CTCTTATTGT AACACAAAAA
CGACATCATA CTCGATTTTT TCCGCTGGAG AGAAGTGGCA ATGTTTTGCC GGGAACAGTT
GTCGACACAA AAGTTATACA CCCTTATGAG TTTGACTTCT ACCTTTGTAG TTACTATGGA
AACCTAGGTA CAAGCAAGCC TACTCATTAC CATGTCTTAT GGGACGAGCA CAAGTTTACA
TCTGATTTAT TGCAGAAGCT AATATACGAG ATGTGTTTCA CCTTTGCAAA GTGCACTAAA
CCTGTATCAT TAGTCCCTCC TGTGTATTAT GCTGACCTTG CTGCTTATAG AGGACGATTA
TACCATGAA
>Rcommunis_29589.t000074
CCCCTGTTGG GAGGTCATTT TTTTCTGATT TGGGGAGCTT GGTATAGAGT ATTGGAGAGG
ATACTATCAG AGTCTTCGCC CAACCCAGAT GGGCTTTCTC TTAACATAGA TTATCAGCTC
GATCATTCTA TGAGCCATTG TACCGACTTT AATACTTGAA GATCGAAGGT GAAAAAGGCT
CTGAAATCAG TGAAAGTGCA AATACTTCAC AGAGCTATAA AGTTACTGGC ATATCAACCC
CTGAATCAGA TTTCTTCAAA TTGGATGAAC AGATATCTCA GTTGTACAGT ATTTCCGTGA
AAAATACAAT ATTGGGCTAA AATATACCCT TGCCTGCCCT TCAAGCTGGG AGTGATGCAA
ACCAATTTAC TGCCTATGGA GCTTGTAAGA TTGTTGATGG TCAGAGGTAT TCAAAGCTTA
ATGAAAGACA AGTAACTGCA CTGCTAAGTG TCATTGCTGA TAATTATTCT CCTGAATCAG
TGGATCT--- ---TATGGTA ATTAAGAGAG TCTGTGAAAC AGAATTGGGA ATTGTTTCTC
AGTGTTGCCA ACCCGACAAG CAGCAAAAGT AACAGTATTT TGAGAATGTT GCTCTCAAAA
TCAATGTTAA GTTGGGGGAA GAAACACTGT GTTGATGCCG TGCAAAGATC CCTTGTGACT
GACCTACAAT TATTTTTGGT GCTGATGTAA CTCATCCCCA GGGGAAGTCC GTCGATAGCA
GCAGTAGTGG CTTCAATGGA CTGGCCTGAG GTAACCAAGT ATAGAGGCAT TGTCTCTGCA
CAGGCGCATC GTGAAGAAAT CATCCAGGAT TTGTATAAAA GCATTCAGGA ATGATCAGAG
AATTGTTTGT TGCATTTAGA AGAGAAACTG GTGAAACCTA AAAGGATTAT ATTCTACAGA
GATGGTGTTA GTGAGGGGCA GTTTAGTCAA GTTTTGCTGT ATGAGATGGA TGCCATAAGA
AAGGCTGTGC CTCACTGAGG AGGGTATCTT CCACCGGTTA CCTTCGTTGT GGTGCAAAAA
AGGCACCATA CACGACTTTT TCCTTGATCG TGGAGTGGCA ATATTCTGCC AGGCACTGTT
ATTGACACTA AGATATGCCA CCAGAGGGAG TTCGACTTCT ACCTGAACAG CCATGCTGGA
ATTCAGGGAA CCAGCAGACC AACTCATTAC CATGTGCTGT ATGATGAAAA TCATTTCACT
GCTGATAATC TGCAAGTGCT GACTAATAAT CTGTGTTACA CGTTTGCCAG GTGTACACGC
TCTGTGTCCA TAGTCCCTCC TGCCTATTAT GCCCATTTAG CAGCTTTCCG TGCTCGATAT
TACATTGAG
>Gmax_Glyma20g28970
GCCCCATTGG GAGGTCCTTC TTTTCTGATA TTAGCGGCTT GGATTAGAGT CATGGTGTGG
ATTTTACCAG AGTATAAGGC CTACACAAAT GGGCTTTCCC TTAATATTGA TTGGCATCTG
CTGCGTTTAT TGAGCCCCAG TGTGGAATTT AGCTATTAGG GATCGAAGAT TAAGAAAGCC
CTTAGAGGAG TTAAAGTTGA AGTAACACAC AGAAGTATCG TGTTTCTGGG TTGACAACCT
ACCAGAGAAC TGTGTTTCCT GTTGATGAAC TATGAAATCA GTAGTTGAAT ACTTCCAAGA
GATGTATGGT TTCACTATTC AATATACACC TTCCTTGCCT TCAAGTAGGA AACCAAAAAA
GGCGAACTAT TACCTATGGA GGCTGCAAAA TTGTTGAGGG GCAACGTTAT ACAAAATTGA
ATGAGAAGCA AATTACGGCT CTATTGAATG CCACTTTTGT TAGCAATATT CCAGAAATAA
TGGGTCT--- CTCTATGGAT CTCAAGCGAA TTTGTGAAAC TGACCTTGGC TTAATTTCAC
AATGCTGTCT GACAAGCATG TCTTCAAACT AACAGTACCT GGCTAATGTG TCTCTGAAGA
TCAATGTGAA GTGGGAGGTA GAAACACTGT ACTGATGCTG TAAGCTGATA CCTGGTTAGT
GACCAACCAT AATTTTTGGA GCAGATGTAA CCCACCCAAT GGAGAAGCCC TTCAATAGCA
GCTGTAGTAG CATCCCAGGA CTGGCCCGAA GTGACAAAAT ATGCCGGTTT GGTATGCGCC
CAAGCTCATA GGCAGGAACT TATACAAGAT TTGTACAAGT TAGTGGTGGC ATGATCCGAG
ATTTACTGGT TTCCTTTAGA AAGGCAACAG GAAAAGCCAC TACGAATTAT ATTTTACAGG
GACGGTGTAA GTGAAGGGCA ATTTTACCAA GTTTTACTTT ATGAGTTGGA TGCGATTCGG
AAGGCTGTGC TTCCTTGAAC CAAATACCAG CCTCCAGTAA CATTCATAGT TGTGCAAAAA
AGACATCATA CCCGGTTATT TGCACTACAG GGAAGTGGGA ATATATTGCC TGGGACTGTC
GTTGATTCCA AAATCTGCCA TCCAACAGAA TTTGATTTTT ATCTCTGCAG CCATGCTGGC

ATCCAGGGTA CTAGTCGGCC AGCTCATTAT CATGTCCTGT GGGATGAAAA CAACTTCACA
GCAGATGGAA TTCAGTCTCT GACAAACAAT CTTTGTTATA CATATGCCAG GTGTACGCGC
TCAGTATCAG TTGTTCCTCC AGCATATTAT GCACATTTAG CAGCGTTCCG AGCACGTTTC
TATATGGAG
>Rcommunis_30093.t000002
TTTATGCTGG TCGAATTTTC CATCCGCCTT ATCCGATCTC GGAATTACAG CTTCTAGAGG
AATCAAACAT ACCCTGAAGC CAACATCCCA GGGCTAGCCT TGTGTCTGGA TATTCAGTTC
TGCCTTTGCT CAAGCACCGG TATAGATTTT AGCATATTCG GCTTTGAAGT GGAGAGAGTT
TTGAAAGAAT TGAAAGTTAC TGTTAATCAC AGAAATTCAA AATAGCAGGT TTAACATGAC
ACACAGGATA TTCATTCGAG GTGGATCGAG GAAAGTTTGG CTTGTTGACT ATTTTAAAGA
AAAATACAAC AAGAATATTA CACACAGATA TACCATGCTT AGATTTAGGT AAAAAGAAAG
GACAAATTAT TTCCTATGGA GTTTGCAGCA TAGCTAAGGG GCAGAGGTTT GCAATGCTGG
ATCGAAATCA ATCCGAAAAG TTGAGGAGGC T--ATTCTTG TTTGTGTCCT CCAAA-----
-GAGGATCCT GGTTACAGAT CTCAAGTGGA TATGTGAGAC CAAAGTTGGC ATTGTAACTC
AATGTTGTTT ATCGAAAATG CATTTAGAAA GCCAATTTCT TGCAAATCTA GCTTTAAAGA
TCAATGCGAA GTTGGAGGCA GTAATGTGGA GCTAAGCAGC CTCAATG--- --TGCAAAGT
AACATGTTAT GTTTATTGGT GCTGATGTTA ACCATCCTCT TACAAAGTCC TTCAATAGCA
GCTGTTGTTG CCACCATGAA TTGGCCTGCA GCAAATCAAT ATGGCGCACT GATTTGTCCT
CAAGATCATC GTGCAGAGAA AATCCTGAAA TTC------- ----GGAGAC ATGTGTTTAG
AGCTTGTGAA TGCTTATGCC CGGCTAAATC ATAAGACCAG AGAATATTGT TGTATTTCGT
GATGGAGTAA GTGAGAGCCA GTTTGATATG GTTCTCAATG AAGAGTTGAA GGATATCAAG
GCGGCTTTGA ATCACTGAA- ----TACTTC CCAACCATCA CTCTTATTGT TGCCCAGAAG
CGACACACAA CTCGTTTGTT TCTATAGTGA TGAGATGAAA ATGTACCTCC GGGCACTGTG
GTGGATACAG TAATTACAAG TCCTTCTGGG TCTGATATCT ATCTTTGCAG CCATTTTGGT
CAAATTGGGA CGAGCAAGCC AGCACACTAC CAGGTGCTGC AGAACGAGAT CGAGTTTACT
CCTAATGAGT TGCAGGAGTT CATTTACAGC ATTTGCTTCA CCTCTGCTCA GTGCACCAAG
CCTGTCTCAC TTGTCCCTCC AGTGATCTAT GCTGATCGTG CAGCTTTTAG AGGTCGGCTT
TACTACAAT
>Mguttatus_mgf011041m
TCTCTATCGA TCGTCACTTT TACTCCAGTT TTAAGATTTT GGTGTTGCAG CGTATCGAGG
CTTCCAATCA ACTTTAAGAC CGACGTCGCA GGGCTGGCCT TGTGCCTCGA CGCTCGGTTT
TGGCGTTTCG TAAGCCGCAG TATGGATTTC AGAATATTCC AGGCGAGAGT TGCACATGCG
TTGAAAGGAT TGACGGTTAG AGTGACTCAC CGCGGTTCAC CATAGCTGGT TTGACAAAAC
ACCCGTGATC TTGGTTCGAT TTTGTCGAGT TAACGTCAGC CTTGTGCAGT ACTTCAGGCA
TAAGTATGGC AAGGACATTG TGTACCAATA TTCCTTGCTT GATTCTTGGC AGAAATAACG
GACGAATCAC TACCTATGGA ATTTGTATCT TGTCTGTCGG ACAACGCTAC AGAAAACTTT
TGGATGAAGT TTCGCAGGAT AAATACGATG CCTATAATAA TATGCGTAAT GCGGA-----
-AAACATCAC GGCTACAAAC CTTAAGTTCG TGTCGGAAAC ACGAATTGGT GTAGTGACTC
AGTGTTGCCT GTCGGTCACG CATTCAGGAC GAAAGTTTCT CGGAAATCTG TGTCTCAAGA
TTAATGCAAA GTCGGAGGAA GTAATGTTGA GTTCAGAGAC TTGCTGA--- --TCGAGGAG
GACATGTTAT GTTCATTGGA GCCGATGTGA ACCATCCTCG AAGAAACTCC ATCCATAGCA
GCAGTTGTCT CTACAGTGAA CTGGCCTGCA GTGAACCGCT ATGCCGCAAG AGTTTGCCCT
CAAGACCACA GAACCGAGAA GATTCTCGAA TTC------- ----GGTTCC ATGTGCCGCG
ATCTCGTCAA CACTTATTTC CAGGTCAACA ATCAAACCGA AGAAGATTGT TGTTTTTCGA
GACGGTGTGA GCGATGGGCA ATTCGATATG GTACTGAACG AAGAATTATC CGACTTAAAA
AGGTCTGC-- ------GATG AGAATACAAG CCAACAATCA CTCTTGTTCT AGCTCAGAAG
AGGCACCAGA CTCGTCTCTT TCTATGTTCT GGAACTGGGA ATGTGCCACC TGGCACCGTT
GTAGACACGA AGATTGTCCA TCCGTTTGAG TTCGATTTTT ACCTCTGCAG TCACTATGGA
AGGATTGGGA CTAGCAAGGC GGTGAGGTAC TGTGTACTGT GGGACGAAAA TTCGTTCACG
TCCGATGAAT TACAGAAGCT TATATATAAT TTATGCTTCA CGTTTGCACG TAGTACGAGG
CCTGTTTCGC TGGTGCCACC TGTATACTAC GCTGACCTGG TCGCTTACAG GGGTCGTATT
TTTCAGGAG
>Sbicolor_Sb06g028510
TTATCCTTGG TAGAGGATTT TACTCAAGCA GC--GACATT GGTGCTGTAG CTATGAAAGG
AACCCAGCAG ACCCTTAAAT ACACTCAGCA AGGCTGATCC TATGTGTTGA CATTCAGTTA
TGCCATTTTA CAAAGCCCGG TATGGATATT AATTAGTACC -----AATCT GATTGAGGAG

CTCAAAGGCC GACGTGTGAC TGTGGTTCAT CGAAGTACAC AGTGCAAGGC TTGACTACCT
GCCATCCAGA TACCTTTGTG GATGCTGACA AACGAAGAGG CTTGTGGATT ATTATGCTCA
GAAACATGAC AAGGTGATTG AGTACCATGC TTCCATGCTT GGATTTGAGC AAGAGCAAAA
ACCAAATCAT TACCAATTGA GCTTGCACTC TTCTTGAAGG ACAGAGGTTT CCAAAAAACT
TGGATAAGAA TTCTGACAGG ATACTGAACT AATCTCCTTT TCTGCCCGAT TCCGA-----
-CAGCACCCA GGGTACAACA CTGAAGCTGA TTTGTGACAC ACAGCTCGGG ATCCTGACCC
AGTGTTTCCT GAGGACCGCG CAAACAAAAT GGCAGTACAT GACCAACCTT GCTCTCAAGA
TTAATGGCAA GTTGGGGGCA GCAACGTTCA GCTGACTCGC TTCCACG--- --TCGGTGGT
GGCCTTTCAT GTTCATCGGT GCTGACGTTA ACCACCCCCC GGGAAAGCCC ATCAATCGCA
GGCGTGGTTG CATCTATCAA C---AGCGGT GCCAACAAGT ATGTGTCAAG AATCCGTGCA
CAGCCACACC GCTGCGAGGT GATCCAGCAG CTG------- ----GGTGAG ATCTGCCTGG
AGCTCATTGG AGTCTTTGTG AAGATAAATC GTGAAGCCAC AGAAGATCAT CTACTTCCGT
GACGGCGTGA GTGACGGGCA GTTTGACATG GTCCTGAACG AGGAGCTGGC TGACCTGGAG
AAGGCATCAA GGTG-----G ACGGTATGCA CCTACCATCA CTGTGATCGT GGCCAAGAAG
CGGCACCACA CGCGGCTGTT CCCACCAGGG CCAACTGGGA ACGTGCCGCC TGGCACGGTG
GTGGACACTG GTGTGGTTGA CCCGTCTGCA TACGACTTCT ACCTGTGCAG CCACACCGGG
CTTCTAGGGA CGAGCAGGCC GACACACTAC TACAGCCTGG TGGACGAGCA CGGCTTCGGG
TCTGACGACC TGCAGAAGCT GATCTACAAC CTGTGCTTCG TGTTCGCGCG GTGCACCAAG
CCGGTGTCAC TGGCGACGCC CGTCTACTAT GCTGACCTCG TGGCGTACCG TGGAAGGGTC
TACTACGAG
>Vvinifera_GSVIVT00031927001
TCTCTGTTGG ACGGAGCTTT TACCCTCTGT TCTCGATCTT GGCATTGTAG CTTCTAGAGG
ATTTTTACAT AGCCTCAAGC CCACGGCCCA GGGTTAACCT TATGTTTGGA CACTCAGTCT
TAGCATTTCG AAAGCCCCGG TATAGATTTC AGCATGTTAA AGGAAGAAGT TGAGGTTGCT
CTAAAAGGAT TAAAAGTTAG AGTCATTCAT CGAAATACAC TATCTCAGGA TTAAGAGGAC
ACAAGGTATC TTCATTTATC GCTGAAGAAA GAAAGTTGGG ATTATTGATT ATTTCAGGGA
AAAATATGGG AAGGATATTA AGTACAAATA TTCCGTGCTT AGATTTGGGG AAAAACAAAG
GAAGAACTAT TACCCATGGA GTTTGCATCT TGACTGAGGG ACAGAGGTTT CTCAAAAACT
TGGATAGAAA TGGGGCTCAG AAGTTGAATC ACTATTCTTG TATGTGTGAT -----GCTAG
GAAGGATCCT GGCTACAAAT CTCAAGTGGT TCTGTGAAAC CAACATTGGG ATAGTTACTC
AATGTTGTTT GTCAGCCCTG CAAATAAAAT GACAGTATCT TGCAAACTTG GCTCTCAAGA
TGAATGCCAA GTTGGAGGCA GCAACGTGGA GCTGACCGGC TTCCTCA--- --TTGAGAAT
GATATGTAAT GTTTGTAGGT GCTGATGTCA ATCATCCGCT TGGAAAGCCC TTCCATAGCT
GCTGTTGTTG CCACTGTAAA TTGGCCTGCT GTAAATCGTT ATGCTGCTCG TGTACGCCCC
CAGCTCCATC GAACTGAGAA GATTCTAAAT TTT------- ----GGGGAC ATGTGCCTGG
AGCTTATTGA GACCTATGCT CAGGTGAATA GCCAAGCCGG ATAAGATTGT GGTGTTTAGG
GATGGGGTGA GCGAGGGCCA ATTTGACATG GTGCTTAATG AAGAATTAGT AGATCTGAAG
GGGGCATCCA G-----AGGG GAAATACAAC CCAACAATCA CACTTATTAT AACCCAGAAG
AGACATCAGA CTCGACTGTT TCCGCAAGAG GGAAATGAGA ATGTGTCTCC AGGCACGGTT
GTAGACACAA CTGTGGTTCA CCCCTTCGAG TTTGATTTTT ATCTTTGTAG CCATTATGGT
GGTATTGGGA CGAGCAAGCC AACACACTAC CATGTCCTCT ATGACGAGCA CAGGTTCTCT
TCTGACCAAC TCCAGAAGCT TATCTACAAC TTGTGTTTCA CCTTTGTGCG GTGTACCAAA
CCTGTCTCCC TTGTTCCTCC TGTATACTAT GCCGACCTTG CTGCATACAG AGGACGGCTC
TACCAT---
>Ptrichocarpa_POPTR_0016s02480
TTCTTGTTCG CCAGTCATTC TTTCATAATC CAAAGATCTG GGAGTCCTTG GATGCCGAGG
ATTTCATTCG AGCTTTCGAG CCTTGCAGGG TGGTTATCCC TAAATATGGA TGTTCGACTA
CAACGATAAT ACAGCCCCAC TATTGACTTT CCAACCAGAA GACTGAAGGC AAAGCGGACA
ATGAAAAATT TGAGGATAAA GGTGTCACCT ACGAGTACAG AATCACTGGC CTAAGATAGT
TGCAAAGAGC AATGTTTTCG CTGAAATCTT TGACATTACA GTTTATGATT ATTTTGTTAA
TCATCGCAGC ATAGATTTAC GTTACTCATT TGCCATGCAT CAATGTTGGC AAGCCTAAAG
GCCTACTTAC TTCCCGTCGA GCTTGTTCAC TGCTTTCTTT GCAACGCTAC ACAAAGCTGA
CTGTCCATCA GAGATCGCAG TTGGTAGAAG ACATTTCTTG TGTGTCTTCT CCTGAAGGAA
GAACTCTGAC ATATATGGCT TGGAAACGAA AGAATCTTGC AGAATATGGA ATTTTCAACC
AATGCCTGGC ACC------- --ACTAGAAT GACAGTATAT ATTGAATGTT CTCCTGAAGA
TAAATGCTAA GTCGGTGGTT TGAATTCTTT GTTGAGCAAT CACGAAAATC CCTTGTTTCA

AACCTACAAT AATATTTGGG ATGGATGTAT CACATGGCCT GGCCAATCCC ATCCATTGCT
GCGGTTGTCA GTTCTAGAAA CTGGCCTCTA CTTTCTCGTT ATAGAGCTTC TGTGCGTAGT
CAGTCACCAA AAGTTGAGAT GGTAGATTCT CTTTTTAAGA TGATTGTGGG ATTGTTAGGG
AATTGTTGTT AGACTACTAT AAGAGTTCTG GCAAAGCCAG CTCAGATAAT CATATTCAGG
GATGGAGTTA GTGAGTCGCA GTTTAATCAA GTCCTCAACA TCGAGCTGGA TCAAATCATT
GAGGCTGCAA GTTCCTGATG AAAGTGGTCA CCCAAGTTCA CTGTAATTGT TGCACAGAAA
AATCACCACA CTAAATTCTT CCAGATCTCC AGA------A ATGTTCCTCC TGGAACTGTT
ATTGATAATG CTGTTTGTCA CCCACAAACC TATGATTTCT ACATGTGTGC CCATGCAGGG
ATGATAGGAA CAACTAGGCC AACACATTAT CATGTTCTTT TAGATGAGAT TGGCTTTTCA
GCTGATGATC TACAGGAGTT GATTCACTCT TTGTCTTATG TGTACCAAAG AAGCACAACA
GCAATATCCC TAGTTGCTCC AGTCCGGTAC GCACACTTGG CAGCAACTCA GATTTCACAA
TTCTTGAAG
>contig11958_taeda
---------- ---------- ---------- ---------- ---------- ----------
---------- ---------- ---------- ---------- ---------- ----------
---------- ---------- ---------- ---------- ---------- ----------
---------- ---------- ---------- ---------- ---------- ----------
---------- ---------- ---------- ---------- ---------- ----------
---------- ---------- ---------- ---------- ---------- ----------
---------- ---------- ---------- ---------- ---------- ----------
---------- ---------- ---------- ---CTTCTTG TTGTTATTTT CCTGAAACAA
TGGTTCT--- TTGTATGGAC TTGAAGCGTA TATGTGAGAC TGACCTTGGT TTGGTTTCCC
AGTGCTGTCT GACAAACATG TCTTTAAAGC AACAATATCT TGCAAATGTT GCTCTTAAGA
TTAATGTAAA GTTGGTGGAA GAAACACTGT CTTGACGCAC TGTCAAGATA CCTGGTCAGT
GACCTACAAT AATCTTTGGA GCAGATGTTA CCCATCCCCT GGAGAAGCCC ATCAATAGCT
GCTGTTGTAG CATCTCAAGA TTGGCCTGAA GTCACTAAAT ATGCAGGCTT AGTTTGTGCT
CAGGCACATC GTCAGGAACT CATTCAGGAT CTTTACAAGC AACTGGAGGC ATGATAAAGG
AATTGTTGAT TTCCTTCAGA AGGAATACAG GACAAGCCGG AGCGTATAAT ATTTTACAGA
GATGGAGTAA GCGAGGGCCA GTTTTATCAA GTTCTGTTGT ATGAGTTAGA TGCTATTCGA
AAGGCTGTGC ATCTCTGAAC CAAATATCAA CCTCCAGTTA CTTTTGTGGT GGTTCAAAAA
CGTCACCATA CTAGACTCTT TGCACCATCA AGAAGTGGAA ACATATTGCC AGGCACTGTA
GTGGATTCCA AAATTTGCCA TCCAACAGAA TTTGACTTTT ACCTCTGCAG CCATGCTGGT
ATTCAGGGAA CTAGTAGGCC TGCCCATTAT CATGTTCTTT GGGATGAAAA CAAATTCACA
GCTGATGGAT TGCAGTCCTT AACCAACAAT CTCTGTTACA CATATGCGAG GTGCACACGG
TCAGTTTCTA TAGTACCCCC AGCGTATTAT GCCCATCTGG CTGCATTTCG GGCTCGGTTT
TATATGGAG
>Gmax_Glyma14g04510
TGCTTGTGCG GCAATCGTTC TTTCACAATC CCAAGATGTA GGTGTACTTG GTTGCAGGGG
CTTCCACTCT AGTTTTAGAA CTACTCAAAG TGGCTGTCTT TGAACATAGA TTCTCAACCA
CCATGATAAT TACCCCCCTG TGTTGACTTC CCAATCAAAA GATTGAAGGC CAAGAGGACA
TTAAAAAACC TGAGGATTAA ATCGAGCCCA TCGAATTCAA AATCACTGGG CTTAGTCCCT
TGCAAAGATC AATGTTTACA TTGAAGAAGA AGAAGTGACA GTATATGATT ATTTTGTTAA
TATCCGCAAG ATAGATCTTC GGTATTCATC TCCCATGTAT CAACGTTGGG AAACCAAAAG
GCCAACTTAC TTCCTCTTGA GCTTGTTCTT TGGTATCACT TCAACGTTAT ACAAAACTAT
CCACACTTCA AAGGTCTTCA TTGGTGGAAG GCATTTCTTC TTTGTCTGCT CCTGAAGGAA
AAATTCTGAT CTTTATGGCA TGGAAGAAGA AGAATCTTGC TGAGTTTGGA ATTGTGACTC
AGTGTATAGC TCCACAAGGG TCAATGA--- --CAATATTT GACTAATGTT CTGTTGAAAA
TCAATGCTAA GTTGGTGGTC TGAACTCAAT GTTGAGCATT CTCCTTCATT CCTTGTTTCT
AGCCAACCAT CATTATTGGC ATGGATGTGT CTCATGGCCA GGGCAATTCC TTCAATTGCT
GCGGTGGTCA GCTCTAGAGA ATGGCCTCTA ATATCAAAGT ATAGGGCCAG TGTCCGTACG
CAGTCTCCAA AGATGGAAAT GATTGATAAT TTGTTCAAGA GGATGAAGGC ATAATGAGGG
AGCTTCTACT TGATTTCTAT ACAAGTTCTG GGAAAGCCCG ATAATATAAT CATATTCAGG
GATGGTGTTA GTGAGTCCCA GTTCAATCAA GTTTTGAACA TTGAACTTGA TCAAATTATC
GAGGCTGCAA GTTTTTGATG AAAATGGAAT CCCAAGTTTT TGGTGATTGT TGCTCAAAAG
AACCATCATA CTAAATTCTT TCAGAGCTCC TGA------A ATGTTCCTCC TGGAACGGTA
ATTGATAACA AAATTTGTCA TCCTCGGAAT TATGATTTCT ACATGTGTGC ACATGCTGGA

ATGATTGGTA CTAGCAGGCC TACACACTAC CATGTTCTCT TAGACGAGAT TGGCTTTTCA
CCTGATGATC TACAGGAGCT TGTGCATTCA TTATCATATG TGTATCAGAG GAGCACAACT
GCCATTTCTG TCGTTGCTCC AATATGCTAT GCTCATCTGG CTGCAACTCA GATGGGTCAA
TTTATGAAA
>Csativus_Cucsa.185140
TTTTGGTAAG GCAGTCGTTC TTTCATGACT CAAGGATGTA GGGGTAACAG GAGTACGGGG
ATTCCATTCT AGCTTTAGGT TGGCACAGGA TGGTTATCAT TGAATATGGA TTTTCTACCA
CAATGATCCT GAAGCCCCAG TATTGATTTC CAAATCAGAA GATTGAAGGC AAAAAAAATG
TTAAAGAATT TGAGAGTCAA GGCAAGACAT CGGAATTTAA AATCATTGGT TTAAGAGCCT
TGTAACCAAC ATTTTTTTCC ATGAAACTGT TGATATTACT GTTTATGAAT ACTTTGTCAG
ACACTGTGGC ATTGAACTGA CTCATTCATT TGCCATGTCT AGATGTTGGG AAACCTAACG
ACCAACTTAT TACCTTTGGA GTTTGCTCAC TTGTTTCACT TCAACGGTAC ACAAAATTGT
CTTCAATGCA AAGAGCATCT TTGGTAGAAG GCATTTATTC TTTGTGTCCT CCAGAAAGAA
AAATTCGAAT ATTTACGGCC TGGAAGAAAA AGTGTTTGTG CGACTTTGGG ATTTTTACAC
AGTGCATTTC CCCACTAAAA TTAATGA--- --CAGTACAT TACTAATGTA CTTCTTAAGA
TTAACTCCAA GTGGGAGGTA TAAACTCATT GTTGAGCATG CATCATGGTT CCTGATAAAA
GACCAACGTT GATCTTGGGA ATGGATGTGT CTCATGGCCT GGACGGTTCC ATCCATTGCT
GCAGTTGTTG GATCCAGATC CTGGCCTTTG ATATCAAGGT ATAGAGCAGC TGTACGGACC
CAGTCGCCTA AGGTGGAAAT GATTGATGCT CTATTTAAAA GGATGATGGT ATCATTAGAG
AATTGCTTTT AGATTTCTAT AGCACCAGCA AGCAAACCAA CTCAGATTGT TGTCTTTAGA
GACGGAGTTA GTGAATCTCA ATTTAATCAA GTTTTGAACA TTGAGTTGGA TCAAATAGTC
AAGGCTACCA ACATCTGGAG AGGTAACATT CCAAAGTTCA CGGTTATTAT AGCACAAAAA
AATCACCATA CAAGATTTTT TCTGTGCCTC TGA------A ATGTTCCACC TGGAACGGTT
GTTGACACGA AAGTTGTACA TCCAAAAAAT TACGACTTCT ACATGTGTGC TCATGCGGGA
ATGATCGGCA CGTCAAGGCC AGCACACTAC CATGTCCTGC TCGATGAAAT AGGTTTTTCT
CCTGATGATC TTCAAAATTT TGTTCATTCA CTTTCATACG TGTATCAAAG GAGCACAACC
GCACTTTCAA TTGCTGCACC AATATGTTAT GCGCATCTTG CTGCAGCACA GATGAGCCAG
TTTATTAAG
>Cpapaya_evm.TU.supercontig_47.31
TTATTGTGGG AAGATCGTTT TTCTCTGACA TGAGAACCTT GGCGTGGACT TTTGGTGGGG
ACATTACCAA AGCTTGAGAC CAACCCAGAT GGGTTATCCC TTAATATTGA TTGTCTGCCA
CGGCATTCTA TCAGCCCTGG TACTGATTTT AGAATTTGAA GATCGAAGGT CAGAAGGGCC
TTGAAATTGT TAAAGGTATC ACTTACCTGT GGGCTTATAA AATTTTTGGC ATATCAGCCA
TTAAGCGAAC TACGTTTTAT CTTGATGACC AAATATGTCC GTGGTCCAAT ATTTTTATGA
AAAATACAAA ACTAAGCTCA AATTTACCTT TGCCTGCAAT TCAAGCTGGA AGTGTATCAG
TCCTGCATTT TGCCTATGGA GGTTGCCAAA TTGCTGATAG ACAGAGATAT ACAAAGTTAA
ACGCTAGACA GATAACTAAC CTGTTAAGTG TCGTTATTGA TTGTAATTTT CCCGA-----
-GTGACTGAC TCATATGGAA ATTAAAAGAG TGTGTGAAAC AGAATTAGGA ATTGTCTCAC
AATGCTGTCA ACCGGACAAG CAAAGAAAGC AACAATACTT TGAAAATATC GCTCTCAAGA
TAAATGTGAA GCTGGGGGAC GAAACACTGT ATTGATGCTA TTGAGGGATT CCTTCTTACT
GACCTACCAT CATTCTTGGA GCTGATGTGA CCCACCCCCT GGCAATTTCC TTCAATAGCA
GCAGTGGTGG CTTCCATGGA CTGGCCTGAA GCAACCAAGT ATCGAGGAAT TGTATCTGCT
CAGTCACACC GAGAAGAAAT TATACAAGAT CTTTATAA-- ---------- ----------
---------- ---------- ---------- -ATCCTCCTG AAAGA----- ----------
---------- ---------- ---------- ---------- ---------- -------GGT
CGTGCTGTGC CTCCCTGAGG AAGGTATCTT CCGCCAGTTA CATTTATTGT GGTGCAGAAA
AGGCATCATA CACGTCTTTT CCCACCACAC AAGAGTGGCA ATATTCTACC AGGTACCGTG
GTTGACACCA AGATTTGCCA TCCCAGAGAA TTCGATTTTT ATCTGAATAG CCATGCTGGA
ATTCAGGGCA CTAGTCGTCC TGTGCACTAC CATGTTCTGT GGGATGAGAA CAAATTCACT
GCTGATGCAT TGCAAGTGCT CACTAATAAT TTGTGTTACA CCTATGCAAG GTGCACACGG
GCTGTTTCAA TTGTGCCTCC TGCTTATTAT GCGCATCTAG CTGCTTTTCG AGCTCGGTAT
TATTTAGAA
>Cpapaya_evm.TU.supercontig_26.59
TTCTTGTTAG GCAGTCTTTC TTTCATGATC CGAAGATGTG GGTGTCTTAG GTTGTAGAGG
ATTTCATTCA AGCTTCAGGG CCTCTCAGGG AGGTTGTCTT TAAATATTGA TTGTCAACTA
CAATGATAAT ACGGCCCCTG TGTGGATTTC AGAATCAAAA GATTGAAGGC TAAAAGAACA

CTGAAAAATT TGAGGATCAA GGCCAATCCC TCGAATACAA AATAACTGGT TTGAGGACCA
TGCAAGGAGC AACGTTTGTA TTGAAGAAGC TGAAGTGACT GTGTATGATT ACTTTGTTAA
TACTCGTGGT ATAGAGTTGA GGTACTCATT TGCCTTGCAT TAATGTTGGA AAGCCGAACG
CCCCACTTTT TCCCACTTGA GCTTGCTCTT TGGTGCCCTT GCAGCGATAC ACAAAACTTT
CAACTCTCCA AAGAGCTTCG CTAGTGGAAG GCATTCCTTC TTTGCTTACT CCTGAAGAAA
GAACTGTGAC ATTTATGGCT TGGAAACGGA AGAATCTTGC TGAATTTGGT ATAGTCACTC
AATGCATTGC TCCACAAGGG TCAATGA--- --CAATATCT CACTAATGTT TTACTGAAGA
TCAATGCAAA GTTGGTGGAT TGAATTCAAT ATTGAACATT CTCCTGCATC CCTTGTTTCA
AACCTACTAT CATCCTTGGG ATGGATGTTT CACACGGCCT GGGCAGTTCC ATCAATTGCA
GCAGTTGTCA GCTCTAGGCA GTGGCCATTG ATCTCACGAT ATAGAGCTTC TGTGCGTACA
CAGTCCCCCA AGGTTGAAAT GATTGATTCT CTTTTCAAGA GGATGATGGT ATCATGAGGG
AGCTGCTGCT GGACTTCTAT ACGAGTTCTG GGAAAACCTG ATCAGATCAT CATATTCAGG
GATGGAGTAA GTGAGTCACA ATTCAATCAA GTTCTTAACA TCGAACTTGA TCAAATTATT
GAGGCTGTAA ATTTCTGATG AATCTGGTCC CCAAAGTTCT TGGTGATCAT TGCACAGAAG
AACCATCATA CGAAATTTTT CCAGCTCCCC AGA------A ATGTTCCACC TGGAACTGTT
ATTGACAGTA AAATTTGTCA TCCACGAAAC AACGATTTTT ATCTATGTGC TCATGCTGGC
ATGATTGGAA CTACAAGGCC TACACACTAT CATGTTCTGT TAGATGAGAT TGGCTTCTCT
GCAGATGATC TCCAAGAGCT AGTTCATTCA CTATCTTATG TATATCAAAG AAGCACAACT
GCCATTTCTG TAGTTGCACC TGTATGCTAT GCTCATCTGG CAGCCACCCA GATGGGACAG
TTTATTAAG
>LOC_Os03g58600
TCACAGTGTC CAGATCCTTC TTCTCCCAGT TTGGGACATT GGACTTGAGT GTTGGAGAGG
TTACTATCAG AGCCTGCGCC CAACACAGAT GGGCTTTCGC TGAATATAGA TTATCTGCAA
CGTCCTTTTT TAAGCCACAG TATCCAATTT AGTTCCTGAA GATCGAAGAT AAAGAAAGCA
TTACGTGGGG TTCGCATTGA AACAAACCAC CAAGATACAA GATAACAGGG ATTACTTCCT
ATGAGCCAGC TATATTTCCT GTTGATGAAC AAGGAAGACT GTTGTTCAGT ACTTCTGGGA
TAGGTACAAT TACAGACTGA AGTACGCCTT GGCCCTGCCT ACAGTCTGGC AGTGATTCCG
CCCTGTATAC TACCTATGGA GGTTGCAAGA TTGTAGAAGG GCAGAGGTAC TCCAAGCTTA
ATGACAAACA AGTGACCAAC ATCCTTAGTG TCACTTTTAA TTGTAATTCT CCTGAGTTAG
TGGTTCT--- ---TATGGAA ATTAAAAGGG TCTGTGAGAC TGACCTTGGC ATTGTATCTC
AATGTTGTTT GCCAGGCATG CCAGCAGAAC AACAATATTT GGAAAATGTT GCACTCAAAA
TCAATGTCAA GTCGGAGGGC GCAACACTGT TCTCGAGCCT TTATCCGATA CCTTGTGTCA
GACCAACAAT CATCTTTGGC GCTGATGTCA CACACCCCCT GGAGAGCATC ATCTATTGCT
GCGGTTGTGG CATCTATGGA TTGGCCTGAA ATCACCAAAT ACCGAGGTCT GGTCTCTGCT
CAACCACATA GACAGGAGAT AATAGAAGAT CTCTTTAGGT AAATGGTGGG ATGATCAGGG
AGTTGCTTAT CGCATTCCGC AAGAAGACTG GGAAGGCCTG AGAGGATAAT CTTCTATAGA
GATGGTGTAA GTGAAGGTCA GTTCAGCCAT GTGCTTCTTC ATGAAATGGA TGCCATCAGA
AAGGCTGTGC ATCTTTGAGG AGGGTATCTA CCACCTGTCA CATTTGTAGT AGTTCAGAAA
AGGCATCACA CAAGGCTTTT CCCTTCATGG GAGAGCGGAA ACATCCTTCC TGGAACTGTC
GTGGACCGTC AGATTTGCCA TCCTACAGAG TTCGATTTCT ACCTGTGTAG CCATGCTGGC
ATACAGGGTA CTAGCAGGCC AACTCATTAC CATGTCCTTT ACGATGAGAA CCATTTTACA
GCCGATGCAC TTCAGTCCCT GACCAACAAT CTTTGCTATA CCTATGCGCG ATGCACCCGG
GCAGTGTCTG TGGTCCCACC GGCCTACTAT GCTCATCTTG CTGCATTCCG CGCTCGCTAC
TACGTGGAA
>Ppatens_1904560_locus
CTCCTGTAGG ACGCTCGTTC TATTCCAATC TGGGCCGTTA GGTCTTGAAA GCTGGCGGGG
CTTTTATCAA AGTATTCGAC CAACTCAAAT GGGTTGTCCC TAAACATTGA CTGTCTTCCA
CAGCGTTCAT AGAACCACTG TATGGAGTTT ATCTCCTCAA GATCGAAGAT CAAGAAAGCT
CTTCGCGGAG TCAAAGTGGA AGTGACTCAT CGAAATACCG TATATCTGGG CTTACAGGCT
ACTAATGAAC TGAATTTCCA GTTGATGAAC ATTGAAGTCT GTCACCGACT ATTTTCGGGA
AACATACGGC TATTTTATCC GTCATCCCTT TGCCGTGCCT TCAAGTGGGT AATTCTCTCG
TCCCAACTAC TTCCCATGGA AGTTGCAAGA TCGTTGAAGG CCAGCGATAT TCGAAATTGA
ATGAGCGCCA AATTACAGCT CTTCTCAATG CCACTCCTTA TTGCAATCCT CCAGAAACAA
CGGGCCT--- TTGTATGGAT TTGAAGAAGC AATGTGAGAC TGTACTGGGT GTGGTTTCTC
AGTGTTGTTT GACAAGCATG TTTTCAAAGC AACAGTACCT GGCAAACGTG GCTCTCAAAA
TTAATGTCAA GTTGGGGGTC GGAACACTGT CCTGATGCAC TTACACGATT CCTCGTCAGT

GACCCACCAT AATATTTGGT GCAGATGTTA CTCATCCCCT GGAGAAGTCC CTCCATTGCT
GCAGTGGTAG CTTCGCAGGA TTGGCCCGAG GTCACCAAGT ATGCAGGGTT GGTGTGTGCT
CAAGCTCACC GTCAGGAGTT GATTCAGGAT CTGTACAAAT GACAGGAGGG ATGATAAAGG
AACTCCTTAT CTCTTTCCGG TGTGCGACCG GAAAAGCCAC TTCGGATTAT CTTTTACAGA
GATGGAGTAA GTGAAGGTCA ATTTTACCAG GTTCTTCTGT ATGAACTGGA CGCAATCAGG
AAAGCTGCGC TTCCCTGAAC CGGATACCAG CCTCCAGTCA CATTTGTGGT CGTTCAGAAA
AGACACCACA CTCGTTTATT TGCATCATAA TGAAGTGGCA ACATTTTACC AGGGACTGTA
GTGGACTCAA AGATCTGTCA TCCTACAGAA TTCGATTTCT ATCTTTGTAG TCATGCTGGG
ATTCAGGGAA CCAGTCGACC TGCGCATTAC CATGTGCTGT GGGATGAGAA CAAATTTTCT
GCGGATAGTT TACAGTCGTT GACAAATAAT CTGTGCTATA CATATGCACG GTGCACGCGC
TCTGTTTCTA TTGTTCCTCC AGCATATTAT GCACATCTAG CTGCTTTTCG TGCCAGGTTC
TACATGGAT
>AT1G48410.2
TTCCGGTGGG CCGGTCCTTT TATTCTGATA TAGGTCATTG GGCTTGGAGA GCTGGCGTGG
ATTCTACCAA AGCATTCGTC CTACACAGAT GGGTTATCAC TCAATATTGA TTGTCATCGA
CAGCCTTCAT AGAGGCCCTG TATTCAGTTT ATTTGCTTAA GATCGAAGAT AAAAAAGGCT
CTTAGAGGTG TCAAAGTTGA AGTGACTCAT CGAAGTACCG CATTTCCGGT TTGACTGGCC
ACTCGGGAAT TACATTCCCA GTAGATGAAC TCAGAAATCT GTTGTAGAAT ACTTCCACGA
AACATATGGT TTTCGCATTC AGCACACAAC TACCATGCTT GCAAGTTGGG AATTCTAAAG
GCCTAATTAC TACCAATGGA GGTTGCAAGA TTGTTGAAGG CCAGCGGTAT TCCAAATTGA
ATGAGAGACA GATCACTGCT TTGCTGAATG TCACTGCTTA TTGTCATTCT CCCGAAATAA
TGGATCA--- TTATACGGAT TTGAAACGCA TATGTGAGAC TGAACTCGGC ATAGTCTCTC
AATGTTGCCT GACAAGCATG TCTTTAAAGC AACAATACAT GGCTAATGTT GCGCTGAAGA
TTAATGTGAA GTTGGAGGAA GAAACACAGT GCTGATGCTC TATCTAGATT CCTAGTCAGT
GACCCACCAT TATATTTGGT GCTGATGTTA CCCACCCCCT GGAGAAGCCC ATCTATTGCT
GCTGTTGTGG CATCTCAGGA TTGGCCTGAA ATCACTAAAT ATGCTGGATT AGTTTGCGCT
CAAGCGCATA GGCAGGAGCT CATTCAGGAT CTGTTCAAGT GACTGGTGGC ATGATAAAGG
AGTTGCTCAT AGCCTTCCGT AGATCAACTG GATAAACCAC TAAGGATCAT CTTCTACAGG
GATGGAGTCA GTGAGGGACA ATTTTACCAA GTTTTGCTCT ATGAACTTGA TGCCATCCGC
AAGGCTGTGC TTCGCTGAAG CAGGTATCAA CCACCAGTGA CATTTGTGGT GGTGCAGAAG
CGTCATCACA CGAGGCTGTT TGCACCACAA TGAAGTGGGA ATATTTTACC TGGCACTGTT
GTGGACTCTA AAATCTGCCA CCCTACAGAG TTTGACTTTT ACCTCTGTAG TCATGCTGGT
ATTCAGGGCA CTTCTCGACC TGCTCATTAC CACGTTCTTT GGGATGAGAA CAACTTTACT
GCAGATGGAC TTCAATCTCT GACCAATAAC TTATGTTACA CGTATGCAAG ATGCACACGC
TCAGTTTCAA TTGTTCCCCC TGCATATTAT GCACATCTAG CAGCTTTTAG GGCTCGATTC
TACATGGAG
>Cpapaya_evm.TU.supercontig_1.68
TATCTGTAGG GAGATCTTTC TATTCTTCAA TGGGGAAATT GGAGCTGTTG GTTTGAGAGG
GTTCTTTCAG AGTCTTCGAC CAACCCAGCA GGGCTTGCTC TCAATGTGGA TTCTCTGTTA
CTGCTTTCCA TGAGAGAAGC GCTCGAGTTT ATCTTCCTCA GAAAGGAAGT AGAGAAGGCA
TTGAGGAACA TCCGGGTTTT TGTTTGCCAT AGAGGTACCG GGTTTACGGC TTAACAAGCT
ACCGAAAATC TTGGTTTGCA GACAGGGAAA GAATCTGAGA CTGCTAACTT ACTTCAAAGA
TCATTACAAC TATGATATAC AATTCAGACT TGCCATGCTT GCAGATTAGT AGAAGC--AA
GCCATGTTAT TTCCTATGGA GCTTGTATGA TATGTGAAGG CCAAAAGTTT CTTGGTCTTT
CAGATGATCA GACTGCAAGA ATACTTAATG CCACTGCTGA TGTGCATAAT GAGAG-----
-AAGCACAAG GGATATGCAT TTGAAGCGAA TAGCAGAGAC AAGTGTGGGA GTAGTAACCC
AATGTTGTTT GTACCAAACC TTAGCAAAGT TCCAATTCCT GGCTAACTTA GCTCTCAAAA
TGAATGCTAA GTTGGGGGCT GCACAGTTGC TTTAACTCGC TACCGTCATT CCTTCAGCTC
GACCTGTGAT GTTTATGGGT GCTGATGTTA CTCATCCCCT CTCGAAGCCC ATCTGTTGCT
GCTGTTGTAG GAAGCATGAA CTGGCCTGCA GCCAACAAGT ATGTTTCAAG AATGAGGTCC
CAAACCCATA GACAAGAAAT TATCCAGGAT CTT------- ----GCTTCG ATGGTAGGGG
AGTTACTAGA TGATTTCCTC CAAGAAATAA CAACTTCCCA AAAGAATCAT ATTCTTCCGG
GATGGAGTAA GTGAAACCCA ATTTAACAAG GTGCTTAAAG AGGAGCTACA AGCTATTAGA
GAAGCTGTTC AAGATT---C CTGGTATAGA CCTCCCATTA CTTTTTCTGT AGTTCAAAAG
AGGCATCACA CAAGGTTGTT TCCCTTCCTC CCAGAAGAAA ACATTCCTCC GGGAACTGTG
GTAGACACCG TGATTACTCA CCCGAGAGAA TTTGATTTCT ACCTGTGTAG CCACTGGGGA

GTAAAAGGAA CCAGCCGTCC AACACATTAC CATGTCCTGT GGGATGAGAA TCATTTCACT
TCTGATGAAC TCCAAAAGCT GGTTTACAAT CTGTGCTACA CATTTGTGAG GTGTACGAAG
CCAGTTTCTT TAGTTCCCCC TGCCTACTAT GCTCACTTGG CTGCATATAG AGGCAGACTT
TACCTTGAG
>Csativus_Cucsa.112480
GTCCAGTGGC CCGATCATTT TACTCAGACC TTGGACACTG GGGCTGGAAA GTTGGCGTGG
TTTCTATCAG AGTATCCGCC CTACTCAAAT GGGCTCTCCC TTAATATTGA TTGTCATCTA
CTGCTTTCAT AGAGCCCATG TATTGAATTT AACTTCTCAA GACCGAAGAT AAAGAAGGCT
CTTCGAGGTG TCAAGGTTGA AGTGACACAT CGAAATATCG CATTTCTGGT CTGACAAGCA
ACACGCGAGC TACTTTCCCT GTCGATGAAC TATGAAGTCA GTGGTTGAGT ACTTCTACGA
AACGTATGGG TTTGTTATCC AACATACAGT GGCCTTGTCT TCAAGTAGGG AATCAGCAAG
ACCTAATTAT TACCGATGGA AGTTGTAAGA TTGTTGAAGG CCAGAGGTAT TCCAAACTTA
ATGAAAGACA AATAACTGCT CTGCTCAATG CCATTGCTTA TTGTTGTATT CCAGAAACAA
CGGATCC--- CTTTATGGAT TTAAAGCGTA TCTGTGAAAC TGATCTTGGC CTCGTTTCCC
AGTGCTGTTT GACAAACATG TTTTCAAAGT AACAGTATTT GGCCAATGTG GCCTTAAAAA
TAAATGTTAA GTTGGGGGAA GGAATACGGT TCTGATGCAT TATCTAGATA CCTGGTTAGC
GACCTACCAT TATATTTGGC GCCGATGTAA CTCATCCCCA GGAGAAGTCC ATCCATTGCT
GCTGTTGTGG CTTCTCAAGA TTGGCCGGAA GTTACAAAAT ATGCTGGTTT GGTTAGTGCT
CAAGCCCACA GGCAAGAACT CATTCAAGAT CTTTTCAAGT AACGGGTGGA ATGATCAAGG
AACTCCTCAT CTCCTTCCGT CGAGCAACTG GAGAAACCTC AACGCATTAT ATTTTACAGG
GATGGGGTTA GCGAAGGACA GTTCTACCAA GTTTTGTTGC ATGAGCTGGA TGCCATTAGA
AAGGCTGTGC TTCTTTGAAC CAAATACCAA CCTCCAGTGA CATTTGTGGT GGTACAGAAG
CGTCATCACA CTAGGTTGTT TGCACCATTC TGAAGTGGGA ACATATTACC TGGCACGGTT
GTCGATTCTA AAATTTGTCA CCCCACGGAG TTCGATTTTT ACCTGTGCAG TCATGCTGGA
ATCCAGGGTA CAAGTCGTCC TGCTCATTAC CATGTTCTAT GGGATGAGAA CAAGTTCACT
GCTGATGGAT TGCAGACCCT AACGAATAAT CTTTGTTACA CATATGCTAG GTGTACTCGC
TCCGTTTCTA TTGTGCCCCC AGCGTACTAC GCTCATCTTG CTGCTTTCCG AGCTCGTTTC
TATATGGAA
>Rcommunis_29813.t000096
TACCTGTAGG GAGATCATTC TATTCTTCAA TGGGGAAATT GGGGCTGTTG GATTGAGAGG
ATTCTTTCAA AGTCTTAGAC CTACACAGCA AGGCTAGCTC TCAATGTAGA CTCTCTGTGA
CTGCTTTTCA TGAAAGAAGC GCTTGATTTT ACCTTCCTCA GAAAGGAAGT AGAGAAAGCC
TTAAAGAACA TCAGGGTCTT CGTTTGTCAC AGCGATACCG GGTTTATGGC CTAACAAGCT
ACAGAAAATC TTGGTTTGCC GACAGAGAAA GAACCTGAGG CTACTGAGTT ATTTCAAGGA
TCACTACAAC TATGATATAA AATTTAGACT TGCCATGCTT GCAAATTAGT AGAAGC--AA
ACCATGTTAT TTCCCATGGA ACTTGTATGA TCTGTGAAGG CCAGAAGTTT CTTGGGCTCT
CAGATGATCA GACAGCAAGA ATACTTAATG CCACTGCTTA TATGTATAAT -----GAGAA
GAGACACAAA GGATATGCAT TTGAAGCGAA TAGCAGAGAC AAGTGTTGGC GTTGTAAGCC
AGTGCTGCTT ATTCCAAATC TTGGGAAAGT TCCAATTTCT GGCTAATTTG GCTCTGAAGA
TCAATGCCAA ATTGGAGGAT GCACAGTTGC TTTAATTCGC TACCCTCATT CCTTCATTCT
GACCTGTGAT CTTTATGGGA GCTGATGTTA CTCATCCCCT CTTGAAGTCC ATCTGTTGCT
GCTGTTGTTG GGAGCATGAA CTGGCCGGCA GCAAACAAGT ATGCCTCGAG GATGAGGTCA
CAAACTCATC GACAAGAAAT TATCCAGGAC CTT------- ----GGTGCA ATGGTGAAAG
AATTGCTAGA TGATTTTTTC CAAGAAGTTG GAACTTCCCA AGCGGATAAT ATTCTTTAGA
GATGGAGTAA GTGAAACCCA GTTTCATAAG GTTCTTCAAG AGGAGTTGCA AGCGATTAGA
GAAGCTGTTC TAGATT---C CTGGTATAGA CCTCCCATTA CTTTTGCAGT AGTCCAAAAG
AGGCATCACA CTAGGTTGTT TCCGCGAAAC TGAGATGAAA ATATACCGCC AGGGACAGTT
GTGGATACCG TGATTACTCA TCCAAAGGAA TTCGATTTCT ATCTATGCAG CCATTGGGGG
GTGAAAGGAA CAAGTAGACC AACTCATTAC CATGTCTTAT GGGATGAGAA CCAATTCACT
TCTGATGAAT TACAGAAGTT GGTTTACAAT CTGTGCTATA CATTCGTAAG GTGCACCAAG
CCAGTTTCTT TAGTCCCTCC TGCTTACTAT GCTCACTTGG CTGCATACAG GGGTAGACTT
TACCTTGAG
>CL344_lactuca
---------- ---------- ---------- ---------- ---------- ----------
---------- ---------- ---------- ---------- ---------- ----------
---------- ---------- ---------- ---------- ---------- ----------

---------- ---------- ---------- ---------- ---------- ----------
---------- ---------- ---------- ---------- ---------- ----------
---------- ---------- ---------- ---------- ---------- ----------
---------- ---------- ---------- ---------- ---------- ----------
---------- ---------- ---------- ---CTGCTCA TTGTCATTCT CCAGAAACAA
TGGATCC--- CTCTATGGAC CTGAAACGAA TCTGTGAGAC TGATCTTGGC GTTGTTTCTC
AGTGCTGTCT GACAAACATG TTTTTAAAGC AACAGTACCT TGCAAACGTG GCTTTGAAGA
TTAACGTGAA GTTGGAGGAA GGAACACGGT CCTGATGCAA TTTCAAGATA CCATGTCAGC
GACCAACTAT CATCTTTGGT GCTGATGTCA CCCATCCCCC GGGGAAGCCC CTCTATTGCA
GCTGTTGTTG CCTCTCAAGA CTGGCCTGAG ATAACAAAGT ATGCTGGGTT GGTTTGTGCA
CAAGCCCACC GTCAAGAACT GATCCAGGAT CTGTACAAAT GTCTGGTGGC ATGGTCAAGG
AACTGCTGAT ATCTTTTCGC AGAGCAACTG GAGAAGCCAA AGCGGATTAT TTTTtacagg
GATGGAGTGA GCGAGGGGCA GTTCTATCAA GTTTTACTTT ATGAGCTTGA TGCGATTCGA
AAGGCTGTGC ATCGTTGAGC CAGATATCAG CCTCCAGTGA CATTTGTGGT GGTTCAAAAA
CGTCATCACA CGCGATTGTT TGCACCATCG TGAAGTGGGA ATATACTGCC AGGCACCGTT
GTCGACTCCA AGATATGTCA TCCAACCGAG TTTGATTTCT ATCTATGCAG CCATGCTGGG
ATTCAAGGGA CAAGCCGTCC GGCCCATTAC CATGTGCTGC GGGATGAGAA CAAGTTCGCA
GCTGATGCTT TGCAATCACT CACAAACAAT CTCTGTTACA CGTATGCAAG GTGCACTCGT
TCTGTGTCCA TTGTGCCACC TGCATACTAT GCTCATCTGG CTGCCTTCCG TGCACGGTTC
TATATGGAG
>Alyrata_16042271_locus
CGTCGATTGG AAGATCGTTT TACTCTTCTA TGGGGAGATT GGAGCTGTTG GACTCAGAGG
GTTTTTCCAG AGTCTTAGGC AGACTCAACA AGGTTAGCAC TTAACATGGA TTCTCAATCA
CAGCTTTCCA TGAAAGAAGC GCTCGAGTTT ACCTTTCTAG GAGGAGAAGT GGAGAAAGCA
CTTAAGAACA TAAGAATCTT TGTTTGCCAT AGAGGTATCG GGTTTACGGG TTAACAGATT
ACCGATAATA TTGGTTTCCG GATAGAGAAA GAACTTAAGG CTTATGAGCT ACTTTAAAGA
TCATTATGGT TATGAGATTC AGTATAAACT TACCGTGTCT GCAAATCAGT AGGGCA--AG
ACCTTGCTAC TTCCTATGGA ACTTGTATGA TCTGTGAAGG TCAAAAGTTT CTTGGGCTTT
CGGATGATCA AGCTGCAAAG ATCATGAATG CCACTGATTA TCTGTGTAAT -----GAGAA
AAAACATAAA GGGTACGGAT CTTAAGCGAA TAGCAGAGAC AAGAATTGGT GTTGTGACGC
AATGCTGCTT ATACCTAACA TCACTAAAGT TCCAGTTCGT TTCAAACTTA GCTCTCAAGA
TAAACGCCAA GTCGGCGGAT CCATGACCGA GCTAACTCAA TACCTTCATC CCTTAGACCC
GACCGGTTAT CTTCATGGGA GCTGATGTAA CGCATCCCCA TTCGAAGCCC TTCAGTAGCG
GCTGTGGTCG GGAGCATAAA CTGGCCAGAA GCTAACAGAT ACGTCTCGAG AATGAGATCT
CAGACTCATA GGCAAGAGAT CATTCAAGAT CTT------- ----GACTTG ATGGTCAAGG
AACTTCTTGA CGATTTCTAC AAAGCGGTAA AAGCTTCCGA ATCGAATCAT ATTCTTCAGA
GACGGTGTGA GCGAGACACA GTTCAAGAAA ATCCTCCAAG AAGAGCTTCA ATCCATCAAA
ATCGCTGCTC TAAGTT---C AGGATACAAT CCAAGCATCA CATTCGCCGT GGTCCAGAAA
AGACACCACA CAAGGCTGTT CCGGCAATCC AGAAATGAGA ACATCCCTCC TGGCACAGTG
GTTGATACAG TGATAACTCA TCCGAAAGAG TTTGATTTCT ATCTCTGTAG CCACTTAGGA
GTAAAAGGCA CGAGCAGGCC AACGCATTAC CACATCCTAT GGGACGAGAA CGAGTTTACT
TCAGACGAAT TGCAGAGACT TGTGTATAAC TTGTGTTACA CTTTCGTGAG GTGCACGAAA
CCTATTTCGA TTGTACCACC GGCTTATTAT GCACACCTTG CTGCGTACAG AGGAAGGCTC
TACATCGAG
>Sbicolor_Sb02g032980
TTGCAGTTGG CCGGTCCTTC TTCTCACTTG TAAAAATCTT GGTGTGGAGG GATGGAATGG
TTTCTATCAG AGCATCAGGC CGACACAGAA GGGCTGTCTG TGGTCGTAGA CTGTCTTCAA
CAGCTTTTGT TCGACCCCAC TATTGAATTT AGATTCTGAA GAGCTAAGCT CAAGAAAGCC
CTCAGGGGTG TGAGGATTGA AGTCACACAC CGAAGTACCG GATTGCCAGC CTGACGTCCT
CCTTCTTTAC ATTCTTTGAA TCGTCCGCGT TCAGAAGTCT GTCGCAGATT ACTTCAGAGA
GGCATACAAT CTGGAAATGC ACTACGACTC TCCCATGCCT CCAAGTTGGC AGTGATGAAG
GCCGAACTAC TCCCTATGGA GGTTGCAAGA TAGTAGCTGG ACAGCAATAC CGGAAGTTGG
ATGGCCAACA AGTCCTTAAT CTAATGGATG CCTCTTTTGC TTGCTATACT CCAGA-----
-AACGGCAAC TTATATGGAT TTCAAAAGGA TCTGCGAGAC AGAGATTGGT ATCATGTCGC
AGTGTTGCCT GGAAAAAATG TTAGAAGGGT CCCCATACTT TGCTAATGTT GCTATTAAGA
TCAATGCCAA GTTGGAGGAA GGAACTTAGA ATT------A ATCCCAATTA CCTTGTTTCG

ATCCAACAAT TATATTTGGT GCCGATGTCA CTCACCCGCT CTGGAGCCCC TTCCATTGCT
TCTGTTGTTG CCTCCCAAGA CTGGCCCACG GTGGCTAACT ATAATGGCAT TGCCCGTGCA
CAAGGTCACC GTAAAGAGCT CATCGATGGC CTG------- ----GAAGAC ATTGTCAAGG
AACTCCTACT TGCATTTCAG GAACGGTCTA AAGAGGCCCA AGCAGCTGAT CTTCTACAGG
GATGGCGTAA GTGAGGGCCA ATTCAAACAA GTGCTGGAAC AAGAAATCCC AGAGATAGAG
AAGGCTGGAA AGCTCTTACA ATGA---AAG CCAAAGATCA CCTTCATAGT GGTGCAGAAG
AGGCACCACA CAAGGCTCTT CCC-----AA TGAAGTGGAA ATATTCTACC TGGCACTGTA
GTTGATAAGA GTATCTGCCA CCCAACAGAA TTTGATTTCT TCCTGTGCAG CCATGCTGGT
ATCAAGGGAA CAAGCCGTCC TACGCATTAC CATGTGCTGC GAGATGACAA TAAGTTCACT
GCAGATGCTC TGCAGTCTCT CACATATAAC TTATGTTACT TGTATTCAAG CTGCACTCGC
TCTGTTTCAA TCGCTCCTCC CGCATACTAC GCCCACAAGC TAGCGTTCCG TGCCCGCTTC
TACATCAAC
>Mesculenta_cassava27530.m1
TATCAATTGG AAGGTCCTTC TATTCTGATA TAAAAAGCTG GGTTTAGAAT CATGGCGGGG
CTTCTACCAA AGTATAAGAC CAACTCAAAT GGGCTATCAC TGAATATTGA TTGTCGGCTA
CGGCTTTCAT TGAACCCTTG TATTGATTTT AAATTTTGTG GATCGAAGGT CAAGAAAGCT
CTTAGAGGTG TCAAAGTTGA AGTCACTCAC AGAAATACCG AATTTCCGGG TTGACAGCCT
ACAAGAGAGC TATCTTCCCA CTCGATGGAA CATGAAATCA GTTGTTGAGT ACTTTCAAGA
GATGTACGGC TATACCATTC AGTATCCATC TACCTTGCCT GCAAGTAGGA AACCAGAGAA
GGTGAACTAT TGCCGATGGA GGCTGCAAGA TAGTCCAGGG ACAAAGATAT ACAAAACTAA
ATGAGAAGCA GATCACTTCT TTGCTAAATG TCATTGCTTA TTGCAATTCT CCTGAAGCAA
TGGCTCA--- TTGTATGGAT TTAAAAAGAA TTTGTGAAAC TGATCTCGGG TTGATTTCCC
AGTGCTGCCT TACAAACATG TCTTCAAAAC AGCAGTACTT GGCAAATGTG TCACTCAAAA
TCAACGTTAA GTGGGAGGAA GAAATACTGT GCTGATGCTT TGAGTTGATT CCTGGTCAGC
GACCAACAAT AATTTTTGGG GCTGATGTAA CACATCCTCA GGAGAAGTCC ATCTATAGCT
GCTGTTGTAG CCTCCCAAGA CTGGCCTGAA GTTACAAAAT ATGCCGGATT AGTATGCGCT
CAGCCTCACC GGCAAGAACT TATTCAAGAT TTATTCAAGT CTCTGGAGGC ATGATCAGGG
AGCTCTTACT TTCATTTAAG AAAGCCACTG GAAAAGCCAT TGAGGATAAT ATTTTACAGG
GATGGTGTAA GCGAAGGACA GTTCTACCAG GTTCTGTTGT ATGAACTTGA CGCGATTCGA
AAGGCTGTGC ATCGCTGAAC CCAGTACCAA CCTCCAGTGA CATTTGTGGT TGTCCAAAAG
CGGCACCATA CTAGACTCTT CACATCACAA CGAAGTGGAA ATATTTTACC TGGTACTGTA
GTGGATTCTA AAATATGTCA TCCGACTGAA TTCGACTTCT ATTTATGTAG TCATGCAGGA
ATCCAGGGCA CCAGCCGGCC TGCCCATTAT CATGTGCTGT GGGATGAGAA CAACTTCACT
GCAGATGAGA TTCAATCTCT GACAAACAAC CTCTGCTATA CGTATGCAAG GTGCACACGC
TCGGTTTCTG TAGTTCCTCC TGCATATTAT GCTCATTTGG CAGCTTATCG AGCTCGGTTT
TACATGGAA
>Ptrichocarpa_POPTR_0001s22120
TTACTGTTGG GAGGTCATTC TTCTCTGATT TGGGGAGCTT GGAATAGAAT ACTGGAGAGG
TTATTATCAA AGTCTTCGGC CAACCCAAAT GGGCTCTCTT TAAATATTGA TTGTCAGCCA
GATCCTTTTA TGAGCCTTGG TACTGAGTTT AGTACTTCAA GATCGAAGGT GAAAAGGGCC
TTAAGAGGAA TCAAAGTGGA AATTTCATAC AGAGCTTCAA GGTTACTGGC ATATCTACCA
GTAGACAAGA CATGTTTACT CTGGATGAAC AAAGGTGTCT GTCCATCAGT ATTTCTGGGA
CAGATACAAT ATTGGGCTGA AATATACCTT TGCCTCCCCT TCAAGCCGGA ACTGATGCAA
ACCCATTTAT TGCCCATGGA GCTTGTAAGA TTGCTGGAGG ACAGCGATAC ACAAAGCTAA
ATGAAAGACA AGTCACTGCA CTTTTAAGTG TCACTGCTGA TCATTATTCT CCTGATTCAG
TGGATCC--- ---TATGGAA ATCAAACGTA TTTGTGAAAC TGAGTTGGGA ATAGTTTCAC
AATGCTGCCA GCCCAGCAGG CAAAGAAAGC AACAATACCT GGAAAATGTT GCTCTCAAAA
TTAATGTGAA GCTGGTGGAC GGAACACTGT GTTGATGCTA TTCAAAGATT CCATGTTACT
GACCTACTAT TATTTTTGGT GCTGATGTGA CCCATCCCCA GGGGAAGCCC GTCGATTGCA
GCAGTAGTGG CTTCTATGGA CTGGCCAGAG GTAACCAAAT ATAGAGGACT TGTCTCTGCA
CAGGCTCATC GCGAGGAAAT TATTCAGGAT CTTTACAAGT TCACAGCGGA ATGATCAGGG
AGCTGTTTAT TGCATTCAGA AGATCAACAG GAAAAACCTC ATAGAATTAT ATTCTATAGA
GATGGTGTAA GTGAAGGCCA ATTCAGCCAA GTTCTGCTAC ATGAGATGCA GGCGATACGA
GAGGCTGTGG CACCCTGAAG AAGGTATTGT CCTCCGGTTA CCTTTGTTGT AGTGCAGAAA
CGGCATCATA CACGCTTCTT TCCACCATAG CAAAGTGGCA ATATCCTACC AGGCACGGTC
GTGGACACTA AAATTTGCCA CCCTACAGAG TTTGATTTCT ACCTTAACAG TCACGCTGGA

ATTCAGGGAA CTAGCAGACC TACACACTAC CATGTATTGT TTGATGAAAA CAACTTCACT
GCTGATGGCT TACAAACCCT CACTAACAAT TTGTGCTACA CGTATGCAAG ATGCACTCGG
TCTGTTTCCA TAGTGCCCCC TGCATATTAC GCCCATTTGG CAGCTTTTAG GGCTCGGTAT
TACATTGAG
>Bdistachyon_Bradi1g36910
TGCCGATAGG GCGCTCGTTC TACTCAGACA TACGCGTCTC GGTCTGCAGT CCTGGTGTGG
GTTCTACCAG AGCATTCGGC CAACTCAGAT GGGTTGTCGC TTAACATCGA TTGTCGTCCA
CTGCATTCAT CGAGCCCCGG TATCGAGTTC AGATTTTAGG AACAGAAGAT CAAGAAAGCA
CTGCGGGGTG TGAAAGTTGA AGTTACTCAC CGAAGTACCG TATTTCAGGG GTGACAACCA
ACACACGAAC TATTTTCCCA ATTGACGAAA TATGAAATCA GTTGTAGAGT ATTTCAAGGA
AATGTATGGG TTCACAATTC AGCATGCATC TTCCTTGCCT TATGGTGGGA AACCAAAAAA
GGCAAACTAT TACCAATGGA GGCTGCAAGA TTGTTGAGGG CCAGAGATAC ACAAAGTTGA
ATGAAAAGCA GATCACCTCG CTGCTAAATG CCACTTCTTT TGGCCATTCT CCTGAAACAA
TGGTGCT--- TTATATGGAT ATCAAACGTA TCTGTGAAAC TGATTTGGGA TTGATATCAC
AATGTTGCTT AACAAGCATG TTTTTAAAGC AACAGTACTT GGCAAATGTT TCGCTTAAAA
TCAATGTTAA GTGGGAGGAA GAAACACTGT GCTGATGCGC TAAGTTGATT CCTGGTCAGT
GACCAACTAT TATATTTGGT GCGGATGTAA CACATCCACC GGGGAAGTCC ATCCATCGCT
GCAGTTGTGG CTTCTCAAGA CTGGCCAGAA GTTACGAAGT ATGCTGGATT GGTGTGCGCG
CAGGCGCATC GGCAAGAGCT CATTCAGGAC CTTTATAAGT CACAGGAGGC ATGGTCAGGG
AACTCTTAAT ATCCTTCAGG AAGGCCACTG GAGAAGCCAT TAAGAATAAT TTTCTACCGT
GATGGTGTTA GCGAAGGCCA ATTCTACCAA GTTCTCCTGT ATGAGTTAGA TGCTATCCGT
AAGGCTGTGC ATCCCTGAAC CAAATACCAA CCTCCTGTAA CATTCGTGGT CGTTCAAAAG
CGTCACCATA CAAGGCTGTT TGCATCACAA GGAAGTGGAA ATATTTTGCC TGGAACTGTT
GTTGATTCGA AGATATGCCA TCCAACGGAG TTTGATTTCT ACCTCTGTAG TCATGCTGGA
ATCCAGGGAA CAAGTAGGCC AGCTCACTAC CATGTCCTCT GGGATGAGAA CAATTTCTCA
GCAGACGAGA TGCAGACGTT GACAAACAAC CTTTGCTACA CGTACGCACG GTGCACACGC
TCTGTTTCTG TCGTCCCTCC TGCATACTAT GCGCATTTGG CTGCATTCCG GGCACGGTTC
TACATGGAG
>Ppatens_1885752_locus
CTCCCGTTGG GCGCTATTTC TATTCCGATC TGGGCCATTA GGTCTTGAAA GCTGGCGGGG
CTTTTATCAG AGTATTCGAC CAACTCAAAT GGGTTGTCCC TAAACATTGA CTGTCTTCCA
CAGCGTTCAT TGAACCACTG TATAGAGTTT ATCTCCTGAG GATCGAAGAT CAAGAAAGCC
CTCCGTGGGG TGAAGGTGGA AGTCACTCAT CGAAGTATCG CATATCTGGG CTCACAGGCC
ACCAGTGAAT TCAATTCCCT GTGGACGAAC TATGAAGTCT GTTACTGACT ACTTTCGCGA
GACTTATAGC TACACAATTC GACATCCCTT TACCCTGTCT CCAAGTGGGA AATACTCACG
TCCCAATTAT TTCCAATGGA AGTTGCAAAA TTGTTGAGGG CCAGCGATAT TCCAAACTGA
ATGAGCGCCA AATTGCTGCA CTTCTACATG TCACTGCTTA TTGCAATTCT CCCGAAACAA
CGGGTCT--- TTGTATGGAT CTTAAGAAAC AGTGTGAGAC TGTCCTGGGG GTCGTGTCTC
AATGTTGTTT GACAAACATG TATTTAAAGC AACAGTACTT GGCAAATGTT GCCCTGAAAA
TCAATGTCAA GTTGGAGGCC GAAATACTGT TCTGACGCTC TTTCTAGATT CCTTGTCAGT
GACCCACCAT TATATTCGGT GCAGATGTCA CTCATCCCCC GGAGAAGCCC TTCTATTGCG
GCTGTTGTGG CATCGCAGGA TTGGCCTGAG GTCACCAAGT ACGCTGGTTT AGTGTGCGCT
CAAACTCACC GTCAAGAGTT GATTGCGGAC CTATTCAAAT GTTTGGAGGG ATGATTAGGG
AACTTCTCAT CTCTTTCCGG AGCGCAACTG GAAAAGCCTC TCCGTATCAT CTTTTACAGA
GATGGAGTAA GCGAGGGCCA GTTTTCACAG GTTCTTCTTC ATGAGCTAGA CGCAATCAGG
CGAGCTGTGC TTCGCTGAAG AAGGTACCAA CCTCCAGTCA CGTTTGTAGT GGTTCAGAAA
AGGCACCACA CTCGTTTGTT TGC------- -GAAGTGGCA ACATTCTACC AGGTACGGTT
GTGGACTCTA CAATCTGTCA CCCTACGGAA TTCGACTTCT ATCTTTGCAG TCACGCTGGA
ATTCAGGGTA CCAGTCGACC TGCACATTAC CATGTATTGT GGGACGAGAA TAGTTTTTCT
GCTGACAGTT TGCAGTCATT GACAAACAAC CTATGCTATA CGTATGCACG GTGCACACGG
TCAGTGTCAA TCGTCCCCCC CGCATATTAT GCTCATTTGG CCGCTTTCCG AGCGAGATTC
TACATGGAT
>Alyrata_16062911_locus
TCTCTGTTGG GAGGTCTTTT TTCCATAGTT TGGGGAGCTT GGTATTGAGT ACTGGAGAGG
TTTTTTCCAA AGTCTAAGGC TGACTCAGAT GGGTTGTCTC TTAACATCGA CTTTCAGCAA
GATCATTCTA TGAACC---G TACTGAGTTT AGTTTCTGAA GATCGAAGGT GAAGAAAGTT

TTGAGGACAC TAAAAGTTAA GTTGCTACAT TGAGTGCCAA GATTAGTGGG ATTTCGTCCC
ATCAGTCAGC TAGGTTCACT TTAGAGGATC AGAGAAGACG GTTATACAAT ATTTTGCTGA
AAAATATAAT TATAGAGTGA AATACCCCTC TACCTGCTAT TCAAACTGGG AGTGACACAG
ACCCGTCTAC TACCTATGGA ACTTGCCAAA TTGACGAAGG GCAAAGATAC ACCAAACTTA
ATGAGAAGCA AGTGACTGCG TTGCTAAGTG CCACTGTTGA TTGTAATATT CCTGAGTGAC
TGGATCA--- ---TATGGAA ATCAAAAGGA TCTGTGAAAC AGAATTGGGG ATCGTCTCTC
AGTGTTGCCA ACCAGACAAG CTTCTAAAAT AACAGTACAT GGAAAATGTT GCCTTGAAGA
TTAATGTCAA GCTGGGGGAA GGAACACTGT TCTAATGATG CTATTAGATA CCTTATTACT
GACCAACCAT AATCATGGGT GCTGATGTGA CTCATCCCCT GGAGAAGTCC TTCTATTGCT
GCTGTTGTGG CCTCTATGGA CTGGCCTGAG ATAACTAAAT ACCGAGGACT GGTTTCTGCT
CAAGCTCATA GGGAAGAAAT TATCCAGGAC CTGTATAAGT CCACTCTGGT TTGATAAGGG
AACATTTCAT AGCATTCAGG AGAGCTACGG GAGATACCTC AAAGGATTAT CTTCTATCGC
GACGGAGTAA GCGAAGGGCA GTTTAGTCAG GTTCTGCTGC ATGAGATGAC AGCTATACGA
AAGGCTGTAA CTCTCTCAAG AGAATATGTT CCTCGTGTCA CTTTCGTGAT TGTCCAGAAA
CGTCACCACA CTCGTTTGTT CCCAACACGG GAAAGTGGCA ATATTCAACC AGGTACTGTC
GTTGACACTA CAATCTGTCA CCCGAATGAG TTCGACTTCT ATTTGAACAG CCACGCTGGT
ATTCAGGGAA CAAGCAGGCC GGCTCATTAC CATGTTCTTC TCGACGAGAA CGGTTTCACT
GCTGATCAGT TGCAAATGCT CACCAACAAC CTCTGCTACA CGTTTGCGAG GTGCACAAGA
TCTGTGTCAA TTGTGCCACC AGCCTACTAC GCTCACTTGG CTGCTTTCCG TGCCCGCTAC
TACATGGAG
>Cpapaya_evm.TU.supercontig_75.90
TATCAGTTGG GAGATTCCTC TATTCTGAGC TCAACAGCTG GGACTGGAGT CATGGCGCGG
ATTTTACCAG AGTATACGGC CTACCCAGAT GGGTTATCAT TGAATATTGA CTGTCAACGA
CAGCATTCAT TGAACCCCGG TATTGAATTT AATTACTGGG GATAGAAGGT GAAGAAAGCT
CTTAGAGGTG TAAAGGTTGA AGTAACACAT AGAAATACCG GATTTCTGGG CTGACAGCCA
ACAAGGGAGC TATTTTCCCT CTTGATGAAA CATGAAATCA GTTGTTGAGT ATTTCCATGA
AATGTATGGT TATACCATTC AATATGCATT TACCTTGCCT TCAAGTCGGA AATCCAAGAA
AATCAACTAT nnnnnnnnnn nnnnnnnnnn nnnnnnnnnn nnnnnnnnnn nnnnnaCTGA
ATGAGAAGCA AATTACTTCG CTTCTAAA-- ---TTACTGA TTGCTATTCT CCAGAAATAA
TGGTTCT--- TTGTATGGAT TTGAAACGAA TTTGTGAAAC AGATCTCGGG TTGATTTCTC
AGTGCTGTCT CACAAACATG TTTTCAAAGC AGCAGTATCT AGCAAACTTG TCACTTAAAA
TCAACGTCAA GTGGGAGGAA GAAACACCGT GCTGATGCTT TAAGCTGATT CCTGGTCAGT
GACCAACAAT TATTTTCGGA GCTGATGTAA CTCATCCACA GGAGAAATCC ATCAATTGCT
GCTGTTGTAG CGTCCCAAGA CTGGCCGGAG GTTACGAAAT ATGCAGGATT GGTATGTGCT
CAGCCTCATC GACAAGAACT TATTCAAGAT TTATACAAGT TAGTGGAGGG ATGATT----
---------- ---------- ---------- ---------- ---------- -------AGG
GATGGTGTAA GTGAAGGACA ATTCTACCAG GTTTTGCTAT TTGAACTTGA TGCCATTCGC
AAGGCTGTGC GTCACTGAAC CCGGTATCAA CCTCCGGTAA CATTTGTTAT AGTCCAAAAG
CGCCACCATA CTAGACTCTT TGCATCACAA CGAAGTGGGA ATATCTTGCC AGGCACGGTT
GTGGACTCTA AGATTTGCCA TCCAACTGAG TTTGACTTCT ATCTATGCAG CCATGCAGGA
ATTCAGGGAA CAAGTAGACC TGCTCATTAT CATGTTCTGT GGGATGAGAA CAATTTTACT
GCTGATGAGA TTCAGACTCT GACAAACAAT CTCTGCTATA CGTATGCTAG ATGCACTCGA
TCGGTATCAG TAGTGCCTCC TGCATATTAT GCTCATTTAG CGGCTTATCG AGCTCGATTC
TACATGGAA
>Vvinifera_GSVIVT00002671001
TTGTTGTCCG ACAGTCATTT TTTGATCTAC CTAGCCTTTG GGTGTTCTTG GATGTCGAGG
TTTCAATTCA AGCTTCAGAG CTACTCAAGG GGGCTATTCT TGAATATGGA TTCTCTACCA
CACTGGTCAT ACAGCCCCAG TAGAGATTTT CTAATCAAAA GACTGAAGGC CAAAAGGATG
CTAAAAAATC TGAGGGTCAA AACTTTACAC TCGAGTGGAA AATCAGTGGA TTGAGGGACC
TGCAGAAATC AACCTTCTTA ATGAAACAGT TGAAGTAACA GTTTATGACT ATTTTGTCAA
ACATCGAAAA ATAAGTTTAC AGTATTCATT TTCCATGCAT CAACGTTGGG AGATCAAACA
CCCCGTCTAT TTCCTCTCGA GCTTGCACAT TGGTTTCCTT GCAACGCTAT ACCAAGTTGT
CTACTCAGCA GAGATCTTCA CTTGTGGACG ACATTTATTC TTTGTATTCT CCACAAAGAA
GAACTGCGAC ATCTATGGCA TGGAAACGGC AATGCCTTTC AGGTTGTGGA GTTCCCATAC
AGTGCATTGC ACCACACCAg taGTGAA--- --CAATACCT CACTAATGTG TTGTTAAAAA
TCAATGCCAA GTTGGGGGTT TGAATTCTTT GTTGGATATT GTCCATCCTT CATGATTTCA

ACCCCACTTT AATCCTTGGA ATGGATGTGT CCCATGGCCT GGTCGGTGCC GTCTATTGCT
GCGGTGGTAA GTTCCAGGCA TTGGCCATCG ATTTCTCAAT ATAGAGCTAC AGTCCGTACA
CAGTCTCCCA AACTTGAGAT GATAGATTCT CTATTCGAAA AGATAGTGGC ATAATCAGGG
GCACCCTGTT GGACTTCTAT AAAACTTCAG CGGAAGCCTG AACACATAAT CATTTTCAGG
GATGGAGTCG GTGAATCGCA ATTCAACCAA GTCTTGAACA TTGAACTGGA ACAAATAATT
GAGGCTGCAA ACTCCTGATG AGCATGGCAT CCAAAGTTCA TGGTGATTAT TGCACAGAAG
AATCACCATA TAAGATTCTT CCAGATCCCC GTC------A ATGTTCCTCC CGGGACAATC
GTGGACAACA CAATATGTCA TCCAAGAAAC AATGATTTCT ATTTGTGTGC ACATGCTGGG
ATGATTGGGA CAAGCCGGCC TACGCATTAC CATGTATTGC TGGATGAGCT TGGCTTTTCA
GCAGATGACT TGCAGCAACT TGTGCATTCC TTGTGTTACG TATATCAGAG GAGCACCACG
GCTGTATCTC TAGTGGCCCC AGTATGCTAT GCTCACCTGG CTGCAGCCCA AGTTGCCCAG
TTTATCAAG
>Sbicolor_Sb01g011870
CATCGATCTC AAAATCGTTT TTCTCaGCAT TTGGCCTCTA GGTGTGGAAT GCTGGAGGGG
TTACTACCAG AGCCTACGCC CTACACAGAT GGGTTGTCCC TTAATATAGA TTTCTGCAA
CGTCATTTTT CAAGGCCCTG TATTGACTTC ATTATCTGAA GATCGAAACT GAAGAAAGCA
CTTAAGGGGG TCCGGGTCGC AACCAAGCAT AGCGCTACAG GATTACGGGG CTAACCTCCA
TTAAATGATT TACGTTT--- ---GATCAAC AAGGGTGTCA GTTGTGCAGT ACTTCAAACA
ACAATATGAC TACTCATTAA AATACACACT GGCCATGCCT TCAAGCTGGC AGTGCTAGAA
GCAGATCTAT TACCTATTGA GGTTGCAGCA TAGTTGAGGG ACAACGCTAC TCGAGTCTGA
ATGAGAATCA AGTCAGGAAT ATCCTGAATG TGATTACTCA TCATAATTTT CCTGAATGAG
TGGT------ TTTTACGGGG ATAAAACGAC TTTGTGAAAC TGAGCTTGGT TTAATCACTC
AGTGTTGTGC GCCAAGAATG TAAGGAAGGA AACAATATCT TGAGAATCTT TCCTTGAAAA
TCAACGTAAA GTTGGTGGGA GGAACACAGT ACTGATGCTT TAAACCGATA CCTTCTGACA
GACCTACAAT AGTCTTTGGA GCTGATGTTA CCCACCCCCT GGGGATCTCC ATCTATTGCA
GCAGTCGTTG CATCCATGGA CTGGCCACAA GTTACAAAGT ACAAATGCTT GGTATCTTCA
CAAGGTCATA GGGTTGAAAT TATAAATGGT CTTTATACGT CAGAGGTGGA ATGATTAGAG
ATTTGCTTTT GTCGTTCCAC AAGTCAACTG GACAAGCCTT CGAGGATTAT ATTCTATCGT
GATGGTGTTA GTGAGGGGCA GTTCAGCCAA GTCTTGCTTT ATGAAATGGA T---------
---GCTGTGC AAGTTTCAGG AGGGTACCAA CCAAGAGTCA CATTCGTTGT TGTGCAAAAG
CGGCATCATA CTCGCCTGTT TCCATCATCG TGCAGTGGAA ACATCCTGCC TGGAACTGTT
GTCGATACGA AGATCTGCCA CCCCAGCGAG TTTGATTTTT ACCTTTGTAG TCATTCTGGT
ATTCAGGGAA CAAGCCGCCC AGCTCATTAT CATGTTCTTA TGGACGAAAA TGGTTTCAGT
GCTGATGCAC TGCAAACCTT GACTTACAAT CTTTGCTACA CCTATGCCCG GTGCACTCGT
TCAGTCTCTA TAGTTCCTCC GGCGTACTAT GCACACCTGG GTGCGTTCCG TGCCCGTTAC
TACATTGAG
>Mesculenta_cassava13489.valid.m1
CAACTGTCGG GAGGTCATTC TTCTCTAATT TGGGGAGCTT GGTATAGAAT ATTGGAGAGG
ATACTATCAA AGCCTTCGCC CAGCCCAGAT GGGCTGTCTT TTAATATTGA TTGTCAGCCA
GATCCTTCTT TGAGCCATGG TACTGACTTT AATACTTTAG GATCGAAGGT GAAAAGGGCC
TTAAGAGGAG TAAAAGTAGA GCTAAGTCAT TGAGCTGCAA GATCATTGAT TTATCAGCCC
CTGAATCAAA TTTCTTCACT TTTGGAGA-- -AGTGTGTCA GTGGTTCAGT ATTTCCGTGA
TCGATACAAT ATTGGTCTTA AATATACCAT TGCCTGCTAT TCAAGCTGGG AGTGATTCAA
GCCAATTTTC TGCCTATGGA GGTTGTAGAA TTGTTGAAGG ACAGAGATAC TCAATGTTGA
ATGATAGGCA AGTAACTGAA TTGTTAAATG TCATTGTTGA TAATCATTCT CCTGAGTCAT
GGGTTCA--- ---TATGGAA ATTAAAAGGA TATGCGAAAC AGAATTGGGT ATTGTATCTC
AGTGCTGTCA ACCAGTCAGG CTGCAAACGT AGCCGTATTT TGAAAATGTT TCCCTCAAAA
TCAATGTTAA GTTGGTGGTC GAAATACTGT GTTGATGCTA TTCAAAGATT CCTAGTGACT
GACCTACCAT TATTTTTGGT GCTGATGTAA CTCATCCCCT GGGGAAATCC GTCAATAGCA
GCAGTAGTGG CTTCAATGGA CTGGCCAGAG GTAACAAAGT ATAGAGGAAA TGTCTCTGCA
CAGGCTCATC GTGAAGAAAT TATCCAGGAT CTCTATAAAT TCATTCCGGA ATGATCAGGG
AATTATGTAT AGCATTTAGG AGAGCAACTG GATAAACCCA ACAGGATGAT ATTCTACAGA
GATGGTGTTA GTGAGGGCCA ATTTAGCCAG GTTTTGCTAC ATGAGATGGA TGCCATACGA
AAGGCTGTTG CTCACTGAGG AGGGTACCTT CCACGTGTTA CCTTCATTGT GGTGCAGAAA
AGGCATCATA CACGGCTTTT TCCTTCAGGG TGGAGTGGCA ATATTCGACC AGGCACAGTT
ATTGATACCA AGATTTGCCA CCAGAATGAA TTTGATTTCT ACCTCAACAG CCATGCTGGA

ATTCAGGGAA CAAGCAGGCC TGCACACTAT CATGTGTTGT ATGATGAGAA TTGTTTCACA
GCTGATAAGT TGCAAGTTCT GACTAATAAC ATGTGTTACA CGTATGCAAG GTGTACTCGC
TCTGTTTCCG TAGTTCCTCC TGCCTATTAT GCCCACTTAG CTGCTTTTCG GGCTCGATAT
TACATTGAG
>Ppatens_1912837_locus
CTCCTGTAGG ACGCTCTTTT TATTCCAATC TGGGCCGCTA GGTCTGGAAA GCTGGCGGGG
CTTCTATCAA AGTATTCGAC CAACTCAAAT GGGCTGTCCC TAAACATTGA CTGTCTTCCA
CAGCGTTCAT AGAACCACTG TATGGAGTTT ATCTCCTCAA GATCGAAGAT CAAGAAAGCT
CTTCGTGGAG TTAAAGTGGA AGTGACTCAT CGAAGTACCG CATATCTGGG CTTACAGGCC
ACCAATGAAT TGAGTTCCCA GTTGATGAAC CTTGAAGTCT GTTACCGACT ATTTTCGGGA
GACGTATGGC TATGTTATCC GGCATCCCTT TGCCTTGCCT CCAAGTAGGG AATGCTCACG
TCCCAACTAT TTCCCATGGA AGTTGCAAGA TCGTTGAGGG CCAGCGGTAC TCCAAACTGA
ATGAGCGCCA AATCACCGCA CTTCTTAATG TCACTCCTCA TAGCTATTCT CCAGAAACAA
CGGGCCT--- TTATATGGAT CTGAAGAAGC AATGTGAGAC TGTCCTGGGA GTGGTTTCAC
AATGTTGTTT GACAAGCATG TCTTCAAAGC AACAGTATTT AGCAAATGTG GCCCTCAAAA
TTAATGTGAA GTCGGAGGTC GGAATACTGT TTTGATGCAC TTTCACGATC CCTTGTCAGT
GACCCACTAT AATATTTGGT GCAGATGTTA CCCACCCCCT GGAGAAGTCC TTCCATAGCT
GCAGTGGTAG CTTCGCAGGA TTGGCCTGAG GTCACTAAAT ATGCCGGATT GGTGTGCGCT
CAAGCTCACC GTCAGGAGTT GATTCAGGAT CTGTACAAAT GACAGGAGGG ATGATCAAGG
AGCTCCTGAT CTCTTTCTGG CGCGCAACAG GAAAAGCCTC TTCGCATCAT TTTTTACAGG
GATGGAGTTA GTGAAGGGCA GTTTTATCAG GTGCTTCTGT TCGAGCTGGA CGCAATCAGG
AAAGCTGCGC TTCCCTGAAC CGGATACCAG CCTCCAGTCA CGTTTGTGGT TGTGCAGAAG
AGGCACCACA CTCGATTATT TGCATCACAA TGAAGTGGCA ACATTTTACC AGGGACTGTT
GTGGACTCGA AGATTTGTCA CCCTACGGAG TTTGACTTTT ATCTTTGCAG TCATGCTGGA
ATTCAGGGGA CTAGTCGACC TGCACATTAT CATGTACTAT GGGATGAAAA TAAATTTTCT
GCGGACAGTT TGCAGTCGTT GACAAACAAT CTTTGCTATA CGTATGCACG GTGCACACGT
TCAGTATCTA TTGTTCCTCC GGCTTATTAT GCGCATTTGG CTGCTTTCCG AGCGCGCTTC
TACATGGAT
>Smoellindorffii_15412065_locus
TTCCCAGTGG ACGTAACTTC TTCGAGTCAT TCGGGCACTA GGTTTTGAGG CCTGGAAAGG
ATTTTACCAG AGTGTTCGTC CAACCATGCA AGGCTCGTGC TGAACGTCGA TTGTCTGCAG
CAGCATTTTA CGAGGCCCAG TCTCGAGTTC AGTCACTCCC GATCGAAGGC CAAAAATCTC
TTGAACCGGC TCAAGGTCGA AGTCACGCAC AGAGATACAG GATTTCGGGA CTGTCGGCCA
ACAAAGGCGT TACGTTCACA ACTGATTCCA GGAGGTGAAG GTTGTGGACT ACTTTTGGAC
GACGTACAAG CATAAGATCC AGTATCCAGT TGCCTTGCCT CGAGTTGCAA GGAAGG--AA
AACTACTTAT TGCCTATGGA AGTTGCAAGC TGGCTGCCGG TCAGAAATAT CAGGGACTCA
ACGAGAGGCA GACAACAAAC ATGCTGAGTG TCACTGGTAG TTTGTCTCAT GACGC-----
-AAGCACCAG ATTTACGGAC CTGAAGAAGC TGTGCGAGAC GGAGCTCGGG CTCGTGACGC
AAGTGTGCCT TAAAAAAATG TGATGAAAAC AGCAGTACCT GGCCAACCTC GCGATGAAGA
TCAACGTCAA ATTGGAGGCC AGAACATGGA TCTGATCTGC GACTCATGTT CCCGATTCTC
GGCCAACAAT CATCTTTGGA GCTGATGTCT CTCATCCGCT CGCGAAGTCC CTCCATCTCC
GCGGTGGTGG CGAGCATGGA CTGGCCATCG GCGGTCAAGT ATCTCGCTCG CGCGAGATCT
CAGAGGGGCA GGGTGGAGAT GATCGAGCAC CTG------- ----CATGAC ATGGTGGTCG
ACCTCATGAG GGCGTTTTTC ACTCACACCA GTCAAACCGG AGAGGCTTCT CTTCTTCCGG
GATGGTGTGA GCGAGGGCCA GTTCTCCGAC GTCCTGAACA ACGAAGTGCA GGCAATCCGG
AGGGCTTCCT TACCCTCAGC CGAATACTGT CCCCAGATAA CTTTCGTGGT CGTCCAGAAG
CGTCACCACA CGAGGTTCTT CCC-----GC GGAAGCAACA ACGTGAGACC GGGGACCGTT
GTGGACACGG AGATCACCCA TCCTCGAGAG TTCGACTTTT ACCTTTGCAG CCACAGGGGT
CTCCAGGGAA CCAGCCGGCC GACGCACTAC CACGTCCTGC TCGACCAGAA TGGCTTCACG
GCCGACCAGC TCCAGACGCT CGTCAACAGC TTATGCTACA CTTATGCCCG GTGTACCAAG
GCGGTCTCGG TGATACCTCC GGCGTACTAC GCTCACCTGG TGGCCTACCG GTCTCGGCTC
CACGTCGAC
>Sbicolor_Sb09g030910
TTTTGGTTAA GCAATCGTTT TTCTACAACC CTTCGACTTG GGCGTGATGG GTTGCCGTGG
ATTTCATTCA AGTTTCCGCG GCACACAGAG TGGCTTTCCC TGAACGTTGA TTCTCAACAA
CAATGATCGT GAAGCCCCTG TATTGATTTT CTAACCAGAA GATTGAAGGC CAAGCGTGCT

CTCAAGGGCT TGAGGATTAG AACCACTCCT GCGAATTCAA GATTTTTGGT CTCAGGGATT
TGTAAAGAAC AACGTTTCCG CTGAGGCAAT TGATATAACT GTCTATGACT ACTATGCGAA
GAAA---GGA ATTGATCTAA AGTACTCATT TGCCTTGTAT AAATACAGGG AGGGCAAACG
TCCAACATAT TTCCAATCGA GCTTGCTGCC TTGTTCCGCT TCAAAGATAC ACCAAATTGT
CTACGCTACA AAGGTCATCC CTTGTTGAAG ACATTCCTCA TGTGCCTTCT CCTGAAGGAA
AAATTGCGAA GTCTATGGCT TGGAAGAGAA AGTGCCTGGC CGAGTTTGGT ATTGTCACAC
AGTGTCTAGC TCC------- --ACAAGAAT GACCGTACCT GCTTAATTTG CTGATGAAGA
TTAATGCAAA GTTGGTGGTC TGAATTCGTT GCTGAAGCAT CTCCATCATA CCATGTGTCG
GACCCACCAT CATCTTGGGT ATGGATGTGT CTCATGGCCA GGACAAGGCC TTCAGTTGCA
GCCGTGGTTA GTTCTCGTCA ATGGCCTCTT ATCTCTAAAT ATAGAGCATC AGTGCACACC
CAATCCGCCA GACTAGAAAT GATGTCCTCC TTGTTTAAGA TGATGATGGC CTCATTAGGG
AATCACTGAT TGACTTCTAC ACTAGCTCTG GGAAAACCAG ACCACATTAT CATTTTCAGG
GATGGAGTCA GCGAAAGTCA GTTTACCCAG GTCATCAACA TTGAACTGGA TCAGATAATT
GAGGCTGTAA GTTTCTGATG AGAATGGTCA CCCAAGTTCA CTGTGATTGT TGCTCAAAAG
AACCACCACA CCAAGTTTTT TCAGATCACC AGA------A ATGTTCTTCC CGGAACTGTC
GTGGACAATA AAGTTTGCCA TCCTAAGAAC TTTGACTTCT ACATGTGTGC ACATGCTGGG
ATGATTGGAA CAACAAGGCC GACCCATTAT CATGTTCTGC ACGATGAGAT AGGTTTCTCT
GCTGACGAAA TGCAGGAGTT TGTTCATTCG CTCTCTTATG TGTATCAAAG GAGCACAACA
GCCATCTCAG TAGTTGCTCC AATCTGCTAT GCCCACCTTG CTGCAGCCCA GGTGGGCACG
TTCCTGAAA
>Mesculenta_cassava43463.valid.m1
TCCTTGTTCG CCAGAACTTT TTTCATGATC CAAGGATGTT GGTGTTCTGG GTTGTAAAGG
GTTTCACTCG AGTTTTAGAA CCACTCAGGG GGGTTATCTC TGAATATTGA TTATCTACAA
CCATGATAAT TCAGCCCCGG TGTGGATTTT CCAACCAAAA GACTGAAGGC AAAACGAACT
CTTAAAAACC TGAGGATCAA AGCTAGCCCC TCGAGTACAA GATAACTGGT CTGAGAACCA
TGCAGAGAGC AATGTTTCAA CTTAAACACT GGAATTAACT GTTTATGACT ATTTTGTTAA
TCATCGTCAT ATAGATTTGC GCTATTCATT TGCCTTGCAT TAACGTTGGG AAGCCAAACG
CCCTACTTAT TTCCGATTGA GCTTGTACTT TGGTGTCCTT GCAACGATAC ACGAAACTCA
ATACTCTCCA AAGAGCTTCC CTGGTGGAAG GCATTCCTAT TGTGTTTACT CCGGAAGGAA
AAACTCAGAT ATATATGGCT TGGAAGAAGA AAAATCTTGC TGAGTTTGGA ATTGTCACCC
AGTGCCTGGC TCCCAAAGGG TCAATGA--- --CAGTATCT GACTAATCTT CTGTTGAAGA
TCAATGCAAA GTTGGTGGGT TAAATTCTAT GTTGAACATA CTCCTTCATT CCTTGTTTCT
AACCTACAAT CATACTTGGG ATGGATGTAT CACATGGCCT GGGCATGCCC GTCCATTGCT
GCGGTGGTCA GTTCCAGGAA TTGGCCATTG ATTTCTCGTT ACAGGGCATC AGTTCGCACT
CAGTCTCCAA AGGTGGAAAT GATAGACTCT CTATACAAGA GGATGAAGGA ATGATTAGGG
AGCTTCTGTT GGATTTTTAT ACCAGTTCAG GGGAAACCAG ATCAGATCAT CATATTCAGG
GATGGTGTAA GTGAATCTCA ATTTAATCAA GTATTGAATA TTGAACTGGA TCAGATAATT
GAGGCTGCAA GTTTCTGATG AGAATGGAAC CCCAAGTTTG TGGTGATTGT AGCTCAGAAG
AACCACCATA CAAAATTCTT CCAGATCTCC TGA------A ATGTTCCACC TGGTACTGTC
ATAGACAATA AAGTCTGTCA TCCAAGGAAC AATGATTTCT ATCTCTGTGC TCATGCAGGA
ATGATTGGTA CTACAAGACC CACACATTAC CATGTTTTAT TAGATGAGGT TGGCTTTTCA
GCAGACGATC TTCAGGACCT AGTGCATTCT CTATCATATG TTTATCAAAG AAGTACAACT
GCCATTTCTG TAGTTGCGCC AATATGCTAT GCTCACCTGG CTGCCACCCA GATGGGATCG
TTCATGAAG
>Vvinifera_GSVIVT00031924001
TATCTTCTGG TCGGAGCTTT TACCAAGACT CAGGGAGCTT GGTATTATAG CTTCTAGAGG
ATTTCAACAT AGTCTCAAAC CCACTGCCCA GGGCTATCCT TGTGCTTGGA CACTCGGTTG
TGCCATTTTT TAATCCTCGG TTTAGAGTTC AGCATGTTTG TACAGGAGGT TGAGGCTGCC
TTAAAGGGAT ATAAAGTTAG AGTGACTCAC CGAAATTCAT TGTTGCAGGT TTAACAAGAC
ACGCGAAACC TTCATTTCTT CCTGAAGAAA GAAAGTAATG CTTGTTGATT ATTTCTATGA
AAAGTATGGC AAGGATATCG AGAACCAATA TTCCCTGCTT AGATGTGGGA AAAAACAAAG
GAAGAATTAT TACCAATGGA GTTTGCATCT TGGTTGAGGG GCAGAGGTAT ACAAAAATTT
TGGATAAAGA GGCTGCTAAG AGGCTGAACA ACTATTCTTG TGTGTGTCAT GCTAG-----
-AAGGATGCT GGCTATGGAT CTTAAGTGGT TTGCTGAGAC CAAATTGGGG ATGGTTACTC
AGTGTTGTTT GTCCGCCCCG CCAACAAAGT GACACCATCT TGCCAACCTT GCTCTCAAGT
TGAATGCTAA GTAGGGGGTA GCAATGTAGA ACTAAACGAC TTCCACG--- --TTGAAGGT

GACATGTGAT GTTTATTGGT GCTGATGTCA ATCACCCTCT CAGAAAGTCC ATCAATAGCA
GCTGTTGTTG CCACAGTGAA TTGGCCTGCA GCAAACCGCT ATGCAGCTCG AATTCGCCCA
CAAGCCCATC GAATGGAGAA AATTCAGAAT TTT------- ----GGGGCA ATGTGCCTGG
AGCTTGTTGA GACTTATGTT CAGGCAAATA ATCAAGCCAG AGAAGATCGT GGTGTTCCGT
GATGGTGTAA GTGAGGGCCA ATTTGACATG GTTCTGAATG AAGAATTACT TGATCTCAAG
AGAGCATCCA G-----GGGG AAAATACTGC CCGACCATCA CTCTTATTGT GGCCCGCAAG
AGACACCTAA CACGTTTGTT TCC--AAGGT AAAAATGGGA ATGTGCCTCC AGGCACTGTT
GTGGACACAA CAGTGGTCCA CCTATCTGAG TTCGACTTCT ATCTTTGCAG CCACTATGGT
ACACTTGGGA CAAGCAAACC CACGCACTAT CATGTCCTAT ACGATGAGCA CAGGTTTAGT
TCTGACCAGA TCCAGAAGCT TACCTATAAC TTGTGTTTCA CCTTTGCTCG GTGTACAAAA
CCCGTCTCGC TGGTCCCCCC AGTGTACTAT GCTGACCTCG CTGCCTATAG AGGAAGGTTG
TACTATGAT
>Ptrichocarpa_POPTR_0015s15780
TCTCTGTTTC CCGTAGCTTC CATTCAGATC AT--CACCTT GGAATCATAG CATCTAGAGG
GTGCCAACAT AGCCTCAAAC CGACCTCCCA AGGATAGCTT TATGCTTGGA CATTCTGTTC
TGTCATTTCA TGAGCCTCTG TATAGAATTC AACATATTTC AGCTTGCTGT GGAAGGTGTG
CTGAAGGGAC TGAAAGTTAG AGTGACTCAC CGAAATATGT TATTACAGGG TTGACATGAC
GCTCAATATA TACATTTCCT CAAGAAGACA GAATGTTAGG CTTGTTGAAT ATTTCAGGCA
AAAATATCAC AGAGATATAG TGCATCAATA TCCCTTGCCT AGAGATGAAA AGCAAG--AT
GAAGAACTAT TACCAATGGA GTTTGTGTCT TGGTTGAGGG GCAAGTATTT CCAAAATATC
TGAAGGAAAC TGAAGCCAAG ATGTTGAACT AGCTTTCTTC TCTGTGTCAT -----TCAAG
GAAGGATCCT GGTTACAAAT CTCAAATGGA TTTCTGAAAC CAAAGTTGGT ATTGTGACAC
AATGTTGTTT GTCACTCCTG CAAATGAAAG GACAGTACCT TGCTAATCTT GGTCTCAAGA
TTAATGCTAA GTTGGAGGAA GCAATGCAGA GCTGGCAGAC TCCCGTA--- --TTGGGAAT
GACGTGTCAT GTTTATTGGG GCTGATGTCA ATCATCCGCT CAAAAAGTCC ATCCATTGCA
GCTGTTGTTG GTACTATAAA TTGGCCAGCT GCAAACCGCT ATGCAGCTCG AGTTCGTCCT
CAGTATCATC GTAAAGAGCA GATTCTGAAT TTT------- ----GGTGAT ATGTGCTTGG
AGCTTGTTGA ATGTTATTCC CGGCTCAATA ACGAAACCTG AGAAGATTGT GATTTTTCGT
GATGGGGTCA GTGAGGGCCA GTTTGATATG GTTCTTAATG AAGAGTTAAC GGACATAATG
AAGGCTTCAA GTCAAT---- --AATACACC CCAACCATAA CACTCATTGT TGCCCAGAAA
CGGCACCAGA CTCGTCTTTT TCCGGGACGA GGGACTGGCA ATGTGTCTCC AGGCACAGTT
GTGGACACAA CAATTGTTCA TCCTTTTGAG TATGACTTTT ACCTCTGTAG CCACTACGGA
AGTCTTGGGA CAAGCAAGCC CACGCATTAT TATGTTCTAT GGGATGAGCA TGGCCTCAGT
TCGGACGACT TGCAGAGGCT CATATACAAC TTGTGCTTCA CATTTGCTCG TTGCACAAAA
CCTGTGTCAT TAGTCCCACC AGTCTACTAT GCTGACCTTG TTGCTTATAG AGGAAGGCTT
TACCATGAG
>LOC_Os02g45070
CTCCAGTTGC AAGGTCATTT TATTCTAACT TAGGCAACTT GGCCTGGAAA GTTGGCGTGG
TTTTTACCAA AGCATACGAC CCACGCAGAT GGGCTTTCTC TGAATATTGA TTGTCATCGA
CAGCATTCAT TGAGCCCCTG TATTGACTTT AGCTTTTGAA GATCGAAGAT CAAGAAGGCC
CTAAGGGGTG TAAAGGTTGA GGTCACACAT AGAAGTATCG CATTTCTGGC CTTACAAGCA
ACACGAGAGT TTCTTTTCCC ATTGATAAAC TGTGAAGACG GTGGTGCAAT ACTTCCAGGA
GACATATGGA TTTAACATTA AGCACACCTT TGCCTTGCTT GCAAGTGGGC AATCAACAAG
GCCAAATTAT TACCAATGGA GGTTGTAAGA TTGTGGAGGG ACAGCGTTAC TCAAAACTAA
ATGAGAAGCA GATAACTGCT CTTCTTAATG CCACTGCTGA TTGCAATATT CCTGAAATAA
TGGTTCT--- CTTTATGGAT CTCAAAAGGA TATGTGAGAC TGATCTTGGA TTGGTCTCGC
AATGCTGTCT TACAAGCATG TTTTTAAAGC AACAGTATTT AGCAAACGTT GCCCTTAAAA
TCAATGTTAA GTGGGAGGAA GAAATACAGT ACTGATGCTT TGACAAGATT CCTTGTGAGT
GACCAACGAT CATTTTTGGT GCTGATGTTA CACATCCCCT GGAGAAGCCC TTCCATTGCA
GCTGTTGTTG CTTCTCAAGA CTGGCCTGAG GTCACCAAAT ATGCCGGATT AGTGAGTGCA
CAGGCCCATC GTCAGGAATT GATACAGGAT CTTTTTAAGT AAGTGGTGGA ATGATCAGAG
AGCTTCTCAT TTCTTTCAAG AGGGCAACTG GAGAAACCCC AGAGGATTAT ATTTTACAGG
GATGGTGTCA GTGAAGGACA GTTTTATCAG GTTCTGTTTT ATGAGCTTGA TGCCATTAGA
AAGGCTGTGC ATCTTTGAAG CCGATATCAG CCACCCGTTA CCTTTGTGGT GGTCCAGAAG
CGTCATCACA CAAGGCTGTT TGCACCACAA GGAAGTGGAA ATATACTACC AGGCACCGTG
GTTGATTCAA AGATATGCCA TCCTACTGAG TTTGATTTCT ACCTGTGTAG CCATGCGGGC

ATTCAGGGAA CAAGTCGCCC TGCACATTAT CATGTTCTGT GGGATGAGAA CAAGTTCACT
GCTGATGGTT TGCAAACTCT GACAAACAAC TTGTGCTACA CCTATGCAAG GTGCACCCGT
TCCGTATCAA TCGTTCCTCC TGCATATTAT GCCCATCTCG CGGCATTTCG AGCTCGATTT
TACATGGAG
>AT2G27040.1
TGCTTGTTCG ACAGTCTTTT TTCCATGATC CAACCCAGTT AACATCTTAG GATGTAGGGG
ATTTCACTCC AGTTTCAGAA CAACGCAGGG TGGATGTCAC TTAATATGGA TTTACAACCA
CCATGATCAT CAAGCCCCAG TGTTGATTTC CTAACCAAAA GACTGAAGGC TAAACGAACC
CTTAAGAACC TAAGGGTAAA GGTCAGCCCC TCGAATTCAA GATAACCGGA TTGAGAGCCT
TGCAGGGAAC AACGTTTGAA TTGAAGAAAC TGAAGTTACA GTTGCTGACT ACTTCCGCGA
TACAAGGCAT ATTGATTTGC AATATTCATT TGCCTTGCAT CAATGTTGGG AAGCCAAACG
ACCCACTTAC TTCCTCTCGA GCTTGCGCGT TGGTTCCACT TCAGAGGTAC ACAAAACTTA
CCACGTTCCA AAGATCTGCC CTTGTTGAAG ACATTCATAC TATGTGTGCT CCTGAAAAAA
GAACAGTGAT CTCTATGGCA TGGAAGAAAA AAAACTTAAC TGAATTTGGC ATTGTTACTC
AATGCATGGC TCCCGGCAAC CTAATGA--- --CAGTATCT TACTAACTTA CTTCTGAAGA
TTAATGCAAA GTTGGAGGCC TGAACTCAAT GTTGAGCGTA CACCTGCTTC ACTGATTTCT
AACCAACCAT TATCCTTGGG ATGGATGTTT CACATGGCCT GGACAGTCCC GTCCATCGCT
GCTGTGGTGA GTTCTAGGGA GTGGCCACTG ATATCCAAAT ATAGAGCATC TGTTCGGACA
CAGCCTTCTA AGGCTGAGAT GATTGAGTCC CTTGTCAAGA AGACGATGGC ATTATCAAGG
AGTTGCTGGT AGATTTCTAC ACCAGCTCGA AGAAAACCAG AGCATATCAT AATTTTCAGG
GATGGTGTGA GTGAATCTCA ATTCAATCAG GTTCTGAATA TTGAACTTGA TCAGATCATC
GAGGCTGCAA GCTCTTGACG CAAATGGAAC CCAAAGTTCC TTTTGTTGGT GGCTCAAAAG
AATCATCATA CCAAGTTCTT CCACGTCTCC TGA------A ATGTTCCTCC AGGGACAATC
ATTGACAACA AAATATGTCA CCCAAAGAAC AATGATTTCT ACCTCTGTGC TCACGCTGGA
ATGATTGGAA CTACCCGCCC AACTCACTAC CACGTCCTGT ATGATGAGAT TGGTTTTTCA
GCTGACGAAC TTCAGGAACT TGTCCACTCG CTCTCCTATG TGTACCAAAG AAGCACCAGT
GCCATTTCTG TTGTTGCGCC GATCTGCTAT GCTCACTTGG CAGCTGCTCA GCTTGGGACG
TTCATGAAG
>LOC_Os04g52540
TTATCATTGG TCAGGGATTT TACTC----C AGGGCCCATT GATGTTGTAG CTCTCAAAGG
AACCCAGCAG ACCCTGAAAT GCACTCAGAA AGGCTGATCC TTTGTGTGGA CATTCGGTTA
TGCCGTTTCG CAAAGCCCTG TTTGGATCTT AGTCTGTGAG -----ACTTT GAAGAATGAA
CTCAAAGGCC AGCGTGTCAC TGTAAATCAT AGAAGTACAT TGTTAAAGGT TTGACAACCT
GCAAGTCAGA TACTTTTGTA GATTCTGACA GACCAAGAAG CTTCTTGATT ACTATTCGCA
GCAGTATGGC AAGGTTATTG AGTATCATGC TTCCATGCTT GGATTTGAGC AAGAGCAAAA
GCAAAACTAT TGCCGATTGA ATTTGTGATC TTCTTGAAGG GCAGAGATAC CCAAAAAGCT
TAAATAGGAA TTCTGATAAA ACACTGAATT GATCTCCTCT TCTGCCCAAT TCTGA-----
-CAGCATCCT GGGTACAACG CTGAAGCTTA TCTGCGAGAC GCAGCTGGGG ATCCAGACCC
AGTGCTTCTT GAGTTCCTCG CGAACAACAG GGCAGTACAT GTCCAACCTT GCTCTGAAGA
TCAACGGCAA GTTGGAGGAA GCAACATCCA ACTGAATCGC TCCCGCG--- ----ATCTCC
GGCCATACAT GTTCATCGGC GCCGACGTGA ATCACCCCCG GGGAAAGCCC GTCGATTGCA
GCAGTGGTGG CCTCGGTGGA T---CAAGGC GCCAGCAAGT ACGTGCCAAG AATCCGCGCT
CAGCCTCACC GCTGCGAGGT GATCCAGCAC CTC------- ----GGCGAC ATGTGCAAGG
AGCTCATCGG CGTGTTCGAG AAGCGGAACC GTGAAGCCCC AGAGGATCAT CTACTTCCGC
GACGGCGTCA GCGACGGTCA GTTCGACATG GTGCTGAACG AGGAGCTGGC GGACATGGAG
AAGGCATCAA G---AC---A AGGATACTCC CCGACGATCA CCGTGATCGT GGCCAAGAAG
CGGCACCACA CCAGGCTGTT CCCACCTGAA CCAAACGGCA ACGTGCTCCC CGGCACGGTG
GTGGACACCG GCGTGGTCGA CCCGGCGGCG TACGACTTCT ACCTGTGCAG CCACAACGGG
CTGATCGGGA CGAGCCGGCC GACGCACTAC TACAGCCTTC TGGACGAGCA CGGCTTCGCC
TCCGACGACC TGCAGAAGCT GGTGTACAAC CTCTGCTTCG TCTTCGCCCG CTGCACCAAG
CCGGTGTCGC TGGCCACGCC CGTCTACTAC GCCGACCTCG CCGCCTACCG CGGCAGGCTC
TACTACGAG
>Mguttatus_mgf006816m
TACCTTCCGG AAGGTCGTTC TACTCCTCAG TGGGGAAATC GGGGCTATTG CACTTAGAGG
ATTCTTTCAA AGCTTGAGAC CTACACAACA AGGCTTGCTC TCAACGTCGA CTCTCGGTTA
CTGCCTTCCA CGAGAGAAAC GCTCGACTTT ACCTATCTTG GAGGAGAAGT GGAGAAAGCA

CTCAAGAATA TGAGGGTTTT TGTCTGCCAT AGAGATACCG AGTTTACGGG TTGACAGGTT
ACGGAAGATC TTGGTTTCCC GATAGAGAAC GAGTATGAGA CTTACGAGCT ATTTCAAGGA
GCAATATGGG TACGATATAC AGTATATATT TGCCTTGCTT GCAGATTAGT AGAAGG--AA
GCCGTGTTAT TGCCTATGGA GCTTGTGTGA TTTGTGAAGG GCAGAAGTTT CTCGGACTCT
CGGATGATCA AACGGCGAAA ATACTCAATG TCATTGCTCG TTTGTGTTAT GAGAG-----
-AAACACAAA GGGTACGCAC TTAAAAAGAA TTGCCGAAAC TAGGATCGGA ATAGTGAGCC
AATGCTGTCT GTATCGAATC TTGAAAAAGC TCCAGTTTCT CGCAAATTTA GCTCTCAAGA
TAAACGCGAA ATCGGTGGAT GCACTGTCGC CCTAACACAC TCCCTTCATC CCTCAGACAA
GACCCGTTAT TTTCATGGGC GCTGATGTCA CTCATCCCCA TTGGAACCCC TTCCGTCGCC
GCTGTTGTAG GCAGTGTGAA CTGGCCCGCT TCGAATAAAT ACGTCTCGAG AATGAGGTCG
CAAACTCACC GGCAAGAAAT AATCGAAGAT CTT------- ----TGTAAT ATGGTTACGG
AAATATTGGA GGATTTCTTA CACGAGCTCT CAACTCCCGA CTAGGATTGT ATTCTTTCGA
GATGGAGTAA GCGAAACGCA GTTCCACAAA GTGATGCATG AGGAGCTGAA AGCTATTAAA
GAGGCTGCTC GAGATT---T CCGATATGCC CCTCCGATCA CTTTCGCTGT GGTGCAGAAG
CGCCACCACA CCAGATTATT CCCAGAATCA GTT------A ATGTTTCGCC GGGGACAGTG
GTCGACAGTG TGATCGTTCA TCCGAGGGAG TTCGATTTCT ATCTTTGTAG CCATTGGGGT
GTGAAGGGGA CGAGCAGGCC GATTCATTAC CATGTATTAT GGGACGAAAA CAAATTTACG
TCCGACGAAG TGCAGAAGTT GGTCTACAAT CTTTGCTACA CGTTTGTGAG GTGTACGAAG
CCTGTATCTA TTGTGCCTCC CGTTTATTAT GCGCATCTTG CTGCATATAG AGGCAGCTTG
TATCTCGAC
>AT1G31290.1
TCACTGTTGG TAAAAGATTC TTTAGTCGGC TA--GACTTT GGAGTTGGAG CTGCGAAAGG
GTTTCACCAC ACTCTCAAGC CCACAGTACA AGGTTATCAT TATGTTTGAA CCCTCTTTGT
TGGCGTTCCG CAAAGCTCAG TATCGAATAC TGTACTTTGG AATTGGACGT GGTACAAGAA
TTGATTGGTT TGAAAGTCAC TGTTGATCAT CGAAATTCAT CATTATGGGG TTGAGACGAC
ACAAAAGATA TAAATTCGAT TTTATTGAAG GAAAATATCC ATTGTTGAGT ATTTCAAGGA
AAAGTATGGA AGAGACATTG ATCACAAATA TTCCTTGCTT GAATTTGGGG AAAAAGGGCG
GGAAAATTTT TACCCATGGA GTTTGTAACT TAGTCGAGGG GCAGATTTTT CCAAAAAAAT
TGTATAGAGA TTCAGCCGCG TGGTTAAATC ACTCTTGTTC TGTGTGCTAT ACTGG-----
-AAGCACGAT GGATACAACT CTGAAATGGA TAGCCGAGAC CAAACTTGGT CTAGTGACTC
AGTGTTTCTT GACTCTGCCA TTAAAGGtct GACAGTACTT GGCAAATCTC GCCCTCAAGA
TAAACGCAAA GTTGGTGGAA CGAACGTGGA GTTAATATTT TCTCTTT--- --TCAAAAAA
GAAAGGTCAT GTTCATTGGT GCTGATGTCA ATCATCCGCT CACGAAGTCC ATCCATTGTT
GCTGTTGTAG GCACTCTTAA CTGGCCTGAA GCTAACCGCT ACGCAGCTAG AGTCAAAGCT
CAGAGTCACC GTAAAGAAGA GATACAAGGG TTT------- ----GGTGAA ACTTGCTGGG
AGCTTATCGA AGCTCATTCT CAGGCCCCCG AAACGACCTA ACAAGATTGT GATATTCCGT
GATGGTGTCA GCGATGGTCA GTTCGATATG GTTCTCAATG TGGAGTTACA GAATGTTAAG
GACGTTTTGC CAAGGT---- --GGTATAAT CCGCAGATAA CTGTAATTGT GGCACAGAAA
CGTCATCAAA CCCGTTTCTT CCCCAACTAG CAAAAGGGCA ATGTGCCTTC AGGTACGGTC
GTTGATACCA CGATCATTCA CCCGTTTGAG TATGATTTCT ACCTCTGTAG TCAACATGGA
GCGATAGGTA CAAGCAAACC AACTCATTAT TATGTTCTTT CGGACGAAAT CGGGTTCAAC
TCGAATCAGA TTCAGAAACT CATCTTTGAC TTGTGCTTCA CGTTTACTCG CTGCACCAAA
CCGGTCGCTC TGGTTCCTCC GGTTTCTTAT GCTGACAAGG CTGCTTCTAG AGGAAGGGTG
TACTACGAG
>Vvinifera_GSVIVT00020067001
GTCCTGTTGG GAGATCCTTC TTTTCTGATA TTAGCGTCTT GGTTTGGAGT CATGGTGTGG
ATTCTACCAG AGTATAAGAC CTACTCAGAT GGGTTGTCAC TGAATATTGA TTGTCTTCGG
CTGCATTCAT TGAAGCCCTG TATAGAGTTT AGCTATTAGG GACCGAAGAT CAAAAAGGCC
CTTAGAGGAG TAAAAGTTGA AGTAACACAC AGAAGTATCG TGTATCAGGA CTAACAACCT
ACAAGAGAAC TGTTTTTCCT GTTGATGAAC CATGAAGTCA GTTGTGGAAT ACTTCCAAGA
AATGTATGGC TTTACAATTC AACATGCACC TTCCTTGCCT TCAAGTAGGA AACCAGAAAA
GGCTAACTAT TACCTTTGGA GGCTGCAAAA TTGTAGAGGG GCAGCGGTAT ACCAAATTGA
ATGAGAGGCA AATTACTGCT CTATTAAATG CCACTTCTAT TAGCTATTTT CCTGAAACAA
TGGGTCT--- TTATATGGAT CTTAAGAGAA TATGTGAAAC CGACCTTGGT CTAATATCAC
AATGCTGTCT CACAAACATG TCTTCAAAGC AACAGTACTT AGCCAATGTG TCTCTGAAGA
TTAATGTTAA GTGGGTGGTA GAAACACTGT CCTGATGCTA TCAGTTGATA CCTAGTTAGT

GACCAACCAT AATATTTGGA GCAGATGTGA CGCATCCAAT GGAGAAGCCC TTCAATTGCT
GCTGTAGTAG CTTCTCAGGA CTGGCCTGAA GTCACAAAAT ATGCAGGATT GGTTTGTGCT
CAGGCTCACA GACAGGAACT GATACAAGAT TTATACAAGT TAGTGGTGGC ATGATTCGGG
ATCTTTTGGT TTCCTTTCGG AAGGCGACAG GAGAAGCCAC TAAGGATTAT ATTTTACAGG
GATGGTGTTA GTGAAGGGCA ATTTTATCAA GTGCTACTTT ATGAACTAGA TGCAATCCGG
AAAGCTGTGC TTCTCTGAAC CAAATATCAA CCACCAGTTA CTTTTATTGT GGTTCAAAAA
CGACATCATA CCCGATTGTT CGCACCACAG GGAAGCGGGA ATATTTTGCC TGGCACAGTG
GTTGATTCTA AAATTTGCCA TCCGACTGAA TTTGATTTTT ATCTCTGCAG CCATGCTGGT
ATTCAGGGGA CAAGTAGGCC TGCTCATTAC CATGTTTTAT GGGATGAGAA CAATTTCACA
GCAGATGGAA TTCAATCCTT GACGAACAAT CTTTGCTACA CATATGCAAG GTGCACACGA
TCGGTGTCTG TTGTTCCTCC AGCATATTAT GCACATTTAG CAGCATTTCG AGCCCGATTC
TACATGGAG
>Smoellindorffii_15408087_locus
CGCCTGTTGG ACGATCGTTT TATTCAGACT TAGGTCCCTC GGCTTAGAAA GCTGGCGAGG
GTTCTACCAG AGTATAAGGC CCACGCAAAT GGGCTTTCTC TGAATATTGA CTGTCATTTA
CTGCTTTCAT TGAGCCCGCG TGTGGACTTT AACTCCTTAA GACCGAAGAT CGAAAAAGCT
CTGAGAGGGG TCAAAGTGGA AGTTACACAT CGAAATACAG GATTTCTGGC TTAACAGCCT
ACTCAAGAAC TATGTTTCCT GTTGATGAAC AATGAAATCG GTAATGGAGT ATTTCCGAGA
TACATATCAC TACACTATAC GAAGCCCCTT TACCTTGTTT ACAAGTTGGA AATCAAGAAG
ACCAAATTAT TGCCAATGGA GGTTGCAAGA TTGTGGAAGG ACAAAGGTAT ACTAAACTCA
ACGAGCGTCA AGTGACAGCC CTTCTAAATG CCATTACTGA TTGCCATATT CCTGAAACAA
TGGCTCA--- CTTTATGGAC TTGAAACGAA TTTGCGAAAC AGACCTGGGA TTGGTTTCTC
AATGCTTCTT GACAAGCACG TCTTTAAGGA AACAGTGTCT TGCTAATGTT GCGTTGAAAA
TCAATGCCAA GTCGGAGGCA GAAACACTGT CTTGATGCAC TATCTCGCTT CCTAGTAAGT
GACCAACAAT TATATTTGGA GCGGATGTTA CGCATCCCCT GGAGAAGTCC CTCGATTGCA
GCGGTA---- -----AGTGA TTGGCCAGAA GTCACGAAAT ACGCAGGTCT AGTGTGCGCT
CAAGCTCACA GACAAGAGTT GATCCAAGAT TTGTATAAAT GAATGGCGGT ATGATAAGAG
AGCTTTTAAT TTCTTTCCGA AGTGCCTCTG GACAAACCTG GAAGAATCAT CTTCTACAGA
GATGGTGTGA GCGAAGGACA GTTCTACCAA GTTCTCCTTC ACGAGCTGGA TGCAATTAGA
AAGGCTGCGC GTCACTGAGC CGAATATCAG CCTCTCGTGA CGTTCGTTGT GGTCCAGAAA
CGGCACCACA CCCGGCTATT TGCATCACGA CGAAGTGGAA ATATCCTGCC AGGCACTGTG
GTGGATTCGA AGATTTGCCA TCCAACGGAG TTTGACTTCT ACCTTTGCAG CCATGGCGGC
ATTCAGGGAA CAAGCAGACC AGCACACTAT CACGTACTTT GGGACGAGAA CAAGTTCACT
GCCGATGGCT TGCAGTCCCT CACCAACAGC CTGTGCTACA CTTACGCTCG CTGCACACGC
TCAGTCTCGA TAGTACCACC TGCATACTAC GCTCACCTGG CTGCGTTCCG CGCGAGATTC
TACATGGAG
>Mguttatus_mgf016601m
--GCTGTTGG GAGGTCCTTC TTTCAGGAGT TTGAGAGCTT GGCCTGGAAT ACTGGAAAGG
GTTTTATCAG AGTCTTCGCC CAACACAGAT TGGCTGTCAC TAAACATTGA TTGTCGGCTA
GAGCATTTTT TGAACCTATG TTCTGAGTTT AGTACCTCAA GATCGAAGGT GAGAAGAGCT
CTGAAAGGTG TTAAGGTTGA GAACAATCAT CACATCATAA GATAACAGGG CTTTCAACCA
ACACAAAGGC TATGTTTTCT GTGGATGAGC ACAAATTTCA GTTTATCAGT ACTTCCGTCA
GAAATACAAT ATTGTGCTGA AGTATCCTTC TGCCAGCACT TCAGGCTGGC AGTGCTGCAG
ACCTATTTAT TTCCAATGGA GGTTGCAAAA TTGTCGCGGG GCAAAGATAC TCCAAATTGA
ACGAGAGACA AGTTACTCAA CTGCTCCGTG CAATTGCTTC TTATTATATT CCCGAGTGAC
TGGTTCA--- ---TATGGTA ATCAAGAGGG TGTGTGAAAC AGAATTAGAC ATTGTATCAC
AATGCTGTCA GCCAAACAGG TTTTGAAAAT ATCAGTACCT CGAAAATGTA TCACTGAAGA
TCAATGTGAA GCTGGCGGGC GAAATACAGT TTTGAGCAGG CACTGCTATG CCACATTAGT
GACCTACCAT AATATTTGGT GCTGATGTCA CTCACCCCCA GGGGAAGTCC TTCTATAGCT
GCTGTGGTGG CCTCAATGGA TTGGCCAGAA GTCACTAAGT ACAGAGGTTT GGTGTCTGCA
CAAGGCCACA GGGAAGAGAT TATTCAGGAT CTCTACACGT CCATGGTGGA TTGATTCGTG
AACATTTGGT TGCATTCTAC AAGAACACTA AGCAAGCCTA GCAGGCTCAT CTTTTACAGG
GATGGTGTGA GCGAAGGACA GTTTAATCAA GTTCTTCTGT ATGAAATTGA TGCAATTAGG
AAGGCTGTGC TTCGCTCAGG CAGATATCAG CCAAGAATAA CTTTTGTTGT GGTTCAAAAG
AGGCATCATA CTCGTCTCTT TCCACCATAG AAGAGTGGTA ACATTTTGCC AGGTACTGTG
GTTGATACCA AGATTTGCCA CCCCAATGAA TTCGATTTCT ATCTTTGCAG CCATGCTGGG

ATCCAGGGAA CTAGTCGTCC AGCACATTAC CATGTGTTGT ACGATGAAAA TCGCTTCAGT
GCAGATGCAC TGCAAATTCT CACCAACAGC TTGTGTTACA CATATGCAAG GTGCACTCGC
TCAGTTTCCA TAGTCCCACC AGCTTACTAC GCACATCTTG CTGCATTCCG AGCCCGTTAC
TACATTGAA
>AT2G27880.1
TCTCTGTTGG GAGGTCTTTT TTCCATAGTT TGGGGAGCTT GGTATTGAGT ACTGGAGAGG
TTATTTCCAA AGTCTAAGGC TGACTCAGAT GGGTTGTCTC TGAACATTGA CTTTCAGCAA
GATCATTTTA TGAACC---G TACTGACTTT AGTTTCTGAA GATCGAAGGT GAAGAAAGTT
TTGAGGACAC TGAAAGTTAA GTTGCTTCAC TGAGTGCCAA AATTAGTGGG ATTTCTACCC
ATCAGGGAGC TAGGTTCACT CTGGAGGATC AGAGAAGACG GTTGTTCAAT ATTTTGCTGA
AAAATATAAT TATAGAGTGA AATACCACTC TACCTGCTAT TCAAACAGGG AGTGACACAG
ACCCGTCTAC TACCAATGGA GCTTGCCAAA TTGACGAAGG GCAAAGATAC ACCAAACTCA
ATGAGAAGCA AGTGACTGCA TTGCTAAATG CCACTGTTGA TTGTAATATT CCTGAGTGAC
TGGATCA--- ---TATGGAA ATAAAAAGGA TCTGTGAAAC AGAATTGGGG ATTGTCTCTC
AGTGTTGCCA ACCAGACAAG TTAATAAAAC AACAGTACAT GGAAAATGTT GCCTTGAAGA
TCAATGTCAA GCTGGGGGAA GGAACACTGT TCTGATGCTA TTAGAAGATA CCTTATTACT
GACCAACCAT CATCATGGGT GCTGATGTGA CTCACCCCCT GGAGAAGTCC TTCTATTGCT
GCTGTTGTGG CCTCTATGGA CTGGCCTGAG ATAAACAAAT ACCGAGGATT GGTTTCTGCT
CAAGCTCATA GGGAAGAAAT TATTCAGGAC CTGTATAAGT CCACTCTGGT TTGATAAGGG
AACATTTCAT AGCATTCAGG AGAGCTACAG GAGATACCTC AAAGGATCAT CTTCTATCGT
GACGGAGTAA GCGAAGGGCA GTTTAGTCAG GTTCTGCTAC ATGAGATGAC TGCTATCCGC
AAGGCTGTAA CTCTCTCAAG AGAATATGTT CCTCGTGTTA CTTTCGTGAT TGTCCAGAAA
CGTCACCACA CACGTTTGTT CCCAACACGG GAAAGTGGCA ATATTCAACC AGGTACTGTC
GTGGACACTA AAATCTGTCA CCCTAATGAA TTCGACTTCT ATTTGAACAG CCATGCTGGT
ATTCAGGGAA CAAGCAGGCC GGCACATTAC CATGTACTTC TCGATGAGAA CGGTTTCACC
GCTGATCAGT TGCAAATGCT CACAAACAAC CTCTGCTACA CGTATGCGAG GTGTACAAAA
TCTGTGTCAA TTGTGCCACC AGCCTACTAC GCTCACTTGG CTGCATTCCG TGCCCGCTAC
TACATGGAG
>Mtruncatula_Medtr5g094940
TGCTTGTGCG CCAAAATTTC TTTCACGACC CAAAGATGTT GGTGTACTTA GTTGCAAAGG
GCTGCATTCC AGCTTTAGAA CCACACAAAG TGGTTGTCTC TGAACATAGA TTGTCGACAA
CCATGATTGT ACGTCCCCTG TGTTGACTTC AAAATCAAAA GACTGAAGGC TAAAAGGACC
CTCAAGAATT TGAGGATCAC AGCTAAACCA TCGAGTACAA GATAACCGGG CTGAGTGTCG
TGCAAAGACC ACTGTTTACC ATGAAGAAGA AGAGATCACG GTTTATGATT ATTTCGTTCA
TCGGCGAAAA ATTGATCTAC AGTACTCGTC TTCCATGTAT TAATGTTGGG AAGCCAAACG
ACCTACTTAT TCCCAATTGA GCTTGCTCAT TGATATCCCT GCAACGATAC ACCAAACTCT
CCACATCTCA ACGATCCTCC CTTGTGGAAG GCATTTCTGC TTTGTCTGCT CCTGAAGGAA
GAACTCTGAT CTTTATGGCA TGGAAGAAGA AGAATCTTGC GGAGTTTGGA ATTGTTACCC
AGTGCATAGC TCCACCAGGG TGAACGA--- --CAGTATCT GACCAATGTT TTGCTGAAGA
TCAATGCAAA GTTGGTGGGA TGAATTCTTG GTTGAACACT CTCGATCATC CCTTGTGTCG
AACCTACTCT CATTTTGGGC ATGGATGTTT CTCATGGCCT GGGCAATTCC TTCAATTGCT
GCGGTCGTAA GCTCGAGGAA ATGGCCCCTG ATATCTAAAT ACAGGGCATG TGTCCGAACT
CAGGGTTCGA AGGTTGAGAT GATTGACAAT CTGTTCAAGA AGATGAAGGA ATTATAAGGG
AACTTTTGCT TGATTTCTTT CATAGTTCTG AGACGACCAG AGAATATAAT CATTTTCAGG
GATGGTGTTA GTGAGTCTCA GTTCAACGAA GTGTTGAACG TTGAACTCAG CCAAATCATC
GAGGCTGTAA ATTTCTGATG AAAATGGAAC CCCAAGTTTA TGGTGATTGT TGCCCAGAAG
AATCATCACA CAAAATTCTT CCAGATCTCC AGA------A ATGTGCCACC TGGAACTGTT
GTGGACAGCA AAATTTGCCA TCCTAGGAAT TATGATTTTT ATATGTGTGC TCATGCTGGA
ATGATTGGTA CAAGCAGACC AACACATTAT CACGTTCTTT TGGATGAGAT TGGTTTTTCT
CCAGATGATT TACAGGAGCT TGTGCACTCT CTATCTTATG TTTATCAGCG GAGCACCACT
GCTATTTCTG TCGTTGCTCC AATCTGCTAT GCACACTTGG CTGCATCTCA AGTTGGACAG
TTCATGAAG
>Bdistachyon_Bradi5g21810
---------- ---------- ---------- ---------- ---------- ----------
---------- ---------- ---------- ---------- --cgccttga c---------
---------- ------ACGA TCTTGAC--- CAACTCTAGG -----CATTT GGAACGTCAT

CTCAAGGGCC TCTACGTTAC CCTGAATTAC CAAAGTACAA GGTTCACGGC CTGACAGCTT
GCCCACCAGA TACCTTTCCG GATTTCAAGA CCAGCGGAAG CTTCTTGAGT ATTAT-----
---------- ---------- ---------- ---------- ---------- --------CG
TCAGCAGTAT ---------- ---------- --------GG GAAGAGGtat cccAAA----
---------- ---------- ---------- -GACTCCTCT TCTGCCCGAT -----TCTGA
GCAGCATCCG GGGTACAACA CTGAAGCAGA TCTGCGAGAC GAAGCTGGGG ATCCAGACTC
AGTGTTTGTT GAGGAAGCCG CGAATAAcgg gaCAGTACAT GTCCAACCTT GCTCTCAAGA
TCAACAGCAA GTCGGGGGCA GCAATGTCCA GCTGACGGGC TTCCAAA--- --TGGCTGGC
AGCATTTCAT GTTCATCGGC GCGGACGTGA ACCATCCCCC AACGAAGTCA CTCGATAGCA
GCTGTGGTCG CGTCCATGGA TTGCCCTGGC GCCAGCAAGT ACGTGCCTAG AATCCGTGCC
CAGAAGAACC GCTGTGAGGA GATCGTGGAG CTC------- ----GGTCAG ATGTGCAAAG
AGCTCATCCA AGTCTACGAG AAGAAGAACG GTCAAGCCAC AGAAGATCAT TTACTTCCGC
GATGGCGTGA GCGATAATCA GTTCGAGATG GTCCTGAAAC AGGAGCTGAA GCAGCTGGAG
AACATCTGAA GGCGCTAAGG AGGGTACTCG CCGACAATCA CAGCGATCGT GGCCAAGAAG
CGGCACCACA CACGGCTGTT CCCAGGACGA AGA------A ACGTGCTCCC TGGCACGGTG
GTCGACACCG ATGTGGTCAA CACGGCAGAC CAAGACTTCT TCCTGTGCAG CCACGACGGG
CTGCACGGGA CGAGCCGGCC AACGCACTAC CACAGGCTCA AGGACGACCA CGGCTTCGAG
CCCGTCGACC TGCAGAAGCT GGTGTACAAC ATGTGCTTCC TGTTCGCGCG CTGCACCAAG
CCGGTGTCGC TCACGACGCC CGTCAAGTAC GCCGACCTTG CGGCGTACCG CGGCAGGGAC
TACTACGAC
>LOC_Os02g07310
CCCAATTTGC TGGTTCATTT TACTCTAACT TAGGCAATTG GTCTTGGAAA GCTGGCGTGG
TTTTCACCAA AGAATACAAG CTACTCAGAT GGGCTTCAAC TGAATATCGA CTGTCATCAT
CAGTGTTCAT CAAGCC---- -GTTAACTTT ---------- ---------- ----------
---------- ---------- ---------- ---------- ---------- ----------
---------- ---------- ---------- ---------- ---------- ----------
---------- ---AGTATTC AGTACCCCTT TGCCTTGCTT GAAAGTGGCT CATTTTGGGA
GACAATATTT TGCCATTGGA GGTTGTAAAA TTGCTGAGGG ACAATGTCAC CAGAAACTCA
ATGCAAAACA TATGGCTGCT CTTCTTCACG ACACTGCTGA TTGTAATATT CCTAAAACAA
TGGATCT--- CTTTATGGAT GTCAAAAGAA TATGTGAGAC AGATATTGGA CTGATCTCTC
AATGTTGTCT TGCAAACATG TTCTTAAAAC AATGGTATCT TGCAAGCGTT GCCCTTAAAA
TCAATGCTAA GTGGGCGGAA GAAATACTGT ACTGATGCTT TAGAAATCTC CCATGTTAGG
GACCAACTAT TGTATTTGGT GCTCATGTCA CCCATCCCCA GGAAAAGTTC TTCCATTGCT
GCTGTTGTTG CTTCTCAAGA CTGGCCCGAG GTTACCAAGT ATGCTGGTTT AATCAGTGTG
CAAGCGTGCC ACCAGGAGTC AATACAAGGT CTTTTTAAAC T---AGCGGA ATGATCAAAG
AGCATCTCAT GTCTTTCTAT CGAGCCACTA AGGAAGCCCG GAAGGATTAT ATTTTACAGG
GATGGTGTGA GCAAAGGACA GCTCCCTCAG GCTTTGATGC ACGAACTTGG TGCCATCAAA
ATGGCTGTGC ATCTATGGAC CCGATATAAT CCATTAGTTA CATACGTGGT GCTCCAGAAG
TGTCGCCATA CACGTCTGTT TGCATAATGC GAAACTGCAA ACATACGGGC TGGTACTGTG
GTTGATTCAA ATATTTGCCA ACCAAATCAG TTTGATTTCT ACTTGTGTAG CCACCGCAGC
ACGCAGGGAA CTAAGCGGCC CAGGTATTAT CATGTTCTGT GGGACGAGAA CGACTTCTTG
GCTGGTTCTT TCCAAGAGCT CACAAACTAC CTCTGCTACA CCTCCGCAAC TTGCACCCAA
TCCATATCAG TTGTGGCCCC TGTGCACTAC GCTCGTCTTC TGTCATCACG AGCTCGATGT
TACATTAAA
>Mtruncatula_Medtr4g114860
TTTCTTTAGG ACGGTGTTTC TTTCCCAATC CT--GATCTT GGAATAATTG CAATTGGAGG
ATTTCAACAC AGTCTCAAGA CCACGGCTCA GGGTTAGCTT TGTGTCTTGA TATTCAGTTT
TGTCTTTTCG AAAGAATCAG TTTGGATTTC ATCATATTAG AAATATTTGT CGAGGAGGTA
CTCTTGGGAT TGAAAGTAAA TGTTACTCAC AGAAATATAC TATTGCTAAG CTAACAAGAT
ACTCGCCACA TACTTTCCCT ATTTTGGAAG AAGCACCTCT CTTCTTGCCT ACTTTAAAGA
TAAACATAAC TATGATATTC AACACAAATA TTCCTGCATT GGATTTTGGG GGAAAC--AA
GACTAATTTC TGCCTATGGA GCTTGCGTCT TGGTTGAGGG TCAGCGATTT CCCAAATATT
TGGACAAGAA TGCTGCCAAG AACTTGAATG CTTTTTCTCC TTTGTGTGAT -----GCCAA
CAAGGATCCA GGTTACAAGC CTCAAGTGGA TTGCTGAGAC CAAGGTTGGC ATAGTGACAC
AGTGCTGCTT ATCGGTAATG CTAATGAAAA GACAATATCT CACAAATCTT GCTTTAAAAA
TCAATGCAAA ATTGGAGGCA GTAACGTTGA GCTAATAGGC TCCCACA--- --TTGAGGAT

GACATGTTAT GTTTATAGGG GCTGATGTCA ATCATCCTCC CGGGAAGTCC ATCAATTGTT
GCAGTGGTTG CCACTACTAA CTGGCCAGCT GCAAATCGCT ATGCAGCACG TGTTTGCGCT
CAAGAGCATT GTACAGAGAA AATTTTGAAT TTT------- ----GGAGAG ATTTGCCTTG
ACCTTGTTAG ACATTATGAG AAGTTGAACA ATCAGGCCCC AAAAAATTGT TATCTTTCGT
GATGGTGTTA GTGAGAGCCA ATTTCACATG GTTCTTGGCG AGGAGTTAAA AGATTTGAAG
ACCGTTTTCA G-----CACT CAAATACTTT CCAACTATCA CTCTTATTGT AGCTCAAAAG
CGCCATCAAA CTCGATTGTT TCCCCGGTGT AAGAGTGGAA ATGTGTTCCC TGGAACAGTT
GTGGACACAA AGGTCGTACA TCCTTTTGAA TTTGACTTTT ACCTGTGTAG TCACTATGGA
AGCCTAGGTA CAAGCAAGCC CACTCACTAT CATGTCTTGT GGGATGAGCA CAGGTTTACT
TCTGATAATT TGCAGAAGCT CATATATGAT ATGTGCTTTA CCTTTGCAAG GTGCACTAAA
CCTGTATCTT TAGTCCCTCC AGTGTACTAT GCTGACCTTG CTGCTTACAG AGGAAGACTA
TACTATGAA
>Mguttatus_mgf009177m
TGTTGGTTCG CCAGTCGTTT TTCCACGACC CGAAGATGTT GGGGTCCTCG GCTGCCGAGG
CTTCCACTCG AGTTTTAGGA CAACCCAGAG TGGTTGTCTT TGAACATTGA TTATCTACAA
CAATGATAAT CCAGCCGATG TGCGAACTTC CTAACCAAAA GATTGAAGGC TAAACGAACG
CTGAAGAATT TGAGGATCAC CGTAAGTCCG ACGAGTTCAA AATAACTGGT CTAAGAGTCT
TGCAGAGAGC AACTTTCACA TTGAAGCAAC AGAAGTGACT GTGTACGACT ACTTTGTGAA
CCAGCGGAAC ATCGACTTGC GATTCTCACT TACCATGCAT TAATGTCGGA AAGCCAAACG
CCCGACTTAC TCCCAGTCGA GCTTGCTCTC TTGTGTCACT GCAACGCTAC ACGAAACTAT
CTACACTTCA AAGAGCTTCA TTGGTCGACG CCATTCCTAC TCTGTTTGCT CCCGACGTAA
AAACTGTGCA CTTTATGGCG TGGAAGCGCA AAAACTTGTC CGAATTCGGA GTTGTTACCC
AATGCCTGGC TCCCAGCGAG TTAACGA--- --CAGTATCT GACAAACCTT CTGCTGAAGA
TAAATGCAAA GTTGGTGGGT TGAACTCCGT GTTGAACTTT CGCCTACATT CCTGGTGTCA
AACCGACATT GATTCTTGGT ATGGACGTCT CTCATGGCCT GGACAATTCC ATCGATTGCT
GCGGTTGTTA GCTCTAGGCA ATGGCCTTCG GTTTCTCGTT ACAGAGCATG TGTTCGCACT
CAATCTCCAA AGATGGAAAT GATTGATTCC CTGTTCAAGA AGATGATGGA ATTATGAGGG
AGGCTCTGCT GGATTTCTAT GTTAGTTCTG GGAAAGCCCG ACCAAATCAT CATTTTCAGG
GATGGTGTGA GTGAATCTCA ATTCAATCAA GTCCTCAATA TTGAACTGAG CCAAATTATA
GAGGCTGCAA ATTCCTGACG AAAATGGAAT CCCAAGTTTG TCGTAATCAT TGCACAGAAG
AATCACCACA CTAAGTTTTT CCAATTCTCC CGA------A ATGTGCAGCC AGGGACTGTG
ATTGATAACA AAGTTTGTCA TCCGAGGAAC AACGACTTCT ACCTGTGTGC CCATGCGGGC
ATGATCGGAA CAACGAGGCC TACACATTAC CATGTTCTGC TCGACGAGAT GGGTTTTTCT
ACCGATGATT TACAGGAGCT TGTTCACTCA CTTTCTTACG TATACCAGCG CAGCACCACT
GCTATATCGA TTGTTGCTCC AATCTGCTAC GCGCACTTGG CAGCGACTCA GTTGGGGCAG
TGGATGAAG
>Bdistachyon_Bradi3g51080
CCCCAGTTGC CAGGTCATTT TATTCTAACT TAGGCAACTC GGCTTGGAAA GTTGGCGTGG
CTTTTACCAG AGCATACGGC CCACACAGAT GGGCTTTCAC TGAATATTGA TTGTCATCTA
CAGCATTCAT CGAGCCCCTG TATTGATTTT AACTTTTGAA GATCGAAGAT CAAGAAGGCT
CTACGAGGTG TAAAGGTTGA GGTCACACAT AGAAGTATCG TATATCTGGC CTTACAAGCA
ACAAGAGAGT TACTTTCCCT ATAGATAAAC TGTGAAGACA GTAGTGCGAT ACTTCCAGGA
GACATACGGT TTTAACATTC AGCATACCTT TGCCTTGCTT GCAAGTGGGC AATCCACAAG
GCCAAATTAC TTCCCATGGA GGTTGTAAGA TTATCGAGGG ACAGCGTTAC TCAAAACTGA
ATGAGAAGCA GATAACTGCT CTTCTTAATG CCACTGCTTA TTGTAATTTT CCTGAAATAA
TGGTTCT--- CTTTATGGAT CTCAAAAGAA TATGTGAGAC AGATCTTGGA TTGGTCTCTC
AATGCTGTCT CACAAACATG TTTTTAAAAC CACAGTATCT TGCAAATGTC GCCCTTAAAA
TCAATGTTAA GTGGGAGGAA GAAATACTGT ACTAATGCTT TGTCAAGATT CCTTGTCAGT
GACCAACCAT TATATTCGGT GCAGATGTTA CCCATCCCCT GGAGAAGCCC TTCCATTGCA
GCTGTTGTTG CTTCTCAAGA TTGGCCTGAG ATCACCAAGT ATGCTGGATT AGTGAGTGCA
CAAACCCGTC GCCAGGAGTT GATACAAGAT CTTTTTAAAT AGCTGGTGGA ATGGTTAGAG
AACTTCTCAT TTCTTTTAAG AGATCAACTG GAGAAACCCC AGAGGATCAT ATTCTATAGA
GATGGTGTTA GTGAAGGGCA GTTCTATCAG GTTCTGTTGT ATGAGCTTGA TGCCATTAGA
AAGGCTGTGC ATCATTGAGC CCAATATCAG CCACCTGTTA CCTTTGTGGT GGTCCAGAAG
CGTCATCATA CACGGCTCTT TGCACCACAA TGAAGTGGAA ACATATTACC TGGAACTGTG
GTTGATTCAA AGATATGTCA TCCTACCGAG TTTGATTTCT ACCTTTGTAG CCATGCTGGC

ATTCAGGGAA CAAGCCGCCC TGCGCATTAC CATGTCCTGT GGGATGAAAA CAAATTTACT
GCCGACGGTT TGCAGACTCT CACGAACAAC TTGTGCTACA CCTATGCAAG ATGCACTCGT
TCTGTATCAA TTGTGCCTCC TGCATACTAT GCTCATCTTG CGGCTTTTCG AGCTCGGTTC
TACTTGGAA
>GRMZM2G007791_T01 cds:_protein_coding
TTATCCTTGG TAGAGGATTT TACTCAAGCA GCAGGACATT GGTGCTGTAG CTATGAAAGG
AACCCAGCAG TCCCTTAAAT CCACTCAGCA AGGTTGATCC TGTGTGTTGA CATTCTGTCA
TGCCGTTTTA CAAAGC---G TATGGATCTT AATTAGTGAG --------CT GGTTGATGAG
CTTAAAGGCC GACGTGTAAC TGTGATTCAT CGAAGTACAC AGTGCAAGGT TTGACTACCT
GCCAGCCAGA TACCTTTGTG GATGCTGACA AACACGGAGG CTCGTGGATT ATTATGCTCA
GAAACATGGC AAGGTGATTG AGTATCATGC TGCCATGCTT GGATTTGAGC AAGAGCAAAA
ACCGAATCAT TTCCAATTGA GCTTGCACTC TTCTTGAAGG ACAGAGGTTT CCAAAAAACT
TGAATCAGAA TTCTGAGAGG ATACTAAAGC TCTCTCCTTT TCTGCCCGAT -----TCCGA
GCAGCATTCG GGGTACAACA CTGAAGCTGA TTTGTGACAC ACAGCTGGGG ATCCTGACCC
AGTGTTTACT GAGGACCGCG CAAACAACAG GACAGTACAT GACGAATCTT GCTCTAAAGA
TCAACGGCAA GTTGGGGGCA GCAACGTTCA GCTGACTCGC TCCCACG--- --TCGGTGGT
GGCCTTTCAT GTTCATCGGT GCTGACGTTA ACCACCCCCC GGTAAAGCCC ATCGATCGCA
GCCGTGGTCG CCTCTGTCAA C---TCTGGT GTCAACAAGT ATGTGACCAG AATCCGTGCC
CAGCCGCACC GCTGCGAGGT GATCCAGCAG CTT------- ----GGTGAG ATCTGCCGGG
AGCTCATTGG AGTCTTTGAG AAGCAGAACC GTGAAGCCGC AGAAGATCAT CTACTTCCGT
GATGGCGTGA GCGACGGGCA GTTCGATATG GTCCTGAACG AGGAGCTGGC TGACCTGGAG
AAGGCATCAA G---GT---A ATGGTATGCG CCAACCATCA CCGTGGTCGT GGCCAAGAAG
CGGCACCACA CTCGGCTGTT CCCACGAACA GCAACCGGGA ACGTGCCGCC TGGCACGGTG
GTGGACACGG GCGTGGTGGA CCCGTCCGCG TACGACTTCT ACCTGTGCAG CCACACTGGG
ATTCTGGGGA CGAGCAGGCC GACGCACTAC TACACCCTGG TGGACGAGCA CGGCTTCGGC
TCCGACGACC TGCAGAAGCT GATCTACAAC CTGTGCTTCG TGTTCGCGCG GTGCACCAAG
CCGGTGTCGC TGGCGACGCC CGTCTACTAT GCCGACCTCG CGGCCTACCG TGGCAGGCTC
TACTACGAG
>Ptrichocarpa_POPTR_0014s15760
TTTTGGTTAG GCAGTCGTTC TTTCATGATT CAAGGATGTG GGTGTAACTG GTGTTAAGGG
TTTCCATTCT AGCTTCCGTA CCACTCAGGG TGGTTGTCTC TTAACATGGA TTGTCCACTA
CAATGATCCT AACTCCCCAG TATTGATTTT TTAATCAAAA GATTGAAGGC CAGAAGGATG
TTGAAAAATT TGAGGGTGAA GACGAAGCAT AAGAGTTTAA AATAATTGGT CTGAGAGCCA
TGCAATCAAC ATATTTTCCT ATGAAACTGT TGAAGTTACT GTATATGATT ATTTCACTAA
ACACTGTGGC ATACAACTTG GTTATTCACC TACCCTGCCT TGATGTTGGT AAACCAAACG
TCCAAACTAC TGCCACTGGA GCTTGTTCAC TTATTTCACT TCAACGGTAT AAAAAATTAT
CTTCAATGCA AAGAGCATCT TTGGTTGACG ACATTTATTC TTTGTGTATT GCAGAAGGAA
AAACTCAGAT ATTTATGGCG TGGAAGAAGA CAAGTCTCAG TGATTTTGGC ATTGTTACAC
AGTGCATATC CCCACGAAGA TTAATGA--- --CAGTATCT TACAAATGTG CTTCTTAAGA
TCAATTCTAA GTAGGAGGAA TAAATTCTCT GTTGAGCACT CCTCACAATT CCTGATAATG
GACCTACAAT GATTCTGGGC ATGGATGTCT CTCATGGCCA GGTCGATGCC ATCAGTGGCT
GCGGTTGTTG GATCTCGATG TTGGCCACTG ATTTCTAGGT ACAGAGCATC TGTAAGAACG
CAATCTCCTA AGGTGGAGAT GATTGATGCT TTGTACAAAA TGATGATGGT ATAATAAGGG
AACTGCTCGT GGATTTCTTT CAAACAAGCA AACAAACCAA AACAAATTAT TGTGTTCAGG
GACGGTGTCA GCGAGTCACA ATTCAATCAG GTGCTGAACA TTGAGCTGGA GCAAATTATC
AAGGCTATCA ACATCTGGTG AGGTGACATA CCGAAGTTCA CAGTAATTGT GGCTCAGAAG
AATCACCACA CAAAGCTTTT TCACTGGTGG TGGACTGAAA ATGTTCCTCC TGGGACAGTT
GTTGACACAA AGATTGTTCA TCCTAGAAAC TATGATTTCT ACATGTGTGC TCATGCAGGC
ATGATTGGAA CTTCAAGGCC AGCACACTAT CATGTCTTGC TCGATGAGAT TGGTTTCTCT
CCAGATGAAT TGCTAAATCT TGTCCACTCT CTTTCATATG TGTATCAAAG GAGTACCACT
GCTGTTTCGA TTGTGGCTCC CATATGTTAT GCTCACCTGG CTGCAGCACA GATAGGGCAG
TTTATGAAG
>Ptrichocarpa_POPTR_0006s02680
TTCTTGTTCG CCAGTCATTC TTTCATGATC CAAAGATCTG GGTGTCCTTG GATGCCGAGG
ATTTCATTCG AGCTTTAGAA CCTCGCAGGG TGGTTATCCC TAAATATAGA TGTTCGACTA
CAACGATAAT ACAGCCCCTC TATTGACTTT CCAACCAGAA GACTGAAGGC TAAGCGAACA

TTGAAAAATC TGAGGATAAG GGTGTCACCT ACGAGTACAG AATCACTGGC TTGAGATACT
TGCAAAGAGC AATGTTCTCT CTGAAATCGT TGACATTACA GTTTACCATT ATTTTGTAAA
TCATCGCAGC ATAGATTTAC GCTACTCATT TGCCTTGCAT CAATGTTGGC AAGCCTAAAG
GCCCACTTAC TTCCTGTCGA GCTTGTTCAC TGCTTCCCTT GCAACGCTAT ATAAAGCTAA
CTGTCCTTCA GAGATCACAG TTAGTAGAAG ACATTTCTCG TGTGTCTTCT CCTGAAGGAA
GAACTCTGAC ATATATGGCT TGGAAAAGAA AGAATCTTGC AGAATATGGA ATTTTCAATC
AATGCCTGGC ACCACTAGAG TTAATGA--- --CAGTATAT ACTTAATGTT CTCCTGAAGA
TAAATGCTAA GTTGGTGGTT TGAATTCTTT GTTGAGCAAT CACGAAAATC CCTCGTCTCG
AACCTACAAT AATATTTGGA ATGGATGTAT CACATGGCCT GGTCAATGCC CTCCATTGCT
GCGGTTGTCA GTTCTAGAAA CTGGCCTCTA CTTTCTCGTT ATAGAGCTTC TGTGCGTAGT
CAGTCACCAA AAGTTGAGAT GGTAGATTCT CTTTTTACGA TGATTCTGGG ATTGTCAGGG
AATTGTTGTT GGACTACTAT AGGAGTTCTG GCAAAACCAG CTCAGATAAT CATATTCAGG
GATGGAGTTA GCGAGTCACA GTTCAATCAA GTCCTCAACA TCGAGCTGGA TCAAATCATT
GAGGCTGCAA GTTCCTGATG AAAGTGGTCA CCCAAGTTCA CTGTTATTGT TGCACAGAAA
AATCATCACA CTAAATTTTT CCAGATCTCC AGA------A ATGTTCCTCC TGGAACCGTT
ATTGATAATG CTGTTTGTCA CCCACAAAGC TATGATTTCT ACATGTGTGC ACATGCAGGG
ATGATAGGAA CAACAAGGCC AACACATTAT CACGTTCTTT TAGATGAGAT TGGCTTTTCA
GCTGATGATC TACAGGAGTT GATTCACTCT TTGTCTTATG TGTACCAAAG AAGCACAACA
GCAATATCTG TAGTTGCTCC TGTCCGTTAT GCTCACTTGG CAGCAACTCA GATTTCACAA
TTCTTGAAG
>AGO1903_Aquilegia
---------- ---------- ---------- ---------- ---------- ----------
---------- ---------- ---------- ---------- ---------- ----------
---------- ---------- ---------- ---------- ---------- ----------
---------- ---------- ---------- ---------- ---------- ----------
---------- ---------- ---------- ---------- ---------- ----------
---------- ---------- ---------- ---------- ---------- ----------
---------- ---------- ---------- ---------- ---------- ----------
---------- ---------- ---------- ---TTTCTTC TTTGTCTTAT CCAGAAGGAA
TGGAACT--- ATATATGGCA TGGAAGAGGA AGTGTCTTGT GGAGTTTGGC ATCATCAATC
AATGCCTTGC ACCATGAGAG TTAATGA--- --CAACTTCT TGCAAATGTT CTCCTAAAGA
TTAATGCAAA GTTGGTGGTT TGAATTCTTT GTTGAACACT CTCGTAAATT CCTGATTTCC
AACCCACTAT GATACTTGGT ATGGATGTAT CACATGGCCA GGCCAATACC ATCTGTTGCT
GCGGTTGTCA GCTCAAGGCA GTGGCCCTCG ATTTCTCGCT ATCGAGCATC TGTGAGAACT
CAGTCACCGA AGCTTGAAAT GGTAGATTCT TTATTCAAGT TGATGAAGGC CTAGTTCGGG
AGCTATTGAT GGATTTCTAT ACTAGCTCAA AGGAAGCCTG ATCAGATAGT CATTTTCAGG
GATGGTGTAA GTGAGTCCCA GTTCAACCAG GTTTTGAACA TTGAATTGGA TCAAATCATT
GAGGCTGTAA GTTCCTGATG AGAATGGCGT CCAAAGTTCA CTCTGATTGT TGCACAAAAA
AACCACCACA CTAAGTTCTT CCAGGTCCCC TGA------A ATGTCCCACC TGGAACTGTG
ATTGATAGCC AGATTTGTCA TCCTAGACAC AATGATTTCT ATATGTGTGC TCATGCTGGA
ATGATTGGGA CAACTAGGCC TACACATTAT CATGTCCTTT TCGATGAAAT TGGCTTTACA
GCTGATGATC TTCAGGAGCT TGTCCATTCA TTATCTTATG TATACCAACG AAGCTCCACG
GCCATTTCTA CTGTTGCCCC TGTTTTCTAT GCTCACCTTG CAGCTGCTCA GATAGGAAAG
TTTATGAAG
>Mguttatus_mgf019782m
--GCTGTTGG AAGGTCCTTC TTTCAGGACT TCGGAAACTC GGCCTGGATT ACTGGAAAGG
ATTTTACCAG AGTCTTCGCC CAACTCAGAT GGGCTGTCAC TGAATATTGA TTGTCCGCTA
GAGCTTTTAT TGAACCTATG TTCTGAGTTT AGTACCTCAA GATCGAAGGT CAAAAGAGCT
CTTAAAGGTG TCAAGGTTGA GAGCATATAT CTCGTTTCAA TATCACAGGC GTGTCAACCC
ACTGAAAGCC TATGTTTACC ATCGATGCGC ACAAATATCT GTTTCTGAGT TCTTCCGTCA
GCAGCACAGT ATCGTGCTTA TGTATCCGTC TGCCTGCTAT TCAGTCCGGC ATTGGG--AG
ACCAATTTAT TTCCAATGGA GTTTGCAAAA TTGTTGAGGG TCAAAGATAC TCTAGGTTGA
ACGGGAGACA AGTTACTGCC CTGCTCCATG CGATTGCTTC TTATTATATT CCCGAGGCAA
CGTGACTGAT TCATACGGTA ATCAAGAGAG TGTGCGAAAC CGAGTTGGGC TTTGTGTCAC
AATGCTGTCA GCCCAACATG TTTTAAAAAT CACAGTACTT GGAGAATGTT TCTCTCAAGA
TCAACGTTAA GTTGGAGGGC GAAATACGGT GCTGAGAAGG CACTCTGATA CCTCGTTAGT

GACCTACCAT AATATTCGGT GCTGATGTCA CTCATCCCCA GGGGAAGTCC TTCTATAGCT
GCGGTGGTGG CCTCAATGGA TTGGCCAGAA GTCTCTAAGT ACAGAGGCTT GGTTTCTGCA
CAGGGCCATA GGGACGAGAT TATCCAGGAT CTCTACat-- ----TCAGGA ATGATTCGCG
AACACTTGGT TGCATTCTAC CAGAACACTA ATGAAGCCTA GCAGGCTCAT CTTTTACAGG
GATGGTGTGA GCGAAGGGCA GTTTAATCAA GTTCTTCTGT ATGAAGTTGA TGCAATTAGG
AAGGCTGTAA TGAACTCAGA CAGATATCAG CCAAGAATAA CTTTTGTTGT CGTTCAAAAG
AAGCATCACA CTCGTCTCTT TCCACCATAC AAGAGTGATA ACATTTTGCC AGGCACTGTG
GTGGATACCA AGATTTGCCA CCCCAACCAA TTGAGTTTCT ATCTTTGCAG CCATGCTGGG
ATCCAGGGAA CGAGCCATCC AGCACATTAC CATGTGTTGT TCGATGAAAA TAAATTCAGT
GCAGATAGTA TGCAAATGCT TACCTACAGC TTGTGTTACA CATATGCTAG GTGCACTCGA
TCAGTATCCA TAGTACCACC AGCTTACTAT GCACATCTTG CTGCATTTCG AGCACGGTAT
TACATCGAA
>AT5G21030.1
TCCTAGTTCG GCAATCTTTC TTCCATGACG CAAAAATATC GGTGTAGATT GTTGCAAAGG
ATTTCATTCA AGCTTCAGAA CTACTCAGGG AGGTTGTCCC TCAATATTGA TTTTCGACTG
CTATGATAGT AAAACCCCTG TGTTGATTTT CAAATCAAGG AACTGAAGGC TAAAAATACT
CTGAAAAATC TTAGAGTTAA AGTCCTCCCT TCGAATACAA GATAACCGGA CTAAGTACAC
TGCAAAGATC AACGTTTACT TGGAAGAAGT TGAGATTACA GTGTCCGATT ACTTCACTAG
GATCCGTGAA ATCGAACTGC GTTACTCGCT TACCTTGTAT CAATGTTGGT AAGCCAAACG
TCCTACCTAC TTCCCATTGA GCTTGTGAGC TTGTATCTCT ACAACGCTAT ACTAAACTAA
CCAAATTCCA GAGGAGTAAC CTTATCAAAG GCATTCCTTC TATGCATACT ---GAAAGAA
AAACTCTGAT GTTTATGA-- ------AAAt cttgttcaat g--------- tggaattgtG
AATGTATTGT TCCCAAAACT TAAATGA--- --CAGTATCT CACAAATCTT CTACTAAAGA
TAAATGCCAA GTTGGTGGAT TGAATTCAGT TTTGATATGG AGCTGTCATG CCTGGTAATG
AGCCTACCAT CATTATTGGA ATGGATGTAT CTCATGGCCT GGACAATACC ATCCATTGCC
GCGGTTGTGA GCTCCAGAGA GTGGCCACTG ATCTCAAAAT ACAGGGCTTG TGTGCGTACA
CAGTCGCCTA AAGTTGAAAT GATCGATAGC CTCTTTAAGA TGATCAAGGT ATCATGAGAG
AGCTCTTGCT TGACTTTCAC TCAAGTTCTG GAGAAACCGA ATCACATTAT CATTTTCAGG
GATGGTGTCA GTGAATCTCA GTTTAACCAA GTTCTTAATA TTGAACTGGA TCAGATGATG
CAAAT----- ---------- ---------- ---------- --------AT GATGCAAATA
AACCACCACA CGAAGTTCTT CCAAAAGCCC TAA------A ATGTTCTTCC AGGAACAATA
ATTGACAGCA ACATCTGTCA CCAACACAAC AACGATTTCT ATCTTTGTGC TCATGCTGGA
AAGATTGGAA CTACAAGGCC AACACATTAC CATGTGCTCT ACGACGAGAT TGGATTTGAC
ACAGATCAAC TCCAAGAACT TGTGCATTCA CTATCCTATG TCTACCAGCG GAGCACAACT
GCCATCTCTC TTGTTGCGCC GATATGTTAT GCTCATTTGG CGGCTGCACA GATGGCAACT
GCAATGAAG
>Alyrata_16055324_locus
TTTTGGTCAG GCAGGCTTTT TTCCA-AGCG ATGGGAAGTT GGTGTAAAAG GTATTCGAGG
TTTCCATTCG AGCTTTCGTC CAACCCATGG AGGCTCTCGC TTAACATTGA TTGTCAACAA
CAATAATCTT AGAACCCCAG TCTTGAATTC CCAATCAGAG GACTGAAGGC TGCGAAAATG
CTTAAACATA TGAGGGTAAA GGCAACACAC CGGAATTTAA AATTATAGGT CTAAGAACCG
TGCAATCAGC ACTGTTTTCG ATGAAGATAG AGATATTACA GTGTATGACT ATTTCAAGCA
AACTTACACA GAACCTATTT CTTCTGCACT TGCCATGCCT TGATGTTGGC AAGCCAAACG
CCCCAACTAT TTCCACTGGA GTTTGTAATC TTGTATCTCT GCAACGTTAT ACAAAATTGT
CCGGAAGGCA AAGAGCTCTA CTTGTTGAAG ACATTTATCC TCTGTGTCCT CCAGACGGAA
AACTTCTGAT ATCTATGGCC TGGAAGAAGA TATGCCTCAC TGAAGAAGGG ATCCACACAC
AATGCATCTG CCC------- --GTCAAAGT GACAATATCT CACCAATGTA CTTCTGAAGA
TTAATTCAAA GTTGGAGGCA TCAATTCTTT GTTGAGTACT CTTACAAATT CCTGATAAAC
AACCCACCTT GATTTTGGGT ATGGATGTAT CTCATGGCCA GGTCGGTTCC TTCAGTAGCA
GCGGTTGTTG GTTCAAAATG CTGGCCCTTA ATCTCAAGGT ATAGGGCAGC TGCAAGAACT
CAGTCACCAC GTCTGGAGAT GATTGATTCG CTCTTCCA-- -GATAACGGT ATTATGAACG
AATTATTTGT AGAGTTCTAC CGGACAAGCA GGGAAGCCTA AGCAGATTAT CATTTTCAGG
GATGGTGTGA GTGAATCACA GTTCAACCAA GTCTTGAACA TCGAGGTGGA CCAAATTATA
AAGGCTATCA ACGTCTGGTG AATCGATGTG CCAAAATTCA CTGTCATTGT GGCTCAGAAG
AACCACCACA CCAAGTTGTT TAAAGGGTCC TGA------A ATGTTCCTGC AGGAACTGTT
GTGGACACCA AGATCGTACA CCCGACAAAC TACGATTTTT ACATGTGTGC TCATGCAGGA

ATAATAGGAA CTTCAAGACC GGCTCATTAC CATGTATTAC TTGATGAGAT TGGTTTCTCC
CCCGATGACT TGCAGAATCT CATCCATTCT CTCTCCTATG TCAACCAACG TAGCACAACT
GCAACTTCGA TTGTGGCTCC AGTGCGATAC GCTCATCTTG CAGCAGCTCA ATTTGCTAAG
TTTGAA---
>GRMZM2G359875_T01 cds:_protein_coding
CTCCTGTCGG TAGGTCATTT TACTCCAACT TAGGAAACTT GGATTGGAAA GTTGGCGTGG
TTTTTACCAA AGCATAAGGC CGACACAGAT GGGCTTTCAC TGAATATTGA TTGTCCTCTA
CTGCATTTAT CGAGCCCCTG TATCGATTTT AGCTTCTTAA GATCGAAGAT TAAAAAAGCC
CTAAGAGGTG TGAAGGTTGA GGTGACTCAC AGAAATATCG CATTTCTGGC CTCACAAGCA
ACAAGAGAGC TTCATTCCCT GTTGATGAAC TGTGAAGACT GTGGTGCAAT ACTTCATGGA
GACTTATGGT TTTAGTATCC AGCACACCTT TACCATGCTT GCAAGTGGGT AATCAACAAG
ACCAAATTAT TGCCTATGGA GGTTGCAAGA TAGTTGAAGG ACAGCGTTAC TCAAAGCTCA
ATGAGAAACA AATCACTGCT CTACTGAATG CCACTGCTGA TCGTAATACT CCTGAAACAA
TGGTTCT--- CTTTATGGAT CTCAAAAGGA TCTGTGAGAC TGAACTCGGA TTGGTCTCCC
AGTGTTGTCT GACAAACATG TTTTTAAAGC AACAGTACCT TGCAAATGTT GCACTCAAAA
TAAATGTTAA GTTGGGGGAA GGAATACTGT ACTGATGCTT TGTCAAGATC CCTTGTCAGT
GACCGACCAT AATATTTGGT GCTGATGTTA CCCATCCCCT GGAGAAGTCC TTCCATTGCA
GCCGTTGTTG CTTCGCAAGA CTGGCCCGAG GTCACGAAAT ACGCTGGACT AGTGAGTGCG
CAAGCCCATC GCCAGGAGCT GATACAGGAT CTTTTCAAGT AACTGGCGGC ATGATAAAGG
AACTTCTCAT TTCTTTCAAG AGGGCAACTG GAGAAGCCCC AGAGGATCAT ATTCTACAGG
GATGGTGTCA GTGAGGGACA GTTCTATCAA GTATTGCTGT ACGAACTTGA TGCCATTAGA
AAGGCTGTGC GTCCCTGAGC CCAATACCAG CCTCCAGTTA CTTTTGTCGT GGTACAGAAG
CGCCATCACA CTAGGCTGTT TGCACCACAG TGAAGCGGAA ACATACTGCC TGGCACCGTG
GTCGATTCGA AGATTTGCCA TCCTACTGAG TTTGACTTCT ACCTGTGTAG CCATGCTGGC
ATTCAGGGAA CGAGCCGCCC TGCTCACTAC CATGTCCTGT GGGACGAGAA CAAGTTCACA
GCTGACGAGC TGCAGACCCT GACGAACAAC CTGTGCTACA CGTACGCTAG GTGCACCCGC
TCCGTGTCCA TCGTGCCCCC GGCGTACTAC GCTCATCTGG CAGCCTTCCG AGCTCGCTTC
TACATGGAG
>Alyrata_16051341_locus
TGACTGTTGG TAAAAGCTTT TTCACTGAGC CAGACGCTTC GGGGTTGTAG CTGCGAAAGG
GTATCGCCAC ACTCTAAAGC CCACAGCACA AGGTTGTCTT TGTGTTTGGA CACTCGGTTT
TGGCATTCCG CAATGCTCGG TATTGACTAC TGTACTTTGG AGGCGGATGT GGAAAAGGAA
CTGACTGGTT TGAAAGTCAC TGTTAACCAT CGAAACTCAC CATTGTAGGG TTGAGACAAC
ACAAAAGATA TACATTCGAT CTTATTGAAG GAAAGTATCC ATTGTTAAGT ATTTCATGGA
AAAGTATGGA AAAGACATCC GTTACAAATA TTCCTTGCCT AAGTTTGGGG AAAAAAGGCG
GCAAAATTAT TACCCATGGA GTTTGTAACT TGGTCGAGGG GCAGATTTAT CCAAAAAAAT
TGAAGGGTAA TTCAGCTTCC CGGTTAAATC CCTCTTGTTC TGTGTGCTAT TCTGG-----
-AAAGTCGAT GGCTACAACT CTGAAATGGC TAGCCGAGAC CAAACTTGGT CTGGTGACTC
AGTGTTTCTT GACGGTTCTG CGAATAGGGT GACAGTACTT GGCAAATCTC GCCCTCAAGA
TAAACGCAAA GTTGGTGGAA CGAACGTGGA GCTAATTATT TCTCTTT--- --TCAATAAA
GAGAGGTCAT GTTCATTGGT GCTGATGTCA ATCATCCGCT CACGAAGTCC ATCCATTGTT
GCTGTTGTAG GCACTCTTAA CTGGCCTGAA GCTAACCGCT ACGCAGCTAG AGTCAAAGCT
CAGACTCACC GTAAAGAAGA GATACAAGGG TTT------- ----GGTGAA ACTTGCTTAG
AGCTTGTCAA TGCTCATTCT AATGCCACCA AAACGACCTA ACAAGATTGT GATATTCCGT
GATGGTGTCA GTGATGGTCA GTTCGATATG GTTCTCAATG TGGAGTTACA GAATGTTAAG
GACACTTTAA AAAGATGAA- ----TATAAT CCGTTGATAA CGGTAATTGT GGCACAGAAG
CGTCATCAAA CCCGTTTCTT CCCCAAGCAA TGAAAGGACA ATGTGCTTTC AGGTACGGTC
GTTGATACAA AGATCATTCA CCCATTTGAG TATGATTTCT ATCTCTGTAG TCACCATGGA
GCGATTGGGA CAAGCAAACC AACTCATTAC TATGTTCTGT ACGACGAAAT CGGGTTCAAG
TCGGATCAGA TTCAGAAGCT CATATTTGAC GTGTGCTTCA CGTTTACTCG CTGCACCAAA
CCTGTCGCTC TGGTTCCTCC AGTTTCTTAT GCTGACAAGG CTGCTTCTAG AGGAAGGTTG
TACTACGAG
>GRMZM2G165242_T02 cds:_protein_coding
TATTAGTAAG GCAATCGTTC TTCCATAATC CTTCGACCTG GGTGTAGTGG GATGTAGAGG
GTTCCATTCT AGTTTTCGTG CAACCCAGAG TGGCTTTCAC TCAATATCGA TTGTCCACCA
CAATGATAGT GAAACCCCTG TATTGACTTT CCAATCAGAA GATTGAAGGC CAAGCGCTCA

CTGAAGAACT TAAGGATAAA AACAAGTCCG GCGAACAGAA GATTGTTGGT CTCAGGGCCT
TGCCGTGAGC ATTATTCACA CTGAAACAGA AGAGATCACT GTTTTGATT ACTTTGTAAA
GAACCGTGGC ATAAAGCTGG AATATTCATC TTCCTTGTAT CAATGTGGGA AAACCAAACG
TCCAACTTAT TTCCAGTTGA GTTTGCAGTC TTCTTCCTTT GCAACGGTAC ACTAAATTGA
GCACACTTCA AAGATCATCA CTCGTTGAAG GCATTTCTTT TGTGTGTTCT GCGGAAGAAA
GAATTCCGAT ATTTATGGCT TGGAAGAAGA AATGCCTTGC TGAATTTGGG ATCGTTACAC
AGTGTGTGGC ACC------- --ACTAGAAT GACAGTATCT TACAAATGTC TTGTTAAAGA
TTAACGCAAA GTGGGTGGCT TGAATTCGTT GCTGAAACAT CCCCAGCATT CCTCGTATCC
AACCAACTAT AATCTTGGGT ATGGACGTAT CACACGGCCT GGACAATACC GTCTGTTGCT
GCTGTTGTTA GTTCTCGTGA ATGGCCTCTT ATCTCAAAAT ACAGAGCATC TGTCCGCACC
CAATCACCAA AAATGGAAAT GATTGACTCG TTGTTTAAGA TGACGATGGT CTGATTCGGG
AGTGTCTGAT TGACTTCTAC ACCAGTTCAG GGAAAGCCCG ACCAAGTCAT CATCTTCAGG
GACGGTGTTA GTGAAAGTCA GTTTAATCAG GTGCTGAACA TTGAGTTGCA ACAAATCATT
GAGGCTGCAA ATTTCTGATG AGAATGGAAT CCCAAGTTCA CATTGATCAT TGCCCAGAAG
AATCACCACA CTAAATTTTT CATGAAAGCC AGA------A ATGTTCCAGC TGGCACTGTT
GTTGACAACA AAGTCTGTCA TCCAAGGAAC TTCGATTTCT ACATGTGTTC ACATGCTGGA
ATGATTGGAA CTACCAGGCC AACTCACTAT CACATCCTGC ATGACGAGAT AGGCTTCAAT
CCTGATGACC TGCAGGAGCT GGTGCACTCG CTCTCTTATG TGTACCAAAG GAGCACAACA
GCCATATCAG TTGGTAAGTT TATCTTGgca ccgtttttcg ttTATGTC-- ----------
----
>Mguttatus_mgf010559m
TTTTGGTTGG TCAATCATTC TTTCATGACT CGCGGATGTT GGGGTGACAG GTGTGAGAGG
TTTCCACTCC AGCTTTCGTC CAACTCTGAG TGGTTGTCTC TGAATATGGA TCATCGACAA
CCTTGATCTT GACGCCCCAG TGTGGACTTC TTAATCAGAA GACTGAAGGC CAAAAAGATG
CTCAAGAATA TGAGGGTTAA GGCGAGGCAC AAGAATTCAA AATTGCAGGT TTGAGAACCT
TGCAATCAGC ATTATTTTCG ATAAAAGTGT GGAGATTACT GTTTACGACT ATTTTGTTAA
ACACCGTAAC ATAGAACTTA TATCTTCATA TGCCATGCAT TGATGTTGGG AAACCGAAAG
ACCGATCTAT TGCCTATAGA GCTTGTTCTC TAGTCTCTCT TCAGAGATAC ACAAAACTGT
CGGTAACACA AAGAGCATCC TTGGTTGAAG GCATTTTTGC TTTGTGTTTT CCCGACGAAA
AAATTGTGAT CTTTATGGCT TGGAAGAGAA AATGTCTATG TAACTTGGGT ATCGTCACCC
AATGCGTCTC TCCATCAAAA TCAACGA--- --CAGTACCT GACAAATGTA CTTCTCAAAA
TGAATTCTAA GTAGGGGGGA TCAACTCATT GTTGAGAATT CCCGTCGATT CCTCATTACG
GACCGACCAT GATCTTGGGA ATGGATGTAT CGCATGGCCT GGTCGATCCC TTCAATTGCT
GCGGTTGTTG GATCTCGGAG TTGGCCATTG ATATCAAGGT ATAGAGCAGC TGTACGAACC
CAATCTTCAA GGGTGGAGAT GATTGAAAGT TTGTTCAAGA GGATGACGGC ATTATGAGGG
AACTGCTTAA GGATTTCTAC GAAACCAGTA AGCAAACCAA CTCAGATTAT TATTTTCAGG
GATGGTGTGA GTGAATCCCA GTTTACACAA GTCATCGACA TTGAACTCAA TCAAATTATC
AAGGCTATCA GCATCTGGCG AGACGAGATT CCGAAATTCA CAGTGATAGT GGCCCAGAAG
AATCACCATA CGAGACTTTT CCACAGCTGC TGA------A ATGTTCCACC CGGTACTGTT
GTGGACACAA ACATTGTTCA TCCTACAAAT TACGATTTCT ATATGTGCTC TCAGGCAGGG
AAAATAGGAA CTTCTCGACC TGCACATTAT CATGTATTGC TCGACGAGAT TGGTTTCTCC
CCAGACGATA TGCAGAATCT CATCCATTCC CTATCATATG TATATCAGAG GAGCACTACT
GCCATCTCCA TTGTTGCACC GGTATGTTAT GCTCATCTTG CAGCCCAACA AATGAGTCAG
TTTATAAAA
>GRMZM2G361518_T01 cds:_protein_coding
CACCGTTTGG GCGATCGTTT TTCTCTGACC TGGGTCCCTT GGAATAGAAA GCTGGCGTGG
GTTTTACCAG AGCATTCGCC CTACTCAAAT GGGTTGTCAT TGAATATTGA TTGTCTGCAA
CTGCTTTCTT TGAGCCCCTG TATAGATTTT AGCTTTTAAA GAACGAAGAT AAAGAAGGCC
TTAAGAGGCG TGAAGGTGGA AGTTACGCAC CGAAGTATCG AATAGCTGGT TTAACAGGAA
ACTCGGGAGC TACTTTTCCT GTTGATCAAC AGTGAAGTCA GTTGTACAGT ATTTTCAAGA
GACCTATGGC TTTGCCATCC AGCACACACC TGCCCTGTCT GCAGGTTGGC AACCAGCACA
CCCAAATTAC TTCCAATGGA GGTTGCAAGA TAGTGGAGGG ACAGAGGTAC TCTAAGTTAA
ACCAGAGTCA GATAAGAGCT CTTTTAGATG CCACTGCTCA TTGGAATACT CCTGAAACAA
CGGTTCT--- CTTTATGGAT CTGAAGCGTA TCTGTGAAAT CGACCTTGGA TTAGTTTCCC
AGTGCTGCTG CGCAAGCAAG TCTTTAAAAC AACAGATACT GGCAAACCTT GCGCTGAAGA
TAAATGTCAA GTCGGAGGAA GGAACACGGT GCTGATGCGG TGTCAAGATT CCTGGTGACT

GACCTACCAT CATATTCGGT GCCGATGTGA CCCATCCCCT GGAGAAGCCC TTCCATTGCT
GCTGTTGTGG CTTCCCAAGA CTGGCCTGAG GTGACAAAGT ATGCTGGTCT AGTTTCTGCT
CAGTCTCATA GGCAAGAGCT GATAGAGGAT CTCTACAAGT TTGTGGTGGC ATGGTCAGGG
AGCTTCTTAT ATCCTTCAAA AAATCAACTG GAAAAGCCTC AGAGGATACT ATTCTACAGG
GATGGTGTGA GTGAAGGGCA GTTCTACCAA GTTCTACTGC ATGAACTGGA TGCTATCCGA
AAGGCTGTGC ATCGCTGAAG CAAATACCAA CCGCAGGTGA CTTTCATCGT CGTCCAGAAA
CGCCACCATA CCAGGCTGTT CGCACCACAA CGAAGCGGCA ACATACTTCC TGGAACTGTC
GTGGACTCGA AGATCTGCCA CCCTACAGAG TTCGACTTCT TCCTGTGCAG CCATGCTGGC
ATCAAGGGCA CCAGCCGTCC TGCTCACTAC CATGTCTTGT GGGACGAGAA CAACTTCACA
GCCGACGCAC TGCAGACCCT CACCAACAAC CTTTGCTACA CCTACGCGAG GTGCACGCGC
TCTGTGTCCA TTGTCCCGCC GGCGTACTAC GCTCACCTGG CCGCATTCCG CGCCCGGTTC
TACATGGAG
>Bdistachyon_Bradi2g14150
TTTTAGTCCG CCAGTCATTT TTCCACAATC CTCGGACCTG GGTGTGTTGG GGTGCCGAGG
ATTTCACTCA AGTTTCCGTG GTACACAGAG TGGCTCTCCC TGAACATTGA TTTTCCACTA
CAATGATTGT TCAACCCCTG TATTGATTTC CTAACCAGAA GACTGAAAGC CAAGCGTGCT
CTTAAGAACT TGAGGATAAA AACAATTCCC GCGAATTCAA AATTGTTGGT TTGAGGAAAT
TGCAATGAAC AACGTTTGAA TGGAGGCAGT TGAAATAACA GTCTATGAGT ACTTCGTTAA
GATTAGAGGC ATAGAACTGC AGTATGGGTC TTCCCTGTAT CAATGTAGGG AGGCCAAACG
TCCAACGTAT TTCCTGCGGA GCTTGCATGC TTCTTCCACT GGAAAGATAC ACCAAACTGT
CTACTCTGCA AAGGTCCTCG CTTGTTGAAG ACAaatATAT ATTCTTTCCT CAACC-----
---------- ------GGCT TGGAAGAAGA AGTGTCTTGC TGATCTTGGT ATTGTTACAC
AATGTCTAGC TCCgcaAGAG TAAATGA--- --CAGTACAT AGATAATGTG CTGTTGAAGA
TAAATGCTAA GTTGGTGGGC TGAACTCATT GCTGAAGTAG AACGCACATA CCTTGTGTCA
AACCTACTAT CATCTTGGGC ATCGACGTGT CACATGGCCT GGGCAAGACC TTCCATTGCC
GCGGTGGTTA GCTCTCGAGA GTGGCCTTAC ATCTCTAAAT ATAGAGCAAC AGTGAACACT
CAGTCACCCA AACTAGAGAT GGTGTCCTCC TTGTTTAAGA TGATGATGGC CTCATTCGGG
TATCACTTAT TGACTTCTAC AACACTAGTG GGCAAACCAG ATCACGTTAT TATTTTCAGG
GATGGAGTTA GTGAAAGCCA ATTTACTCAG GTCATAAACA TCGAGCTTGA AAAGATCATT
GAGGCTGCAA GTTCCTGATG AGAATGGTCG CCTAAGTTCA CAGTCATTGT TGCTCAGAAG
AACCATCATA CCAAATTTTT CCAGATCCCC GGA------A ATGTTCCTCC CGGCACTGTG
GTGGACAAAC AAGTGTGTCA TCCCAAGAAT TTCGACTTCT ACATGTGTGC ACATGCTGGC
ATGATCGGAA CATCAAGGCC AACACATTAT CATGTTCTGC ACGATGAGAT CGGCTTCACA
GCTGACGAAC TTGAGGAATT TGTGCATTCA CTCTCCTATG TGTACCAGAG GAGCACAACG
GCGGTATCAG TCGTTGCTCC AATTTGCTAT GCGCATCTGG CGGCTGCCCA GGTCGGCACA
TTCCTGAAG
>LOC_Os03g33650
TCCTTGTGGG TCGGTCACTG TATGCCTCCA TGGGGACATT GGTGCTGTTG GATTAAGAGG
TTTCTTTCAG AGATTGAGGC CAACCAAGCA AGGCTTGCCC TTAATGTTGA TTCTCACTCT
CAGCTTTCCA CGAGAGAAGC GTGTGACTTC ACCTTCCACA CACAGGAGGT GGAGAAAGCA
TTGAAAAATA TCCGGGTATT TGTGTGCCAT CGAGGTACCA TGTGCATAGC TTGACAGACA
ACAGAGAACC TAAGTTTCGC GACCGAAGAA GGATCTTATG GTGGTGGATT ACTTCAAGGA
GCACTATAAC CATGATATAC AATTCAGACC TTCCATGCTT GCAGATTGGC AGGAGC--AA
GCCATGTTAT TGCCAATGGA GCTTGTGTAG TTTGTGAGGG CCAGAAGTTT CTTGGCCTGT
CTGATGAACA AACTTCTAAG ATTCTGAATG TGATTGCTAA TCTGTGTCAT -----GAGAG
GAGGCACCAA GGCTATGCAT CTGAAGCGAA TTGCAGAAAC ATCCATTGGT GTTGTGACAC
AATGTTGCCT TTATCCAACT TGAGCAAACC TCCAATTCTT GACGAATTTG GCTTTGAAGA
TCAATGCGAA ATCGGTGGCT GCAATATTGC CCTAGCAGCT TTCCATGATT CCTTTTGTCG
GACCGGTGAT GTTCATGGGT GCTGATGTCA CACACCCCCC CTTGAAGTCC ATCAGTGGTT
GCTGTAGTTG CAAGCATGAA TTGGCCGTCA GCAAATAAGT ACATCTCCAG GATGAGATCA
CAGACACACC GGAAAGAAAT CATTGAGCAA CTG------- ----GATGTT ATGGCTGGTG
AACTGCTTGA AGAGTTTCTA AAAGAAGTGG GAGCTCCCAA GCAGAATCAT ATTCTTCAGA
GATGGTGTGA GCGAGACACA GTTCTACAAG GTGCTGAAGG AGGAGATGCA TGCAGTGCGC
ACAACTGTTC GAGGTA---C CGGGTACAAA CCCTTGATCA CATTCATCGT AGTTCAGAAG
AGGCATCACA CTAGACTCTT CCAGGGAGAG GAAGATCAGA ACATACCACC AGGAACAGTT
GTGGACACTG TGATTACACA CCCAAGGGAA TTTGATTTCT ATCTGTGCAG CCACTGGGGC

ACCAAGGGGA CGAGCCGGCC AACTCATTAT CATGTTCTGT GGGATGAGAA TAACTTCCGT
TCCGACGAAG TGCAGCAGTT GATACACAAT CTTTGCTACA CATTTGCTCG GTGCACCAGG
CCAGTTTCTC TTGTCCCACC GGCTTACTAC GCACATCTCG CGGCATATAG AGGCAGGCTG
TACCTTGAG
>LOC_Os01g16870
TTTTGGTTCG CCAATCCTTT TTCCACAATC CTTCGACTTG GGTGTGATGG GCTGTAGGGG
ATTCCATTCA AGCTTCCGAG CTACACAGAG TGGCTTTCAC TCAATATCGA TTGTCCACAA
CGATGATTGT GAAACCCCTG TGTAGATTTT CCAACCAGAA GACTGAAGGC CAAGCGTGCT
CTGAAGAATT TAAGGATAAA AACAAGTCCA GCGAATACAA GATTGTTGGT TTGAGGAAAC
TGCTATGAAC AATGTTCACT TTGAAGCAGT GGAAGTATCT GTTTACGAGT ACTTTGTGAA
GAATCGGGGC ATAGAGTTGA GATACTCATT TTCCCTGTAT CAATGTGGGG AAACCAAACG
GCCAACTTAT TTCCAATTGA GCTTGCTCTC TTGTGCCTTT GCAAAGGTAT ACCAAGTTGA
GTACACTACA GAGGTCTTCG CTTGTTGAAG GCATTTCTGC TCTGTGTTCT GCTGAAGGAA
GAATTCAGAT ATTTATGGCC TGGAAGCGGA AATGTCTTGC TGAATTTGGG ATTATTACAC
AATGTGTGGC CCCACTAGGG TCAATGA--- --CAGTATAT TACAAATGTA CTGTTAAAGA
TAAATGCAAA GTTGGTGGCT TGAACTCCCT GCTGAAACAT CCCCTTCATT CCTTGTATCC
AACCGACAAT AATATTGGGA ATGGATGTTT CCCATGGCCT GGACAATACC TTCAATTGCT
GCGGTCGTTA GTTCTCGTGA ATGGCCTCTT GTATCAAAAT ACAGGGCTTC AGTCCGTTCT
CAGTCACCTA AGTTAGAAAT GATTGATGGA CTATTTAAGA AGATGATGGC CTCATTCGGG
AGTTATTAGT TGACTTCTAT ACCAGTACTG GGTAAGCCTG ATCAAGTCAT TATCTTCAGG
GATGGGGTCA GTGAGAGCCA GTTTACCCAG GTGCTGAACA TTGAGCTGGA TCAAATAATT
GAGGCTGCAA GTTTCTGACG AAAATGGTCT CCAAAGTTTA CACTGATTGT TGCACAGAAG
AATCATCATA CCAAATTTTT TGTGATCTCA AAA------A ACGTTCCTCC TGGTACTGTT
GTGGACAACG CAGTCTGTCA TCCAAGGAAC AATGACTTCT ACATGTGTGC GCACGCTGGA
ATGATTGGAA CTACAAGGCC TACACATTAT CATATCCTTC ATGATGAGAT AGGCTTCTCT
GCTGACGATC TTCAGGAGTT GGTCCACTCC CTTTCCTATG TCTACCAGAG GAGCACTACA
GCCATATCAG TCGTTGCACC CATTTGCTAC GCGCATCTCG CTGCTGCTCA GGTCAGCCAG
TTCATCAAG
>LOC_Os04g52550
TTGTCCTTGG TCAGGGATTT TAC--GCAGA GCGGgccatc AATATTGTAG CTCTCAAAGG
AACCCAGCAG AGCCTTAAAT GCACTCAGAA AGGCTGATCC TGTGTGTGGA CATTCGGTTT
TGCCATGTTG GAAAGCTCTG TTTGGACCTT CTATGaagTT GACCAAAATT GAACAATGCG
CTCAAAGGCC TGTGTGTTAC TGTAAGTCAC AGAAGTACAC TGTTAAAGGC TTGACAACCT
GCCGACCAGA TACTTTTAAA GACTCTAACA GACGACGAAG CTTATCGAGT ACTATAAGGA
GACGTATAAG AAAGAGATTG AGCATCCTGC TTCCATGCTT GGATTTGAGC AAGAGCAAAA
ACAAAACTAT TGCCGATTGA ATTTGTAATA TTCCTGAAGG GGAGAGGTAT CCAGTGTTAG
ATGACAAGAA GTCTGATAAT AAGGGCGACA AGACTCCTCT TCTGCCCGAT -----CTCAA
CCGGTGCCAT GGGTACAACC CTGAAGTTGA TGTGCGAGAC GGAGCTGGGG ATCCAGACCC
AGTGCTTCTT GAG---ACCG CCGCAAAAAA CGCAGTACAT TACCAACCTT GCTCTGAAGA
TCAACGGCAA GTCGGGGGTA GCAACATGCA GCTGACTCG- -------ATC CCTGGTGTCC
GCGATTTCAT GTTCATCGGT GCGGACGTGA ACCACCCCCT GGGAAatcCC GTCCATAGCA
GCCGTGGTGG CCTCCGTTGA T---AAAGGC GCCAGCAAGT ACGTGACAAG GATCCGCGCC
CAGTATCACC GGTGCGAGAT GATCCAGAAC CTC------- ----GGTGAT ATCTGCAAGG
AGCTCATCGG CGCGTATGAG AAGGTAAACA AAGAAGCCTG ATAGCATCAT CTACTTCCGC
GACGGCGTCA GTGACGGTCA GTTCGACATG GTGCTGAACG AGGAGCTGGC GGACATGGAG
AATAA----- ----ATATGG TGGGGACTAC CCGAAGATCA CCGTGATCGT TGCCAAGAAG
AGGCACCACA CGCGGCTGTT CCCACAGGAA CCAAACGGCA ACGTGCTCCC CGGCACGGTG
GTGGACACCG ACGTGGTCGA CCCGACGGCG TACGACTTCT ACCTGTGCAG CCACAAGGGG
GAGGTCGGGA CGAGCCGGCC GACGCACTAC TACAGCCTTC TGGACGAGCA CGGCTTCGCC
TCCGACGACC TGCAGAAGCT GGTGTACAAC CTCTGCTTCG TCTTCGCCCG CTGCACCAAG
CCGGTGTCGC TGGCCACTCC CGTCTACTAC GCCGACCTCG CCGCCTACCG CGGCAGGCTC
TACTACGAG
>AT2G32940.1
TTTTGGTCAG ACAGGCATTT TTCCACGATG GGCAAAAGTT GGTGTTATAG GTATTCGAGG
TTTACATTCG AGCTTTCGTC CAACTCATGG AGGCTCTCGC TTAACATTGA TTGTCAACAA
CAATGATCTT AGAACCCCAG TATTGAATTC CCAATCAGAG GACTGAAGGC TGCGAAAATG

CTTAAACATA TGAGGGTTAA GGCAACACAC CGGAATTTAA AATTATAGGT CTAAGAACCG
TGCAATCAGC ACTGTTTTCA ATGAAGATAG AGAGATTACT GTGTATGACT ATTTCAAGCA
AACCTACACA GAGCCTATTT CTTCTGCACT TTCCATGCCT TGATGTTGGC AAGCCAGACG
GCCCAACTAT TTCCACTGGA GTTTGTAATC TTGTATCTCT GCAACGTTAT ACAAAATTGT
CAGGGAGGCA AAGAGTTTTA CTTGTTGAAG ACATTTATCC TCTGTATTCT CCAGACGGAA
AACTTCTGAT ATCTATGGCC TGGAAGAAGA TATGCCTCAC TGAAGAAGGG ATCCACACAC
AATGCATCTG CCCATCAAGA TCAGTGA--- --CAATATCT CACCAATGTA CTTCTGAAGA
TAAATTCGAA GTTGGAGGCA TCAATTCTCT GTTGAGTACT CTTACAAATT CCTGATAAAC
AACCCACCTT GATTTTGGGT ATGGATGTAT CTCATGGCCA GGTCGGTTCC TTCAGTAGCA
GCGGTTGTTG GTTCAAAATG CTGGCCCTTA ATCTCAAGGT ATAGGGCAGC TGTAAGAACT
CAGTCACCAC GTCTGGAGAT GATTGATTCG CTCTTCCAGG AGATAACGGT ATCATGAACG
AATTATTTGT AGAGTTCTAT CGGACAAGCA GGGAAACCTA AGCAGATTAT CATTTTCAGG
GATGGTGTGA GTGAATCACA GTTCGAGCAA GTCTTGAAGA TCGAAGTGGA CCAAATTATA
AAGGCTATCA ACGTCTGGTG AATCGATGTG CCAAAATTCA CTGTCATTGT GGCTCAGAAG
AACCACCACA CCAAGTTGTT TCAAGGGTCC TGA------A ATGTTCCTGC AGGAACTGTC
GTGGACACCA AGATCGTACA CCCGACAAAC TACGATTTTT ACATGTGTGC TCATGCAGGA
AAAATAGGAA CTTCAAGACC GGCTCATTAC CATGTATTAC TTGATGAGAT TGGTTTCTCC
CCCGATGACT TGCAGAATCT CATCCATTCT CTCTCCTATG TCAACCAACG TAGCACAACT
GCAACTTCGA TCGTGGCTCC AGTACGATAC GCTCATCTTG CAGCAgctca agttGCGCAA
TTCACAAAG
>LOC_Os06g51310
CACCCTTTGG TCGATCCTTC TTCTCTGACC TGGGTCCCTC GGACTAGAAA CCTGGCGTGG
GTTTTATCAG AGCATTCGTC CTACTCAAAT GGGTTGTCAC TGAATATTGA TTGTCGGCAA
CTGCTTTCTT TGAGCCCCAG TATAGATTTT AGCTTTTAAA GAGCGAAGAT CAAGAAGGCC
TTAAGAGGAG TGAAGGTAGG AGTTACTCAC CGAAGTATCG GATATCTGGT TTGACAGGCA
ACTCGGGAAC TACTTTTCCT GTTGATCAAC AGTGAAATCT GTTGTACAAT ATTTTCAAGA
GACATATGGA TTTGCGATCC AGCATACATC TTCCTTGTCT GCAAGTTGGC AATCAGCACG
TCCAAATTAC TACCAATGGA GGTTGCAAAA TAGTGGAAGG ACAGAGGTAC TCCAAGCTGA
ACCAGAATCA GATAAGGGCT CTTTTAGATG TCATTACTTA TCGGACTGCT CCTGAAACAA
TGGTTCT--- CTTTATGGAT TTGAAGCGTA TATGCGAAAT TGACCTTGGA TTGGTTTCCC
AGTGCTGTTG CACAAGCAAG TGTTTAAAAC AACAAATCCT AGCAAATCTT GCTCTGAAGA
TAAATGTGAA GTTGGGGGAA GGAACACTGT ACTGATGCAG TGTCGAGATT CCTGGTAACT
GACCTACTAT TATATTCGGT GCTGATGTCA CCCATCCCCT GGCGAAGCCC ATCCATTGCT
GCTGTTGTGG CCTCCCAAGA TTGGCCTGAA GTGACAAAGT ATGCTGGTTT AGTTTCTGCT
CAATCTCACA GACAAGAGTT AATAGATGAT CTGTATAAAT CTGCGGTGGA ATGGTCAGGG
AACTTCTTAT ATCCTTCAAA AGATCAACTG GAAAAGCCTC AACGGATAAT ATTCTATAGG
GATGGTGTTA GTGAAGGGCA ATTTTACCAG GTTCTATTGC ATGAGCTTGA TGCAATCCGA
AAGGCTGTGC ATCACTGAAG CAAATACCAA CCGCAGGTGA CTTTCATTGT GGTTCAGAAG
CGCCACCACA CGAGATTATT TGCACCACAA TGAAGTGGGA ACATATTGCC TGGTACTGTT
GTTGACTCCA AGATTTGCCA TCCTACGGAG TTTGACTTCT TCTTGTGCAG CCATGCTGGA
ATCAAGGGCA CAAGCCGTCC CGCTCATTAC CACGTCCTGT GGGATGAAAA CAACTTCACA
GCTGATGCGT TGCAGACCCT CACCAACAAC CTCTGTTACA CTTATGCGAG GTGCACACGA
TCCGTATCTA TTGTTCCACC AGCATACTAT GCTCATCTGG CGGCGTTCAG AGCCCGTTTC
TACATGGAG
>Bdistachyon_Bradi1g29580
CATCATATGG TCGATCCTTC TTCTCTGACC TGGGTCCCTT GGAATAGAAA GCTGGCGTGG
GTTTTATCAG AGCATTCGTC CTACTCAGAT GGGTTATCAC TAAATATCGA CTGTCAGCGA
CATCTTTCTT CGAGCCCCTG TATTGATTTT AGCTTCTAAA GATCGAAGAT CAAGAAGGCC
TTAAGAGGAG TGAAGGTGGA AGTTACTCAT CGAAGTACCG TATATCTGGT TTAACAGGCA
ACTCGGGAGT TAGTTTTCCT GTTGATCAAT GGTGAAGTCT GTTGTACAAT ATTTTCAGGA
GACATATGGT TTTGCTATCC AACACACACC TTCCCTGTCT GCAAGTTGGT AATCAGCACG
TCCAAATTAT TCCCCATGGA GGTTGCAAAA TAGTGGAGGG ACAGAGATAT TCCAAGCTGA
ACCAGAGTCA GATAAGAGTT CTCTTAGATG TCATTGCTTA TTGGAATACT CCTGAAACAA
TGGCTCA--- CTTTATGGAC TTGAAGCGTG TCTGTGAAAT CGACCTTGGG CTAGTTTCCC
AGTGCTGTTT AACAAACAAG TGTTTAAAAC AACAGATCCT GGCAAATCTT GCGCTGAAGA
TAAATGTCAA GTTGGGGGAA GGAACACTGT ACTGATGCAT TGTCAAGATT CCTGGTTACT

GACCTACGAT TATATTTGGT GCCGATGTCA CCCATCCCCT GGTGAAGCCC TTCCATTGCT
GCAGTTGTGG CCTCCCAGGA TTGGCCTGAG GTGACAAAAT ATGCTGGCCT AGTTTCTGCT
CAATCTCACA GGCAAGAATT GATAGAGGAT CTATATAAAT CCATGGTGGC ATGATCAGGG
AGCTTCTTAT ATCCTTTAAA AGGTCAACTG GAAAAGCCTC AGCGAATTAT ATTCTATAGG
GATGGTGTCA GTGAAGGCCA GTTTTACCAA GTTCTACTGC ATGAGCTTGA TGCGATCCGA
AAGGCTGTGC ATCGCTGAAG CAAATACCAA CCGCAGGTTA CTTTCGTCGT GGTTCAGAAG
CGCCACCACA CCAGGTTATT TGCACCACAA TGAAGCGGCA ACATACTTCC TGGTACTGTT
GTTGACTCCA AGATCTGCCA TCCTACAGAG TTTGACTTCT TCCTGTGCAG CCATGCTGGC
ATTAAGGGCA CAAGCCGTCC TGCTCATTAC CATGTACTGT GGGATGAAAA CAACTTCACC
GCCGATGGGC TGCAGACCCT CACTAACAAC CTCTGCTACA CTTATGCAAG GTGCACGCGT
TCTGTATCTA TTGTTCCTCC TGCATATTAT GCTCATCTGG CTGCCTTCCG TGCCCGTTTC
TACATGGAG
>GRMZM2G354867_T01 cds:_protein_coding
TTATCCTTGG TAGAGGATTT TACTCAAGCA GCAGGACATT GGTGTTGTAG CTATGAAAGG
AACCCAGCAG ACACTTAAAT ACACTCAACA AGGTTGAACC TGTGTGTTGA TATTCAGTTA
TGCCATTTTA CAAAGCCCGG TATGGACCTT AAATAGTGGG -----AATCT GGTTGATGAG
CTTAAAGGCC GACGTGTAAC TGTGATTCAT CGAAGTACAC AGTGCAAGGC TTGACTACCT
GCCAGCCAGA TACCTTTGTG GATGCTGACA AACAAAGTGT CTTGTGGAGT ATTATGCTCA
GAAACATGGC ATTGTGATTG AGTATCATGC TGCCATGCTT GGATTTGAGC AAGAGCAAAA
ACCGAATCAT TCCCAATTGA GCTTGCACTC TTCTTGAAGG ACAGAGGTTT CCAAAAAACT
TGGATAAGAA TTCTGGCAGG ATACTAAAGC TCTCTCCTTT TCTGCCCGAT -----TCCGA
GCAGCATCCA GGGTACAACA CTGAAGCTGA TTTGCGATAC ACAGCTTGGG ATCATGACCC
AGTGTTTCCT GGGGACCGCG CAAACAACAG GACAGTACAT GACCAACCTT GCCCTCAAGA
TAAACGGCAA GTTGGGGGCA GCAACGTCCA GCTGACTCGC TCCCACG--- ----GTCGGT
GGCCTTTCAT GTTCATCGGT GCTGACGTCA ACCACCCCCG GGGAAAGCCC ATCGATTGCA
GCCGTGGTTG CGTCTATCAA C---TCCGGT GTCAGCAAGT ACGTGACAAG AATCCGTGCC
CAGCCGCACC GCTGTGAGGT GATCCAGCAG CTC------- ----GGCGAG ATCTGCCTGG
AGCTCATCGG AGTCTTCGAG AAGCGAAACC GTGAAGCCGC AGAAGATCAT CTACTTCCGC
GACGGCGTGA GCGACGGGCA GTTCGACATG GTCCTGAACG AGGAGCTGGC GGACCTGGAG
AAGGCATCAA G---GT---G GCGGTACGCG CCGACCGTCA CCGTGATCGT GGCCAAGAAG
CGGCACCACA CGCGCCTGTT CCCACCCCAG CCAAACGGGA ACGTGCCGCC CGGCACGGTG
GTGGACACGG GCGTGGTGGA CCCGTCCGCG TACGACTTCT ACCTGTGCAG CCACGCCGGG
ATCCTGGGCA CGAGCAGGCC GACGCACTAC TACAGCCTGG TGGACGAGCA CGGCTTCCGG
TCCGACGACC TGCAGAAGCT GGTCTACAAC CTCTGCTTCG TGTTCGCGCG GTGCACCAAG
CCCGTGTCGC TGGCGACGCC CGTCTACTAC GCCGACCTCG CGGCGTACCG TGGCAGGCTC
TACTACGAG
>Bdistachyon_Bradi5g18540
CTCCAGTTGG CCGATCATTT TATTCAAACT TAGGAAACTT GGTTTGGAAA GTTGGCGTGG
ATTTTACCAA AGTATAAGAC CTACACAGAT GGGCTATCGC TTAATATTGA TTGTCTTCTA
CTGCATTTAT TGAGCCCCTG TATTGAATTT AGCTTCTGTG GATCGAAGAT TAAAAAGGCG
TTGCGAGGTG TAAAGGTGGA GGTGACGCAT CGAAATATCG TATATCTGGT CTAACAAGCT
ACACGAGAGC TTCATTCCCT GTCGATGAAC TGTAAAAACC GTGGTGCAAT ACTTCCTGGA
GACATATGGT TTTAATATTC AGCACACCTC TGCCTTGCTT GCAAGTGGGC AATCAGCAAG
GCCGAATTAT TTCCTATGGA GGTTGTAAGA TTGTTGAAGG ACAACGTTAC TCGAAGCTGA
ATGAGAAACA GATAACTGCT CTACTGAATG CCACTGCTAA TTGTTATACT CCTGAAATAA
TGGTTCT--- CTTTATGGAT CTCAAAAGGA TTTGCGAGAC TGATCTGGGA TTGGTATCTC
AATGTTGTCT CACAAGCACG TCTTTAAAGC AACAGTATCT TGCCAATGTA GCACTTAAAA
TTAATGTTAA GTAGGAGGAA GGAATACTGT ACTGATGCTT TGACAAGATT CCTTGTTAGT
GACCAACCAT AATATTTGGT GCTGATGTTA CACATCCCCT GGTGAAGTCC TTCCATTGCC
GCAGTGGTTG CTTCCCAAGA CTGGCCTGAG GTCACCAAGT ATGCAGGATT AGTGAGCGCA
CAAGCCCATA GACAAGAGTT GATACAGGAT CTATTTAAGT GACTGGTGGC ATGATCAAGG
AGCTTCTCAT TTCTTTCAAG AGGGCAACTG GAAAAGCCAC AGCGAATCAT ATTTTACAGG
GATGGTGTCA GCGAAGGACA GTTCTATCAA GTTCTGTTGT ATGAGCTTGA TGCCATTAGA
AAGGCTGTGC ATCCTTGAGC CAAATATCAG CCTCCAGTTA CCTTCGTAGT TGTCCAGAAG
CGTCATCATA CCAGGCTTTT TGCACCACAA CGAAGCGGGA ATATACTGCC TGGCACTGTT
GTTGATTCCA AGATTTGCCA TCCGACAGAG TTTGATTTCT ACTTGTGTAG CCATGCTGGC

ATTCAGGGAA CAAGCCGCCC TGCTCATTAT CATGTTCTGT GGGATGAGAA CAAATTTACT
GCTGATGAGT TGCAAACTCT CACAAACAAC TTGTGCTACA CGTACGCAAG GTGCACCCGC
TCTGTATCAA TAGTGCCGCC GGCATATTAT GCACATTTGG CGGCCTTCCG AGCAAGATTT
TACATGGAG
>Ptrichocarpa_POPTR_0012s11920
TCTCTGTTGG TCGTGGCTTC CATTCTAGAG CTCATACCTT GGAATCATAG CATCTAAAGG
TTGTCAACAT AGCCTCAAGC CGACCTCCCA GGGCTAGCTT TATGCTTGGA TATTCTGTTC
TGTCATTTCA TGAGCCTCTG TATAGATTTC AACATATTTG AGATGGATGT GGAAATTGCA
TTGAAAGGAC TCAAAGTTAG AGTGACTCAC CGAAATATGT TATTGTTGGG TTGACATGAC
ACACGAGATA TACATTTTCT CAAGAAGACA GAATGTTAGG CTTGTTGATT ATTTCAGGCA
AAAATATGGC AGGGATATAG TGCATCAATA TCCCTTGCCT AGAAATGAAA AGCAAC--AT
GAGAAACTAT TACCGATGGA GTATGTGTCT TGGTTGAAGG GCAAGTATTT CCAAAACTGC
AGAGAGACGA AGCCCAGATG TTGAAGGACT AGCTTTCTTC TCTGTGTCAT -----TCAAA
GAAGGATCCT GGTTACAAAT CTCAAATGGA TTTGTGAAAC CAAAGTTGGT ATTGTGACAC
AATGCTGTCT GTCCGTTCTG CGAATAAAAT GACAGTACCT TGCCAATATT GGTCTCAAGA
TTAATGCTAA GTTGGAGGAA GCAATGCAGA GCTGACAGAC TTCCATA--- --TTGGGGAT
GACACATCAT GTTTATTGGG GCTGATGTCA ACCATCCGCT CGAAAAGTCC ATCCATTGCT
GCTGTTGTTG GTACTACAAA TTGGCCAGCT GCAAATCGCT ATGCAGCTCG AGTTCGTCCT
CAGGACCATC GTTGTGAGAA GATTCTGAAT TTT------- ----GGTGAT ATGTGTTTGG
AGCTTGTTGA ATTTTATTCT CGGCTCAATA ACAAAACCTG AGAAGATTGT GATATTCCGT
GATGGGGTCA GTGAGGGCCA GTTTGATATG GTTCTCAATG ACGAGTTAAT GGACATCAAG
AGGGCAGGTC AATCAT---- ----TACACC CCAACCATCA CACTCATTGT TGCCCAGAAG
CGGCACCAGA CTCGTCTTTT TCTAGGATGG GGGATAGGCA ATGTGTCTCC GGGTACAGTT
GTGGACACAA AAATTGTCCA TCCTTTCGAG TATGATTTTT ACCTCTGCAG CCACTACGGA
AGCCTTGGGA CAAGCAAGCC CACACATTAC CATGTTCTAT GGGATGAGCA TGGCCTCAGT
TCTGATCAGT TGCAGAAGCT CATATATGAC ATGTGCTTTA CATTTGCTCG TTGCACTAAA
CCTGTGTCGT TAGTTCCACC TGTCTACTAT GCTGACCTTG TTGCTTATAG AGGAAGGCTC
TACCATGAG
>Ptrichocarpa_POPTR_0008s01100
TGTTGGTCCG ACAGTCGTTT TTCCATAATC CAAGGAGTTG GGTGTCATGG GATGCCGTGG
TTTTCATTCA AGTTTTCGAG CTGCCCAGGA TGGCTATCCC TGAATATTGA TTATCAACCA
CCATGATAGT TAAACCCCAG TGTGGACTTC TGAATCAGAA GACTGAAGGC TAAGAGGATG
CTTAAAAATT TGAGGATTAA AACTAATCAC TCGAGTACAA AATCACTGGA TTGACAATCC
TGCAGAGAGC AACTTTCTCG CTAAACCAAT TGAGGTTACG GTTTATGATT ACTTTGTCAA
TCATCGCAAC ATGGGATTGC AGTATTCATT TCCCGTGCAT TAATGTTGGG AAACCAAACG
CCCATCGTAT TTCCTCTTGA GCTTGCAATC TGGTTTCGTT ACAACGCTAC ACCAAATTGT
CCAGCCTGCA AAGGGCTTCC CTTGTGGACG GCATTTCTGT TGTGTATTCT CCAGAAGGAA
GAACTCTGAC ATTTATGGCA TGGAAAAGGA AAAATCTTTC TGATTTAGGG ATCGTCACAC
AGTGCATTGC ACCACAAAGG TGAATGA--- --CAGTACCT GACAAATGTG CTACTGAAAA
TCAATGCCAA GTTGGTGGAA TGAATTCCTT GTTGAGCATG CTCCTTCATA CCTGGTGTCT
AACCCACCTT AATACTTGGG ATGGATGTAT CACATGGCCT GGTCAGTGCC ATCTATTGCT
GCGGTGGTAA GTTCCAGGCA CTGGCCATTA ATTTCTCGCT ACAGAGCTTC TGTTCGCACC
CAGTCACAGA AGGTTGAAAT GATTGCAAAT CTATTCAAGA GGATCAGGGC ATTATCAGGG
AGTCACTTCT GGACTTCTAC TCGAGCTCAG GGAAAGCCTG ATCAGATAAT TATTTTCAGG
GATGGAGTGA GCGAGTCACA GTTCATCCAA GTTCTAAACA TTGAACTTGA ACAAATTATT
GAGGCTGCAA GTTCTTGATG AGAATGGTGC CCGAAATTCA TGGTGATTGT TGCCCAGAAA
AATCACCATA CCAAGTTCTT CCAGATCACC TGA------A ATGTTCCACC TGGTACTGTC
ATTGACAACA AGGTTTGCCA TCCCAGAAAC AATGACTTCT ACATGTGTGC TCATGCTGGG
ATGATTGGAA CGACTCGGCC TACTCATTAC CATGTTCTAC ATGATGAGCT TGGTTTTTCT
GCAGATGATT TACAAGAGCT TGTGCACTCC TTGTCATATG TGTATCAGAG GAGCACCACT
GCCATATCTG TAGTTGCTCC AATTTGCTAT GCCCACTTAG CTGCAAGCCA GATGACTCAG
TTTATCAAA
>Gmax_Glyma16g34300
GTCCTGTTGG AAGATCATTT TATTCTGATT TGGGCCTTTA GGATTGGAAA GCTGGCGTGG
TTTCTACCAG AGTATTCGGC CTACACAGAT GGGCTATCAC TGAACATTGA TTGTCTTCCA
CTGCATTTAT TGAGCCCCAG TATTGATTTC AACTGCTGAA GATCGAAGAT CAAGAAAGCT

CTCCGGGGTA TCAAAGTTGA AGTGACACAT CGAAATATCG TATCTCTGGT CTGACAGGCA
ACCAGAGAAT TACATTCCCG GTAGATGAAC CATGAAATCT GTTGTGGAGT ACTTCTATGA
GACATATGGG TTTGTCATTC AACATACAGT GGCCTTGTCT GCAAGTTGGC AATGCACAAG
ACCAAACTAT TGCCAATGGA GGTTGCAAGA TAGTGGAGGG TCAAAGGTAC TCGAAACTTA
ATGAGAGGCA AATCACTAAT TTGCTGAGTG CCATTGCTCA TCGTTATCTT CCCGAAATAA
TGGATCT--- CTTTATGGAT CTCAAACGGA TATGTGAGAC TGACCTAGGA CTTGTTTCAC
AATGTTGCTT AACAAGCATG TCTTCAAAGC AACAGTACCT TGCAAATGTT GCTTTGAAAA
TTAATGTCAA ATTGGAGGGA GAAACACTGT ACTGATGCGC TCTCACGATT CCTGGTCAGT
GACCTACAAT TATTTTTGGA GCTGATGTGA CTCATCCCCT GGAGAAGCCC ATCAATTGCA
GCAGTTGTGG CTTCGCAAGA CTATCCTGAA ATTACAAAGT ATGCTGGTTT AGTTTGTGCC
CAAGTTCATA GGCAGGAACT CATTCAGGAT CTTTTCAAGT GACTGGTGGA ATGATCAAGG
AACTTCTTAT ATCTTTTAGG AGAGCTACAG GAAAAGCCAC AACGCATCAT ATTTTATAGG
GATGGTGTGA GTGAGGGACA GTTTTATCAG GTTCTACTGT TTGAGCTTGA TGCTATTCGA
AAGGCTGTGC ATCCCTGAAC CCAATATCAG CCTCCTGTGA CTTTTGTGGT GGTTCAAAAG
CGTCACCACA CAAGGCTCTT TGCACCATCA TGAAGTGGCA ACATATTGCC TGGCACTGTT
GTTGACTCCA AAATCTGCCA TCCCACCGAA TTTGACTTTT ATCTCTGCAG CCATGCTGGA
ATACAGGGTA CAAGCCGTCC TGCTCACTAC CATGTGTTGT GGGATGAAAA CAATTTTACT
GCTGATGCCT TGCAAACACT CACCAACAAT CTTTGCTACA CATATGCTCG GTGCACCCGG
TCTGTTTCAA TCGTGCCTCC TGCATACTAT GCTCACCTTG CTGCATTCCG CGCAAGGTTT
TACATGGAA
>Vvinifera_GSVIVT00023378001
TTCTTGTCCG TCAATCATTC TTTCATAACT CAAGGACTTG GGTGTCCTTG GATGCAGAGG
GTTTCATTCC AGTTTTCGAG CCACACAAGG TGGTTATCTC TAAATGTTGA TGGTCCACTA
CTACAATCAT ACAGCCCCGC TGTTGATTTT CAAACCAAAA GACTGAAGGC TAAACGGACT
CTCAAAAATT TAAGGATAAA GGTAAAACCT TCGAGTACAG AATTGTTGGA CTGAGGTCCT
TGCAAAGAGC AATGTTCACC CTAAAAAAAT AGAAGTGACG GTTTATGATT ATTTTGTTAA
CTATCGCCAG ATAGAGTTGC GTTATTCACT TACCTTGCAT CAATGTTGGG AAACCAAAAG
GCCTACGTAC TGCCTATTGA GCTTGTTTTT TGGTTTCTTT ACAACGCTAT ACGAAGTTAA
CTGTTCATCA AAGGTCCACC TTGGTAGAAG ACATTTATTG TCTGCCTTCT CCTGAAGGAA
GAACTCTGAC ATATATGGCA TGGAAAAGGA AGTGCCTTGC AGAATTTGGA ATTTTTAATC
AATGCCTTGC ACCACTAGAG TTAATGA--- --CAGTATAT CATGAATGTA CTTTTGAAGA
TCAATGCCAA ATTGGTGGTT TGAATTCTCT GTTGCCATTG AACCATCATA CCTGGTTTCC
AACCTACCAT AATCTTTGGA ATGGATGTGT CACATGGCCT GGCCAATTCC ATCTGTTGCT
GCTGTTGTGA GTTCTAGGTG TTGGCCACTG ATCTCTCGTT ACAGAGCTTC TGTTCGTACA
CAGTCACCGA AAGTTGAAAT GATAGATTCG CTTTTTAAAA AGATTTAGGC ATTGTCAGGG
AGCTGCTGTT GGACTTTTAT GTGAGTTCAG GCAAAGCCCA CTCAAATAAT CATTTTCAGG
GATGGAGTCA GTGAATCGCA GTTCAACCAA GTCTTGAACA TTGAGCTAGA TCAAATTATT
GAGGCTGCAA GTTCCTGATG AGAATGGACT CCTAAATTCA CAATAATTAT TGCCCAGAAA
AATCACCACA CAAAATTCTT CCAGGTCTCA AGA------A ATGTGCCTCC TGGAACTGTA
ATTGACAGCA AAGTCTGTCA TCCAACGCAC AACGATTTCT ACATGTGCGC ACATGCAGGG
ATGATAGGGA CAACAAGACC AACACATTAC CATGTTCTTC TAGACGAGAT AGGCTTTTCA
GCTGATGATA TGCAAGAACT CATCCATTCT CTGTCCTATG TGTATCAGAG AAGCACTACT
GCCATATCCA TCGTCGCCCC AGTTCGCTAT GCACACCTGG CGGCTACACA GGTTTCACAG
TTCATGAAG
>GRMZM2G411082_T01 cds:_protein_coding
CACCGTTTGG GCGATCGTTT TTCTCTGACC TGGGTCCCTT GGAATAGAAA GCTGGCGTGG
GTTTTACCAG AGCATTCGCC CTACTCAAAT GGGTTGTCAT TGAATATTGA TTGTCTGCAA
CTGCTTTCTT TGAGCCCCTG TATAGATTTT AGCTTTTAAA GAACGAAGAT AAAGAAGGCC
TTAAGAGGCG TGAAGGTGGA AGTTACGCAC CGAAGTATCG AATAGCTGGT TTAACAGGAA
ACTCGGGAGC TACTTTTCCT GTTGATCAAC AGTGAAGTCA GTTGTACAGT ATTTTCAAGA
GACCTATGGC TTTGCCATCC AGCACACACC TGCCCTGTCT GCAGGTTGGC AACCAGCACA
CCCAAATTAC TTCCAATGGA GGTTGCAAGA TAGTGGAGGG ACAGAGGTAC TCTAAGTTAA
ACCAGAGTCA GATAAGAGCT CTTTTAGATG CCACTGCTCA TTGGAATACT CCTGAAACAA
CGGTTCT--- CTTTATGGAT CTGAAGCGTA TCTGTGAAAT CGACCTTGGA TTAGTTTCCC
AGTGCTGCTG CGCAAGCAAG TCTTTAAAAC AACAGATACT GGCAAACCTT GCGCTGAAGA
TAAATGTCAA GTCGGAGGAA GGAACACGGT GCTGATGCGG TGTCAAGATT CCTGGTGACT

GACCTACCAT CATATTCGGT GCCGATGTGA CCCATCCCCT GGAGAAGCCC TTCCATTGCT
GCTGTTGTGG CTTCCCAAGA CTGGCCTGAG GTGACAAAGT ATGCTGGTCT AGTTTCTGCT
CAGTCTCATA GGCAAGAGCT GATAGAGGAT CTCTACAAGT TTGTGGTGGC ATGGTCAGGG
AGCTTCTTAT ATCCTTCAAA AAATCAACTG GAAAAGCCTC AGAGGATACT ATTCTACAGG
GATGGTGTGA GTGAAGGGCA GTTCTACCAA GTTCTACTGC ATGAACTGGA TGCTATCCGA
AAGGCTGTGC ATCGCTGAAG CAAATACCAA CCGCAGGTGA CTTTCATCGT CGTCCAGAAA
CGCCACCATA CCAGGCTGTT CGCACCACAA CGAAGCGGCA ACATACTTCC TGGAACTGTC
GTGGACTCGA AGATCTGCCA CCCTACAGAG TTCGACTTCT TCCTGTGCAG CCATGCTGGC
ATCAAGGGCA CCAGCCGTCC TGCTCACTAC CATGTCTTGT GGGACGAGAA CAACTTCACA
GCCGACGCAC TGCAGACCCT CACCAACAAC CTTTGCTACA CCTACGCGAG GTGCACGCGC
TCTGTGTCCA TTGTCCCGCC GGCGTACTAC GCTCACCTGG CCGCATTCCG CGCCCGGTTC
TACATGGAG
>Gmax_Glyma02g44260
TGCTTGTGAG GCAATCGTTC TTCCATGATC CCAAGATGTA GGTGTACTTG GATGCAGGGG
TTTCCATTCT AGCTTTAGAA CTACACAAAG TGGCTGTCTT TGAACATAGA TTCTCAACCA
CGATGATAAT TACCCCCCTG TGTGGACTTC CCAATCAAAA GACTGAAGGC CAAGAGGACA
TTAAAAAATC TGAGGATTAA AGCGAGCCCA TCGAATTCAA AATAACTGGG ATTAGTCCCT
TGCAAAGATC AACGTTTACC TTGAAGAGGA AGAAGTGACA GTATATGATT ATTTTGTTAA
TATCCGCAAG ATAGATCTTC GATATTCATC TCCCATGTAT TAACGTTGGA AAGCCAAAAG
GCCAACTTAT TCCCTCTTGA GCTTGCTCTT TGGTATCCCT GCAACGTTAT ACAAAACTAT
CCACTCTTCA AAGGGCTTCA TTGGTGGAAG GCATTTCTTC TTTGTCTGCT CCTGAAGGAA
AAATTCTGAT CTTTATGGCA TGGAAGAAGA AGAATCTTGC TGAGTTTGGA ATCGTGACTC
AGTGTATA-- -GC---CCGA CC---AGAAT GACAATATTT GACTAATGTT CTGTTGAAAA
TCAATGCTAA GTTGGTGGCC TGAACTCAAT ACTGAGCATT CTCCTTCATT CCTTGTTTCT
AGCCAACCAT TATTATTGGC ATGGATGTGT CTCATGGCCA GGGCAATTCC TTCAATTGCC
GCGGTGGTCA GCTCCCGAGA ATGGCCACTA ATATCAAAGT ATAGGGCTAG CGTCCGTACC
CAGTCTCCAA AGATGGAAAT GATTGATAAT TTGTTCAA-- ----GAAGGC ATAATGAGGG
AGCTTCTACT TGATTTCTAT ACAAGTTCTG GGAAAGCCCG ATAATATAAT CATATTCAGG
GACGGTGTTA GTGAGTCCCA GTTCAATCAA GTTTTGAACA TTGAACTTGA TCAAATTATC
GAGGCTGCAA GTTTTTGATG AAAATGGAAC CCCAAGTTTT TGGTGATTGT TGCTCAAAAG
AACCATCATA CTAAATTCTT TCA--GGAGC TCC------A ATGTTCCTCC TGGAACTGTA
ATTGATAACA AAATTTGCCA TCCTCGGAAT TATGATTTCT ATATGTGTGC ACATGCTGGA
ATGATTGGTA CTAGCAGGCC CACACACTAC CATGTTCTGT TAGACGAGAT TGGCTTTTCA
CCTGATGATC TACAAGAGCT TGTACATTCA TTATCATATG TGTATCAGAG GAGCACCACT
GCCATTTCTG TCGTTGCTCC AATTTGCTAT GCTCATCTGG CTGCTACTCA GATGGGTCAG
TTTATGAAA
>AGO1906_Aquilegia
---------- ---------- ---------- ---------- ---------- ----------
---------- ---------- ---------- ---------- ---------- ----------
---------- ---------- ---------- ---------- ---------- ----------
---------- ---------- ---------- ---------- ---------- ----------
---------- ---------- ---------- ---------- ---------- ----------
---------- ---------- ---------- ---------- ---------- ----------
---------- ---------- ---------- ---------- ---------- ----------
---------- ---------- ---------- ---TTACTAA TTGTTATTTT CCAGAAATAA
TGGTTCT--- CTTTATGGAT TTGAAACGAA TATGTGAGAC AGATCTTGGT CTTGTTTCAC
AGTGTTGTTT AACAAGCATG TTTTTAGAGT AACAGTATAT GGCAAATGTA GCTCTTAAGA
TTAATGTGAA GTTGGTGGAA GGAACACAGT GCTGATGCGT TGTCAAGATA CCTGGTTAGT
GACCTACTAT CATTTTTGGT GCTGATGTTA CTCATCCCCT GGAGAAGCCC GTCAATTGCA
GCTGTTGTCG CTTCTCAAGA TTGGCCAGAA GTTACTAAAT ATGCTGGTTT GGTATGTGCT
CAAGCCCATC GCCAAGAACT CATTCAAGAT CTTTACAAAT GCATGGGGGC ATGGTGAAGG
AACTTTTGAT ATCCTTCCGC AGAGCAACTG GAGAAGCCTG AGCGCATTAT CTTTTACAGG
GATGGGGTCA GCGAGGGACA GTTTTACCAG GTCTTGCTGT ATGAGCTGGA TGCAATTAGA
AAGGCTGTGC CTCCCTGAGC CGAATACCAG CCACCTGTTA CTTTTGTTGT GGTTCAGAAG
CGTCACCACA CACGACTGTT TGCACCATGC AGAAGTGGCA ACATTTTGCC AGGAACGGTG
GTAGACTCAA AGATCTGTCA TCCGACAGAG TTTGACTTTT ATCTTTGCAG TCACGCTGGG

ATACAGGGCA CTAGTCGTCC AGCCCATTAT CATGTTCTCT GGGATGAGAA CAAATTTACT
GCCGATGGAC TCCAGACTTT AACCAATAAT CTCTGTTACA CATATGCGCG ATGCACCCGC
TCAGTTTCCA TTGTACCTCC GGCATACTAT GCCCACTTGG CTGCCTTTCG TGCACGTTTT
TACATGGAG
>Ptrichocarpa_POPTR_0010s09150
GCCCTGTTGG AAGATCCTTT TTTTCCGATA TAAGCGACTA GGCTTGGAAT CCTGGTGCGG
ATTTTACCAG AGTATAAGAC CGACTCAAAT GGGCTGTCCT TAAACATTGA TTGGCCTCGG
CAGCATTCAT TGAGCCCCTG TATAGAGTTT AGCTTCTAGG GATCGAAGAT TAAAAAAGGT
CTAAGAGGAG TGAAAGTTGA AGTAACTCAC AGAAGTATCG TGTCTCGGGC TTGACAGCCT
ACAAGAGAAC TGTGTTTCCT GTTGATGAAC AATGAAGTCA GTTGTTGAAT ACTTCCAAGA
GATGTATGGC TTCACCATTC AACATGCATC TACCTTGCCT TCAGGTTGGA AACCAGAAAA
AGCAAATTAT TACCCATGGA GGCTGCAAAA TTGTGGAGGG GCAACGGTAT ACAAAATTGA
ATGAGAGGCA AATTACTGCC CTTCTAAATG TCACTTCTCT TAGCTATTCT CCTGAAACAA
TGGGTCC--- CTATATGGAT CTGAAGCGAA TATGCGAAAC TGATCTTGGT TTACTAACTC
AATGCTGTCT CTCAAACATG TGTTCAAAGT AACAGTACTT GGCAAATGTG TCCCTCAAGA
TCAATGTAAA GTGGGTGGTA GAAATACTGT CCTGATGCTA TCAGCTGATA CCTAGTTAGC
GACCAACCAT TATTTTTGGA GCAGATGTGA CTCACCCAAT GGAGAAGCCC CTCAATAGCT
GCTGTGGTGG CTTCTCAGGA CTGGCCTGAA GTAACAAAAT ATGCTGGACT AGTTTGTGCT
CAAGCTCACA GACAGGAACT CATACAGGAC TTGTACAAGT TAGCGGTGGC ATGATCAGAG
ATCTTCTGAT TTCTTTCAGG AAAGCAACTG GAAAAGCCGC TAAGGATCAT ATTTTACAGG
GATGGTGTTA GTGAAGGACA ATTTTATCAA GTTCTGCTTT ATGAGTTGGA TGCGATTCGG
AAGGCTGTGC GTCTTTGAGC CAAATATCAG CCACCAGTAA CTTTCATAGT TGTACAAAAA
CGTCACCACA CTAGATTGTT TGCACCATAG GGAAGTGGAA ACATACTGCC TGGCACTGTG
GTTGATTCTA AAATCTGTCA TCCAACAGAG TTTGACTTCT ATCTCTGTAG CCATGCTGGT
ATTCAGGGGA CAAGTAGGCC AGCGCACTAT CATGTTTTGT GGGATGAGAA CAACTTCACA
GCTGATGGAA TCCAGTCTTT GACGAACAAT CTCTGCTACA CATATGCTAG GTGCACTCGC
TCCGTTTCAG TAGTTCCTCC GGCATACTAT GCACATTTAG CTGCATTTCG TGCTCGATTT
TACACGGAG
>Mesculenta_cassava7327.valid.m1
TTTCTGTCGG CCGCAGCTTT CACTCAGCTA ATGAGACCTT GGCATCACAG CTTCCAGAGG
GTTTCAACAT AGCCTTAAGC CCACCTTCCA GGGCTAGCCA TGTGCTTGGA CATTCTGTCC
TGGCATTTCG CAAGCGCCAG TATAAATTTT AGCATATTCC AGCTTGATGT TGAAAATGCA
CTGAAGGGAC TGAAAGTTAC AGTGACTCAC CGAAATATAC TATAGCTGGA CTTACATAAT
GCACTGTATC TTCATTTTTG TCTGAAGAAA GAAAGTACAT CTTGTTGAAT ATTTCAGGCA
AAAATACCAG ---GATATAA AATTCAAACA TTCCTTGCCT TGATTTGGGC ACAAACAACG
AAAAAATTAT TACCCATGGA GTTTGTGTCC TAGTTGAGGG TCAGATTTAT CCAAAGCATT
TGGATAGAAA TGCAGCCCTC TTTTTGAATC GCTTTTATTC TTTGTGTGAT -----TCTAG
GAAGGATTCT GGTTACAAAT CTTAAGTGGA TTTCTGAGAC CAGAGTTGGT GTAGTGACTC
AATGTTGCTT GTCGATCTTG CTAACAAAAT GACAGTATCT TGCTAATCTT GCTCTGAAGA
TAAATGCAAA GTTGGAGGTA GCAATGTGGA GCTGATCGAC TTCCATT--- --TTGAAGGT
GACATGTTAT GTTTGTTGGG GCTGATGTTA ATCATCCTCC CGGAAAGTCC ATCTATAGCT
GCTGTTGTTG CCACTGTAAA TTGGCCTGCT GCAAACCGCT ATGCAGCTCG TGTTCGTCCC
CAGGAACATC GTAAGGAGAA GATTATCAAT TTT------- ----GGAGAT ATGTGTGTTG
AGCTTGTTGA AACTTATGTT CGGCTGAATA GTCAAACCAG GTAATATTGT AATATTCCGT
GACGGGGTAA GTGAGGGCCA GTTTGATATG GTTCTCAATG AAGAGTTAAT CGATCTCAAG
AGAGCTTTAG ATCAAT---- --AATATGCA CCAACTGTTA CACTTATTGT GGCCCAAAAG
CGGCACCAGA CTCGTTTGTT TCCCAGGCAG GAGAATGGGA ATGTGTCTCC TGGCACAGTT
GTGGACTCTA AAATTGTGCA TCCATTTGAG TATGATTTCT ATCTTTGTAG CCATTACGGA
AGCCTTGGGA CGAGCAAGCC CACACACTAT CATGTACTGT GGGATGAACA TGGTTTCAGT
TCTGATCAAT TGCAGAAGCT CATATATAAT ATGTGCTACA CATTTGCTCG ATGCACAAAA
CCTGTGTCCT TGGTTCCACC AGTGTACTAT GCTGACCTTG TTGCTTACAG GGGAAGGCTG
TATTATGAA
>Csativus_Cucsa.082260
TATCAGTTGG ACGATCCTTT TACTCTTGTA TTAACATGTT GGTCTGCAAG CATGGCGAGG
CTTCTATCAA AGCATCCGGC CTACTCAAAT GGGTTGTCTC TAAATATTGA CTGTCATCCA
CTGCATTCAT TGAAGCCCGG TATTGATTTT AACTCTTAAA GATCGAAGGT CAAAAAAGTA

CTTAGAGGTG TAAAAGTTGA AGTTACACAT AGAAATATCG GATTTCAGGG CTAACAGCCT
ACAAGAGAGC TATTTTCCCT CTTGATGAAA CATGAAATCT GTAGTTGAGT ACTTCCAAGA
GATGTATGGG TATACCATTC AGTATACATT TGCCTTGCCT TCAAGTAGGA AACCAGAAAA
GGTGAATTAT TACCCATGGA GGCTGTAAGA TACTCAAAGG ACAGAGATAC ACAAAACTTA
ACGAAAAGCA AATAACTTCC TTGTTGAATG CCATTGCTGA TAGCTATTCT CCTGAAACAA
TGGCTCA--- TTATATGGAT CTTAAAAGGA TCTGTGAAAC AGAATTGGGA TTGATATCCC
AGTGTTGTCT AACAAACATG TCTTCAAAGC AGCAGTACTT GGCGAACGTG TCTCTTAAGA
TCAATGTCAA GTGGGTGGAA GAAACACTGT TCTGATGCTT TACGGGCATT CCTAGTCAGT
GACCAACCAT TATTTTTGGA GCTGACGTCA CACATCCTCT GGAGACTTCC ATCAATAGCA
GCTGTTGTGG CTTCCCAAGA TTGGCCAGAA GTTACAAAAT ATGCTGGATT GGTATGTGCG
CAGCCGCACA GGGAGGAACT AATTCAAGAT TTATTCAAGT TGCCGGGGGT ATGATAAGGG
AGCTCTTGCT TTCATTTAAG AAGGCCACTG GAAAAACCAT TAAGGATAAT ATTCTATAGG
GATGGGGTCA GTGAGGGTCA GTTCTACCAG GTTCTACTGC ACGAACTCGA TGCCATACGC
AAGGCTGTGC TTCACTGAAC CCAGTACCAA CCTCCAGTAA CTTTTATCAT AGTCCAAAAG
CGACACCACA CCAGACTCTT TGCATCACAA CGAAGTGGTA ACATTTTACC AGGTACTGTC
GTGGATTCAA AAATATGTCA TCCAACTGAG TTCGACTTTT ATCTATGCAG TCACGCAGGA
ATCCAGGGAA CAAGTCGTCC TGCACATTAT CACGTTCTTT GGGATGAGAA CAATTTCAGT
GCGGATGAAA TTCAATCTCT GACTAATAAT CTCTGTTACA CGTATGCTCG GTGCACAAGA
TCGGTCTCAG TAGTGCCTCC AGCATACTAC GCTCATTTAG CAGCATACAG AGCTCGATTC
TACGTAGAA
>Gmax_Glyma20g02820
TTTCCTTGGG GCGGTGCTTC TTCCCCCCTC CTTTGATCTT GGCATAATTG CGATTGGAGG
GTTTCAGCAG AGTCTTAAGT CTACTTCTCA GGGTTGTCCT TGTGCCTGGA CATTCGGTTT
TGTCCTTTCG GAAGAATTGG TTTGGATTTT AGCATATTAG AGGAGCAAGT TGAGCATGTA
CTTATTGGGT TGAAGGTTAA TGTTAAACAC CGAAGTACAC TATTACTAGG TTGACAGGTT
ACGAGACATA TACATTCCCT ATTTTGGAAA GGAAGCTACT CTGGTTGGTT ACTTTCTAGA
GAAGTATGGT GTGAACATTG AATACAAACA TTCCTGCCTT GGATTTTGGA GGCAAC--AA
GACGAATTTT TGCCTATGGA GTTTGTGAGT TGGTTGAGGG GCAGAGATAT CCCAAATTGG
ACAAATATGC TGCCAAGGAC TTAAAAGAGT GCATTTCTTC TGTGTGTGAT -----TCCGA
CAAGCATCAA GGTTACAAGC CTCAAATGGA TTGCTGAGAC CAAGGTTGGC ATAGTGACAC
AATGCTGCTT GTCGGTATTG CTAATGAAAG GACAATATCT TACAAATCTT GCCCTCAAGA
TCAATGCCAA ATTGGAGGAA GTAATGTGGA GCTAATAGGC TACCACA--- --TTGAGGGT
GACATGTTAT GTTCATAGGG GCTGATGTCA ATCATCCTCC CGGGAAGTCC ATCAATTGCT
GCTGTAGTTG CCACTGTTAA TTGGCCTGCT GCAAATCGCT ATGCAGCACG TGTTTGTGCT
CAAGGTCATC GGGTTGAGAA AATTTTGAAT TTT------- ----GGGAGA ATTTGCTATG
AACTTGTTTC GTATTACGAT AGGCTGAACA ATCAGGCCTG AAAAAATTGT TGTCTTTCGT
GATGGCGTGA GCGAAAGTCA ATTCCATATG GTTCTCACAG AGGAGTTACA AGATTTGAAA
TCGGTTTTAG T-----GATG CAAATACTTC CCAACCATCA CTATTATTGT CGCACAAAAG
CGACATCAAA CTCGATTTTT TCCTGGGTCC AAAAATGGCA ATGTGTTTCC AGGTACAGTT
GTGGACACAA AAGTAGTACA TCCTTTTGAA TTTGACTTTT ACCTTTGTAG TCACTATGGA
AGCTTGGGTA CTAGTAAGCC CACTCACTAT CATGTCTTAT GGGATGAGCA TAAATTTAAC
TCTGATGATT TGCAGAAACT GATATATGAC ATGTGCTTTA CCTTTGCAAG GTGCACTAAA
CCTGTATCTT TAGTCCCTCC AGTGTACTAT GCTGATCTCA CTGCATATAG AGGACGGTTA
TACTATGAA
>Rcommunis_29828.t000011
TTCTTGTTCG TCAATCATTC TTTCATGATT CGAGGATCTG GGTGTACTCG GATGCAGGGG
ATTTCATTCA AGTTTAGAG TCTCACAAGG CGGTTATCAC TGAATATTGA TGGTCAACTA
CAACAATAAT ACAGCCCCAT TATTGACTTT CCAACCAACA GATTGAAGGC TAAACGTACC
CTGAAAAATT TGAGGATAAG GGTATCACCC ACGAGTACAG AATCACTGGC TTGAGATCTT
TGCAAGGACC AATATTCTCA ATGAAATCGT CGATATTACA GTTTATGAAT ATTTCGTTAA
TCATCGTAAT ATAGACTTGC GCTACTCATT TGCCTTGCAT TAATGTTGGA AGGCCTAACG
ACCTACTTTC TTCCTATTGA GCTTGTTCAT TGCTTCCGTT GCAACGCTAT ACAAAGTTAT
CTGTTATTCA GAGGTCAAAG TTGGTTGACG TCATTTCTTC TGTCTATTTT CCTGAAGGAA
GAATTCTGAC ATATATGGCA TGGAAAAGGA AGAATCTGGC AGAATTTGGA ATTTTCAATC
AATGCCTTTG TTCAATAGAC TCAGTGA--- --ATGTATGT TACGAATGTT CTCATGAAGA
TAAATGCCAA GTTGGTGGTT TAAATACTTT CTTGAACAAT CACGAAAGTC CCTCGTTTCA

AACCTACAAT AATTTTTGGT ATGGATGTTT CACATGGCCT GGACAGTGCC ATCTATTGCT
GCGGTGGTAA GTTCTAGGAA TTGGCCACTA CTATCTCGTT ATAGAGCTTC TGTTCATAGT
CAGTCACCAA AGGTTGAGAT GATTGATTCC CTTTTTAAGA TGATGATGGA ATAATCAGGG
AGTTGCTGCT GGACTTCTAT AGGAGTTCTG GCAAAGCCGG CTCAGATAAT CATATTCAGG
GATGGAGTCA GTGAGTCACA GTTTAATCAA GTCCTCAACA TCGAGTTAAA TCAAATCATT
GAGGCTGCAA GTTCCTGATG AAAGTGGTCG CCTAAGTTCA CTGTGATTGT AGCACAAAAG
AATCACCACA CTAAATTCTT CCA------- ---GCAGAAA ATGTCCCTCC AGGAACTGTT
GTAGATAATG GTGTTTGCCA CCCACAAAGC AATGATTTCT ACATGTGTGC CCACGCAGGG
ATGATTGGGA CAACCAGGCC AACACATTAC CATGTTCTGT TAGATGAAAT TGGCTTTTCA
GCTGATGATC TACAAGAACT CATTCATTCT CTATCCTATG TGTATCAGAG AAGCACAAGT
GCAGTATCTG TAGTTGCTCC TGTTCGTTAT GCTCATTTGG CAGCAACTCA AATAAGGCTA
TTCATGAAG
>Ppatens_1888444_locus
TGCTTGTGAG AGACAATTTC TTCCAGAGTT TGGGGATTTG GGTGTGGAAG GTTGGAGGGG
TTATCACTCC AGCGTGAGAC CGACGCTGAT GGGTTGATGT TAAATTTAGA TCCACTATGA
CGGTGGTACT GAAGCC---G TGATGAGTTT AAAGGTTCAA GATTGAAAGC GAAGGACATG
TTGAAAAACG TCAGAATAGA AACGACTCAC ACAAGTACAG AATATCGGGC TTCAGGGTCC
ATACGAGAAT TAAGTTTATG AAGGGGACGA GGAAATTTCA GTATATAATT ATTTTTTTGA
TACTTACTCG CGCAAACTAA AAAACCTATT TTCCAGCGCT GGATCTTGGA AACAGCAGAA
GCCAATATAC TGCCCATAGA ATTTGCAAGA TAGTTAGTGG ACAGCGCTAT ACCAAGCTGT
CCAGCAAGCA GAGGATGGCA CAAATCGGAA GCATTCATTC TTGCTATTCT GCAGA---AA
AGACAGTCCC ATTTATGTCT TTCAAGCGGT TGTGTGAAAT TAGACTTGGA ATAATCTCGC
AGTGCATGGT GAACGAACTC TGAACGA--- --CAGTTCCT AGGAAACCTT GCGTTGAAGA
TTAATTTGAA GTGGGAGGAT TGAACTCGCC ATTCGGATGT TACATTGCTT GG--------
--TCGACCAT AATCTTCGGC ATGGATGTGA CCCATGGCCT GGGGAATACC CTCAATTGCG
GCAGTAGTCG CAACGAAGAA TTGGCCCGAG GTATTCCATT ATTCGACTCA AGTTAAGGTC
CAACCAGCCA GGATGGAGAT GATTCAAGGG CTGTATGA-- ----GGTGGC ATGGTACGAG
AATTGCTCAT GTCGTTCTAT TCGACATGCG CCGAAGCCAT CCCAAATTAT AATTTACAGG
GATGGAGTTA GCGATAGCAT GTTTGCGAAA TGTTTGGAAG TTGAGTTCGT CGCATTTAAA
CGAGCTGTGC AGAACTGAAG CGGGTACAAT CCCGGAATAA CGTTCATTGT GGCTAAAAAG
CGTCACGGCA CGCGCTTCTT TCCAGAGTCG AGAAATGGGA ACGTTCTACC AGGAACTGTT
GTCGACAAGG ATGCGTGCCA CCCTCGGAAT TTTGACTTCT TCCTCATCTC TCAGGCCGGC
CTCATTGGCA CAGCTCGGCC TACTCACTAC ACAATACTGG TGAATGAAAA CCAGCTTGGG
CCTGATGATA TTCAGACCTT GACCAACAAA CTGTGCTACA CGTTTGGACG CTGTACCTCA
TCCATCTCGA TGGCGGCTCC AGCCGCATAT GCTCATATTC TGGCATCAAG ATATCGGAAG
TTGATGAGT
>Csativus_Cucsa.284770
TACCTGTGGG AAGATCTCTG TATTCTTCAA TGTGGAAATT GGAGTTGTTG GCTTAAGAGG
CTTTTTCCAA AGCCTTAGAC CCACACAACA AGGTTAGCTA TGAATGTAGA TGTTCTGTCA
CTGCTTTCCA TGAAAGAAAC GCTTGATTTT ATCTTTCTCA AAGAAGAAGT GGAGAAGGCT
TTAATGCATA TCAGAGTATT TGTTTGTCAC AGAGATATAG AGTTTATGGC TTGACAACCT
ACTGAGAGTC TTGGTTTGCT GATAGAAAAA GAATCTAAGA TTGGTGGGTT ACTTCAAGGA
TCATTATAAC TATGACATTC AATTCAGATT TGCCTTGTTT GCAGATTAGT AGGAGT--AA
GCCATGTTAT TTCCTATGGA GCTTGTATGA TTTGTGAAGG GCAAAAGTTT CTTGGGCTCA
CTGATGAACA GACTACAAGA ATGCTTAATG CCACTCTTGA TATGTGTGAT GAGAG-----
-AAACACAAA GGCTATGCAT TTGAAACGCA TTGCCGAGAC CAGTATCGGC GTTGTAAGTC
AATGTTGCCT TTACCTAATC TTGCCAAAGC TCCAATTTCT GGCTAATTTG GCTCTTAAGA
TTAATGCCAA GTTGGTGGTT GCACTGTTGC TTTAATTCGT TACATTCGTA CCTCCGAATC
GACCTGTGAT CTTCATGGGT GCAGATGTAA CTCATCCCCC TTGGAAGCCC CTCCATCGCT
GCTGTGGTTG GTAGCATGAA TTGGCCAGCA GCAAACAAGT ACGTCTCCAG AATGAGATCA
CAAACACACA GACAAGAAAT TATTGTGGAT CTT------- ----GGAACA ATGGTCGAAG
AGCTGCTTGA GGAATTCTAT CAAGAAGTGA AAATTGCCAT CACGAATCAT ATTCTTCAGA
GACGGTGTAA GTGAAACCCA GTTCTACAAG GTGCTTCAAG AGGAATTGCA AGCTATAAAA
ACAGCTGTTC TAGGTT---C TCAATATAAA CCTCCTATAA CTTTTGCTGT TGTTCAGAAG
AGACATCACA CAAGATTGTT CCCCTTCTAA TCAGATGAAA ATATTCCTCC CGGGACAGTC
GTTGATACCG TAATTACGCA CCCGAAGGAA TTCGATTTCT ATCTCTGCAG CCATTGGGGG

GTGAAGGGAA CAAGCAGGCC TACACATTAT CATATTCTGT GTGATGAGAA TCAATTCACT
TCTGATGAAC TACAAAAGCT AGTTTACAAC TTATGCTACA CATACAACAG GTGCACAAAG
CCTGTTTCAT TAGTACCTCC AGCTTATTAT GCTCATCTTG CTGCTTATAG AGGCAGACTT
TACCTTGAA
>Vvinifera_GSVIVT00016822001
TATCAGTTGG GAGATGCTTA TATTCTGATA TTAACAACTA GGTTTACAAT CATGGCAAGG
CTTCTATAAG AGTATAAGGC CAACTCAGAT GGGTTATCAT TGAACATCGA TTGTCATCAA
CTGCATTCAT TGAACCCCTG TATTGACTTT AACTTTTGGA GACCGAAGGT TAAGAAAGCT
CTTAGAGGTG TTAAAGTTGA AGTTACGCAC AGAAATATCG GATTTCAGGA CTGACAACCT
ACAAGGGAAC TATTTTCCCA GTTGATGAAA CATGAAATCA GTTGTTGAGT ACTTTCAGGA
GATGTATGGA TTTACCATTC GATATTCATC TACCTTGCCT CCAAGTAGGT AACCAGAGAA
AGTGAATTAC TACCAATGGA GGCTGTAAGA TTATTGGGGG ACAGAGATAT ACCAAACTGA
CTGACAAGCA GATAACTTCC TTGTTAAATG CCATTACTCA TTGCCATTCT CCAGAAACAA
TGGCTCT--- TTGTATGGAT TTGAAGCGGA TTTGTGACAC AGATCTGGGG TTGATTTCTC
AGTGCTGTCT TACAAAAATG TCTACAAAGC AACAGTACCT GGCAAACGTG TCACTTAAAA
TCAATGTTAA GTGGGTGGAA GAAATACTGT GCTGATGCTT TGAGTTCATT CCTGGTTAGT
GACCAACAAT CATATTTGGA GCCGATGTAA CTCATCCACT GGAGATGTCC ATCAATTGCT
GCTGTAGTAG CCTCCCAAGA CTGGCCAGAA GTCACCAAGT ATGCTGGATT GGTATGTGCT
CAGGCTCATC GGCAAGAACT TATTCAAGAT TTGTATAAGT TACTGGAGGC ATGATCAGAG
AGCTTTTACT TTCATTTAAG GCAGCCACTG GAAAAACCAT TGAGGATAAT ATTTTACAGG
GATGGTGTCA GTGAAGGGCA GTTCTACCAG GTTCTACTAT ATGAACTTGA TGCCATTCGT
AAGGCTGCGC ATCATTGAAC CTAGTACCAA CCTCCAGTGA CATTTGTTGT CGTCCAAAAA
CGGCACCACA CTAGACTCTT TGCACCACAA TGAAGTGGGA ACATCTTACC TGGTACTGTG
GTCGATTCGA AGATCTGCCA TCCCAGTGAG TTTGACTTTT ATCTATGCAG TCATGCAGGA
ATCCAGGGGA CCAGCCGACC CGCTCATTAT CATGTTCTCT GGGATGAGAA CAACTTTACA
GCAGATGAGA TCCAATCTTT GACGAACAAC CTCTGTTACA CGTATGCAAG GTGCACTCGG
TCTGTTTCTC TAGTTCCTCC TGCGTATTAT GCTCATCTGG CAGCCTACAG AGCTCGATTC
TACATGGAA
>Ptrichocarpa_POPTR_0010s17100
TACCTGTGGG AAGATCACTG TATTCTTCAA TGGGGAAATT GGAGCGGTTG CATTAAGAGG
GTTCTTTCAA AGTCTAAGGC CAACTCAACA AGGCTAGCTC TCAATGTGGA TTCTCTGTGA
CTGCTTTTCA TGAAAGAAGC GCTAGAATTT ACCTTCCTCA GAAGAGAAGT GGAGAAGGCC
TTAAAGAACA TCAGGATCTT TGTTTGTCAC AGAGATACCG GGTGTTTGGC CTAACAAGCT
ACAGAAAATC TTGGTTTTCT GACAGGGAAA AAATCTGAGG TTGCTGAATT ACTTCAAGGA
TCACTACAAC TACGATATAC AATTCAGACT TACCATGCTT GCAGATTAGT AGGAGC--AA
ACCATGTTAT TTCCTATGGA ACTTGTATGA TTTGTGAAGG CCAGAAGTTT CTCGGGCTAT
CTGATGATCA GACTGCAAGG ATACTTAATG CCACTGCTTA TATGCGTAAT GAGAA-----
-AAACACAAA GGGTACGCAT TTGAAGCGAA TAGCAGAGAC AAGTGTTGGT GTCGTAACCC
AATGCTGCTT GTATTAAATC TTGGCAAAGC TCCAATTTCT GGCTAATTTG GCTCTCAAGA
TCAATGCCAA ATTGGTGGGT GCACAGTTGC TTTAATTCGT TACCCTCATT CCTTCGTTCC
AACCTGTGAT CTTCATGGGA GCTGATGTGA CTCATCCCCG CTCGAAGTCC ATCTGTTGCT
GCTGTGGTTG GTAGCATGAA CTGGCCTGCA GCAAACAAGT ATGTCTCAAG AATGAGGTCG
CAAACACATC GACAAGAAAT CATCCAAGAC CTT------- ----GGTGAA ATGGTGAAAG
AATTACTAGA CGACTTTTAC CAAGAATTGA AAACTTCCCA AAAGAATTAT ATTCTTTAGG
GACGGGGTAA GCGAAACCCA ATTTTATAAG GTCCTTAAAG AGGAGTTGCA AGCCATTAGA
GAAGCTGTTC TAGATT---C CTGGTATAGA CCTCCCATTA CTTTTGCAGT AGTCCAGAAG
AGACATCACA CAAGGTTGTT TCCAAACTGA TCCGACGAAA ATATACCCCC TGGGACTGTC
GTGGACACTG TGATTACACA TCCAAGGGAA TTTGATTTCT ATCTATGCAG CCATTGGGGA
GTGAAAGGTA CAAGCAGGCC AACACATTAC CATGTCCTGT GGGATGAGAA CCAATTCACT
TCTGATGAAC TACAGAAGTT GGTTTACAAT CTGTGCTACA CATTTGTAAG GTGCACCAAG
CCAGTTTCTT TAGTGCCTCC AGCTTACTAT GCTCACTTGG CTGCATATAG AGGCAGACTT
TACCTTGAG
>contig9005_taeda
---------- ---------- ---------- ---------- ---------- ----------
---------- ---------- ---------- ---------- ---------- ----------
---------- ---------- ---------- ---------- ---------- ----------

---------- ---------- ---------- ---------- ---------- ----------
---------- ---------- ---------- ---------- ---------- ----------
---------- ---------- ---------- ---------- ---------- ----------
---------- ---------- ---------- ---------- ---------- ----------
---------- ---------- ---------- ---------- ---------- ----------
---------- ---------- ---------- --------AC AGACCTTGGT ATAGTTTCTC
AATGCTGTTT AGTAAGCATG TATTGAAAGC AACAATACAT GGCGAATGTA GCCTTAAAGA
TAAATGTTAA GCCGGAGGCA GGAACACTGT ACTGATGCTC TTAGAAGATA TCTGGTGAGT
GACCAACAAT AATATTTGGT GCAGATGTAA CTCATCCCCT GGAGAAGCCC ATCCATAGCA
GCCGTTGTGG CTTCTCAAGA TTGGCCAGAA GTCACAAAAT ATGTTGGTCT AGTTTCTGCT
CAGGCACATC GTCAAGAGAT GATTGAAGAC CTTTTTAAGT CGTTCGAAGC ATGATAAGAG
ATCTTTTGCG TTCCTTCTAC ACTGCAACAA GAGAAACCTA TGAGGATCAT CTTCTACAGA
GATGGTGTAA GTGAGGGGCA GTTTTACCAA GTCCTGCTGG ATGAGTTAGA GGCCATACGC
AGGGCTGCAT GTCAATGACA AGGATATCAG CCTCCTGTGA CTTTTATAGT TGTTCAAAAA
CGTCATCACA CACGTTTATT TGCATCATCG TGAAGTGGTA ACATATTGCC AGGTACCGTT
GTTGATTCCA CAATTTGTCA TCCAACAGAG TTTGATTTCT ACCTTTGCAG TCATGCTGGT
ATTCAGGGAA CAAGCAGGCC AGCTCATTAT CATGTTCTGT GGGATGATAA TAGATTCAGT
GCAGATGCTT TGCAGTCTTT GACAAATAAT CTCTGTTACA CATATGCTCG GTGTACTCGC
TCCGTTTCAA TCGTTCCTCC AGCATATTAT GCGCATCTGG CTGCATTTCG AGCTCGCTTC
TACATAGAG
>GRMZM2G079080_T02 cds:_protein_coding
TGTCCATAGG GCGGTCCTTC TACTCGGACA TCAGCGGCTC GGCCTGCAGT CGTGGCGTGG
GTTCTACCAG AGCATCCGGC CGACCCAGAT GGGTTGTCGC TTAACATCGA CTGTCGTCCA
CGGCATTTAT TGAACCCCGG TATCGAGTTC AGATATTAGG AACCGAAGAT CAAGAAGGCA
TTGCGGGGTG TAAAAGTTGA GGTCACTCAC CGAAGTATCG CATTTCAGGC CTCACAGCCA
ACTCATGAAT TATTTTCCCG ATTGATGAAA TATGAAATCT GTCGTGGAAT ACTTCAAGGA
AATGTATGGT TTCACCATTC AGCATCCATC TTCCCTGCCT TCAGGTTGGA AACCAAAAAA
GGCAAACTAT TACCCATGGA GGCTGCAAGA TCATTGAAGG CCAGAGATAC ACAAAGCTGA
ATGAAAAACA GATCACATCG CTGCTAAATG CCACTTCTTT TGGCTATCCT CCTGAAACAA
TGGGCAG--- TTATATGGAC ATCAAACGTA TTTGTGAAAC TGATTTGGGG TTGATATCAC
AATGTTGCTT AACAAGCATG TTTTTAAAGC AACAGTACTT GGCAAATGTC TCACTGAAAA
TTAATGTTAA GTGGGAGGAA GAAACACTGT GCTGACGCAA TAAGTTGATT CCTGGTCAGT
GACCAACTAT TATATTTGGT GCAGATGTAA CACATCCACC GGGGAAGTCC ATCGATTGCT
GCCGTTGTTG CTTCTCAAGA TTGGCCAGAA GTTACAAAGT ATGCTGGATT GGTTTGTGCT
CAGGCACACC GGCAAGAGCT CATTCAAGAC CTTTACAAGT AACAGGCGGC ATGATCAGGG
AGCTCTTAAT ATCCTTCAGG AAGGCCACTG GAGAAGCCAT TGAGAATAAT ATTCTACAGG
GACGGTGTTA GTGAAGGTCA GTTCTATCAA GTTCTCCTTT ACGAGTTAGA TGCCATCCGG
AAGGCTGTGC ATCCCTGAAC CAAATACCAG CCTCCTGTAA CATTTGTGGT GGTTCAAAAA
CGTCATCATA CAAGACTATT TGCATCACAA AGAAGTGGAA ATATTTTGCC AGGAACCGTT
GTTGATTCTA AGATATGCCA CCCAACGGAG TTTGATTTCT ACCTCTGTAG TCATGCTGGA
ATCCAGGGAA CGAGTAGGCC TGCTCACTAC CATGTCCTCT GGGATGAGAA CAATTTCACA
GCAGACGAAA TGCAGACATT GACAAACAAC CTTTGCTACA CTTATGCCCG GTGCACACGC
TCGGTTTCTG TTGTCCCTCC TGCATACTAC GCACACTTGG CAGCATTCCG GGCTCGGTTC
TACATGGAA
>Bdistachyon_Bradi2g10370
TTTTAGTCCG CCAATCCTTT TTCCACAATC CATCGATCTG GGTGTAATGG GCTGCAGGGG
ATTTCACTCA AGCTTCCGAG CCACAAAGAG CGGCTTTCTC TTAATATTGA TTATCTACAA
CAATGATTGT GAAACCGCTG TGTTGATTTT CCAACCAGAA GATTGAAGGC TAAGCGTGCA
CTGAAGAATC TAAGGATAAA AACAAGTCCA GCGAATTTAA GATTGTTGGA CTGAGGGAAC
TGTTATGAAC AATGTTTTCA CTGAAGCAAT AGAAATATCT GTTTATGATT ACTTTGTGAA
GAATCGGGGC ATAGAGCTAA GATATTCATT TCCCCTGTAT CAATGTGGGG AAACCAAACG
CCCAACATAT TTCCTATTGA GCTTGCTCGC TTGTGCCTTT GCAAAGATAT ACCAAGTTAA
GTACTCTCCA AAGGTCCTCT CTTGTCGAAG GCATTTTTGT TGTGTATCCT GCTGAAGGAA
GAATTCAGAT GTTTATGGCT TGGAAGCGAA AATGTCTAGC TGAATTTGGG ATTGTCACAC
AATGTGTGGC CCCACTAGGG TCAATGA--- --CAATATCT GACAAATGTC CTGCTAAAAA
TAAATGCAAA GTTGGTGGGA TGAATTCTTT GCTGAACTGT CCCCTGCATC CCTTGTATCA

AACCAACTAT GATTTTGGGC ATGGATGTGT CCCATGGCCT GGACAACACC ATCTATTGCT
GCAGTCGTTA GTTCTCGTGA ATGGCCTCTT GTCTCAAAGT ACAGGGCCTC AGTGCGTTCT
CAGTCACCAA AATCAGAAAT GATTGATTCA CTGTTTAAGA AGACGATGGC CTTATTAGGG
AGTGCCTAAT AGACTTCTAT ACCAGTTCTG GGAAAACCTG ATCAGATTAT AATCTTCAGG
GATGGTGTTA GTGAGAGCCA ATTTACTCAG GTGCTAAACA AGGAGTTAGA TCAAATAAAT
GAGGCTGCAA ATTCTTGATG AAAGTGGTCC CCCAAATTCA CACTAATTGT TGCGCAGAAA
AACCACCACA CAAAGTTTTT CATGATCACC GGA------A ATGTCCCTCC CGGCACTGTT
GTTGATAACG TAGTTTGCCA TCCAAAGAAC TATGATTTCT ACATGTGCGC ACATGCAGGA
ATGATAGGAA CAACAAGGCC AACACATTAC CATATCCTGC ATGATGATAT ACACTTTACC
GCAGATGATC TGCAGGATCT CGTGCACTCG CTCTCTTATG TGTACCAAAG AAGCACAACG
GCCATATCAG TAGTCTCTCC AATCTGCTAT GCCCATCTTG CTGCTGCCCA AGTGTCACAG
TTCGTCAAG
>Sbicolor_Sb02g005150
TCACTCTTTC TCGATCCTTC TTCTCGAAGT TTGAGACATT GGGCTAGAGA GCTGGAGCGG
ATATTACCAG AGCTTGCGCC CAACTCAAAT GGGCTCTCAT TGAACATTGA TTATGCTCGA
CTTCATTTTA CCAATCCCTG TGTAAAATTT ATTGTCTTGG GATCGAAGCT TAAGAAAGCC
CTGCGTGGAG TTCGTGTTGA GACTACACAC CAGCCTACAA GATAACTGGG ATTACTTCCA
TTGGCTCAGC TAGCTTTTCT TGTAACGACC TCAGTTGACT GTTGTCCAGT ACTTTGCTGA
ACGGTACAAC TACCGGTTGC GCTATACCTT GGCCCTGCCT TCAGTCCGGC AATGATTCAA
GCCGATATAT TACCTATGGA GGTTGCCAAA TCATTGAAGG ACAGAGGTAC CCTAGGCTCA
GCGACACACA GGTGACCAAT ATACTGAATG TAACTGCTTA TTGTCGTTCT CCAGAGTTTC
TGGTCAT--- ---TATGGAA ATTAAGAGGA TATGCGAGAC TGACCTTGGT ATAGTATCTC
AGTGCATCAA TCCAAGAAG- -----AAAAC AACAGTATTT TGAAAATGTC GCCCTTAAAA
TCAATGTGAA GTGGGAGGGC GCAATACAGT GCTAGAGCCT TTGTGCCATA CCTTGTCTCA
GACCAACAAT CATTTTTGGT GCTGATGTTA CCCATCCGCA GGAGATCGGC TTCTATTGCA
GCTGTGGTTG CATCCATGGA CTGGCCACAA GTCACAACAT ATAAAGCACT AGTCTCGGCG
CAAGCACATA GGGAAGAGAT TATACAAAAT CTGTTCTGGT GAACGGTGGA ATGATAAGGG
AGTTGCTGAC TTCATTCTTT AAGAGGACTG GGAAAGCCCA AAAGGATTAT ATTTTACAGG
GATGGTGTAA GTGAGGGACA ATTCAGCCAC GTTTTGCTCC ATGAAATGGA CGCAATCAGG
AAGGCTGTGC CTCTATGAAG ATGGTATCTA CCACCAGTGA CATTCGTGGT GGTACAGAAA
AGGCACCACA CAAGGCTCTT CCCTTCATGG AAGAGCGGAA ACATTCTTCC TGGAACTGTG
GTTGATACTA GCATTTGTCA TCCCAGCGAG TTTGATTTCT ACCTCTGTAG CCATGCTGGA
ATTAAGGGAA CAAGCAGGCC AACACACTAT CATGTCCTCT ATGATGAGAA CCGTTTCTCG
GCTGATGCTC TGCAGTTTCT CACAAACAAC CTTTGCTACA CATACGCACG CTGCACACGC
GCTGTTTCTG TTGTTCCACC AGCCTACTAC GCTCACCTGG CAGCATTCCG CGCAAGGTAC
TATGACGAA
>Mesculenta_cassava1330.valid.m1
TACCTGTGGG GAGATCATTC TATTCTTCAA TGGGGAAATT GGAGCCATTG GATTGAGAGG
ATTCTTTCAA AGTCTCAGAC CCACCCAACA AGGTTAGCTC TCAATGTGGA TTTTCAGTGA
CTGCTTTTCA TGAAAGAAGC GCTCAAGTTT ACCTCCCACA GAAAGGAAGT GGAGAAGGCT
TTGAAGAACA TCAGGGTCTT TGTTTGCCAC AGAGGTACCG AGTTTTCGGC TTAACAAGCT
ACGGACAATC TTGGTTCGCG GACAGGGAAA GAATCTGAGG CTGGTGAGTT ACTTCAAGGA
CCACTACAAT TATGATATAA AATTCCGACT TGCCATGTTT GCAGATCAGT AGAAGT--AA
ACCATGTTAT TTCCTATGGA GCTTGTATGA TATGTGAAGG CCAGAAGTTT CTTGGTCTGT
CAGATGATCA GACTGCAAAA ATACTCAATG CCACTGCTTA TTTGTATAAT -----GAGAA
GAGACACAGA GGATATGCAT TTGAAGCGGA TAGCAGAGAC AAATGTAGGT GTTGTAAGCC
AGTGCTGCTT GTTACAAATC TTGGCAAAGT TCCAATTTCT TTCCAATTTG TCTCTCAAGA
TCAATGCCAA ATTGGAGGAT GTACAGTTGC TTTAATTCAT TACCCTCATT CCTTCATTCC
GACCCGTGAT CTTCATGGGA GCTGACGTCA CTCATCCCCT CTCGAAGTCC ATCAGTTGCT
GCTGTTGTTG GTAGCATGAA CTGGCCAGCA GCAAATAAGT ATGCATCTCG AATGAGGTCG
CAAACGCATC GCCAAGAAAT CATCCAGGAT CTT------- ----GGTTCA ATGGTGAAAG
AATTGCTAGA TGATTTTTAC AAAGAAGCAA AAACTTCCCA AAAGGATAAT GTTCTTCAGG
GATGGAGTAA GTGAAACTCA GTTTCATAAG GTTCTCCAAG AAGAGCTGAA ATCAATTCGA
GAAGCTGTTC TAGATT---C CTTGTATAAA CCTCCCATTA CCTTTGCAGT TGTCCAAAAG
AGGCATCATA CAAGGTTGTT TCCGCGAAAC AGAAATGAAA ATATCCCACC AGGGACAGTT
GTGGACACTG TGATTACACA TCCAAAGGAA TTTGATTTTT ATTTGTGCAG CCATTGGGGG

GTGAAAGGAA CAAGCAGGCC AACACATTAC CATGTGTTGT GGGATGAGAA CCAATTCACT
TCTGATGAAT TACAGAAGTT GGTATACAAT CTCTGCTACA CATTTGTAAG ATGCACCAAG
CCAGTTTCTT TGGTGCCTCC TGCTTACTAC GCCCACTTAG CTGCATACAG GGGCAGGCTT
TACATTGAA
>Mesculenta_cassava2314.valid.m1
TACCTGTAGG GAGATCATTC TATTCTTTAA TGGGGAAATT GGAGCTGTTG GATTGAGAGG
ATTCTTTCAA AGTCTTAGAC CCACACAACA AGGCTAGCTC TCAATGTAGA TTCTCCGTGA
CCGCTTTTCA TGAAAGAAGC GCTCAAGTTT ACCTTCCTCA GAAAGGAAGT GGAGATAACT
TTGAAGAATA TCAGGGTCTT TGTTTGCCAC AGAGATACCG GGTTTATGGC TTGACAAGCT
ACGGATAATA TTGGTTCGCC GACAGGGAAA GAAACTGAGG CTGGTGAGTT ACTTCAAGGA
TCACTACAAT TATGACATAA AATTCAGACT TGCCATGTTT GCAGATTAGT AGAAGT--AA
ACCATGTTAT TTCCCATGGA GCTTGTATGA TTTGTGAAGG CCAGAAGTTT CTTGGGCTGT
CCGATGATCA GACAGCAAAA ATTCTCAATG CCACTGCTTA TTTGTATAAT -----GAGAA
GAGACACAAA GGATATGCAT TTGAAGCGAA TAGCAGAAAC AAATGTTGGT GTTGTAAGCC
AGTGCTGCTT GTTCCGAACC TCGGCAAAAT CCCAATTTCT AGCTAATTTG GCTCTCAAGA
TCAATGCTAA ATTGGAGGAT GCACAGTCGC TTTAATTCGT TACCCTCATT CCTTCATTCT
GACCTGTGAT CTTCATGGGA GCTGATGTAA CTCATCCCCT CTTGAAGTCC ATCAGTTGCT
GCTGTTGTTG GTAGCATGAA CTGGCCAGAA ACAAACAAGT ATGCATCACG AATGAGGTCG
CAAACGCATC GACAAGAAAT TATTCAGGAC CTT------- ----GGTGCA ATGGTGAAAG
AATTGCTAGA TGAATTTTAT CAAGAAGCAA GAACTTCCCA AGAGGATAAT ATTCTTCAGG
GATGGAGTAA GCGAAACCCA GTTCTACAAG GTGCTTCAAG AGGAGTTGCG GGCGATTCAA
GAAGCTGTTC GAGATA---C CAGGTATAGA CCTCTCATTA CATTTGCAGT TGTCCAAAAG
AGACACCATA CAAGGTTGTT TCAGTGAAAC AGAGGCGAAA ACATACCCCC AGGAACAGTT
GTGGATACTG TTATTACTCA TCCAAAGGAA TTTGATTTTT ATCTATGCAG CCATTGGGGA
GTAAAAGGAA CAAGTAGGCC AACTCATTAC CATGTCTTGT GGGATGAGAA CCAATTCACT
TCTGATGAAT TACAGAAGCT AGTTTACAAT CTCTGCTACA CATTTGTAAG GTGCACAAAG
CCAATTTCTT TGGTGCCTCC TGCTTACTAT GCACACTTAG CTGCATACAG GGGCAGGCTT
TACCTCGAA
>GRMZM2G441583_T01 cds:_protein_coding
GTCCTGTTGG TAGATCATTT TATTCCAATT TAGGCAACTT GGTTTGGAAA CTTGGCGTGG
TTTCTATCAA AGCATAAGGC CCACACAGAT GGGCTTTCTC TGAATATTGA TTGTCCTCTA
CTGCATTTAT TGAGCCCCTG TATTGATTTT AGCTTCTTGA GATCGAAGAT TAAAAAAGCC
CTACGAGGTG TGAAAGTCGA GGTCACACAC CGAAGTATCG GATATCTGGC CTCACAAGCA
ACAAGGGAGT TTCATTCCCT ATTGATGAAC TGTTAAGACT GTGGTGCAAT ACTTCCTGGA
GACTTATGGC TTTAATATTC AGCACACCTT TACCTTGTTT GCAAGTGGGC AATCAGCAAG
AATAAATTAT TGCCTATGGA GGTTGTAAGA TAGTTGAGGG ACAGCGTTAC TCAAAACTCA
ATGAGAAACA GATCACTGCT CTACTGAATG CCATTGCTGA TGGTAATACT CCTGAAATAA
TGGCTCT--- CTTTACGGAT CTTAAAAGGA TATGTGAGAC TGATCTTGGA TTGGTCTCCC
AATGCTGTCT GACAAACATG TTTTCAAAAC AACAGTATCT TGCAAATGTT GCCCTGAAAA
TAAATGTTAA GTTGGGGGAA GGAATACTGT ACTGATGCTT TGGCAAGATC CCTTGTCAGT
GAGCTACTAT TATCTTTGGT GCTGATGTGA CCCATCCCCT GGGGAAGTCC TTCCATTGCA
GCTGTGGTTG CTTCTCAAGA CTGGCCTGAG GTTACGAAGT ATGCAGGATT AGTGAGTGCT
CAAACCCATC GCCAAGAATT GATACAGGAT CTTTTCAAGT CTCTGGTGGC ATGATTAGGG
AACTTCTCAT TTCTTTCTGG AGGGCGACTG GAAAAACCCA AGAGGATCAT ATTCTACAGG
GATGGTGTCA GTGAGGGACA GTTCTACCAA GTTCTGTTGT ACGAACTTGA TGCCATTAGA
AAGGCTGTGC ATCATTGAGT CTGATACCAG CCTCCAGTTA CCTTTGTCGT GGTCCAGAAG
CGTCATCACA CCAGGTTGTT TGTATCACAA TGAAGTGGCA ACATACTGCC GGGCACTGTG
GTGGACTCGA AGATTTGCCA TCCAACCGAG TTTGATTTCT ACCTGTGCAG CCATGCTGGC
ATTCAGGGAA CAAGCCGTCC TGCCCATTAT CATGTTCTGT GGGATGAGAA CAAATTTACG
GCTGATGGGT TGCAAACTCT CACCAACAAC TTGTGTTACA CGTATGCCAG GTGCACACGC
TCAGTATCAA TCGTTCCTCC TGCATACTAT GCTCATCTGG CAGCCTTCCG AGCTCGGTTT
TACATGGAG
>Gmax_Glyma06g23920
TGTCCATTGG GAGGTTTCTG TATTCTGATG TAAGCAGTTG GGTCTTGAAT CATGGAGGGG
CTTCTATCAG AGTATAAGGC CTACTCAGAT GGGTTGTCAC TTAACATTGA TTGTCATCAA
TGGCATTTAT CGAACCCCTG TATTGACTTT AAATTTTGGG GATCGAAGAT CAAAAAGGCC

CTAAGAGGTG TAAAAGTTGA AGTTACACAT AGAAGTATAG AATATCAGGA CTGACAGCCT
ACAAGGGAGC TATTTTCCCT CTTGATGAAA CATGAAATCA GTAGTTGATT ATTTTCAAGA
AATGTATGGA TTTACAATCA AATATTCATC TACCTTGCCT TCAAGTAGGA AGTCAAAGAA
GGTGAACTAT TGCCAATGGA GGCTGCAAGA TAGTTGGGGG CCAGAGATAT ACAAAACTAA
ATGAAAAGCA GATAACTTCT CTGCTGAATG TCATTGCTGA TTGCCCTTCT CCAGAAATAA
TGGCTCT--- TTGTATGGAT CTCAAAAGAA TCTGCGAAAC AGATCTGGGG TTGATTTCTC
AATGCTGCCT TACAAACATG TATTCAAAAT AGCAGTACCT GGCTAACGTG GCACTAAAAA
TCAATGTCAA GTGGGAGGAA GAAACACAGT ACTGATGCTT TAAGTTGATT CCTGGTTAGT
GACCAACAAT AATATTTGGA GCTGATGTAA CTCATCCTCA GGAGATGTCC ATCCATTGCT
GCTGTTGTAG CCTCCCAGGA CTGGCCTGAA GTAACAAAGT ATGCAGGGTT GGTTTGTGCT
CAGCCTCATC GAGAAGAACT AATTCAAGAT CTTTTCCGAT GTACGGTGGT ATGATCAGGG
AGCTTTTACT ATCATTTAAG AAGGCAACTG GAAAAACCAT TGAGAATAAT CTTTTACAGG
GATGGGGTAA GTGAGGGACA GTTCTACCAG GTTTTGCTAT ATGAGCTTGA TGCCATACGC
AAGGCTGTGC ATCTTTGAAC CTAGTACCAA CCTCCAGTAA CATTTGTTAT TGTTCAAAAA
CGGCATCACA CTAGACTCTT CGCATCATGA TGAAGTGGGA ATATCTTACC TGGTACTGTG
GTAGATTCTA AGATCTGTCA TCCTTCGGAA TTCGACTTCT ACTTGTGCAG TCATGCAGGA
ATTCAGGGTA CAAGTAGACC AGCACACTAT CACGTCCTCT GGGACGAGAA CAATTTCACT
GCAGATGAGA TTCAGTCTCT AACCAACAAT TTGTGCTACA CTTATGCAAG ATGTACGCGG
TCTGTTTCAG TAGTGCCTCC AGCATACTAT GCTCATCTAG CAGCCTACAG GGCTCGATTC
TACATGGAA
>CL5705_sinensis
CAGTTGTGGG AAGGTCATTT TTCTCTGATC TTGGCAGCTT GGCGTTGAAT ATTGGCGTGG
ATATTTTCAG AGTCTTCGCC CAACCCAGAT GGGCTATCTC TTAATATTGA TTTTCAGCCC
GCTCCTTTTA TGAGCCCCAG TACTGAGTTT ATTATTGCAG GTGCGAAGGT GAAAAAGGCA
TTGAAGGGAA TAAAGGTAGT GCTCACGCAT ATAGTTACAA GATCACTGGA ATATCAACCG
ATGAGCCGAC TATGTTTACT GATGACAAAC AAGGTTGTCA GTGGTTCAAT ATTTTCGTCA
AAGATATAAT ATTGGGCTTC AATTCACCAT TGCCTGCTCT TGTAGCTGGA AGTGAAGCAG
GCCTATTTAT TGCCAATGGA GCTTCTAGGA TTGCCGCGGG ACAGAGATAT ACTAAATTAA
ATGAAAGGAC AGTAACTGCT CTTTTAGA-- ---------- ---------- ----------
---------- ---------- ---------- ---------- ---------- ----------
---------- ---------- ---------- ---------- ---------- ----------
---------- ---------- ---------- ---------- ---------- ----------
---------- ---------- ---------- ---------- ---------- ----------
---------- ---------- ---------- ---------- ---------- ----------
---------- ---------- ---------- ---------- ---------- ----------
---------- ---------- ---------- ---------- ---------- ----------
---------- ---------- ---------- ---------- ---------- ----------
---------- ---------- ---------- ---------- ---------- ----------
---------- ---------- ---------- ---------- ---------- ----------
---------- ---------- ---------- ---------- ---------- ----------
---------- ---------- ---------- ---------- ---------- ----------
---------- ---------- ---------- ---------- ---------- ----------
---------- ---------- ---------- ---------- ---------- ----------
----
>Vvinifera_GSVIVT00001880001
TGCCTGTGGG GAGATCCCTG TACTCCTCAA TGGGGATATT GGAGCGGTTG GATTAAGAGG
GTTCTTTCAG AGTCTTAGAC CAACCCAACA AGGCTTGCTC TCAATGTGGA TTTTCAGTGA
CTGCTTTCCA TGAGAGAAGC GGTTGAGTTT ACCTTTCCCA AGGAAGAAGT GGAGAAGGCA
TTGAAGAATA TTAGGGTCTT TGTACGTCAT AGAGATATCG GGTGCATAGC TTAACAAACT
ACAGAAAATC TTGGTTTGAA GACAGGGAAA GATTCTGAGG CTGGTGAATT ACTTCAAGGA
TCACTATGGC TACGATATAC AGTTCAGACT TGCCATGTTT GCAGATTAGT AGGAGC--AA
ACCATGCTAT TTCCTATGGA GCTTGTATGA TCTGTGAAGG CCAAAAATTT CTTGGGCTCT
CTGATGATCA AACTGCAAGA ATACTTAATG CCACTCCTTA TATGCATAAT -----GAGAG
AAAGCACAAA GGGTATGCAT TTGAAGCGAA TTGCTGAGAC CAGCATTGGG GTTGTAAGTC
AGTGCTGCTT GTACAAAATC TTGGCAAAGT TCCAGTTTCT GGCAAACCTG GCTCTCAAGA
TCAATGCCAA ATGGGAGGAT GCACTGTTGC CTTAATTCAC TTCCTTCATA CCTTCGGCCT

GACCAGTCAT CTTTATGGGT GCTGATGTGA CTCATCCCCT CTTGAAGCCC CTCCATTGCT
GCTGTGGTTG GGAGCATGAA CTGGCCAGCA GCTAACAAGT ATGTTTCAAG AATGAGGTCC
CAGACCCATC GTCAAGAAAT CATCCAGGAT CTT------- ----GGTGCC ATGGTTGGAG
AAATACTGGA TGATTTTTAT CAGCAAGTAT CAACTCCCCA AGAGGATCAT TTTTTTCAGG
GATGGAGTGA GCGAAACCCA GTTCTATAAG GTGCTCCAAG AGGAGTTGCA AGCTATAAGA
GTGGCTGTTG TAGATT---C CCAATACAGA CCTCCCATTA CTTTTGCAGT GGTGCAGAAG
AGGCACCACA CGAGGTTGTT TCGATGAAAG CAAGAGGAGA ACATTCCCCC AGGGACAGTT
GTGGATGCTG TGATTACACA CCCAAGGGAA TTTGATTTCT ATCTTTGTAG CCATTGGGGG
GTGAAGGGAA CAAGCCGACC AACCCACTAC CATATATTGT GGGATGAAAA CCATTTCACT
TCTGATGAAG TACAGAAGCT GGTTTACAGT CTATGCTACA CATTTGTGAG ATGTACAAAG
CCTGTGTCGT TGGTCCCACC AGCTTACTAT GCCCACCTGG CTGCATACAG AGGCAGACTC
TACCTGGAG
>Mesculenta_cassava35367.valid.m1
GTCCCATAGG AAGATCCTTC TTTTCAGATA TTAGCGGCTT GGCTTGGAGT CTTGGTGTGG
GTTTTACCAG AGTATAAGGC CTACACAAAT GGGCTCTCTT TAAATATTGA TTGGCTTCTG
CTGCATTCAT CGAGCCCCTG TATTGAGTTT AGCTTCTAGG GATAGAAGAT TAAAAAGGCC
CTCAGGGGAG TGAAAGTTGA AGTAACTCAT AGAAGTATCG TGTCTCAGGA TTGACAACCT
ACAAGAGAAC TGTATTTCCT GTTGATGAAC TATGAAGTCA GTAGTTGAGT ACTTCCAAGA
GATGTATGGC TTCACCATTC AGCATTCATC TCCCTTGCCT TCAAGTAGGA AACCAGAAAA
GGCAAACTAT TCCCGATGGA GGCTGCAAAA TTGTAGAAGG TCAACGATAC ACTAAATTGA
ATGAGAGACA AATTACTGCA CTGTTGAATG CCACTTTTAT TAGCTATTCT CCCGAAACAA
CGGATCC--- CTTTATGGAC CTTAAGCGGA TATGTGAAAC TGATCTTGGT TTAATATCAC
AATGCTGTCT TACAAACATG TCTTCAAAGC AACAGTATTT GGCTAATGTG TCACTGAAGA
TTAATGTTAA GTGGGTGGTA GAAACACTGT CCTGACGCCA TTAGCTGATA CCTAGTTAGT
GACCAACCAT TATATTTGGA GCAGATGTGA CTCACCCAAT GGGGAAGCCC TTCAATTGCA
GCAGTAGTAG CTTCTCAAGA CTGGCCTGAA GTGACAAAAT ATGCTGGATT AGTTTGTGCT
CAAGCTCACA GACAAGAACT CATACAAGAC TTGTATAAGT TAGTGGGGGC ATGATCAGAG
ATCTTCTGGT TTCCTTTAGG AAGGCAACAG GAGAAGCCGC TGAGGATTAT ATTCTACAGG
GATGGTGTTA GCGAAGGGCA ATTCTATCAA GTTCTGCTTT ATGAATTAGA TGCAATCCGG
AAGGCTGTGC CTCTTTGAAC CAAATATCAA CCACCTGTGA CTTTCATAGT TGTACAGAAA
CGGCATCATA CTCGATTATT TGCACCACAG GGAAGTGGGA ACATATTACC TGGTACTGTG
GTTGACTCTA AAATCTGTCA CCCTACAGAA TTTGATTTTT ATCTCTGTAG CCATGCTGGT
ATTCAGGGAA CAAGTCGGCC TGCTCACTAC CATGTTTTGT GGGATGAAAA CAACTTCACT
GCTGATGGAA TCCAGTCATT GACAAACAAT CTATGCTATA CATATGCAAG ATGCACTCGC
TCTGTTTCTG TCGTTCCTCC AGCATATTAT GCACATTTGG CTGCTTTTCG CGCTCGATTT
TACATGGAG
>GRMZM2G039455_T01 cds:_protein_coding
CTCCTGTTGG TAGGTCATTT TATTCCAACT TAGGCAACTT GGTTTGGAAA GTTGGCGCGG
TTTTTACCAA AGCATAAGGC CGACACAGAT GGGCTTTCAC TGAATATTGA TTGTCCTCTA
CTGCATTTAT CGAGCCCCTG TATTGATTTT AGCTTCTTAA GATCGAAGAT CAAAAAAGCC
TTAAGAGGTG TGAAGGTTGA GGTCACTCAC AGAAGTATCG CATTTCTGGC CTCACAAGCA
ACAAGAGAGC TTCATTCCCT GTTGATGAAC TGTGAAGACT GTGGTCCAAT ACTTCATGGA
GACTTATGGT TTTAGCATCC AGCACACCTT TACCGTGCTT GCAAGTGGGC AATCAACAAG
ACCAAATTAT TGCCTATGGA GGTTGCAAGA TAGTTGAAGG ACAGCGTTAC TCAAAGCTCA
ATGAGAAACA AATCACTGCT TTACTGAATG CCACTGCTGA TTGTAATACT CCTGAATTAA
TGGTTCC--- TTATATGGAT CTCAAAAGGA TCTGTGAGAC TGATCTCGGA TTGGTCTCCC
AGTGTTGTCT GACAAACATG TTTTTAAAGC AACAGTATCT TGCAAATGTT GCACTCAAAA
TAAATGTTAA GTTGGTGGAA GGAATACTGT ACTGATGCTT TGACAAGATC CCTTGTCAGT
GACCGACCAT AATATTTGGT GCTGATGTTA CCCATCCCCT GGAGAAGTCC TTCCATTGCA
GCTGTGGTTG CTTCGCAAGA CTGGCCTGAG GTCACCAAAT ATGCTGGACT AGTGAGTGCC
CAAGCCCATC GCCAGGAGCT GATACAGGAT CTTTTCAAGT AACTGGTGGC ATGATAAAGG
AACTTCTCAT TTCTTTCAAG AGAGCAACTG GAGAAGCCCC AGAGGATCAT ATTCTACAGG
[truncated: 98,655 more chars]
